# A yeast phenomic model for the influence of Warburg metabolism on genetic buffering of doxorubicin

**DOI:** 10.1186/s40170-019-0201-3

**Published:** 2019-10-23

**Authors:** Sean M. Santos, John L. Hartman

**Affiliations:** Department of Genetics, University of Alabama at Birmingham, Birmingham, AL USA

**Keywords:** Genetic buffering, Yeast phenomics, Quantitative high throughput cell array phenotyping (Q-HTCP), Cell proliferation parameters (CPPs), Doxorubicin, Warburg metabolism, Differential gene interaction networks, Recursive expectation-maximization clustering (REMc), Pharmacogenomics, Human-like/HL yeast media

## Abstract

**Background:**

The influence of the Warburg phenomenon on chemotherapy response is unknown. *Saccharomyces cerevisiae* mimics the Warburg effect, repressing respiration in the presence of adequate glucose. Yeast phenomic experiments were conducted to assess potential influences of Warburg metabolism on gene-drug interaction underlying the cellular response to doxorubicin. Homologous genes from yeast phenomic and cancer pharmacogenomics data were analyzed to infer evolutionary conservation of gene-drug interaction and predict therapeutic relevance.

**Methods:**

Cell proliferation phenotypes (CPPs) of the yeast gene knockout/knockdown library were measured by quantitative high-throughput cell array phenotyping (Q-HTCP), treating with escalating doxorubicin concentrations under conditions of respiratory or glycolytic metabolism. Doxorubicin-gene interaction was quantified by departure of CPPs observed for the doxorubicin-treated mutant strain from that expected based on an interaction model. Recursive expectation-maximization clustering (REMc) and Gene Ontology (GO)-based analyses of interactions identified functional biological modules that differentially buffer or promote doxorubicin cytotoxicity with respect to Warburg metabolism. Yeast phenomic and cancer pharmacogenomics data were integrated to predict differential gene expression causally influencing doxorubicin anti-tumor efficacy.

**Results:**

Yeast compromised for genes functioning in chromatin organization, and several other cellular processes are more resistant to doxorubicin under glycolytic conditions. Thus, the Warburg transition appears to alleviate requirements for cellular functions that buffer doxorubicin cytotoxicity in a respiratory context. We analyzed human homologs of yeast genes exhibiting gene-doxorubicin interaction in cancer pharmacogenomics data to predict causality for differential gene expression associated with doxorubicin cytotoxicity in cancer cells. This analysis suggested conserved cellular responses to doxorubicin due to influences of homologous recombination, sphingolipid homeostasis, telomere tethering at nuclear periphery, actin cortical patch localization, and other gene functions.

**Conclusions:**

Warburg status alters the genetic network required for yeast to buffer doxorubicin toxicity. Integration of yeast phenomic and cancer pharmacogenomics data suggests evolutionary conservation of gene-drug interaction networks and provides a new experimental approach to model their influence on chemotherapy response. Thus, yeast phenomic models could aid the development of precision oncology algorithms to predict efficacious cytotoxic drugs for cancer, based on genetic and metabolic profiles of individual tumors.

## Background

The Warburg effect refers to the phenomena of cancer cells undergoing a metabolic transition from respiration to aerobic glycolysis and has been documented for over 90 years, yet there remains a lack of consensus regarding how this contributes to cancer [1–3]. In humans, aerobic glycolysis is a cancer-specific metabolic transition; however, yeast normally represses respiration in the presence of adequate glucose [4–6]. Although not possible in a single cell organism to ascertain the role of the Warburg transition in oncogenesis, we wondered whether it might influence chemotherapeutic response, and particularly in the context of vulnerabilities created by genomic instability and unique to individual patient’s cancers. Using doxorubicin as a model anti-cancer agent, we examined whether doxorubicin-gene interaction manifests differentially under glycolytic vs. respiratory conditions in yeast and how genetic insights from the yeast model might lead to predicting variable efficacy in killing cancer cells. It is also possible that the model could be informative regarding dose-limiting toxicity observed in cardiomyocytes, which have respiratory rates among the highest of all cell types [7].

Doxorubicin is used widely in oncology to treat both hematologic cancer and solid tumors [8]. Proposed mechanisms of doxorubicin cytotoxicity include topoisomerase II poisoning, DNA adduct formation, oxidative stress, and ceramide overproduction [8–13]. Topoisomerase II is an ATP-dependent enzyme that relieves the DNA torsional stress occurring with replication or transcription by catalyzing a double-stranded DNA (dsDNA) break, relaxing positive and negative DNA supercoiling, and finally re-ligating the DNA [14]. Inhibiting this activity can result in irreparable DNA damage and induction of apoptosis, selectively killing rapidly dividing proliferating cells [15–17]. Doxorubicin also causes histone eviction leading to chromatin trapping and damage [9, 18–20]. In addition to its potent anti-cancer therapeutic properties, doxorubicin is known for dose-limiting cardiomyocyte toxicity, causing cardiomyopathy and heart failure years post-treatment [21]. In this regard, topoisomerase IIB is highly expressed specifically in myocardiocytes, where tissue-specific deletion suppresses cardiac toxicity in mice [22]. Clinical guidelines recommend a maximum cumulative lifetime dose of 500 mg/m^2^; however, doxorubicin toxicity is variable and has a genetic basis [23]. Thus, a detailed understanding of drug-gene interaction could advance the rationale for more precisely prescribing doxorubicin (among other cytotoxic agents) and also predicting toxicity, based on the unique genetic context of each patient’s tumor genetic profile as well as germline functional variation.

To address these questions, this work establishes a yeast phenomic model to understand genetic pathways that buffer doxorubicin toxicity [24–30], and how the Warburg effect influences the doxorubicin-gene interaction network. We conducted yeast phenomic analysis of doxorubicin-gene interaction, consisting of quantitative high throughput cell array phenotyping (Q-HTCP) of the yeast knockout and knockdown (YKO/KD) libraries, using multiple growth inhibitory concentrations of doxorubicin in either dextrose- (HLD) or ethanol/glycerol-based (HLEG) media. Q-HTCP provided cell proliferation parameters (CPPs) with which to quantify doxorubicin-gene interaction and determine its dependence on respiratory vs*.* glycolytic metabolism [31–33]. The yeast phenomic model was used to predict causality underlying correlations between doxorubicin sensitivity and increased or decreased expression of the homologous human gene in pharmacogenomics data from cancer cell lines. Thus, the work details genetic pathways for buffering doxorubicin toxicity in yeast, including the influence of Warburg metabolism on the network, and applies the information to predict interactions between doxorubicin and functional genetic variation that could be present in cancers from different, individual patients.

## Methods

### Strains and media

The yeast gene knockout strain library (YKO) was obtained from Research Genetics (Huntsville, AL, USA). The knockdown (KD) collection, also known as the Decreased Abundance of mRNA Production (DAmP) library, was obtained from Open Biosystems (Huntsville, AL, USA). The genetic background for the YKO library was BY4741 (S288C MAT**a** *ura3-∆0 his3-∆1 leu2-∆0 met17-∆0*). Additional information and lists of strains can be obtained at https://dharmacon.horizondiscovery.com/cdnas-and-orfs/non-mammalian-cdnas-and-orfs/yeast/#all. Some mutants appear multiple times in the library and they are treated independently in our analysis. HL yeast media, a modified synthetic complete media [27], was used with either 2% dextrose (HLD) or 3% ethanol and 3% glycerol (HLEG) as the carbon source.

### Quantitative high throughput cell array phenotyping (Q-HTCP)

Phenomic data was obtained by Q-HTCP, a custom, automated method of collecting growth curve phenotypes for the YKO/KD library arrayed onto agar media [33]. A Caliper Sciclone 3000 liquid handling robot was used for cell array printing, integrated with a custom imaging robot (Hartman laboratory) and Cytomat 6001 (Thermo Fisher Scientific, Asheville, NC, USA) incubator. Three hundred eighty-four-culture array images were obtained approximately every 2 h and analyzed as previously described [28, 33]. To obtain CPPs, image data were fit to the logistic equation, *G*(*t*) = K/(1 + *e*^−*r*(*t*−*l*)^), assuming *G*(0) < *K*, where *G*(*t*) is the image intensity of a spotted culture vs. time, *K* is the carrying capacity, *r* is the maximum specific growth rate, and *l* is the moment of maximal absolute growth rate, occurring when *G*(*t*) = *K*/2 (the time to reach half of carrying capacity) [31]. The resulting CPPs were used as phenotypes to measure doxorubicin-gene interaction.

### Quantification of doxorubicin-gene interaction

Gene interaction was defined by departure of the corresponding YKO/KD strain from its expected phenotypic response to doxorubicin. The expected phenotype was determined by cell proliferation phenotypes of the mutant without doxorubicin, together with those of the reference strain with and without doxorubicin [24–26, 28]. The concentrations of doxorubicin (0, 2.5, 5, 7.5, and 15 ug/mL) were chosen based on phenotypic responses being functionally discriminating in the parental strain. We tested for effects of mating type or ploidy on doxorubicin growth inhibition (Additional file 1: Figure S1) and noted only small differences between the YKO/KD parental strain genotypes, BY4741 (MAT**a** *ura3-∆0 his3-∆1 leu2-∆0 met17-∆0*), BY4742 (MAT**α** *ura3-∆0 his3-∆1 leu2-∆0 lys2∆0*), BY4741R (MAT**a** *ura3-∆0 his3-∆1 leu2-∆0 lys2∆0*), BY4742R (MAT**α** *ura3-∆0 his3-∆1 leu2-∆0 met17-∆0*), and diploid strains derived from these haploids. In this regard, haploid *MET17/lys2-∆0* was associated with a lower carrying capacity in HLD media (Additional file 1: Figure S1), but genome-wide experiments were not performed in this background.

Interaction scores were calculated as previously described [28], with slight modifications, as summarized below. Variables were defined as follows:

*D*_*i*_ = concentration (dose) of doxorubicin

*R*_*i*_ = observed mean growth parameter for parental reference strain at *D*_*i*_

*Y*_*i*_ = observed growth parameter for the YKO/KD mutant strain at *D*_*i*_

*K*_*i*_ = *Y*_*i*_–*R*_*i*_, the difference in growth parameter between the YKO/KD mutant (*Y*_*i*_) and reference (*R*_*i*_) at *D*_*i*_

*K*_0_ = *Y*_0_ –*R*_0_, the effect of gene KO/KD on the observed phenotype in the absence of doxorubicin; this value is annotated as “shift” and is subtracted from all *K*_*i*_ to obtain *L*_*i*_

*L*_*i*_ = *K*_*i*_–*K*_0_, the interaction between (specific influence of) the KO/KD mutation on doxorubicin response, at *D*_*i*_

For cultures not generating a growth curve, *Y*_*i*_ = 0 for *K* and *r*, and the *L* parameter was assigned *Y*_*i*_ max, defined as the maximum observed *Y*_*i*_ among all cultures exhibiting a minimum carrying capacity (*K*) within 2 standard deviations (SD) of the parental reference strain mean at *D*_*i*_. *Y*_*i*_ max was also assigned to outlier values (i.e., if *Y*_*i*_ > *Y*_*i*_ max).

**Fig. 1:**
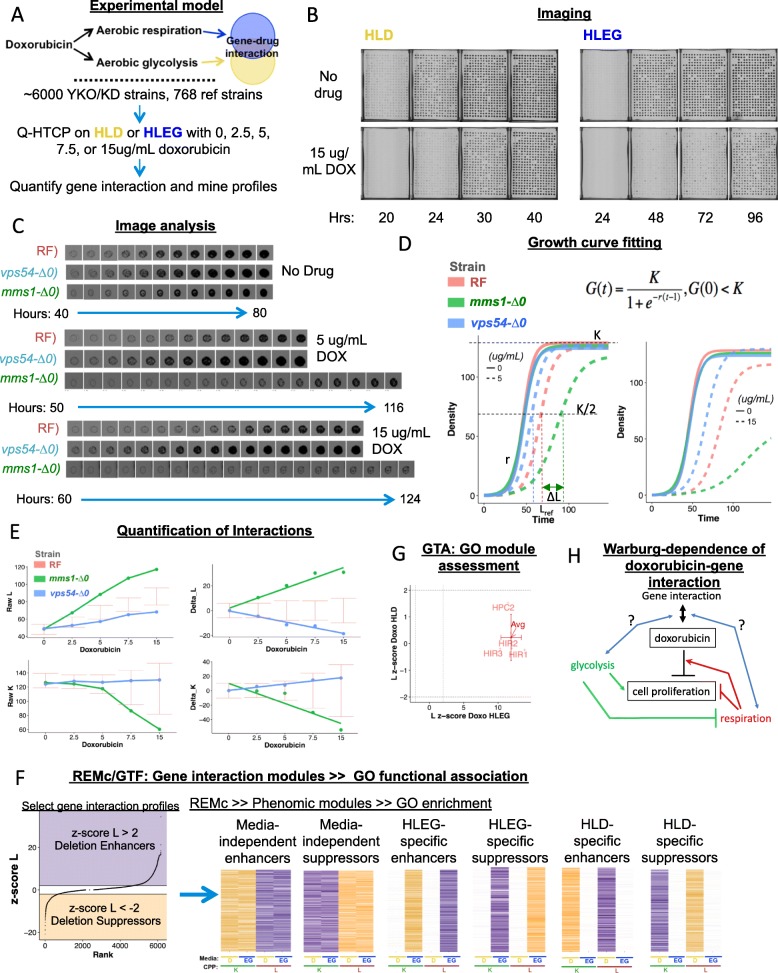
Experimental strategy to characterize differential doxorubicin-gene interaction, with respect to the Warburg metabolic transition. **a** The phenomic model incorporates treatment of individually grown cultures of the YKO/KD collection with increasing doxorubicin (0, 2.5, 5, 7.5, and 15 ug/mL) in “fermentable/glycolytic” (HLD) or “non-fermentable/respiratory” (HLEG) media. **b** Representative cell array images, treated and untreated with 15 ug/mL doxorubicin. **c** Time series of individual culture images, exemplifying gene deletion suppression (*vps54-∆0*) and gene deletion enhancement (*mms1-∆0*), relative to parental control (“RF1”) in HLEG media with indicated concentrations (0, 5, and 15 ug/mL) of doxorubicin. **d** After image analysis, data time series are fit to a logistic growth function, G(*t*), to obtain the cell proliferation parameters (CPPs), *K* (carrying capacity), *L* (time at which K/2 is reached), and *r* (maximum-specific rate) for each culture. “∆*L*” (left panel) indicates *K*_*i*_ (see the “Methods” section). **e** Interaction is quantified by linear regression of *L*_*i*_ (indicated “Delta_L” and “Delta_K” in right panels; see the “Methods” section) across the entire dose range, which is converted to a *z*-score by dividing with the variance of the parental reference control (see the “Methods” section). **f** Gene interaction profiles were grouped by recursive expectation-maximization clustering (REMc) to reveal deletion-enhancing and deletion-suppressing doxorubicin-gene interaction modules and the influence of the Warburg effect. Resulting clusters were analyzed with GOTermFinder (GTF) to identify enriched biological functions. **g** Gene Ontology Term Averaging (GTA) was used as a complement to REMc/GTF. **h** The model for genetic buffering of doxorubicin cytotoxicity incorporates primary and interaction effects involving glycolysis (green), and respiration (red), to explain the influence of Warburg context (blue) on doxorubicin-gene interaction (black)

The interaction was calculated by the following steps:

1. Compute the average value of the 768 reference cultures (*R*_*i*_) at each dose (*D*_*i*_)
2. Assign *Y*_*i*_ max (defined above) if the growth curve is observed at *D*_0_, but not at *D*_*i*_, or if observed *Y*_*i*_ is greater than *Y*_*i*_ max
3. Calculate *K*_*i*_ = *Y*_*i*_–*R*_*i*_
4. Calculate *L*_*i*_ = *K*_*i*_–*K*_0_
5. Fit data by linear regression (least squares): *L*_*i*_ = *A* + B*D_*i*_
6. Compute the interaction value “INT” at the max dose: INT = *L*_*i*_ max = *A* + B*D_max_
7. Calculate the mean and standard deviation of interaction scores for reference strains, mean (REF_INT_) and SD (REF_INT_); mean (REF_INT_) is expected to be approximately zero, but SD (REF_INT_) is useful for standardizing against variance (Additional files 2, 3 and 4).
8. Calculate interaction *z*-scores (Fig. 1d):

$$ z-\mathrm{score}\ \left(\mathrm{YKO}/{\mathrm{KD}}_{\mathrm{INT}}\right)=\left(\mathrm{YKO}/{\mathrm{KD}}_{\mathrm{INT}}-\mathrm{mean}\ \left({\mathrm{REF}}_{\mathrm{INT}}\ \right)\right)/\mathrm{SD}\ \left({\mathrm{REF}}_{\mathrm{INT}}\right) $$

*z*-score (YKO/KD_INT_) > 2 for *L* or ≤ 2 for *K* are referred to as gene deletion enhancers of doxorubicin cytotoxicity, and conversely, *L* interaction score ≤ 2 or *K* interaction scores > 2 are considered gene deletion suppressors (Fig. 1e).

### Recursive expectation-maximization clustering (REMc) and heatmap generation

REMc is a probability-based clustering method and was performed as previously described [34]. Clusters obtained by Weka 3.5, an EM-optimized Gaussian mixture-clustering module, were subjected to hierarchical clustering in R (http://www.r-project.org/) to further aid visualization with heatmaps. REMc was performed using *L* and *K* interaction *z*-scores (Fig. 1f). REMc uses an expectation-maximization algorithm to define clusters probabilistically and is applied recursively to resolve gene interaction profile clusters. REMc terminates when a round of clustering reveals no new clusters. The cluster naming convention is “A-B.C.D-X”, where “*A*” = the round of clustering, “*B*” = 0, and “C.D-X” indicates the cluster pedigree. For example, 1-0-0 refers to the first cluster of the first round, 2-0.0-3 the fourth cluster derived from 1-0-0 (in round 2 of REMc), 3-0.0.3-1 indicates the second cluster derived from 2-0.0-3 (in round 3), and so on [34]. The main effect of the gene KO or KD on cell proliferation, i.e., *K*_*i*_ in the absence of doxorubicin (*D*_0_) is also referred to as “shift.” The shift was not subjected to REMc, but was included for hierarchical clustering and visualization by heatmaps after REMc. *K*_*i*_ is termed shift, because this value is subtracted from the data series for each YKO/KD to obtain *L*_*i*_ values, which are fit by linear regression for calculating drug-gene interaction. Additional file 5 contains REMc results in text files with associated data also displayed as heatmaps. In cases where a culture did not grow in the absence of drug, 0.0001 was assigned as the interaction score, and associated data were colored red (“NA”) in the shift columns of the heatmaps.

### Gene ontology term finder (GTF)

A python script was used to format REMc clusters for analysis with the command line version of the GO Term Finder (GTF) tool downloaded from http://search.cpan.org/dist/GO-TermFinder/ [35]. GTF reports on the enrichment of Gene Ontology (GO) terms by comparing the ratio of genes assigned to a term within a cluster to the respective ratio involving all genes tested. Additional file 5 contains GTF analysis of all REMc clusters. GO-enriched terms from REMc were investigated with respect to genes representing the term and literature underlying their annotations [36].

### Gene ontology term averaging (GTA)

In addition to using GTF to survey functional enrichment in REMc clusters, we developed GTA as a complementary workflow, using the GO information on SGD at https://downloads.yeastgenome.org/curation/literature/ to perform the following analysis:

1. Calculate the average and SD for interaction values of all genes in a GO term.
2. Filter results to obtain terms having GTA value greater than 2 or less than − 2.
3. Obtain GTA scores defined as |GTA value|—gtaSD; filter for GTA score > 2.

The GTA analysis is contained in Additional file 6 as tables and interactive plots created using the R *plotly* package https://CRAN.R-project.org/package=plotly. GTA results were analyzed primarily using the *L* interaction scores; however, GTA results with *K* interaction scores are included in Additional file 6: File D.

### Validation of doxorubicin-gene interaction

**Fig. 2:**
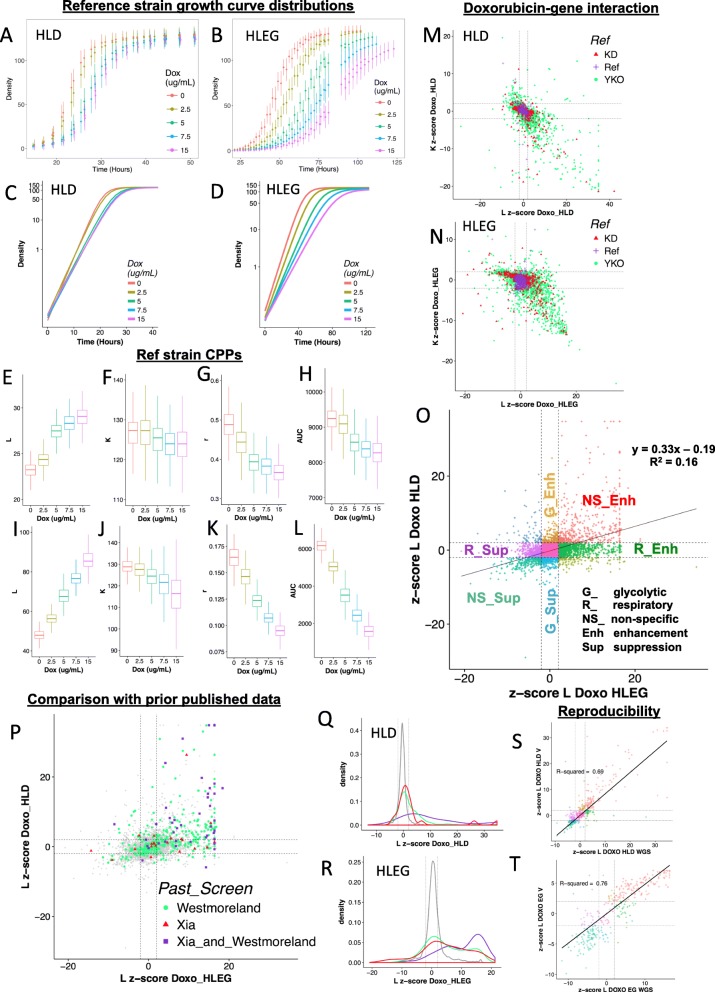
Q-HTCP provides cell proliferation parameters as phenotypes to quantify gene interaction. **a**, **b** Average pixel intensity and standard deviation for 768 reference strain cultures at indicated times after exposure to escalating doxorubicin concentrations in **a** HLD or **b** HLEG media. **c**, **d** Semi-log plots after fitting the data plotted above for **c** HLD or **d** HLEG to a logistic function (see Fig. 1d). **e**–**l** CPP distributions from data depicted in panels A-D for **e**–**h** HLD and **i**, **j** HLEG, including *L* (**e**, **i**), *K* (**f, j**), *r* (**g**, **k**), and (**h**, **l**) AUC. **m**, **n** Comparison of doxorubicin-gene interaction scores using the *L* vs*. K* CPP in the context of either **m** HLD or **n** HLEG media. Score distributions of knockout (YKO, green), knockdown/DAmP (YKD, red), and non-mutant parental (Ref, purple) strain cultures are indicated along with thresholds for deletion enhancement and suppression (dashed lines at ± 2). **o** Differential doxorubicin-gene interaction (using *L* as the CPP) for HLD vs. HLEG, classified with respect to Warburg metabolism as non-specific (NS), respiratory-specific (R), or glycolysis-specific (G) deletion enhancement (Enh) or deletion suppression (Sup). **p**–**r** Comparisons between genome-wide studies of doxorubicin-gene interaction: **p** Genes reported from Westmoreland et al. (green), Xia et al. (red), or both studies (purple) are plotted overlying *L* interaction scores (gray) in HLD vs*.* HLEG. **q**, **r** *L* interaction scores (gray) for genes reported by Westmoreland et al. (green), Xia et al. (red), or both studies (purple) in **q** HLD or **r** HLEG media. **s**, **t** Doxorubicin-gene interaction from whole-genome (WGS) and validation (V) studies on **s** HLD or **t** HLEG media

We retested 364 YKO/KD strains having human homologs in the P-POD database [37] and *L* interaction scores greater than 2 or less than − 2 in at least one media type. Strains were struck to obtain four single colonies and arranged on replicate 384-well plates along with 20 reference strain controls and reanalyzed by Q-HTCP on HLD and HLEG, as in the genome-wide experiment. Results are summarized in Fig. 2s, t, Additional file 2: Tables S5–S8, and Additional files 3 and 4: Files C-D.

### Prediction of human homologs that influence tumor response to doxorubicin

PharmacoDB reports on pharmacogenomics data from cancer cell lines, including transcriptomics and drug sensitivity [38]. The *PharmacoGx* R/Bioconductor package [39] was used to analyze the GDSC1000 (https://pharmacodb.pmgenomics.ca/datasets/5) and gCSI (https://pharmacodb.pmgenomics.ca/datasets/4) datasets, which contained transcriptomic and doxorubicin sensitivity results. A *p* value < 0.05 was used for differential gene expression and doxorubicin sensitivity. For gene expression, the sign of the standardized coefficient denotes increased (+) or decreased (−) expression. The *biomaRt* R package [40, 41] was used with the Ensembl database [42] to match yeast and human homologs from the phenomic and transcriptomic data, classifying yeast-human homology as one to one, one to many, and many to many.

## Results

### Phenomic characterization of doxorubicin response genes

The workflow for analyzing doxorubicin-gene interaction and differential buffering of doxorubicin with respect to the Warburg effect is summarized in Fig. 1. Alternately, in a respiratory or glycolytic (HLEG or HLD media, respectively) context (Fig. 1a), Q-HTCP technology was used for high throughput kinetic imaging of 384-culture cell arrays plated on agar media (Fig. 1b), image analysis (Fig. 1c), and growth curve fitting (Fig. 1d) to obtain the CPPs, *L* (time to reach half-carrying capacity), *K* (carrying capacity), and *r* (maximum specific rate) [28, 31, 33], which were used to measure doxorubicin-gene interaction across the entire YKO/KD library. The departure of the observed CPP from the expected doxorubicin response for each YKO/KD strain was derived using distributions from many replicate reference strain control cultures and summarized across all doxorubicin concentrations by linear regression (Fig. 1e). Interaction scores with absolute value greater than two were considered as gene *deletion enhancement* (*z*-score_L ≥ 2 or *z*-score_K ≤ − 2) or *deletion suppression* (*z*-score_L ≤ − 2 or *z*-score_K ≥ 2) of doxorubicin cytotoxicity. Gene deletion enhancement (e.g., *mms1-∆0*) and suppression (e.g., *vps54-∆0*) reveal functions that buffer or confer doxorubicin cytotoxicity, respectively. Doxorubicin-gene interaction profiles (selected if they contained *L* interaction scores with absolute value greater than 2, in either HLD or HLEG media) were analyzed by REMc and assessed for GO term enrichment (Fig. 1f)**.** As a complement to clustering gene interaction profiles, functional enrichment was analyzed by GTA (see the “Methods” section), systematically querying all GO processes, functions, and components (Fig. 1g and the “Methods” section) with respect to CPPs and Warburg status. Taken together, REMc and GTA reveal genetic modules that buffer doxorubicin, and how they are influenced by Warburg metabolism (Fig. 1h).

Doxorubicin cytotoxicity was greater in HLEG than HLD media, evidenced by the reference strain being more growth inhibited (Fig. 2a–l, Additional file 1: Figure S1). The “*L*” parameter was the most sensitive CPP, while *K* reported larger phenotypic effects (Fig. 2m, n) (Additional file 1: Figure S2). We noted a positive correlation between doxorubicin-gene interaction in HLEG and HLD; however, the interaction was media-specific and more abundant in the context of respiration, i.e., with HLEG media (Fig. 2o).

We compared our results with two prior studies of doxorubicin cytotoxicity in the yeast knockout collections [43, 44]. One study was conducted in SC media with the haploid (BY4741) YKO library and identified 71 deletion enhancers of cytotoxicity [43]. A second study reported on the homozygous diploid (BY4743) YKO collection in YPD media, identifying 376 enhancers [44]. Overlap between these studies and ours is shown in Fig. 2p–r and in Additional file 7: Table S9–10. While many genes overlapped between the studies, differing results were also observed, possibly attributable to strain background, media conditions, and methods for scoring interactions [27, 45]. To assess within-study reproducibility, we sub-cloned four colonies from glycerol stocks used in the first experiment and retested doxorubicin-gene interaction, revealing higher correlation and overall reproducibility within-study than between-study (Fig. 2s, t).

### Identification of functional gene interaction modules

**Fig. 3:**
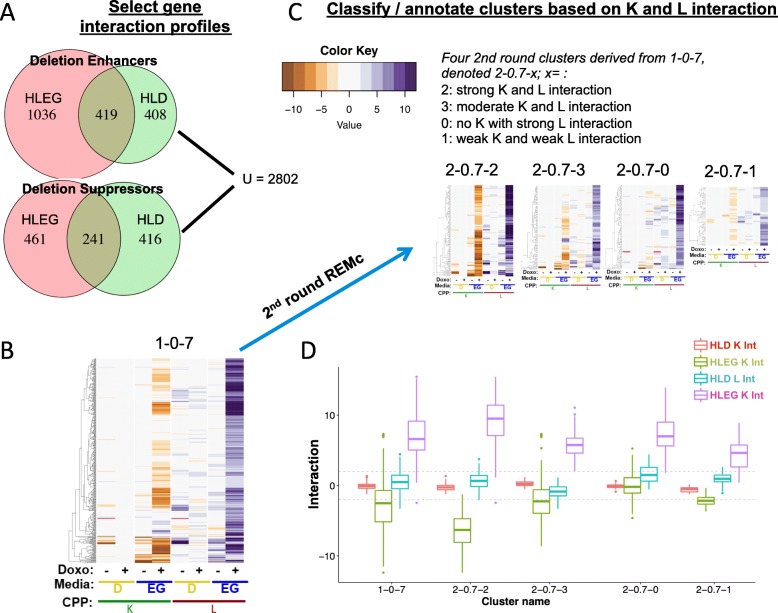
Characterization of Warburg-differential, doxorubicin-gene interaction profiles. **a** The union of enhancers (*L z*-score > 2) or suppressors (*L z*-score ≤ 2) from the HLD and HLEG analyses totaled 2802 gene interaction profiles that were subjected to REMc (see the “Methods” section). **b**, **c** The column order is the same for all heatmaps; “+” indicates doxorubicin-gene interaction and “−“ indicates “shift” (*K*_*0*_; see the “Methods” section). Interactions by *K* are negative (brown) if enhancing and positive (purple) if suppressing, while the signs of interaction are reversed for *L* (see the “Methods” section). The heatmap color scale is incremented by twos; red indicates no growth curve in the absence of doxorubicin. **b** First round cluster 1-0-7 has a gene interaction profile indicative of HLEG-specific deletion enhancement. **c** Second round clusters (2-0.7-X) are ordered left to right by strength of influence. **d** The pattern of distributions for the different doxorubicin-gene interaction scores (“+” columns only from panel **c**) summarizes respective clusters from panel **c**. Deletion enhancement is considered to be qualitatively stronger if observed for *K* in addition to *L*

Gene interaction profiles were analyzed by REMc (Fig. 3, Additional file 1: Figure S3), as described previously (see the “Methods” section) [34]. GO TermFinder [35] was used to associate enrichment of biological functions with particular patterns of doxorubicin-gene interaction identified by REMc (Fig. 3, Table 1, Additional file 1: Figure S3, and Additional file 5: File C). We expect that clustering by gene interaction profiles should, in general, increase GO enrichment [34]. While true overall, as evidenced by the first two rounds of REMc revealed distinctive profiles of gene interaction in respiratory vs. glycolytic media (Additional file 1: Figure S3), later round clusters only sometimes exhibited greater GO term enrichment as other times it was reduced by further clustering, highlighting the importance of reviewing the heatmaps and GTF files associated with each clustering round (see Additional file 8).

**Table 1:** GO Terms enriched in REMc clusters

Media	INT	GTA HLEG	GTA HLD	Clust	GO term name	*p* value	Genes
Resp	Enh	5.0	2.8	1-0-7	nucleosome organization	1.1E−07	VPS71 RSC2 SWR1 LDB7 HHF1 RSC4 IES1 ISW1 ARP6 RTT106 HIR3 SWC3 HPC2 YAF9 HIR1 HIR2 HTB1 NHP6A SWC5 NHP10
Resp	Enh	7.1	0.1	1-0-7	Set1C/COMPASS complex	5.5E−04	SPP1 SDC1 SWD1 SWD3 BRE2
Resp	Enh	3.9	− 0.6	1-0-7	histone methylation	4.1E−03	SPP1 SDC1 LGE1 NOP1 SWD3 HHF1 SWD1 BRE2
Resp	Enh	3.4	3.0	1-0-7	protein import into mitochondrial matrix	6.4E−03	MGR2 TOM7 YME1 TOM70 PAM17 TIM17 TIM23 TOM6
Resp	Enh	0.6	0.8	2-0.7-1	ER membrane protein complex	4.6E−06	EMC6 EMC4 EMC3 EMC5
Resp	Enh	4.6	0.2	2-0.7-2	Sin3-type complex	1.5E−05	RCO1 RXT2 SAP30 PHO23 DEP1 UME1
Resp	Enh	5.2	− 0.1	2-0.7-2	Rpd3L complex	7.1E−05	RXT2 SAP30 PHO23 DEP1 UME1
Resp	Enh	7.3	1.6	2-0.7-2	Swr1 complex	1.2E−06	SWC3 SWC5 VPS71 YAF9 SWR1 ARP6
Resp	Enh	5.9	2.1	2-0.7-2	histone exchange	5.7E−06	SWC3 SWC5 VPS71 YAF9 SWR1 ARP6
Resp	Enh	5.0	3.4	2-0.7-2	ATP-dependent chromatin remodeling	2.4E−04	SWC3 SWC5 VPS71 YAF9 SWR1 LDB7 ARP6
Resp	Enh	11.9	0.2	2-0.7-2	HIR complex	6.6E−06	HPC2 HIR1 HIR3 HIR2
Resp	Enh	11.4	3.2	2-0.7-2	DNA replication-independent nucleosome assembly	4.5E−04	HPC2 HIR1 HIR3 HIR2
Resp	Enh	11.0	1.7	1-0-8	respiratory chain complex III assembly	4.2E−02	QCR9 CBP4 FMP25
Resp	Enh	7.9	0.7	2-0.8-0	DNA topological change	2.6E−02	TOP3 MUS81
Resp	Enh	14.9	− 0.4	2-0.8-1	NatC complex	5.6E−03	MAK31 MAK3
Resp	Sup	− 2.6	− 1.5	2-0.3-1	regulation of fatty acid beta-oxidation	2.1E−02	ADR1 OAF1 PIP2
Resp	Sup	− 0.3	6.7	2-0.3-5	translation reinitiation	2.0E−02	TMA20 TIF34 TMA22
Glyc	Enh	1.1	0.5	2-0.2-2	ribonucleoprotein complex subunit organization	1.9E−02	RSA4 HBS1 BRR1 SDO1 RPS17A DHH1 CLF1 RRP7 TIF6 RPS14A RPS27B PRP9
Glyc	Sup	− 2.2	− 3.0	2-0.4-0	7-methylguanosine cap hypermethylation	5.6E−03	SWM2 TGS1
Glyc	Sup	1.5	− 0.4	2-0.4-2	mRNA 3'-end processing	8.6E−04	MPE1 CDC73 YSH1 KIN28 RNA14 NRD1
Glyc	Sup	1.3	0.9	2-0.4-2	mRNA cleavage	3.3E−02	MPE1 YSH1 POP8 RNA14
Glyc	Sup	− 0.8	− 2.9	2-0.4-2	meiotic chromosome condensation	3.4E−03	SMC2 YCG1 YCS4
Glyc	Sup	− 1.0	− 2.7	2-0.4-2	condensin complex	2.8E−03	SMC2 YCG1 YCS4
Both	Enh	2.9	2.3	1-0-6	cellular response to DNA damage stimulus	4.1E−08	CTK3 SIT4 RTT109 RVB1 RAD54 MMS22 CDC1 RAD55 PSF3 RAD50 BUD25 RAD51 MRE11 ARP8 ARP4 RAD57 TFB1 CDC7 RAD52 NPL6
Both	Enh	5.0	5.0	1-0-6	double-strand break repair via homologous recombination	2.9E−07	PSF3 RAD50 RAD51 MRE11 RAD54 MMS22 RAD57 CDC7 RAD52 RAD55
Both	Enh	7.7	9.7	1-0-6	double-strand break repair via synthesis-dependent strand annealing	4.3E−06	RAD54 RAD57 RAD51 RAD52 MRE11 RAD55
Both	Enh	9.2	5.0	2-0.6-1	ATP-dependent 3'-5' DNA helicase activity	1.9E−04	RVB1 ARP5 ARP8 ARP4
Both	Enh	9.0	2.5	2-0.6-1	Ino80 complex	2.1E−05	RVB1 IES6 ARP5 ARP8 ARP4
Both	Enh	3.4	1.6	2-0.6-1	histone acetylation	4.1E−02	RTT109 RVB1 NGG1 SPT20 ARP4
Resp	Enh	7.4	1.1	2-0.2-1	protein urmylation	1.1E−03	URM1 URE2 UBA4 ELP2
Both	Enh	9.9	3.9	2-0.2-1	Lst4-Lst7 complex	3.1E−02	LST7 LST4
Both	Sup	− 4.5	− 2.3	2-0.4-1	cellular sphingolipid homeostasis	9.6E−05	VPS53 VPS52 VPS54 VPS51
Both	Sup	− 12.2	− 7.0	2-0.4-1	fatty acid elongase activity	2.9E−02	ELO3 ELO2
Both	Sup	− 3.0	− 1.3	2-0.4-1	actin cortical patch localization	8.1E−03	RVS167 LSB3 RVS161 VRP1
Both	Sup	− 9.0	− 3.5	2-0.4-1	Rvs161p-Rvs167p complex	1.7E−02	RVS167 RVS161
Both	Sup	− 4.4	− 0.6	2-0.4-1	telomere tethering at nuclear periphery	1.8E−02	NUP60 MLP1 NUP120 NUP133

The table headers are defined as follows: For the column, “Media,” “Resp,” “Glyc,” and “Both” refer to whether the gene interaction type observed for the REMc cluster associated with the term was prominent in HLEG, HLD, or both media (see Additional file 1: Figure S3). For the column, “INT,” “Enh,” and “Sup” indicate deletion-enhancing or deletion-suppressing. The column “GTA” refers to GO term average. The column “Clust” refers to REMc ID

GTA score revealed 129 GO terms, 39 of which were found by REMc/GTF (Table 2 and Additional file 6: Files A–C). GTA identifies functions of smaller GO terms, e.g., protein complexes. GTA with *K* interaction scores yielded only 35 GO terms (Additional file 6: File D), with only 3 being unique from GTA with *L* interaction; thus, we focused on *L* interaction for GTA analysis. Interactive scatter plots (html files in which points contain embedded information) were used to visualize significant GO terms from both REMc and GTA (Additional file 6: File B). GO term-specific heatmaps further aided visualization of relationships between genes and the GO terms (see Figs. 5, 6, 7, 8, 9 and 10 and Additional file 9) by systematically displaying, for all genes attributed to a parent term and its children, uniformity vs. pleiotropy of interaction effects across different conditions.

**Table 2:** GO terms identified by GTA

GO term name	Media	INT	HLEG GTA	HLEG gtaSD	HLD GTA	HLD gtaSD	Genes	REMc related	*p* value
HIR complex	Resp	Enh	11.9	1.5	0.2	0.9	HIR1 HIR2 HPC2 HIR3	2-0.7-2	6.6E−06
histone monoubiquitination	Resp	Enh	11.4	7.0	0.1	1.2	RAD6 BRE1	NA	NA
Ino80 complex	Resp	Enh	9.0	6.8	2.5	7.7	RVB1 IES6 ARP5 ARP8 ARP4 ARP7 IES5 IES3 NHP10 IES2 IES1 RVB2 IES4 TAF14	3-0.6.1-1	1.5E−06
histone H4 acetylation	Resp	Enh	8.0	4.8	− 0.8	2.1	ESA1 NGG1 ELP4 EAF3 HAT1	NA	NA
mitochondrial respiratory chain complex III assembly	Resp	Enh	11.0	6.8	1.7	2.0	QCR7 CBP6 CBP4 BCS1 QCR9 FMP25 FMP36 CBP3	1-0-8	4.2E−02
mitochondrial respiratory chain supercomplex assembly	Resp	Enh	15.9	0.6	0.8	0.1	RCF1 COX13	1-0-8	7.0E−02
mitochondrial outer membrane translocase complex	Resp	Enh	9.1	6.4	0.7	3.1	TOM22 TOM5 TOM6 TOM70 TOM7 TOM40	NA	NA
protein urmylation	Resp	Enh	7.4	2.6	1.1	0.9	ELP2 URM1 NCS2 UBA4 ELP6 URE2	2-0.2-1	1.1E−03
Elongator holoenzyme complex	Resp	Enh	8.9	3.6	0.0	0.9	TUP1 IKI3 ELP4 ELP2 ELP3 IKI1 ELP6	3-0.7.2-0	1.4E−04
NatC complex	Resp	Enh	14.9	1.7	− 0.4	0.6	MAK31 MAK10 MAK3	2-0.8-1	5.6E−03
DNA topological change	Resp	Enh	7.9	5.7	0.7	2.6	RFA2 TOP3 MUS81 RMI1 TOP1 SGS1 RFA1 RAD4 TOP2	2-0.8-0	2.6E−02
tRNA (m1A) methyltransferase complex	Resp	Enh	17.0	0.8	9.3	17.4	GCD10 GCD14	NA	NA
MUB1-RAD6-UBR2 ubiquitin ligase complex	Resp	Enh	12.9	3.1	0.9	0.5	RAD6 MUB1 UBR2	NA	NA
malonyl-CoA biosynthetic process	Resp	Enh	11.1	7.4	1.5	0.1	HFA1 ACC1	NA	NA
pyridoxal 5'-phosphate salvage	Resp	Enh	11.1	8.7	1.5	5.3	PDX3 BUD16 BUD17	NA	NA
maintenance of transcriptional fidelity during DNA-templated transcription elongation from RNA polymerase II promoter	Resp	Enh	11.1	7.5	− 0.4	4.2	RPB9 DST1	NA	NA
RNA polymerase II transcription corepressor activity	Resp	Enh	11.0	7.6	2.2	1.7	SIN3 MED8 SRB7	NA	NA
pyruvate dehydrogenase activity	Resp	Enh	10.6	6.4	2.8	0.9	PDA1 LPD1 PDB1	NA	NA
eukaryotic translation initiation factor 2 complex	Resp	Enh	10.3	4.7	8.2	8.7	SUI2 GCD11	NA	NA
L-aspartate:2-oxoglutarate aminotransferase activity	Resp	Sup	− 3.9	0.5	− 0.9	0.8	AAT2 AAT1	2-0.4-3	5.9E−04
nuclear pore outer ring	Resp	Sup	− 6.3	3.7	1.4	7.6	NUP145 SEH1 NUP84 NUP120 NUP133	3-0.4.1-0	9.7E−02
positive regulation of fatty acid beta-oxidation	Resp	Sup	− 2.6	0.5	− 1.5	0.2	OAF1 ADR1 PIP2	2-0.3-1	2.1E−02
EKC/KEOPS complex	Resp	Sup	− 7.9	4.6	− 1.8	1.1	KAE1 CGI121 GON7 BUD32	NA	NA
spermine biosynthetic process	Resp	Sup	− 2.6	0.3	− 0.3	0.7	SPE4 SPE2	NA	NA
Dom34-Hbs1 complex	Glyc	Enh	0.3	2.1	2.7	0.3	HBS1 DOM34	NA	NA
Ubp3-Bre5 deubiquitination complex	Glyc	Enh	− 1.1	3.2	8.8	2.2	BRE5 UBP3	NA	NA
Cul4-RING E3 ubiquitin ligase complex	Glyc	Enh	1.8	4.1	4.6	2.3	HRT1 PRP46 SOF1	NA	NA
dTTP biosynthetic process	Glyc	Enh	− 1.3	3.2	7.0	0.6	CDC21 CDC8	NA	NA
GDP-mannose transport	Glyc	Enh	1.5	1.9	9.5	5.6	VRG4 HVG1	NA	NA
7-methylguanosine cap hypermethylation	Glyc	Sup	− 2.2	1.5	− 3.0	0.9	SWM2 TGS1	2-0.4-0	5.6E−03
meiotic chromosome condensation	Glyc	Sup	− 0.8	0.9	− 2.9	0.9	SMC2 YCG1 SMC4 YCS4	2-0.4-2	3.4E−03
histone deubiquitination	Glyc	Sup	1.8	1.9	− 3.4	1.1	SEM1 UBP8 SGF73 SGF11	NA	NA
HDA1 complex	Both	Enh	8.9	0.3	4.0	1.1	HDA2 HDA1 HDA3	NA	NA
CTDK-1 complex	Both	Enh	15.6	0.7	3.8	0.9	CTK2 CTK3 CTK1	1-0-8	5.3E−02
Cul8-RING ubiquitin ligase complex	Both	Enh	9.1	4.5	6.1	1.1	MMS22 MMS1 RTT101 HRT1 RTT107	1-0-2	1.0E−01
Lst4-Lst7 complex	Both	Enh	9.9	1.4	3.9	0.3	LST7 LST4	2-0.2-1	3.1E−02
MCM complex	Both	Enh	4.2	1.4	4.9	2.6	MCM7 MCM6 MCM5 MCM2 MCM3	NA	NA
histone H3-K56 acetylation	Both	Enh	10.3	7.0	8.6	4.3	RTT109 SPT10	NA	NA
fatty acid elongase activity	Both	Sup	− 12.2	2.4	− 7.0	1.1	SUR4 FEN1	2-0.4-1	2.9E−02
GARP complex	Both	Sup	− 6.8	0.9	− 3.5	0.8	VPS53 VPS54 VPS52 VPS51	3-0.4.1-0	6.9E−07
nuclear cap binding complex	Both	Sup	− 4.7	0.5	− 3.4	0.5	STO1 CBC2	3-0.4.1-0	9.9E−03
Rvs161p-Rvs167p complex	Both	Sup	− 9.0	0.4	− 3.5	0.2	RVS167 RVS161	3-0.4.1-0	9.9E−03

The table headers are defined as follows: “gtaSD” refers to the standard deviation of GTA, “REMc related” refers to an REMc cluster ID if GTA-identified term was also found by REMc/GTF, and “*p* value” reports results from REMc/GTF. See Table 1 for other header definitions

**Fig. 4:**
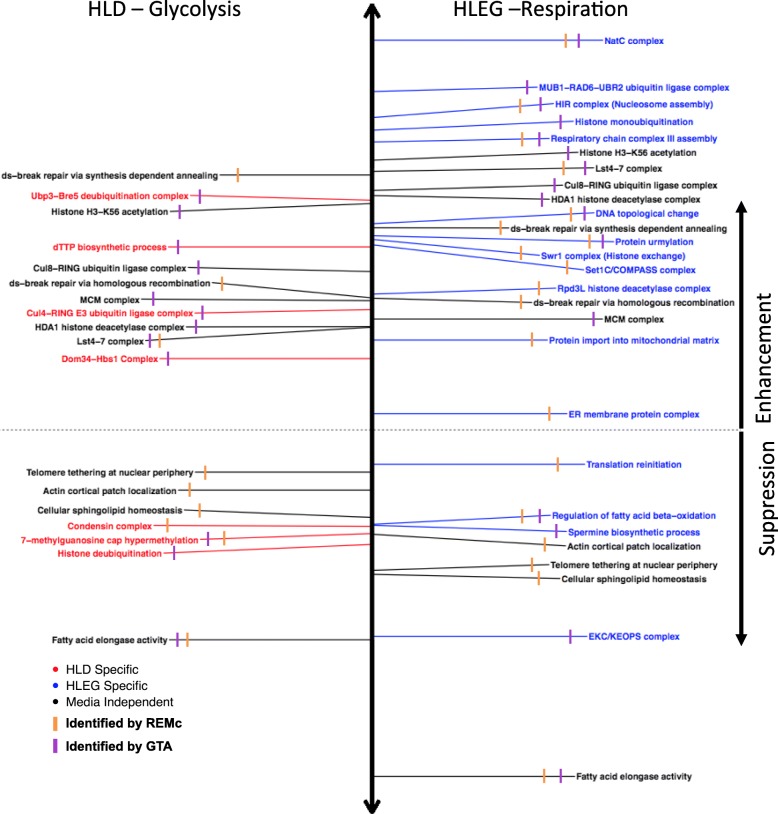
GO annotations associated with deletion enhancement or suppression of doxorubicin cytotoxicity, with respect to Warburg-dependence. Representative GO terms are listed, which were identified by REMc/GTF (orange), GTA (purple), or both methods, for HLD (left, red), HLEG (right, blue), or both media types (black), and for enhancement (above dashed line) or suppression (below dashed line) of doxorubicin cytotoxicity. Distance above or below the horizontal dashed line indicates the GTA value for terms identified by REMc or the GTA score if identified by GTA (see the “Methods” section). See Additional files 5 and 6, respectively, for all REMc and GTA results

In summary, we used REMc, GTA, and GO term-specific scatterplots and heatmaps to discover genetic modules that alternatively buffer (i.e., deletion enhancing) or confer (i.e., deletion suppressing) doxorubicin cytotoxicity and to determine whether the Warburg-transition exerts influence on their effects (Fig. 4).

### Warburg transition-dependent doxorubicin gene interaction modules

Despite both longstanding and renewed interest in the importance of the Warburg effect to oncogenesis, whether it influences cellular responses to chemotherapeutic agents is unknown. Thus, YKO/KD strains that display differential resistance to doxorubicin under respiratory (non-fermentable HLEG media) vs. glycolytic (fermentable HLD media) media provide new insight both into genes that function in pathways that may buffer or promote doxorubicin cytotoxicity and whether such pathways are potentially influenced by the Warburg transition. The phenomic assessments described below systematically quantify the contribution of each and every individual yeast gene to doxorubicin cell proliferation phenotypes. In addition, the influence of the Warburg effect on this network is detailed by differential doxorubicin-gene interaction on glycolytic (HLD) vs. respiratory (HLEG) media. In addition to direct implication of cellular pathways by the identification of genes annotated to their functions, functional enrichment among all genes was ascertained by GO term enrichment in gene clusters having similar gene-doxorubicin interaction profiles (REMc/GTF) or by systematic analysis of the average gene interaction value in Gene Ontology terms (GTA).

### Respiration-specific gene deletion enhancement

**Fig. 5:**
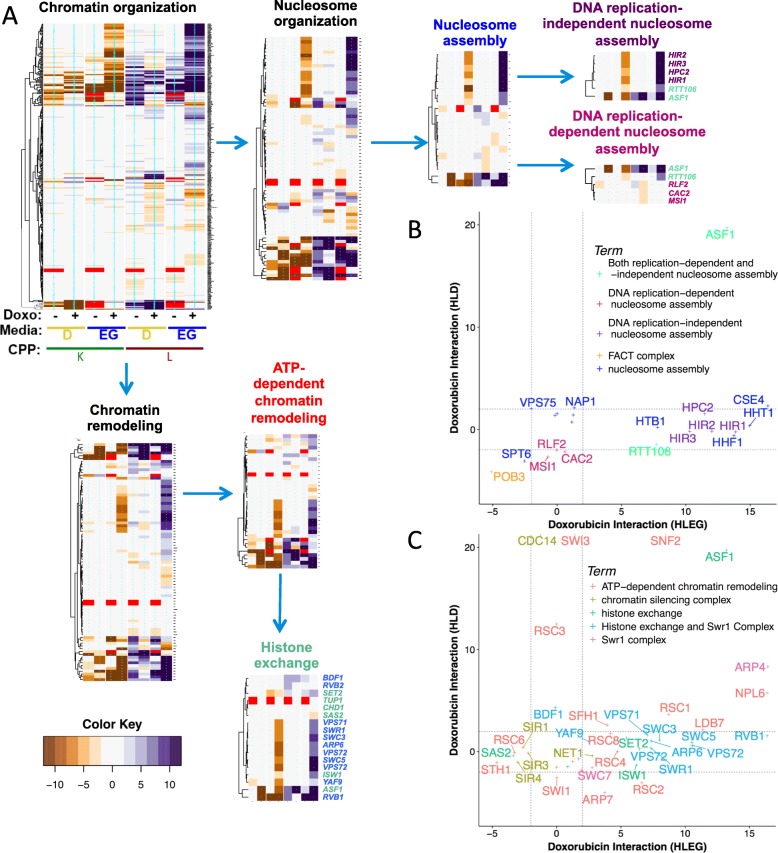
Respiration increases the role for chromatin organization in buffering doxorubicin toxicity. **a** GO term-specific heatmaps for chromatin organization and its child terms (indicated by arrows) clarify related but distinct biological functions that buffer doxorubicin, with respect to Warburg status. **b**, **c** *L*-based doxorubicin-gene interaction scores associated with GO terms that were enriched in cluster 2-0.7-2. Dashed lines indicate *z*-score thresholds for enhancers (> 2) and suppressors (≤ 2). Sub-threshold gene interaction values are plotted, but not labeled

**Fig. 6:**
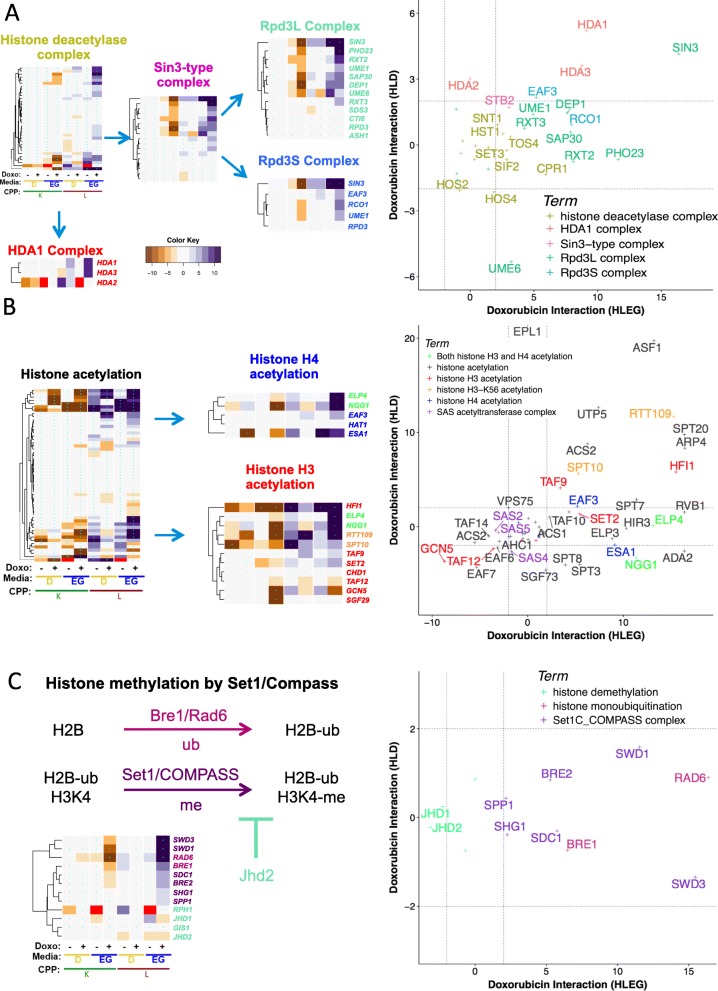
Distinct histone modifications differentially influence doxorubicin cytotoxicity. **a** Rpd3L and Rpd3S complexes exert strong HLEG-specific doxorubicin-enhancing influence relative to other Sin3-type histone deacetylases and the HDA1 complex. **b** In contrast to histone deacetylation (panel **a**), histone acetylation exhibits deletion enhancement that is Warburg-independent. **c** Histone H3K4 methylation by the Set1C/COMPASS complex, which requires histone mono-ubiquitination of H2B by the Bre1/Rad6 complex, is opposed by Jhd2, a histone H3K4 demethylase. The respiration-specific deletion-enhancing interactions suggest the Warburg transition can protect tumors promoted by certain types of chromatin deregulation from doxorubicin

**Fig. 7:**
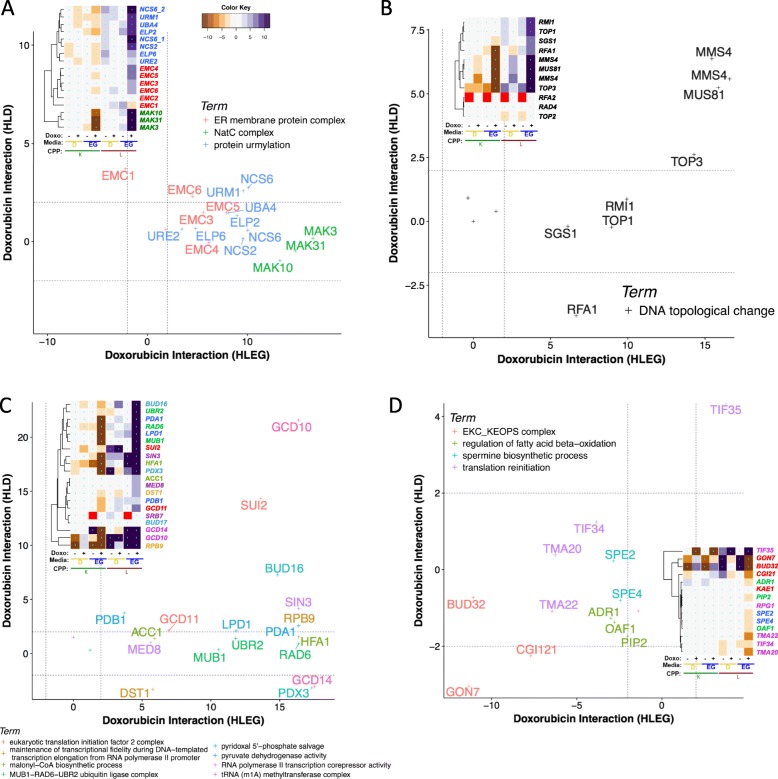
Additional respiration-specific deletion-enhancing and deletion-suppressing functions that influence doxorubicin cytotoxicity. Heatmaps depicting complete phenotypic profiles are the inset, corresponding to the plots of *L*-based doxorubicin-gene interaction. **a** Protein folding in endoplasmic reticulum and the N-terminal protein-acetylating NatC complex are largely respiratory-dependent in their deletion-enhancing influence. **b** DNA topological change exerts deletion-enhancing interactions in both respiratory and glycolytic contexts. **c** GTA-identified terms tend to be smaller in number and display greater variability in the Warburg dependence among genes sharing the same functional annotation. **d** Functions implicated in respiratory-dependent deletion suppression of doxorubicin toxicity

Respiration-specific deletion-enhancing clusters (see Additional file 1: Figure S3: 1-0-7 and 1-0-8) revealed GO term enrichment for *histone modification and chromatin organization*, *respiratory chain complex III assembly*, *protein import into mitochondria, protein urmylation*, the *NatC complex*, *protein folding in endoplasmic reticulum*, *and DNA topological change* (Figs. 5, 6, and 7; Additional file 5: File C). Additional modules were identified using GTA (Fig. 7c and Additional file 6: File A).

### Chromatin organization and histone modification

REMc/GTF and GTA identified several chromatin-related processes that buffer doxorubicin toxicity in a respiration-specific manner, including *DNA replication-independent nucleosome assembly*, *histone exchange*, *histone deacetylation*, and *histone methylation* (Figs. 5 and 6).

#### (i) DNA replication-independent nucleosome assembly (HIR complex)

REMc/GTF identified the HIR complex (*HIR1-3* and *HPC2*), which functions as a histone chaperone in chromatin assembly and disassembly, in cluster 2-0.7-2 (Additional file 1: Figure S3 and Table 1) [46]. Along with Asf1 and Rtt106, the HIR complex is involved in DNA replication-independent (i.e., RNA transcriptional) histone deposition and regulates transcription of three of the four histone genes [46–48]. Furthermore, genes encoding for *HTA1/HTB1*, *HHT1/2*, and *HHF1/2* were also respiratory-specific deletion enhancers. Asf1 and Rtt106 function in nucleosome assembly in both DNA replication and DNA replication-independent contexts. Asf1, which functions in the Rad53-dependent DNA damage response [49], enhanced doxorubicin toxicity in both respiratory and glycolytic media, like other DNA repair genes (see below). In further contrast, genes associated with *replication-dependent nucleosome assembly* (*RLF2*, *CAC2*, *MSI1*) by the chromatin assembly factor complex, CAF-1, [50] were HLD-specific suppressors (Fig. 5a, b).

Prior studies have reported enhanced doxorubicin cytotoxicity due to nucleosome disassembly and “chromatin trapping” by the FACT complex, referring to binding and resulting damage to disassembled chromatin in the context of doxorubicin exposure [20]. *POB3-DAmP*, the only member of the FACT complex represented in the YKO/KD library, resulted in suppression of doxorubicin cytotoxicity (Fig. 5b), presumably by suppressing its effect of trapping and damaging disassembled chromatin.

#### (ii) Histone exchange (Swr1 complex)

The Swr1 complex (enriched in cluster 2-0.7-2) uses ATP hydrolysis to replace the H2A nucleosome with the H2AZ variant [51]. Swr1 complex genes showing respiration-specific buffering of doxorubicin toxicity included *RVB1*, *SWC3*, *SWC5*, *ARP6*, *SWR1*, *VPS71*, and *VPS72* (Fig. 5c). Accordingly, the H2AZ variant, Htz1, which is enriched at most gene promoters in euchromatin [52–54], was also an HLEG-specific deletion enhancer. The Swr1 complex is recruited for repair of dsDNA breaks, where the H2AZ variant is incorporated [55]; however, the interaction profile of the Swr1 complex more closely resembles other respiratory specific enhancers involved in transcriptional regulation, whereas dsDNA-break repair by homologous recombination buffered doxorubicin toxicity independent of Warburg context (see cluster 1-0-6 from Additional file 1: Figure S3 and Table 1). The Swr1 complex can also inhibit subtelomeric spread of heterochromatin by impeding SIR-dependent silencing [56]. Consistent with knockout of Swr1 promoting silencing and having a deletion-enhancing effect, deletion of *SIR1*, *SIR3*, or *SIR4* (which disrupts chromatin silencing) also exerted respiratory-specific suppression of doxorubicin toxicity (Fig. 5c).

#### (iii) Histone deacetylation (Sin3-type and HDA1 complexes)

Deletion of genes functioning in the Rpd3L and Rpd3S histone deacetylase complexes (HDAC) was associated with strong respiratory enhancement of doxorubicin toxicity (cluster 2-0.7-2, Fig. 6a); however, genes constituting the Hda1 complex exerted weaker effects, but in both respiratory and glycolytic media (Fig. 6a and Table 2). The yeast Rpd3 deacetylase histone complexes are homologous to mammalian class I Rpd3-like proteins (Hdac1-3,8), while the yeast Hda1 complex is homologous to mammalian class II Hda1-like proteins (Hdac4-5,7,9) [57]. Hda1 and Rpd3 complexes both deacetylate histones H3 and H4; however, deletion of *RPD*3 vs*. HDA1* revealed different degrees of H4 lysine 5 and K12 hyperacetylation [58], implicating this functional distinction in Warburg-differential doxorubicin response.

Histone acetylation was GO-enriched in cluster 2-0.6-1, which displayed a Warburg-independent gene interaction profile (Additional file 1: Figure S3 and Table 1). GTA analysis confirmed H3K56 acetylation (*SPT10* and *RTT109*) and histone H3 acetylation (*TAF9* and *HFI1*) as media-independent, but also histone H4 acetylation (*EAF3*, *ESA1*, *NGG1*, and *ELP4*), which was relatively respiratory-specific in its deletion enhancement (Fig. 6b and Table 2). Rtt109 promotes H3K56 acetylation, which is associated with elongating RNA polymerase II [59], and can be persistent in the setting of DNA damage [60]. Warburg-independent deletion enhancement suggests its role in DNA repair becomes invoked.

The SAS acetyltransferase complex was deletion suppressing; *SAS2* and *SAS5* were HLEG-specific, and *SAS4* was HLD-specific (Fig. 6b). The Sas2 acetyltransferase complex creates a barrier against spread of heterochromatin at telomeres by opposing Sir protein deacetylation via effects on histone H4K16 [61]. The deacetylating SIR proteins (*SIR1*, *SIR3*, *SIR4*) were also HLEG-specific suppressors (Fig. 5c), suggesting dynamic regulation of telomeric histones (not simply acetylation or deacetylation), or perhaps a function of Sas2 acetyltransferase that is independent of SIR protein functions, confers doxorubicin cytotoxicity in respiring cells.

#### (iv) Histone methylation (Set1C/COMPASS complex)

Histone methylation differentially influences gene transcription, depending on the histone residues modified and the number of methyl groups added [62]. The Set1C/COMPASS complex, which catalyzes mono-, di-, and tri-methylation of H3K4 [63–66], was enriched in cluster 1-0-7 (Additional file 1: Figure S3 and Table 1). All genes tested from the Set1C/COMPASS complex (*SPP1*, *SDS1*, *SWD1*, *SWD3*, *BRE2*, *SHG1*; *SET1* not in YKO/KD) were EG-specific deletion enhancers (Fig. 6c). The Set1C/COMPASS complex and H3K4 trimethylation localize at transcription start sites of actively transcribed genes [67, 68]. Furthermore, the Rad6-Bre1 complex, which mono-ubiquitinates histone H2B before Set1C/COMPASS methylates histone H3K4 [69–71], shared the same interaction profile, cross-implicating the Set1C/COMPASS and Rad6-Bre1 functions (Fig. 6c). The Rad6-Bre1 complex is additionally involved in the DNA damage response checkpoint to activate Rad53 [72]; however, its HLEG-specific enhancing profile was more closely shared with transcriptional regulation modules, indicating its latter role is better related. *JHD1* and *JHD2* are JmjC domain family histone demethylases that act on H3-K36 and H3-K4 respectively, and their deletion suppression interactions are further evidence that histone methylation contributes to buffering doxorubicin cytotoxicity, especially in a respiratory context (Fig. 6c).

Based on the findings above, it appears buffering of doxorubicin-mediated cellular toxicity by some transcription-associated chromatin regulators is alleviated by the transition from respiratory to glycolytic metabolism, whereas buffering by those more associated with DNA repair is relatively independent of metabolic context.

### Mitochondrial functions

The greater number of deletion-enhancing doxorubicin-gene interactions in HLEG media, relative to HLD media (Fig. 2o), caused us to examine genes annotated to mitochondrial function more systematically. Many mitochondrial gene deletion strains grew very poorly on HLEG media and exhibited reduced carrying capacity on HLD media, as would be associated with petite mutants. Completely respiratory-deficient mutants clustered together in 1-0-0; however, many mitochondrial mutants maintained some or all respiratory capacity. For example, the *respiratory chain complex III assembly* and *protein import into mitochondrial matrix* terms were enriched in deletion-enhancing clusters, 1-0-7 and 1-0-8 (Table 1 and Additional file 1: Figures S3-4). Some of these strains appeared respiratory sufficient, yet the genes were required to buffer doxorubicin cytotoxicity under respiratory conditions. For example, evolutionarily conserved genes functioning in complex IV assembly (*RCF1/YML030W* and *COA6*) reached carrying capacity on HLEG media, yet exerted strong deletion enhancement of doxorubicin growth inhibition (Additional file 1: Figure S4A). In contrast, HLEG-specific deletion-enhancing complex IV assembly components (*COA2*, *CMC1*, *RCF2*) and complex III assembly genes (*FMP25*, *FMP36*, *QCR9*, *CBP4*) were either not conserved in humans or exhibited strong respiratory defects (in absence of doxorubicin) (Additional file 1: Figure S4A-B). These findings appear to establish relevance of the yeast model to studies in cardiomyocytes, for which it was reported that doxorubicin toxicity is exacerbated by depletion of cytochrome c or cardiolipin, leading to reduced workload capacity, and accelerated aging [73, 74]. Likewise, functionally conserved (*TOM70*, *TIM10*, *TIM17*, *TIM23*, and *MGR2*) and yeast-specific (*TOM6* and *TOM7*) genes in *protein import into mitochondrial matrix* buffered doxorubicin cytotoxicity (Additional file 1: Figure S4C-E), perhaps relating to increased oxidative stress [75], which also enhances doxorubicin toxicity in mammalian cells [8, 11].

Systematic examination of the GO annotation, *mitochondrion* (Additional file 1: Figure S5), revealed several additional respiratory-competent gene-deletion strains exhibiting HLEG-specific enhancing interactions. *COX13* encodes subunit VIa of cytochrome c oxidase, which functions with Rcf1 in the formation of respirasomes (also called “supercomplexes”) [76, 77]. Others included *COX8*, encoding subunit VIII of cytochrome c oxidase [78]; *MPC1*, encoding a mitochondrial pyruvate carrier [79, 80]; *MME1*, encoding an inner mitochondrial membrane magnesium exporter [81]; *OMS1*, an inner membrane protein predicted to have methyltransferase activity [82]*; GUF1*, a matrix-localized GTPase that binds mitochondrial ribosomes and influences cytochrome oxidase assembly [83]*;* and *MIC10 (YCL057C-A)*, encoding a component of the MICOS complex, functioning in inner membrane organization and membrane contact site formation [84].

### Protein folding, localization, and modification pathways

Protein biogenesis and modification pathways enriched in HLEG-specific enhancement clusters included the *endoplasmic reticulum membrane complex* (EMC) (2-0.7-1), *protein urmylation* (2-0.2-1), and N-terminal acetylation by the *NatC complex* (2-0.8-1) (Additional file 1: Figure S3 and Table 1).

#### (i) Protein folding in endoplasmic reticulum (ER membrane protein complex)

The ER membrane complex (*EMC1-6*, Fig. 7a) functions in protein folding in the ER [85] and together with the ER-mitochondria encounter structure (ERMES), the EMC enables ER-mitochondria phosphatidylserine transfer and tethering [86]. The EMC physically interacts with the mitochondrial translocase of the outer membrane (e.g., *TOM5*, *6*, *7*, *22*, *70*; described above) for the process of ER-mitochondria phosphatidylserine transfer [86]. The shared respiratory-specific, deletion-enhancing profiles suggest cooperative functions of the EMC and mitochondrial outer membrane translocase (Additional file 1: Figure S4D) in buffering doxorubicin cytotoxicity. In contrast to the EMC, genes involved in the ERMES complex (1-0-0; Additional file 5: File B-C) were essential for respiration, and thus, their influence on doxorubicin cytotoxicity could not be addressed with knockout mutants in HLEG media.

#### (ii) Protein urmylation, Elongator complex, and tRNA wobble uridine thiolation

*ELP2*, *UBA4*, *URM1*, and *URE2* clustered together in 2-0.2-1, constituting GO-enrichment in protein urmylation, the covalent modification of lysine residues with the ubiquitin-related modifier, Urm1 [87]. Other protein urmylation genes, *ELP6*, *NCS2,* and *NCS6/YGL211W,* displayed similar interaction profiles and clustered together in 1-0-7 (Fig. 7a). *ELP2* and *ELP6* also function in the Elongator holoenzyme complex (*IKI1, IKI3, ELP2, ELP3, ELP4,* and *ELP6*), associated with similar interaction profiles (Additional file 1: Figure S6). *URM1*, *UBA4, NCS2,* and *NCS6* further function in tRNA wobble position uridine thiolation, where Urm1 functions as a sulfur carrier [88–90]. Genes uniquely annotated to these terms (*IKI1, IKI3, ELP3, ELP4, TUM1, URE2*) also displayed related profiles (Additional file 1: Figure S6). Thus, protein urmylation, Elongator complex function, and tRNA wobble thiolation appear to be distinct modules, comprised of shared genes, buffering doxorubicin specifically in a respiratory context.

#### (iii) N-terminal acetylation by the NatC complex

The NatC complex (Mak3, Mak10, and Mak31) specifically acetylates methionine-starting hydrophobic N-terminal proteins (Met-Leu, Met-Phe, Met-Ile, Met-Tyr) [91], neutralizing positive charge on the alpha-amino group, and impeding turnover by ubiquitination or other modifications [92]. N-acetylation occurs on around half of the soluble yeast proteome and over 80% in humans [93]. NatC-mediated N-terminal acetylation facilitates Golgi or inner nuclear membrane localization of some [94–97], but not most proteins [98]. The three genes encoding the NatC complex clustered together (Fig. 7a); however, NatC substrates were not enriched among doxorubicin-gene interactions (Additional file 7: Table S11). Perhaps a select few NatC targets or a novel function for NatC underlies its compensatory effects.

### DNA topological change

DNA topological change, which refers to remodeling the turns of a double stranded DNA helix, was enriched in cluster 2-0.8-0 (Additional file 1: Figure S3 and Table 1). Representative genes were *SGS1*, *TOP1*, *RFA1*, *RMI1*, *TOP3*, *MMS4*, and *MUS81* (Fig. 7b). Types I and II topoisomerases resolve supercoiling during replication and transcription [99, 100]. Top1 is a type IB topoisomerase, which relaxes positive and negative supercoils [101, 102], compared to Top3, a type IA topoisomerase that specifically acts on negative supercoiling [103]. The Mms4-Mus81 endonuclease has overlapping functions with Top3 and Sgs1 in DNA repair [104]; however, their respective influences on doxorubicin toxicity were quantitatively distinct in both respiratory and glycolytic contexts, with a greater requirement for the *MMS4/MUS81* than *SGS1*, *TOP3*, *RFA1*, and *RMI1* (Fig. 7b); the latter four, functioning together for decatenation and unknotting of dsDNA [105].

### GTA reveals additional biological functions that buffer doxorubicin toxicity

GTA is a method complementary to REMc/GTF for discovering GO functions in Q-HTCP-derived phenomic data. Whereas GTF scores GO enrichment among genes within a cluster, GTA is independent of clustering and systematically assesses all genes in every GO term for interaction (see the “Methods” section).

GTA revealed 71 respiratory-specific deletion-enhancing GO terms, 24 of which were also found by REMc/GTF (see Additional file 6: File A). Strong enhancing terms (GTA value > 10) with functions relatively distinct from those identified above by REMc were *tRNA (m1A) methyltransferase complex*, *MUB1-RAD6-UBR2 ubiquitin ligase complex*, *malonyl-CoA biosynthetic process*, *pyridoxal 5'-phosphate salvage*, *maintenance of transcriptional fidelity during DNA-templated transcription elongation from RNA polymerase II promoter*, *RNA polymerase II transcription corepressor activity*, *pyruvate dehydrogenase activity*, and *eukaryotic translation initiation factor 2 complex* (Fig. 7c). Most terms identified by GTA consisted of 2–3 genes and did not necessarily cluster together by REMc.

### Respiration-specific gene deletion suppression of doxorubicin cytotoxicity

Clusters exhibiting respiration-specific gene deletion suppression revealed GO term enrichment for *regulation of fatty acid beta-oxidation (*cluster 2-0.3-1) and *translation reinitiation* (cluster 2-0.3-5) (Additional file 1: Figure S3 and Table 1). By GTA analysis, the *EKC/KEOPS complex* and *spermine biosynthetic process* were additionally found to confer HLEG-specific deletion suppression (Fig. 7d and Table 2).

### Regulation of fatty acid beta-oxidation

*ADR1*, *OAF1*, and *PIP2* were grouped together in cluster 2-0.3-1 (Additional file 1: Figure S3 and Table 1), displaying HLEG-specific gene deletion suppression (Fig. 7d). The Pip2-Oaf1 complex binds to oleate response elements and, along with *ADR1*, regulates transcription of peroxisomal genes [106, 107]. Doxorubicin inhibits beta-oxidation of long-chain fatty acids in cardiac tissues, which is reversed by supplementing with propionyl-l-carnitine, and alleviates effects of doxorubicin cardiotoxicity [108]. Thus, the yeast model may be informative for investigating related gene networks in greater depth.

### Translation reinitiation

In the respiratory-specific deletion suppressing cluster 2-0.3-5 (Additional file 1: Figure S3), *TMA20*, *TMA22*, and *TIF34* represented enrichment for translation reinitiation, which is necessary after termination of short upstream open reading frames (uORFs) [109] (Fig. 7d). Some uORFs function in translational regulation of a downstream protein; for example, *GCN4* expression is regulated in response to amino acid starvation [109]. However, using the Welsh two sample *t* test, we found no significant difference in means of interaction scores between the distribution of proteins regulated or not by uORFs [110] (*p* value = 0.8357) (Additional file 7: Table S12).

### Spermine biosynthetic process

Loss of spermine biosynthesis, specifically *SPE2* (*S*-adenosylmethionine decarboxylase) and *SPE4* (spermine synthase)*,* suppressed doxorubicin toxicity in HLEG media (Fig. 7d). The pathways of polyamine metabolism and their physiologic effects on cancer are complex [111, 112], and although our data suggest spermine metabolism contributes to doxorubicin cytotoxicity, how this occurs mechanistically and specifically in respiring cells awaits further study [113].

### EKC/KEOPS complex

GTA revealed the EKC/KEOPS complex (*CGI121, GON7,* and *BUD32*) as HLEG-specific deletion suppressing (Fig. 7d). The EKC/KEOPS complex is involved in threonyl carbamoyl adenosine (t6A) tRNA modification [114], which strengthens the A-U codon–anticodon interaction [115]. EKC/KEOPS has also been characterized with respect to telomere maintenance [116] and transcription [117]. Deletion of *GON7, BUD32,* or to a lesser extent, *CGI121,* inhibited cell proliferation in the absence of doxorubicin treatment, indicating that translational and/or transcriptional activity of the EKC/KEOPS complex function contributes to doxorubicin sensitivity.

### Glycolysis-specific gene deletion enhancement of doxorubicin cytotoxicity

HLD-specific deletion enhancement of doxorubicin cytotoxicity could represent lethal vulnerabilities that emerge when a tumor undergoes the Warburg transition. In this regard, several genes, but few enriched GO terms, were identified by REMc (Additional file 1: Figure S3, clusters 1-0-5, 2-0.3-0, and 2-0.2-2; Additional file 5: File A). *Ribonucleoprotein complex subunit organization* was suggested (Table 1); however, the term-specific heatmap revealed doxorubicin-gene interaction within this cellular process to be pleiotropic (Additional file 1: Figure S7).

### Glycolysis-specific deletion-enhancing terms identified by GTA

**Fig. 8:**
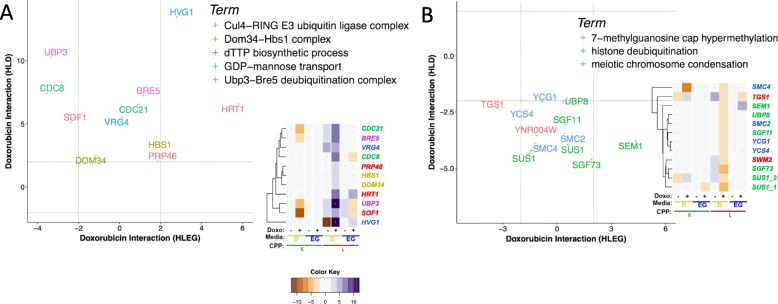
Glycolysis-specific enhancement and suppression of doxorubicin cytotoxicity. Doxorubicin-gene interaction profiles for HLD-specific GO terms identified by GTA are depicted for **a** deletion enhancement and **b** deletion suppression

GTA analysis revealed HLD-specific deletion-enhancing genes encoding the Cul4-RING E3 ubiquitin ligase, the Dom34-Hbs1 complex, and the Ubp3-Bre5 deubiquitinase. *GDP-Mannose Transport* and *dTTP biosynthesis* were also revealed (Fig. 8a and Additional file 6: File A). *SOF1, HRT1,* and *PRP46* were computationally inferred to form the Cul4-RING E3 ubiquitin ligase complex [118]. Yeast Sof1 is an essential protein that is required for 40s ribosomal biogenesis, and overexpression of its human ortholog, *DCAF13/WDSOF1*, is associated with aggressive tumors and poorer survival in hepatocellular carcinoma [119]. *DOM34/PELO* and *HBS1/HBS1L* facilitate recycling of stalled ribosomes by promoting dissociation of large and small subunits through a process called no-go decay [120–122]. Knockdown by siRNA of either *WDSOF1* or *HBS1L* was synthetic lethal in a KRAS-driven tumor model [123]. The Ubp3-Bre5 deubiquitination complex regulates anterograde and retrograde transport between the ER and Golgi [124, 125]. Vrg4 and Hvg1 transport GDP-mannose into the Golgi lumen for protein glycosylation [126, 127]. Reduced dTTP pools, evidenced by *CDC8/DTYMK* and *CDC21/TYM*S, can increase doxorubicin cytotoxicity in cancer cell lines [128]. The human homologs of *UBP3*, *CDC8*, and *CDC21* were identified in genome-wide siRNA synthetic interaction studies in cancer cell line models [129–131].

For several examples above, like *SOF1/DCAF13,* genes could be targeted as both a driver of the tumor and as a sensitizer to doxorubicin. To systematically identify all candidate vulnerabilities specific to glycolytic tumor cells (not constrained by GO enrichment), we filtered the overall data set, limiting the list to genes with human homologs and to YKO/KD strains that were growth sufficient (low shift on HLD) (Additional file 1: Figure S8). The human homologs, along with functional descriptions, are provided in Additional file 10: Table S13.

### Glycolysis-specific gene deletion suppression of doxorubicin cytotoxicity

Deletion suppression points to genes that could potentially increase doxorubicin toxicity if overexpressed. GTA identified *histone deubiquitination* (Table 2), and HLD-specific deletion suppression clusters (Additional file 1: Figure S3, clusters 2-0.1-0, 2-0.4-0, 2-0.4-2, and 3-0.3.3-1) had GO term enrichment for terms related to mRNA processing and *meiotic chromosome condensation*.

### Histone deubiquitination

Histone deubiquitination was identified by GTA and includes *SUS1, SGF11, SGF73, UBP8*, *and SEM1* (Fig. 8b); all except *SEM1* are part of the DUBm complex, which mediates histone H2B deubiquitination and mRNA export [132]. Loss of histone H2B ubiquitination resulting in HLEG-specific enhancement (Fig. 6c) is consistent with loss of the DUBm deubiquitinase being suppressing. Together, they implicate regulation by histone ubiquitination as a mechanism of doxorubicin response. The human homologs of *UBP8*, *USP22*, and *USP51* were identified in an RNAi screen for resistance to ionizing radiation [133].

### RNA processing

HLD-specific deletion suppression clusters (2-0.4-0, 2-0.4-2; Additional file 1: Figure S3) were enriched for mRNA processing-related terms including *mRNA 3’ end processing*, *mRNA cleavage*, and *7-methylguanosine cap hypermethylation* (Table 1), but the term-specific heatmaps revealed pleiotropic gene interaction profiles (Additional file 1: Figure S9). *SWM2/YNR004W* and *TGS1* function in 7-methylguanosine (m^7^G) cap trimethylation (cluster 2-0.4-0); however, the *tgs1-∆0* allele also exerted deletion suppression in a respiratory context (Fig. 8b). m^7^G cap trimethylation protects small nuclear RNAs (snRNAs), and small nucleolar RNAs (snoRNAs) from degradation by exonucleases [134, 135], and promotes efficient pre-rRNA processing and ribosome biogenesis [136].

### Meiotic chromosome condensation

*SMC2, SMC4, YCG1*, and *YCS4* constitute the nuclear condensin complex, which functions in chromosome condensation and segregation. The condensin complex associates with chromosomal sites bound by TFIIIC and the RNA Pol III transcription machinery [137], where it facilitates clustering of tRNA genes at the nucleolus [138] (Fig. 8b). The condensin complex has been suggested as a potential therapeutic target for cancer [139], and human homologs *YCG1/NCAPG2*, *YCS4/NCAPD2*, and *SMC4/SMC4* are synthetic lethal with the Ras oncogene [123].

### Warburg transition-independent doxorubicin gene-interaction modules:

Since cancers may have both respiratory and glycolytic cell populations, targeting Warburg-independent interactions could be especially efficacious, as described below.

#### Deletion enhancement

**Fig. 9:**
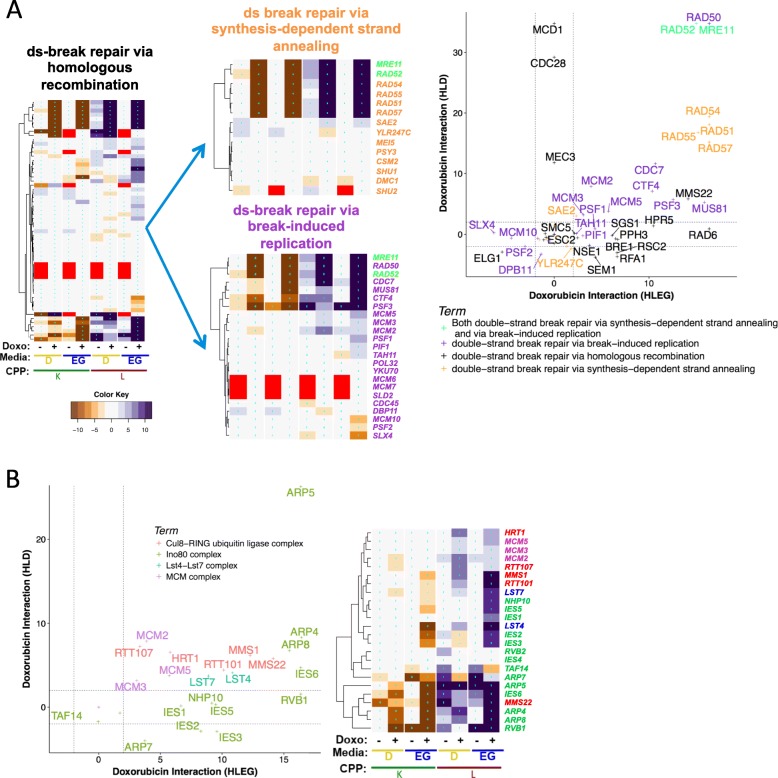
Warburg-independent deletion enhancement of doxorubicin cytotoxicity. Gene interaction profiles showing deletion enhancement in both respiratory and glycolytic context included: **a** *double-strand break repair via homologous recombination*, and its child terms (indicated by arrows), and **b** the *Cul8-RING ubiquitin ligase*, *Ino80 complex*, *Lst4-7 complex*, and *MCM complex*

Cluster 1-0-6 (Additional file 1: Figure S3) had a strong deletion-enhancing profile in both metabolic contexts with GO term enrichment for DNA repair (Fig. 9), as well as histone acetylation (discussed above, Fig. 6b). GTA analysis additionally revealed the Lst4-Lst7, the Cul8-RING ubiquitin ligase, and MCM complexes (Fig. 9b).

#### DNA repair

Warburg-independent, deletion-enhancing pathways included homologous recombination and break-induced replication repair (Fig. 9a), along with the Ino80 complex (Fig. 9b), the latter explained by its role of histone acetylation in the recruitment of DNA repair machinery to dsDNA break sites [51]. The Ino80 complex influences doxorubicin response in fission yeast [140, 141], further suggesting evolutionary conservation of this interaction, and thus potential relevance to mammalian systems [142]. DNA repair pathways, such as those involving *RAD52* and *INO80*, are evolutionarily conserved, involved in genome instability and tumorigenesis [143], and predictive of therapeutic response in some cancers [144], thus representing potential tumor-specific biomarkers for chemotherapeutic efficacy.

### Complexes identified by GTA

Warburg-independent deletion-enhancing modules identified by GTA were weaker, in many cases, than the dsDNA break repair pathways found by REMc, some of which had strong *K* parameter interactions (Fig. 9, Additional file 9). GTA-identified terms included (1) the Cul8-RING ubiquitin ligase complex, which is encoded by *RTT101*, *RTT107*, *MMS1*, *MMS22*, and *HRT1* and functions in replication-associated DNA repair [145]. Cul8/Rtt101, in fact, contributes to multiple complexes that regulate DNA damage responses, including Rtt101-Mms1-Mms22, which is required for Eco1-catalyzed Smc3 acetylation for normal sister chromatid cohesion establishment during S phase [146]; (2) The Lst4-Lst7 complex, which functions in general amino acid permease (*GAP1*) trafficking [147], threonine uptake, and maintenance of deoxyribonucleotide (dNTP) pools [26], clustered with *thr1-∆0* (threonine biosynthesis) in 2-0.2-1 (Additional file 5: File B); and (3) the mini-chromosome maintenance (MCM) complex, which licenses and initiates DNA replication [148], was evidenced by the *mcm2-DAmP*, *mcm3-DAmP*, and *mcm5-DAmP* YKD strains (Fig. 9b). Work in pea plants showed that doxorubicin inhibits the *MCM6* DNA helicase activity [149]. Prior genome-wide experiments with doxorubicin did not analyze YKD mutants; thus, the MCM complex highlights the utility of the DAmP collection in drug-gene interaction studies.

### Media-independent deletion suppression

Loss of genes functioning in processes that augment doxorubicin toxicity results in suppression of its growth inhibitory effect. This was suggested in both respiratory and glycolytic contexts for *sphingolipid homeostasis*, *telomere tethering at nuclear periphery*, and *actin cortical patch localization* (Additional file 1: Figure S3, clusters 2-0.4-1 and 2-0.3-3). Conversely, their overexpression in cancer could potentiate toxicity and therapeutic efficacy.

### Sphingolipid homeostasis and metabolism

**Fig. 10:**
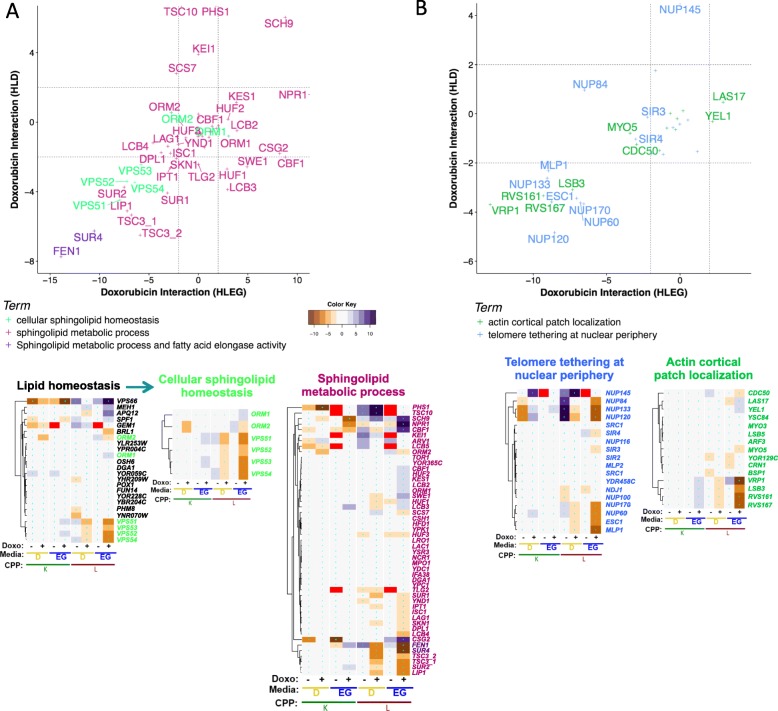
Warburg-independent deletion suppression of doxorubicin cytotoxicity. Doxorubicin-gene interaction profiles and *L*-interaction plots for genes associated with deletion suppression in HLEG or HLD media, including **a** *cellular sphingolipid homeostasis*, along with its parent term, *lipid homeostasis*, and related term *sphingolipid metabolism* and **b** *actin cortical patch localization* and *telomere tethering at nuclear periphery*

From cluster 2-0.4-1, *VPS51, VPS52, VPS53*, and *VPS54* (Fig. 10a) form the Golgi-associated retrograde protein (GARP) complex, which is required for endosome-to-Golgi retrograde vesicular transport. GARP deficiency results in accumulation of sphingolipid synthesis intermediates [150]. Also, from this cluster came fatty acid elongase activity (*FEN1/ELO2* and *SUR4/ELO3*), which when deficient leads to reduced ceramide production and phytosphingosine accumulation [151, 152].

Since the GARP genes and fatty acid elongase activity genes function together in sphingolipid metabolism, we searched all genes annotated to this term and found other media-independent suppressors to include *TSC3*, *LIP1*, *SUR1*, *SUR2*, *IPT1*, and *SKN1* (Fig. 10a). Doxorubicin treatment induces accumulation of ceramide [12, 13], which mediates anti-proliferative responses and apoptosis in yeast and human and appears to mechanistically underlie the influence of this gene group [153] (Additional file 1: Figure S10). These findings were further supported by the deletion enhancer, *SCH9*, which negatively regulates ceramide production by inducing ceramidases and negatively regulating *ISC1* (Fig. 10a) [154]. Multidrug-resistant HL-60/MX2 human promyelocytic leukemia cells are sensitized to doxorubicin by *N*,*N*-dimethyl phytosphingosine [155].

Taken together, the model provides genetic detail regarding how disruption of sphingolipid metabolism increases resistance to doxorubicin and that this occurs in a Warburg-independent manner, seemingly by reducing apoptosis associated with doxorubicin-induced ceramide overproduction [12, 156, 157].

### Telomere tethering at nuclear periphery

Enrichment for *telomere tethering at nuclear periphery* in cluster 2-0.4-1 was comprised of *NUP60, NUP170, MLP1,* and *ESC1*. Although growth deficient on HLD media, *NUP84, NUP120,* and *NUP133* also exerted deletion suppression in HLEG (Fig. 10b). Nuclear pore functions include coordinating nuclear-cytoplasmic transport and localizing proteins and/or chromosomes at the nuclear periphery, which contributes to DNA repair, transcription, and chromatin silencing [158]. Thus, deletion of nuclear pore genes could influence doxorubicin resistance by multiple potential mechanisms involving altering chromatin states, transcriptional regulation, maintenance of telomeric regions, and DNA repair. Doxorubicin-gene interaction profiles for all nuclear pore-related genes are provided in Additional file 1: Figure S11A.

### Actin cortical patch localization

Cluster 2-0.4-1 was enriched for *actin cortical patch localization*, including *RVS167, LSB3, RVS161*, and *VRP1* (Fig. 10b). Related terms (*Arp2/3 protein complex* and *actin cortical patch*) exhibited similar doxorubicin-gene interaction profiles, including *ARC15*, *ARC18*, *ARC35*, *INP52*, *INP53*, *ARP2*, *ARP3*, *GTS1*, *RSP5*, and *FKS1 (*see Additional file 1: Figure S11B-C). This result corroborates studies in mouse embryonic fibroblasts where deletion of *ROCK1* increased doxorubicin resistance by altering the actin cytoskeleton and protecting against apoptosis [159, 160]. Additional literature indicates the importance of actin-related processes for doxorubicin cytotoxicity [161–163], highlighting the utility of yeast phenomics to understand these effects in greater depth.

## Respiratory-deficient doxorubicin-gene interaction modules

From cluster 1-0-0, we noted that respiratory deficient YKO/KD strains (those not generating a growth curve on HLEG) also had low *K* and/or increased *L* “shift” values on HLD, as would be expected of petite strains [164]. Strains in this category tended to display deletion enhancement (Additional file 1: Figure S3) and function primarily in mitochondrial processes (Additional file 5: File C; see GO enrichment for cluster 1-0-0 and derivative clusters), including *mitochondrial translation*, *mitochondrion-ER tethering*, *protein localization into mitochondria*, *mitochondrial genome maintenance*, *respiratory chain complex assembly*, and *proton transport*. Compromise of mitochondrial respiration leading to sensitization of cells to doxorubicin is of interest given recent findings that some glycolytic cancers are respiratory deficient [165, 166].

## Phenomics-based predictions of doxorubicin-gene interaction in cancer cell lines

**Fig. 11:**
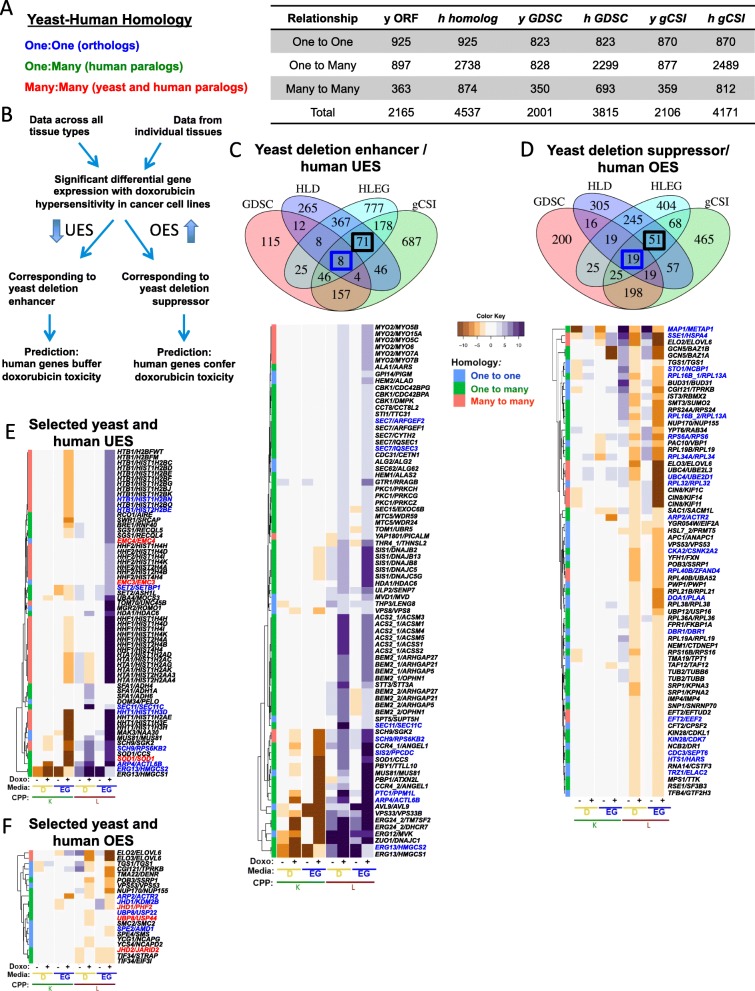
Use of the yeast phenomic model to predict doxorubicin-gene interaction in cancer cells. **a** *BiomaRt* was used to assign yeast-human gene homology for the GDSC and gCSI datasets. **b** *PharmacoGx* was used to retrieve differential gene expression for doxorubicin sensitive cell lines from the gCSI and GDSC databases, searching data from individual tissues or across data aggregated from all tissues. Human genes that are underexpressed in doxorubicin sensitive cell lines (UES) with yeast homologs that are deletion enhancers are predicted to be causal in their phenotypic association. Similarly, human genes that are overexpressed in doxorubicin sensitive cancer cell lines (OES) would be predicted to be causal if the yeast homolog was a deletion suppressor in the phenomic dataset. **c**, **d** Boxes inside of Venn diagrams indicate the genes for which gene interaction profiles are shown in the heatmaps below. Gene names are to the right of heatmaps, with *blue* labels indicating genes identified in both the GDSC and gCSI databases and *black* labels indicating genes found only in the gCSI dataset. The category of homology (see panel **a**) is indicated in the left column of each heatmap. **c** Deletion enhancement by yeast genes predicts human functions that buffer doxorubicin cytotoxicity, and thus, reduced expression of homologs in cancer cell lines is predicted to increase doxorubicin sensitivity. **d** Deletion suppression by yeast genes predicts functions that mediate cytotoxicity and is shown for human homologs having significant association of overexpression in cancer cell lines with increased doxorubicin sensitivity. **e**, **f** Genes representing enhancing or suppressing modules from REMc or GTA that are **e** UES or **f** OES in at least one of the two databases. *Red* labels indicate genes found only in the GDSC database. Additional file 11 reports all results from the analysis described above, including assessment of individual tissues

We next investigated how measures of enhancing and suppressing interactions from the yeast phenomic model could serve to predict and prioritize candidate effectors of cancer cell line sensitivity and transcriptomic data [167, 168]. Differential gene expression, by itself, has been clearly shown to be a poor predictor of whether protein function affects proliferative response to a particular drug [169]. Yeast doxorubicin-gene interaction was matched by homology to differential gene expression in doxorubicin-sensitive cancer cell lines, using *PharmacoGx* [39] and *biomaRt* [40, 41]) in conjunction with the GDSC1000 [170, 171] or gCSI [172, 173] databases (Fig. 11). Differential gene expression analysis was performed for individual tissues and for data aggregated for all tissues. Yeast gene deletion enhancers were used to predict causality for human homologs underexpressed in doxorubicin-sensitive cancer cell lines, termed “UES.” Conversely, yeast gene deletion suppressors were matched to human homologs overexpressed in doxorubicin sensitive cells, termed “OES” (Additional file 11).

There was higher correspondence for yeast gene interactions with differential gene expression in the gCSI vs*.* the GDSC database, partially explained by the greater number of genes reported in gCSI than GDSC (Additional file 12). Such differences are possibly due to sensitivity for measuring gene expression arising from distinct platforms used to measure gene expression and cell cytotoxicity, and different sample sizes in the respective data (https://pharmacodb.pmgenomics.ca/drugs/273). The gCSI data reported more UES and OES genes than GDSC (Additional file 11: Files B-E and Additional file 12) and consequently greater overlap with the yeast phenomic data.

Warburg status was not available for the cancer cell lines, so we prioritized the analysis by focusing on Warburg-independent yeast gene interactions having homology to differentially expressed genes in both the gCSI and GDSC datasets, aggregated across all tissues. These constraints (agreement across all tissues, from both databases, and in both yeast media types) led to prediction of eight UES (*ARP4/ACTL6B, ERG13/HMGCS2, PTC1/PPM1L, SCH9/RPS6KB2, SEC11/SEC11C, SEC7/ARFGEF2, SEC7/IQSEC3,* and *SIS2/PPCDC*) and 18 OES genes (*ARP2/ACTR2, CDC3/SEPT6, CKA2/CSNK2A2, DBR1/DBR1, DOA1/PLAA, EFT2/EEF2, HTS1/HARS, KIN28/CDK7, MAP 1/METAP1, RPL16B/RPL13A*, *RPL32/RPL32, RPL34A/RPL34, RPL40B/ZFAND4,* RPS6A/RPS6, *SSE1/HSPA4, STO1/NCBP1, TRZ1/ELAC2,* and *UBC4/UBE2D1*) as having causal influences on the doxorubicin sensitivity phenotype (Fig. 11c, d).

As detailed in Tables 3 and 4, we expanded the analysis to genes representative of GO term enrichments revealed by the yeast phenomic model having human homologs differentially expressed across all cancer tissues, but without restricting by Warburg-independence or gCSI/GDSC co-evidence. Results for individual tissues are also provided in Additional file 11: File A. We considered whether correlations between doxorubicin-gene interaction in yeast with pharmacogenomic results could be enhanced by particular combinations of the data from this study and prior yeast studies. To briefly summarize, there was a higher correlation for deletion enhancers under respiratory (27%) than glycolytic (19%) conditions, and considering both media, there was higher correspondence of deletion suppressors (43%) than deletion enhancers (25%). The complete data are provided in Additional file 7 and Additional file 11 to enable comparisons between data sets, and example comparisons are summarized in Additional file 12.

**Table 3:** Yeast-human homologs with deletion enhancement and UES across all tissues

hGene	yGene	DB	Fig.	GO term	HLD L|K	HLEG L|K	GDSC pval	gCSI pval	Ref	H	Description hGene
ACTL6B	ARP4	Both	12E	Ino80 Complex	8.3|− 10	16.4|− 12.6	3.3E−02	3.8E−02	[172]	2	Actin like 6B
HMGCS2	ERG13	Both	S7B	N/A	34.8|− 21.4	3.7|− 3.3	2.4E−02	7.9E−04	[186]	2	3-Hydroxy-3-methylglutaryl-CoA synthase 2
PPM1L	PTC1	Both	12C	N/A	15.2|− 4.7	14.7|− 13	3.1E−04	1.6E−02	[173, 174]	2	Protein phosphatase, Mg2+/Mn2+ dependent 1L
RPS6KB2	SCH9	Both	12E	Sphingolipid Metabolic Process	6|− 3.7	8.8|− 9.8	3.5E−02	4.2E−03	NA	3	Ribosomal protein S6 kinase B2
SEC11C	SEC11	Both	S7B	N/A	11.6|− 1.3	2.8|0	3.5E−04	3.5E−04	[175]	2	SEC11 homolog C, signal peptidase complex subunit
ARFGEF2	SEC7	Both	12C	N/A	2.9|0	2.6|0.8	7.5E−03	1.5E−08	[176, 177]	2	ADP ribosylation factor guanine nucleotide exchange factor 2
IQSEC3	SEC7	Both	12C	N/A	2.9|0	2.6|0.8	7.5E−03	4.9E−02	NA	2	IQ motif and Sec7 domain 3
PPCDC	SIS2	Both	12C	N/A	7.3|− 3.5	12.1|− 9.8	3.9E−02	4.7E−03	NA	2	Phosphopantothenoylcysteine decarboxylase
CCS	CCS1	gCSI	NA	N/A	2.4|− 0.4	5.6|− 3.7	3.4E−01	1.2E−02	NA	2	Copper chaperone for superoxide dismutase
HMGCS1	ERG13	gCSI	S7B	N/A	34.8|− 21.4	3.7|− 3.3	9.5E−01	1.4E−02	[185]	3	3-Hydroxy-3-methylglutaryl-CoA synthase 1
HDAC6	HDA1	gCSI	7A	HDA1 Complex	5.2|− 1	9.1|− 1.8	9.1E−01	1.7E−03	[235, 236]	2	Histone deacetylase 6
MUS81	MUS81	gCSI	8B	DNA Topological Change	5.2|− 2.4	15.9|− 11.1	6.9E−02	1.9E−04	[181, 182]	2	MUS81 structure-specific endonuclease subunit
SGK2	SCH9	gCSI	12E	Sphingolipid Metabolic Process	6|− 3.7	8.8|− 9.8	4.6E−01	8.5E−04	NA	1	SGK2, serine/threonine kinase 2
CCS	SOD1	gCSI	12E	N/A	6.2|− 0.5	8.1|− 10.7	3.4E−01	1.2E−02	NA	2	Copper chaperone for superoxide dismutase
SOD1	SOD1	GDSC	12E	N/A	6.2|− 0.5	8.1|− 10.7	4.3E−02	7.9E−01	NA	2	Superoxide dismutase 1
PELO	DOM34	gCSI	9A	Dom34-Hbs1 Complex	2.5|− 0.7	− 1.2|1.1	NA	1.7E−02	NA	2	Pelota mRNA surveillance and ribosome rescue factor
ADH1A	SFA1	gCSI	12E	N/A	4.8|0	0.9|− 0.3	1.1E−01	2.9E−02	[183, 184]	2	Alcohol dehydrogenase 1A (class I), alpha polypeptide
ADH4	SFA1	gCSI	12E	N/A	4.8|0	0.9|− 0.3	3.6E−01	3.6E−03	[184]	3	Alcohol dehydrogenase 4 (class II), pi polypeptide
ADH6	SFA1	gCSI	12E	N/A	4.8|0	0.9|− 0.3	8.6E−01	3.3E−03	[183]	1	Alcohol dehydrogenase 6 (class V)
HIST1H3D	HHT1	Both	6A-B	Nucleosome Assembly	0.4|− 0.2	15|− 10.2	4.2E−02	2.1E−02	NA	2	Histone cluster 1 H3 family member d
HIST1H2BN	HTB1	Both	6A-B	Nucleosome Assembly	0.2|− 0.6	7.8|− 5.8	4.0E−02	3.0E−06	NA	2	Histone cluster 1 H2B family member n
HIST2H2BE	HTB1	Both	6A-B	Nucleosome Assembly	0.2|− 0.6	7.8|− 5.8	4.0E−02	2.3E−08	NA	2	Histone cluster 2 H2B family member e
SETBP1	SET2	Both	6A,C	Histone exchange	1.3|− 1.6	5.4|− 2.5	7.3E−07	3.0E−04	NA	3	SET binding protein 1
RNF40	BRE1	gCSI	7C	Histone Monoubiquitination	− 0.7|0.5	6.5|− 4.9	7.4E−01	8.5E−03	NA	2	Ring finger protein 40
HIST1H4D	HHF1	gCSI	6A-B	Nucleosome Assembly	− 0.6|0.2	13.7|− 3.8	NA	8.9E−03	NA	3	Histone cluster 1 H4 family member d
HIST1H4H	HHF1	gCSI	6A-B	Nucleosome Assembly	− 0.6|0.2	13.7|− 3.8	8.6E−02	2.8E−06	NA	3	Histone cluster 1 H4 family member h
HIST1H4I	HHF1	gCSI	6A-B	Nucleosome Assembly	− 0.6|0.2	13.7|− 3.8	NA	3.8E−02	NA	2	Histone cluster 1 H4 family member i
HIST1H4K	HHF1	gCSI	6A-B	Nucleosome Assembly	− 0.6|0.2	13.7|− 3.8	NA	8.0E−03	NA	3	Histone cluster 1 H4 family member k
HIST2H4A	HHF1	gCSI	6A-B	Nucleosome Assembly	− 0.6|0.2	13.7|− 3.8	NA	4.8E−02	NA	3	Histone cluster 2 H4 family member a
HIST2H4B	HHF1	gCSI	6A-B	Nucleosome Assembly	− 0.6|0.2	13.7|− 3.8	NA	3.7E−03	NA	3	Histone cluster 2 H4 family member b
HIST4H4	HHF1	gCSI	6A-B	Nucleosome Assembly	− 0.6|0.2	13.7|− 3.8	5.4E−02	2.4E−02	NA	3	Histone cluster 4 H4
HIST1H4D	HHF2	gCSI	12E	Chromatin Assembly or Disassembly	− 1.6|0.4	4.3|− 0.1	NA	8.9E−03	NA	3	Histone cluster 1 H4 family member d
HIST1H4H	HHF2	gCSI	12E	Chromatin Assembly or Disassembly	− 1.6|0.4	4.3|− 0.1	8.6E−02	2.8E−06	NA	3	Histone cluster 1 H4 family member h
HIST1H4I	HHF2	gCSI	12E	Chromatin Assembly or Disassembly	− 1.6|0.4	4.3|− 0.1	NA	3.8E−02	NA	3	Histone cluster 1 H4 family member i
HIST1H4K	HHF2	gCSI	12E	Chromatin Assembly or Disassembly	− 1.6|0.4	4.3|− 0.1	NA	8.0E−03	NA	3	Histone cluster 1 H4 family member k
HIST2H4A	HHF2	gCSI	12E	Chromatin Assembly or Disassembly	− 1.6|0.4	4.3|− 0.1	NA	4.8E−02	NA	3	Histone cluster 2 H4 family member a
HIST2H4B	HHF2	gCSI	12E	Chromatin Assembly or Disassembly	− 1.6|0.4	4.3|− 0.1	NA	3.7E−03	NA	3	Histone cluster 2 H4 family member b
HIST4H4	HHF2	gCSI	12E	Chromatin Assembly or Disassembly	− 1.6|0.4	4.3|− 0.1	5.4E−02	2.4E−02	NA	3	Histone cluster 4 H4
HIST1H2AE	HHT1	gCSI	6A-B	Nucleosome Assembly	0.4|− 0.2	15|− 10.2	NA	1.1E−02	NA	3	Histone cluster 1 H2A family member e
HIST1H3E	HHT1	gCSI	6A-B	Nucleosome Assembly	0.4|− 0.2	15|− 10.2	NA	1.3E−02	NA	3	Histone cluster 1 H3 family member e
HIST1H3H	HHT1	gCSI	6A-B	Nucleosome Assembly	0.4|− 0.2	15|− 10.2	NA	6.8E−03	NA	3	Histone cluster 1 H3 family member h
HIST1H2AC	HTA1	gCSI	12E	Chromatin Assembly or Disassembly	− 3.5|0.8	13.5|− 5.2	7.1E−01	2.9E−05	NA	3	Histone cluster 1 H2A family member c
HIST1H2AD	HTA1	gCSI	12E	Chromatin Assembly or Disassembly	− 3.5|0.8	13.5|− 5.2	3.7E−01	5.8E−03	NA	3	Histone cluster 1 H2A family member d
HIST1H2AG	HTA1	gCSI	12E	Chromatin Assembly or Disassembly	− 3.5|0.8	13.5|− 5.2	5.6E−01	1.4E−02	NA	3	Histone cluster 1 H2A family member g
HIST1H2AK	HTA1	gCSI	12E	Chromatin Assembly or Disassembly	− 3.5|0.8	13.5|− 5.2	NA	6.3E−04	NA	3	Histone cluster 1 H2A family member k
HIST2H2AA3	HTA1	gCSI	12E	Chromatin Assembly or Disassembly	− 3.5|0.8	13.5|− 5.2	NA	2.6E−02	NA	3	Histone cluster 2 H2A family member a3
HIST2H2AA4	HTA1	gCSI	12E	Chromatin Assembly or Disassembly	− 3.5|0.8	13.5|− 5.2	NA	5.3E−03	NA	3	Histone cluster 2 H2A family member a4
H2BFM	HTB1	gCSI	6A-B	Nucleosome Assembly	0.2|− 0.6	7.8|− 5.8	NA	3.0E−02	NA	3	H2B histone family member M
H2BFWT	HTB1	gCSI	6A-B	Nucleosome Assembly	0.2|− 0.6	7.8|− 5.8	3.3E−01	6.5E−04	NA	3	H2B histone family member W, testis specific
HIST1H2BC	HTB1	gCSI	6A-B	Nucleosome Assembly	0.2|− 0.6	7.8|− 5.8	9.8E−01	5.3E−05	NA	3	Histone cluster 1 H2B family member c
HIST1H2BD	HTB1	gCSI	6A-B	Nucleosome Assembly	0.2|− 0.6	7.8|− 5.8	4.7E−01	3.0E−06	NA	3	Histone cluster 1 H2B family member d
HIST1H2BE	HTB1	gCSI	6A-B	Nucleosome Assembly	0.2|− 0.6	7.8|− 5.8	NA	2.5E−04	NA	3	Histone cluster 1 H2B family member e
HIST1H2BF	HTB1	gCSI	6A-B	Nucleosome Assembly	0.2|− 0.6	7.8|− 5.8	NA	5.3E−03	NA	3	Histone cluster 1 H2B family member f
HIST1H2BG	HTB1	gCSI	6A-B	Nucleosome Assembly	0.2|− 0.6	7.8|− 5.8	NA	5.8E−04	NA	3	Histone cluster 1 H2B family member g
HIST1H2BJ	HTB1	gCSI	6A-B	Nucleosome Assembly	0.2|− 0.6	7.8|− 5.8	9.2E−02	1.5E−03	NA	3	Histone cluster 1 H2B family member j
HIST1H2BK	HTB1	gCSI	6A-B	Nucleosome Assembly	0.2|− 0.6	7.8|− 5.8	NA	9.0E−04	NA	3	Histone cluster 1 H2B family member k
HIST1H2BO	HTB1	gCSI	6A-B	Nucleosome Assembly	0.2|− 0.6	7.8|− 5.8	NA	2.9E−02	NA	3	Histone cluster 1 H2B family member o
NAA30	MAK3	gCSI	8A	NatC Complex	0.2|− 0.5	16.6|− 11.6	8.5E−01	2.9E−02	[180]	2	N (alpha)-acetyltransferase 30, NatC catalytic subunit
ROMO1	MGR2	gCSI	S3C	Protein import into mitochondrial matrix	0|− 0.2	10.3|0.1	7.1E−02	4.1E−02	[178, 179]	2	Reactive oxygen species modulator 1
AIRE	RCO1	gCSI	7A	Rpd3S Complex	0.9|− 0.5	7.9|− 4.4	5.5E−01	1.3E−03	NA	3	Autoimmune regulator
ASH1L	SET2	gCSI	6A,C	Histone exchange	1.3|− 1.6	5.4|− 2.5	6.6E−01	5.5E−04	NA	3	ASH1 like histone lysine methyltransferase
RECQL4	SGS1	gCSI	8B	DNA Topological Change	− 0.2|0.7	6.1|− 2.5	7.3E−01	3.2E−02	NA	3	RecQ like helicase 4
RECQL5	SGS1	gCSI	8B	DNA Topological Change	− 0.2|0.7	6.1|− 2.5	2.7E−01	3.0E−04	NA	1	RecQ like helicase 5
SRCAP	SWR1	gCSI	7A	Swr1 complex	0.4|− 0.5	7.3|− 6.2	NA	5.1E−04	NA	3	Snf2 related CREBBP activator protein
UNC45B	TOM70	gCSI	S3C	Protein import into mitochondrial matrix	0.8|− 0.4	12.4|− 0.3	7.4E−01	1.6E−02	NA	2	unc-45 myosin chaperone B
MOCS3	UBA4	gCSI	8A	protein urmylation	1.5|− 3.3	8.1|− 3.4	8.0E−01	3.0E−02	NA	1	Molybdenum cofactor synthesis 3
EMC3	EMC3	GDSC	8A	ER Membrane Protein Complex	1.5|− 0.8	5.6|− 1.8	1.1E−02	NA	NA	2	ER membrane protein complex subunit 3
EMC4	EMC4	GDSC	8A	ER Membrane Protein Complex	− 0.1|− 0.3	6.2|− 1.6	2.6E−02	NA	NA	2	ER membrane protein complex subunit 4

For column “DB”: “gCSI,” “GDSC”, or “Both” indicate UES in the gCSI, GDSC, or both databases. Column “Fig.” refers to specific figures. Columns “HLD L|K” and “HLEG L|K” contain the L and K interaction scores for HLD and HLEG media, respectively. “GDSC pval” and “gCSI pval” refer to the significance of differential gene expression in the respective databases. “Ref” refers to relevant literature citations. “H” refers to homology type: “1,” “2,” and “3” indicate 1:1, 1:many, and many:many, respectively

**Table 4:** Yeast-human homologs with deletion suppression and OES across all tissues

hGene	yGene	DB	Fig	GO term	HLD L|K	HLEG L|K	GDSC pval	gCSI pval	Ref	H	Description hGene
ACTR2	ARP2	Both	12D	Arp2/3 Protein Complex	− 3.7|1.4	− 3.3|− 6.9	3.2E−02	6.0E−05	[188]	1	ARP2 actin-related protein 2 homolog
SEPT6	CDC3	Both	12D	N/A	− 2.1|0.7	− 2.6|0.3	1.7E−04	2.8E−05	NA	1	Septin 6
CSNK2A2	CKA2	Both	12D	N/A	− 5.5|1	− 4|1.2	4.3E−03	3.6E−03	NA	2	Casein kinase 2 alpha 2
DBR1	DBR1	Both	12D	N/A	− 2.1|0.6	− 3.5|1	4.3E−02	9.8E−04	NA	1	Debranching RNA lariats 1
PLAA	DOA1	Both	12D	N/A	− 2.2|0.7	− 7.7|1.9	2.6E−02	1.5E−04	NA	3	Phospholipase A2 activating protein
EEF2	EFT2	Both	12D	N/A	− 2.7|0.2	− 2.1|1.1	1.9E−02	9.7E−06	[189]	2	Eukaryotic translation elongation factor 2
HARS	HTS1	Both	12D	N/A	− 2.4|0.6	− 2.9|0.5	4.1E−03	1.6E−03	NA	1	Histidyl-tRNA synthetase
CDK7	KIN28	Both	12D	N/A	− 2.2|1.1	− 2.5|− 1.2	2.4E−02	2.6E−04	[190–192]	3	Cyclin-dependent kinase 7
METAP1	MAP 1	Both	12D	N/A	− 4.7|3.3	− 4.2|− 0.6	8.9E−03	2.3E−02	NA	1	Methionyl aminopeptidase 1
RPL13A	RPL16B	Both	12D	N/A	− 4.5|3.7	− 5.8|1.4	1.5E−03	9.5E−05	NA	2	Ribosomal protein L13a
RPL32	RPL32	Both	12D	N/A	− 3.9|1	− 11.3|1.1	6.9E−03	3.6E−03	[196]	2	Ribosomal protein L32
RPL34	RPL34A	Both	12D	N/A	− 4.8|2.3	− 7.2|2.4	1.5E−02	4.4E−03	[193–195]	3	Ribosomal protein L34
ZFAND4	RPL40B	Both	12D	N/A	− 4.1|1.1	− 5.7|1.1	3.7E−02	1.7E−02	NA	2	Zinc finger AN1-type containing 4
RPS6	RPS6A	Both	12D	N/A	− 5.7|1.8	− 6|2.6	2.0E−04	2.5E−07	[197, 198]	2	Ribosomal protein S6
HSPA4	SSE1	Both	12D	N/A	− 6.3|3	− 13.7|4.4	1.5E−02	4.2E−07	NA	2	Heat shock protein family A (Hsp70) member 4
NCBP1	STO1	Both	12D	N/A	− 3|1.7	− 4.3|1.3	2.3E−03	3.5E−04	NA	2	Nuclear cap-binding protein subunit 1
ELAC2	TRZ1	Both	12D	N/A	− 2.3|0.6	− 2.6|0.1	1.1E−05	1.5E−08	NA	3	ElaC ribonuclease Z 2
UBE2D1	UBC4	Both	12D	N/A	− 4.6|2.2	− 12.3|2.6	1.0E−02	8.1E−03	[199]	1	Ubiquitin conjugating enzyme E2 D1
TPRKB	CGI121	gCSI	12F	EKC/KEOPS Complex	− 2.2|− 0.8	− 7.7|2.1	1.3E−01	7.6E−04	NA	1	TP53RK binding protein
ELOVL6	ELO2	gCSI	12F	Fatty Acid Elongase Activity	− 7.7|1.4	− 13.9|4.1	5.1E−01	2.7E−02	[205]	2	ELOVL fatty acid elongase 6
ELOVL6	ELO3	gCSI	12F	Fatty Acid Elongase Activity	− 6.3|1.3	− 10.5|1.9	5.1E−01	2.7E−02	[205]	1	ELOVL fatty acid elongase 6
NUP155	NUP170	gCSI	12F	Telomere tethering at the nuclear periphery	− 3.7|0.6	− 6.5|1.3	1.0E−01	4.4E−02	[215–217]	1	Nucleoporin 155
SSRP1	POB3	gCSI	12F	FACT Complex	− 4.1|1.2	− 5.1|1.4	6.0E−02	2.2E−06	[20]	2	Structure-specific recognition protein 1
TGS1	TGS1	gCSI	12F	7-methylguanosine cap hypermethylation	− 2.4|2.6	− 3.3|0.7	8.5E−02	2.0E−03	NA	2	Trimethylguanosine synthase 1
VPS53	VPS53	gCSI	12F	Cellular sphingolipid homeostasis	− 2.4|1.8	− 5.8|1.4	2.0E−01	2.4E−02	[201–204]	2	VPS53, GARP complex subunit
USP22	UBP8	Both	12F	histone deubiquitination	− 2|0.8	0.3|0.3	2.3E−02	1.2E−02	NA	1	Ubiquitin-specific peptidase 22
SMC2	SMC2	gCSI	12F	meiotic chromosome condensation	− 3.5|1.2	0.4|− 0.9	1.1E−01	4.3E−02	[138]	3	Structural maintenance of chromosomes 2
NCAPG	YCG1	gCSI	12F	meiotic chromosome condensation	− 2|0.8	− 0.8|− 0.6	7.9E−01	9.2E−06	[138]	3	Non-SMC condensin I complex subunit G
NCAPD2	YCS4	gCSI	12F	meiotic chromosome condensation	− 2.4|0.8	− 1.7|− 0.9	2.3E−01	1.7E−03	NA	2	Non-SMC condensin I complex subunit D2
USP44	UBP8	GDSC	12F	histone deubiquitination	− 2|0.8	0.3|0.3	4.1E−04	6.1E−01	NA	2	Ubiquitin specific peptidase 44
KDM2B	JHD1	Both	12F	Histone Demethylation	0.2|− 0.2	− 2.3|1.9	4.6E−02	3.5E−02	[206–208]	1	Lysine demethylase 2B
AMD1	SPE2	Both	12F	spermine biosynthetic process	0.2|− 0.2	− 2.8|0.5	1.7E−02	1.5E−04	[200]	1	Adenosylmethionine decarboxylase 1
SMS	SPE4	gCSI	12F	spermine biosynthetic process	− 0.8|0.4	− 2.4|1	NA	3.9E−02	NA	1	Spermine synthase
EIF3I	TIF34	gCSI	12F	translation reinitiation	1.2|0	− 3.9|1.4	8.2E−01	7.1E−05	NA	2	Eukaryotic translation initiation factor 3 subunit I
STRAP	TIF34	gCSI	12F	translation reinitiation	1.2|0	− 3.9|1.4	6.7E−01	9.1E−03	NA	2	Serine/threonine kinase receptor-associated protein
DENR	TMA22	gCSI	12F	translation reinitiation	− 1.1|0.6	− 6.4|1.9	4.0E−01	1.9E−02	[210–214]	1	Density regulated re-initiation and release factor
PHF2	JHD1	GDSC	12F	Histone Demethylation	0.2|− 0.2	− 2.3|1.9	1.9E−03	6.8E−02	NA	1	PHD finger protein 2
JARID2	JHD2	GDSC	12F	Histone Demethylation	− 0.2|0.1	− 3.2|1	1.9E−03	2.5E−02	[209]	2	Jumonji and AT-rich interaction domain containing 2

For column “DB”: “gCSI,” “GDSC,” or “Both” indicate UES in the gCSI, GDSC, or both databases. Column “Fig.” refers to specific figures. Columns “HLD L|K” and “HLEG L|K” contain the L and K interaction scores for HLD and HLEG media, respectively. “GDSC pval” and “gCSI pval” refer to the significance of differential gene expression in the respective databases. “Ref” refers to relevant literature citations. “H” refers to homology type: “1,” “2,” and “3” indicate 1:1, 1:many, and many:many, respectively

### Deletion enhancers with UES homologs

Concordance between deletion-enhancing doxorubicin-gene interaction in yeast and UES observed for the corresponding human homologs in cancer cells suggests synergistic targets for inhibition and biomarkers of genetic vulnerabilities that may increase therapeutic efficacy for doxorubicin (Table 3 and Fig. 11c). Many of these genes function in processes identified by the yeast phenomic model (Tables 1, 2, 3, and 4) and have annotated roles in cancer biology.

Doxorubicin-enhancing interactions that were UES in both gCSI and GDSC included *ACTL6B*, identified as a candidate tumor suppressor gene in primary hepatocellular carcinoma tissue [174]; *PPM1L*, which regulates ceramide trafficking at ER-Golgi membrane contact sites [175] and exhibits reduced expression in familial adenomatous polyposis [176]; *RPS6KB2*, which was UES in the breast, ovary, and bone in gCSI, while *RPS6KA1*, *A2*, *A5,* and *A6* were UES in select tissues in both databases (Additional file 11: File A); *SEC11/SEC11C*, which is upregulated in response to hypoxia in non-small cell lung cancer tissue [177] and for which deletion enhancement was stronger in glycolytic media (Additional file 1: Figure S8); *SEC7/ARFGEF2* (alias *BIG2)* which exhibits increased gene and protein expression in pancreatic cancer [178], and shRNA knockdown of *ARFGEF2* can reduce Burkitt’s lymphoma cell survival [179].

**Fig. 12:**
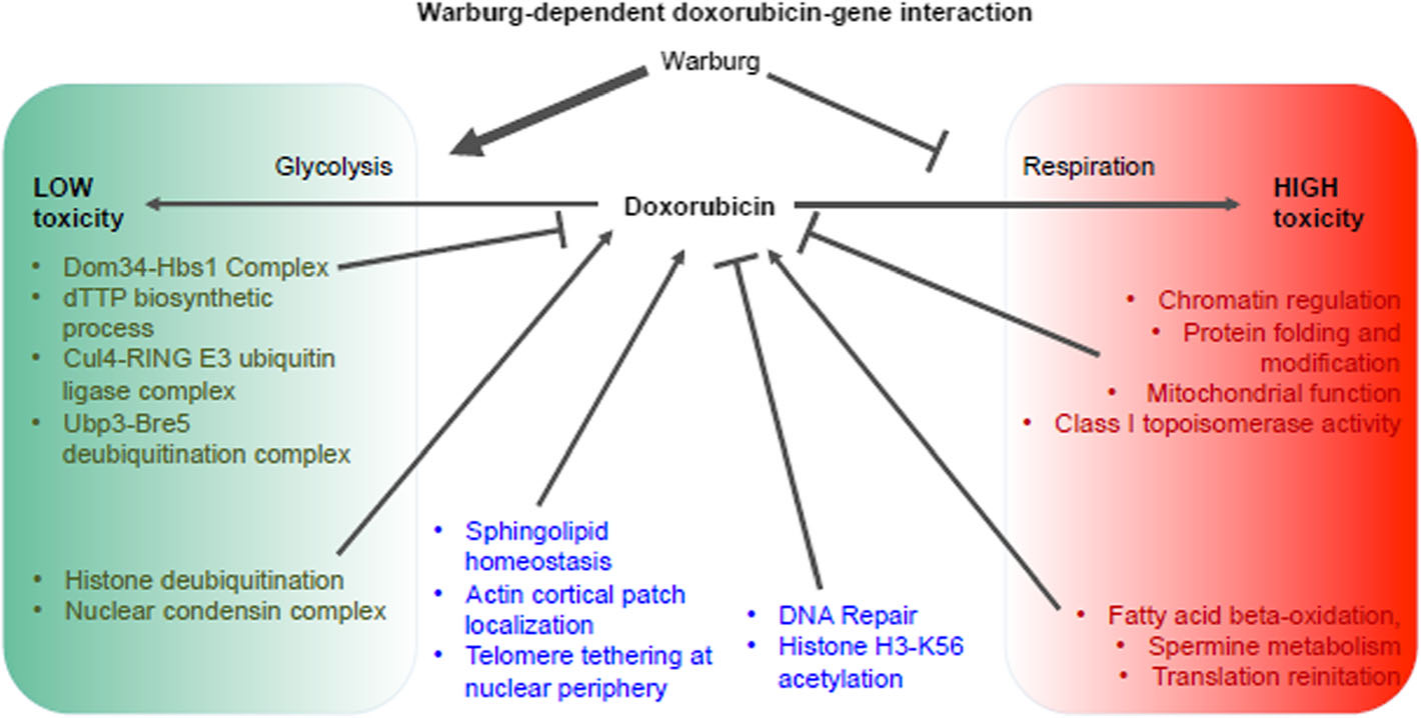
Yeast phenomic model for the influence of Warburg metabolism on doxorubicin-gene interaction. Shaded areas indicate influences that are relatively Warburg-dependent, being red or green if their effects are relatively specific to a respiratory or glycolytic context, respectively. Processes that influence doxorubicin cytotoxicity in a more Warburg-independent manner are unshaded. Arrowheads indicate processes for which genes predominantly transduce doxorubicin toxicity, based on their loss of function suppressing its growth inhibitory effects. Conversely, a perpendicular bar at the line head indicates a process that buffers doxorubicin toxicity, as genetic compromise of its function enhances the growth inhibitory effects of doxorubicin

We expanded the analysis above by matching yeast gene deletion enhancers to human UES genes in either database, i.e., not requiring that genes be significant in both datasets (Fig. 12e, f). The result highlighted chromatin-related buffering processes, including nucleosome assembly (*HTA1, HTB1, HHF1, HHF2, HHT1, HHF1)*, histone exchange (*SET2/SETBP1* and *SWR1/SRCAP)*, and histone modifiers (*BRE1, HDA1, RCO1*) (Fig. 11e and Table 3). Other functions predicted by the yeast model to buffer doxorubicin toxicity in cancer cells included DNA topological change (*MUS81, SGS1*), mitochondrial maintenance (*MGR2, TOM70*), protein acetylation (*MAK3*), and metabolism (*SFA1, ERG13, SOD1*).

*MGR2/ROMO1* is involved in protein import into the mitochondrial matrix and overexpression of *ROMO1* has been associated with poor prognosis in colorectal [181] and non-small cell lung cancer patients [182]. *MAK3/NAA30,* a component of the NatC complex (Fig. 8a), induces p53-dependent apoptosis when knocked down in cancer cell lines [183]. *MUS81* knockdown enhances sensitivity of colon cancer lines to epirubicin (doxorubicin analog) [184], cisplatin, and other chemotherapy agents by activating the *CHK1* pathway (Fig. 7b) [180].

The glycolysis-specific deletion enhancer, *SFA1,* has seven human homologs, of which three (*ADH4, ADH1A,* and *ADH6*) were UES in gCSI data (Additional file 1: Figure S8). High expression of *ADH1A* or *ADH6* was predictive of improved prognosis for pancreatic adenocarcinoma [185], and high expression of *ADH1A* or *ADH4* had improved prognosis for non-small cell lung cancer [186]. The *ERG13* homolog, *HMGCS1*, has been suggested as a synthetic lethal target for BRAF^V600E^-positive human cancers [187], and *HMGCS2* plays a role in invasion and metastasis in colorectal and oral cancer [188]. These data suggest doxorubicin treatment may have anti-tumor efficacy specifically in glycolytic tumors with reduced expression of *SFA1* and *ERG13* homologs.

### Deletion suppressors with OES homologs

Genes that promote toxicity of a drug could lead to increased sensitivity if overexpressed in cancer cell lines or, correspondingly, deletion suppression by yeast phenomic analysis. Choosing chemotherapeutic agents for patients based on their tumors exhibiting high expression of genes known to increase sensitivity represents a targeted strategy to increase therapeutic efficacy and could be particularly effective if the sensitizing overexpressed genes happen to also be drivers [189]. Human genes that were OES and homologous to yeast deletion suppressors are highlighted in Table 4 and Fig. 11d. *ARP2/ACTR2* is a member of the Arp2/3 protein complex (see Additional file 1: Figure S11C), and silencing of the Arp2/3 protein complex reduces migration of pancreatic cancer cell lines [190]. *EEF2* protein is overexpressed in multiple cancer types, where shRNA knockdown inhibits growth [191]. *CDK7* overexpression in breast [192, 193] and gastric [194] cancer is predictive of poor prognosis. *RPL34* overexpression promotes proliferation, invasion, and metastasis in pancreatic [195], non-small cell lung [196], and squamous cell carcinoma [197], while *RPL32* was also overexpressed in a prostate cell cancer model [198]. In contrast to Rps6k family members being UES/deletion enhancing, Rps6 was OES/deletion suppressing in ovarian tissue. *RPS6* overexpression portends reduced survival for patients with renal carcinoma [199] and hyperphosphorylation of Rps6 confers poor prognosis in non-small cell lung cancer [200]. Overexpression of *UBE2D1* is associated with decreased survival in lung squamous cell carcinoma tissue [201], and numerous additional ubiquitin-conjugating enzyme family members were OES in analysis of individual tissues (Additional file 11: File A).

We expanded the analysis, similar to the way described above for the deletion enhancers, by relaxing the matching criteria in order to identify additional deletion suppressing pathways revealed by the yeast model (Additional file 11). The extended analysis identified yeast-human conserved functions in metabolism (*SPE2, SPE4, VPS53, ELO2, ELO4*), histone demethylation (*JHD1, JHD2*), translation reinitiation (*TMA22, TIF32*), the condensin complex (*YCG1*, *YCS4*, *SMC2*), and telomere tethering at the nuclear periphery (*NUP170*) (Table 4 and Fig. 11f). *SPE2/AMD1* is required for spermidine and spermine biosynthesis, and upregulation of *AMD1* by mTORC1 rewires polyamine metabolism in prostate cancer cell lines and mouse models [202]. *VPS53*, a component of the GARP complex involved in sphingolipid homeostasis, is a tumor suppressor in hepatocellular carcinoma [203–205]; additionally, transfection with *VPS53* transcript induces apoptosis and sensitizes cervical cancer cells to doxorubicin [206], consistent with our model. Inhibition of *ELOVL6* (homologous to yeast *ELO2* and *ELO3*) in mice reduces tumor growth and increases survival [207]. The histone demethylase, *JHD1/KDM2B*, is overexpressed in pancreatic cancer [208] and is associated with poor prognosis in glioma [209] and triple-negative breast cancer [210]. A second homolog, *JHD2/JARID2*, is required for tumor initiation in bladder cancer [211]. The yeast model also predicts causality underlying OES associated with genes involved in translation reinitiation, *TMA22/DENR* (translation machinery associated) and *TIF32/EIF31. DENR-MCT-1* regulates a class of mRNAs encoding oncogenic kinases [212–214], and its overexpression in hepatocellular carcinoma is associated with metastasis [215]. *TMA22/DENR* also exerts evolutionarily conserved influence on telomeric function and cell proliferation [216]. *YCG1/NCAPG* and *SMC2/SMC2* are components of the condensin complex, which are overexpressed in cancer [139]. *NUP170/NUP155*, which functions in telomere tethering at the nuclear periphery (Fig. 10b), is hyper-methylated in association with breast cancer [217, 218], where its reduced expression contributes to a signature for bone metastasis [219].

## Discussion

A relatively comprehensive and quantitative phenotypic model of the gene-drug interaction network underlying the cell proliferative response to doxorubicin is presented. We demonstrate how a yeast phenomic model is predictive of genetic vulnerabilities to a cytotoxic agent that arise in cancer cells due to differential gene expression. Although an unbiased, experimental model of genetic interaction is largely descriptive with respect to molecular mechanisms, we propose it to nevertheless be a unique and powerful resource to model genotype-phenotype networks at the cellular and organismal level. Genes that promote or buffer a cell proliferative response to defined perturbations report on molecular networks that potentially influence a variety of phenotypes [220, 221]. For disease phenotypes involving evolutionarily conserved cellular processes, such as cell cycle and DNA repair (e.g., cancer) or folding of biogenesis of polytopic membrane proteins (e.g., cystic fibrosis), phenomic models can account for biological context and potential genetic modifiers of disease [25, 28], serving to further inform the disease literature while also generating in an unbiased experimental manner new hypotheses that can be tested across species [26, 222–225].

To create the doxorubicin-gene interaction model for cancer, we used advances in Q-HTCP for phenomic analysis of the YKO/KD library and developed customized GO tools to mine cancer pharmacogenomics data via homology information. The information resulting from the model generates new hypotheses, further integrating the yeast and cancer literature, thus providing new insights for investigators with complementary expertise to further clarify the mechanistic basis of the observed gene-drug interaction network [27]. We propose that it may be further possible to leverage yeast phenomics to advance precision oncology models somewhat independently of molecular mechanisms; i.e., if genetic interactions that determine the response to cytotoxic chemotherapy can be successfully predicted, it may not be necessary to detail all interactions mechanistically in order for the model to be a useful tool for decision-making in precision oncology.

Many genes are implicated in oncogenesis and in chemotherapeutic response, with varying degrees of tissue-specific influence and yeast-human homology. The ability to assess mutation, differential gene expression, and other molecular correlates of cancer and chemotherapeutic efficacy is growing, but the direct assessment of drug-gene interaction (i.e., phenotypic/cell proliferative responses) remains a challenge due to the complex genetics and tissue-specific aspects of cancer. In contrast to this daunting complexity, yeast is a single-cell eukaryotic organism that is uniquely amenable to precise and genome-wide measures of drug-gene interaction, and from which fundamental contributions to our understanding of human disease are well established [226–230]. We show that yeast, which naturally exhibits aerobic glycolysis, can be used to explore the potential of the Warburg effect to influence the anti-cancer efficacy of doxorubicin, and thus potentially other chemotherapeutic agents [1, 231]. From an unbiased systems perspective, we observed that a less extensive genetic network is required to buffer doxorubicin in glycolytic vs. respiring cells. The doxorubicin-gene interaction network suggested GO-enriched genetic vulnerabilities that may occur in cancer cells due to genomic instability, including defective chromatin regulation, protein folding and modification, mitochondrial function, and DNA topology; while impeding the ability to buffer doxorubicin toxicity in a respiratory context, such vulnerabilities can be relieved of by the Warburg transition to glycolytic metabolism. Also specific to respiratory conditions, the yeast model predicts that doxorubicin is *less* toxic to cells if functions for fatty acid beta-oxidation, spermine metabolism, and translation reinitiation are compromised by mutation (Fig. 12 and Tables 1 and 2). On the other hand, cells that transition to glycolytic metabolism need dTTP biosynthesis and protein complexes including the Cul4-RING E3 ubiquitin ligase, and the Ubp3-Bre5 deubuiquitinase, as well as Dom34-Hbs1, which functions in ‘no-go’ mRNA decay, in order to buffer doxorubicin (Fig. 12 and Table 2). These findings indicate that the metabolic status of cancer cells further influences the vulnerabilities to chemotherapy that may emerge from genetic alterations occurring in cancer. Thus, along with monitoring Warburg status in conjunction with cancer genetic profiling, yeast phenomic models that predict potential influences of Warburg status on chemotherapy response and cancer vulnerabilities emerging from somatic mutations unique to each individual patient, may help better predict therapeutic outcomes and thus be useful to develop more efficacious treatment algorithms.

Since the Warburg metabolic status is not monitored clinically, we thought the most relevant genes from the yeast phenomic model for predicting genetic modifiers of doxorubicin cytotoxicity in pharmacogenomics data would be those influencing doxorubicin cytotoxicity similarly in either metabolic context. Functionally enriched genes in this category represented DNA repair and histone H3-K56 acetylation, along with deletion suppressing pathways, including sphingolipid homeostasis, actin cortical patch localization, and telomere tethering at the nuclear periphery (Fig. 12 and Table 3). We expanded the analysis to genes that were not GO-enriched, because genes may have evolutionarily conserved influences on phenotype independent of prior functional annotation and also independent of enrichment all together. Thus, we examined homologs systematically (regardless of GO enrichment) for yeast phenomic–PharmacoDB correlation and if evident specifically in either the glycolytic or respiratory context (Fig. 11, Tables 3 and 4, and Additional file 11). As discussed in the “Results” section, many genes not identified solely by GO-enrichment were nevertheless representative of enriched buffering processes from the phenomic model. The supplemental data files provided enable mining the pharmacogenomics data with the yeast model, filtering on combinations of desired criteria, including metabolic status, tissue type, and pharmacogenomics data set (Additional file 11).

Regarding the Warburg influence on chemotherapy response highlighted by the yeast phenomic model, work with cancer cell lines, mice, and acute myeloid leukemia blast cells from patients have suggested histone eviction, increased mutation rates at active promoter sites are important mechanisms of doxorubicin toxicity [18, 19, 232], including accumulation of damage from chromatin trapping by the FACT complex [20]. Further support of the importance of chromatin regulation was suggested by transcriptional control and assembly of histones, as well as histone modifications, as also suggested by differential gene expression from the pharmacogenomics data. The yeast model suggests that most of these effects are particularly important in a respiratory context; thus, from a precision medicine perspective, tumors that are promoted by genetic compromise in chromatin regulation [233, 234] would be potentially more susceptible to treatment, but only if they have not undergone the Warburg transition to glycolysis. Analogously, patients with germline variation resulting in functional compromise of chromatin regulation may have normal tissue (e.g., cardiac muscle) that is susceptible to doxorubicin and thus may suffer greater toxic side effects of cancer treatment.

The genetic and phenotypic resolution of the yeast model can help resolve differential buffering by related complexes or pathways. In the example of histone deacetylase complexes, the class I (RPD3L and 3S complexes) and class II (HDA1 complex) HDAC genes interact differentially with doxorubicin. The Sin3-type class I HDAC complex exerts stronger deletion enhancement that is respiration specific, while the Class II (HDA1) complex shows weaker deletion enhancement that is relatively independent of Warburg status (Fig. 6a). These observations suggest that stratifying cancers based on their Warburg metabolic status could be informative for clarifying the clinical efficacy of different HDAC inhibitors in combination with doxorubicin. Consistent with the yeast model, pan-HDAC inhibitors have been shown to enhance the anti-cancer efficacy of doxorubicin, as well as its cardiotoxicity [235, 236]. Interestingly, shRNA-mediated inhibition of *HDAC6* enhanced doxorubicin cytotoxicity in transformed cells [237], but protected against doxorubicin induced cardiotoxicity [238]—findings which could relate to the reduced toxicity associated with loss of class II vs. class I histone deacetylase function. Given that cancers can be driven by epigenetic plasticity [233, 234], such as could occur by loss of histone deacetylase function, information about the Warburg metabolic status could help clarify the likely impact of mutations in Sin3-type (class I) vs. HDA1-like (class II) histone deacetylase complexes. While speculative, this example illustrates the possible utility of yeast phenomic models to generate unbiased, systems-level experimental insights and may be of interest given the availability of HDAC6-specific inhibitors [239].

The examples of integrating yeast phenomic data with cancer cell line pharmacogenomics data to predict therapeutic efficacy are not limited to doxorubicin and/or the Warburg phenomenon. Analogous phenomic models could be generated for other cytotoxic agents and/or metabolic states, so long as the corresponding targets and buffering networks are conserved. Consistent with prior studies in yeast examining the question [169], we found the global correlation of human UES and OES with yeast deletion suppressors and enhancers to be low, further indicating the value of phenomic models for interpreting associations of gene expression with actual traits that are directly subject to natural selection. We anticipate that future integrative studies and ultimately clinical trials will further clarify how yeast phenomic studies can contribute to personalizing therapeutic efficacy for patients.

Although we focused the yeast model on predicting causality among differentially expressed genes that were associated with doxorubicin sensitivity in pharmacogenomic experiments, it can also be directly informative even if not correlated with gene expression. For example, genes required for DNA recombinational repair can be functionally regulated relatively independently of transcription [169]. Accordingly, these were detected more strongly from the yeast phenomics than the pharmacogenomics data.

In summary, we envision yeast phenomic drug-gene interaction models as a complement to existing cancer pharmacogenomics, providing an experimental platform to quantitatively derive drug-gene interaction network knowledge that can be integrated with DNA, RNA, protein, epigenetic, metabolite profiling, and/or cell proliferation data collected from tumors. Examples of experimental validation of the yeast model in cancer cell lines, as described through the manuscript, are summarized in Table 5. As a future step, predictions regarding treatment response of cancer to specific cytotoxic agents could be tested prospectively with patient samples, *in vitro* or in patient-derived xenograft models. Such a strategy could also be extended to before and after treatment(s) to understand how cancers evolve to buffer the drug’s toxicities. Analyses of patient-derived tumor organoids, for example, could include predictive modeling and experimental validation for the development of treatment strategies, both initially and with recurrence [240–242]. Though we have focused on a single cytotoxic agent for demonstrating the principle here, yeast phenomics would also accommodate modeling of combination chemotherapy, both for anti-cancer efficacy and host toxicity [243]. The influence of the Warburg effect or other influences of metabolic or nutrient status could also be integrated into such personalized models of cancer chemotherapy efficacy [244]. Thus, yeast phenomic models can be tailored to examine increasingly complex interactions: also including background genetic factors such as homologous recombination deficiency [144]. Yeast phenomics provides the experimental capabilities and genetic tractability to model genetic buffering networks relevant to human disease at high precision and resolution. However, advanced strategies for applying yeast phenomics to predict genetic influences on human disease biology remain to be developed.

**Table 5:** Literature supporting the yeast phenomic doxorubicin model

y/hGene	Process	PharmacoDB	Description	Ref	Doxorubicin relevance/validation
Enhancement/UES					
MUS81/MUS81	Topological change	MUS81	MUS81 structure-specific endonuclease subunit	[103, 181, 182]	Knockdown (shRNA) increases cisplatin and epirubicin (doxorubicin analog)-induced apoptosis of HCC cells.
SOD1/SOD1/CCS	Oxidative stress: complexes III and IV; protein import into mito matrix	SOD1; CCS	Superoxide dismutase	[8, 11, 72–74]	Doxorubicin causes depletion of cardiolipin and cytochrome c in cardiomyocytes, which reduces workload capacity and accelerates aging; TIM/TOM deficiency induces oxidative stress, oxidative stress enhances doxorubicin toxicity.
CDC8/DTYMK; CDC21/TYMS	dTTP biosynthetic process	NA	Thymidylate kinase; thymidylate synthetase	[127]	shRNA silencing of DTYMK enhances doxorubicin in cancer cell lines.
HDA1/HDAC6	Histone deacetylation	HDAC6	Histone deacetylase 6	[235, 236]	shRNA inhibition of HDAC6 enhances doxorubicin treatment; HDAC6 inhibition reduces cardiomyocyte toxicity.
Suppression/OES					
VPS53/VPS53	Sphingolipid homeostasis	VPS53	GARP complex subunit	[204]	Transfection with VPS53 transcript induces apoptosis and sensitizes cervical cancer cells to doxorubicin.
ELO2/3/ELOVL6	Fatty acid elongase activity; ceramide/phytosphingosine	ELOVL6	ELOVL fatty acid elongase 6; doxorubicin induces ceramide overproduction contributing to doxorubicin induced apoptosis	[12, 13, 150–156]	Loss of genes involved in sphingolipid/ceramide metabolism suppress doxorubicin cytotoxicity in our experiment; treatment with by *N*,*N*-dimethyl phytosphingosine sensitizes leukemia cells to doxorubicin.
POB3/SSRP1	FACT complex	SSRP1	Structure-specific recognition protein 1	[20]	FACT complex binds and “traps” disassembled chromatin in response to doxorubicin induced nucleosome disassembly, which induces chromatin damage.
ARP2/ACTR2	Actin cortical patch localization; APR2/3 complex	ACTR2	ARP2 actin-related protein 2 homolog	[158–162, 188]	ROCK1 deletion enhances doxorubicin resistance in fibroblasts by altering the actin cytoskeleton and protecting from apoptosis.

A major premise of precision medicine should be to comprehensively and quantitatively account for the contribution of genetic variance to phenotypes as well as influential interacting factors such as cell energy metabolism, age, drugs, or other environmental factors. This is an overwhelming challenging in humans, as functional genetic variation, as exemplified in cancer, is essentially too abundant to resolve at a systems level, particularly with respect to higher-order interactions as undoubtedly occur with combination chemotherapy. Thus, yeast phenomics, which can define gene interaction networks and genetic buffering in a systematic and global way [28, 245, 246], offers the potential to help resolve gene interaction networks that contribute to disease and therapeutic response [24, 247].

## Conclusions

A yeast phenomic model for the influence of Warburg metabolism on doxorubicin cytotoxicity revealed that glycolysis reduces the cellular reliance on genetic buffering networks. The model reports gene deletion-enhancing and deletion-suppression pathways and leverages yeast phenomic results to predict differentially expressed human genes that are causal in their association with doxorubicin killing from cancer cell line pharmacogenomics data. As such, this yeast model provides systems-level information about gene networks that buffer doxorubicin, serving as example of how Q-HTCP applied to the YKO/KD enables experimental designs to quantify gene interaction globally at high resolution; in this case, resolving how gene networks buffer doxorubicin cytotoxicity differentially with respect to Warburg metabolic status. Understanding cytotoxicity in terms of differential gene interaction networks has the potential to inform systems medicine by increasing the precision and rationale for personalizing the choice of cytotoxic agents, improving anti-tumor efficacy, and thereby reducing host toxicity. Yeast phenomics is a scalable experimental platform that can, in principle, be expanded to other cytotoxic chemotherapeutic agents and metabolic states, singly or in combination, thus providing versatile, tractable models to map drug-gene interaction networks and understand their complex influence on cell proliferation.

## Supplementary information

**Additional file 1: Figure S1.** Doxorubicin dose responses of the YKO/KD parental strains, BY4741a, BY4742alpha, and BY4743a/alpha diploid. **Figure S2.** Correlation between interaction scores based on L *vs.* other CPPs (K, r, and AUC), for both HLD and HLEG media. **Figure S3.** A summary of the first and second rounds of REMc. First round clusters are at the left end of each row of heatmap thumbnails; second round clusters derived from each first round cluster are ordered to the right by relative strength. Rows are grouped into panels by similarity in their gene interaction profiles. The columns in each heatmap have the same order from left to right (see inset panel), with K to the left and L to the right. Within the K and L groups, HLD is to the left and HLEG to the right. Within each of the CPP-media groupings, ‘shift’ (-) is left of the doxorubicin-gene interaction (+). (A) Respiration-specific enhancement. (B) Warburg-independent enhancement. (C) Glycolysis-specific enhancement. (D) HLD and HLEG suppression modules. (E) Respiratory deficiency. **Figure S4.** Doxorubicin-gene interaction profiles for selected mitochondrial GO terms. **Figure S5.** Deletion of mitochondrial genes tends to influence doxorubicin-gene interaction in a respiratory (HLEG media) more so than a glycolytic (HLD media) context. **Figure S6.** Heatmaps for GO terms comprised of overlapping gene sets. **Figure S7.** Pleiotropic phenotypic influences from genetic perturbation of ribonucleoprotein complex subunit organization. **Figure S8.** HLD-specific deletion enhancement of doxorubicin toxicity by evolutionarily conserved genes. *See also* Additional file 10: Table S13. **Figure S9.** GO term-specific heatmaps for *mRNA 3’ end processing* and *mRNA cleavage* gene interaction profiles. **Figure S10.** Suppression of doxorubicin cytotoxicity by perturbation of sphingolipid and ceramide metabolism. **Figure S11.** Deletion suppressing doxorubicin-gene interaction for nuclear pore and actin cortical patch functions is relatively Warburg-independent.

**Additional file 2. **Doxorubicin-gene interaction data; **Tables S1-S8. Tables S1-S4** are the genome-wide experiment: **Table S1.** YKO/KD strains in HLEG. **Table S2.** Reference cultures in HLEG. **Table S3.** YKO/KD strains in HLD. **Table S4.** Reference cultures in HLD. **Tables S5-S8** are the validation study: **Table S5.** YKO/KD strains in HLEG. **Table S6.** Reference cultures in HLEG. **Table S7.** YKO/KD strains in HLD. **Table S8.** Reference cultures in HLD.

**Additional file 3.** Interaction plots for HLEG. (A, B) Genome-wide and (C, D) validation analyses for (A, C) YKO/KD and (B, D) reference strains in HLEG. See also methods and Additional file 2.

**Additional file 4.** Interaction plots for HLD. (A, B) Genome-wide and (C, D) validation analyses. (A, C) YKO/KD and (B, D) reference strains in HLD media. See also methods and Additional file 2.

**Additional file 5. **REMc results with doxorubicin-gene interaction profile heatmaps and Gene Ontology enrichment (GO Term Finder; GTF) results. **File A** contains REMc results and associated gene interaction and shift data. **File B** is the heatmap representation of each REMc cluster after incorporating shift values and hierarchical clustering. **File C** contains the GTF results obtained for REMc clusters for the three ontologies – process, function, and component.

**Additional file 6. **Gene Ontology Term Averaging (GTA) results and interactive plots. **File A** contains all GTA values, cross-referenced with REMc-enriched terms. **File B** displays GTA values associated with above-threshold GTA scores (see note below) plotted for HLD *vs.* HLEG. GTA values for REMc-enriched terms are also included (regardless of whether |GTA score| >2). **File C** displays a subset of File B, containing only GO Terms with above-threshold GTA scores and that were enriched by REMc/GTF. **File D** reports GTA value using the K parameter. **Files B-D** should be opened in an Internet web browser so that embedded information from **File A** can be viewed by scrolling over points on the graphs. Subsets in each of the plots can be toggled off and on by clicking on the respective legend label. In the embedded information, X1 represents HLEG and X2 represents HLD information. Note: The GTA score threshold (for L) indicates that GTA-gtaSD > 2 for enhancers or GTA+gtaSD < -2 for suppressors, in at least one media.

**Additional file 7. **Systematic comparisons involving genome-wide studies of doxorubicin-gene interaction. **Table S9.** Genes with deletion-enhancing doxorubicin-gene interaction from *Xia et al. 2007* and *Westmoreland et al. 2009.* **Table S10.** Summary of experimental details associated with Table S9. **Table S11.** Test of enrichment for doxorubicin-gene interaction among genes encoding proteins predicted as substrates of the NatC complex. **Table S12.** Test of enrichment for doxorubicin-gene interaction among genes predicted to be regulated by conserved uORFs (*Cvijovic et al. 2007*).

**Additional file 8. **Quantitative summaries of REMc clusters. **File A** depicts REMc results, in terms of cluster distributions of L and K interaction (‘shift’ is not used for REMc and thus is not displayed), as a way to visualize cluster differences quantitatively. **File B** is organized by first round clusters and plots the change in p-value for significant terms with respect to round of clustering. Clusters derived from one another and sharing enrichment of the same GO term are connected by a line. Only GO terms with a background size of 500 or smaller are included. Scroll over a symbol to see embedded detail about each GO term. The square root of the p-value is used on the y-axis to evenly distribute data.

**Additional file 9.** GO term-specific heatmaps for REMc/GTF-enriched clusters. GO term-specific heatmaps for significant GO process terms were generated as described in methods and Figs. 3 and 4. Any related child terms are presented in subsequent pages of the parent file name. GO terms with more than 100 children, with 2 or fewer genes annotated to the term, or a file size over 300KB are not shown. All heatmaps are generated with the same layout (see Figs. 3 and 4).

**Additional file 10: Table S13.** HLD-specific gene deletion enhancement, not associated with ‘shift’ / growth deficiency. Data were selected for yeast-human homologs if the respective YKO/KD strains generated growth curves in both HLD and HLEG media (in the absence doxorubicin), and either of the following two sets of criteria were met: (1) HLD L interaction > 2 and HLEG L interaction < 2; these data were further filtered for *HLD L Interaction* - *HLD L Shift* > 4, and are presented in Additional file 1: Figure S8A.; or (2) *HLD L Interaction* – *HLEG L interaction* > 4 and HLEG K interaction > - 10; these data were further filtered for *HLD L Interaction* - *HLD L Shift* > 4, and are presented in Additional file 1: Figure S8B. Data included in Additional file 1: Figure S8 are indicated in the last column.

**Additional file 11.** Integration of yeast phenomic and cancer cell line pharmacogenomic data to predict human genes that modify doxorubicin toxicity in cancer cells. (A) Tables of UES and OES human genes and whether their yeast homologs were found to be deletion enhancing or deletion suppressing, respectively. (B-C) Overlap between the gCSI and GDSC1000 databases with regard to UES and OES human genes (B) across all tissues or (C) for individual tissues. Note: the intersection of UES with OES between gCSI and GDSC was used as a negative control for assessing UES and OES overlap. (D-E) Yeast phenomic doxorubicin-gene interaction profiles for homologs of human UES or OES genes, sub-classified according to interaction type (deletion enhancing or suppressing) and Warburg-dependence of the interaction, for the (D) gCSI or (E) GDSC1000 databases. Similar to Fig. 11, yeast-human homology relationships are shown to the left of heatmaps (blue - one to one; green - one to many; red - many to many). (F-I) Interactive plots for yeast-human homologs, comparing the p-value of human genes to L interaction scores for yeast counterparts in (F, G) HLD or (H, I) HLEG from (F, H) gCSI or (G, I) GDSC1000. For the standardized coefficient (‘estimate’; color gradient), a negative value (purple) indicates UES, while a positive value (orange) indicates OES. Thus, the model would predict causality for a human gene if its yeast homolog has a positive L interaction (deletion enhancing) and is colored purple (UES), or a negative L interaction (deletion suppressing) and colored orange (OES). Genes are only plotted if the human homolog was significant (p-value < 0.05).

**Additional file 12.** Comparisons between yeast studies of doxorubicin in the context of integrating cancer pharmacogenomics data. Overlapping and unique sets of genes reported from the different studies of doxorubicin, using the YKO/KD libraries, are assessed with regard to correlation with cancer pharmacogenomics data.

## Data Availability

All data generated or analyzed during this study are either included in this published article and supplementary files or will be freely supplied upon request.

## Glossary of terms

CPPs: Cell proliferation parameters: parameters of the logistic growth equation used to fit cell proliferation data obtained by Q-HTCP. The CPPs used to assess gene interaction in this study were *K* (carrying capacity) and *L* (time required to reach half of carrying capacity, K/2) [27, 28, 31, 33]
DAmP: Decreased abundance of mRNA production: refers to the method of making the yeast knockdown alleles, where the 3’ UTR of essential genes is disrupted, reducing mRNA stability and therefore gene dosage [248]
DE: Deletion enhancer: gene loss of function (knockout or knockdown) that results in enhancement/increase of drug sensitivity [28]
dNTP: Deoxyribonucleotide triphosphate
DS: Deletion suppressor: gene loss of function (knockout or knockdown) that results in suppression/reduction of drug sensitivity [28]
dsDNA: Double-stranded DNA
EMC: Endoplasmic reticulum membrane complex: an evolutionarily conserved protein complex involved in protein biogenesis via the ER [28, 85]
ER: Endoplasmic reticulum
ERMES: ER-mitochondria encounter structure: mitochondrial outer membrane complex regulated by the evolutionarily conserved Rho GTPase, Gem1 [249]
GARP complex: Golgi-associated retrograde protein complex [250]
gCSI: The Genentech Cell Line Screening Initiative: one of the two pharmacogenomics datasets curated by PharmacoDB that reported both cancer cell line gene expression and doxorubicin sensitivity data. Details regarding use of CellTiter Glo for pharmacological studies and Illumina RNA-seq for gene expression studies are provided at https://pharmacodb.pmgenomics.ca/datasets/4
GDSC1000: Genomics of Drug Sensitivity in Cancer: one of the two pharmacogenomics datasets curated by PharmacoDB that reported both cancer cell line gene expression and doxorubicin sensitivity data. Details regarding use of Syto60 for pharmacological studies and Affymetrix HG-U133A for gene expression studies are provided at (https://pharmacodb.pmgenomics.ca/datasets/5)
GO: Gene ontology
GTF: Gene ontology term finder: an algorithm to assess GO term enrichment among a list of genes; applied to REMc (clustering) results [35]
GTA: Gene ontology term averaging: an assessment of GO term function obtained by averaging the gene interaction values for all genes of a GO term
GTA value: Gene ontology term average value: see GTA
gtaSD: standard deviation of GTA value: see GTA
GTA score: (GTA value - gtaSD): see GTA
HDAC: Histone deacetylase complex
HLD: Human-like media with dextrose [27]: the yeast media used in this study to induce glycolytic metabolism
HLEG: Human-like media with ethanol and glycerol [27]: the yeast media used to induce respiratory metabolism
INT: Interaction score
m7G: 7-methylguanosine
MCM: Mini-chromosome maintenance
OES: Overexpressed in doxorubicin sensitive cells: refers to genes having an association of above average expression with doxorubicin sensitivity in pharmacogenomics data [38]
PharmacoDB: The resource used to analyze the gCSI and GDSC pharmacogenomics datasets [38]
Q-HTCP: Quantitative high throughput cell array phenotyping: a method of robotic imaging and image analysis that analyzes cell proliferation of yeast spot cultures arrayed onto agar media [31, 33]
Ref: Reference: the “reference” culture from which the YKO/KD strain library was derived
REMc: Recursive expectation maximization clustering: a probabilistic clustering algorithm that determines a discrete number of clusters from a data matrix [34]
ROS: Reactive oxygen species
RPA: Replication Protein A
SD: Standard deviation
SGD: Saccharomyces cerevisiae genome database
snoRNAs: Small nucleolar RNA
snRNA: Small nuclear RNA
t6A: Threonyl carbamoyl adenosine
UES: Underexpressed in doxorubicin sensitive cells: refers to genes having an association of below average expression with doxorubicin sensitivity in pharmacogenomics data [38]
uORF: Upstream open reading frames
YKO: Yeast knockout: complete gene deletion, constructed in a haploid cell for non-essential genes
YKD: Yeast knockdown: DAmP allele, constructed in a haploid cell for essential genes
YKO/KD: Yeast knockout or knockdown

## References

[1] Liberti MV, Locasale JW (2016). The Warburg effect: how does it benefit cancer cells?. Trends Biochem Sci.

[2] Schwartz L, Supuran CT, Alfarouk KO (2017). The Warburg effect and the hallmarks of cancer. Anticancer Agents Med Chem.

[3] Xu XD, Shao SX, Jiang HP, Cao YW, Wang YH, Yang XC, Wang YL, Wang XS, Niu HT (2015). Warburg effect or reverse Warburg effect? A review of cancer metabolism. Oncol Res Treat.

[4] Diaz-Ruiz R, Rigoulet M, Devin A (1807). The Warburg and Crabtree effects: on the origin of cancer cell energy metabolism and of yeast glucose repression. Biochim Biophys Acta.

[5] Diaz-Ruiz R, Uribe-Carvajal S, Devin A, Rigoulet M (2009). Tumor cell energy metabolism and its common features with yeast metabolism. Biochim Biophys Acta.

[6] Takeda M (1981). Glucose-induced inactivation of mitochondrial enzymes in the yeast Saccharomyces cerevisiae. Biochem J.

[7] Ventura-Clapier R, Garnier A, Veksler V, Joubert F (2011). Bioenergetics of the failing heart. Biochim Biophys Acta.

[8] Thorn CF, Oshiro C, Marsh S, Hernandez-Boussard T, McLeod H, Klein TE, Altman RB (2011). Doxorubicin pathways: pharmacodynamics and adverse effects. Pharmacogenet Genomics.

[9] Yang F, Teves SS, Kemp CJ, Henikoff S (2014). Doxorubicin, DNA torsion, and chromatin dynamics. Biochim Biophys Acta.

[10] Swift LP, Rephaeli A, Nudelman A, Phillips DR, Cutts SM (2006). Doxorubicin-DNA adducts induce a non-topoisomerase II-mediated form of cell death. Cancer Res.

[11] Angsutararux P, Luanpitpong S, Issaragrisil S (2015). Chemotherapy-induced cardiotoxicity: overview of the roles of oxidative stress. Oxid Med Cell Longev.

[12] Delpy E, Hatem SN, Andrieu N, de Vaumas C, Henaff M, Rucker-Martin C, Jaffrezou JP, Laurent G, Levade T, Mercadier JJ (1999). Doxorubicin induces slow ceramide accumulation and late apoptosis in cultured adult rat ventricular myocytes. Cardiovasc Res.

[13] Kawase M, Watanabe M, Kondo T, Yabu T, Taguchi Y, Umehara H, Uchiyama T, Mizuno K, Okazaki T (2002). Increase of ceramide in adriamycin-induced HL-60 cell apoptosis: detection by a novel anti-ceramide antibody. Biochim Biophys Acta.

[14] Chen SH, Chan NL, Hsieh TS (2013). New mechanistic and functional insights into DNA topoisomerases. Annu Rev Biochem.

[15] Tewey KM, Rowe TC, Yang L, Halligan BD, Liu LF (1984). Adriamycin-induced DNA damage mediated by mammalian DNA topoisomerase II. Science.

[16] Nitiss JL (2009). Targeting DNA topoisomerase II in cancer chemotherapy. Nat Rev Cancer.

[17] Coldwell KE, Cutts SM, Ognibene TJ, Henderson PT, Phillips DR (2008). Detection of Adriamycin-DNA adducts by accelerator mass spectrometry at clinically relevant Adriamycin concentrations. Nucleic Acids Res.

[18] Pang B, Qiao X, Janssen L, Velds A, Groothuis T, Kerkhoven R, Nieuwland M, Ovaa H, Rottenberg S, van Tellingen O (2013). Drug-induced histone eviction from open chromatin contributes to the chemotherapeutic effects of doxorubicin. Nat Commun.

[19] Yang F, Kemp CJ, Henikoff S (2013). Doxorubicin enhances nucleosome turnover around promoters. Curr Biol.

[20] Nesher E, Safina A, Aljahdali I, Portwood S, Wang ES, Koman I, Wang J, Gurova KV (2018). Role of chromatin damage and chromatin trapping of FACT in mediating the anticancer cytotoxicity of DNA-binding small-molecule drugs. Cancer Res.

[21] Singal PK, Iliskovic N (1998). Doxorubicin-induced cardiomyopathy. N Engl J Med.

[22] Zhang S, Liu X, Bawa-Khalfe T, Lu LS, Lyu YL, Liu LF, Yeh ET (2012). Identification of the molecular basis of doxorubicin-induced cardiotoxicity. Nat Med.

[23] Visscher H, Ross CJ, Rassekh SR, Barhdadi A, Dube MP, Al-Saloos H, Sandor GS, Caron HN, van Dalen EC, Kremer LC (2012). Pharmacogenomic prediction of anthracycline-induced cardiotoxicity in children. J Clin Oncol.

[24] Hartman JL, Garvik B, Hartwell L (2001). Principles for the buffering of genetic variation. Science.

[25] Hartman JL, Tippery NP (2004). Systematic quantification of gene interactions by phenotypic array analysis. Genome Biol.

[26] Hartman JL (2007). Buffering of deoxyribonucleotide pool homeostasis by threonine metabolism. Proc Natl Acad Sci U S A.

[27] Hartman JL, Stisher C, Outlaw DA, Guo J, Shah NA, Tian D, Santos SM, Rodgers JW, White RA (2015). Yeast phenomics: an experimental approach for modeling gene interaction networks that buffer disease. Genes (Basel).

[28] Louie RJ, Guo J, Rodgers JW, White R, Shah N, Pagant S, Kim P, Livstone M, Dolinski K, McKinney BA (2012). A yeast phenomic model for the gene interaction network modulating CFTR-∆F508 protein biogenesis. Genome Med.

[29] Veit G, Oliver K, Apaja PM, Perdomo D, Bidaud-Meynard A, Lin ST, Guo J, Icyuz M, Sorscher EJ, Hartman JI, Lukacs GL (2016). Ribosomal stalk protein silencing partially corrects the DeltaF508-CFTR functional expression defect. PLoS Biol.

[30] Hartman JL, Rodriguez R, Kaput J (2006). Genetic and molecular buffering of phenotypes. Nutritional Genomics: Discovering the Path to Personalized Nutrition. Volume 1.

[31] Shah NA, Laws RJ, Wardman B, Zhao LP, Hartman JL (2007). Accurate, precise modeling of cell proliferation kinetics from time-lapse imaging and automated image analysis of agar yeast culture arrays. BMC Syst Biol.

[32] Mani R, St Onge RP, Hartman JL, Giaever G, Roth FP (2008). Defining genetic interaction. Proc Natl Acad Sci U S A.

[33] Rodgers J, Guo J, Hartman JL (2014). Phenomic assessment of genetic buffering by kinetic analysis of cell arrays. Methods Mol Biol.

[34] Guo J, Tian D, McKinney BA, Hartman JL (2010). Recursive expectation-maximization clustering: a method for identifying buffering mechanisms composed of phenomic modules. Chaos.

[35] Boyle EI, Weng S, Gollub J, Jin H, Botstein D, Cherry JM, Sherlock G (2004). GO::TermFinder--open source software for accessing Gene Ontology information and finding significantly enriched Gene Ontology terms associated with a list of genes. Bioinformatics.

[36] Cherry JM, Hong EL, Amundsen C, Balakrishnan R, Binkley G, Chan ET, Christie KR, Costanzo MC, Dwight SS, Engel SR (2012). Saccharomyces Genome Database: the genomics resource of budding yeast. Nucleic Acids Res.

[37] Heinicke S, Livstone MS, Lu C, Oughtred R, Kang F, Angiuoli SV, White O, Botstein D, Dolinski K (2007). The Princeton Protein Orthology Database (P-POD): a comparative genomics analysis tool for biologists. PLoS One.

[38] Smirnov P, Kofia V, Maru A, Freeman M, Ho C, El-Hachem N, Adam GA, Ba-Alawi W, Safikhani Z, Haibe-Kains B (2018). PharmacoDB: an integrative database for mining in vitro anticancer drug screening studies. Nucleic Acids Res.

[39] Smirnov P, Safikhani Z, El-Hachem N, Wang D, She A, Olsen C, Freeman M, Selby H, Gendoo DM, Grossmann P (2016). PharmacoGx: an R package for analysis of large pharmacogenomic datasets. Bioinformatics.

[40] Durinck S, Moreau Y, Kasprzyk A, Davis S, De Moor B, Brazma A, Huber W (2005). BioMart and Bioconductor: a powerful link between biological databases and microarray data analysis. Bioinformatics.

[41] Durinck S, Spellman PT, Birney E, Huber W (2009). Mapping identifiers for the integration of genomic datasets with the R/Bioconductor package biomaRt. Nat Protoc.

[42] Zerbino DR, Achuthan P, Akanni W, Amode MR, Barrell D, Bhai J, Billis K, Cummins C, Gall A, Giron CG (2018). Ensembl 2018. Nucleic Acids Res.

[43] Xia L, Jaafar L, Cashikar A, Flores-Rozas H (2007). Identification of genes required for protection from doxorubicin by a genome-wide screen in Saccharomyces cerevisiae. Cancer Res.

[44] Westmoreland TJ, Wickramasekara SM, Guo AY, Selim AL, Winsor TS, Greenleaf AL, Blackwell KL, Olson JA, Marks JR, Bennett CB (2009). Comparative genome-wide screening identifies a conserved doxorubicin repair network that is diploid specific in Saccharomyces cerevisiae. PLoS One.

[45] Smith DL, Maharrey CH, Carey CR, White RA, Hartman JL (2016). Gene-nutrient interaction markedly influences yeast chronological lifespan. Exp Gerontol.

[46] Amin AD, Vishnoi N, Prochasson P (2013). A global requirement for the HIR complex in the assembly of chromatin. Biochim Biophys Acta.

[47] Green EM, Antczak AJ, Bailey AO, Franco AA, Wu KJ, Yates JR, Kaufman PD (2005). Replication-independent histone deposition by the HIR complex and Asf1. Curr Biol.

[48] Imbeault D, Gamar L, Rufiange A, Paquet E, Nourani A (2008). The Rtt106 histone chaperone is functionally linked to transcription elongation and is involved in the regulation of spurious transcription from cryptic promoters in yeast. J Biol Chem.

[49] Emili A, Schieltz DM, Yates JR, Hartwell LH (2001). Dynamic interaction of DNA damage checkpoint protein Rad53 with chromatin assembly factor Asf1. Mol Cell.

[50] Kaufman PD, Kobayashi R, Stillman B (1997). Ultraviolet radiation sensitivity and reduction of telomeric silencing in Saccharomyces cerevisiae cells lacking chromatin assembly factor-I. Genes Dev.

[51] Bao Y, Shen X (2011). SnapShot: chromatin remodeling: INO80 and SWR1. Cell.

[52] Zhang H, Roberts DN, Cairns BR (2005). Genome-wide dynamics of Htz1, a histone H2A variant that poises repressed/basal promoters for activation through histone loss. Cell.

[53] Raisner RM, Hartley PD, Meneghini MD, Bao MZ, Liu CL, Schreiber SL, Rando OJ, Madhani HD (2005). Histone variant H2A.Z marks the 5’ ends of both active and inactive genes in euchromatin. Cell.

[54] Guillemette B, Bataille AR, Gevry N, Adam M, Blanchette M, Robert F, Gaudreau L (2005). Variant histone H2A.Z is globally localized to the promoters of inactive yeast genes and regulates nucleosome positioning. PLoS Biol.

[55] Seeber A, Hauer M, Gasser SM (2013). Nucleosome remodelers in double-strand break repair. Curr Opin Genet Dev.

[56] Meneghini MD, Wu M, Madhani HD (2003). Conserved histone variant H2A.Z protects euchromatin from the ectopic spread of silent heterochromatin. Cell.

[57] Seto E, Yoshida M (2014). Erasers of histone acetylation: the histone deacetylase enzymes. Cold Spring Harb Perspect Biol.

[58] Rundlett SE, Carmen AA, Kobayashi R, Bavykin S, Turner BM, Grunstein M (1996). HDA1 and RPD3 are members of distinct yeast histone deacetylase complexes that regulate silencing and transcription. Proc Natl Acad Sci U S A.

[59] Schneider J, Bajwa P, Johnson FC, Bhaumik SR, Shilatifard A (2006). Rtt109 is required for proper H3K56 acetylation: a chromatin mark associated with the elongating RNA polymerase II. J Biol Chem.

[60] Masumoto H, Hawke D, Kobayashi R, Verreault A (2005). A role for cell-cycle-regulated histone H3 lysine 56 acetylation in the DNA damage response. Nature.

[61] Gartenberg MR, Smith JS (2016). The nuts and bolts of transcriptionally silent chromatin in Saccharomyces cerevisiae. Genetics.

[62] Greer EL, Shi Y (2012). Histone methylation: a dynamic mark in health, disease and inheritance. Nat Rev Genet.

[63] Miller T, Krogan NJ, Dover J, Erdjument-Bromage H, Tempst P, Johnston M, Greenblatt JF, Shilatifard A (2001). COMPASS: a complex of proteins associated with a trithorax-related SET domain protein. Proc Natl Acad Sci U S A.

[64] Krogan NJ, Dover J, Khorrami S, Greenblatt JF, Schneider J, Johnston M, Shilatifard A (2002). COMPASS, a histone H3 (Lysine 4) methyltransferase required for telomeric silencing of gene expression. J Biol Chem.

[65] Roguev A, Schaft D, Shevchenko A, Pijnappel WW, Wilm M, Aasland R, Stewart AF (2001). The Saccharomyces cerevisiae Set1 complex includes an Ash2 homologue and methylates histone 3 lysine 4. EMBO J.

[66] Shilatifard A (2012). The COMPASS family of histone H3K4 methylases: mechanisms of regulation in development and disease pathogenesis. Annu Rev Biochem.

[67] Krogan NJ, Dover J, Wood A, Schneider J, Heidt J, Boateng MA, Dean K, Ryan OW, Golshani A, Johnston M (2003). The Paf1 complex is required for histone H3 methylation by COMPASS and Dot1p: linking transcriptional elongation to histone methylation. Mol Cell.

[68] Ng HH, Dole S, Struhl K (2003). The Rtf1 component of the Paf1 transcriptional elongation complex is required for ubiquitination of histone H2B. J Biol Chem.

[69] Dover J, Schneider J, Tawiah-Boateng MA, Wood A, Dean K, Johnston M, Shilatifard A (2002). Methylation of histone H3 by COMPASS requires ubiquitination of histone H2B by Rad6. J Biol Chem.

[70] Sun ZW, Allis CD (2002). Ubiquitination of histone H2B regulates H3 methylation and gene silencing in yeast. Nature.

[71] Wood A, Schneider J, Dover J, Johnston M, Shilatifard A (2003). The Paf1 complex is essential for histone monoubiquitination by the Rad6-Bre1 complex, which signals for histone methylation by COMPASS and Dot1p. J Biol Chem.

[72] Giannattasio M, Lazzaro F, Plevani P, Muzi-Falconi M (2005). The DNA damage checkpoint response requires histone H2B ubiquitination by Rad6-Bre1 and H3 methylation by Dot1. J Biol Chem.

[73] Nicolay K, de Kruijff B (1987). Effects of adriamycin on respiratory chain activities in mitochondria from rat liver, rat heart and bovine heart. Evidence for a preferential inhibition of complex III and IV. Biochim Biophys Acta.

[74] Pereira GC, Pereira SP, Tavares LC, Carvalho FS, Magalhaes-Novais S, Barbosa IA, Santos MS, Bjork J, Moreno AJ, Wallace KB, Oliveira PJ (2016). Cardiac cytochrome c and cardiolipin depletion during anthracycline-induced chronic depression of mitochondrial function. Mitochondrion.

[75] MacKenzie JA, Payne RM (2007). Mitochondrial protein import and human health and disease. Biochim Biophys Acta.

[76] Taanman JW, Capaldi RA (1993). Subunit VIa of yeast cytochrome c oxidase is not necessary for assembly of the enzyme complex but modulates the enzyme activity. Isolation and characterization of the nuclear-coded gene. J Biol Chem.

[77] Vukotic M, Oeljeklaus S, Wiese S, Vogtle FN, Meisinger C, Meyer HE, Zieseniss A, Katschinski DM, Jans DC, Jakobs S (2012). Rcf1 mediates cytochrome oxidase assembly and respirasome formation, revealing heterogeneity of the enzyme complex. Cell Metab.

[78] Patterson TE, Poyton RO (1986). COX8, the structural gene for yeast cytochrome c oxidase subunit VIII. DNA sequence and gene disruption indicate that subunit VIII is required for maximal levels of cellular respiration and is derived from a precursor which is extended at both its NH2 and COOH termini. J Biol Chem.

[79] Herzig S, Raemy E, Montessuit S, Veuthey JL, Zamboni N, Westermann B, Kunji ER, Martinou JC (2012). Identification and functional expression of the mitochondrial pyruvate carrier. Science.

[80] Bricker DK, Taylor EB, Schell JC, Orsak T, Boutron A, Chen YC, Cox JE, Cardon CM, Van Vranken JG, Dephoure N (2012). A mitochondrial pyruvate carrier required for pyruvate uptake in yeast, Drosophila, and humans. Science.

[81] Cui Y, Zhao S, Wang J, Wang X, Gao B, Fan Q, Sun F, Zhou B (2015). A novel mitochondrial carrier protein Mme1 acts as a yeast mitochondrial magnesium exporter. Biochim Biophys Acta.

[82] Lemaire C, Guibet-Grandmougin F, Angles D, Dujardin G, Bonnefoy N (2004). A yeast mitochondrial membrane methyltransferase-like protein can compensate for oxa1 mutations. J Biol Chem.

[83] Bauerschmitt H, Funes S, Herrmann JM (2008). The membrane-bound GTPase Guf1 promotes mitochondrial protein synthesis under suboptimal conditions. J Biol Chem.

[84] Pfanner N, van der Laan M, Amati P, Capaldi RA, Caudy AA, Chacinska A, Darshi M, Deckers M, Hoppins S, Icho T (2014). Uniform nomenclature for the mitochondrial contact site and cristae organizing system. J Cell Biol.

[85] Jonikas MC, Collins SR, Denic V, Oh E, Quan EM, Schmid V, Weibezahn J, Schwappach B, Walter P, Weissman JS, Schuldiner M (2009). Comprehensive characterization of genes required for protein folding in the endoplasmic reticulum. Science.

[86] Lahiri S, Chao JT, Tavassoli S, Wong AK, Choudhary V, Young BP, Loewen CJ, Prinz WA (2014). A conserved endoplasmic reticulum membrane protein complex (EMC) facilitates phospholipid transfer from the ER to mitochondria. PLoS Biol.

[87] van der Veen AG, Ploegh HL (2012). Ubiquitin-like proteins. Annu Rev Biochem.

[88] Judes A, Bruch A, Klassen R, Helm M, Schaffrath R (2016). Sulfur transfer and activation by ubiquitin-like modifier system Uba4*Urm1 link protein urmylation and tRNA thiolation in yeast. Microb Cell.

[89] Nakai Y, Nakai M, Hayashi H (2008). Thio-modification of yeast cytosolic tRNA requires a ubiquitin-related system that resembles bacterial sulfur transfer systems. J Biol Chem.

[90] Noma A, Sakaguchi Y, Suzuki T (2009). Mechanistic characterization of the sulfur-relay system for eukaryotic 2-thiouridine biogenesis at tRNA wobble positions. Nucleic Acids Res.

[91] Polevoda B, Norbeck J, Takakura H, Blomberg A, Sherman F (1999). Identification and specificities of N-terminal acetyltransferases from Saccharomyces cerevisiae. EMBO J.

[92] Varland S, Osberg C, Arnesen T (2015). N-terminal modifications of cellular proteins: the enzymes involved, their substrate specificities and biological effects. Proteomics.

[93] Arnesen T, Van Damme P, Polevoda B, Helsens K, Evjenth R, Colaert N, Varhaug JE, Vandekerckhove J, Lillehaug JR, Sherman F, Gevaert K (2009). Proteomics analyses reveal the evolutionary conservation and divergence of N-terminal acetyltransferases from yeast and humans. Proc Natl Acad Sci U S A.

[94] Setty SR, Strochlic TI, Tong AH, Boone C, Burd CG (2004). Golgi targeting of ARF-like GTPase Arl3p requires its Nalpha-acetylation and the integral membrane protein Sys1p. Nat Cell Biol.

[95] Behnia R, Panic B, Whyte JR, Munro S (2004). Targeting of the Arf-like GTPase Arl3p to the Golgi requires N-terminal acetylation and the membrane protein Sys1p. Nat Cell Biol.

[96] Behnia R, Barr FA, Flanagan JJ, Barlowe C, Munro S (2007). The yeast orthologue of GRASP65 forms a complex with a coiled-coil protein that contributes to ER to Golgi traffic. J Cell Biol.

[97] Murthi A, Hopper AK (2005). Genome-wide screen for inner nuclear membrane protein targeting in Saccharomyces cerevisiae: roles for N-acetylation and an integral membrane protein. Genetics.

[98] Aksnes H, Osberg C, Arnesen T (2013). N-terminal acetylation by NatC is not a general determinant for substrate subcellular localization in Saccharomyces cerevisiae. PLoS One.

[99] Garinther WI, Schultz MC (1997). Topoisomerase function during replication-independent chromatin assembly in yeast. Mol Cell Biol.

[100] Champoux JJ (2001). DNA topoisomerases: structure, function, and mechanism. Annu Rev Biochem.

[101] Goto T, Wang JC (1985). Cloning of yeast TOP1, the gene encoding DNA topoisomerase I, and construction of mutants defective in both DNA topoisomerase I and DNA topoisomerase II. Proc Natl Acad Sci U S A.

[102] Thrash C, Bankier AT, Barrell BG, Sternglanz R (1985). Cloning, characterization, and sequence of the yeast DNA topoisomerase I gene. Proc Natl Acad Sci U S A.

[103] Wang JC (1996). DNA topoisomerases. Annu Rev Biochem.

[104] Kaliraman V, Mullen JR, Fricke WM, Bastin-Shanower SA, Brill SJ (2001). Functional overlap between Sgs1-Top3 and the Mms4-Mus81 endonuclease. Genes Dev.

[105] Cejka P, Plank JL, Dombrowski CC, Kowalczykowski SC (2012). Decatenation of DNA by the S. cerevisiae Sgs1-Top3-Rmi1 and RPA complex: a mechanism for disentangling chromosomes. Mol Cell.

[106] Rottensteiner H, Kal AJ, Hamilton B, Ruis H, Tabak HF (1997). A heterodimer of the Zn2Cys6 transcription factors Pip2p and Oaf1p controls induction of genes encoding peroxisomal proteins in Saccharomyces cerevisiae. Eur J Biochem.

[107] Karpichev IV, Luo Y, Marians RC, Small GM (1997). A complex containing two transcription factors regulates peroxisome proliferation and the coordinate induction of beta-oxidation enzymes in Saccharomyces cerevisiae. Mol Cell Biol.

[108] Sayed-Ahmed MM, Salman TM, Gaballah HE, Abou El-Naga SA, Nicolai R, Calvani M (2001). Propionyl-L-carnitine as protector against adriamycin-induced cardiomyopathy. Pharmacol Res.

[109] Gunisova S, Hronova V, Mohammad MP, Hinnebusch AG, Valasek LS (2018). Please do not recycle! Translation reinitiation in microbes and higher eukaryotes. FEMS Microbiol Rev.

[110] Cvijovic M, Dalevi D, Bilsland E, Kemp GJ, Sunnerhagen P (2007). Identification of putative regulatory upstream ORFs in the yeast genome using heuristics and evolutionary conservation. BMC Bioinformatics.

[111] Murray-Stewart TR, Woster PM, Casero RA (2016). Targeting polyamine metabolism for cancer therapy and prevention. Biochem J.

[112] Casero RA, Celano P, Ervin SJ, Porter CW, Bergeron RJ, Libby PR (1989). Differential induction of spermidine/spermine N1-acetyltransferase in human lung cancer cells by the bis (ethyl) polyamine analogues. Cancer Res.

[113] Ulrich S, Huwiler A, Loitsch S, Schmidt H, Stein JM (2007). De novo ceramide biosynthesis is associated with resveratrol-induced inhibition of ornithine decarboxylase activity. Biochem Pharmacol.

[114] Srinivasan M, Mehta P, Yu Y, Prugar E, Koonin EV, Karzai AW, Sternglanz R (2011). The highly conserved KEOPS/EKC complex is essential for a universal tRNA modification, t6A. EMBO J.

[115] Murphy FV, Ramakrishnan V, Malkiewicz A, Agris PF (2004). The role of modifications in codon discrimination by tRNA (Lys)UUU. Nat Struct Mol Biol.

[116] Downey M, Houlsworth R, Maringele L, Rollie A, Brehme M, Galicia S, Guillard S, Partington M, Zubko MK, Krogan NJ (2006). A genome-wide screen identifies the evolutionarily conserved KEOPS complex as a telomere regulator. Cell.

[117] Kisseleva-Romanova E, Lopreiato R, Baudin-Baillieu A, Rousselle JC, Ilan L, Hofmann K, Namane A, Mann C, Libri D (2006). Yeast homolog of a cancer-testis antigen defines a new transcription complex. EMBO J.

[118] Gaudet P, Livstone MS, Lewis SE, Thomas PD (2011). Phylogenetic-based propagation of functional annotations within the Gene Ontology consortium. Brief Bioinform.

[119] Cao J, Hou P, Chen J, Wang P, Wang W, Liu W, Liu C, He X (2017). The overexpression and prognostic role of DCAF13 in hepatocellular carcinoma. Tumour Biol.

[120] Doma MK, Parker R (2006). Endonucleolytic cleavage of eukaryotic mRNAs with stalls in translation elongation. Nature.

[121] Tsuboi T, Kuroha K, Kudo K, Makino S, Inoue E, Kashima I, Inada T (2012). Dom34:hbs1 plays a general role in quality-control systems by dissociation of a stalled ribosome at the 3' end of aberrant mRNA. Mol Cell.

[122] Shoemaker CJ, Eyler DE, Green R (2010). Dom34:Hbs1 promotes subunit dissociation and peptidyl-tRNA drop-off to initiate no-go decay. Science.

[123] Luo J, Emanuele MJ, Li D, Creighton CJ, Schlabach MR, Westbrook TF, Wong KK, Elledge SJ (2009). A genome-wide RNAi screen identifies multiple synthetic lethal interactions with the Ras oncogene. Cell.

[124] Cohen M, Stutz F, Belgareh N, Haguenauer-Tsapis R, Dargemont C (2003). Ubp3 requires a cofactor, Bre5, to specifically de-ubiquitinate the COPII protein, Sec23. Nat Cell Biol.

[125] Cohen M, Stutz F, Dargemont C (2003). Deubiquitination, a new player in Golgi to endoplasmic reticulum retrograde transport. J Biol Chem.

[126] Dean N, Zhang YB, Poster JB (1997). The VRG4 gene is required for GDP-mannose transport into the lumen of the Golgi in the yeast, Saccharomyces cerevisiae. J Biol Chem.

[127] Ballou L, Hitzeman RA, Lewis MS, Ballou CE (1991). Vanadate-resistant yeast mutants are defective in protein glycosylation. Proc Natl Acad Sci U S A.

[128] Hu CM, Chang ZF (2008). Synthetic lethality by lentiviral short hairpin RNA silencing of thymidylate kinase and doxorubicin in colon cancer cells regardless of the p53 status. Cancer Res.

[129] Safran M, Dalah I, Alexander J, Rosen N, Iny Stein T, Shmoish M, Nativ N, Bahir I, Doniger T, Krug H (2010). GeneCards Version 3: the human gene integrator. Database (Oxford).

[130] Vizeacoumar FJ, Arnold R, Vizeacoumar FS, Chandrashekhar M, Buzina A, Young JT, Kwan JH, Sayad A, Mero P, Lawo S (2013). A negative genetic interaction map in isogenic cancer cell lines reveals cancer cell vulnerabilities. Mol Syst Biol.

[131] Krastev DB, Slabicki M, Paszkowski-Rogacz M, Hubner NC, Junqueira M, Shevchenko A, Mann M, Neugebauer KM, Buchholz F (2011). A systematic RNAi synthetic interaction screen reveals a link between p53 and snoRNP assembly. Nat Cell Biol.

[132] Kohler A, Schneider M, Cabal GG, Nehrbass U, Hurt E (2008). Yeast Ataxin-7 links histone deubiquitination with gene gating and mRNA export. Nat Cell Biol.

[133] Hurov KE, Cotta-Ramusino C, Elledge SJ (2010). A genetic screen identifies the Triple T complex required for DNA damage signaling and ATM and ATR stability. Genes Dev.

[134] Mouaikel J, Verheggen C, Bertrand E, Tazi J, Bordonne R (2002). Hypermethylation of the cap structure of both yeast snRNAs and snoRNAs requires a conserved methyltransferase that is localized to the nucleolus. Mol Cell.

[135] Boon KL, Kos M (2010). Deletion of Swm2p selectively impairs trimethylation of snRNAs by trimethylguanosine synthase (Tgs1p). FEBS Lett.

[136] Colau G, Thiry M, Leduc V, Bordonne R, Lafontaine DL (2004). The small nucle (ol) ar RNA cap trimethyltransferase is required for ribosome synthesis and intact nucleolar morphology. Mol Cell Biol.

[137] D'Ambrosio C, Schmidt CK, Katou Y, Kelly G, Itoh T, Shirahige K, Uhlmann F (2008). Identification of cis-acting sites for condensin loading onto budding yeast chromosomes. Genes Dev.

[138] Haeusler RA, Pratt-Hyatt M, Good PD, Gipson TA, Engelke DR (2008). Clustering of yeast tRNA genes is mediated by specific association of condensin with tRNA gene transcription complexes. Genes Dev.

[139] Wang HZ, Yang SH, Li GY, Cao X (2018). Subunits of human condensins are potential therapeutic targets for cancers. Cell Div.

[140] Tay Z, Eng RJ, Sajiki K, Lim KK, Tang MY, Yanagida M, Chen ES (2013). Cellular robustness conferred by genetic crosstalk underlies resistance against chemotherapeutic drug doxorubicin in fission yeast. PLoS One.

[141] Nguyen TT, Lim JS, Tang RM, Zhang L, Chen ES (2015). Fitness profiling links topoisomerase II regulation of centromeric integrity to doxorubicin resistance in fission yeast. Sci Rep.

[142] Ding J, Yu C, Sui Y, Wang L, Yang Y, Wang F, Yao H, Xing F, Liu H, Li Y, et al. The chromatin remodeling protein INO80 contributes to the removal of H2A.Z at the p53-binding site of the p21 gene in response to doxorubicin. FEBS J. 2018. 10.1111/febs.14615 30055111

[143] Galanos P, Pappas G, Polyzos A, Kotsinas A, Svolaki I, Giakoumakis NN, Glytsou C, Pateras IS, Swain U, Souliotis VL (2018). Mutational signatures reveal the role of RAD52 in p53-independent p21-driven genomic instability. Genome Biol.

[144] Konstantinopoulos PA, Ceccaldi R, Shapiro GI, D’Andrea AD (2015). Homologous recombination deficiency: exploiting the fundamental vulnerability of ovarian cancer. Cancer Discov.

[145] Mimura S, Yamaguchi T, Ishii S, Noro E, Katsura T, Obuse C, Kamura T (2010). Cul8/Rtt101 forms a variety of protein complexes that regulate DNA damage response and transcriptional silencing. J Biol Chem.

[146] Zhang J, Shi D, Li X, Ding L, Tang J, Liu C, Shirahige K, Cao Q, Lou H (2017). Rtt101-Mms1-Mms22 coordinates replication-coupled sister chromatid cohesion and nucleosome assembly. EMBO Rep.

[147] Roberg KJ, Bickel S, Rowley N, Kaiser CA (1997). Control of amino acid permease sorting in the late secretory pathway of Saccharomyces cerevisiae by SEC13, LST4, LST7 and LST8. Genetics.

[148] Symeonidou IE, Taraviras S, Lygerou Z (2012). Control over DNA replication in time and space. FEBS Lett.

[149] Tran NQ, Pham XH, Tuteja R, Tuteja N (2011). Inhibition of unwinding and ATPase activities of pea MCM6 DNA helicase by actinomycin and nogalamycin. Plant Signal Behav.

[150] Frohlich F, Petit C, Kory N, Christiano R, Hannibal-Bach HK, Graham M, Liu X, Ejsing CS, Farese RV, Walther TC. The GARP complex is required for cellular sphingolipid homeostasis. Elife. 2015;4. 10.7554/eLife.08712 PMC4600884 26357016

[151] Oh CS, Toke DA, Mandala S, Martin CE (1997). ELO2 and ELO3, homologues of the Saccharomyces cerevisiae ELO1 gene, function in fatty acid elongation and are required for sphingolipid formation. J Biol Chem.

[152] Hwang S, Gustafsson HT, O'Sullivan C, Bisceglia G, Huang X, Klose C, Schevchenko A, Dickson RC, Cavaliere P, Dephoure N, Torres EM (2017). Serine-dependent sphingolipid synthesis is a metabolic liability of aneuploid cells. Cell Rep.

[153] Ponnusamy S, Meyers-Needham M, Senkal CE, Saddoughi SA, Sentelle D, Selvam SP, Salas A, Ogretmen B (2010). Sphingolipids and cancer: ceramide and sphingosine-1-phosphate in the regulation of cell death and drug resistance. Future Oncol.

[154] Swinnen E, Wilms T, Idkowiak-Baldys J, Smets B, De Snijder P, Accardo S, Ghillebert R, Thevissen K, Cammue B, De Vos D (2014). The protein kinase Sch9 is a key regulator of sphingolipid metabolism in Saccharomyces cerevisiae. Mol Biol Cell.

[155] Kim BM, Choi YJ, Lee YH, Joe YA, Hong SH (2010). N,N-Dimethyl phytosphingosine sensitizes HL-60/MX2, a multidrug-resistant variant of HL-60 cells, to doxorubicin-induced cytotoxicity through ROS-mediated release of cytochrome c and AIF. Apoptosis.

[156] Liu YY, Yu JY, Yin D, Patwardhan GA, Gupta V, Hirabayashi Y, Holleran WM, Giuliano AE, Jazwinski SM, Gouaze-Andersson V (2008). A role for ceramide in driving cancer cell resistance to doxorubicin. FASEB J.

[157] Martinez R, Navarro R, Lacort M, Ruiz-Sanz JI, Ruiz-Larrea MB (2009). Doxorubicin induces ceramide and diacylglycerol accumulation in rat hepatocytes through independent routes. Toxicol Lett.

[158] Ibarra A, Hetzer MW (2015). Nuclear pore proteins and the control of genome functions. Genes Dev.

[159] Wei L, Surma M, Gough G, Shi S, Lambert-Cheatham N, Chang J, Shi J (2015). Dissecting the mechanisms of doxorubicin and oxidative stress-induced cytotoxicity: the involvement of actin cytoskeleton and ROCK1. PLoS One.

[160] Shi J, Wu X, Surma M, Vemula S, Zhang L, Yang Y, Kapur R, Wei L (2013). Distinct roles for ROCK1 and ROCK2 in the regulation of cell detachment. Cell Death Dis.

[161] Colombo R, Necco A, Vailati G, Saracco B, Milzani A, Scari G (1984). Doxorubicin affects actin assembly in vitro. Cell Biol Int Rep.

[162] Colombo R, Necco A, Vailati G, Milzani A (1988). Dose-dependence of doxorubicin effect on actin assembly in vitro. Exp Mol Pathol.

[163] Colombo R, Dalle Donne I, Milzani A (1990). Metal ions modulate the effect of doxorubicin on actin assembly. Cancer Biochem Biophys.

[164] Chen XJ, Clark-Walker GD (2000). The petite mutation in yeasts: 50 years on. Int Rev Cytol.

[165] Courtney KD, Bezwada D, Mashimo T, Pichumani K, Vemireddy V, Funk AM, Wimberly J, McNeil SS, Kapur P, Lotan Y (2018). Isotope tracing of human clear cell renal cell carcinomas demonstrates suppressed glucose oxidation in vivo. Cell Metab.

[166] Sanderson SM, Locasale JW (2018). Revisiting the Warburg effect: some tumors hold their breath. Cell Metab.

[167] O'Neil NJ, Bailey ML, Hieter P (2017). Synthetic lethality and cancer. Nat Rev Genet.

[168] McGary KL, Park TJ, Woods JO, Cha HJ, Wallingford JB, Marcotte EM (2010). Systematic discovery of nonobvious human disease models through orthologous phenotypes. Proc Natl Acad Sci U S A.

[169] Birrell GW, Brown JA, Wu HI, Giaever G, Chu AM, Davis RW, Brown JM (2002). Transcriptional response of Saccharomyces cerevisiae to DNA-damaging agents does not identify the genes that protect against these agents. Proc Natl Acad Sci U S A.

[170] Garnett MJ, Edelman EJ, Heidorn SJ, Greenman CD, Dastur A, Lau KW, Greninger P, Thompson IR, Luo X, Soares J (2012). Systematic identification of genomic markers of drug sensitivity in cancer cells. Nature.

[171] Yang W, Soares J, Greninger P, Edelman EJ, Lightfoot H, Forbes S, Bindal N, Beare D, Smith JA, Thompson IR (2013). Genomics of Drug Sensitivity in Cancer (GDSC): a resource for therapeutic biomarker discovery in cancer cells. Nucleic Acids Res.

[172] Klijn C, Durinck S, Stawiski EW, Haverty PM, Jiang Z, Liu H, Degenhardt J, Mayba O, Gnad F, Liu J (2015). A comprehensive transcriptional portrait of human cancer cell lines. Nat Biotechnol.

[173] Haverty PM, Lin E, Tan J, Yu Y, Lam B, Lianoglou S, Neve RM, Martin S, Settleman J, Yauch RL, Bourgon R (2016). Reproducible pharmacogenomic profiling of cancer cell line panels. Nature.

[174] Revill K, Wang T, Lachenmayer A, Kojima K, Harrington A, Li J, Hoshida Y, Llovet JM, Powers S (2013). Genome-wide methylation analysis and epigenetic unmasking identify tumor suppressor genes in hepatocellular carcinoma. Gastroenterology.

[175] Saito S, Matsui H, Kawano M, Kumagai K, Tomishige N, Hanada K, Echigo S, Tamura S, Kobayashi T (2008). Protein phosphatase 2Cepsilon is an endoplasmic reticulum integral membrane protein that dephosphorylates the ceramide transport protein CERT to enhance its association with organelle membranes. J Biol Chem.

[176] Thean LF, Loi C, Ho KS, Koh PK, Eu KW, Cheah PY (2010). Genome-wide scan identifies a copy number variable region at 3q26 that regulates PPM1L in APC mutation-negative familial colorectal cancer patients. Genes Chromosomes Cancer.

[177] Leithner K, Wohlkoenig C, Stacher E, Lindenmann J, Hofmann NA, Galle B, Guelly C, Quehenberger F, Stiegler P, Smolle-Juttner FM (2014). Hypoxia increases membrane metallo-endopeptidase expression in a novel lung cancer ex vivo model - role of tumor stroma cells. BMC Cancer.

[178] Park JY, Kim SA, Chung JW, Bang S, Park SW, Paik YK, Song SY (2011). Proteomic analysis of pancreatic juice for the identification of biomarkers of pancreatic cancer. J Cancer Res Clin Oncol.

[179] Corso J, Pan KT, Walter R, Doebele C, Mohr S, Bohnenberger H, Strobel P, Lenz C, Slabicki M, Hullein J (2016). Elucidation of tonic and activated B-cell receptor signaling in Burkitt's lymphoma provides insights into regulation of cell survival. Proc Natl Acad Sci U S A.

[180] Wu F, Su SC, Tan GQ, Yan L, Li TY, Zhang HL, Yu JS, Wang BL (2017). Mus81 knockdown sensitizes colon cancer cells to chemotherapeutic drugs by activating CHK1 pathway. Clin Res Hepatol Gastroenterol.

[181] Kim HJ, Jo MJ, Kim BR, Kim JL, Jeong YA, Na YJ, Park SH, Lee SY, Lee DH, Lee HS (2017). Reactive oxygen species modulator-1 (Romo1) predicts unfavorable prognosis in colorectal cancer patients. PLoS One.

[182] Kim HJ, Jo MJ, Kim BR, Kim JL, Jeong YA, Na YJ, Park SH, Lee SY, Lee DH, Kim BH (2018). Overexpression of Romo1 is an unfavorable prognostic biomarker and a predictor of lymphatic metastasis in non-small cell lung cancer patients. Onco Targets Ther.

[183] Starheim KK, Gromyko D, Evjenth R, Ryningen A, Varhaug JE, Lillehaug JR, Arnesen T (2009). Knockdown of human N alpha-terminal acetyltransferase complex C leads to p53-dependent apoptosis and aberrant human Arl8b localization. Mol Cell Biol.

[184] Wu F, Chen WJ, Yan L, Tan GQ, Li WT, Zhu XJ, Ge XC, Liu JW, Wang BL (2016). Mus81 knockdown improves chemosensitivity of hepatocellular carcinoma cells by inducing S-phase arrest and promoting apoptosis through CHK1 pathway. Cancer Med.

[185] Liao X, Huang R, Liu X, Han C, Yu L, Wang S, Sun N, Li B, Ning X, Peng T (2017). Distinct prognostic values of alcohol dehydrogenase mRNA expression in pancreatic adenocarcinoma. Onco Targets Ther.

[186] Wang P, Zhang L, Huang C, Huang P, Zhang J (2018). Distinct prognostic values of alcohol dehydrogenase family members for non-small cell lung cancer. Med Sci Monit.

[187] Zhao L, Fan J, Xia S, Pan Y, Liu S, Qian G, Qian Z, Kang HB, Arbiser JL, Pollack BP (2017). HMG-CoA synthase 1 is a synthetic lethal partner of BRAF(V600E) in human cancers. J Biol Chem.

[188] Chen SW, Chou CT, Chang CC, Li YJ, Chen ST, Lin IC, Kok SH, Cheng SJ, Lee JJ, Wu TS (2017). HMGCS2 enhances invasion and metastasis via direct interaction with PPARalpha to activate Src signaling in colorectal cancer and oral cancer. Oncotarget.

[189] Castro-Giner F, Ratcliffe P, Tomlinson I (2015). The mini-driver model of polygenic cancer evolution. Nat Rev Cancer.

[190] Rauhala HE, Teppo S, Niemela S, Kallioniemi A (2013). Silencing of the ARP2/3 complex disturbs pancreatic cancer cell migration. Anticancer Res.

[191] Oji Y, Tatsumi N, Fukuda M, Nakatsuka S, Aoyagi S, Hirata E, Nanchi I, Fujiki F, Nakajima H, Yamamoto Y (2014). The translation elongation factor eEF2 is a novel tumorassociated antigen overexpressed in various types of cancers. Int J Oncol.

[192] Patel H, Abduljabbar R, Lai CF, Periyasamy M, Harrod A, Gemma C, Steel JH, Patel N, Busonero C, Jerjees D (2016). Expression of CDK7, Cyclin H, and MAT1 is elevated in breast cancer and is prognostic in estrogen receptor-positive breast cancer. Clin Cancer Res.

[193] Li B, Ni Chonghaile T, Fan Y, Madden SF, Klinger R, O'Connor AE, Walsh L, O'Hurley G, Mallya Udupi G, Joseph J (2017). Therapeutic rationale to target highly expressed CDK7 conferring poor outcomes in triple-negative breast cancer. Cancer Res.

[194] Wang Q, Li M, Zhang X, Huang H, Huang J, Ke J, Ding H, Xiao J, Shan X, Liu Q (2016). Upregulation of CDK7 in gastric cancer cell promotes tumor cell proliferation and predicts poor prognosis. Exp Mol Pathol.

[195] Wei F, Ding L, Wei Z, Zhang Y, Li Y, Qinghua L, Ma Y, Guo L, Lv G, Liu Y (2016). Ribosomal protein L34 promotes the proliferation, invasion and metastasis of pancreatic cancer cells. Oncotarget.

[196] Yang S, Cui J, Yang Y, Liu Z, Yan H, Tang C, Wang H, Qin H, Li X, Li J (2016). Over-expressed RPL34 promotes malignant proliferation of non-small cell lung cancer cells. Gene.

[197] Dai J, Wei W (2017). Influence of the RPL34 gene on the growth and metastasis of oral squamous cell carcinoma cells. Arch Oral Biol.

[198] Karan D, Kelly DL, Rizzino A, Lin MF, Batra SK (2002). Expression profile of differentially-regulated genes during progression of androgen-independent growth in human prostate cancer cells. Carcinogenesis.

[199] Knoll M, Macher-Goeppinger S, Kopitz J, Duensing S, Pahernik S, Hohenfellner M, Schirmacher P, Roth W (2016). The ribosomal protein S6 in renal cell carcinoma: functional relevance and potential as biomarker. Oncotarget.

[200] Chen B, Tan Z, Gao J, Wu W, Liu L, Jin W, Cao Y, Zhao S, Zhang W, Qiu Z (2015). Hyperphosphorylation of ribosomal protein S6 predicts unfavorable clinical survival in non-small cell lung cancer. J Exp Clin Cancer Res.

[201] Hou L, Li Y, Wang Y, Xu D, Cui H, Xu X, Cong Y, Yu C (2018). UBE2D1 RNA expression was an independent unfavorable prognostic indicator in lung adenocarcinoma, but not in lung squamous cell carcinoma. Dis Markers.

[202] Zabala-Letona A, Arruabarrena-Aristorena A, Martin-Martin N, Fernandez-Ruiz S, Sutherland JD, Clasquin M, Tomas-Cortazar J, Jimenez J, Torres I, Quang P (2017). mTORC1-dependent AMD1 regulation sustains polyamine metabolism in prostate cancer. Nature.

[203] Zhao X, Li J, He Y, Lan F, Fu L, Guo J, Zhao R, Ye Y, He M, Chong W (2001). A novel growth suppressor gene on chromosome 17p13.3 with a high frequency of mutation in human hepatocellular carcinoma. Cancer Res.

[204] Xu HN, Huang WD, Cai Y, Ding M, Gu JF, Wei N, Sun LY, Cao X, Li HG, Zhang KJ (2011). HCCS1-armed, quadruple-regulated oncolytic adenovirus specific for liver cancer as a cancer targeting gene-viro-therapy strategy. Mol Cancer.

[205] Gan Y, Gu J, Cai X, Hu J, Liu XY, Zhao X (2008). Adenovirus-mediated HCCS1 overexpression elicits a potent antitumor efficacy on human colorectal cancer and hepatoma cells both in vitro and in vivo. Cancer Gene Ther.

[206] Kim TE, Kim YW, Hwang SY, Shin SM, Shin JW, Lee YH, Shin SY, Han KT, Lee JM, Namkoong SE, Kim JW (2002). Candidate tumor suppressor, HCCS-1, is downregulated in human cancers and induces apoptosis in cervical cancer. Int J Cancer.

[207] Su YC, Feng YH, Wu HT, Huang YS, Tung CL, Wu P, Chang CJ, Shiau AL, Wu CL (2018). Elovl6 is a negative clinical predictor for liver cancer and knockdown of Elovl6 reduces murine liver cancer progression. Sci Rep.

[208] Tzatsos A, Paskaleva P, Ferrari F, Deshpande V, Stoykova S, Contino G, Wong KK, Lan F, Trojer P, Park PJ, Bardeesy N (2013). KDM2B promotes pancreatic cancer via Polycomb-dependent and -independent transcriptional programs. J Clin Invest.

[209] Wang Y, Zang J, Zhang D, Sun Z, Qiu B, Wang X (2018). KDM2B overexpression correlates with poor prognosis and regulates glioma cell growth. Onco Targets Ther.

[210] Zheng Q, Fan H, Meng Z, Yuan L, Liu C, Peng Y, Zhao W, Wang L, Li J, Feng J (2018). Histone demethylase KDM2B promotes triple negative breast cancer proliferation by suppressing p15INK4B, p16INK4A, and p57KIP2 transcription. Acta Biochim Biophys Sin (Shanghai).

[211] Zhu XX, Yan YW, Ai CZ, Jiang S, Xu SS, Niu M, Wang XZ, Zhong GS, Lu XF, Xue Y (2017). Jarid2 is essential for the maintenance of tumor initiating cells in bladder cancer. Oncotarget.

[212] Schleich S, Strassburger K, Janiesch PC, Koledachkina T, Miller KK, Haneke K, Cheng YS, Kuechler K, Stoecklin G, Duncan KE, Teleman AA (2014). DENR-MCT-1 promotes translation re-initiation downstream of uORFs to control tissue growth. Nature.

[213] Wang YW, Lin KT, Chen SC, Gu DL, Chen CF, Tu PH, Jou YS (2013). Overexpressed-eIF3I interacted and activated oncogenic Akt1 is a theranostic target in human hepatocellular carcinoma. Hepatology.

[214] Qi J, Dong Z, Liu J, Zhang JT (2014). EIF3i promotes colon oncogenesis by regulating COX-2 protein synthesis and beta-catenin activation. Oncogene.

[215] Wang C, Jin G, Jin H, Wang N, Luo Q, Zhang Y, Gao D, Jiang K, Gu D, Shen Q (2015). Clusterin facilitates metastasis by EIF3I/Akt/MMP13 signaling in hepatocellular carcinoma. Oncotarget.

[216] Torrance V, Lydall D (2018). Overlapping open reading frames strongly reduce human and yeast STN1 gene expression and affect telomere function. PLoS Genet.

[217] Widschwendter M, Apostolidou S, Raum E, Rothenbacher D, Fiegl H, Menon U, Stegmaier C, Jacobs IJ, Brenner H (2008). Epigenotyping in peripheral blood cell DNA and breast cancer risk: a proof of principle study. PLoS One.

[218] Khakpour G, Pooladi A, Izadi P, Noruzinia M, Tavakkoly Bazzaz J (2015). DNA methylation as a promising landscape: a simple blood test for breast cancer prediction. Tumour Biol.

[219] Savci-Heijink CD, Halfwerk H, Koster J, van de Vijver MJ (2016). A novel gene expression signature for bone metastasis in breast carcinomas. Breast Cancer Res Treat.

[220] Horlbeck MA, Xu A, Wang M, Bennett NK, Park CY, Bogdanoff D, Adamson B, Chow ED, Kampmann M, Peterson TR (2018). Mapping the genetic landscape of human cells. Cell.

[221] Costanzo M, Kuzmin E, van Leeuwen J, Mair B, Moffat J, Boone C, Andrews B (2019). Global genetic networks and the genotype-to-phenotype relationship. Cell.

[222] Chen G, Wang J (2014). Threonine metabolism and embryonic stem cell self-renewal. Curr Opin Clin Nutr Metab Care.

[223] Shyh-Chang N, Locasale JW, Lyssiotis CA, Zheng Y, Teo RY, Ratanasirintrawoot S, Zhang J, Onder T, Unternaehrer JJ, Zhu H (2013). Influence of threonine metabolism on S-adenosylmethionine and histone methylation. Science.

[224] Wang J, Alexander P, Wu L, Hammer R, Cleaver O, McKnight SL (2009). Dependence of mouse embryonic stem cells on threonine catabolism. Science.

[225] Shurtleff MJ, Itzhak DN, Hussmann JA, Schirle Oakdale NT, Costa EA, Jonikas M, Weibezahn J, Popova KD, Jan CH, Sinitcyn P, et al. The ER membrane protein complex interacts cotranslationally to enable biogenesis of multipass membrane proteins. Elife. 2018;7. 10.7554/eLife.37018 PMC5995541 29809151

[226] Kornberg RD (2007). The molecular basis of eucaryotic transcription. Cell Death Differ.

[227] Hartwell LH (2002). Nobel lecture. Yeast and cancer. Biosci Rep.

[228] Blackburn EH (2010). Telomeres and telomerase: the means to the end (Nobel lecture). Angew Chem Int Ed Engl.

[229] Schekman R, Sudhof T (2014). An interview with Randy Schekman and Thomas Sudhof. Trends Cell Biol.

[230] Mizushima N (2017). The exponential growth of autophagy-related research: from the humble yeast to the Nobel Prize. FEBS Lett.

[231] Bhattacharya B, Mohd Omar MF, Soong R (2016). The Warburg effect and drug resistance. Br J Pharmacol.

[232] Yang F, Kemp CJ, Henikoff S (2015). Anthracyclines induce double-strand DNA breaks at active gene promoters. Mutat Res.

[233] Flavahan WA, Gaskell E, Bernstein BE. Epigenetic plasticity and the hallmarks of cancer. Science. 2017;357. 10.1126/science.aal2380 PMC5940341 28729483

[234] de Cubas AA, Rathmell WK (2018). Epigenetic modifiers: activities in renal cell carcinoma. Nat Rev Urol.

[235] Ververis K, Rodd AL, Tang MM, El-Osta A, Karagiannis TC (2011). Histone deacetylase inhibitors augment doxorubicin-induced DNA damage in cardiomyocytes. Cell Mol Life Sci.

[236] Tu Y, Hershman DL, Bhalla K, Fiskus W, Pellegrino CM, Andreopoulou E, Makower D, Kalinsky K, Fehn K, Fineberg S (2014). A phase I-II study of the histone deacetylase inhibitor vorinostat plus sequential weekly paclitaxel and doxorubicin-cyclophosphamide in locally advanced breast cancer. Breast Cancer Res Treat.

[237] Namdar M, Perez G, Ngo L, Marks PA (2010). Selective inhibition of histone deacetylase 6 (HDAC6) induces DNA damage and sensitizes transformed cells to anticancer agents. Proc Natl Acad Sci U S A.

[238] Song R, Yang Y, Lei H, Wang G, Huang Y, Xue W, Wang Y, Yao L, Zhu Y (2018). HDAC6 inhibition protects cardiomyocytes against doxorubicin-induced acute damage by improving alpha-tubulin acetylation. J Mol Cell Cardiol.

[239] Li T, Zhang C, Hassan S, Liu X, Song F, Chen K, Zhang W, Yang J (2018). Histone deacetylase 6 in cancer. J Hematol Oncol.

[240] Yang C, Choy E, Hornicek FJ, Wood KB, Schwab JH, Liu X, Mankin H, Duan Z. Histone deacetylase inhibitor (HDACI) PCI-24781 potentiates cytotoxic effects of doxorubicin in bone sarcoma cells. Cancer Chemother Pharmacol. 2011;67:439–46. 10.1007/s00280-010-1344-7 20461381

[241] Grandori C, Kemp CJ (2018). Personalized cancer models for target discovery and precision medicine. Trends Cancer.

[242] Puca L, Bareja R, Prandi D, Shaw R, Benelli M, Karthaus WR, Hess J, Sigouros M, Donoghue A, Kossai M (2018). Patient derived organoids to model rare prostate cancer phenotypes. Nat Commun.

[243] Tecza K, Pamula-Pilat J, Lanuszewska J, Butkiewicz D, Grzybowska E (2018). Pharmacogenetics of toxicity of 5-fluorouracil, doxorubicin and cyclophosphamide chemotherapy in breast cancer patients. Oncotarget.

[244] Kim MM, Parolia A, Dunphy MP, Venneti S (2016). Non-invasive metabolic imaging of brain tumours in the era of precision medicine. Nat Rev Clin Oncol.

[245] Costanzo M, VanderSluis B, Koch EN, Baryshnikova A, Pons C, Tan G, Wang W, Usaj M, Hanchard J, Lee SD, et al. A global genetic interaction network maps a wiring diagram of cellular function. Science. 2016;353. 10.1126/science.aaf1420 PMC5661885 27708008

[246] Ihmels J, Collins SR, Schuldiner M, Krogan NJ, Weissman JS (2007). Backup without redundancy: genetic interactions reveal the cost of duplicate gene loss. Mol Syst Biol.

[247] Phillips PC (2008). Epistasis--the essential role of gene interactions in the structure and evolution of genetic systems. Nat Rev Genet.

[248] Breslow DK, Cameron DM, Collins SR, Schuldiner M, Stewart-Ornstein J, Newman HW, Braun S, Madhani HD, Krogan NJ, Weissman JS (2008). A comprehensive strategy enabling high-resolution functional analysis of the yeast genome. Nat Methods.

[249] Kornmann B, Osman C, Walter P (2011). The conserved GTPase Gem1 regulates endoplasmic reticulum-mitochondria connections. Proc Natl Acad Sci U S A.

[250] Conibear E, Stevens TH (2000). Vps52p, Vps53p, and Vps54p form a novel multisubunit complex required for protein sorting at the yeast late Golgi. Mol Biol Cell.

[256] Boyle EI, Weng S, Gollub J, Jin H, Botstein D, Cherry JM, Sherlock G. GO::TermFinder—open source software for accessing Gene Ontology information and finding significantly enriched Gene Ontology terms associated with a list of genes. Bioinformatics. 2004;20:3710–5. 10.1093/bioinformatics/bth456 PMC3037731 15297299

