# Supplementary material for: A yeast phenomic model for the influence of Warburg metabolism on genetic buffering of doxorubicin
Source: Cancer Metab. 2019 Oct 23;7:9. doi: 10.1186/s40170-019-0201-3 (PMC6806529; doi:10.1186/s40170-019-0201-3)
Supplement: Supplementary file 1 — Additional file 1: Figure S1. Doxorubicin dose responses of the YKO/KD parental strains, BY4741a, BY4742alpha, and BY4743a/alpha diploid. Figure S2. Correlation between interaction scores based on L vs. other CPPs (K, r, and AUC), for both HLD and HLEG media. Figure S3. A summary of the first and second rounds of REMc. First round clusters are at the left end of each row of heatmap thumbnails; second round clusters derived from each first round cluster are ordered to the right by relative strength. Rows are grouped into panels by similarity in their gene interaction profiles. The columns in each heatmap have the same order from left to right (see inset panel), with K to the left and L to the right. Within the K and L groups, HLD is to the left and HLEG to the right. Within each of the CPP-media groupings, ‘shift’ (-) is left of the doxorubicin-gene interaction (+). (A) Respiration-specific enhancement. (B) Warburg-independent enhancement. (C) Glycolysis-specific enhancement. (D) HLD and HLEG suppression modules. (E) Respiratory deficiency. Figure S4. Doxorubicin-gene interaction profiles for selected mitochondrial GO terms. Figure S5. Deletion of mitochondrial genes tends to influence doxorubicin-gene interaction in a respiratory (HLEG media) more so than a glycolytic (HLD media) context. Figure S6. Heatmaps for GO terms comprised of overlapping gene sets. Figure S7. Pleiotropic phenotypic influences from genetic perturbation of ribonucleoprotein complex subunit organization. Figure S8. HLD-specific deletion enhancement of doxorubicin toxicity by evolutionarily conserved genes. See also Additional file 10: Table S13. Figure S9. GO term-specific heatmaps for mRNA 3’ end processing and mRNA cleavage gene interaction profiles. Figure S10. Suppression of doxorubicin cytotoxicity by perturbation of sphingolipid and ceramide metabolism. Figure S11. Deletion suppressing doxorubicin-gene interaction for nuclear pore and actin cortical patch functions is relatively Warburg-i [file 40170_2019_201_MOESM1_ESM.pptx]

## Slide 1
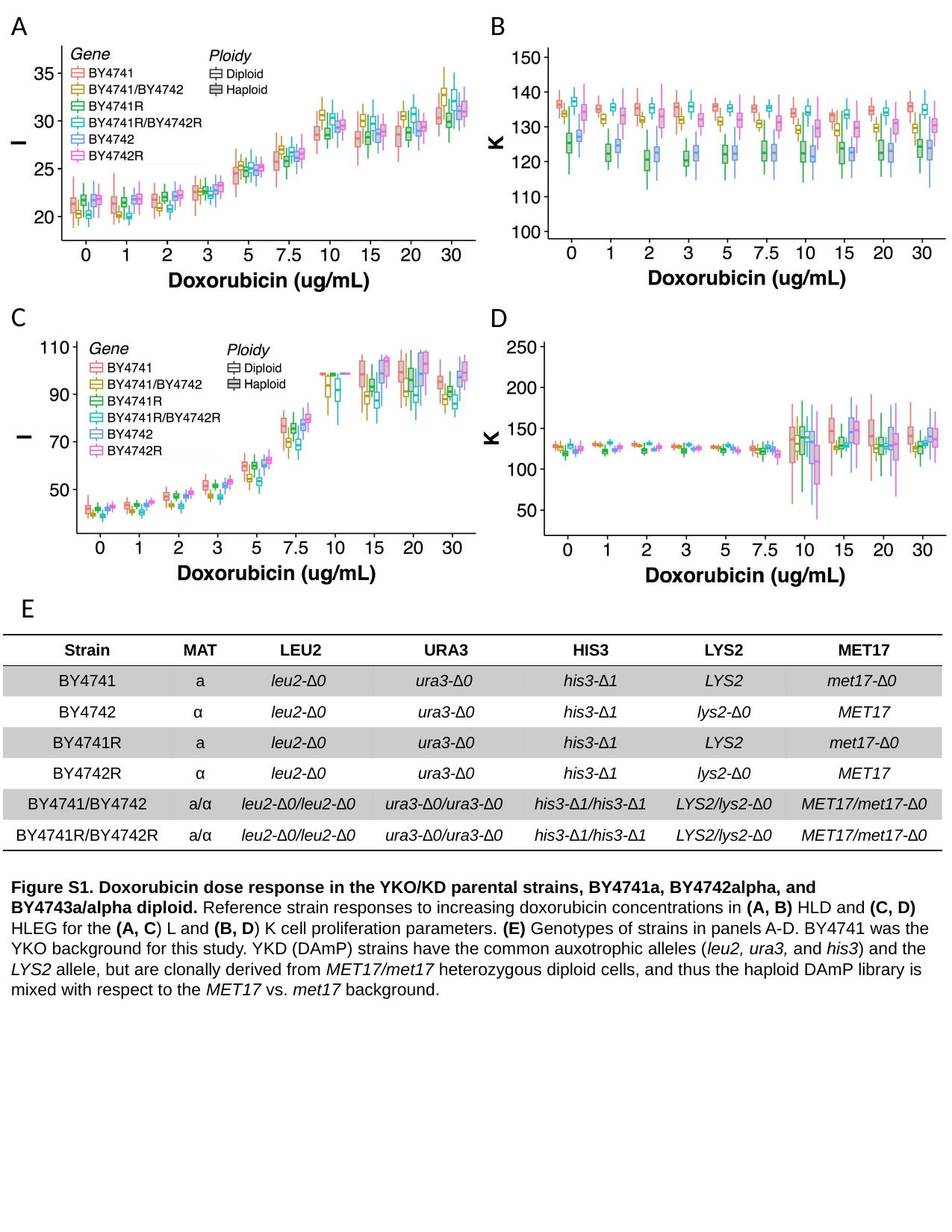

A
B
C
D
E
| Strain | MAT | LEU2 | URA3 | HIS3 | LYS2 | MET17 |
| --- | --- | --- | --- | --- | --- | --- |
| BY4741 | a | leu2-∆0 | ura3-∆0 | his3-∆1 | LYS2 | met17-∆0 |
| BY4742 | α | leu2-∆0 | ura3-∆0 | his3-∆1 | lys2-∆0 | MET17 |
| BY4741R | a | leu2-∆0 | ura3-∆0 | his3-∆1 | LYS2 | met17-∆0 |
| BY4742R | α | leu2-∆0 | ura3-∆0 | his3-∆1 | lys2-∆0 | MET17 |
| BY4741/BY4742 | a/α | leu2-∆0/leu2-∆0 | ura3-∆0/ura3-∆0 | his3-∆1/his3-∆1 | LYS2/lys2-∆0 | MET17/met17-∆0 |
| BY4741R/BY4742R | a/α | leu2-∆0/leu2-∆0 | ura3-∆0/ura3-∆0 | his3-∆1/his3-∆1 | LYS2/lys2-∆0 | MET17/met17-∆0 |
Figure S1. Doxorubicin dose response in the YKO/KD parental strains, BY4741a, BY4742alpha, and BY4743a/alpha diploid. Reference strain responses to increasing doxorubicin concentrations in (A, B) HLD and (C, D) HLEG for the (A, C) L and (B, D) K cell proliferation parameters. (E) Genotypes of strains in panels A-D. BY4741 was the YKO background for this study. YKD (DAmP) strains have the common auxotrophic alleles (leu2, ura3, and his3) and the LYS2 allele, but are clonally derived from MET17/met17 heterozygous diploid cells, and thus the haploid DAmP library is mixed with respect to the MET17 vs. met17 background.

## Slide 2
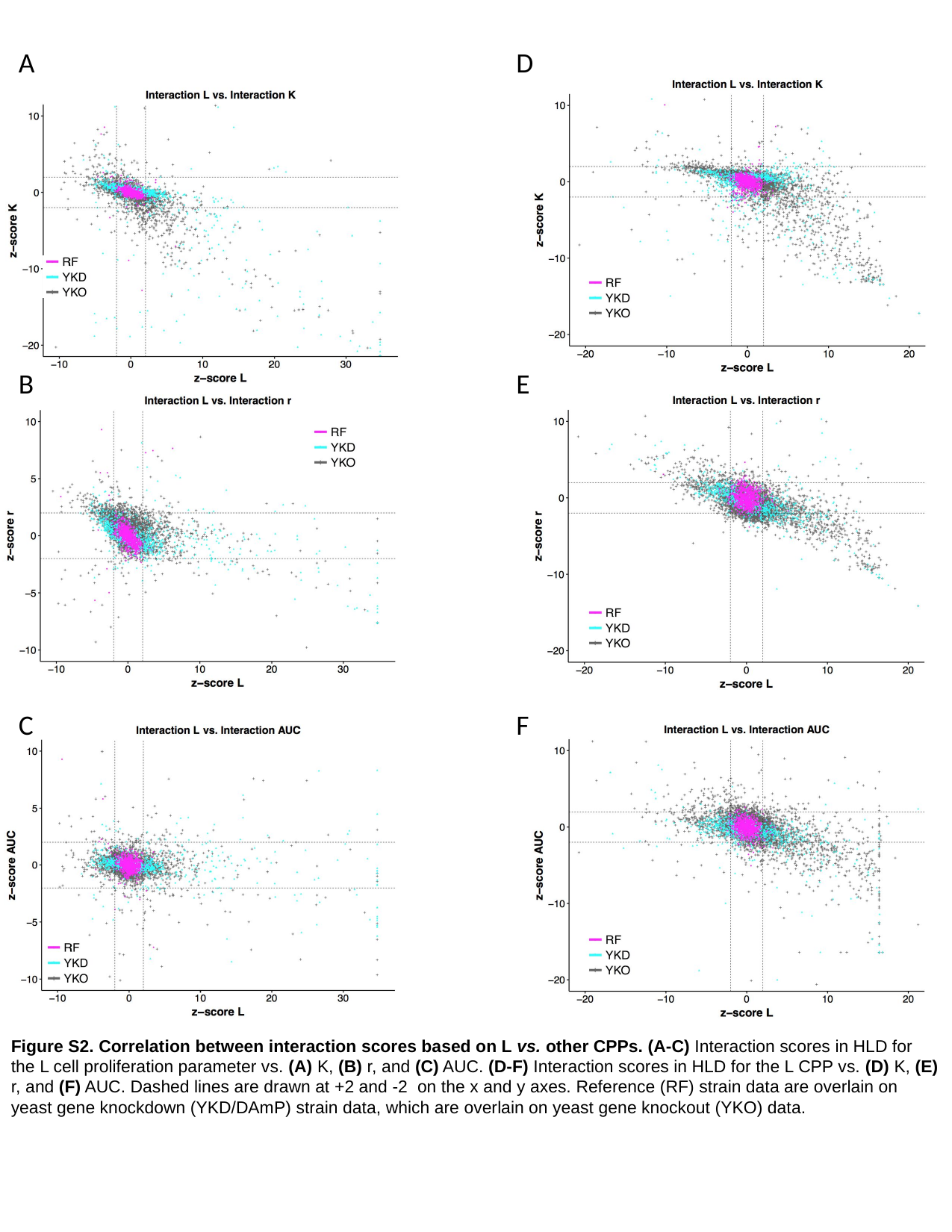

A
D
B
E
C
F
Figure S2. Correlation between interaction scores based on L vs. other CPPs. (A-C) Interaction scores in HLD for the L cell proliferation parameter vs. (A) K, (B) r, and (C) AUC. (D-F) Interaction scores in HLD for the L CPP vs. (D) K, (E) r, and (F) AUC. Dashed lines are drawn at +2 and -2 on the x and y axes. Reference (RF) strain data are overlain on yeast gene knockdown (YKD/DAmP) strain data, which are overlain on yeast gene knockout (YKO) data.

## Slide 3
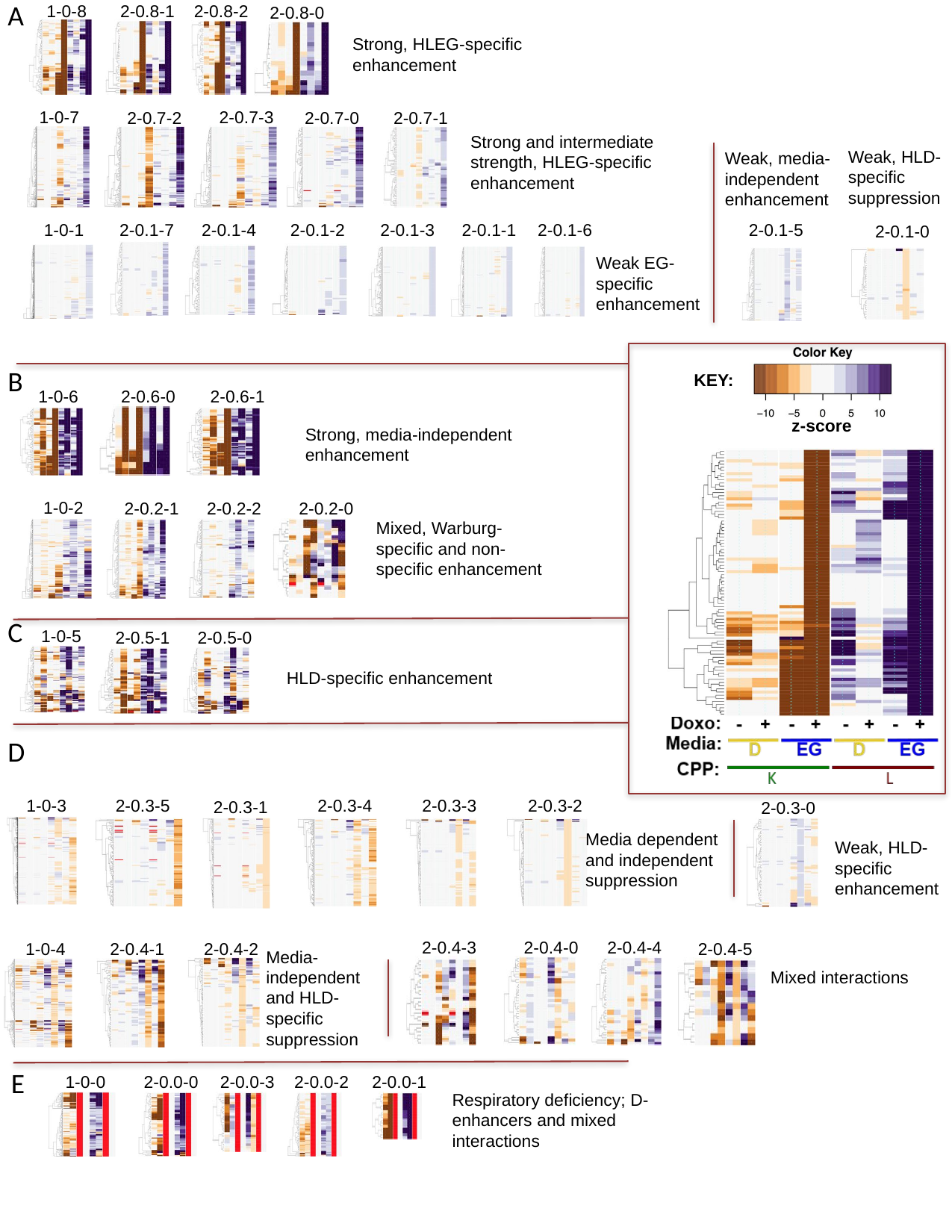

A
1-0-8
2-0.8-1
2-0.8-2
2-0.8-0
Strong, HLEG-specific enhancement
1-0-7
2-0.7-3
2-0.7-2
2-0.7-0
2-0.7-1
Strong and intermediate strength, HLEG-specific enhancement
Weak, HLD-specific suppression
Weak, media-independent enhancement
2-0.1-7
2-0.1-4
2-0.1-3
2-0.1-1
2-0.1-6
1-0-1
2-0.1-2
2-0.1-5
2-0.1-0
Weak EG-specific enhancement
KEY:
z-score
B
1-0-6
2-0.6-0
2-0.6-1
Strong, media-independent enhancement
1-0-2
2-0.2-1
2-0.2-2
2-0.2-0
Mixed, Warburg-specific and non-specific enhancement
C
1-0-5
2-0.5-1
2-0.5-0
HLD-specific enhancement
D
2-0.3-2
2-0.3-5
2-0.3-3
2-0.3-4
1-0-3
2-0.3-1
2-0.3-0
Weak, HLD-specific enhancement
Media dependent and independent suppression
2-0.4-3
2-0.4-0
2-0.4-4
2-0.4-2
2-0.4-5
2-0.4-1
1-0-4
Media-independent and HLD-specific suppression
Mixed interactions
E
1-0-0
2-0.0-3
2-0.0-1
2-0.0-0
2-0.0-2
Respiratory deficiency; D-enhancers and mixed interactions

## Slide 4
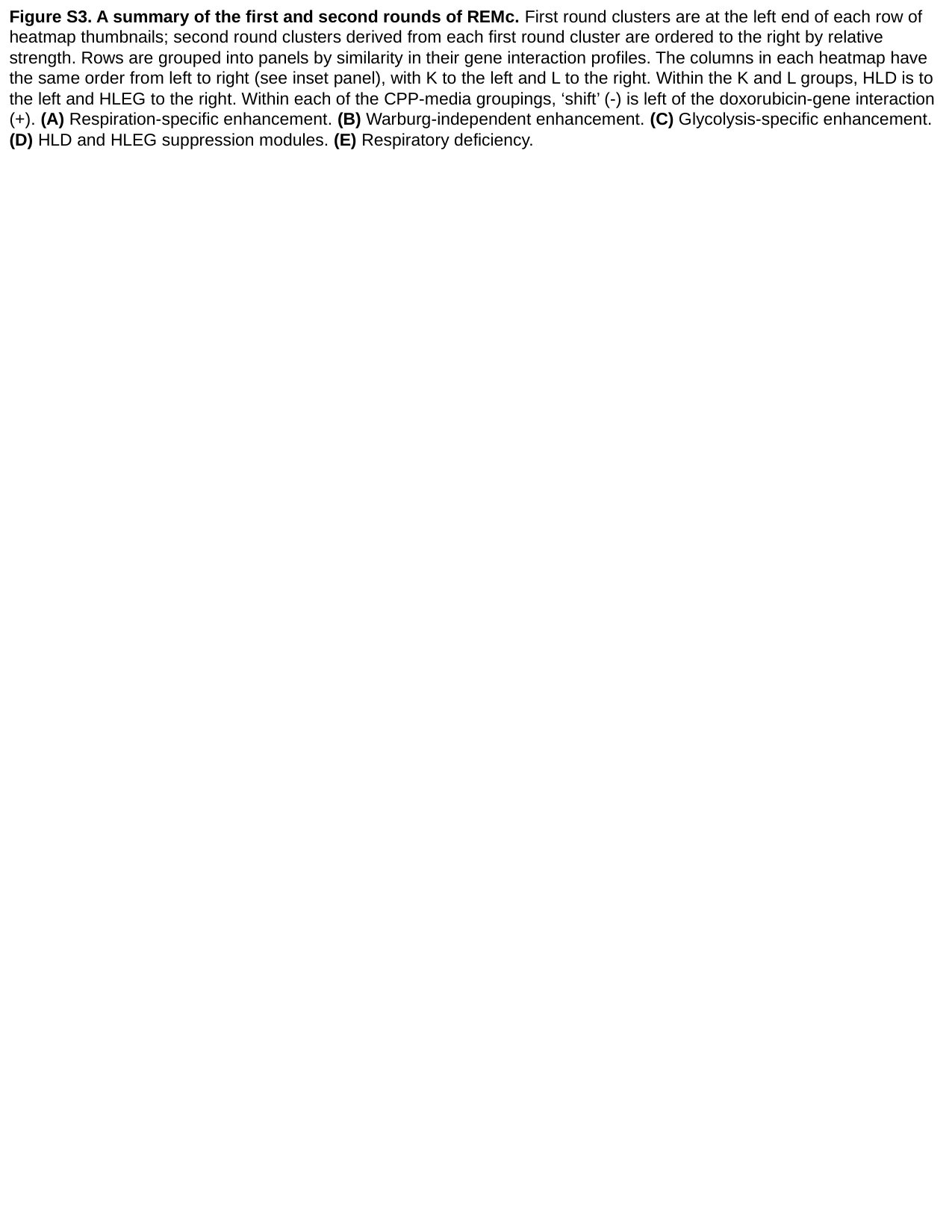

Figure S3. A summary of the first and second rounds of REMc. First round clusters are at the left end of each row of heatmap thumbnails; second round clusters derived from each first round cluster are ordered to the right by relative strength. Rows are grouped into panels by similarity in their gene interaction profiles. The columns in each heatmap have the same order from left to right (see inset panel), with K to the left and L to the right. Within the K and L groups, HLD is to the left and HLEG to the right. Within each of the CPP-media groupings, ‘shift’ (-) is left of the doxorubicin-gene interaction (+). (A) Respiration-specific enhancement. (B) Warburg-independent enhancement. (C) Glycolysis-specific enhancement. (D) HLD and HLEG suppression modules. (E) Respiratory deficiency.

## Slide 5
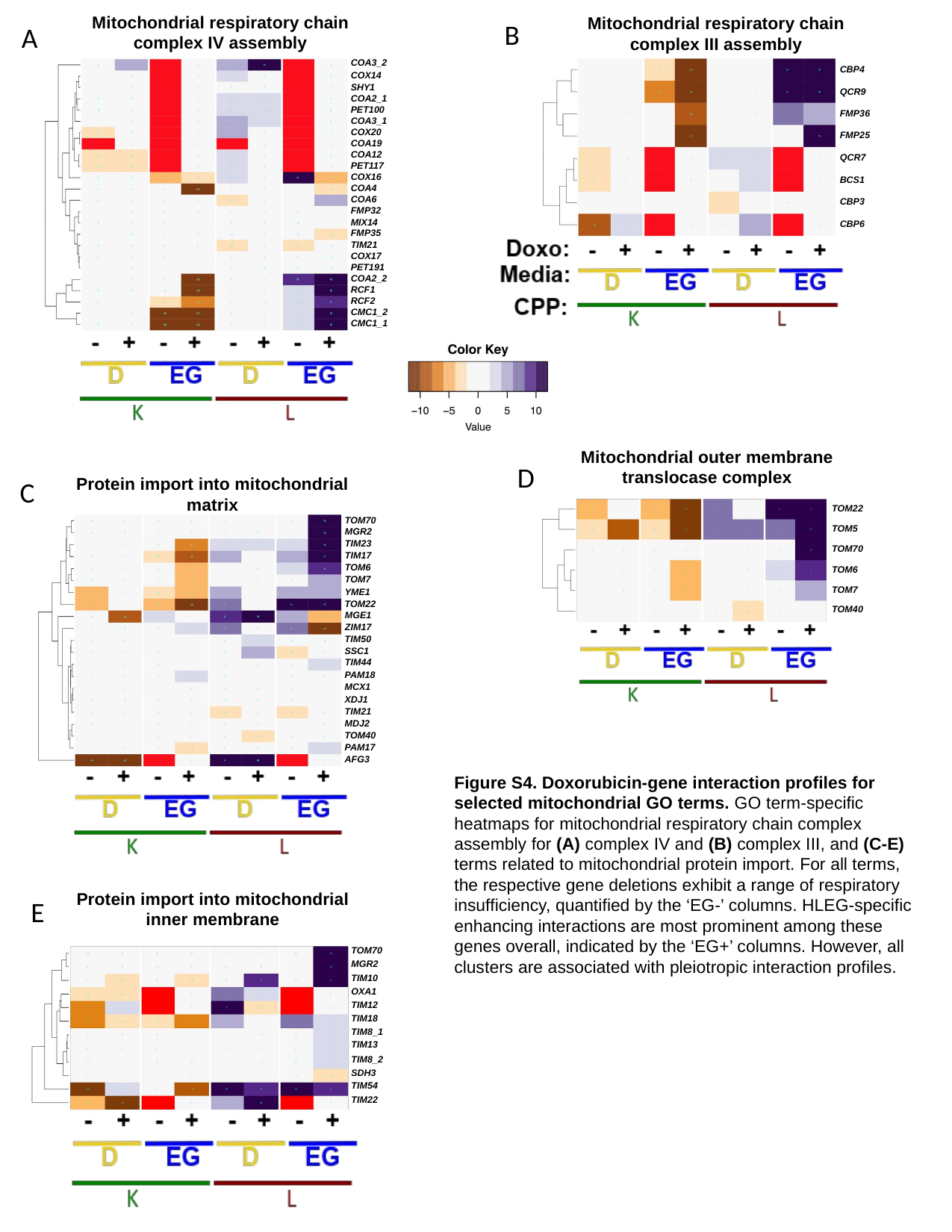

Mitochondrial respiratory chain complex IV assembly
A
COA3_2
COX14
SHY1
COA2_1
PET100
COA3_1
COX20
COA19
COA12
PET117
COX16
COA4
COA6
FMP32
MIX14
FMP35
TIM21
COX17
PET191
COA2_2
RCF1
RCF2
CMC1_2
CMC1_1
Mitochondrial respiratory chain complex III assembly
B
CBP4
QCR9
FMP36
FMP25
QCR7
BCS1
CBP3
CBP6
Mitochondrial outer membrane translocase complex
D
Protein import into mitochondrial matrix
C
TOM22
TOM70
TOM5
MGR2
TIM23
TOM70
TIM17
TOM6
TOM6
TOM7
TOM7
YME1
TOM22
TOM40
MGE1
ZIM17
TIM50
SSC1
TIM44
PAM18
MCX1
XDJ1
TIM21
MDJ2
TOM40
PAM17
AFG3
Figure S4. Doxorubicin-gene interaction profiles for selected mitochondrial GO terms. GO term-specific heatmaps for mitochondrial respiratory chain complex assembly for (A) complex IV and (B) complex III, and (C-E) terms related to mitochondrial protein import. For all terms, the respective gene deletions exhibit a range of respiratory insufficiency, quantified by the ‘EG-’ columns. HLEG-specific enhancing interactions are most prominent among these genes overall, indicated by the ‘EG+’ columns. However, all clusters are associated with pleiotropic interaction profiles.
Protein import into mitochondrial inner membrane
E
TOM70
MGR2
TIM10
OXA1
TIM12
TIM18
TIM8_1
TIM13
TIM8_2
SDH3
TIM54
TIM22

## Slide 6
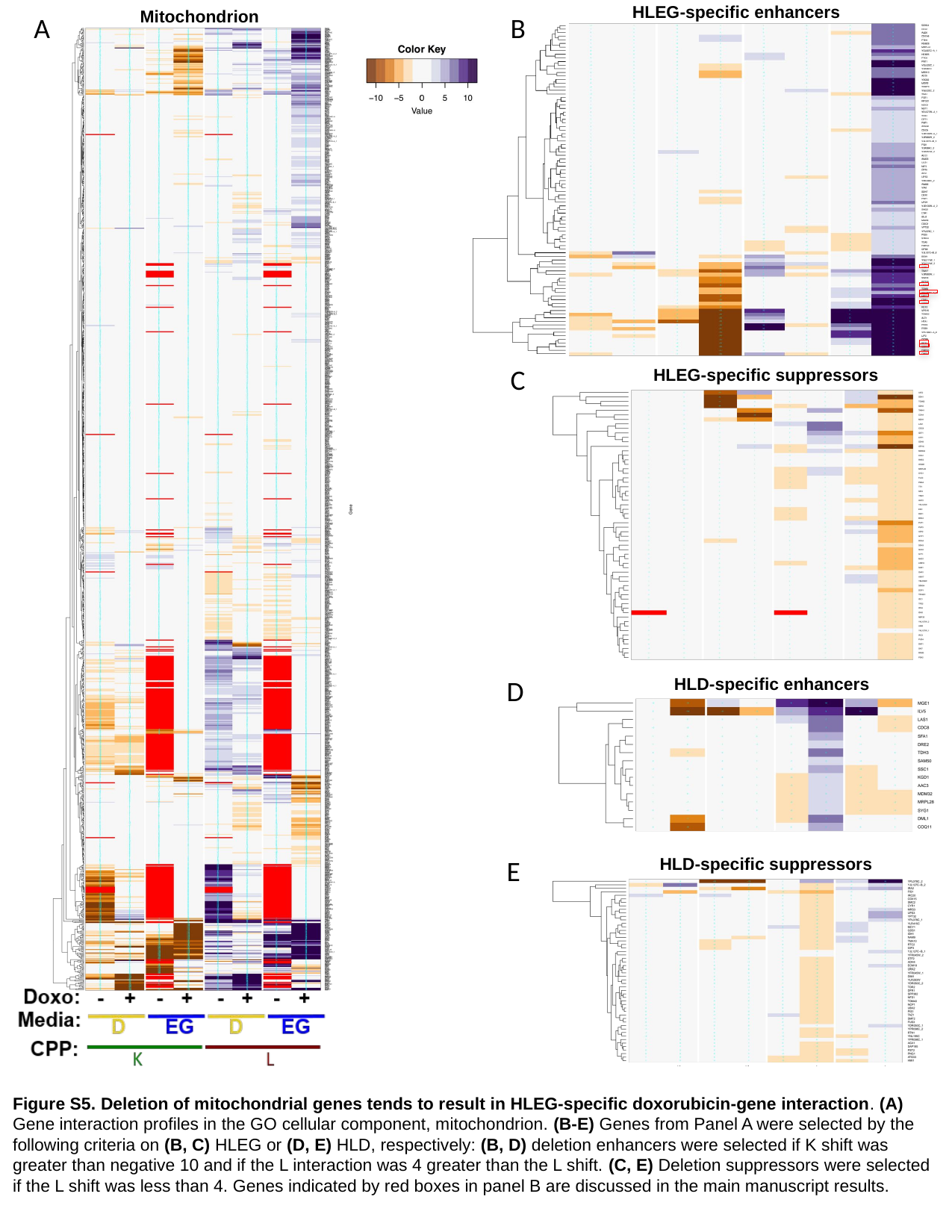

HLEG-specific enhancers
Mitochondrion
B
A
C
HLEG-specific suppressors
D
HLD-specific enhancers
E
HLD-specific suppressors
Figure S5. Deletion of mitochondrial genes tends to result in HLEG-specific doxorubicin-gene interaction. (A) Gene interaction profiles in the GO cellular component, mitochondrion. (B-E) Genes from Panel A were selected by the following criteria on (B, C) HLEG or (D, E) HLD, respectively: (B, D) deletion enhancers were selected if K shift was greater than negative 10 and if the L interaction was 4 greater than the L shift. (C, E) Deletion suppressors were selected if the L shift was less than 4. Genes indicated by red boxes in panel B are discussed in the main manuscript results.

## Slide 7
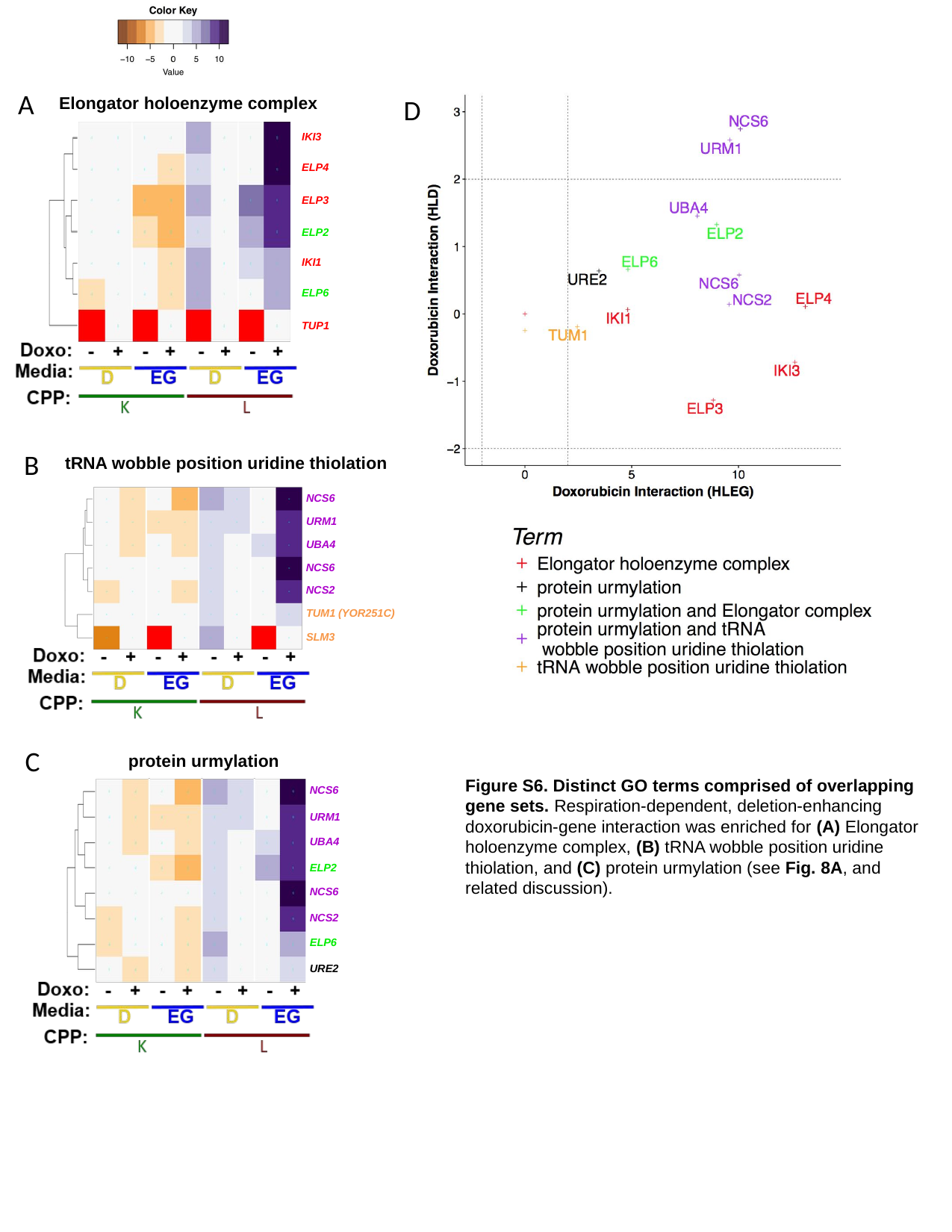

A
Elongator holoenzyme complex
D
IKI3
ELP4
ELP3
ELP2
IKI1
ELP6
TUP1
B
tRNA wobble position uridine thiolation
NCS6
URM1
UBA4
NCS6
NCS2
TUM1 (YOR251C)
SLM3
C
protein urmylation
Figure S6. Distinct GO terms comprised of overlapping gene sets. Respiration-dependent, deletion-enhancing doxorubicin-gene interaction was enriched for (A) Elongator holoenzyme complex, (B) tRNA wobble position uridine thiolation, and (C) protein urmylation (see Fig. 8A, and related discussion).
NCS6
URM1
UBA4
ELP2
NCS6
NCS2
ELP6
URE2

## Slide 8
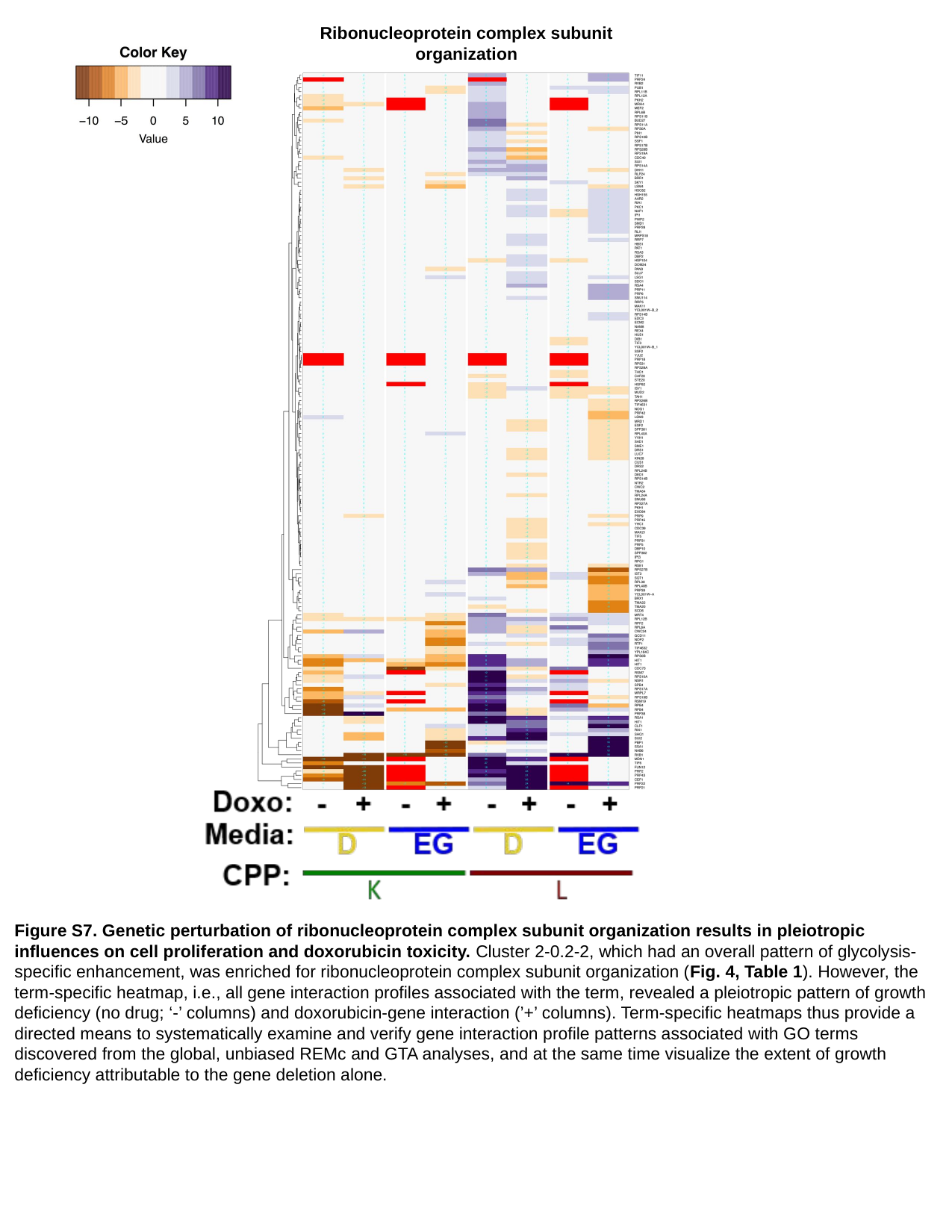

Ribonucleoprotein complex subunit organization
Figure S7. Genetic perturbation of ribonucleoprotein complex subunit organization results in pleiotropic influences on cell proliferation and doxorubicin toxicity. Cluster 2-0.2-2, which had an overall pattern of glycolysis-specific enhancement, was enriched for ribonucleoprotein complex subunit organization (Fig. 4, Table 1). However, the term-specific heatmap, i.e., all gene interaction profiles associated with the term, revealed a pleiotropic pattern of growth deficiency (no drug; ‘-’ columns) and doxorubicin-gene interaction (’+’ columns). Term-specific heatmaps thus provide a directed means to systematically examine and verify gene interaction profile patterns associated with GO terms discovered from the global, unbiased REMc and GTA analyses, and at the same time visualize the extent of growth deficiency attributable to the gene deletion alone.

## Slide 9
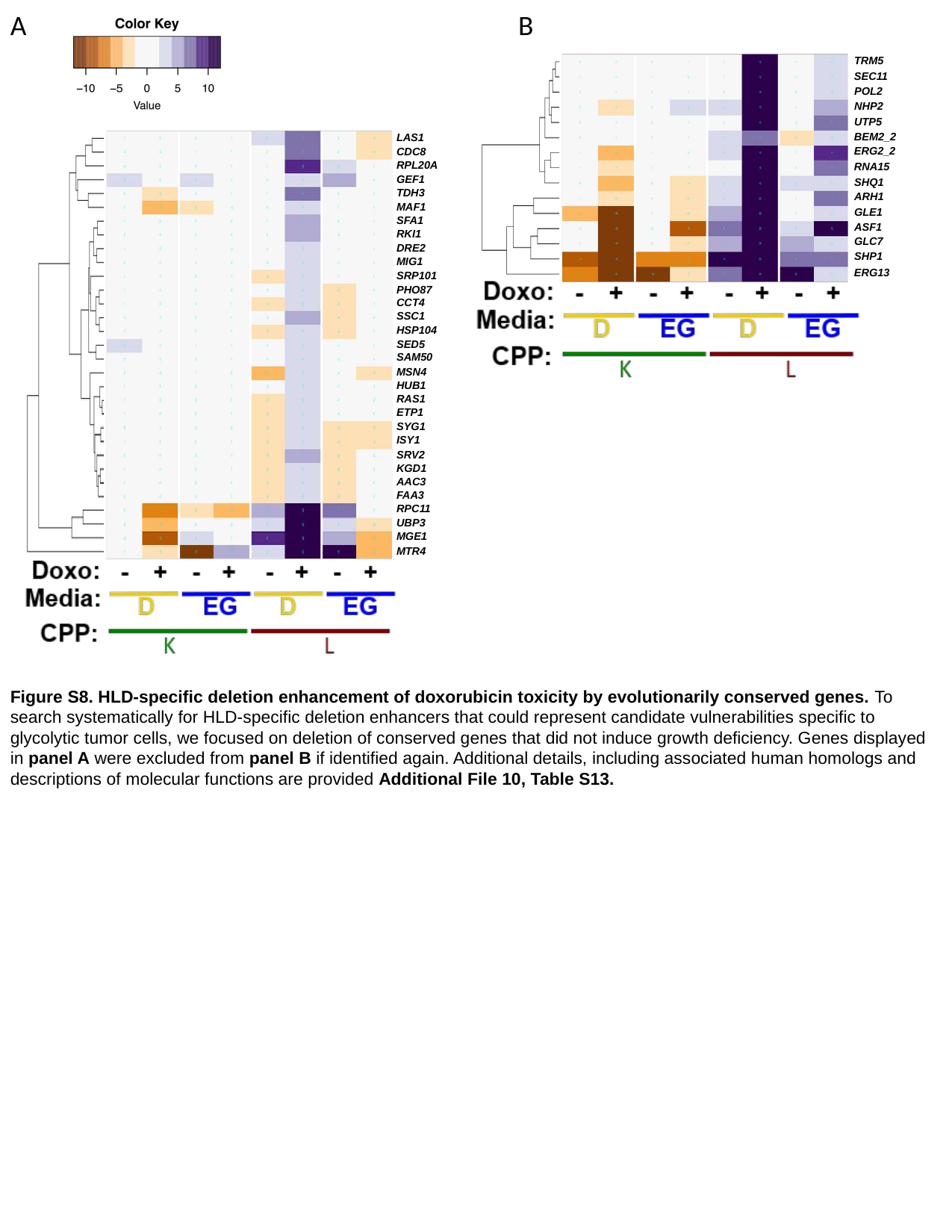

A
B
TRM5
SEC11
POL2
NHP2
UTP5
BEM2_2
LAS1
ERG2_2
CDC8
RPL20A
RNA15
GEF1
SHQ1
TDH3
ARH1
MAF1
GLE1
SFA1
ASF1
RKI1
GLC7
DRE2
SHP1
MIG1
ERG13
SRP101
PHO87
CCT4
SSC1
HSP104
SED5
SAM50
MSN4
HUB1
RAS1
ETP1
SYG1
ISY1
SRV2
KGD1
AAC3
FAA3
RPC11
UBP3
MGE1
MTR4
Figure S8. HLD-specific deletion enhancement of doxorubicin toxicity by evolutionarily conserved genes. To search systematically for HLD-specific deletion enhancers that could represent candidate vulnerabilities specific to glycolytic tumor cells, we focused on deletion of conserved genes that did not induce growth deficiency. Genes displayed in panel A were excluded from panel B if identified again. Additional details, including associated human homologs and descriptions of molecular functions are provided Additional File 10, Table S13.

## Slide 10
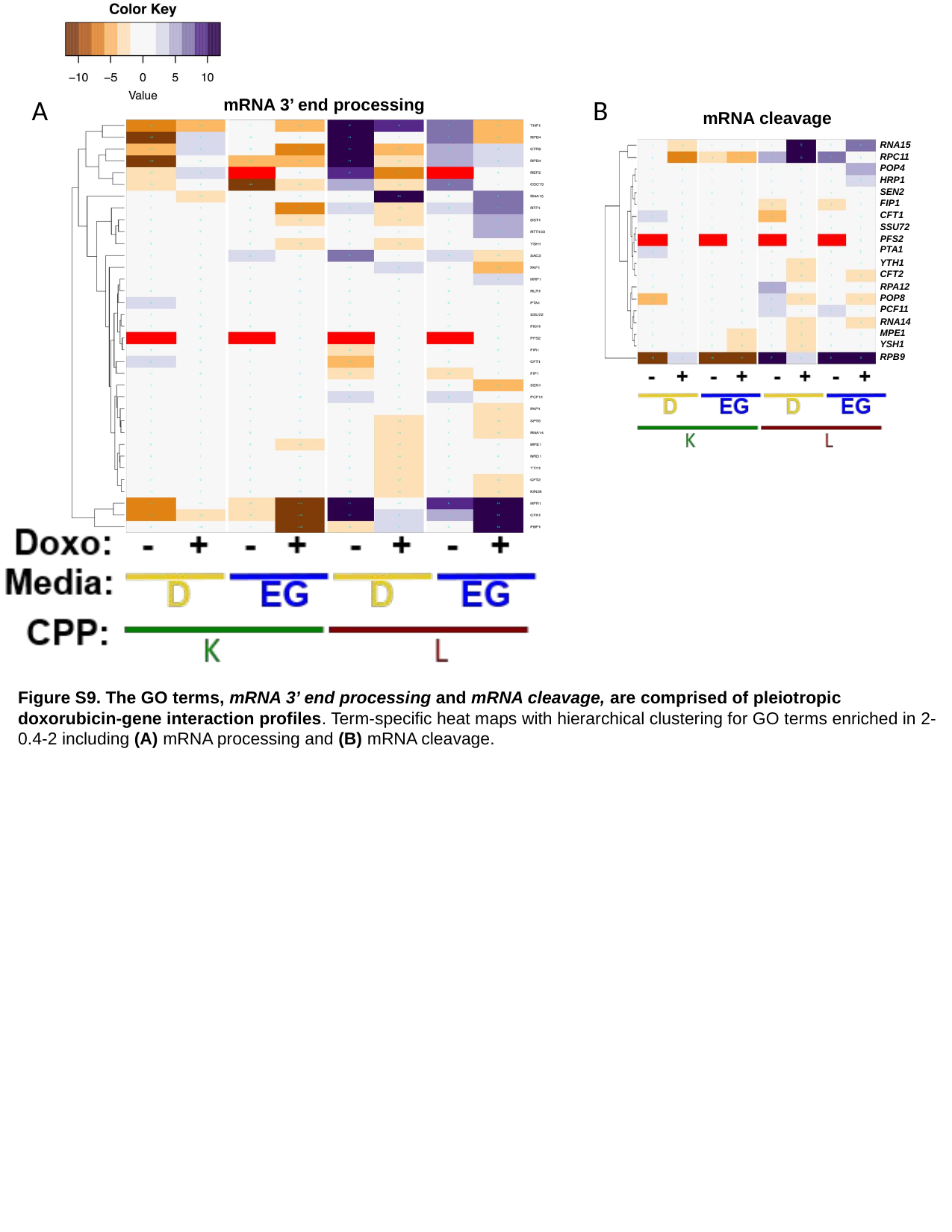

A
B
mRNA 3’ end processing
mRNA cleavage
RNA15
RPC11
POP4
HRP1
SEN2
FIP1
CFT1
SSU72
PFS2
PTA1
YTH1
CFT2
RPA12
POP8
PCF11
RNA14
MPE1
YSH1
RPB9
Figure S9. The GO terms, mRNA 3’ end processing and mRNA cleavage, are comprised of pleiotropic doxorubicin-gene interaction profiles. Term-specific heat maps with hierarchical clustering for GO terms enriched in 2-0.4-2 including (A) mRNA processing and (B) mRNA cleavage.

## Slide 11
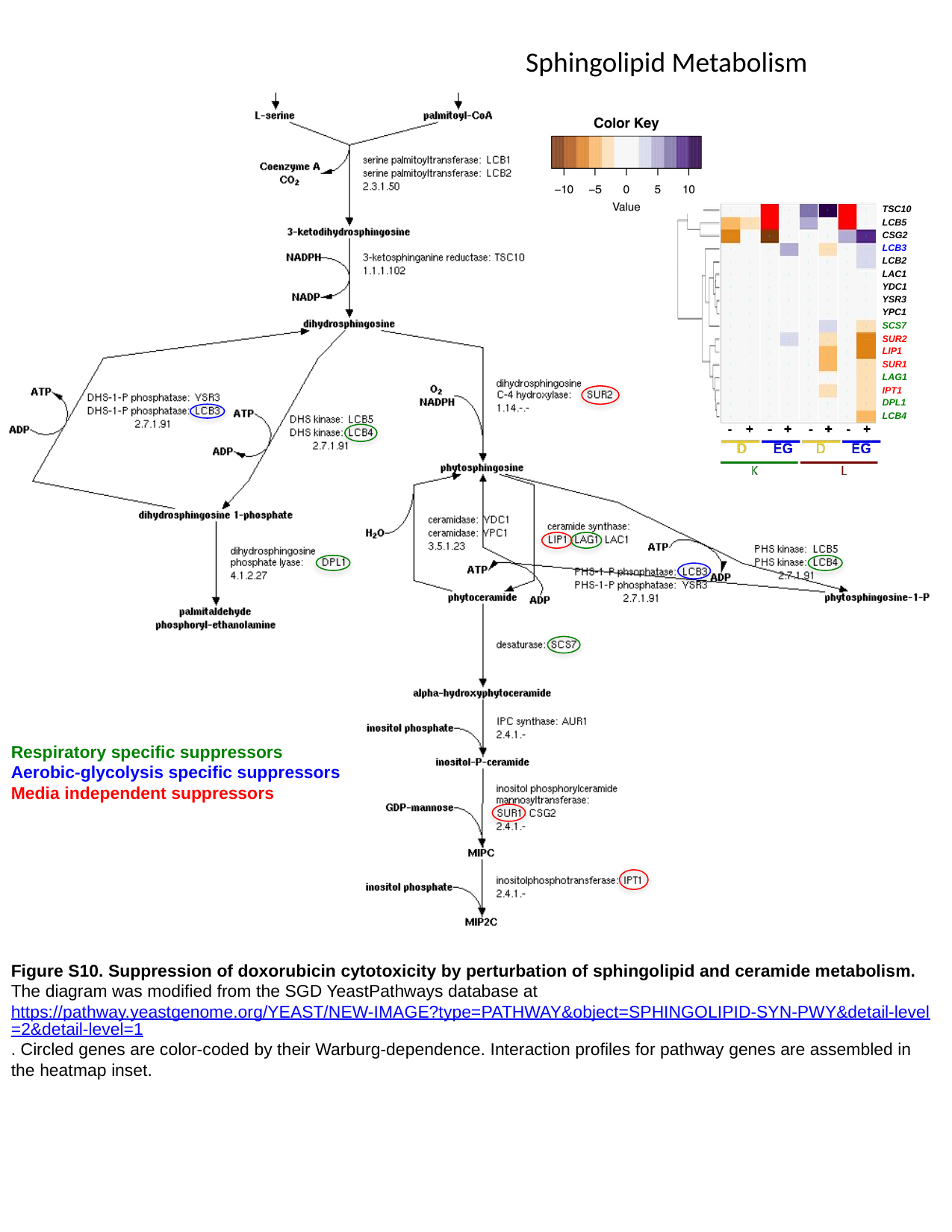

Sphingolipid Metabolism
TSC10
LCB5
CSG2
LCB3
LCB2
LAC1
YDC1
YSR3
YPC1
SCS7
SUR2
LIP1
SUR1
LAG1
IPT1
DPL1
LCB4
Respiratory specific suppressors
Aerobic-glycolysis specific suppressors
Media independent suppressors
Figure S10. Suppression of doxorubicin cytotoxicity by perturbation of sphingolipid and ceramide metabolism. The diagram was modified from the SGD YeastPathways database at https://pathway.yeastgenome.org/YEAST/NEW-IMAGE?type=PATHWAY&object=SPHINGOLIPID-SYN-PWY&detail-level=2&detail-level=1. Circled genes are color-coded by their Warburg-dependence. Interaction profiles for pathway genes are assembled in the heatmap inset.

## Slide 12
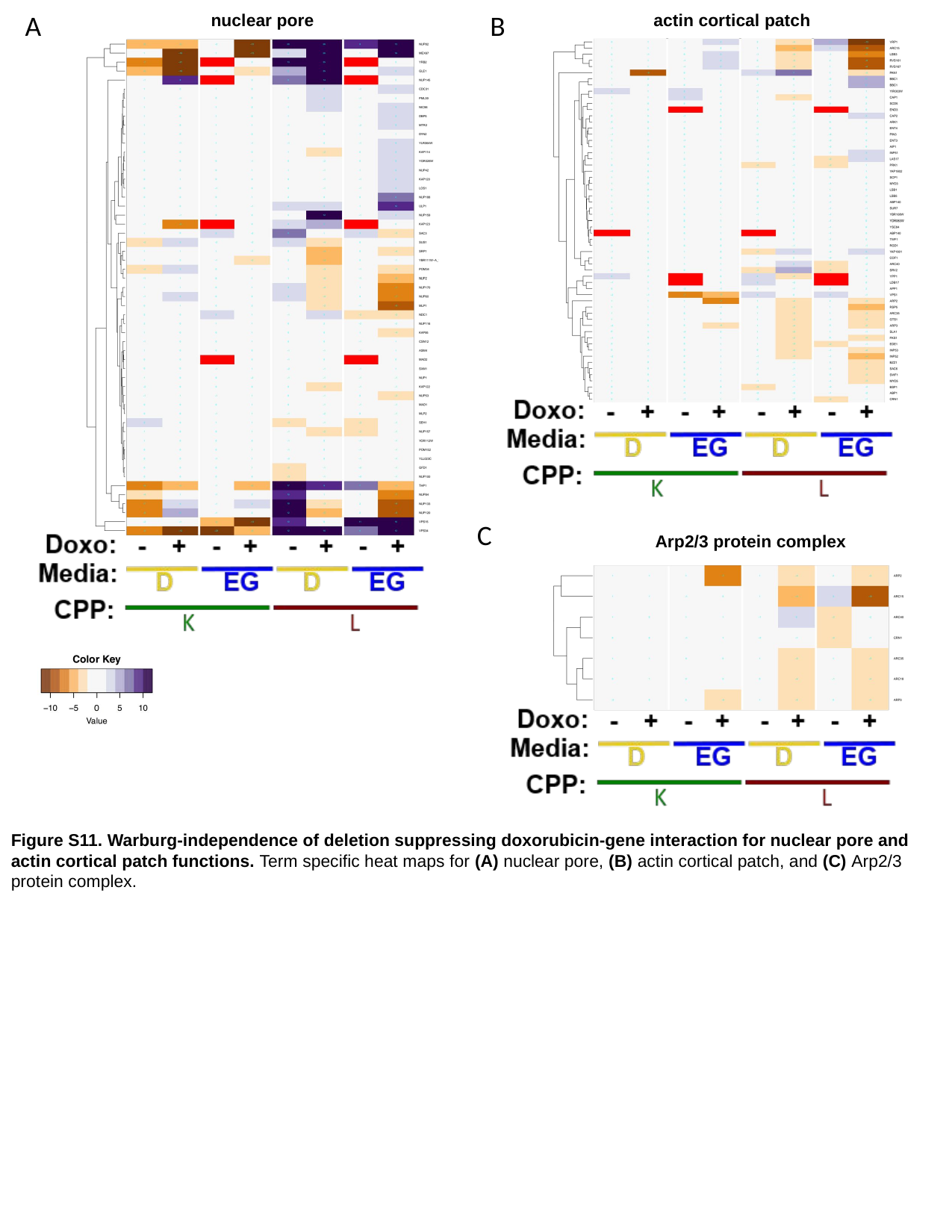

A
B
nuclear pore
actin cortical patch
C
Arp2/3 protein complex
Figure S11. Warburg-independence of deletion suppressing doxorubicin-gene interaction for nuclear pore and actin cortical patch functions. Term specific heat maps for (A) nuclear pore, (B) actin cortical patch, and (C) Arp2/3 protein complex.
