# Supplementary material for: A yeast phenomic model for the influence of Warburg metabolism on genetic buffering of doxorubicin
Source: Cancer Metab. 2019 Oct 23;7:9. doi: 10.1186/s40170-019-0201-3 (PMC6806529; doi:10.1186/s40170-019-0201-3)
Supplement: Supplementary file 6 — Additional file 6. Gene Ontology Term Averaging (GTA) results and interactive plots. File A contains all GTA values, cross-referenced with REMc-enriched terms. File B displays GTA values associated with above-threshold GTA scores (see note below) plotted for HLD vs. HLEG. GTA values for REMc-enriched terms are also included (regardless of whether |GTA score| >2). File C displays a subset of File B, containing only GO Terms with above-threshold GTA scores and that were enriched by REMc/GTF. File D reports GTA value using the K parameter. Files B-D should be opened in an Internet web browser so that embedded information from File A can be viewed by scrolling over points on the graphs. Subsets in each of the plots can be toggled off and on by clicking on the respective legend label. In the embedded information, X1 represents HLEG and X2 represents HLD information. Note: The GTA score threshold (for L) indicates that GTA-gtaSD > 2 for enhancers or GTA+gtaSD < -2 for suppressors, in at least one media. [file 40170_2019_201_MOESM6_ESM.bz2 › Additional_File6_GTA/B - All_GTA_Above_Threshold_and_REMc_enriched_Terms.html]

<!DOCTYPE html>
