# Supplementary material for: A yeast phenomic model for the influence of Warburg metabolism on genetic buffering of doxorubicin
Source: Cancer Metab. 2019 Oct 23;7:9. doi: 10.1186/s40170-019-0201-3 (PMC6806529; doi:10.1186/s40170-019-0201-3)
Supplement: Supplementary file 8 — Additional file 8. Quantitative summaries of REMc clusters. File A depicts REMc results, in terms of cluster distributions of L and K interaction (‘shift’ is not used for REMc and thus is not displayed), as a way to visualize cluster differences quantitatively. File B is organized by first round clusters and plots the change in p-value for significant terms with respect to round of clustering. Clusters derived from one another and sharing enrichment of the same GO term are connected by a line. Only GO terms with a background size of 500 or smaller are included. Scroll over a symbol to see embedded detail about each GO term. The square root of the p-value is used on the y-axis to evenly distribute data. [file 40170_2019_201_MOESM8_ESM.bz2 › Additional_File8_REMc_SignificanceByRound/A - Clust_Scores_by_first_rd_boxplots.pdf]

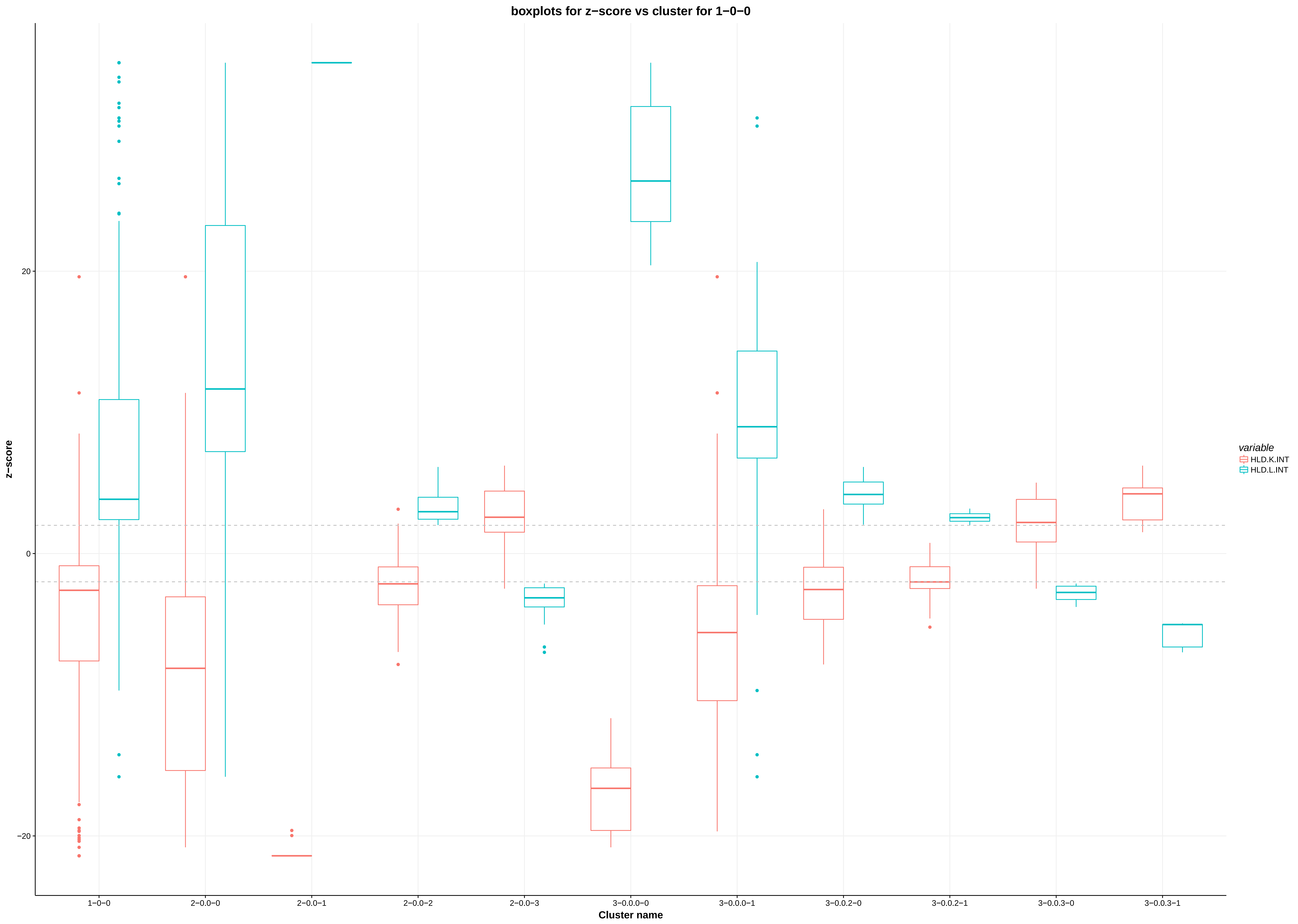

boxplots for z−score vs cluster for 1−0−0
z−score
20
0
−20
1−0−0
2−0.0−0
2−0.0−1
2−0.0−2
2−0.0−3
3−0.0.0−0
3−0.0.0−1
3−0.0.2−0
3−0.0.2−1
3−0.0.3−0
3−0.0.3−1
Cluster name
variable
HLD.K.INT
HLD.L.INT

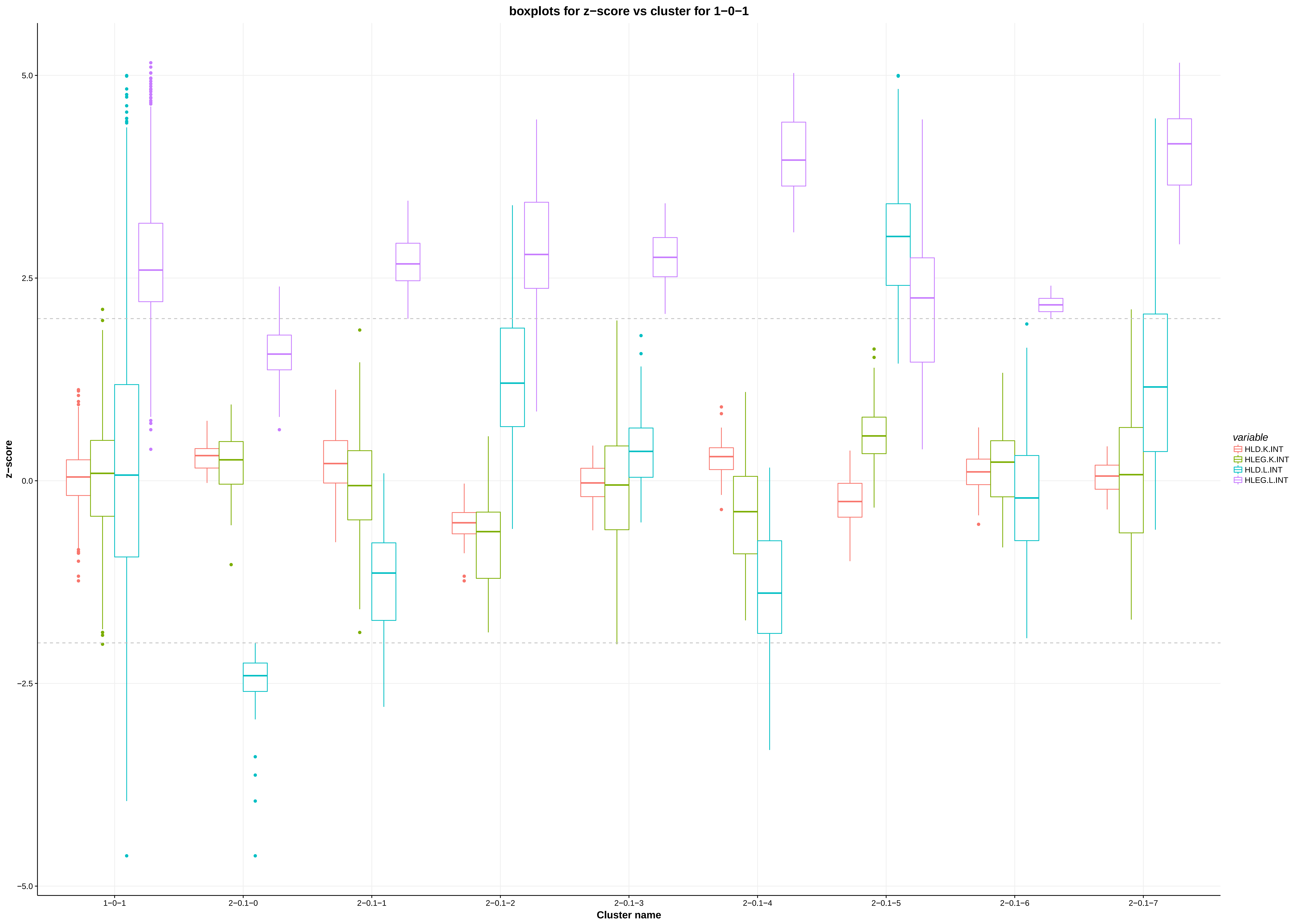
boxplots for z−score vs cluster for 1−0−1
z−score
5.0
2.5
0.0
−2.5
−5.0
1−0−1
2−0.1−0
2−0.1−1
2−0.1−2
2−0.1−3
2−0.1−4
2−0.1−5
2−0.1−6
2−0.1−7
Cluster name
variable
HLD.K.INT
HLEG.K.INT
HLD.L.INT
HLEG.L.INT

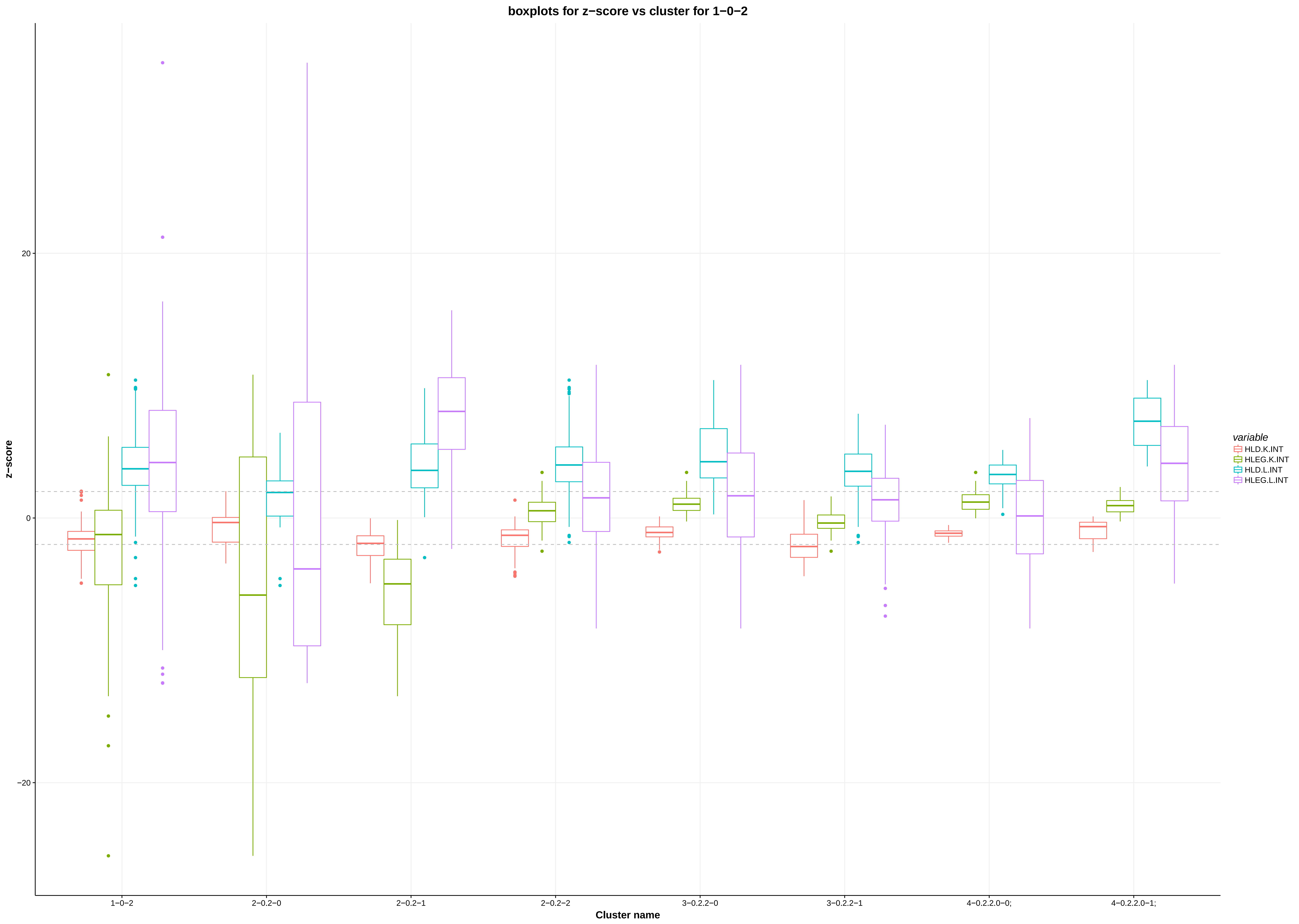

boxplots for z−score vs cluster for 1−0−2
z−score
20
0
−20
Cluster name
1−0−2
2−0.2−0
2−0.2−1
2−0.2−2
3−0.2.2−0
3−0.2.2−1
4−0.2.2.0−0;
4−0.2.2.0−1;
variable
HLD.K.INT
HLEG.K.INT
HLD.L.INT
HLEG.L.INT

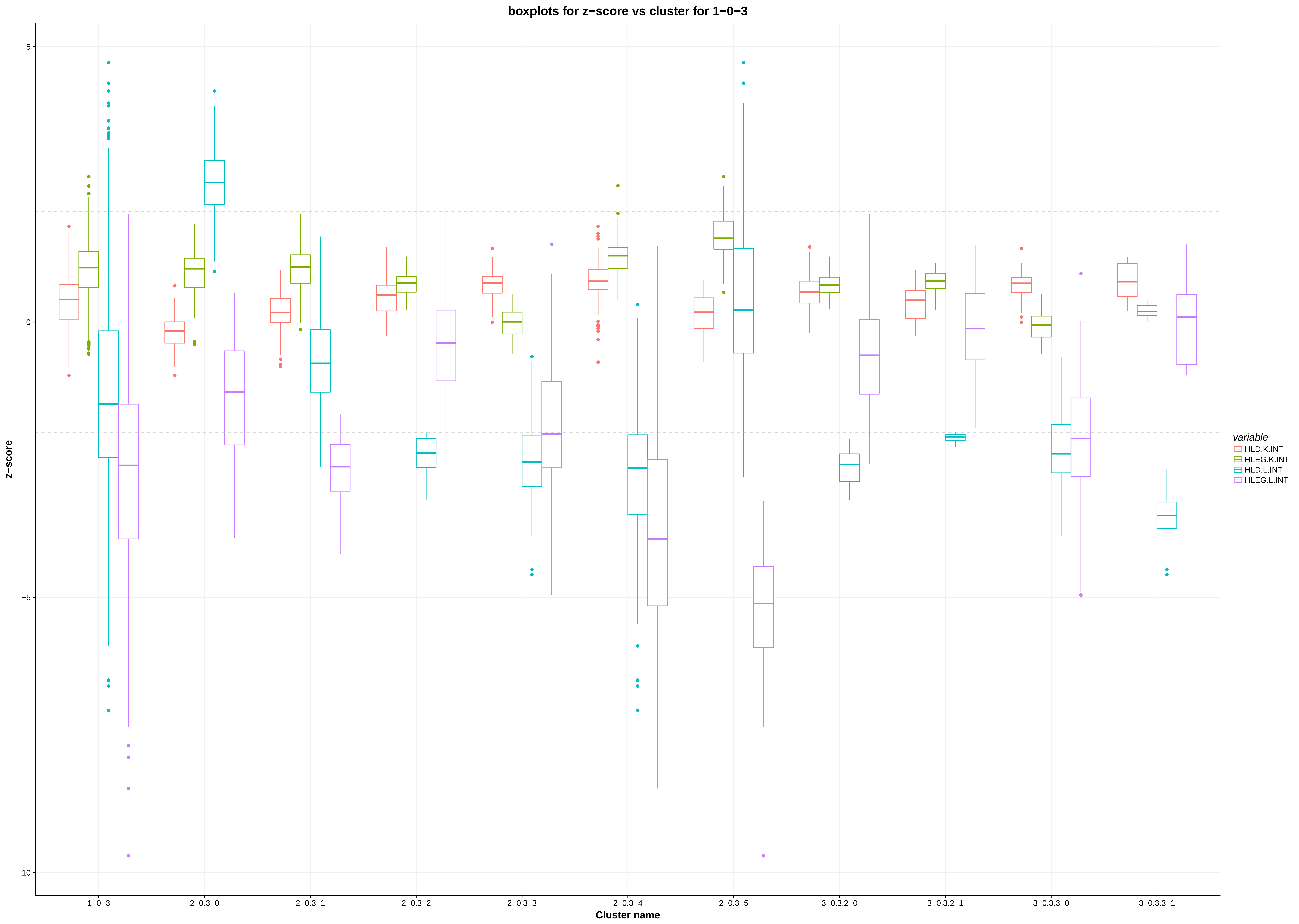

boxplots for z−score vs cluster for 1−0−3
z−score
5
0
−5
−10
Cluster name
1−0−3
2−0.3−0
2−0.3−1
2−0.3−2
2−0.3−3
2−0.3−4
2−0.3−5
3−0.3.2−0
3−0.3.2−1
3−0.3.3−0
3−0.3.3−1
variable
HLD.K.INT
HLEG.K.INT
HLD.L.INT
HLEG.L.INT

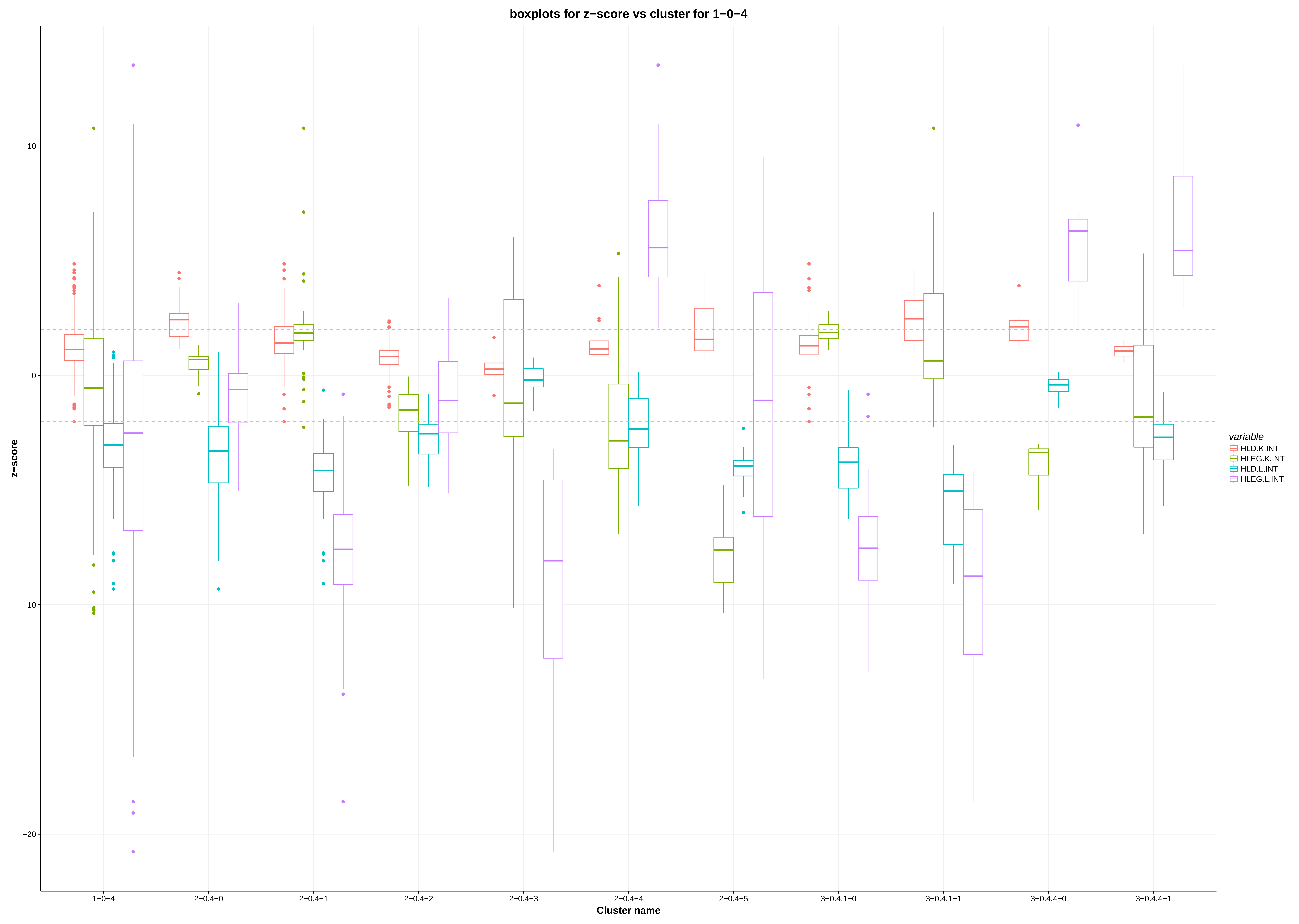
boxplots for z−score vs cluster for 1−0−4
z−score
10
0
−10
−20
Cluster name
1−0−4
2−0.4−0
2−0.4−1
2−0.4−2
2−0.4−3
2−0.4−4
2−0.4−5
3−0.4.1−0
3−0.4.1−1
3−0.4.4−0
3−0.4.4−1
variable
HLD.K.INT
HLEG.K.INT
HLD.L.INT
HLEG.L.INT

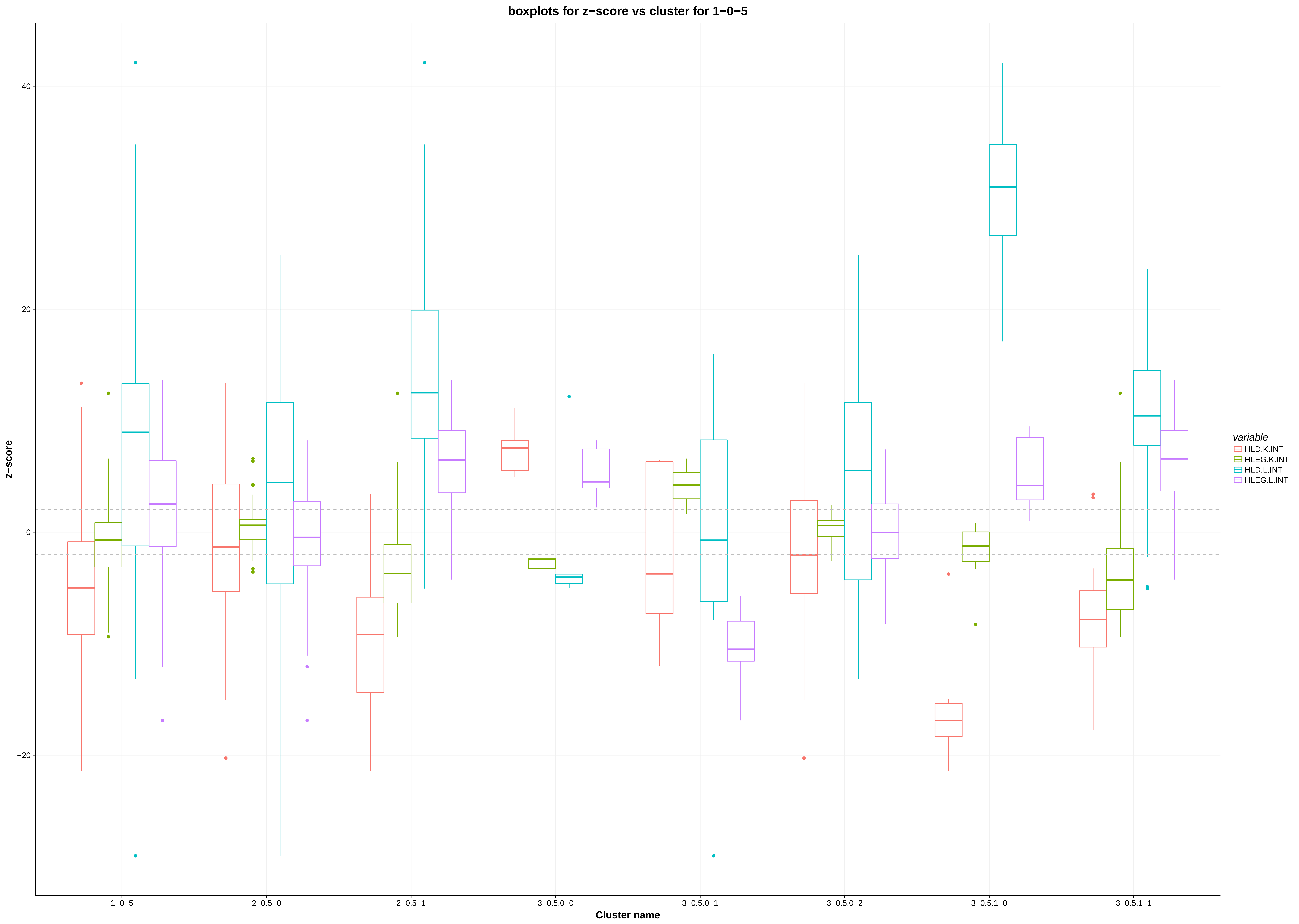
boxplots for z−score vs cluster for 1−0−5
z−score
40
20
0
−20
Cluster name
1−0−5
2−0.5−0
2−0.5−1
3−0.5.0−0
3−0.5.0−1
3−0.5.0−2
3−0.5.1−0
3−0.5.1−1
variable
HLD.K.INT
HLEG.K.INT
HLD.L.INT
HLEG.L.INT

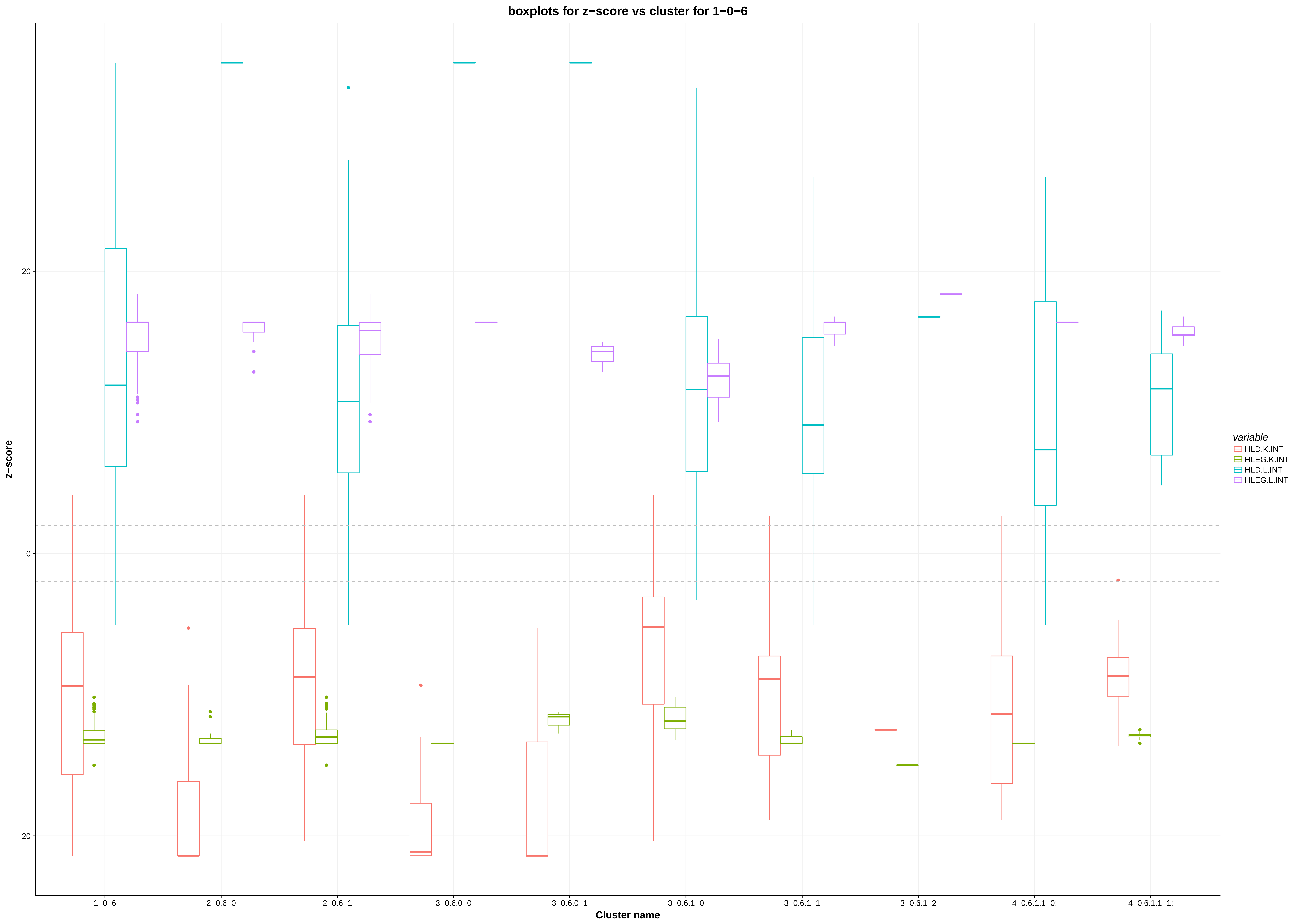

boxplots for z−score vs cluster for 1−0−6
z−score
20
0
−20
Cluster name
1−0−6
2−0.6−0
2−0.6−1
3−0.6.0−0
3−0.6.0−1
3−0.6.1−0
3−0.6.1−1
3−0.6.1−2
4−0.6.1.1−0;
4−0.6.1.1−1;
variable
HLD.K.INT
HLEG.K.INT
HLD.L.INT
HLEG.L.INT

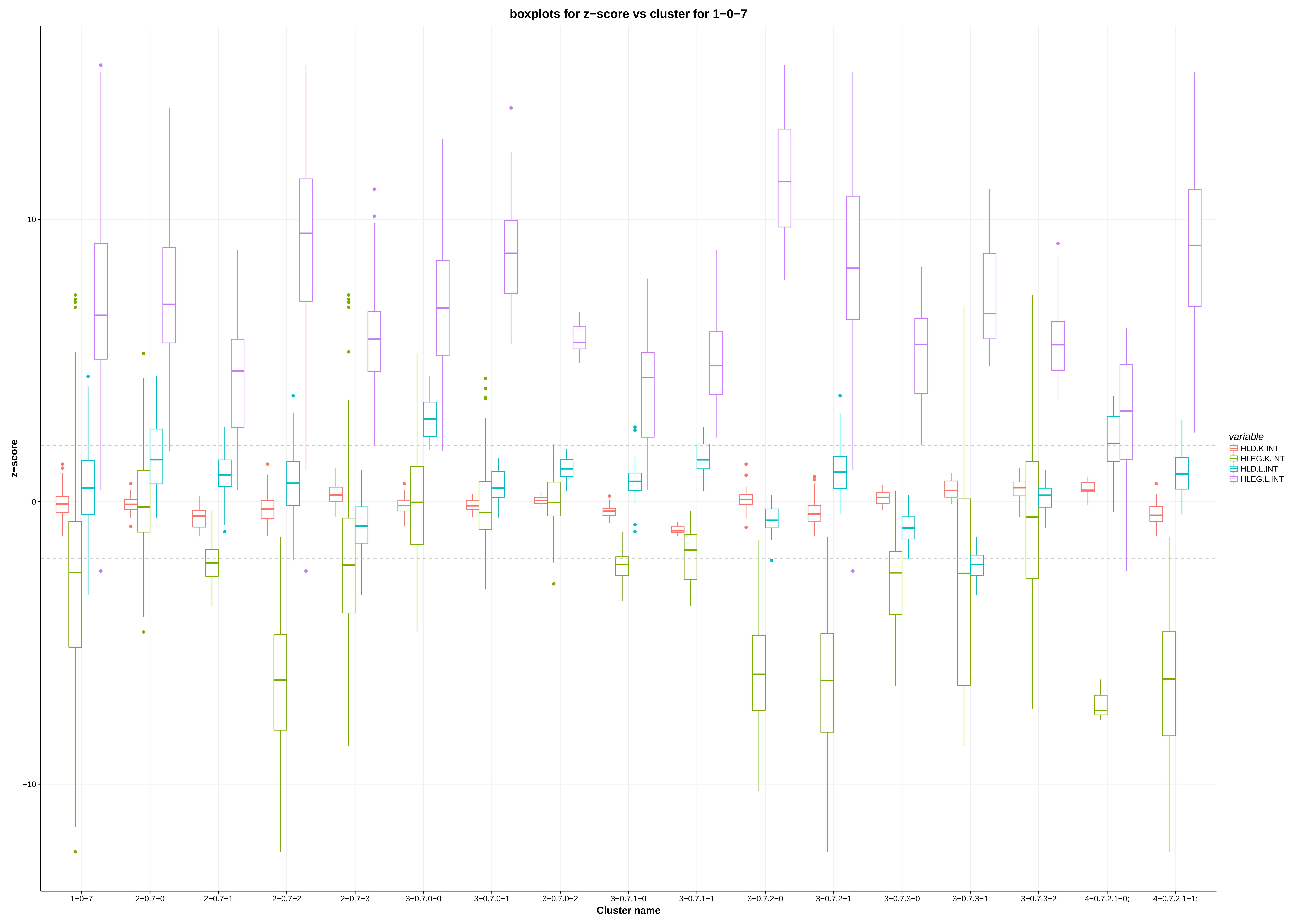

boxplots for z−score vs cluster for 1−0−7
z−score
10
0
−10
Cluster name
1−0−7
2−0.7−0
2−0.7−1
2−0.7−2
2−0.7−3
3−0.7.0−0
3−0.7.0−1
3−0.7.0−2
3−0.7.1−0
3−0.7.1−1
3−0.7.2−0
3−0.7.2−1
3−0.7.3−0
3−0.7.3−1
3−0.7.3−2
4−0.7.2.1−0;
4−0.7.2.1−1;
variable
HLD.K.INT
HLEG.K.INT
HLD.L.INT
HLEG.L.INT

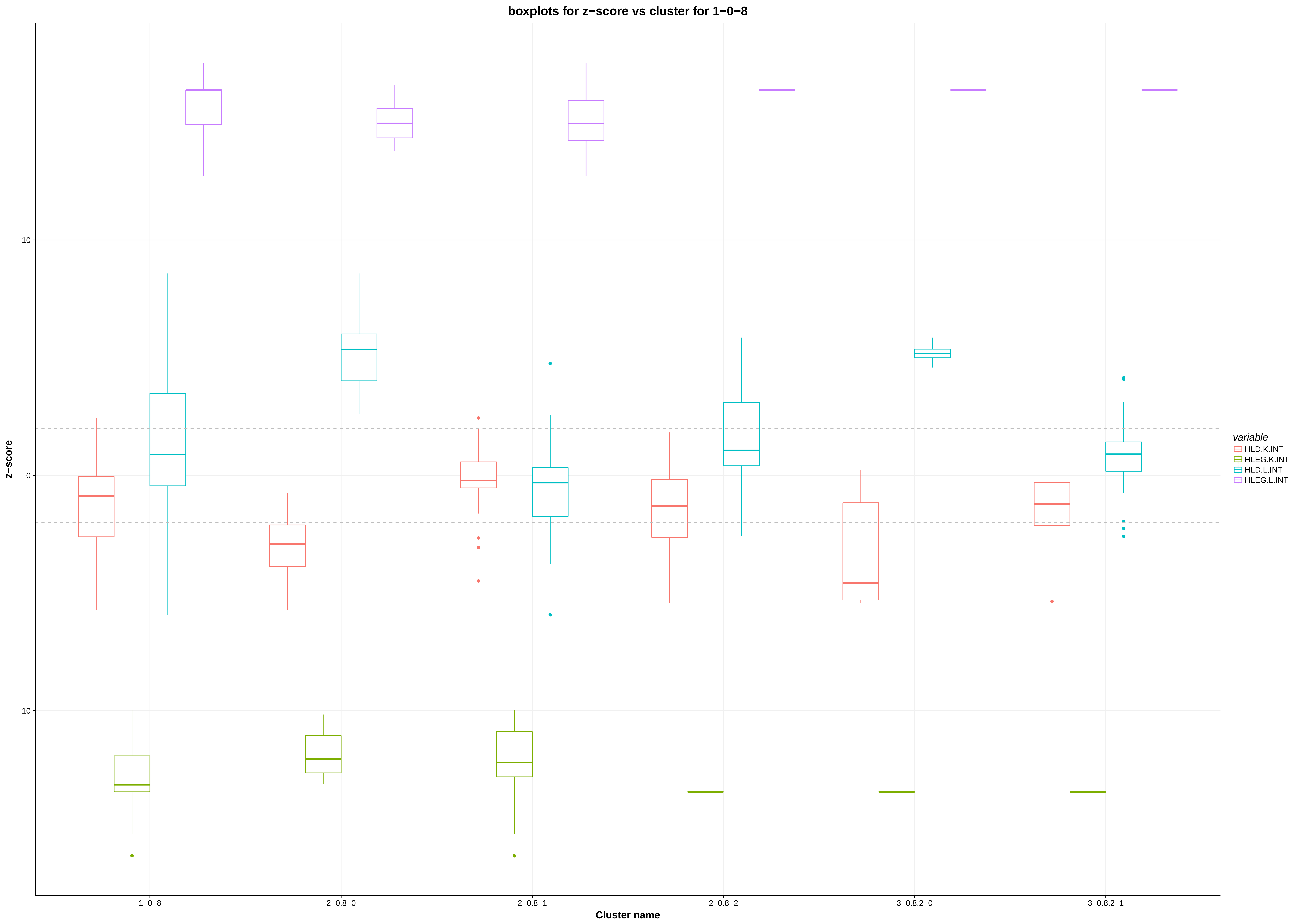

boxplots for z−score vs cluster for 1−0−8
z−score
10
0
−10
Cluster name
1−0−8
2−0.8−0
2−0.8−1
2−0.8−2
3−0.8.2−0
3−0.8.2−1
variable
HLD.K.INT
HLEG.K.INT
HLD.L.INT
HLEG.L.INT
