# Supplementary material for: A yeast phenomic model for the influence of Warburg metabolism on genetic buffering of doxorubicin
Source: Cancer Metab. 2019 Oct 23;7:9. doi: 10.1186/s40170-019-0201-3 (PMC6806529; doi:10.1186/s40170-019-0201-3)
Supplement: Supplementary file 9 — Additional file 9. GO term-specific heatmaps for REMc/GTF-enriched clusters. GO term-specific heatmaps for significant GO process terms were generated as described in methods and Figs. 3 and 4. Any related child terms are presented in subsequent pages of the parent file name. GO terms with more than 100 children, with 2 or fewer genes annotated to the term, or a file size over 300KB are not shown. All heatmaps are generated with the same layout (see Figs. 3 and 4). [file 40170_2019_201_MOESM9_ESM.bz2 › Additional_File9_GOTermHeatmaps/Additional_File9_GOTermHeatmap/ATP_hydrolysis_coupled_cation_transmembrane_transport.pdf]

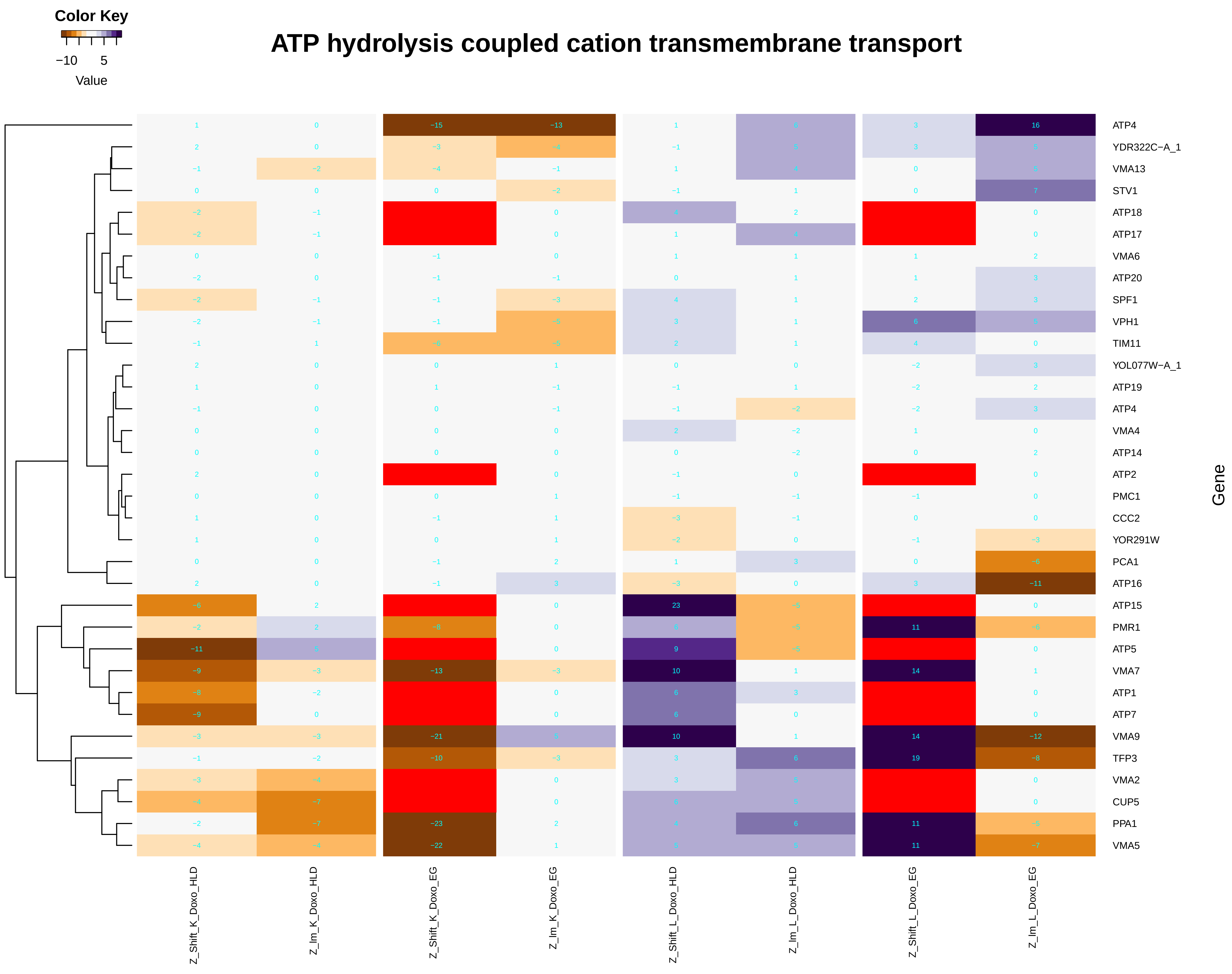

Color Key
−10
5
Value
ATP hydrolysis coupled cation transmembrane transport
ATP4
YDR322C−A_1
VMA13
STV1
ATP18
ATP17
VMA6
ATP20
SPF1
VPH1
TIM11
YOL077W−A_1
ATP19
ATP4
VMA4
ATP14
ATP2
PMC1
CCC2
YOR291W
PCA1
ATP16
ATP15
PMR1
ATP5
VMA7
ATP1
ATP7
VMA9
TFP3
VMA2
CUP5
PPA1
VMA5
Gene
Z_Shift_K_Doxo_HLD
Z_Im_K_Doxo_HLD
Z_Shift_K_Doxo_EG
Z_Im_K_Doxo_EG
Z_Shift_L_Doxo_HLD
Z_Im_L_Doxo_HLD
Z_Shift_L_Doxo_EG
Z_Im_L_Doxo_EG

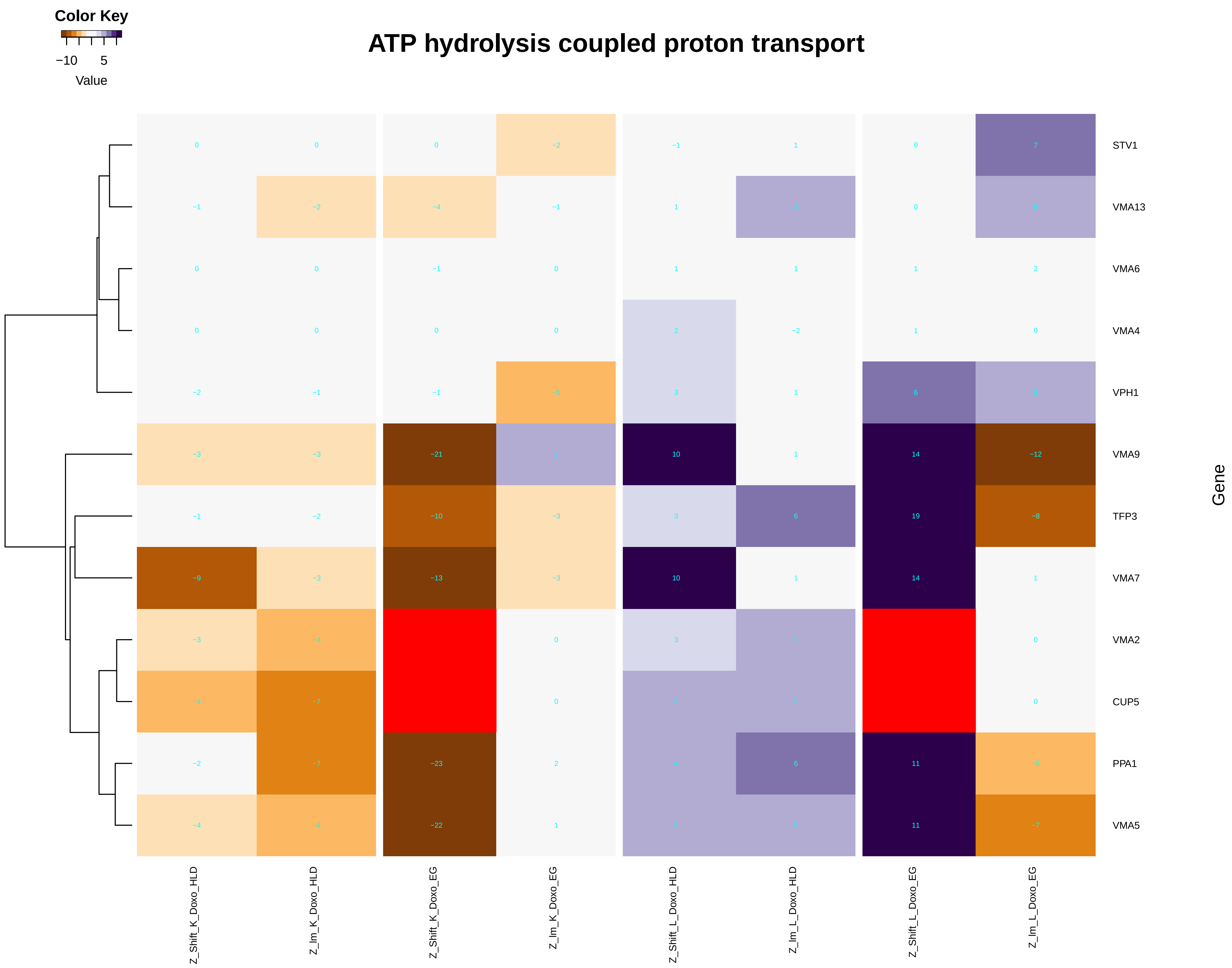
Color Key
−10
5
Value
ATP hydrolysis coupled proton transport
STV1
VMA13
VMA6
VMA4
VPH1
VMA9
TFP3
VMA7
VMA2
CUP5
PPA1
VMA5
Gene
Z_Shift_K_Doxo_HLD
Z_Im_K_Doxo_HLD
Z_Shift_K_Doxo_EG
Z_Im_K_Doxo_EG
Z_Shift_L_Doxo_HLD
Z_Im_L_Doxo_HLD
Z_Shift_L_Doxo_EG
Z_Im_L_Doxo_EG
