# Supplementary material for: A yeast phenomic model for the influence of Warburg metabolism on genetic buffering of doxorubicin
Source: Cancer Metab. 2019 Oct 23;7:9. doi: 10.1186/s40170-019-0201-3 (PMC6806529; doi:10.1186/s40170-019-0201-3)
Supplement: Supplementary file 9 — Additional file 9. GO term-specific heatmaps for REMc/GTF-enriched clusters. GO term-specific heatmaps for significant GO process terms were generated as described in methods and Figs. 3 and 4. Any related child terms are presented in subsequent pages of the parent file name. GO terms with more than 100 children, with 2 or fewer genes annotated to the term, or a file size over 300KB are not shown. All heatmaps are generated with the same layout (see Figs. 3 and 4). [file 40170_2019_201_MOESM9_ESM.bz2 › Additional_File9_GOTermHeatmaps/Additional_File9_GOTermHeatmap/cell_cycle_DNA_replication.pdf]

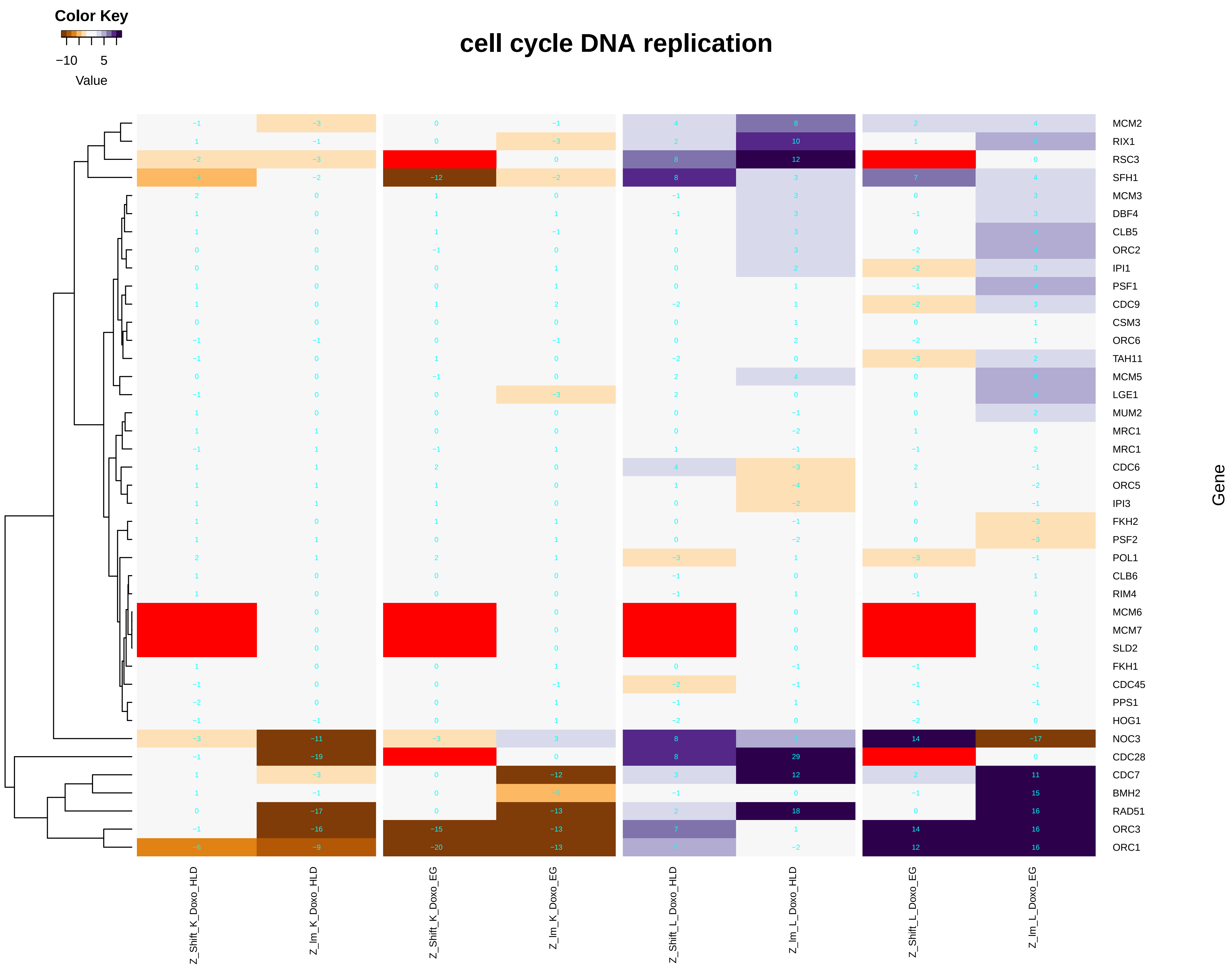

Color Key
−10
5
Value
cell cycle DNA replication
MCM2
RIX1
RSC3
SFH1
MCM3
DBF4
CLB5
ORC2
IPI1
PSF1
CDC9
CSM3
ORC6
TAH11
MCM5
LGE1
MUM2
MRC1
MRC1
CDC6
ORC5
IPI3
FKH2
PSF2
POL1
CLB6
RIM4
MCM6
MCM7
SLD2
FKH1
CDC45
PPS1
HOG1
NOC3
CDC28
CDC7
BMH2
RAD51
ORC3
ORC1
Gene
Z_Shift_K_Doxo_HLD
Z_Im_K_Doxo_HLD
Z_Shift_K_Doxo_EG
Z_Im_K_Doxo_EG
Z_Shift_L_Doxo_HLD
Z_Im_L_Doxo_HLD
Z_Shift_L_Doxo_EG
Z_Im_L_Doxo_EG

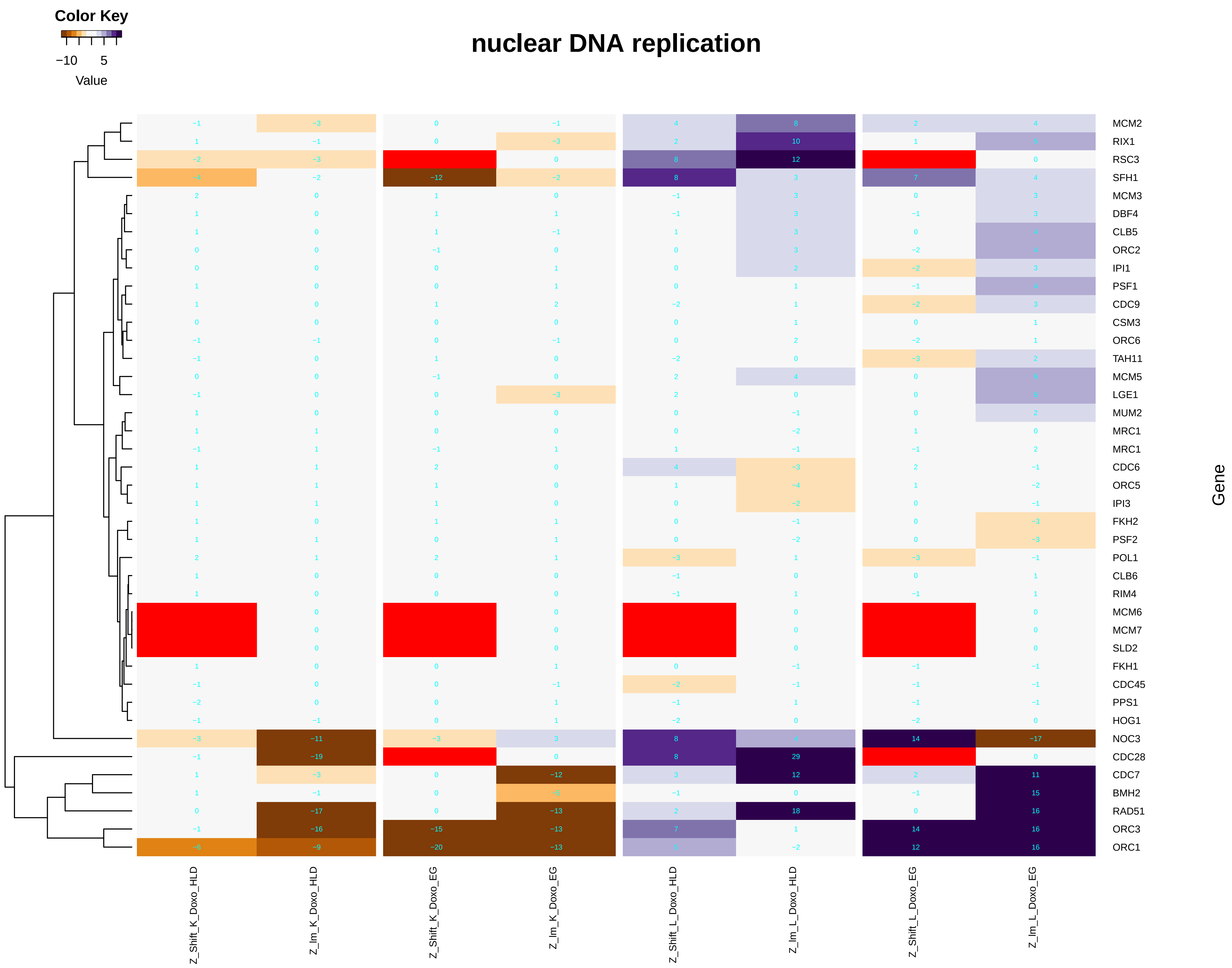

Color Key
−10
5
Value
nuclear DNA replication
MCM2
RIX1
RSC3
SFH1
MCM3
DBF4
CLB5
ORC2
IPI1
PSF1
CDC9
CSM3
ORC6
TAH11
MCM5
LGE1
MUM2
MRC1
MRC1
CDC6
ORC5
IPI3
FKH2
PSF2
POL1
CLB6
RIM4
MCM6
MCM7
SLD2
FKH1
CDC45
PPS1
HOG1
NOC3
CDC28
CDC7
BMH2
RAD51
ORC3
ORC1
Gene
Z_Shift_K_Doxo_HLD
Z_Im_K_Doxo_HLD
Z_Shift_K_Doxo_EG
Z_Im_K_Doxo_EG
Z_Shift_L_Doxo_HLD
Z_Im_L_Doxo_HLD
Z_Shift_L_Doxo_EG
Z_Im_L_Doxo_EG

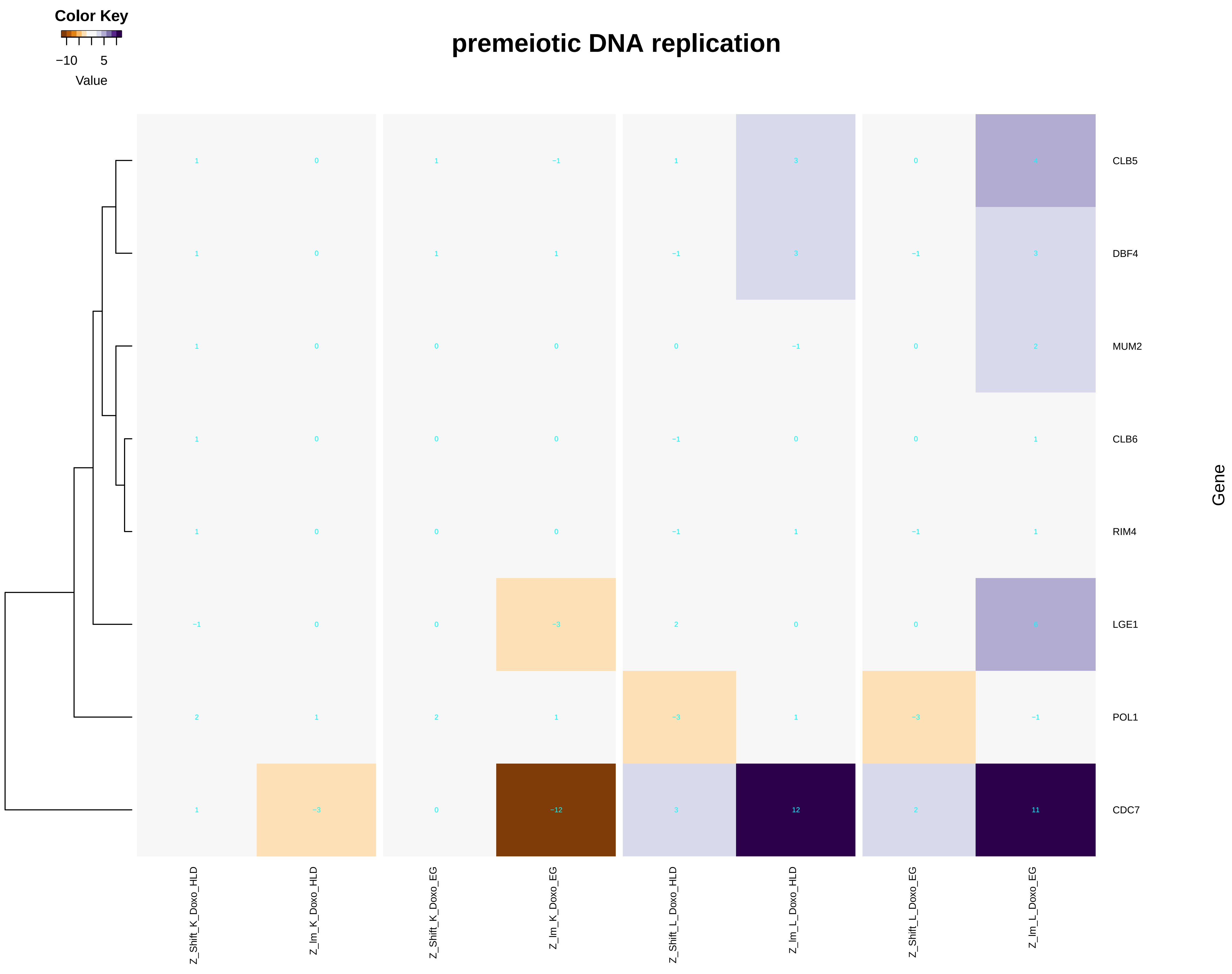

Color Key
−10 5
Value
premeiotic DNA replication
CLB5
DBF4
MUM2
CLB6
RIM4
LGE1
POL1
CDC7
Gene
Z_Shift_K_Doxo_HLD
Z_Im_K_Doxo_HLD
Z_Shift_K_Doxo_EG
Z_Im_K_Doxo_EG
Z_Shift_L_Doxo_HLD
Z_Im_L_Doxo_HLD
Z_Shift_L_Doxo_EG
Z_Im_L_Doxo_EG

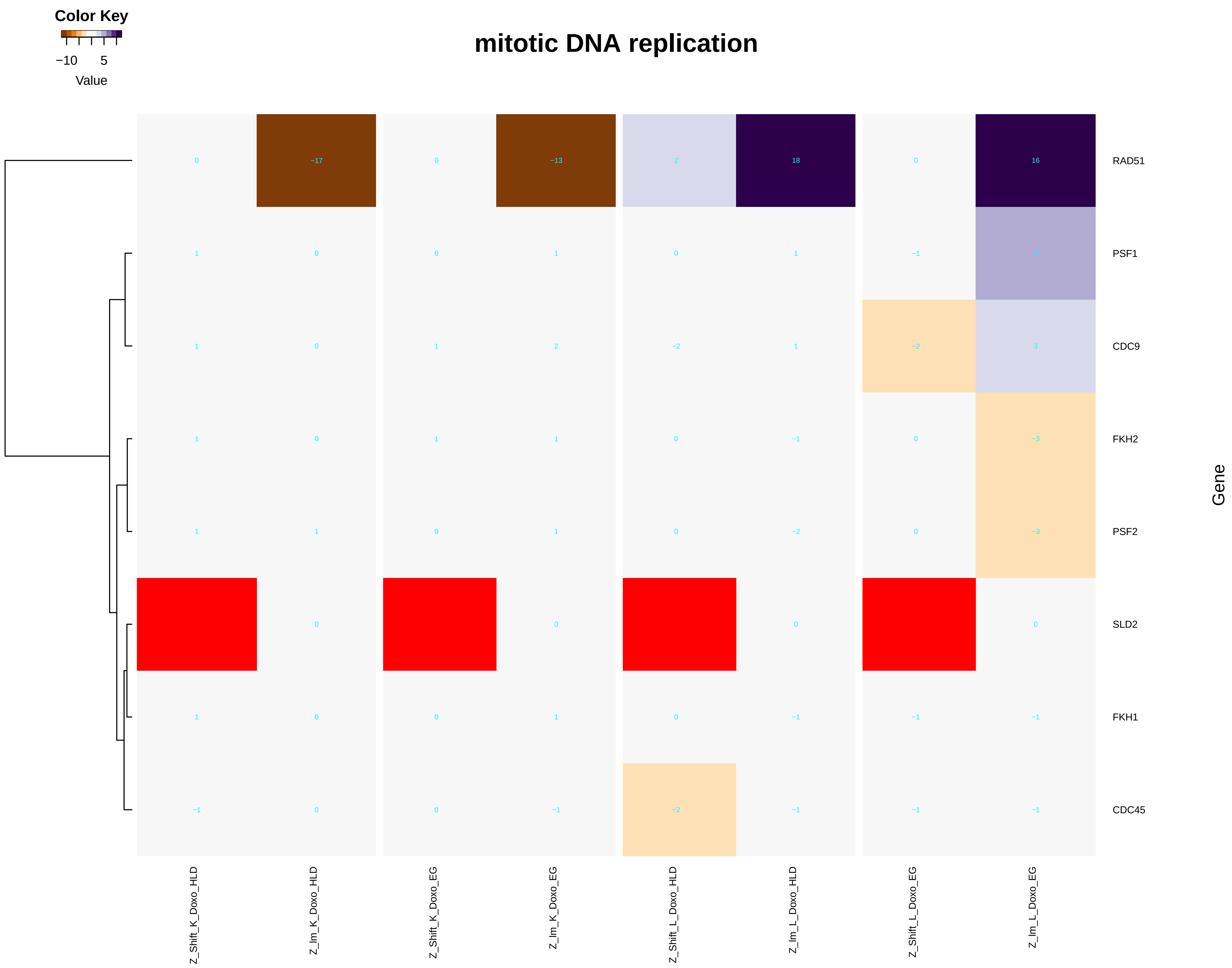

mitotic DNA replication
Color Key
−10 5
Value
0 −17 0 −13 2 18 0 16
1 0 0 1 0 1 −1 4
1 0 1 2 −2 1 −2 3
1 0 1 1 0 −1 0 −3
1 1 0 1 0 −2 0 −3
0 0 0 0
1 0 0 1 0 −1 −1 −1
−1 0 0 −1 −2 −1 −1 −1
RAD51
PSF1
CDC9
FKH2
PSF2
SLD2
FKH1
CDC45
Gene
Z_Shift_K_Doxo_HLD
Z_Im_K_Doxo_HLD
Z_Shift_K_Doxo_EG
Z_Im_K_Doxo_EG
Z_Shift_L_Doxo_HLD
Z_Im_L_Doxo_HLD
Z_Shift_L_Doxo_EG
Z_Im_L_Doxo_EG
