# Supplementary material for: A yeast phenomic model for the influence of Warburg metabolism on genetic buffering of doxorubicin
Source: Cancer Metab. 2019 Oct 23;7:9. doi: 10.1186/s40170-019-0201-3 (PMC6806529; doi:10.1186/s40170-019-0201-3)
Supplement: Supplementary file 9 — Additional file 9. GO term-specific heatmaps for REMc/GTF-enriched clusters. GO term-specific heatmaps for significant GO process terms were generated as described in methods and Figs. 3 and 4. Any related child terms are presented in subsequent pages of the parent file name. GO terms with more than 100 children, with 2 or fewer genes annotated to the term, or a file size over 300KB are not shown. All heatmaps are generated with the same layout (see Figs. 3 and 4). [file 40170_2019_201_MOESM9_ESM.bz2 › Additional_File9_GOTermHeatmaps/Additional_File9_GOTermHeatmap/cleavage_involved_in_rRNA_processing.pdf]

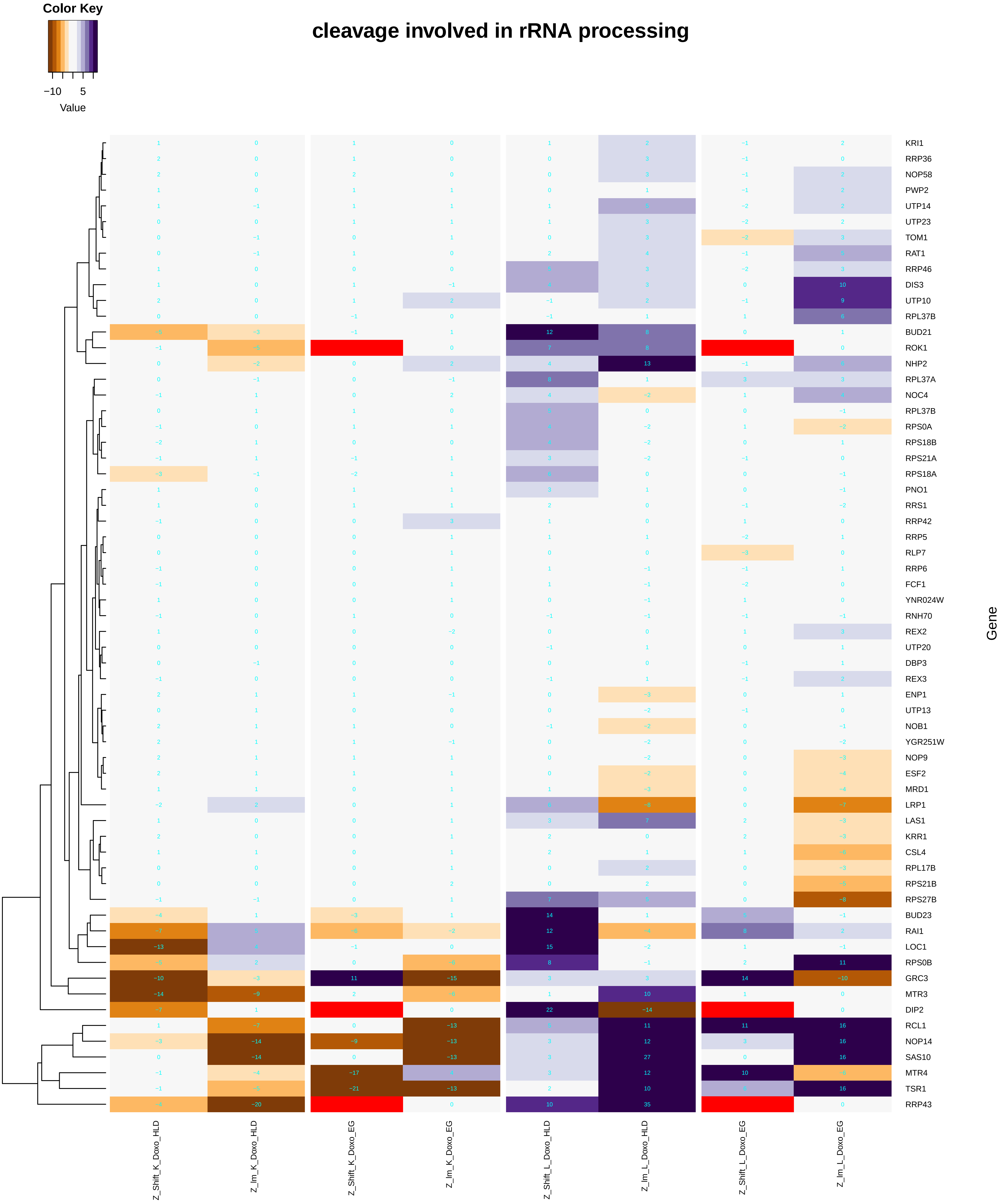
Color Key
−10
5
Value
cleavage involved in rRNA processing
KRI1
RRP36
NOP58
PWP2
UTP14
UTP23
TOM1
RAT1
RRP46
DIS3
UTP10
RPL37B
BUD21
ROK1
NHP2
RPL37A
NOC4
RPL37B
RPS0A
RPS18B
RPS21A
RPS18A
PNO1
RRS1
RRP42
RRP5
RLP7
RRP6
FCF1
YNR024W
RNH70
REX2
UTP20
DBP3
REX3
ENP1
UTP13
NOB1
YGR251W
NOP9
ESF2
MRD1
LRP1
LAS1
KRR1
CSL4
RPL17B
RPS21B
RPS27B
BUD23
RAI1
LOC1
RPS0B
GRC3
MTR3
DIP2
RCL1
NOP14
SAS10
MTR4
TSR1
RRP43
Gene
Z_Shift_K_Doxo_HLD
Z_Im_K_Doxo_HLD
Z_Shift_K_Doxo_EG
Z_Im_K_Doxo_EG
Z_Shift_L_Doxo_HLD
Z_Im_L_Doxo_HLD
Z_Shift_L_Doxo_EG
Z_Im_L_Doxo_EG

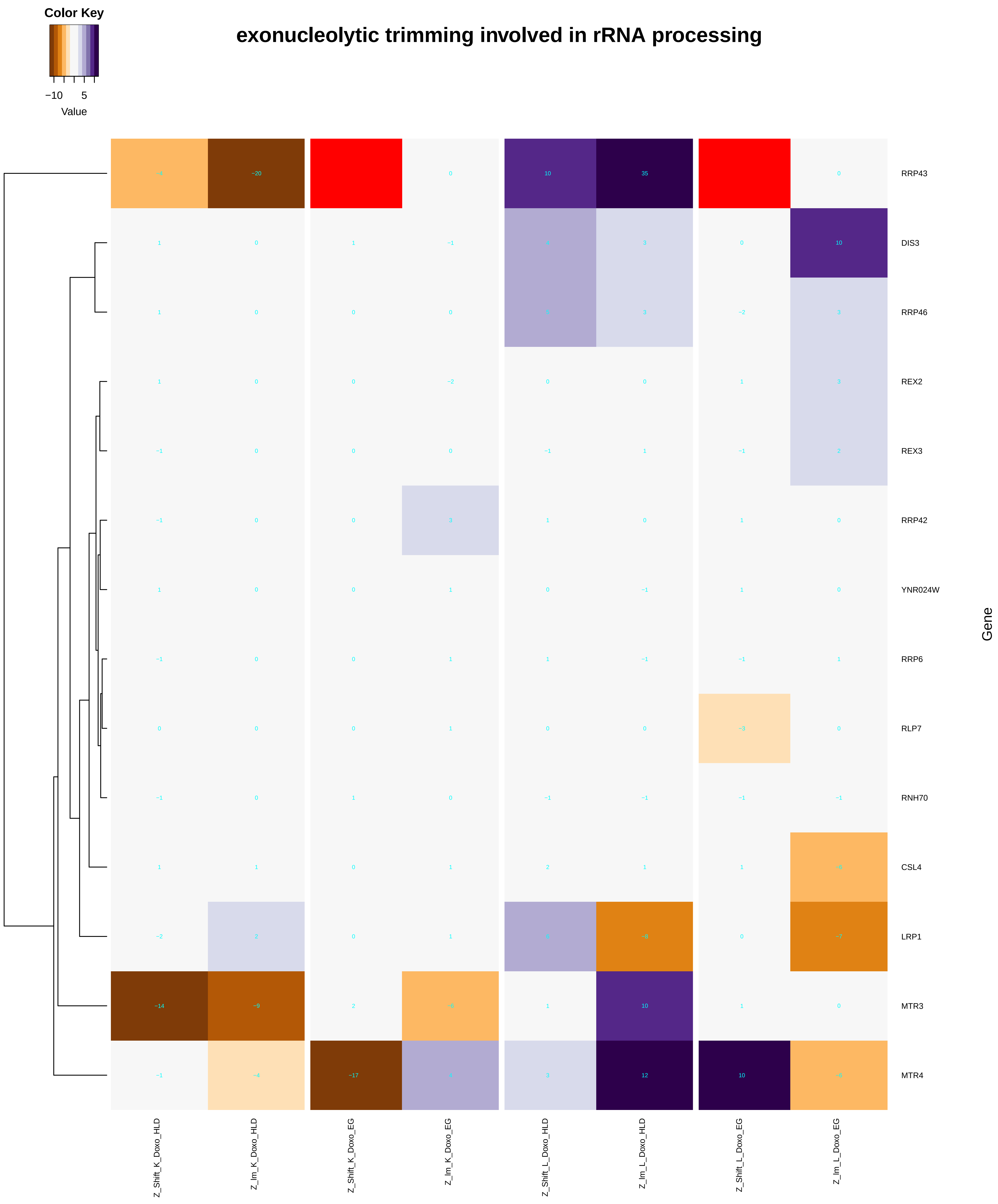

exonucleolytic trimming involved in rRNA processing
Color Key
−10
5
Value
RRP43
DIS3
RRP46
REX2
REX3
RRP42
YNR024W
RRP6
RLP7
RNH70
CSL4
LRP1
MTR3
MTR4
Gene
Z_Shift_K_Doxo_HLD
Z_Im_K_Doxo_HLD
Z_Shift_K_Doxo_EG
Z_Im_K_Doxo_EG
Z_Shift_L_Doxo_HLD
Z_Im_L_Doxo_HLD
Z_Shift_L_Doxo_EG
Z_Im_L_Doxo_EG

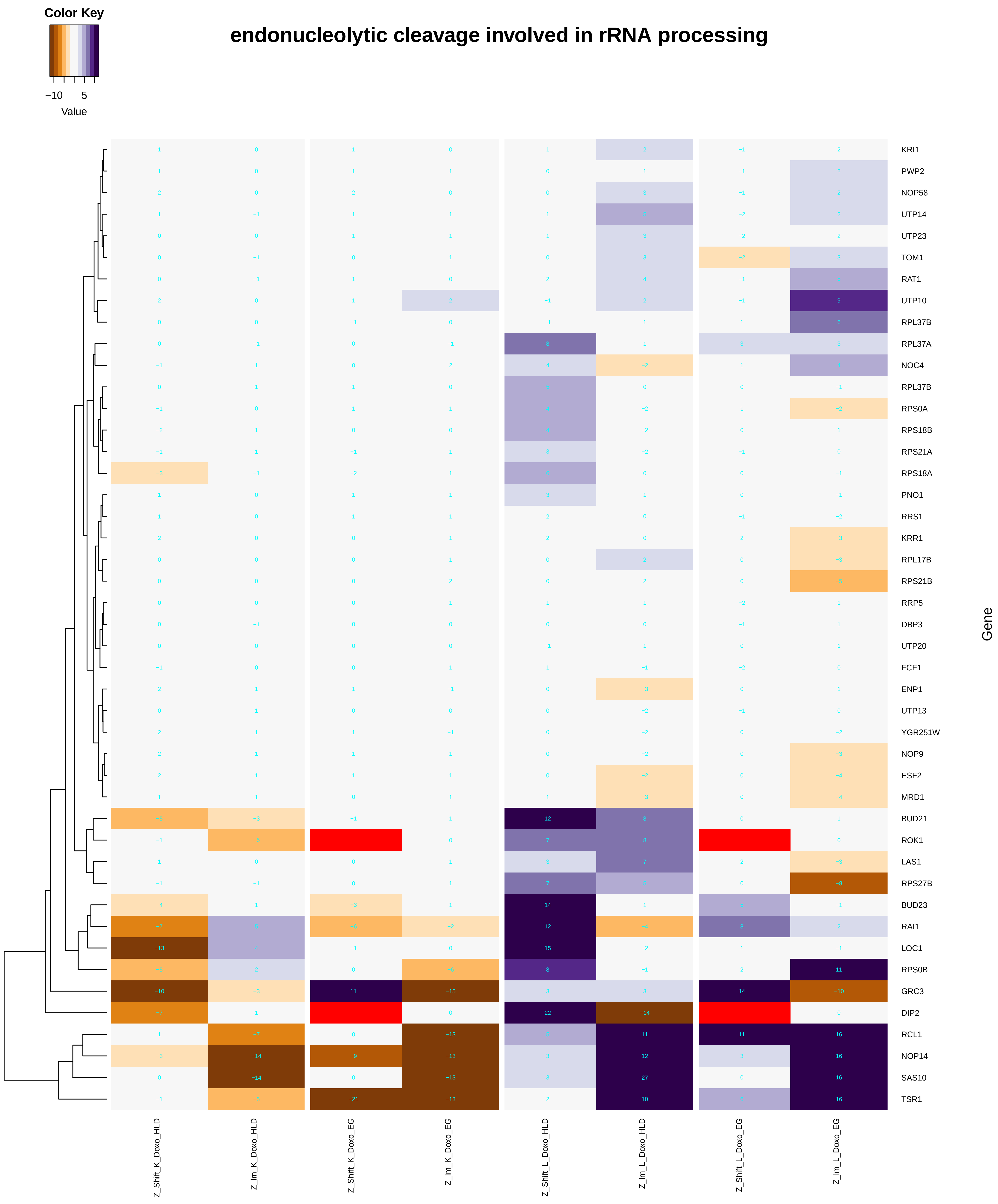
Color Key
−10
5
Value
endonucleolytic cleavage involved in rRNA processing
KRI1
PWP2
NOP58
UTP14
UTP23
TOM1
RAT1
UTP10
RPL37B
RPL37A
NOC4
RPL37B
RPS0A
RPS18B
RPS21A
RPS18A
PNO1
RRS1
KRR1
RPL17B
RPS21B
RRP5
DBP3
UTP20
FCF1
ENP1
UTP13
YGR251W
NOP9
ESF2
MRD1
BUD21
ROK1
LAS1
RPS27B
BUD23
RAI1
LOC1
RPS0B
GRC3
DIP2
RCL1
NOP14
SAS10
TSR1
Gene
Z_Shift_K_Doxo_HLD
Z_lm_K_Doxo_HLD
Z_Shift_K_Doxo_EG
Z_lm_K_Doxo_EG
Z_Shift_L_Doxo_HLD
Z_lm_L_Doxo_HLD
Z_Shift_L_Doxo_EG
Z_lm_L_Doxo_EG

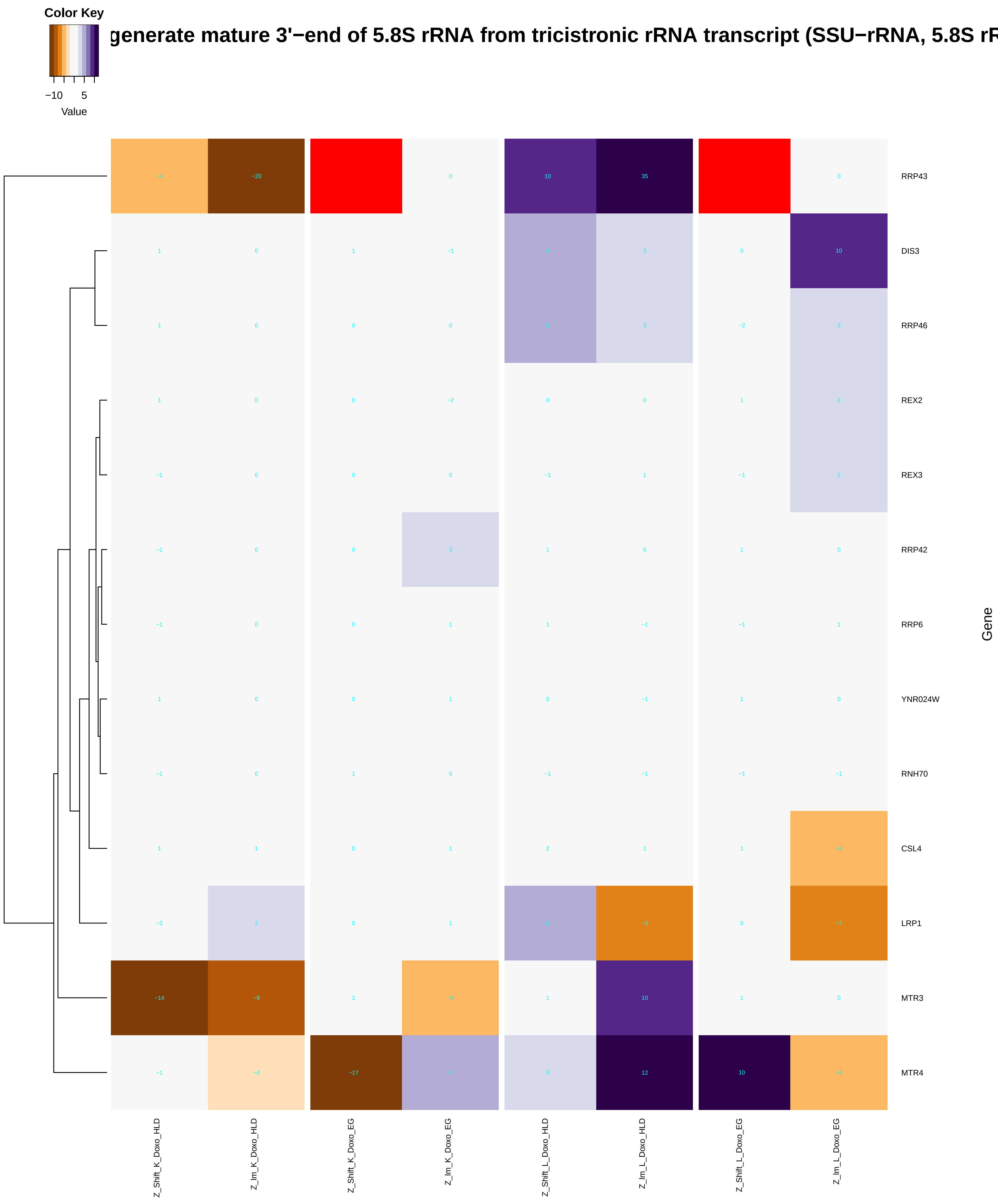
Color Key
−10
5
Value
generate mature 3'−end of 5.8S rRNA from tricistronic rRNA transcript (SSU−rRNA, 5.8S rR
RRP43
DIS3
RRP46
REX2
REX3
RRP42
RRP6
YNR024W
RNH70
CSL4
LRP1
MTR3
MTR4
Gene
Z_Shift_K_Doxo_HLD
Z_lm_K_Doxo_HLD
Z_Shift_K_Doxo_EG
Z_lm_K_Doxo_EG
Z_Shift_L_Doxo_HLD
Z_lm_L_Doxo_HLD
Z_Shift_L_Doxo_EG
Z_lm_L_Doxo_EG

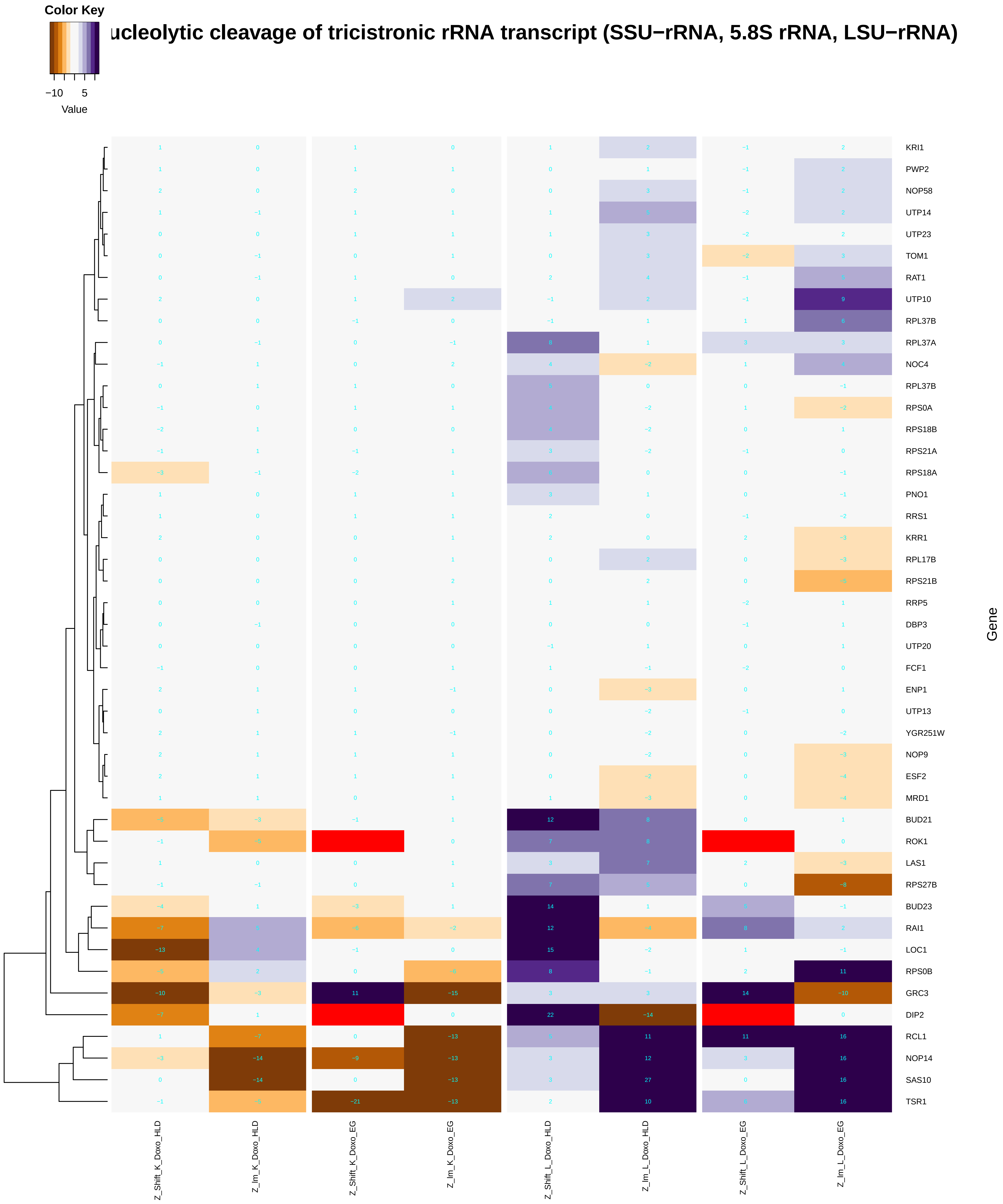
Color Key
ucleolytic cleavage of tricistronic rRNA transcript (SSU−rRNA, 5.8S rRNA, LSU−rRNA)
−10
5
Value
KRI1
PWP2
NOP58
UTP14
UTP23
TOM1
RAT1
UTP10
RPL37B
RPL37A
NOC4
RPL37B
RPS0A
RPS18B
RPS21A
RPS18A
PNO1
RRS1
KRR1
RPL17B
RPS21B
RRP5
DBP3
UTP20
FCF1
ENP1
UTP13
YGR251W
NOP9
ESF2
MRD1
BUD21
ROK1
LAS1
RPS27B
BUD23
RAI1
LOC1
RPS0B
GRC3
DIP2
RCL1
NOP14
SAS10
TSR1
Gene
Z_Shift_K_Doxo_HLD
Z_Im_K_Doxo_HLD
Z_Shift_K_Doxo_EG
Z_Im_K_Doxo_EG
Z_Shift_L_Doxo_HLD
Z_Im_L_Doxo_HLD
Z_Shift_L_Doxo_EG
Z_Im_L_Doxo_EG

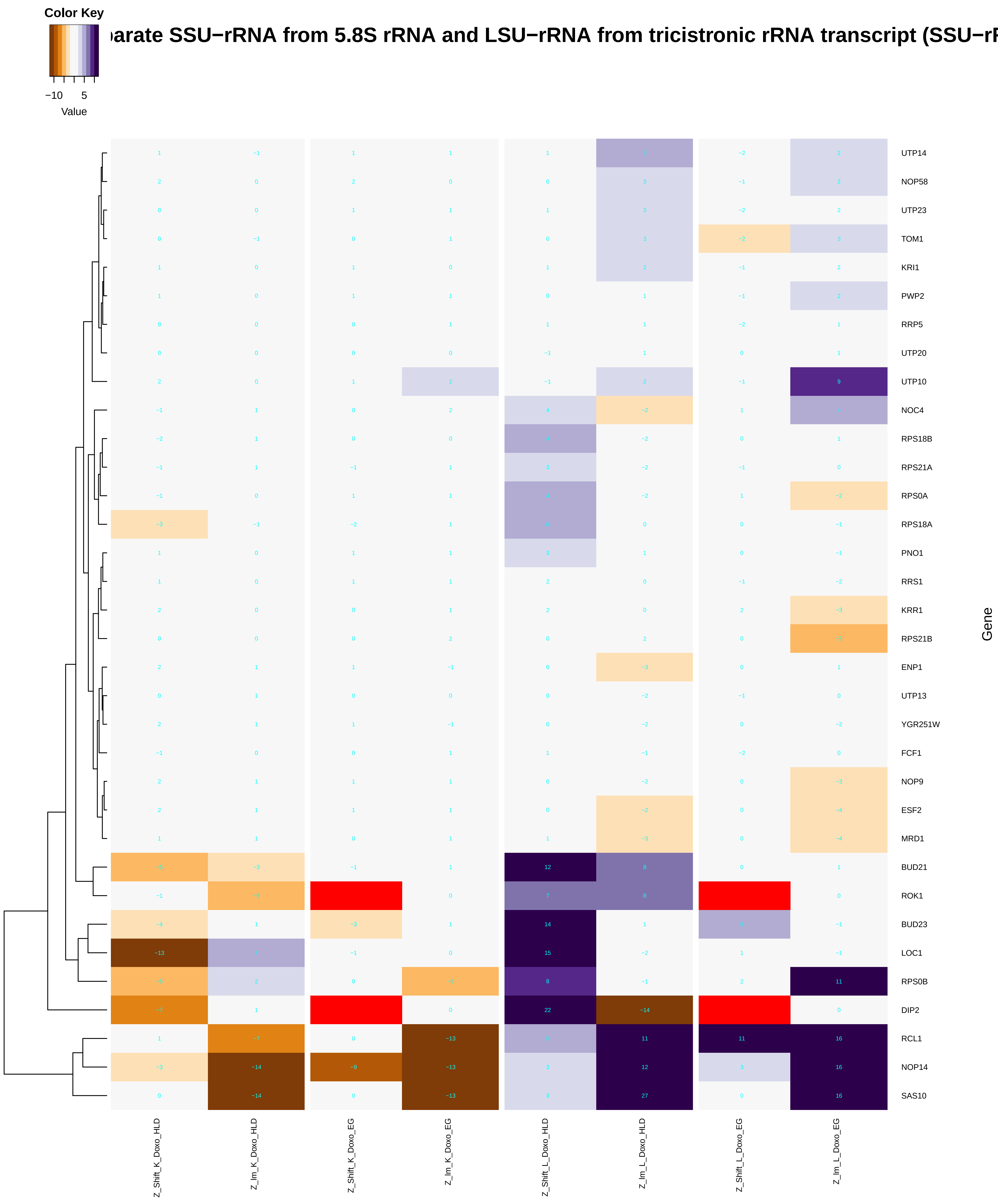
Color Key
−10
5
Value
parate SSU−rRNA from 5.8S rRNA and LSU−rRNA from tricistronic rRNA transcript (SSU−rR
UTP14
NOP58
UTP23
TOM1
KRI1
PWP2
RRP5
UTP20
UTP10
NOC4
RPS18B
RPS21A
RPS0A
RPS18A
PNO1
RRS1
KRR1
RPS21B
ENP1
UTP13
YGR251W
FCF1
NOP9
ESF2
MRD1
BUD21
ROK1
BUD23
LOC1
RPS0B
DIP2
RCL1
NOP14
SAS10
Gene
Z_Shift_K_Doxo_HLD
Z_lm_K_Doxo_HLD
Z_Shift_K_Doxo_EG
Z_lm_K_Doxo_EG
Z_Shift_L_Doxo_HLD
Z_lm_L_Doxo_HLD
Z_Shift_L_Doxo_EG
Z_lm_L_Doxo_EG

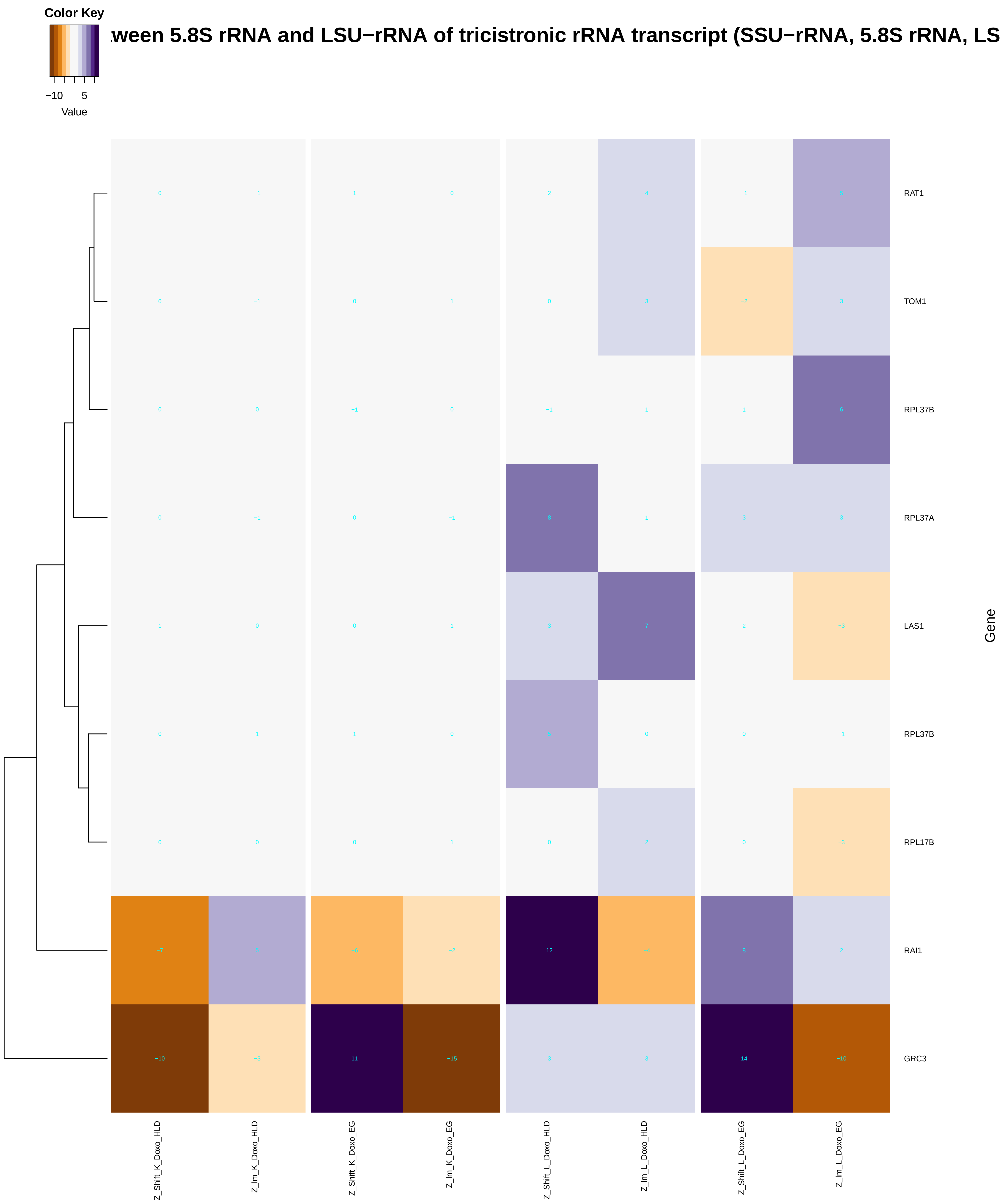

ween 5.8S rRNA and LSU−rRNA of tricistronic rRNA transcript (SSU−rRNA, 5.8S rRNA, LS
Color Key
−10
5
Value
RAT1
TOM1
RPL37B
RPL37A
LAS1
RPL37B
RPL17B
RAI1
GRC3
Gene
Z_Shift_K_Doxo_HLD
Z_Im_K_Doxo_HLD
Z_Shift_K_Doxo_EG
Z_Im_K_Doxo_EG
Z_Shift_L_Doxo_HLD
Z_Im_L_Doxo_HLD
Z_Shift_L_Doxo_EG
Z_Im_L_Doxo_EG

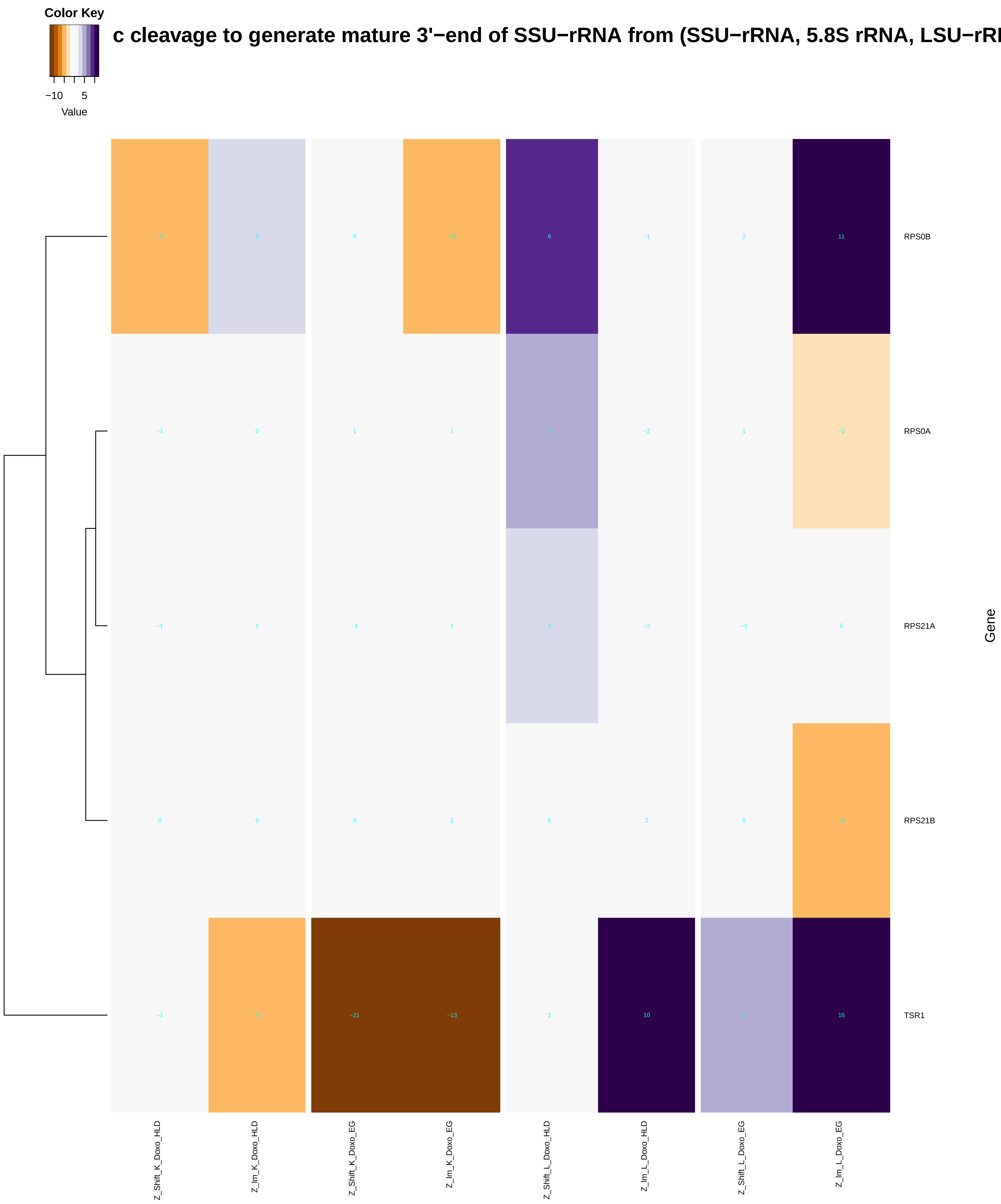

Color Key
−10
5
Value
c cleavage to generate mature 3'−end of SSU−rRNA from (SSU−rRNA, 5.8S rRNA, LSU−rR
RPS0B
RPS0A
RPS21A
RPS21B
TSR1
Gene
Z_Shift_K_Doxo_HLD
Z_lm_K_Doxo_HLD
Z_Shift_K_Doxo_EG
Z_lm_K_Doxo_EG
Z_Shift_L_Doxo_HLD
Z_lm_L_Doxo_HLD
Z_Shift_L_Doxo_EG
Z_lm_L_Doxo_EG

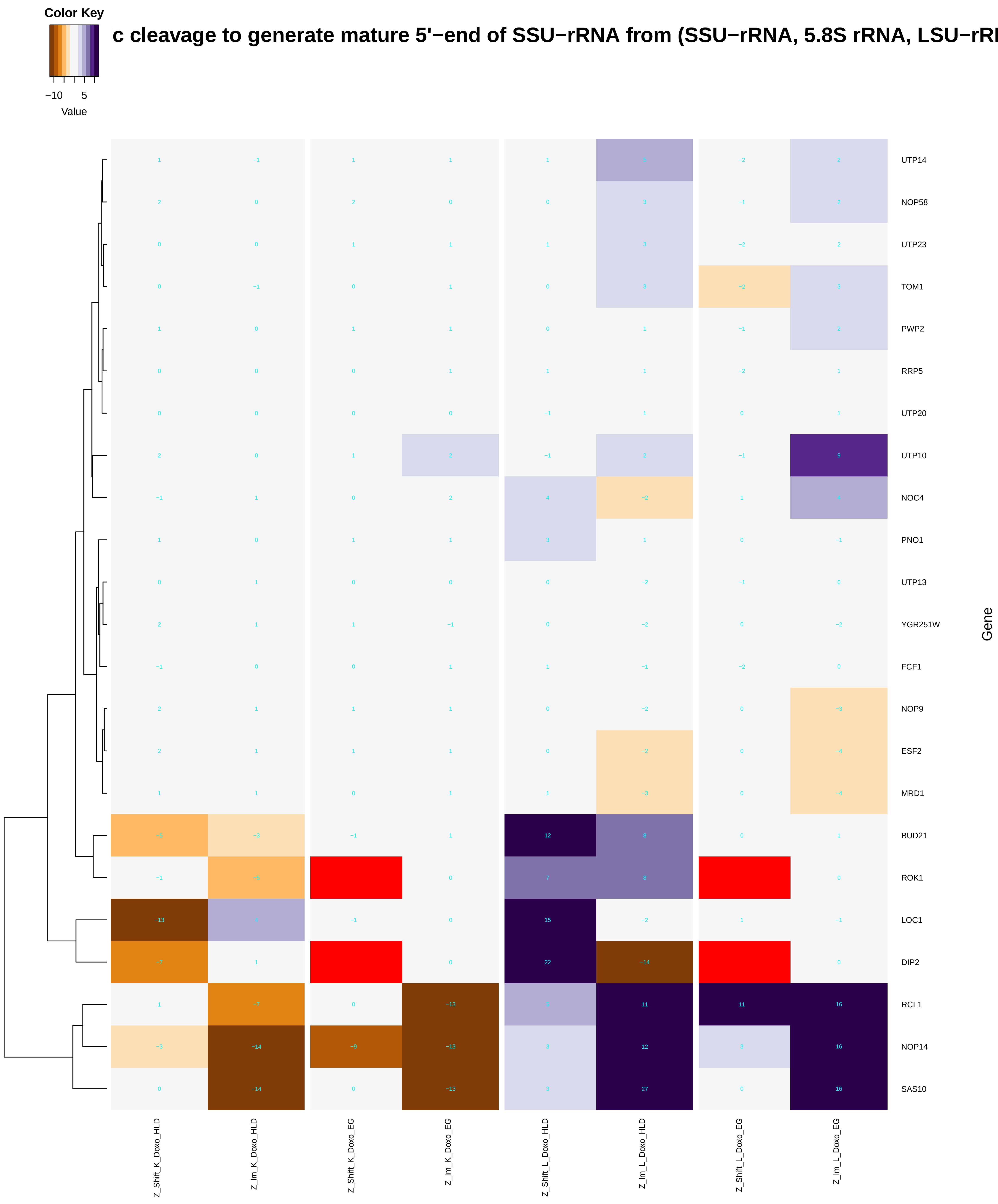

Color Key
−10
5
Value
c cleavage to generate mature 5'−end of SSU−rRNA from (SSU−rRNA, 5.8S rRNA, LSU−rRI
UTP14
NOP58
UTP23
TOM1
PWP2
RRP5
UTP20
UTP10
NOC4
PNO1
UTP13
YGR251W
FCF1
NOP9
ESF2
MRD1
BUD21
ROK1
LOC1
DIP2
RCL1
NOP14
SAS10
Gene
Z_Shift_K_Doxo_HLD
Z_lm_K_Doxo_HLD
Z_Shift_K_Doxo_EG
Z_lm_K_Doxo_EG
Z_Shift_L_Doxo_HLD
Z_lm_L_Doxo_HLD
Z_Shift_L_Doxo_EG
Z_lm_L_Doxo_EG

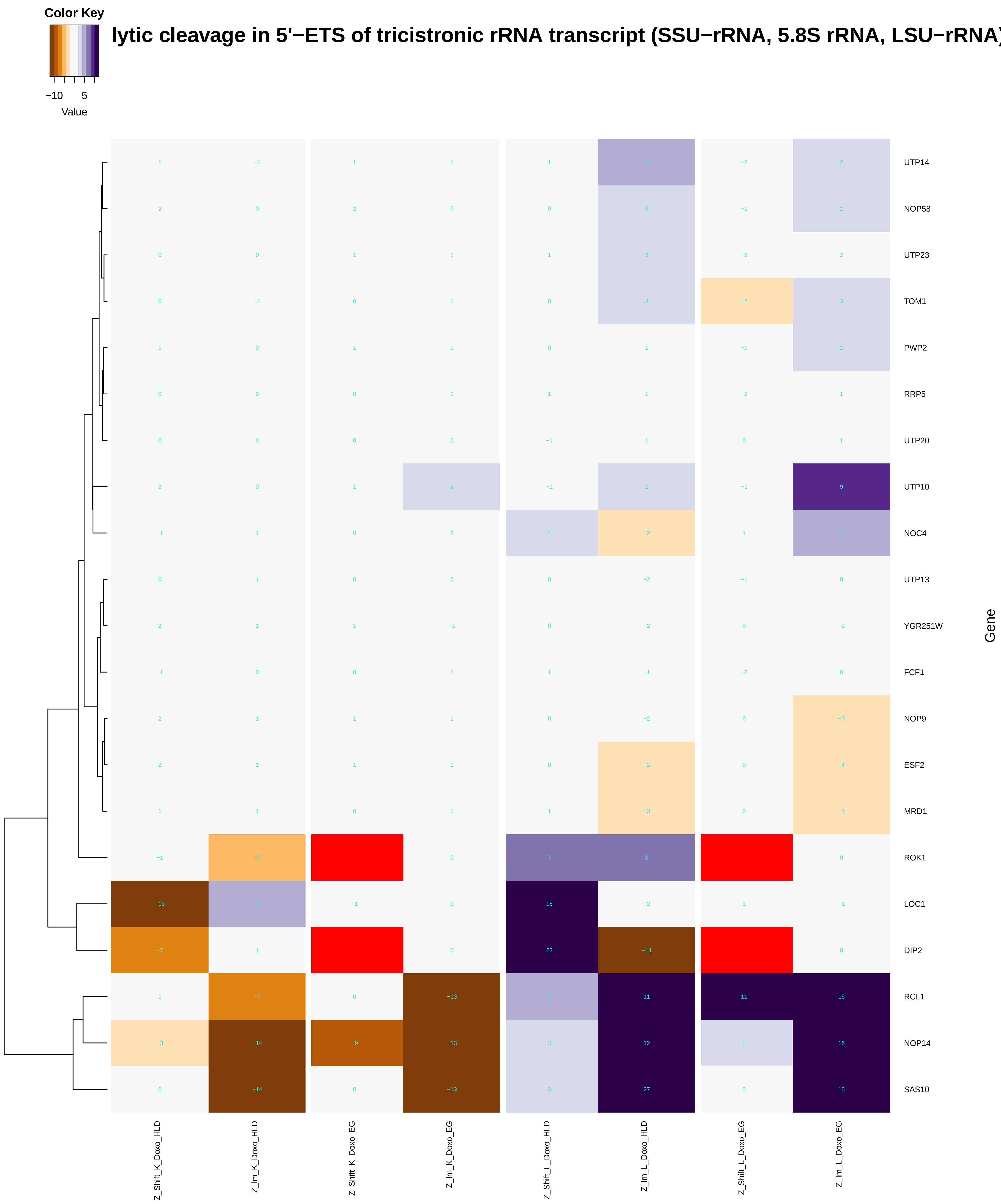
Color Key
−10
5
Value
lytic cleavage in 5'−ETS of tricistronic rRNA transcript (SSU−rRNA, 5.8S rRNA, LSU−rRNA
UTP14
NOP58
UTP23
TOM1
PWP2
RRP5
UTP20
UTP10
NOC4
UTP13
YGR251W
FCF1
NOP9
ESF2
MRD1
ROK1
LOC1
DIP2
RCL1
NOP14
SAS10
Gene
Z_Shift_K_Doxo_HLD
Z_lm_K_Doxo_HLD
Z_Shift_K_Doxo_EG
Z_lm_K_Doxo_EG
Z_Shift_L_Doxo_HLD
Z_lm_L_Doxo_HLD
Z_Shift_L_Doxo_EG
Z_lm_L_Doxo_EG
