# Supplementary material for: A yeast phenomic model for the influence of Warburg metabolism on genetic buffering of doxorubicin
Source: Cancer Metab. 2019 Oct 23;7:9. doi: 10.1186/s40170-019-0201-3 (PMC6806529; doi:10.1186/s40170-019-0201-3)
Supplement: Supplementary file 9 — Additional file 9. GO term-specific heatmaps for REMc/GTF-enriched clusters. GO term-specific heatmaps for significant GO process terms were generated as described in methods and Figs. 3 and 4. Any related child terms are presented in subsequent pages of the parent file name. GO terms with more than 100 children, with 2 or fewer genes annotated to the term, or a file size over 300KB are not shown. All heatmaps are generated with the same layout (see Figs. 3 and 4). [file 40170_2019_201_MOESM9_ESM.bz2 › Additional_File9_GOTermHeatmaps/Additional_File9_GOTermHeatmap/cytochrome_complex_assembly.pdf]

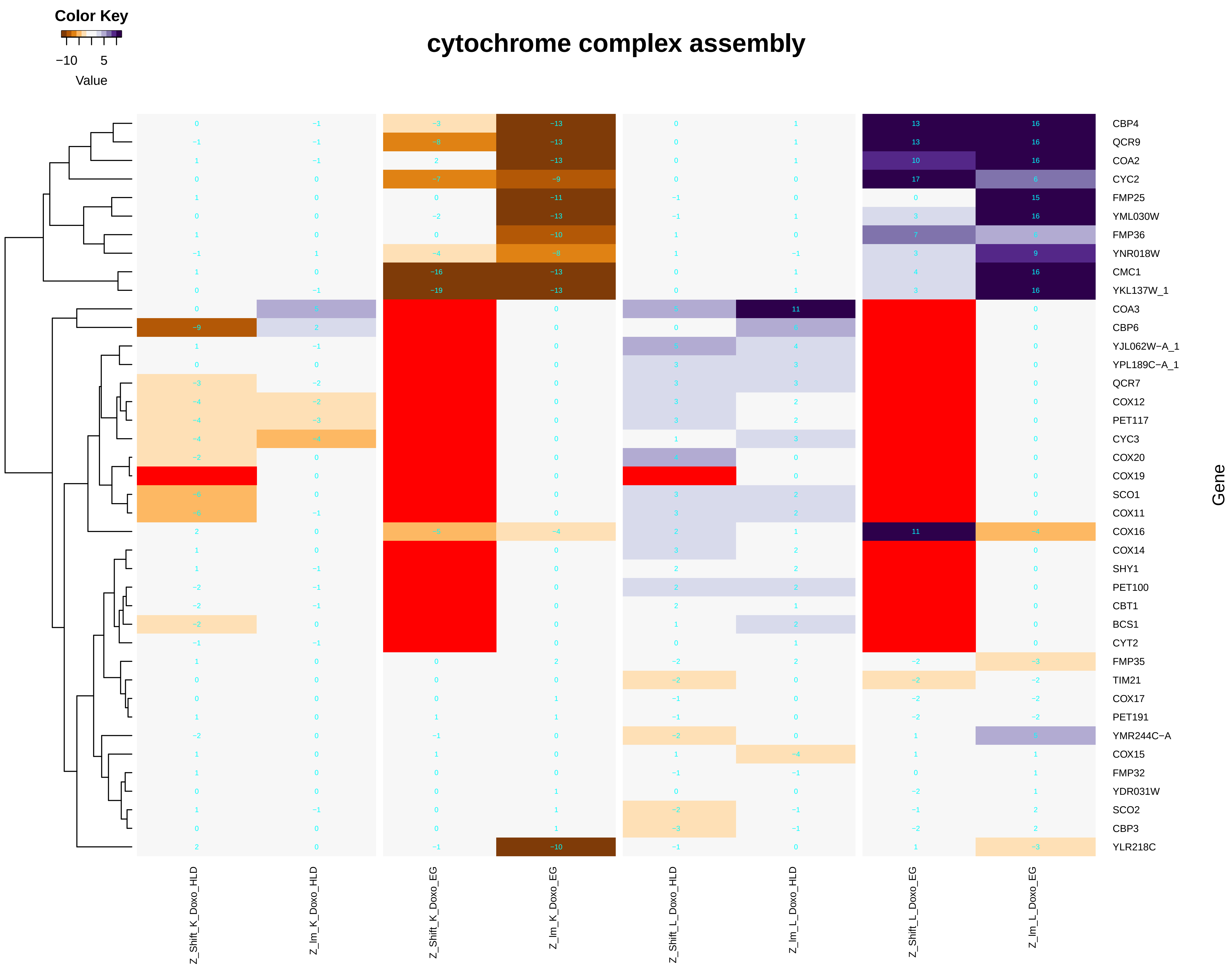

Color Key
−10
5
Value
cytochrome complex assembly
CBP4
QCR9
COA2
CYC2
FMP25
YML030W
FMP36
YNR018W
CMC1
YKL137W_1
COA3
CBP6
YJL062W−A_1
YPL189C−A_1
QCR7
COX12
PET117
CYC3
COX20
COX19
SCO1
COX11
COX16
COX14
SHY1
PET100
CBT1
BCS1
CYT2
FMP35
TIM21
COX17
PET191
YMR244C−A
COX15
FMP32
YDR031W
SCO2
CBP3
YLR218C
Gene
Z_Shift_K_Doxo_HLD
Z_Im_K_Doxo_HLD
Z_Shift_K_Doxo_EG
Z_Im_K_Doxo_EG
Z_Shift_L_Doxo_HLD
Z_Im_L_Doxo_HLD
Z_Shift_L_Doxo_EG
Z_Im_L_Doxo_EG

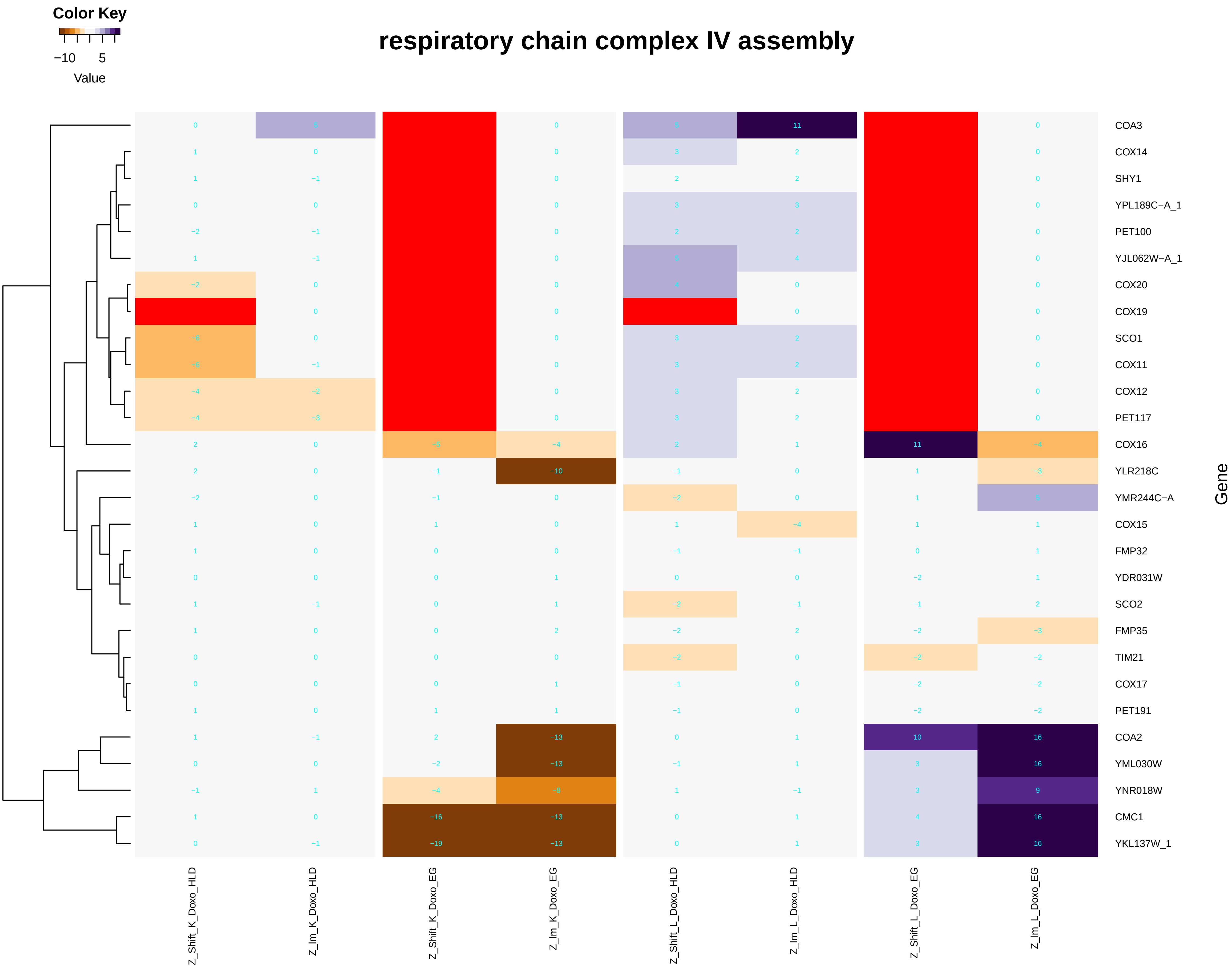
Color Key
−10 5
Value
respiratory chain complex IV assembly
COA3
COX14
SHY1
YPL189C−A_1
PET100
YJL062W−A_1
COX20
COX19
SCO1
COX11
COX12
PET117
COX16
YLR218C
YMR244C−A
COX15
FMP32
YDR031W
SCO2
FMP35
TIM21
COX17
PET191
COA2
YML030W
YNR018W
CMC1
YKL137W_1
Gene
Z_Shift_K_Doxo_HLD
Z_Im_K_Doxo_HLD
Z_Shift_K_Doxo_EG
Z_Im_K_Doxo_EG
Z_Shift_L_Doxo_HLD
Z_Im_L_Doxo_HLD
Z_Shift_L_Doxo_EG
Z_Im_L_Doxo_EG

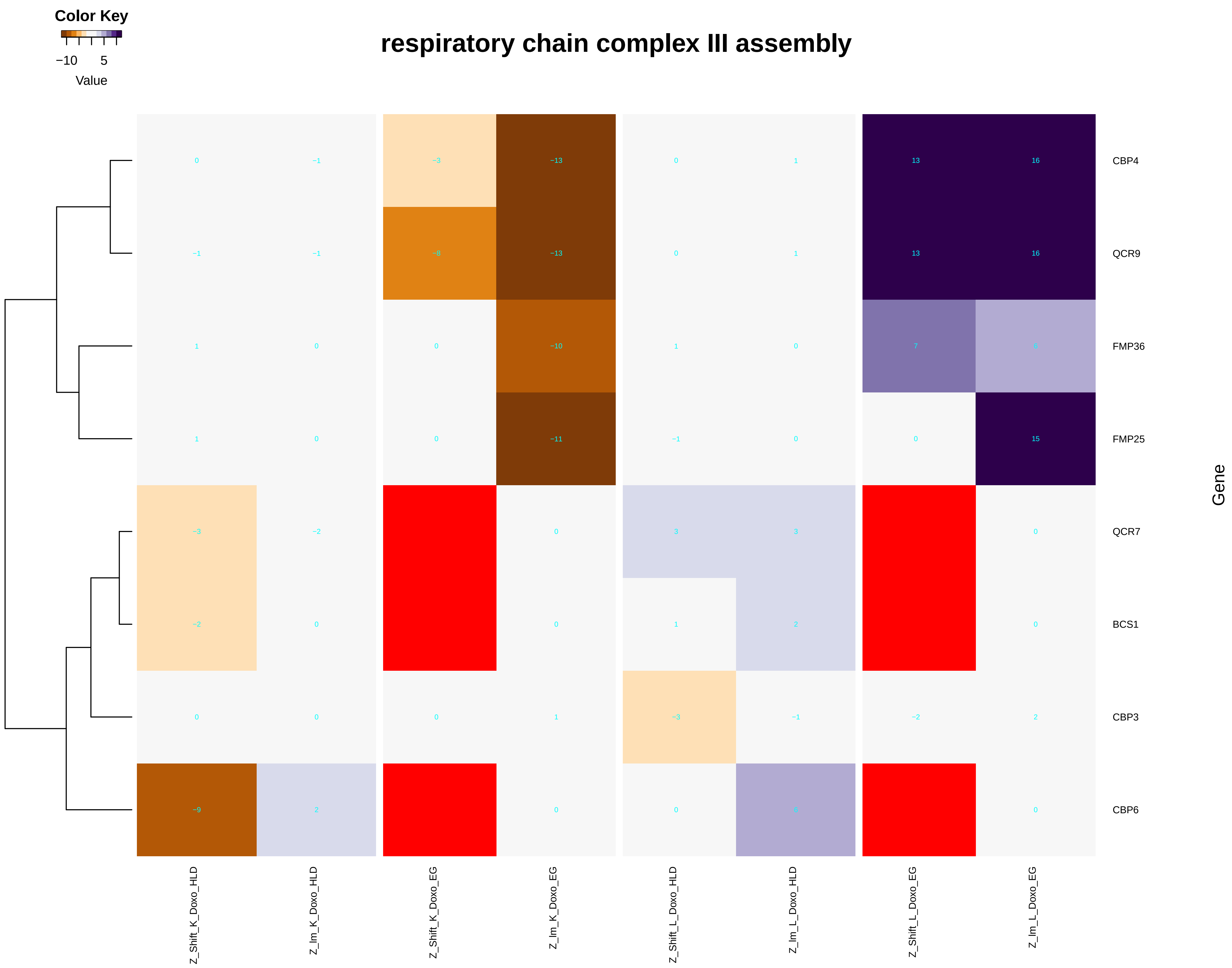

Color Key
−10
5
Value
respiratory chain complex III assembly
CBP4
QCR9
FMP36
FMP25
QCR7
BCS1
CBP3
CBP6
Gene
Z_Shift_K_Doxo_HLD
Z_Im_K_Doxo_HLD
Z_Shift_K_Doxo_EG
Z_Im_K_Doxo_EG
Z_Shift_L_Doxo_HLD
Z_Im_L_Doxo_HLD
Z_Shift_L_Doxo_EG
Z_Im_L_Doxo_EG
0 −1 −3 −13 0 1 13 16
−1 −1 −8 −13 0 1 13 16
1 0 0 −10 1 0 7
1 0 0 −11 −1 0 0 15
−3 −2 0 3 3 0
−2 0 0 1 2 0
0 0 0 1 −3 −1 −2 2
−9 2 0 0 0

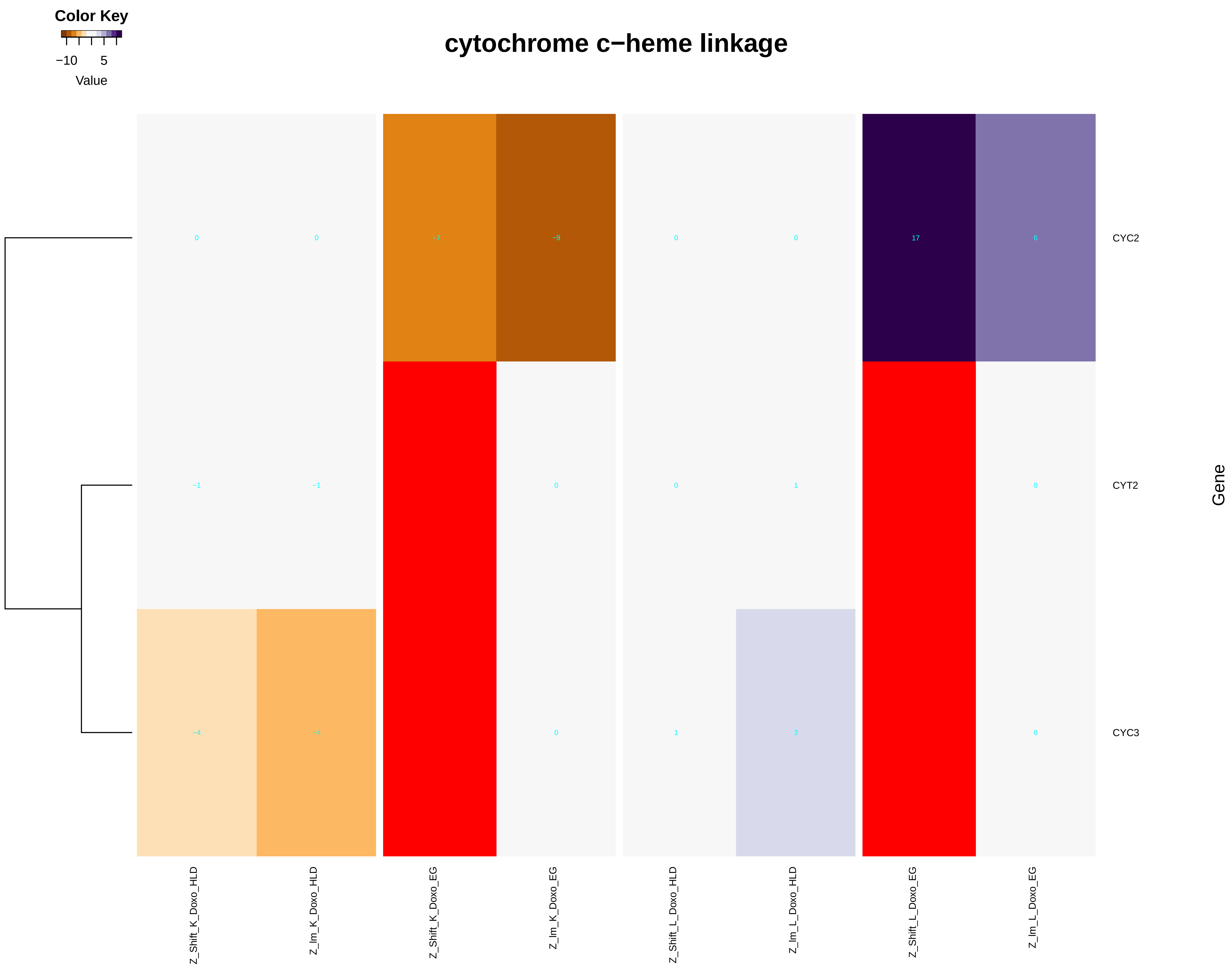
Color Key
−10
5
Value
cytochrome c−heme linkage
0
0
−7
−9
0
0
17
6
CYC2
−1
−1
0
0
1
0
CYT2
−4
−4
0
1
3
0
CYC3
Gene
Z_Shift_K_Doxo_HLD
Z_Im_K_Doxo_HLD
Z_Shift_K_Doxo_EG
Z_Im_K_Doxo_EG
Z_Shift_L_Doxo_HLD
Z_Im_L_Doxo_HLD
Z_Shift_L_Doxo_EG
Z_Im_L_Doxo_EG

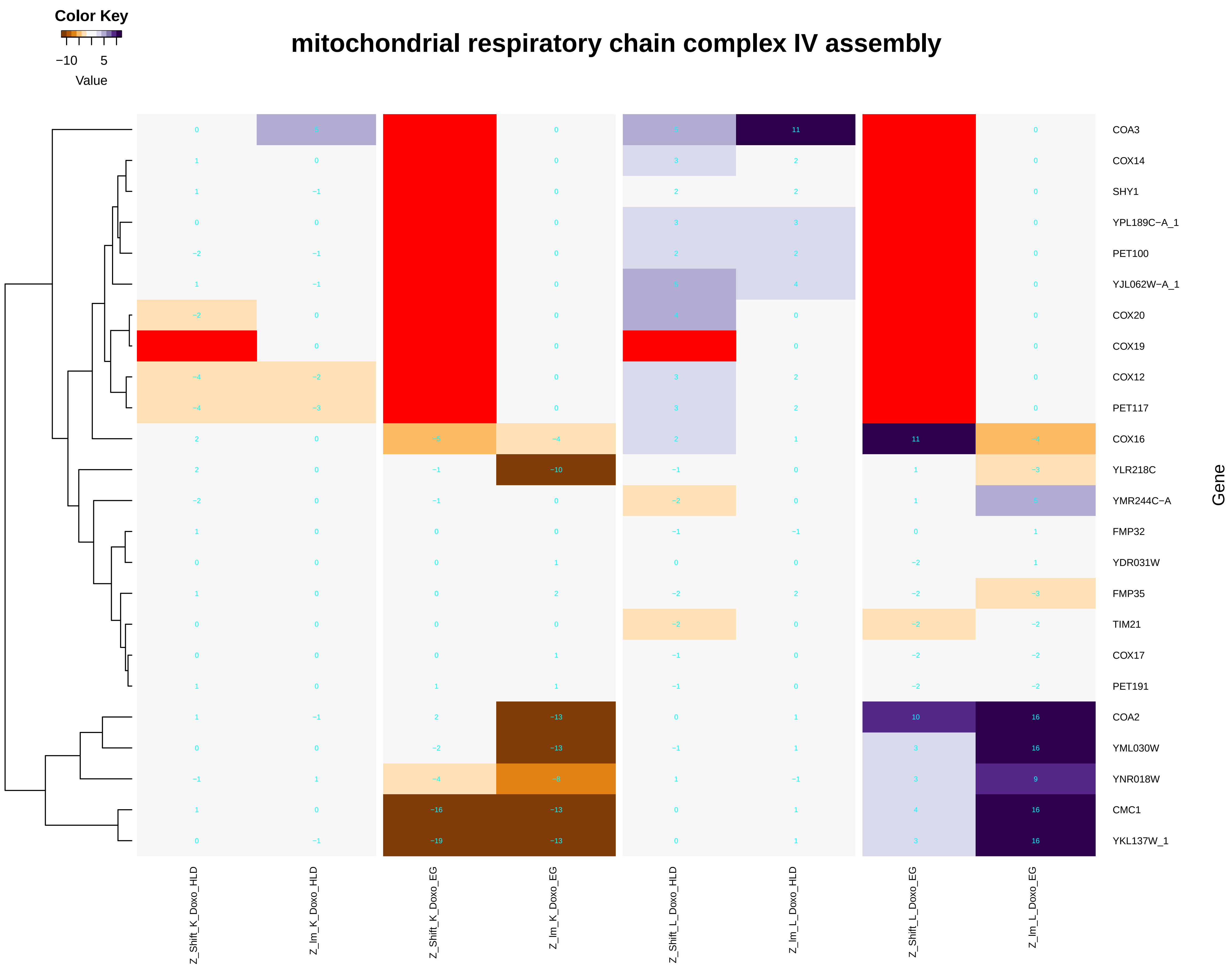

Color Key
−10
5
Value
mitochondrial respiratory chain complex IV assembly
COA3
COX14
SHY1
YPL189C−A_1
PET100
YJL062W−A_1
COX20
COX19
COX12
PET117
COX16
YLR218C
YMR244C−A
FMP32
YDR031W
FMP35
TIM21
COX17
PET191
COA2
YML030W
YNR018W
CMC1
YKL137W_1
Gene
Z_Shift_K_Doxo_HLD
Z_Im_K_Doxo_HLD
Z_Shift_K_Doxo_EG
Z_Im_K_Doxo_EG
Z_Shift_L_Doxo_HLD
Z_Im_L_Doxo_HLD
Z_Shift_L_Doxo_EG
Z_Im_L_Doxo_EG

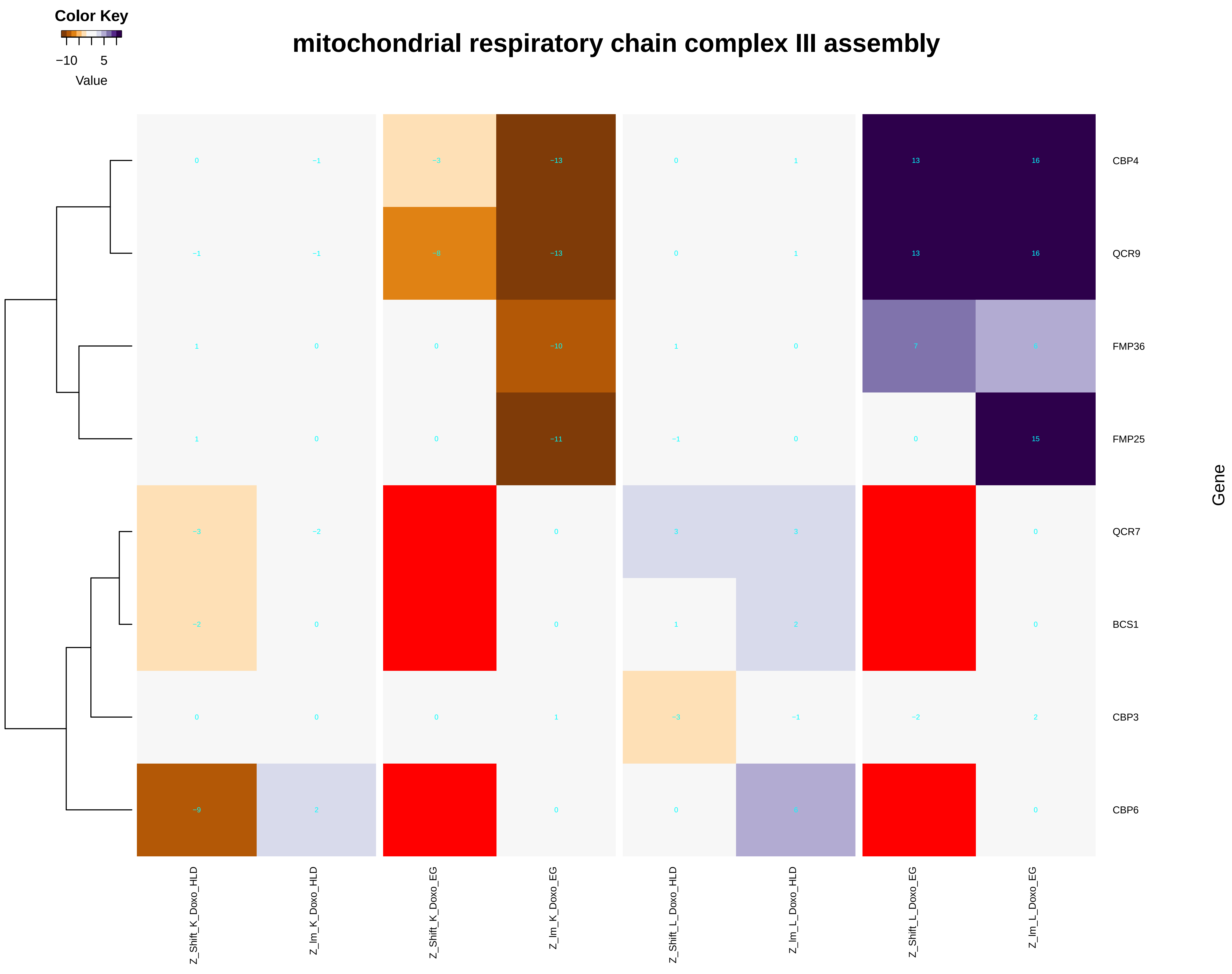
Color Key
−10
5
Value
mitochondrial respiratory chain complex III assembly
CBP4
QCR9
FMP36
FMP25
QCR7
BCS1
CBP3
CBP6
Gene
Z_Shift_K_Doxo_HLD
Z_Im_K_Doxo_HLD
Z_Shift_K_Doxo_EG
Z_Im_K_Doxo_EG
Z_Shift_L_Doxo_HLD
Z_Im_L_Doxo_HLD
Z_Shift_L_Doxo_EG
Z_Im_L_Doxo_EG
