# Supplementary material for: A yeast phenomic model for the influence of Warburg metabolism on genetic buffering of doxorubicin
Source: Cancer Metab. 2019 Oct 23;7:9. doi: 10.1186/s40170-019-0201-3 (PMC6806529; doi:10.1186/s40170-019-0201-3)

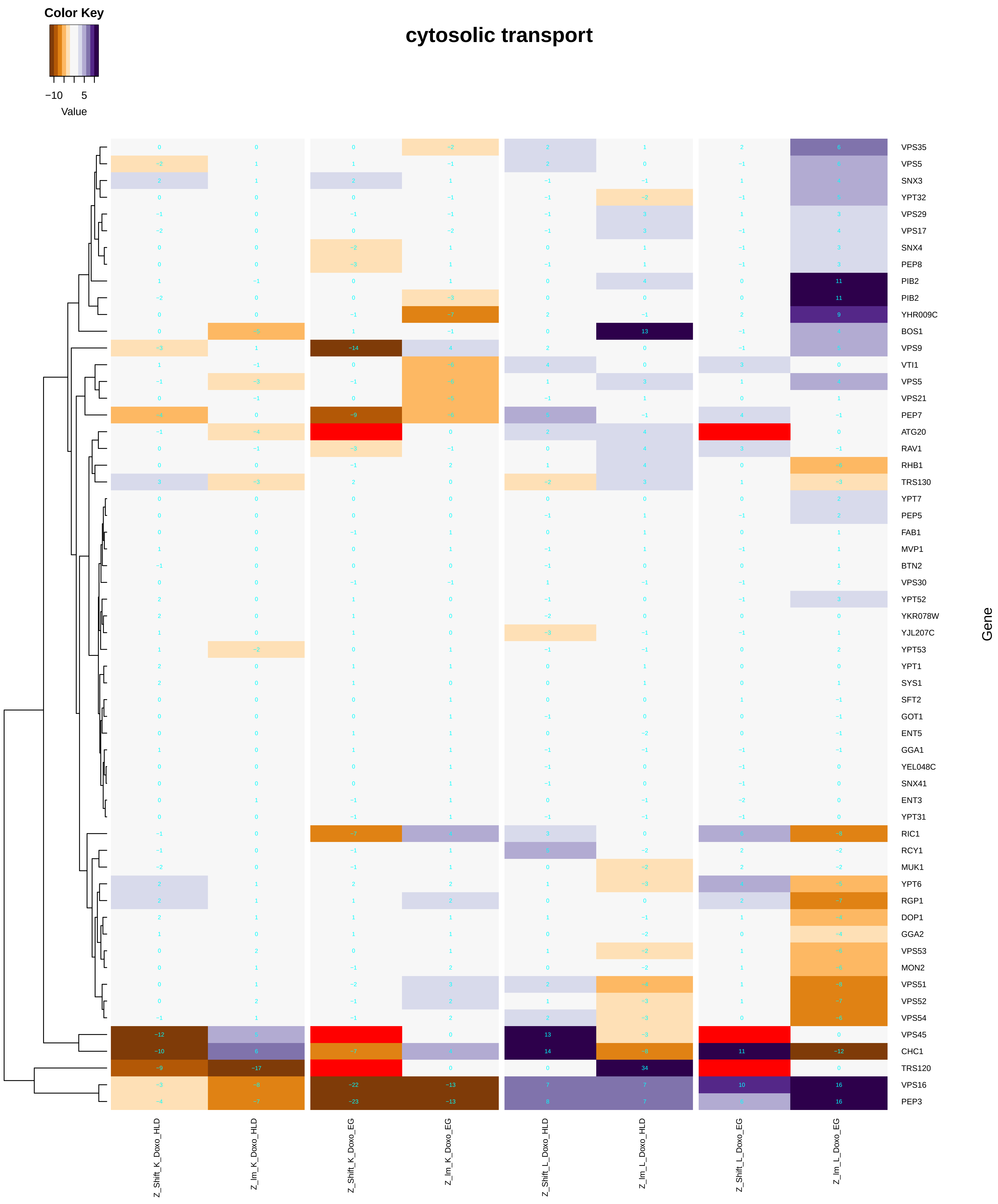
Color Key
−10
5
Value
cytosolic transport
VPS35
VPS5
SNX3
YPT32
VPS29
VPS17
SNX4
PEP8
PIB2
PIB2
YHR009C
BOS1
VPS9
VTI1
VPS5
VPS21
PEP7
ATG20
RAV1
RHB1
TRS130
YPT7
PEP5
FAB1
MVP1
BTN2
VPS30
YPT52
YKR078W
YJL207C
YPT53
YPT1
SYS1
SFT2
GOT1
ENT5
GGA1
YEL048C
SNX41
ENT3
YPT31
RIC1
RCY1
MUK1
YPT6
RGP1
DOP1
GGA2
VPS53
MON2
VPS51
VPS52
VPS54
VPS45
CHC1
TRS120
VPS16
PEP3
Gene
Z_Shift_K_Doxo_HLD
Z_Im_K_Doxo_HLD
Z_Shift_K_Doxo_EG
Z_Im_K_Doxo_EG
Z_Shift_L_Doxo_HLD
Z_Im_L_Doxo_HLD
Z_Shift_L_Doxo_EG
Z_Im_L_Doxo_EG

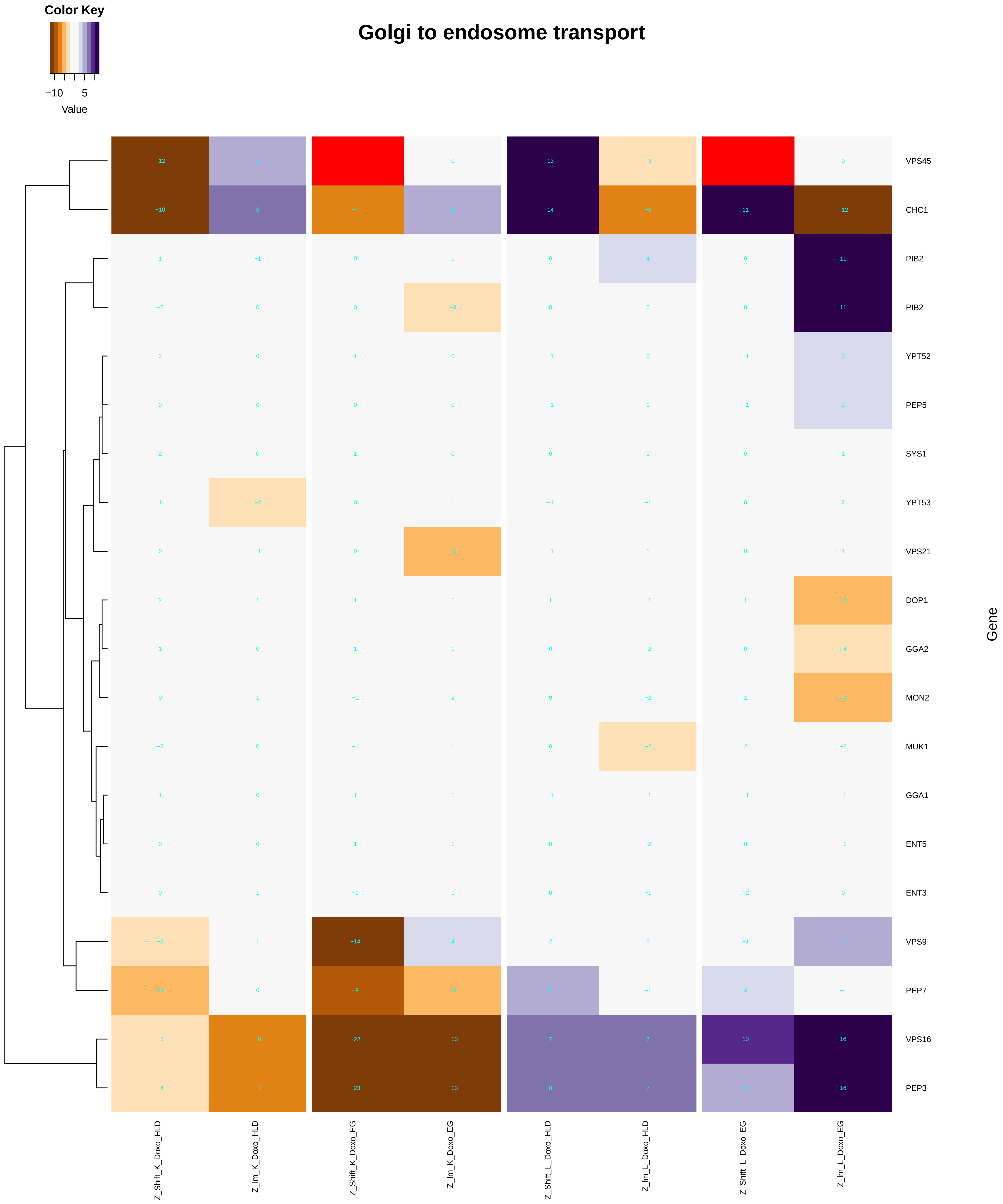
Color Key
−10
5
Value
Golgi to endosome transport
VPS45
CHC1
PIB2
PIB2
YPT52
PEP5
SYS1
YPT53
VPS21
DOP1
GGA2
MON2
MUK1
GGA1
ENT5
ENT3
VPS9
PEP7
VPS16
PEP3
Gene
Z_Shift_K_Doxo_HLD
Z_Im_K_Doxo_HLD
Z_Shift_K_Doxo_EG
Z_Im_K_Doxo_EG
Z_Shift_L_Doxo_HLD
Z_Im_L_Doxo_HLD
Z_Shift_L_Doxo_EG
Z_Im_L_Doxo_EG

retrograde transport, endosome to Golgi

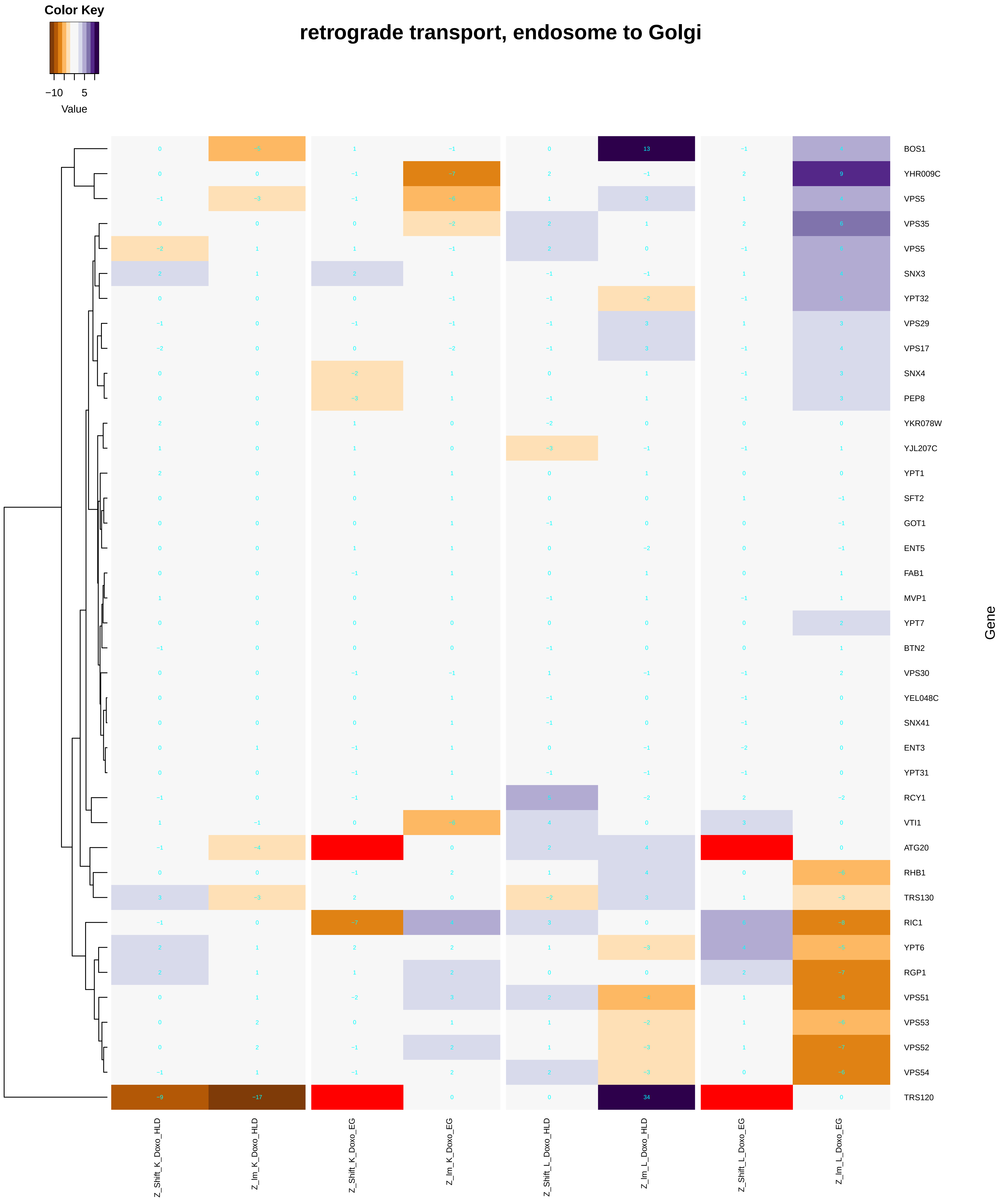

early endosome to Golgi transport

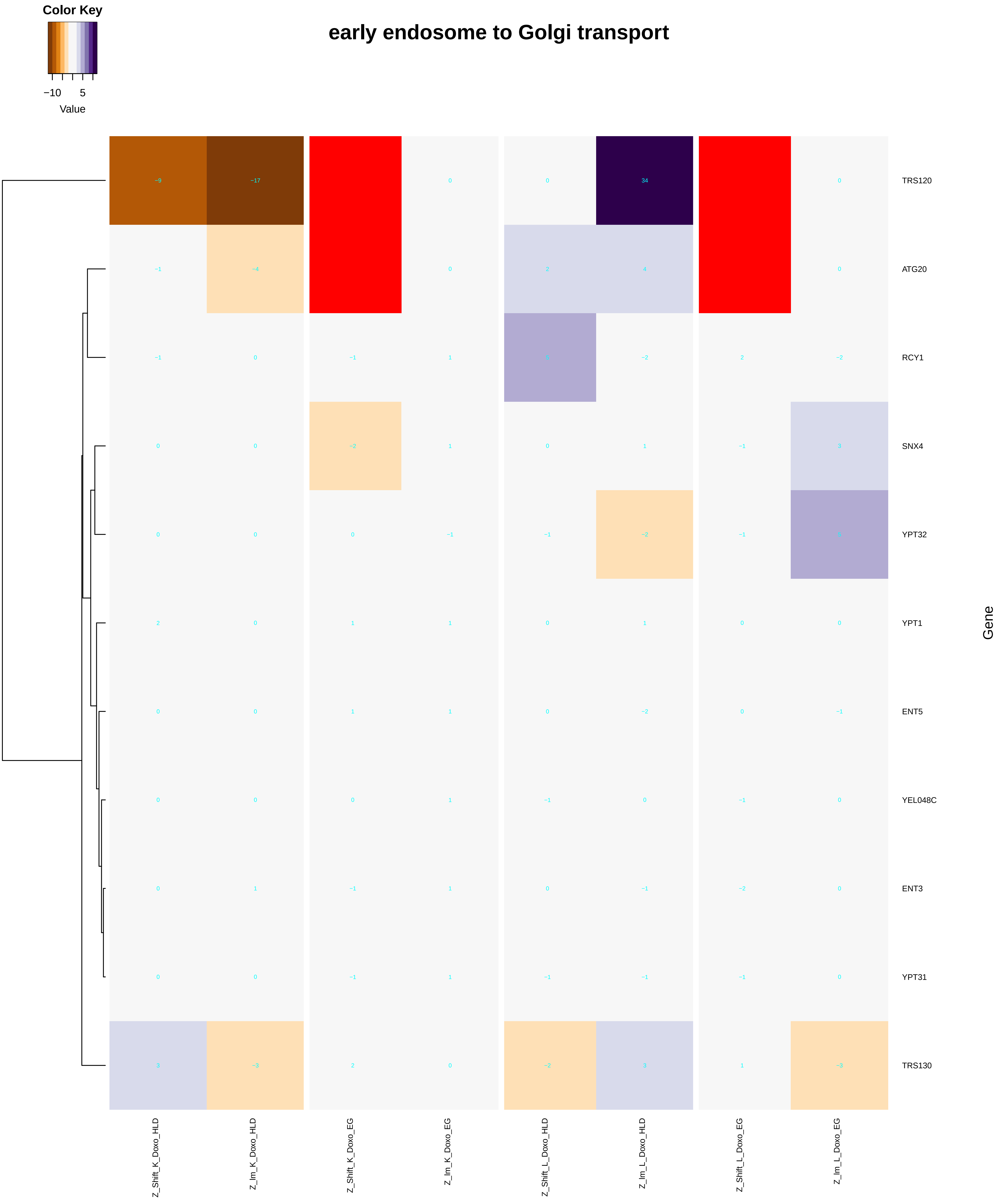

Supplement: Supplementary file 9 — Additional file 9. GO term-specific heatmaps for REMc/GTF-enriched clusters. GO term-specific heatmaps for significant GO process terms were generated as described in methods and Figs. 3 and 4. Any related child terms are presented in subsequent pages of the parent file name. GO terms with more than 100 children, with 2 or fewer genes annotated to the term, or a file size over 300KB are not shown. All heatmaps are generated with the same layout (see Figs. 3 and 4). [file 40170_2019_201_MOESM9_ESM.bz2 › Additional_File9_GOTermHeatmaps/Additional_File9_GOTermHeatmap/cytosolic_transport.pdf]
