# Supplementary material for: A yeast phenomic model for the influence of Warburg metabolism on genetic buffering of doxorubicin
Source: Cancer Metab. 2019 Oct 23;7:9. doi: 10.1186/s40170-019-0201-3 (PMC6806529; doi:10.1186/s40170-019-0201-3)
Supplement: Supplementary file 9 — Additional file 9. GO term-specific heatmaps for REMc/GTF-enriched clusters. GO term-specific heatmaps for significant GO process terms were generated as described in methods and Figs. 3 and 4. Any related child terms are presented in subsequent pages of the parent file name. GO terms with more than 100 children, with 2 or fewer genes annotated to the term, or a file size over 300KB are not shown. All heatmaps are generated with the same layout (see Figs. 3 and 4). [file 40170_2019_201_MOESM9_ESM.bz2 › Additional_File9_GOTermHeatmaps/Additional_File9_GOTermHeatmap/DNA_repair.pdf]

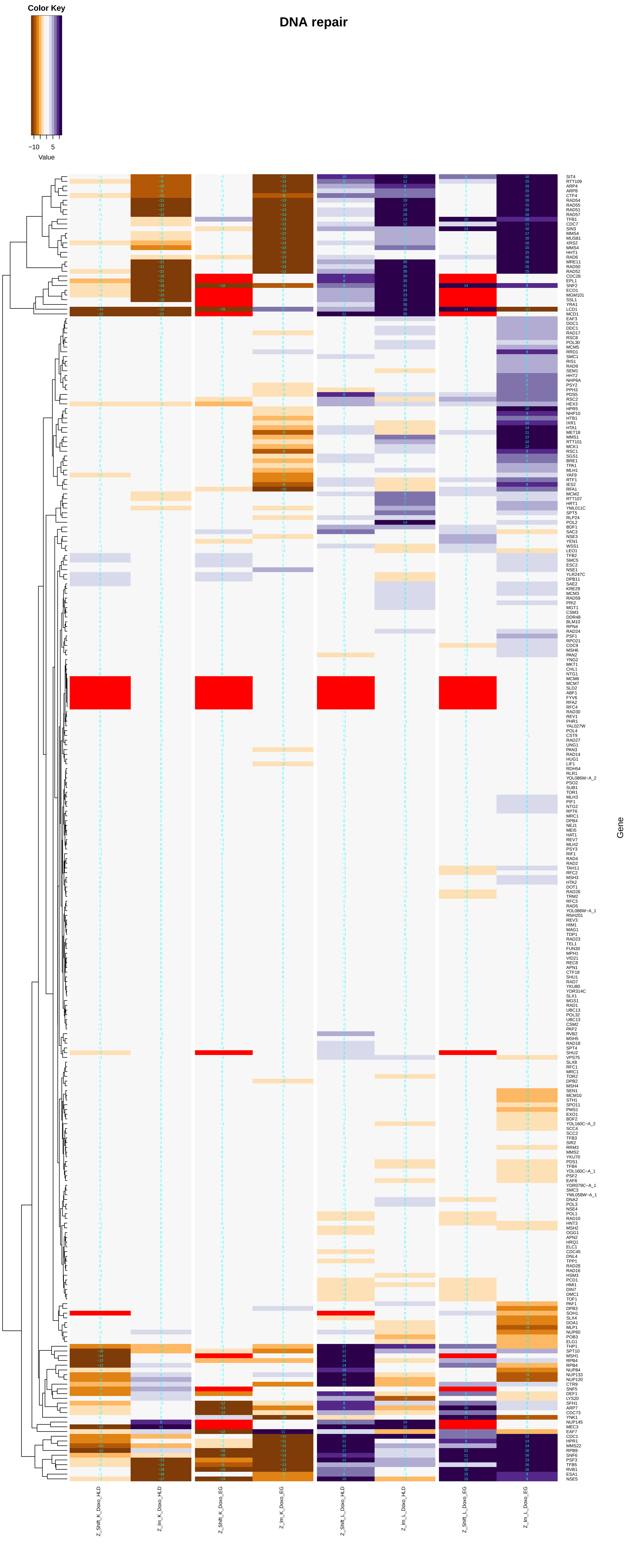

Color Key
−10
5
Value
DNA repair
Gene
Z_Shift_K_Doxo_HLD
Z_Im_K_Doxo_HLD
Z_Shift_K_Doxo_EG
Z_Im_K_Doxo_EG
Z_Shift_L_Doxo_HLD
Z_Im_L_Doxo_HLD
Z_Shift_L_Doxo_EG
Z_Im_L_Doxo_EG

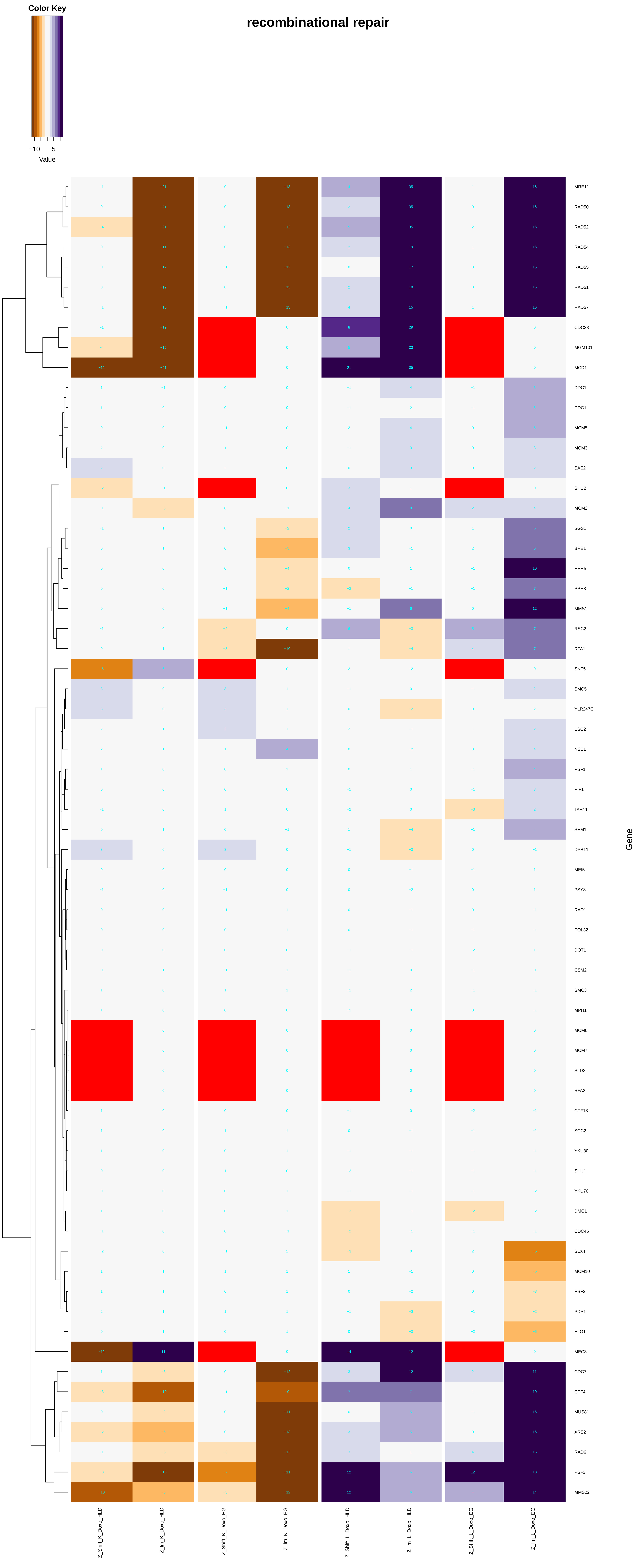

Color Key
−10
5
Value
recombinational repair
MRE11
RAD50
RAD52
RAD54
RAD55
RAD51
RAD57
CDC28
MGM101
MCD1
DDC1
DDC1
MCM5
MCM3
SAE2
SHU2
MCM2
SGS1
BRE1
HPR5
PPH3
MMS1
RSC2
RFA1
SNF5
SMC5
YLR247C
ESC2
NSE1
PSF1
PIF1
TAH11
SEM1
DPB11
MEI5
PSY3
RAD1
POL32
DOT1
CSM2
SMC3
MPH1
MCM6
MCM7
SLD2
RFA2
CTF18
SCC2
YKU80
SHU1
YKU70
DMC1
CDC45
SLX4
MCM10
PSF2
PDS1
ELG1
MEC3
CDC7
CTF4
MUS81
XRS2
RAD6
PSF3
MMS22
Gene
Z_Shift_K_Doxo_HLD
Z_Im_K_Doxo_HLD
Z_Shift_K_Doxo_EG
Z_Im_K_Doxo_EG
Z_Shift_L_Doxo_HLD
Z_Im_L_Doxo_HLD
Z_Shift_L_Doxo_EG
Z_Im_L_Doxo_EG

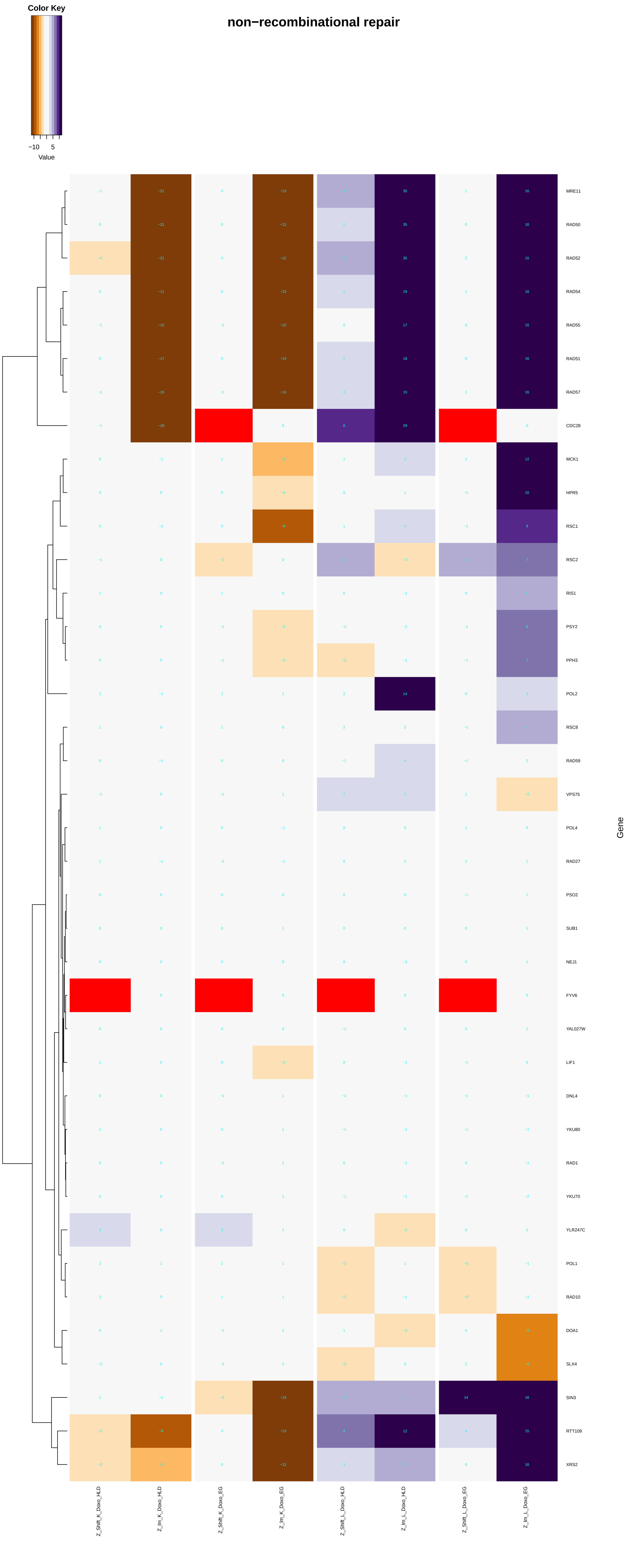
Color Key
−10
5
Value
non−recombinational repair
MRE11
RAD50
RAD52
RAD54
RAD55
RAD51
RAD57
CDC28
MCK1
HPR5
RSC1
RSC2
RIS1
PSY2
PPH3
POL2
RSC8
RAD59
VPS75
POL4
RAD27
PSO2
SUB1
NEJ1
FYV6
YAL027W
LIF1
DNL4
YKU80
RAD1
YKU70
YLR247C
POL1
RAD10
DOA1
SLX4
SIN3
RTT109
XRS2
Gene
Z_Shift_K_Doxo_HLD
Z_Im_K_Doxo_HLD
Z_Shift_K_Doxo_EG
Z_Im_K_Doxo_EG
Z_Shift_L_Doxo_HLD
Z_Im_L_Doxo_HLD
Z_Shift_L_Doxo_EG
Z_Im_L_Doxo_EG

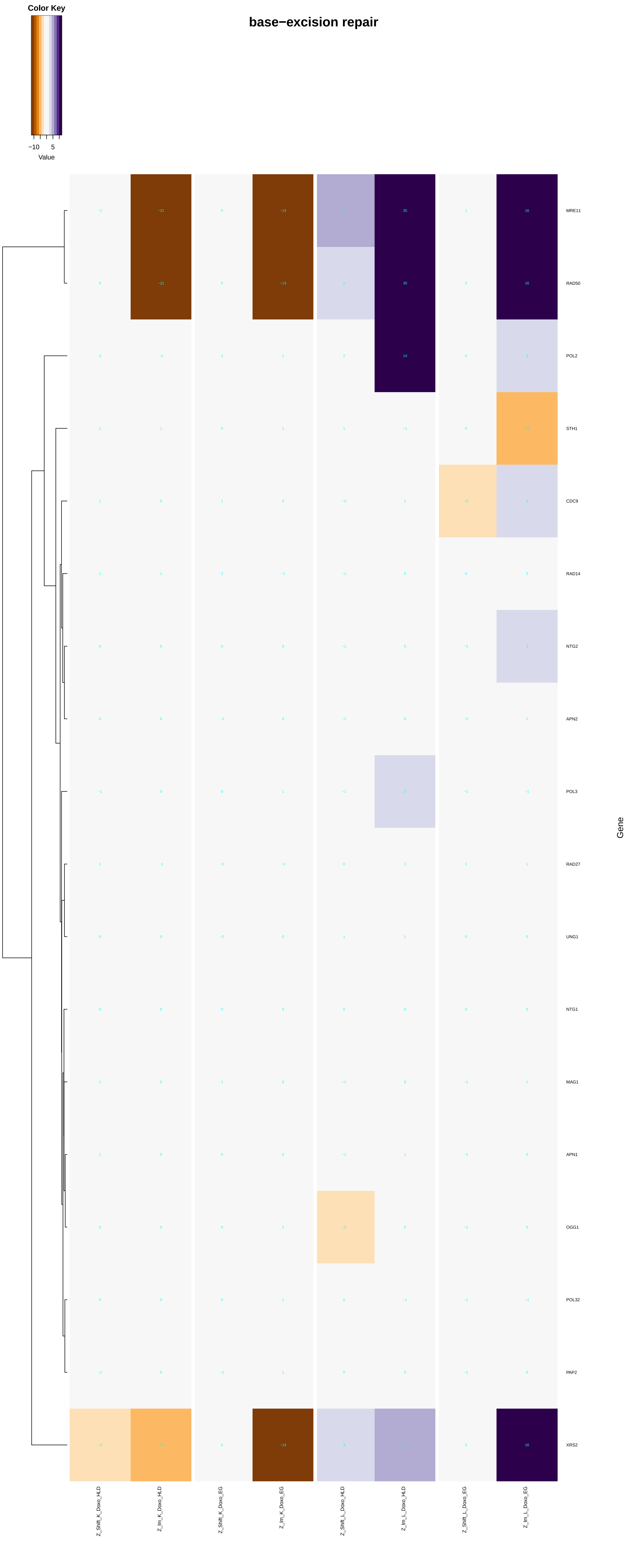

Color Key
−10 5
Value
base−excision repair
MRE11
RAD50
POL2
STH1
CDC9
RAD14
NTG2
APN2
POL3
RAD27
UNG1
NTG1
MAG1
APN1
OGG1
POL32
PAP2
XRS2
Gene
Z_Shift_K_Doxo_HLD
Z_Im_K_Doxo_HLD
Z_Shift_K_Doxo_EG
Z_Im_K_Doxo_EG
Z_Shift_L_Doxo_HLD
Z_Im_L_Doxo_HLD
Z_Shift_L_Doxo_EG
Z_Im_L_Doxo_EG

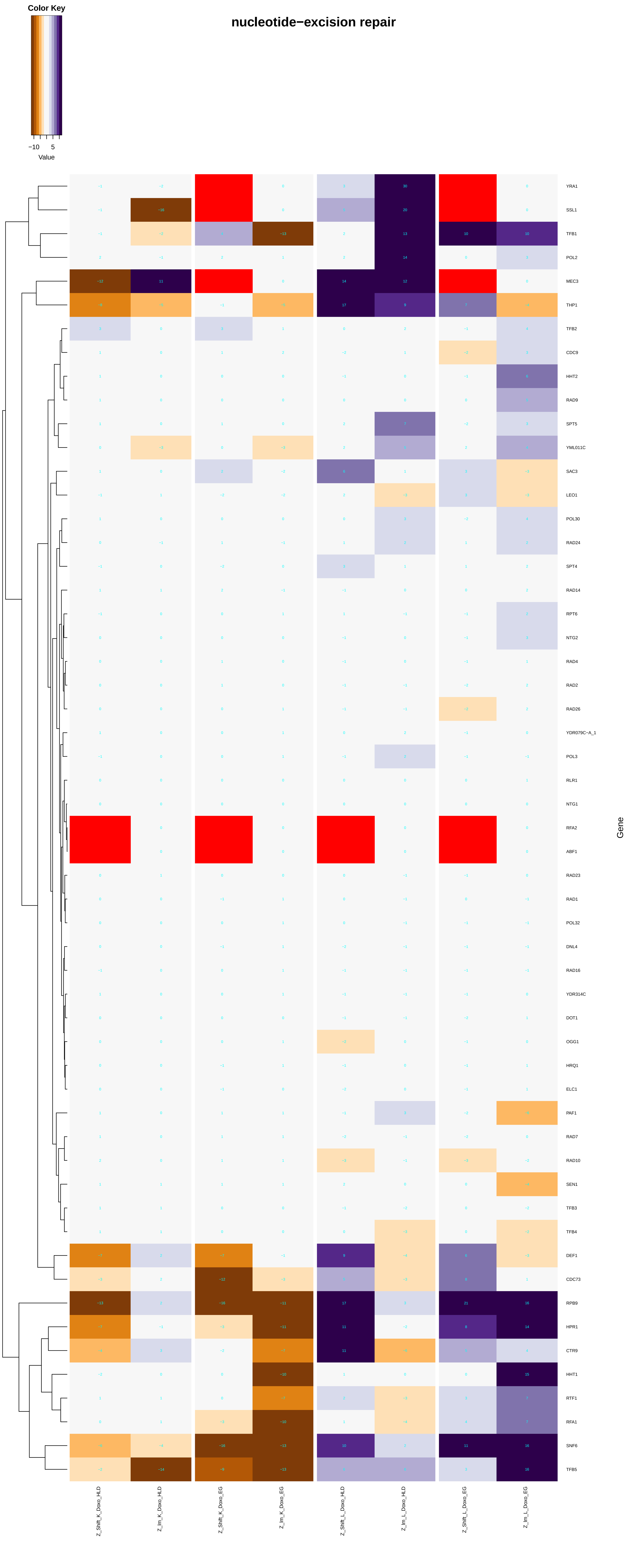
Color Key
−10 5
Value
nucleotide−excision repair
YRA1
SSL1
TFB1
POL2
MEC3
THP1
TFB2
CDC9
HHT2
RAD9
SPT5
YML011C
SAC3
LEO1
POL30
RAD24
SPT4
RAD14
RPT6
NTG2
RAD4
RAD2
RAD26
YDR079C−A_1
POL3
RLR1
NTG1
RFA2
ABF1
RAD23
RAD1
POL32
DNL4
RAD16
YDR314C
DOT1
OGG1
HRQ1
ELC1
PAF1
RAD7
RAD10
SEN1
TFB3
TFB4
DEF1
CDC73
RPB9
HPR1
CTR9
HHT1
RTF1
RFA1
SNF6
TFB5
Gene
Z_Shift_K_Doxo_HLD
Z_Im_K_Doxo_HLD
Z_Shift_K_Doxo_EG
Z_Im_K_Doxo_EG
Z_Shift_L_Doxo_HLD
Z_Im_L_Doxo_HLD
Z_Shift_L_Doxo_EG
Z_Im_L_Doxo_EG

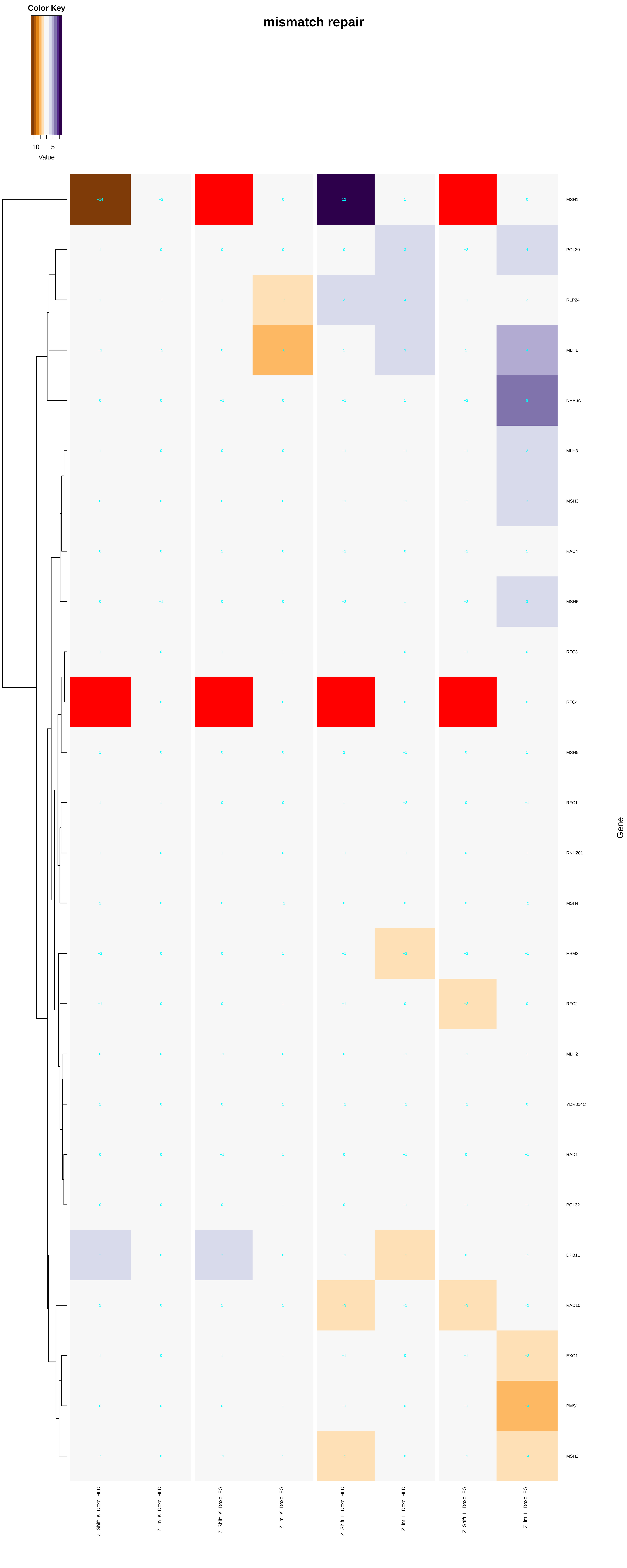
Color Key
−10
5
Value
mismatch repair
MSH1
POL30
RLP24
MLH1
NHP6A
MLH3
MSH3
RAD4
MSH6
RFC3
RFC4
MSH5
RFC1
RNH201
MSH4
HSM3
RFC2
MLH2
YDR314C
RAD1
POL32
DPB11
RAD10
EXO1
PMS1
MSH2
Gene
Z_Shift_K_Doxo_HLD
Z_Im_K_Doxo_HLD
Z_Shift_K_Doxo_EG
Z_Im_K_Doxo_EG
Z_Shift_L_Doxo_HLD
Z_Im_L_Doxo_HLD
Z_Shift_L_Doxo_EG
Z_Im_L_Doxo_EG

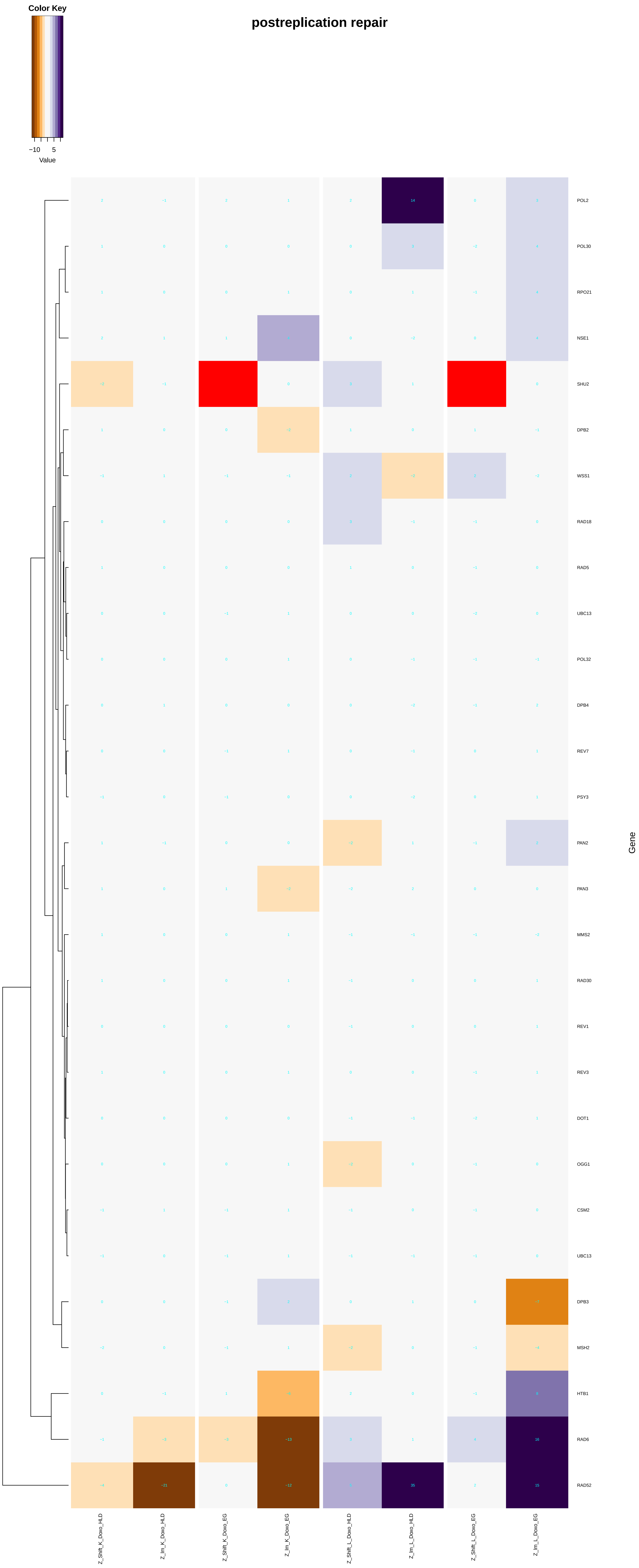
Color Key
−10
5
Value
postreplication repair
POL2
POL30
RPO21
NSE1
SHU2
DPB2
WSS1
RAD18
RAD5
UBC13
POL32
DPB4
REV7
PSY3
PAN2
PAN3
MMS2
RAD30
REV1
REV3
DOT1
OGG1
CSM2
UBC13
DPB3
MSH2
HTB1
RAD6
RAD52
Gene
Z_Shift_K_Doxo_HLD
Z_lm_K_Doxo_HLD
Z_Shift_K_Doxo_EG
Z_lm_K_Doxo_EG
Z_Shift_L_Doxo_HLD
Z_lm_L_Doxo_HLD
Z_Shift_L_Doxo_EG
Z_lm_L_Doxo_EG

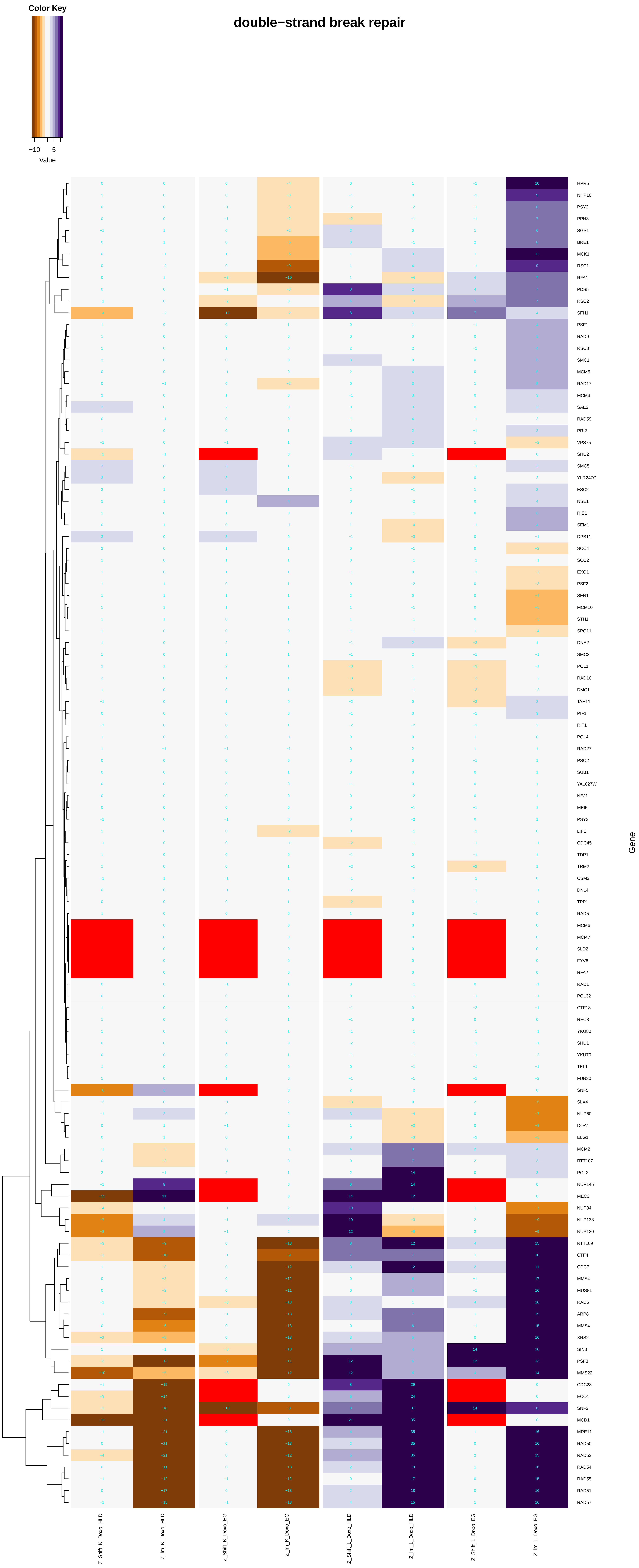
double−strand break repair
Color Key
−10
5
Value
HPR5
NHP10
PSY2
PPH3
SGS1
BRE1
MCK1
RSC1
RFA1
PDS5
RSC2
SFH1
PSF1
RAD9
RSC8
SMC1
MCM5
RAD17
MCM3
SAE2
RAD59
PRI2
VPS75
SHU2
SMC5
YLR247C
ESC2
NSE1
RIS1
SEM1
DPB11
SCC4
SCC2
EXO1
PSF2
SEN1
MCM10
STH1
SPO11
DNA2
SMC3
POL1
RAD10
DMC1
TAH11
PIF1
RIF1
POL4
RAD27
PSO2
SUB1
YAL027W
NEJ1
MEI5
PSY3
LIF1
CDC45
TDP1
TRM2
CSM2
DNL4
TPP1
RAD5
MCM6
MCM7
SLD2
FYV6
RFA2
RAD1
POL32
CTF18
REC8
YKU80
SHU1
YKU70
TEL1
FUN30
SNF5
SLX4
NUP60
DOA1
ELG1
MCM2
RTT107
POL2
NUP145
MEC3
NUP84
NUP133
NUP120
RTT109
CTF4
CDC7
MMS4
MUS81
RAD6
ARP8
MMS4
XRS2
SIN3
PSF3
MMS22
CDC28
ECO1
SNF2
MCD1
MRE11
RAD50
RAD52
RAD54
RAD55
RAD51
RAD57
Gene
Z_Shift_K_Doxo_HLD
Z_lm_K_Doxo_HLD
Z_Shift_K_Doxo_EG
Z_lm_K_Doxo_EG
Z_Shift_L_Doxo_HLD
Z_lm_L_Doxo_HLD
Z_Shift_L_Doxo_EG
Z_lm_L_Doxo_EG

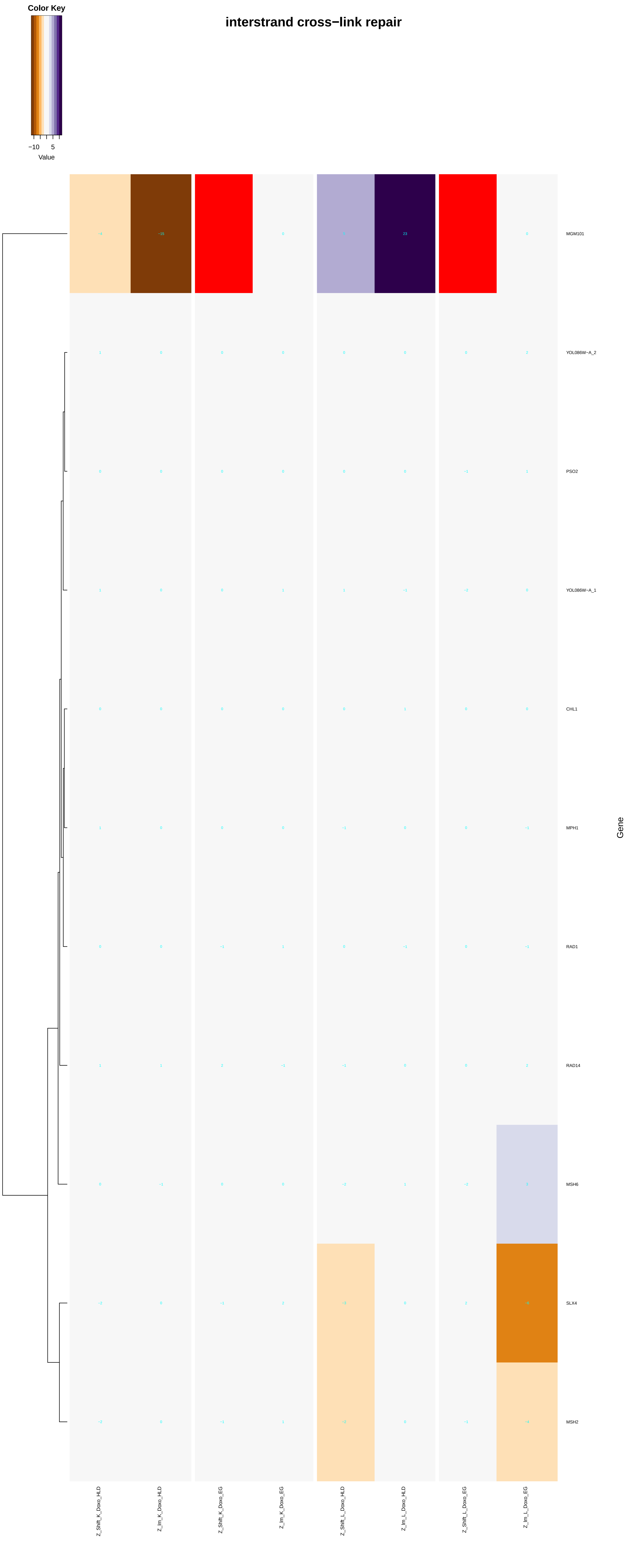

Color Key
−10
5
Value
interstrand cross−link repair
MGM101
YOL086W−A_2
PSO2
YOL086W−A_1
CHL1
MPH1
RAD1
RAD14
MSH6
SLX4
MSH2
Gene
Z_Shift_K_Doxo_HLD
Z_lm_K_Doxo_HLD
Z_Shift_K_Doxo_EG
Z_lm_K_Doxo_EG
Z_Shift_L_Doxo_HLD
Z_lm_L_Doxo_HLD
Z_Shift_L_Doxo_EG
Z_lm_L_Doxo_EG

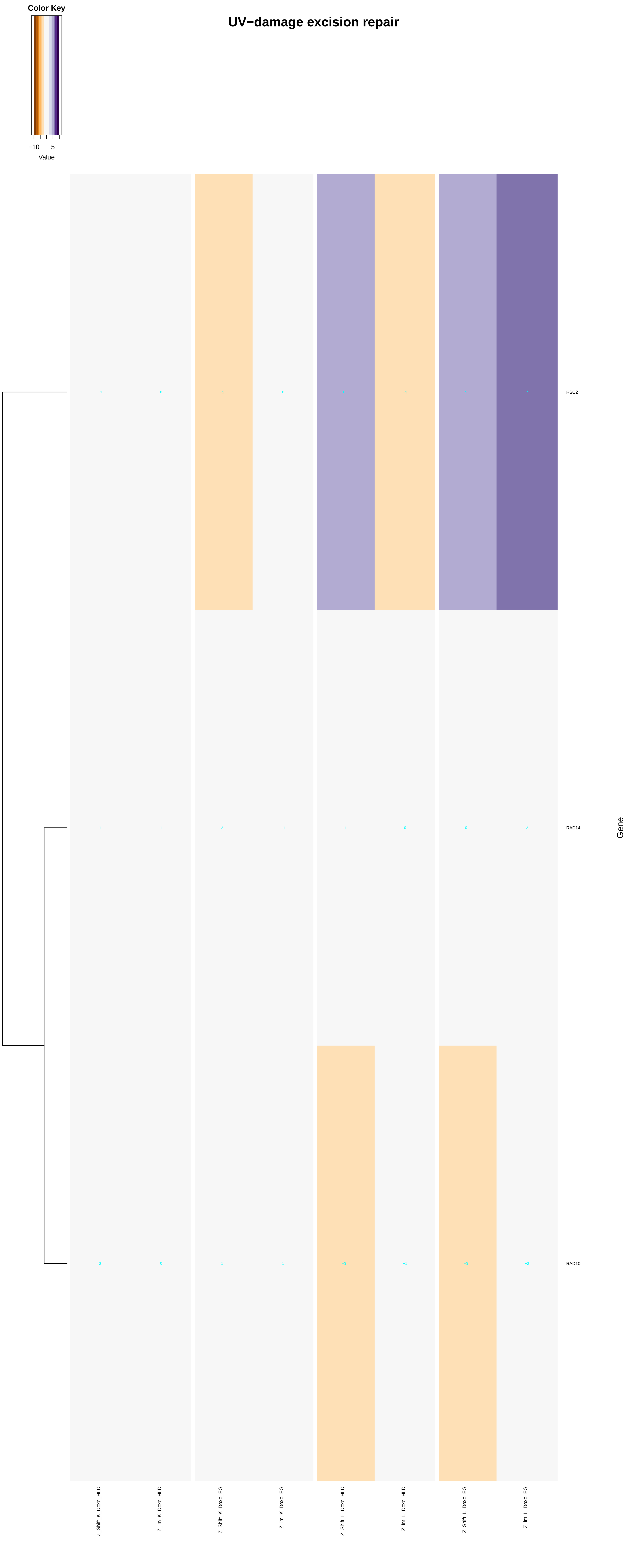
UV−damage excision repair
Color Key
−10
5
Value
RSC2
RAD14
RAD10
Gene
Z_Shift_K_Doxo_HLD
Z_Im_K_Doxo_HLD
Z_Shift_K_Doxo_EG
Z_Im_K_Doxo_EG
Z_Shift_L_Doxo_HLD
Z_Im_L_Doxo_HLD
Z_Shift_L_Doxo_EG
Z_Im_L_Doxo_EG

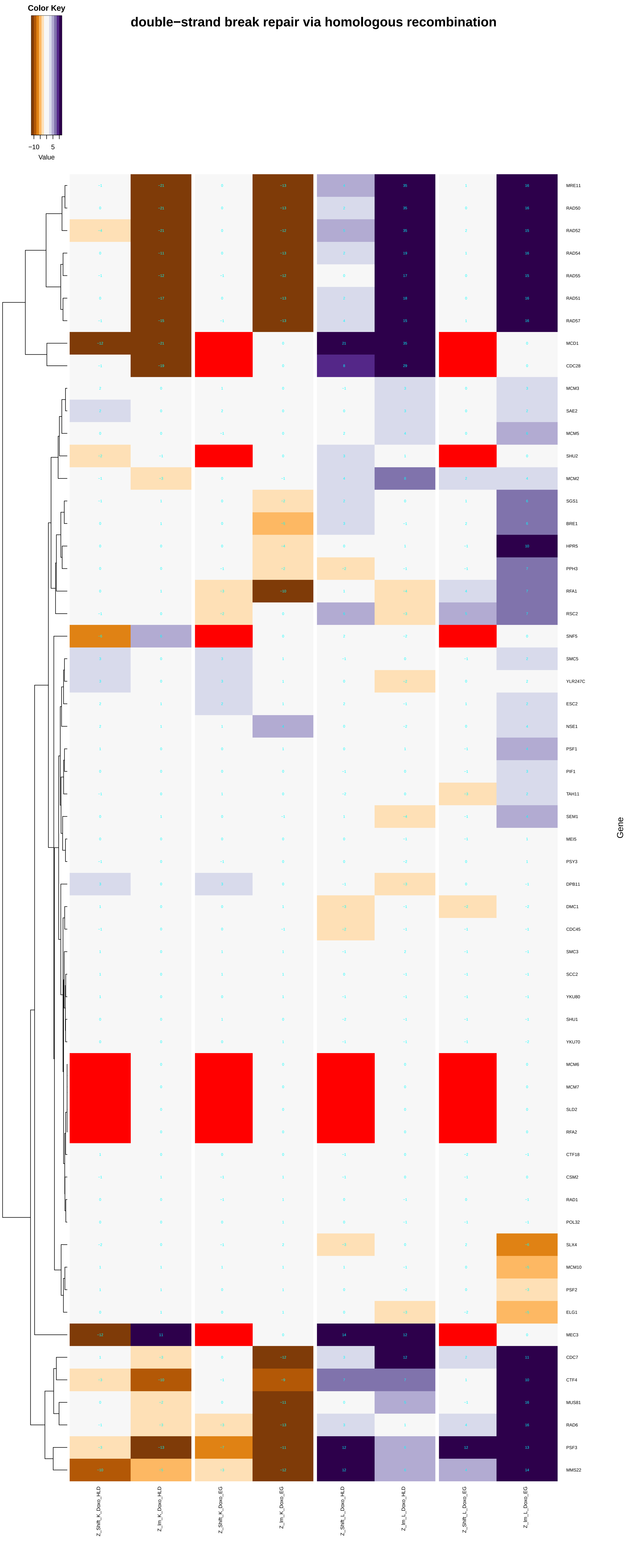
double−strand break repair via homologous recombination
Color Key
−10
5
Value
Gene
MRE11
RAD50
RAD52
RAD54
RAD55
RAD51
RAD57
MCD1
CDC28
MCM3
SAE2
MCM5
SHU2
MCM2
SGS1
BRE1
HPR5
PPH3
RFA1
RSC2
SNF5
SMC5
YLR247C
ESC2
NSE1
PSF1
PIF1
TAH11
SEM1
MEI5
PSY3
DPB11
DMC1
CDC45
SMC3
SCC2
YKU80
SHU1
YKU70
MCM6
MCM7
SLD2
RFA2
CTF18
CSM2
RAD1
POL32
SLX4
MCM10
PSF2
ELG1
MEC3
CDC7
CTF4
MUS81
RAD6
PSF3
MMS22
Z_Shift_K_Doxo_HLD
Z_lm_K_Doxo_HLD
Z_Shift_K_Doxo_EG
Z_lm_K_Doxo_EG
Z_Shift_L_Doxo_HLD
Z_lm_L_Doxo_HLD
Z_Shift_L_Doxo_EG
Z_lm_L_Doxo_EG

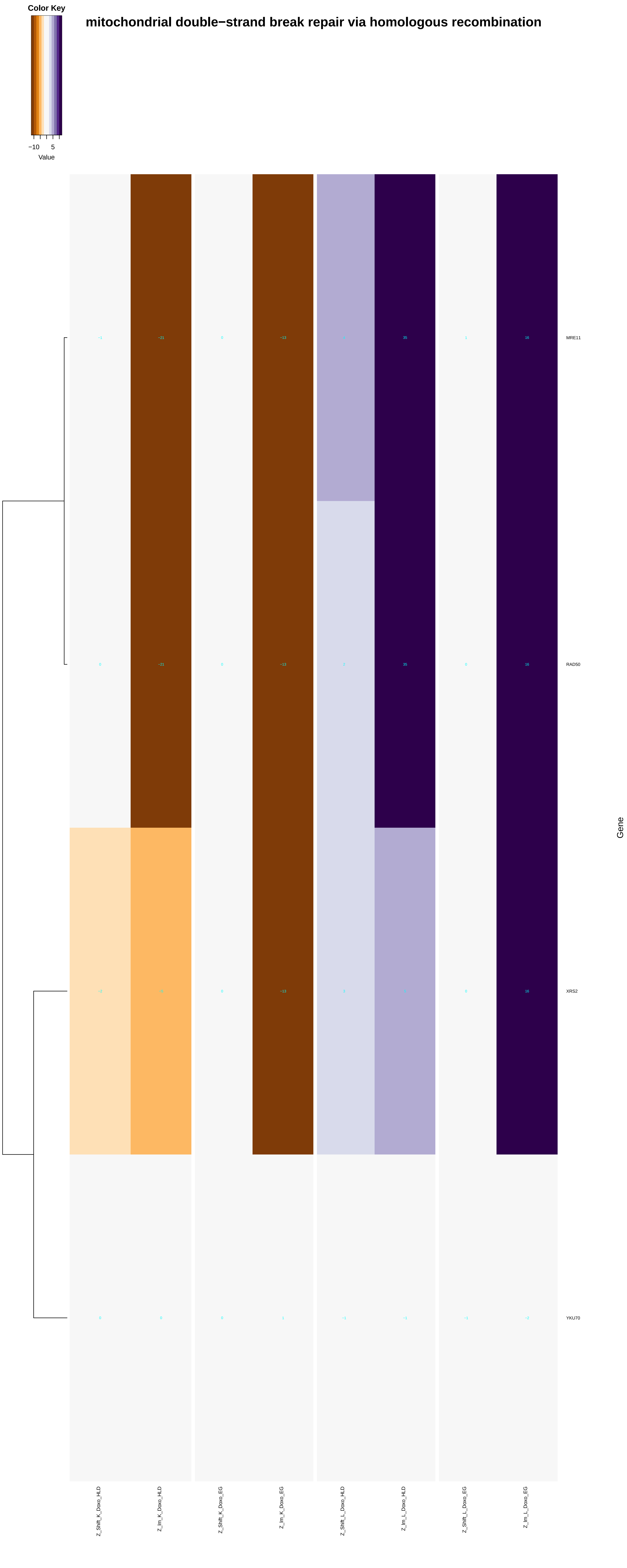

Color Key
−10
5
Value
mitochondrial double−strand break repair via homologous recombination
−1
−21
0
−13
4
35
1
16
MRE11
0
−21
0
−13
2
35
0
16
RAD50
−2
−5
0
−13
3
5
0
16
XRS2
0
0
0
1
−1
−1
−1
−2
YKU70
Gene
Z_Shift_K_Doxo_HLD
Z_Im_K_Doxo_HLD
Z_Shift_K_Doxo_EG
Z_Im_K_Doxo_EG
Z_Shift_L_Doxo_HLD
Z_Im_L_Doxo_HLD
Z_Shift_L_Doxo_EG
Z_Im_L_Doxo_EG

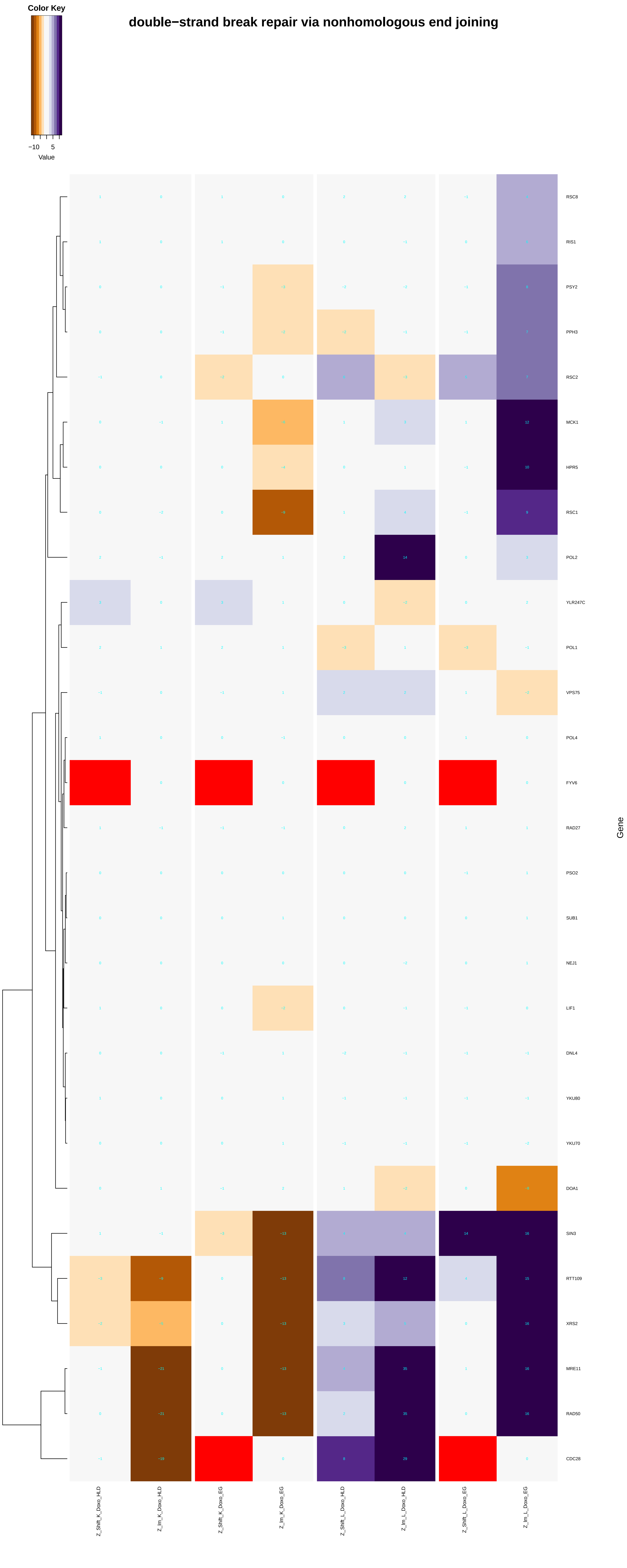
double−strand break repair via nonhomologous end joining
Color Key
−10
5
Value
RSC8
RIS1
PSY2
PPH3
RSC2
MCK1
HPR5
RSC1
POL2
YLR247C
POL1
VPS75
POL4
FYV6
RAD27
PSO2
SUB1
NEJ1
LIF1
DNL4
YKU80
YKU70
DOA1
SIN3
RTT109
XRS2
MRE11
RAD50
CDC28
Gene
Z_Shift_K_Doxo_HLD
Z_lm_K_Doxo_HLD
Z_Shift_K_Doxo_EG
Z_lm_K_Doxo_EG
Z_Shift_L_Doxo_HLD
Z_lm_L_Doxo_HLD
Z_Shift_L_Doxo_EG
Z_lm_L_Doxo_EG

double−strand break repair via single−strand annealing

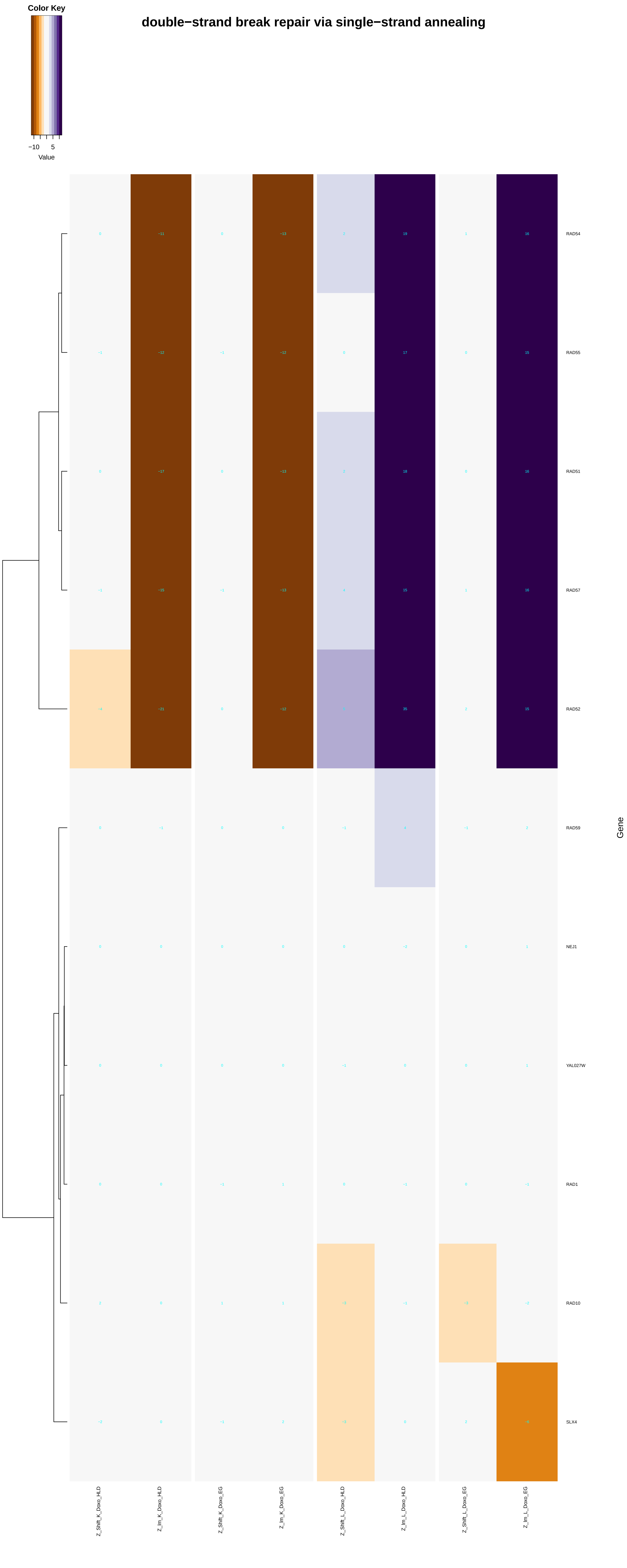

transcription−coupled nucleotide−excision repair

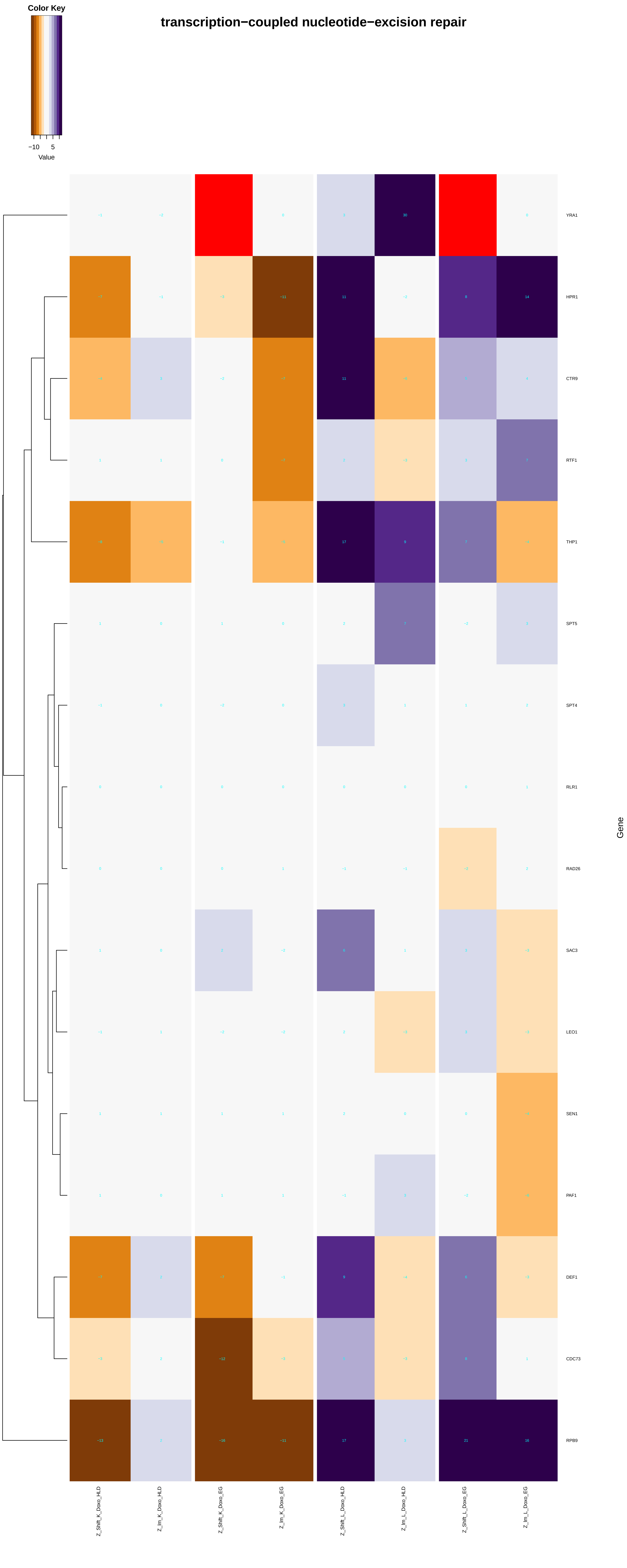

global genome nucleotide−excision repair

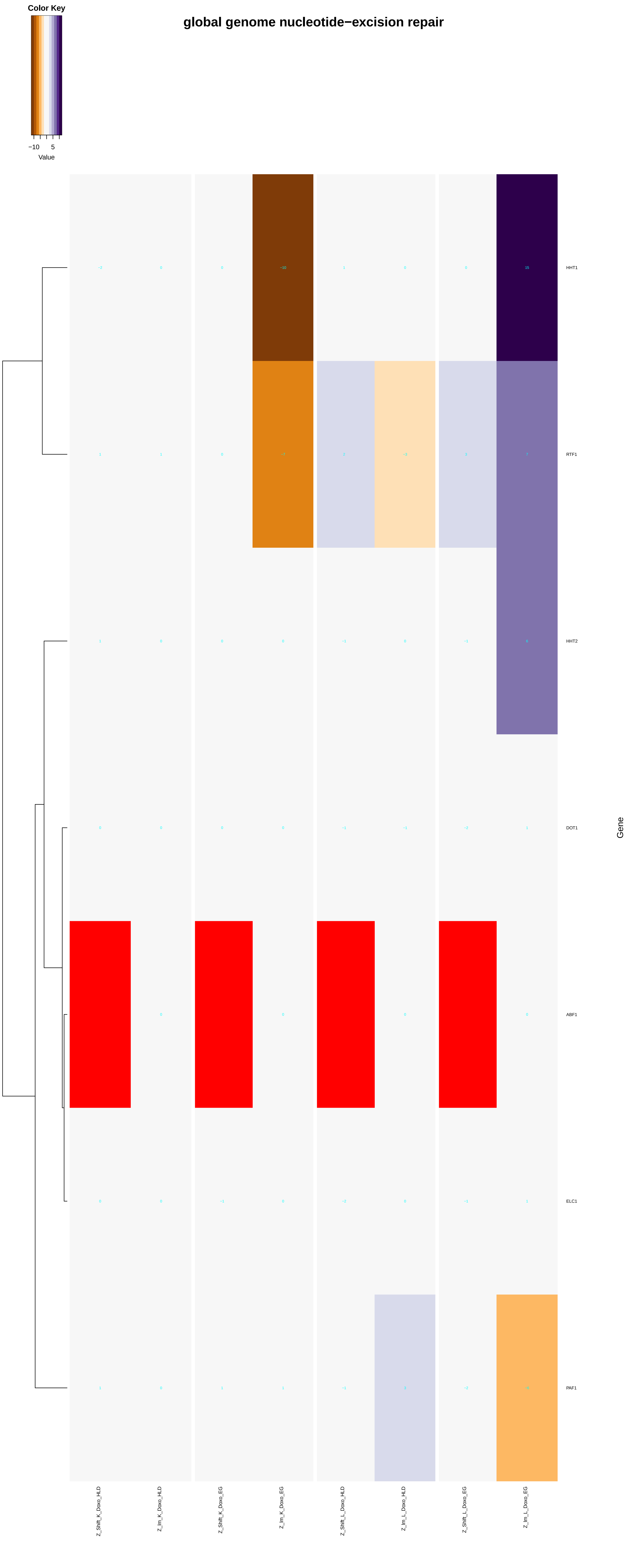

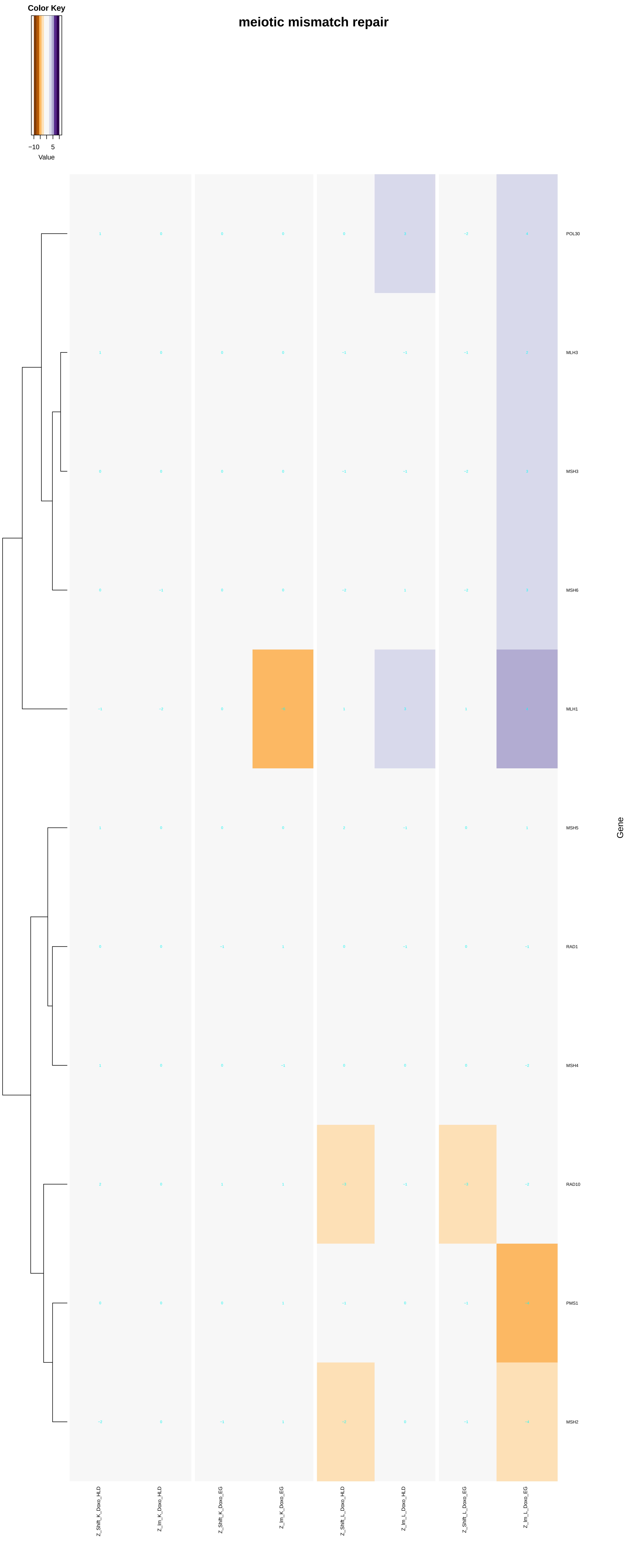
meiotic mismatch repair
Color Key
−10
5
Value
POL30
MLH3
MSH3
MSH6
MLH1
MSH5
RAD1
MSH4
RAD10
PMS1
MSH2
Gene
Z_Shift_K_Doxo_HLD
Z_Im_K_Doxo_HLD
Z_Shift_K_Doxo_EG
Z_Im_K_Doxo_EG
Z_Shift_L_Doxo_HLD
Z_Im_L_Doxo_HLD
Z_Shift_L_Doxo_EG
Z_Im_L_Doxo_EG

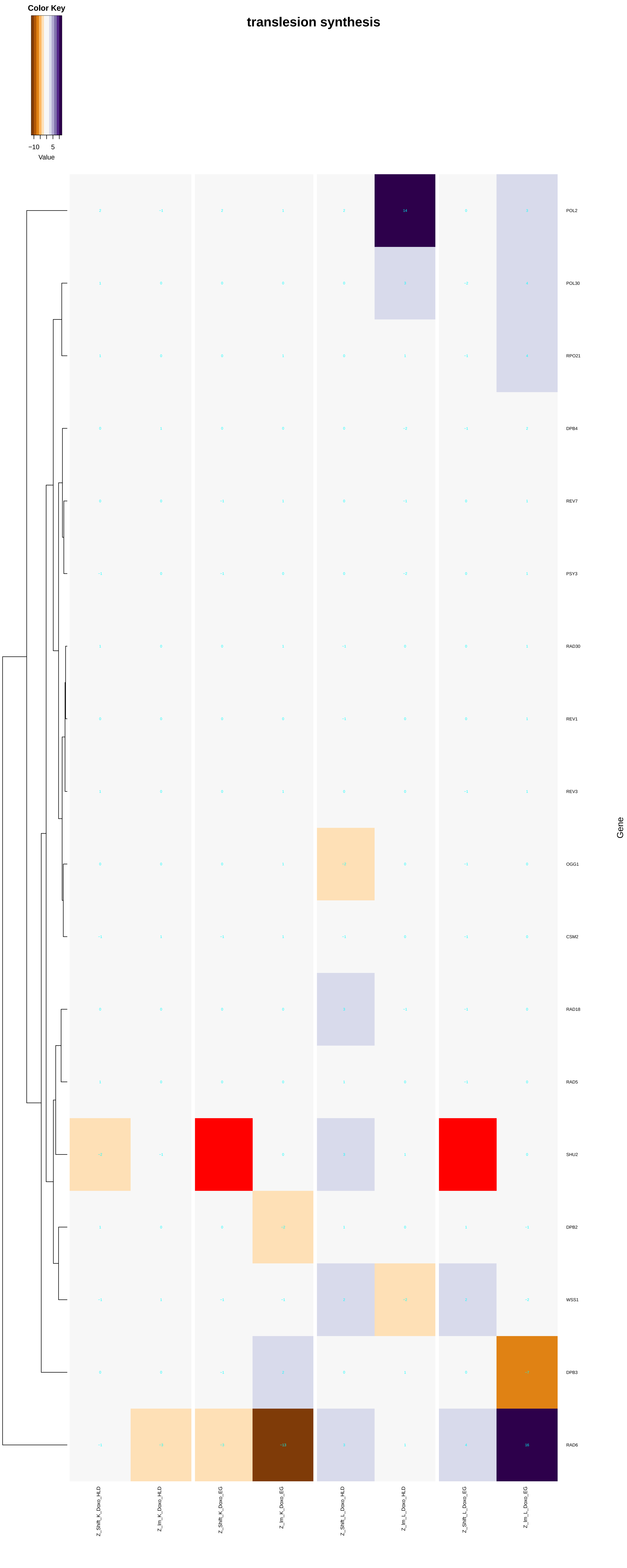

Color Key
−10
5
Value
translesion synthesis
POL2
POL30
RPO21
DPB4
REV7
PSY3
RAD30
REV1
REV3
OGG1
CSM2
RAD18
RAD5
SHU2
DPB2
WSS1
DPB3
RAD6
Gene
Z_Shift_K_Doxo_HLD
Z_Im_K_Doxo_HLD
Z_Shift_K_Doxo_EG
Z_Im_K_Doxo_EG
Z_Shift_L_Doxo_HLD
Z_Im_L_Doxo_HLD
Z_Shift_L_Doxo_EG
Z_Im_L_Doxo_EG

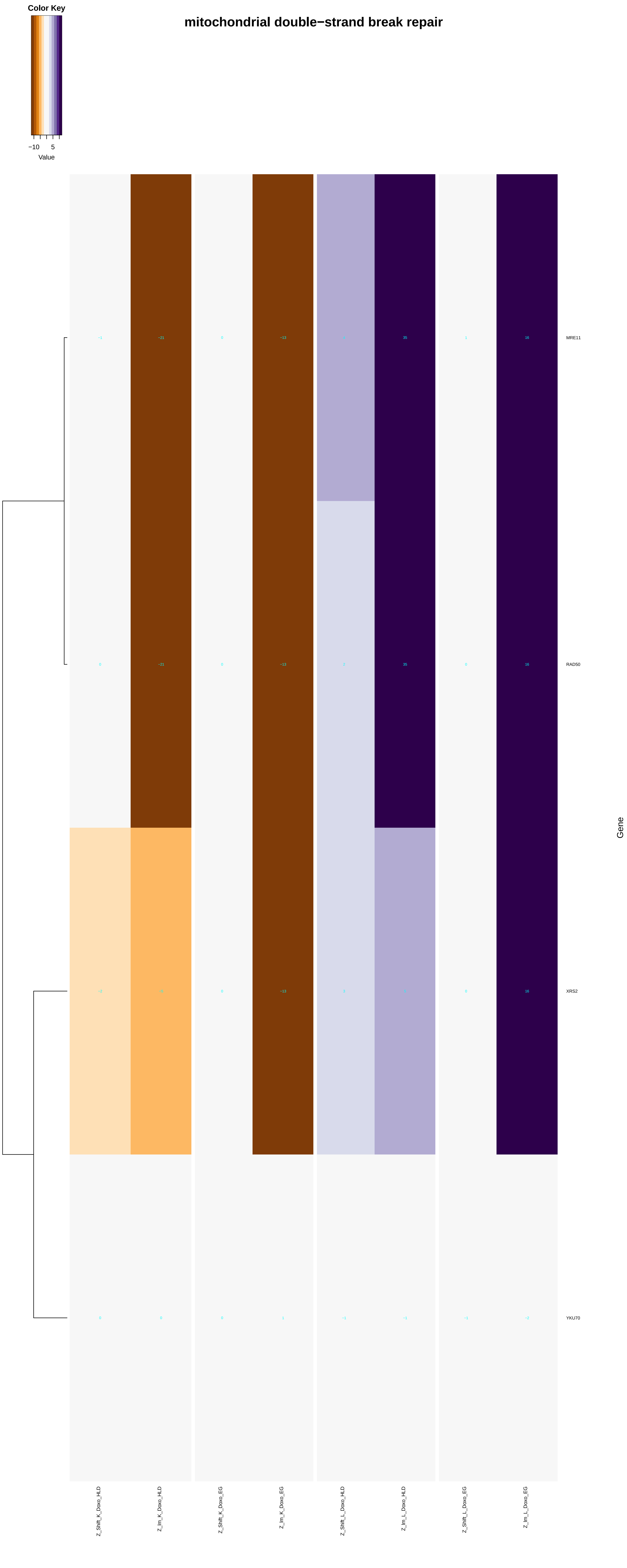

mitochondrial double−strand break repair
Color Key
−10
5
Value
MRE11
RAD50
XRS2
YKU70
Gene
Z_Shift_K_Doxo_HLD
Z_Im_K_Doxo_HLD
Z_Shift_K_Doxo_EG
Z_Im_K_Doxo_EG
Z_Shift_L_Doxo_HLD
Z_Im_L_Doxo_HLD
Z_Shift_L_Doxo_EG
Z_Im_L_Doxo_EG

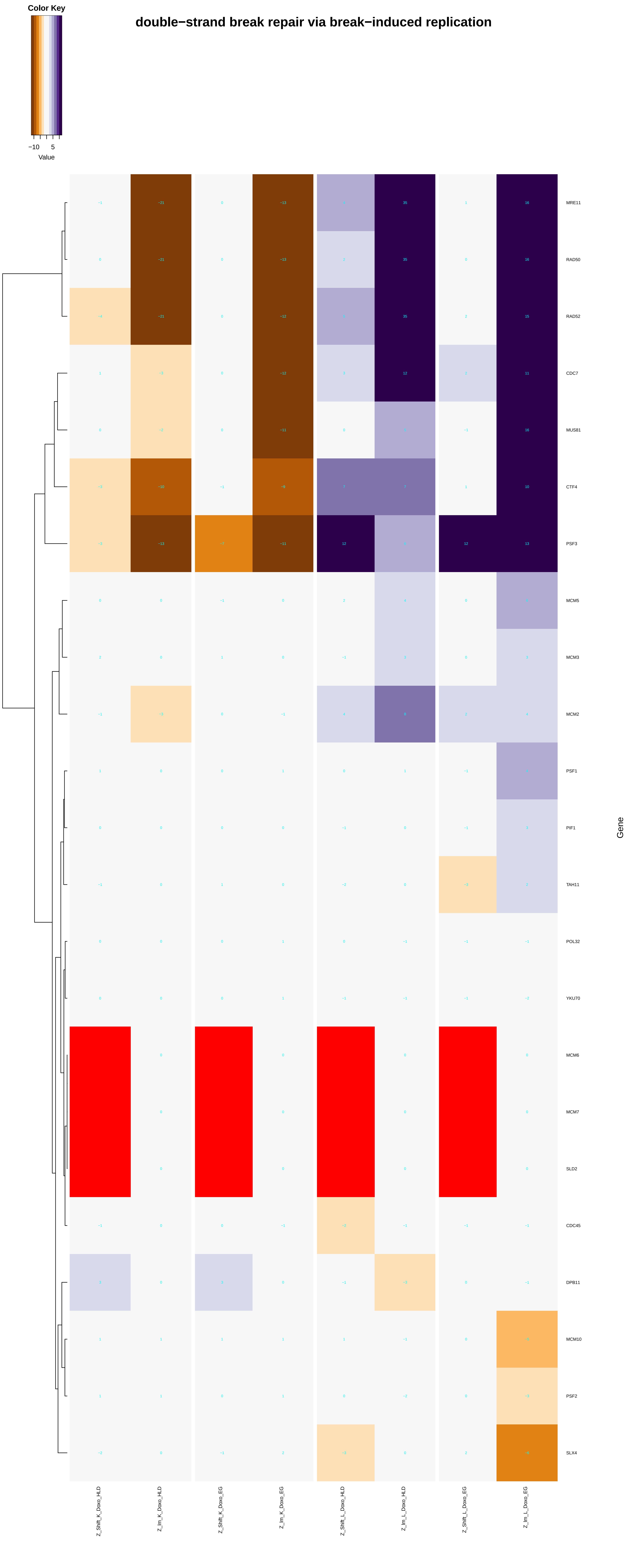
double−strand break repair via break−induced replication
Color Key
−10
5
Value
MRE11
RAD50
RAD52
CDC7
MUS81
CTF4
PSF3
MCM5
MCM3
MCM2
PSF1
PIF1
TAH11
POL32
YKU70
MCM6
MCM7
SLD2
CDC45
DPB11
MCM10
PSF2
SLX4
Gene
Z_Shift_K_Doxo_HLD
Z_Im_K_Doxo_HLD
Z_Shift_K_Doxo_EG
Z_Im_K_Doxo_EG
Z_Shift_L_Doxo_HLD
Z_Im_L_Doxo_HLD
Z_Shift_L_Doxo_EG
Z_Im_L_Doxo_EG

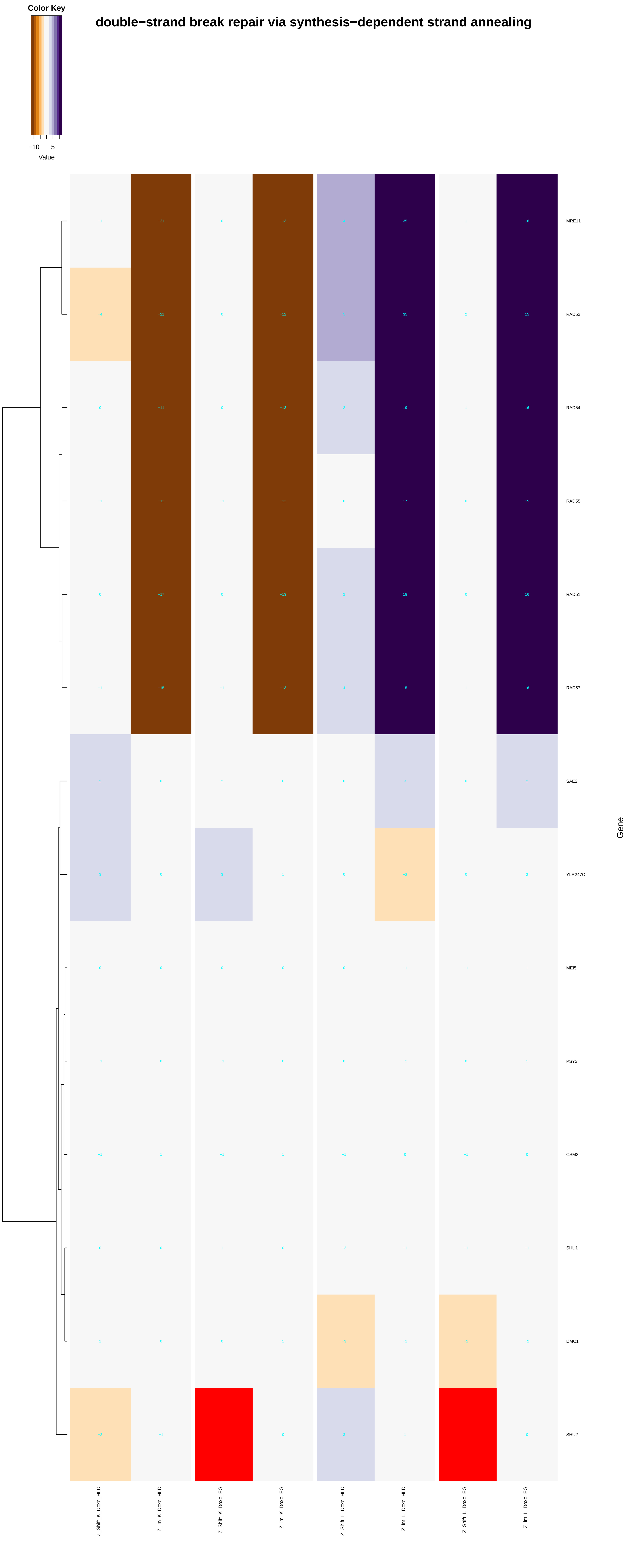
Color Key
−10
5
Value
double−strand break repair via synthesis−dependent strand annealing
MRE11
RAD52
RAD54
RAD55
RAD51
RAD57
SAE2
YLR247C
MEI5
PSY3
CSM2
SHU1
DMC1
SHU2
Gene
Z_Shift_K_Doxo_HLD
Z_Im_K_Doxo_HLD
Z_Shift_K_Doxo_EG
Z_Im_K_Doxo_EG
Z_Shift_L_Doxo_HLD
Z_Im_L_Doxo_HLD
Z_Shift_L_Doxo_EG
Z_Im_L_Doxo_EG

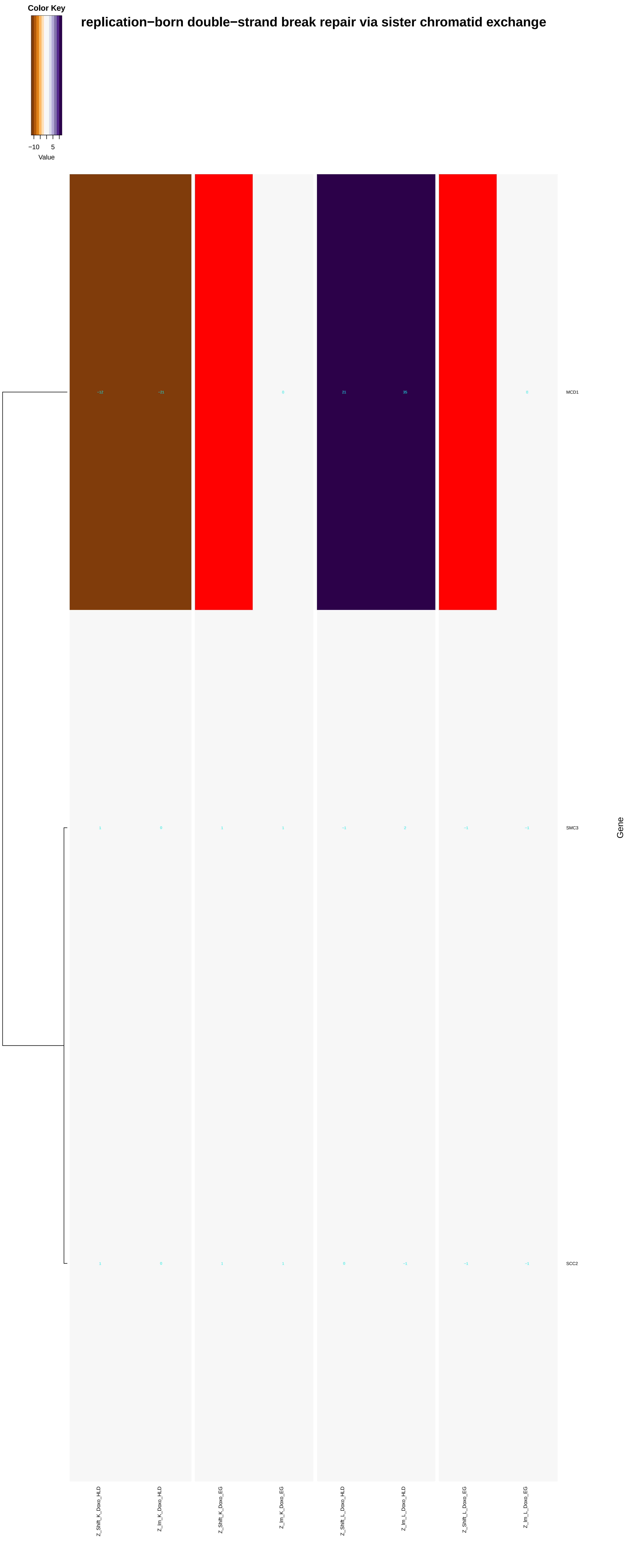
Color Key
−10
5
Value
replication−born double−strand break repair via sister chromatid exchange
MCD1
SMC3
SCC2
Gene
Z_Shift_K_Doxo_HLD
Z_lm_K_Doxo_HLD
Z_Shift_K_Doxo_EG
Z_lm_K_Doxo_EG
Z_Shift_L_Doxo_HLD
Z_lm_L_Doxo_HLD
Z_Shift_L_Doxo_EG
Z_lm_L_Doxo_EG

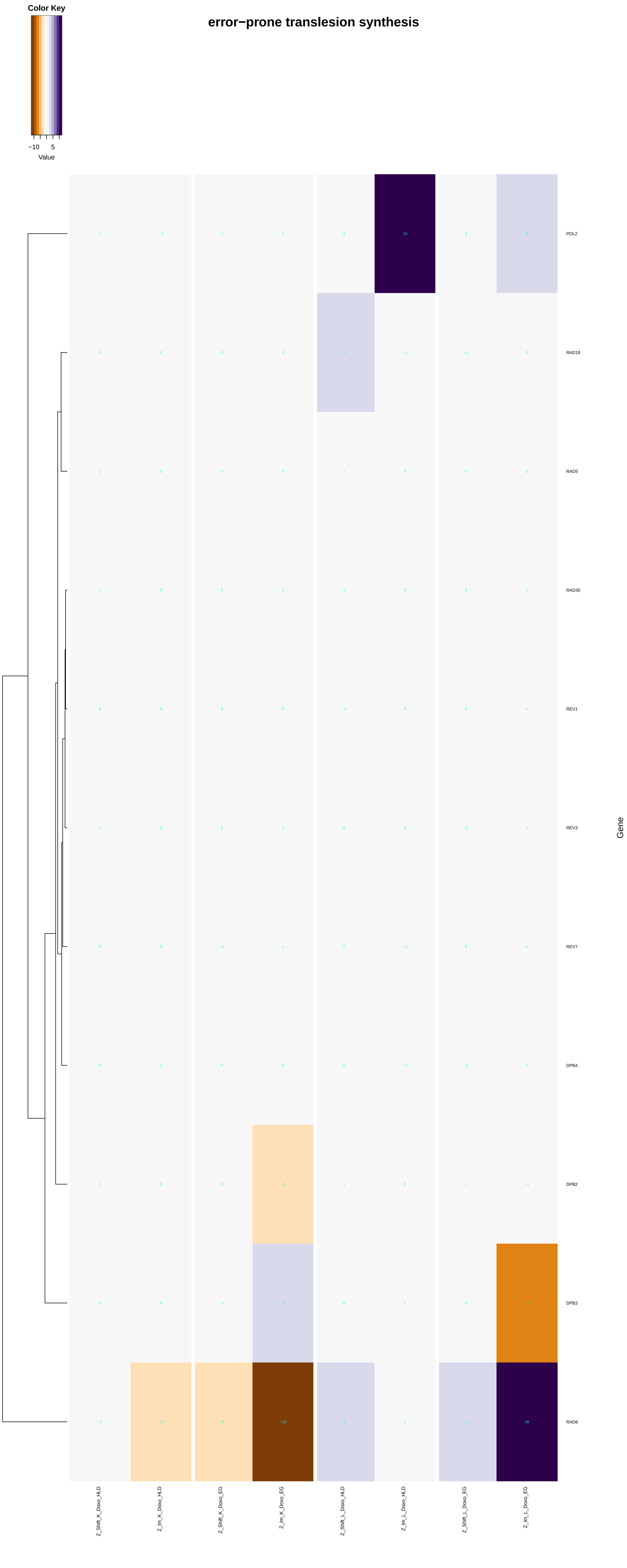
error−prone translesion synthesis
Color Key
−10
5
Value
POL2
RAD18
RAD5
RAD30
REV1
REV3
REV7
DPB4
DPB2
DPB3
RAD6
Gene
Z_Shift_K_Doxo_HLD
Z_Im_K_Doxo_HLD
Z_Shift_K_Doxo_EG
Z_Im_K_Doxo_EG
Z_Shift_L_Doxo_HLD
Z_Im_L_Doxo_HLD
Z_Shift_L_Doxo_EG
Z_Im_L_Doxo_EG

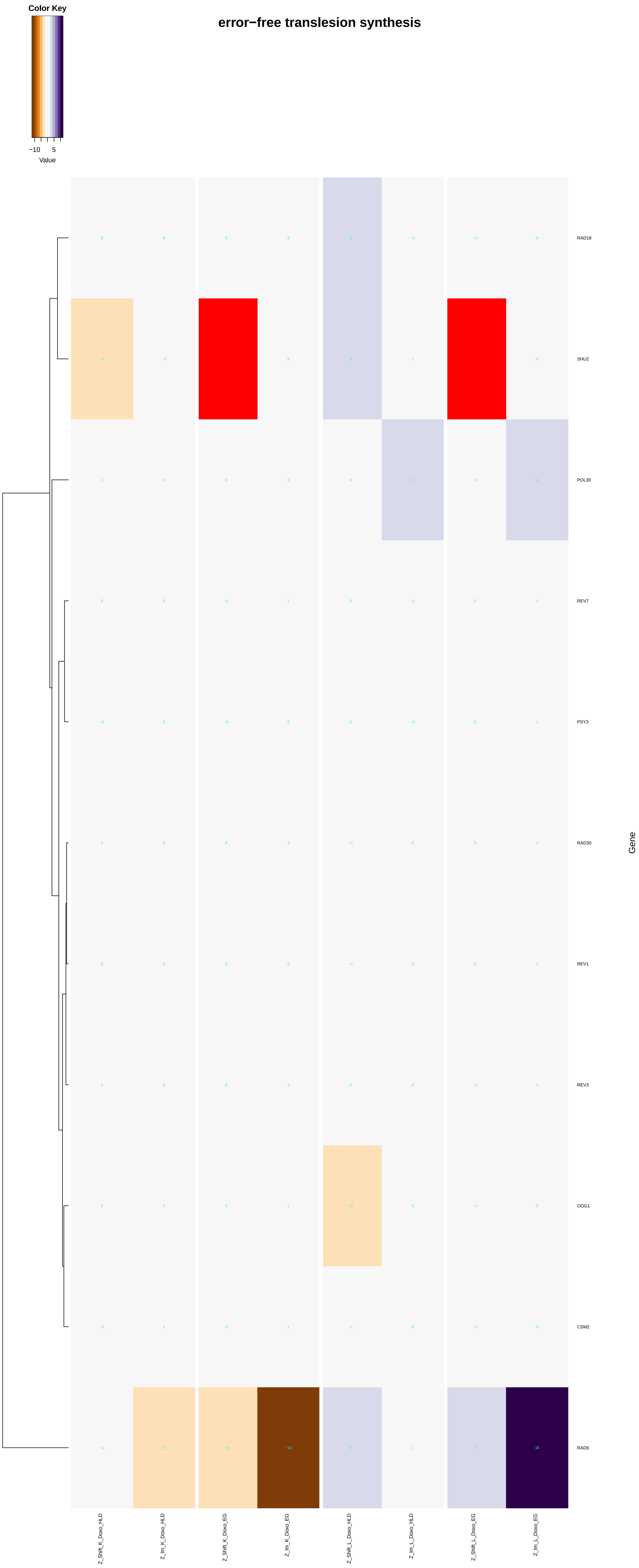

error−free translesion synthesis
Color Key
−10
5
Value
RAD18
SHU2
POL30
REV7
PSY3
RAD30
REV1
REV3
OGG1
CSM2
RAD6
Gene
Z_Shift_K_Doxo_HLD
Z_Im_K_Doxo_HLD
Z_Shift_K_Doxo_EG
Z_Im_K_Doxo_EG
Z_Shift_L_Doxo_HLD
Z_Im_L_Doxo_HLD
Z_Shift_L_Doxo_EG
Z_Im_L_Doxo_EG
