# Supplementary material for: A yeast phenomic model for the influence of Warburg metabolism on genetic buffering of doxorubicin
Source: Cancer Metab. 2019 Oct 23;7:9. doi: 10.1186/s40170-019-0201-3 (PMC6806529; doi:10.1186/s40170-019-0201-3)

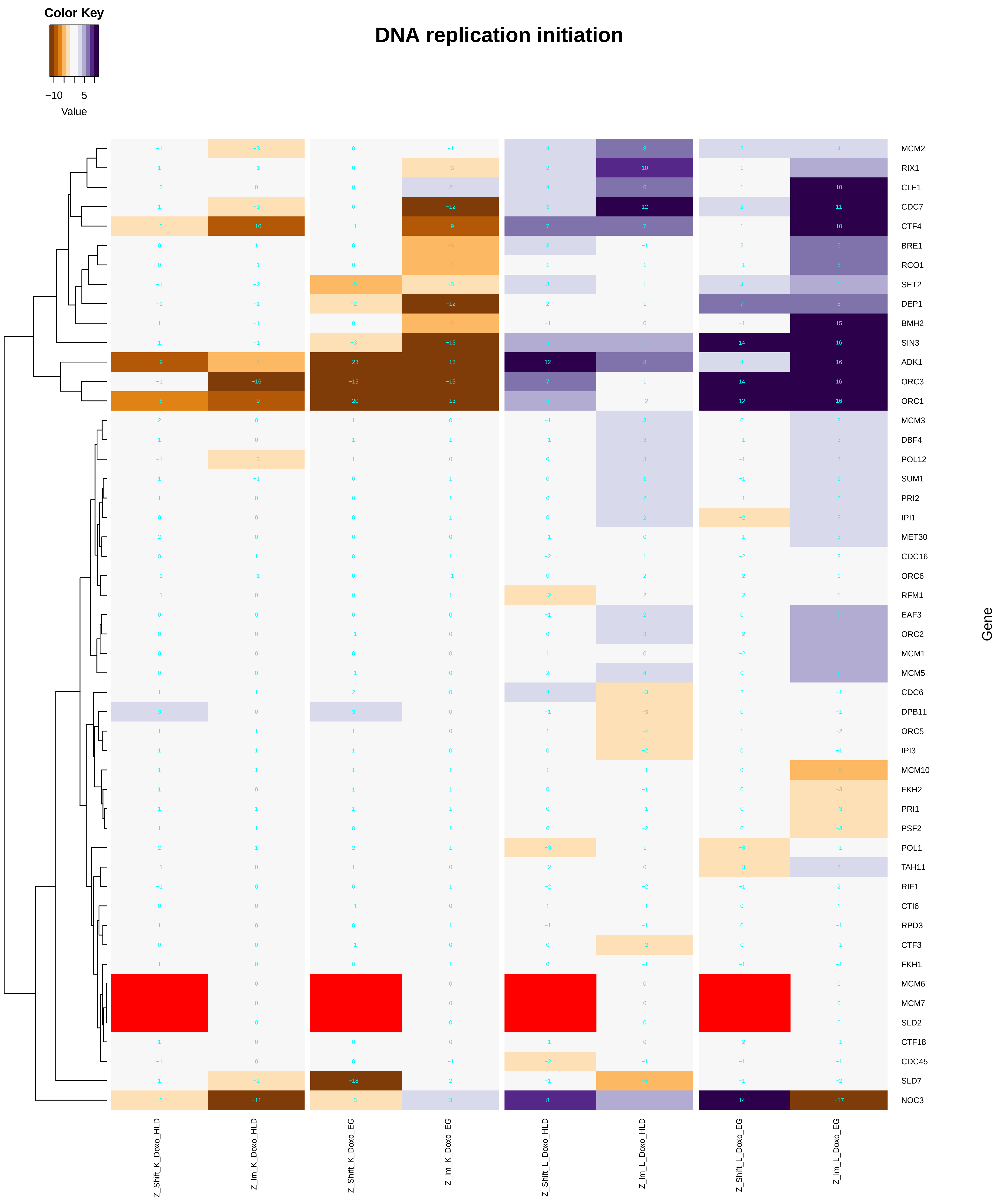
Color Key
−10 5
Value
DNA replication initiation
MCM2
RIX1
CLF1
CDC7
CTF4
BRE1
RCO1
SET2
DEP1
BMH2
SIN3
ADK1
ORC3
ORC1
MCM3
DBF4
POL12
SUM1
PRI2
IPI1
MET30
CDC16
ORC6
RFM1
EAF3
ORC2
MCM1
MCM5
CDC6
DPB11
ORC5
IPI3
MCM10
FKH2
PRI1
PSF2
POL1
TAH11
RIF1
CTI6
RPD3
CTF3
FKH1
MCM6
MCM7
SLD2
CTF18
CDC45
SLD7
NOC3
Gene
Z_Shift_K_Doxo_HLD
Z_Im_K_Doxo_HLD
Z_Shift_K_Doxo_EG
Z_Im_K_Doxo_EG
Z_Shift_L_Doxo_HLD
Z_Im_L_Doxo_HLD
Z_Shift_L_Doxo_EG
Z_Im_L_Doxo_EG

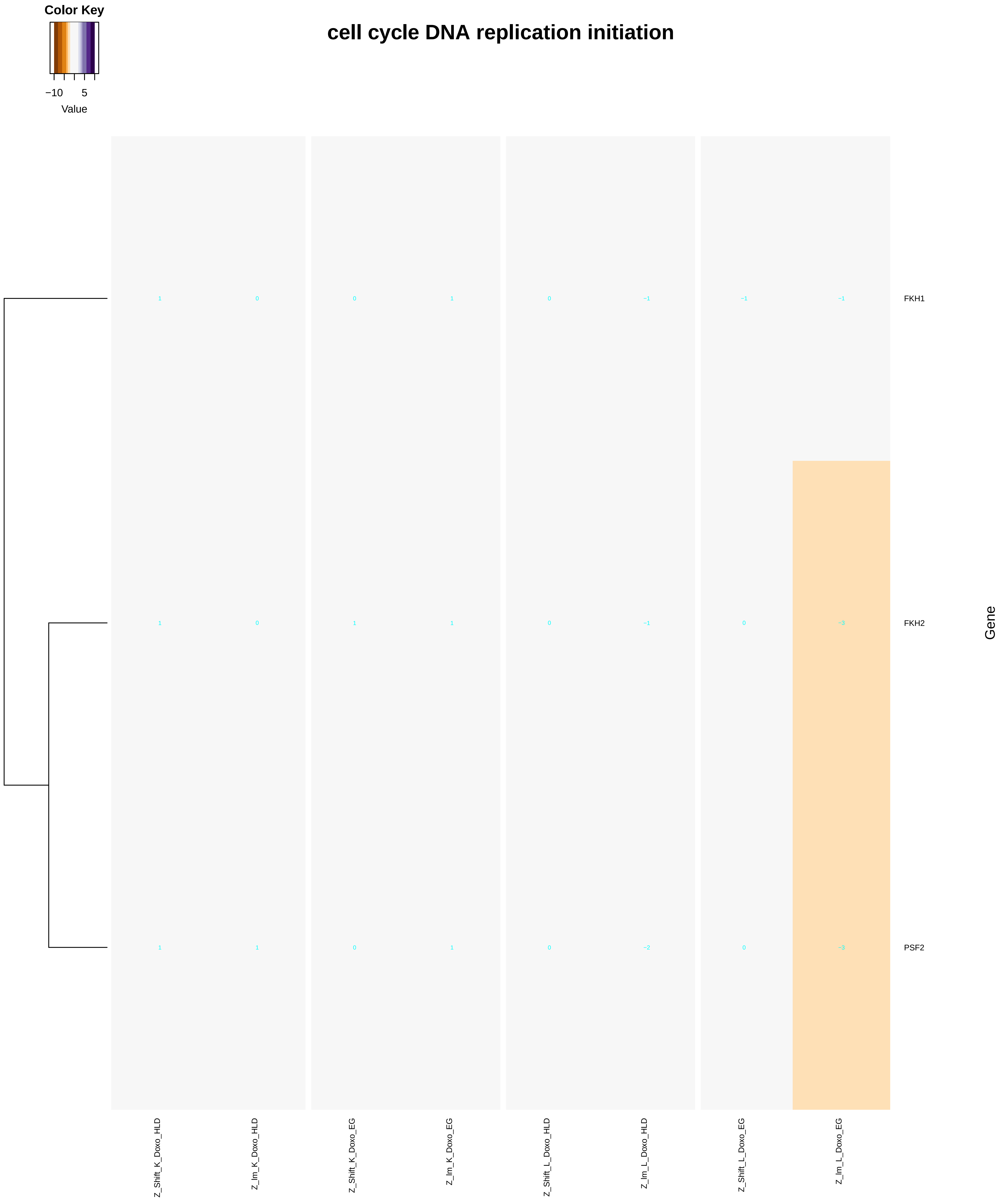
Color Key
−10
5
Value
cell cycle DNA replication initiation
1
0
0
1
0
−1
−1
−1
FKH1
1
0
1
1
0
−1
0
−3
FKH2
1
1
0
1
0
−2
0
−3
PSF2
Gene
Z_Shift_K_Doxo_HLD
Z_Im_K_Doxo_HLD
Z_Shift_K_Doxo_EG
Z_Im_K_Doxo_EG
Z_Shift_L_Doxo_HLD
Z_Im_L_Doxo_HLD
Z_Shift_L_Doxo_EG
Z_Im_L_Doxo_EG

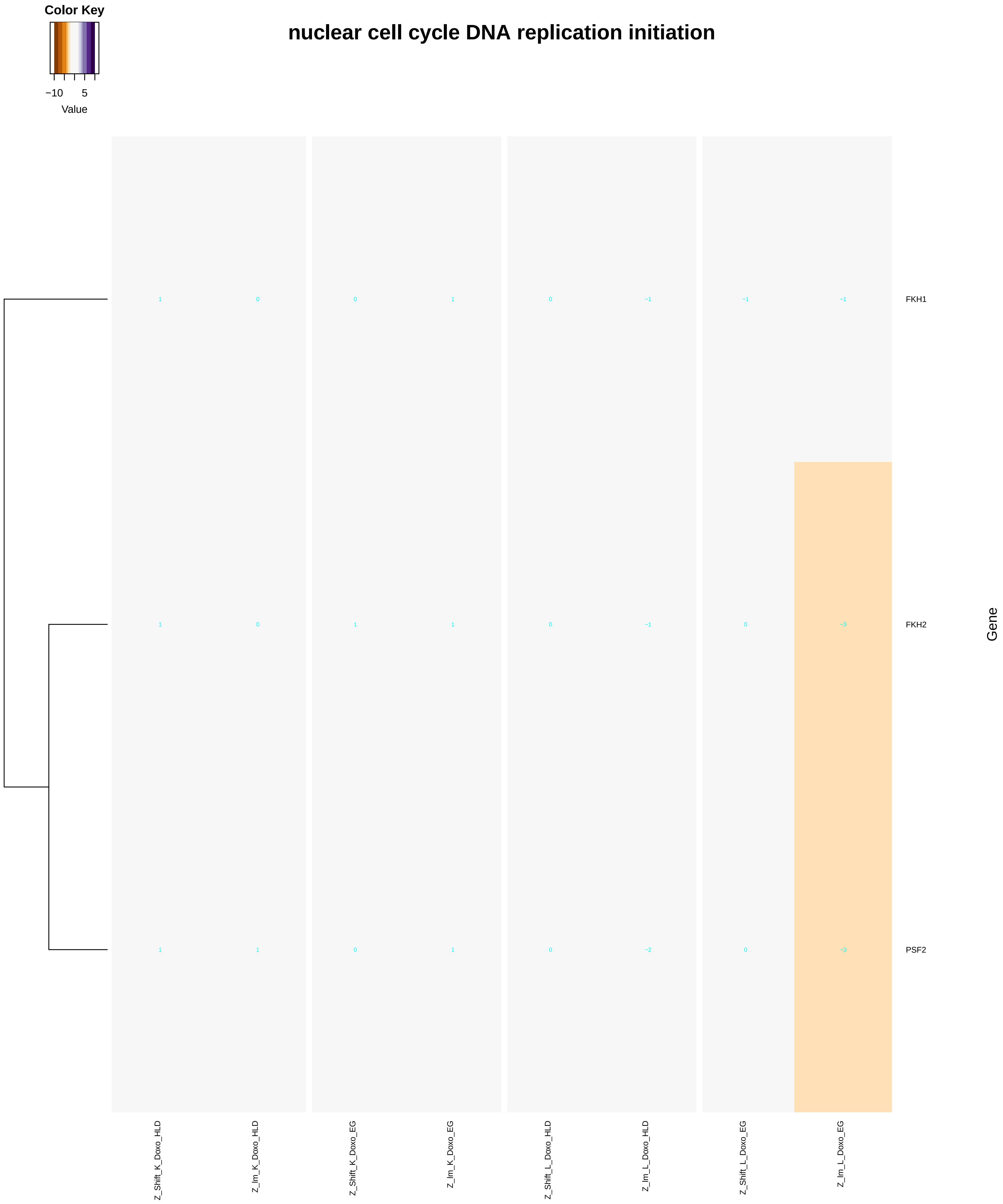
nuclear cell cycle DNA replication initiation
Color Key
−10
5
Value
FKH1
FKH2
PSF2
Gene
Z_Shift_K_Doxo_HLD
Z_Im_K_Doxo_HLD
Z_Shift_K_Doxo_EG
Z_Im_K_Doxo_EG
Z_Shift_L_Doxo_HLD
Z_Im_L_Doxo_HLD
Z_Shift_L_Doxo_EG
Z_Im_L_Doxo_EG
1 0 0 1 0 −1 −1 −1
1 0 1 1 0 −1 0 −3
1 1 0 1 0 −2 0 −3

mitotic DNA replication initiation

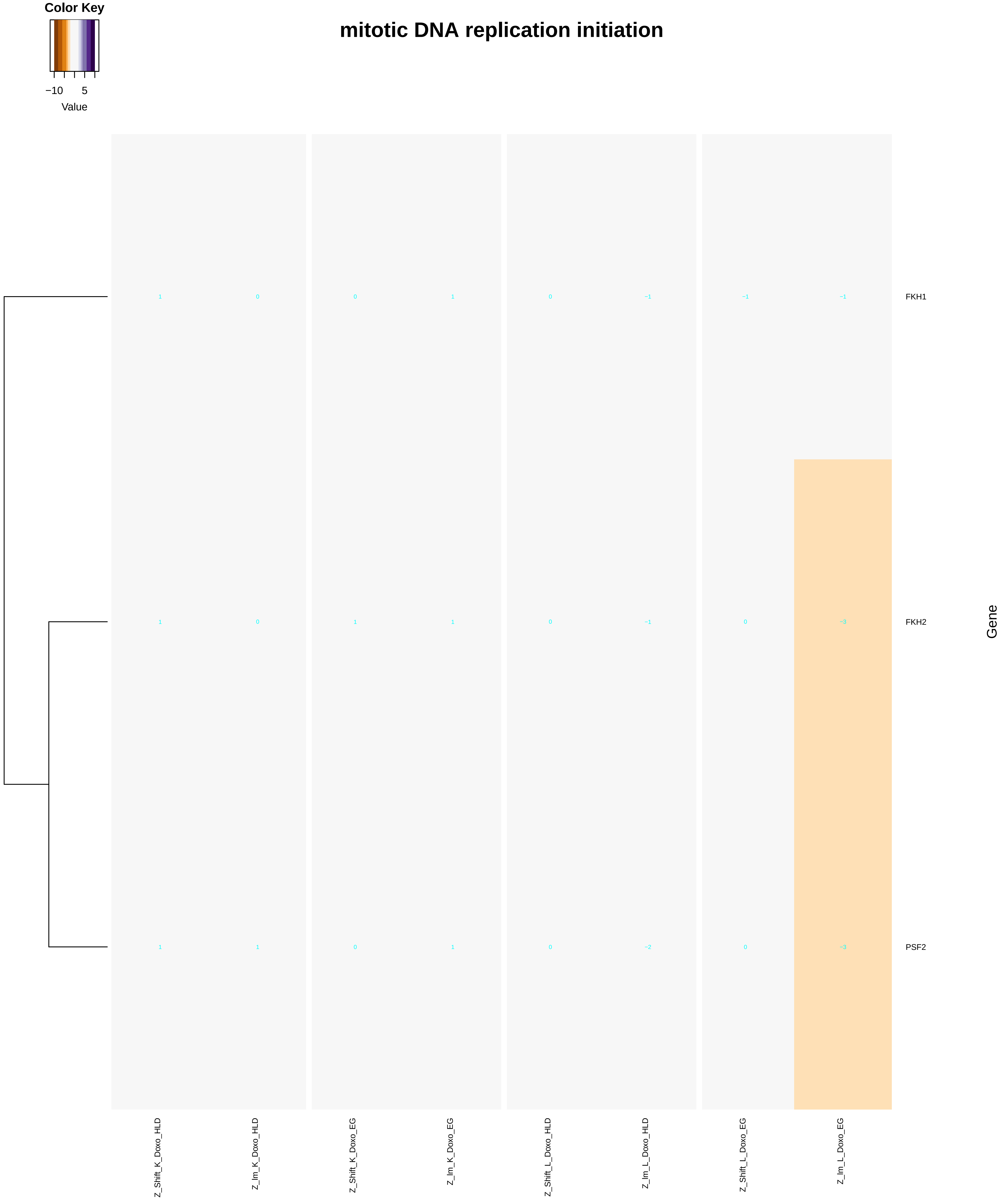

Supplement: Supplementary file 9 — Additional file 9. GO term-specific heatmaps for REMc/GTF-enriched clusters. GO term-specific heatmaps for significant GO process terms were generated as described in methods and Figs. 3 and 4. Any related child terms are presented in subsequent pages of the parent file name. GO terms with more than 100 children, with 2 or fewer genes annotated to the term, or a file size over 300KB are not shown. All heatmaps are generated with the same layout (see Figs. 3 and 4). [file 40170_2019_201_MOESM9_ESM.bz2 › Additional_File9_GOTermHeatmaps/Additional_File9_GOTermHeatmap/DNA_replication_initiation.pdf]
