# Supplementary material for: A yeast phenomic model for the influence of Warburg metabolism on genetic buffering of doxorubicin
Source: Cancer Metab. 2019 Oct 23;7:9. doi: 10.1186/s40170-019-0201-3 (PMC6806529; doi:10.1186/s40170-019-0201-3)
Supplement: Supplementary file 9 — Additional file 9. GO term-specific heatmaps for REMc/GTF-enriched clusters. GO term-specific heatmaps for significant GO process terms were generated as described in methods and Figs. 3 and 4. Any related child terms are presented in subsequent pages of the parent file name. GO terms with more than 100 children, with 2 or fewer genes annotated to the term, or a file size over 300KB are not shown. All heatmaps are generated with the same layout (see Figs. 3 and 4). [file 40170_2019_201_MOESM9_ESM.bz2 › Additional_File9_GOTermHeatmaps/Additional_File9_GOTermHeatmap/fatty_acid_biosynthetic_process.pdf]

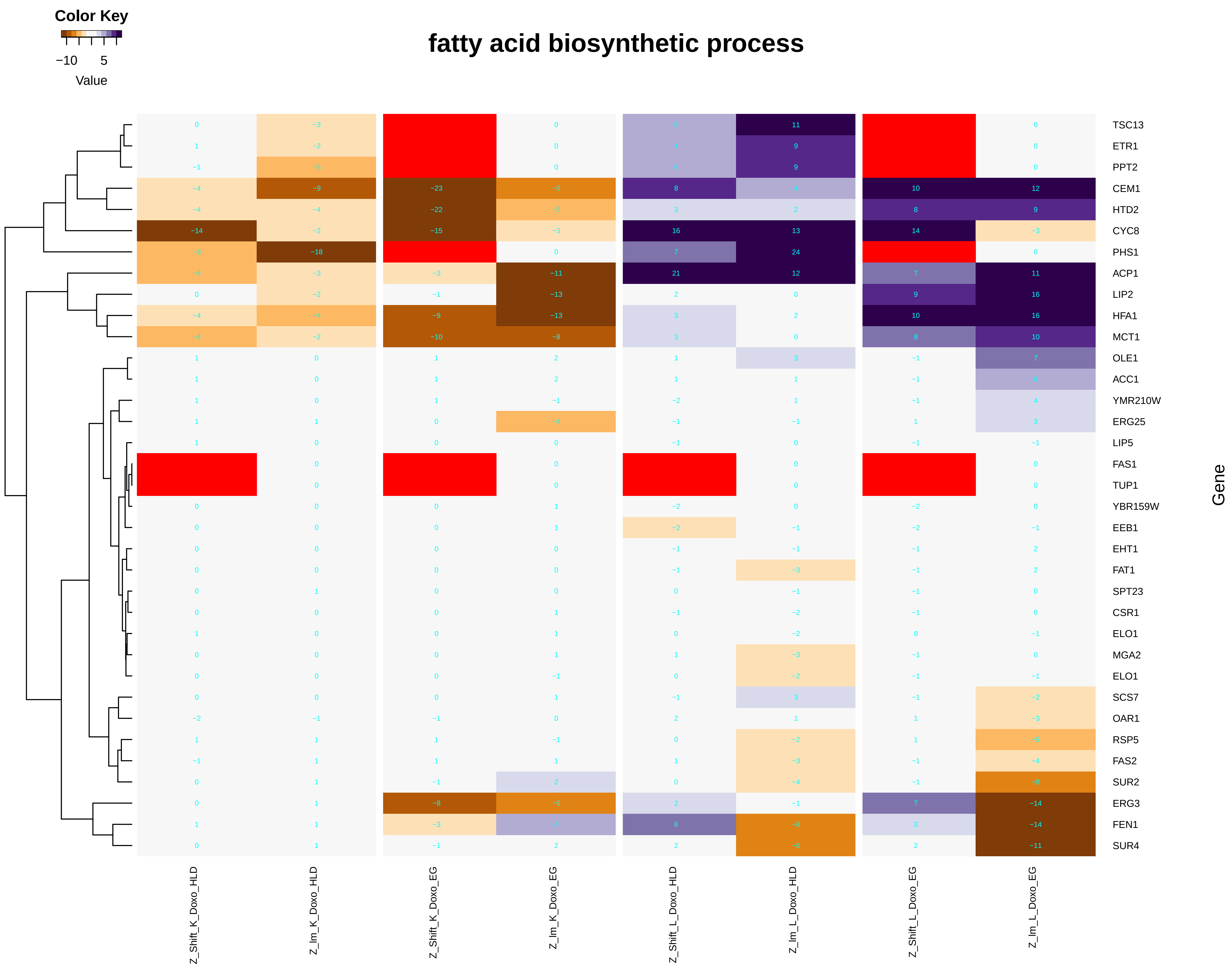
fatty acid biosynthetic process
Color Key
−10
5
Value
TSC13
ETR1
PPT2
CEM1
HTD2
CYC8
PHS1
ACP1
LIP2
HFA1
MCT1
OLE1
ACC1
YMR210W
ERG25
LIP5
FAS1
TUP1
YBR159W
EEB1
EHT1
FAT1
SPT23
CSR1
ELO1
MGA2
ELO1
SCS7
OAR1
RSP5
FAS2
SUR2
ERG3
FEN1
SUR4
Gene
Z_Shift_K_Doxo_HLD
Z_Im_K_Doxo_HLD
Z_Shift_K_Doxo_EG
Z_Im_K_Doxo_EG
Z_Shift_L_Doxo_HLD
Z_Im_L_Doxo_HLD
Z_Shift_L_Doxo_EG
Z_Im_L_Doxo_EG

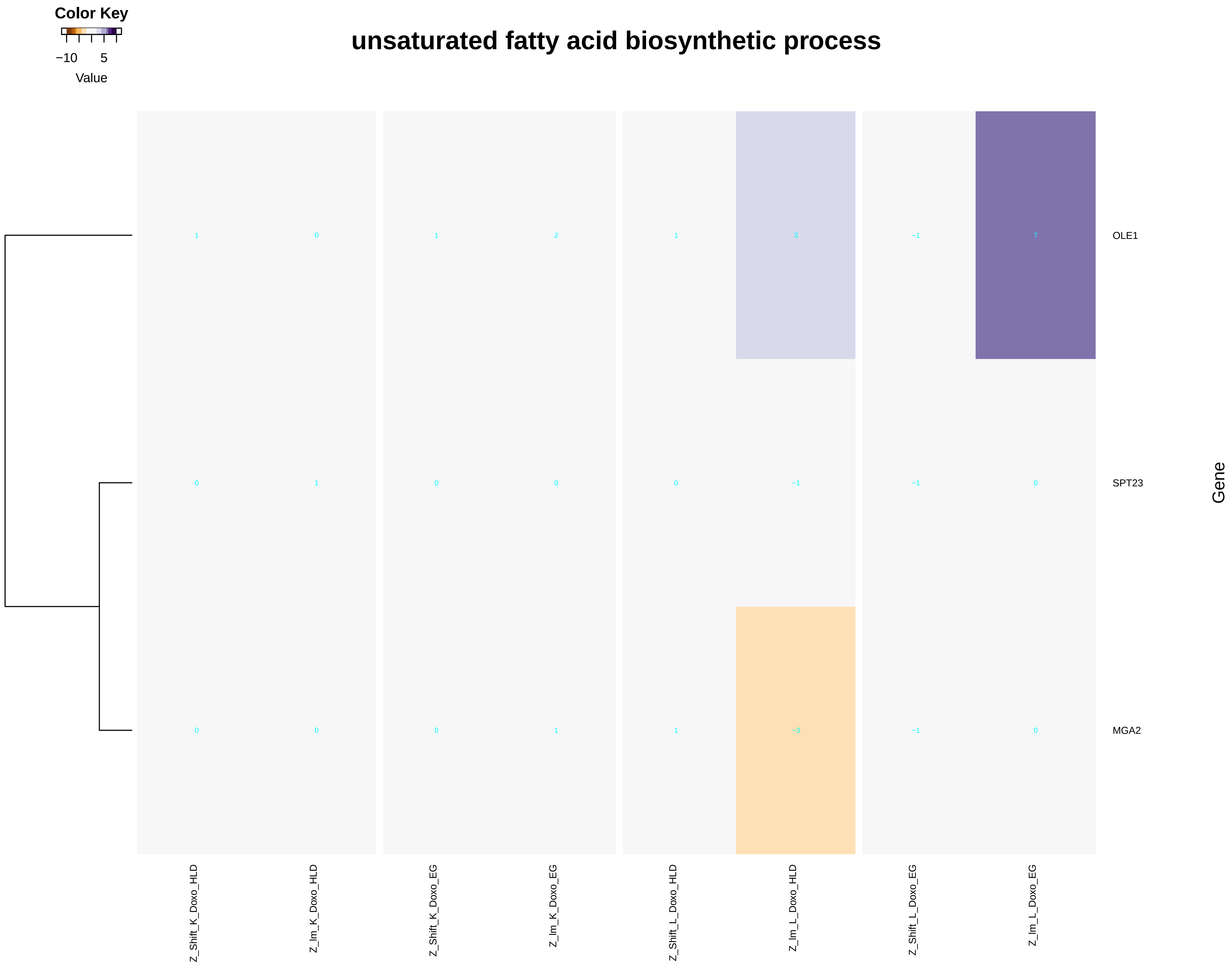

Color Key
−10
5
Value
unsaturated fatty acid biosynthetic process
1
0
1
2
1
3
−1
7
OLE1
0
1
0
0
0
−1
−1
0
SPT23
0
0
0
1
1
−3
−1
0
MGA2
Gene
Z_Shift_K_Doxo_HLD
Z_Im_K_Doxo_HLD
Z_Shift_K_Doxo_EG
Z_Im_K_Doxo_EG
Z_Shift_L_Doxo_HLD
Z_Im_L_Doxo_HLD
Z_Shift_L_Doxo_EG
Z_Im_L_Doxo_EG

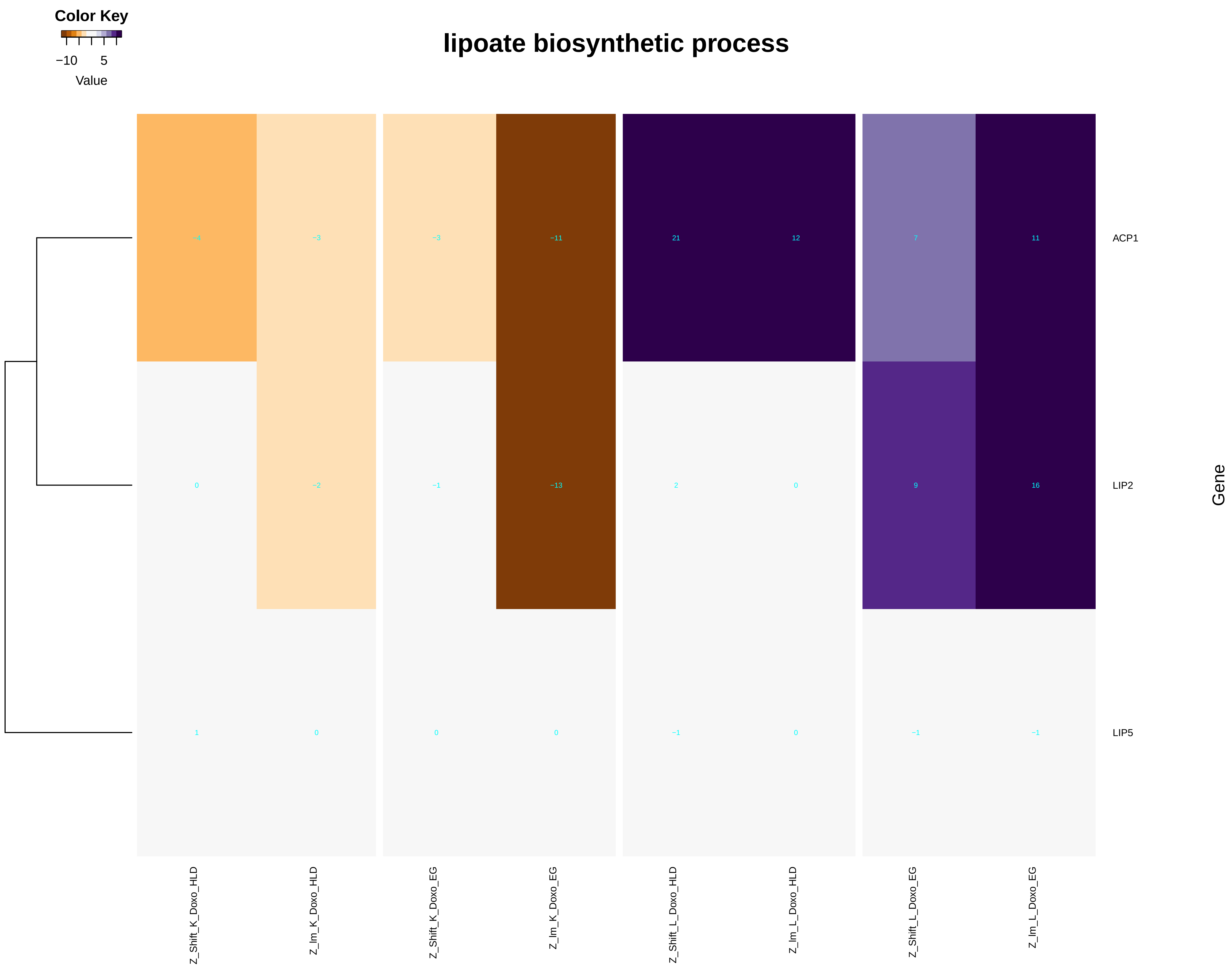

Color Key
−10
5
Value
lipoate biosynthetic process
−4
−3
−3
−11
21
12
7
11
ACP1
0
−2
−1
−13
2
0
9
16
LIP2
1
0
0
0
−1
0
−1
−1
LIP5
Gene
Z_Shift_K_Doxo_HLD
Z_lm_K_Doxo_HLD
Z_Shift_K_Doxo_EG
Z_lm_K_Doxo_EG
Z_Shift_L_Doxo_HLD
Z_lm_L_Doxo_HLD
Z_Shift_L_Doxo_EG
Z_lm_L_Doxo_EG

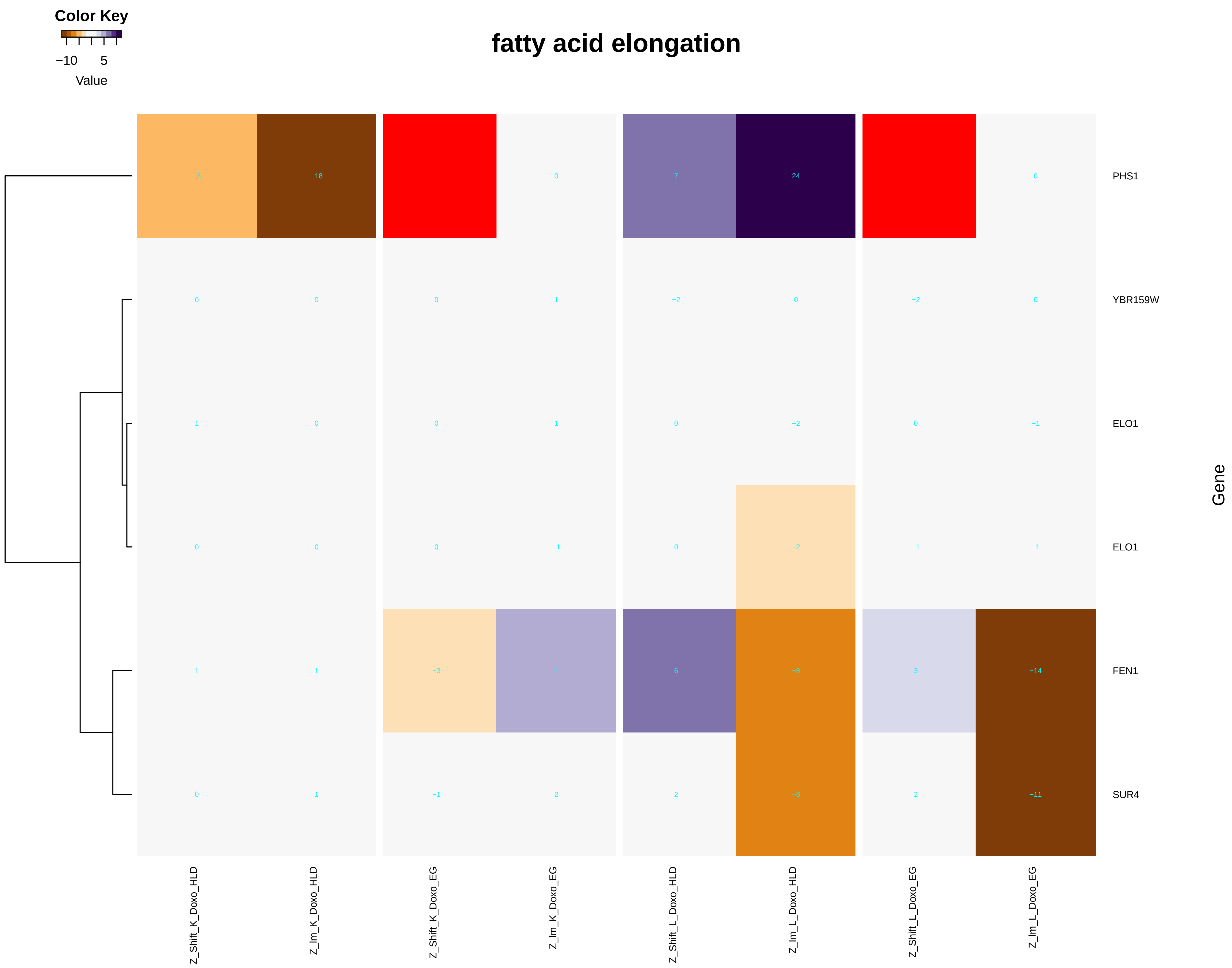
fatty acid elongation
Color Key
−10 5
Value
PHS1
YBR159W
ELO1
ELO1
FEN1
SUR4
Gene
Z_Shift_K_Doxo_HLD
Z_Im_K_Doxo_HLD
Z_Shift_K_Doxo_EG
Z_Im_K_Doxo_EG
Z_Shift_L_Doxo_HLD
Z_Im_L_Doxo_HLD
Z_Shift_L_Doxo_EG
Z_Im_L_Doxo_EG
−6 −18 0 7 24 0
0 0 0 1 −2 0 −2 0
1 0 0 1 0 −2 0 −1
0 0 0 −1 0 −2 −1 −1
1 1 −3 6 −8 3 −14
0 1 −1 2 2 −6 2 −11

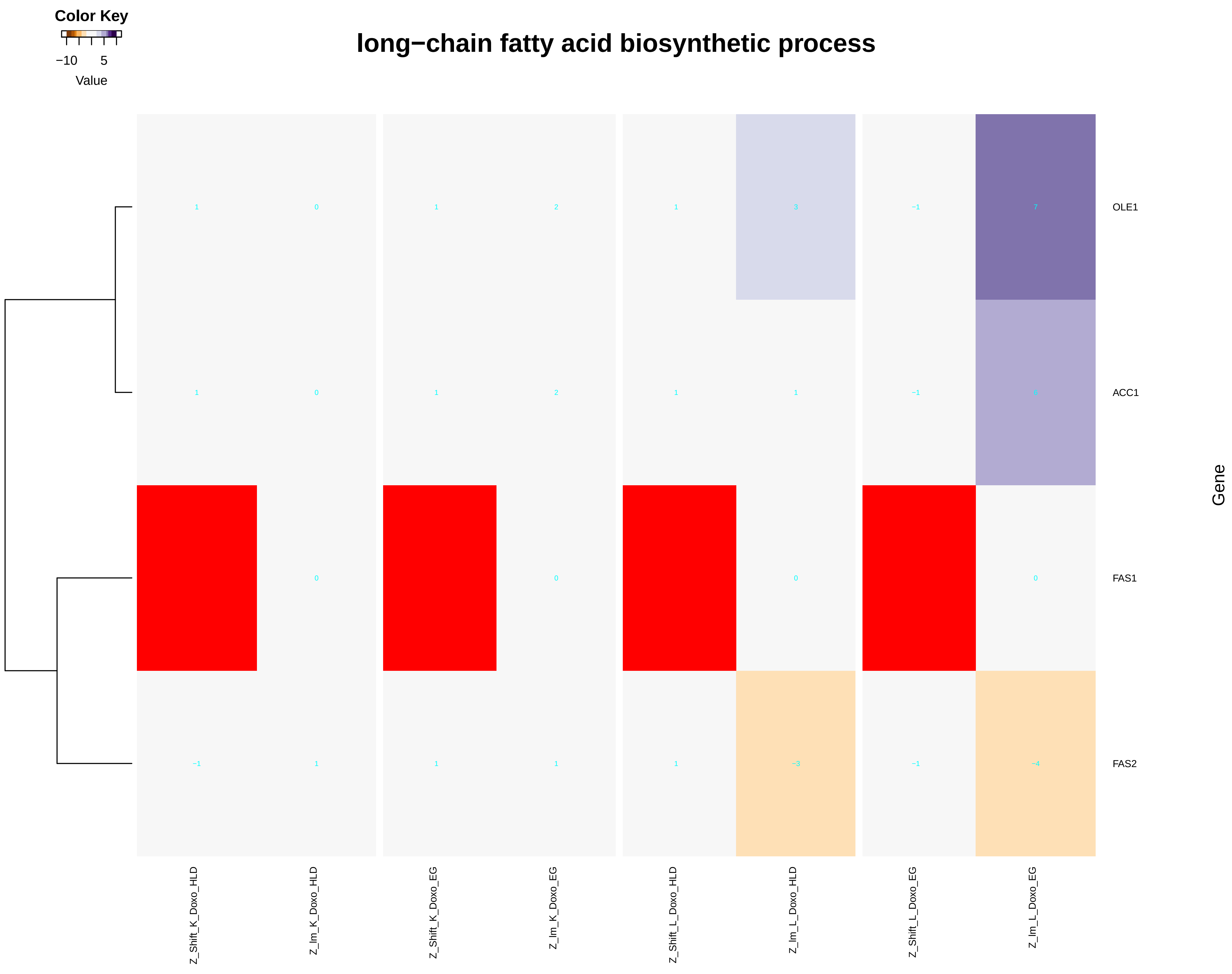
Color Key
−10
5
Value
long−chain fatty acid biosynthetic process
1
0
1
2
1
3
−1
7
OLE1
1
0
1
2
1
1
−1
ACC1
0
0
0
0
FAS1
−1
1
1
1
1
−3
−1
−4
FAS2
Gene
Z_Shift_K_Doxo_HLD
Z_Im_K_Doxo_HLD
Z_Shift_K_Doxo_EG
Z_Im_K_Doxo_EG
Z_Shift_L_Doxo_HLD
Z_Im_L_Doxo_HLD
Z_Shift_L_Doxo_EG
Z_Im_L_Doxo_EG

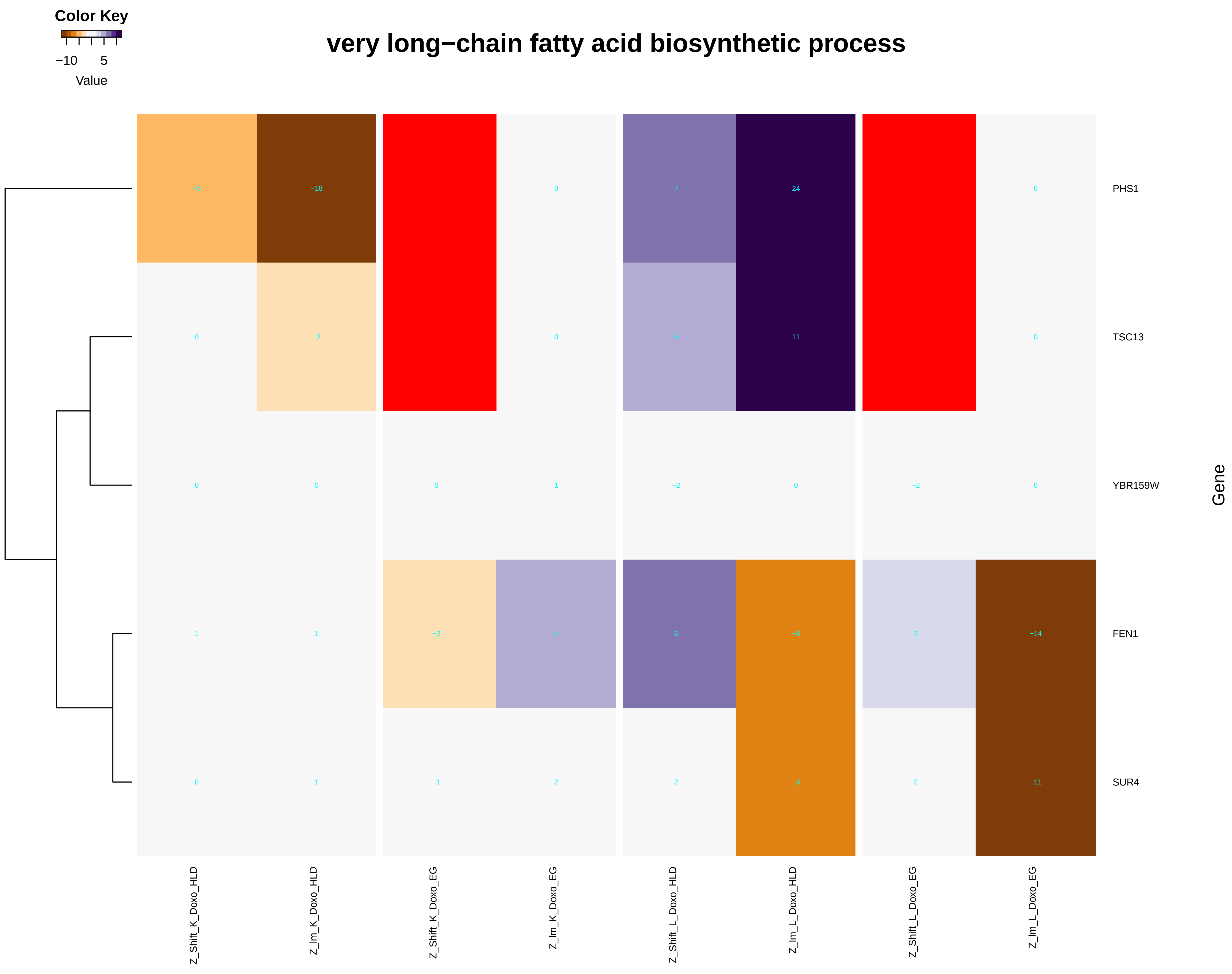
Color Key
−10
5
Value
very long−chain fatty acid biosynthetic process
PHS1
TSC13
YBR159W
FEN1
SUR4
Gene
Z_Shift_K_Doxo_HLD
Z_lm_K_Doxo_HLD
Z_Shift_K_Doxo_EG
Z_lm_K_Doxo_EG
Z_Shift_L_Doxo_HLD
Z_lm_L_Doxo_HLD
Z_Shift_L_Doxo_EG
Z_lm_L_Doxo_EG

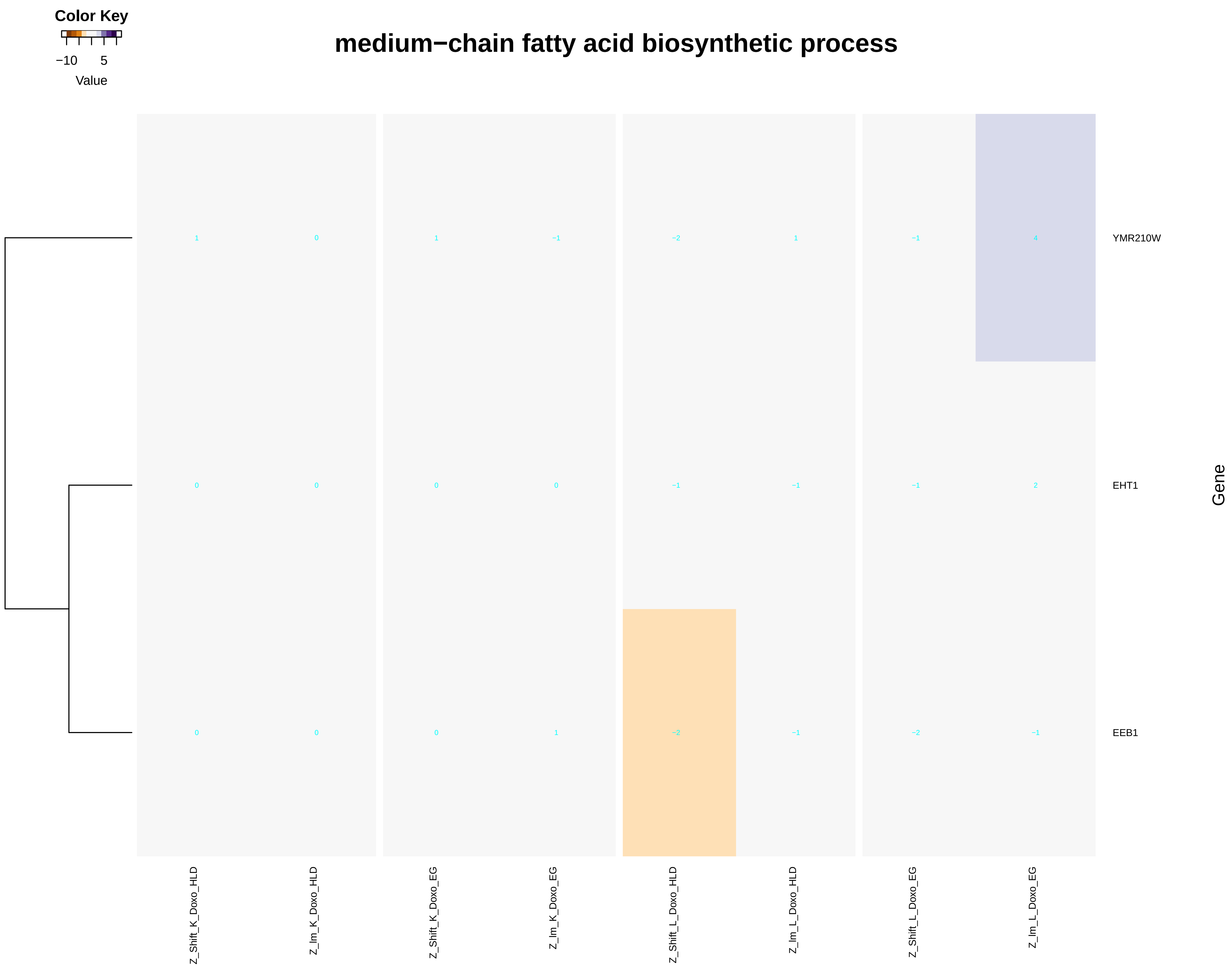
Color Key
−10
5
Value
medium−chain fatty acid biosynthetic process
1
0
1
−1
−2
1
−1
4
YMR210W
0
0
0
0
−1
−1
−1
2
EHT1
0
0
0
1
−2
−1
−2
−1
EEB1
Gene
Z_Shift_K_Doxo_HLD
Z_lm_K_Doxo_HLD
Z_Shift_K_Doxo_EG
Z_lm_K_Doxo_EG
Z_Shift_L_Doxo_HLD
Z_lm_L_Doxo_HLD
Z_Shift_L_Doxo_EG
Z_lm_L_Doxo_EG
