# Supplementary material for: A yeast phenomic model for the influence of Warburg metabolism on genetic buffering of doxorubicin
Source: Cancer Metab. 2019 Oct 23;7:9. doi: 10.1186/s40170-019-0201-3 (PMC6806529; doi:10.1186/s40170-019-0201-3)
Supplement: Supplementary file 9 — Additional file 9. GO term-specific heatmaps for REMc/GTF-enriched clusters. GO term-specific heatmaps for significant GO process terms were generated as described in methods and Figs. 3 and 4. Any related child terms are presented in subsequent pages of the parent file name. GO terms with more than 100 children, with 2 or fewer genes annotated to the term, or a file size over 300KB are not shown. All heatmaps are generated with the same layout (see Figs. 3 and 4). [file 40170_2019_201_MOESM9_ESM.bz2 › Additional_File9_GOTermHeatmaps/Additional_File9_GOTermHeatmap/histone_methylation.pdf]

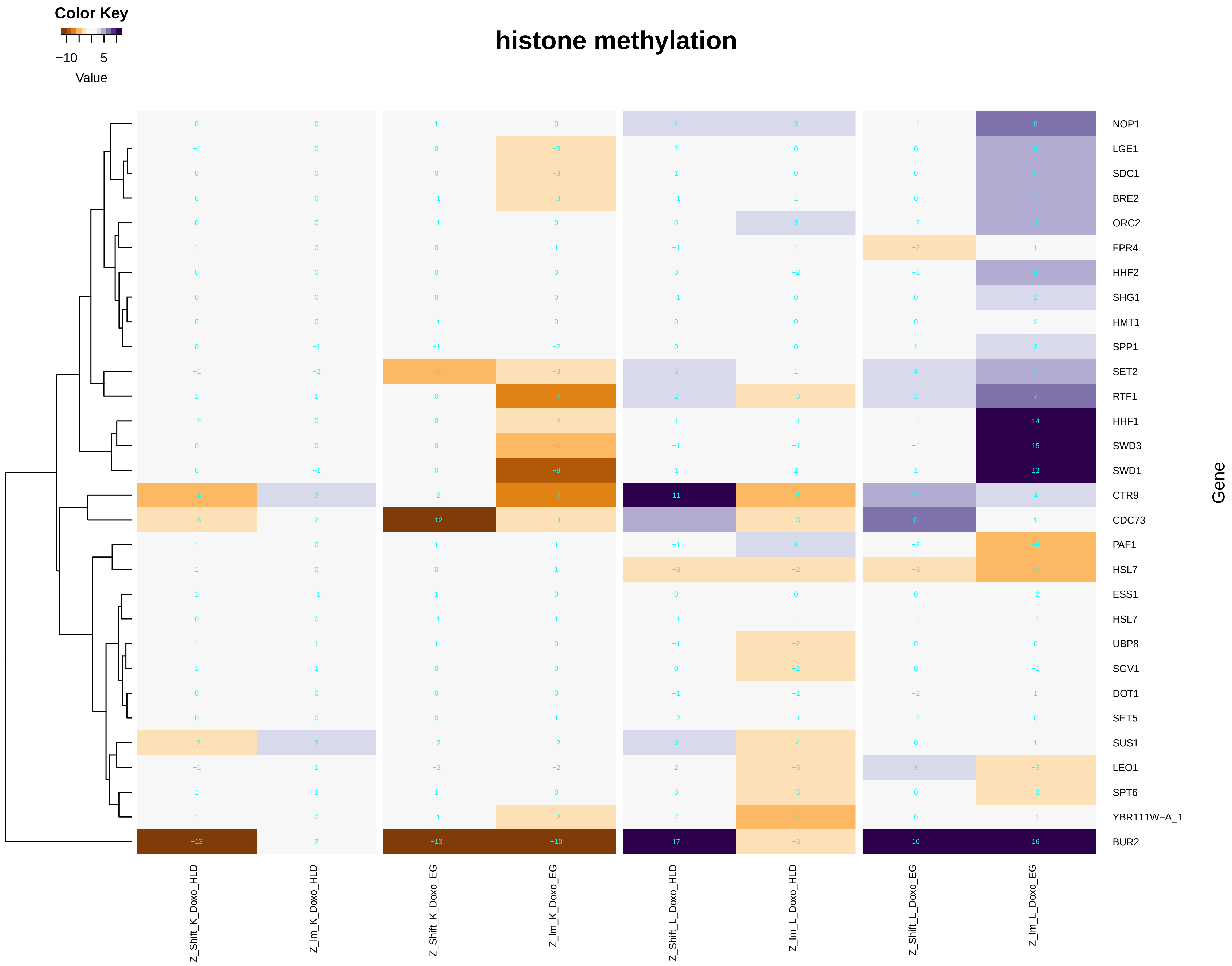
histone methylation
Color Key
−10
5
Value
NOP1
LGE1
SDC1
BRE2
ORC2
FPR4
HHF2
SHG1
HMT1
SPP1
SET2
RTF1
HHF1
SWD3
SWD1
CTR9
CDC73
PAF1
HSL7
ESS1
HSL7
UBP8
SGV1
DOT1
SET5
SUS1
LEO1
SPT6
YBR111W−A_1
BUR2
Gene
Z_Shift_K_Doxo_HLD
Z_Im_K_Doxo_HLD
Z_Shift_K_Doxo_EG
Z_Im_K_Doxo_EG
Z_Shift_L_Doxo_HLD
Z_Im_L_Doxo_HLD
Z_Shift_L_Doxo_EG
Z_Im_L_Doxo_EG

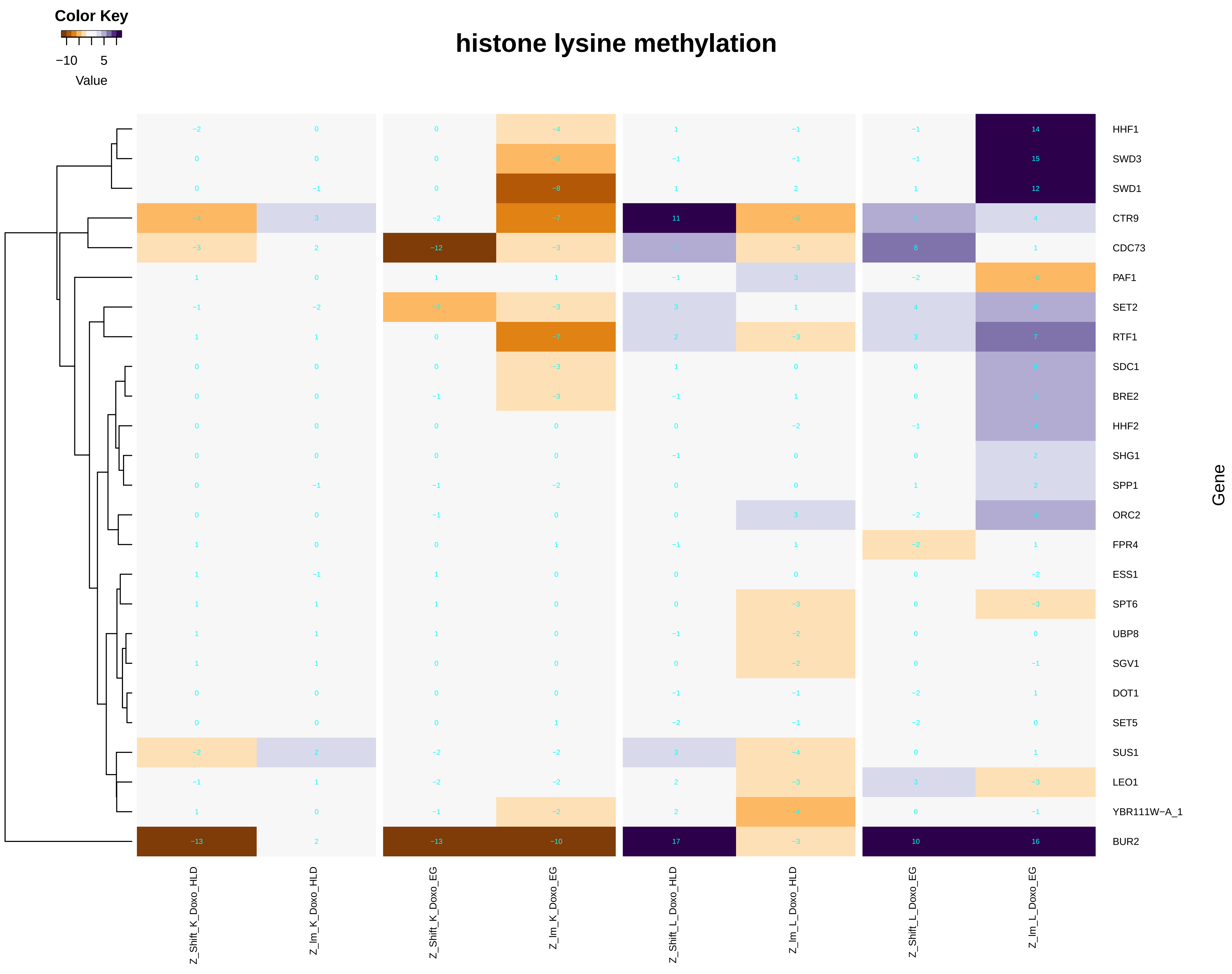
Color Key
−10
5
Value
histone lysine methylation
HHF1
SWD3
SWD1
CTR9
CDC73
PAF1
SET2
RTF1
SDC1
BRE2
HHF2
SHG1
SPP1
ORC2
FPR4
ESS1
SPT6
UBP8
SGV1
DOT1
SET5
SUS1
LEO1
YBR111W−A_1
BUR2
Gene
Z_Shift_K_Doxo_HLD
Z_Im_K_Doxo_HLD
Z_Shift_K_Doxo_EG
Z_Im_K_Doxo_EG
Z_Shift_L_Doxo_HLD
Z_Im_L_Doxo_HLD
Z_Shift_L_Doxo_EG
Z_Im_L_Doxo_EG

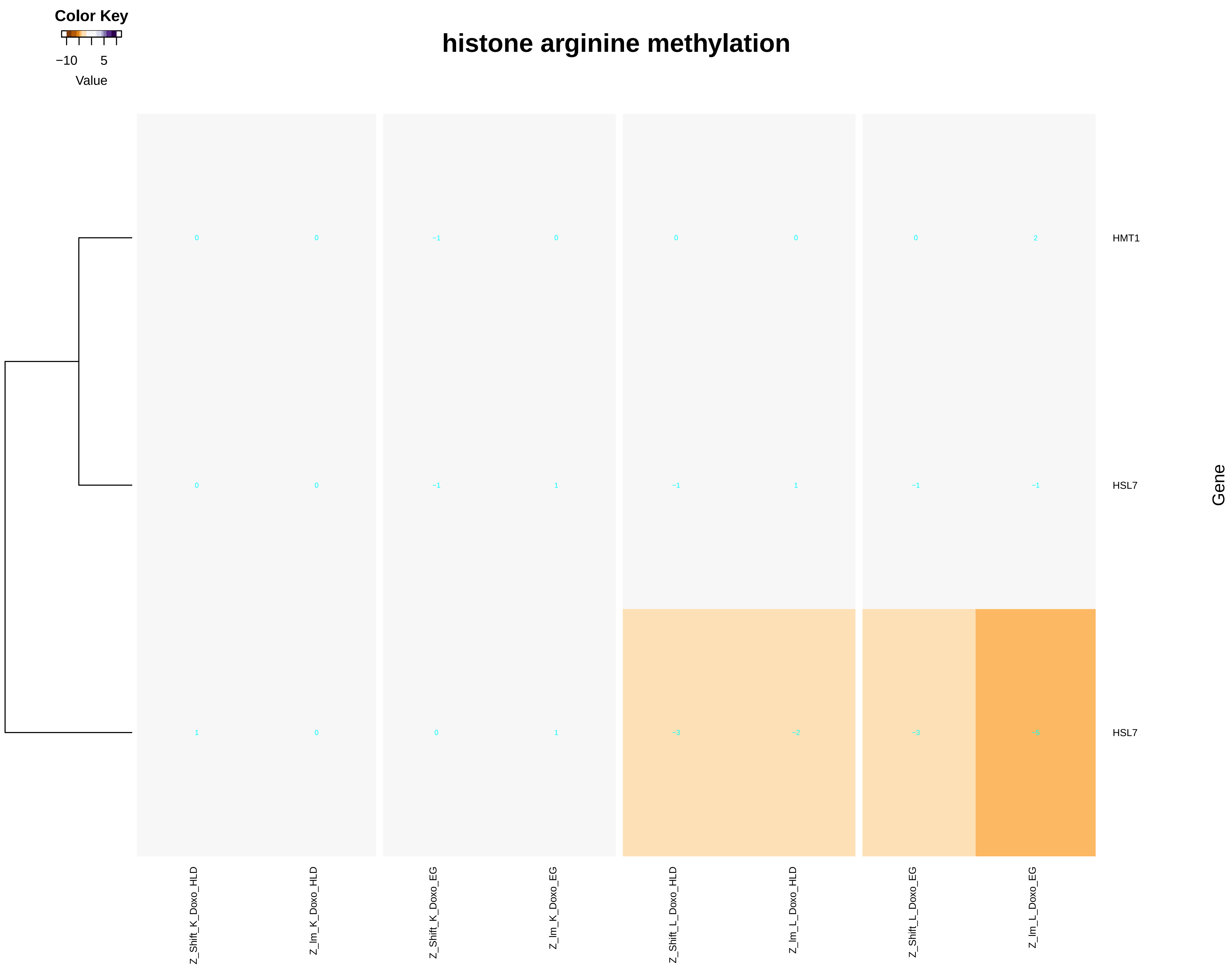
Color Key
−10
5
Value
histone arginine methylation
0
0
−1
0
0
0
0
2
HMT1
0
0
−1
1
−1
1
−1
−1
HSL7
1
0
0
1
−3
−2
−3
−5
HSL7
Gene
Z_Shift_K_Doxo_HLD
Z_Im_K_Doxo_HLD
Z_Shift_K_Doxo_EG
Z_Im_K_Doxo_EG
Z_Shift_L_Doxo_HLD
Z_Im_L_Doxo_HLD
Z_Shift_L_Doxo_EG
Z_Im_L_Doxo_EG

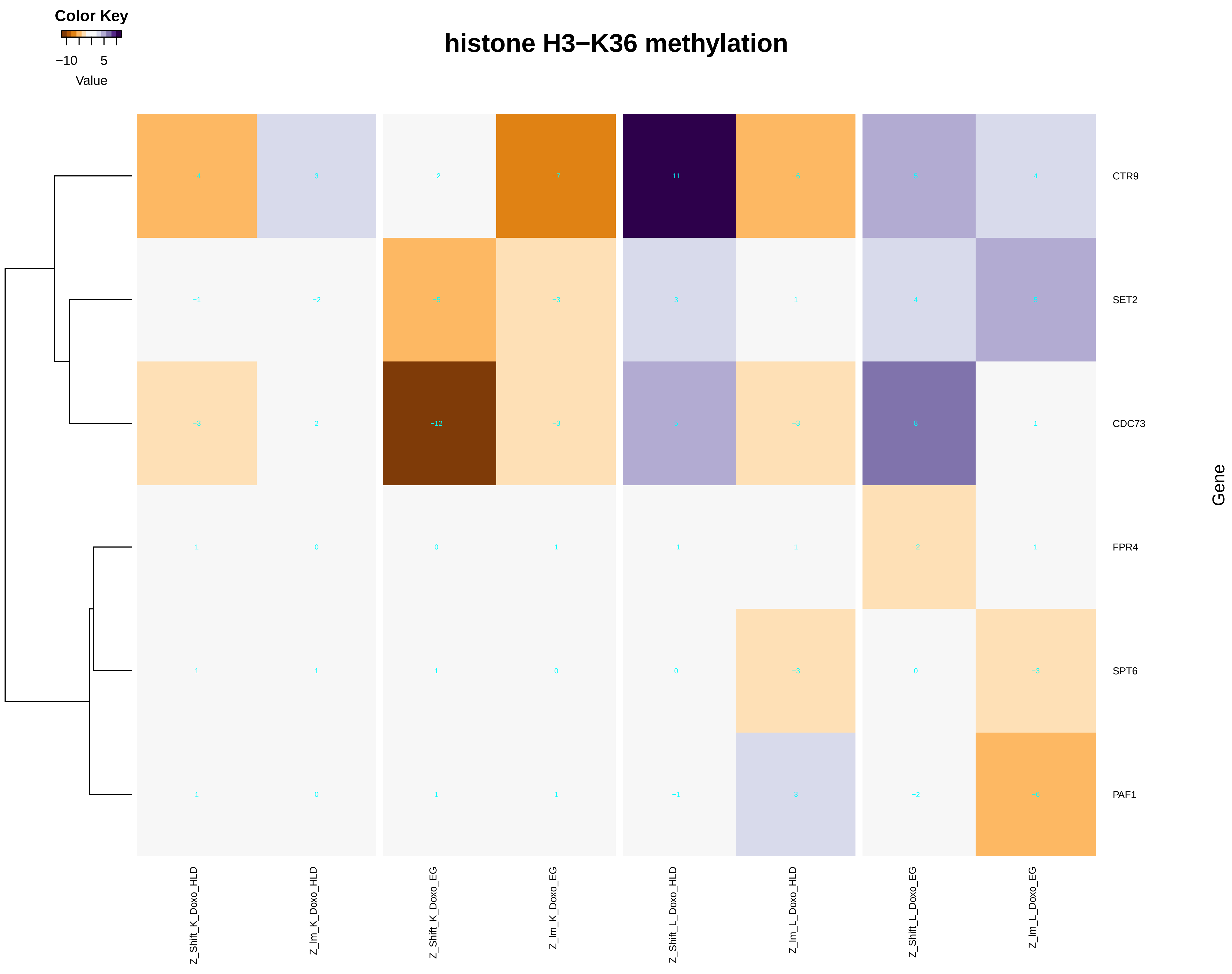

Color Key
−10
5
Value
histone H3−K36 methylation
CTR9
SET2
CDC73
FPR4
SPT6
PAF1
Gene
Z_Shift_K_Doxo_HLD
Z_Im_K_Doxo_HLD
Z_Shift_K_Doxo_EG
Z_Im_K_Doxo_EG
Z_Shift_L_Doxo_HLD
Z_Im_L_Doxo_HLD
Z_Shift_L_Doxo_EG
Z_Im_L_Doxo_EG

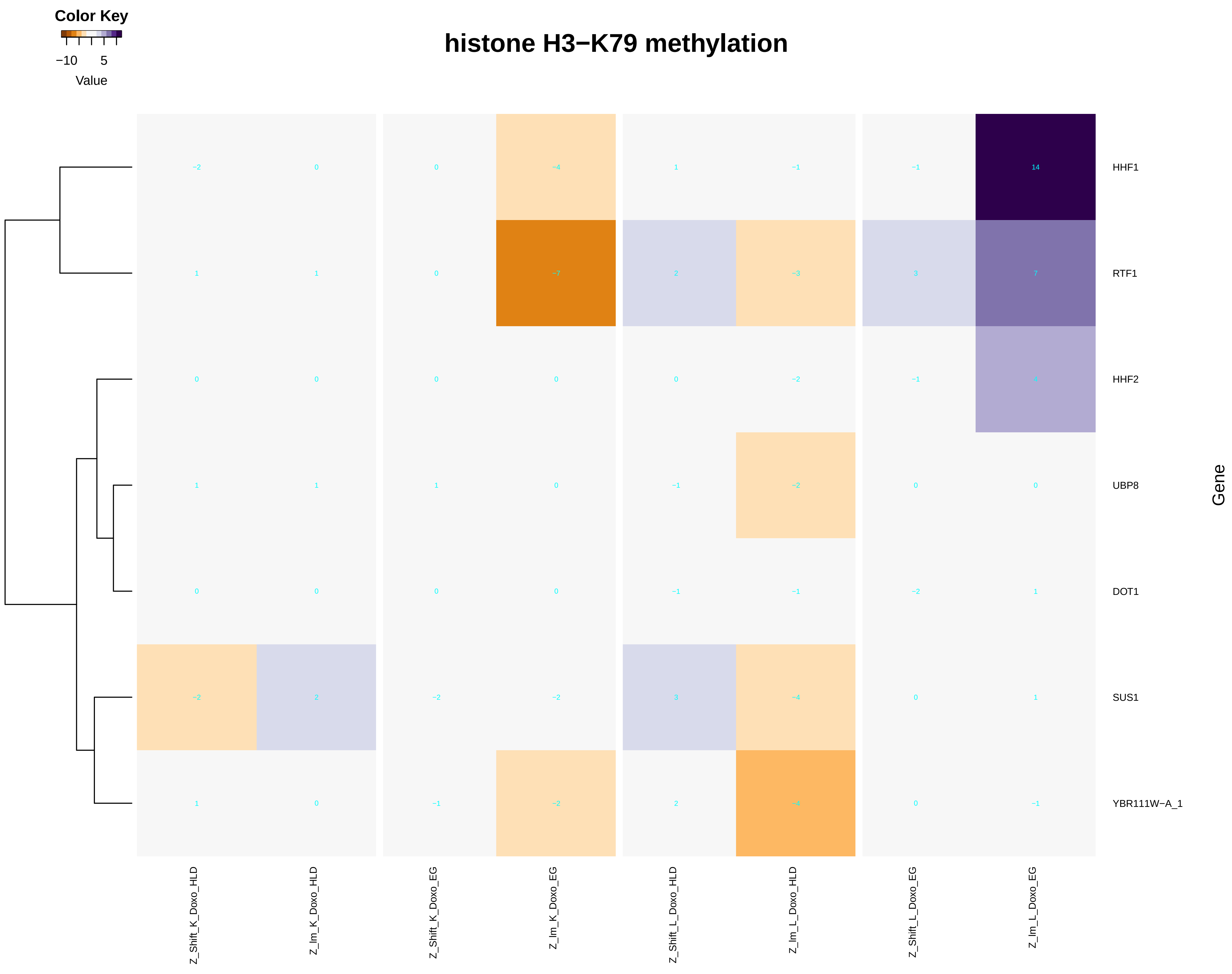
Color Key
−10
5
Value
histone H3−K79 methylation
HHF1
RTF1
HHF2
UBP8
DOT1
SUS1
YBR111W−A_1
Gene
Z_Shift_K_Doxo_HLD
Z_Im_K_Doxo_HLD
Z_Shift_K_Doxo_EG
Z_Im_K_Doxo_EG
Z_Shift_L_Doxo_HLD
Z_Im_L_Doxo_HLD
Z_Shift_L_Doxo_EG
Z_Im_L_Doxo_EG
−2 0 0 −4 1 −1 −1 14
1 1 0 −7 2 −3 3 7
0 0 0 0 0 −2 −1
1 1 1 0 −1 −2 0 0
0 0 0 0 −1 −1 −2 1
−2 2 −2 −2 3 −4 0 1
1 0 −1 −2 2 0 −1

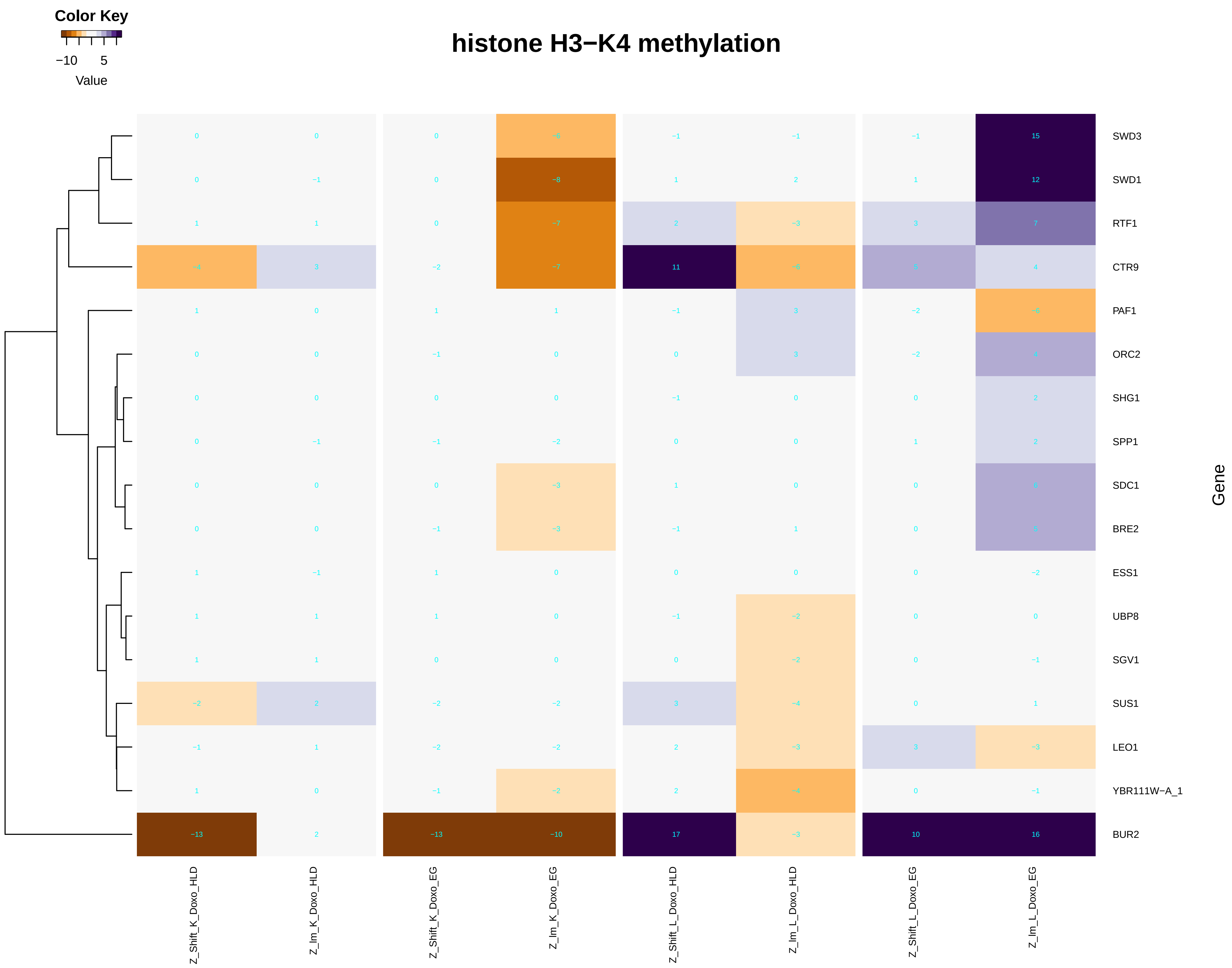
histone H3−K4 methylation
Color Key
−10
5
Value
SWD3
SWD1
RTF1
CTR9
PAF1
ORC2
SHG1
SPP1
SDC1
BRE2
ESS1
UBP8
SGV1
SUS1
LEO1
YBR111W−A_1
BUR2
Gene
Z_Shift_K_Doxo_HLD
Z_Im_K_Doxo_HLD
Z_Shift_K_Doxo_EG
Z_Im_K_Doxo_EG
Z_Shift_L_Doxo_HLD
Z_Im_L_Doxo_HLD
Z_Shift_L_Doxo_EG
Z_Im_L_Doxo_EG

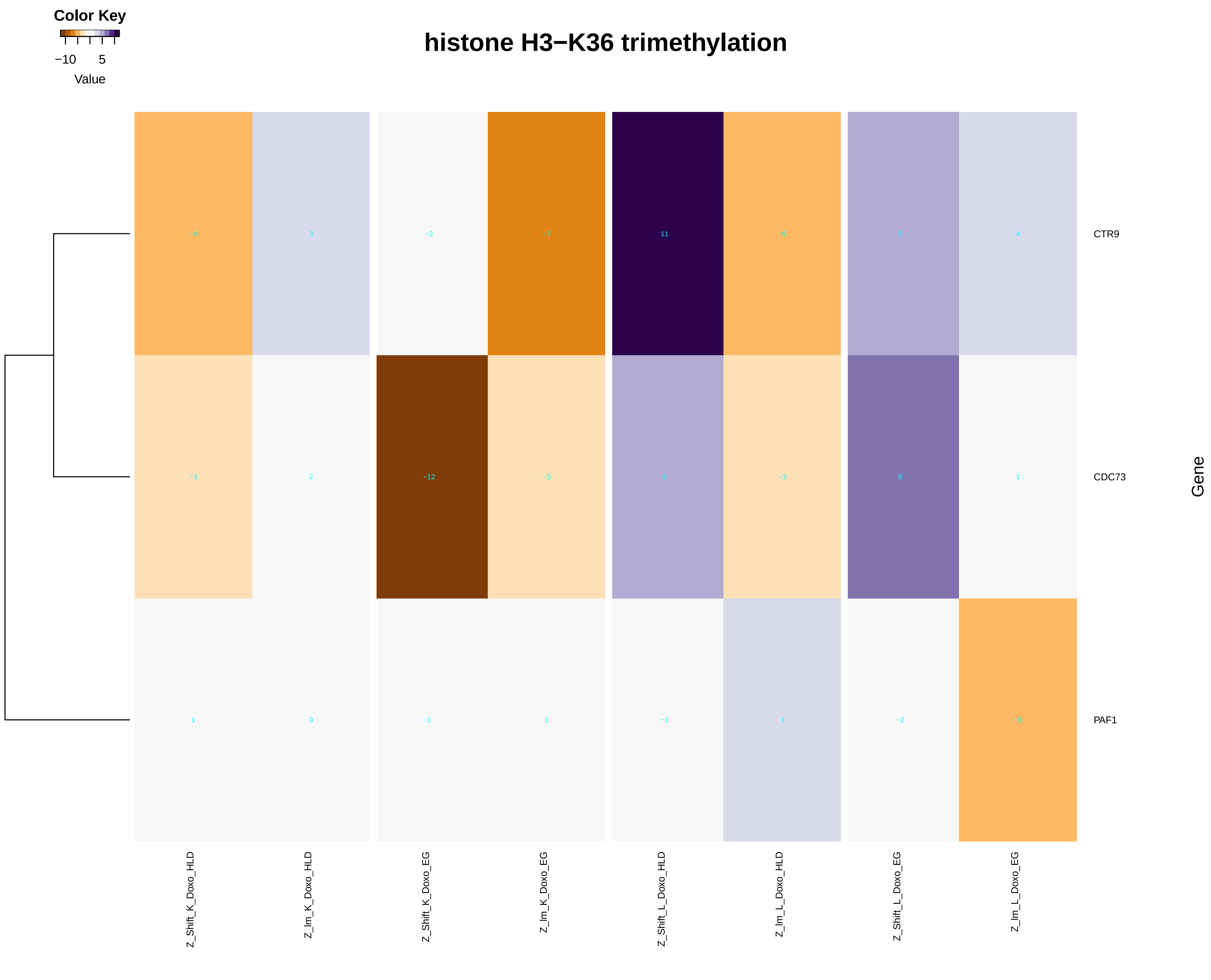
Color Key
−10
5
Value
histone H3−K36 trimethylation
CTR9
CDC73
PAF1
Gene
Z_Shift_K_Doxo_HLD
Z_Im_K_Doxo_HLD
Z_Shift_K_Doxo_EG
Z_Im_K_Doxo_EG
Z_Shift_L_Doxo_HLD
Z_Im_L_Doxo_HLD
Z_Shift_L_Doxo_EG
Z_Im_L_Doxo_EG

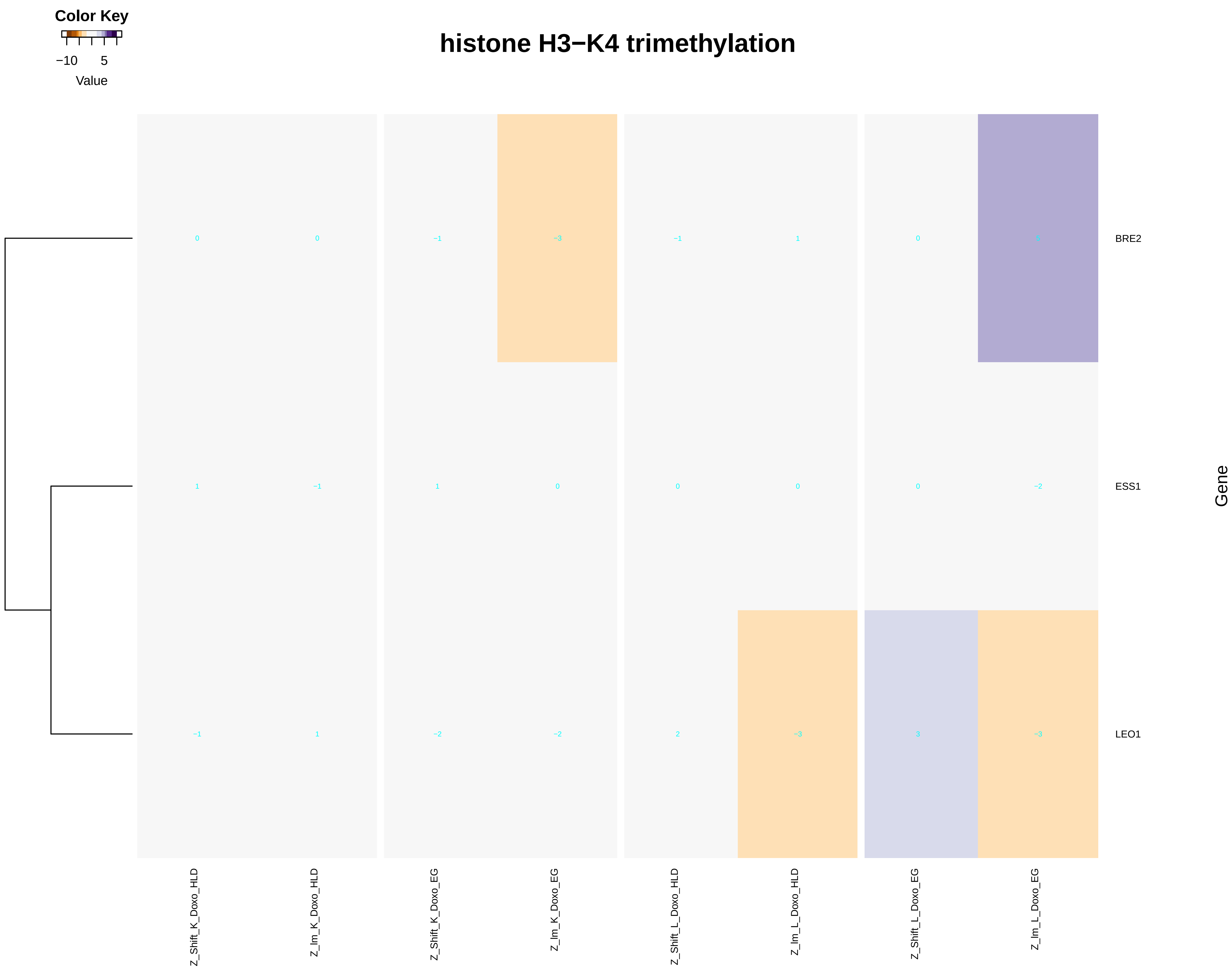
histone H3−K4 trimethylation
Color Key
−10
5
Value
BRE2
ESS1
LEO1
Gene
Z_Shift_K_Doxo_HLD
Z_Im_K_Doxo_HLD
Z_Shift_K_Doxo_EG
Z_Im_K_Doxo_EG
Z_Shift_L_Doxo_HLD
Z_Im_L_Doxo_HLD
Z_Shift_L_Doxo_EG
Z_Im_L_Doxo_EG
0 0 −1 −3 −1 1 0 5
1 −1 1 0 0 0 0 −2
−1 1 −2 −2 2 −3 3 −3
