# Supplementary material for: A yeast phenomic model for the influence of Warburg metabolism on genetic buffering of doxorubicin
Source: Cancer Metab. 2019 Oct 23;7:9. doi: 10.1186/s40170-019-0201-3 (PMC6806529; doi:10.1186/s40170-019-0201-3)
Supplement: Supplementary file 9 — Additional file 9. GO term-specific heatmaps for REMc/GTF-enriched clusters. GO term-specific heatmaps for significant GO process terms were generated as described in methods and Figs. 3 and 4. Any related child terms are presented in subsequent pages of the parent file name. GO terms with more than 100 children, with 2 or fewer genes annotated to the term, or a file size over 300KB are not shown. All heatmaps are generated with the same layout (see Figs. 3 and 4). [file 40170_2019_201_MOESM9_ESM.bz2 › Additional_File9_GOTermHeatmaps/Additional_File9_GOTermHeatmap/internal_protein_amino_acid_acetylation.pdf]

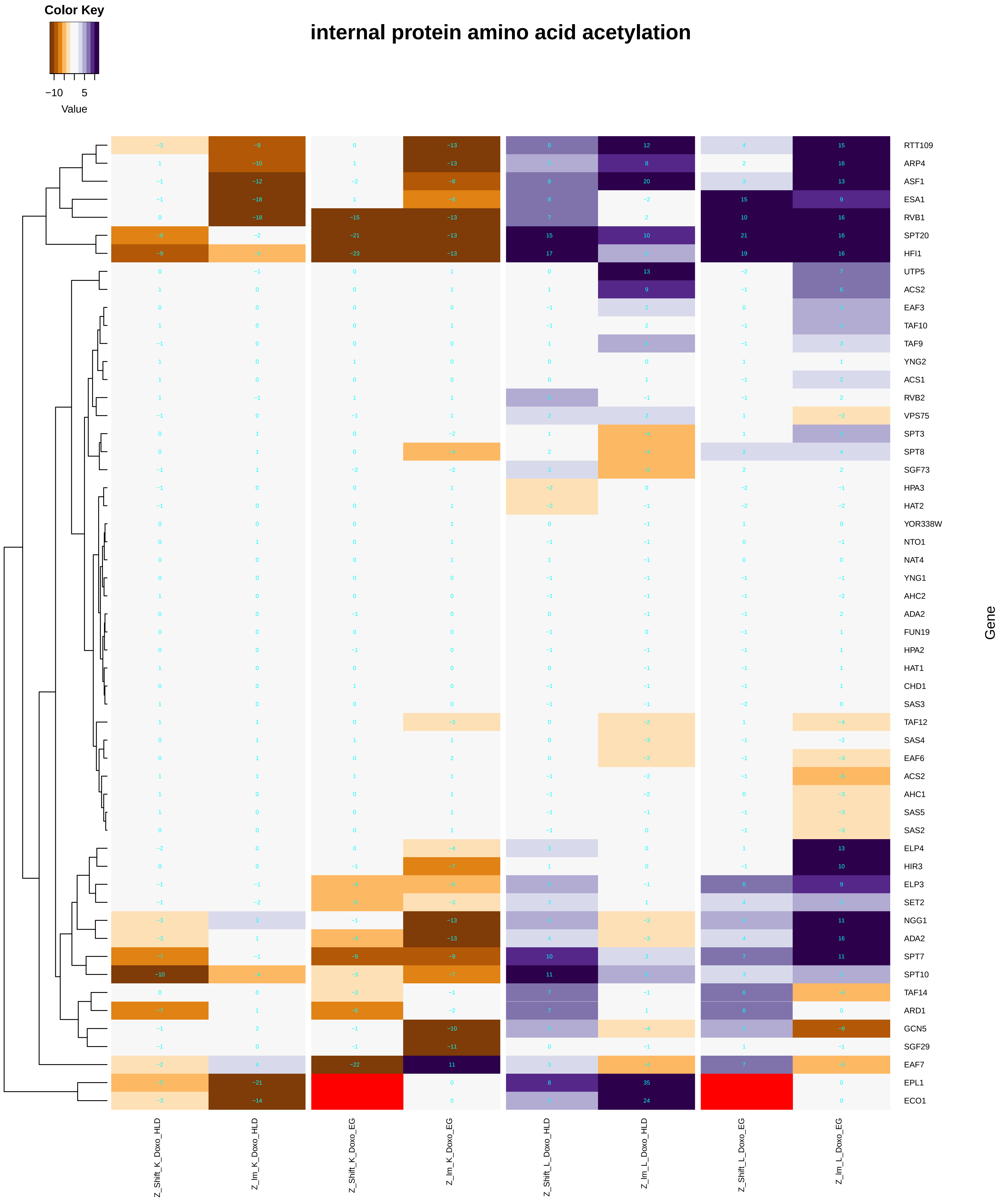

Color Key
−10
5
Value
internal protein amino acid acetylation
RTT109
ARP4
ASF1
ESA1
RVB1
SPT20
HFI1
UTP5
ACS2
EAF3
TAF10
TAF9
YNG2
ACS1
RVB2
VPS75
SPT3
SPT8
SGF73
HPA3
HAT2
YOR338W
NTO1
NAT4
YNG1
AHC2
ADA2
FUN19
HPA2
HAT1
CHD1
SAS3
TAF12
SAS4
EAF6
ACS2
AHC1
SAS5
SAS2
ELP4
HIR3
ELP3
SET2
NGG1
ADA2
SPT7
SPT10
TAF14
ARD1
GCN5
SGF29
EAF7
EPL1
ECO1
Gene
Z_Shift_K_Doxo_HLD
Z_Im_K_Doxo_HLD
Z_Shift_K_Doxo_EG
Z_Im_K_Doxo_EG
Z_Shift_L_Doxo_HLD
Z_Im_L_Doxo_HLD
Z_Shift_L_Doxo_EG
Z_Im_L_Doxo_EG

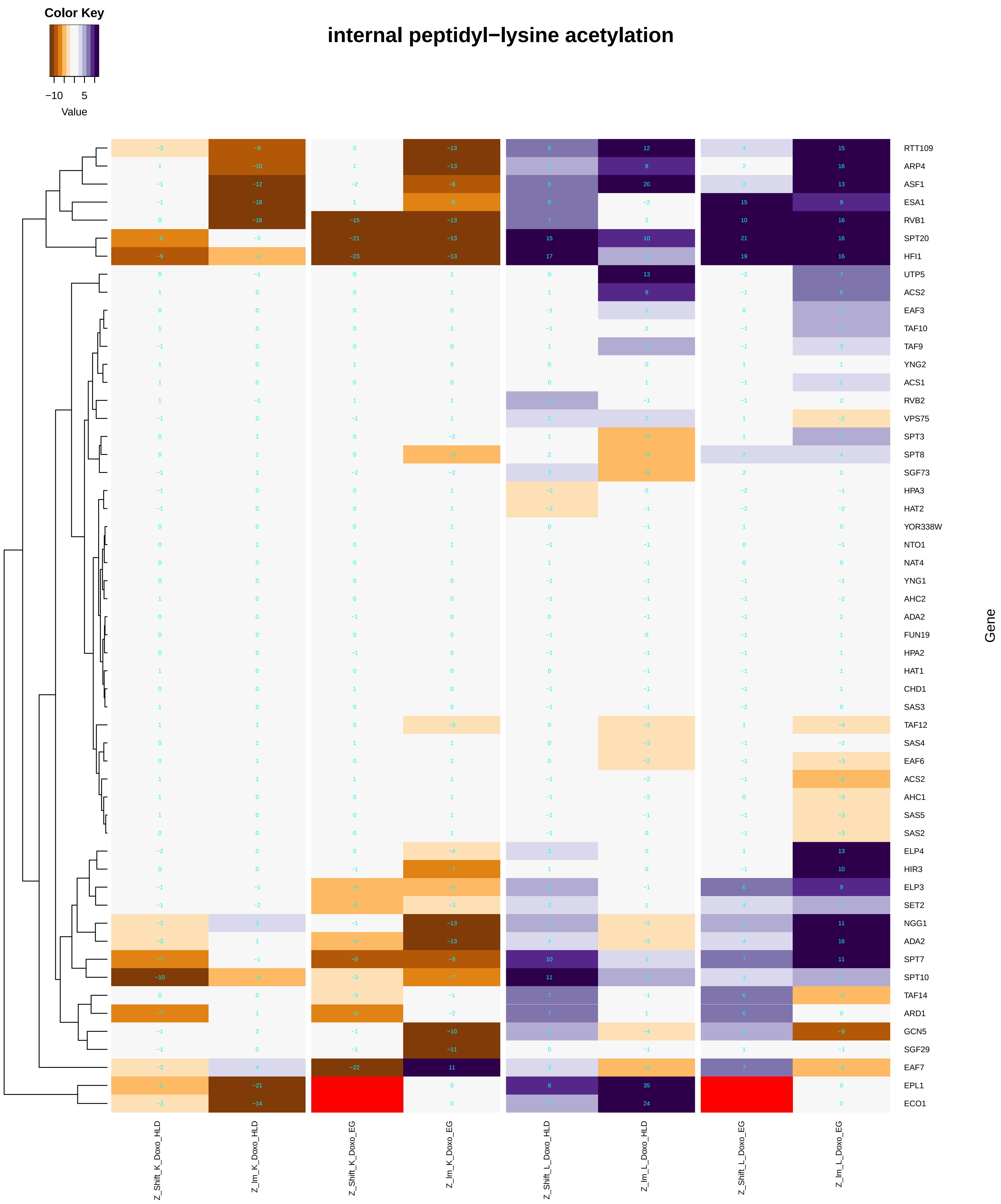
internal peptidyl−lysine acetylation
Color Key
−10
5
Value
RTT109
ARP4
ASF1
ESA1
RVB1
SPT20
HFI1
UTP5
ACS2
EAF3
TAF10
TAF9
YNG2
ACS1
RVB2
VPS75
SPT3
SPT8
SGF73
HPA3
HAT2
YOR338W
NTO1
NAT4
YNG1
AHC2
ADA2
FUN19
HPA2
HAT1
CHD1
SAS3
TAF12
SAS4
EAF6
ACS2
AHC1
SAS5
SAS2
ELP4
HIR3
ELP3
SET2
NGG1
ADA2
SPT7
SPT10
TAF14
ARD1
GCN5
SGF29
EAF7
EPL1
ECO1
Gene
Z_Shift_K_Doxo_HLD
Z_Im_K_Doxo_HLD
Z_Shift_K_Doxo_EG
Z_Im_K_Doxo_EG
Z_Shift_L_Doxo_HLD
Z_Im_L_Doxo_HLD
Z_Shift_L_Doxo_EG
Z_Im_L_Doxo_EG

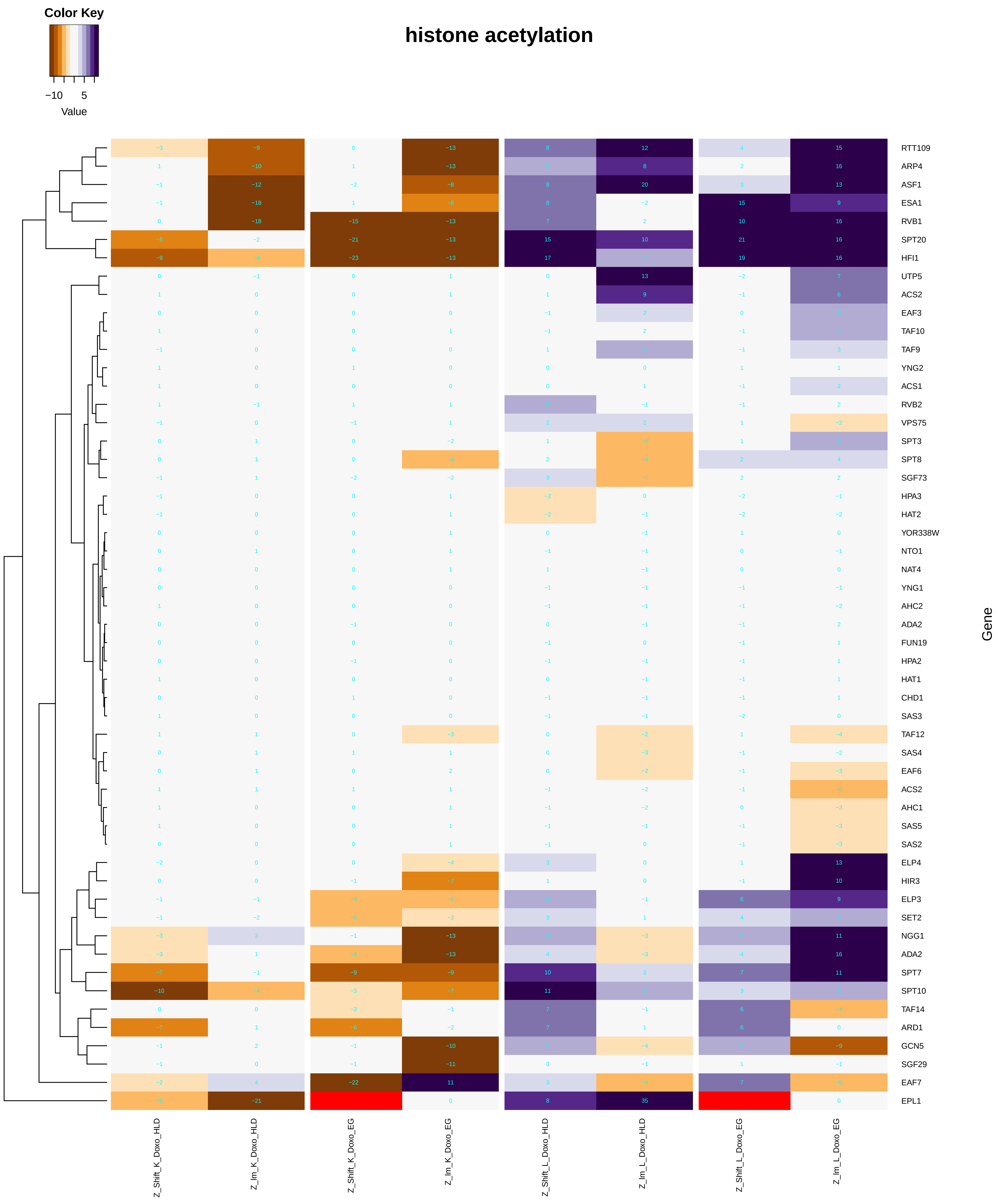

histone acetylation
Color Key
−10
5
Value
RTT109
ARP4
ASF1
ESA1
RVB1
SPT20
HFI1
UTP5
ACS2
EAF3
TAF10
TAF9
YNG2
ACS1
RVB2
VPS75
SPT3
SPT8
SGF73
HPA3
HAT2
YOR338W
NTO1
NAT4
YNG1
AHC2
ADA2
FUN19
HPA2
HAT1
CHD1
SAS3
TAF12
SAS4
EAF6
ACS2
AHC1
SAS5
SAS2
ELP4
HIR3
ELP3
SET2
NGG1
ADA2
SPT7
SPT10
TAF14
ARD1
GCN5
SGF29
EAF7
EPL1
Gene
Z_Shift_K_Doxo_HLD
Z_Im_K_Doxo_HLD
Z_Shift_K_Doxo_EG
Z_Im_K_Doxo_EG
Z_Shift_L_Doxo_HLD
Z_Im_L_Doxo_HLD
Z_Shift_L_Doxo_EG
Z_Im_L_Doxo_EG

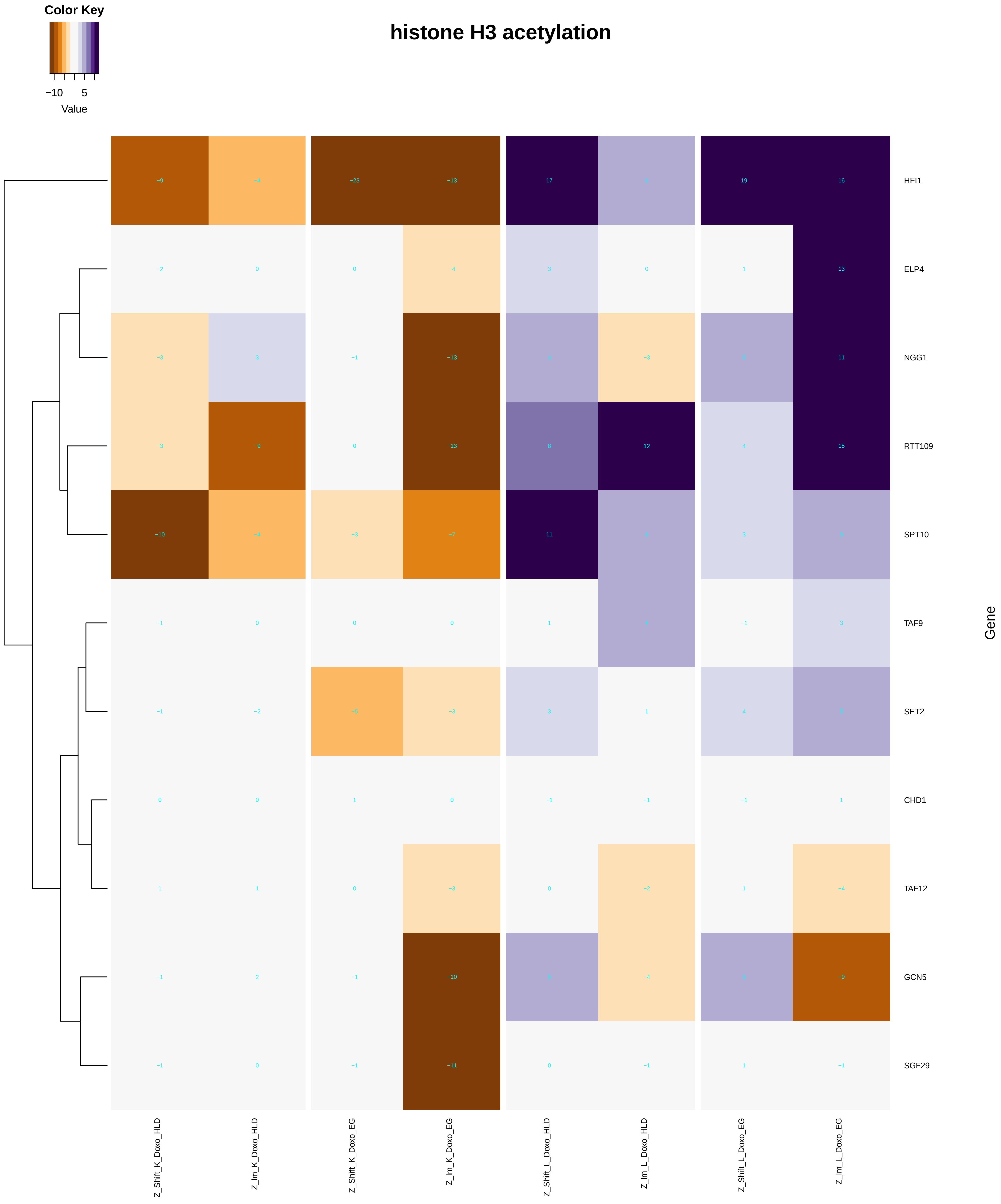
Color Key
−10
5
Value
histone H3 acetylation
HFI1
ELP4
NGG1
RTT109
SPT10
TAF9
SET2
CHD1
TAF12
GCN5
SGF29
Gene
Z_Shift_K_Doxo_HLD
Z_lm_K_Doxo_HLD
Z_Shift_K_Doxo_EG
Z_lm_K_Doxo_EG
Z_Shift_L_Doxo_HLD
Z_lm_L_Doxo_HLD
Z_Shift_L_Doxo_EG
Z_lm_L_Doxo_EG

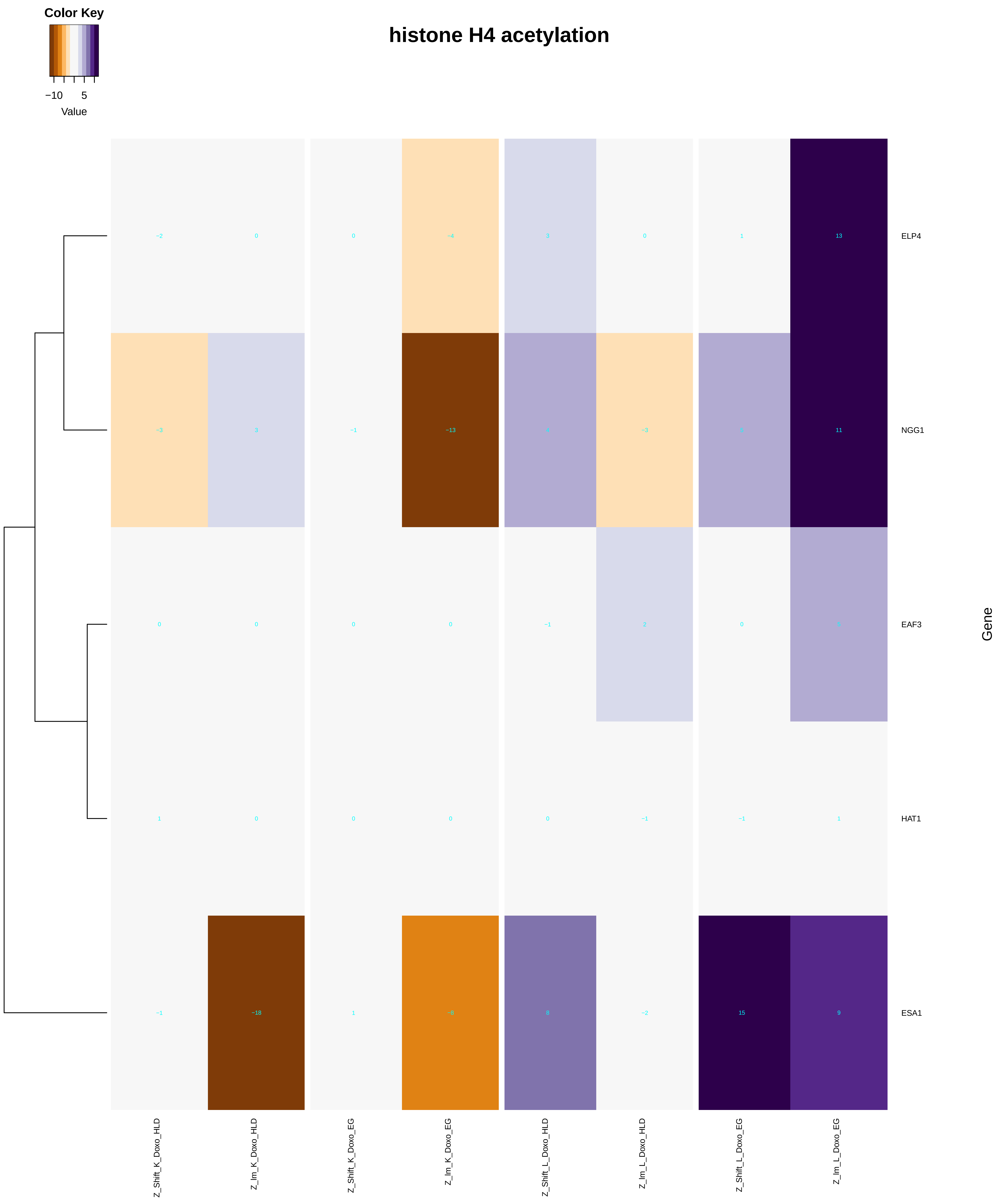
Color Key
−10
5
Value
histone H4 acetylation
ELP4
NGG1
EAF3
HAT1
ESA1
Gene
Z_Shift_K_Doxo_HLD
Z_Im_K_Doxo_HLD
Z_Shift_K_Doxo_EG
Z_Im_K_Doxo_EG
Z_Shift_L_Doxo_HLD
Z_Im_L_Doxo_HLD
Z_Shift_L_Doxo_EG
Z_Im_L_Doxo_EG

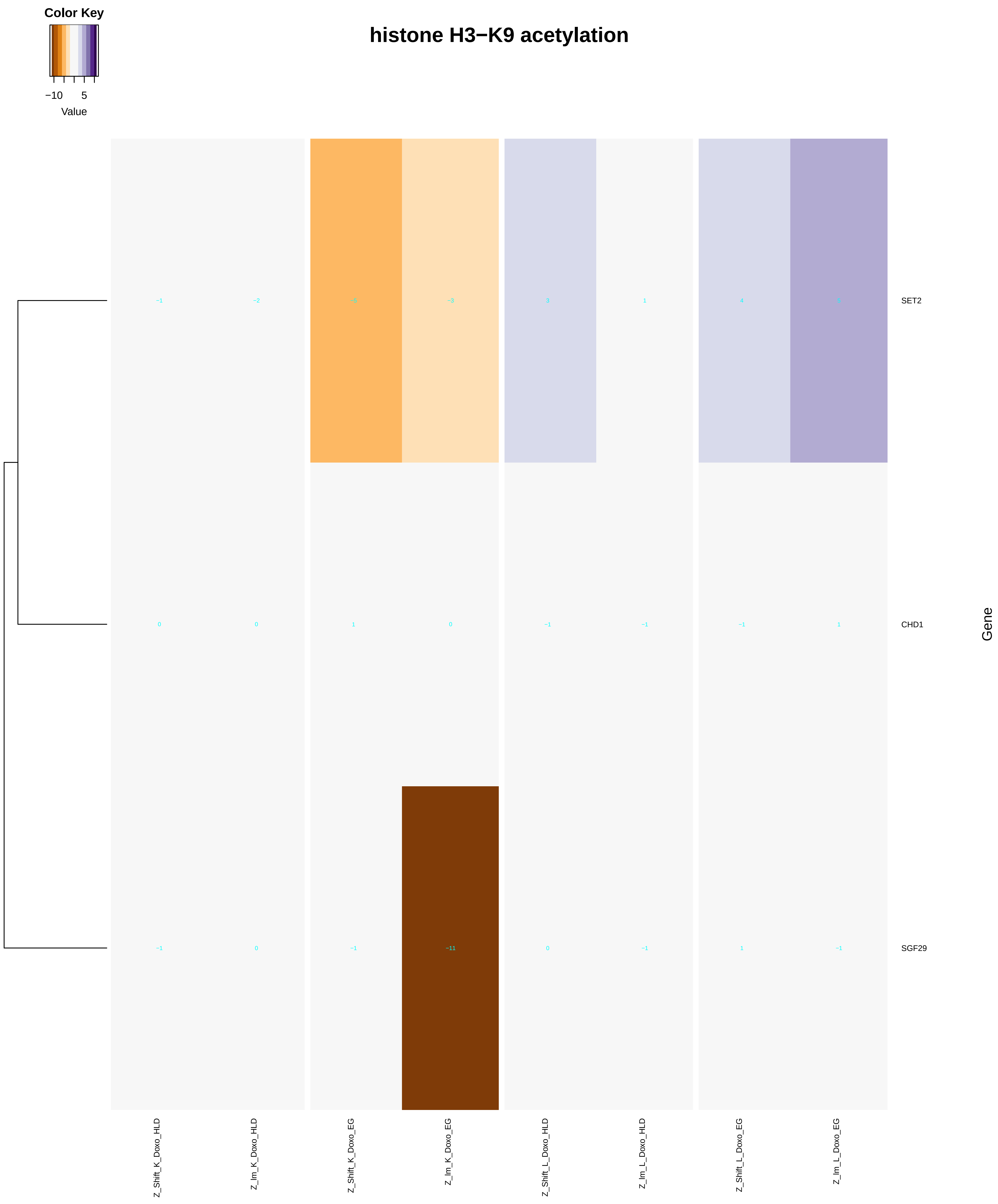
histone H3−K9 acetylation
Color Key
−10
5
Value
SET2
CHD1
SGF29
Gene
Z_Shift_K_Doxo_HLD
Z_lm_K_Doxo_HLD
Z_Shift_K_Doxo_EG
Z_lm_K_Doxo_EG
Z_Shift_L_Doxo_HLD
Z_lm_L_Doxo_HLD
Z_Shift_L_Doxo_EG
Z_lm_L_Doxo_EG

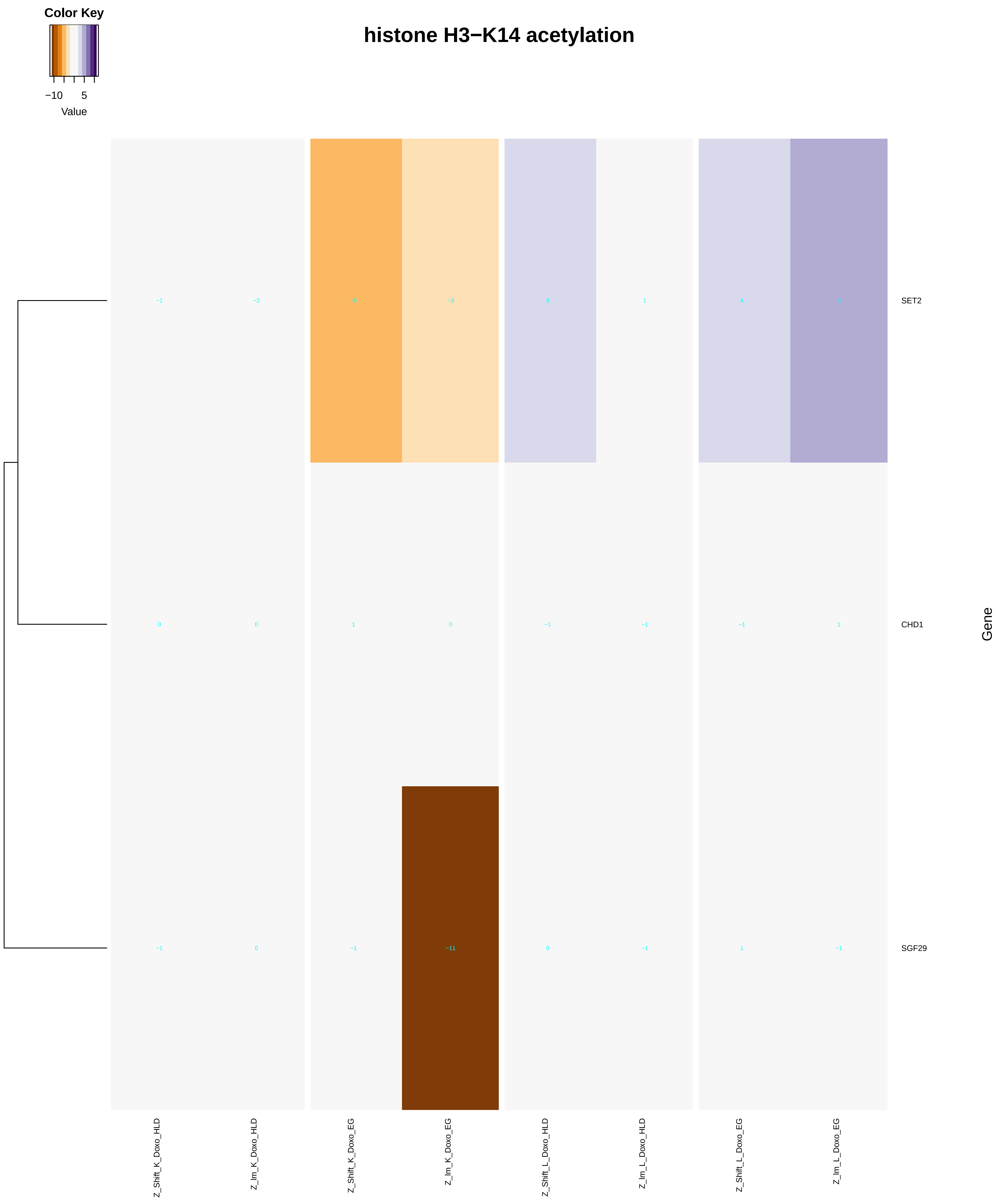
Color Key
−10
5
Value
histone H3−K14 acetylation
SET2
CHD1
SGF29
Gene
Z_Shift_K_Doxo_HLD
Z_Im_K_Doxo_HLD
Z_Shift_K_Doxo_EG
Z_Im_K_Doxo_EG
Z_Shift_L_Doxo_HLD
Z_Im_L_Doxo_HLD
Z_Shift_L_Doxo_EG
Z_Im_L_Doxo_EG
