# Supplementary material for: A yeast phenomic model for the influence of Warburg metabolism on genetic buffering of doxorubicin
Source: Cancer Metab. 2019 Oct 23;7:9. doi: 10.1186/s40170-019-0201-3 (PMC6806529; doi:10.1186/s40170-019-0201-3)
Supplement: Supplementary file 9 — Additional file 9. GO term-specific heatmaps for REMc/GTF-enriched clusters. GO term-specific heatmaps for significant GO process terms were generated as described in methods and Figs. 3 and 4. Any related child terms are presented in subsequent pages of the parent file name. GO terms with more than 100 children, with 2 or fewer genes annotated to the term, or a file size over 300KB are not shown. All heatmaps are generated with the same layout (see Figs. 3 and 4). [file 40170_2019_201_MOESM9_ESM.bz2 › Additional_File9_GOTermHeatmaps/Additional_File9_GOTermHeatmap/mitochondrial_membrane_organization.pdf]

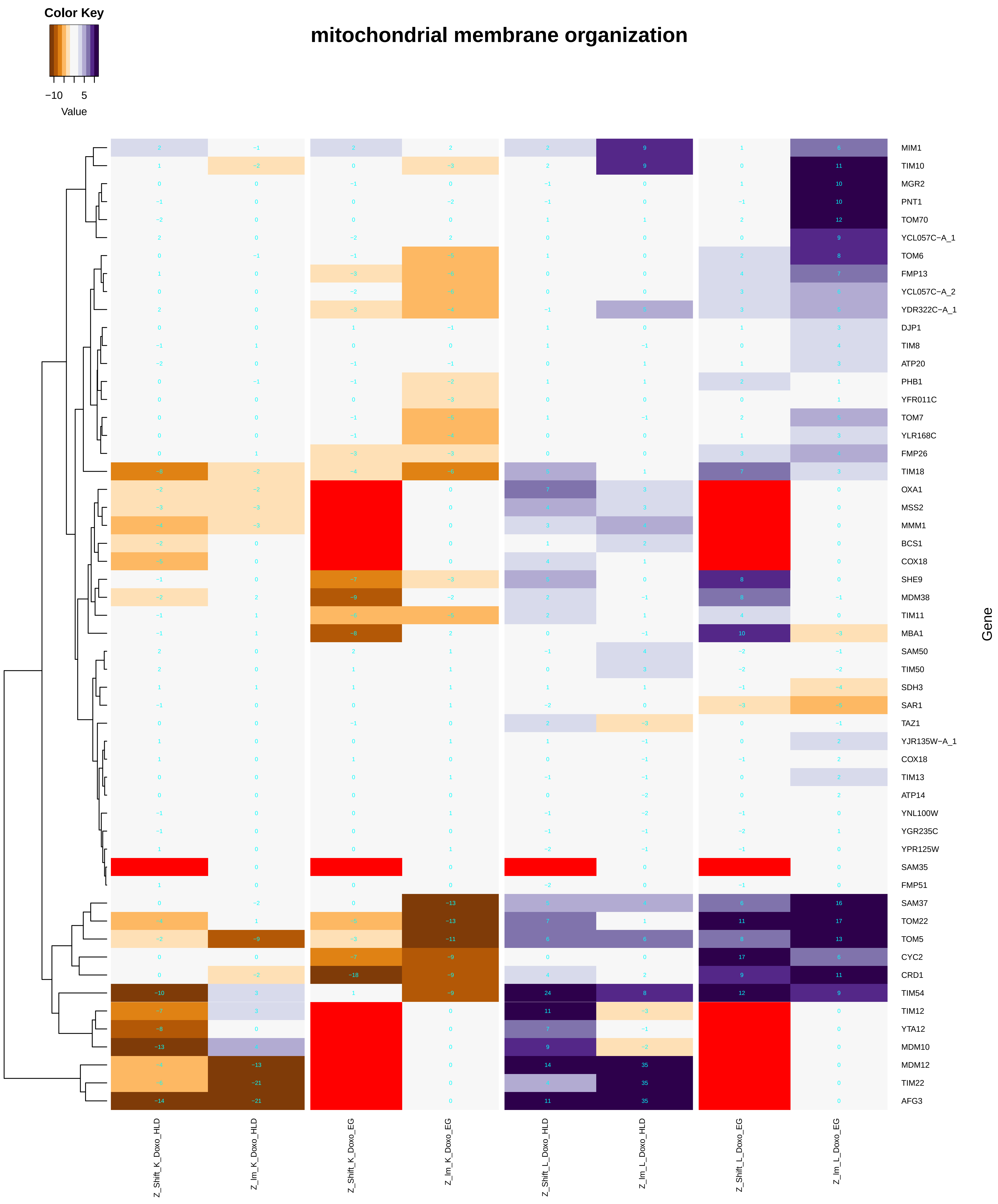
Color Key
−10 5
Value
mitochondrial membrane organization
MIM1
TIM10
MGR2
PNT1
TOM70
YCL057C−A_1
TOM6
FMP13
YCL057C−A_2
YDR322C−A_1
DJP1
TIM8
ATP20
PHB1
YFR011C
TOM7
YLR168C
FMP26
TIM18
OXA1
MSS2
MMM1
BCS1
COX18
SHE9
MDM38
TIM11
MBA1
SAM50
TIM50
SDH3
SAR1
TAZ1
YJR135W−A_1
COX18
TIM13
ATP14
YNL100W
YGR235C
YPR125W
SAM35
FMP51
SAM37
TOM22
TOM5
CYC2
CRD1
TIM54
TIM12
YTA12
MDM10
MDM12
TIM22
AFG3
Gene
Z_Shift_K_Doxo_HLD
Z_Im_K_Doxo_HLD
Z_Shift_K_Doxo_EG
Z_Im_K_Doxo_EG
Z_Shift_L_Doxo_HLD
Z_Im_L_Doxo_HLD
Z_Shift_L_Doxo_EG
Z_Im_L_Doxo_EG

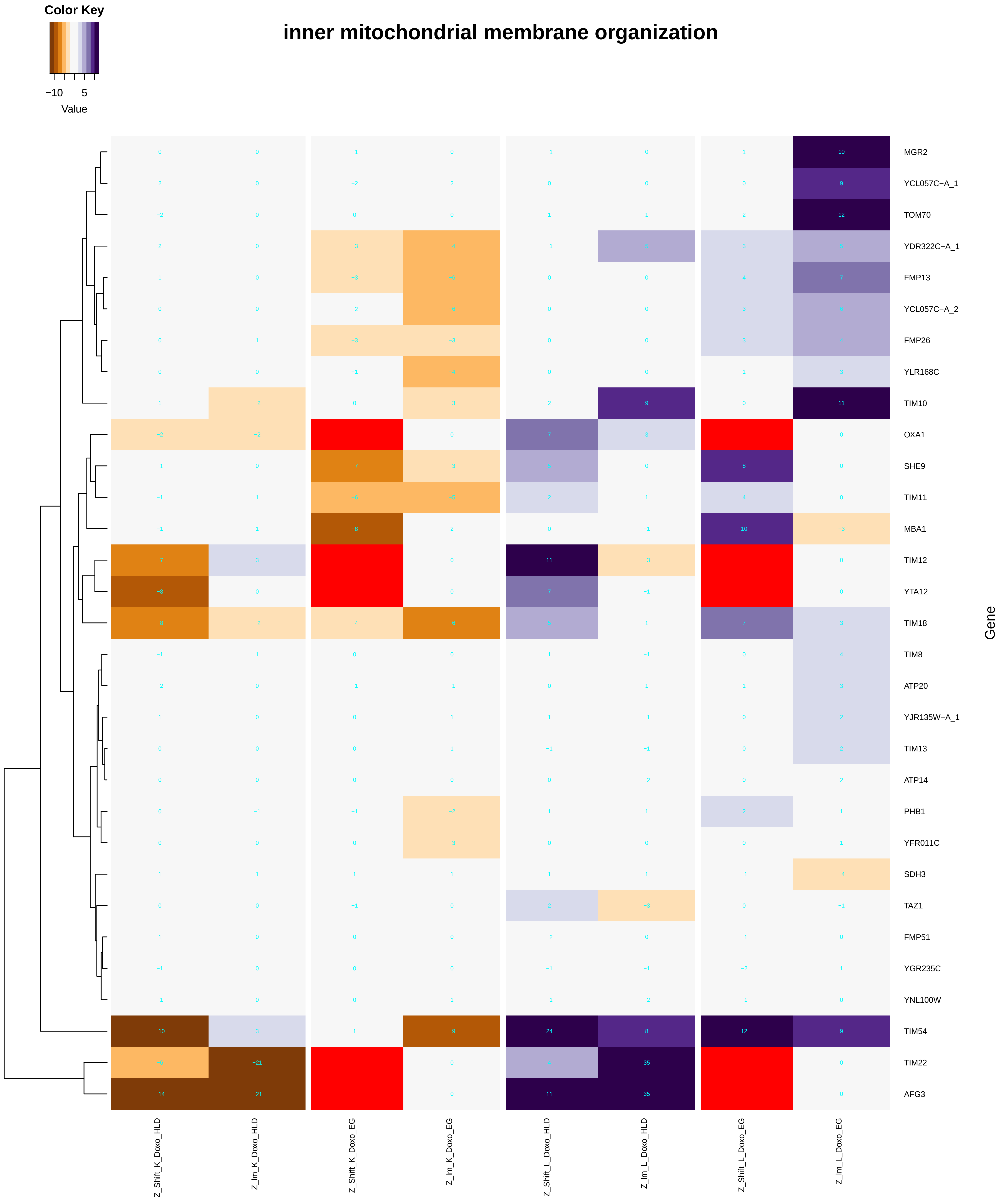

inner mitochondrial membrane organization
Color Key
−10
5
Value
MGR2
YCL057C−A_1
TOM70
YDR322C−A_1
FMP13
YCL057C−A_2
FMP26
YLR168C
TIM10
OXA1
SHE9
TIM11
MBA1
TIM12
YTA12
TIM18
TIM8
ATP20
YJR135W−A_1
TIM13
ATP14
PHB1
YFR011C
SDH3
TAZ1
FMP51
YGR235C
YNL100W
TIM54
TIM22
AFG3
Gene
Z_Shift_K_Doxo_HLD
Z_lm_K_Doxo_HLD
Z_Shift_K_Doxo_EG
Z_lm_K_Doxo_EG
Z_Shift_L_Doxo_HLD
Z_lm_L_Doxo_HLD
Z_Shift_L_Doxo_EG
Z_lm_L_Doxo_EG

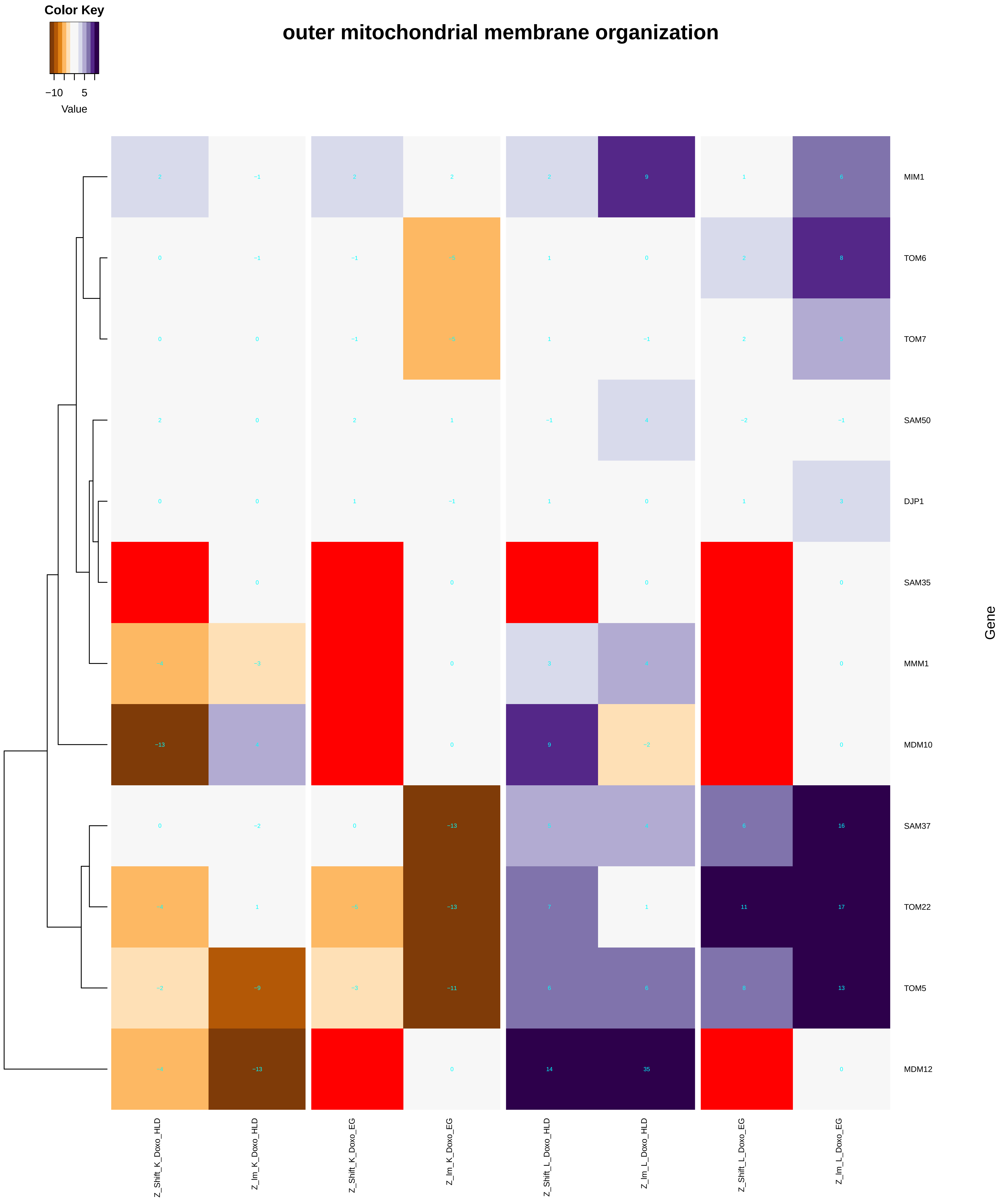

outer mitochondrial membrane organization
Color Key
−10
5
Value
MIM1
TOM6
TOM7
SAM50
DJP1
SAM35
MMM1
MDM10
SAM37
TOM22
TOM5
MDM12
Gene
Z_Shift_K_Doxo_HLD
Z_lm_K_Doxo_HLD
Z_Shift_K_Doxo_EG
Z_lm_K_Doxo_EG
Z_Shift_L_Doxo_HLD
Z_lm_L_Doxo_HLD
Z_Shift_L_Doxo_EG
Z_lm_L_Doxo_EG

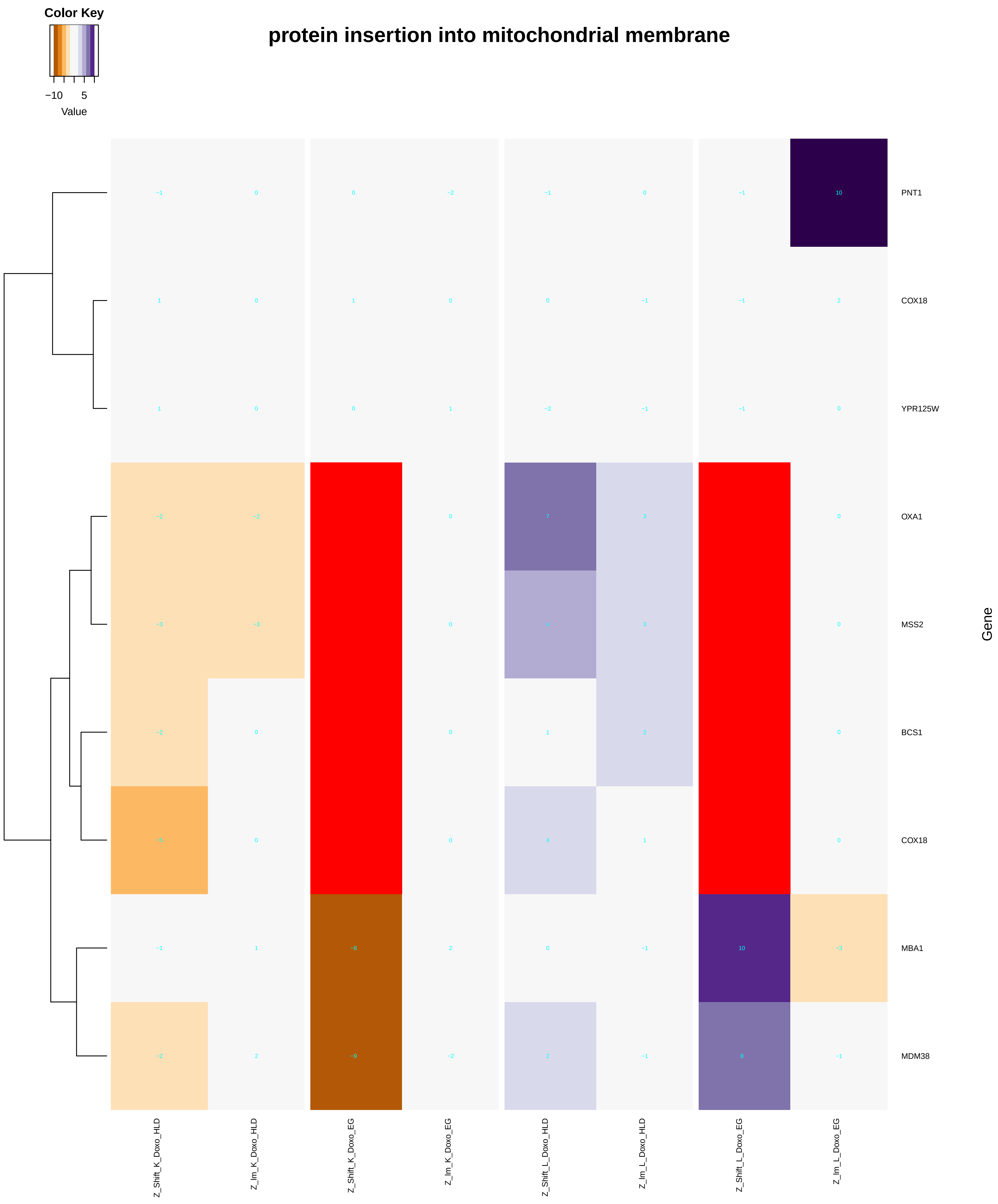
protein insertion into mitochondrial membrane
Color Key
−10
5
Value
PNT1
COX18
YPR125W
OXA1
MSS2
BCS1
COX18
MBA1
MDM38
Gene
Z_Shift_K_Doxo_HLD
Z_Im_K_Doxo_HLD
Z_Shift_K_Doxo_EG
Z_Im_K_Doxo_EG
Z_Shift_L_Doxo_HLD
Z_Im_L_Doxo_HLD
Z_Shift_L_Doxo_EG
Z_Im_L_Doxo_EG

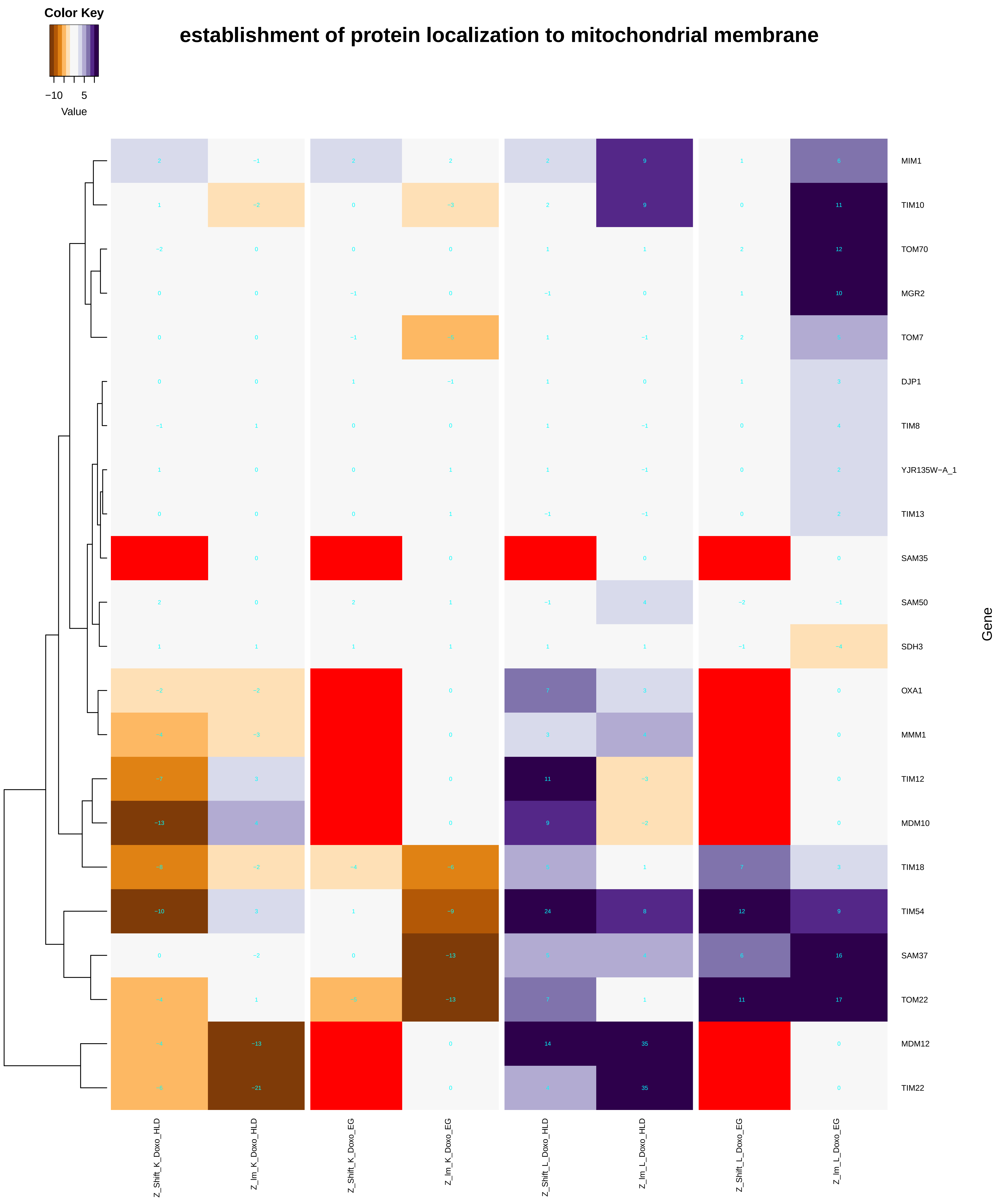
Color Key
−10
5
Value
establishment of protein localization to mitochondrial membrane
MIM1
TIM10
TOM70
MGR2
TOM7
DJP1
TIM8
YJR135W−A_1
TIM13
SAM35
SAM50
SDH3
OXA1
MMM1
TIM12
MDM10
TIM18
TIM54
SAM37
TOM22
MDM12
TIM22
Gene
Z_Shift_K_Doxo_HLD
Z_Im_K_Doxo_HLD
Z_Shift_K_Doxo_EG
Z_Im_K_Doxo_EG
Z_Shift_L_Doxo_HLD
Z_Im_L_Doxo_HLD
Z_Shift_L_Doxo_EG
Z_Im_L_Doxo_EG

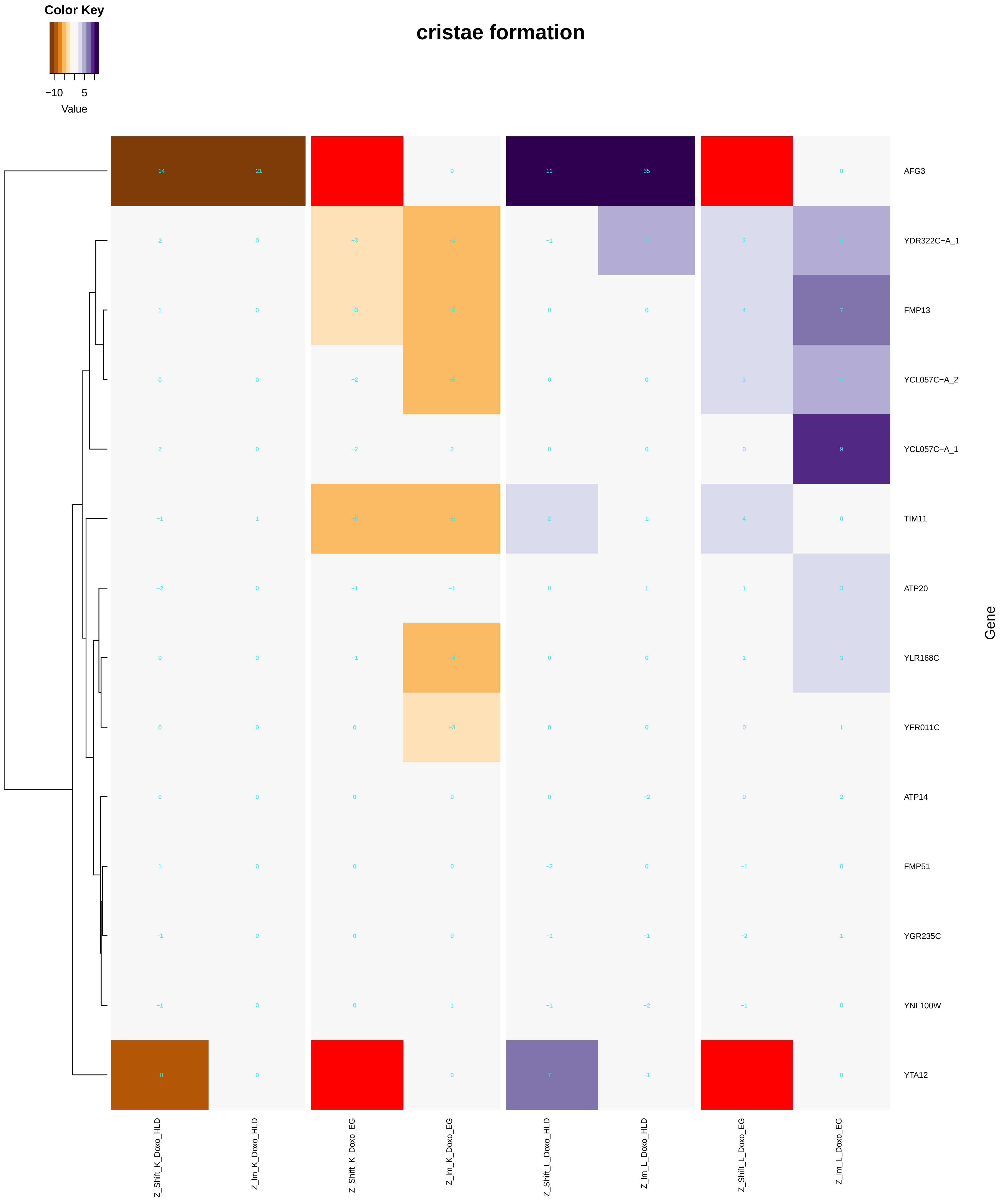
Color Key
−10
5
Value
cristae formation
AFG3
YDR322C−A_1
FMP13
YCL057C−A_2
YCL057C−A_1
TIM11
ATP20
YLR168C
YFR011C
ATP14
FMP51
YGR235C
YNL100W
YTA12
Gene
Z_Shift_K_Doxo_HLD
Z_lm_K_Doxo_HLD
Z_Shift_K_Doxo_EG
Z_lm_K_Doxo_EG
Z_Shift_L_Doxo_HLD
Z_lm_L_Doxo_HLD
Z_Shift_L_Doxo_EG
Z_lm_L_Doxo_EG

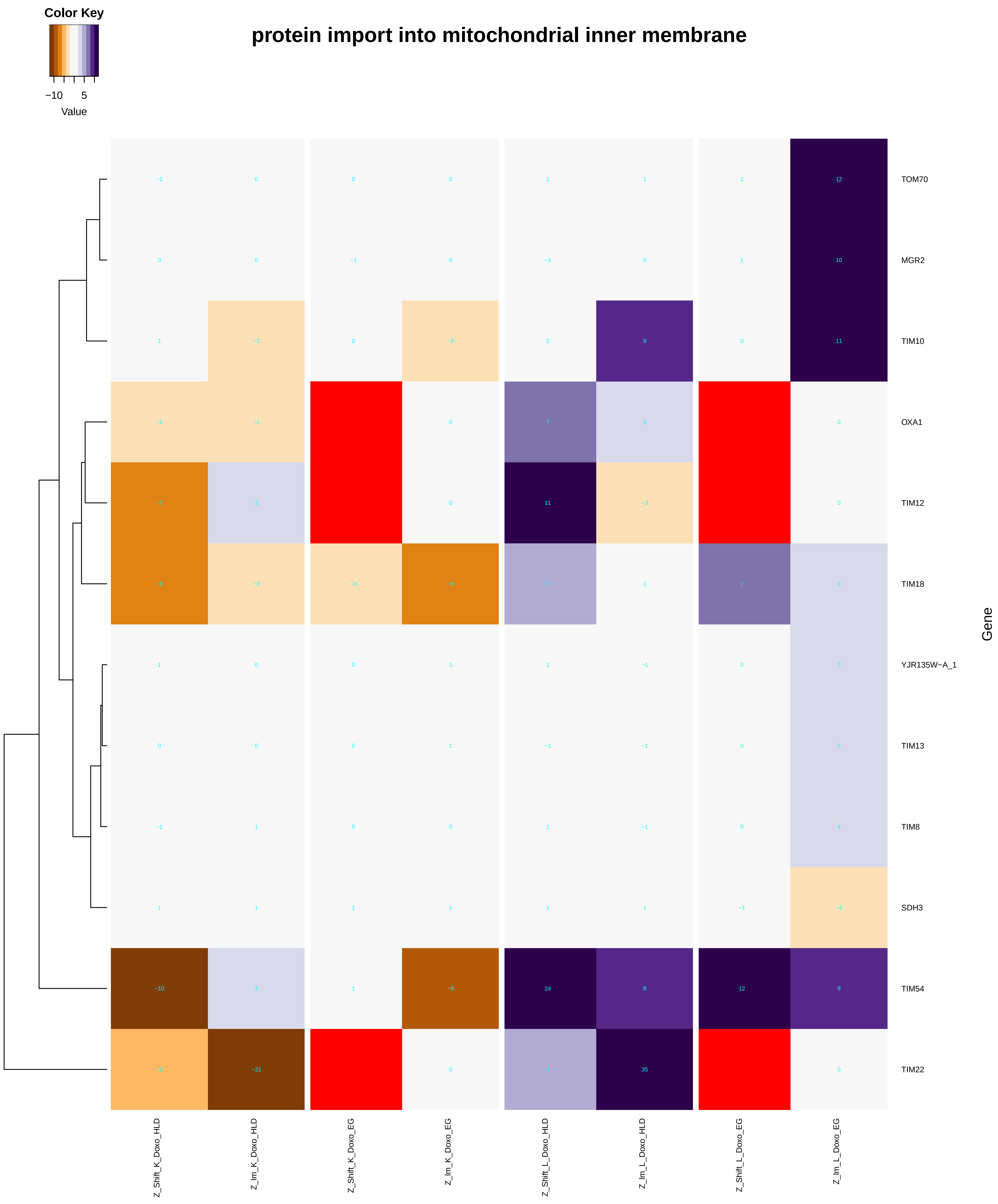
Color Key
−10
5
Value
protein import into mitochondrial inner membrane
TOM70
MGR2
TIM10
OXA1
TIM12
TIM18
YJR135W−A_1
TIM13
TIM8
SDH3
TIM54
TIM22
Gene
Z_Shift_K_Doxo_HLD
Z_Im_K_Doxo_HLD
Z_Shift_K_Doxo_EG
Z_Im_K_Doxo_EG
Z_Shift_L_Doxo_HLD
Z_Im_L_Doxo_HLD
Z_Shift_L_Doxo_EG
Z_Im_L_Doxo_EG

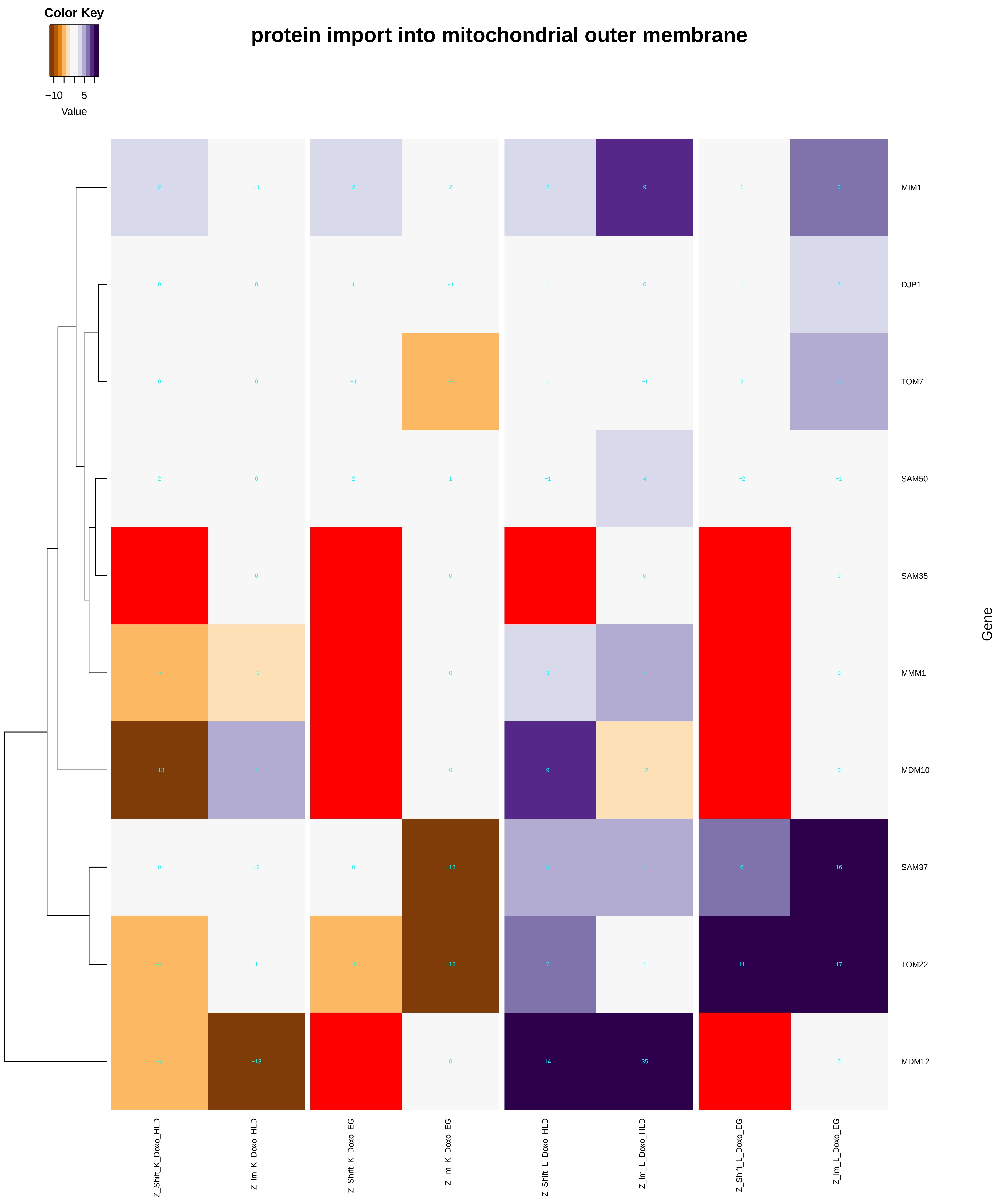
Color Key
−10
5
Value
protein import into mitochondrial outer membrane
MIM1
DJP1
TOM7
SAM50
SAM35
MMM1
MDM10
SAM37
TOM22
MDM12
Gene
Z_Shift_K_Doxo_HLD
Z_Im_K_Doxo_HLD
Z_Shift_K_Doxo_EG
Z_Im_K_Doxo_EG
Z_Shift_L_Doxo_HLD
Z_Im_L_Doxo_HLD
Z_Shift_L_Doxo_EG
Z_Im_L_Doxo_EG

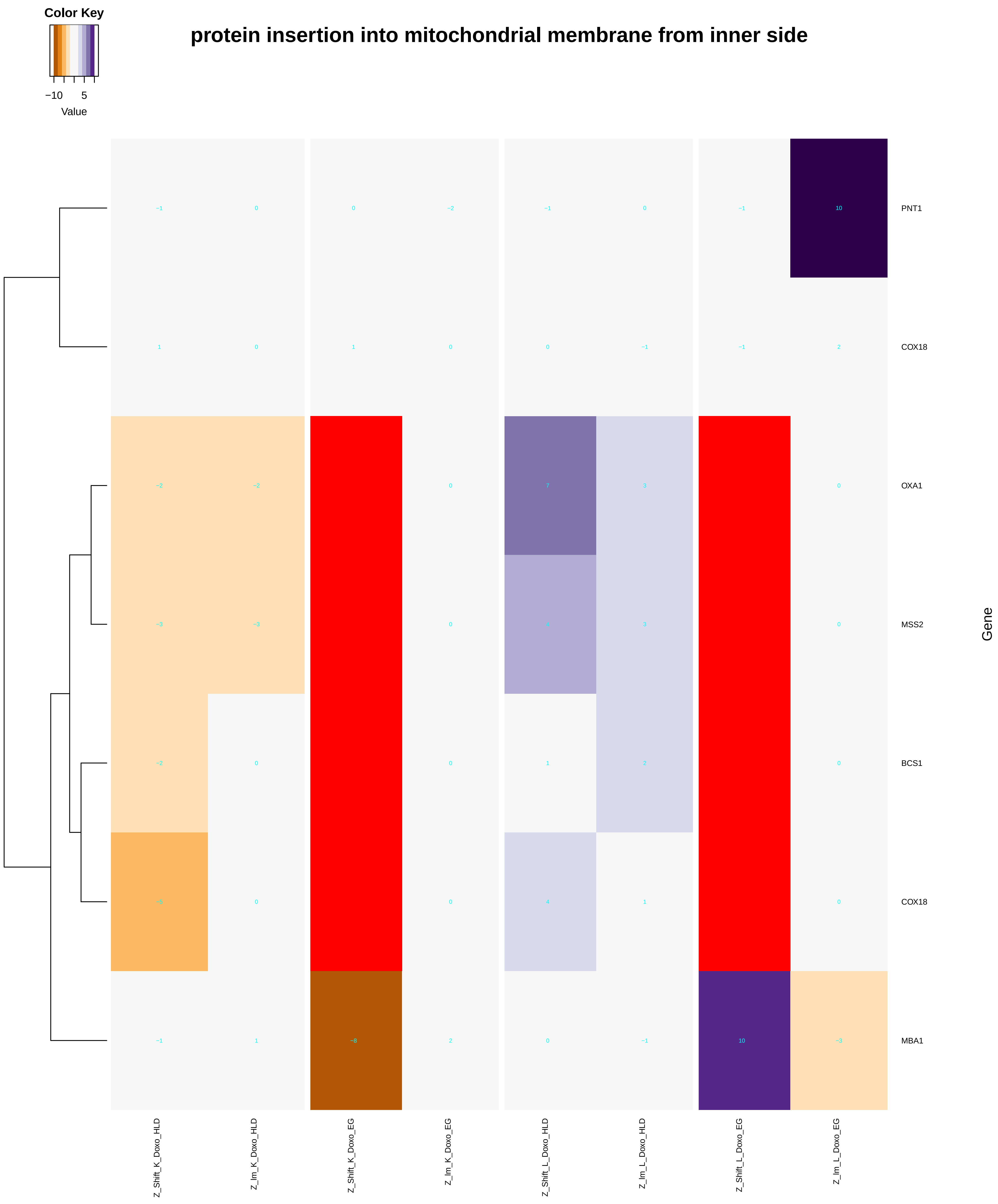
Color Key
−10
5
Value
protein insertion into mitochondrial membrane from inner side
PNT1
COX18
OXA1
MSS2
BCS1
COX18
MBA1
Gene
Z_Shift_K_Doxo_HLD
Z_Im_K_Doxo_HLD
Z_Shift_K_Doxo_EG
Z_Im_K_Doxo_EG
Z_Shift_L_Doxo_HLD
Z_Im_L_Doxo_HLD
Z_Shift_L_Doxo_EG
Z_Im_L_Doxo_EG
