# Supplementary material for: A yeast phenomic model for the influence of Warburg metabolism on genetic buffering of doxorubicin
Source: Cancer Metab. 2019 Oct 23;7:9. doi: 10.1186/s40170-019-0201-3 (PMC6806529; doi:10.1186/s40170-019-0201-3)
Supplement: Supplementary file 9 — Additional file 9. GO term-specific heatmaps for REMc/GTF-enriched clusters. GO term-specific heatmaps for significant GO process terms were generated as described in methods and Figs. 3 and 4. Any related child terms are presented in subsequent pages of the parent file name. GO terms with more than 100 children, with 2 or fewer genes annotated to the term, or a file size over 300KB are not shown. All heatmaps are generated with the same layout (see Figs. 3 and 4). [file 40170_2019_201_MOESM9_ESM.bz2 › Additional_File9_GOTermHeatmaps/Additional_File9_GOTermHeatmap/mitochondrial_respiratory_chain_complex_assembly.pdf]

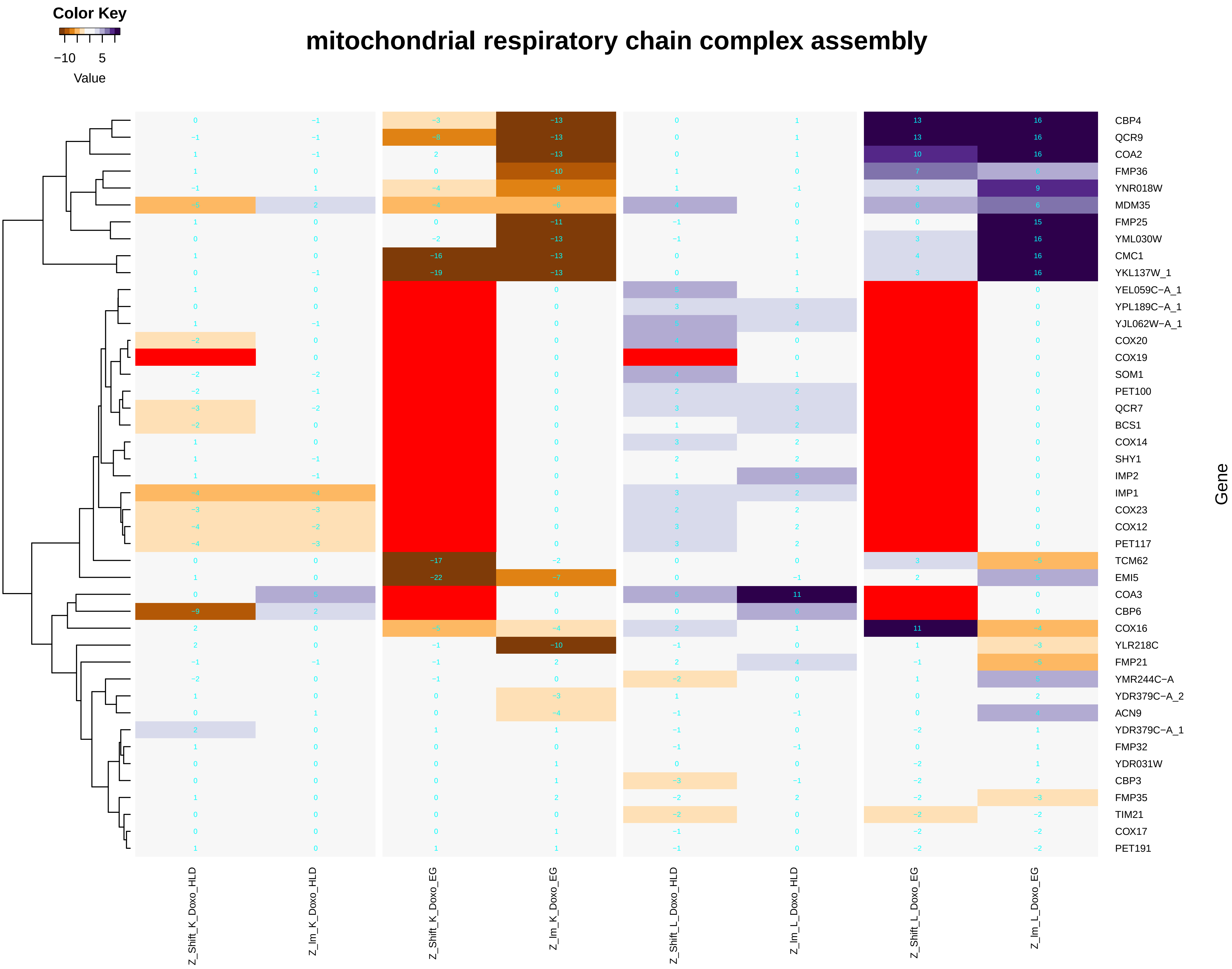
mitochondrial respiratory chain complex assembly
Color Key
−10
5
Value
CBP4
QCR9
COA2
FMP36
YNR018W
MDM35
FMP25
YML030W
CMC1
YKL137W_1
YEL059C−A_1
YPL189C−A_1
YJL062W−A_1
COX20
COX19
SOM1
PET100
QCR7
BCS1
COX14
SHY1
IMP2
IMP1
COX23
COX12
PET117
TCM62
EMI5
COA3
CBP6
COX16
YLR218C
FMP21
YMR244C−A
YDR379C−A_2
ACN9
YDR379C−A_1
FMP32
YDR031W
CBP3
FMP35
TIM21
COX17
PET191
Gene
Z_Shift_K_Doxo_HLD
Z_Im_K_Doxo_HLD
Z_Shift_K_Doxo_EG
Z_Im_K_Doxo_EG
Z_Shift_L_Doxo_HLD
Z_Im_L_Doxo_HLD
Z_Shift_L_Doxo_EG
Z_Im_L_Doxo_EG

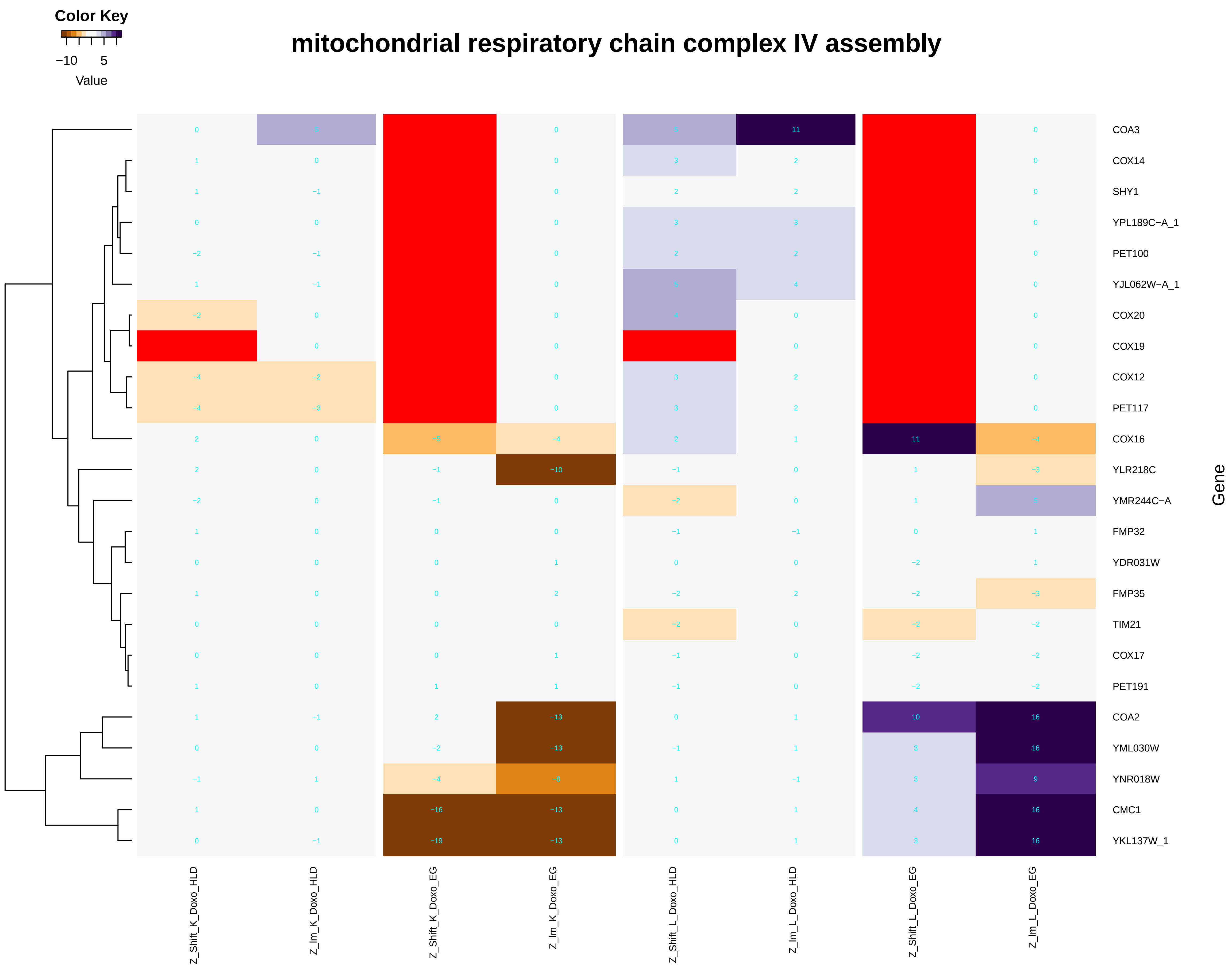

Color Key
−10 5
Value
mitochondrial respiratory chain complex IV assembly
COA3
COX14
SHY1
YPL189C−A_1
PET100
YJL062W−A_1
COX20
COX19
COX12
PET117
COX16
YLR218C
YMR244C−A
FMP32
YDR031W
FMP35
TIM21
COX17
PET191
COA2
YML030W
YNR018W
CMC1
YKL137W_1
Gene
Z_Shift_K_Doxo_HLD
Z_Im_K_Doxo_HLD
Z_Shift_K_Doxo_EG
Z_Im_K_Doxo_EG
Z_Shift_L_Doxo_HLD
Z_Im_L_Doxo_HLD
Z_Shift_L_Doxo_EG
Z_Im_L_Doxo_EG

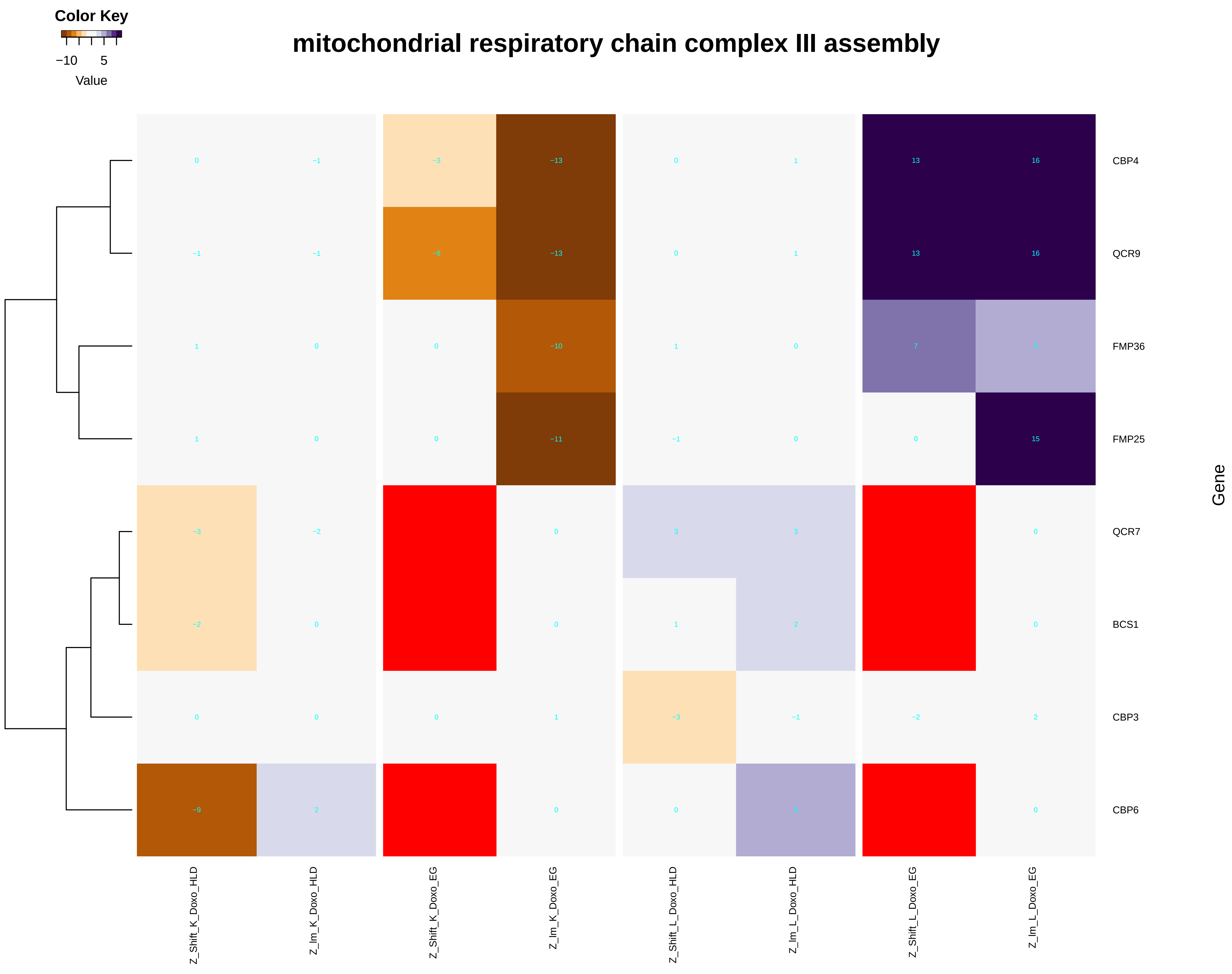
Color Key
−10
5
Value
mitochondrial respiratory chain complex III assembly
CBP4
QCR9
FMP36
FMP25
QCR7
BCS1
CBP3
CBP6
Gene
Z_Shift_K_Doxo_HLD
Z_Im_K_Doxo_HLD
Z_Shift_K_Doxo_EG
Z_Im_K_Doxo_EG
Z_Shift_L_Doxo_HLD
Z_Im_L_Doxo_HLD
Z_Shift_L_Doxo_EG
Z_Im_L_Doxo_EG

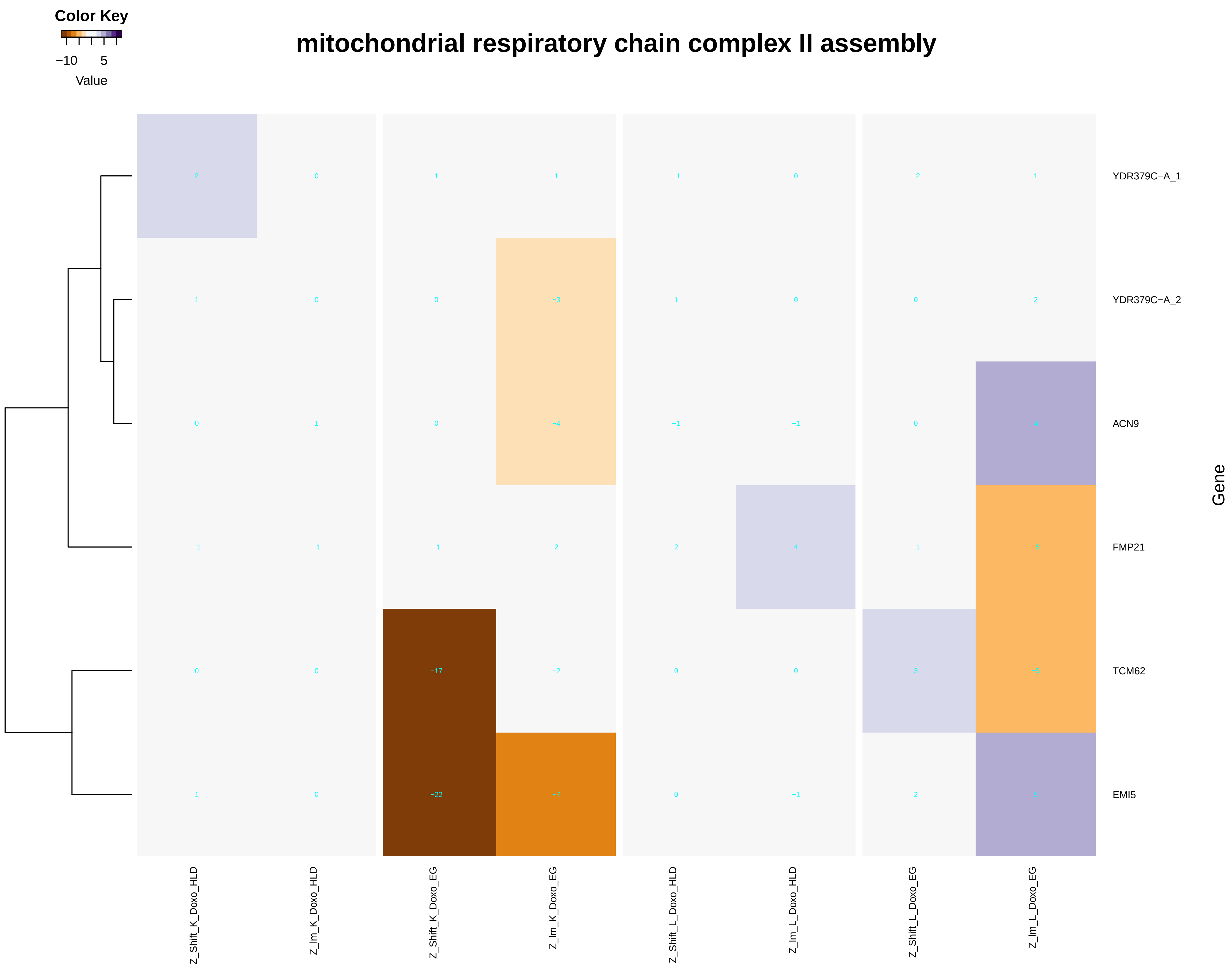

Color Key
−10
5
Value
mitochondrial respiratory chain complex II assembly
YDR379C−A_1
YDR379C−A_2
ACN9
FMP21
TCM62
EMI5
Gene
Z_Shift_K_Doxo_HLD
Z_Im_K_Doxo_HLD
Z_Shift_K_Doxo_EG
Z_Im_K_Doxo_EG
Z_Shift_L_Doxo_HLD
Z_Im_L_Doxo_HLD
Z_Shift_L_Doxo_EG
Z_Im_L_Doxo_EG
