# Supplementary material for: A yeast phenomic model for the influence of Warburg metabolism on genetic buffering of doxorubicin
Source: Cancer Metab. 2019 Oct 23;7:9. doi: 10.1186/s40170-019-0201-3 (PMC6806529; doi:10.1186/s40170-019-0201-3)
Supplement: Supplementary file 9 — Additional file 9. GO term-specific heatmaps for REMc/GTF-enriched clusters. GO term-specific heatmaps for significant GO process terms were generated as described in methods and Figs. 3 and 4. Any related child terms are presented in subsequent pages of the parent file name. GO terms with more than 100 children, with 2 or fewer genes annotated to the term, or a file size over 300KB are not shown. All heatmaps are generated with the same layout (see Figs. 3 and 4). [file 40170_2019_201_MOESM9_ESM.bz2 › Additional_File9_GOTermHeatmaps/Additional_File9_GOTermHeatmap/ncRNA_3'-end_processing.pdf]

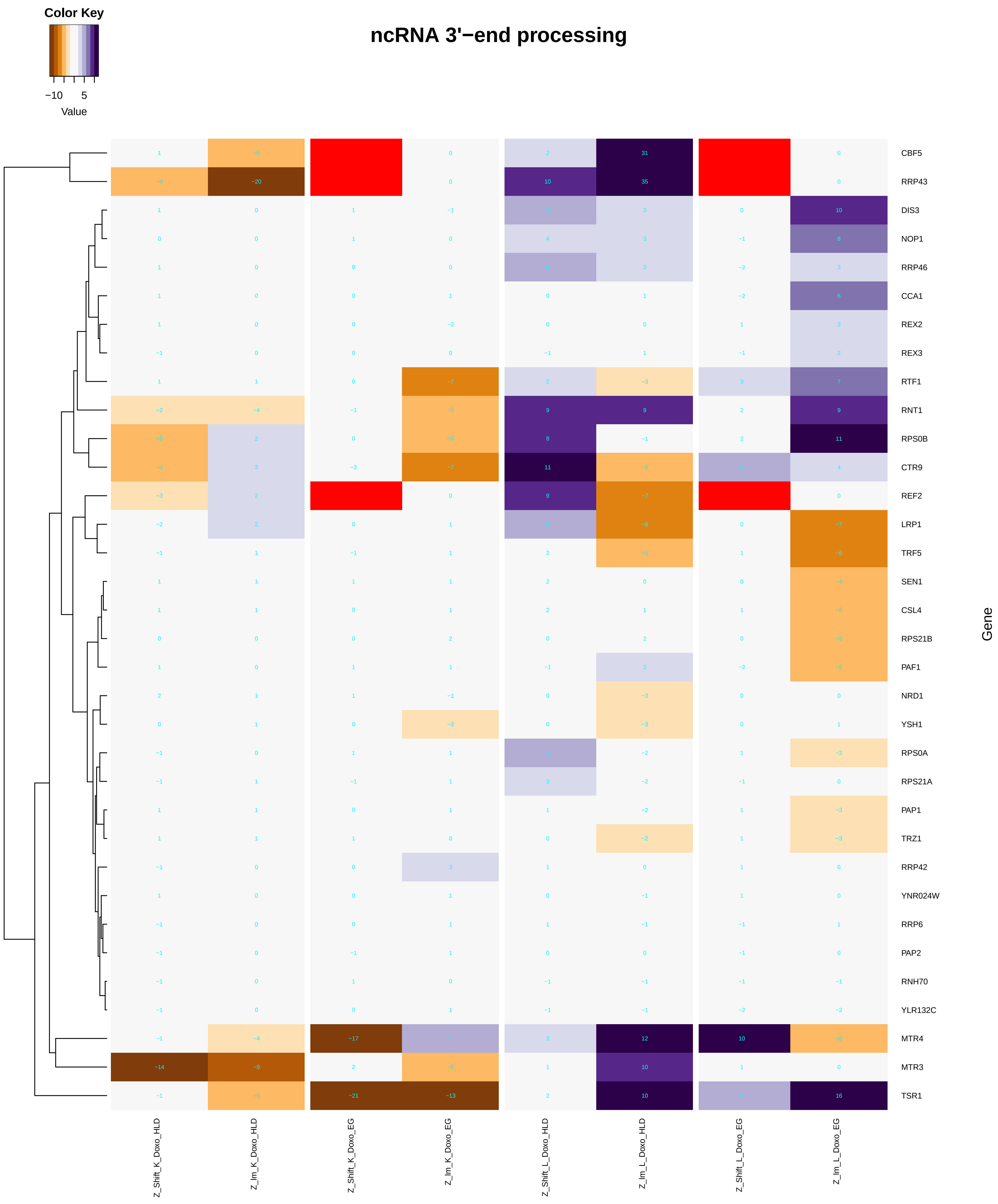
ncRNA 3'−end processing
Color Key
−10
5
Value
CBF5
RRP43
DIS3
NOP1
RRP46
CCA1
REX2
REX3
RTF1
RNT1
RPS0B
CTR9
REF2
LRP1
TRF5
SEN1
CSL4
RPS21B
PAF1
NRD1
YSH1
RPS0A
RPS21A
PAP1
TRZ1
RRP42
YNR024W
RRP6
PAP2
RNH70
YLR132C
MTR4
MTR3
TSR1
Gene
Z_Shift_K_Doxo_HLD
Z_lm_K_Doxo_HLD
Z_Shift_K_Doxo_EG
Z_lm_K_Doxo_EG
Z_Shift_L_Doxo_HLD
Z_lm_L_Doxo_HLD
Z_Shift_L_Doxo_EG
Z_lm_L_Doxo_EG

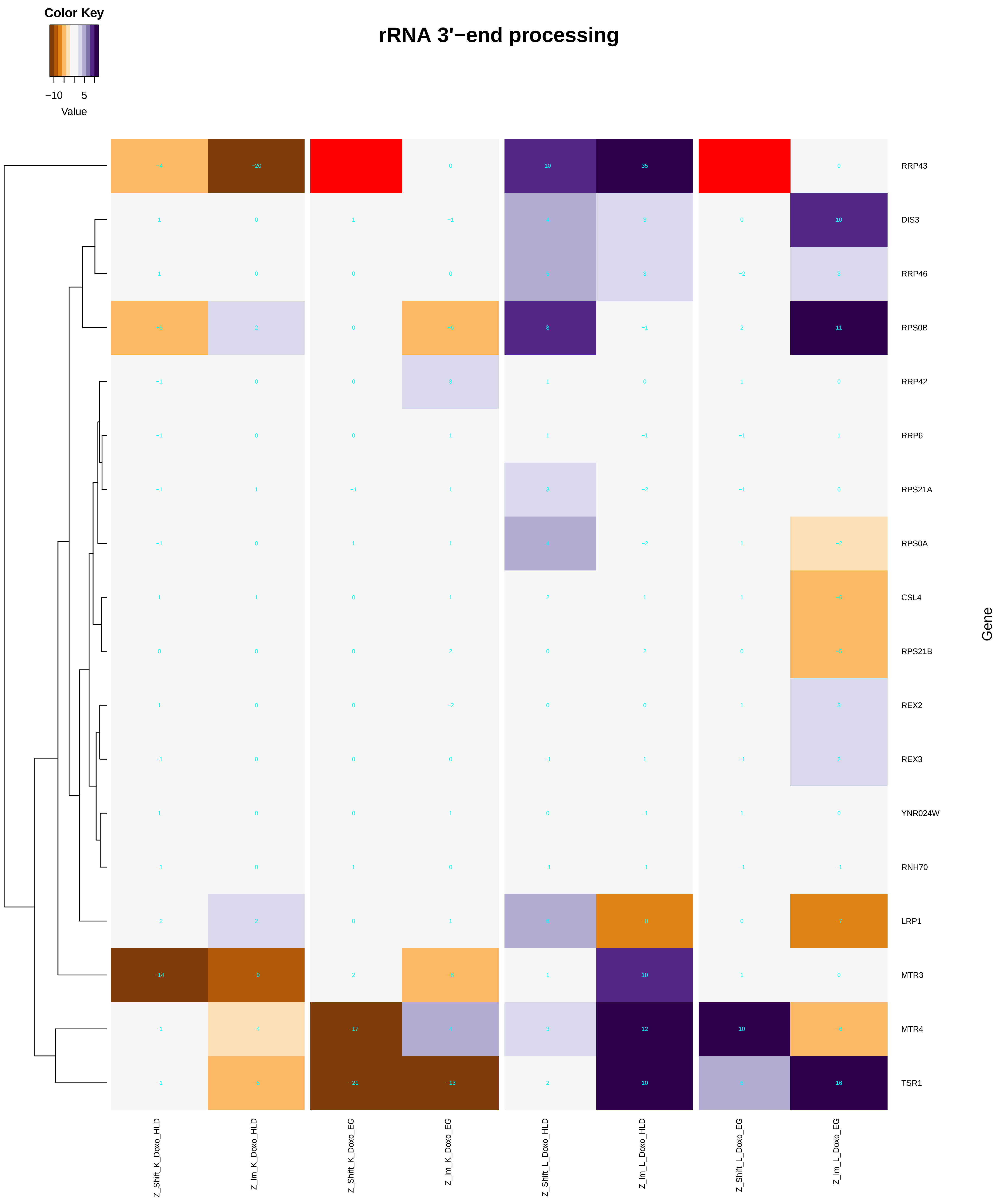
Color Key
−10
5
Value
rRNA 3'−end processing
RRP43
DIS3
RRP46
RPS0B
RRP42
RRP6
RPS21A
RPS0A
CSL4
RPS21B
REX2
REX3
YNR024W
RNH70
LRP1
MTR3
MTR4
TSR1
Gene
Z_Shift_K_Doxo_HLD
Z_Im_K_Doxo_HLD
Z_Shift_K_Doxo_EG
Z_Im_K_Doxo_EG
Z_Shift_L_Doxo_HLD
Z_Im_L_Doxo_HLD
Z_Shift_L_Doxo_EG
Z_Im_L_Doxo_EG

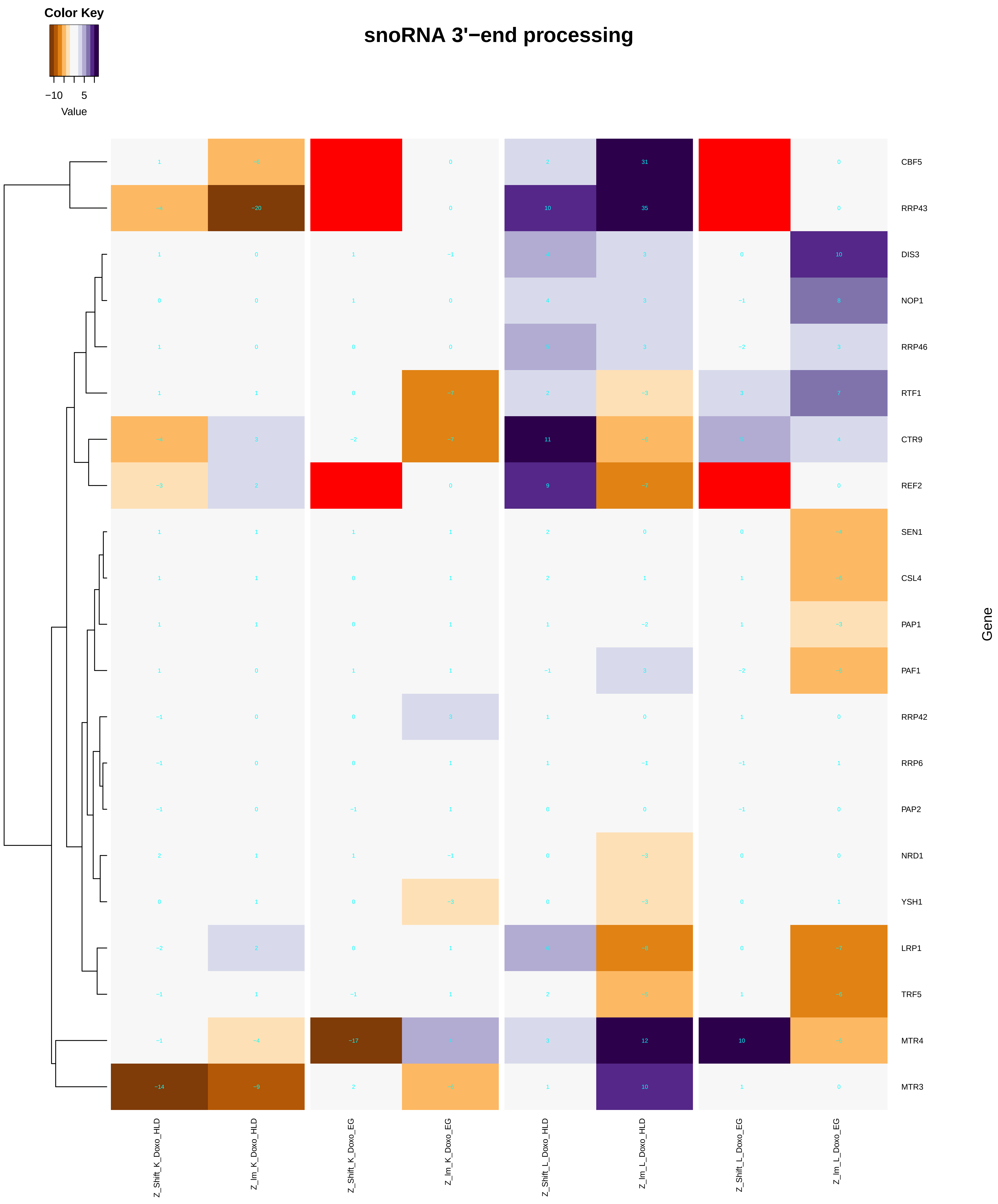
Color Key
−10 5
Value
snoRNA 3'−end processing
CBF5
RRP43
DIS3
NOP1
RRP46
RTF1
CTR9
REF2
SEN1
CSL4
PAP1
PAF1
RRP42
RRP6
PAP2
NRD1
YSH1
LRP1
TRF5
MTR4
MTR3
Gene
Z_Shift_K_Doxo_HLD
Z_Im_K_Doxo_HLD
Z_Shift_K_Doxo_EG
Z_Im_K_Doxo_EG
Z_Shift_L_Doxo_HLD
Z_Im_L_Doxo_HLD
Z_Shift_L_Doxo_EG
Z_Im_L_Doxo_EG

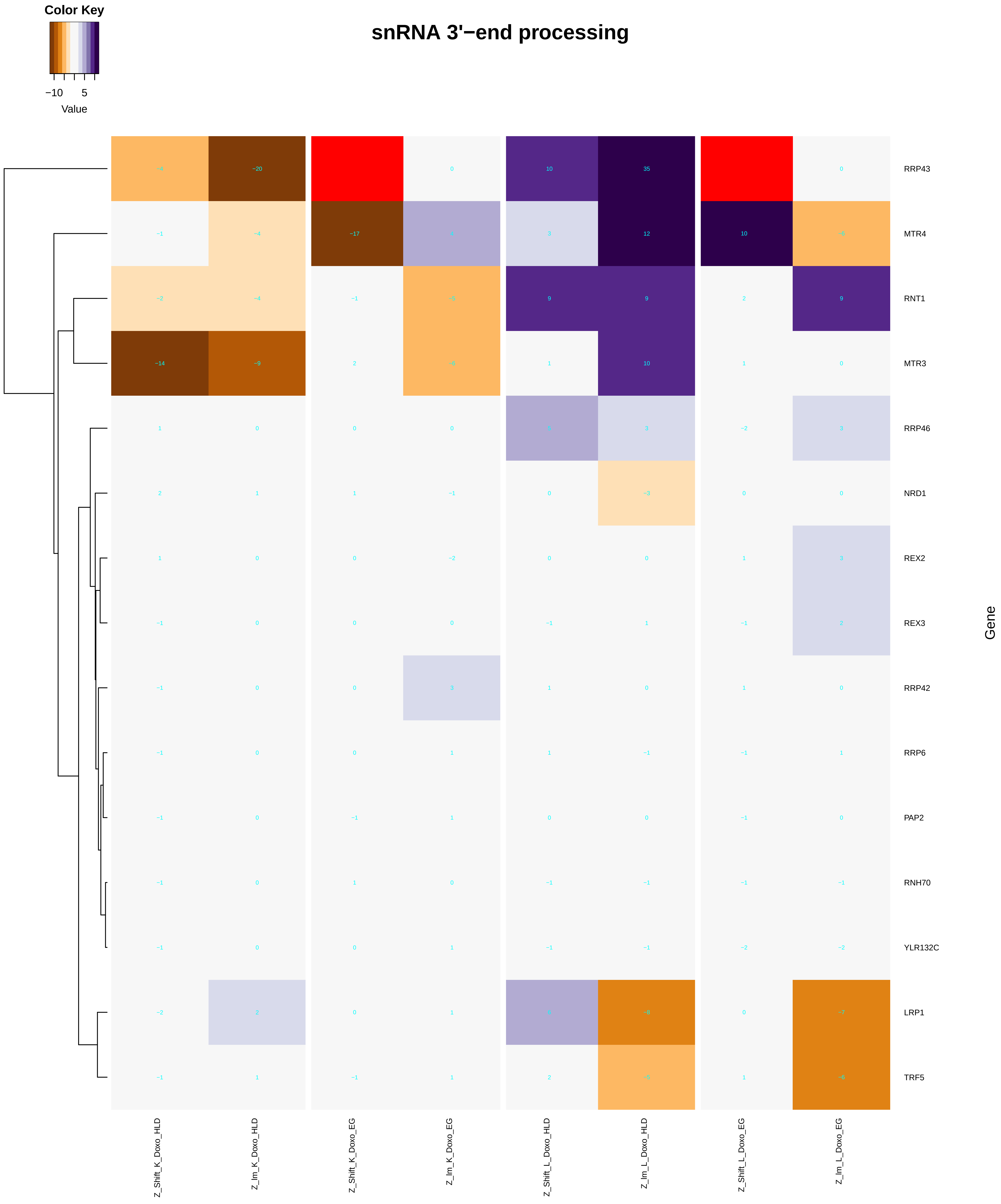
snRNA 3'−end processing
Color Key
−10
5
Value
RRP43
MTR4
RNT1
MTR3
RRP46
NRD1
REX2
REX3
RRP42
RRP6
PAP2
RNH70
YLR132C
LRP1
TRF5
Gene
Z_Shift_K_Doxo_HLD
Z_Im_K_Doxo_HLD
Z_Shift_K_Doxo_EG
Z_Im_K_Doxo_EG
Z_Shift_L_Doxo_HLD
Z_Im_L_Doxo_HLD
Z_Shift_L_Doxo_EG
Z_Im_L_Doxo_EG

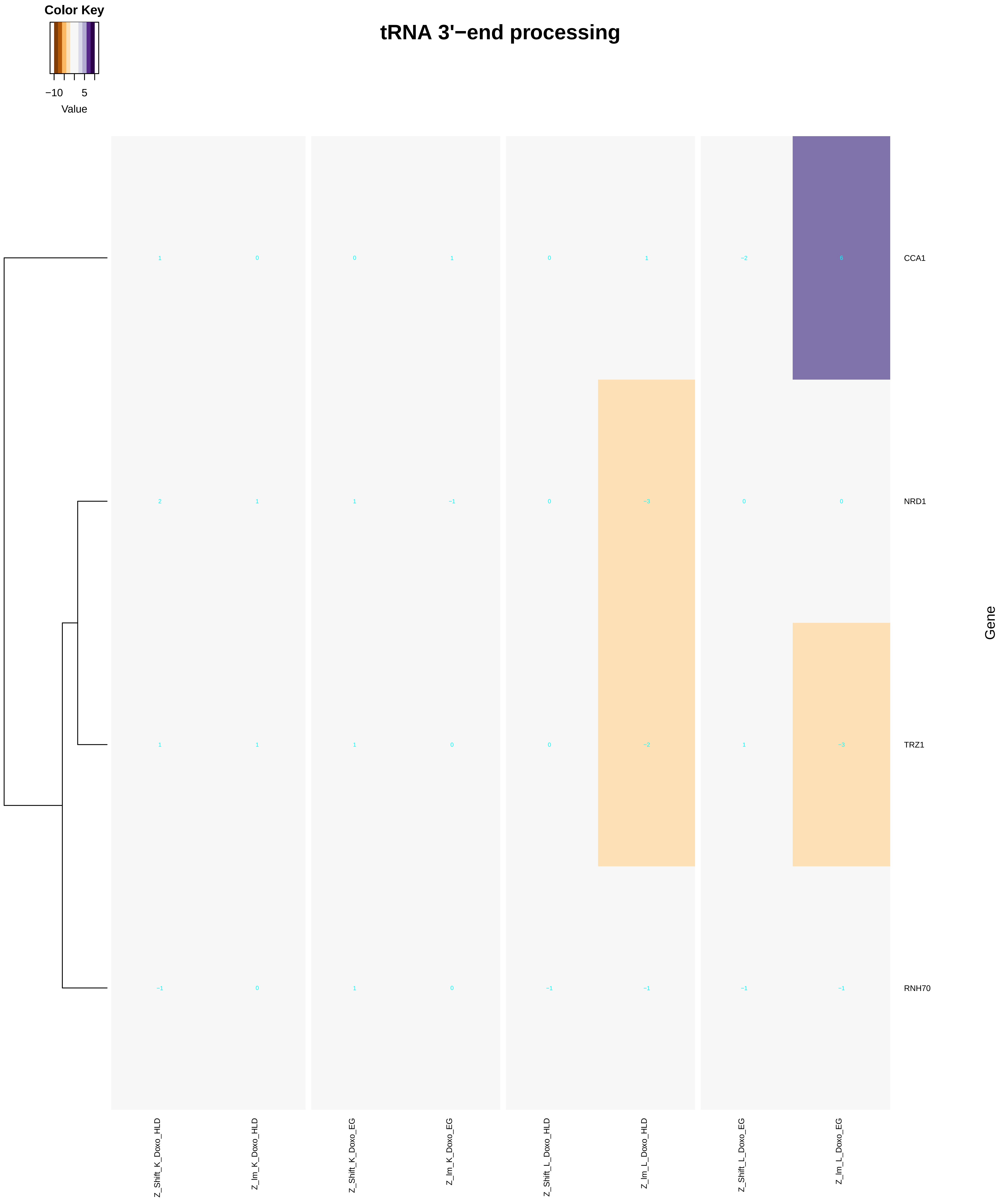
Color Key
−10
5
Value
tRNA 3'−end processing
CCA1
NRD1
TRZ1
RNH70
Gene
1 0 0 1 0 1 −2 6
2 1 1 −1 0 −3 0 0
1 1 1 0 0 −2 1 −3
−1 0 1 0 −1 −1 −1 −1
Z_Shift_K_Doxo_HLD
Z_Im_K_Doxo_HLD
Z_Shift_K_Doxo_EG
Z_Im_K_Doxo_EG
Z_Shift_L_Doxo_HLD
Z_Im_L_Doxo_HLD
Z_Shift_L_Doxo_EG
Z_Im_L_Doxo_EG

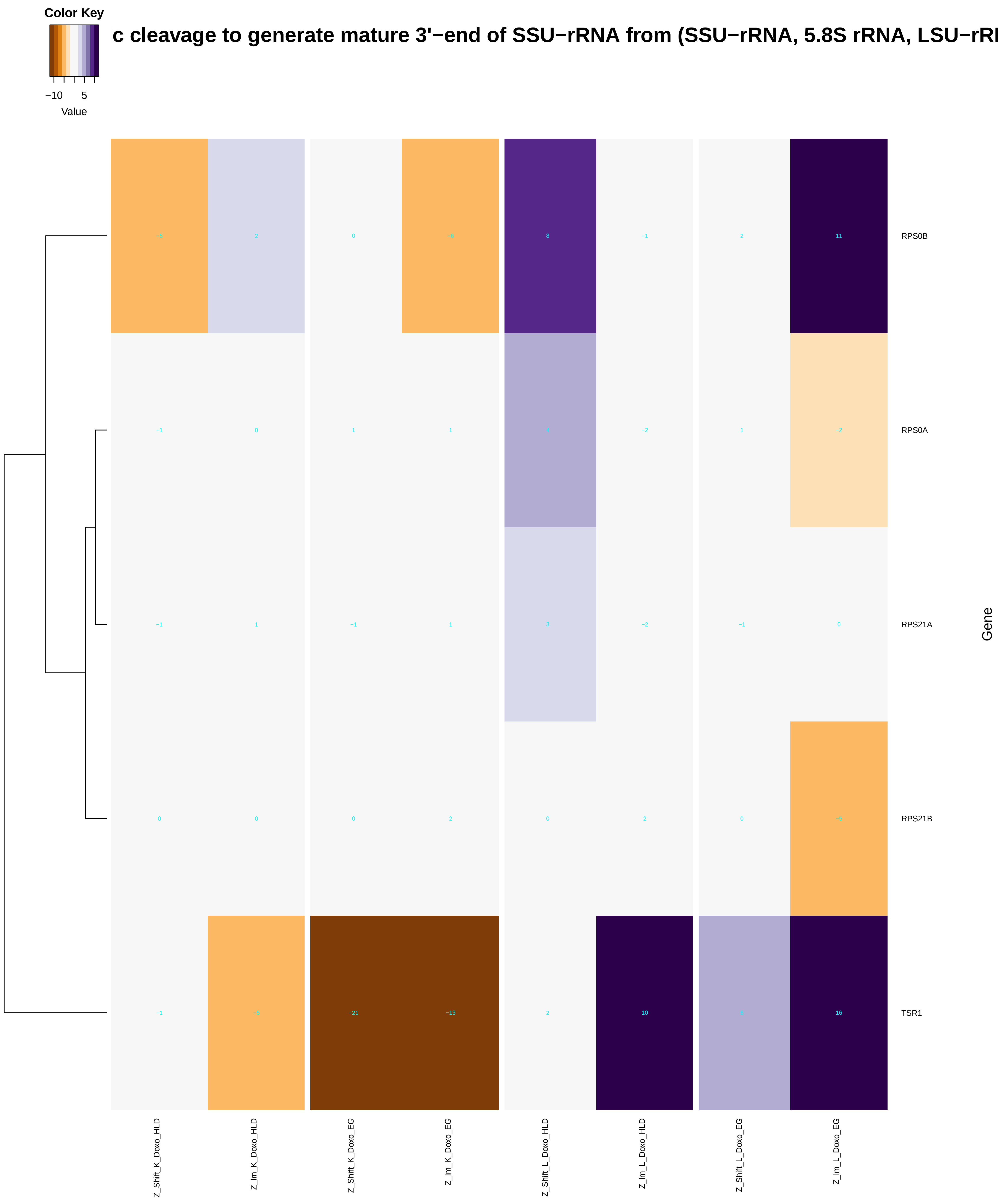
Color Key
−10
5
Value
c cleavage to generate mature 3'−end of SSU−rRNA from (SSU−rRNA, 5.8S rRNA, LSU−rR
−5
2
0
−6
8
−1
2
11
RPS0B
−1
0
1
1
4
−2
1
−2
RPS0A
−1
1
−1
1
3
−2
−1
0
RPS21A
0
0
0
2
0
2
0
−5
RPS21B
−1
−5
−21
−13
2
10
6
16
TSR1
Gene
Z_Shift_K_Doxo_HLD
Z_lm_K_Doxo_HLD
Z_Shift_K_Doxo_EG
Z_lm_K_Doxo_EG
Z_Shift_L_Doxo_HLD
Z_lm_L_Doxo_HLD
Z_Shift_L_Doxo_EG
Z_lm_L_Doxo_EG

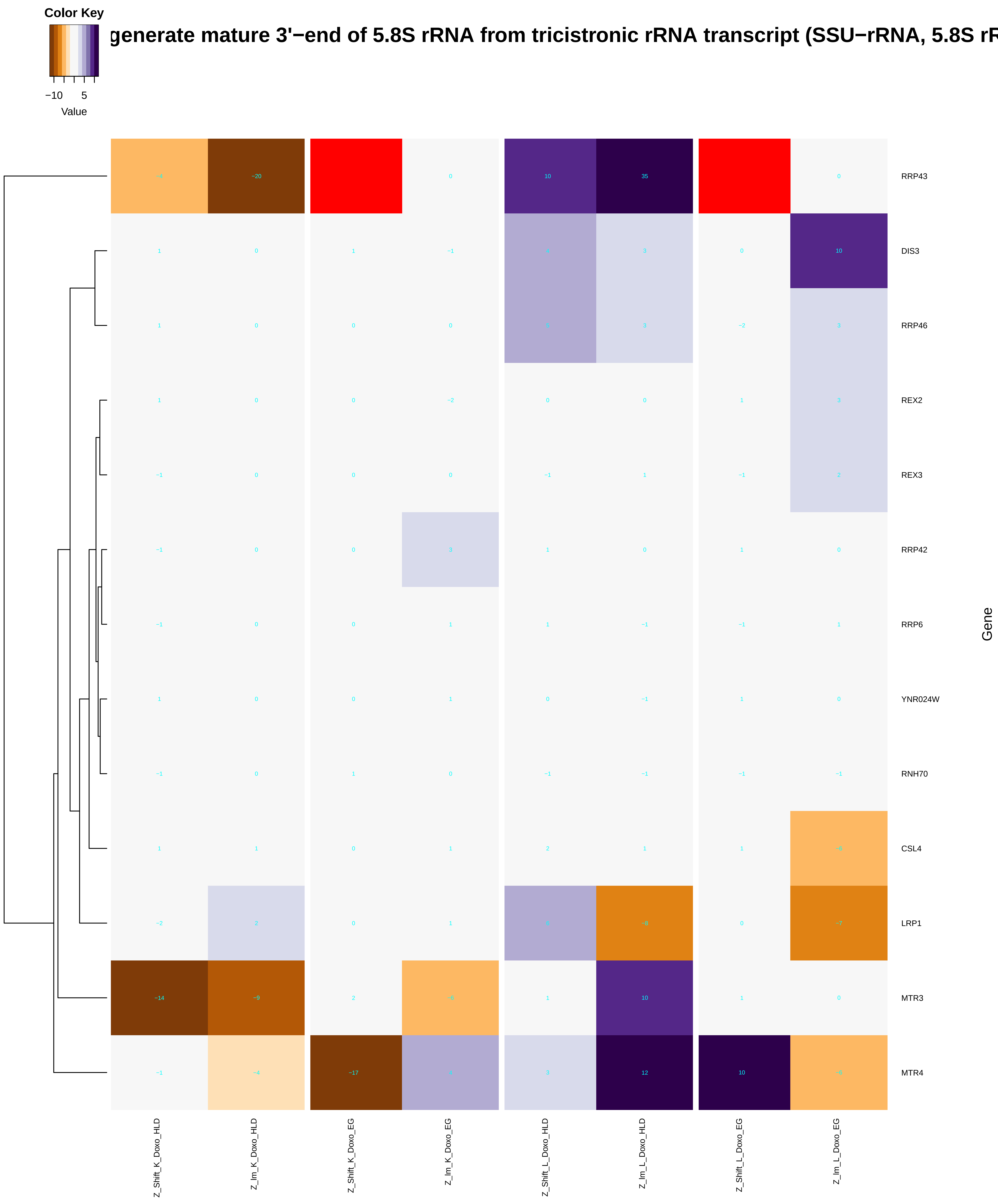
Color Key
−10
5
Value
generate mature 3'−end of 5.8S rRNA from tricistronic rRNA transcript (SSU−rRNA, 5.8S rR
RRP43
DIS3
RRP46
REX2
REX3
RRP42
RRP6
YNR024W
RNH70
CSL4
LRP1
MTR3
MTR4
Gene
Z_Shift_K_Doxo_HLD
Z_lm_K_Doxo_HLD
Z_Shift_K_Doxo_EG
Z_lm_K_Doxo_EG
Z_Shift_L_Doxo_HLD
Z_lm_L_Doxo_HLD
Z_Shift_L_Doxo_EG
Z_lm_L_Doxo_EG

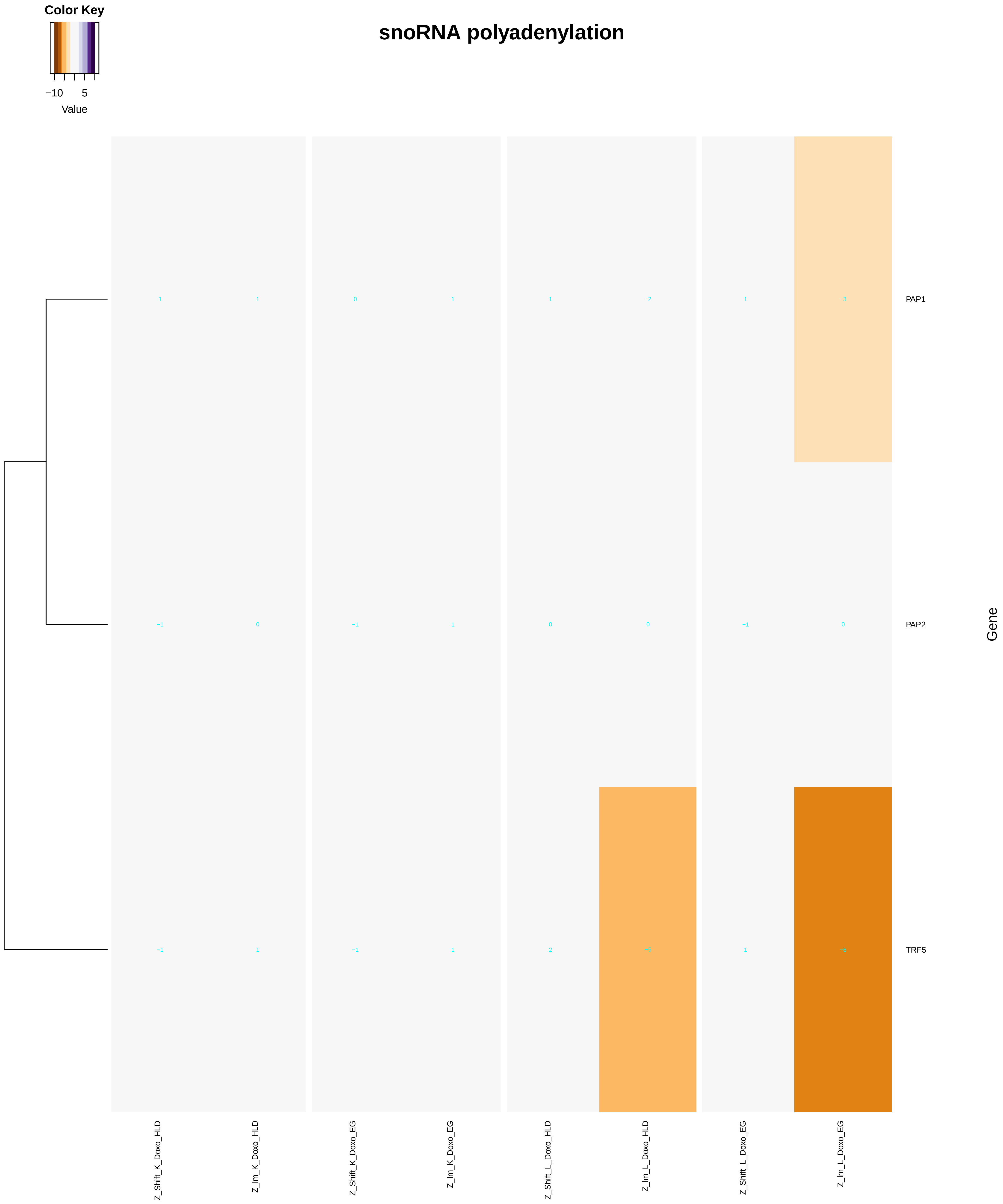
Color Key
−10
5
Value
snoRNA polyadenylation
PAP1
PAP2
TRF5
Gene
Z_Shift_K_Doxo_HLD
Z_Im_K_Doxo_HLD
Z_Shift_K_Doxo_EG
Z_Im_K_Doxo_EG
Z_Shift_L_Doxo_HLD
Z_Im_L_Doxo_HLD
Z_Shift_L_Doxo_EG
Z_Im_L_Doxo_EG

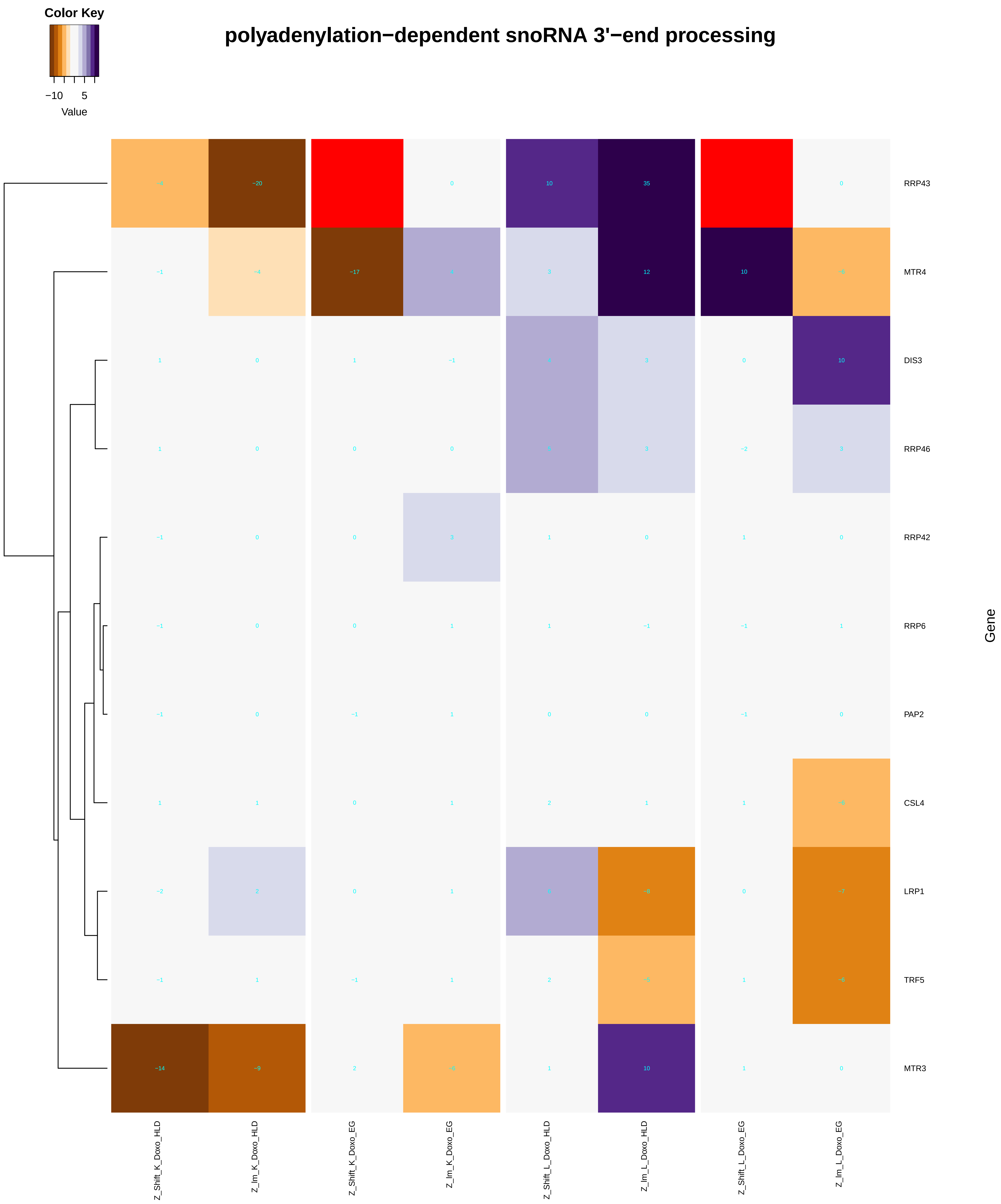
polyadenylation−dependent snoRNA 3'−end processing
Color Key
−10
5
Value
RRP43
MTR4
DIS3
RRP46
RRP42
RRP6
PAP2
CSL4
LRP1
TRF5
MTR3
Gene
Z_Shift_K_Doxo_HLD
Z_Im_K_Doxo_HLD
Z_Shift_K_Doxo_EG
Z_Im_K_Doxo_EG
Z_Shift_L_Doxo_HLD
Z_Im_L_Doxo_HLD
Z_Shift_L_Doxo_EG
Z_Im_L_Doxo_EG

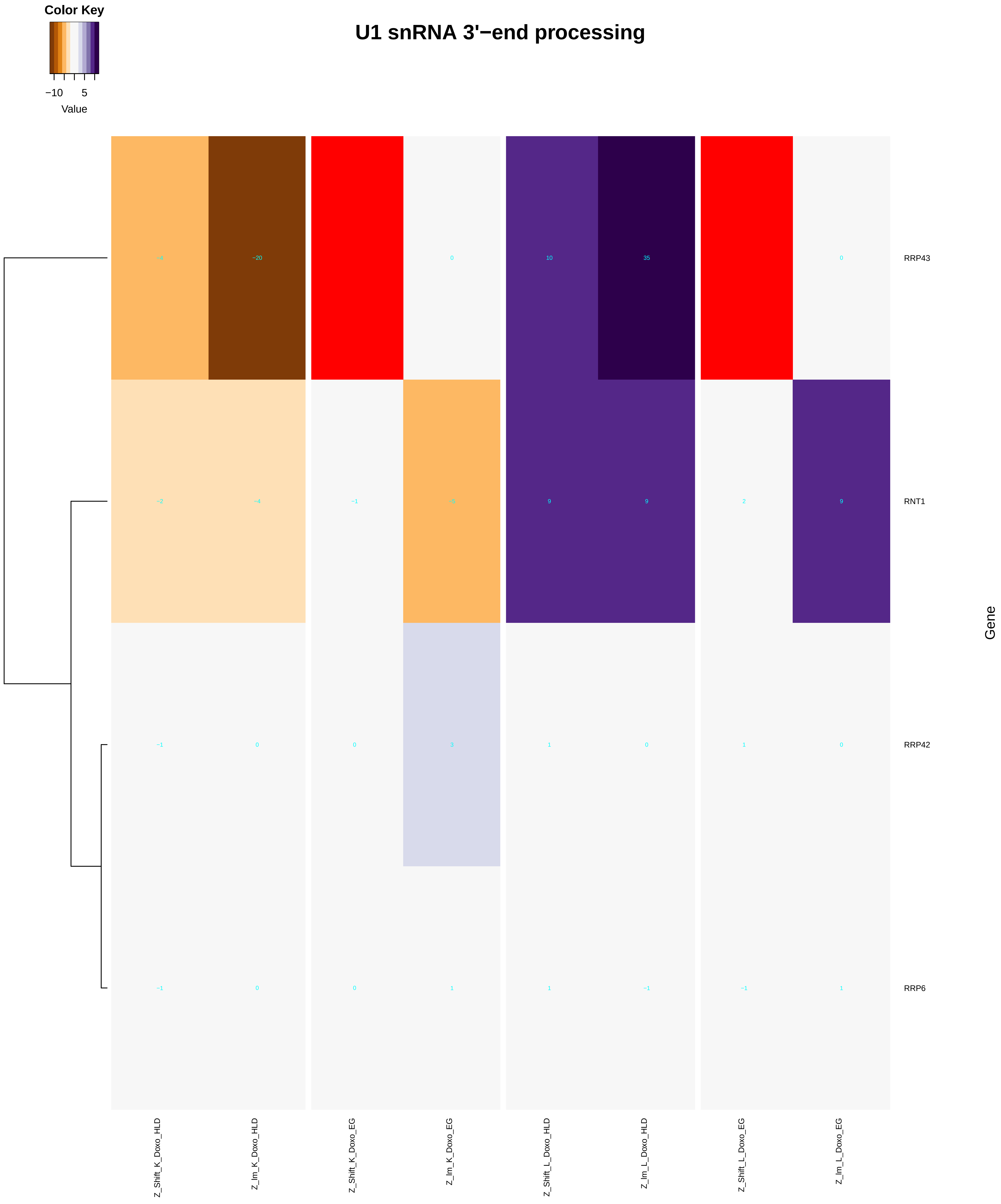
Color Key
−10
5
Value
U1 snRNA 3'−end processing
−4
−20
0
10
35
0
RRP43
−2
−4
−1
−5
9
9
2
9
RNT1
−1
0
0
3
1
0
1
0
RRP42
−1
0
0
1
1
−1
−1
1
RRP6
Gene
Z_Shift_K_Doxo_HLD
Z_Im_K_Doxo_HLD
Z_Shift_K_Doxo_EG
Z_Im_K_Doxo_EG
Z_Shift_L_Doxo_HLD
Z_Im_L_Doxo_HLD
Z_Shift_L_Doxo_EG
Z_Im_L_Doxo_EG

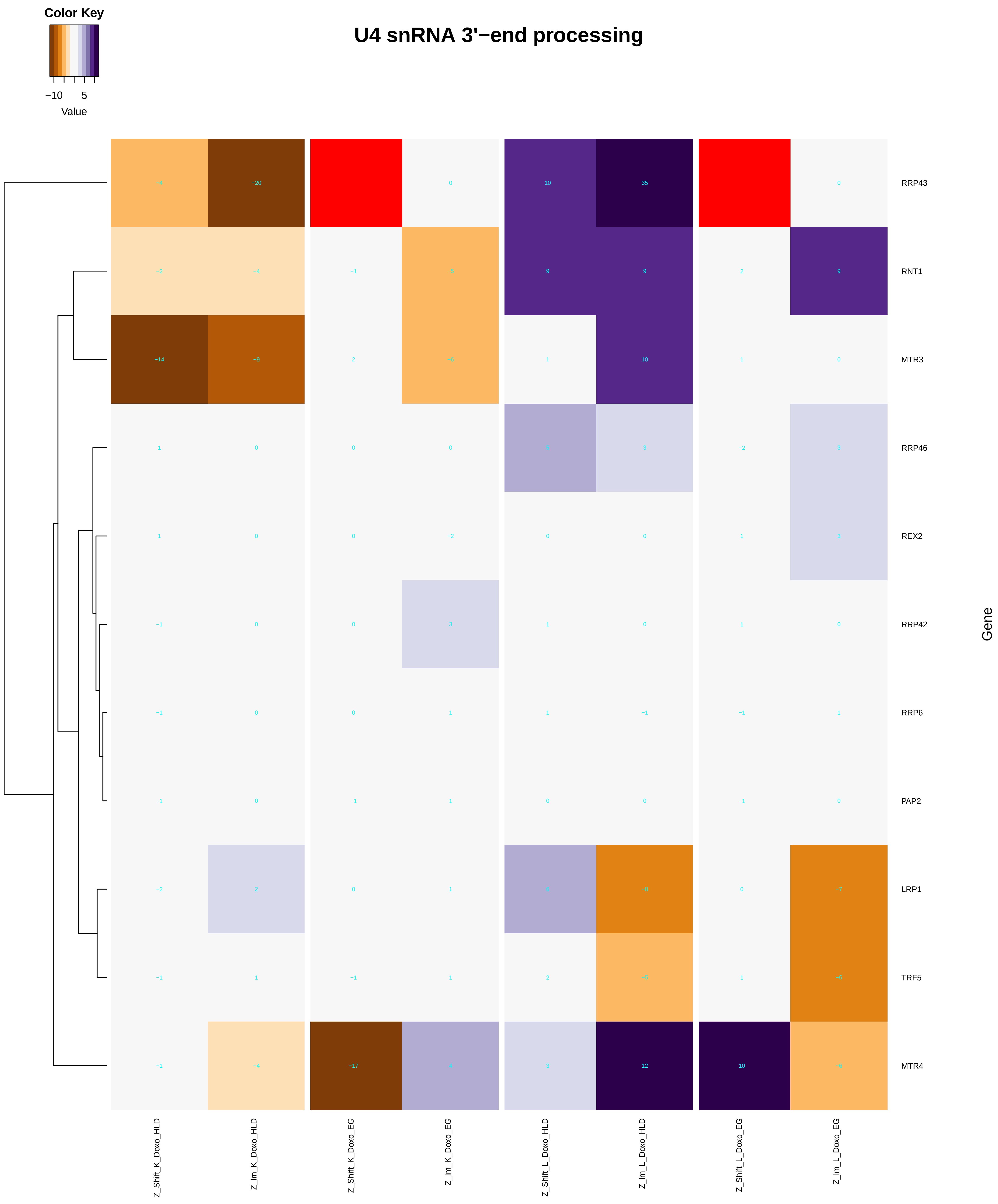
Color Key
−10 5
Value
U4 snRNA 3'−end processing
RRP43
RNT1
MTR3
RRP46
REX2
RRP42
RRP6
PAP2
LRP1
TRF5
MTR4
Gene
Z_Shift_K_Doxo_HLD
Z_lm_K_Doxo_HLD
Z_Shift_K_Doxo_EG
Z_lm_K_Doxo_EG
Z_Shift_L_Doxo_HLD
Z_lm_L_Doxo_HLD
Z_Shift_L_Doxo_EG
Z_lm_L_Doxo_EG

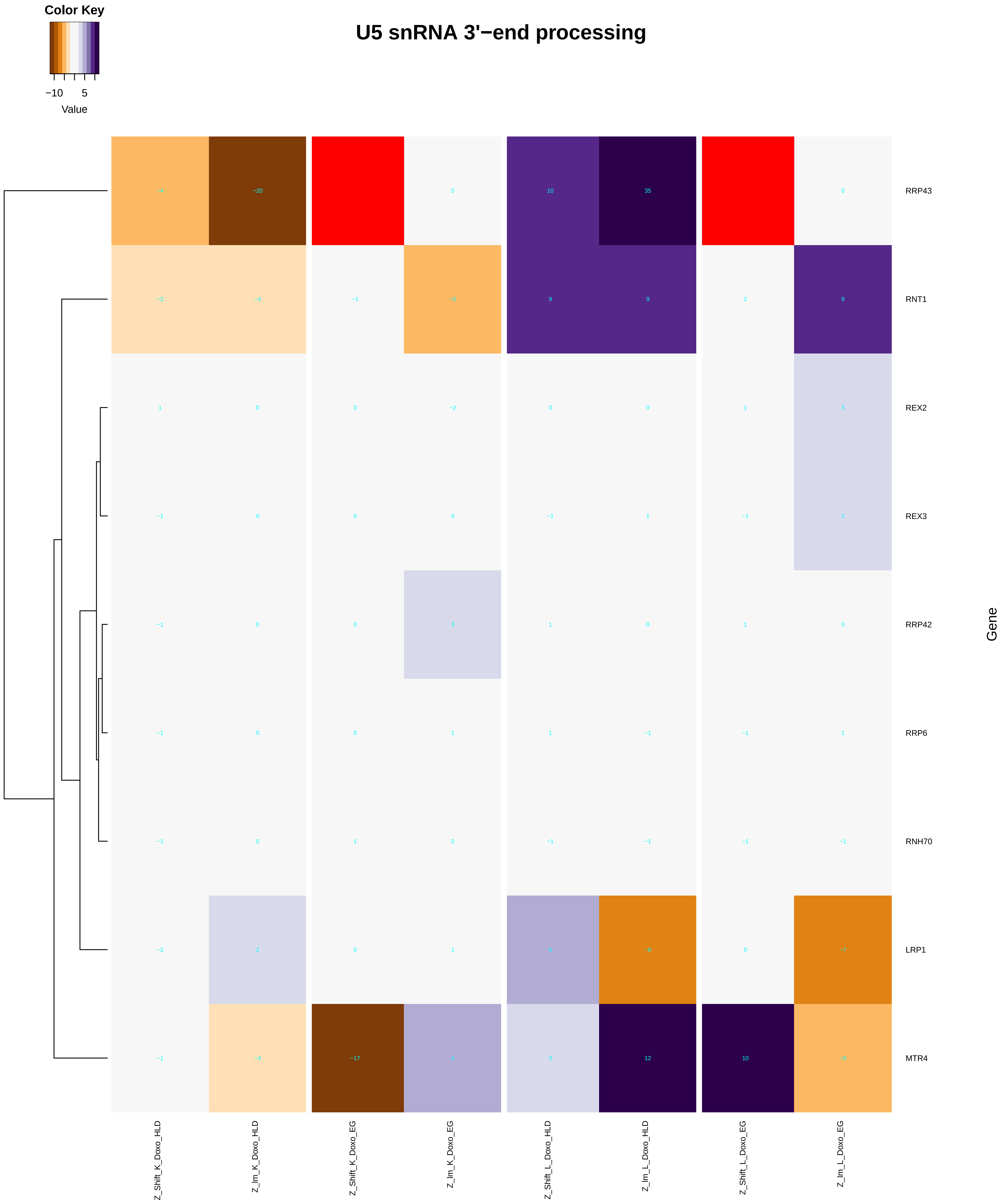
U5 snRNA 3'−end processing
Color Key
−10
5
Value
RRP43
RNT1
REX2
REX3
RRP42
RRP6
RNH70
LRP1
MTR4
Gene
Z_Shift_K_Doxo_HLD
Z_Im_K_Doxo_HLD
Z_Shift_K_Doxo_EG
Z_Im_K_Doxo_EG
Z_Shift_L_Doxo_HLD
Z_Im_L_Doxo_HLD
Z_Shift_L_Doxo_EG
Z_Im_L_Doxo_EG
