# Supplementary material for: A yeast phenomic model for the influence of Warburg metabolism on genetic buffering of doxorubicin
Source: Cancer Metab. 2019 Oct 23;7:9. doi: 10.1186/s40170-019-0201-3 (PMC6806529; doi:10.1186/s40170-019-0201-3)
Supplement: Supplementary file 9 — Additional file 9. GO term-specific heatmaps for REMc/GTF-enriched clusters. GO term-specific heatmaps for significant GO process terms were generated as described in methods and Figs. 3 and 4. Any related child terms are presented in subsequent pages of the parent file name. GO terms with more than 100 children, with 2 or fewer genes annotated to the term, or a file size over 300KB are not shown. All heatmaps are generated with the same layout (see Figs. 3 and 4). [file 40170_2019_201_MOESM9_ESM.bz2 › Additional_File9_GOTermHeatmaps/Additional_File9_GOTermHeatmap/ncRNA_transcription.pdf]

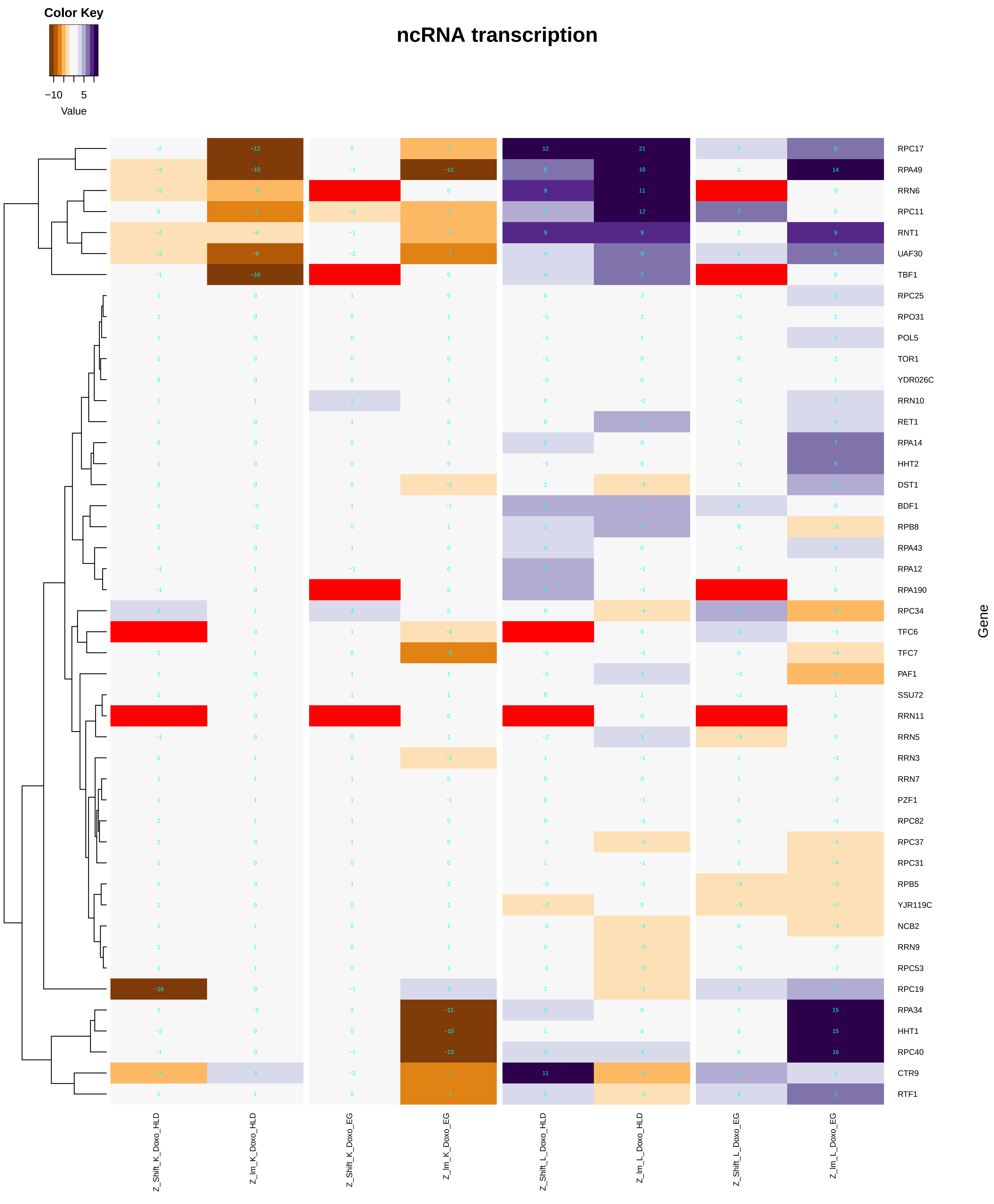
ncRNA transcription
Color Key
−10
5
Value
RPC17
RPA49
RRN6
RPC11
RNT1
UAF30
TBF1
RPC25
RPO31
POL5
TOR1
YDR026C
RRN10
RET1
RPA14
HHT2
DST1
BDF1
RPB8
RPA43
RPA12
RPA190
RPC34
TFC6
TFC7
PAF1
SSU72
RRN11
RRN5
RRN3
RRN7
PZF1
RPC82
RPC37
RPC31
RPB5
YJR119C
NCB2
RRN9
RPC53
RPC19
RPA34
HHT1
RPC40
CTR9
RTF1
Gene
Z_Shift_K_Doxo_HLD
Z_lm_K_Doxo_HLD
Z_Shift_K_Doxo_EG
Z_lm_K_Doxo_EG
Z_Shift_L_Doxo_HLD
Z_lm_L_Doxo_HLD
Z_Shift_L_Doxo_EG
Z_lm_L_Doxo_EG

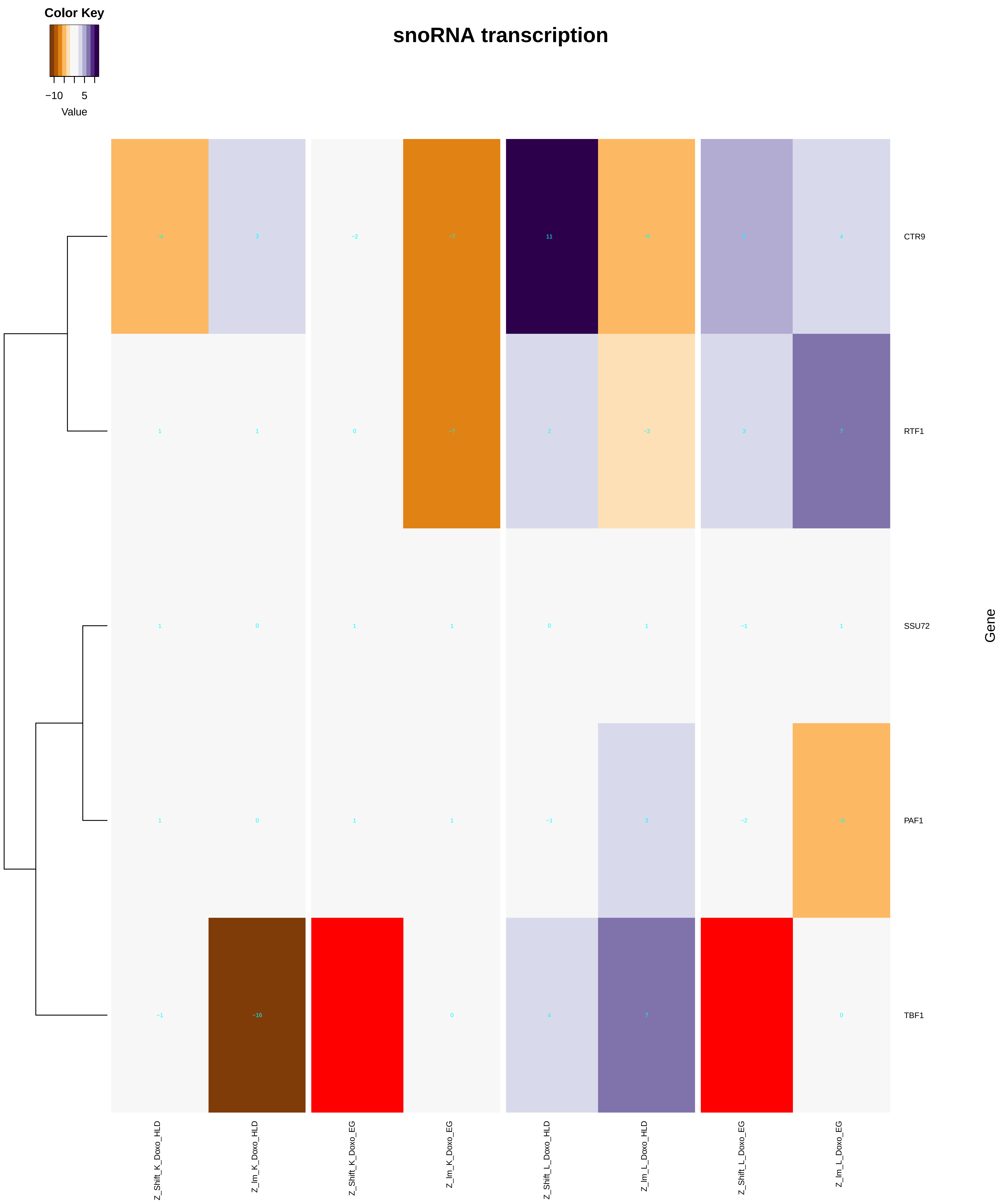
Color Key
−10
5
Value
snoRNA transcription
CTR9
RTF1
SSU72
PAF1
TBF1
Gene
Z_Shift_K_Doxo_HLD
Z_Im_K_Doxo_HLD
Z_Shift_K_Doxo_EG
Z_Im_K_Doxo_EG
Z_Shift_L_Doxo_HLD
Z_Im_L_Doxo_HLD
Z_Shift_L_Doxo_EG
Z_Im_L_Doxo_EG

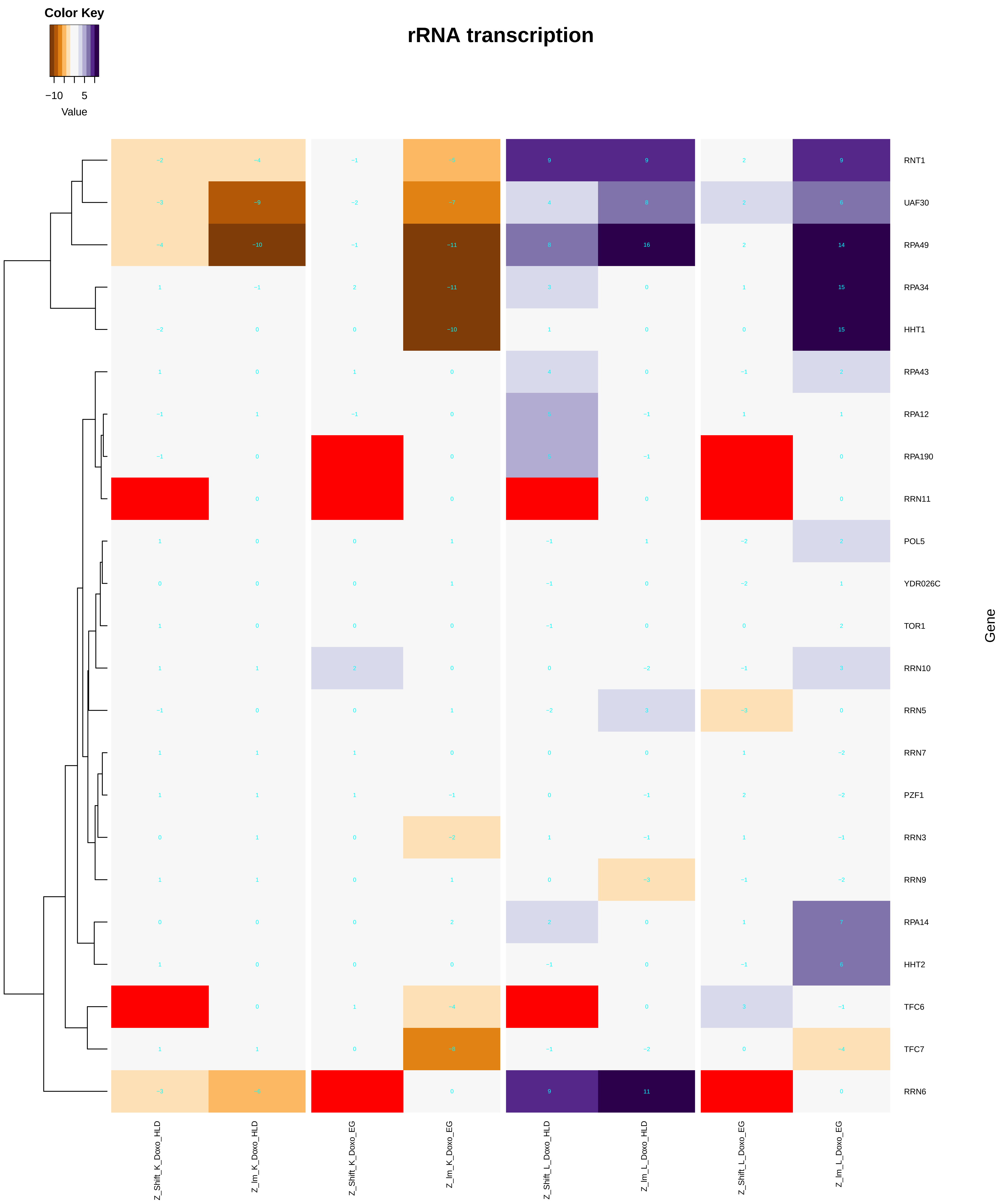

rRNA transcription
Color Key
−10
5
Value
Gene
RNT1
UAF30
RPA49
RPA34
HHT1
RPA43
RPA12
RPA190
RRN11
POL5
YDR026C
TOR1
RRN10
RRN5
RRN7
PZF1
RRN3
RRN9
RPA14
HHT2
TFC6
TFC7
RRN6
Z_Shift_K_Doxo_HLD
Z_lm_K_Doxo_HLD
Z_Shift_K_Doxo_EG
Z_lm_K_Doxo_EG
Z_Shift_L_Doxo_HLD
Z_lm_L_Doxo_HLD
Z_Shift_L_Doxo_EG
Z_lm_L_Doxo_EG

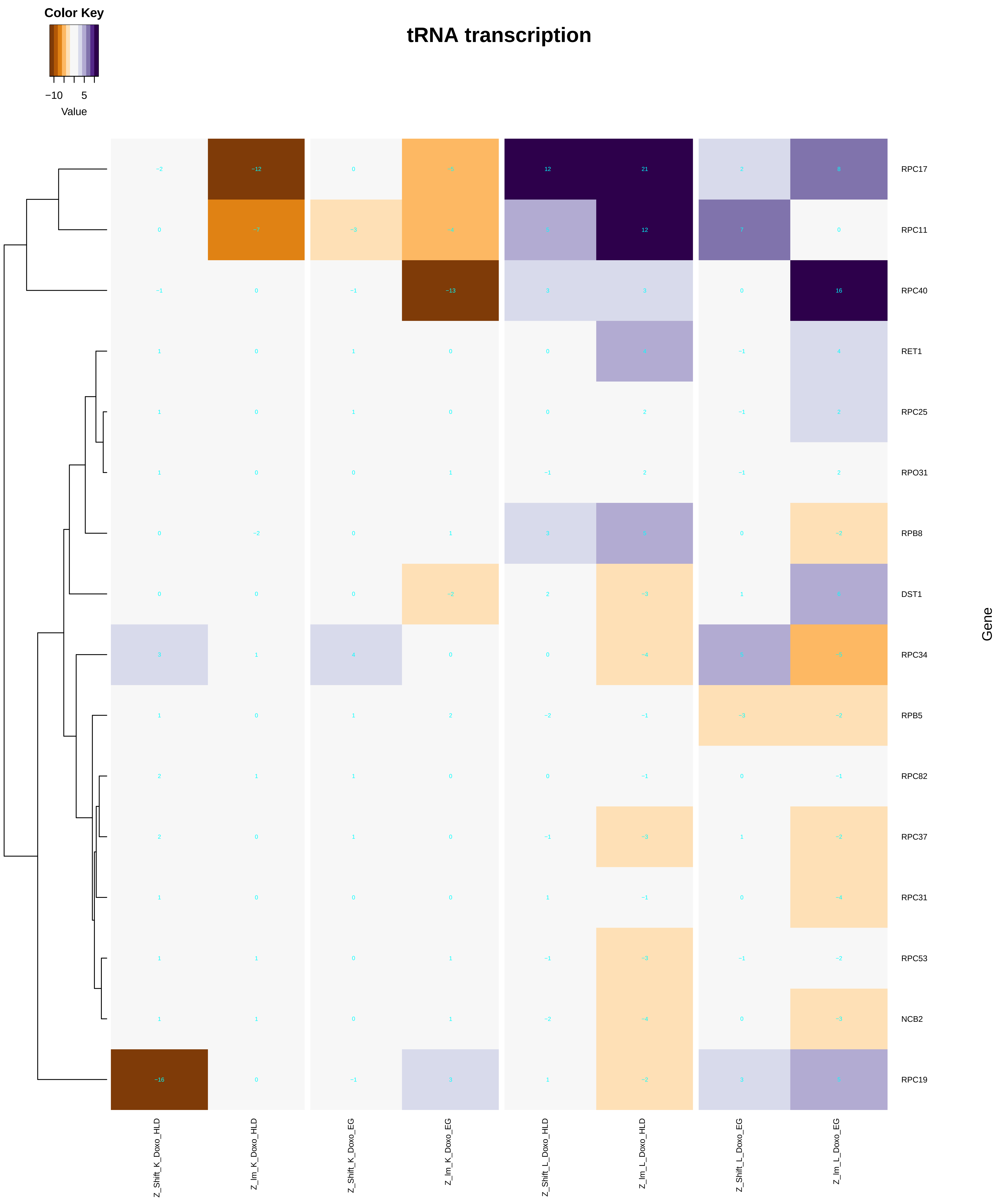

Color Key
−10
5
Value
tRNA transcription
RPC17
RPC11
RPC40
RET1
RPC25
RPO31
RPB8
DST1
RPC34
RPB5
RPC82
RPC37
RPC31
RPC53
NCB2
RPC19
Gene
Z_Shift_K_Doxo_HLD
Z_Im_K_Doxo_HLD
Z_Shift_K_Doxo_EG
Z_Im_K_Doxo_EG
Z_Shift_L_Doxo_HLD
Z_Im_L_Doxo_HLD
Z_Shift_L_Doxo_EG
Z_Im_L_Doxo_EG

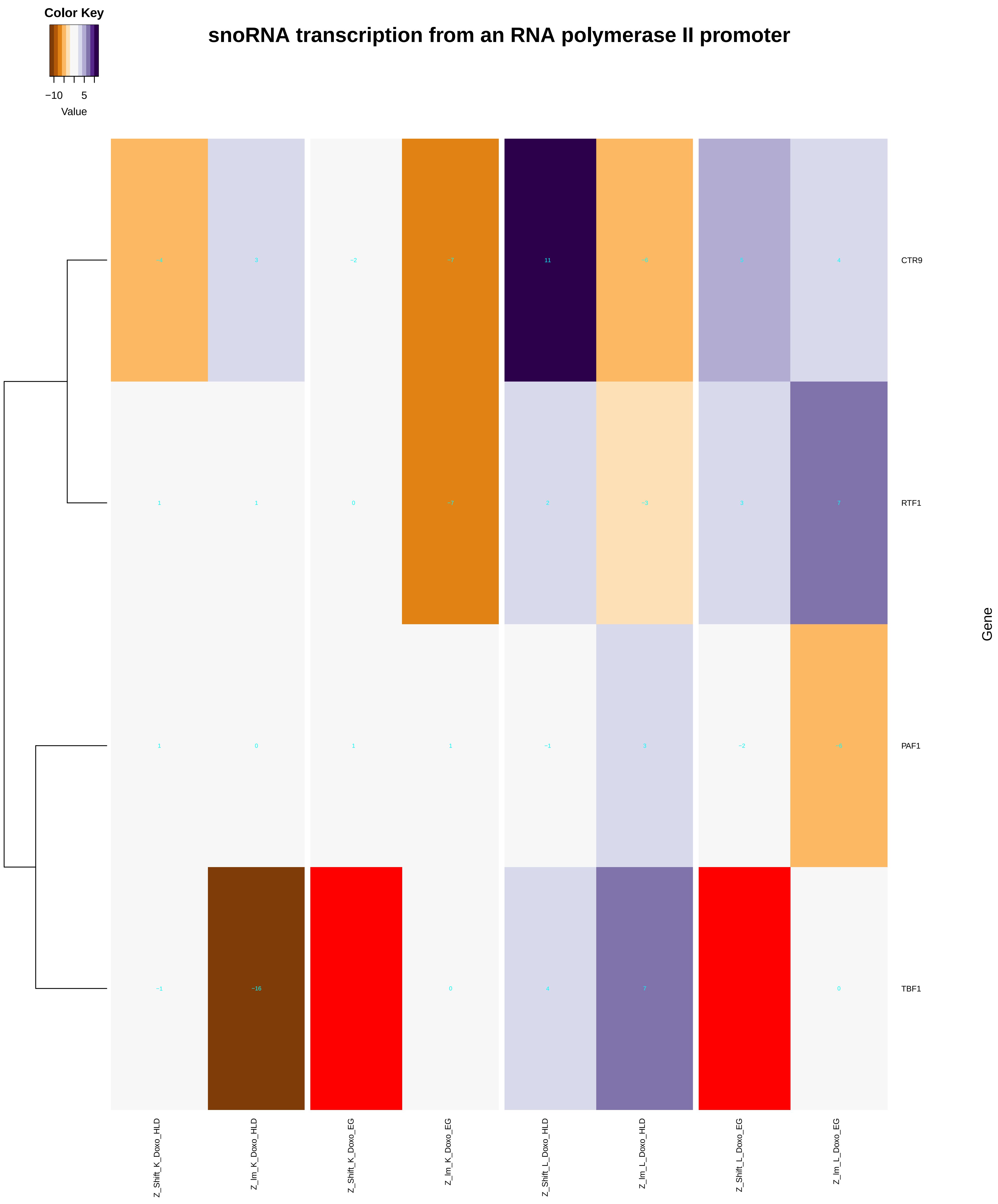
Color Key
−10
5
Value
snoRNA transcription from an RNA polymerase II promoter
CTR9
RTF1
PAF1
TBF1
Gene
Z_Shift_K_Doxo_HLD
Z_Im_K_Doxo_HLD
Z_Shift_K_Doxo_EG
Z_Im_K_Doxo_EG
Z_Shift_L_Doxo_HLD
Z_Im_L_Doxo_HLD
Z_Shift_L_Doxo_EG
Z_Im_L_Doxo_EG

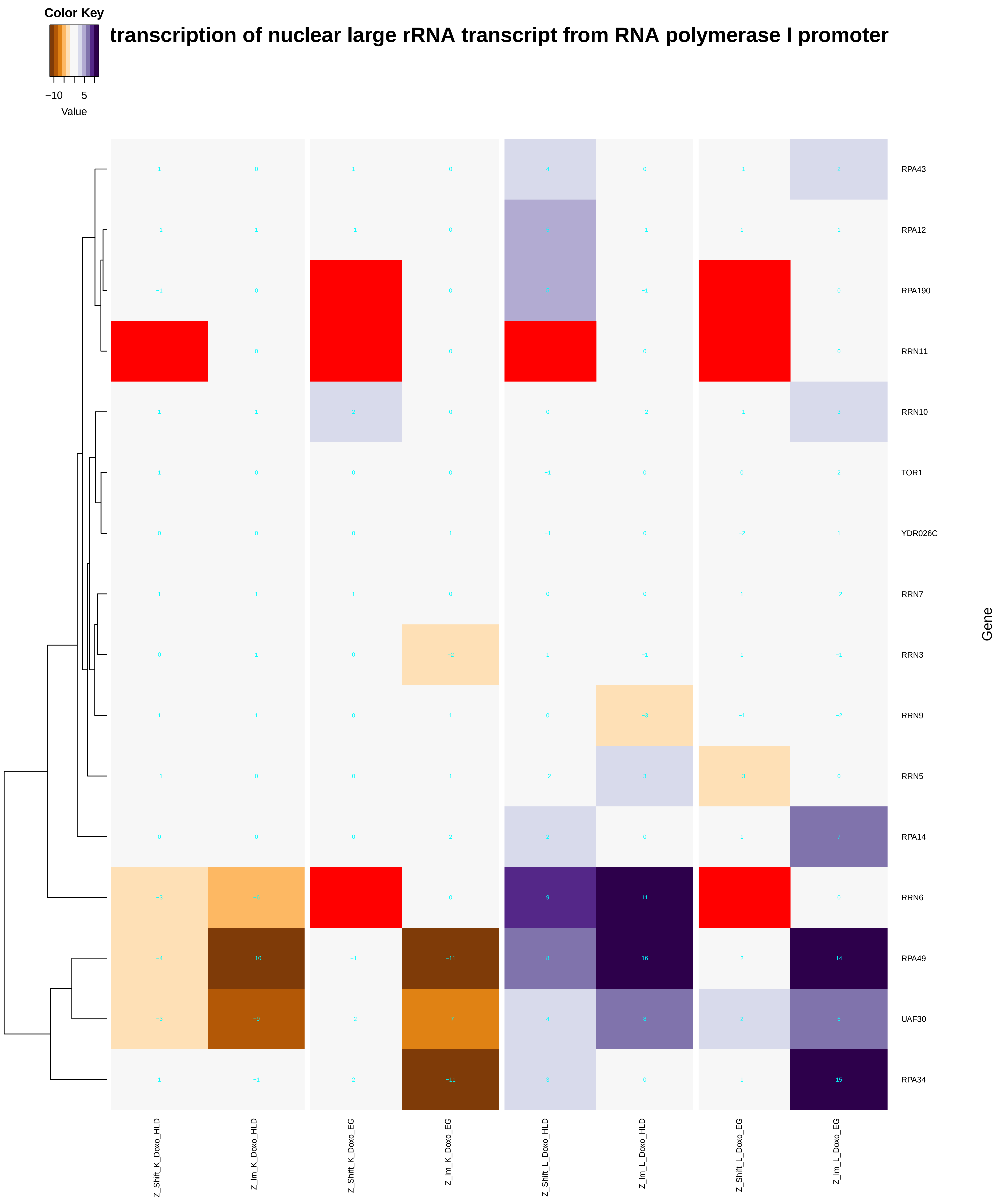

Color Key
−10
5
Value
transcription of nuclear large rRNA transcript from RNA polymerase I promoter
RPA43
RPA12
RPA190
RRN11
RRN10
TOR1
YDR026C
RRN7
RRN3
RRN9
RRN5
RPA14
RRN6
RPA49
UAF30
RPA34
Gene
Z_Shift_K_Doxo_HLD
Z_Im_K_Doxo_HLD
Z_Shift_K_Doxo_EG
Z_Im_K_Doxo_EG
Z_Shift_L_Doxo_HLD
Z_Im_L_Doxo_HLD
Z_Shift_L_Doxo_EG
Z_Im_L_Doxo_EG

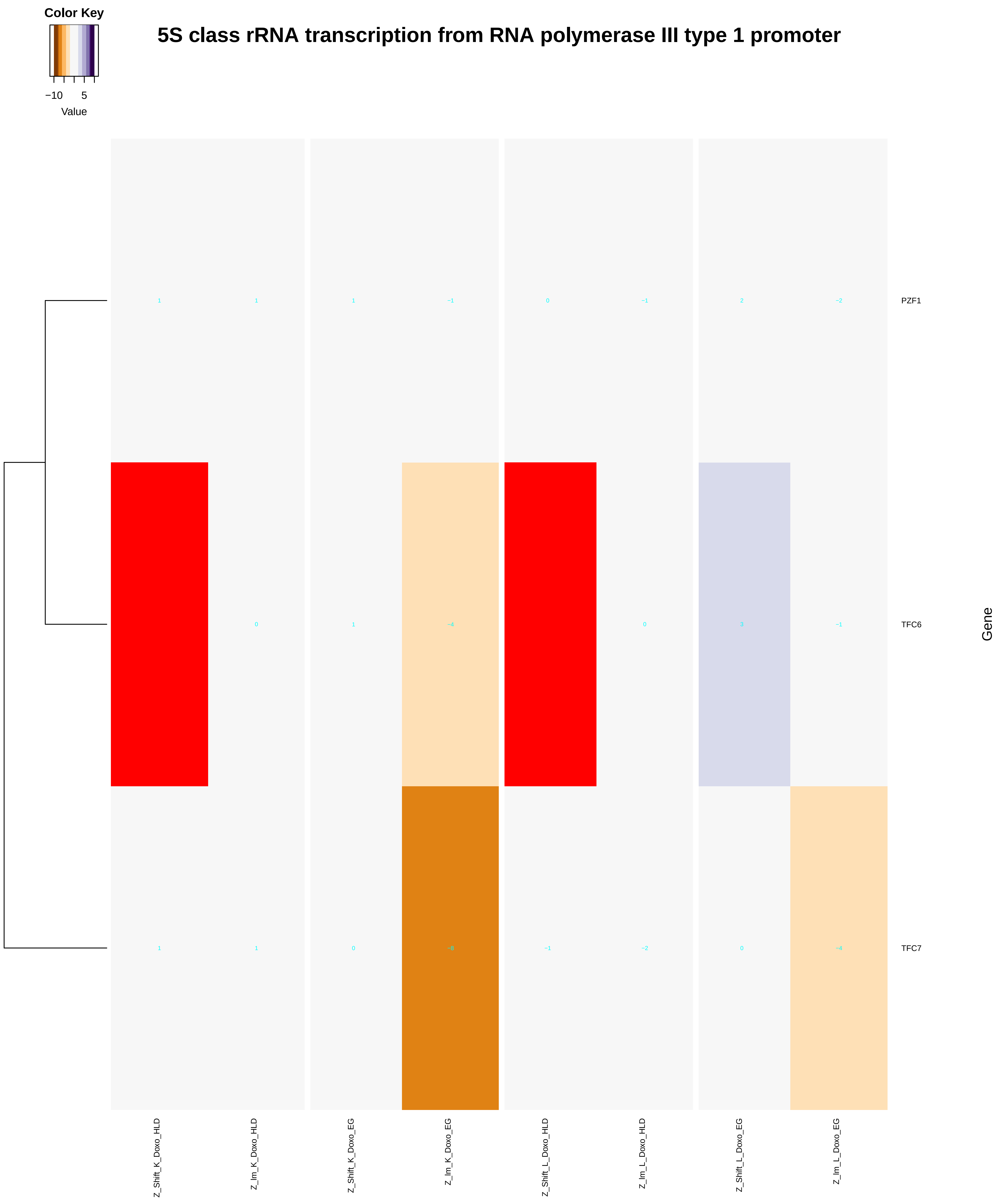

Color Key
−10
5
Value
5S class rRNA transcription from RNA polymerase III type 1 promoter
PZF1
TFC6
TFC7
Gene
Z_Shift_K_Doxo_HLD
Z_Im_K_Doxo_HLD
Z_Shift_K_Doxo_EG
Z_Im_K_Doxo_EG
Z_Shift_L_Doxo_HLD
Z_Im_L_Doxo_HLD
Z_Shift_L_Doxo_EG
Z_Im_L_Doxo_EG

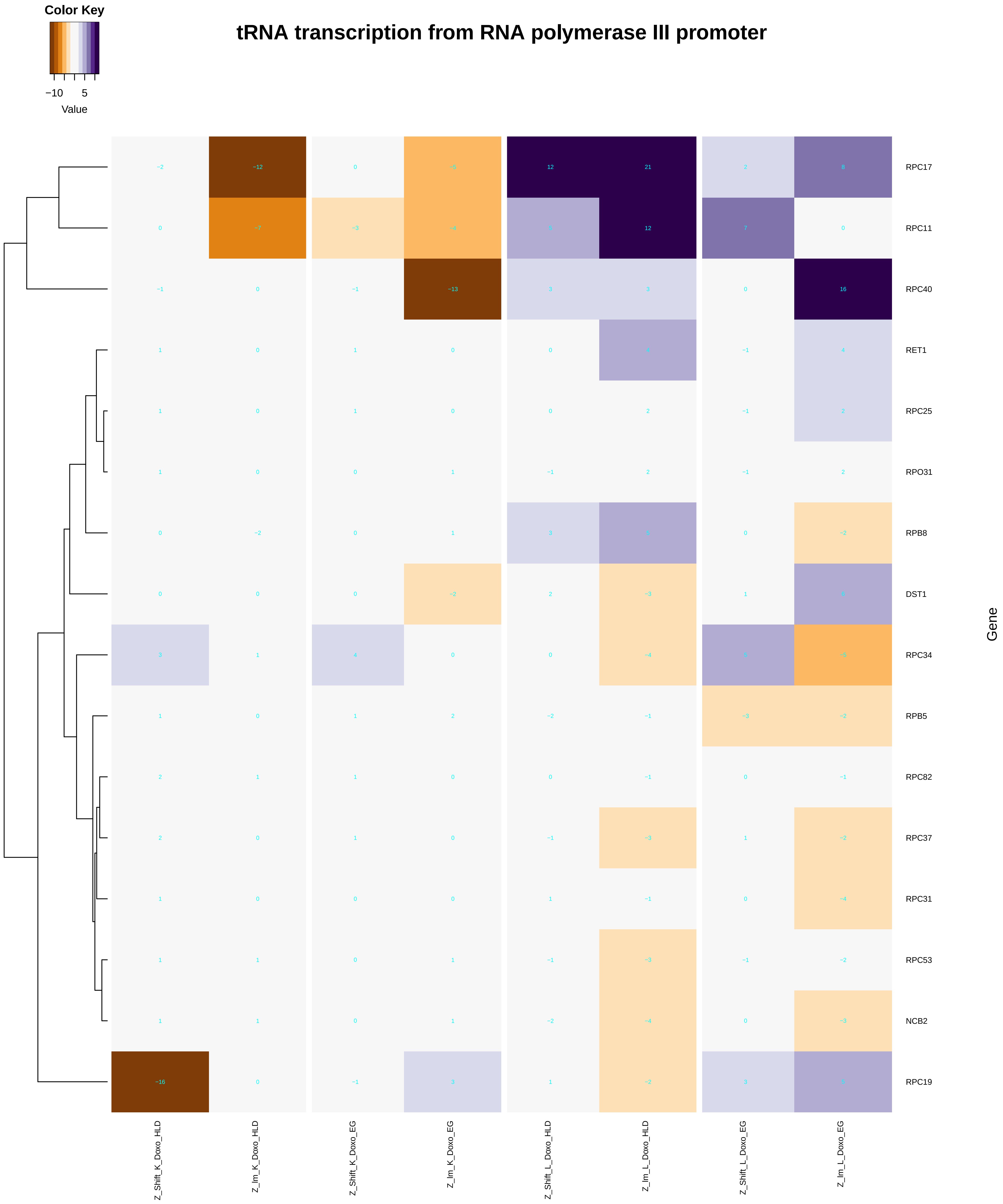
tRNA transcription from RNA polymerase III promoter
Color Key
−10
5
Value
RPC17
RPC11
RPC40
RET1
RPC25
RPO31
RPB8
DST1
RPC34
RPB5
RPC82
RPC37
RPC31
RPC53
NCB2
RPC19
Gene
Z_Shift_K_Doxo_HLD
Z_Im_K_Doxo_HLD
Z_Shift_K_Doxo_EG
Z_Im_K_Doxo_EG
Z_Shift_L_Doxo_HLD
Z_Im_L_Doxo_HLD
Z_Shift_L_Doxo_EG
Z_Im_L_Doxo_EG
