# Supplementary material for: A yeast phenomic model for the influence of Warburg metabolism on genetic buffering of doxorubicin
Source: Cancer Metab. 2019 Oct 23;7:9. doi: 10.1186/s40170-019-0201-3 (PMC6806529; doi:10.1186/s40170-019-0201-3)
Supplement: Supplementary file 9 — Additional file 9. GO term-specific heatmaps for REMc/GTF-enriched clusters. GO term-specific heatmaps for significant GO process terms were generated as described in methods and Figs. 3 and 4. Any related child terms are presented in subsequent pages of the parent file name. GO terms with more than 100 children, with 2 or fewer genes annotated to the term, or a file size over 300KB are not shown. All heatmaps are generated with the same layout (see Figs. 3 and 4). [file 40170_2019_201_MOESM9_ESM.bz2 › Additional_File9_GOTermHeatmaps/Additional_File9_GOTermHeatmap/negative_regulation_of_chromatin_silencing.pdf]

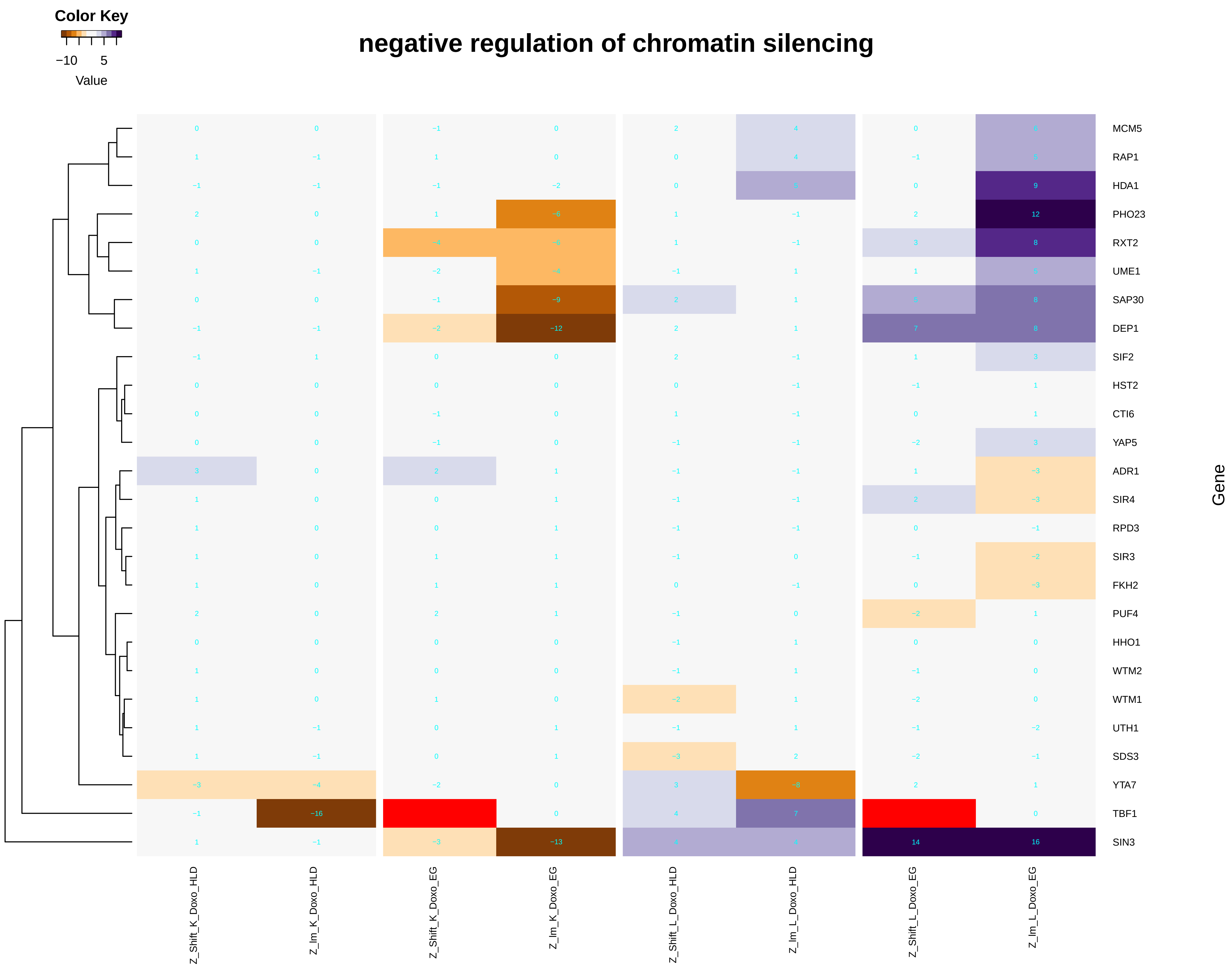
negative regulation of chromatin silencing
Color Key
−10
5
Value
MCM5
RAP1
HDA1
PHO23
RXT2
UME1
SAP30
DEP1
SIF2
HST2
CTI6
YAP5
ADR1
SIR4
RPD3
SIR3
FKH2
PUF4
HHO1
WTM2
WTM1
UTH1
SDS3
YTA7
TBF1
SIN3
Gene
Z_Shift_K_Doxo_HLD
Z_Im_K_Doxo_HLD
Z_Shift_K_Doxo_EG
Z_Im_K_Doxo_EG
Z_Shift_L_Doxo_HLD
Z_Im_L_Doxo_HLD
Z_Shift_L_Doxo_EG
Z_Im_L_Doxo_EG

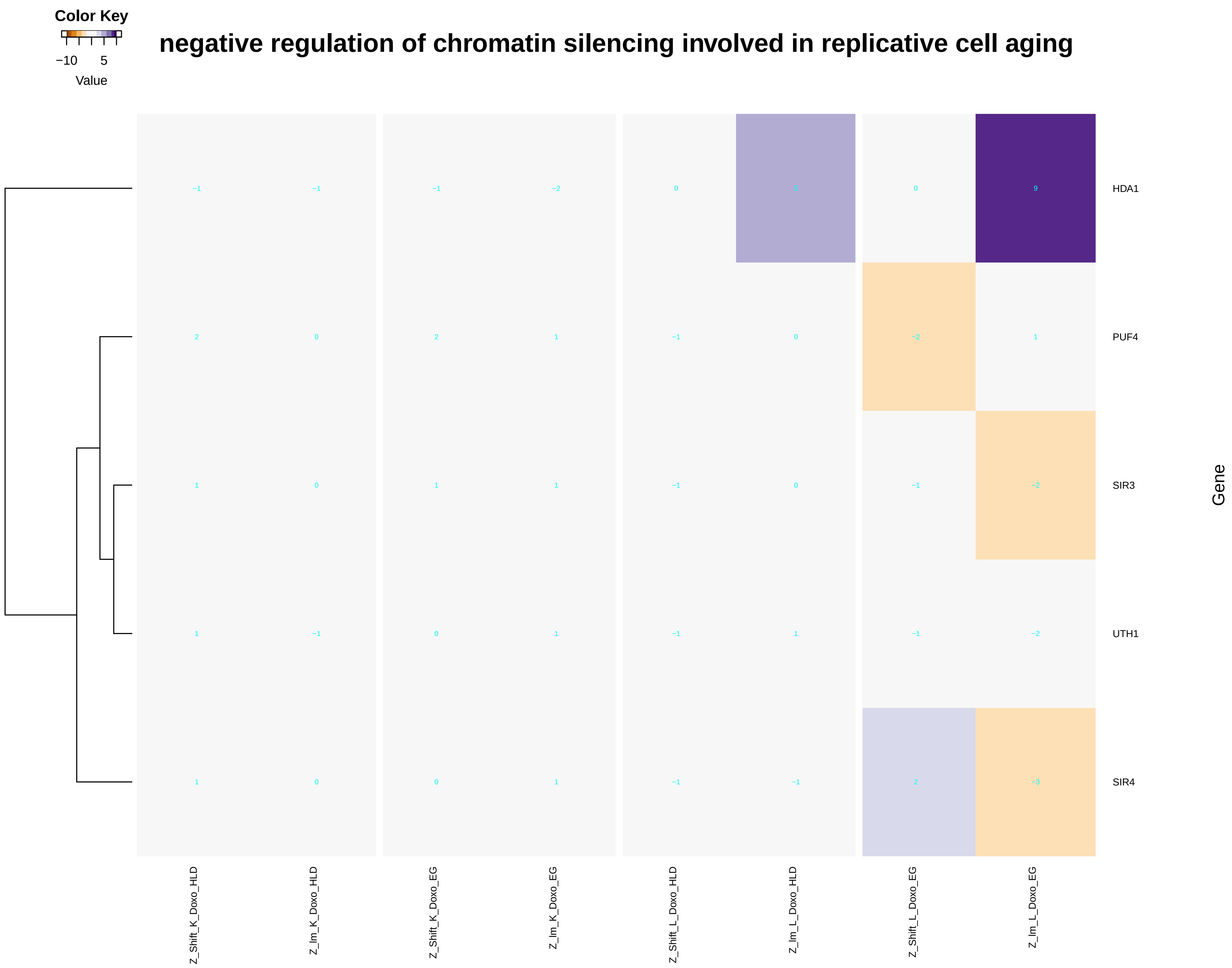

Color Key
−10
5
Value
negative regulation of chromatin silencing involved in replicative cell aging
−1
−1
−1
−2
0
5
0
9
HDA1
2
0
2
1
−1
0
−2
1
PUF4
1
0
1
1
−1
0
−1
−2
SIR3
1
−1
0
1
−1
1
−1
−2
UTH1
1
0
0
1
−1
−1
2
−3
SIR4
Gene
Z_Shift_K_Doxo_HLD
Z_Im_K_Doxo_HLD
Z_Shift_K_Doxo_EG
Z_Im_K_Doxo_EG
Z_Shift_L_Doxo_HLD
Z_Im_L_Doxo_HLD
Z_Shift_L_Doxo_EG
Z_Im_L_Doxo_EG

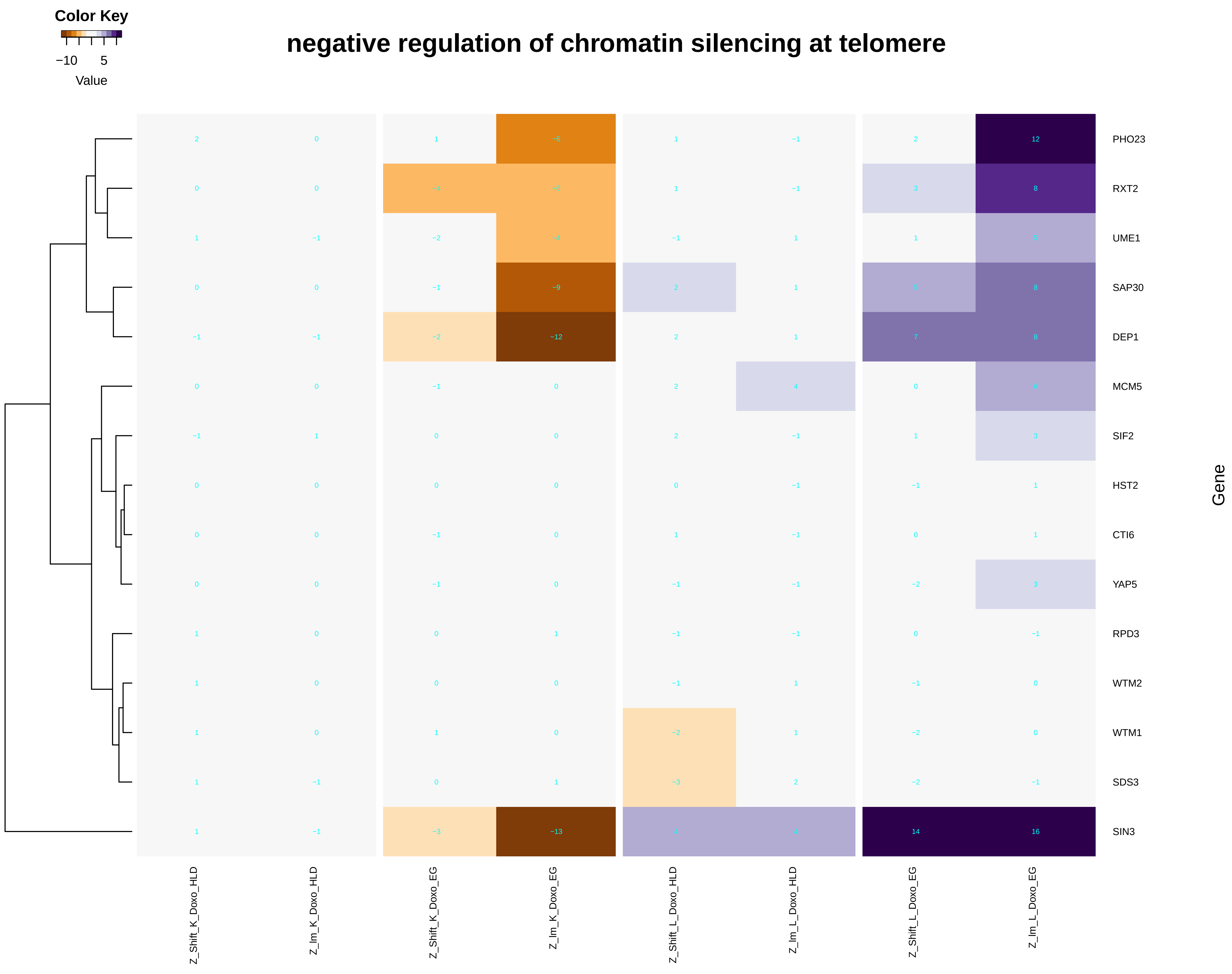

Color Key
−10
5
Value
negative regulation of chromatin silencing at telomere
PHO23
RXT2
UME1
SAP30
DEP1
MCM5
SIF2
HST2
CTI6
YAP5
RPD3
WTM2
WTM1
SDS3
SIN3
Gene
Z_Shift_K_Doxo_HLD
Z_Im_K_Doxo_HLD
Z_Shift_K_Doxo_EG
Z_Im_K_Doxo_EG
Z_Shift_L_Doxo_HLD
Z_Im_L_Doxo_HLD
Z_Shift_L_Doxo_EG
Z_Im_L_Doxo_EG

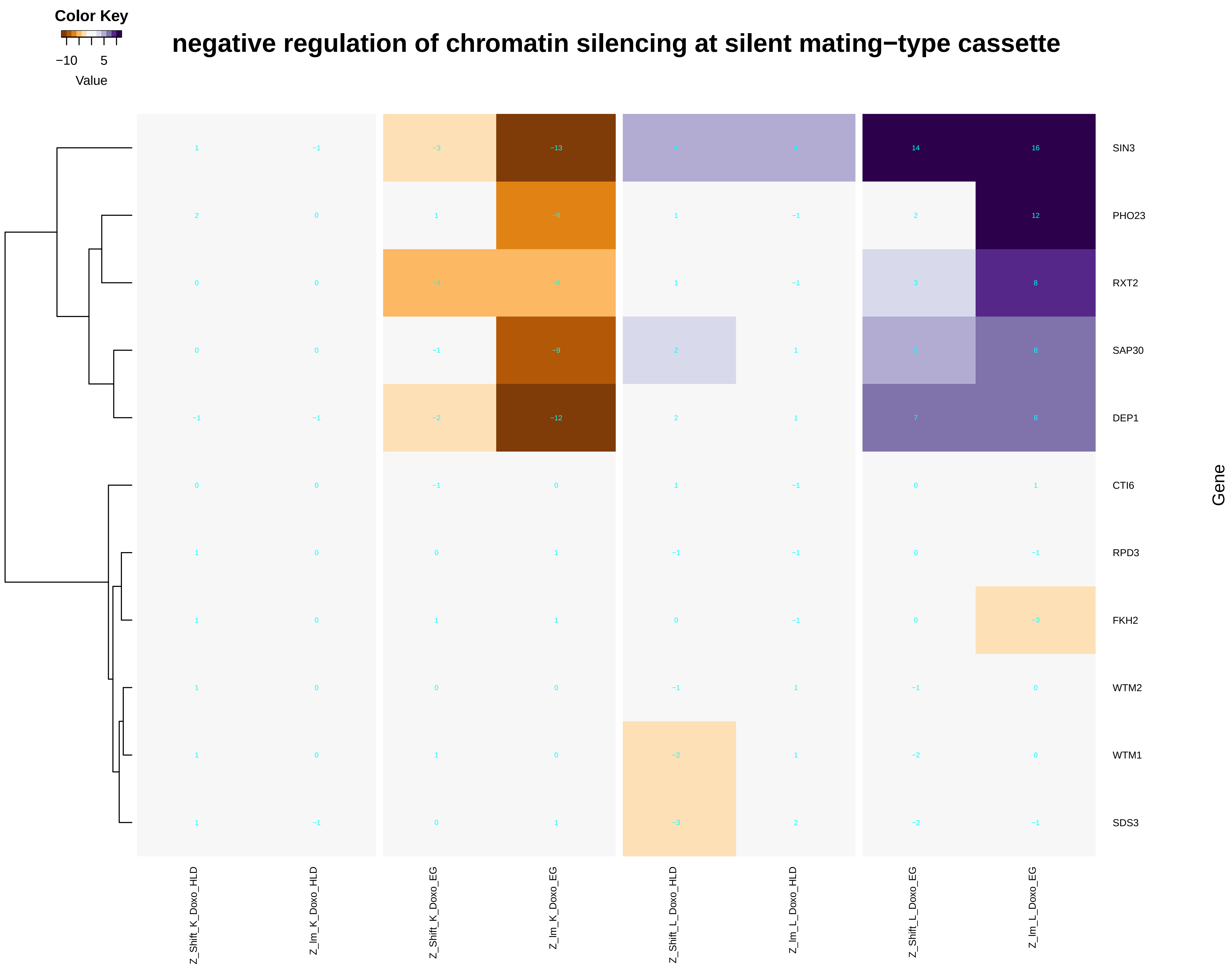

Color Key
−10
5
Value
negative regulation of chromatin silencing at silent mating−type cassette
SIN3
PHO23
RXT2
SAP30
DEP1
CTI6
RPD3
FKH2
WTM2
WTM1
SDS3
Gene
Z_Shift_K_Doxo_HLD
Z_Im_K_Doxo_HLD
Z_Shift_K_Doxo_EG
Z_Im_K_Doxo_EG
Z_Shift_L_Doxo_HLD
Z_Im_L_Doxo_HLD
Z_Shift_L_Doxo_EG
Z_Im_L_Doxo_EG

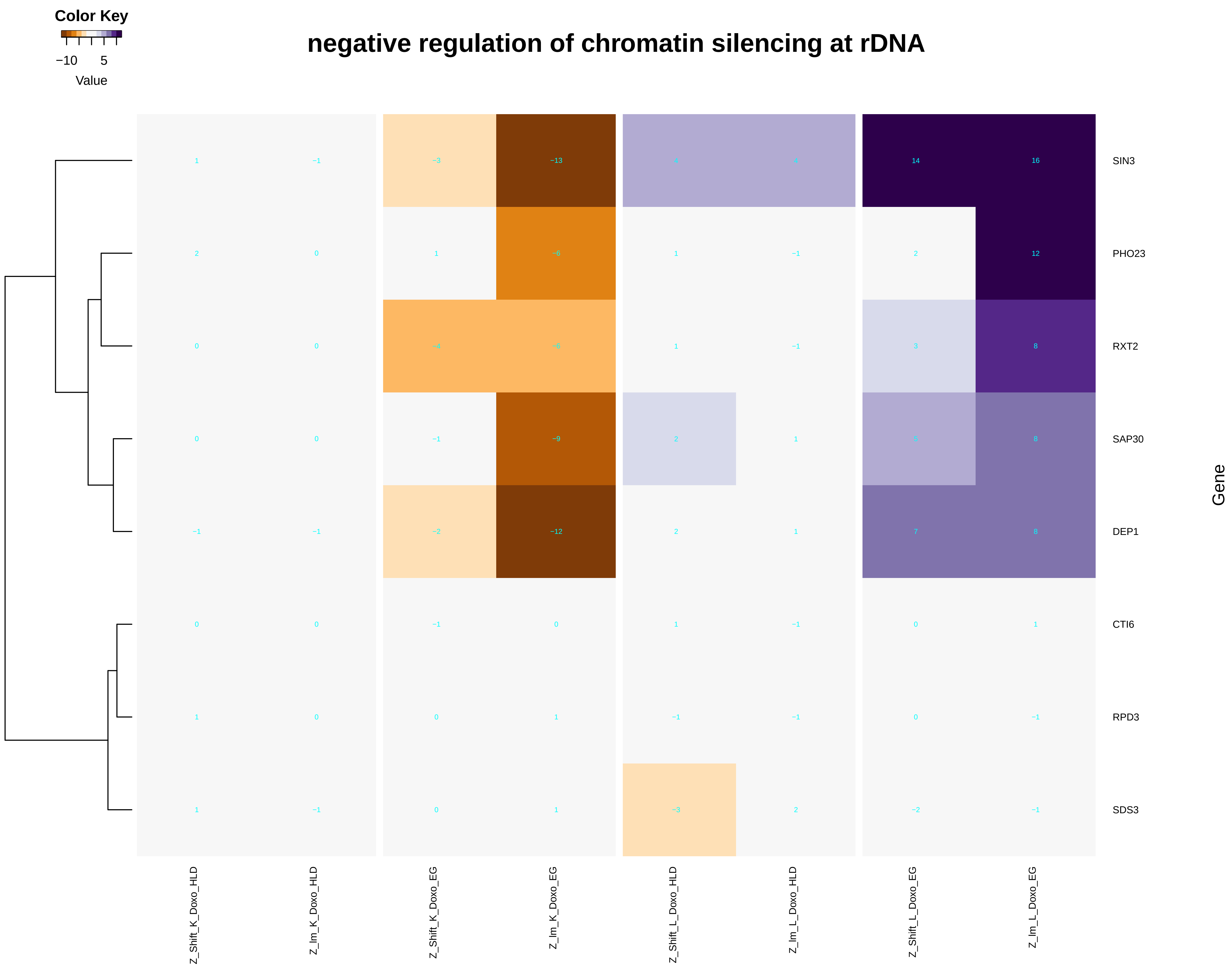

Color Key
−10
5
Value
negative regulation of chromatin silencing at rDNA
SIN3
PHO23
RXT2
SAP30
DEP1
CTI6
RPD3
SDS3
Gene
Z_Shift_K_Doxo_HLD
Z_Im_K_Doxo_HLD
Z_Shift_K_Doxo_EG
Z_Im_K_Doxo_EG
Z_Shift_L_Doxo_HLD
Z_Im_L_Doxo_HLD
Z_Shift_L_Doxo_EG
Z_Im_L_Doxo_EG
