# Supplementary material for: A yeast phenomic model for the influence of Warburg metabolism on genetic buffering of doxorubicin
Source: Cancer Metab. 2019 Oct 23;7:9. doi: 10.1186/s40170-019-0201-3 (PMC6806529; doi:10.1186/s40170-019-0201-3)
Supplement: Supplementary file 9 — Additional file 9. GO term-specific heatmaps for REMc/GTF-enriched clusters. GO term-specific heatmaps for significant GO process terms were generated as described in methods and Figs. 3 and 4. Any related child terms are presented in subsequent pages of the parent file name. GO terms with more than 100 children, with 2 or fewer genes annotated to the term, or a file size over 300KB are not shown. All heatmaps are generated with the same layout (see Figs. 3 and 4). [file 40170_2019_201_MOESM9_ESM.bz2 › Additional_File9_GOTermHeatmaps/Additional_File9_GOTermHeatmap/negative_regulation_of_transcription_from_RNA_polymerase_II_promoter.pdf]

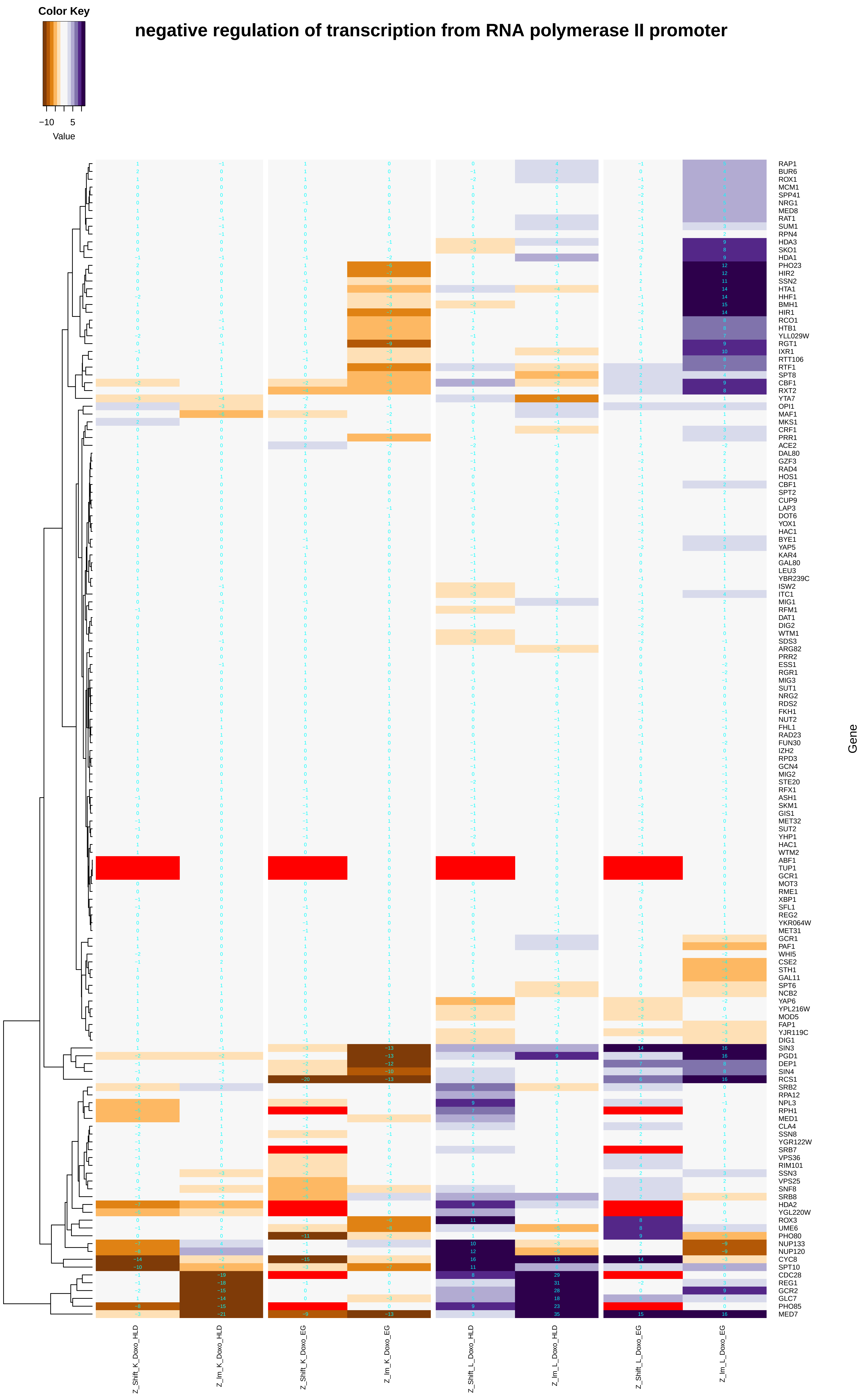
Color Key
−10
5
Value
negative regulation of transcription from RNA polymerase II promoter
Z_Shift_K_Doxo_HLD
Z_Im_K_Doxo_HLD
Z_Shift_K_Doxo_EG
Z_Im_K_Doxo_EG
Z_Shift_L_Doxo_HLD
Z_Im_L_Doxo_HLD
Z_Shift_L_Doxo_EG
Z_Im_L_Doxo_EG
Gene
RAP1
BUR6
ROX1
MCM1
SPP41
NRG1
MED8
RAT1
SUM1
RPN4
HDA3
SKO1
HDA1
PHO23
HIR2
SSN2
HTA1
HHF1
BMH1
HIR1
RCO1
HTB1
YLL029W
RGT1
IXR1
RTT106
RTF1
SPT8
CBF1
RXT2
YTA7
OPI1
MAF1
MKS1
CRF1
PRR1
ACE2
DAL80
GZF3
RAD4
HOS1
CBF1
SPT2
CUP9
LAP3
DOT6
YOX1
HAC1
BYE1
YAP5
KAR4
GAL80
LEU3
YBR239C
ISW2
ITC1
MIG1
RFM1
DAT1
DIG2
WTM1
SDS3
ARG82
PRR2
ESS1
RGR1
MIG3
SUT1
NRG2
RDS2
FKH1
NUT2
FHL1
RAD23
FUN30
IZH2
RPD3
GCN4
MIG2
STE20
RFX1
ASH1
SKM1
GIS1
MET32
SUT2
YHP1
HAC1
WTM2
ABF1
TUP1
GCR1
MOT3
RME1
XBP1
SFL1
REG2
YKR064W
MET31
GCR1
PAF1
WHI5
CSE2
STH1
GAL11
SPT6
NCB2
YAP6
YPL216W
MOD5
FAP1
YJR119C
DIG1
SIN3
PGD1
DEP1
SIN4
RCS1
SRB2
RPA12
NPL3
RPH1
MED1
CLA4
SSN8
YGR122W
SRB7
VPS36
RIM101
SSN3
VPS25
SNF8
SRB8
HDA2
YGL220W
ROX3
UME6
PHO80
NUP133
NUP120
CYC8
SPT10
CDC28
REG1
GCR2
GLC7
PHO85
MED7

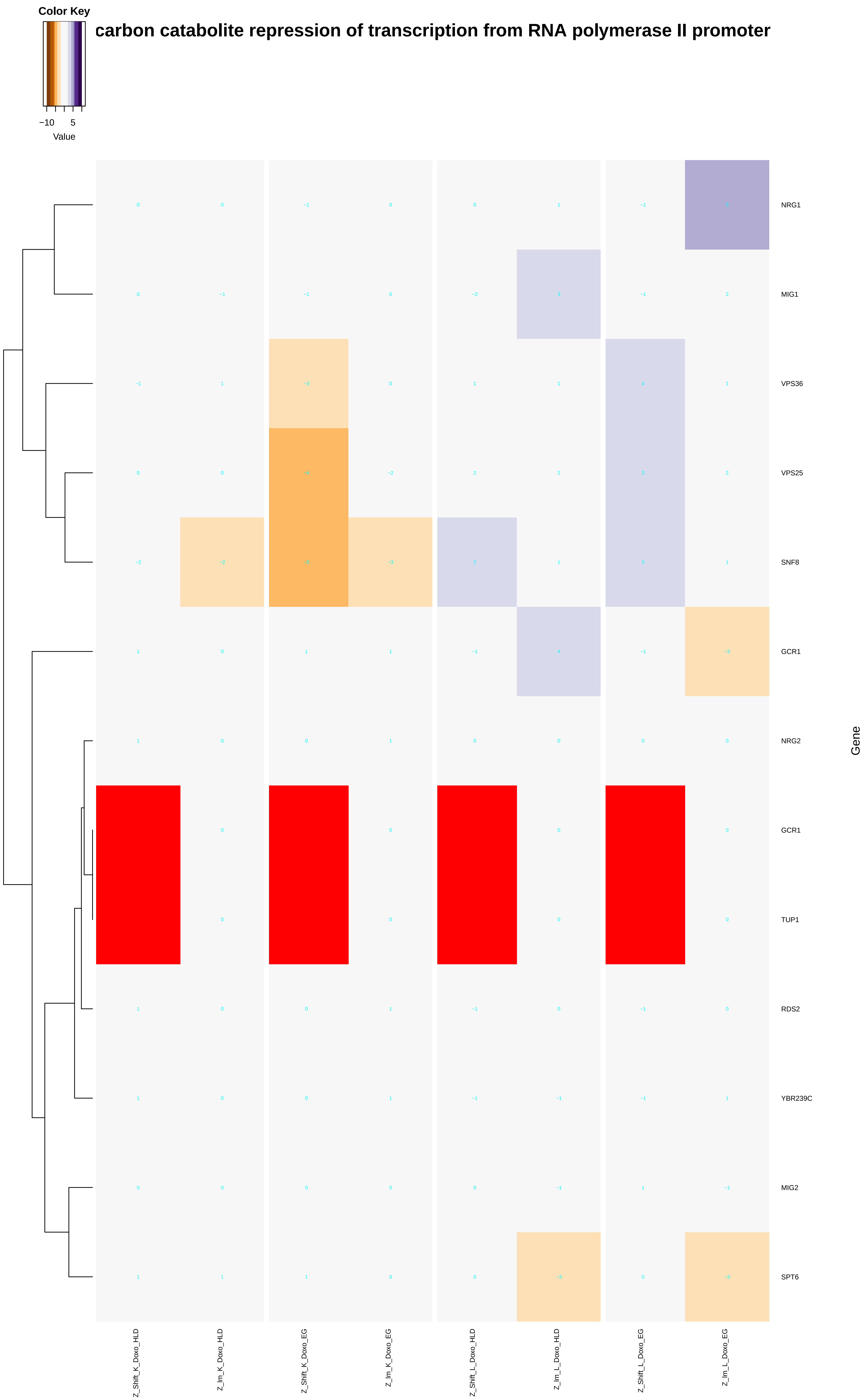

Color Key
−10
5
Value
carbon catabolite repression of transcription from RNA polymerase II promoter
NRG1
MIG1
VPS36
VPS25
SNF8
GCR1
NRG2
GCR1
TUP1
RDS2
YBR239C
MIG2
SPT6
Gene
Z_Shift_K_Doxo_HLD
Z_lm_K_Doxo_HLD
Z_Shift_K_Doxo_EG
Z_lm_K_Doxo_EG
Z_Shift_L_Doxo_HLD
Z_lm_L_Doxo_HLD
Z_Shift_L_Doxo_EG
Z_lm_L_Doxo_EG

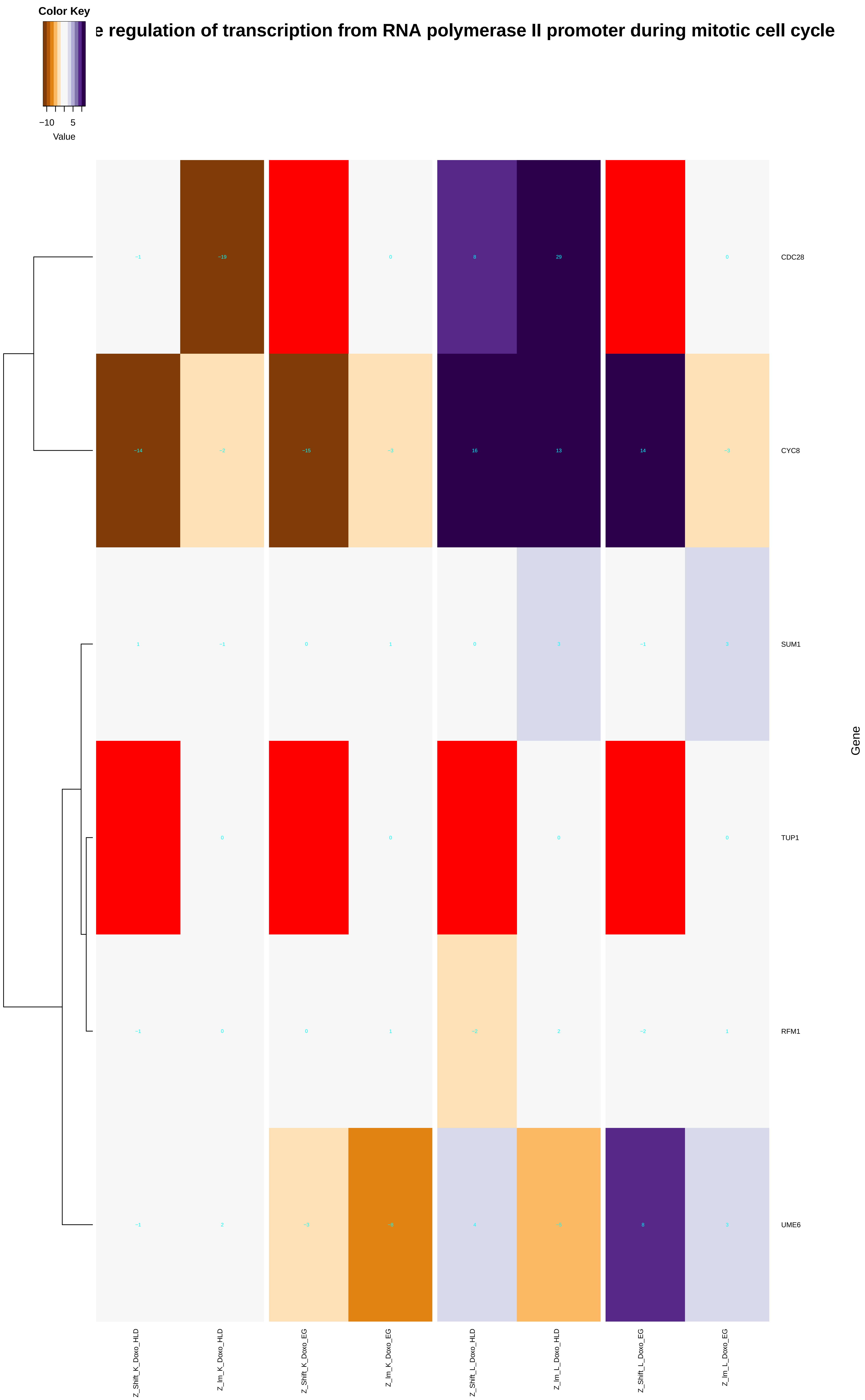
Color Key
−10
5
Value
e regulation of transcription from RNA polymerase II promoter during mitotic cell cycle
CDC28
CYC8
SUM1
TUP1
RFM1
UME6
Gene
Z_Shift_K_Doxo_HLD
Z_Im_K_Doxo_HLD
Z_Shift_K_Doxo_EG
Z_Im_K_Doxo_EG
Z_Shift_L_Doxo_HLD
Z_Im_L_Doxo_HLD
Z_Shift_L_Doxo_EG
Z_Im_L_Doxo_EG

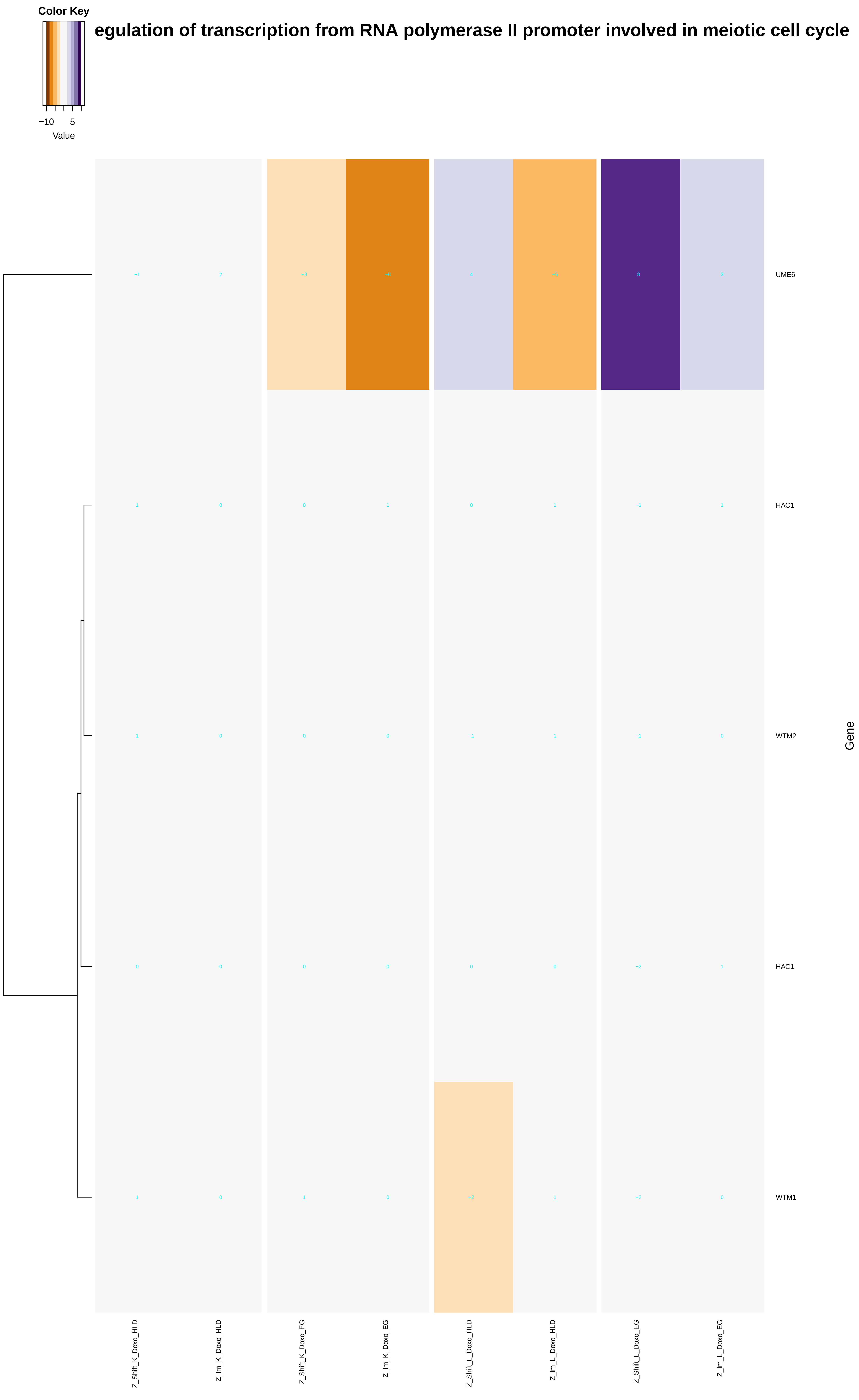
Color Key
−10
5
Value
egulation of transcription from RNA polymerase II promoter involved in meiotic cell cycle
UME6
HAC1
WTM2
HAC1
WTM1
Gene
Z_Shift_K_Doxo_HLD
Z_Im_K_Doxo_HLD
Z_Shift_K_Doxo_EG
Z_Im_K_Doxo_EG
Z_Shift_L_Doxo_HLD
Z_Im_L_Doxo_HLD
Z_Shift_L_Doxo_EG
Z_Im_L_Doxo_EG

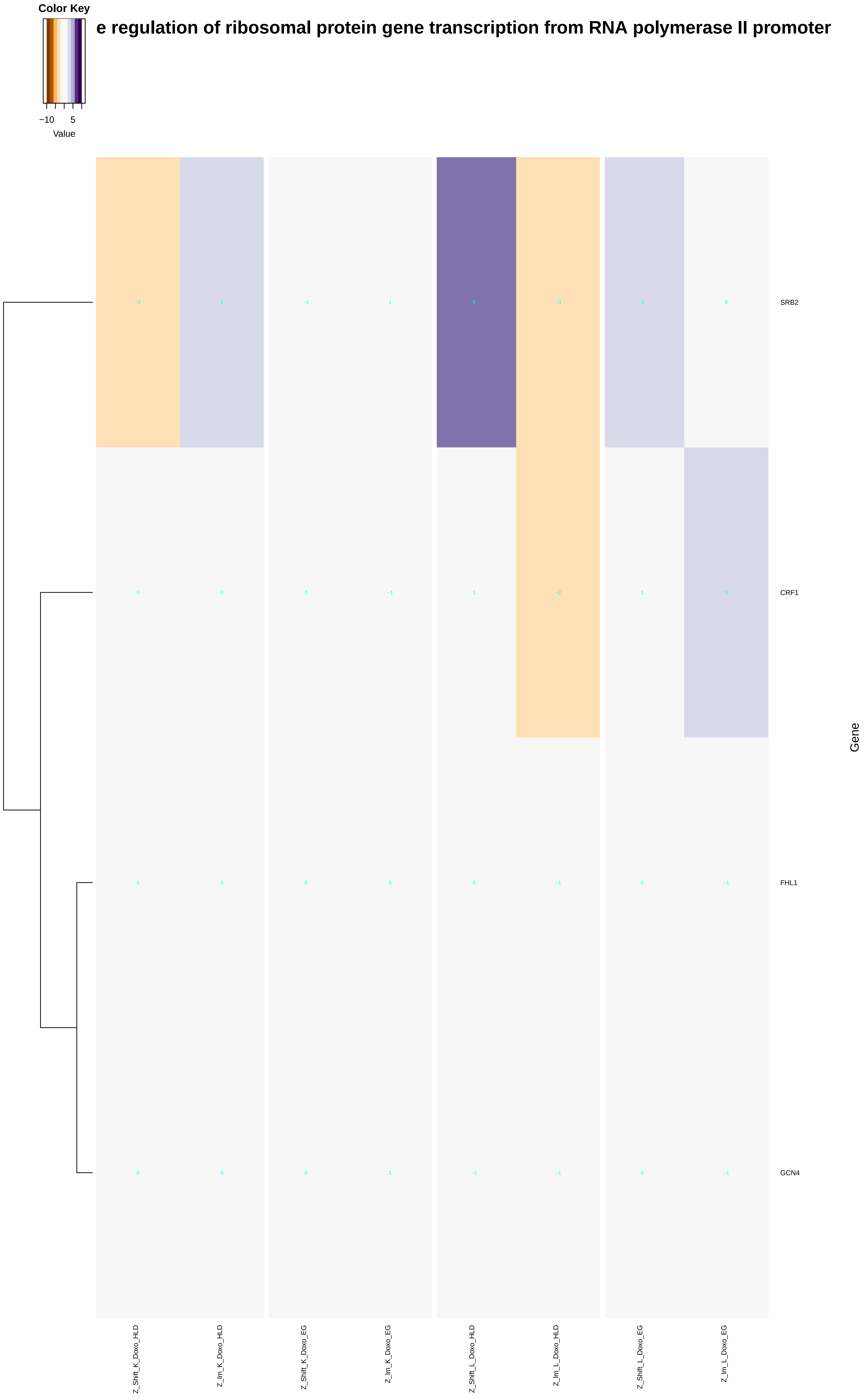
e regulation of ribosomal protein gene transcription from RNA polymerase II promoter
Color Key
−10
5
Value
SRB2
CRF1
FHL1
GCN4
Gene
Z_Shift_K_Doxo_HLD
Z_Im_K_Doxo_HLD
Z_Shift_K_Doxo_EG
Z_Im_K_Doxo_EG
Z_Shift_L_Doxo_HLD
Z_Im_L_Doxo_HLD
Z_Shift_L_Doxo_EG
Z_Im_L_Doxo_EG
−2 2 −1 1 6 −3 3 0
0 0 0 −1 1 −2 1 3
1 1 0 0 0 −1 0 −1
0 0 0 1 −1 −1 0 −1

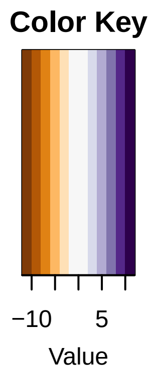

of oligopeptide transport by negative regulation of transcription from RNA polymerase II p

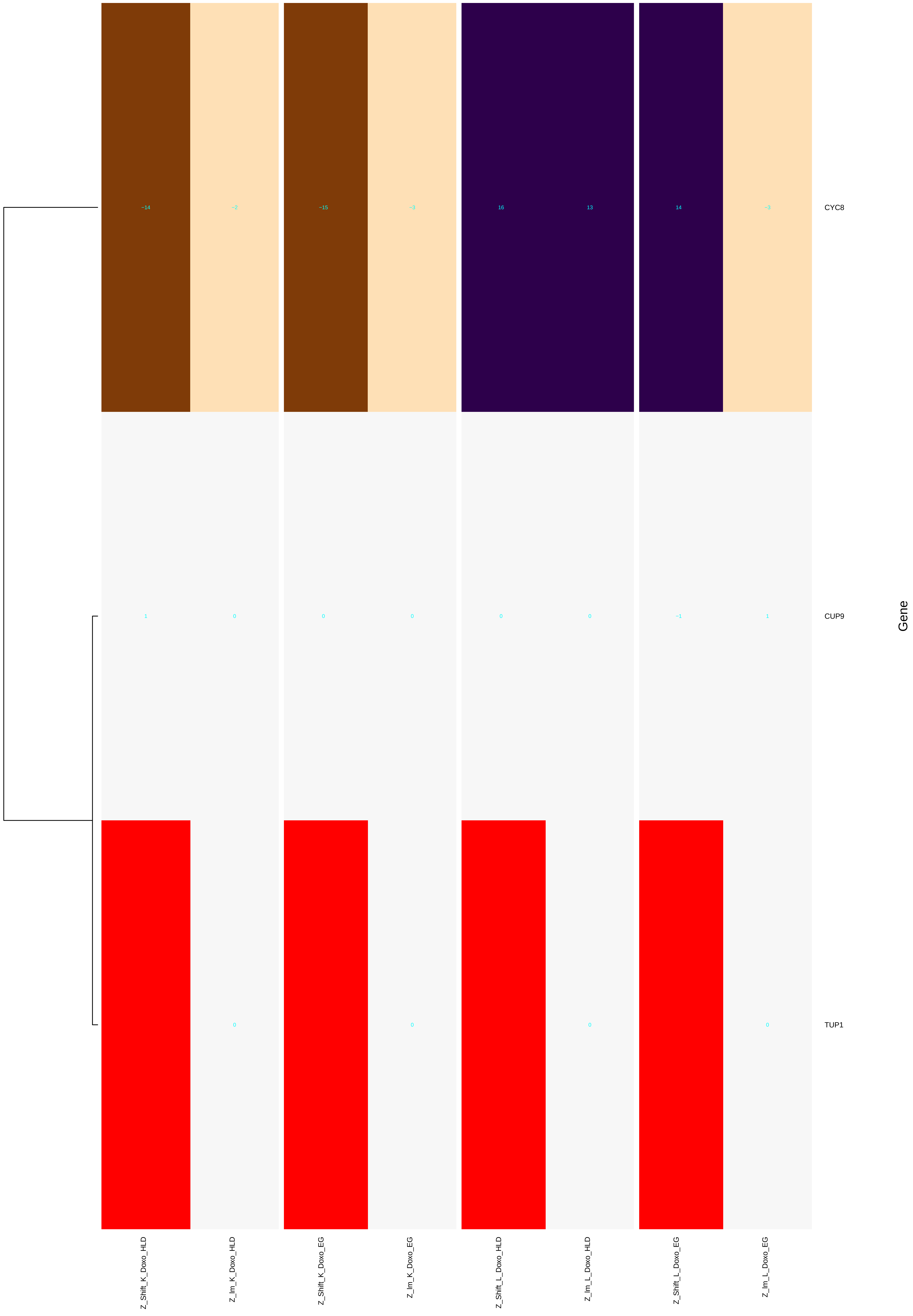

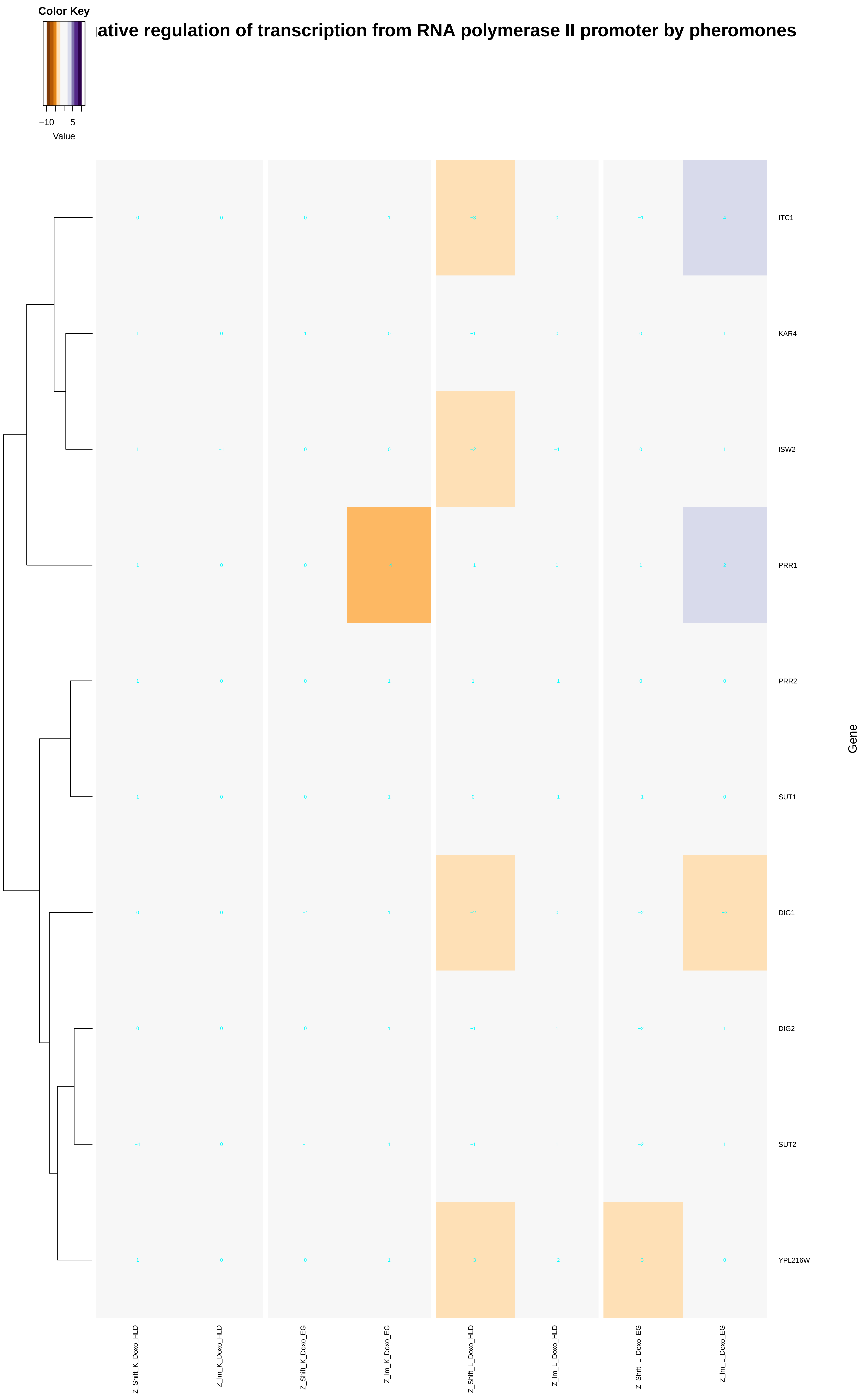
Color Key
−10
5
Value
ative regulation of transcription from RNA polymerase II promoter by pheromones
ITC1
KAR4
ISW2
PRR1
PRR2
SUT1
DIG1
DIG2
SUT2
YPL216W
Gene
Z_Shift_K_Doxo_HLD
Z_Im_K_Doxo_HLD
Z_Shift_K_Doxo_EG
Z_Im_K_Doxo_EG
Z_Shift_L_Doxo_HLD
Z_Im_L_Doxo_HLD
Z_Shift_L_Doxo_EG
Z_Im_L_Doxo_EG

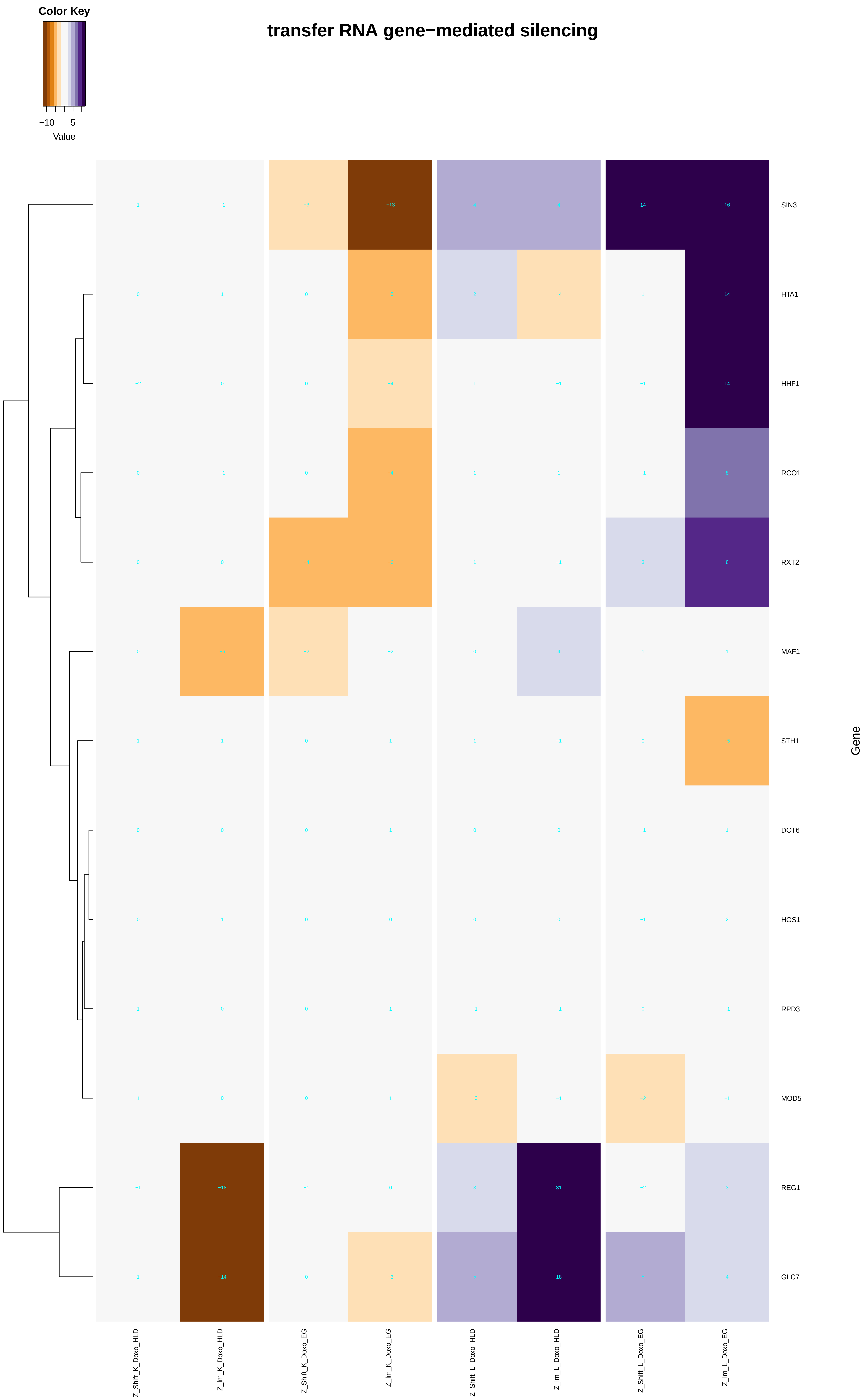
transfer RNA gene−mediated silencing
Color Key
−10 5
Value
SIN3
HTA1
HHF1
RCO1
RXT2
MAF1
STH1
DOT6
HOS1
RPD3
MOD5
REG1
GLC7
Gene
Z_Shift_K_Doxo_HLD
Z_lm_K_Doxo_HLD
Z_Shift_K_Doxo_EG
Z_lm_K_Doxo_EG
Z_Shift_L_Doxo_HLD
Z_lm_L_Doxo_HLD
Z_Shift_L_Doxo_EG
Z_lm_L_Doxo_EG

of lipid transport by negative regulation of transcription from RNA polymerase II promoter

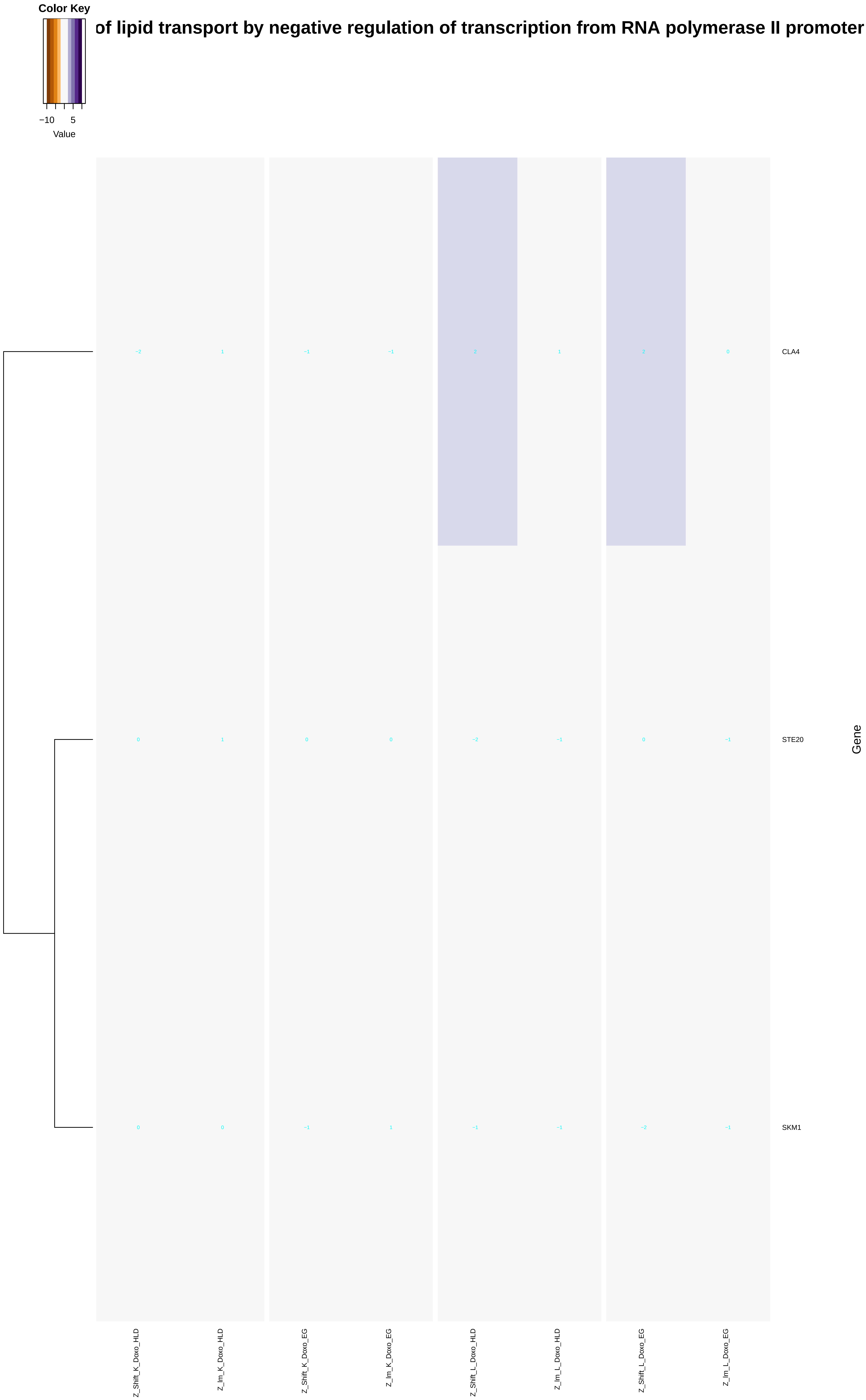

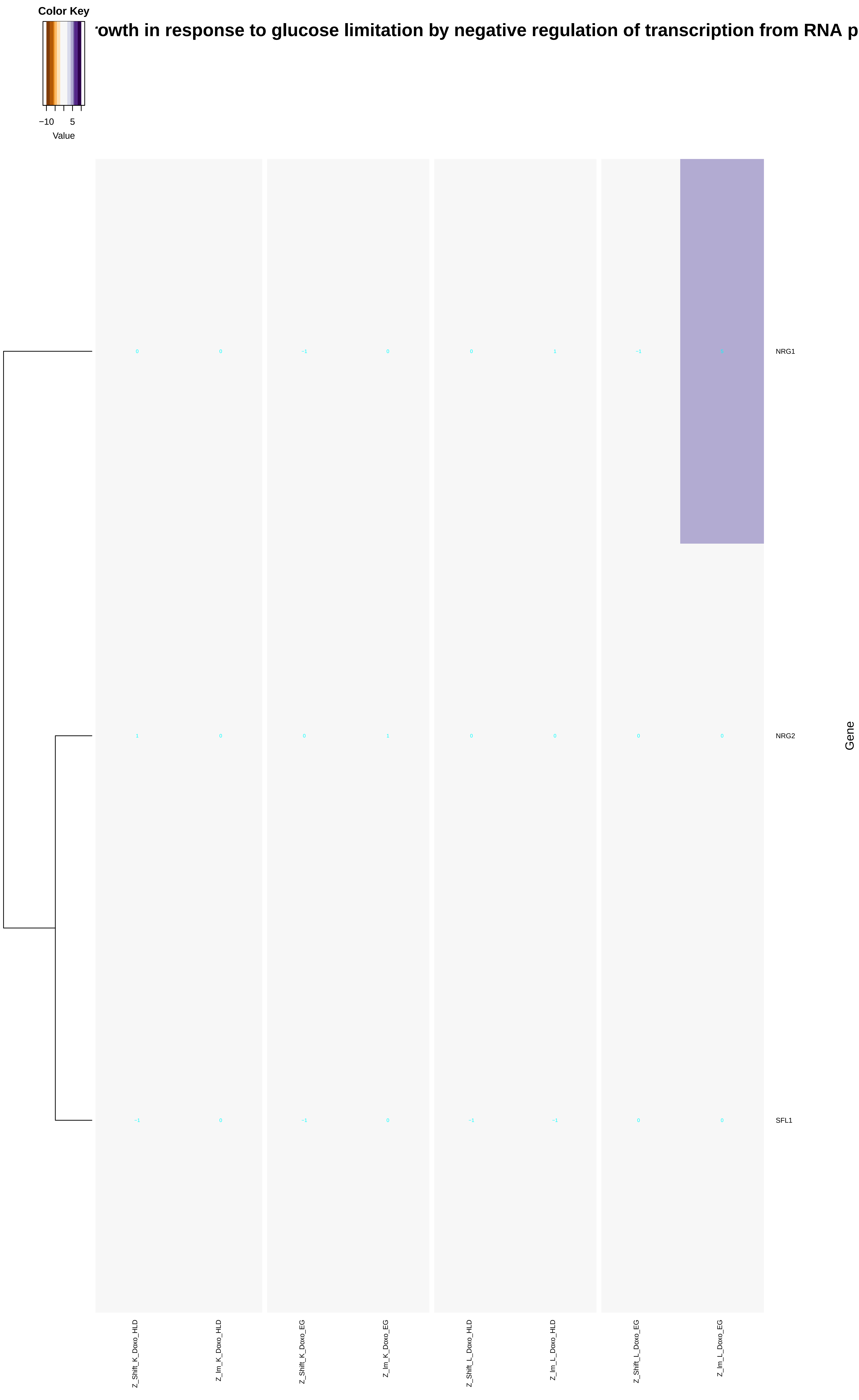

Color Key
−10
5
Value
rowth in response to glucose limitation by negative regulation of transcription from RNA p
NRG1
NRG2
SFL1
Gene
Z_Shift_K_Doxo_HLD
Z_Im_K_Doxo_HLD
Z_Shift_K_Doxo_EG
Z_Im_K_Doxo_EG
Z_Shift_L_Doxo_HLD
Z_Im_L_Doxo_HLD
Z_Shift_L_Doxo_EG
Z_Im_L_Doxo_EG

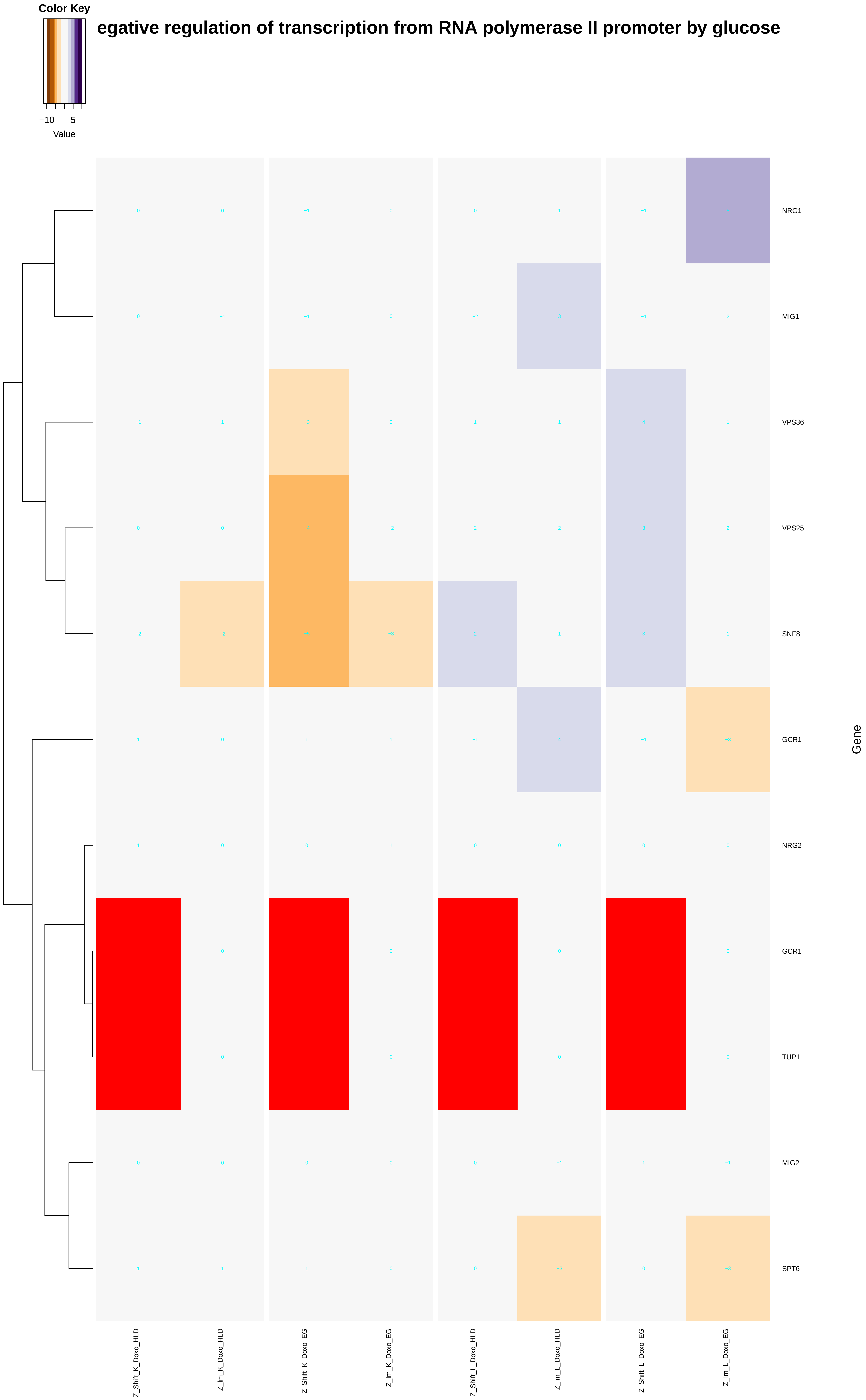

Color Key
egative regulation of transcription from RNA polymerase II promoter by glucose
−10
5
Value
NRG1
MIG1
VPS36
VPS25
SNF8
GCR1
NRG2
GCR1
TUP1
MIG2
SPT6
Gene
Z_Shift_K_Doxo_HLD
Z_Im_K_Doxo_HLD
Z_Shift_K_Doxo_EG
Z_Im_K_Doxo_EG
Z_Shift_L_Doxo_HLD
Z_Im_L_Doxo_HLD
Z_Shift_L_Doxo_EG
Z_Im_L_Doxo_EG

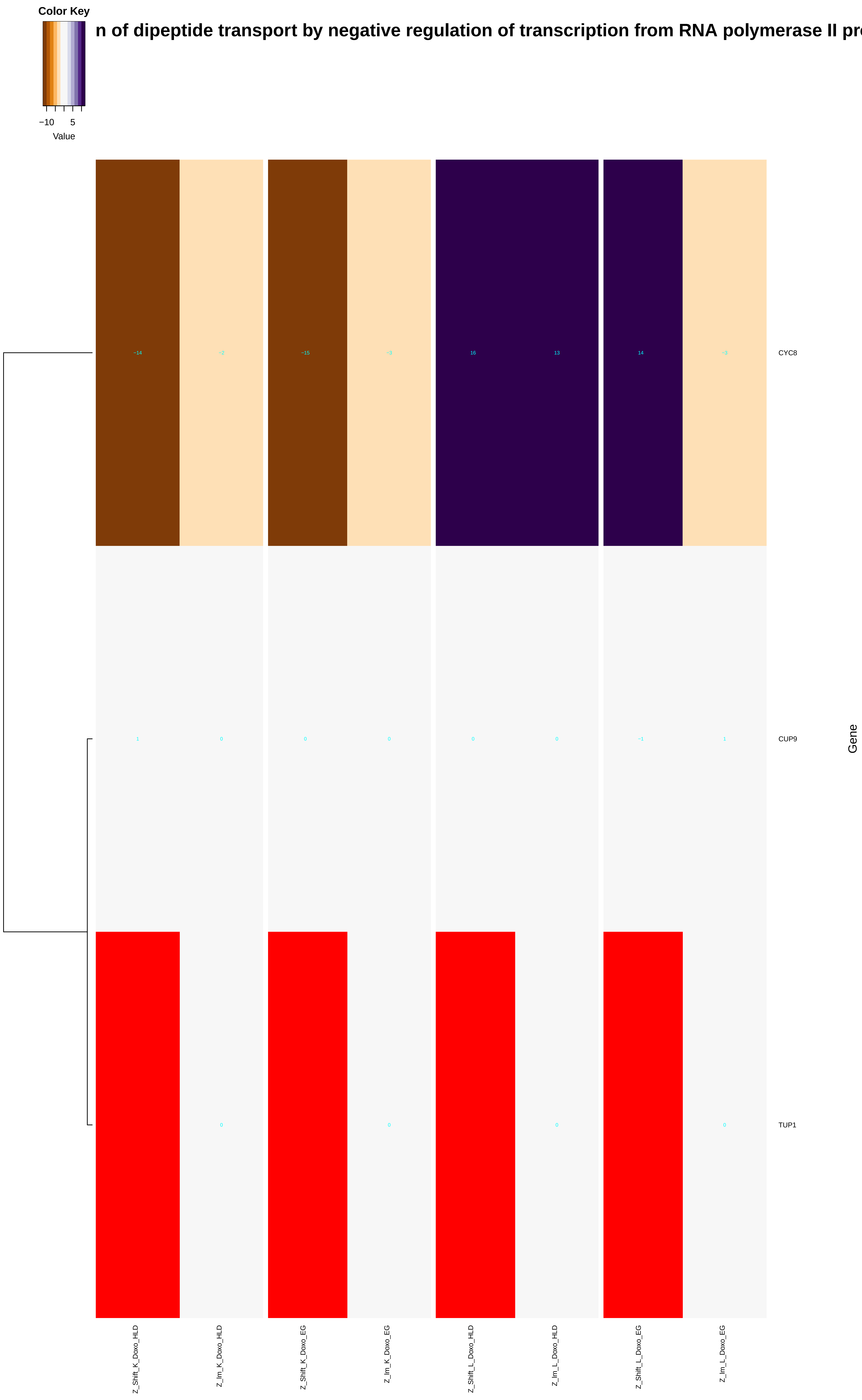
Color Key
−10 5
Value
n of dipeptide transport by negative regulation of transcription from RNA polymerase II pr
−14
−2
−15
−3
16
13
14
−3
CYC8
1
0
0
0
0
0
−1
1
CUP9
0
0
0
0
TUP1
Gene
Z_Shift_K_Doxo_HLD
Z_lm_K_Doxo_HLD
Z_Shift_K_Doxo_EG
Z_lm_K_Doxo_EG
Z_Shift_L_Doxo_HLD
Z_lm_L_Doxo_HLD
Z_Shift_L_Doxo_EG
Z_lm_L_Doxo_EG

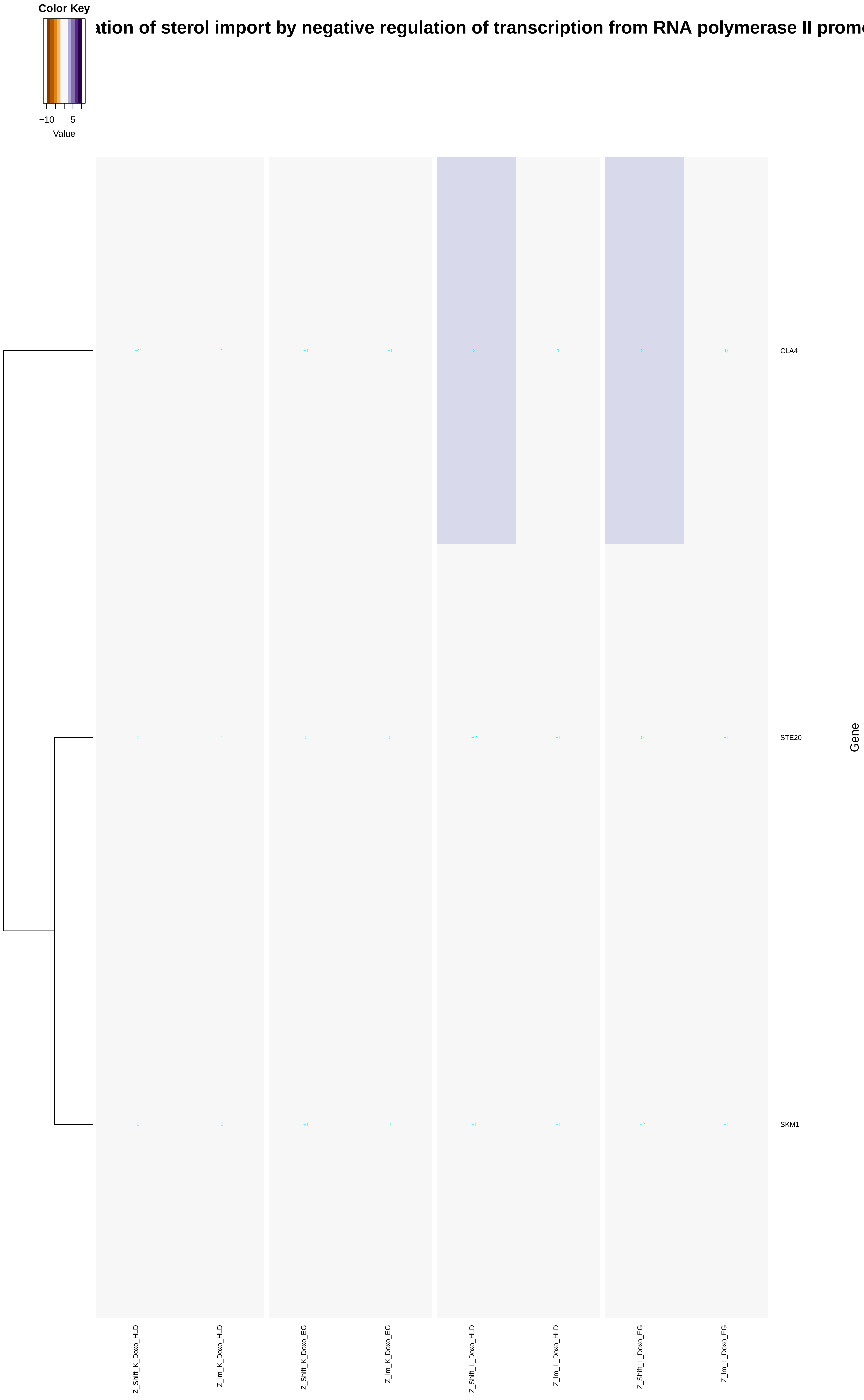

Color Key
−10
5
Value
ation of sterol import by negative regulation of transcription from RNA polymerase II prome
CLA4
STE20
SKM1
Gene
−2
1
−1
−1
2
1
2
0
0
1
0
0
−2
−1
0
−1
0
0
−1
1
−1
−1
−2
−1
Z_Shift_K_Doxo_HLD
Z_Im_K_Doxo_HLD
Z_Shift_K_Doxo_EG
Z_Im_K_Doxo_EG
Z_Shift_L_Doxo_HLD
Z_Im_L_Doxo_HLD
Z_Shift_L_Doxo_EG
Z_Im_L_Doxo_EG
