# Supplementary material for: A yeast phenomic model for the influence of Warburg metabolism on genetic buffering of doxorubicin
Source: Cancer Metab. 2019 Oct 23;7:9. doi: 10.1186/s40170-019-0201-3 (PMC6806529; doi:10.1186/s40170-019-0201-3)
Supplement: Supplementary file 9 — Additional file 9. GO term-specific heatmaps for REMc/GTF-enriched clusters. GO term-specific heatmaps for significant GO process terms were generated as described in methods and Figs. 3 and 4. Any related child terms are presented in subsequent pages of the parent file name. GO terms with more than 100 children, with 2 or fewer genes annotated to the term, or a file size over 300KB are not shown. All heatmaps are generated with the same layout (see Figs. 3 and 4). [file 40170_2019_201_MOESM9_ESM.bz2 › Additional_File9_GOTermHeatmaps/Additional_File9_GOTermHeatmap/non-recombinational_repair.pdf]

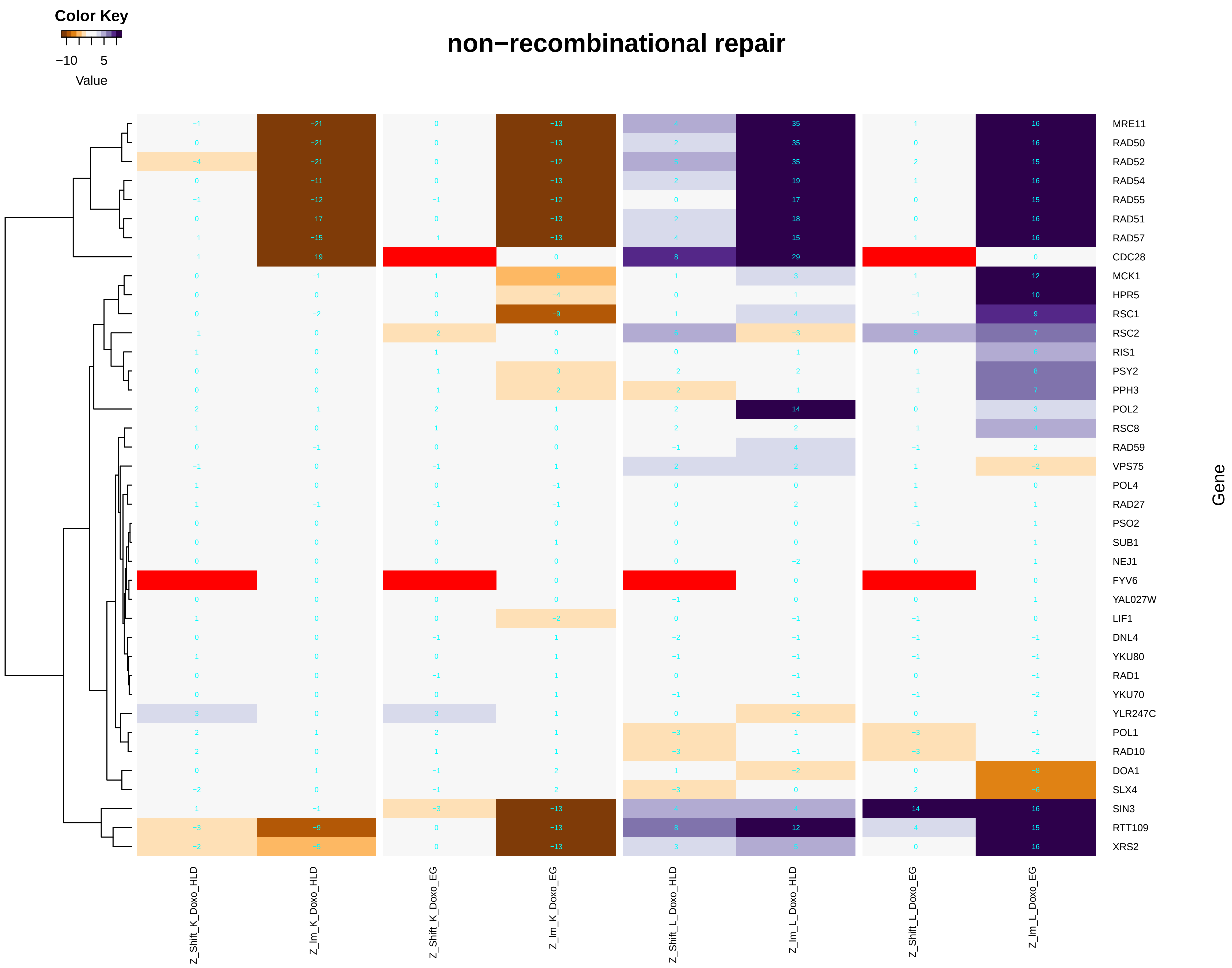

Color Key
−10
5
Value
non−recombinational repair
MRE11
RAD50
RAD52
RAD54
RAD55
RAD51
RAD57
CDC28
MCK1
HPR5
RSC1
RSC2
RIS1
PSY2
PPH3
POL2
RSC8
RAD59
VPS75
POL4
RAD27
PSO2
SUB1
NEJ1
FYV6
YAL027W
LIF1
DNL4
YKU80
RAD1
YKU70
YLR247C
POL1
RAD10
DOA1
SLX4
SIN3
RTT109
XRS2
Gene
Z_Shift_K_Doxo_HLD
Z_Im_K_Doxo_HLD
Z_Shift_K_Doxo_EG
Z_Im_K_Doxo_EG
Z_Shift_L_Doxo_HLD
Z_Im_L_Doxo_HLD
Z_Shift_L_Doxo_EG
Z_Im_L_Doxo_EG

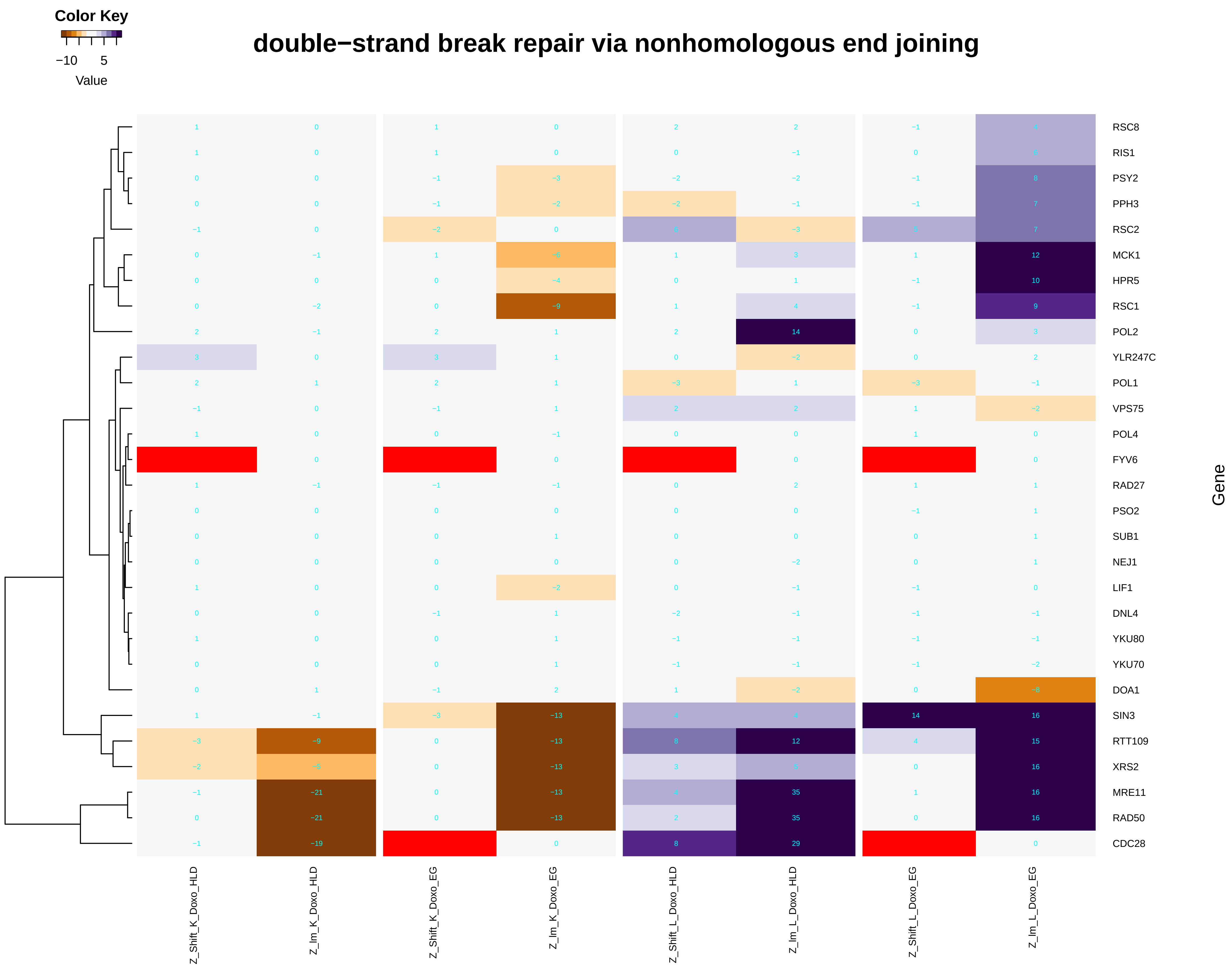
Color Key
−10
5
Value
double−strand break repair via nonhomologous end joining
RSC8
RIS1
PSY2
PPH3
RSC2
MCK1
HPR5
RSC1
POL2
YLR247C
POL1
VPS75
POL4
FYV6
RAD27
PSO2
SUB1
NEJ1
LIF1
DNL4
YKU80
YKU70
DOA1
SIN3
RTT109
XRS2
MRE11
RAD50
CDC28
Gene
Z_Shift_K_Doxo_HLD
Z_lm_K_Doxo_HLD
Z_Shift_K_Doxo_EG
Z_lm_K_Doxo_EG
Z_Shift_L_Doxo_HLD
Z_lm_L_Doxo_HLD
Z_Shift_L_Doxo_EG
Z_lm_L_Doxo_EG

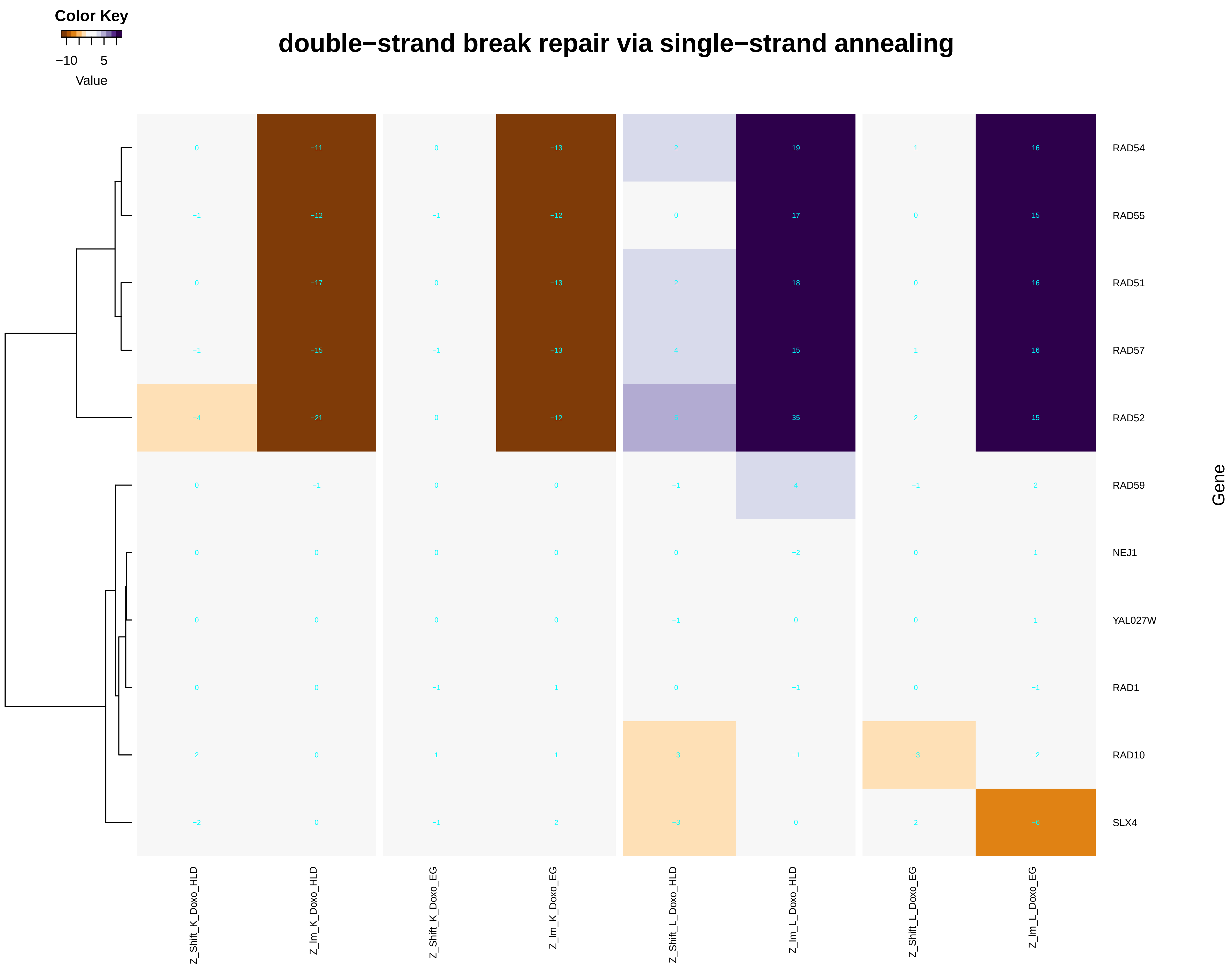

double−strand break repair via single−strand annealing
Color Key
−10
5
Value
RAD54
RAD55
RAD51
RAD57
RAD52
RAD59
NEJ1
YAL027W
RAD1
RAD10
SLX4
Gene
Z_Shift_K_Doxo_HLD
Z_Im_K_Doxo_HLD
Z_Shift_K_Doxo_EG
Z_Im_K_Doxo_EG
Z_Shift_L_Doxo_HLD
Z_Im_L_Doxo_HLD
Z_Shift_L_Doxo_EG
Z_Im_L_Doxo_EG
