# Supplementary material for: A yeast phenomic model for the influence of Warburg metabolism on genetic buffering of doxorubicin
Source: Cancer Metab. 2019 Oct 23;7:9. doi: 10.1186/s40170-019-0201-3 (PMC6806529; doi:10.1186/s40170-019-0201-3)
Supplement: Supplementary file 9 — Additional file 9. GO term-specific heatmaps for REMc/GTF-enriched clusters. GO term-specific heatmaps for significant GO process terms were generated as described in methods and Figs. 3 and 4. Any related child terms are presented in subsequent pages of the parent file name. GO terms with more than 100 children, with 2 or fewer genes annotated to the term, or a file size over 300KB are not shown. All heatmaps are generated with the same layout (see Figs. 3 and 4). [file 40170_2019_201_MOESM9_ESM.bz2 › Additional_File9_GOTermHeatmaps/Additional_File9_GOTermHeatmap/nuclear-transcribed_mRNA_catabolic_process,_nonsense-mediated_decay.pdf]

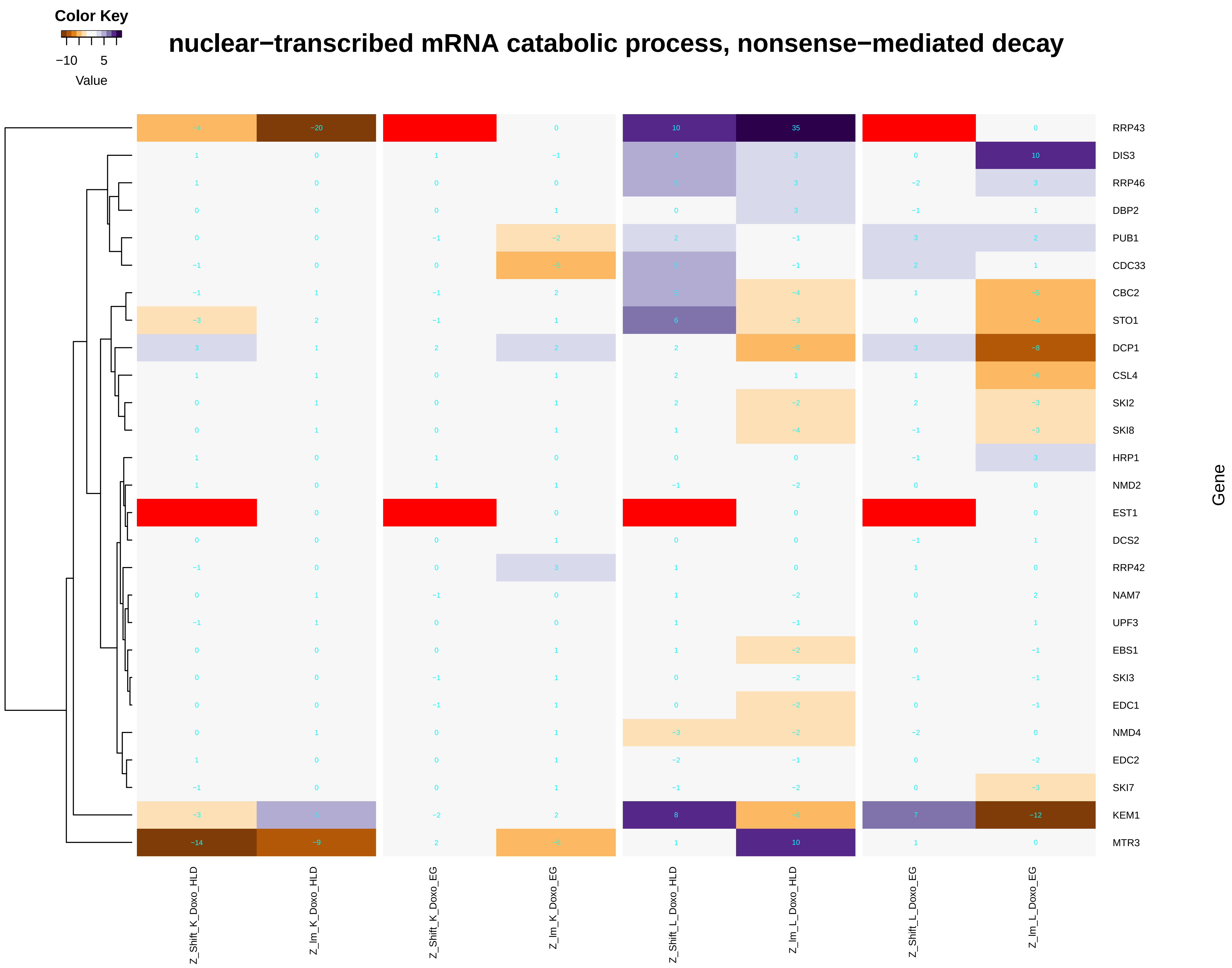
Color Key
−10 5
Value
nuclear−transcribed mRNA catabolic process, nonsense−mediated decay
RRP43
DIS3
RRP46
DBP2
PUB1
CDC33
CBC2
STO1
DCP1
CSL4
SKI2
SKI8
HRP1
NMD2
EST1
DCS2
RRP42
NAM7
UPF3
EBS1
SKI3
EDC1
NMD4
EDC2
SKI7
KEM1
MTR3
Gene
Z_Shift_K_Doxo_HLD
Z_Im_K_Doxo_HLD
Z_Shift_K_Doxo_EG
Z_Im_K_Doxo_EG
Z_Shift_L_Doxo_HLD
Z_Im_L_Doxo_HLD
Z_Shift_L_Doxo_EG
Z_Im_L_Doxo_EG

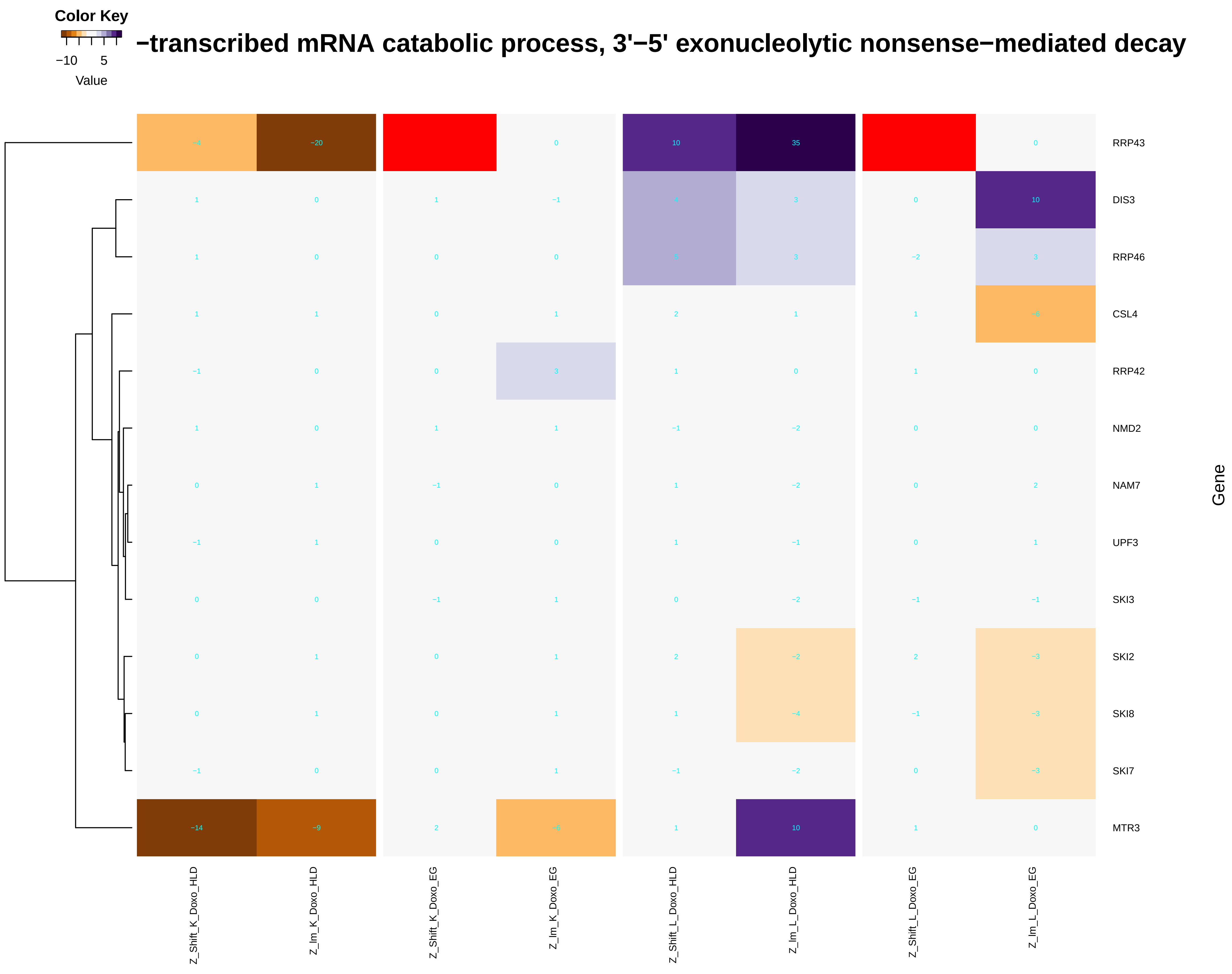

Color Key
−10 5
Value
−transcribed mRNA catabolic process, 3'−5' exonucleolytic nonsense−mediated decay
RRP43
DIS3
RRP46
CSL4
RRP42
NMD2
NAM7
UPF3
SKI3
SKI2
SKI8
SKI7
MTR3
Gene
Z_Shift_K_Doxo_HLD
Z_Im_K_Doxo_HLD
Z_Shift_K_Doxo_EG
Z_Im_K_Doxo_EG
Z_Shift_L_Doxo_HLD
Z_Im_L_Doxo_HLD
Z_Shift_L_Doxo_EG
Z_Im_L_Doxo_EG
