# Supplementary material for: A yeast phenomic model for the influence of Warburg metabolism on genetic buffering of doxorubicin
Source: Cancer Metab. 2019 Oct 23;7:9. doi: 10.1186/s40170-019-0201-3 (PMC6806529; doi:10.1186/s40170-019-0201-3)
Supplement: Supplementary file 9 — Additional file 9. GO term-specific heatmaps for REMc/GTF-enriched clusters. GO term-specific heatmaps for significant GO process terms were generated as described in methods and Figs. 3 and 4. Any related child terms are presented in subsequent pages of the parent file name. GO terms with more than 100 children, with 2 or fewer genes annotated to the term, or a file size over 300KB are not shown. All heatmaps are generated with the same layout (see Figs. 3 and 4). [file 40170_2019_201_MOESM9_ESM.bz2 › Additional_File9_GOTermHeatmaps/Additional_File9_GOTermHeatmap/nucleoside_triphosphate_biosynthetic_process.pdf]

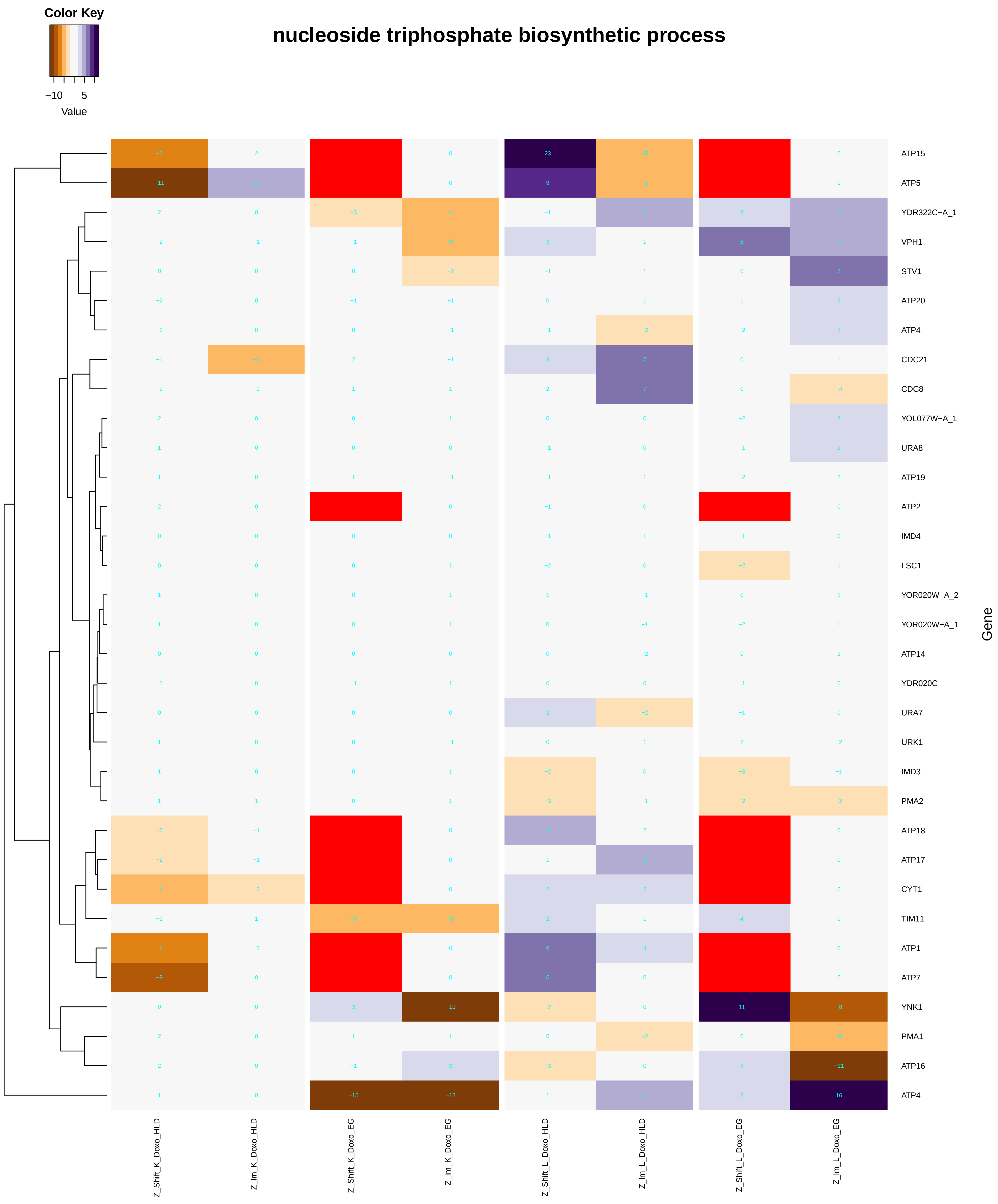
Color Key
−10
5
Value
nucleoside triphosphate biosynthetic process
ATP15
ATP5
YDR322C−A_1
VPH1
STV1
ATP20
ATP4
CDC21
CDC8
YOL077W−A_1
URA8
ATP19
ATP2
IMD4
LSC1
YOR020W−A_2
YOR020W−A_1
ATP14
YDR020C
URA7
URK1
IMD3
PMA2
ATP18
ATP17
CYT1
TIM11
ATP1
ATP7
YNK1
PMA1
ATP16
ATP4
Gene
Z_Shift_K_Doxo_HLD
Z_Im_K_Doxo_HLD
Z_Shift_K_Doxo_EG
Z_Im_K_Doxo_EG
Z_Shift_L_Doxo_HLD
Z_Im_L_Doxo_HLD
Z_Shift_L_Doxo_EG
Z_Im_L_Doxo_EG

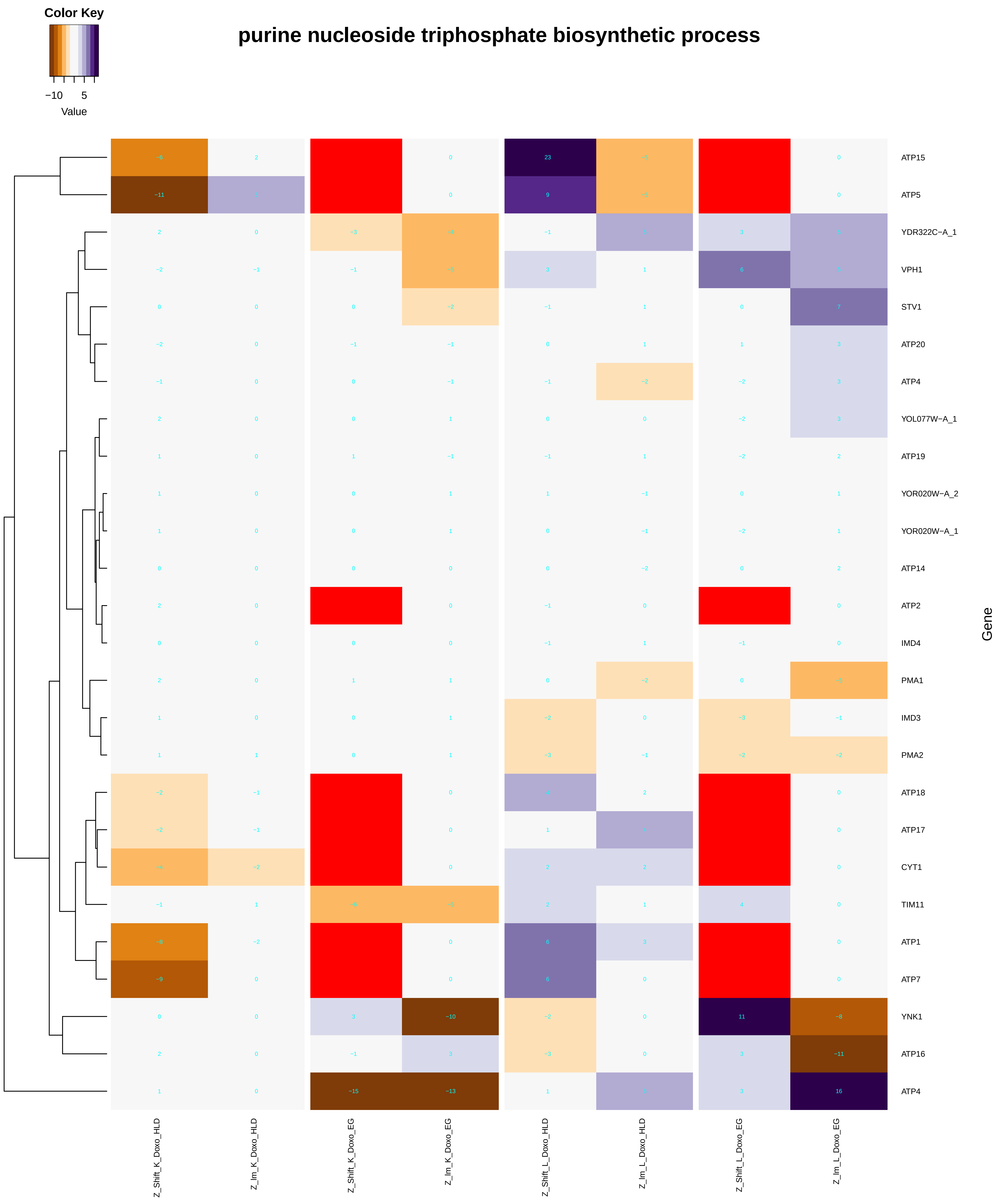
Color Key
−10
5
Value
purine nucleoside triphosphate biosynthetic process
ATP15
ATP5
YDR322C−A_1
VPH1
STV1
ATP20
ATP4
YOL077W−A_1
ATP19
YOR020W−A_2
YOR020W−A_1
ATP14
ATP2
IMD4
PMA1
IMD3
PMA2
ATP18
ATP17
CYT1
TIM11
ATP1
ATP7
YNK1
ATP16
ATP4
Gene
Z_Shift_K_Doxo_HLD
Z_lm_K_Doxo_HLD
Z_Shift_K_Doxo_EG
Z_lm_K_Doxo_EG
Z_Shift_L_Doxo_HLD
Z_lm_L_Doxo_HLD
Z_Shift_L_Doxo_EG
Z_lm_L_Doxo_EG

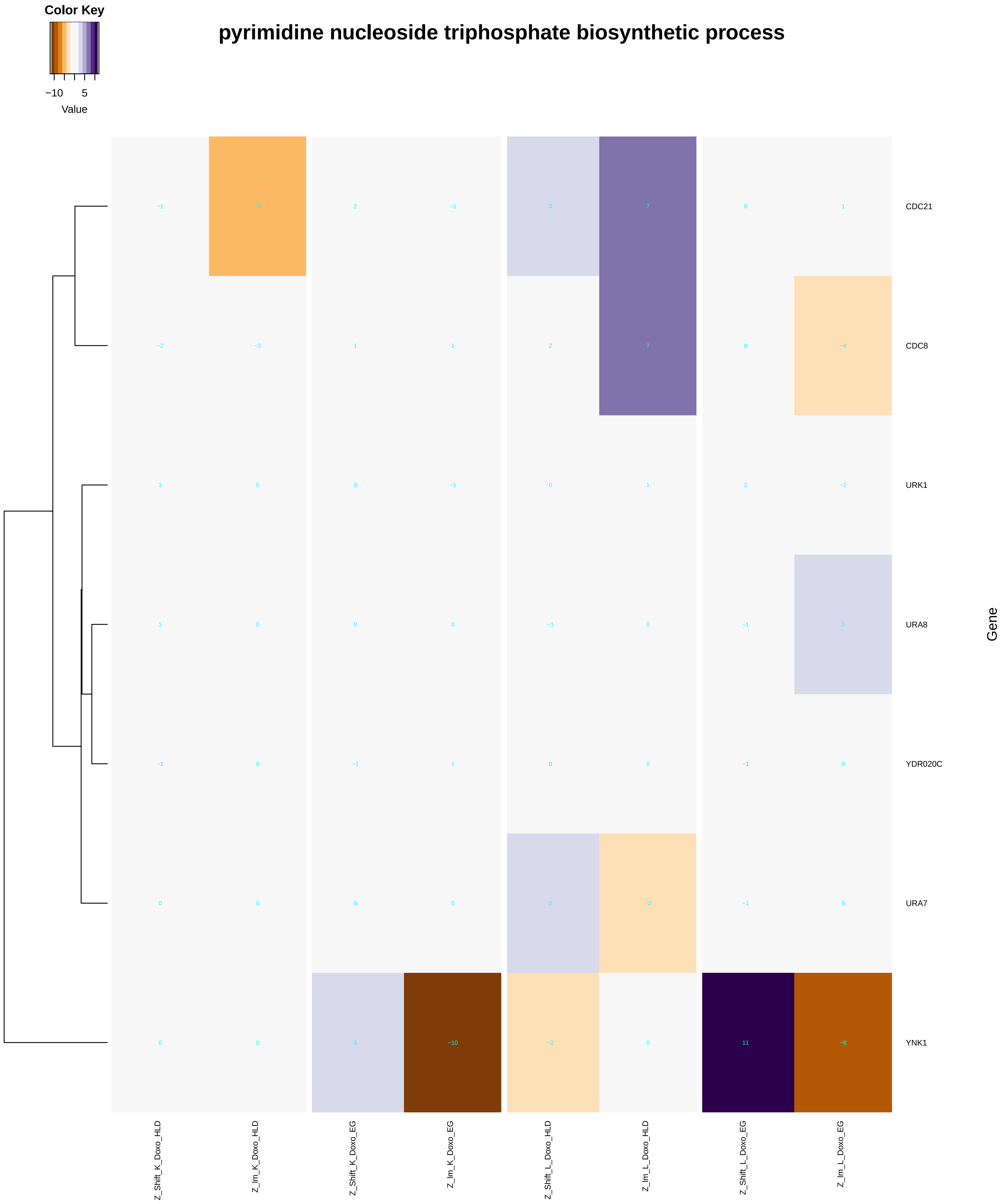

pyrimidine nucleoside triphosphate biosynthetic process
Color Key
−10
5
Value
CDC21
CDC8
URK1
URA8
YDR020C
URA7
YNK1
Gene
Z_Shift_K_Doxo_HLD
Z_lm_K_Doxo_HLD
Z_Shift_K_Doxo_EG
Z_lm_K_Doxo_EG
Z_Shift_L_Doxo_HLD
Z_lm_L_Doxo_HLD
Z_Shift_L_Doxo_EG
Z_lm_L_Doxo_EG

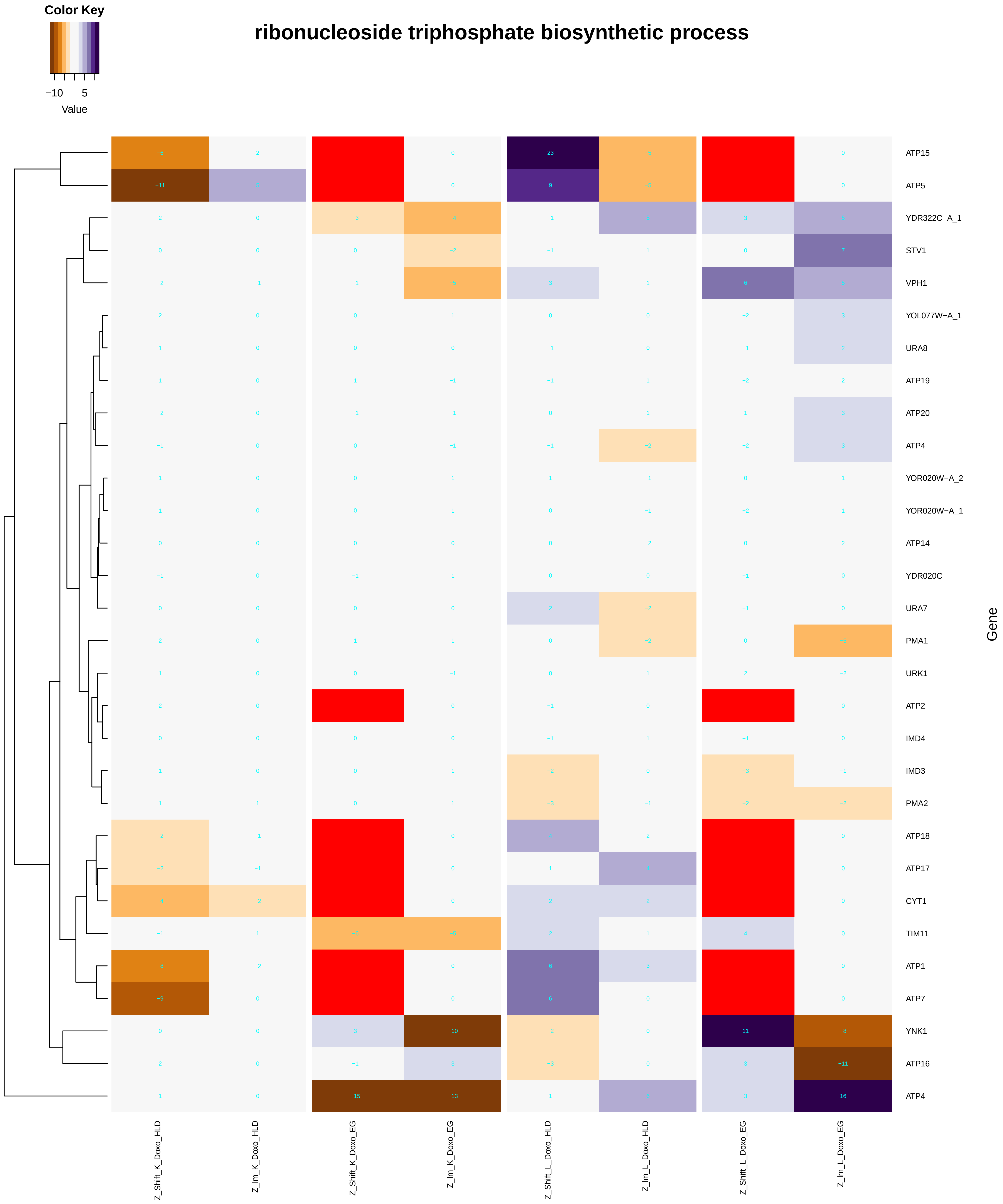

Color Key
−10
5
Value
ribonucleoside triphosphate biosynthetic process
ATP15
ATP5
YDR322C−A_1
STV1
VPH1
YOL077W−A_1
URA8
ATP19
ATP20
ATP4
YOR020W−A_2
YOR020W−A_1
ATP14
YDR020C
URA7
PMA1
URK1
ATP2
IMD4
IMD3
PMA2
ATP18
ATP17
CYT1
TIM11
ATP1
ATP7
YNK1
ATP16
ATP4
Gene
Z_Shift_K_Doxo_HLD
Z_Im_K_Doxo_HLD
Z_Shift_K_Doxo_EG
Z_Im_K_Doxo_EG
Z_Shift_L_Doxo_HLD
Z_Im_L_Doxo_HLD
Z_Shift_L_Doxo_EG
Z_Im_L_Doxo_EG

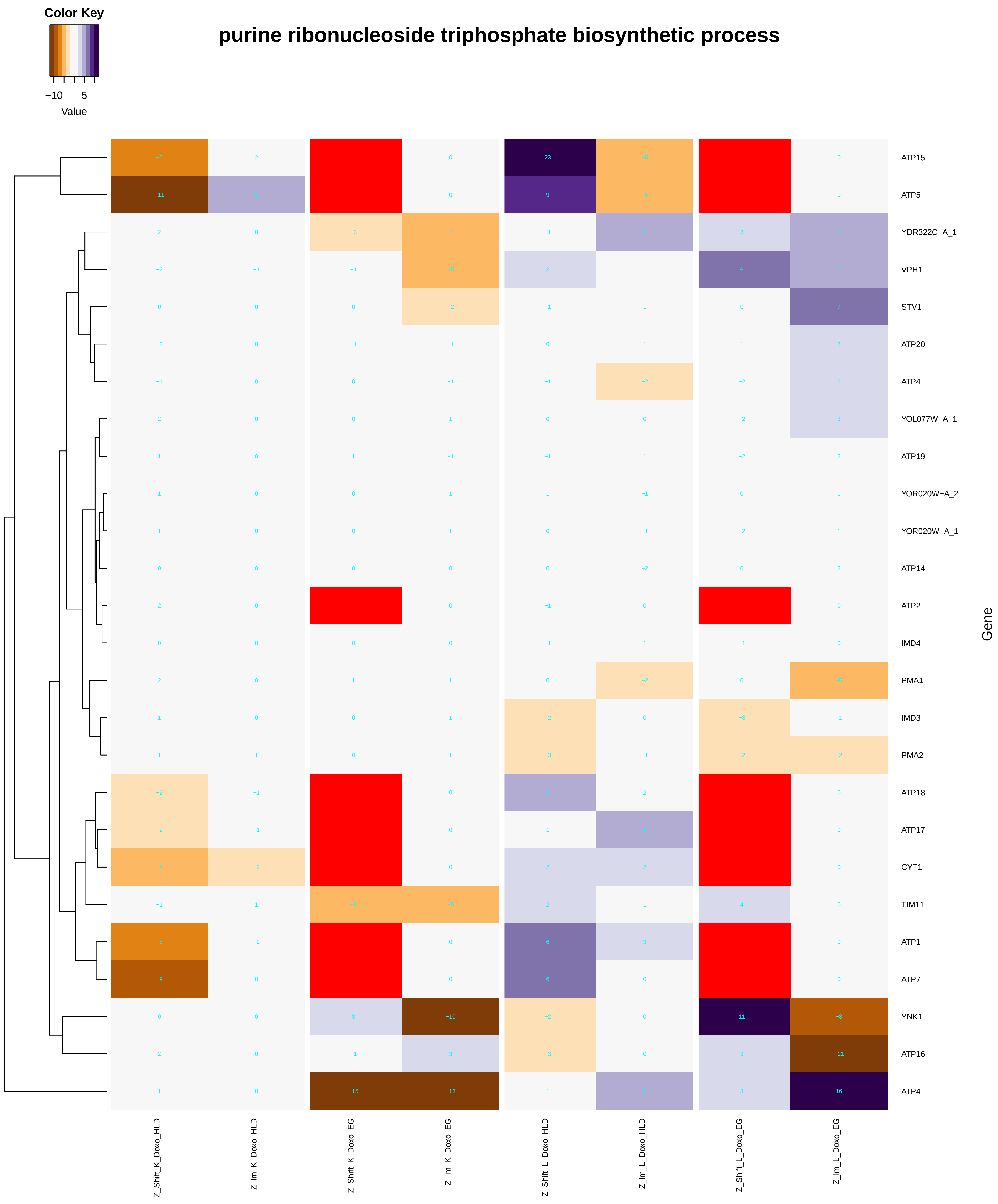

Color Key
−10 5
Value
purine ribonucleoside triphosphate biosynthetic process
ATP15
ATP5
YDR322C−A_1
VPH1
STV1
ATP20
ATP4
YOL077W−A_1
ATP19
YOR020W−A_2
YOR020W−A_1
ATP14
ATP2
IMD4
PMA1
IMD3
PMA2
ATP18
ATP17
CYT1
TIM11
ATP1
ATP7
YNK1
ATP16
ATP4
Gene
Z_Shift_K_Doxo_HLD
Z_Im_K_Doxo_HLD
Z_Shift_K_Doxo_EG
Z_Im_K_Doxo_EG
Z_Shift_L_Doxo_HLD
Z_Im_L_Doxo_HLD
Z_Shift_L_Doxo_EG
Z_Im_L_Doxo_EG

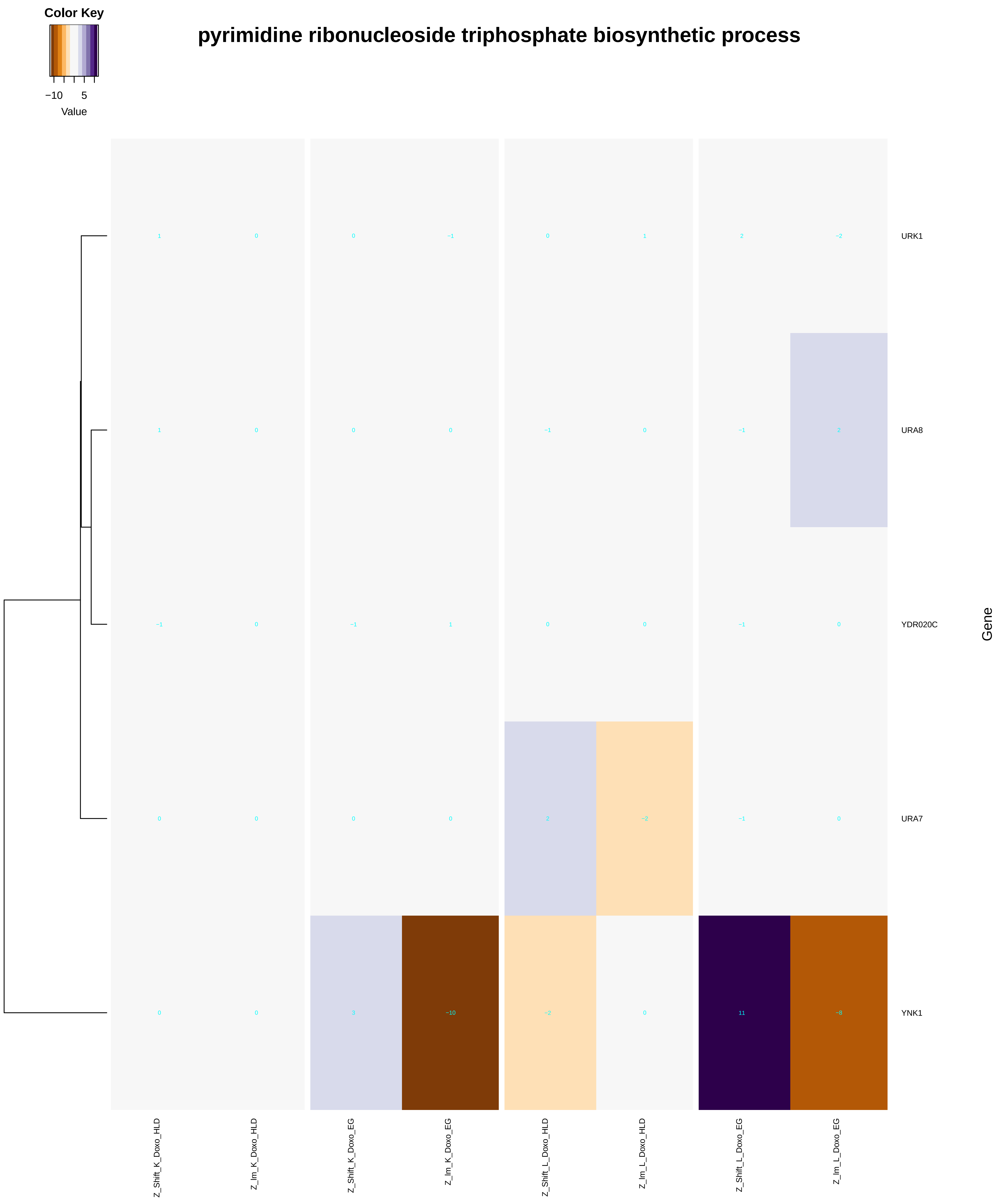
Color Key
−10
5
Value
pyrimidine ribonucleoside triphosphate biosynthetic process
URK1
URA8
YDR020C
URA7
YNK1
Gene
Z_Shift_K_Doxo_HLD
Z_lm_K_Doxo_HLD
Z_Shift_K_Doxo_EG
Z_lm_K_Doxo_EG
Z_Shift_L_Doxo_HLD
Z_lm_L_Doxo_HLD
Z_Shift_L_Doxo_EG
Z_lm_L_Doxo_EG

GTP biosynthetic process

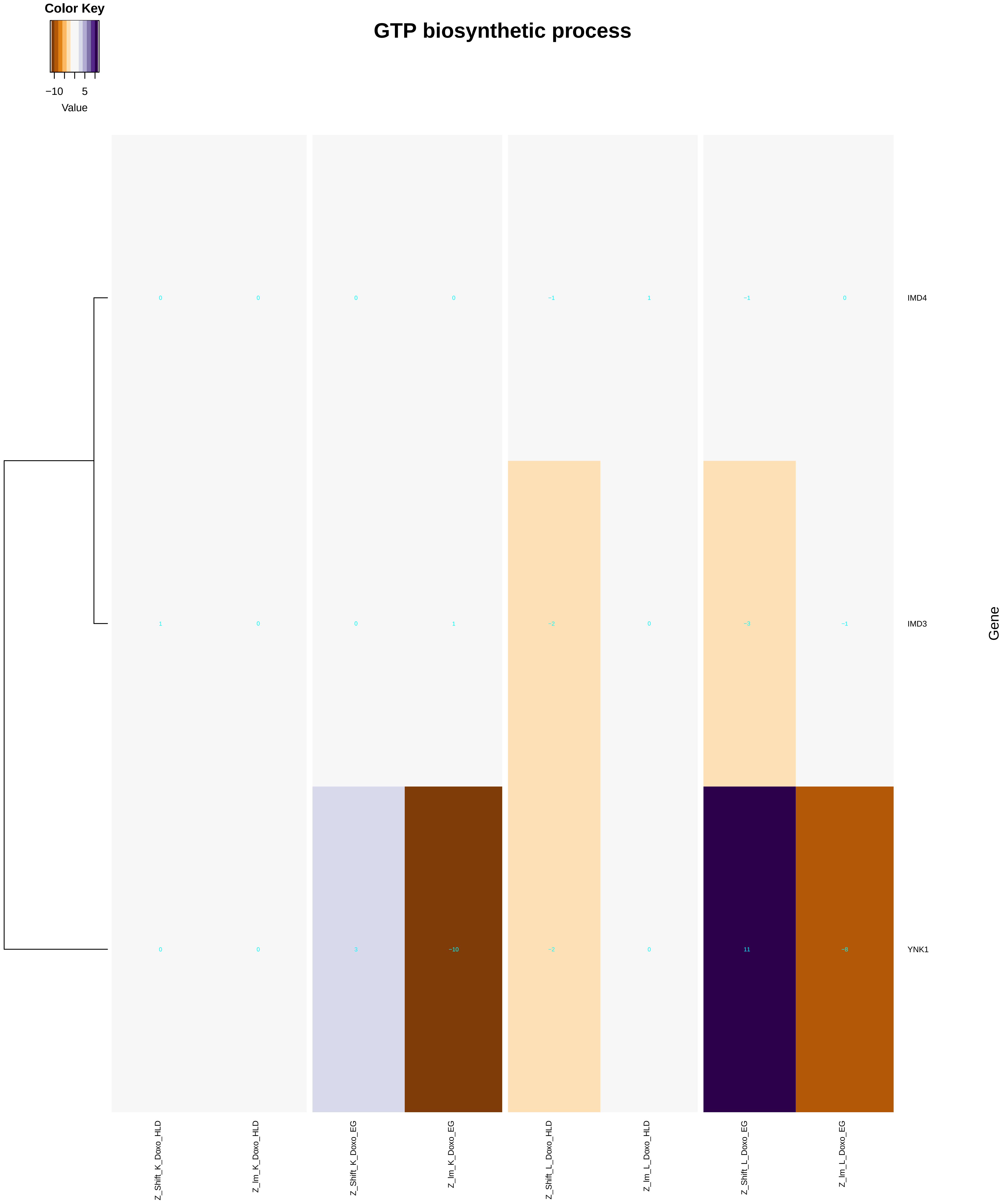

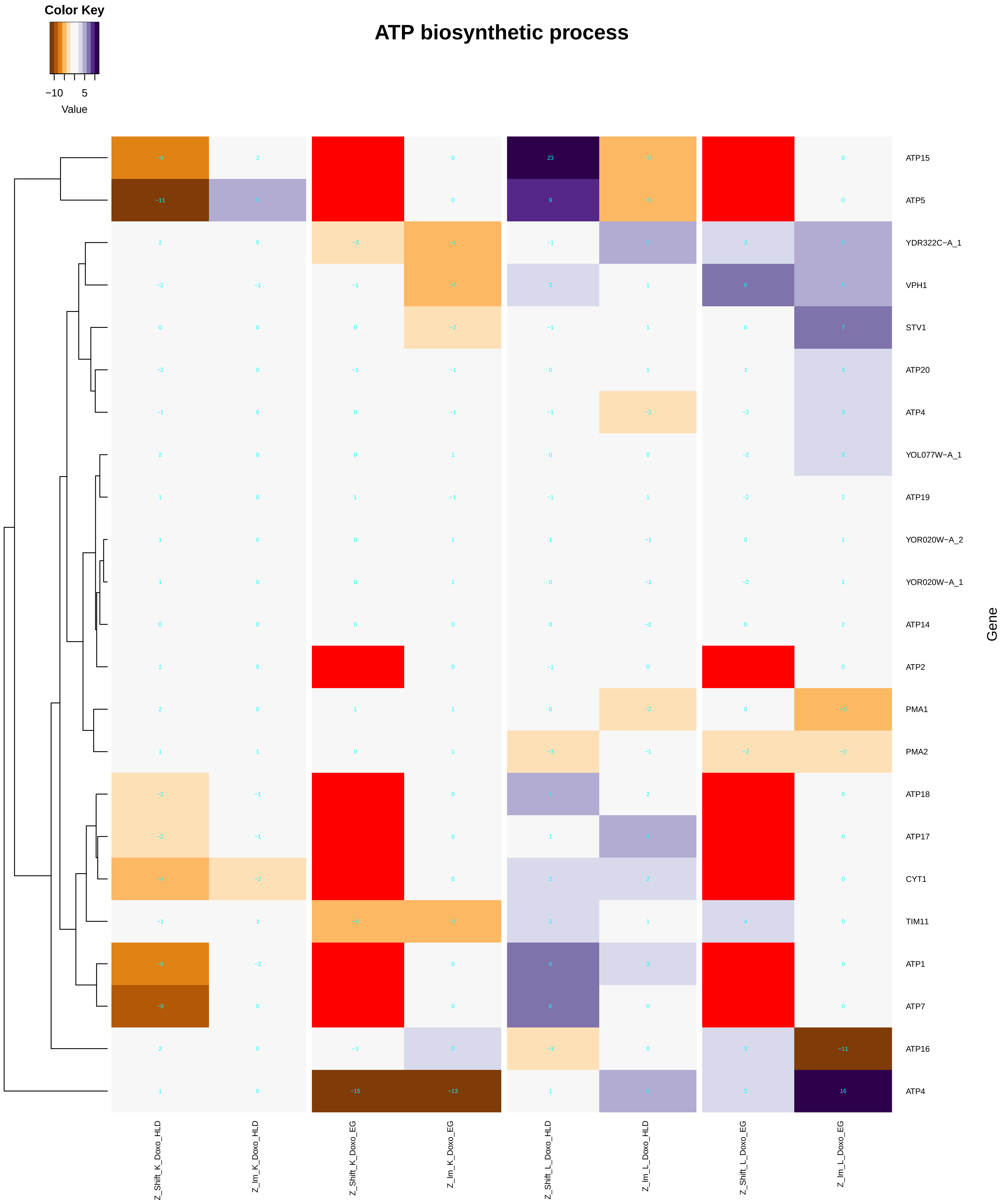
Color Key
−10
5
Value
ATP biosynthetic process
ATP15
ATP5
YDR322C−A_1
VPH1
STV1
ATP20
ATP4
YOL077W−A_1
ATP19
YOR020W−A_2
YOR020W−A_1
ATP14
ATP2
PMA1
PMA2
ATP18
ATP17
CYT1
TIM11
ATP1
ATP7
ATP16
ATP4
Gene
Z_Shift_K_Doxo_HLD
Z_Im_K_Doxo_HLD
Z_Shift_K_Doxo_EG
Z_Im_K_Doxo_EG
Z_Shift_L_Doxo_HLD
Z_Im_L_Doxo_HLD
Z_Shift_L_Doxo_EG
Z_Im_L_Doxo_EG

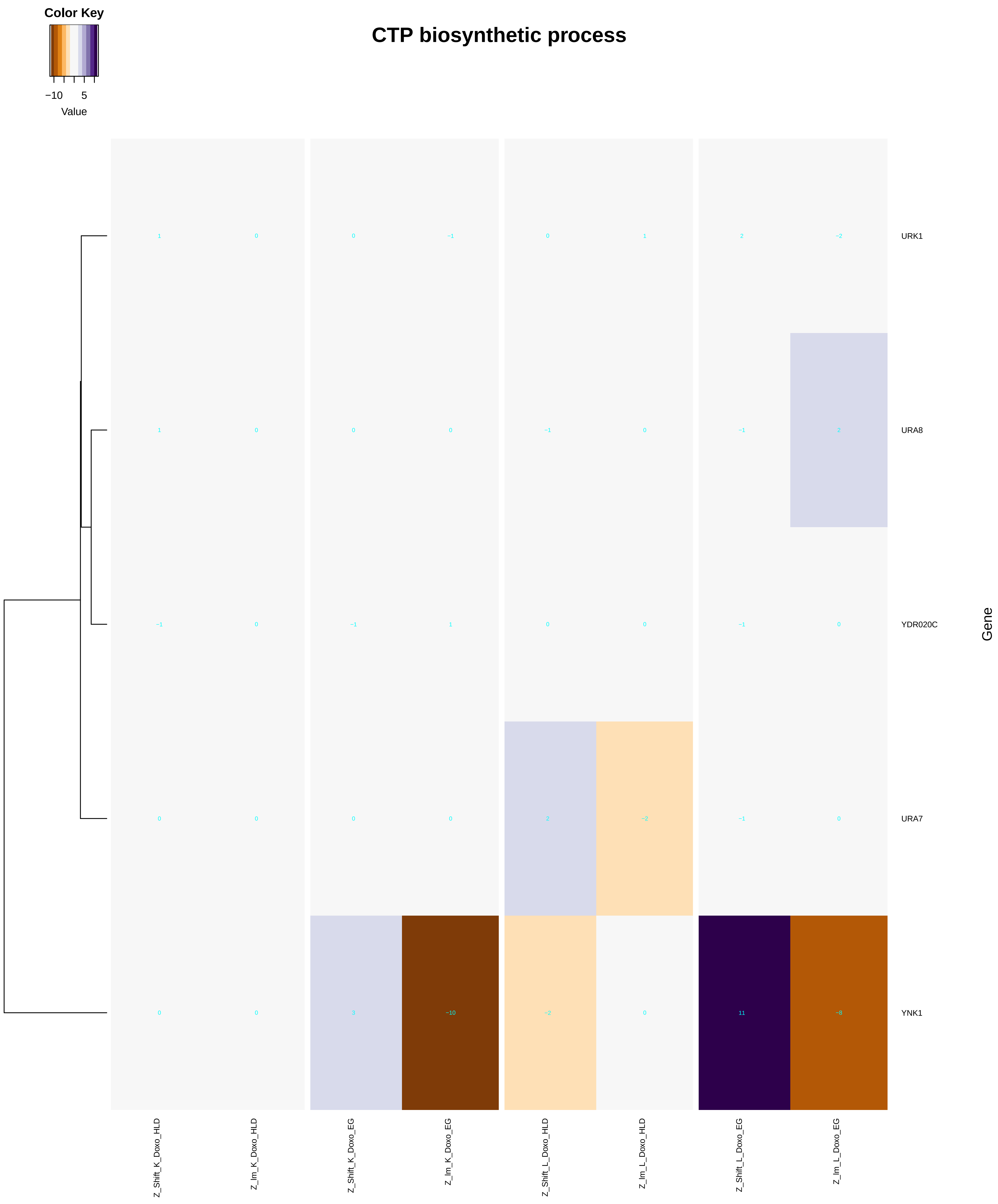
Color Key
−10
5
Value
CTP biosynthetic process
URK1
URA8
YDR020C
URA7
YNK1
Gene
Z_Shift_K_Doxo_HLD
Z_Im_K_Doxo_HLD
Z_Shift_K_Doxo_EG
Z_Im_K_Doxo_EG
Z_Shift_L_Doxo_HLD
Z_Im_L_Doxo_HLD
Z_Shift_L_Doxo_EG
Z_Im_L_Doxo_EG

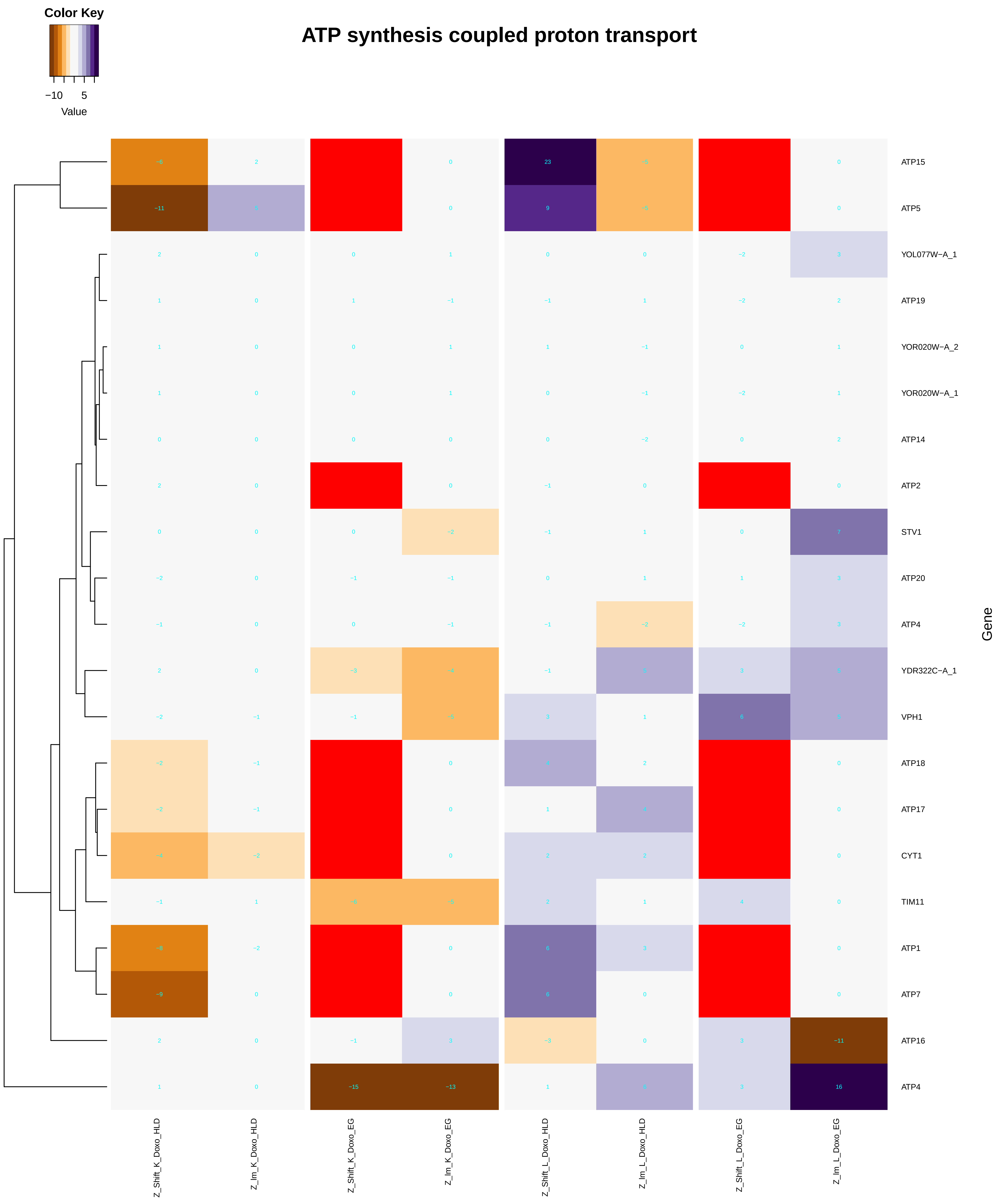
ATP synthesis coupled proton transport
Color Key
−10
5
Value
ATP15
ATP5
YOL077W−A_1
ATP19
YOR020W−A_2
YOR020W−A_1
ATP14
ATP2
STV1
ATP20
ATP4
YDR322C−A_1
VPH1
ATP18
ATP17
CYT1
TIM11
ATP1
ATP7
ATP16
ATP4
Gene
Z_Shift_K_Doxo_HLD
Z_Im_K_Doxo_HLD
Z_Shift_K_Doxo_EG
Z_Im_K_Doxo_EG
Z_Shift_L_Doxo_HLD
Z_Im_L_Doxo_HLD
Z_Shift_L_Doxo_EG
Z_Im_L_Doxo_EG
