# Supplementary material for: A yeast phenomic model for the influence of Warburg metabolism on genetic buffering of doxorubicin
Source: Cancer Metab. 2019 Oct 23;7:9. doi: 10.1186/s40170-019-0201-3 (PMC6806529; doi:10.1186/s40170-019-0201-3)
Supplement: Supplementary file 9 — Additional file 9. GO term-specific heatmaps for REMc/GTF-enriched clusters. GO term-specific heatmaps for significant GO process terms were generated as described in methods and Figs. 3 and 4. Any related child terms are presented in subsequent pages of the parent file name. GO terms with more than 100 children, with 2 or fewer genes annotated to the term, or a file size over 300KB are not shown. All heatmaps are generated with the same layout (see Figs. 3 and 4). [file 40170_2019_201_MOESM9_ESM.bz2 › Additional_File9_GOTermHeatmaps/Additional_File9_GOTermHeatmap/positive_regulation_of_gene_expression,_epigenetic.pdf]

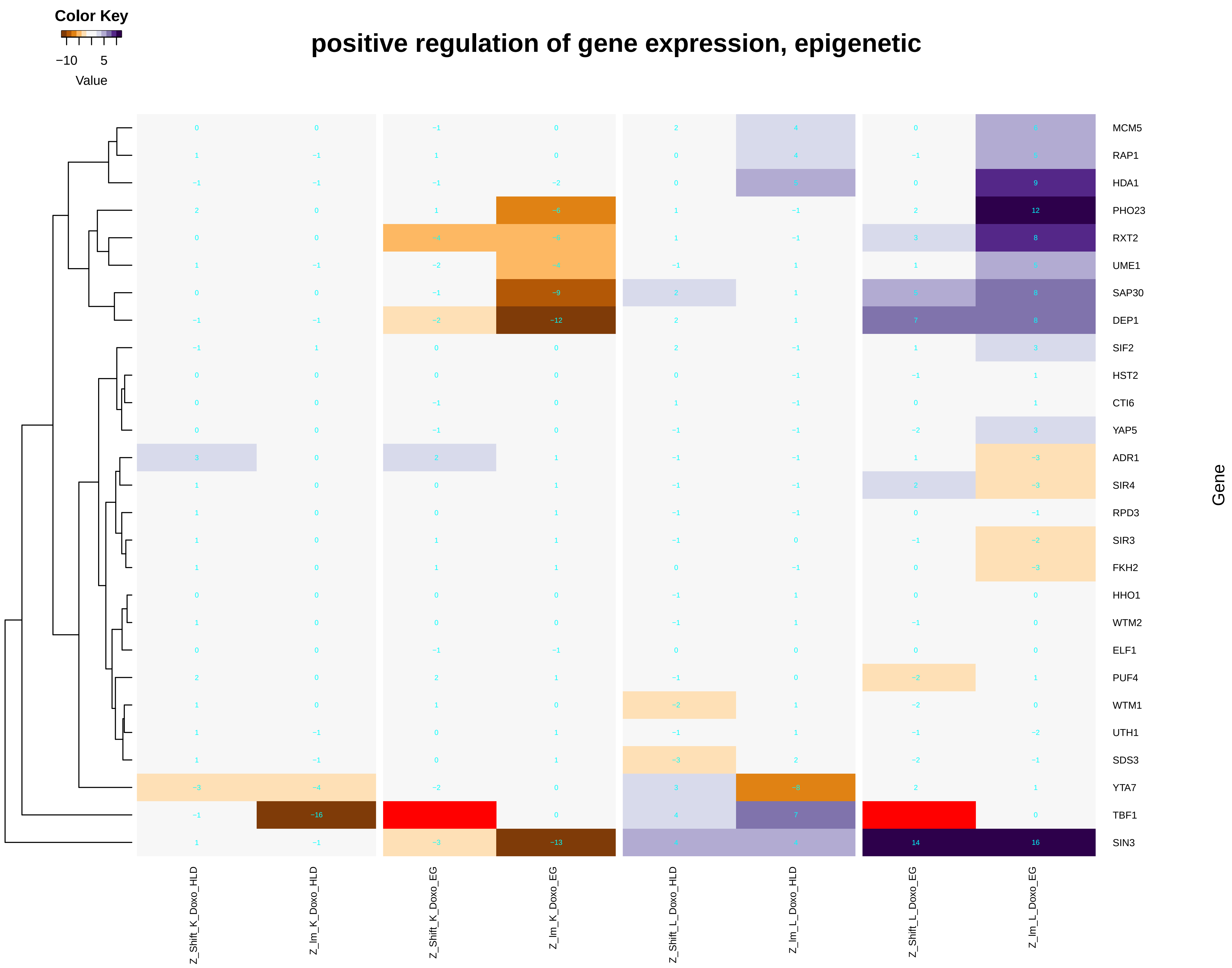

positive regulation of gene expression, epigenetic
Color Key
−10
5
Value
Gene
MCM5
RAP1
HDA1
PHO23
RXT2
UME1
SAP30
DEP1
SIF2
HST2
CTI6
YAP5
ADR1
SIR4
RPD3
SIR3
FKH2
HHO1
WTM2
ELF1
PUF4
WTM1
UTH1
SDS3
YTA7
TBF1
SIN3
Z_Shift_K_Doxo_HLD
Z_Im_K_Doxo_HLD
Z_Shift_K_Doxo_EG
Z_Im_K_Doxo_EG
Z_Shift_L_Doxo_HLD
Z_Im_L_Doxo_HLD
Z_Shift_L_Doxo_EG
Z_Im_L_Doxo_EG

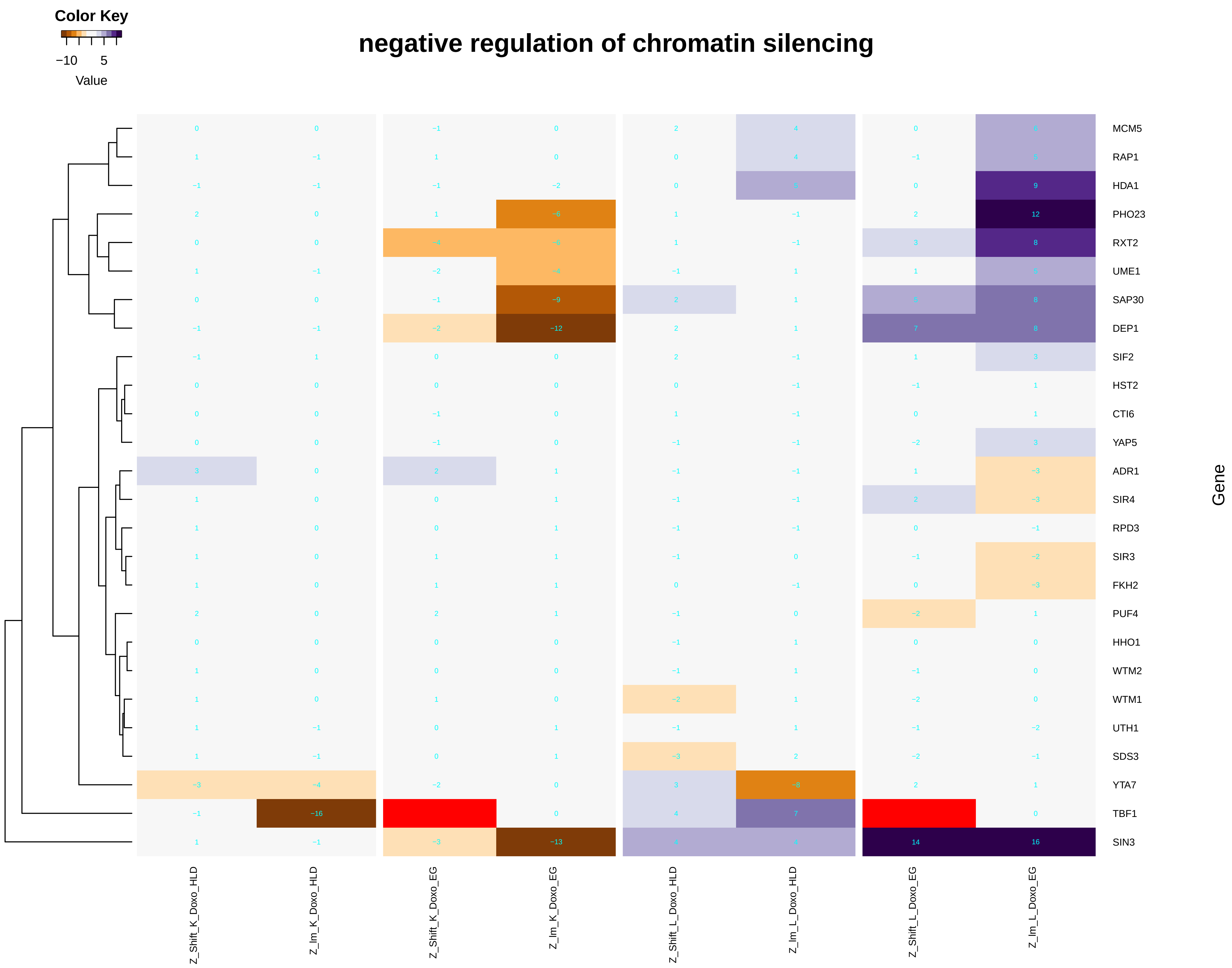
Color Key
−10
5
Value
negative regulation of chromatin silencing
MCM5
RAP1
HDA1
PHO23
RXT2
UME1
SAP30
DEP1
SIF2
HST2
CTI6
YAP5
ADR1
SIR4
RPD3
SIR3
FKH2
PUF4
HHO1
WTM2
WTM1
UTH1
SDS3
YTA7
TBF1
SIN3
Gene
Z_Shift_K_Doxo_HLD
Z_Im_K_Doxo_HLD
Z_Shift_K_Doxo_EG
Z_Im_K_Doxo_EG
Z_Shift_L_Doxo_HLD
Z_Im_L_Doxo_HLD
Z_Shift_L_Doxo_EG
Z_Im_L_Doxo_EG

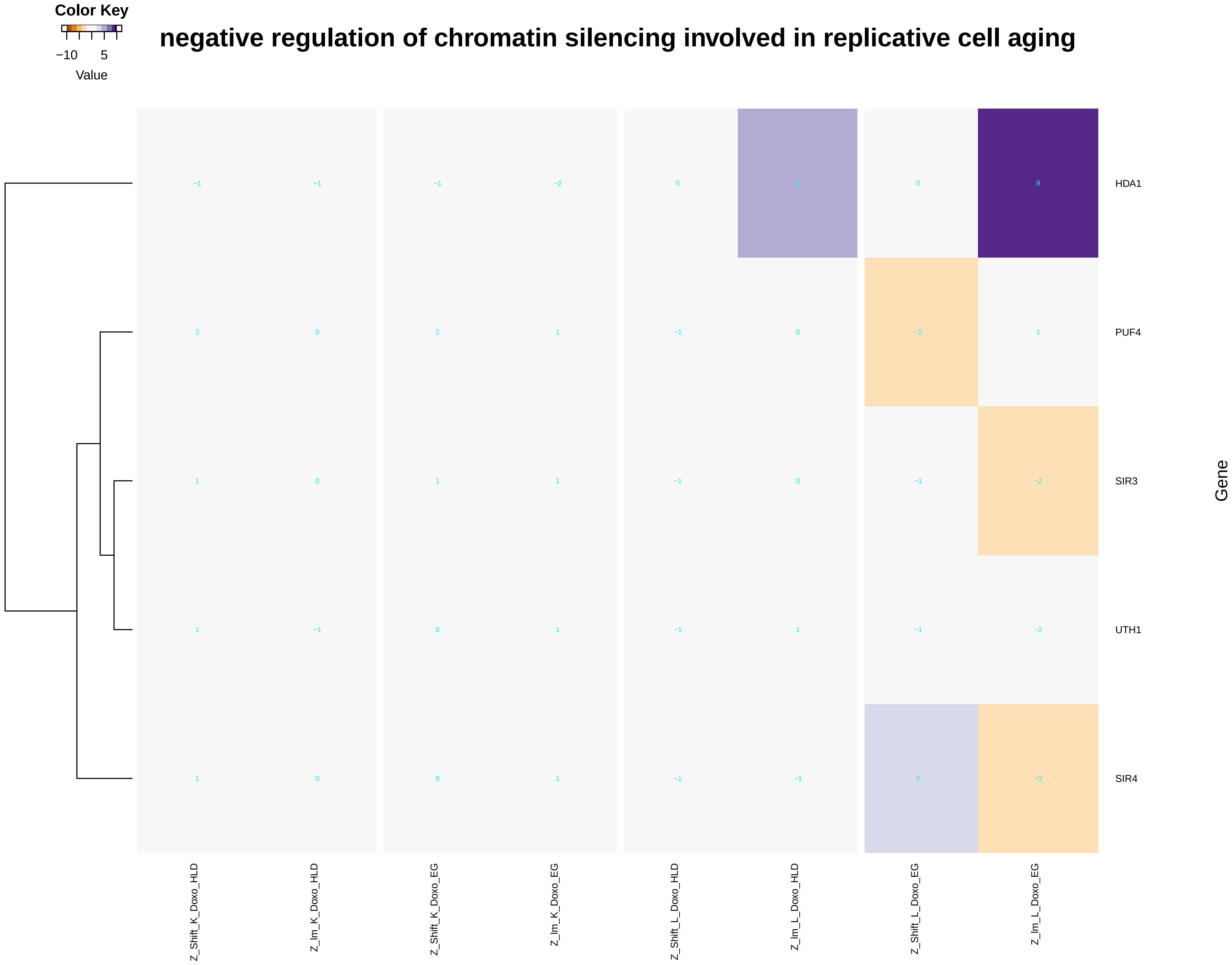
Color Key
−10
5
Value
negative regulation of chromatin silencing involved in replicative cell aging
HDA1
PUF4
SIR3
UTH1
SIR4
Gene
Z_Shift_K_Doxo_HLD
Z_Im_K_Doxo_HLD
Z_Shift_K_Doxo_EG
Z_Im_K_Doxo_EG
Z_Shift_L_Doxo_HLD
Z_Im_L_Doxo_HLD
Z_Shift_L_Doxo_EG
Z_Im_L_Doxo_EG

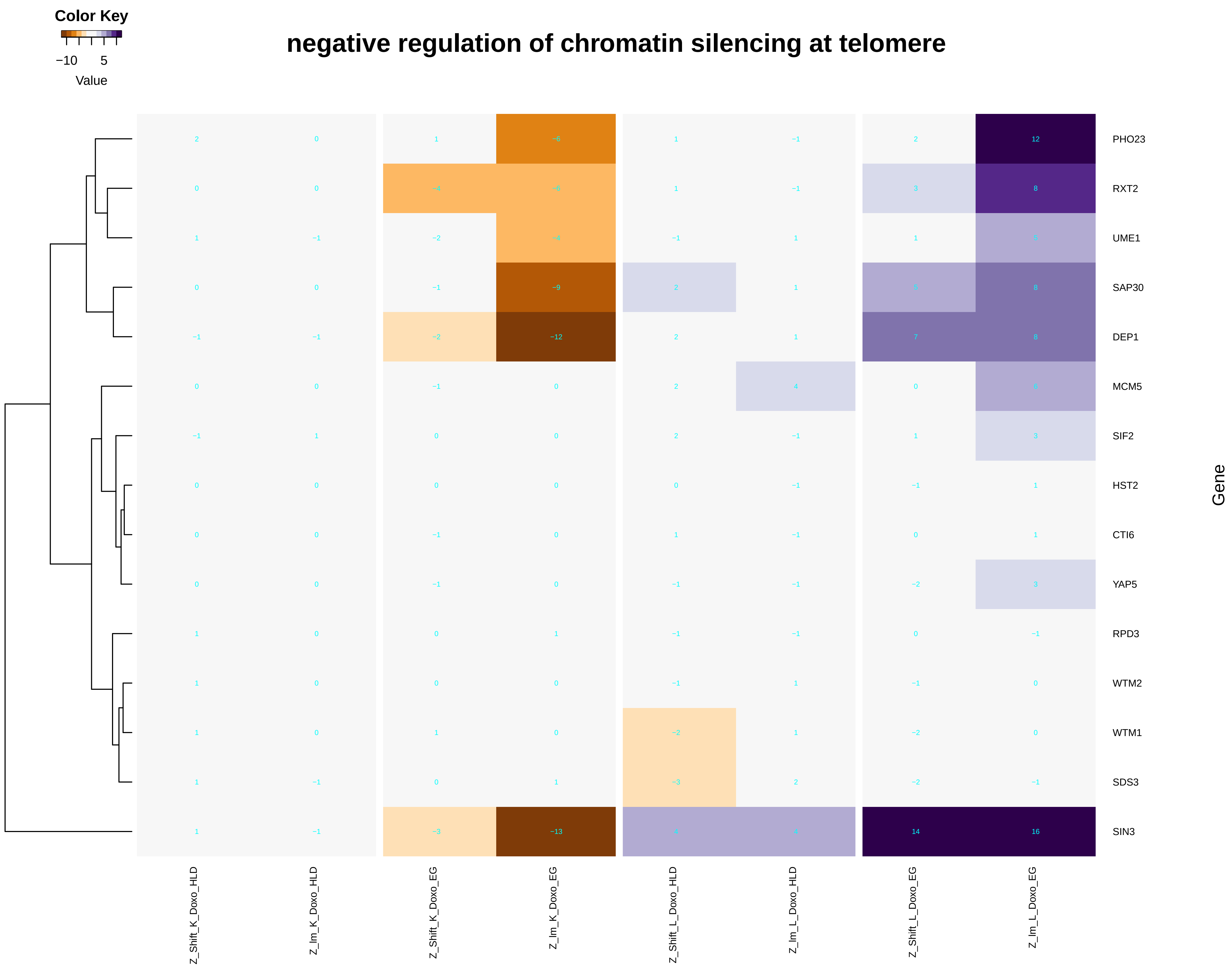

Color Key
−10
5
Value
negative regulation of chromatin silencing at telomere
PHO23
RXT2
UME1
SAP30
DEP1
MCM5
SIF2
HST2
CTI6
YAP5
RPD3
WTM2
WTM1
SDS3
SIN3
Gene
Z_Shift_K_Doxo_HLD
Z_Im_K_Doxo_HLD
Z_Shift_K_Doxo_EG
Z_Im_K_Doxo_EG
Z_Shift_L_Doxo_HLD
Z_Im_L_Doxo_HLD
Z_Shift_L_Doxo_EG
Z_Im_L_Doxo_EG

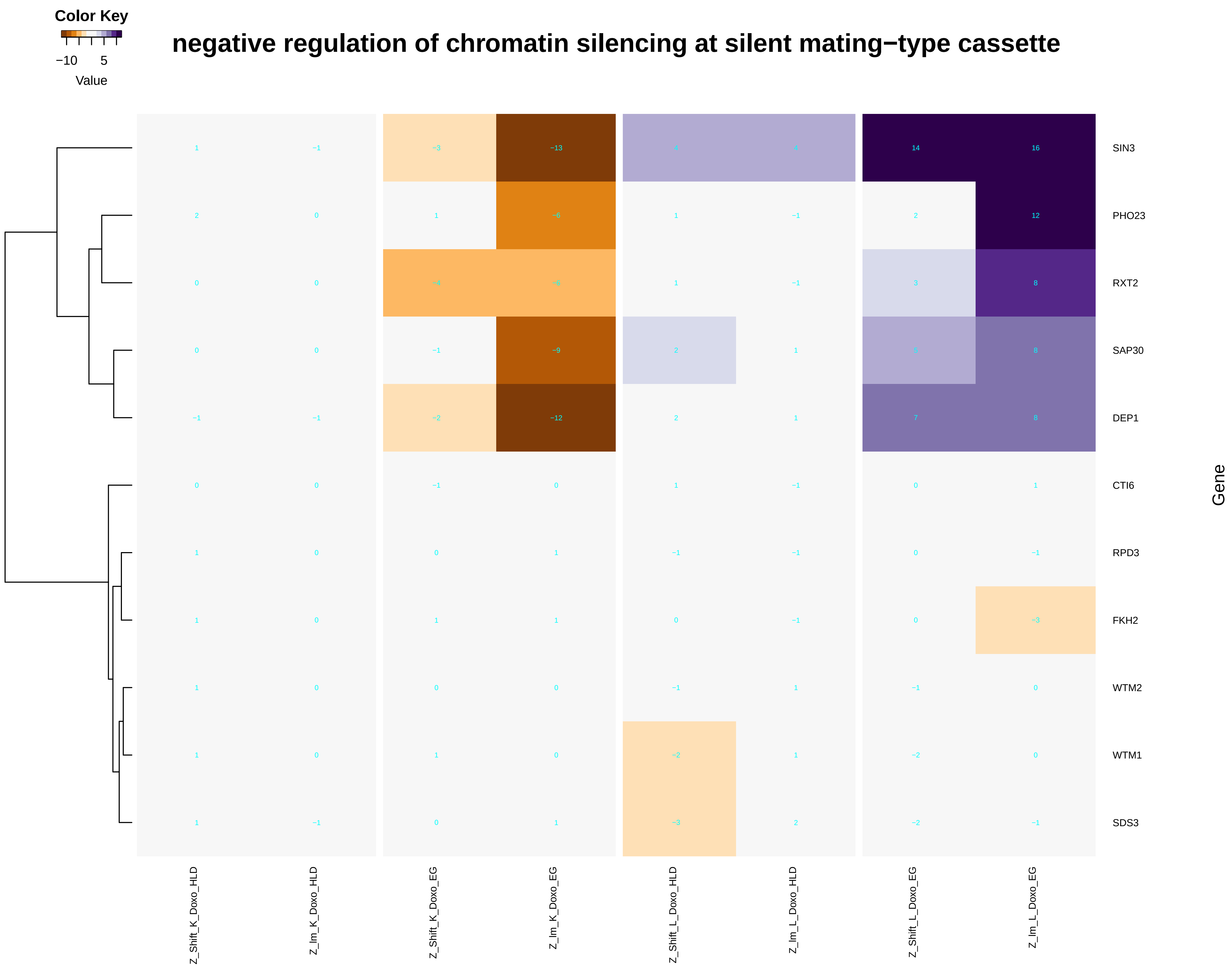
Color Key
−10
5
Value
negative regulation of chromatin silencing at silent mating−type cassette
SIN3
PHO23
RXT2
SAP30
DEP1
CTI6
RPD3
FKH2
WTM2
WTM1
SDS3
Gene
Z_Shift_K_Doxo_HLD
Z_Im_K_Doxo_HLD
Z_Shift_K_Doxo_EG
Z_Im_K_Doxo_EG
Z_Shift_L_Doxo_HLD
Z_Im_L_Doxo_HLD
Z_Shift_L_Doxo_EG
Z_Im_L_Doxo_EG

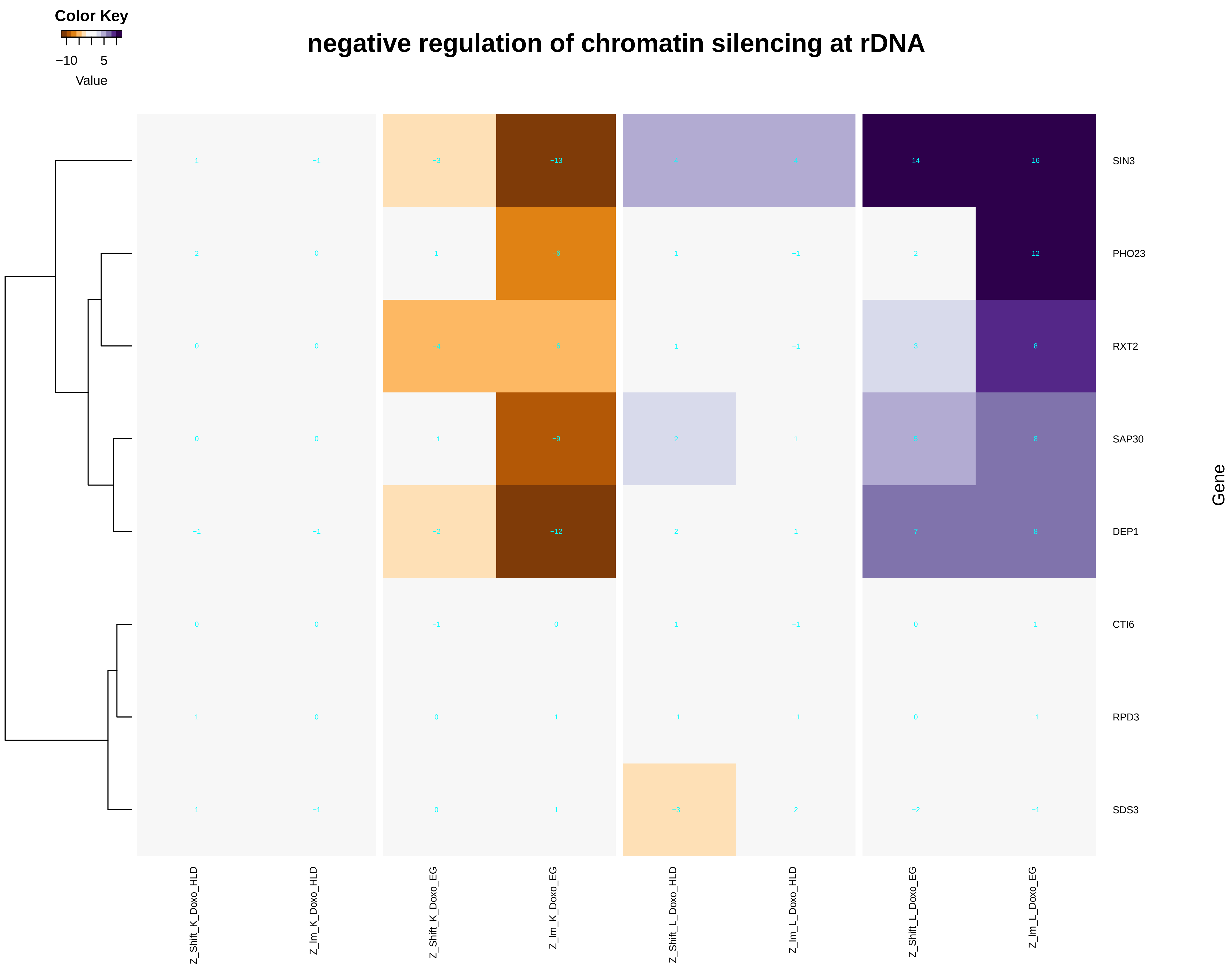
negative regulation of chromatin silencing at rDNA
Color Key
−10
5
Value
SIN3
PHO23
RXT2
SAP30
DEP1
CTI6
RPD3
SDS3
Gene
Z_Shift_K_Doxo_HLD
Z_lm_K_Doxo_HLD
Z_Shift_K_Doxo_EG
Z_lm_K_Doxo_EG
Z_Shift_L_Doxo_HLD
Z_lm_L_Doxo_HLD
Z_Shift_L_Doxo_EG
Z_lm_L_Doxo_EG
