# Supplementary figures and images for: A yeast phenomic model for the influence of Warburg metabolism on genetic buffering of doxorubicin (part 2 of 2)
Source: Cancer Metab. 2019 Oct 23;7:9. doi: 10.1186/s40170-019-0201-3 (PMC6806529; doi:10.1186/s40170-019-0201-3)

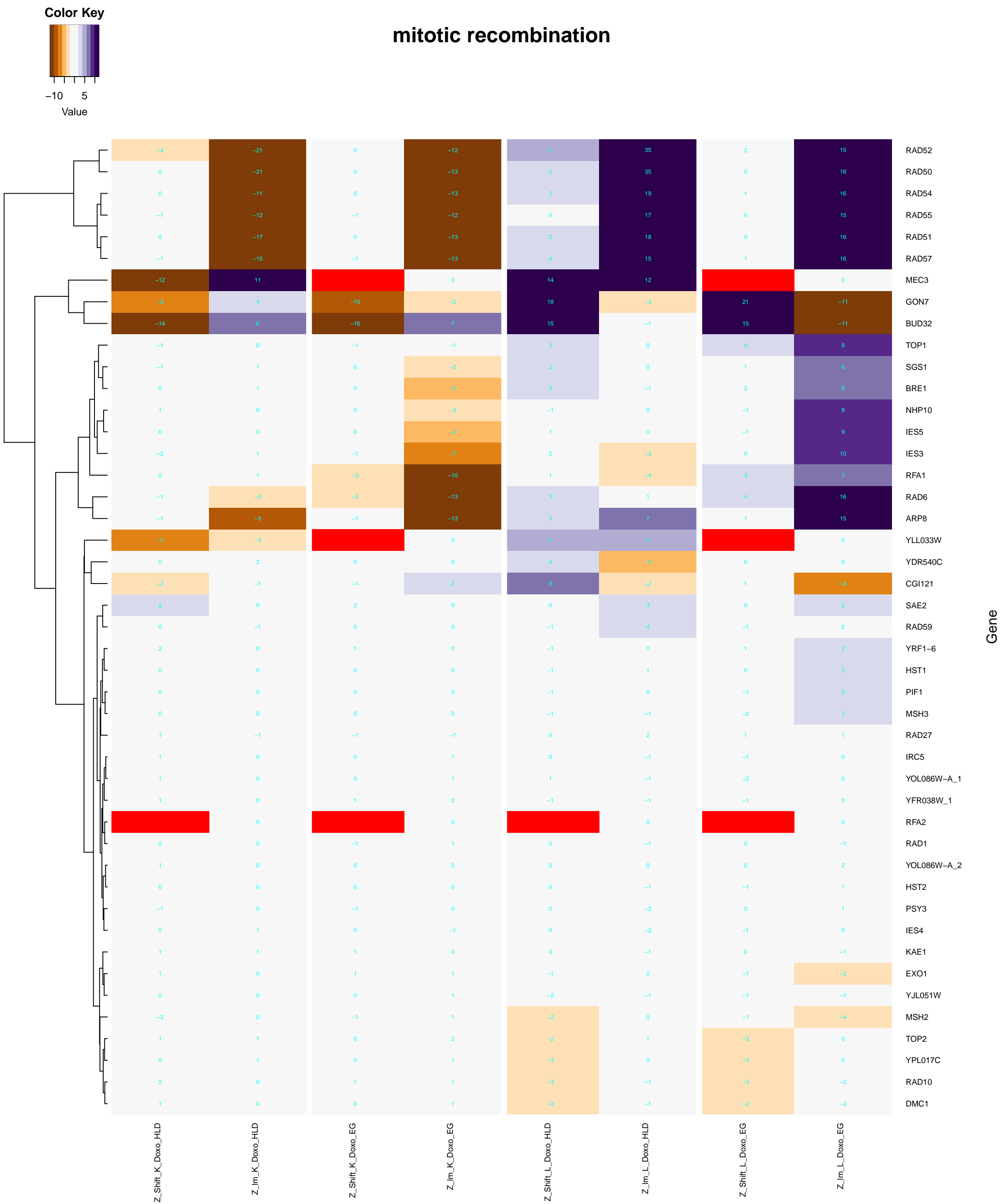

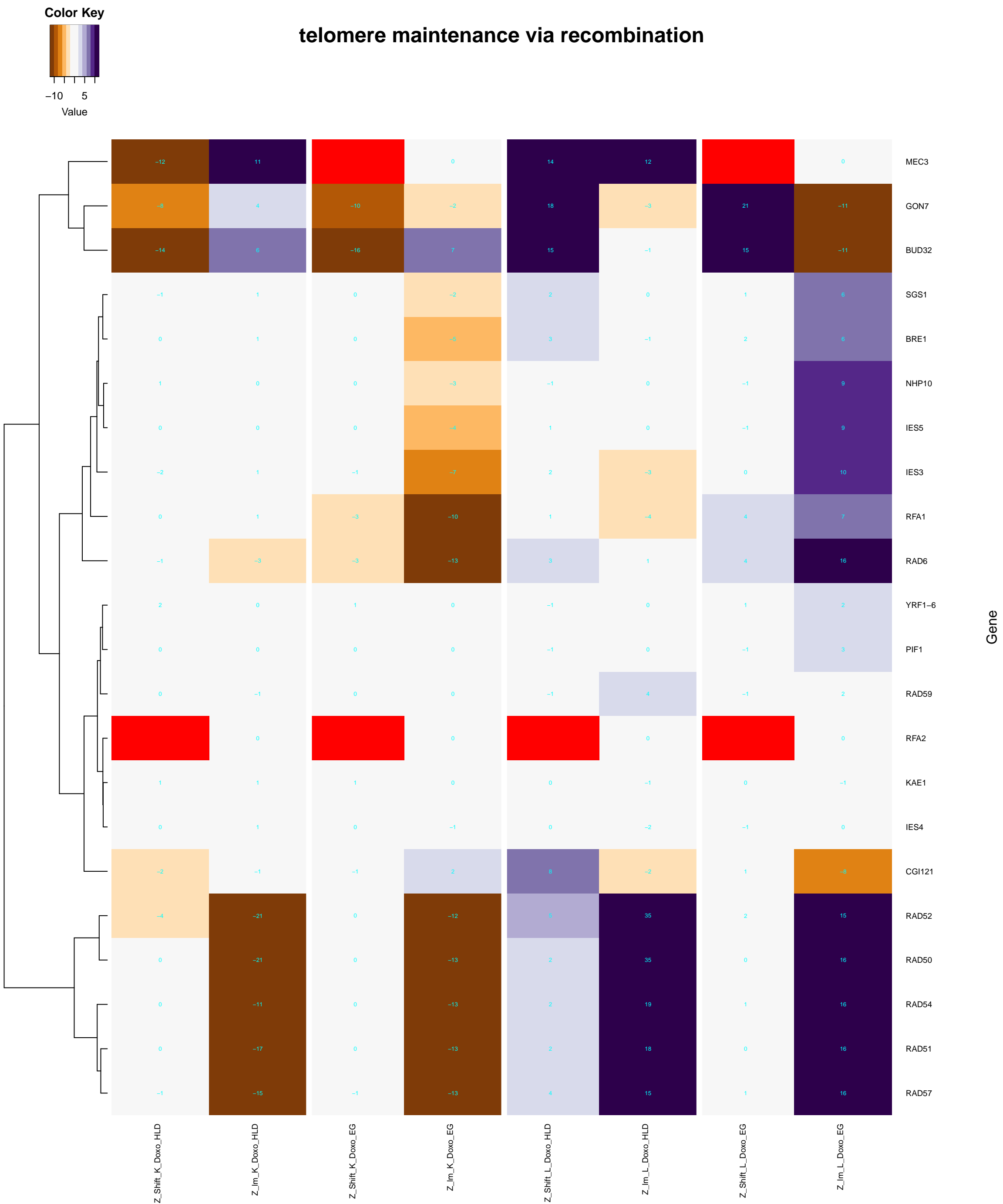

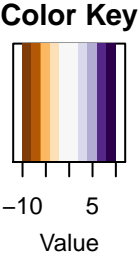

gene conversion at mating-type locus

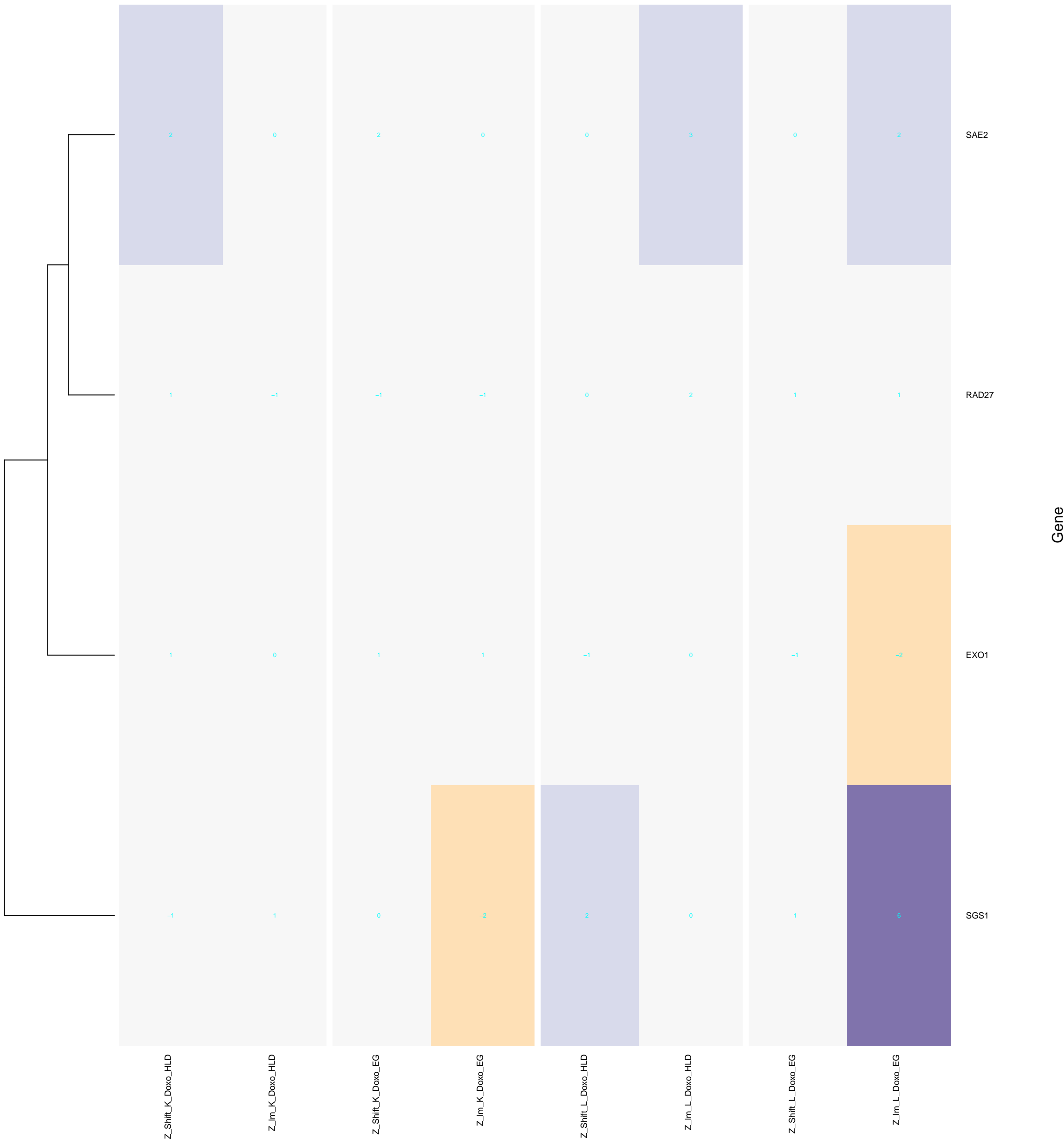

Supplement: Supplementary file 9 — Additional file 9. GO term-specific heatmaps for REMc/GTF-enriched clusters. GO term-specific heatmaps for significant GO process terms were generated as described in methods and Figs. 3 and 4. Any related child terms are presented in subsequent pages of the parent file name. GO terms with more than 100 children, with 2 or fewer genes annotated to the term, or a file size over 300KB are not shown. All heatmaps are generated with the same layout (see Figs. 3 and 4). [file 40170_2019_201_MOESM9_ESM.bz2 › Additional_File9_GOTermHeatmaps/Additional_File9_GOTermHeatmap/mitotic_recombination.pdf]

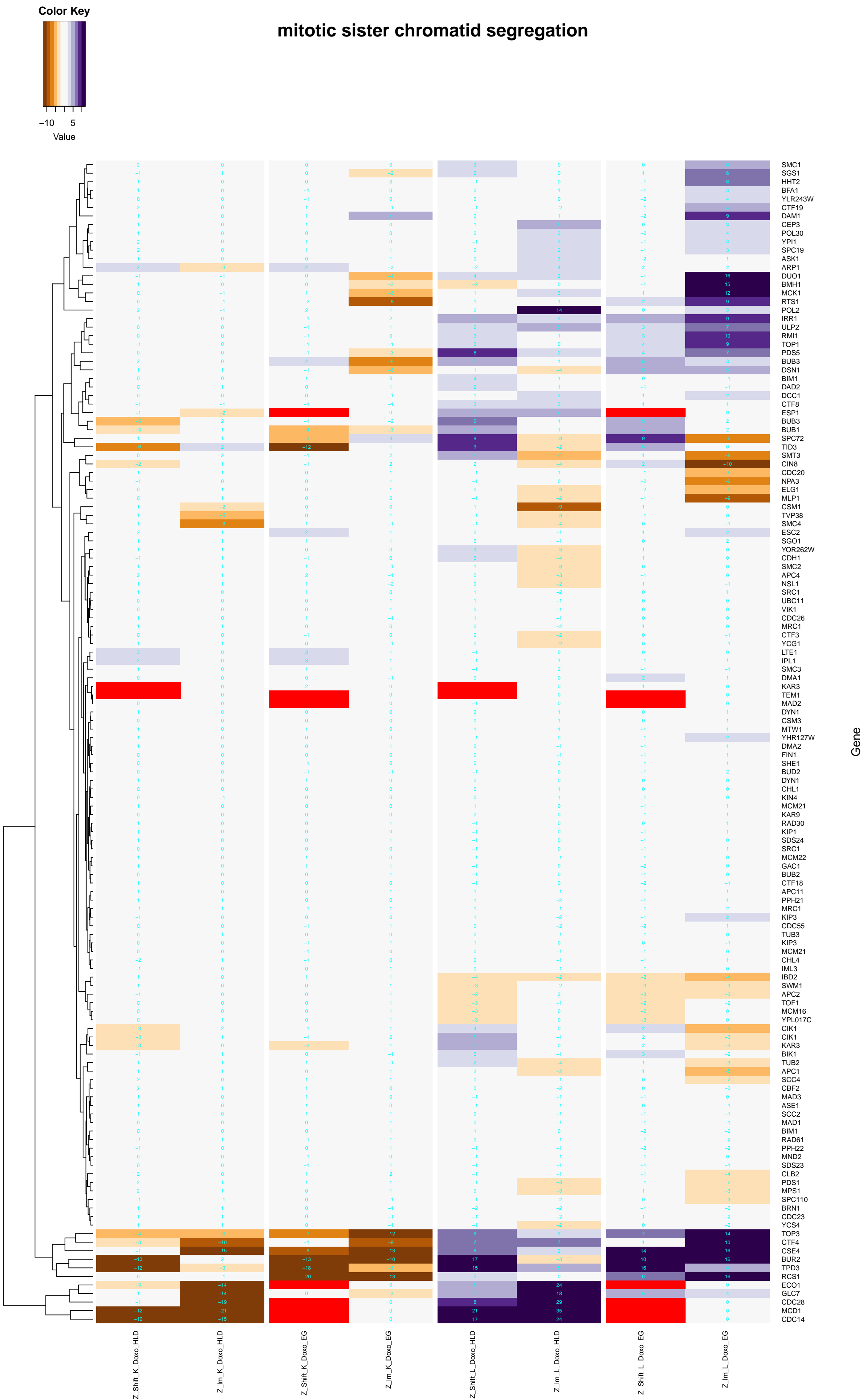

Supplement: Supplementary file 9 — Additional file 9. GO term-specific heatmaps for REMc/GTF-enriched clusters. GO term-specific heatmaps for significant GO process terms were generated as described in methods and Figs. 3 and 4. Any related child terms are presented in subsequent pages of the parent file name. GO terms with more than 100 children, with 2 or fewer genes annotated to the term, or a file size over 300KB are not shown. All heatmaps are generated with the same layout (see Figs. 3 and 4). [file 40170_2019_201_MOESM9_ESM.bz2 › Additional_File9_GOTermHeatmaps/Additional_File9_GOTermHeatmap/mitotic_sister_chromatid_segregation.pdf]

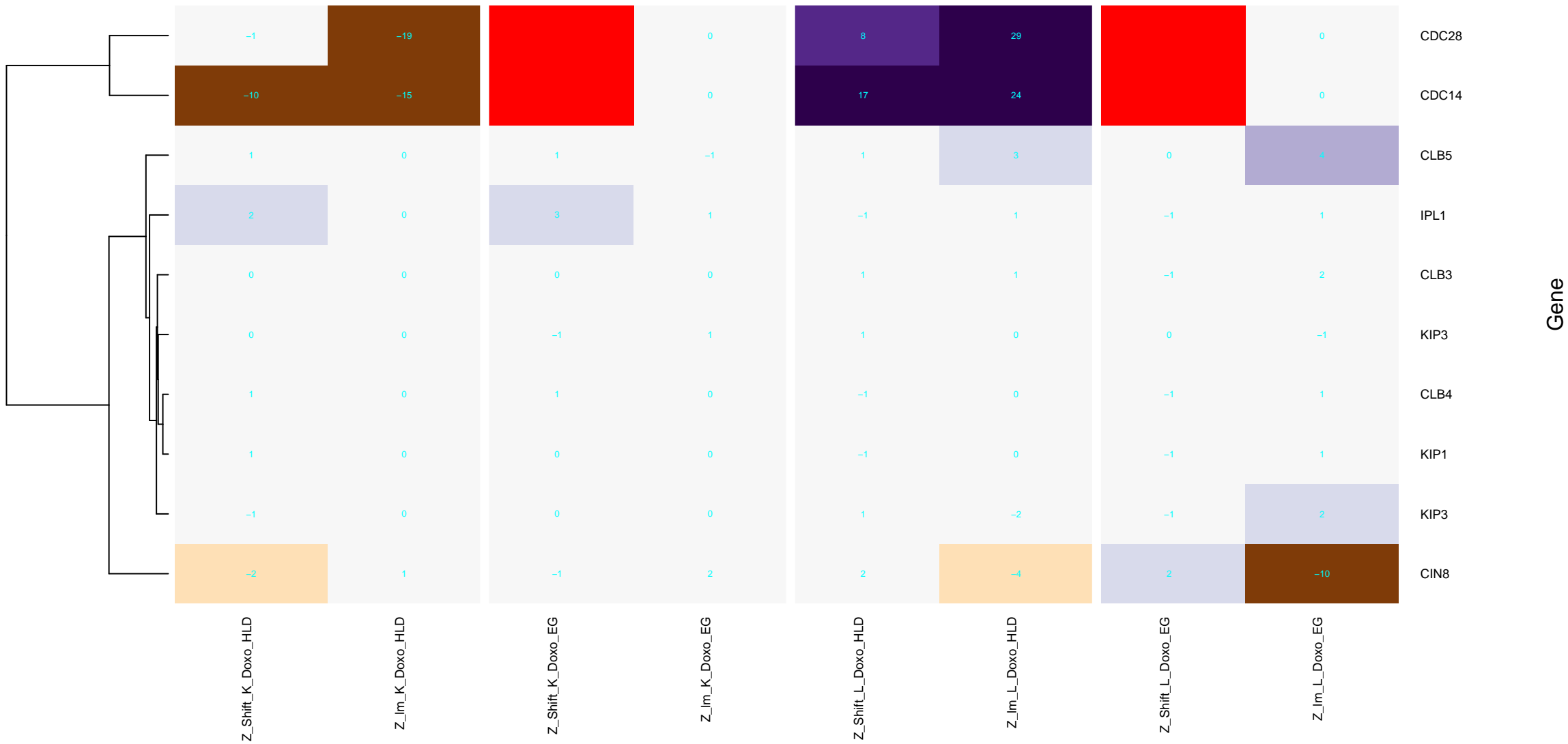

Supplement: Supplementary file 9 — Additional file 9. GO term-specific heatmaps for REMc/GTF-enriched clusters. GO term-specific heatmaps for significant GO process terms were generated as described in methods and Figs. 3 and 4. Any related child terms are presented in subsequent pages of the parent file name. GO terms with more than 100 children, with 2 or fewer genes annotated to the term, or a file size over 300KB are not shown. All heatmaps are generated with the same layout (see Figs. 3 and 4). [file 40170_2019_201_MOESM9_ESM.bz2 › Additional_File9_GOTermHeatmaps/Additional_File9_GOTermHeatmap/mitotic_spindle_assembly.pdf]

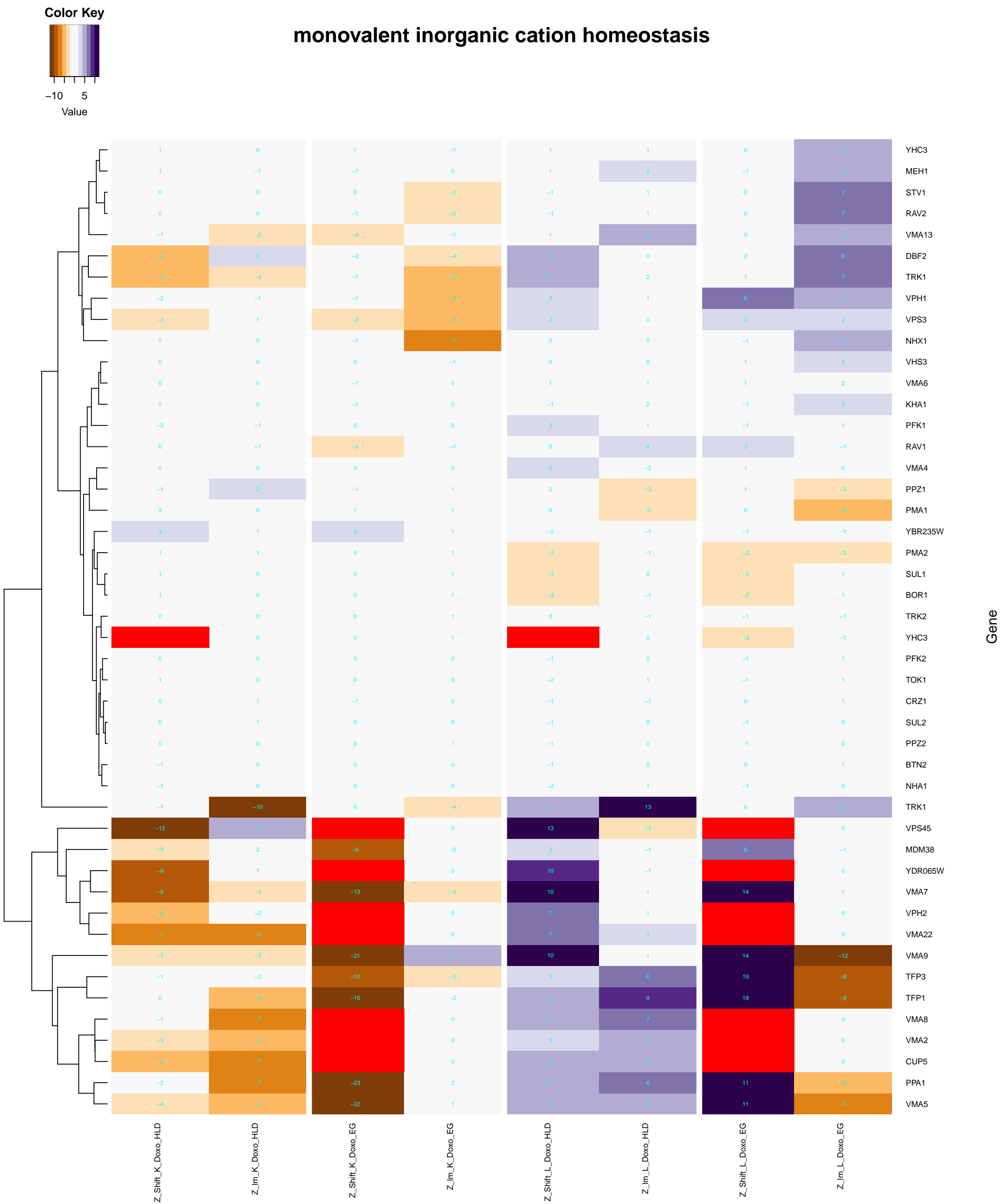

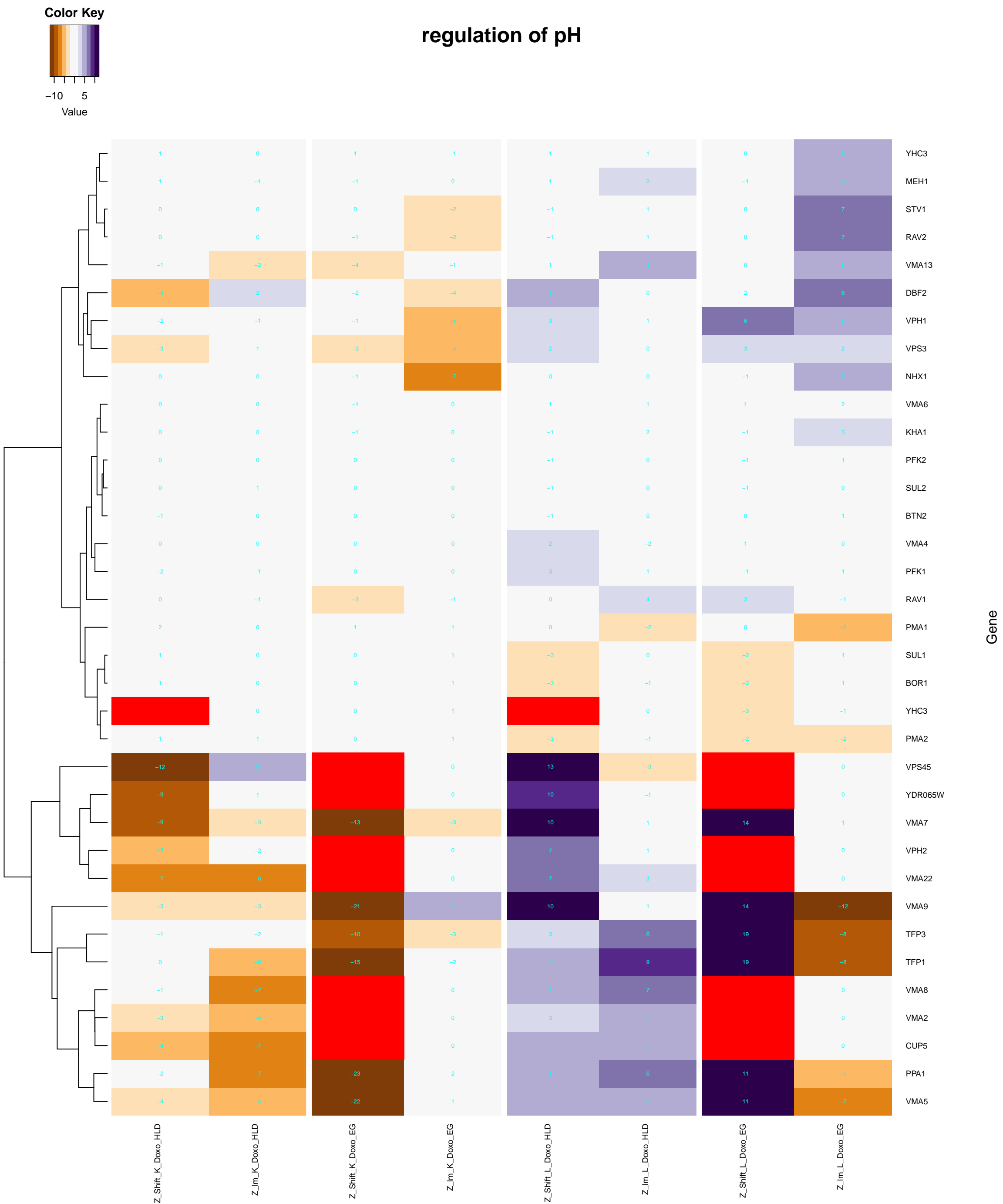

## cellular monovalent inorganic cation homeostasis

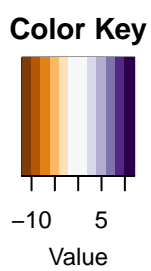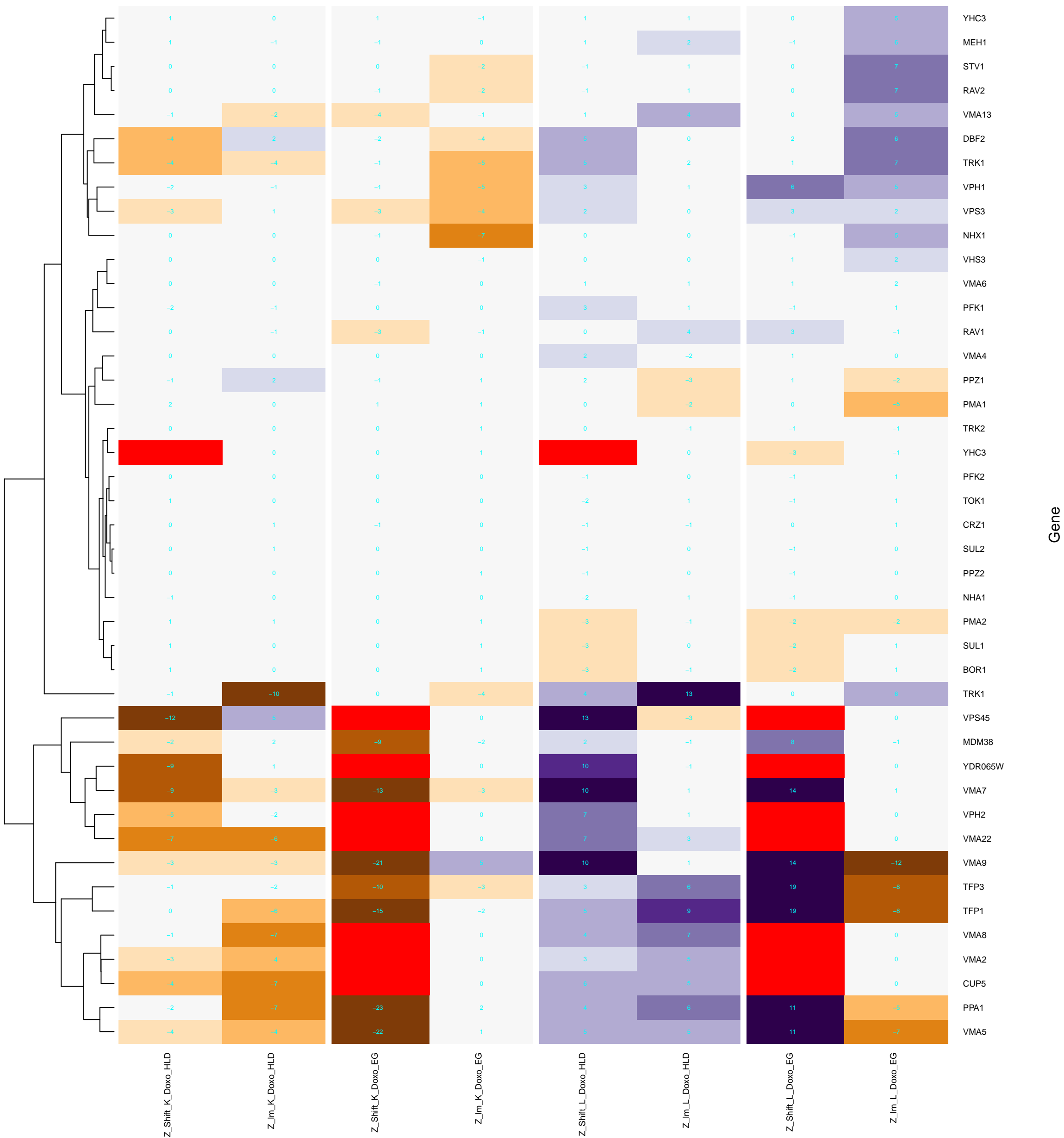

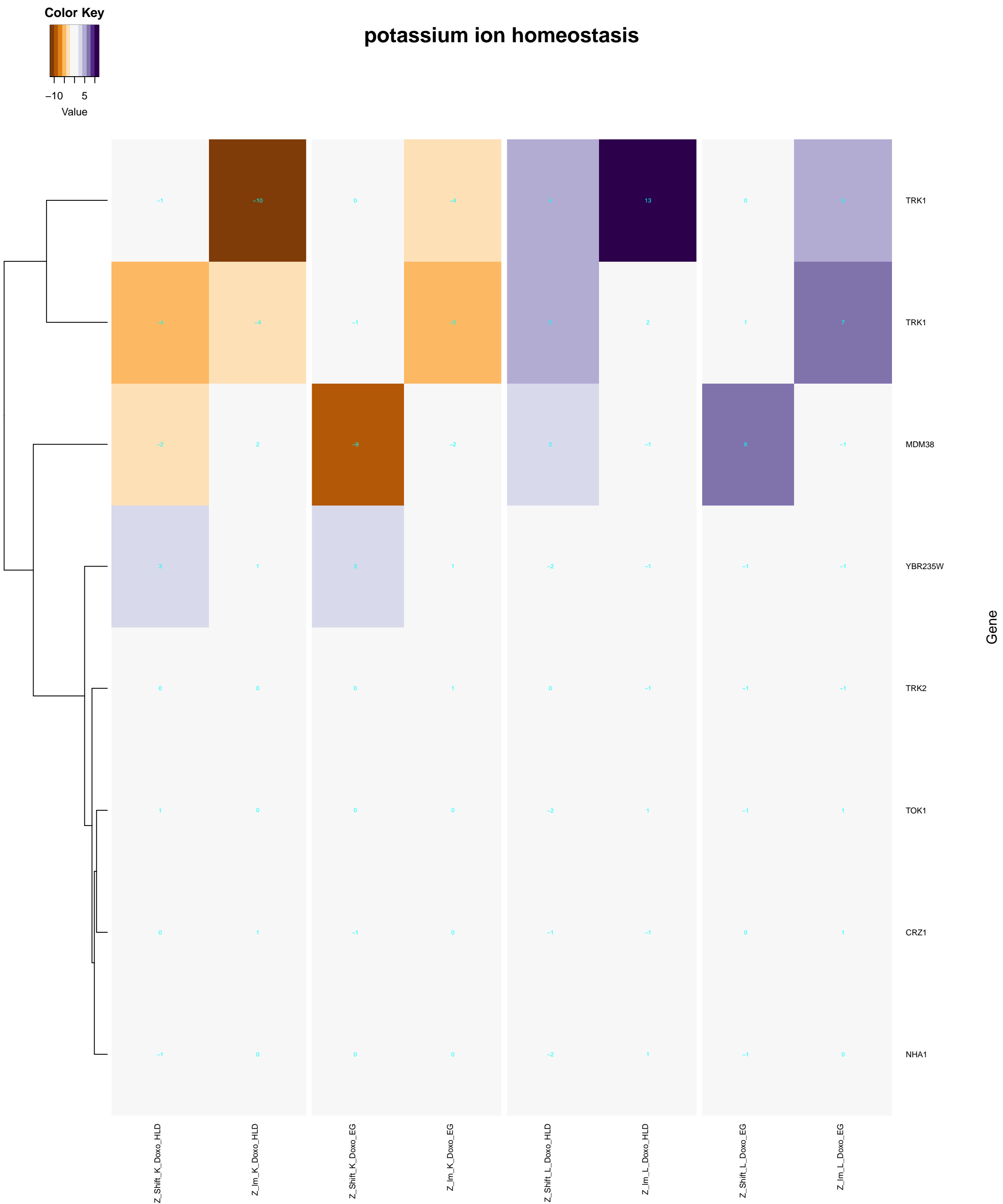

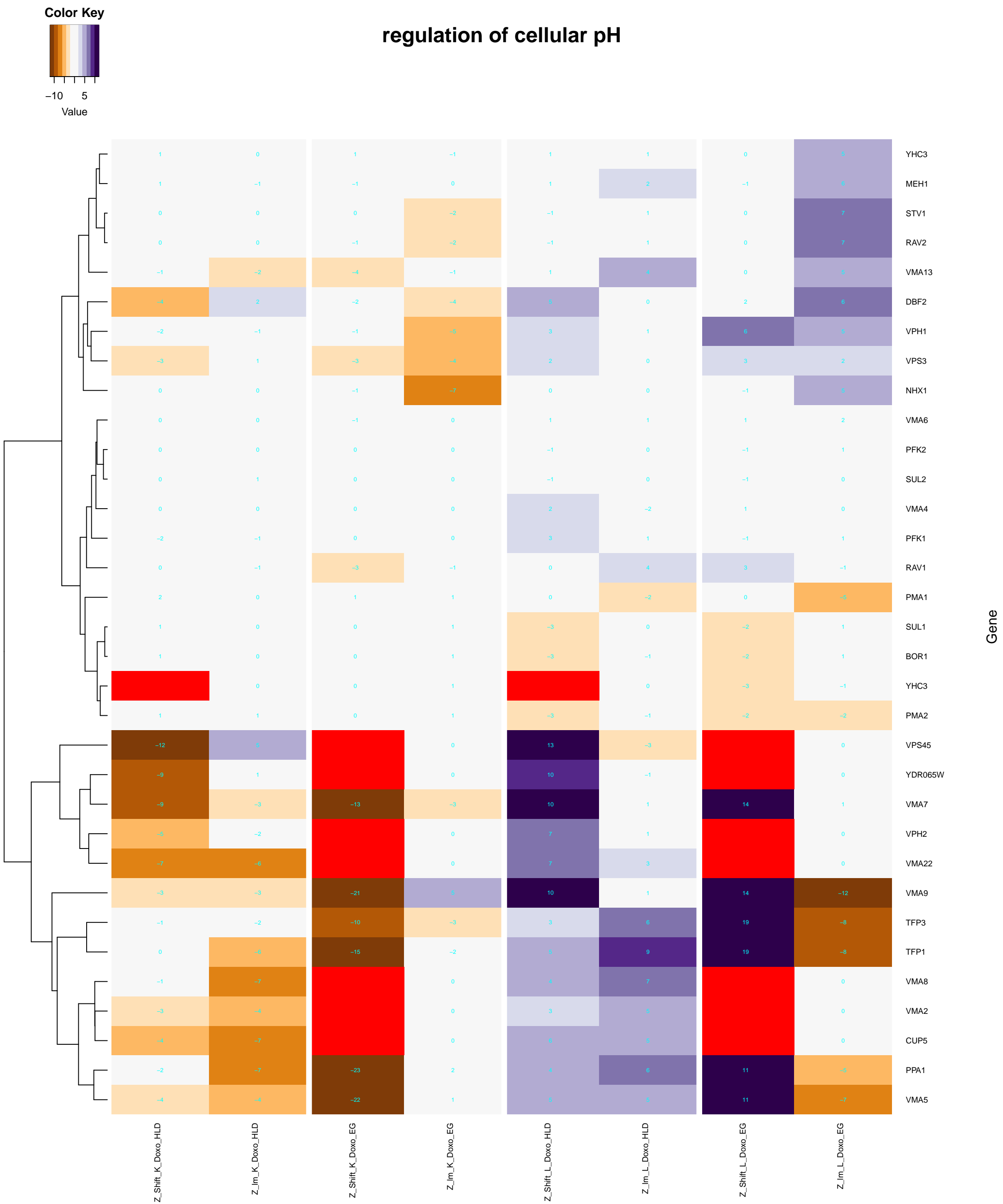

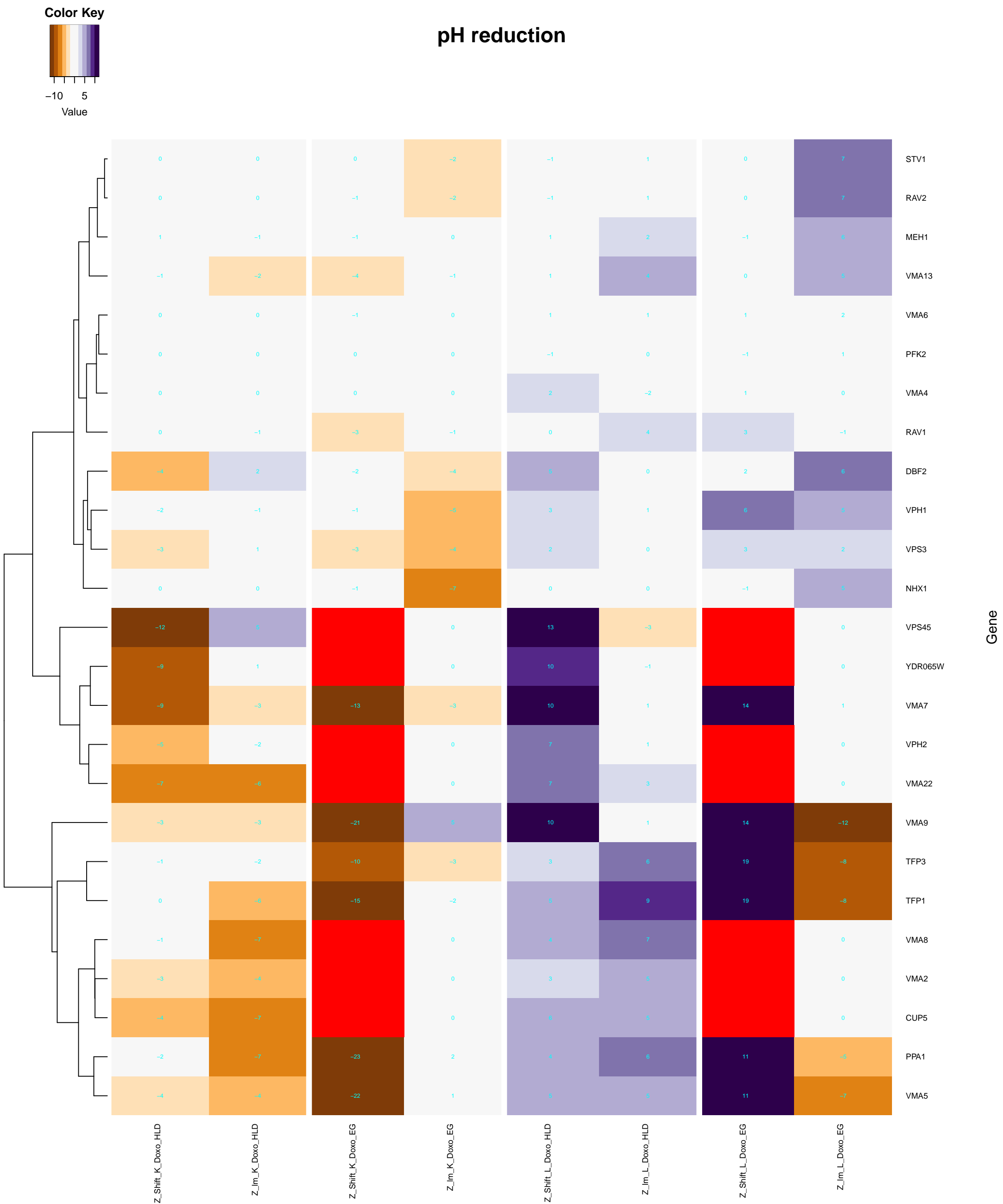

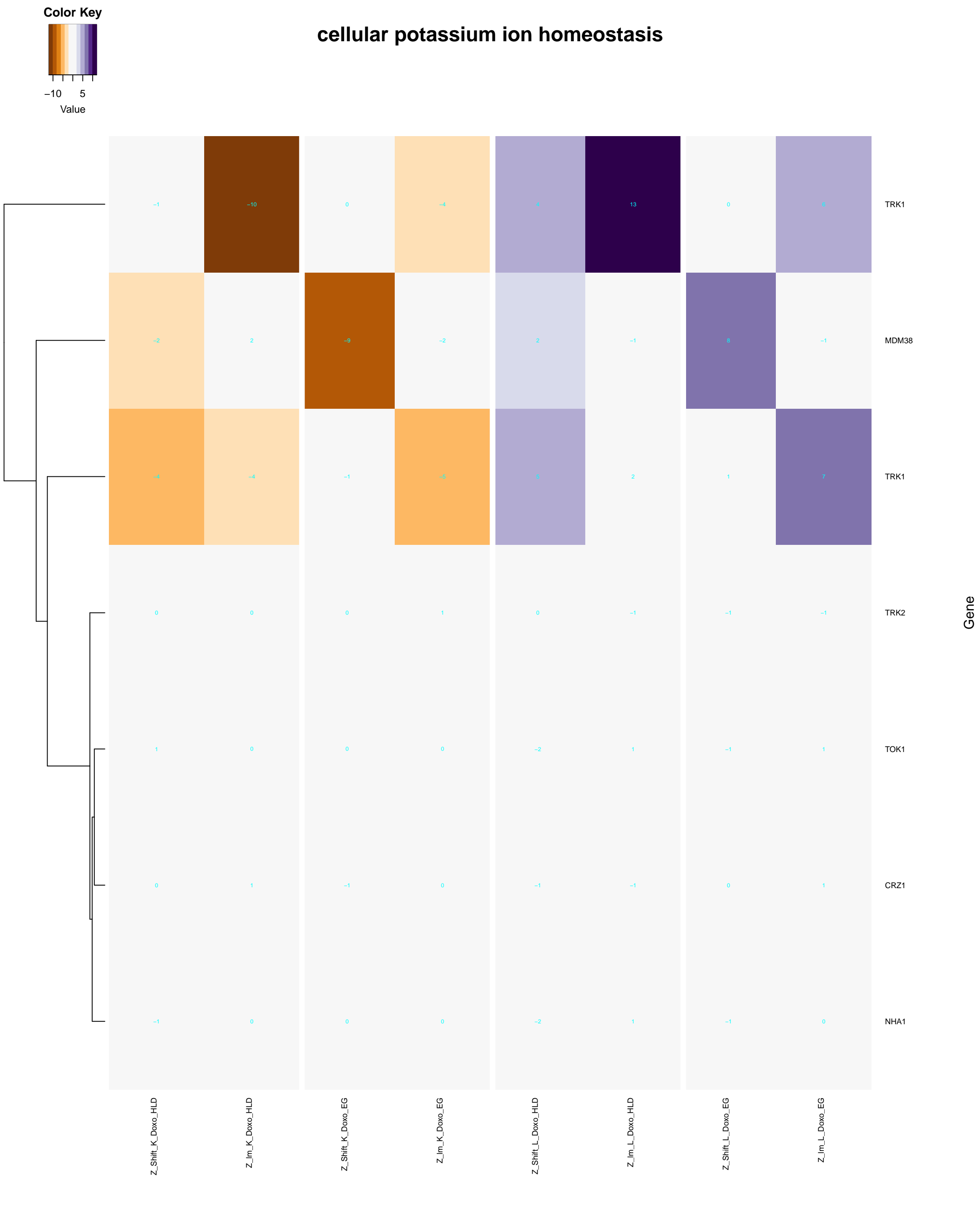

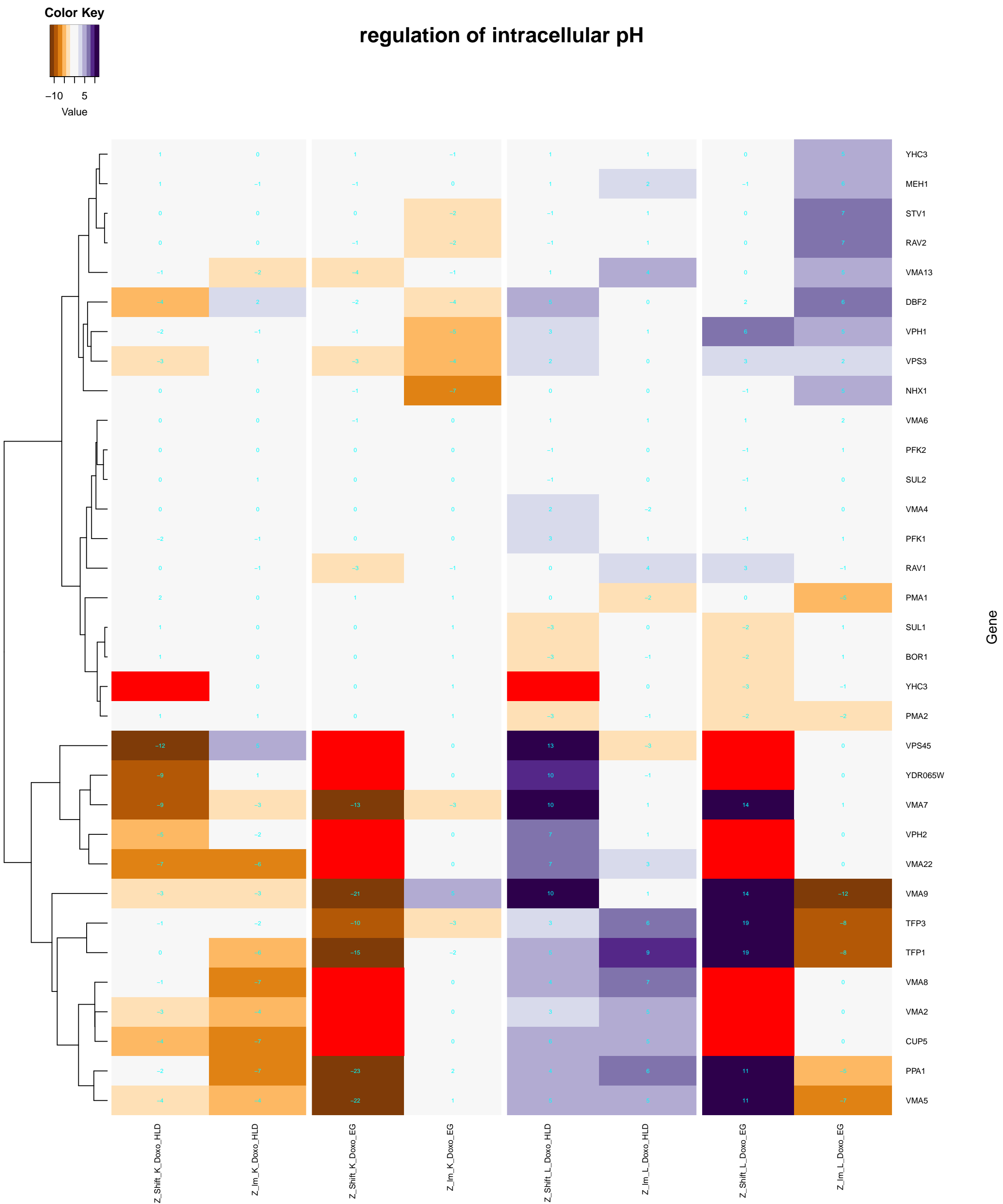

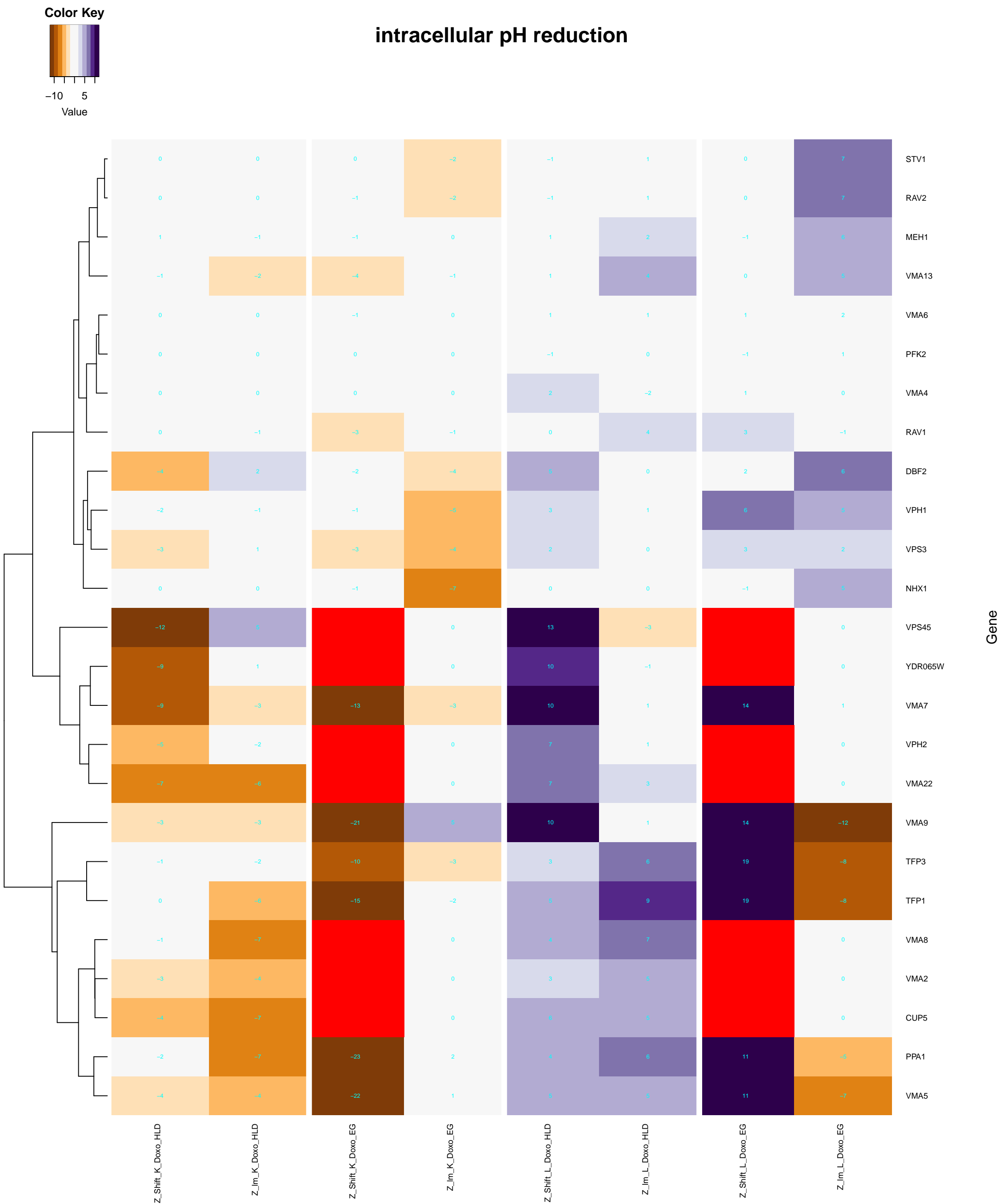

Supplement: Supplementary file 9 — Additional file 9. GO term-specific heatmaps for REMc/GTF-enriched clusters. GO term-specific heatmaps for significant GO process terms were generated as described in methods and Figs. 3 and 4. Any related child terms are presented in subsequent pages of the parent file name. GO terms with more than 100 children, with 2 or fewer genes annotated to the term, or a file size over 300KB are not shown. All heatmaps are generated with the same layout (see Figs. 3 and 4). [file 40170_2019_201_MOESM9_ESM.bz2 › Additional_File9_GOTermHeatmaps/Additional_File9_GOTermHeatmap/monovalent_inorganic_cation_homeostasis.pdf]

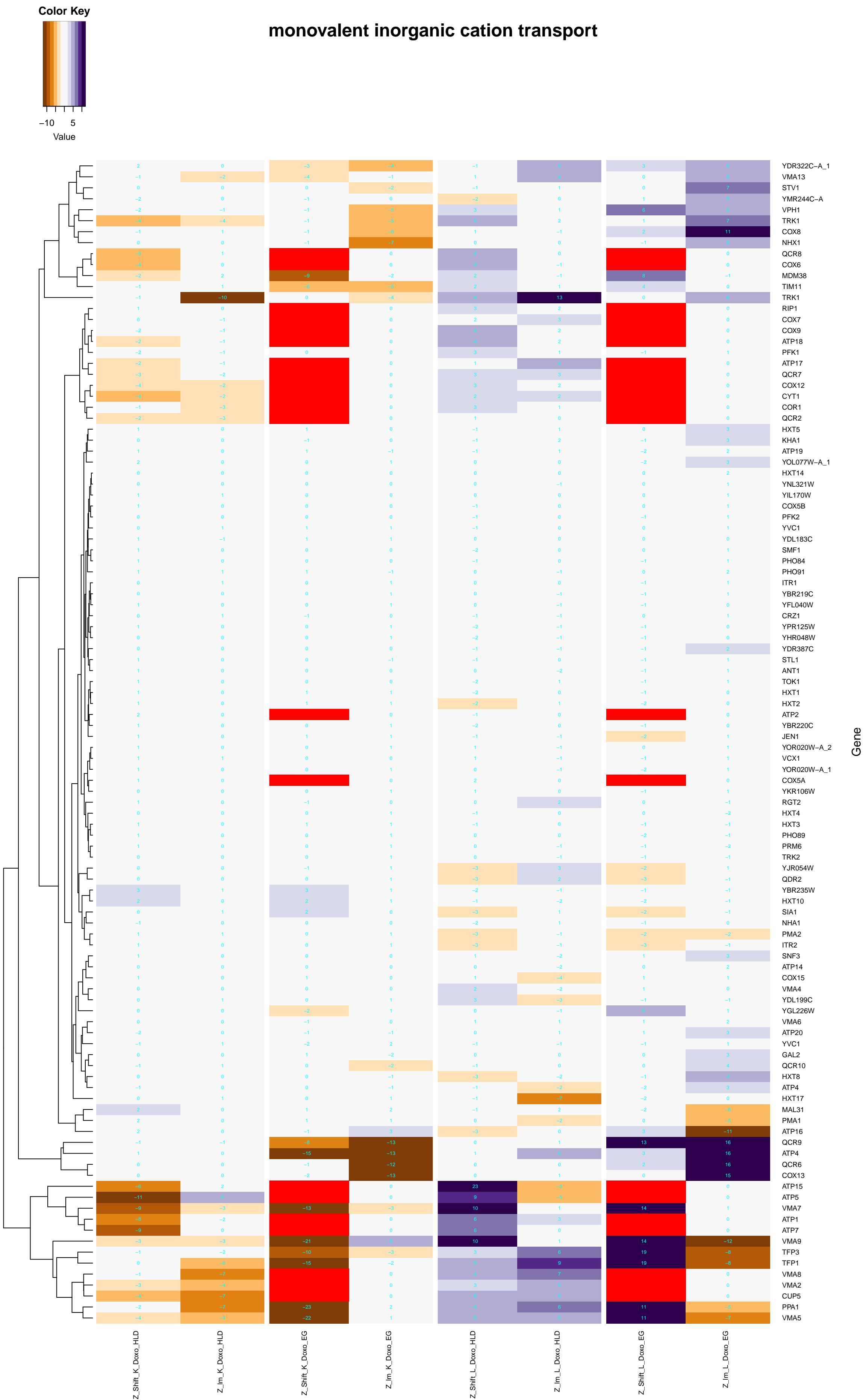

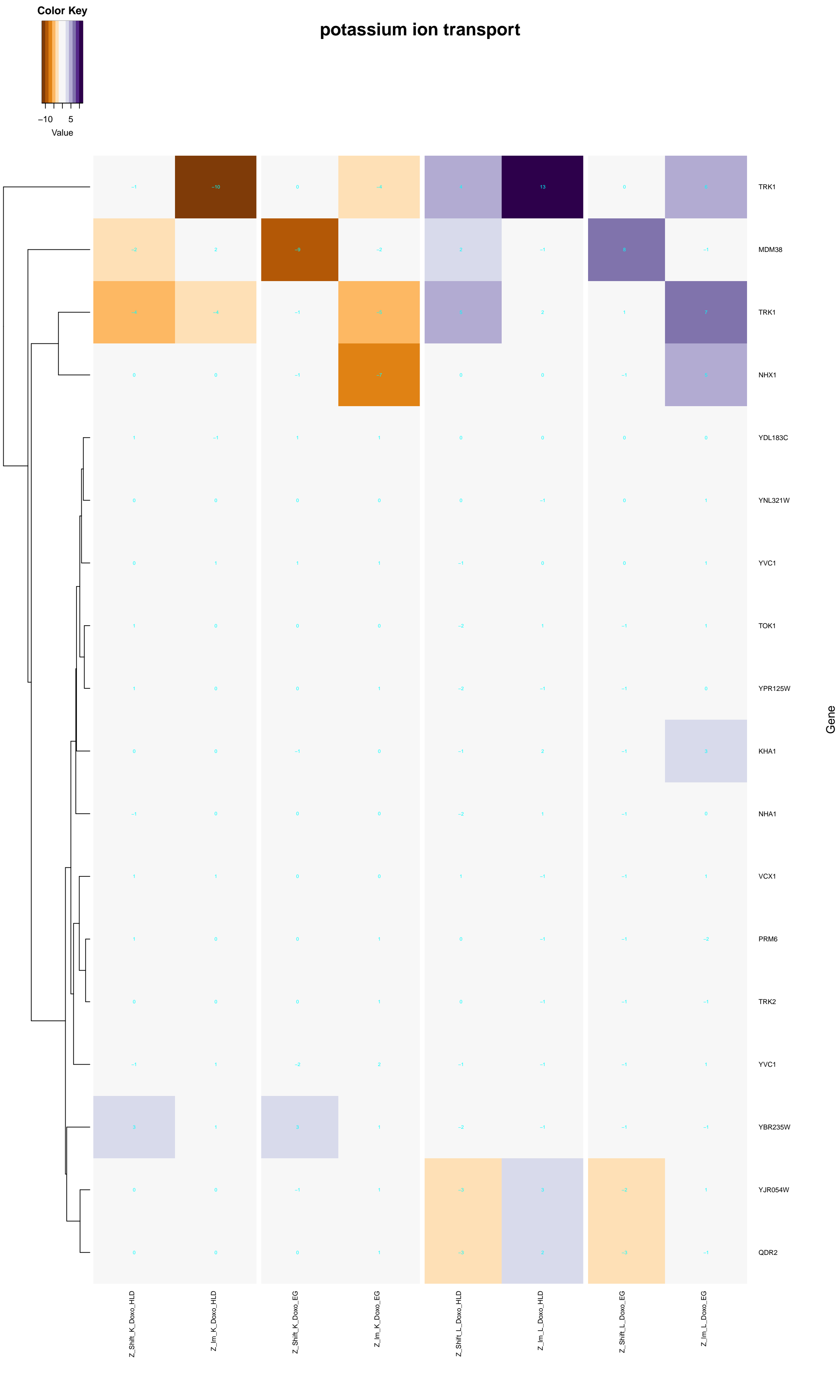

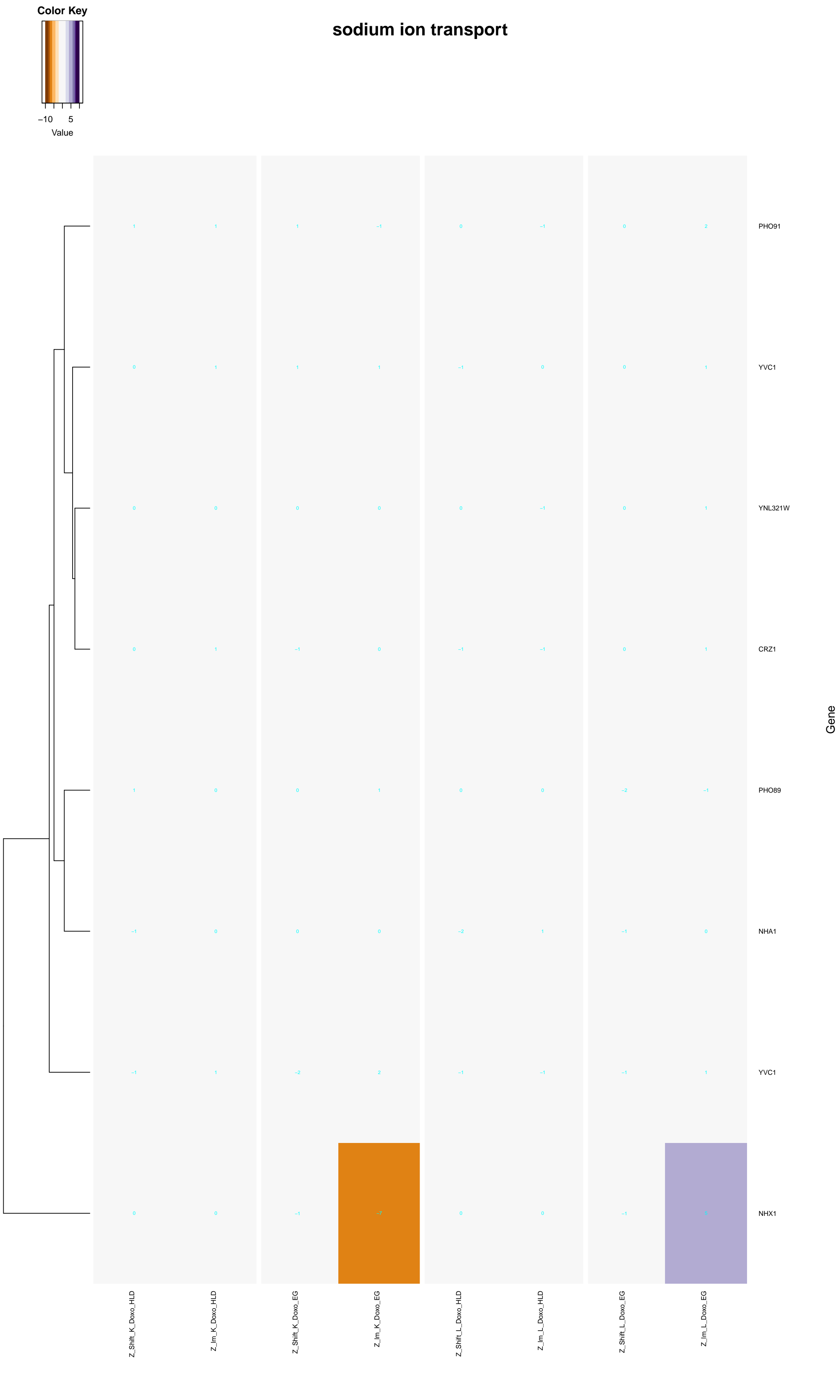

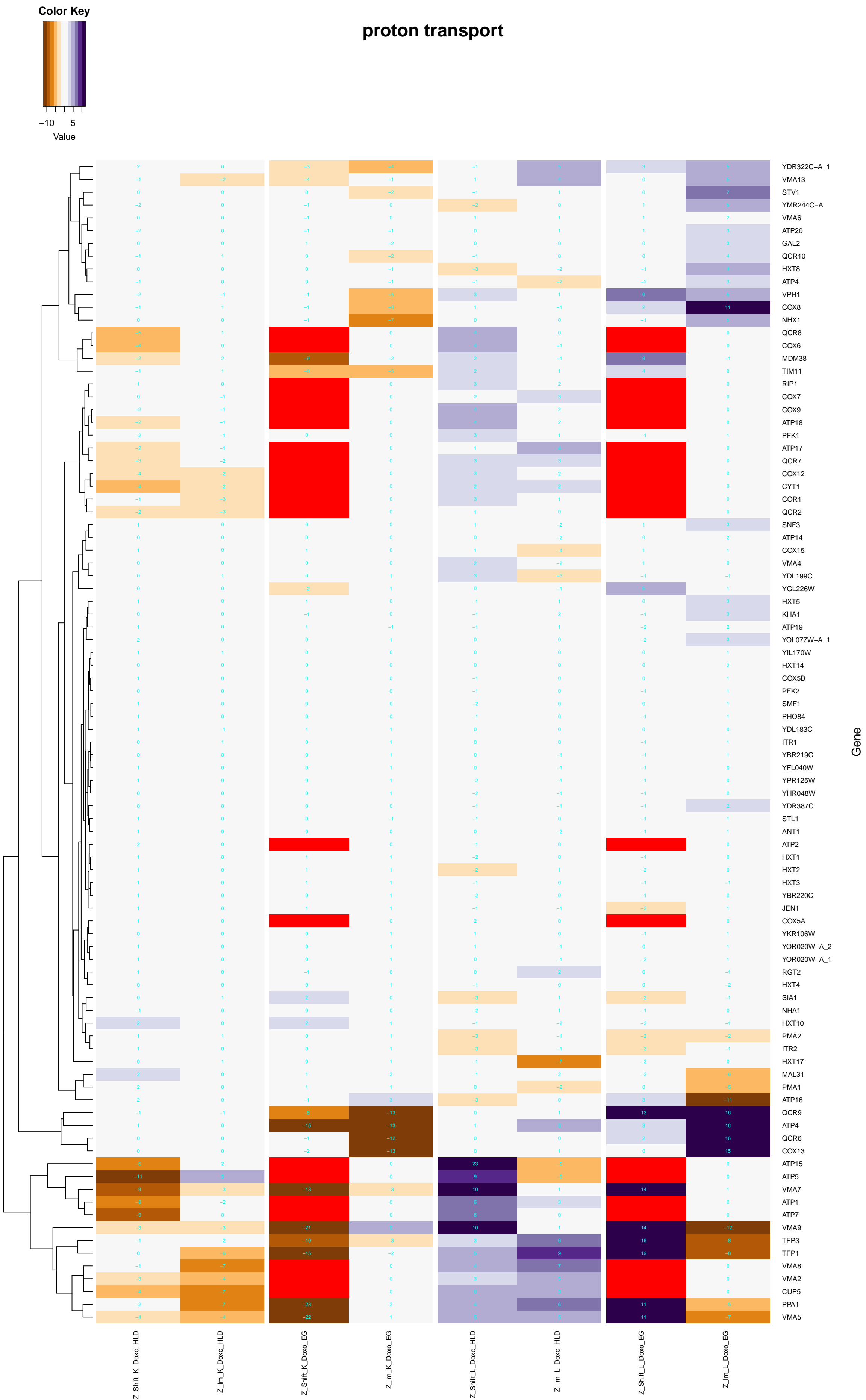

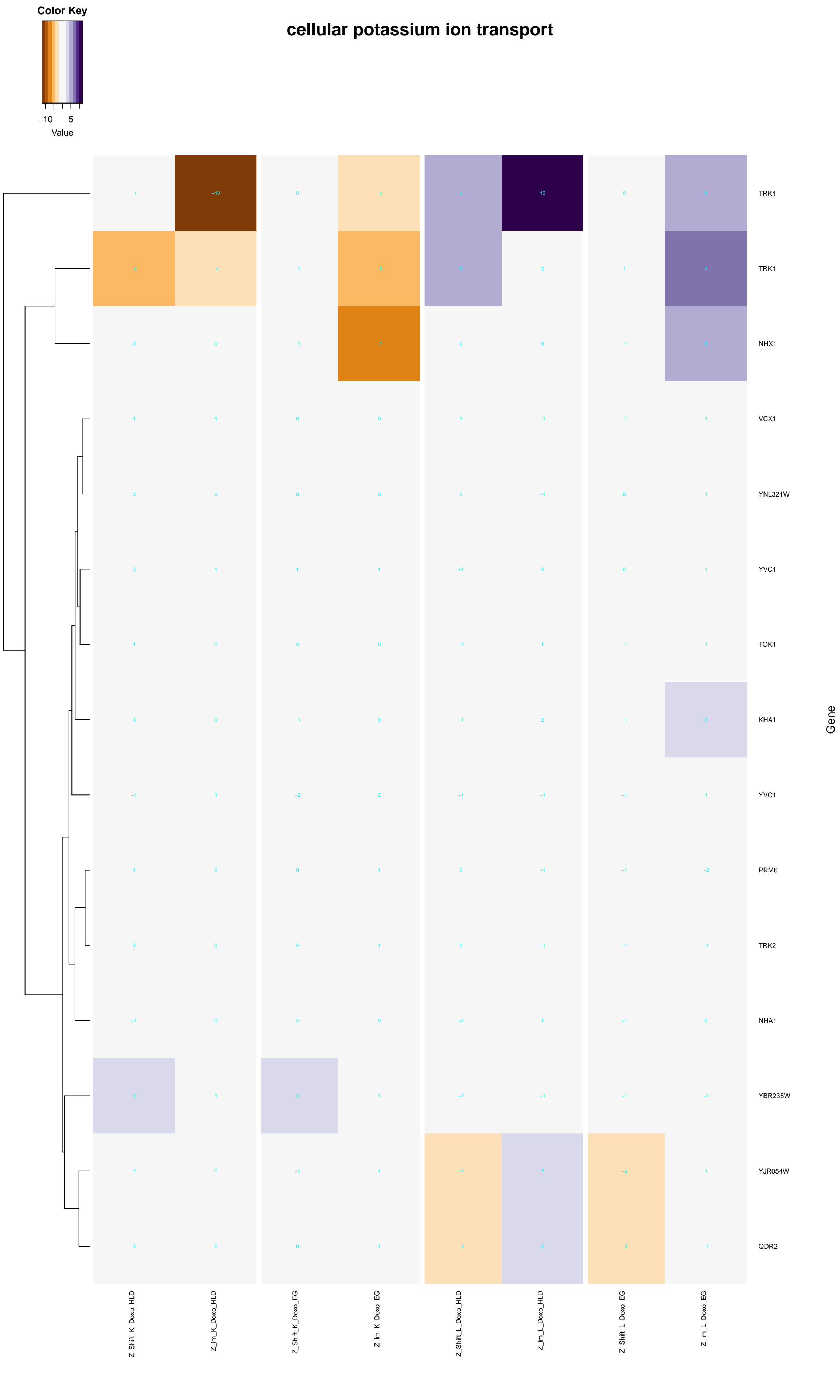

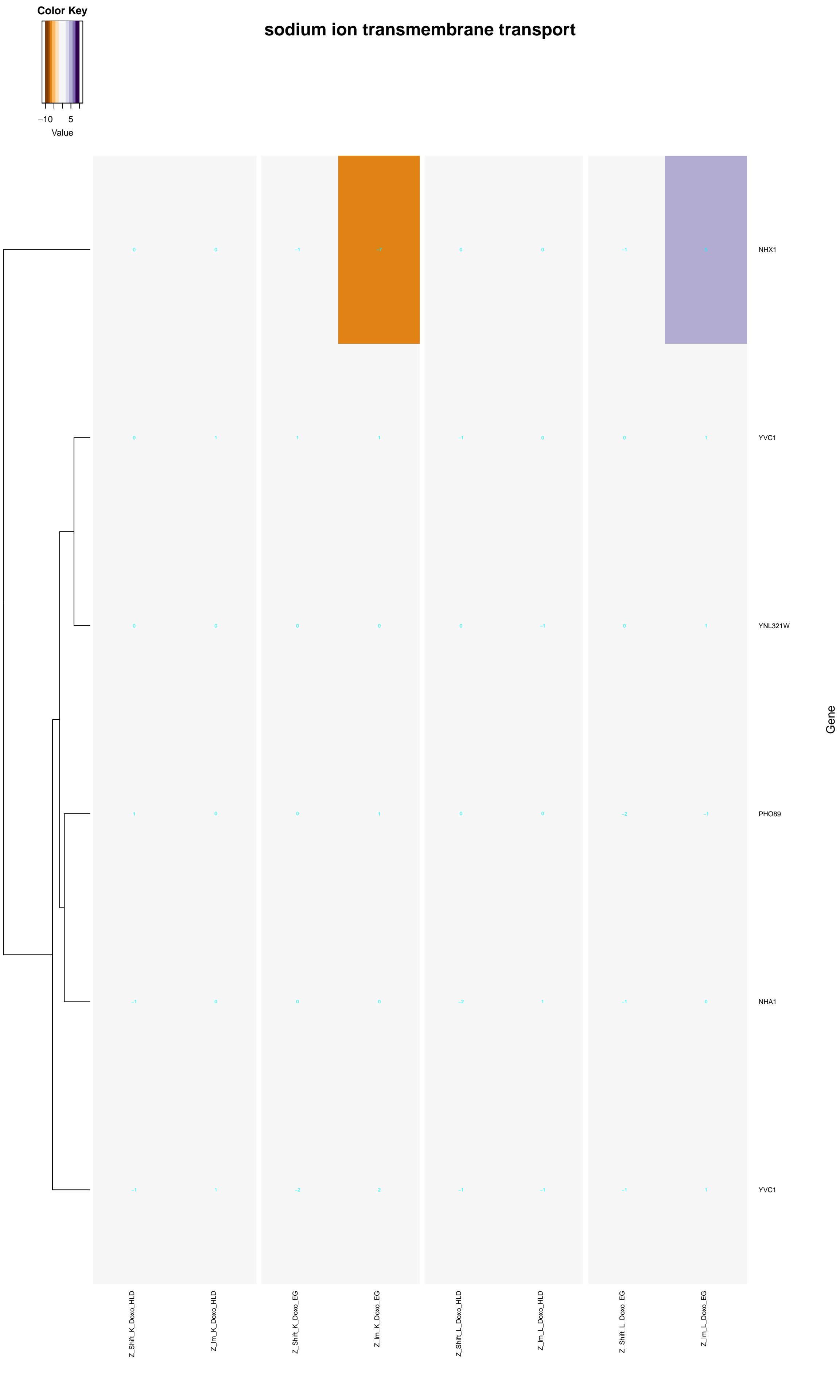

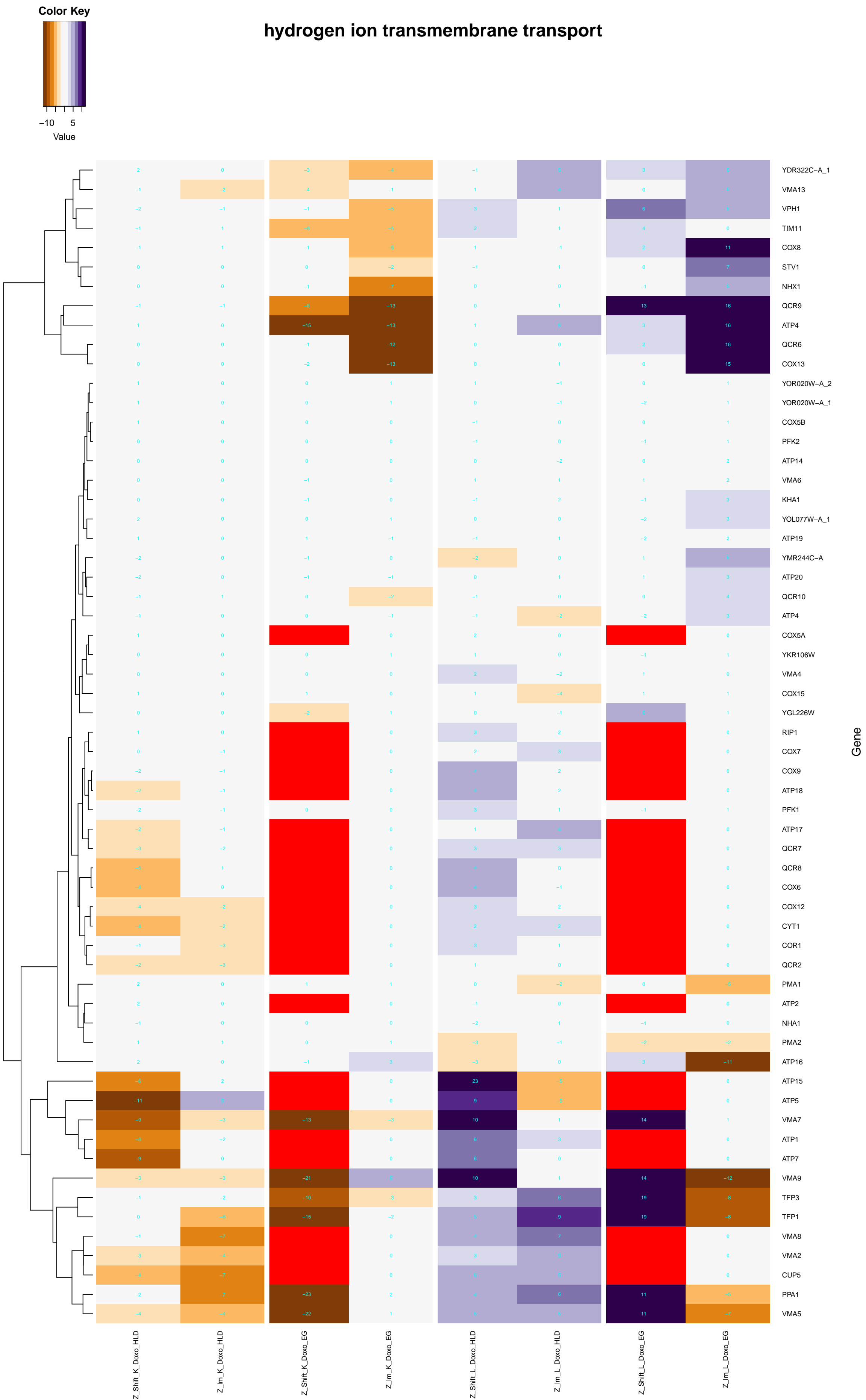

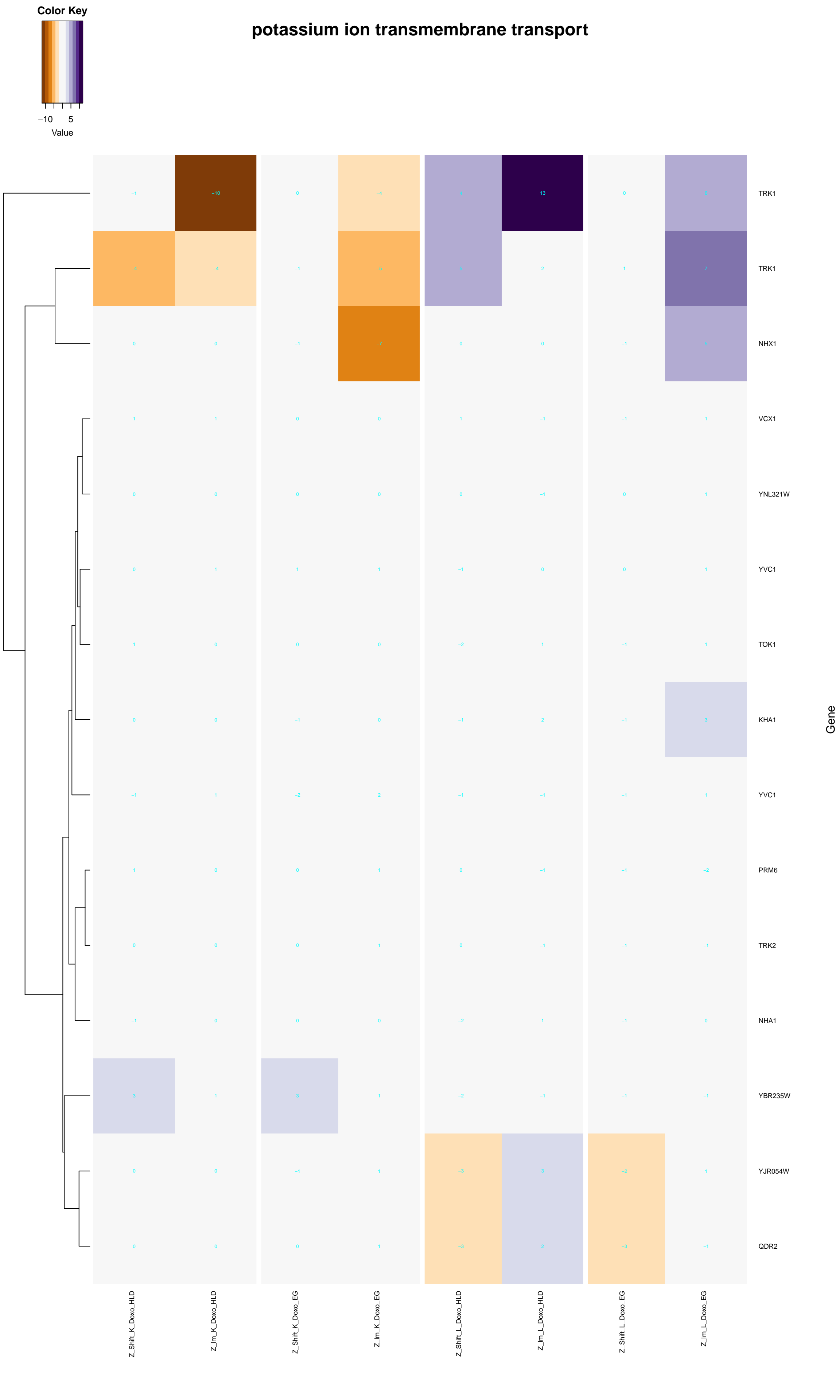

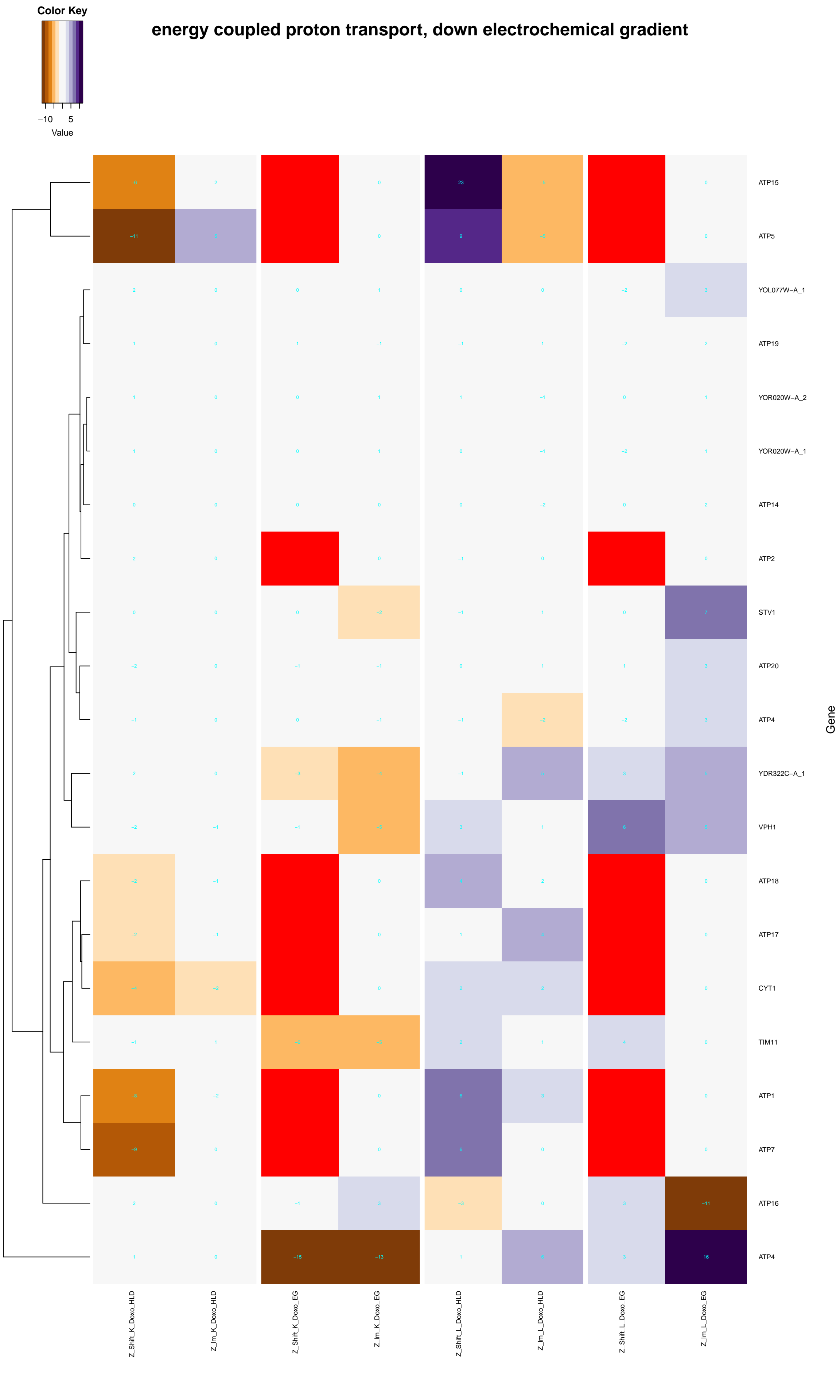

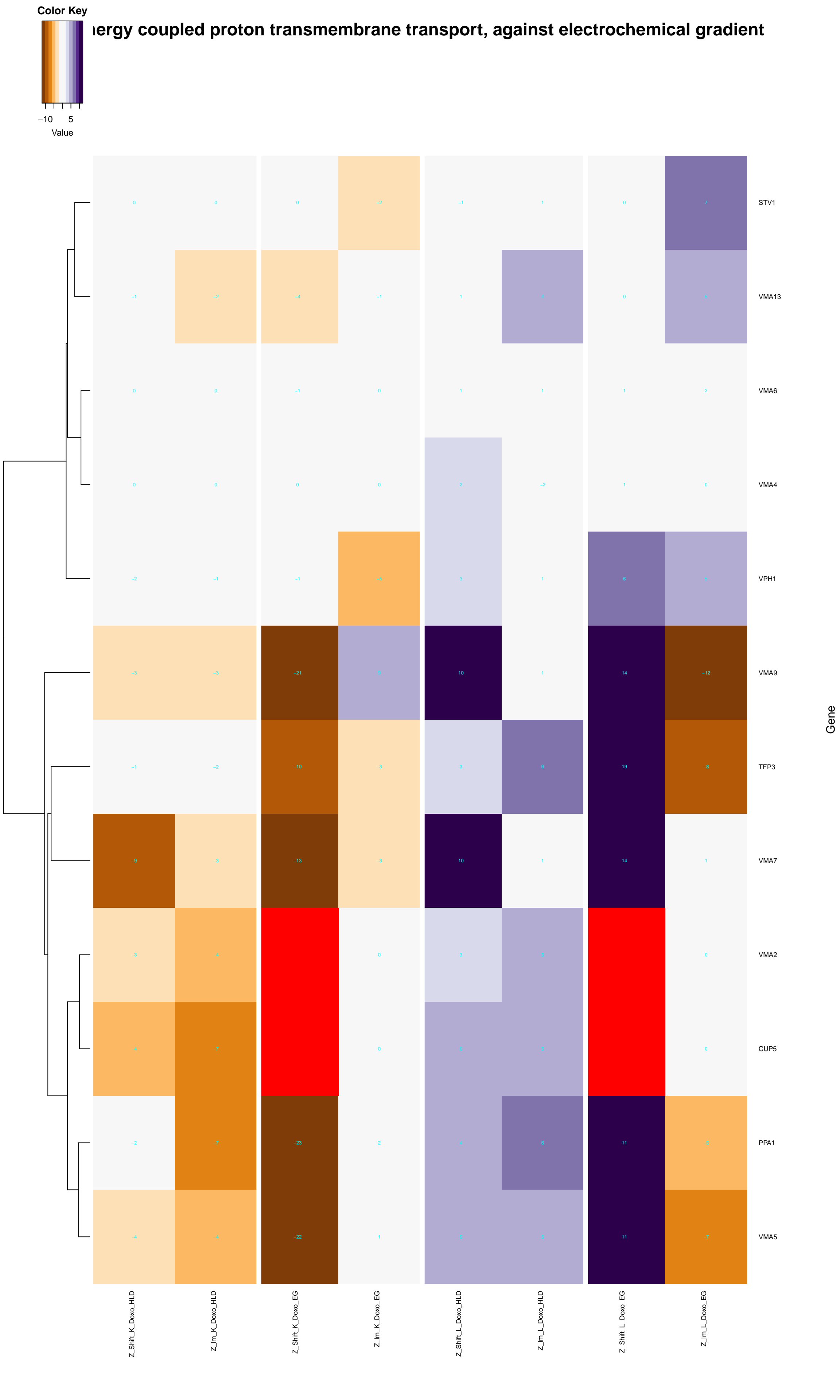

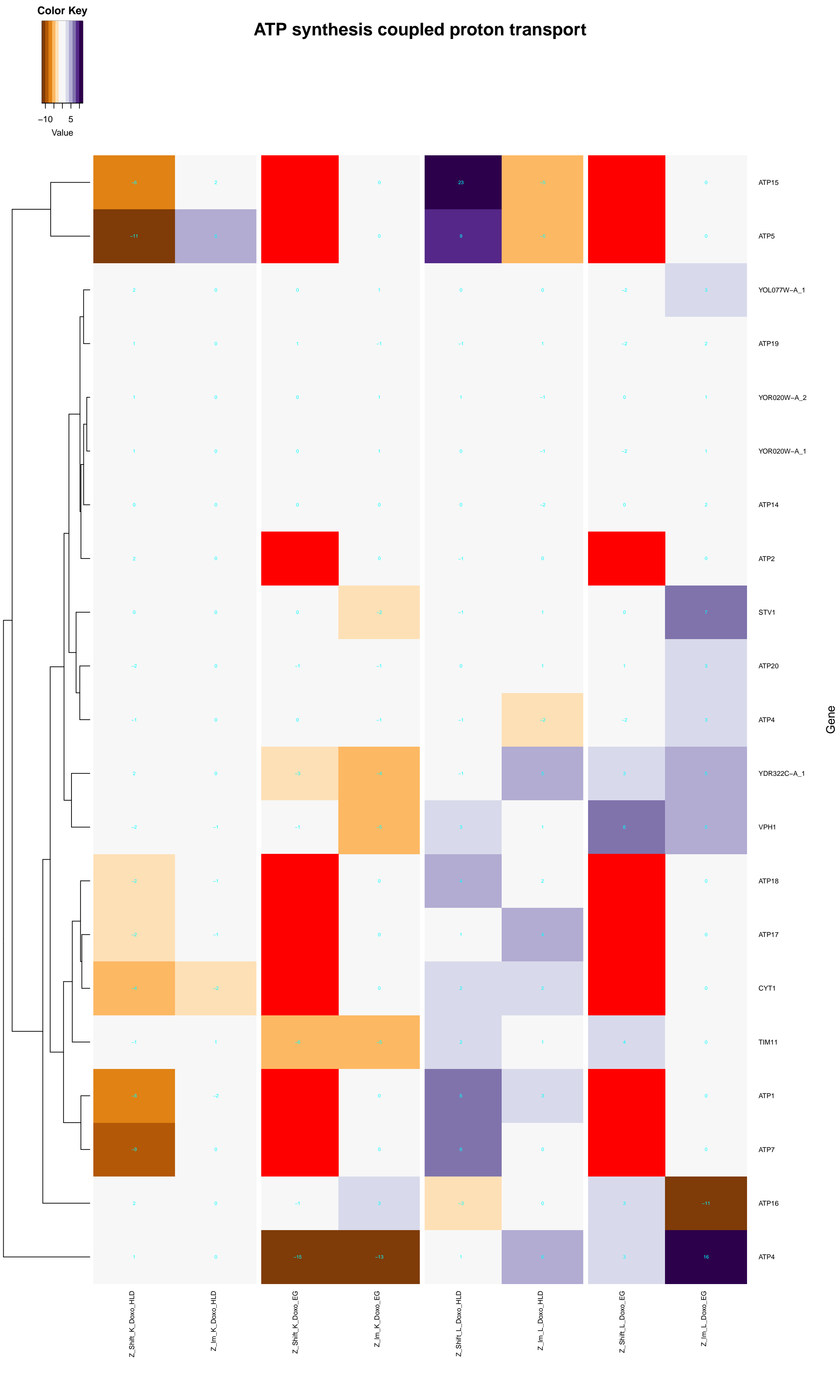

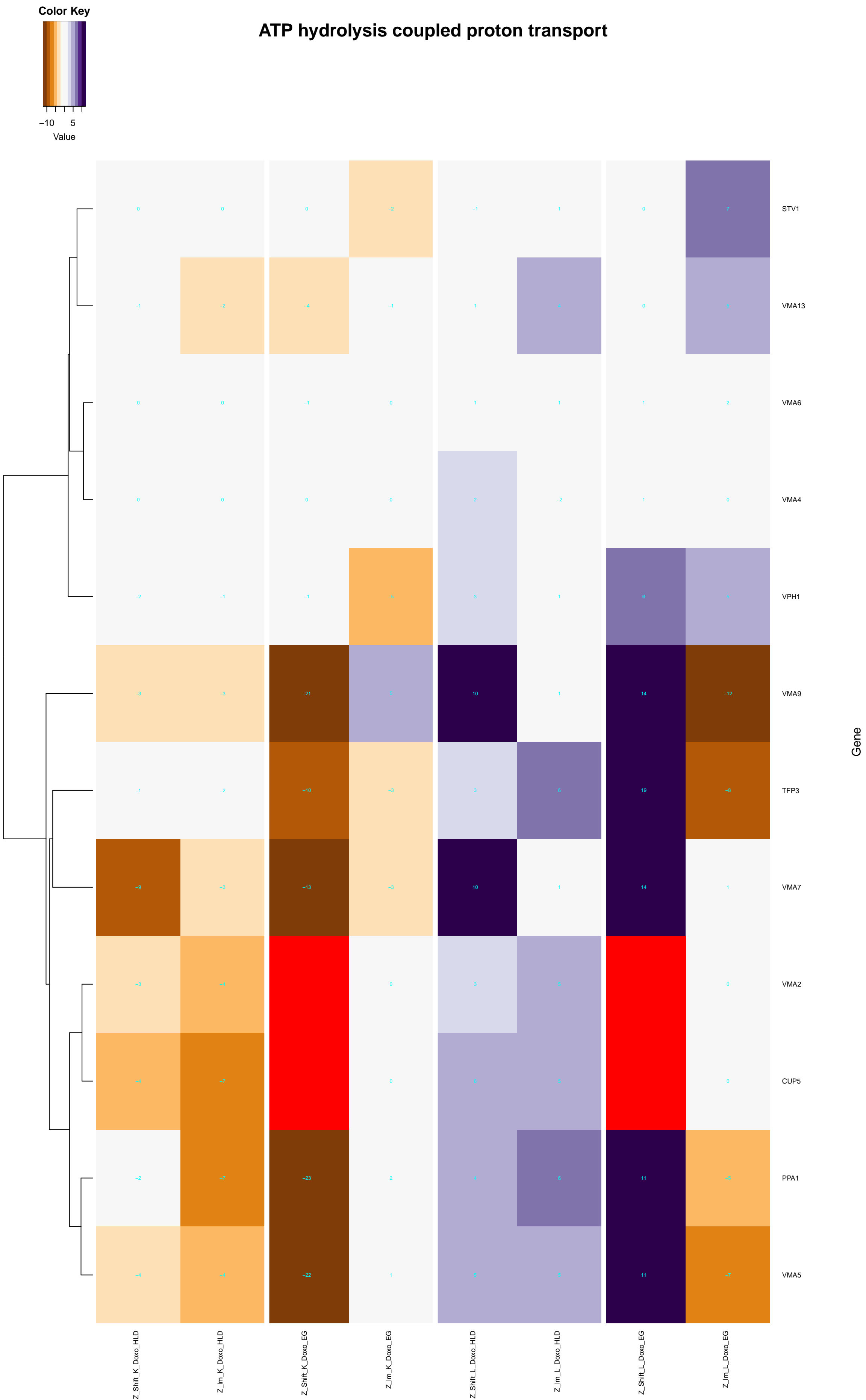

Supplement: Supplementary file 9 — Additional file 9. GO term-specific heatmaps for REMc/GTF-enriched clusters. GO term-specific heatmaps for significant GO process terms were generated as described in methods and Figs. 3 and 4. Any related child terms are presented in subsequent pages of the parent file name. GO terms with more than 100 children, with 2 or fewer genes annotated to the term, or a file size over 300KB are not shown. All heatmaps are generated with the same layout (see Figs. 3 and 4). [file 40170_2019_201_MOESM9_ESM.bz2 › Additional_File9_GOTermHeatmaps/Additional_File9_GOTermHeatmap/monovalent_inorganic_cation_transport.pdf]

## mRNA-containing ribonucleoprotein complex export from nucleus

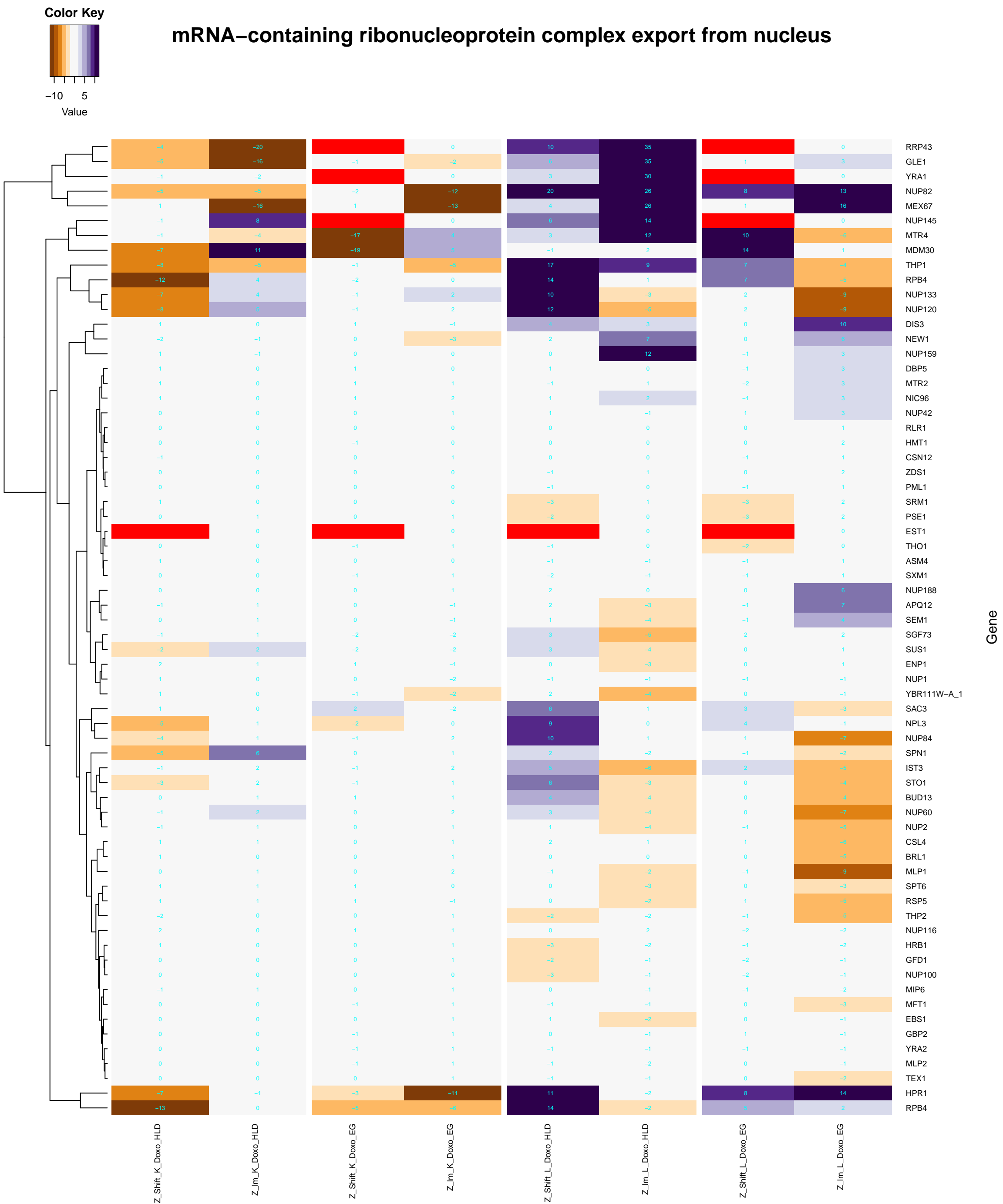

Supplement: Supplementary file 9 — Additional file 9. GO term-specific heatmaps for REMc/GTF-enriched clusters. GO term-specific heatmaps for significant GO process terms were generated as described in methods and Figs. 3 and 4. Any related child terms are presented in subsequent pages of the parent file name. GO terms with more than 100 children, with 2 or fewer genes annotated to the term, or a file size over 300KB are not shown. All heatmaps are generated with the same layout (see Figs. 3 and 4). [file 40170_2019_201_MOESM9_ESM.bz2 › Additional_File9_GOTermHeatmaps/Additional_File9_GOTermHeatmap/mRNA-containing_ribonucleoprotein_complex_export_from_nucleus.pdf]

## mRNA 3'-end processing

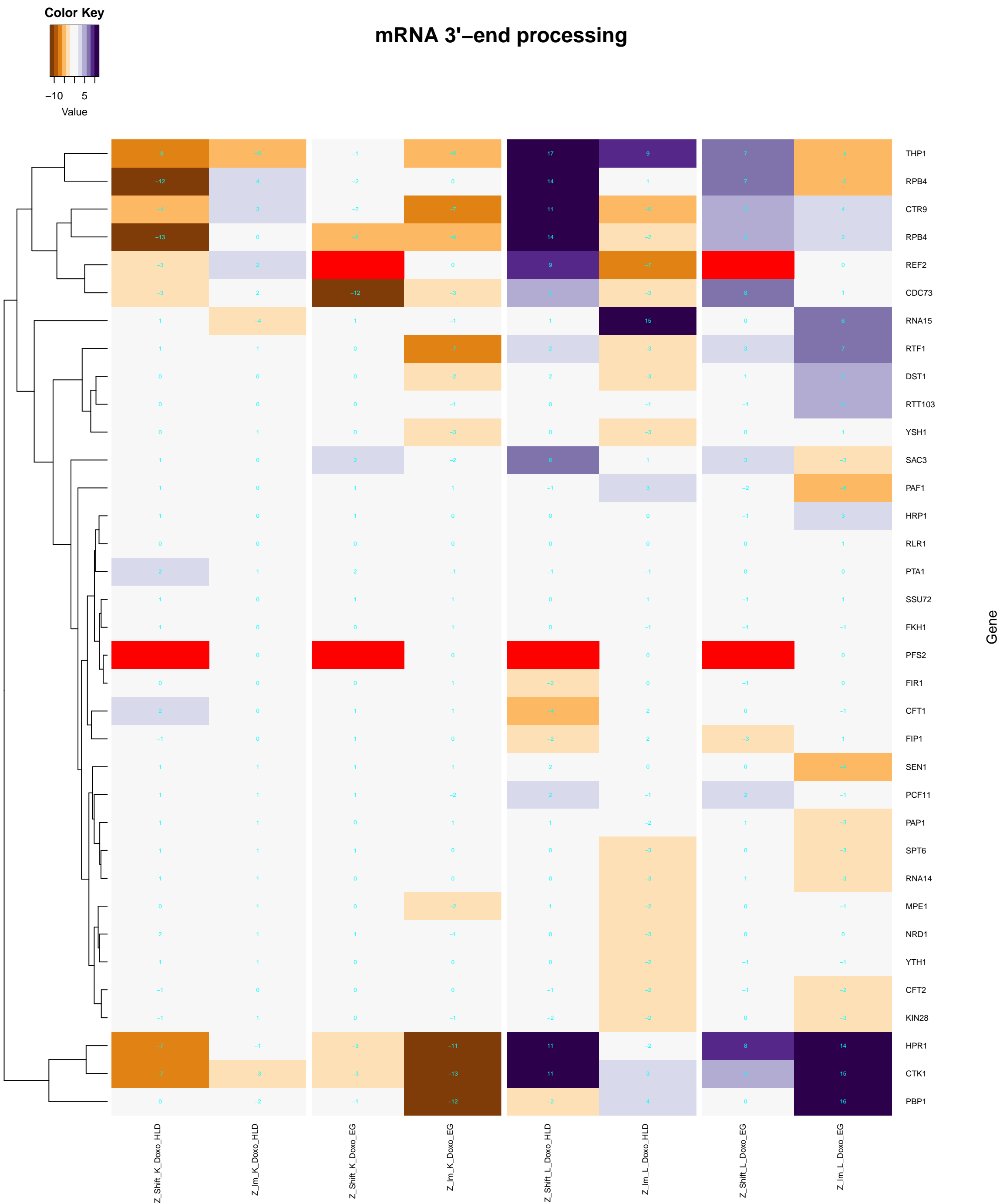

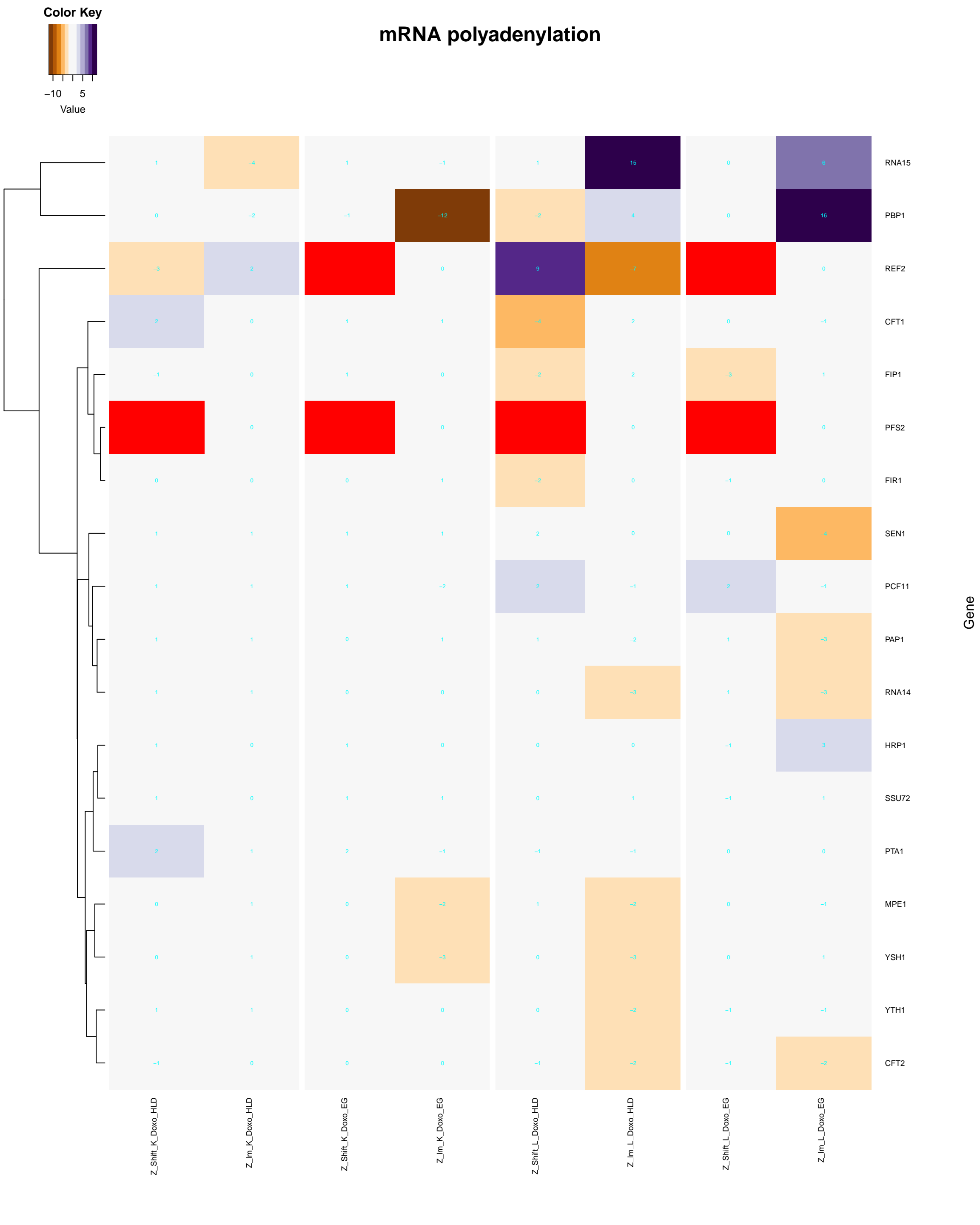

Supplement: Supplementary file 9 — Additional file 9. GO term-specific heatmaps for REMc/GTF-enriched clusters. GO term-specific heatmaps for significant GO process terms were generated as described in methods and Figs. 3 and 4. Any related child terms are presented in subsequent pages of the parent file name. GO terms with more than 100 children, with 2 or fewer genes annotated to the term, or a file size over 300KB are not shown. All heatmaps are generated with the same layout (see Figs. 3 and 4). [file 40170_2019_201_MOESM9_ESM.bz2 › Additional_File9_GOTermHeatmaps/Additional_File9_GOTermHeatmap/mRNA_3'-end_processing.pdf]

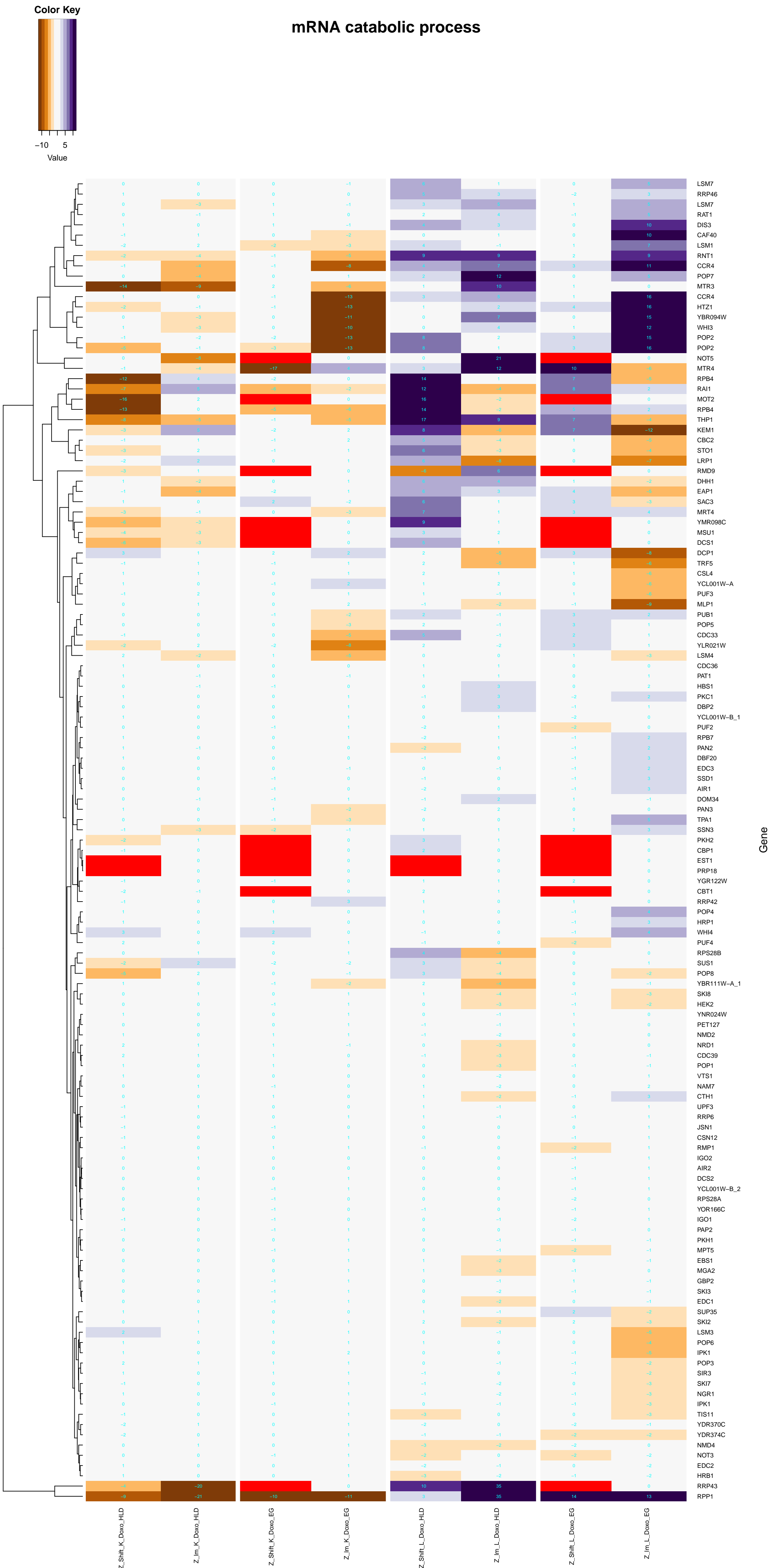



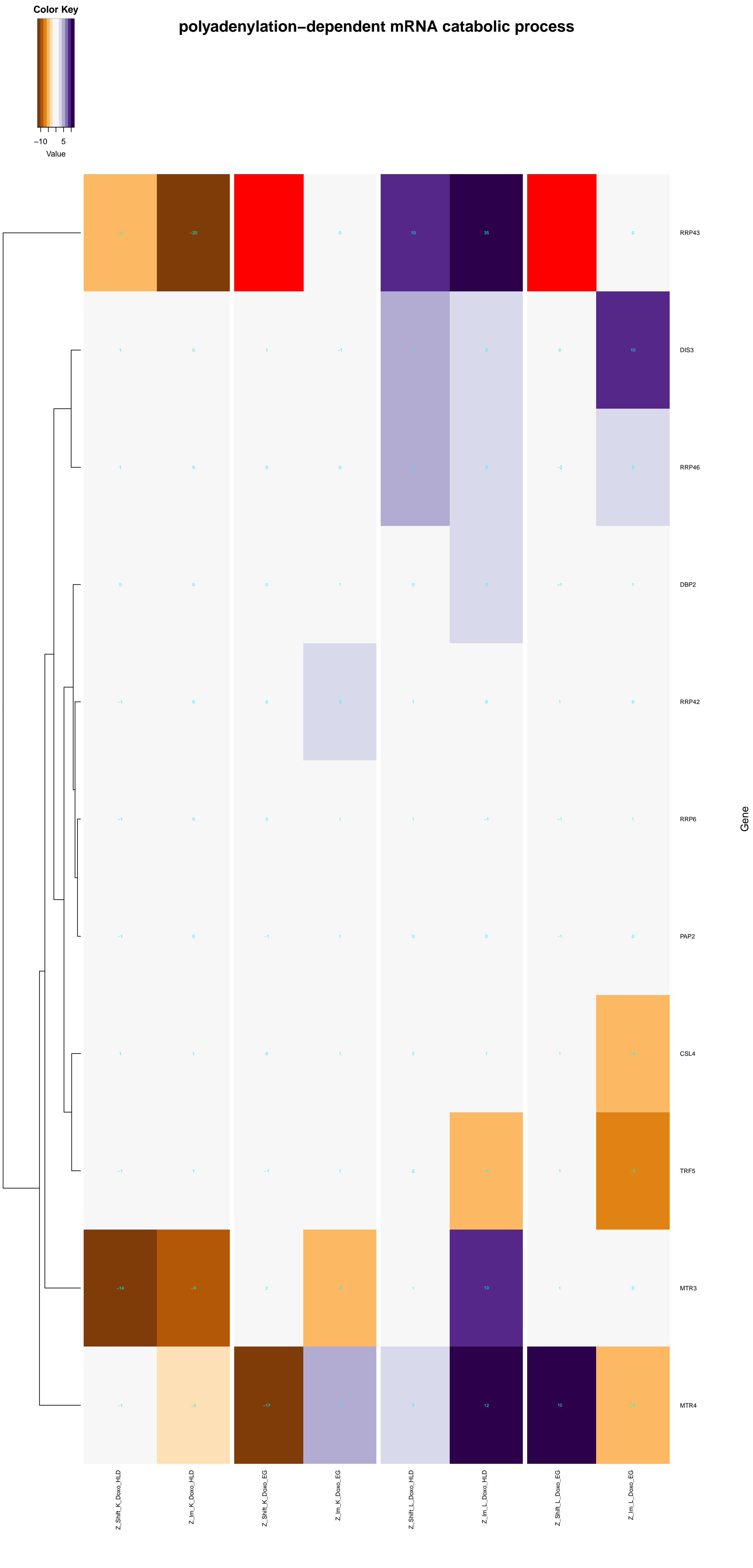

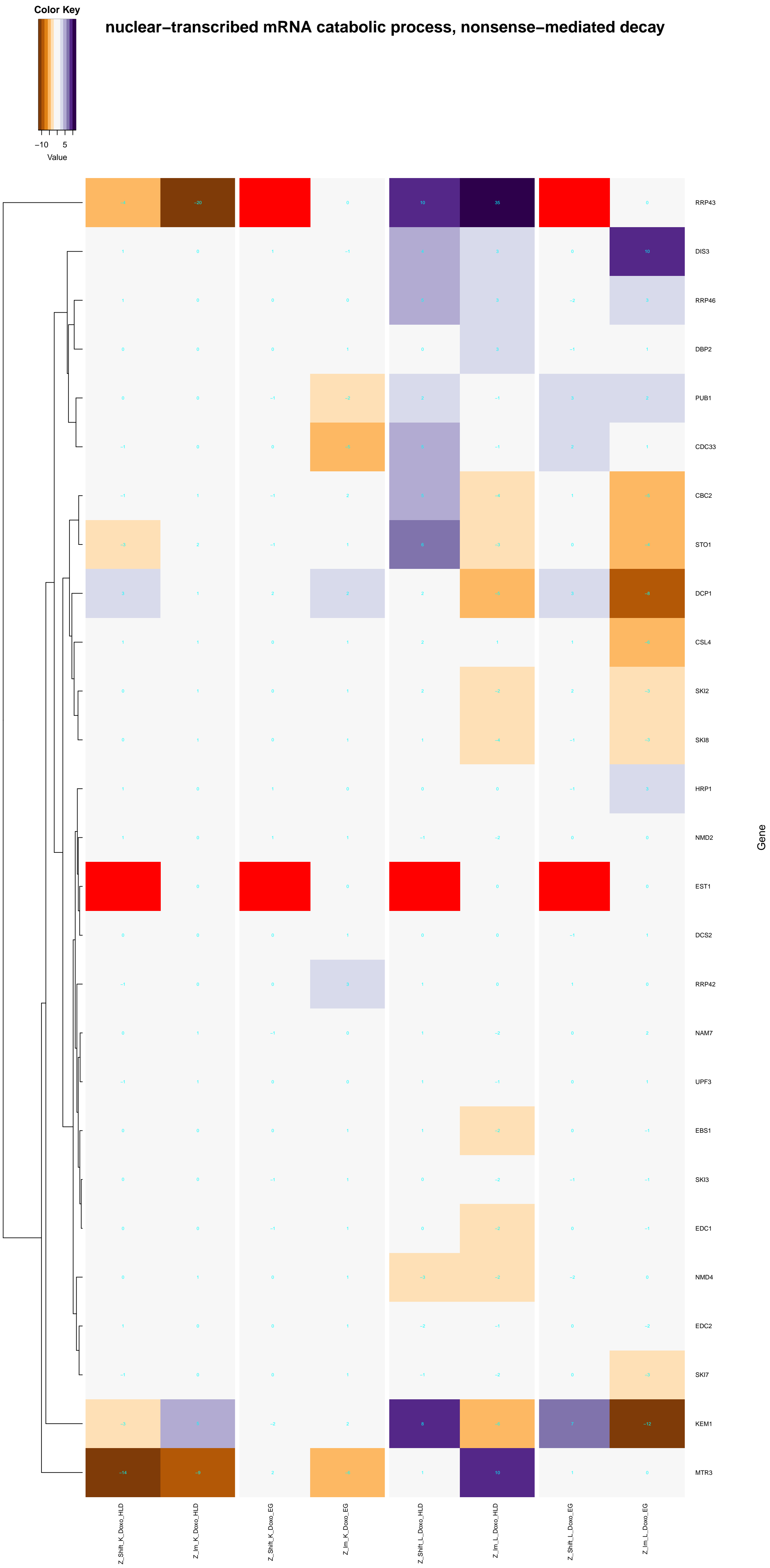

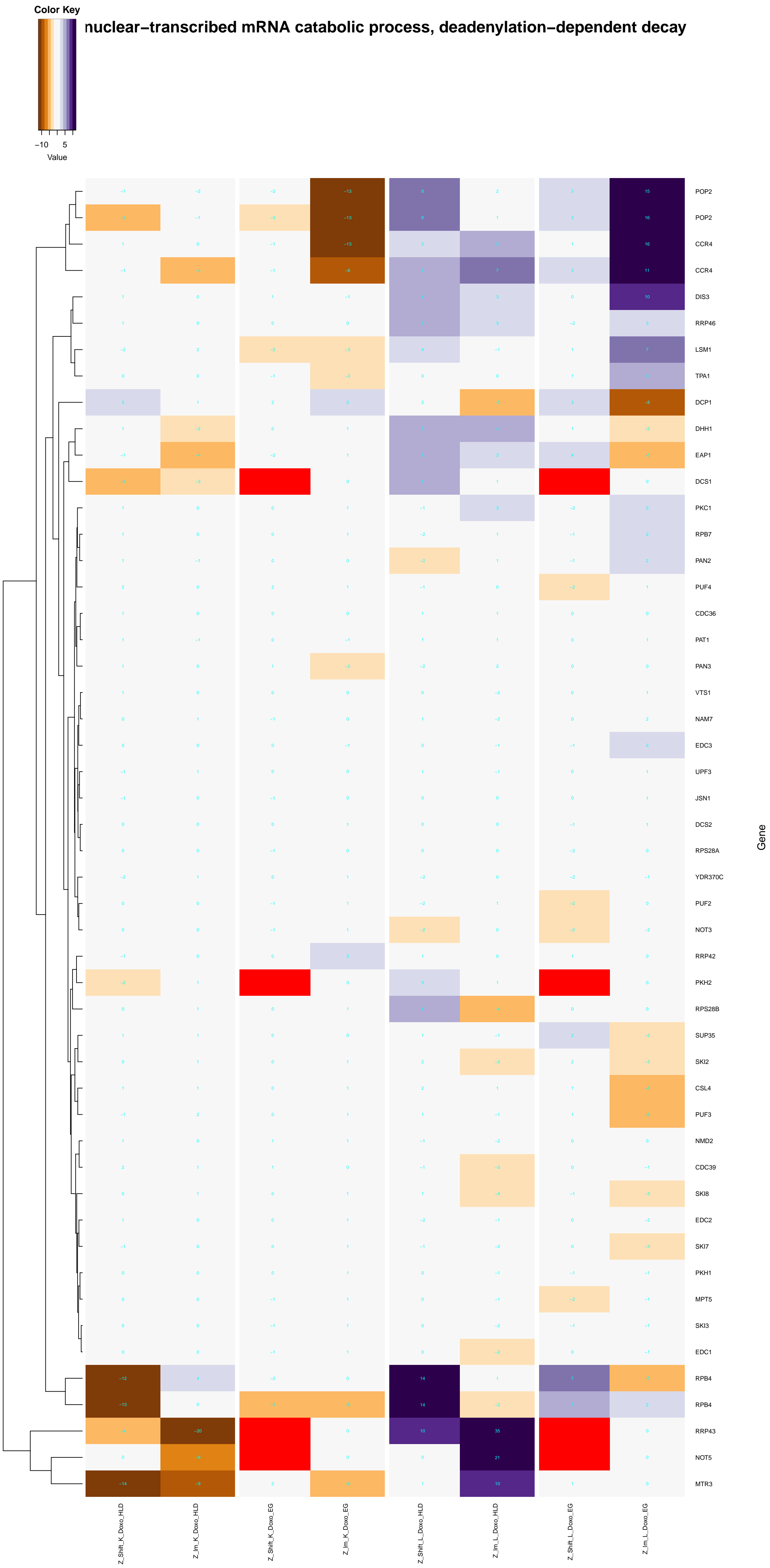

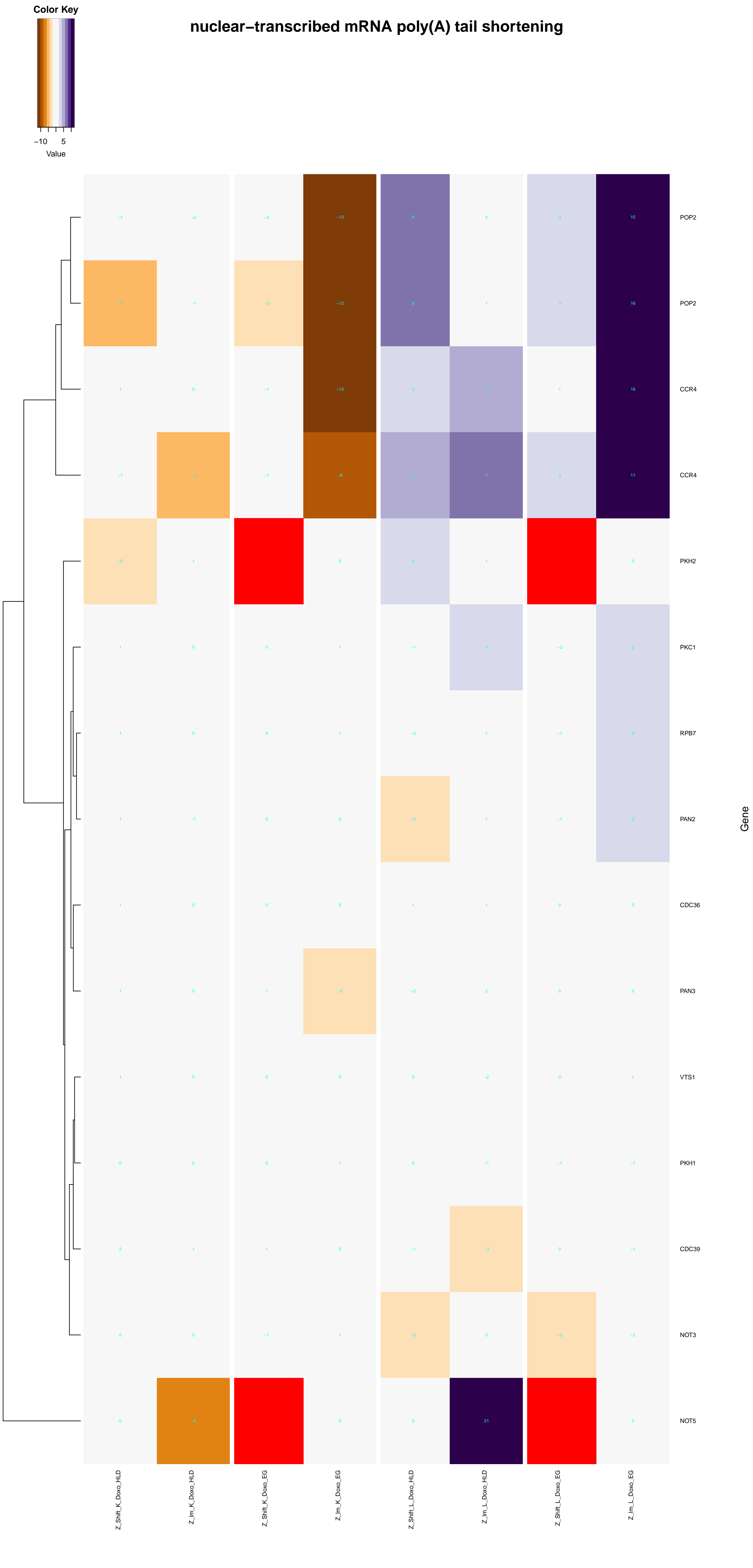

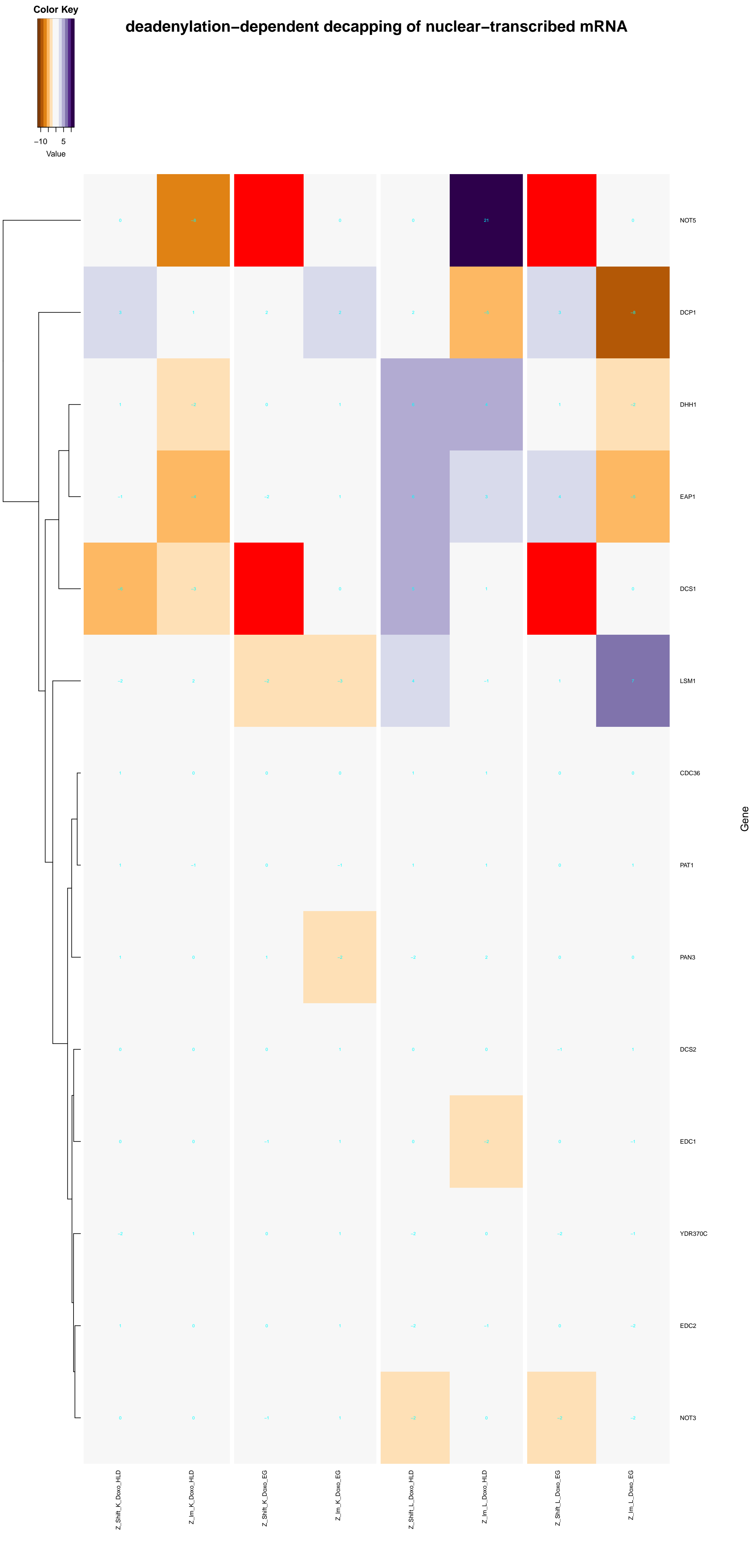

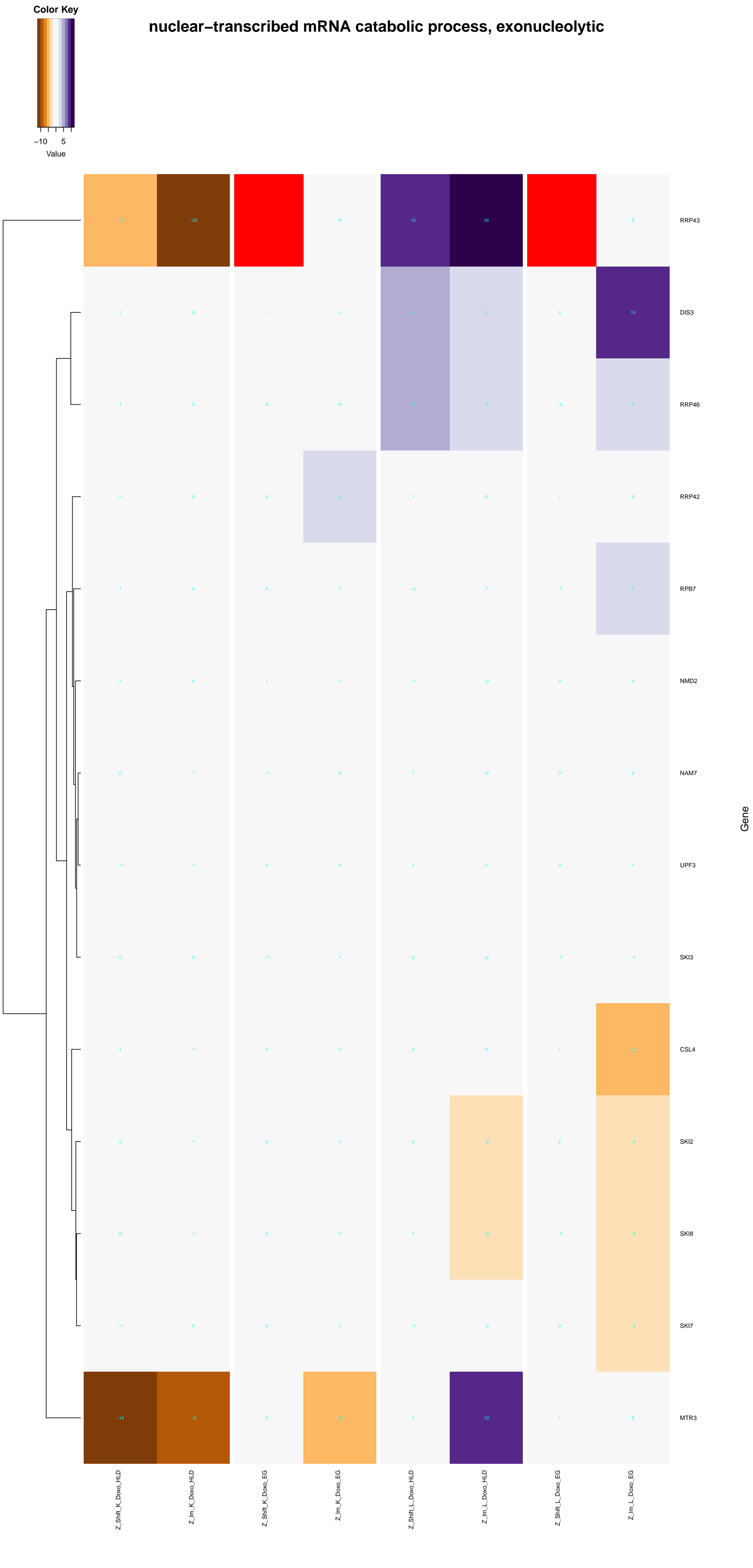

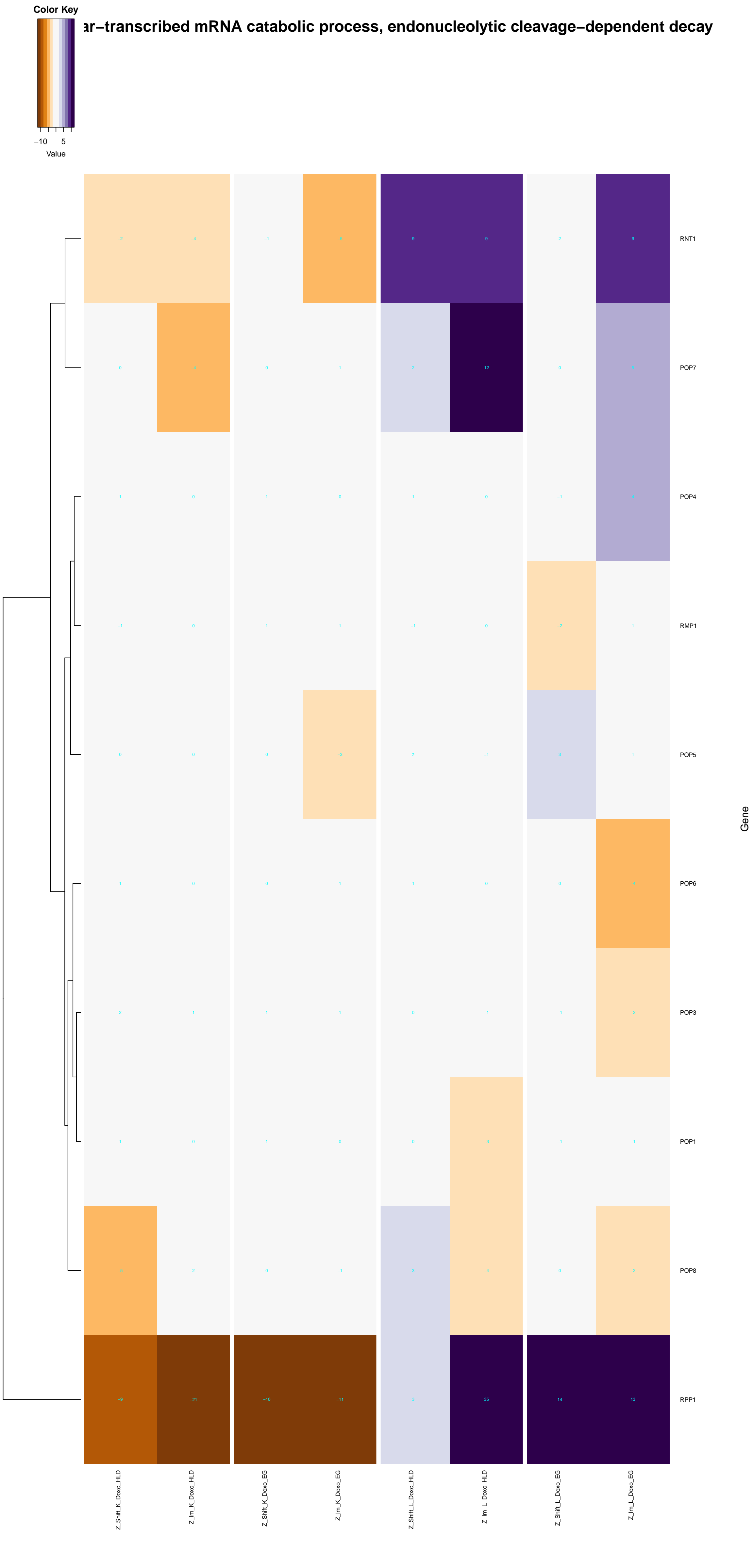

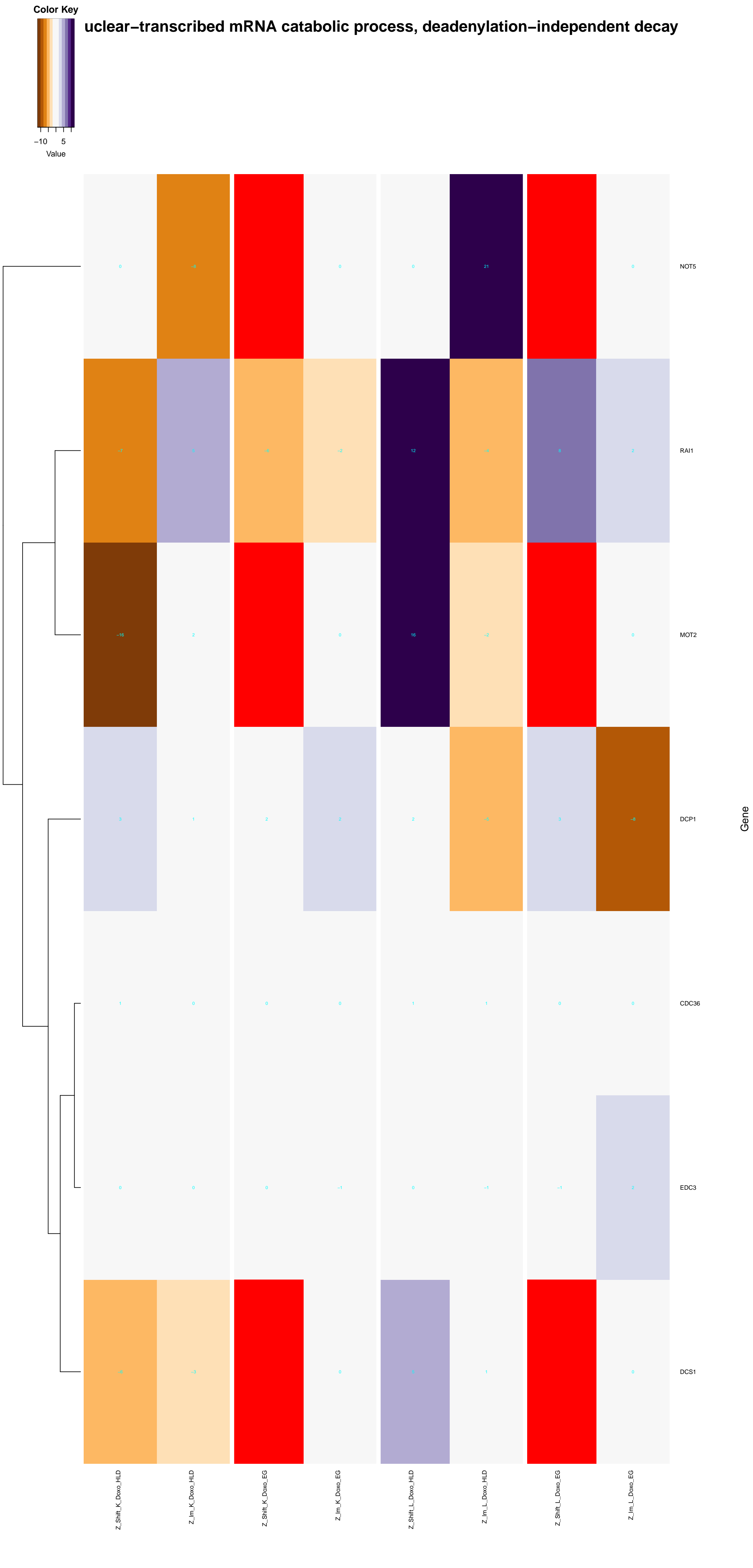

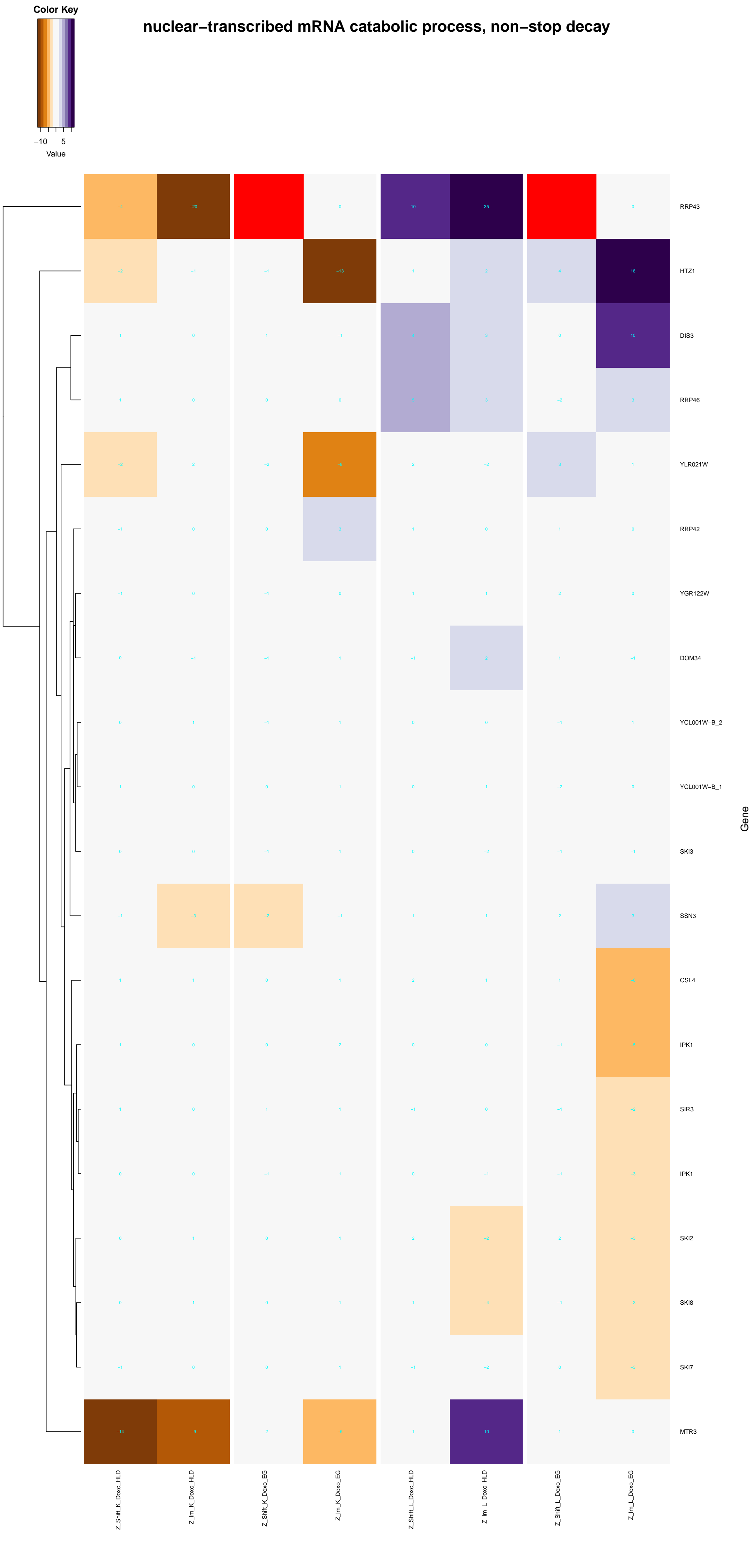

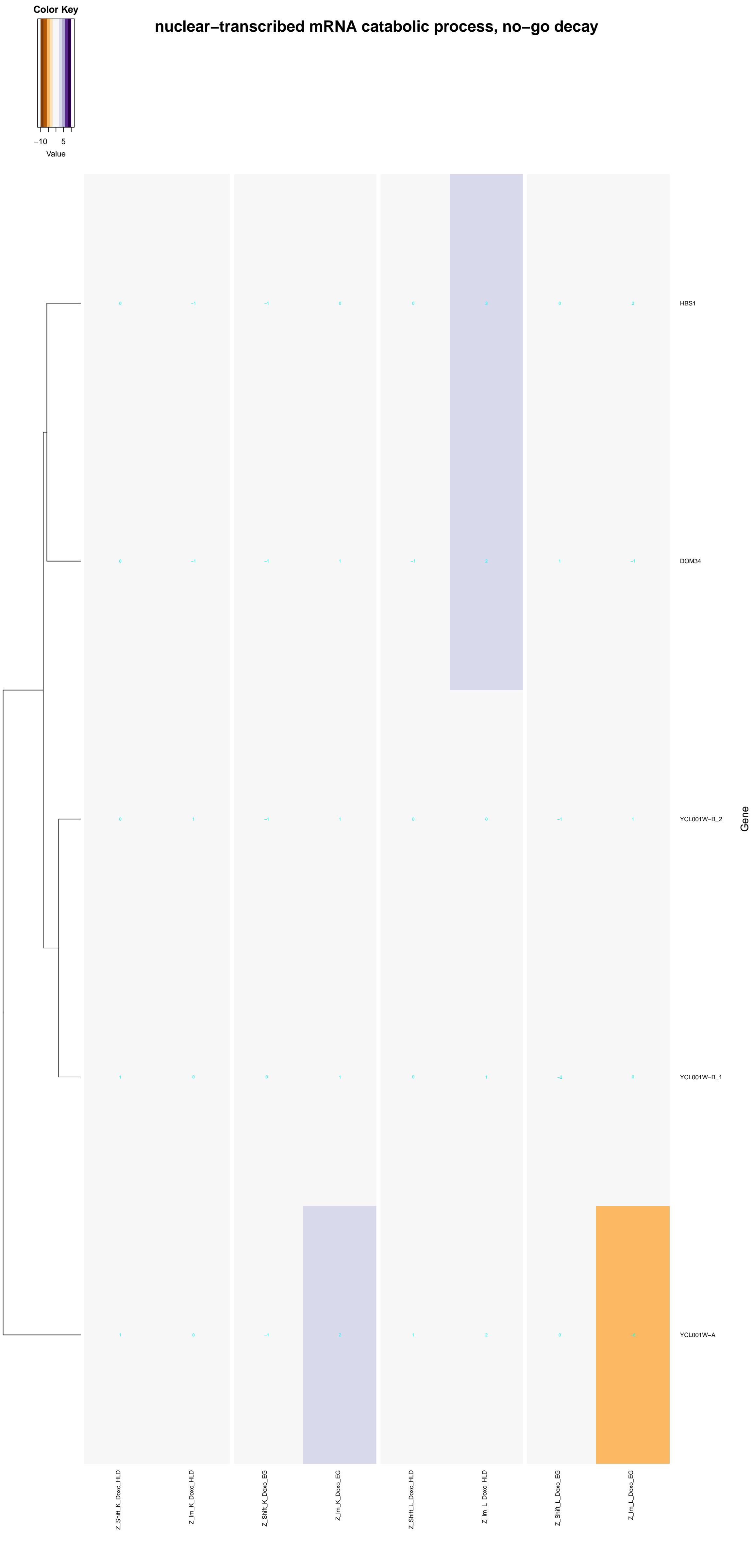

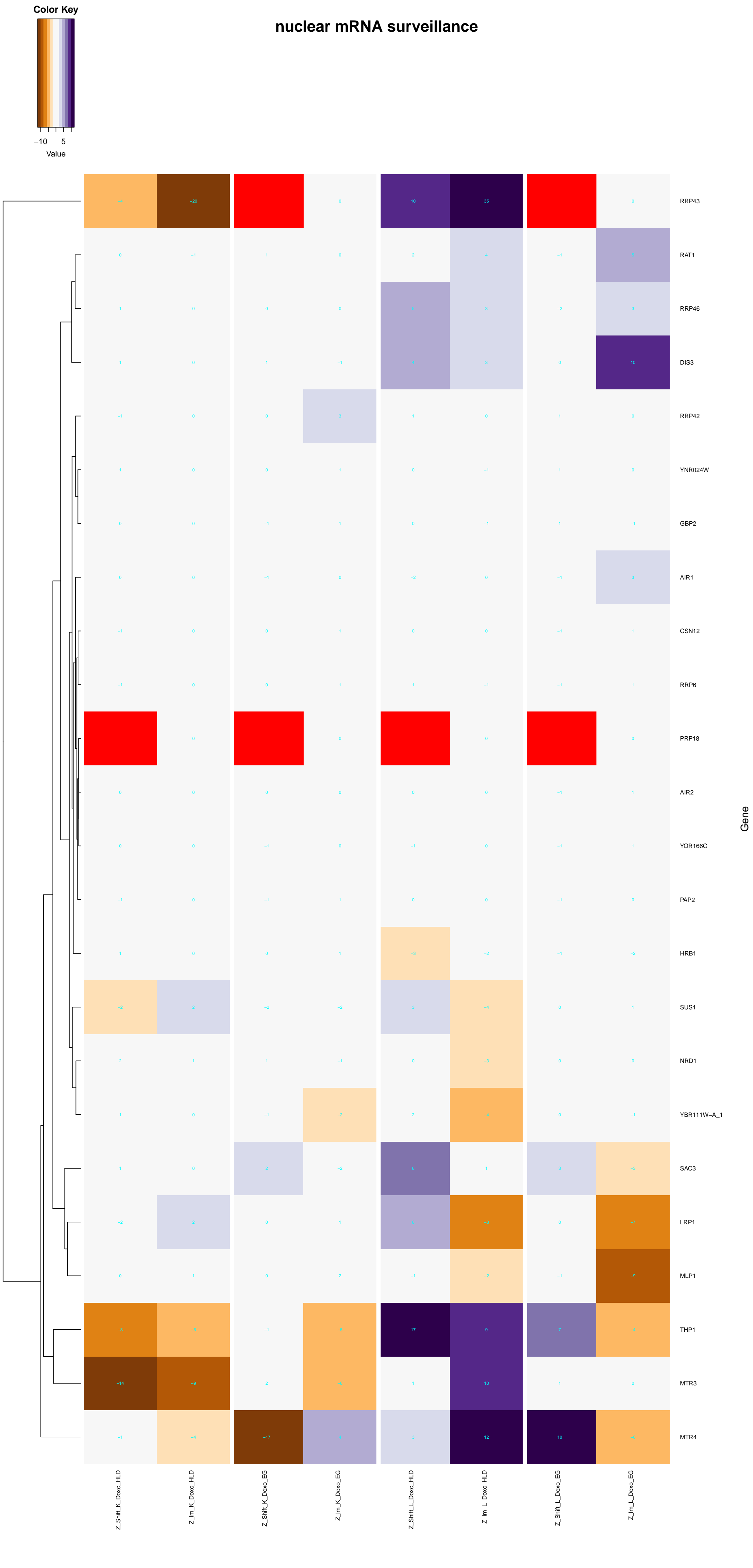

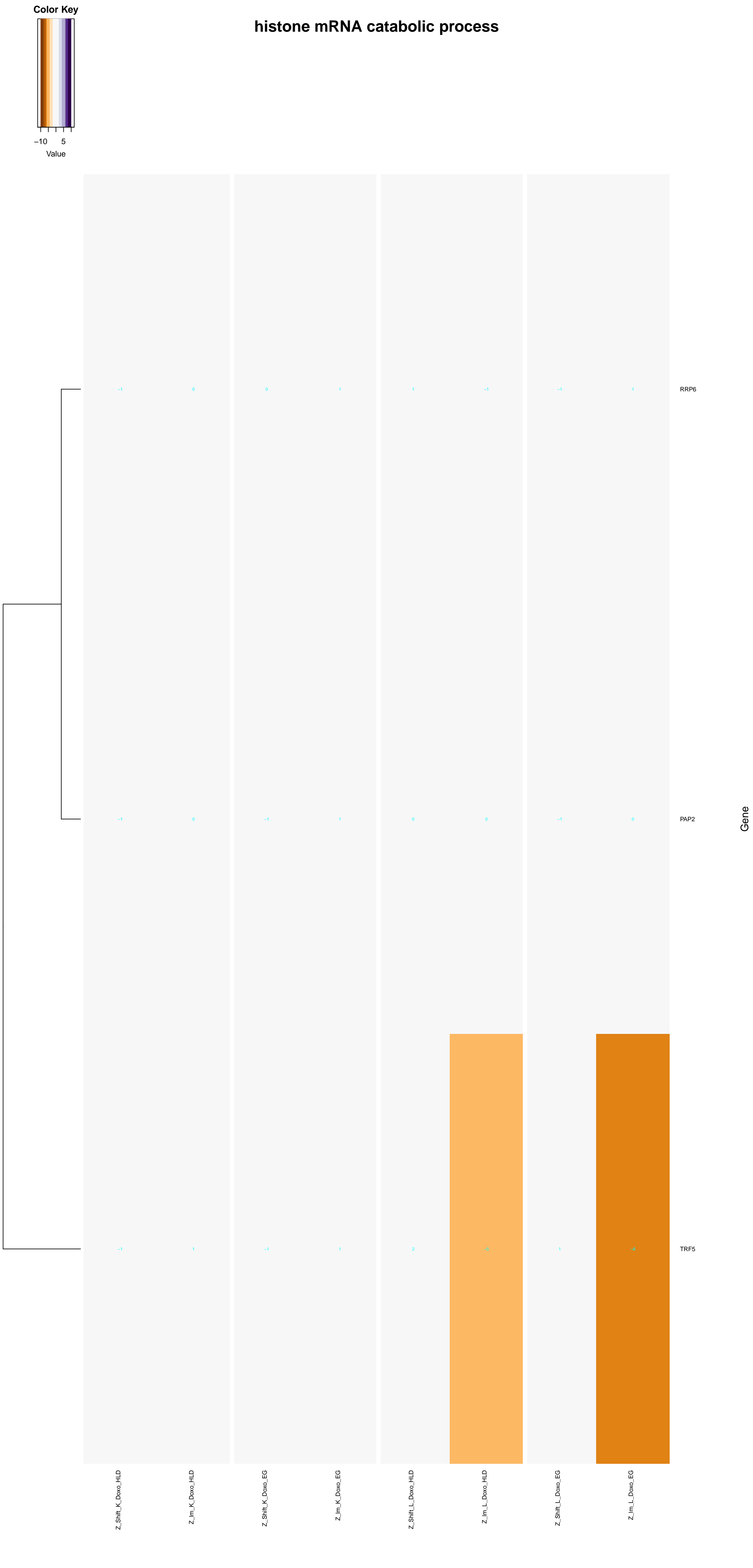

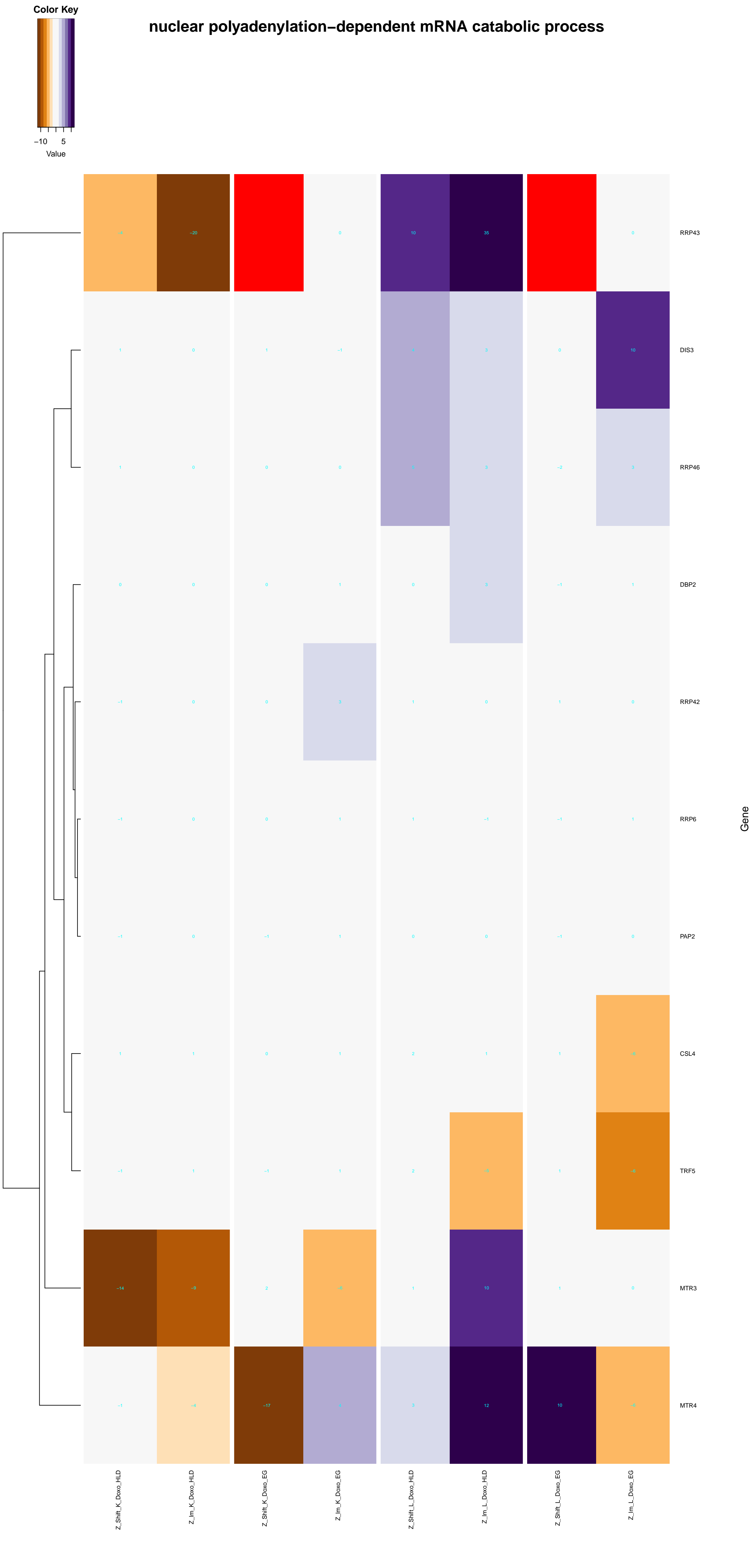

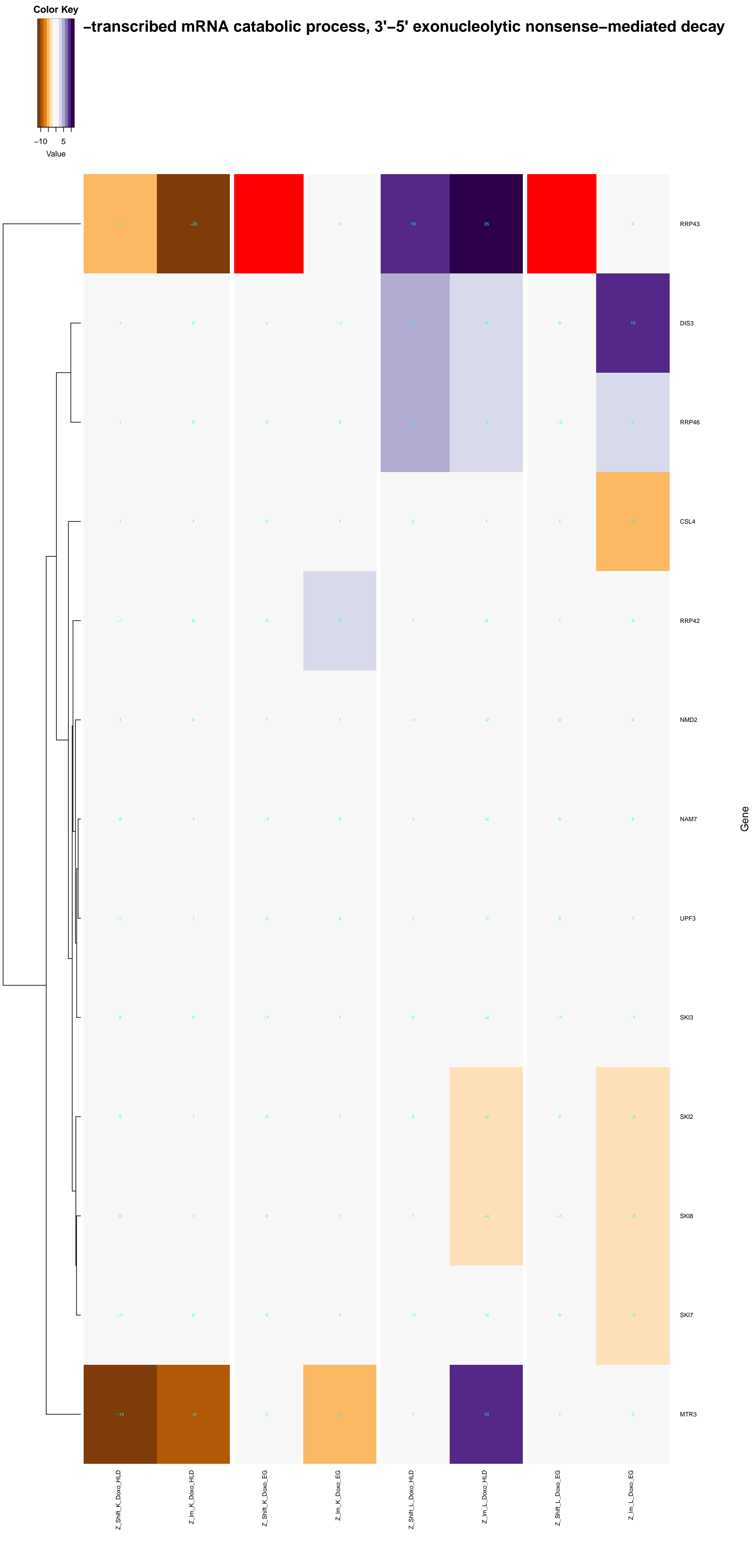

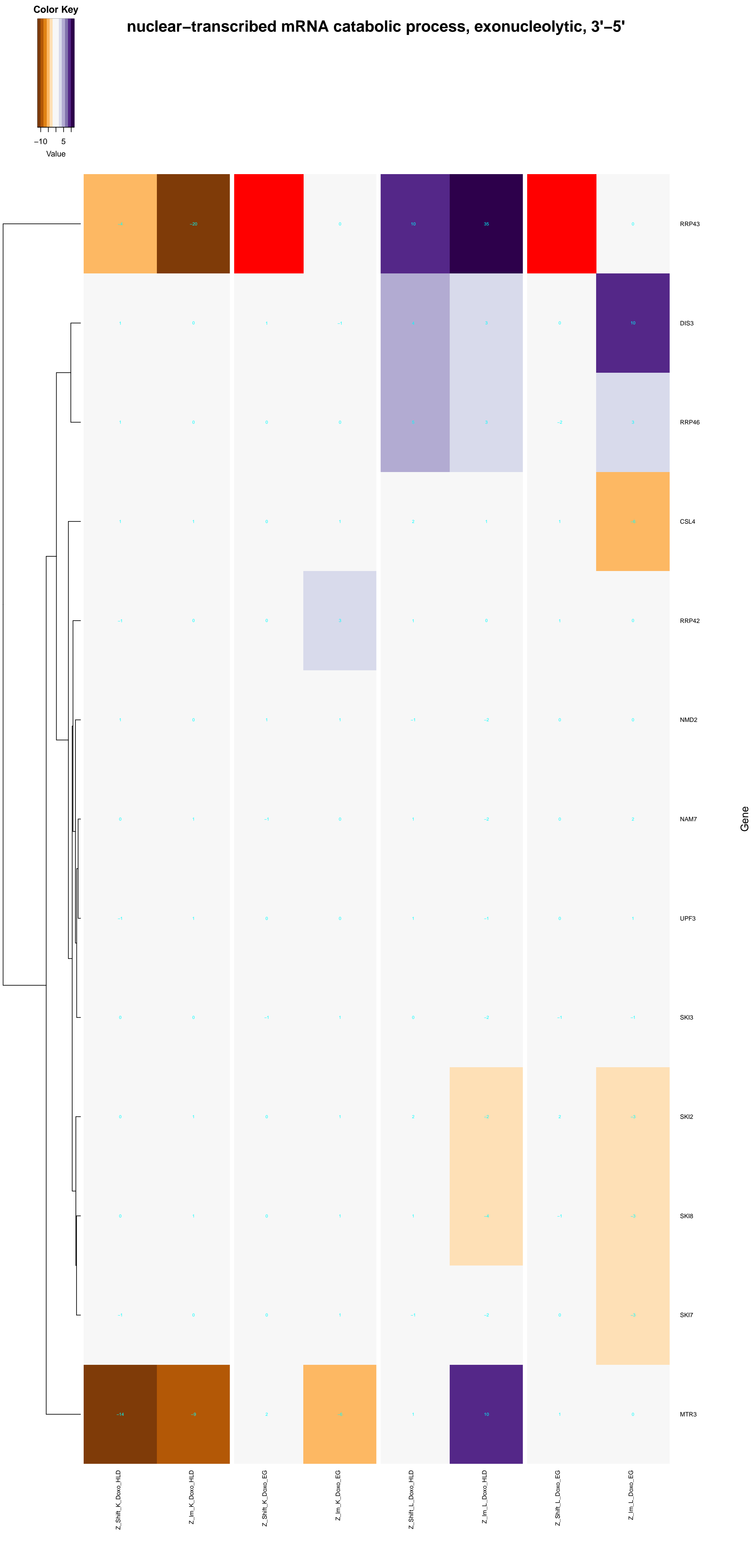

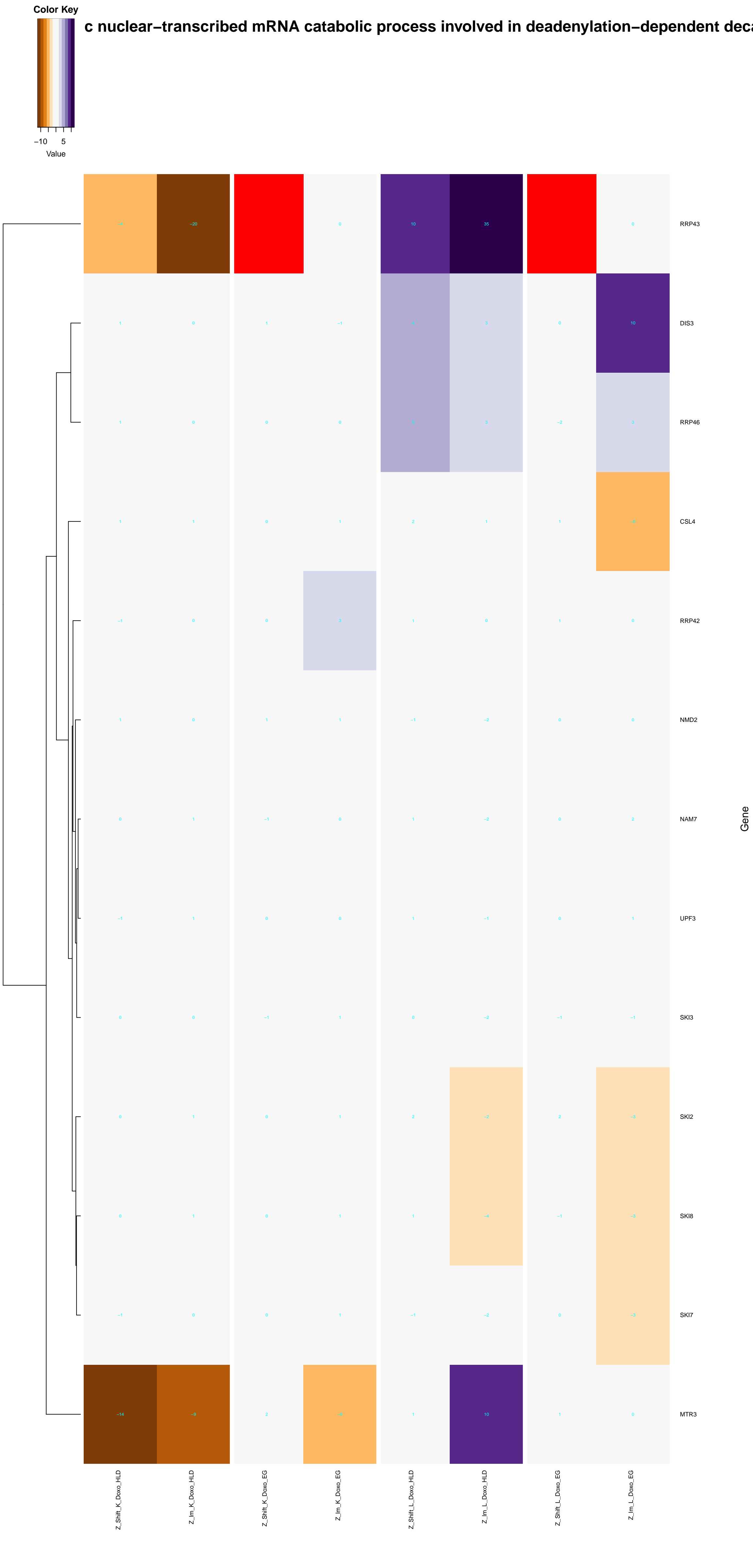

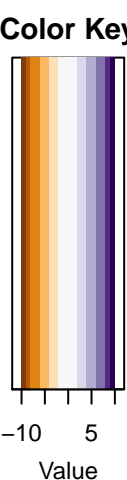

nuclear mRNA surveillance of spliceosomal pre-mRNA splicing

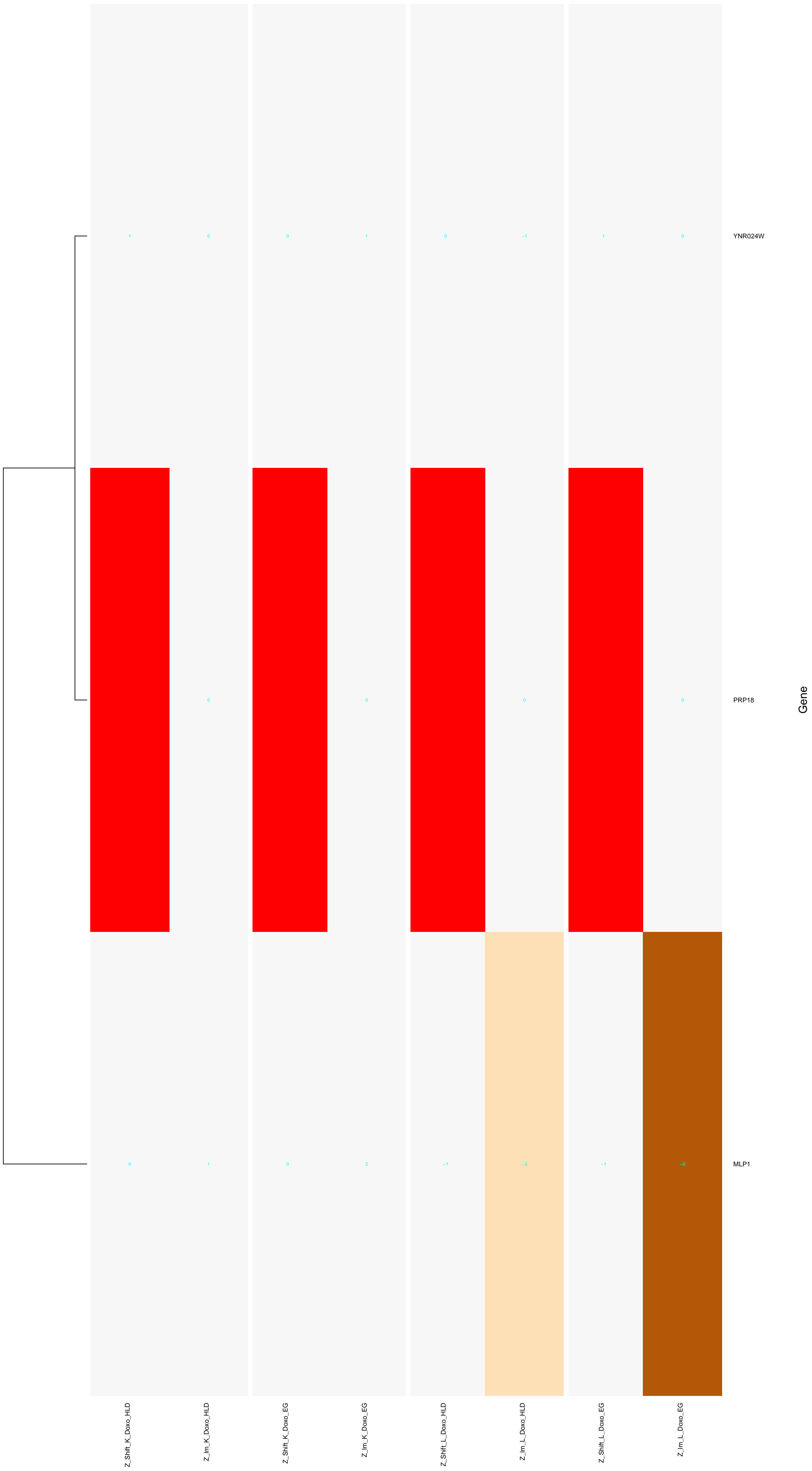

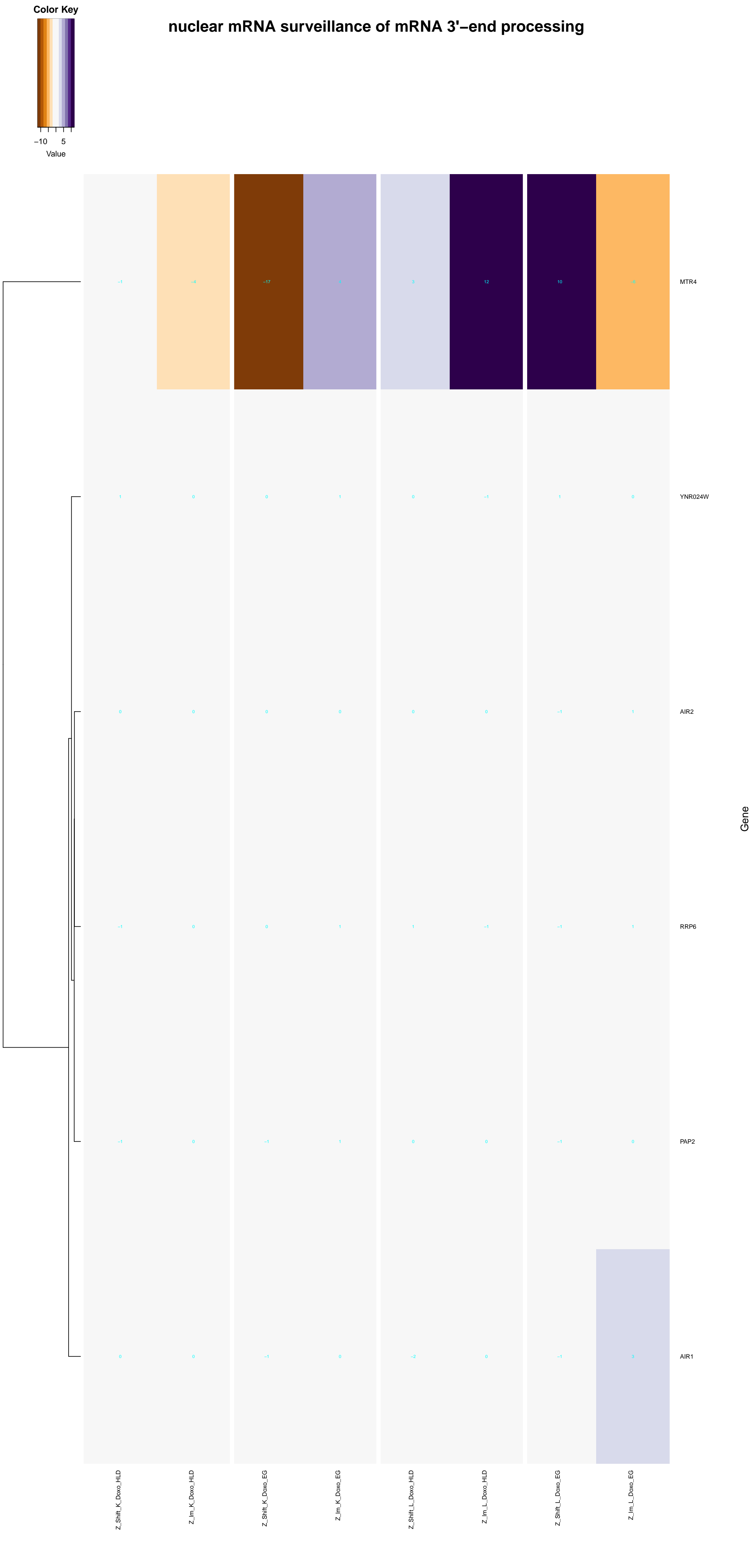

Supplement: Supplementary file 9 — Additional file 9. GO term-specific heatmaps for REMc/GTF-enriched clusters. GO term-specific heatmaps for significant GO process terms were generated as described in methods and Figs. 3 and 4. Any related child terms are presented in subsequent pages of the parent file name. GO terms with more than 100 children, with 2 or fewer genes annotated to the term, or a file size over 300KB are not shown. All heatmaps are generated with the same layout (see Figs. 3 and 4). [file 40170_2019_201_MOESM9_ESM.bz2 › Additional_File9_GOTermHeatmaps/Additional_File9_GOTermHeatmap/mRNA_catabolic_process.pdf]

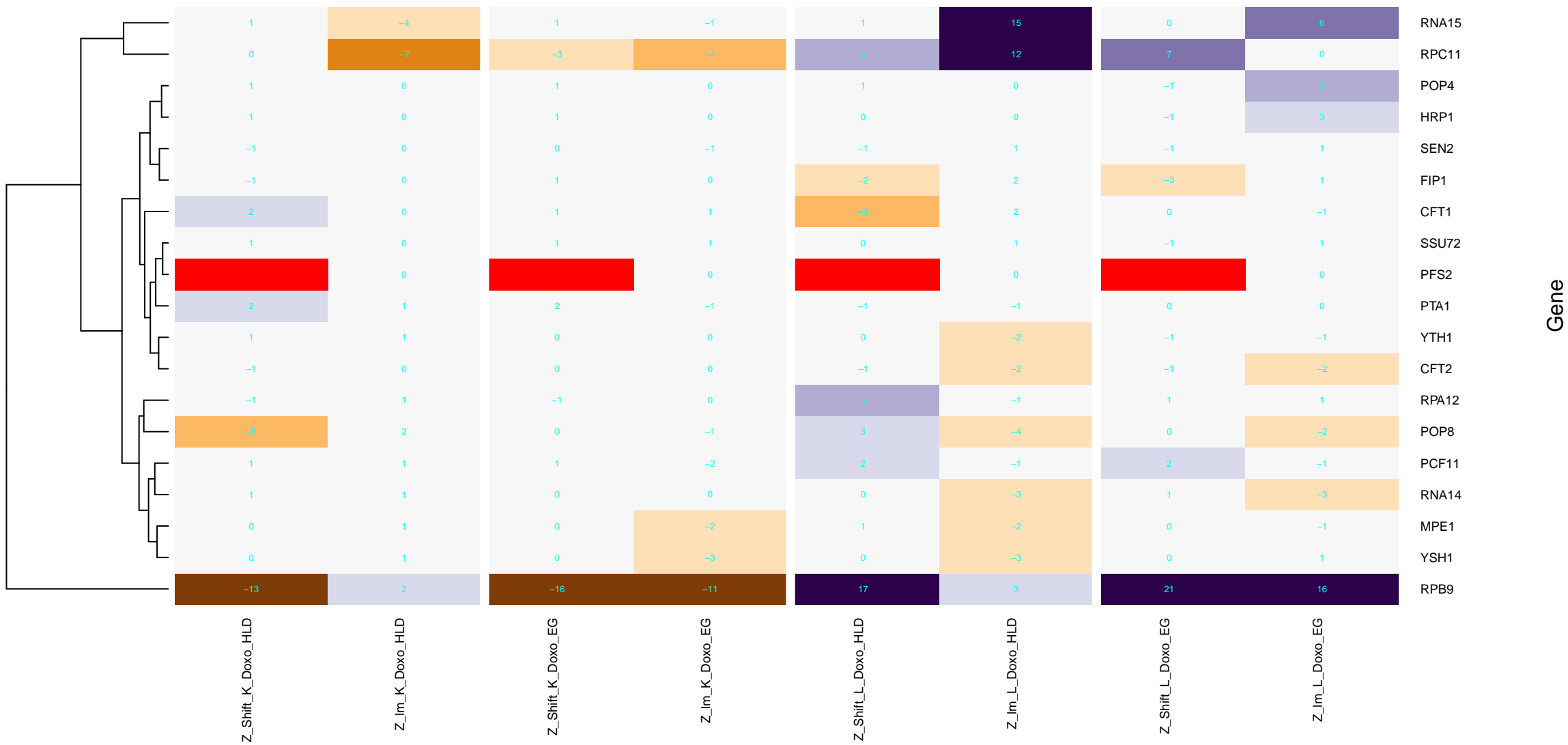

Supplement: Supplementary file 9 — Additional file 9. GO term-specific heatmaps for REMc/GTF-enriched clusters. GO term-specific heatmaps for significant GO process terms were generated as described in methods and Figs. 3 and 4. Any related child terms are presented in subsequent pages of the parent file name. GO terms with more than 100 children, with 2 or fewer genes annotated to the term, or a file size over 300KB are not shown. All heatmaps are generated with the same layout (see Figs. 3 and 4). [file 40170_2019_201_MOESM9_ESM.bz2 › Additional_File9_GOTermHeatmaps/Additional_File9_GOTermHeatmap/mRNA_cleavage.pdf]

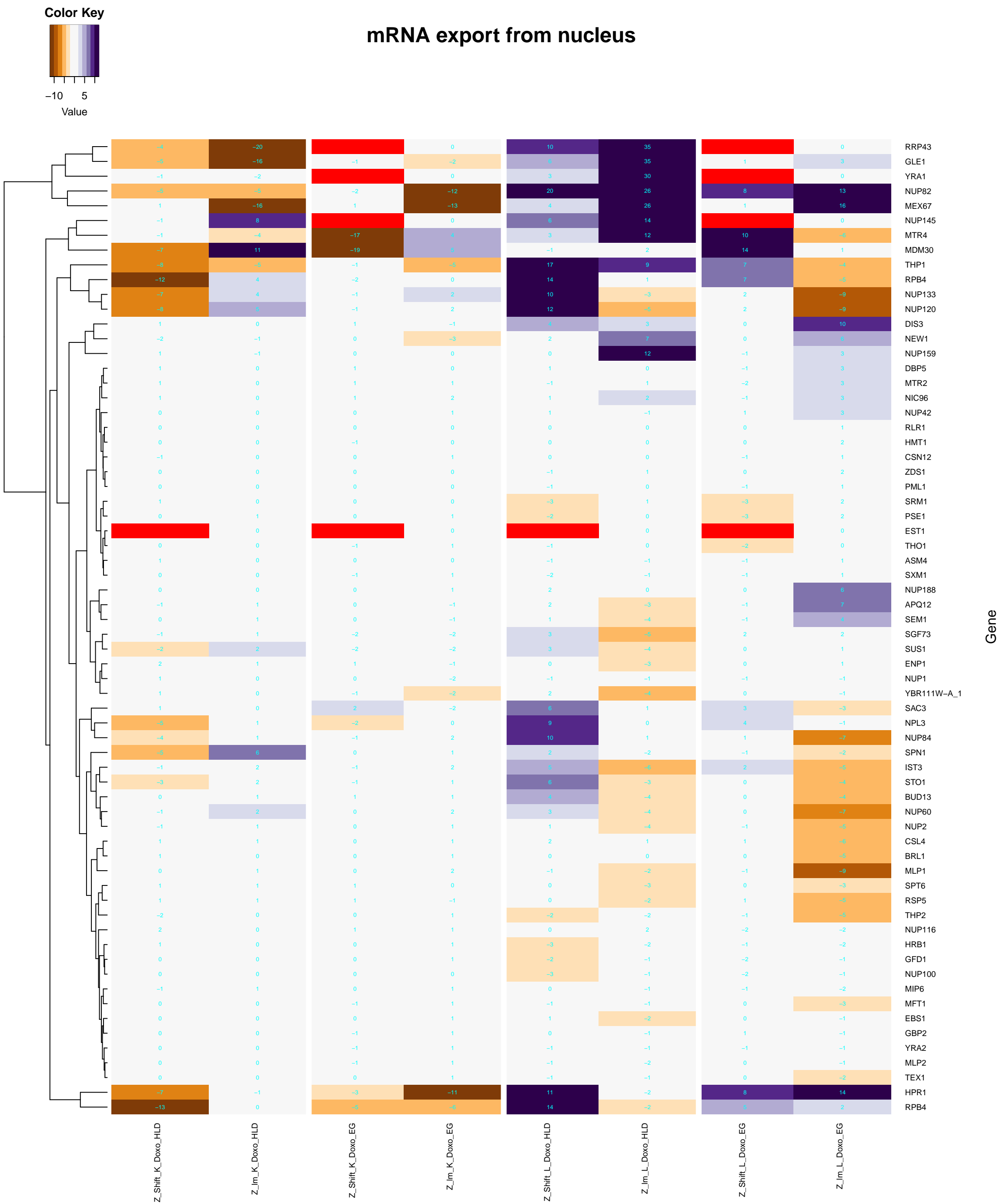



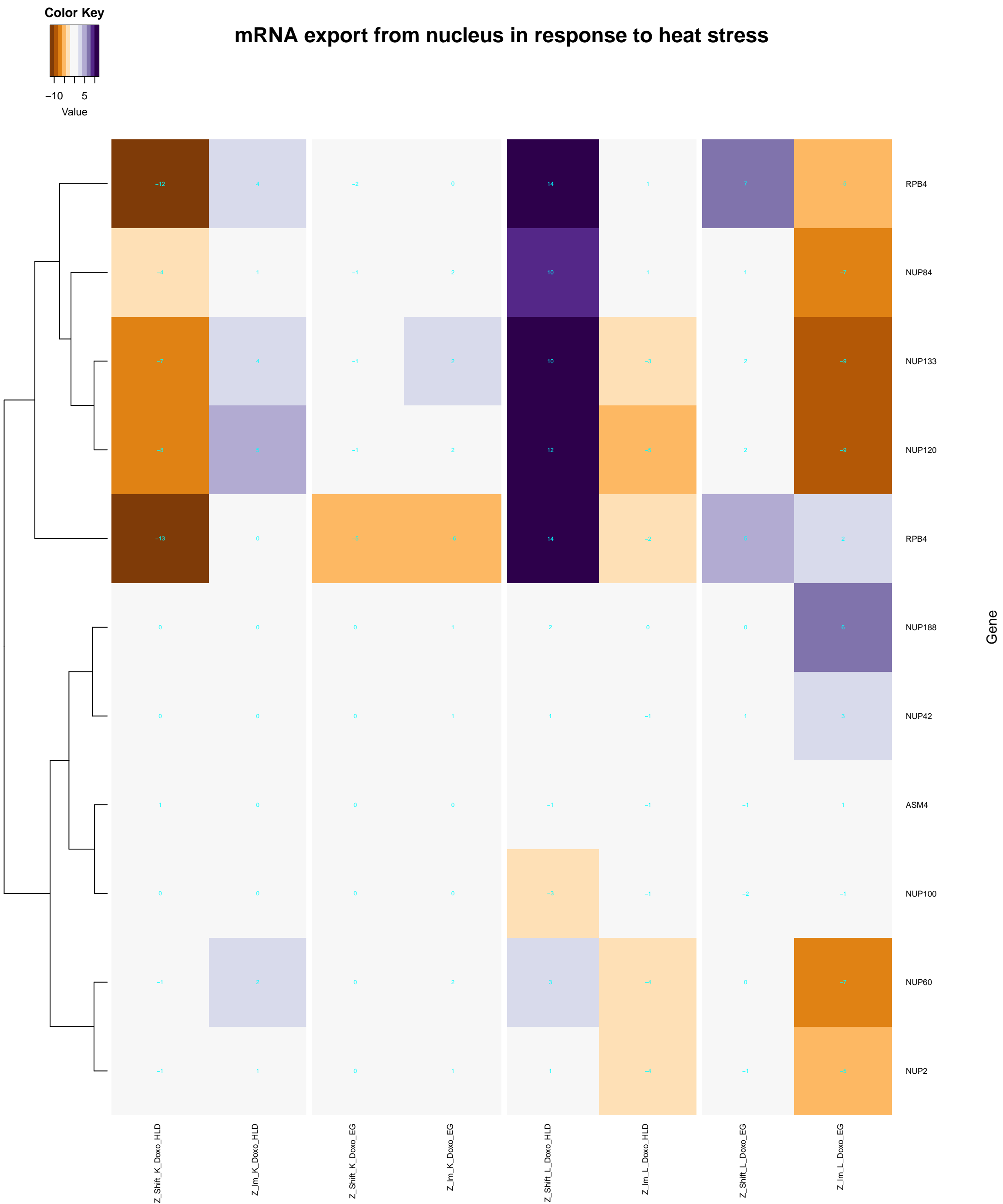

Supplement: Supplementary file 9 — Additional file 9. GO term-specific heatmaps for REMc/GTF-enriched clusters. GO term-specific heatmaps for significant GO process terms were generated as described in methods and Figs. 3 and 4. Any related child terms are presented in subsequent pages of the parent file name. GO terms with more than 100 children, with 2 or fewer genes annotated to the term, or a file size over 300KB are not shown. All heatmaps are generated with the same layout (see Figs. 3 and 4). [file 40170_2019_201_MOESM9_ESM.bz2 › Additional_File9_GOTermHeatmaps/Additional_File9_GOTermHeatmap/mRNA_export_from_nucleus.pdf]

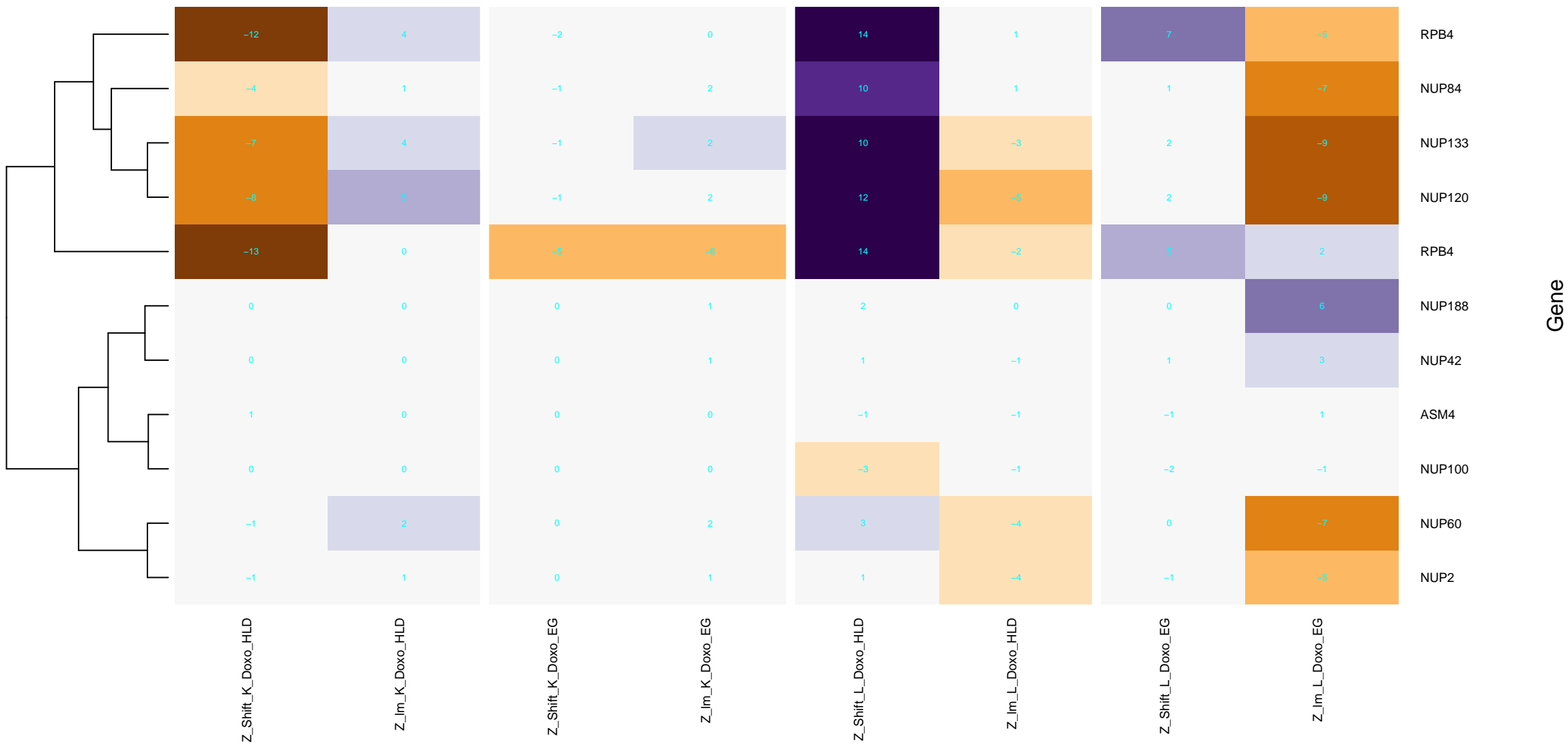

Supplement: Supplementary file 9 — Additional file 9. GO term-specific heatmaps for REMc/GTF-enriched clusters. GO term-specific heatmaps for significant GO process terms were generated as described in methods and Figs. 3 and 4. Any related child terms are presented in subsequent pages of the parent file name. GO terms with more than 100 children, with 2 or fewer genes annotated to the term, or a file size over 300KB are not shown. All heatmaps are generated with the same layout (see Figs. 3 and 4). [file 40170_2019_201_MOESM9_ESM.bz2 › Additional_File9_GOTermHeatmaps/Additional_File9_GOTermHeatmap/mRNA_export_from_nucleus_in_response_to_heat_stress.pdf]

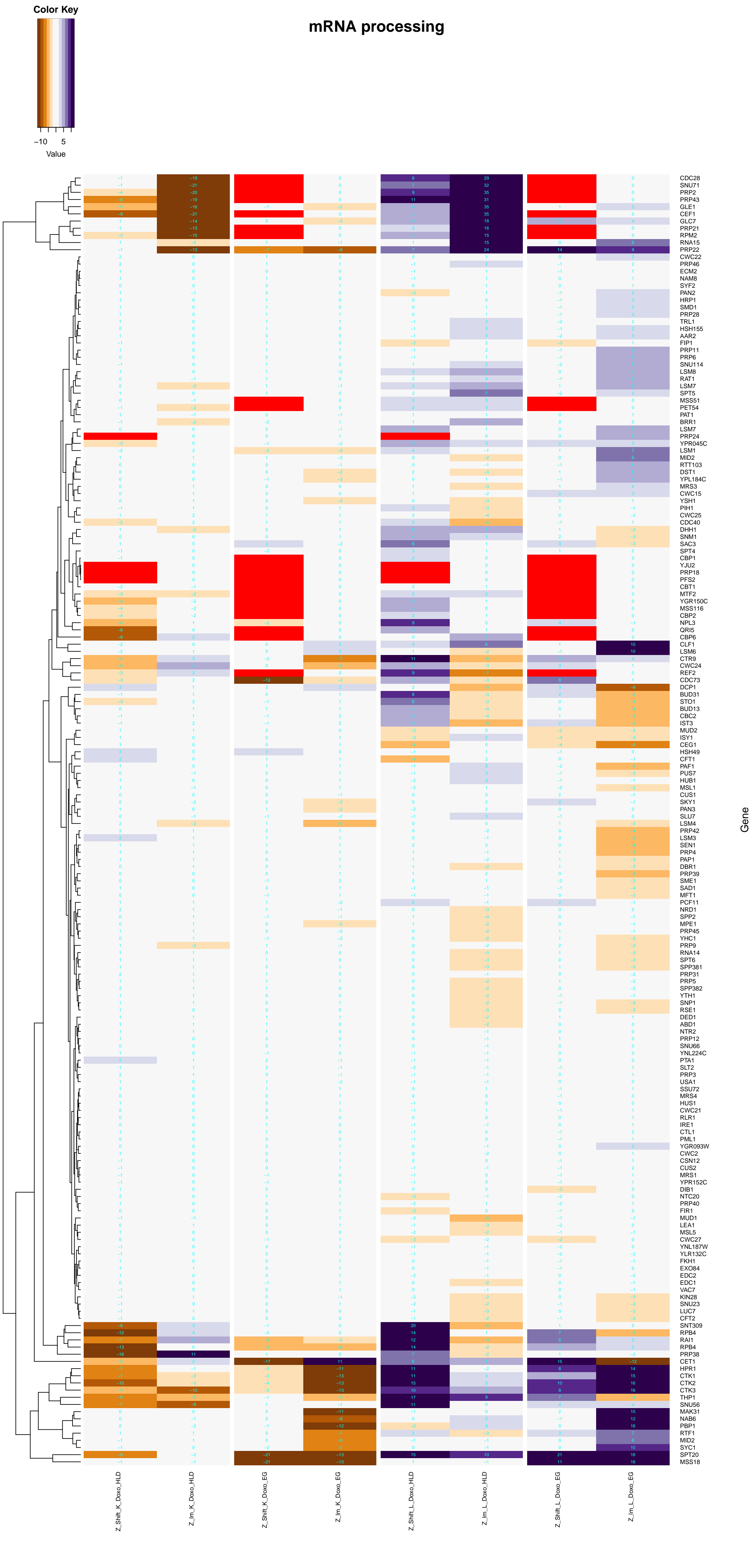

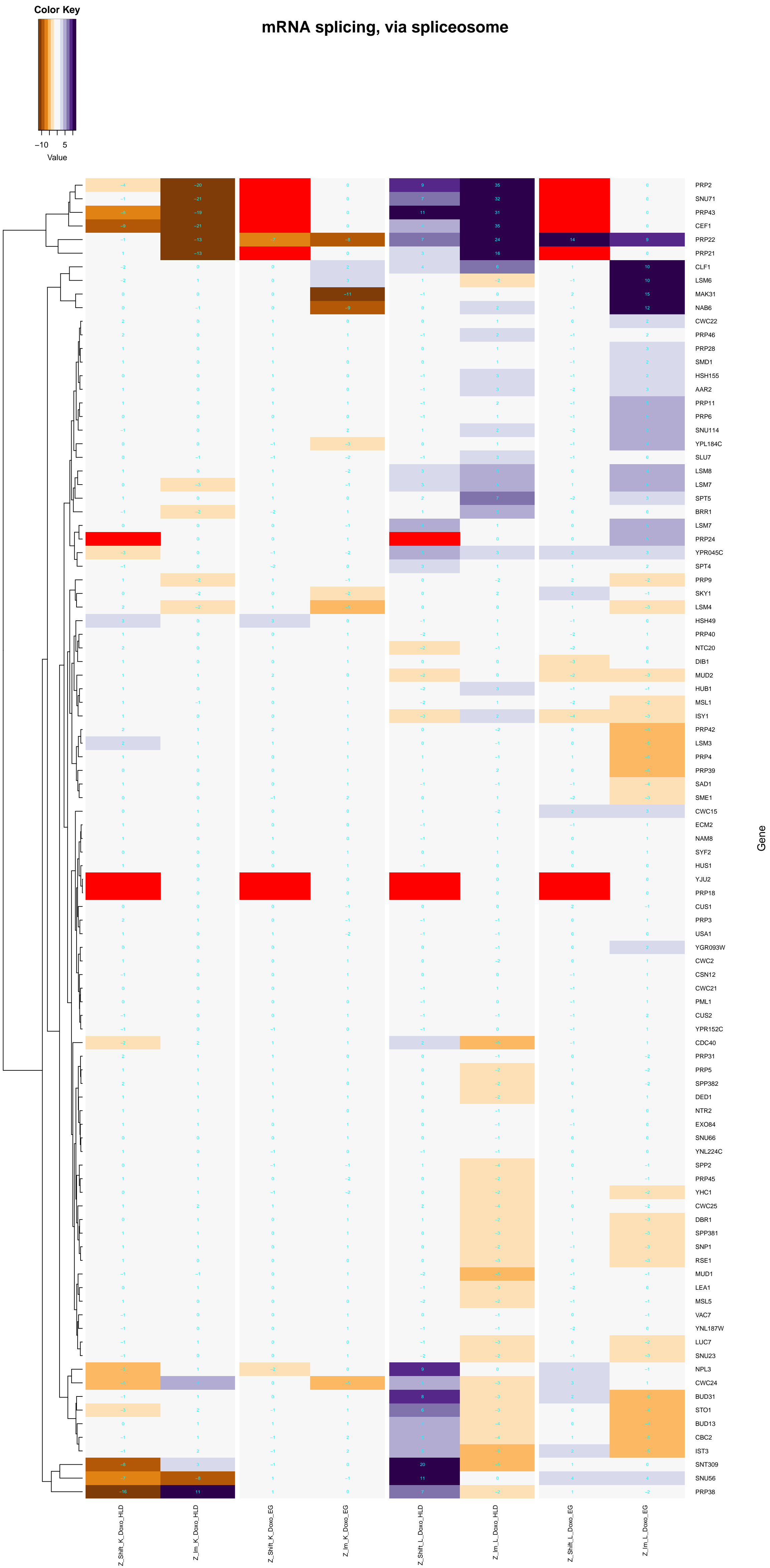

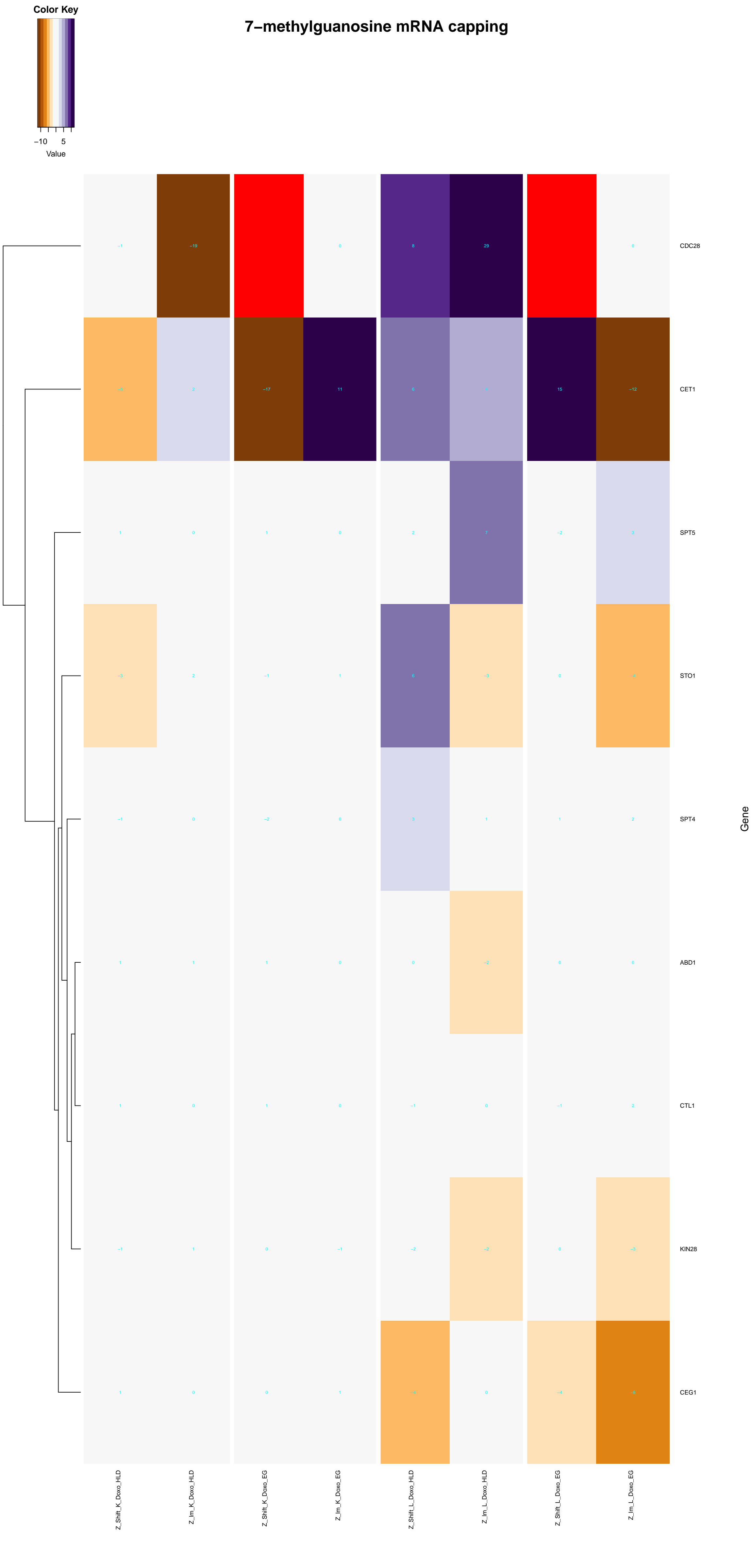

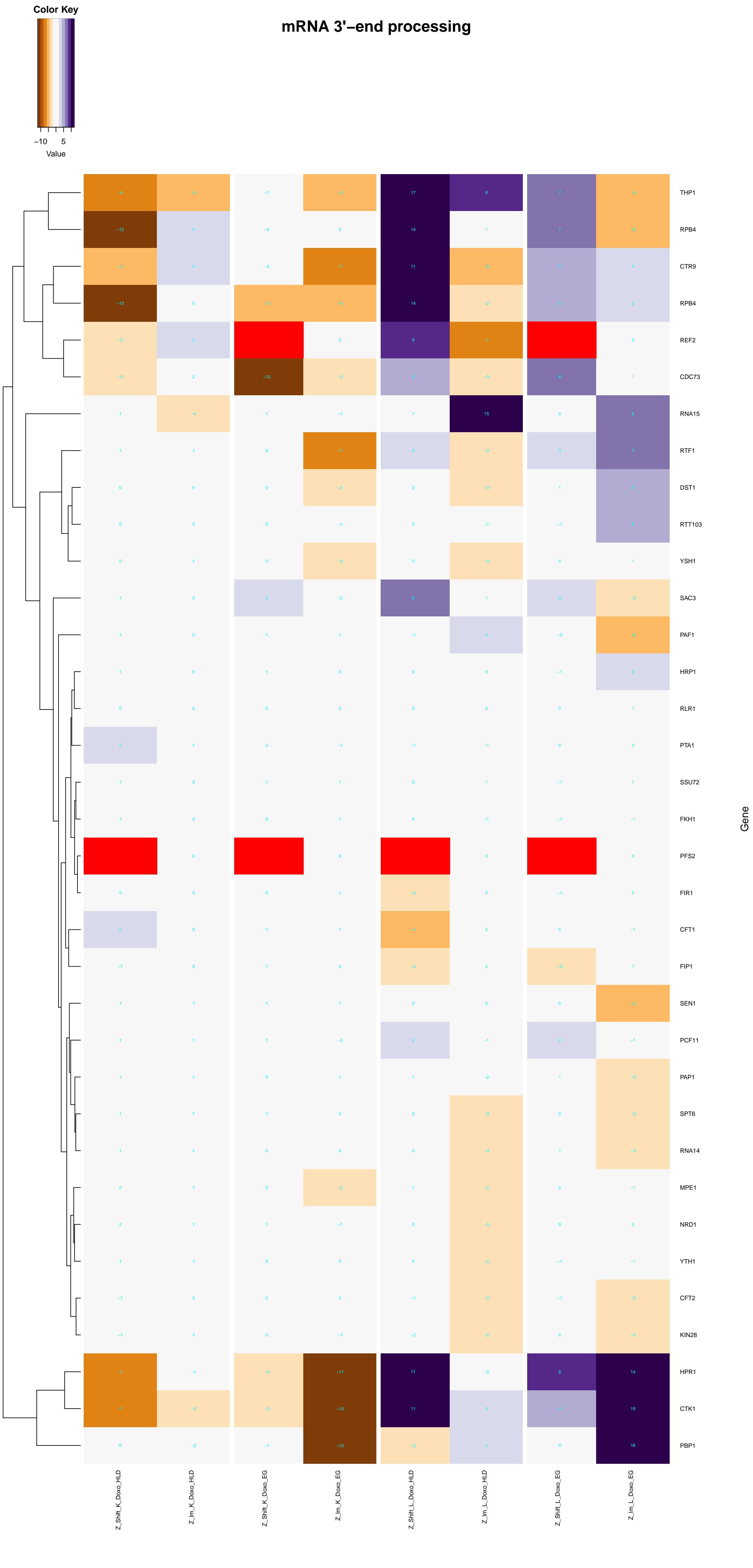

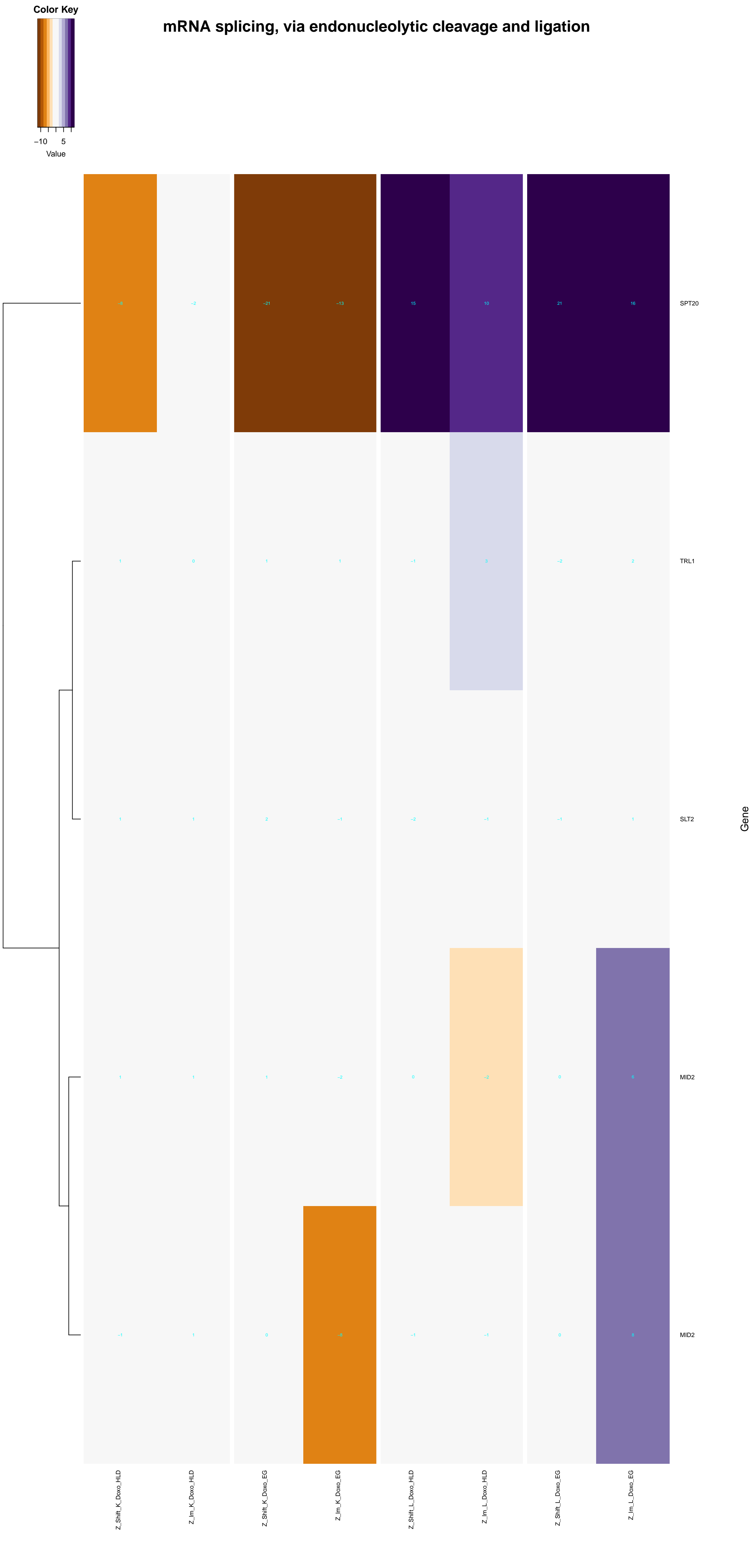

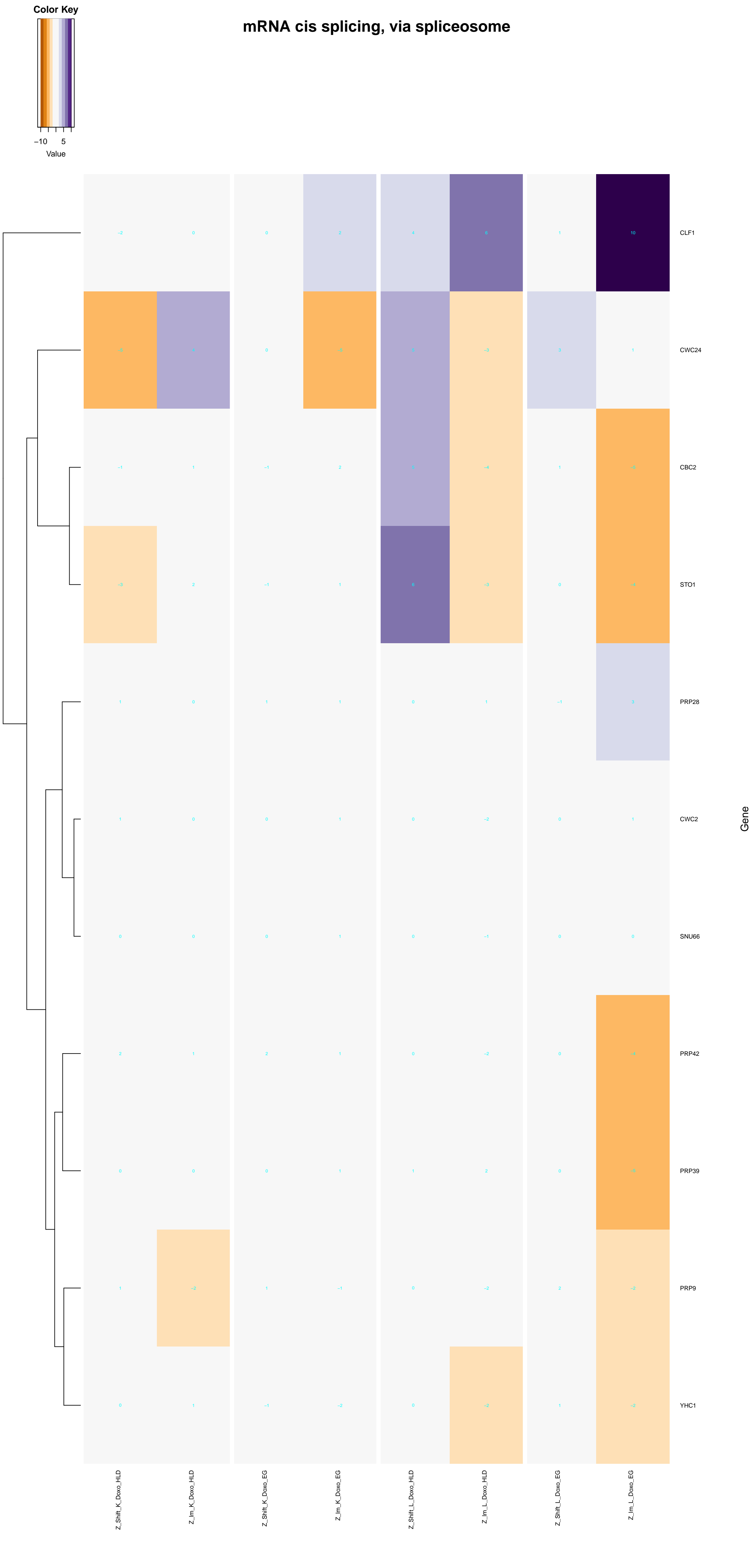

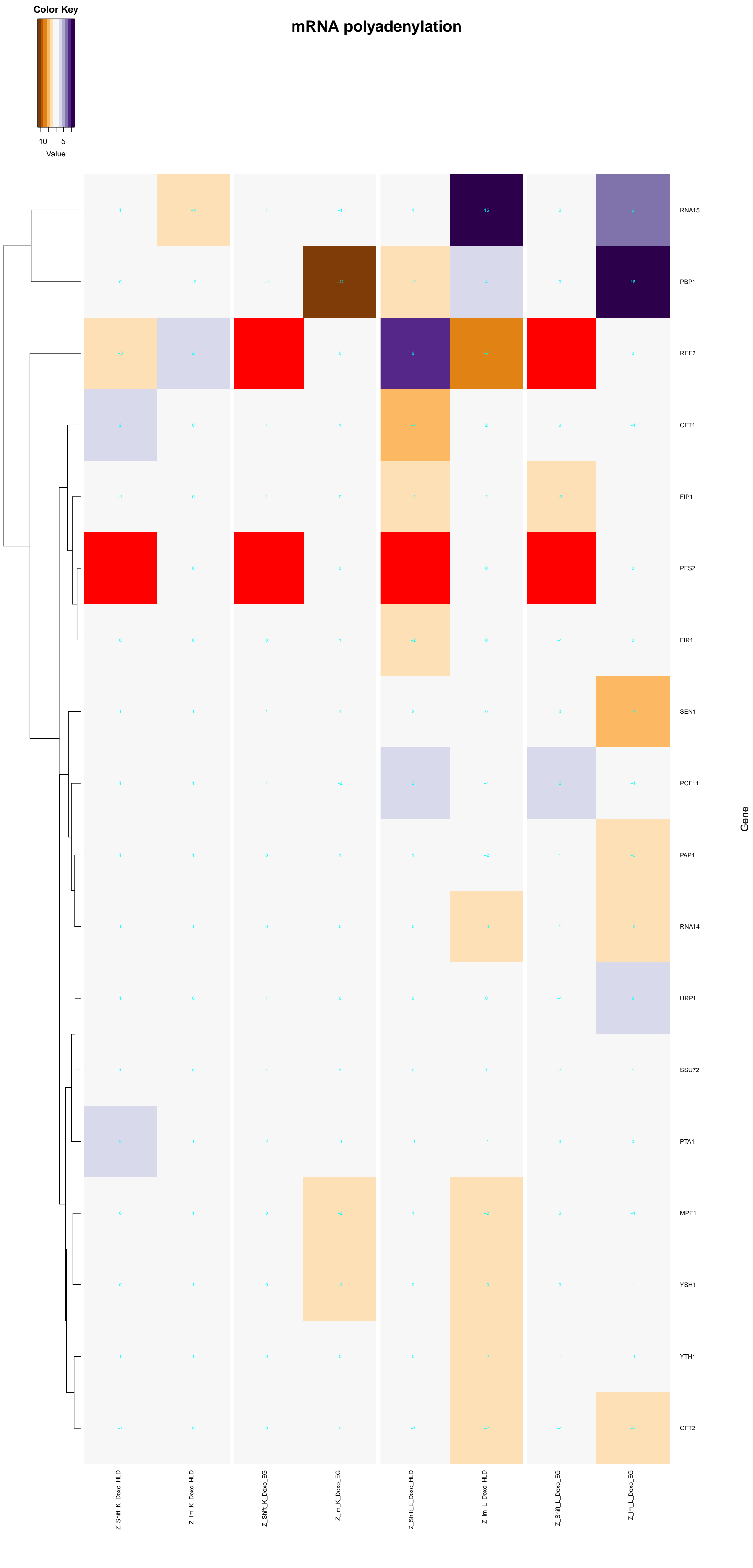

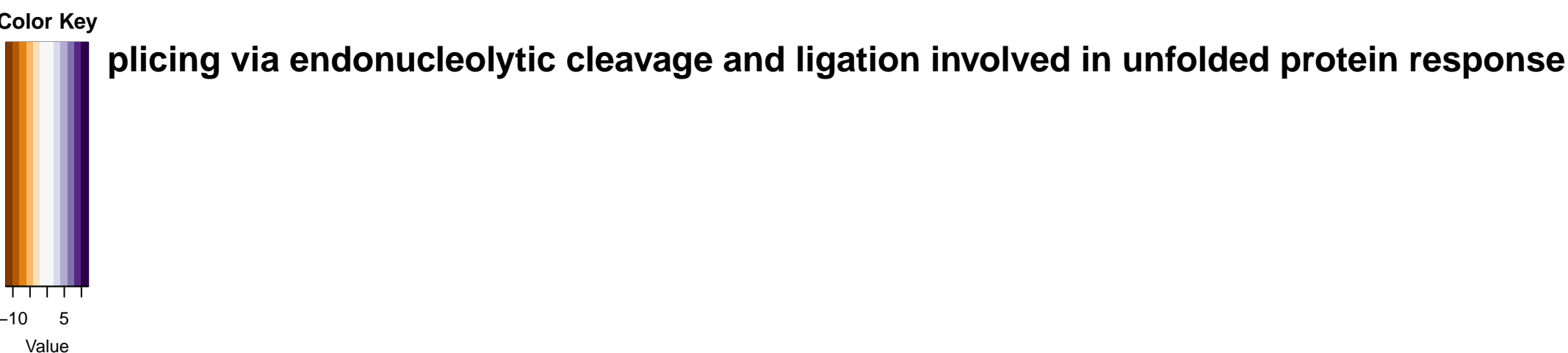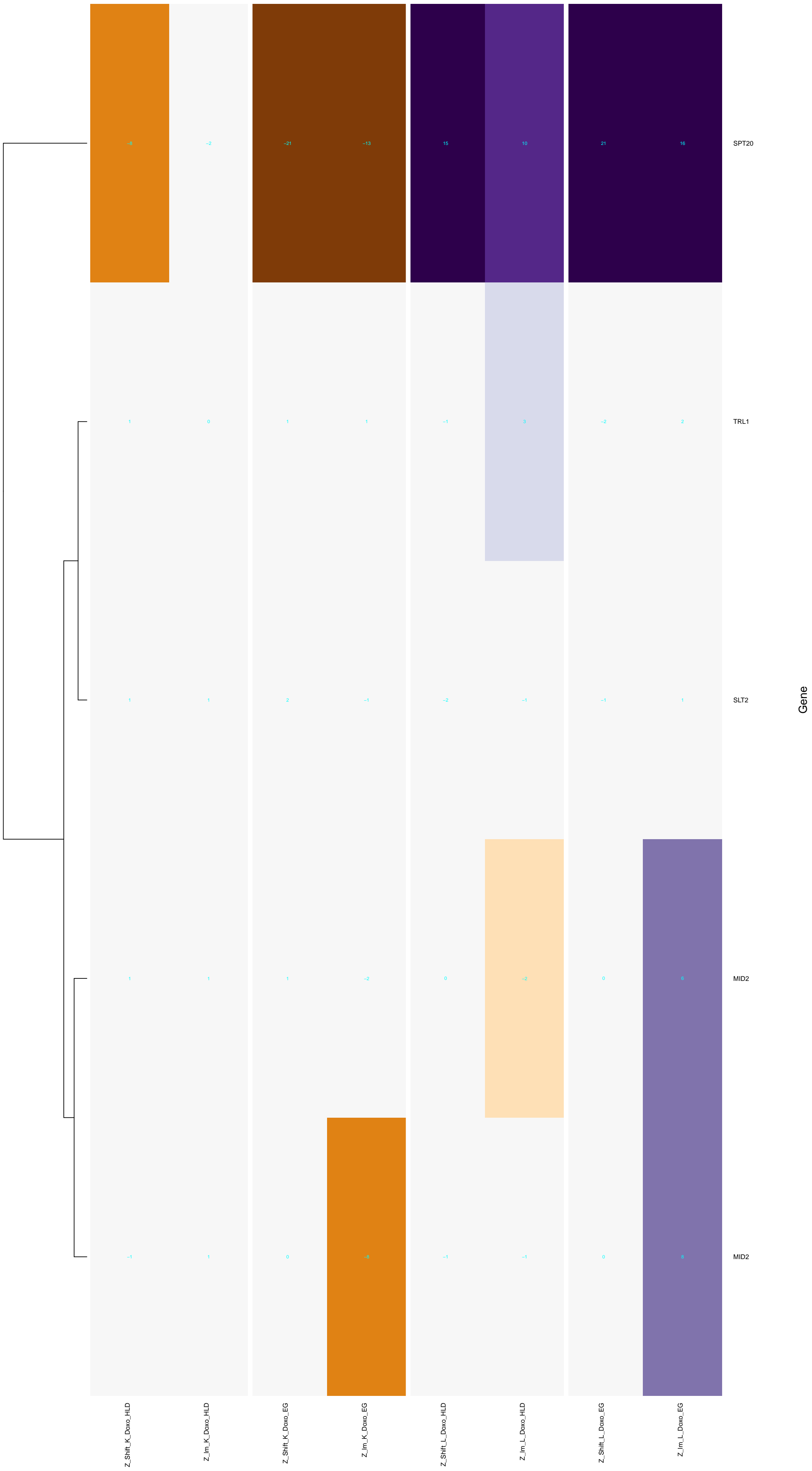

Supplement: Supplementary file 9 — Additional file 9. GO term-specific heatmaps for REMc/GTF-enriched clusters. GO term-specific heatmaps for significant GO process terms were generated as described in methods and Figs. 3 and 4. Any related child terms are presented in subsequent pages of the parent file name. GO terms with more than 100 children, with 2 or fewer genes annotated to the term, or a file size over 300KB are not shown. All heatmaps are generated with the same layout (see Figs. 3 and 4). [file 40170_2019_201_MOESM9_ESM.bz2 › Additional_File9_GOTermHeatmaps/Additional_File9_GOTermHeatmap/mRNA_processing.pdf]

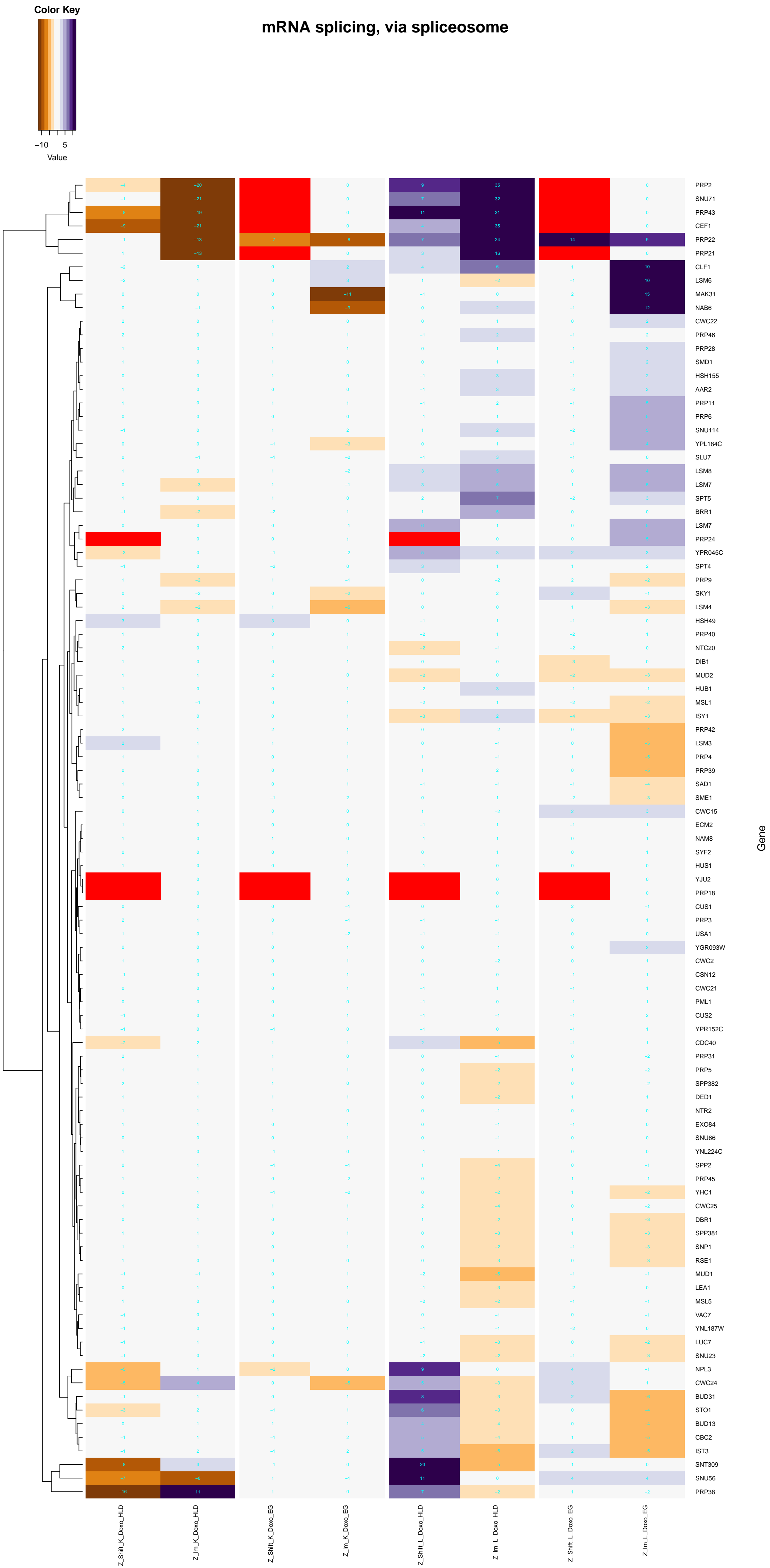

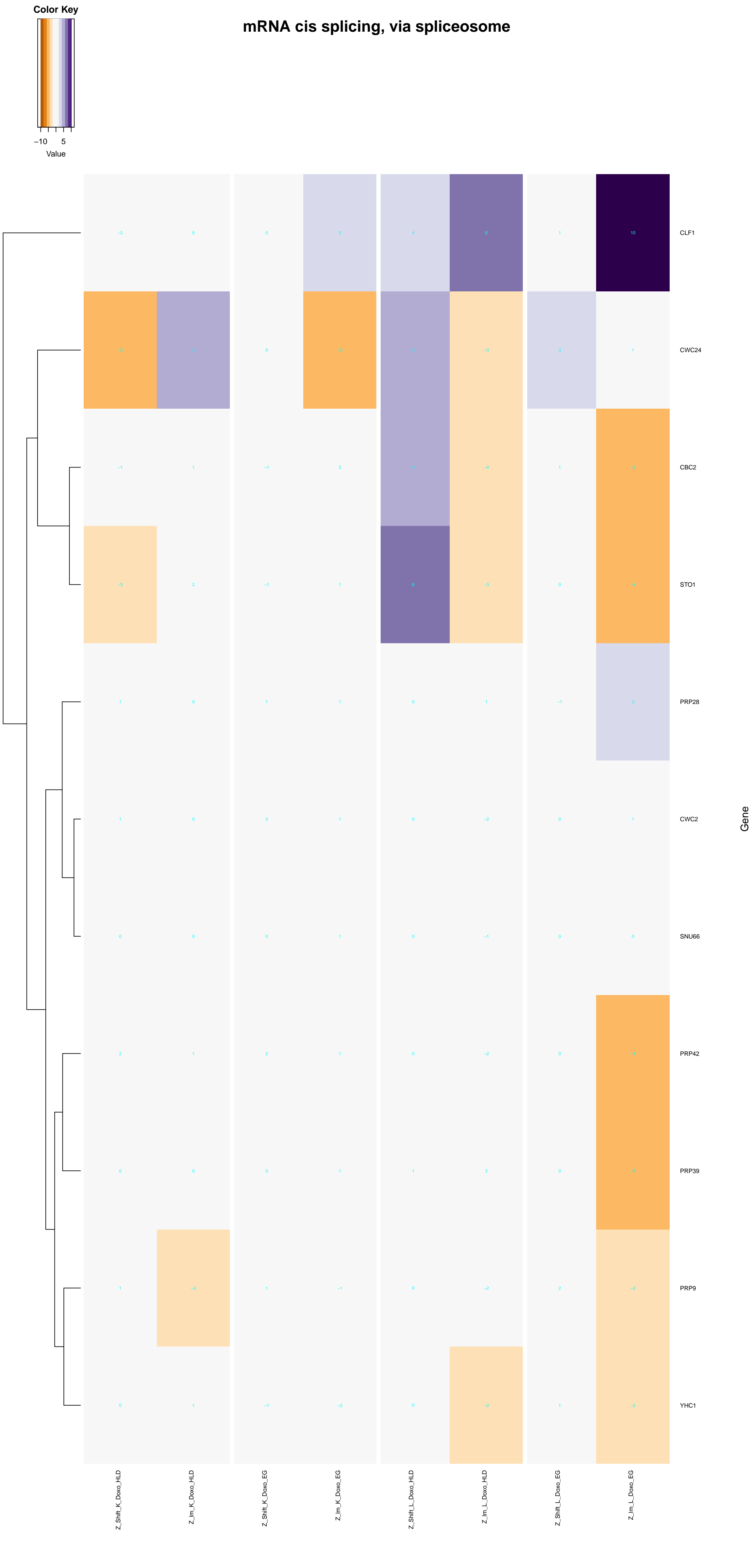

Supplement: Supplementary file 9 — Additional file 9. GO term-specific heatmaps for REMc/GTF-enriched clusters. GO term-specific heatmaps for significant GO process terms were generated as described in methods and Figs. 3 and 4. Any related child terms are presented in subsequent pages of the parent file name. GO terms with more than 100 children, with 2 or fewer genes annotated to the term, or a file size over 300KB are not shown. All heatmaps are generated with the same layout (see Figs. 3 and 4). [file 40170_2019_201_MOESM9_ESM.bz2 › Additional_File9_GOTermHeatmaps/Additional_File9_GOTermHeatmap/mRNA_splicing,_via_spliceosome.pdf]

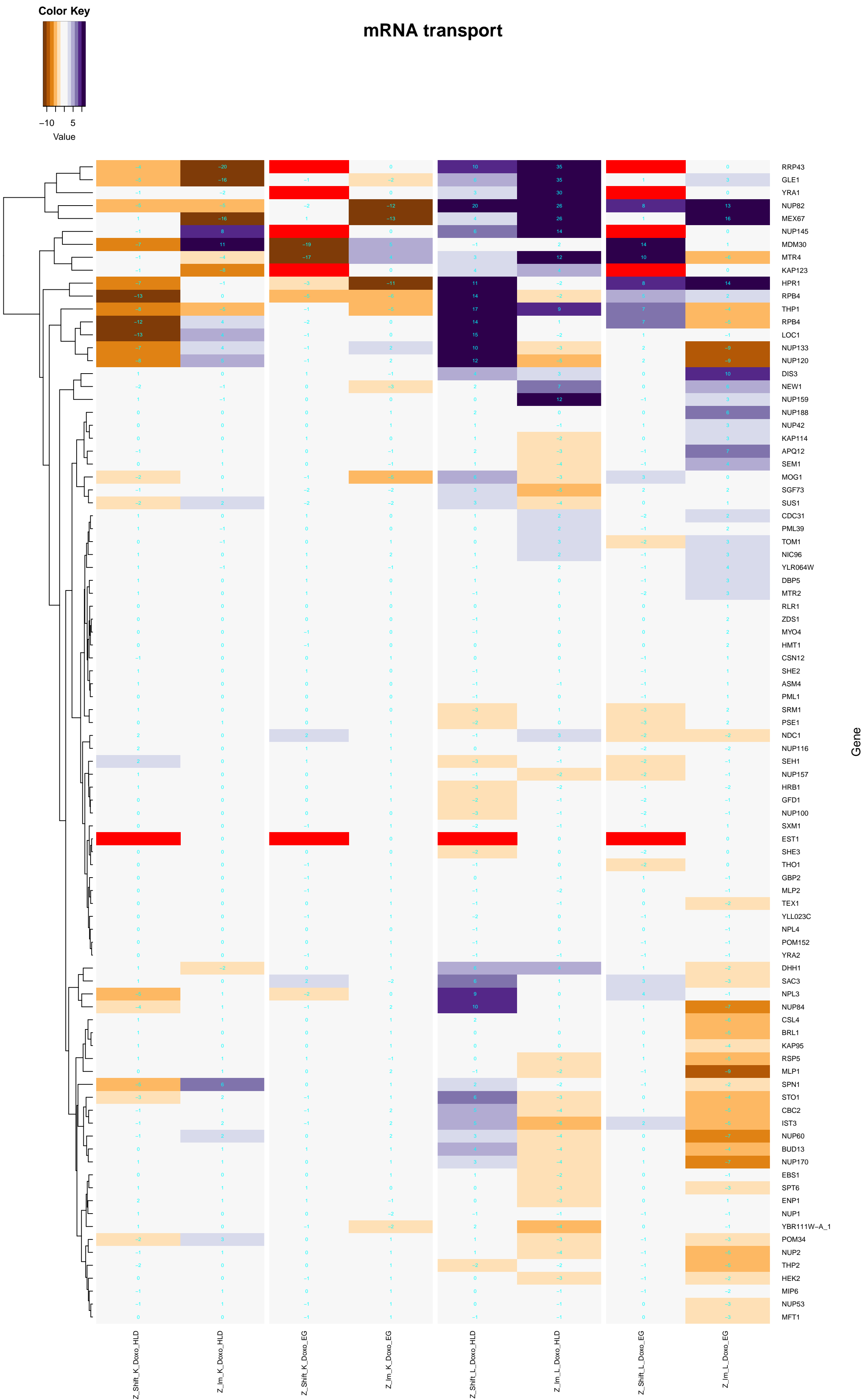

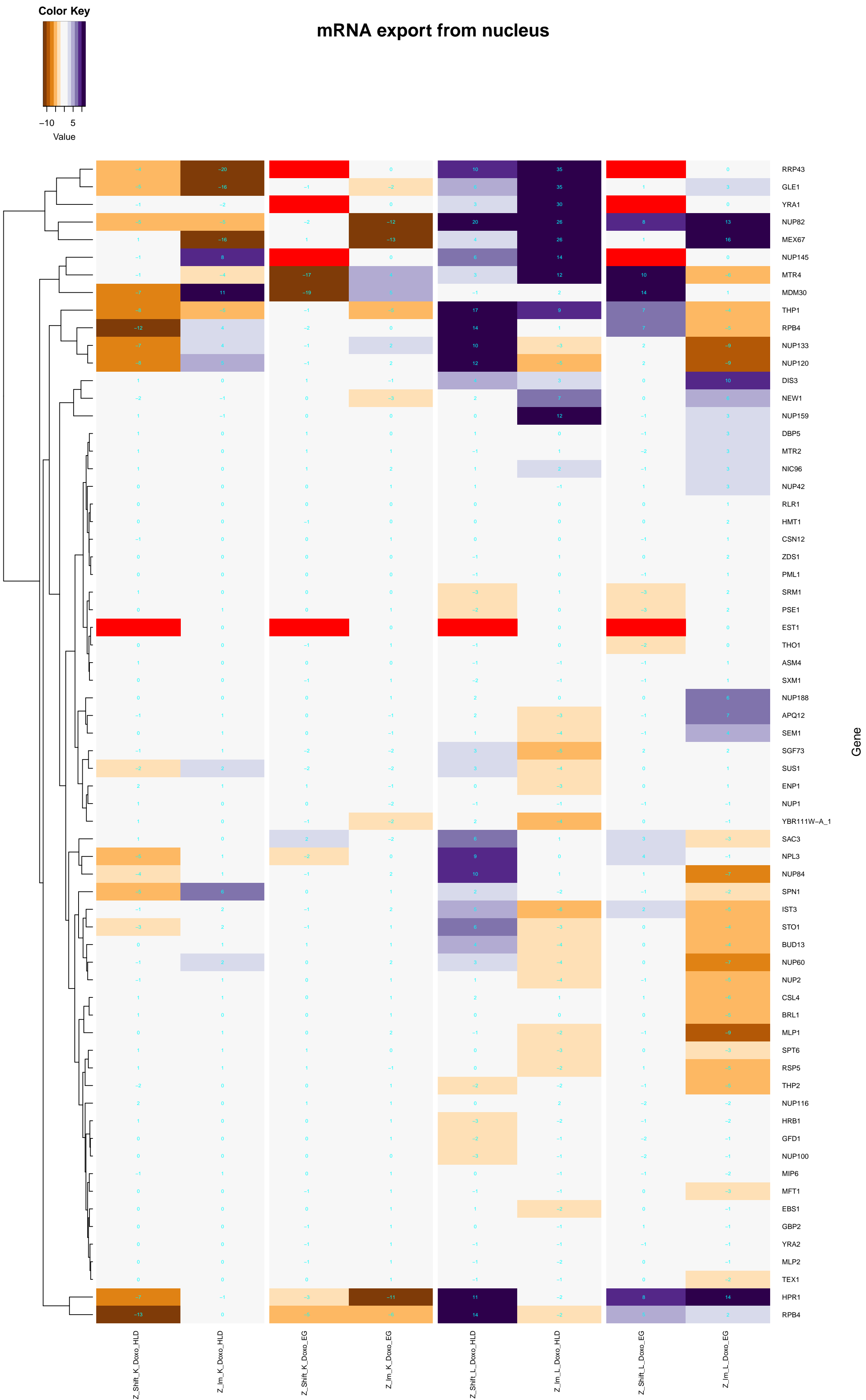

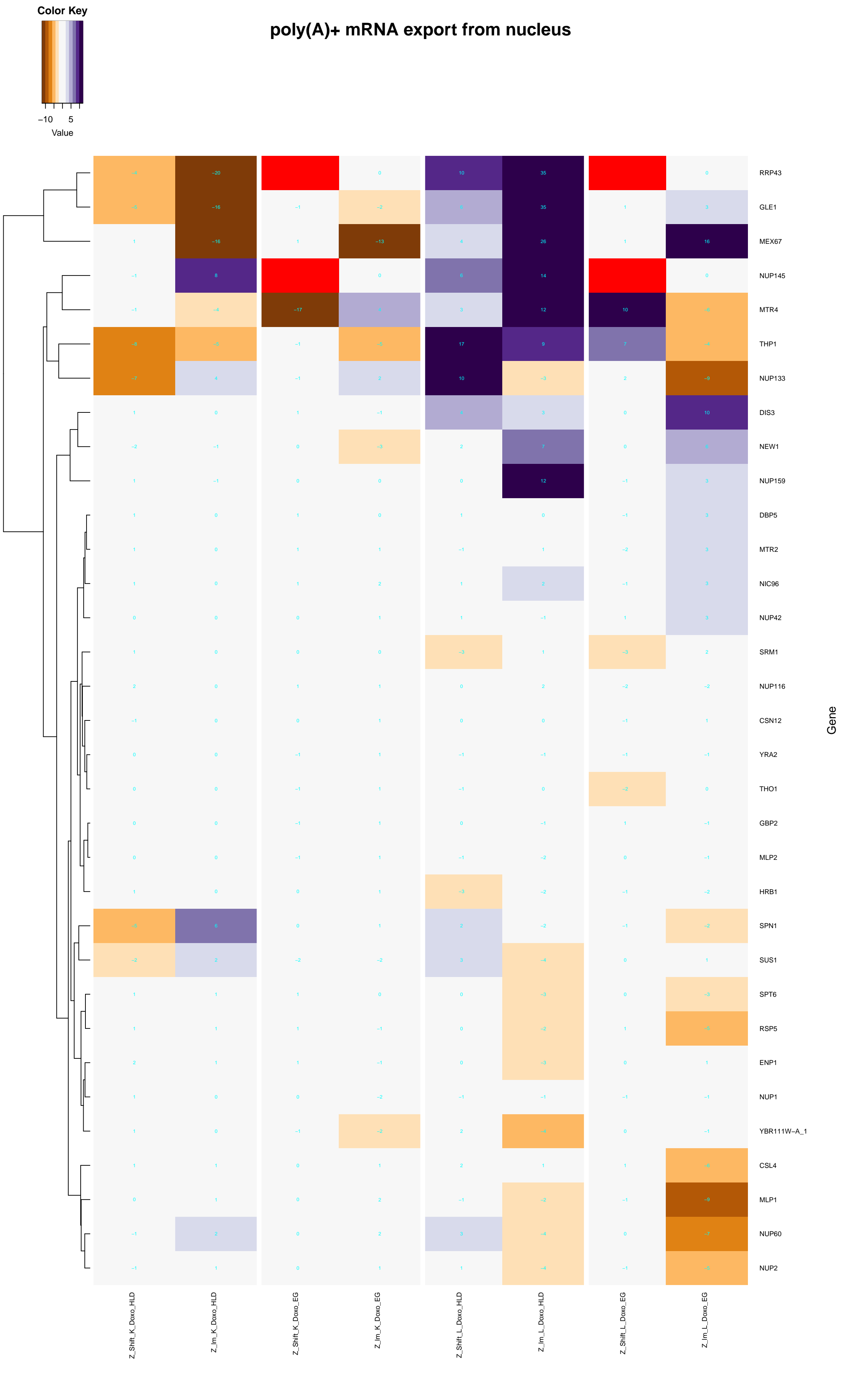

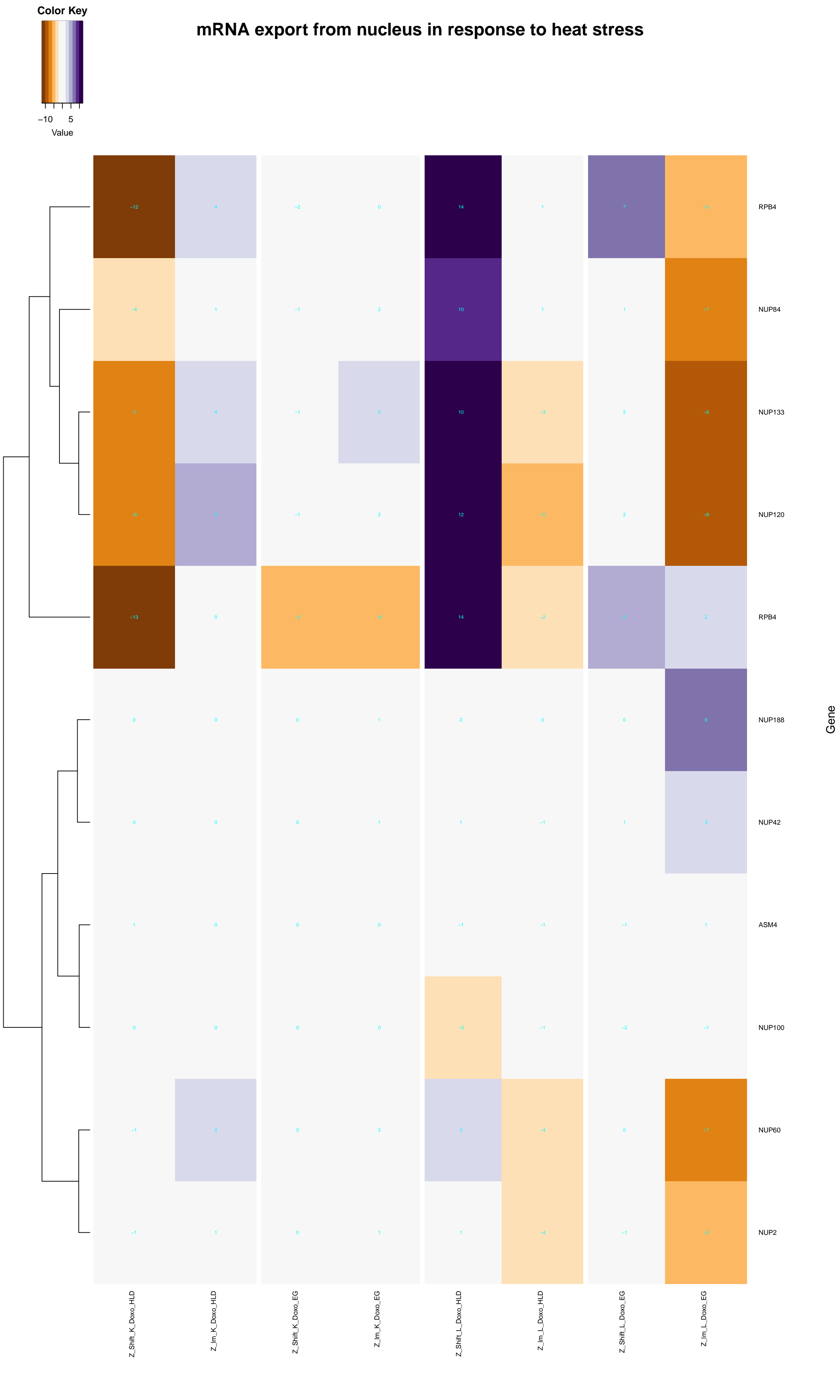

Supplement: Supplementary file 9 — Additional file 9. GO term-specific heatmaps for REMc/GTF-enriched clusters. GO term-specific heatmaps for significant GO process terms were generated as described in methods and Figs. 3 and 4. Any related child terms are presented in subsequent pages of the parent file name. GO terms with more than 100 children, with 2 or fewer genes annotated to the term, or a file size over 300KB are not shown. All heatmaps are generated with the same layout (see Figs. 3 and 4). [file 40170_2019_201_MOESM9_ESM.bz2 › Additional_File9_GOTermHeatmaps/Additional_File9_GOTermHeatmap/mRNA_transport.pdf]

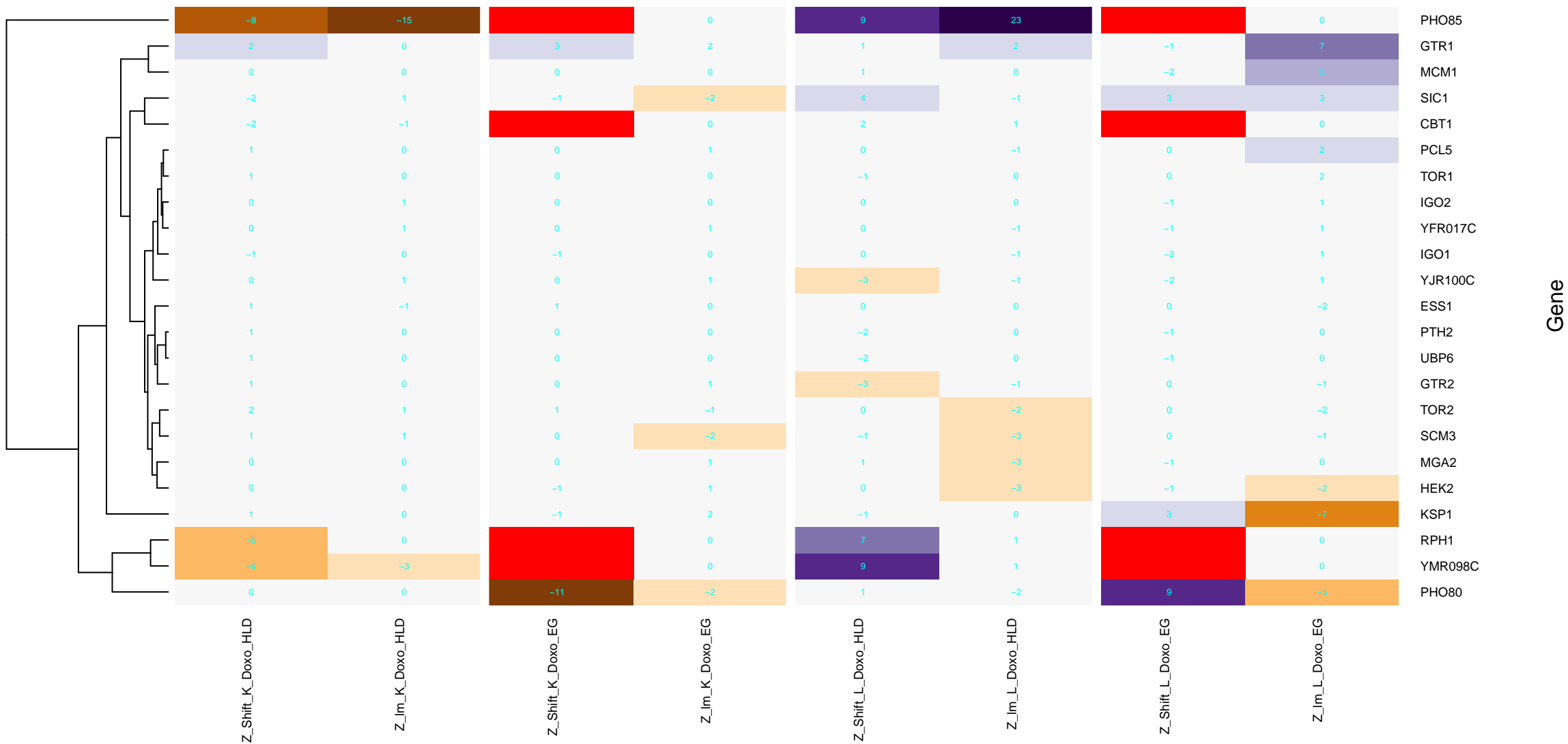

Supplement: Supplementary file 9 — Additional file 9. GO term-specific heatmaps for REMc/GTF-enriched clusters. GO term-specific heatmaps for significant GO process terms were generated as described in methods and Figs. 3 and 4. Any related child terms are presented in subsequent pages of the parent file name. GO terms with more than 100 children, with 2 or fewer genes annotated to the term, or a file size over 300KB are not shown. All heatmaps are generated with the same layout (see Figs. 3 and 4). [file 40170_2019_201_MOESM9_ESM.bz2 › Additional_File9_GOTermHeatmaps/Additional_File9_GOTermHeatmap/negative_regulation_of_cellular_catabolic_process.pdf]

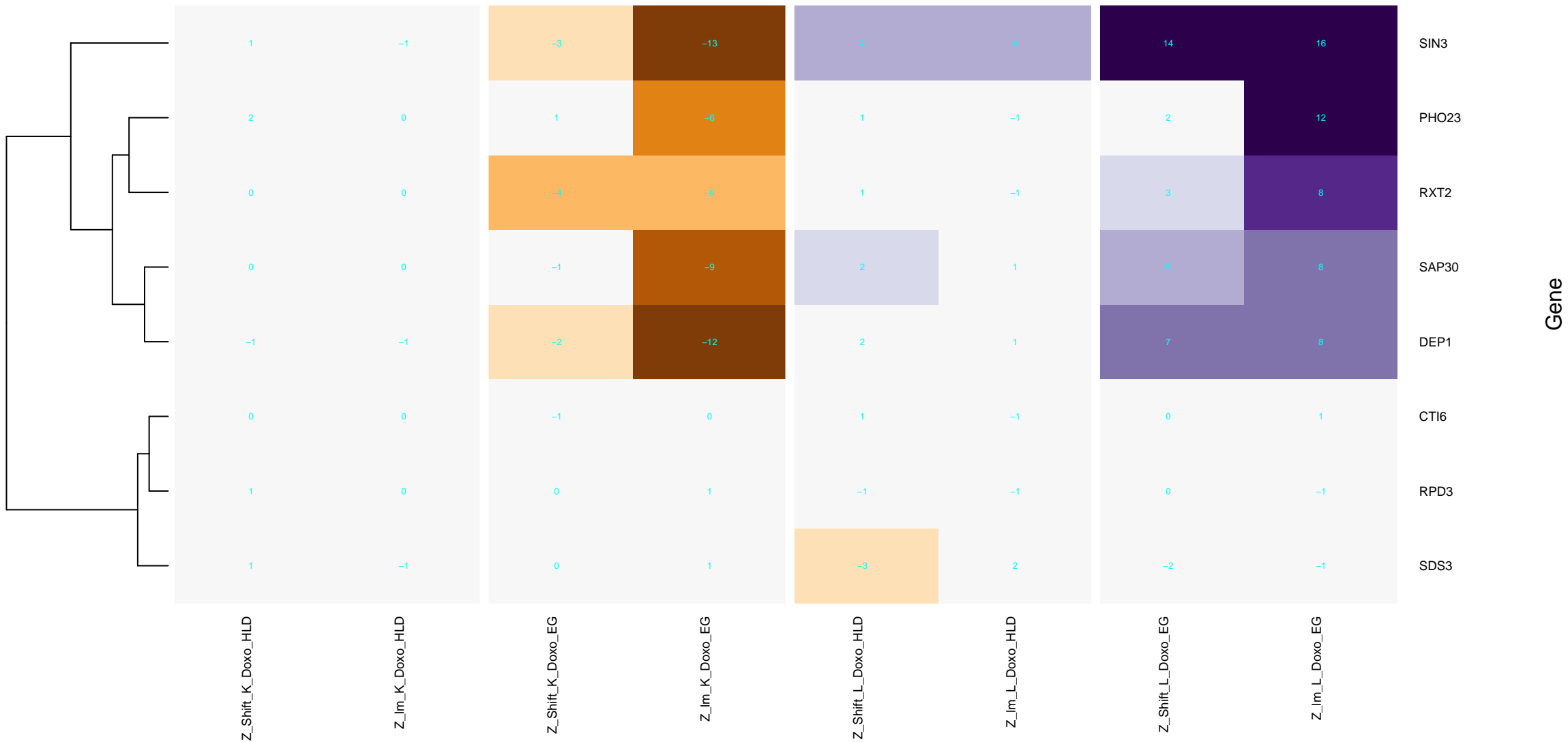

Supplement: Supplementary file 9 — Additional file 9. GO term-specific heatmaps for REMc/GTF-enriched clusters. GO term-specific heatmaps for significant GO process terms were generated as described in methods and Figs. 3 and 4. Any related child terms are presented in subsequent pages of the parent file name. GO terms with more than 100 children, with 2 or fewer genes annotated to the term, or a file size over 300KB are not shown. All heatmaps are generated with the same layout (see Figs. 3 and 4). [file 40170_2019_201_MOESM9_ESM.bz2 › Additional_File9_GOTermHeatmaps/Additional_File9_GOTermHeatmap/negative_regulation_of_chromatin_silencing_at_rDNA.pdf]

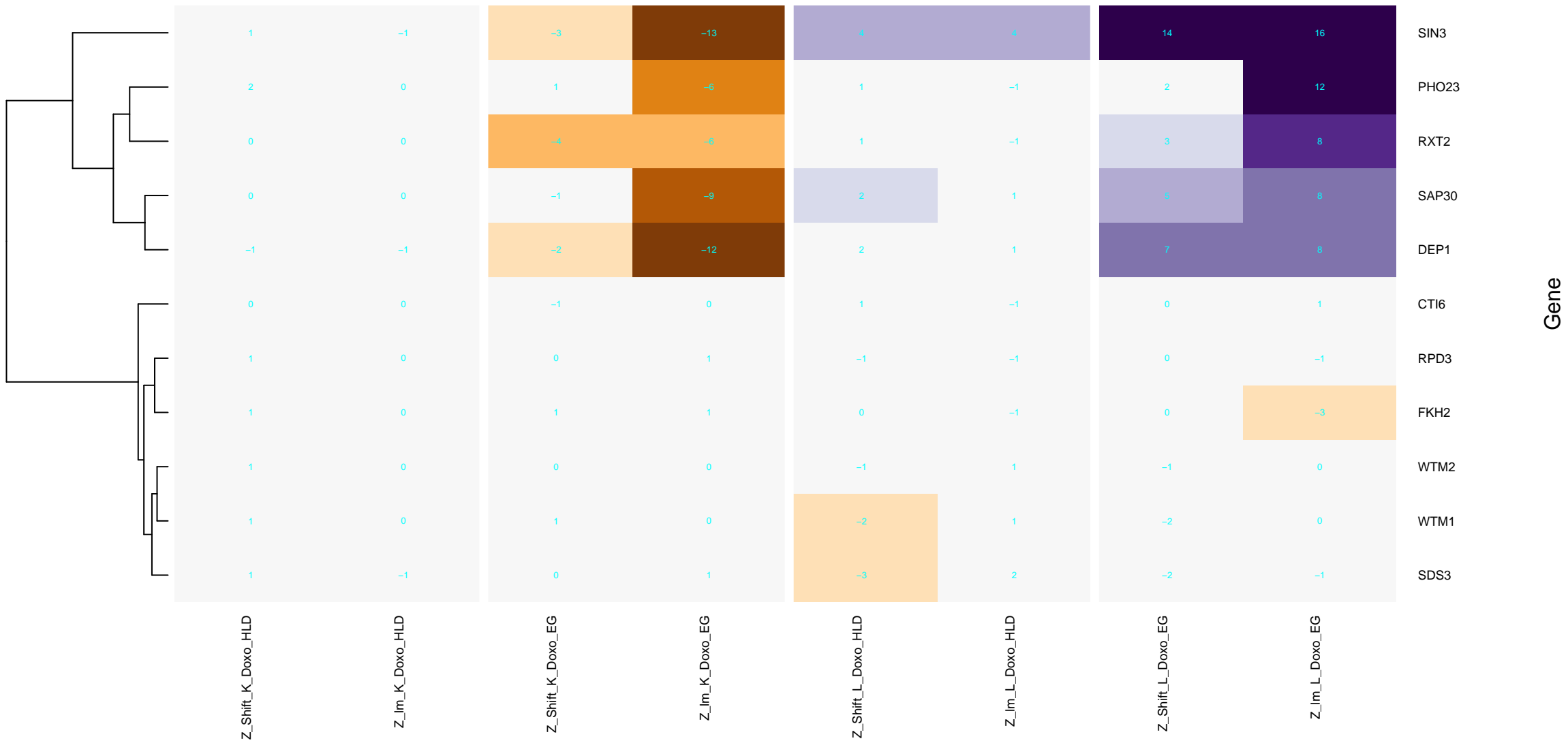

Supplement: Supplementary file 9 — Additional file 9. GO term-specific heatmaps for REMc/GTF-enriched clusters. GO term-specific heatmaps for significant GO process terms were generated as described in methods and Figs. 3 and 4. Any related child terms are presented in subsequent pages of the parent file name. GO terms with more than 100 children, with 2 or fewer genes annotated to the term, or a file size over 300KB are not shown. All heatmaps are generated with the same layout (see Figs. 3 and 4). [file 40170_2019_201_MOESM9_ESM.bz2 › Additional_File9_GOTermHeatmaps/Additional_File9_GOTermHeatmap/negative_regulation_of_chromatin_silencing_at_silent_mating-type_cassette.pdf]

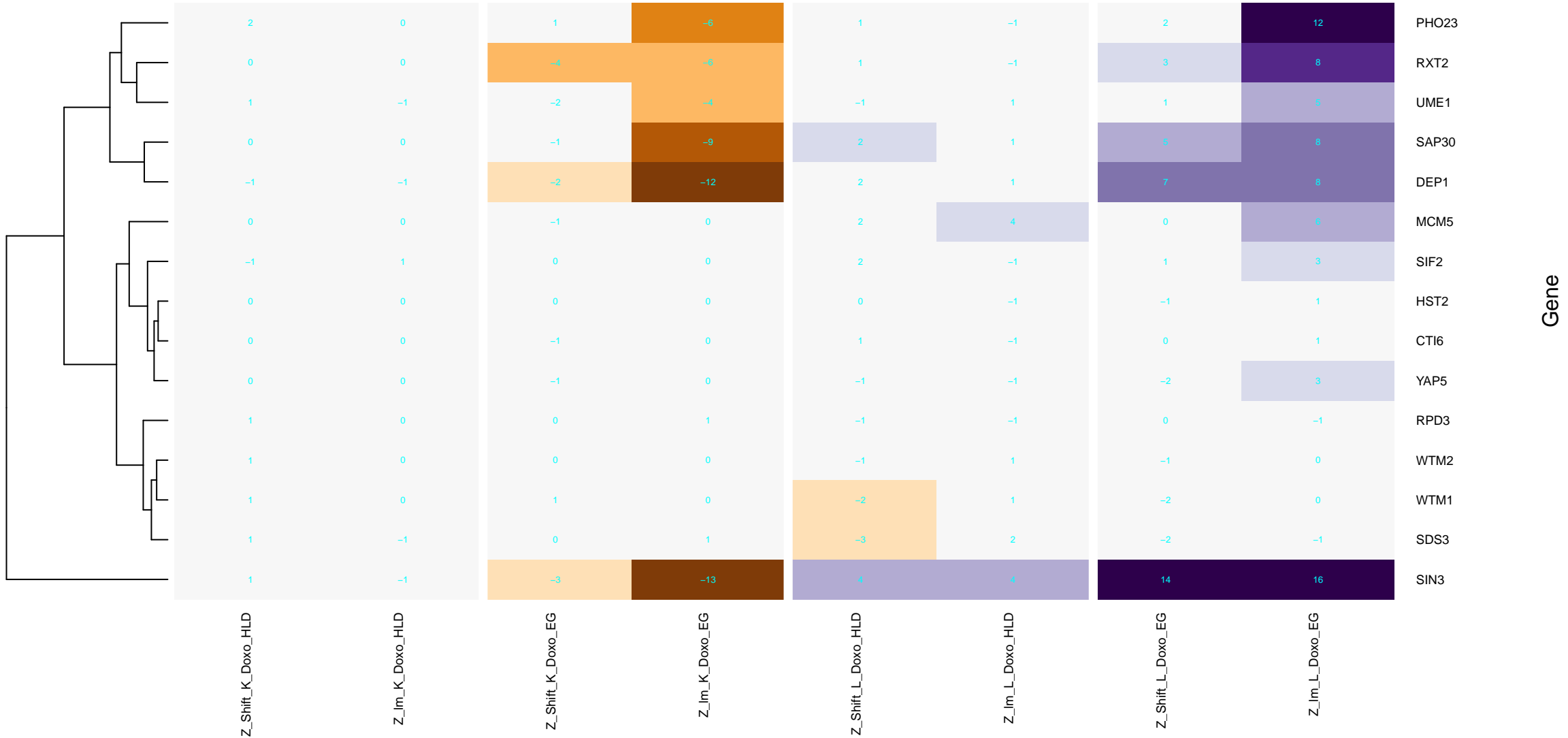

Supplement: Supplementary file 9 — Additional file 9. GO term-specific heatmaps for REMc/GTF-enriched clusters. GO term-specific heatmaps for significant GO process terms were generated as described in methods and Figs. 3 and 4. Any related child terms are presented in subsequent pages of the parent file name. GO terms with more than 100 children, with 2 or fewer genes annotated to the term, or a file size over 300KB are not shown. All heatmaps are generated with the same layout (see Figs. 3 and 4). [file 40170_2019_201_MOESM9_ESM.bz2 › Additional_File9_GOTermHeatmaps/Additional_File9_GOTermHeatmap/negative_regulation_of_chromatin_silencing_at_telomere.pdf]

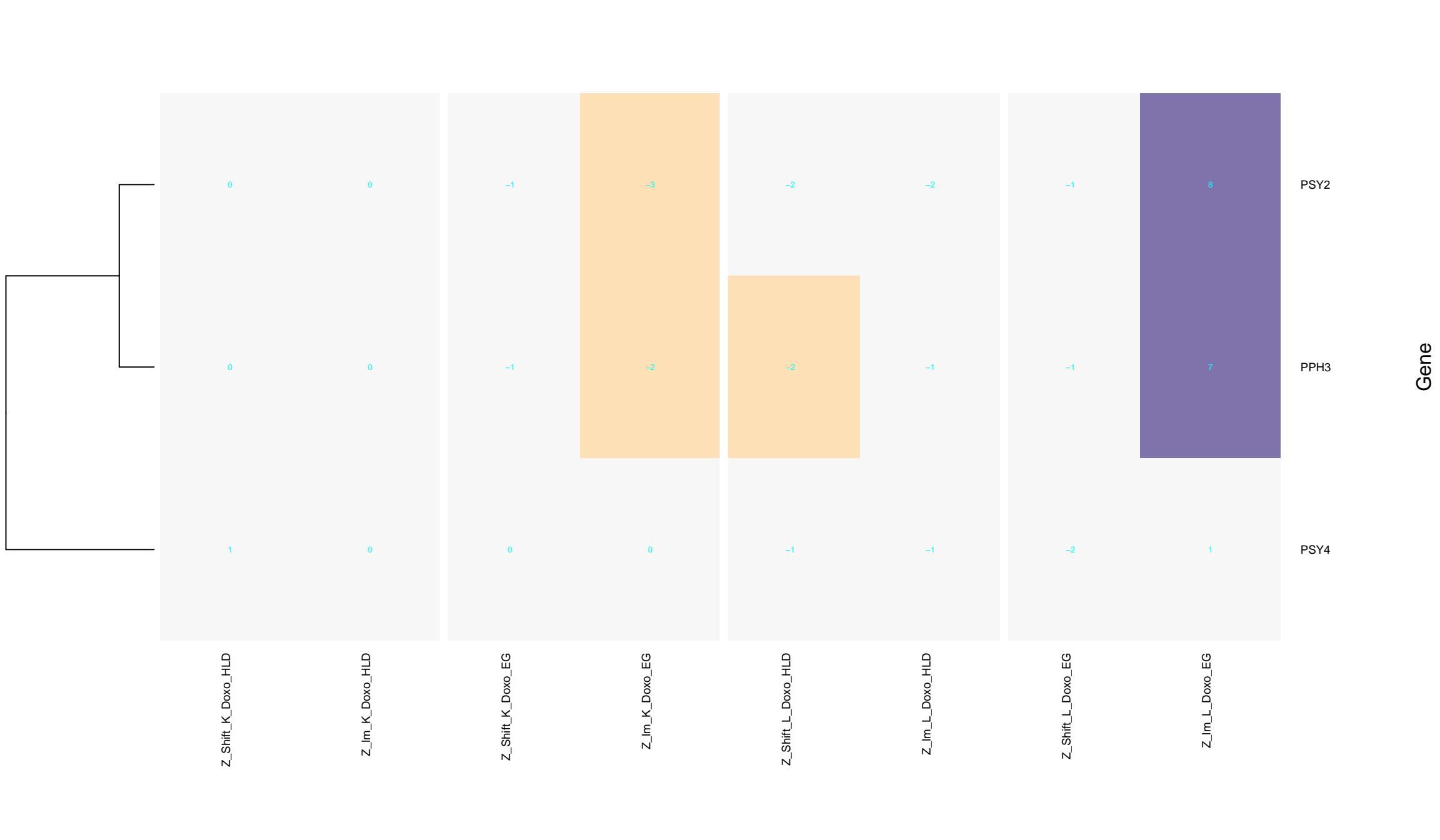

Supplement: Supplementary file 9 — Additional file 9. GO term-specific heatmaps for REMc/GTF-enriched clusters. GO term-specific heatmaps for significant GO process terms were generated as described in methods and Figs. 3 and 4. Any related child terms are presented in subsequent pages of the parent file name. GO terms with more than 100 children, with 2 or fewer genes annotated to the term, or a file size over 300KB are not shown. All heatmaps are generated with the same layout (see Figs. 3 and 4). [file 40170_2019_201_MOESM9_ESM.bz2 › Additional_File9_GOTermHeatmaps/Additional_File9_GOTermHeatmap/negative_regulation_of_DNA_damage_checkpoint.pdf]

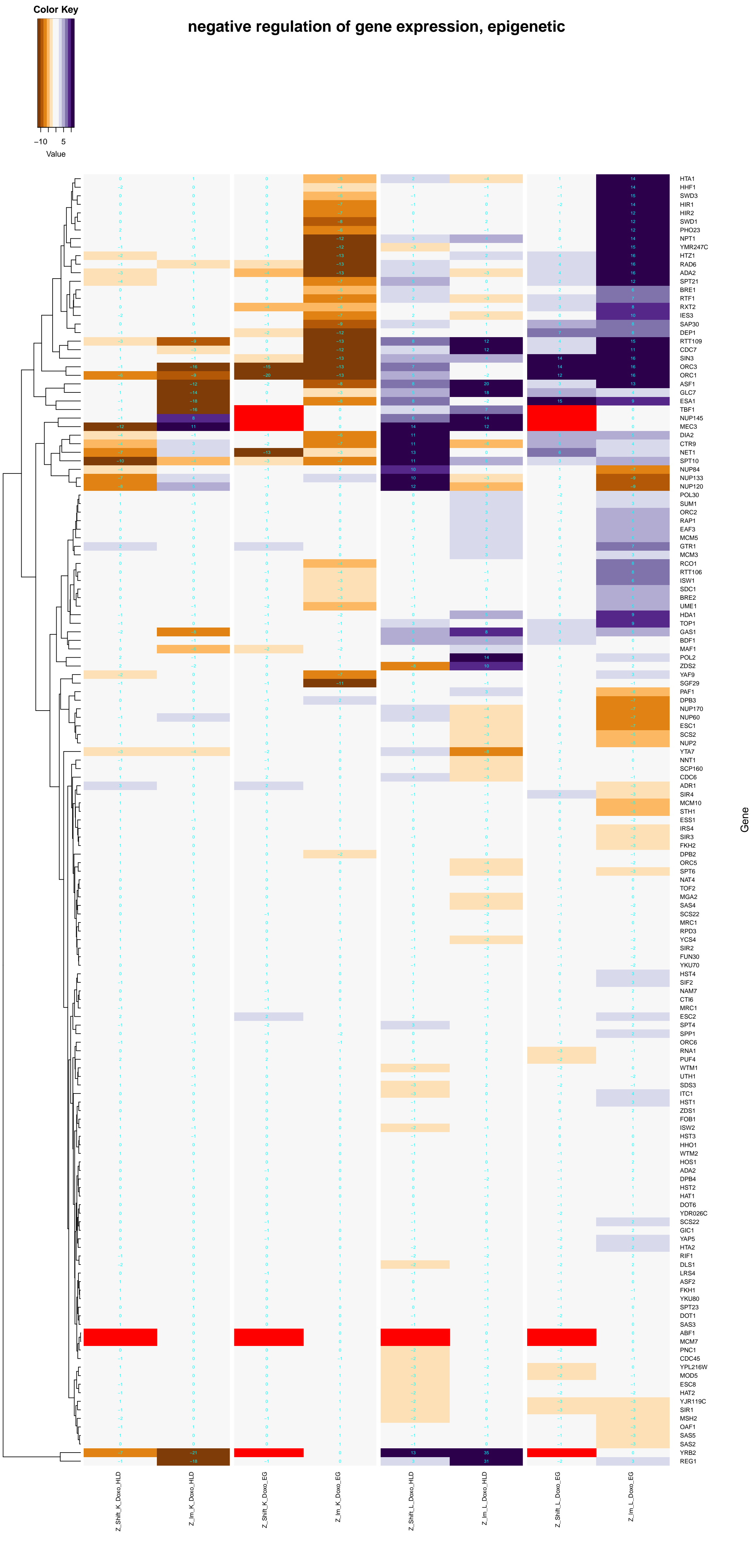

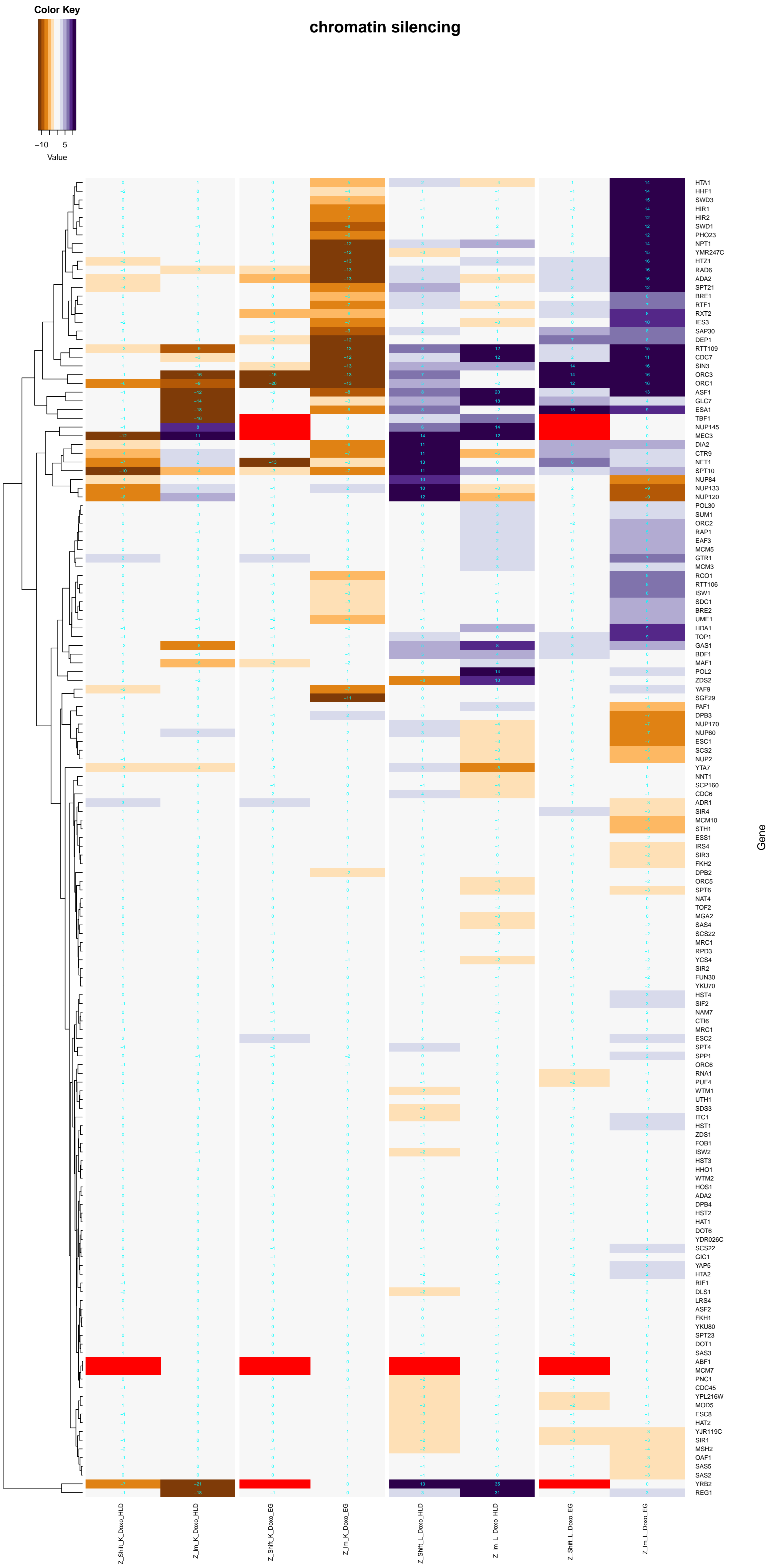

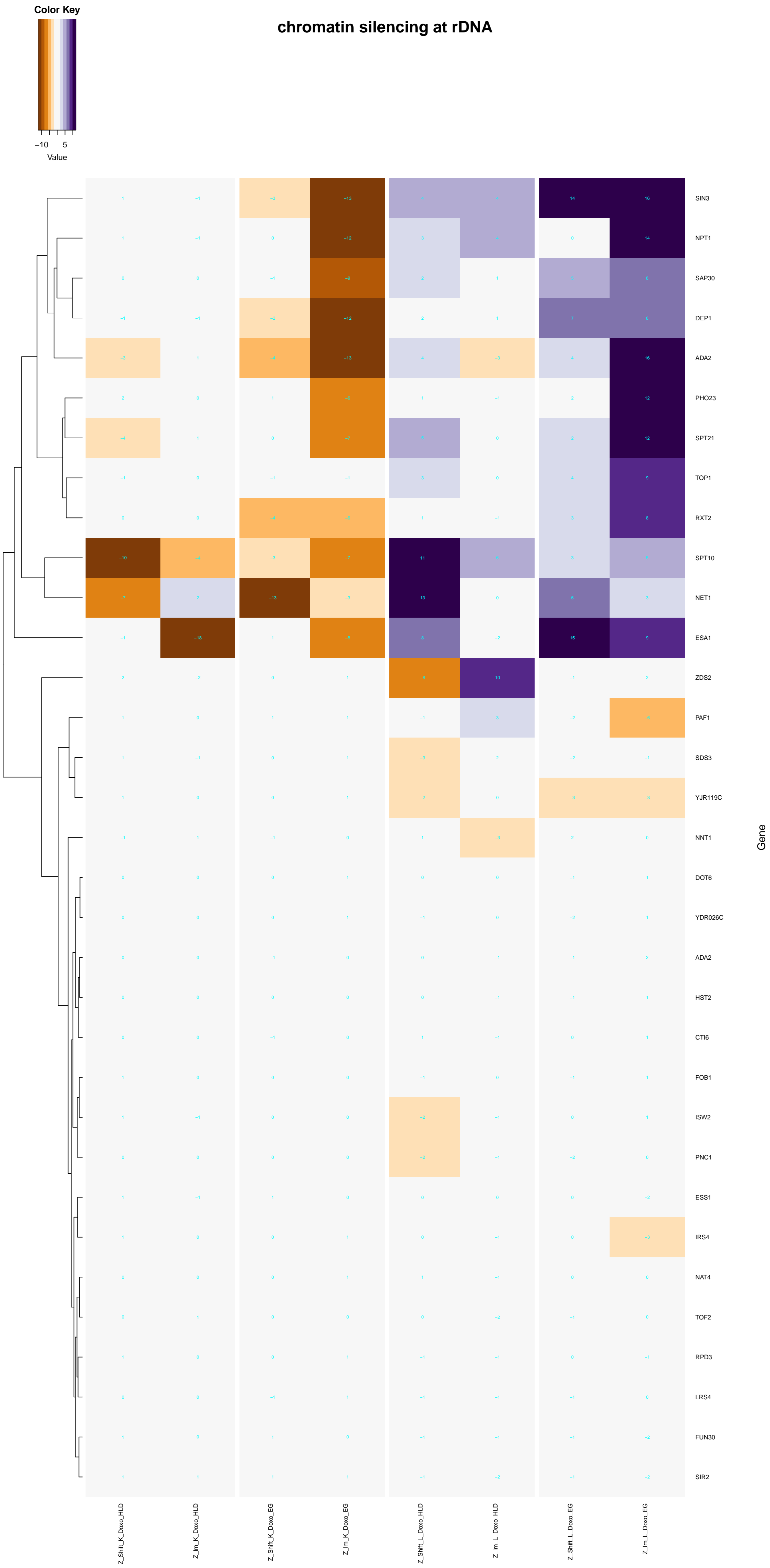



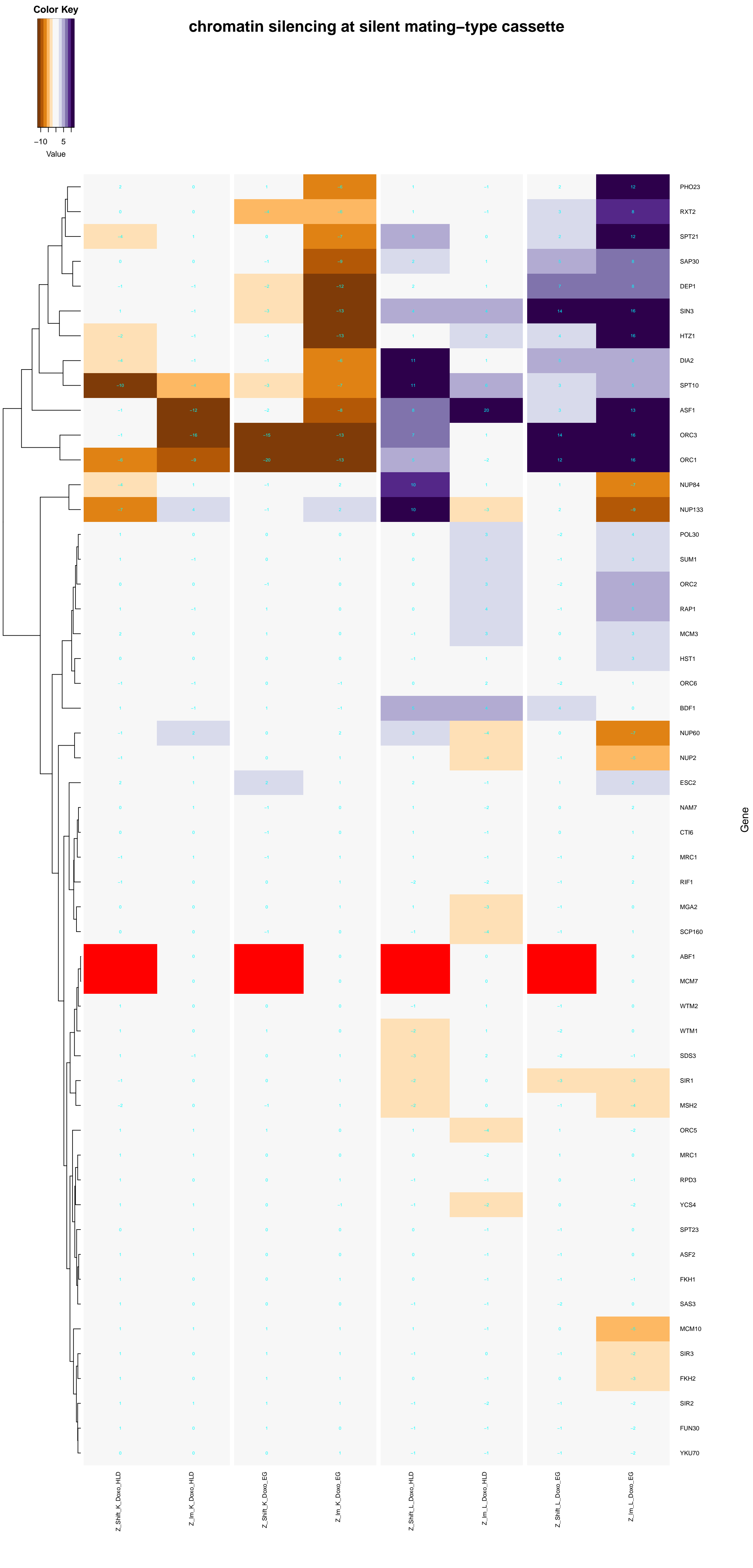

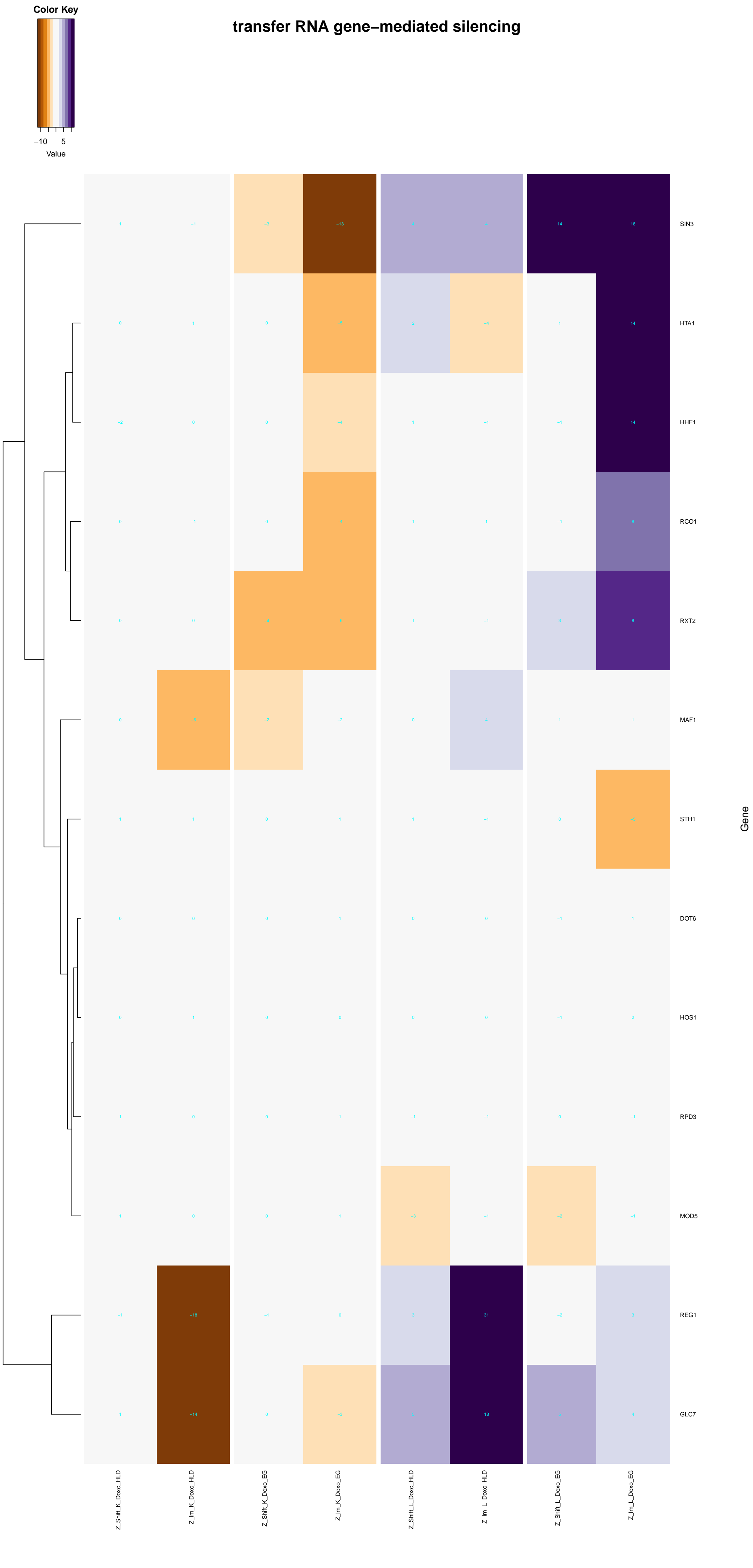

Supplement: Supplementary file 9 — Additional file 9. GO term-specific heatmaps for REMc/GTF-enriched clusters. GO term-specific heatmaps for significant GO process terms were generated as described in methods and Figs. 3 and 4. Any related child terms are presented in subsequent pages of the parent file name. GO terms with more than 100 children, with 2 or fewer genes annotated to the term, or a file size over 300KB are not shown. All heatmaps are generated with the same layout (see Figs. 3 and 4). [file 40170_2019_201_MOESM9_ESM.bz2 › Additional_File9_GOTermHeatmaps/Additional_File9_GOTermHeatmap/negative_regulation_of_gene_expression,_epigenetic.pdf]

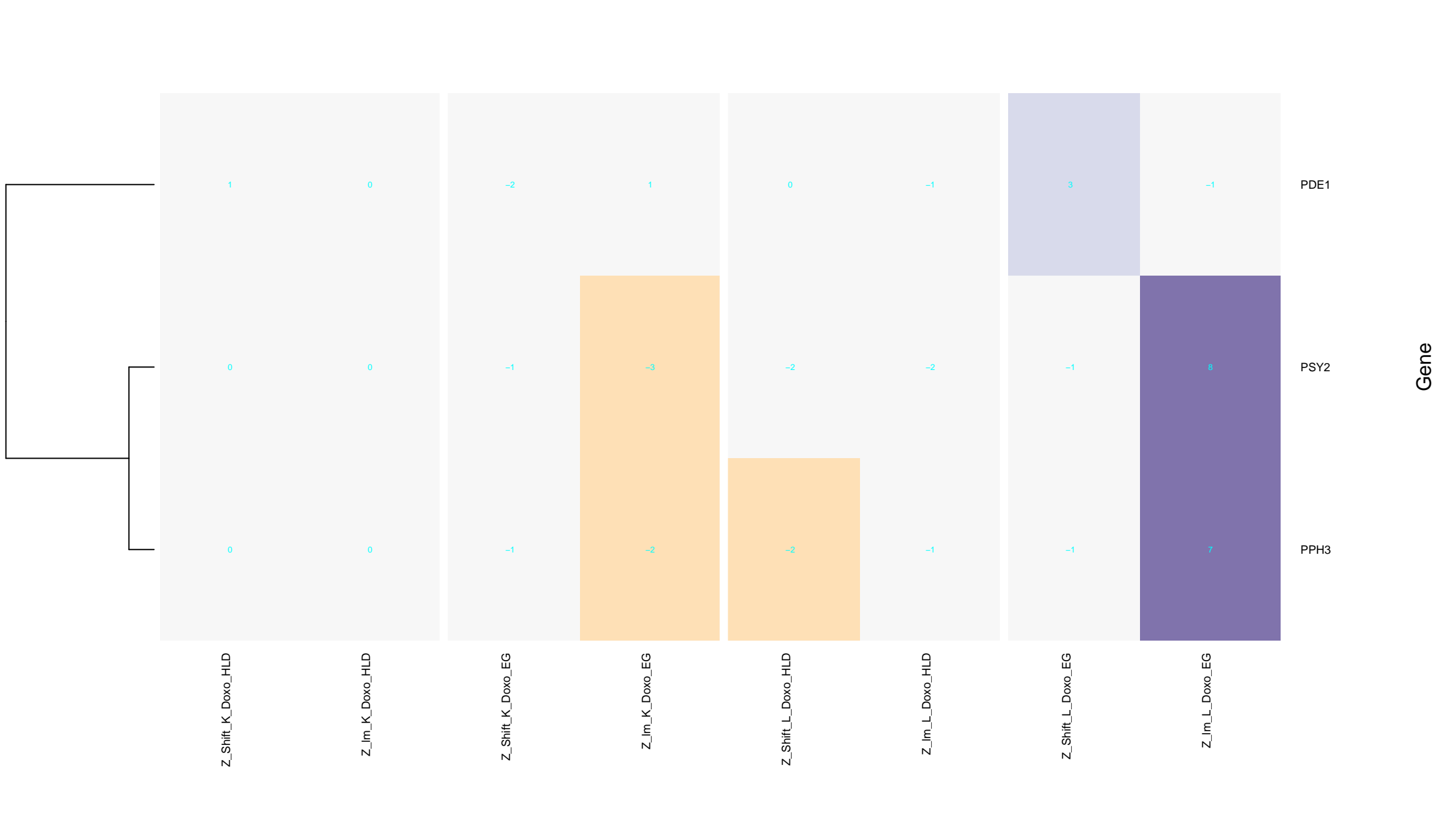

Supplement: Supplementary file 9 — Additional file 9. GO term-specific heatmaps for REMc/GTF-enriched clusters. GO term-specific heatmaps for significant GO process terms were generated as described in methods and Figs. 3 and 4. Any related child terms are presented in subsequent pages of the parent file name. GO terms with more than 100 children, with 2 or fewer genes annotated to the term, or a file size over 300KB are not shown. All heatmaps are generated with the same layout (see Figs. 3 and 4). [file 40170_2019_201_MOESM9_ESM.bz2 › Additional_File9_GOTermHeatmaps/Additional_File9_GOTermHeatmap/negative_regulation_of_glucose_mediated_signaling_pathway.pdf]

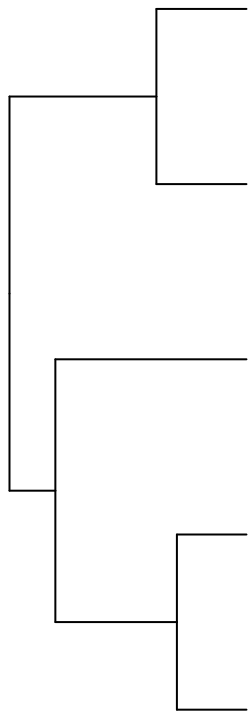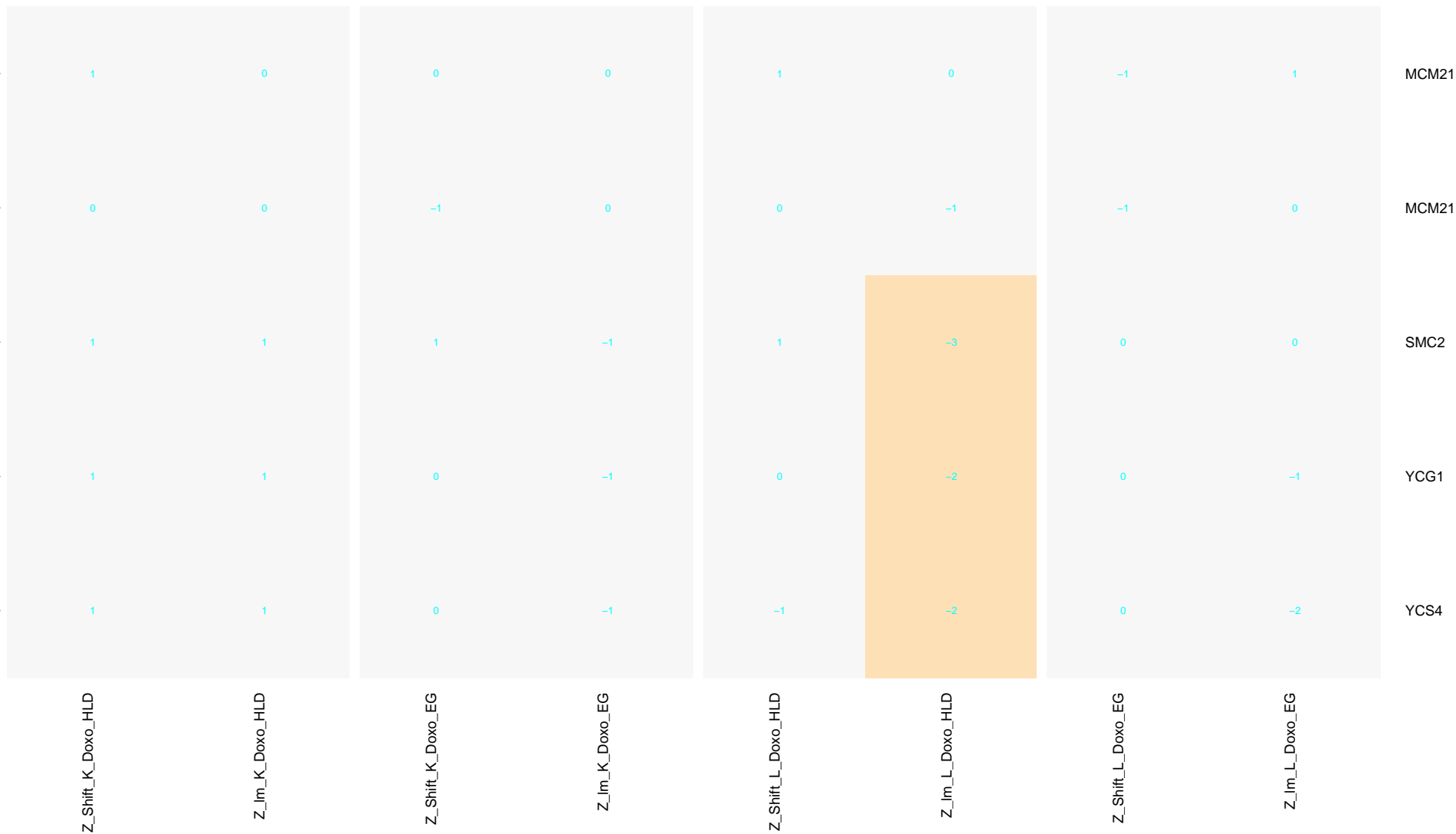

Supplement: Supplementary file 9 — Additional file 9. GO term-specific heatmaps for REMc/GTF-enriched clusters. GO term-specific heatmaps for significant GO process terms were generated as described in methods and Figs. 3 and 4. Any related child terms are presented in subsequent pages of the parent file name. GO terms with more than 100 children, with 2 or fewer genes annotated to the term, or a file size over 300KB are not shown. All heatmaps are generated with the same layout (see Figs. 3 and 4). [file 40170_2019_201_MOESM9_ESM.bz2 › Additional_File9_GOTermHeatmaps/Additional_File9_GOTermHeatmap/negative_regulation_of_meiotic_DNA_double-strand_break_formation.pdf]

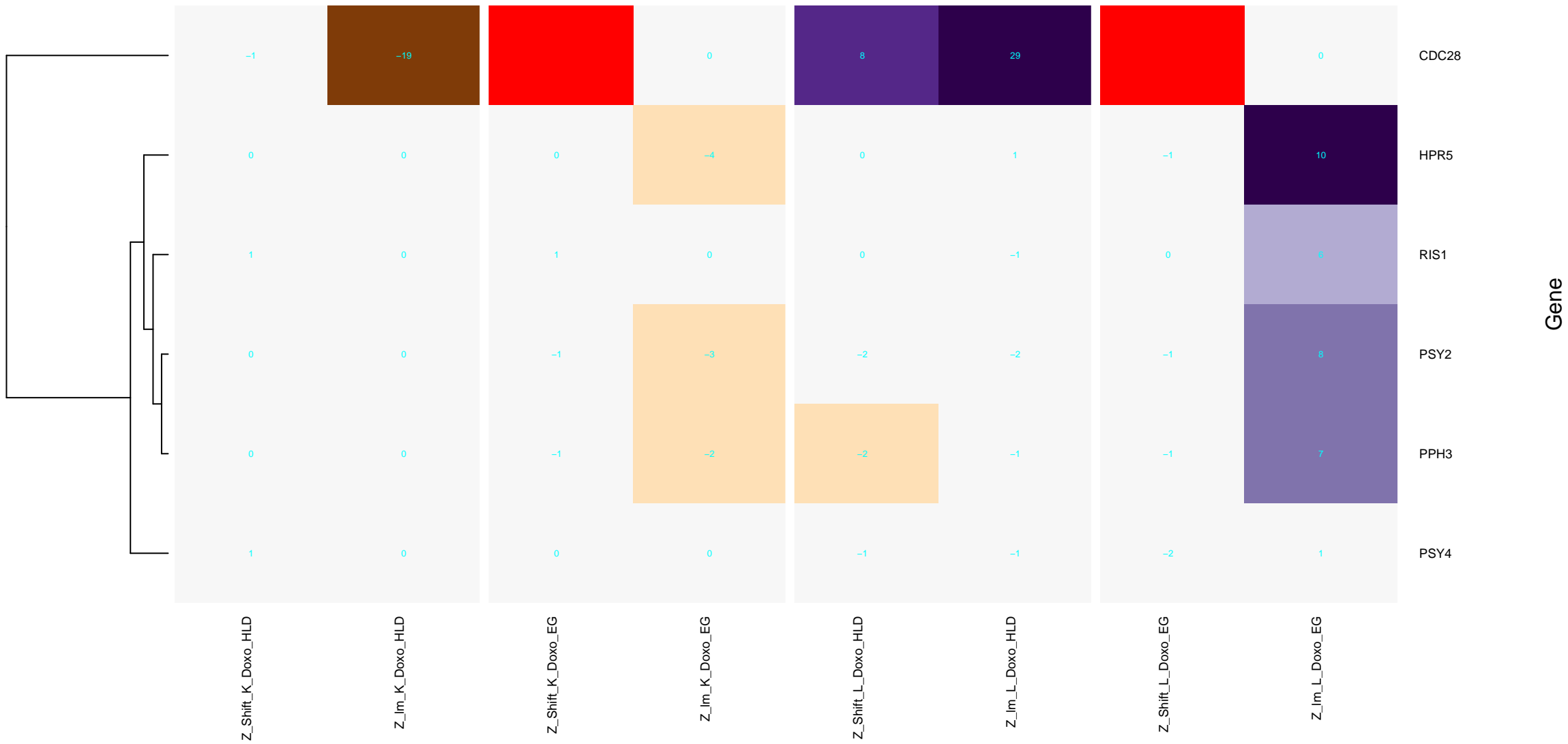

Supplement: Supplementary file 9 — Additional file 9. GO term-specific heatmaps for REMc/GTF-enriched clusters. GO term-specific heatmaps for significant GO process terms were generated as described in methods and Figs. 3 and 4. Any related child terms are presented in subsequent pages of the parent file name. GO terms with more than 100 children, with 2 or fewer genes annotated to the term, or a file size over 300KB are not shown. All heatmaps are generated with the same layout (see Figs. 3 and 4). [file 40170_2019_201_MOESM9_ESM.bz2 › Additional_File9_GOTermHeatmaps/Additional_File9_GOTermHeatmap/negative_regulation_of_response_to_DNA_damage_stimulus.pdf]

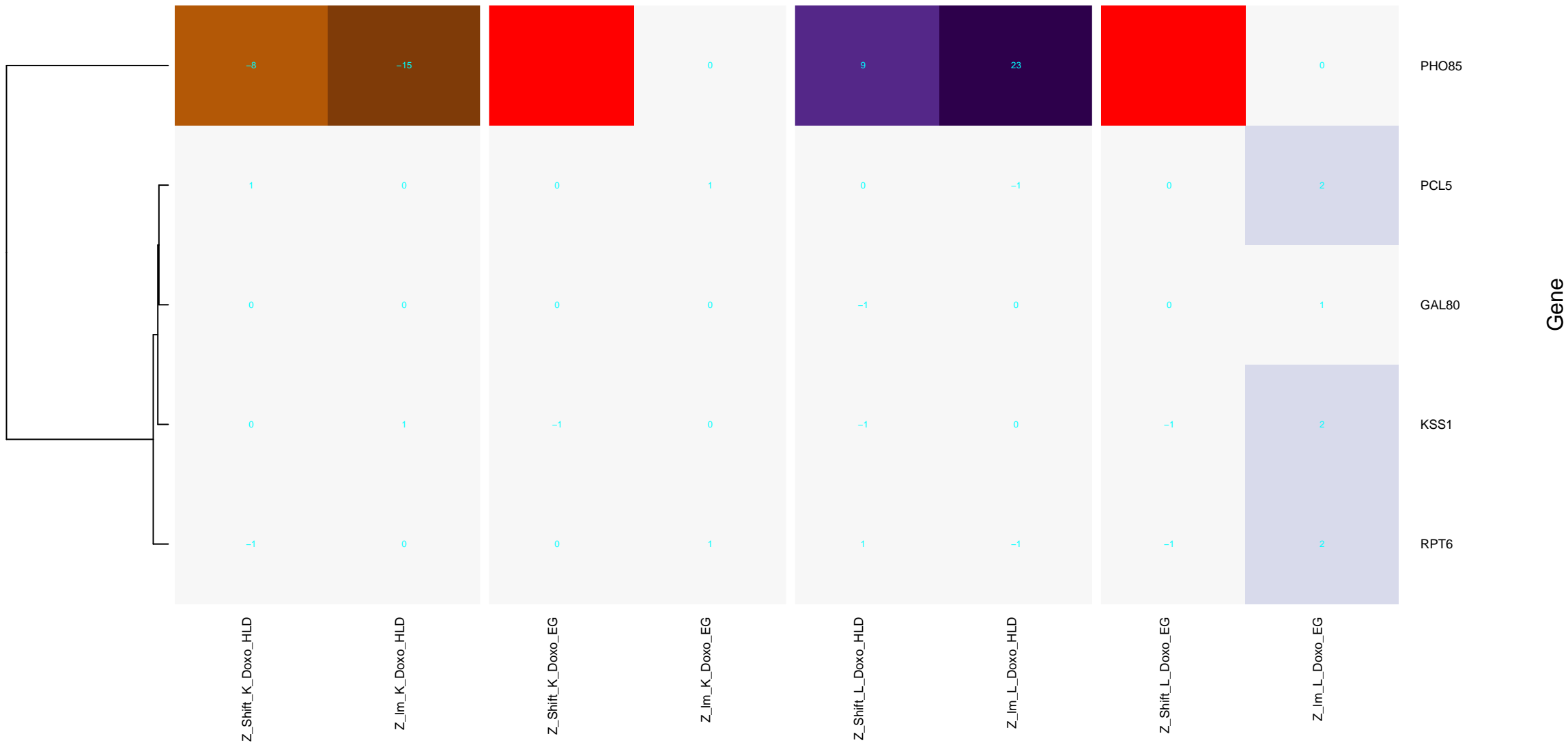

Supplement: Supplementary file 9 — Additional file 9. GO term-specific heatmaps for REMc/GTF-enriched clusters. GO term-specific heatmaps for significant GO process terms were generated as described in methods and Figs. 3 and 4. Any related child terms are presented in subsequent pages of the parent file name. GO terms with more than 100 children, with 2 or fewer genes annotated to the term, or a file size over 300KB are not shown. All heatmaps are generated with the same layout (see Figs. 3 and 4). [file 40170_2019_201_MOESM9_ESM.bz2 › Additional_File9_GOTermHeatmaps/Additional_File9_GOTermHeatmap/negative_regulation_of_sequence-specific_DNA_binding_transcription_factor_activity.pdf]

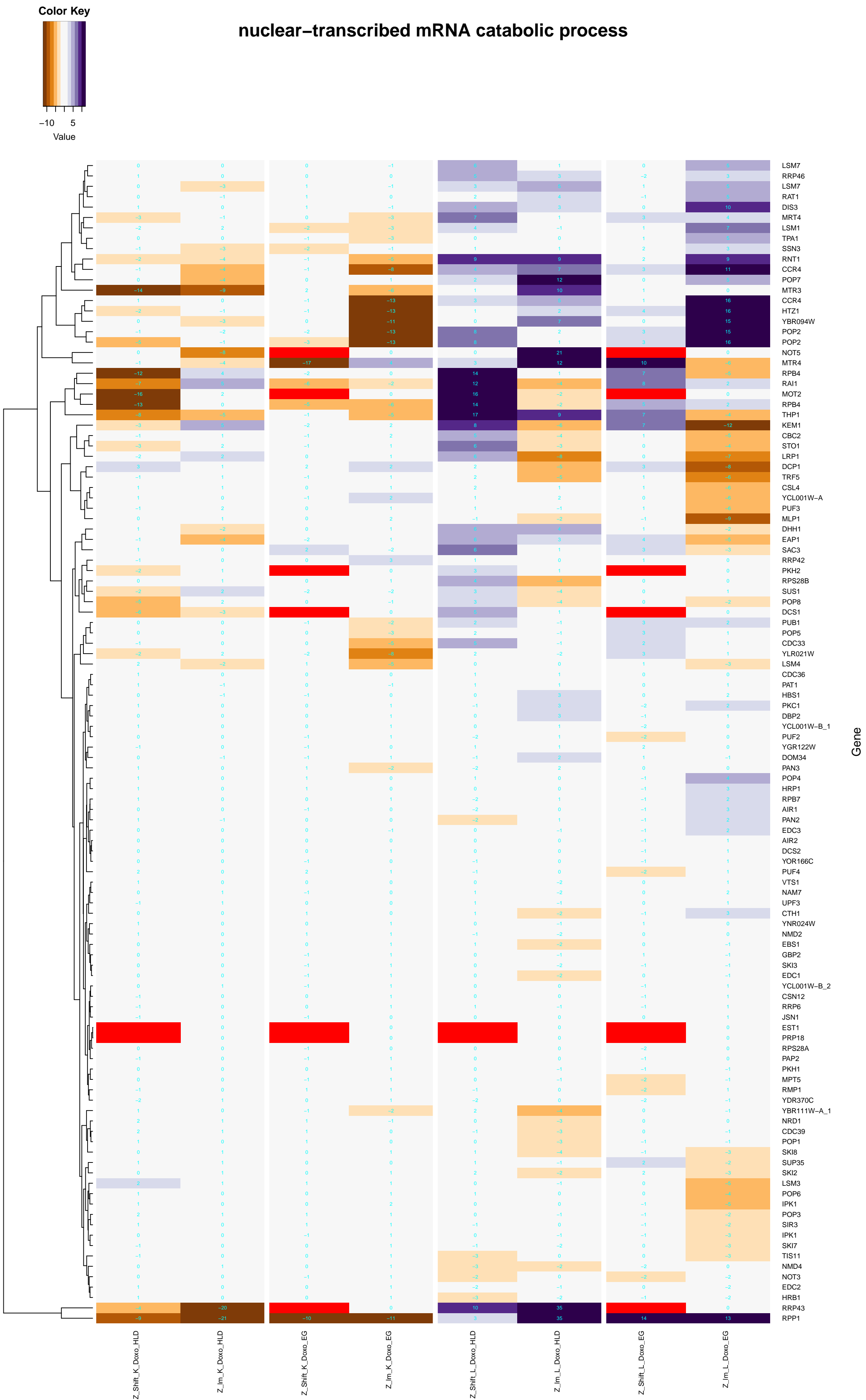

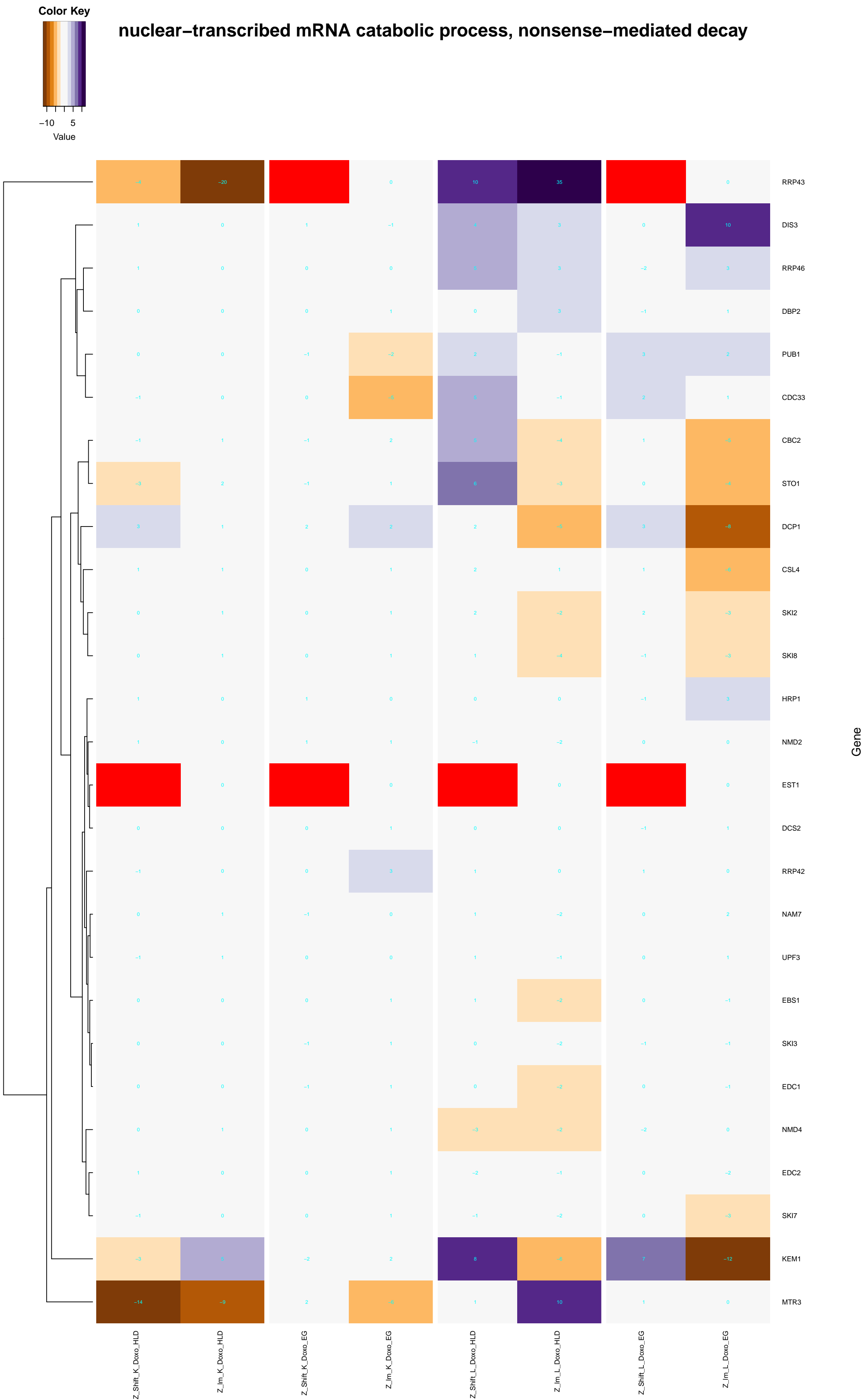

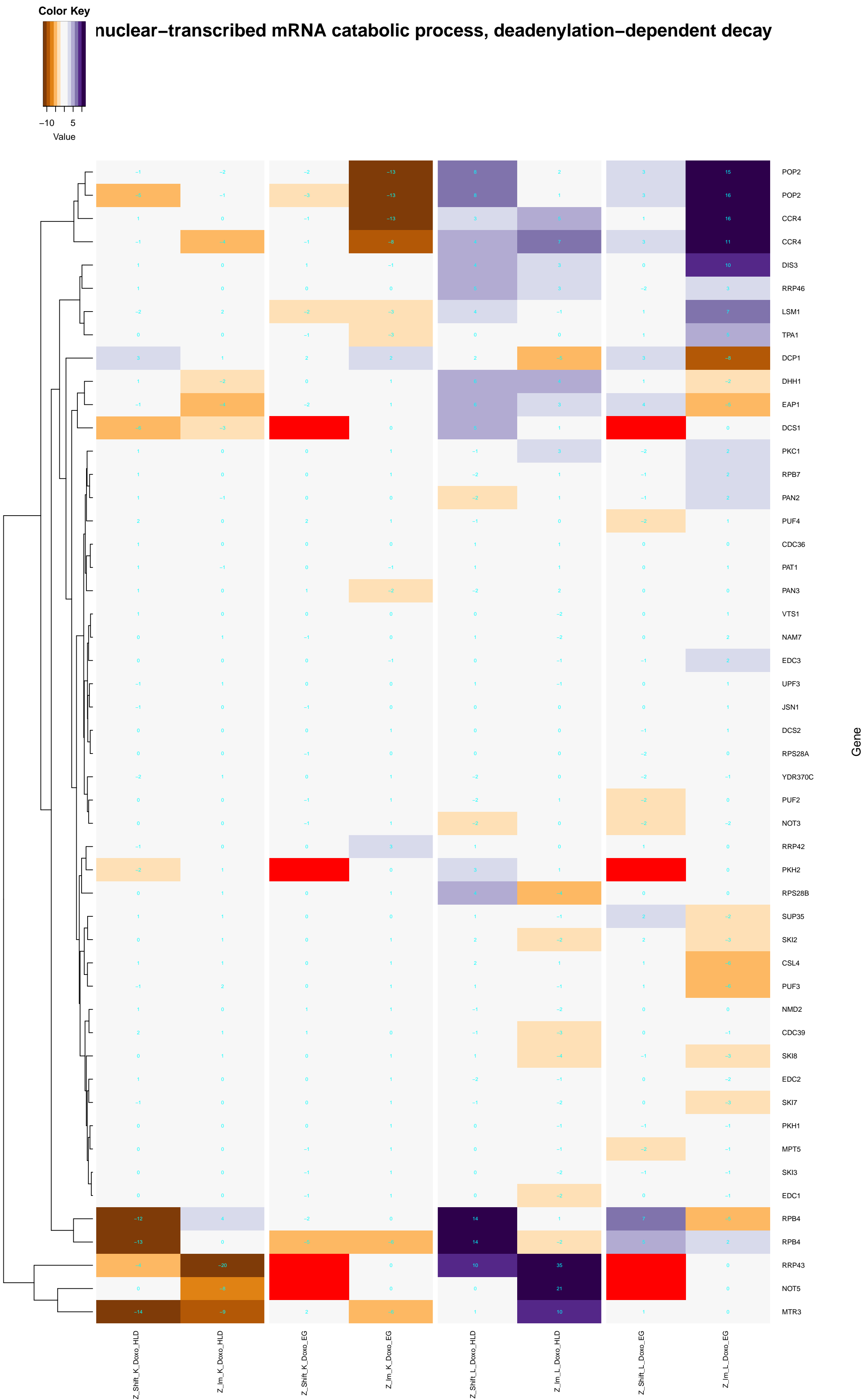

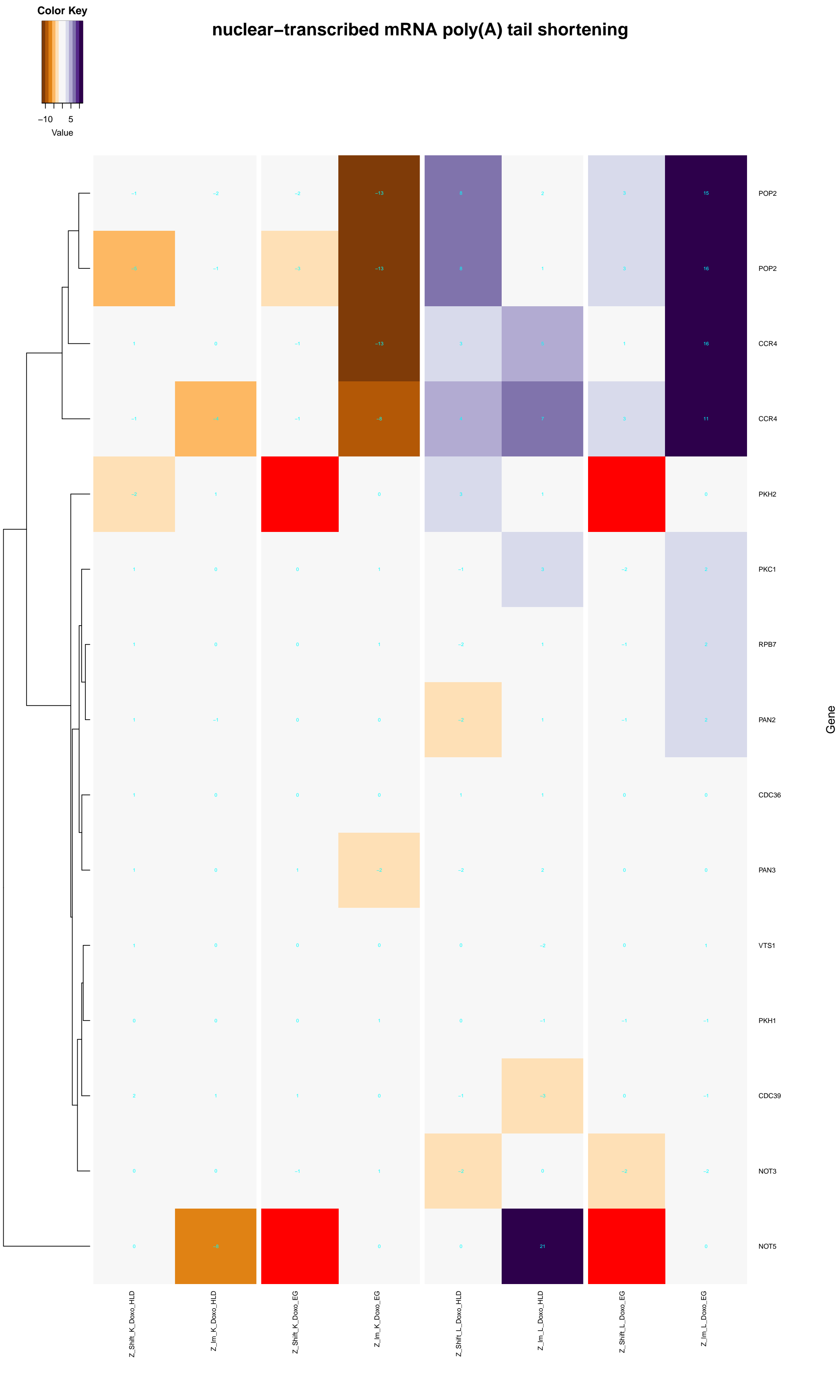

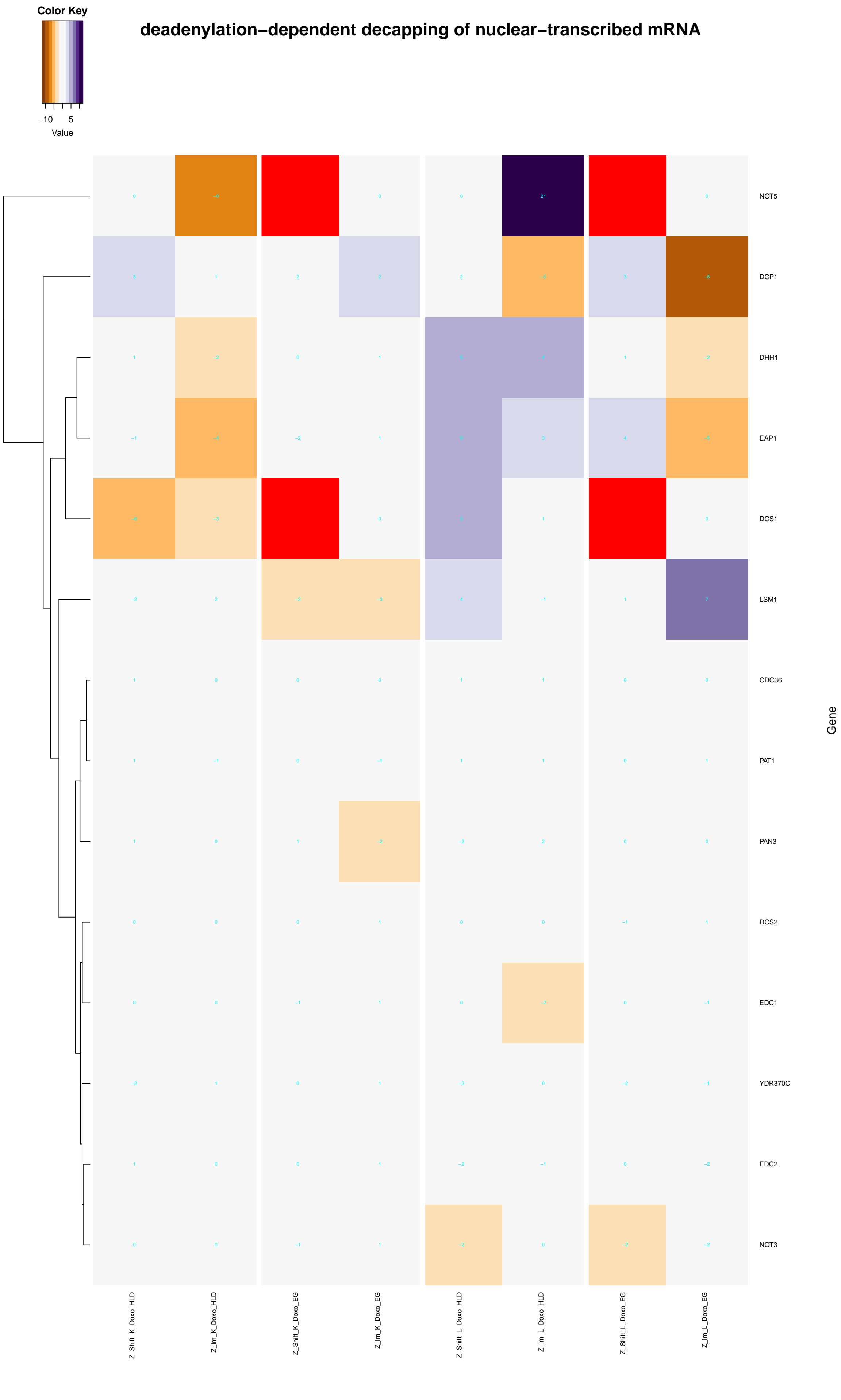

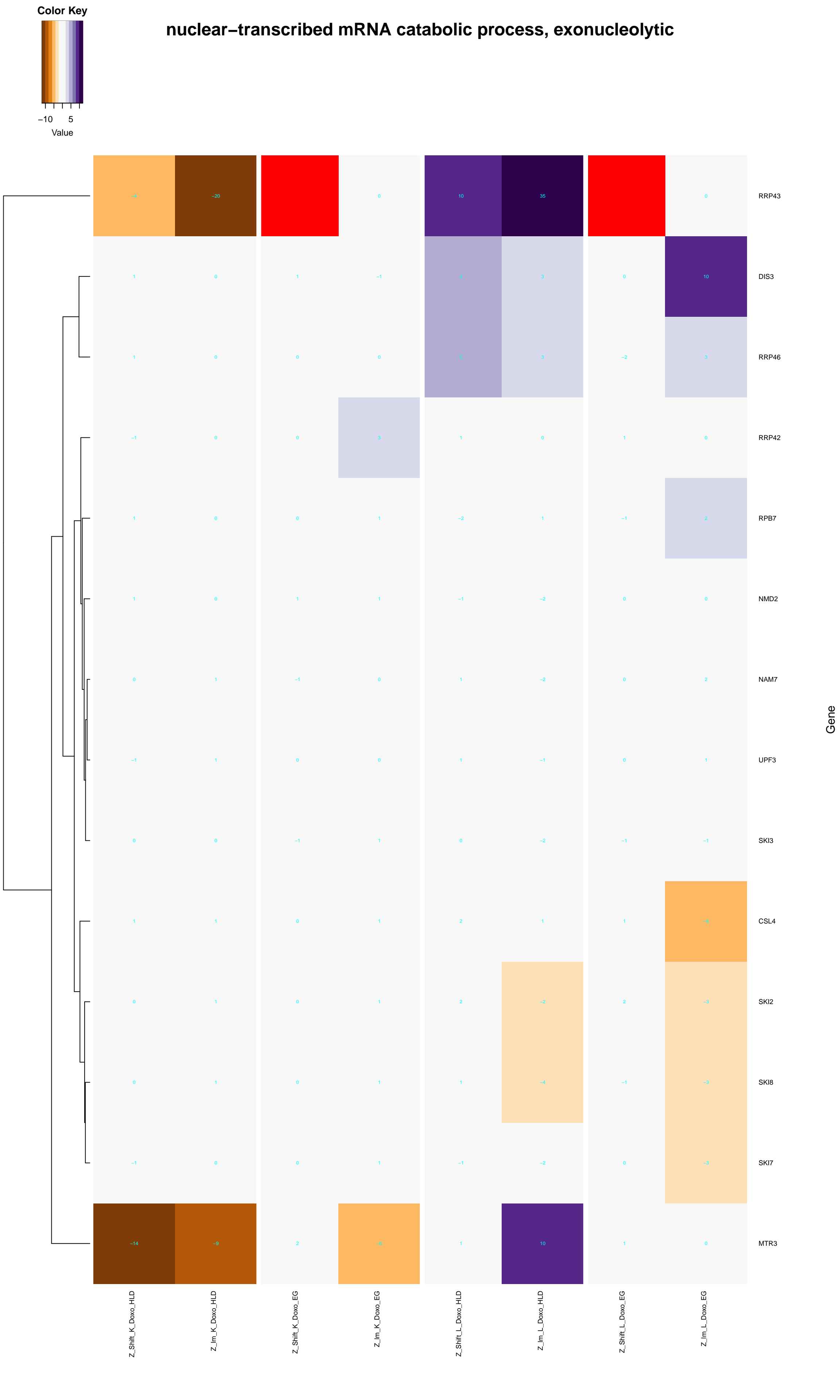

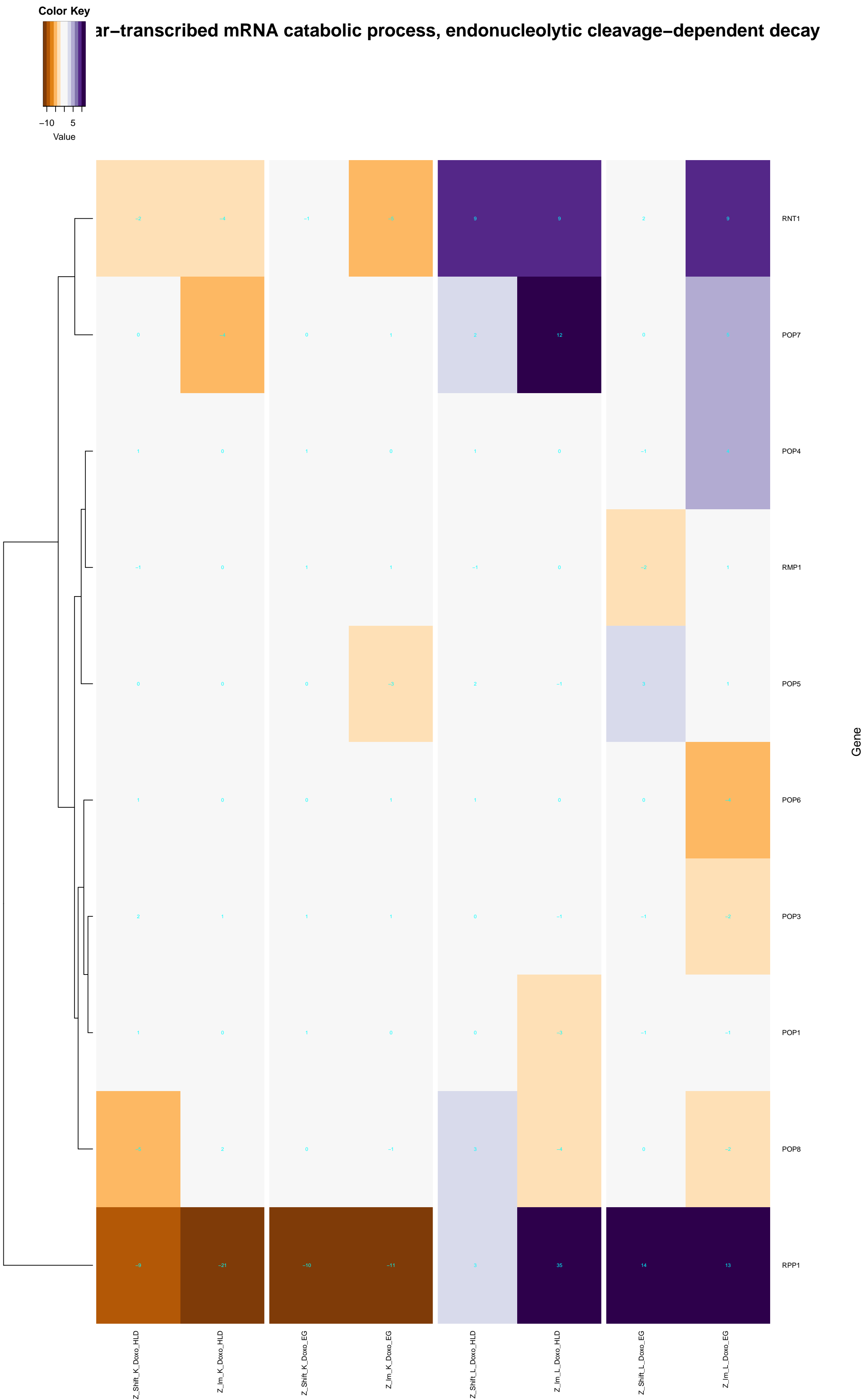

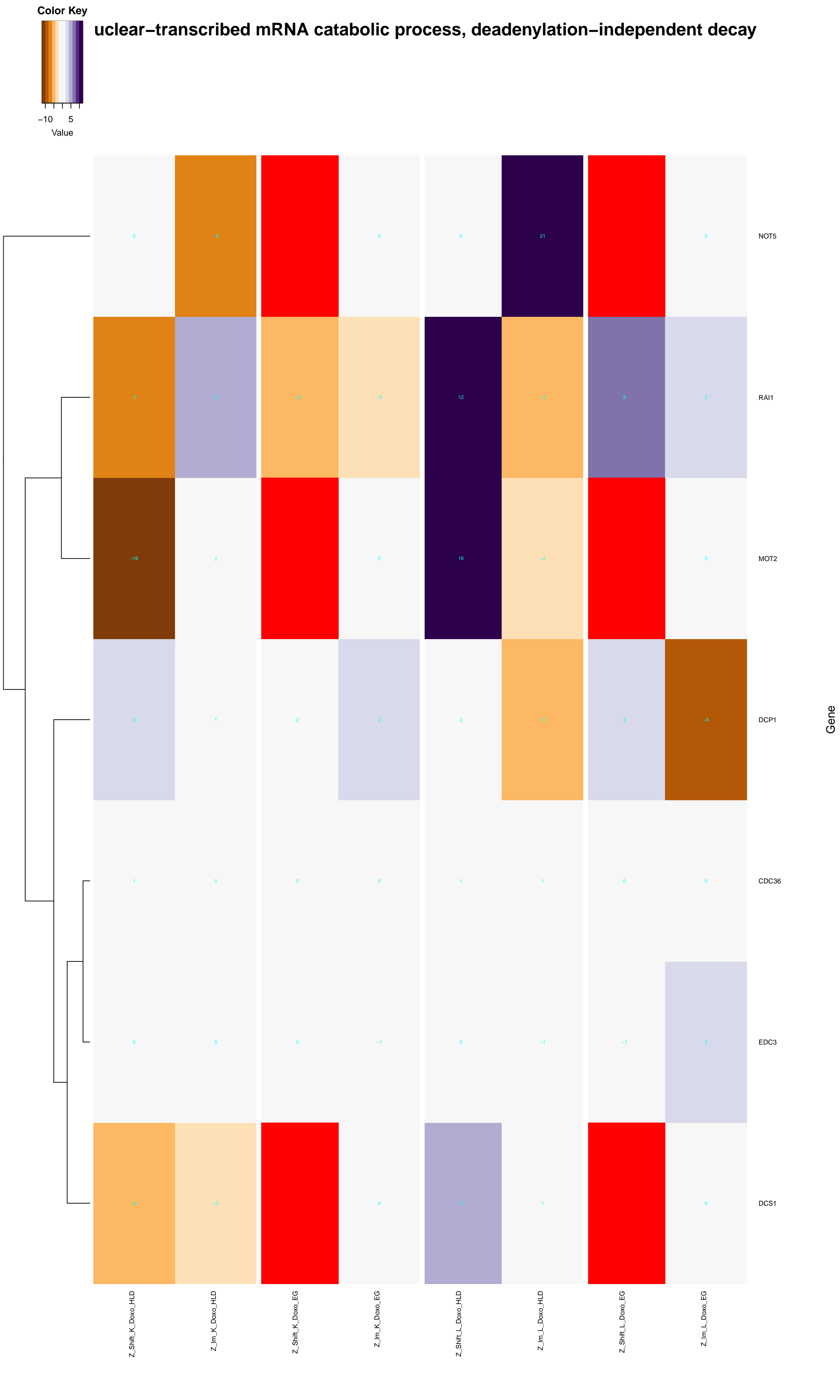

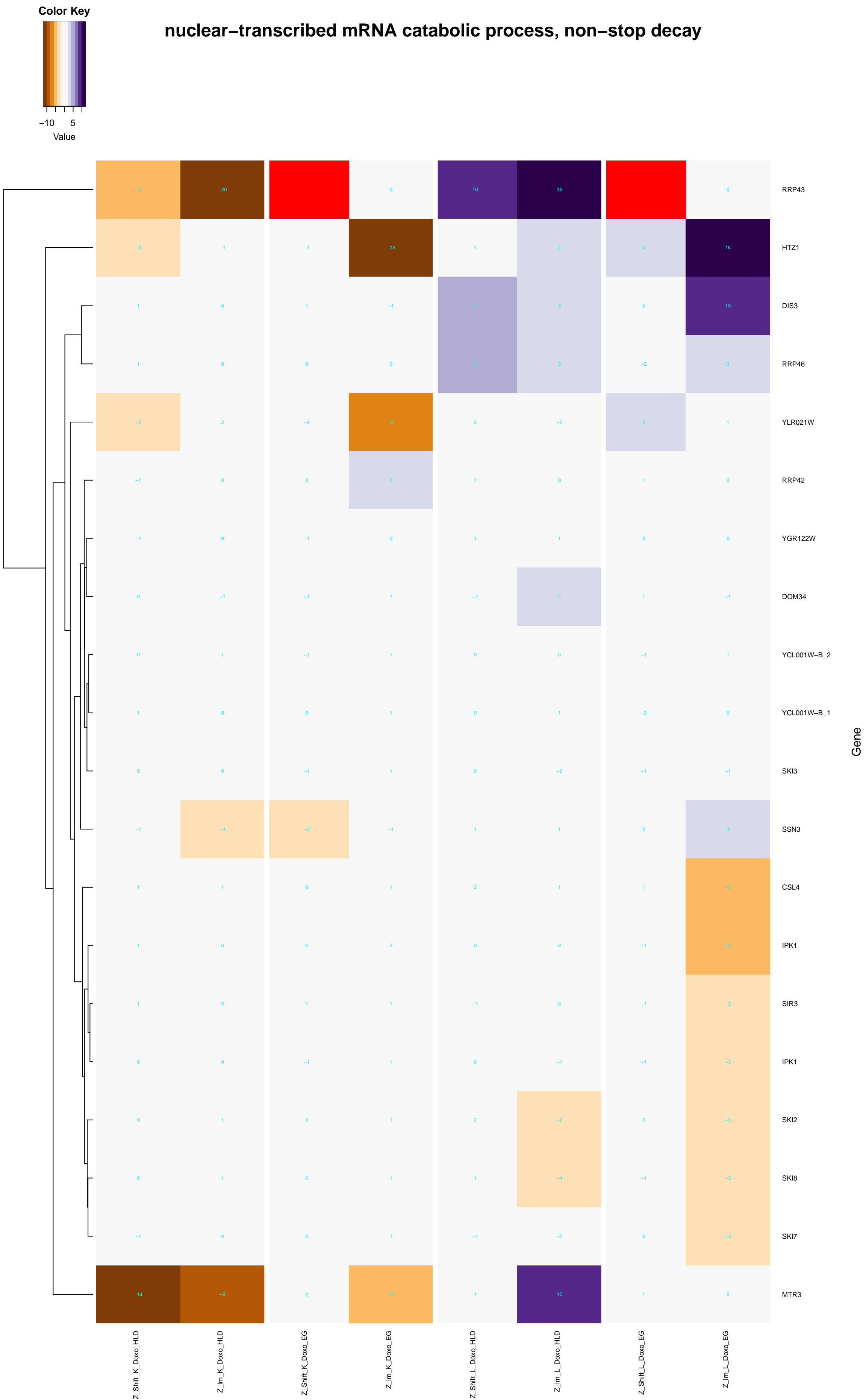

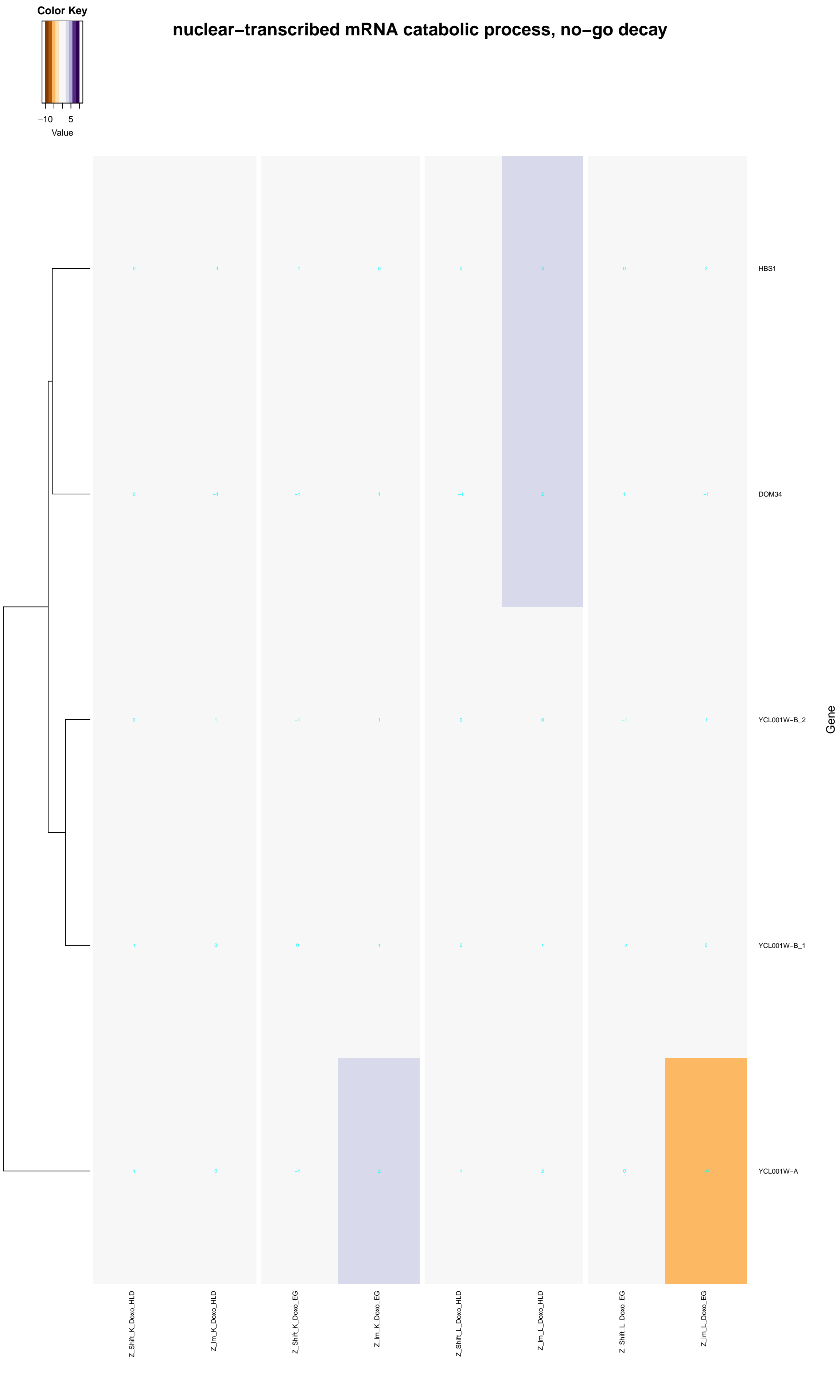

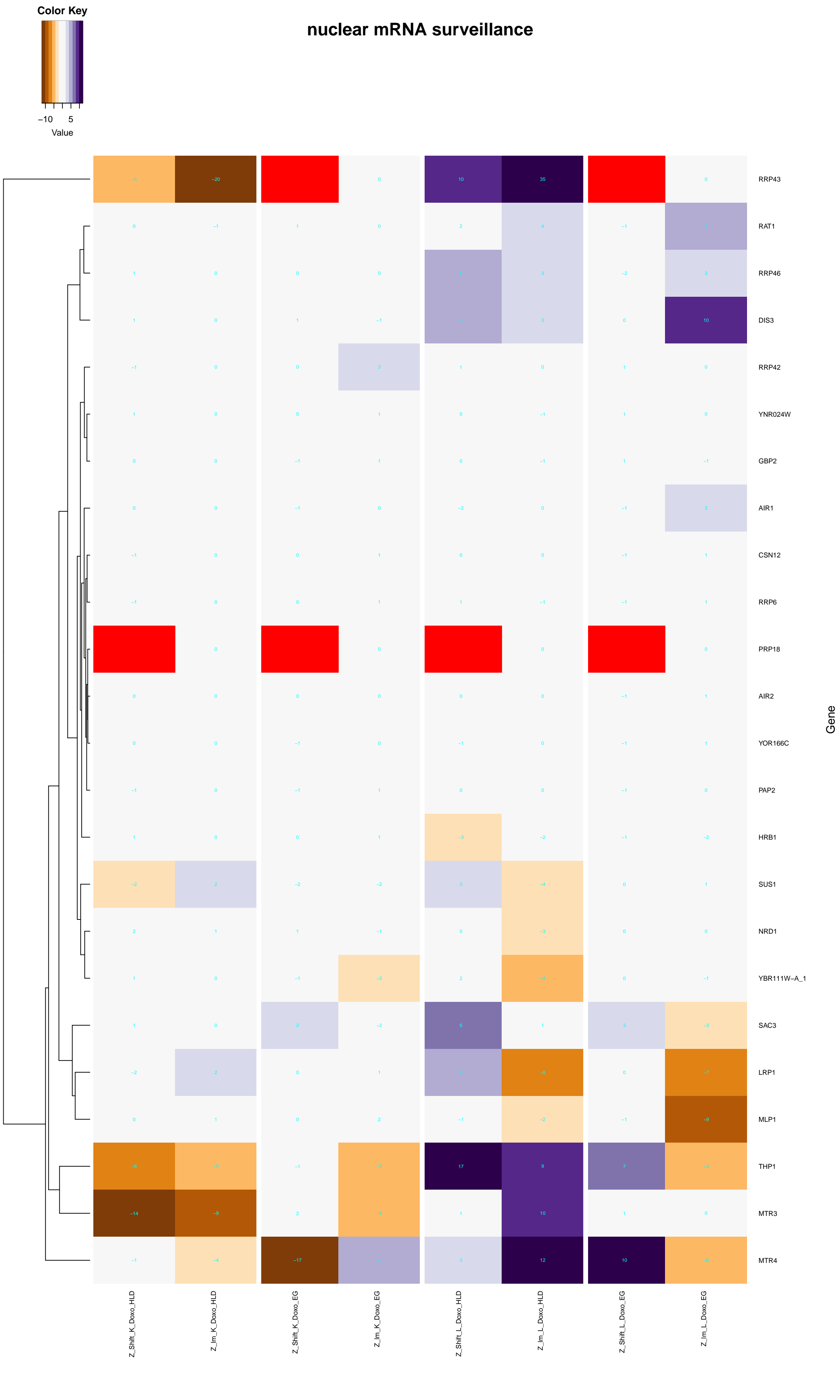

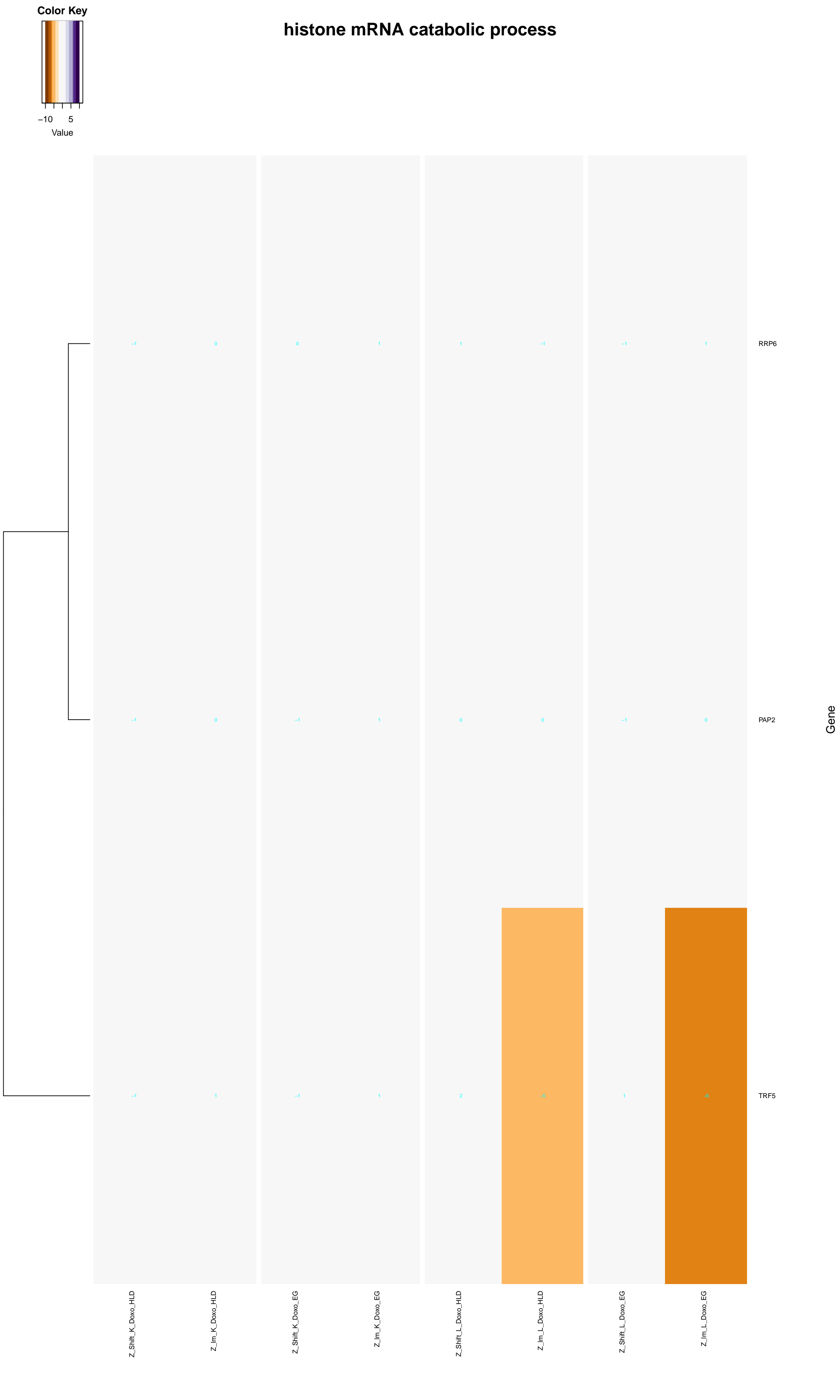

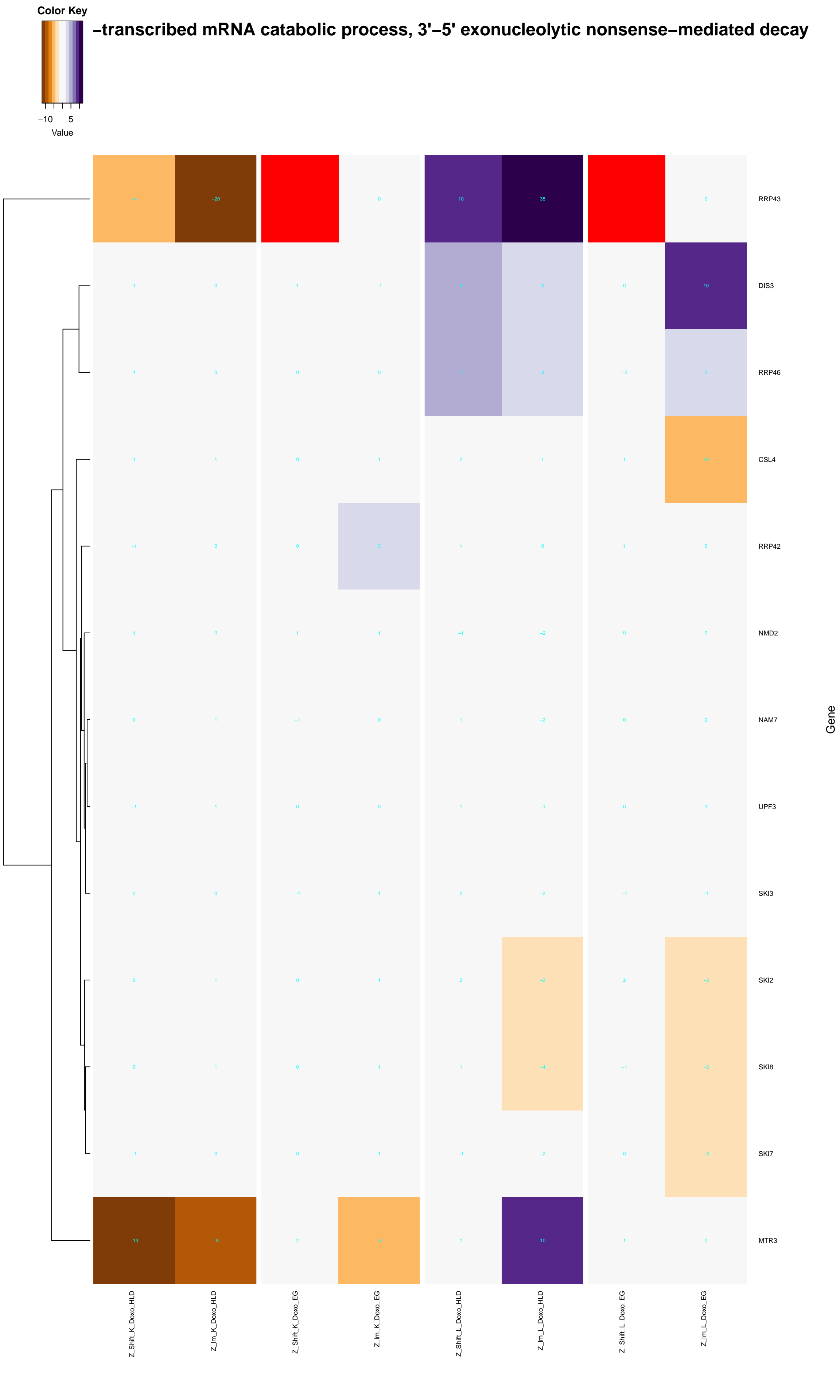

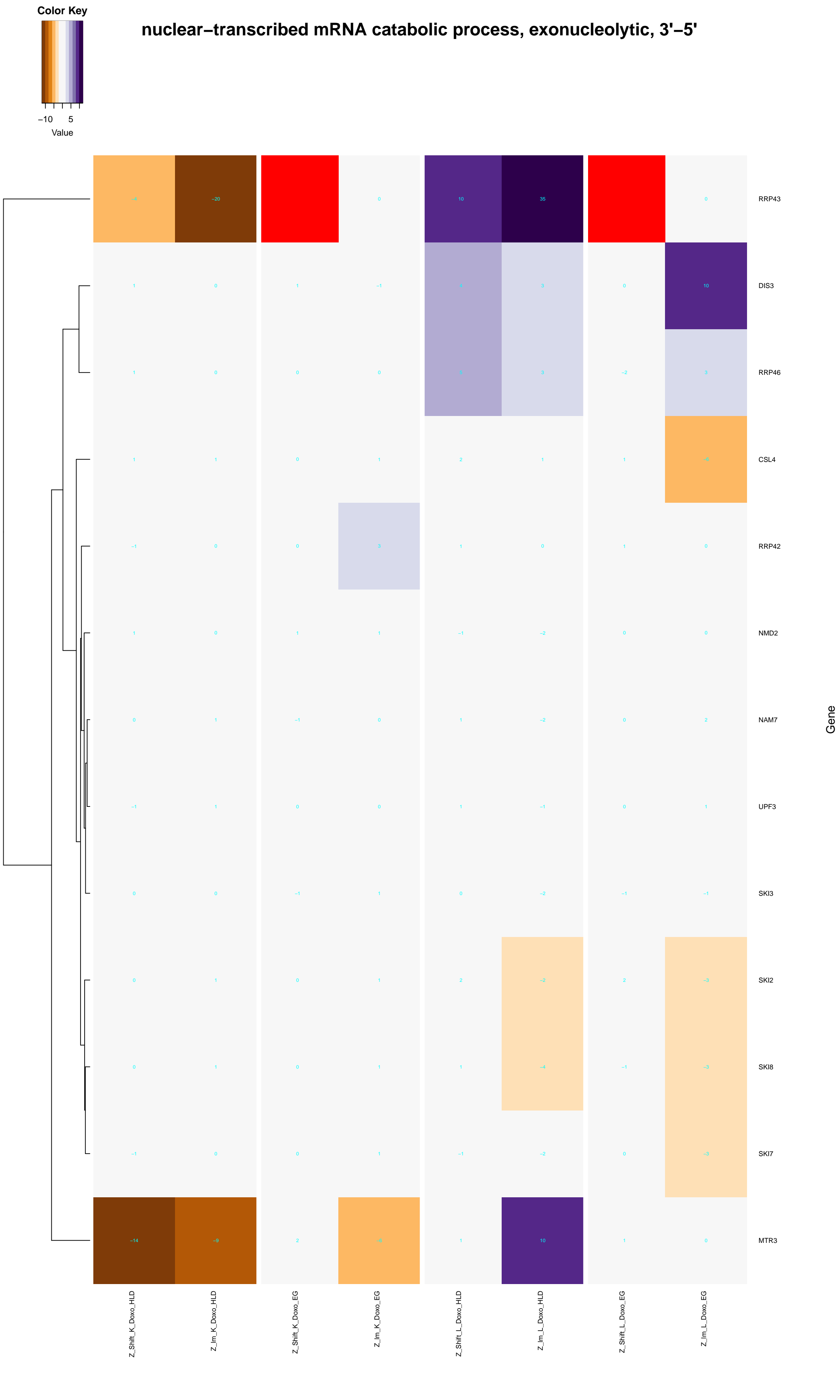

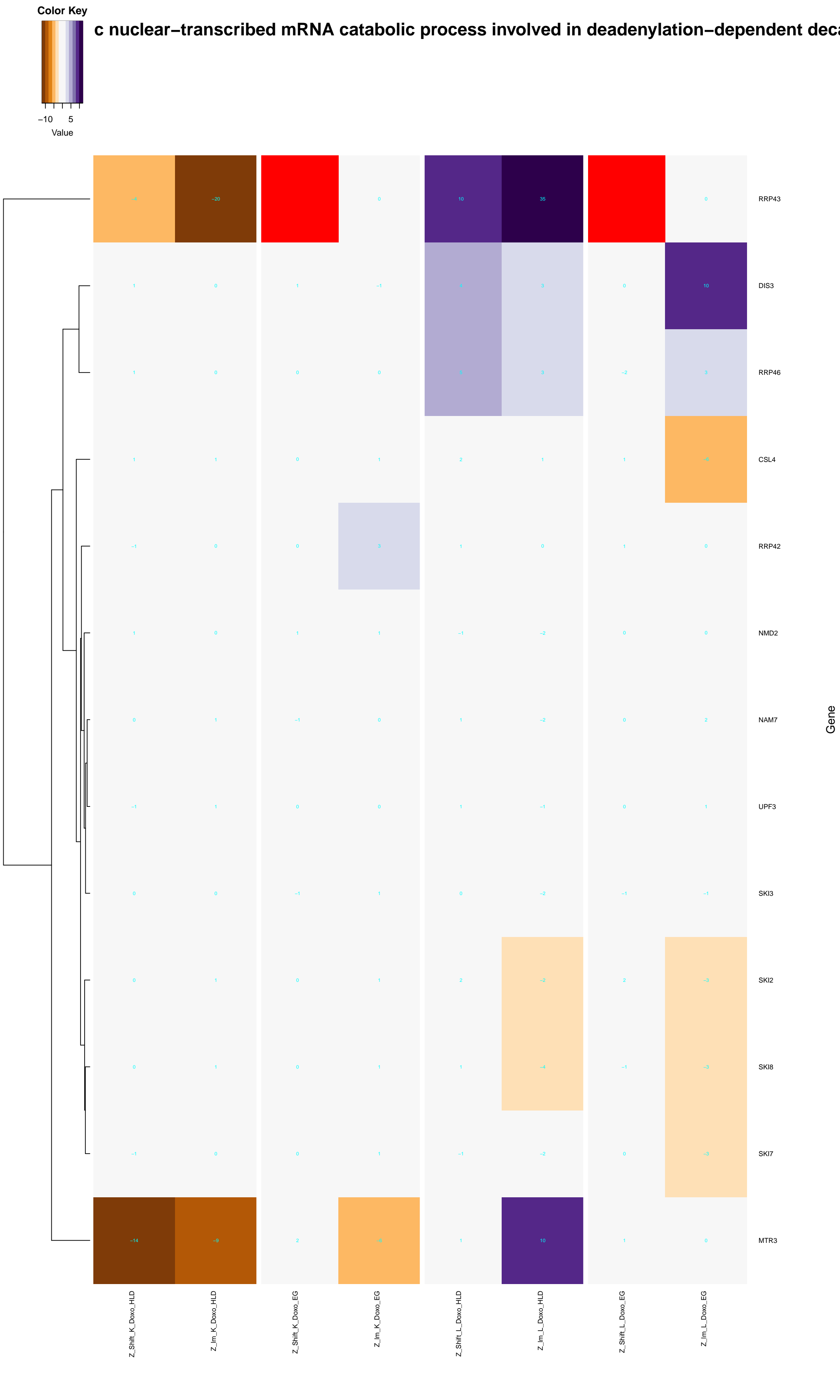

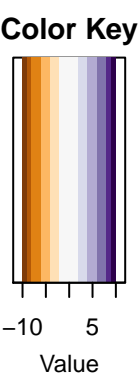

nuclear mRNA surveillance of spliceosomal pre-mRNA splicing

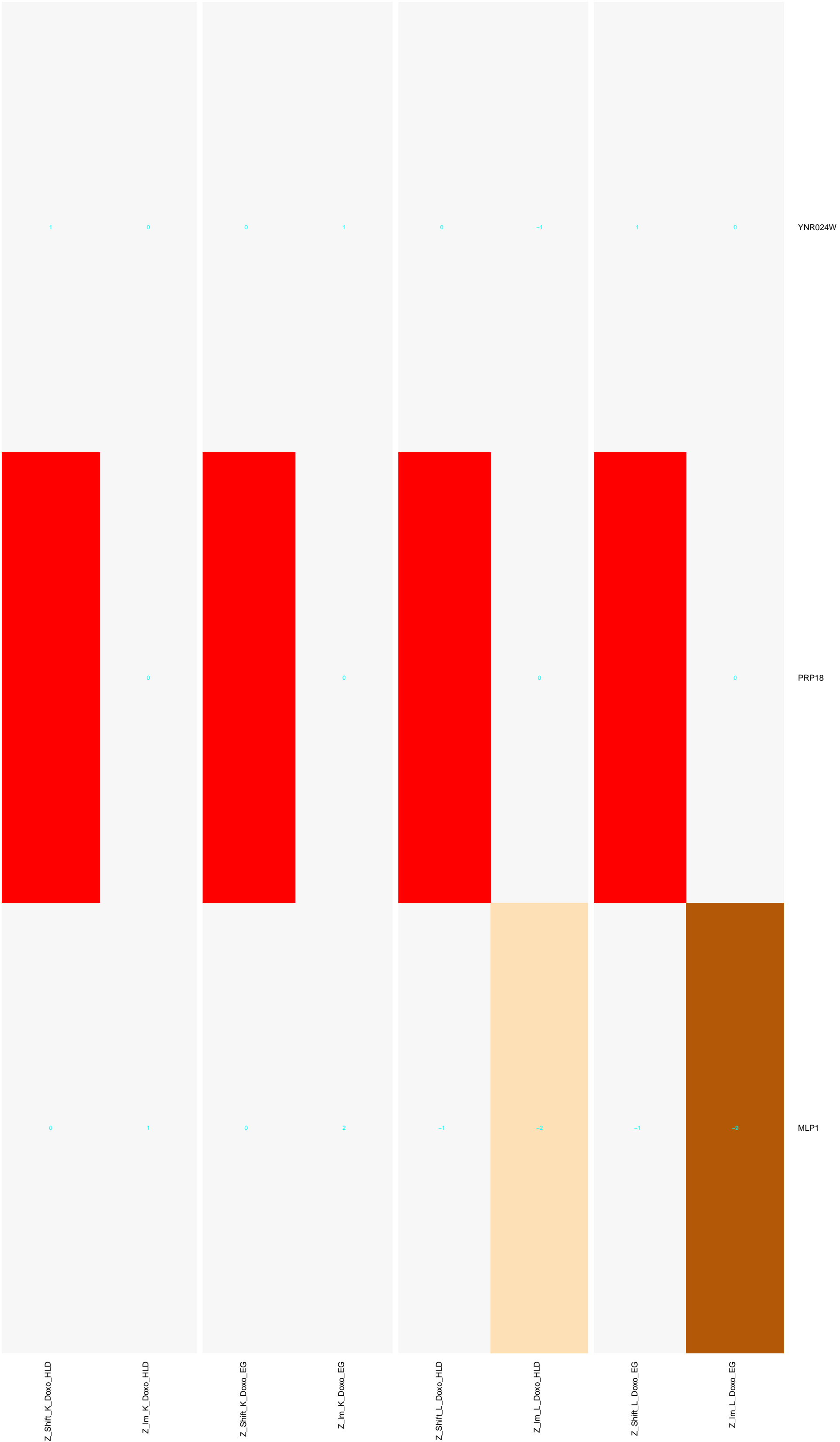

Gene

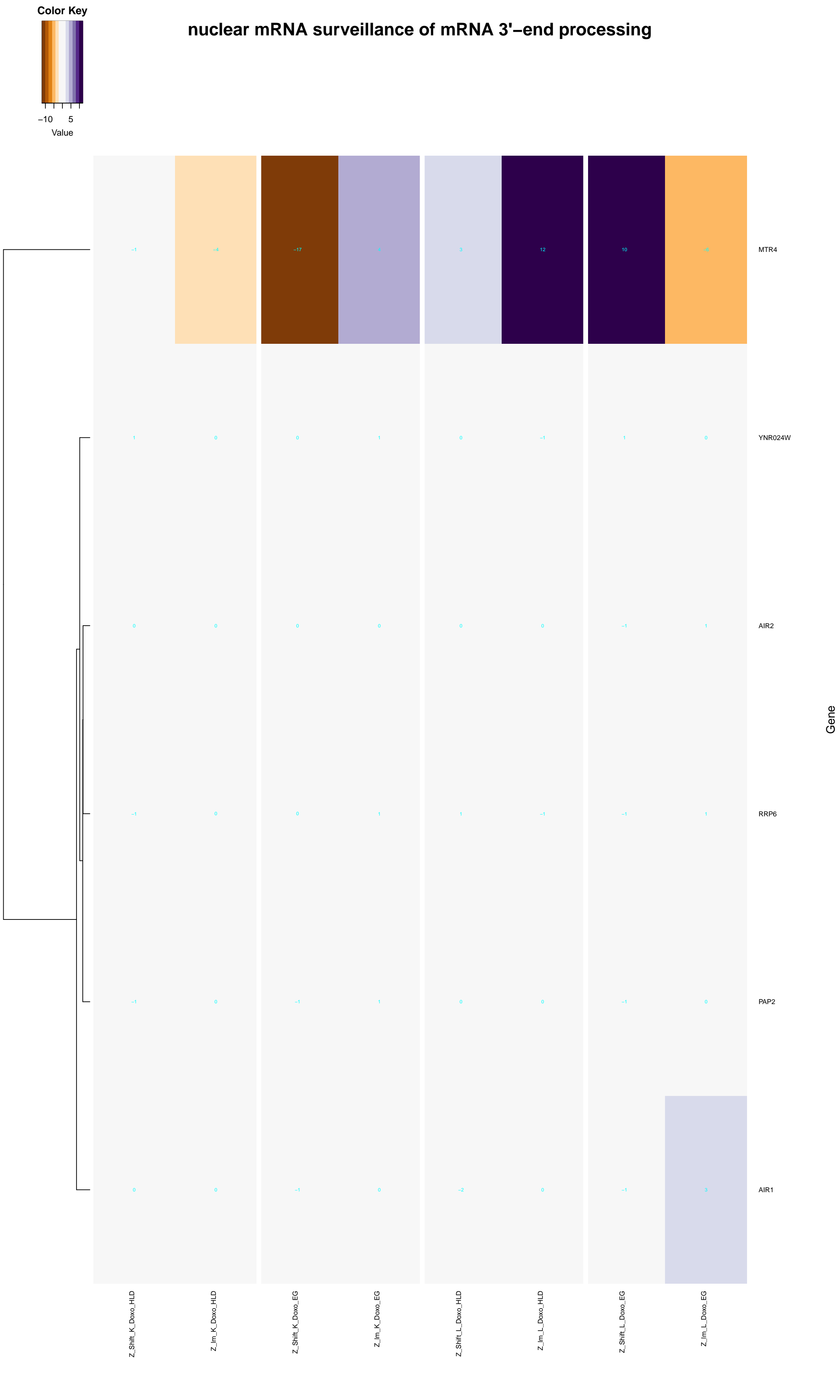

Supplement: Supplementary file 9 — Additional file 9. GO term-specific heatmaps for REMc/GTF-enriched clusters. GO term-specific heatmaps for significant GO process terms were generated as described in methods and Figs. 3 and 4. Any related child terms are presented in subsequent pages of the parent file name. GO terms with more than 100 children, with 2 or fewer genes annotated to the term, or a file size over 300KB are not shown. All heatmaps are generated with the same layout (see Figs. 3 and 4). [file 40170_2019_201_MOESM9_ESM.bz2 › Additional_File9_GOTermHeatmaps/Additional_File9_GOTermHeatmap/nuclear-transcribed_mRNA_catabolic_process.pdf]

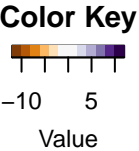

nuclear DNA replication

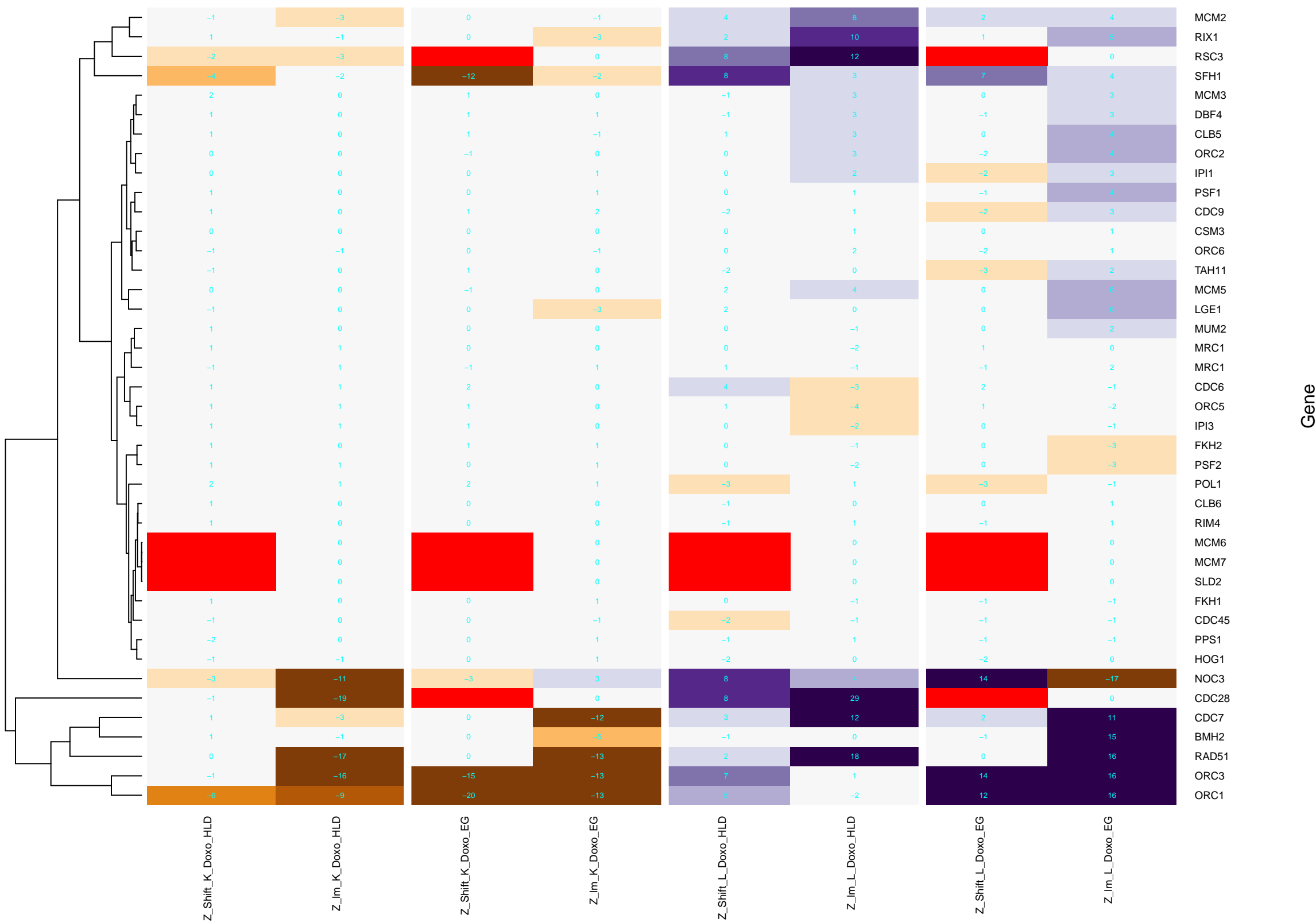

Color Key

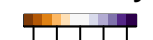

-10 5

Value

# premeiotic DNA replication

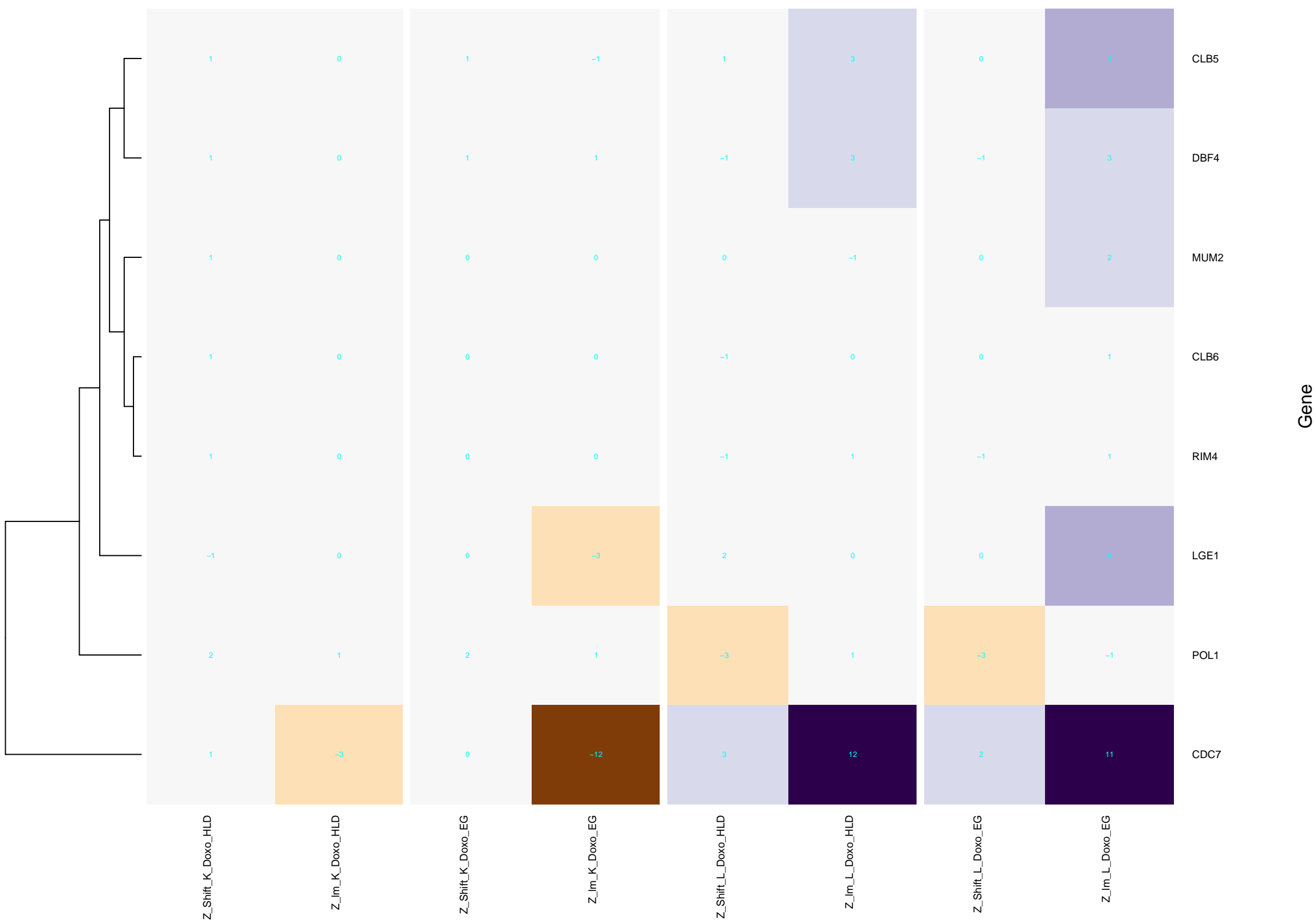

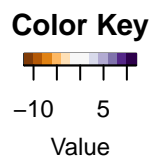

# mitotic DNA replication

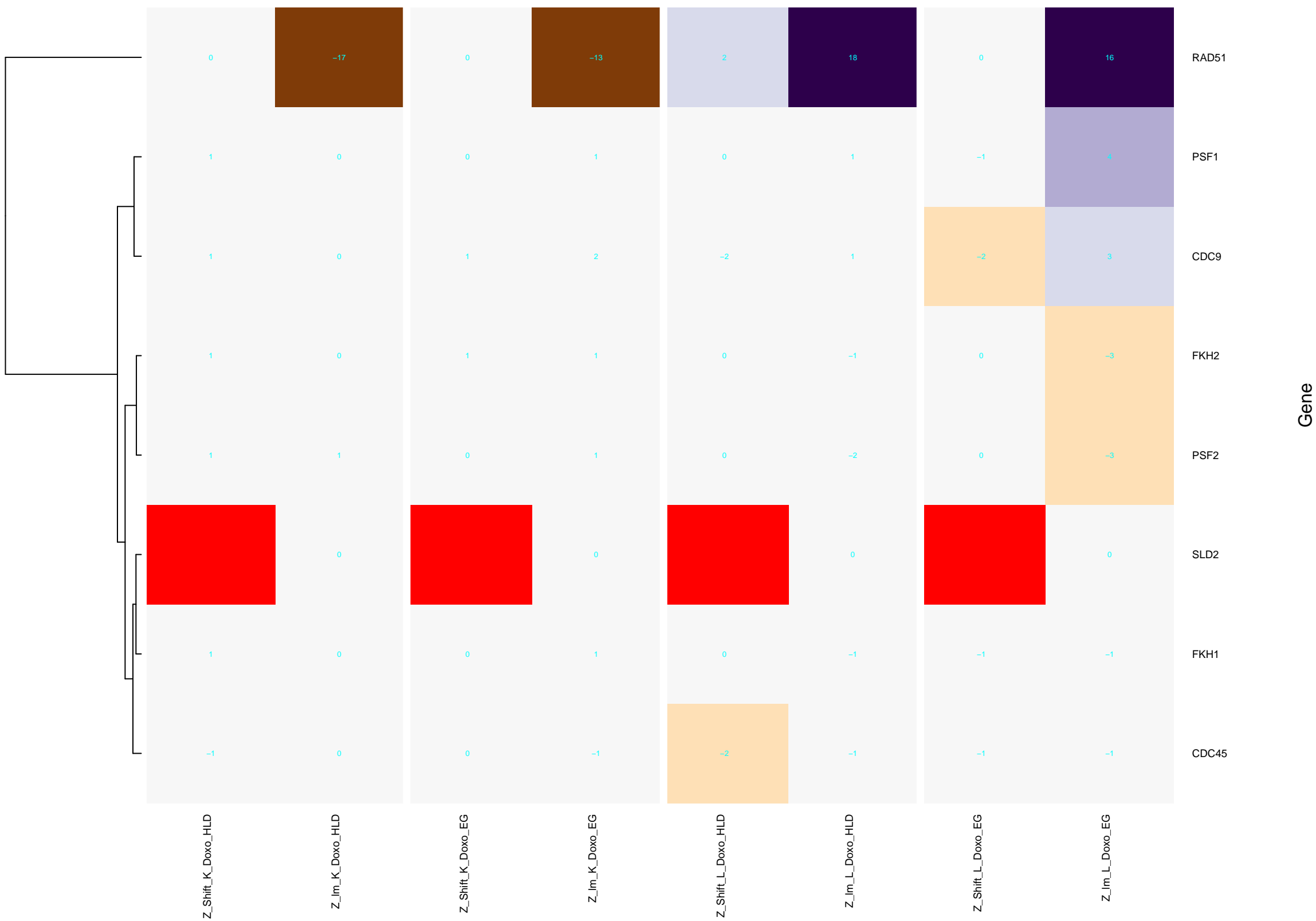

Supplement: Supplementary file 9 — Additional file 9. GO term-specific heatmaps for REMc/GTF-enriched clusters. GO term-specific heatmaps for significant GO process terms were generated as described in methods and Figs. 3 and 4. Any related child terms are presented in subsequent pages of the parent file name. GO terms with more than 100 children, with 2 or fewer genes annotated to the term, or a file size over 300KB are not shown. All heatmaps are generated with the same layout (see Figs. 3 and 4). [file 40170_2019_201_MOESM9_ESM.bz2 › Additional_File9_GOTermHeatmaps/Additional_File9_GOTermHeatmap/nuclear_DNA_replication.pdf]

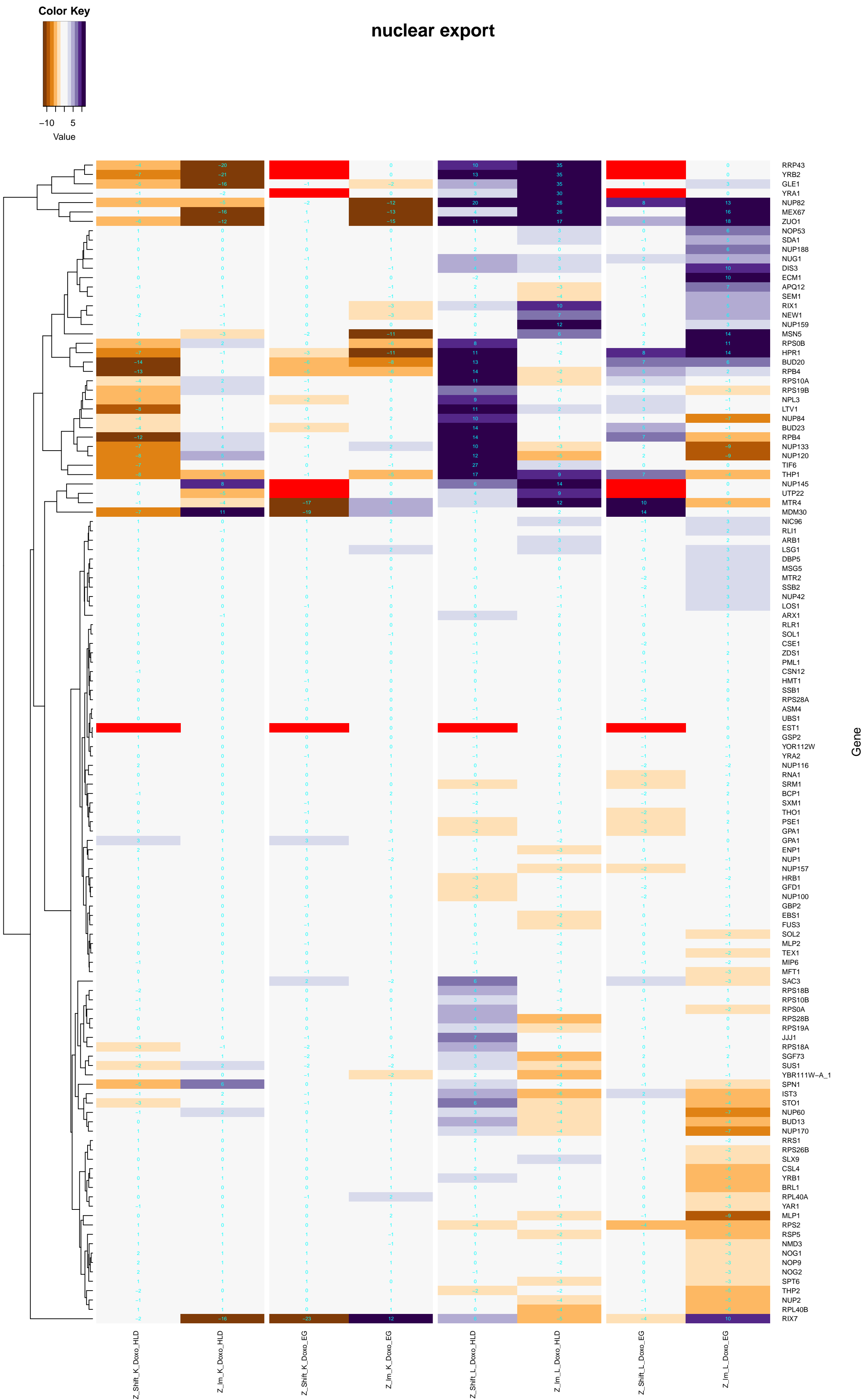

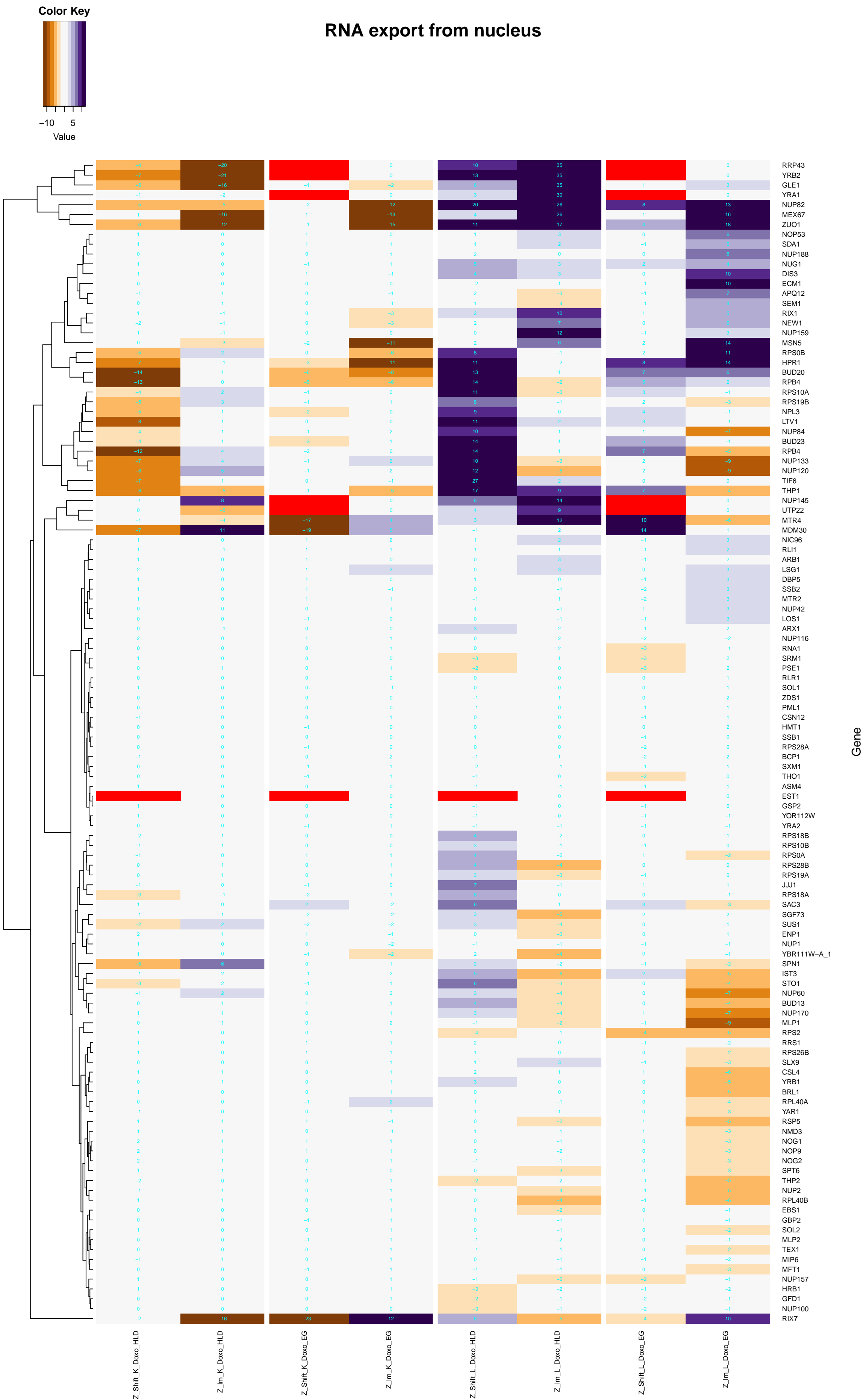

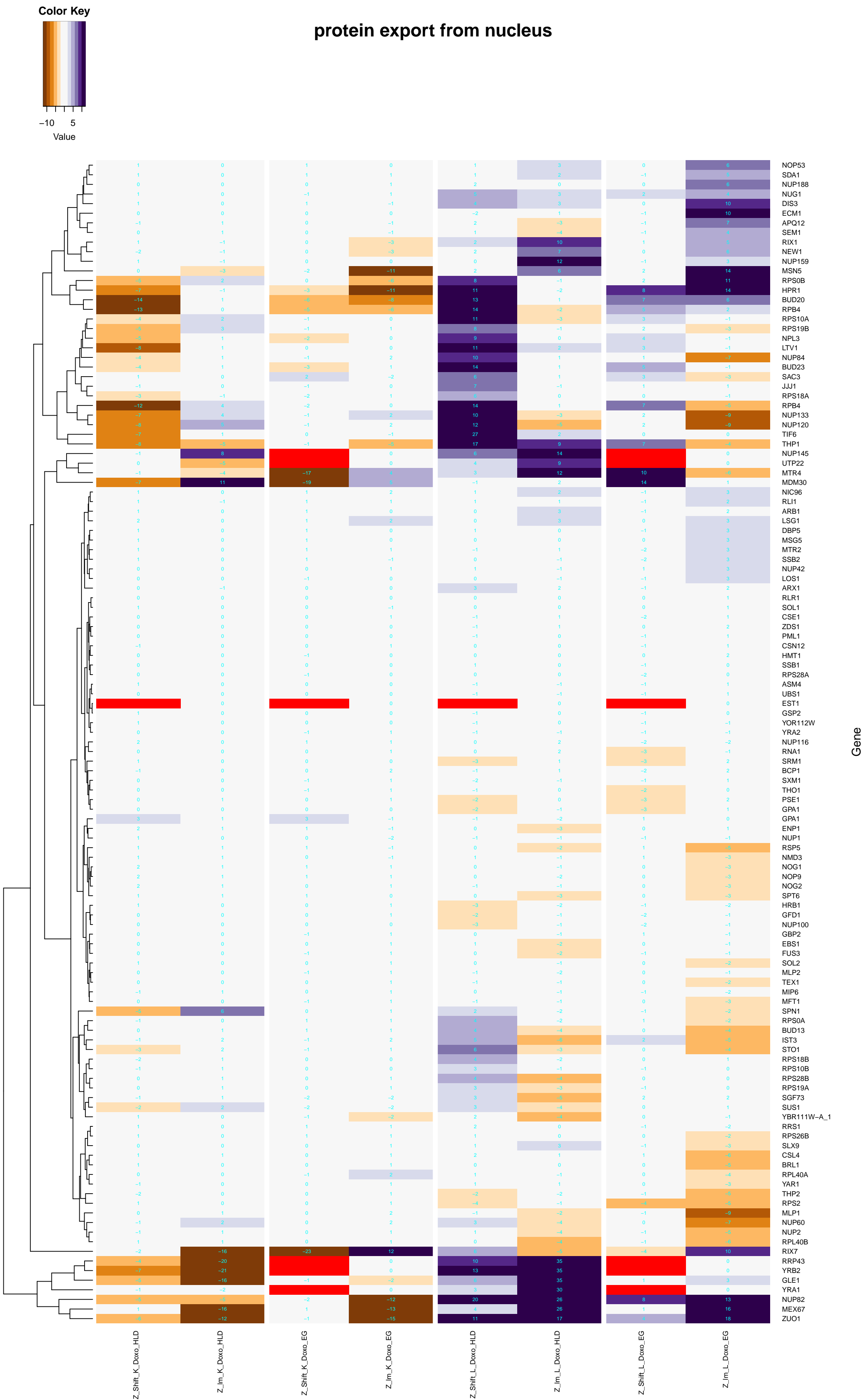

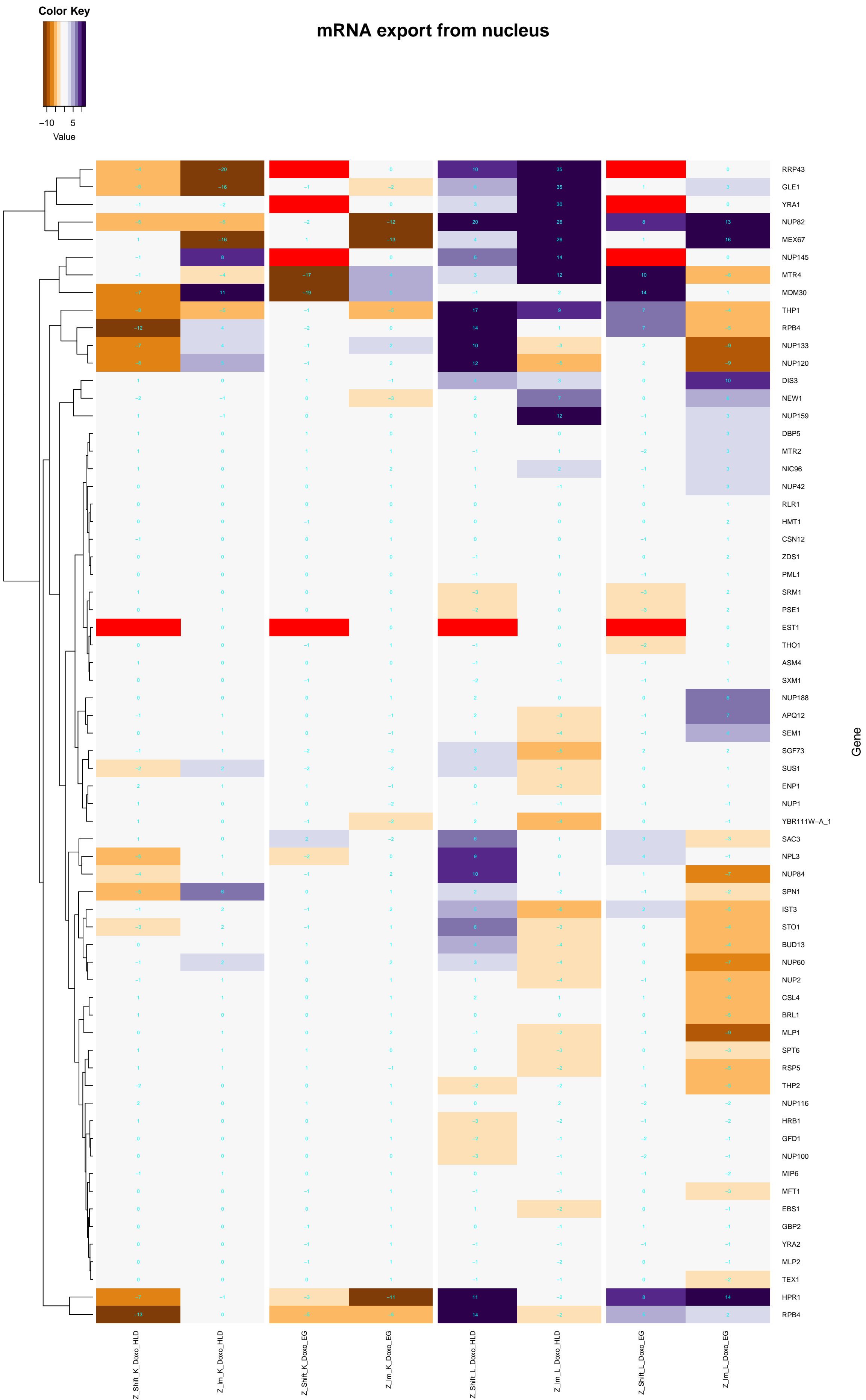

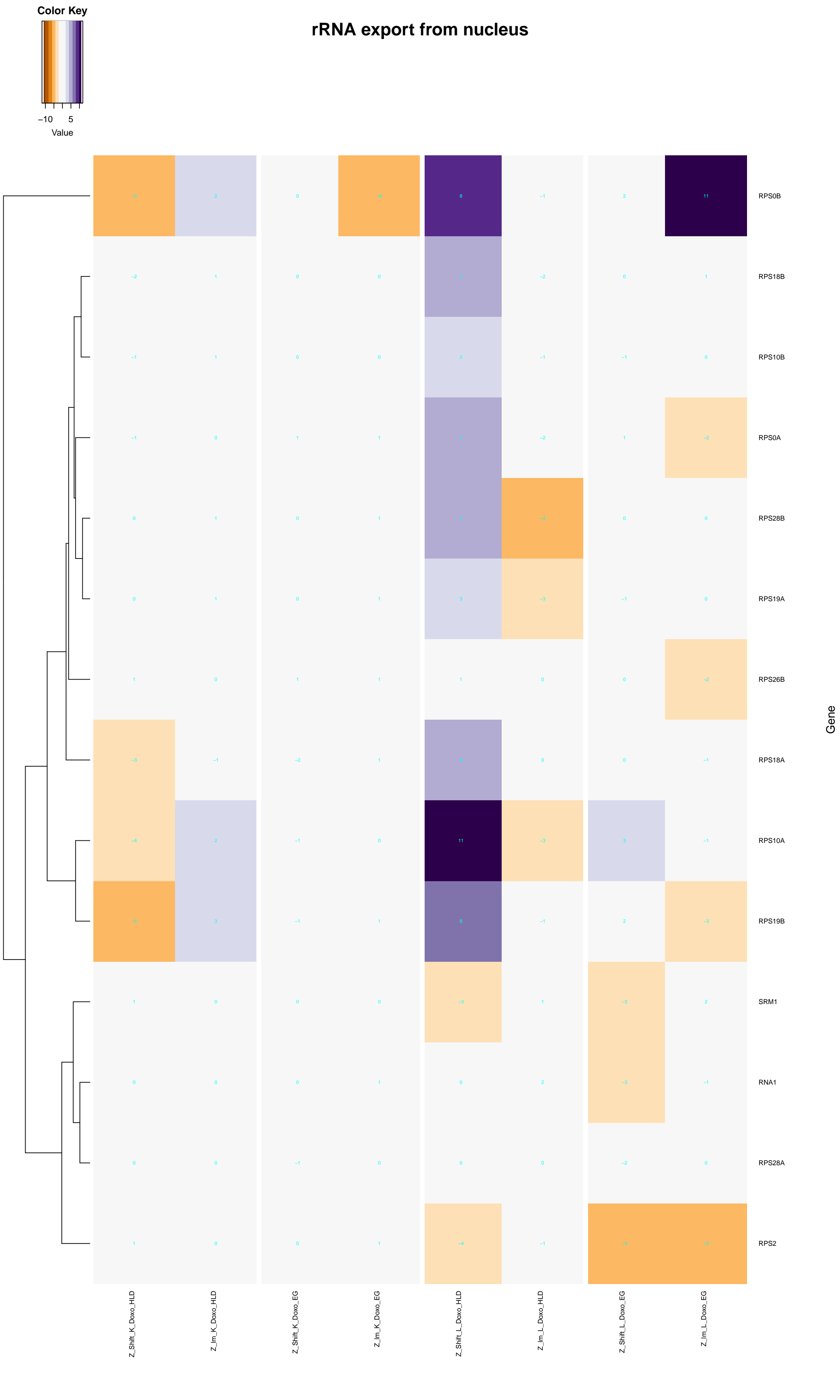

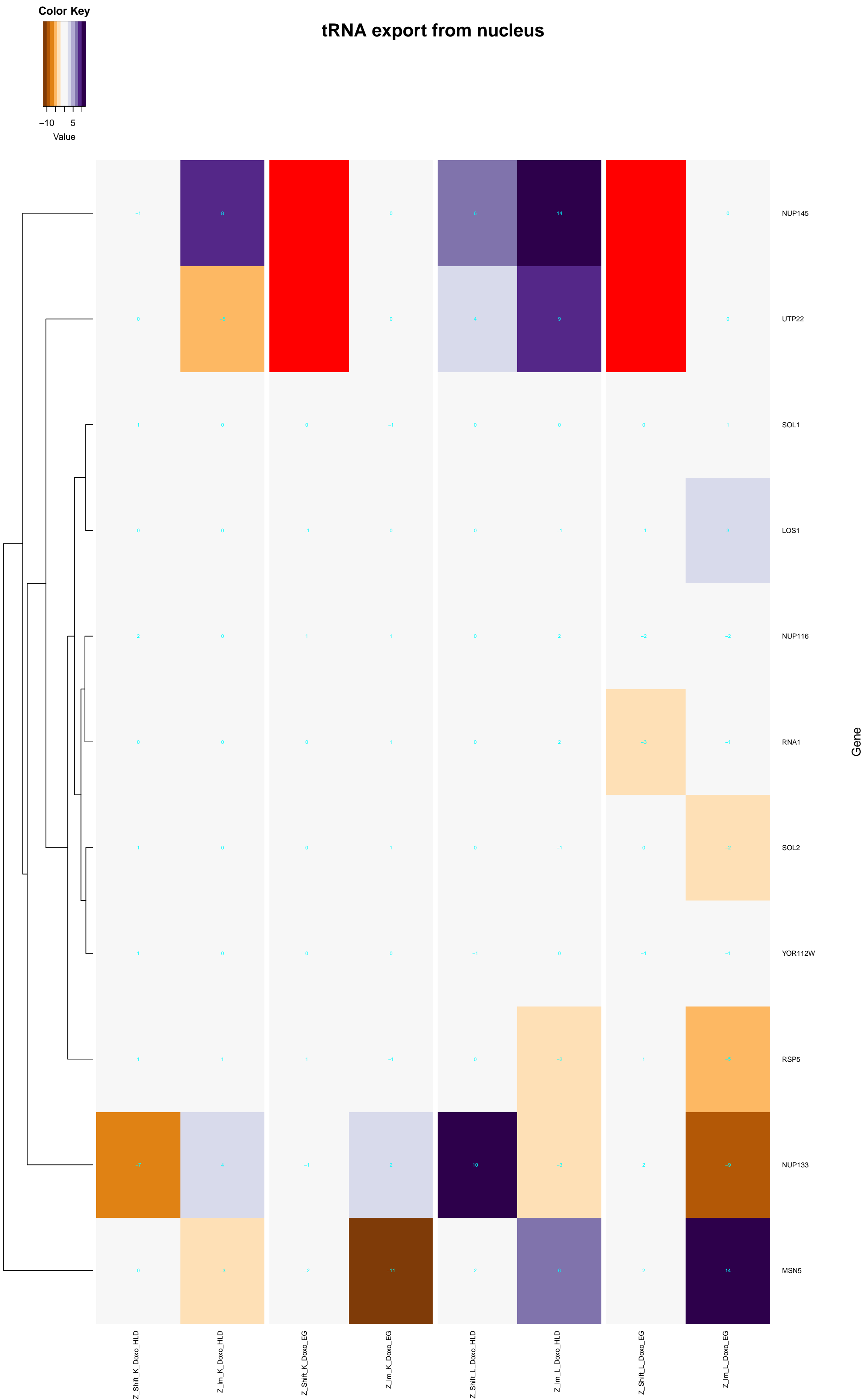

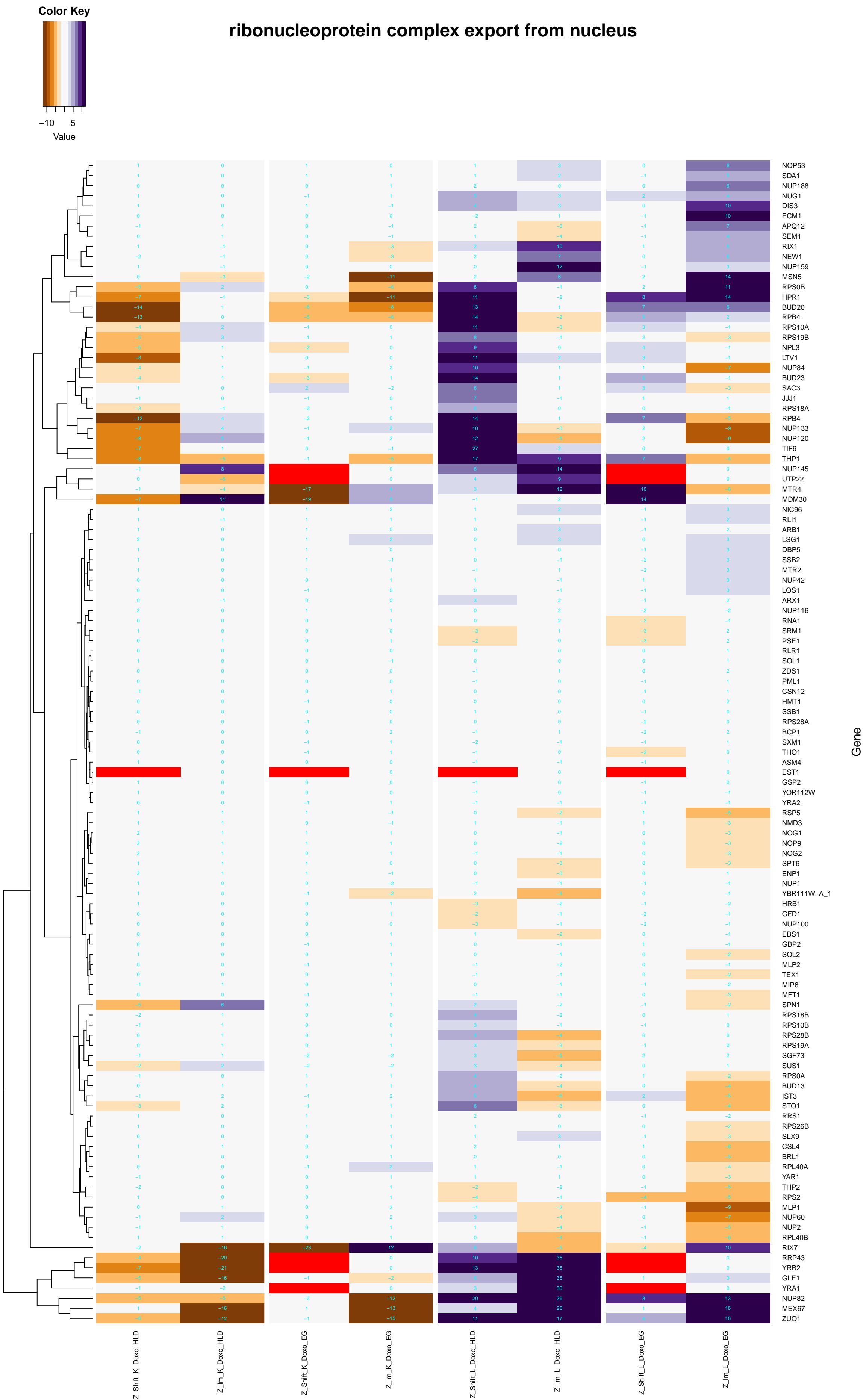

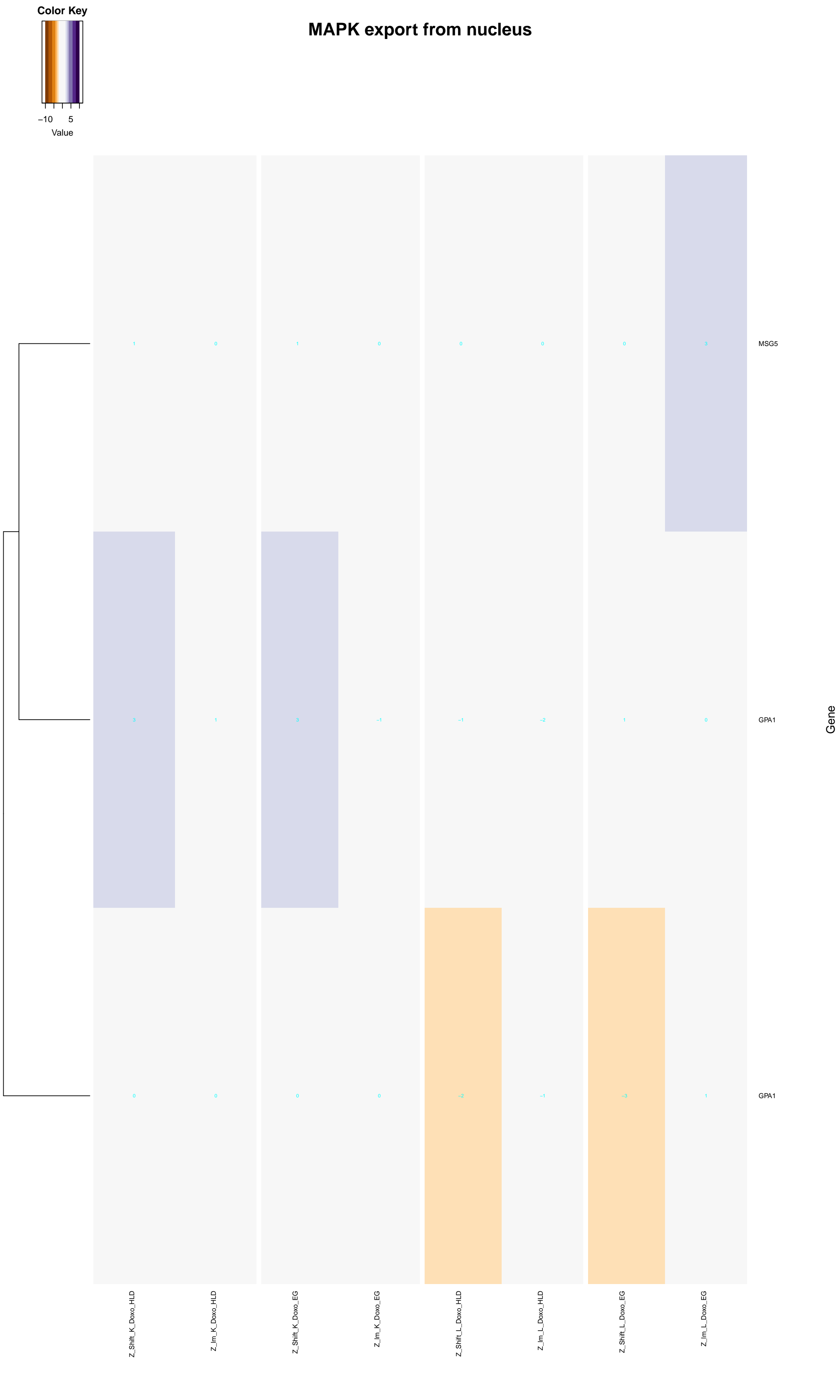

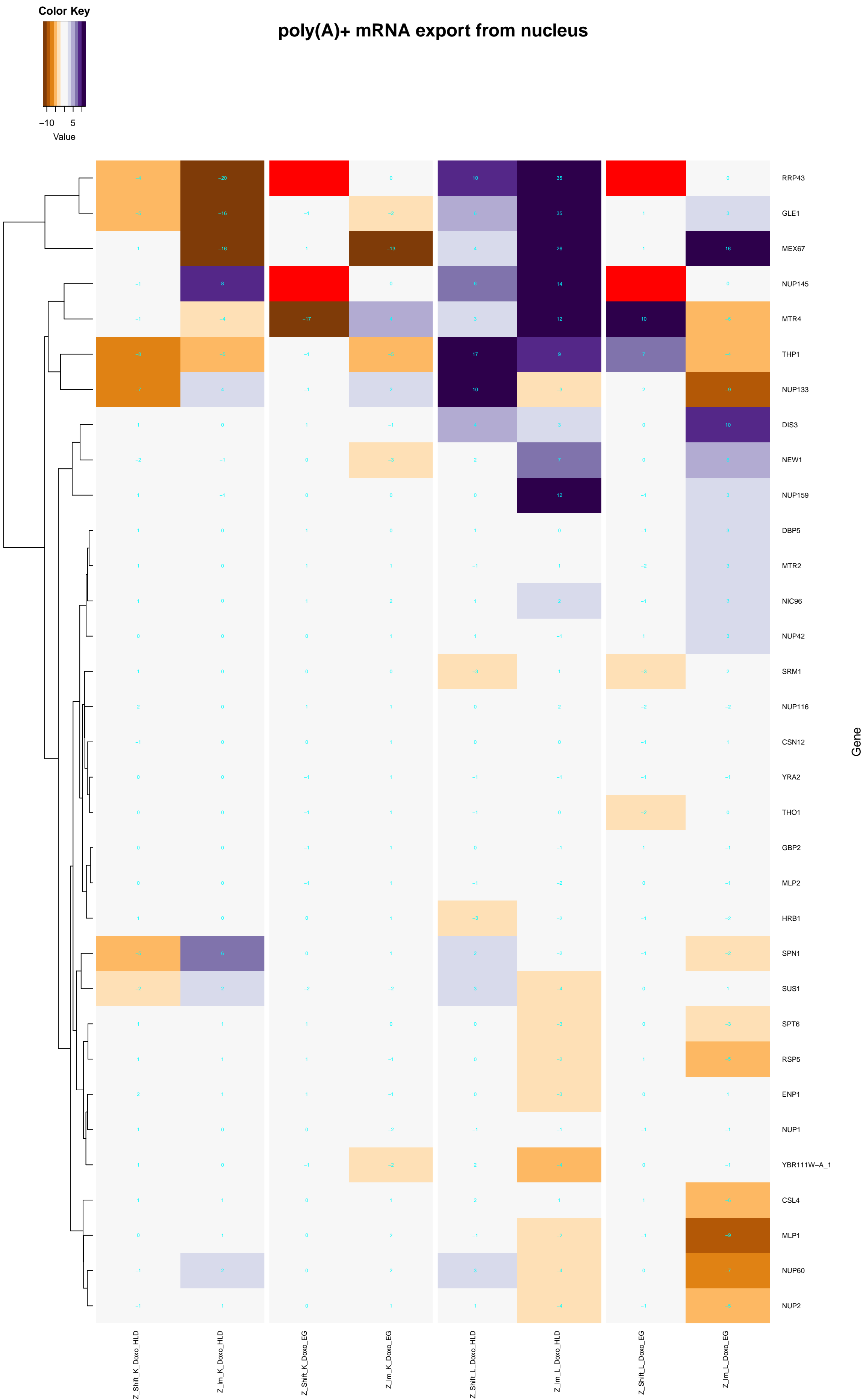

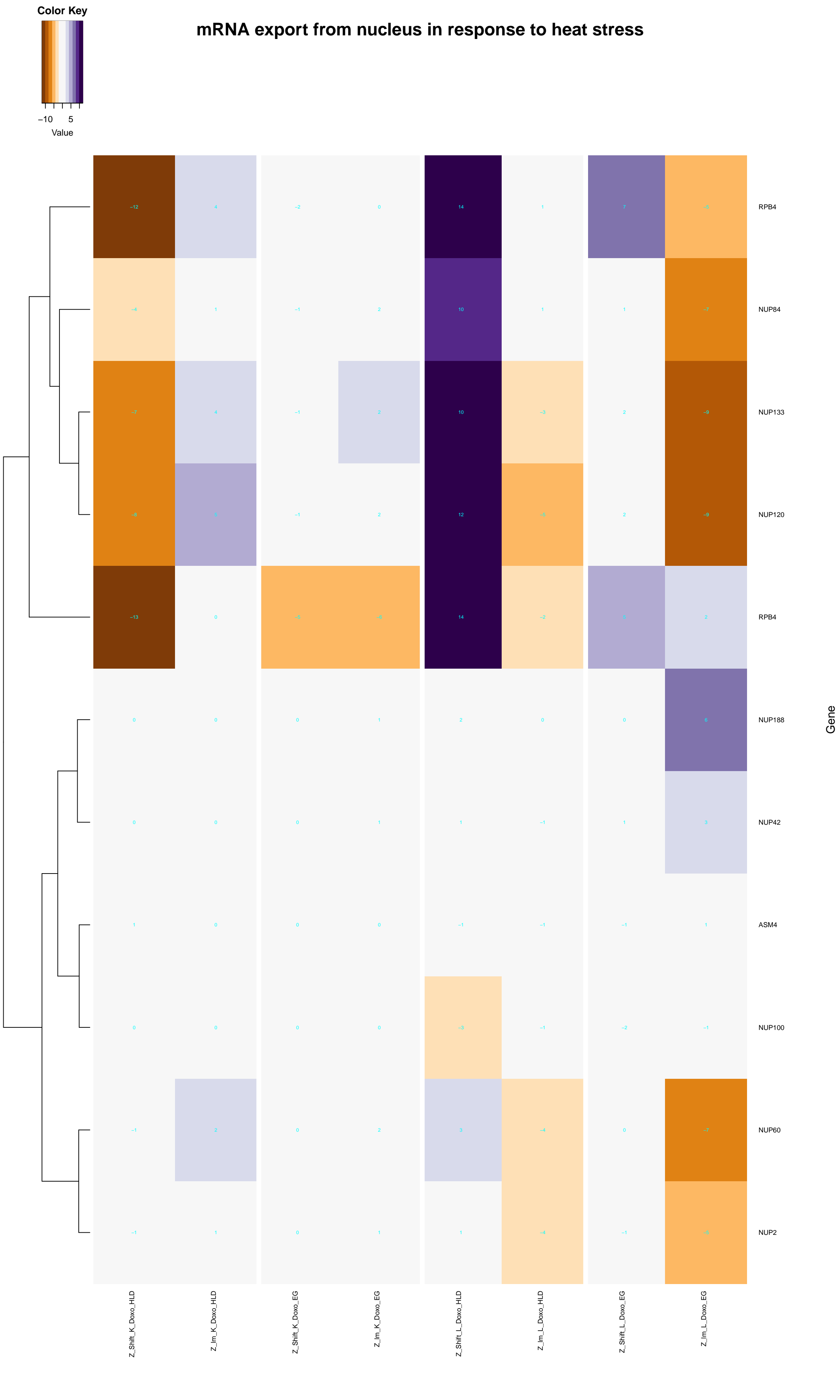

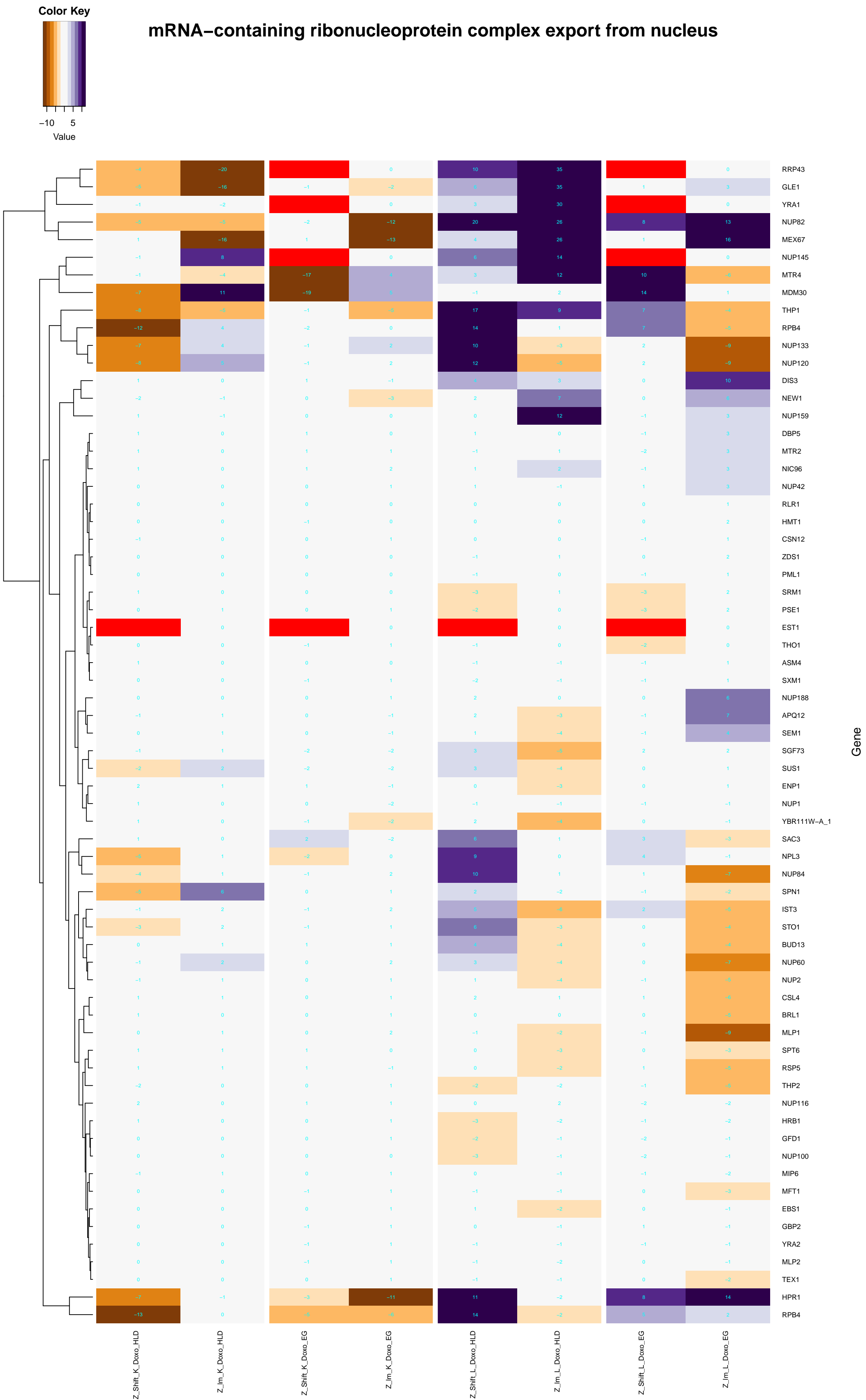

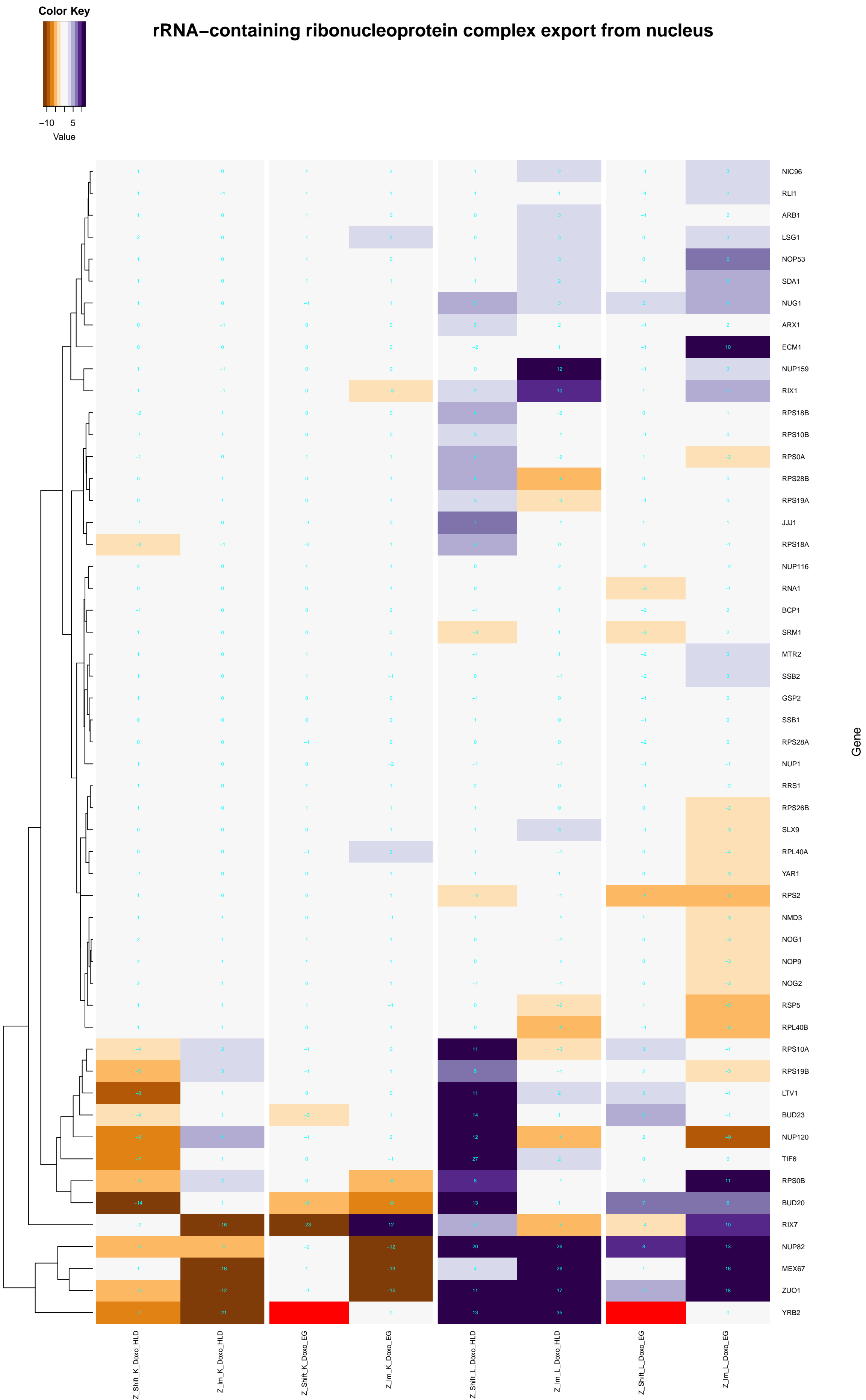

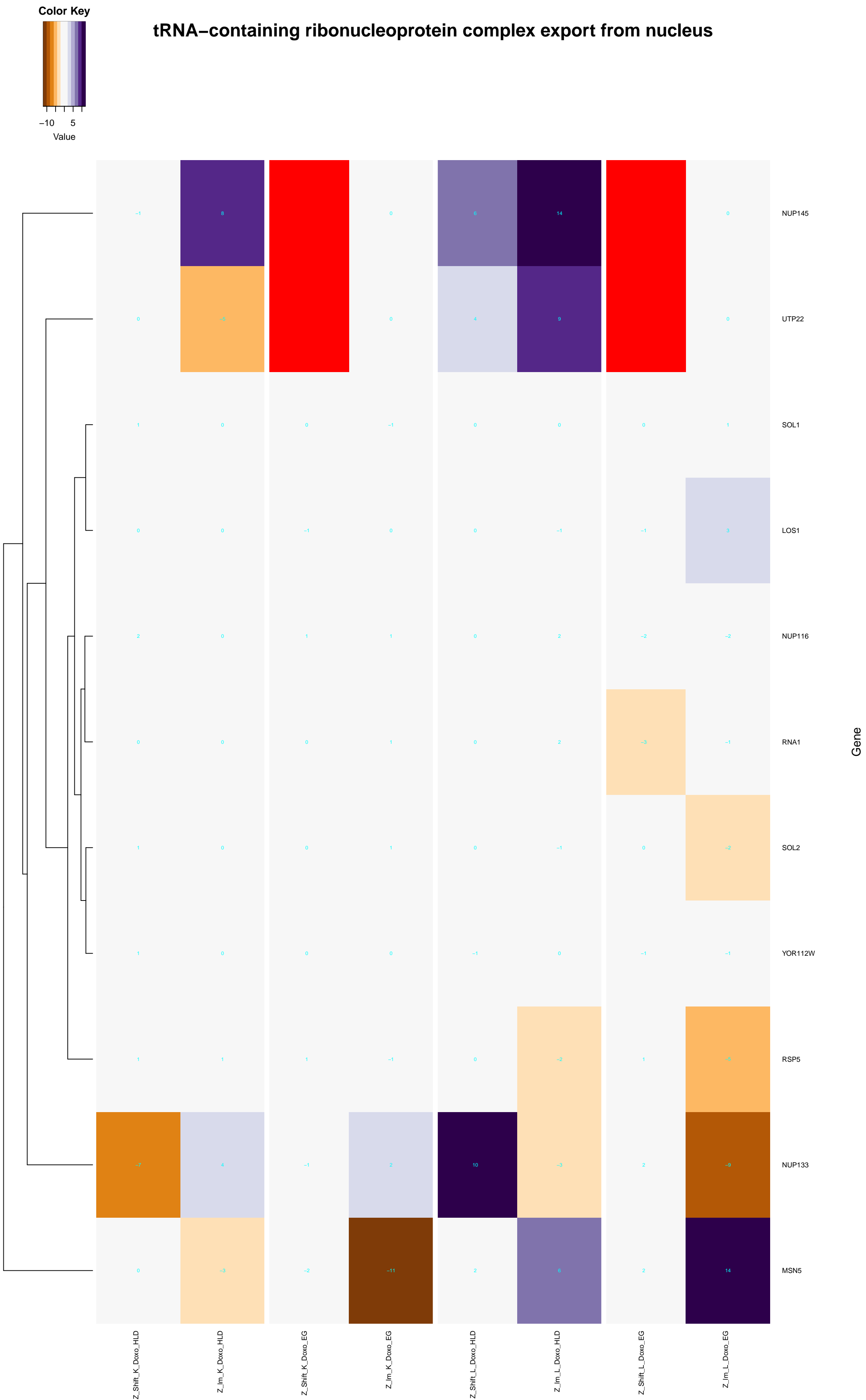

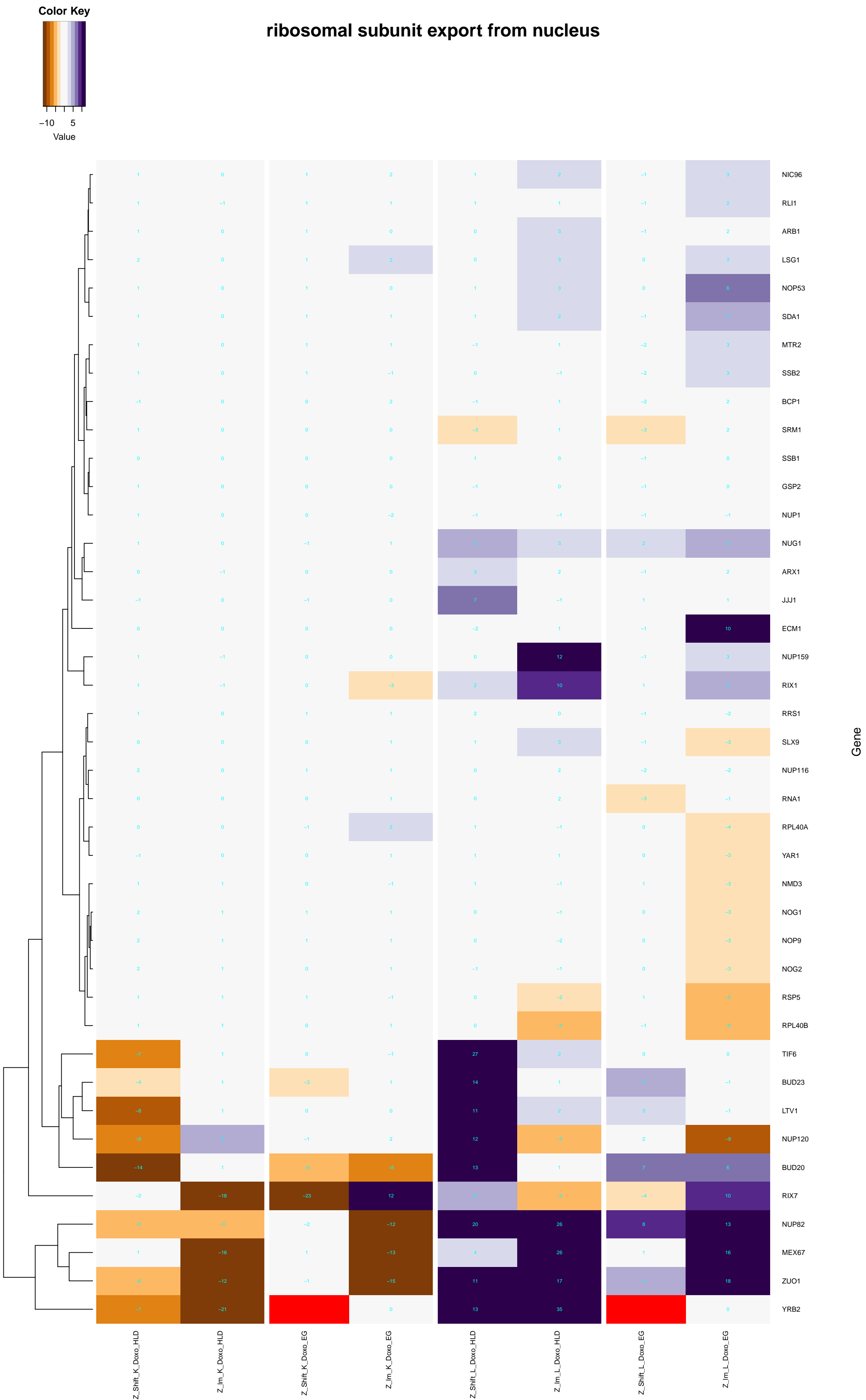

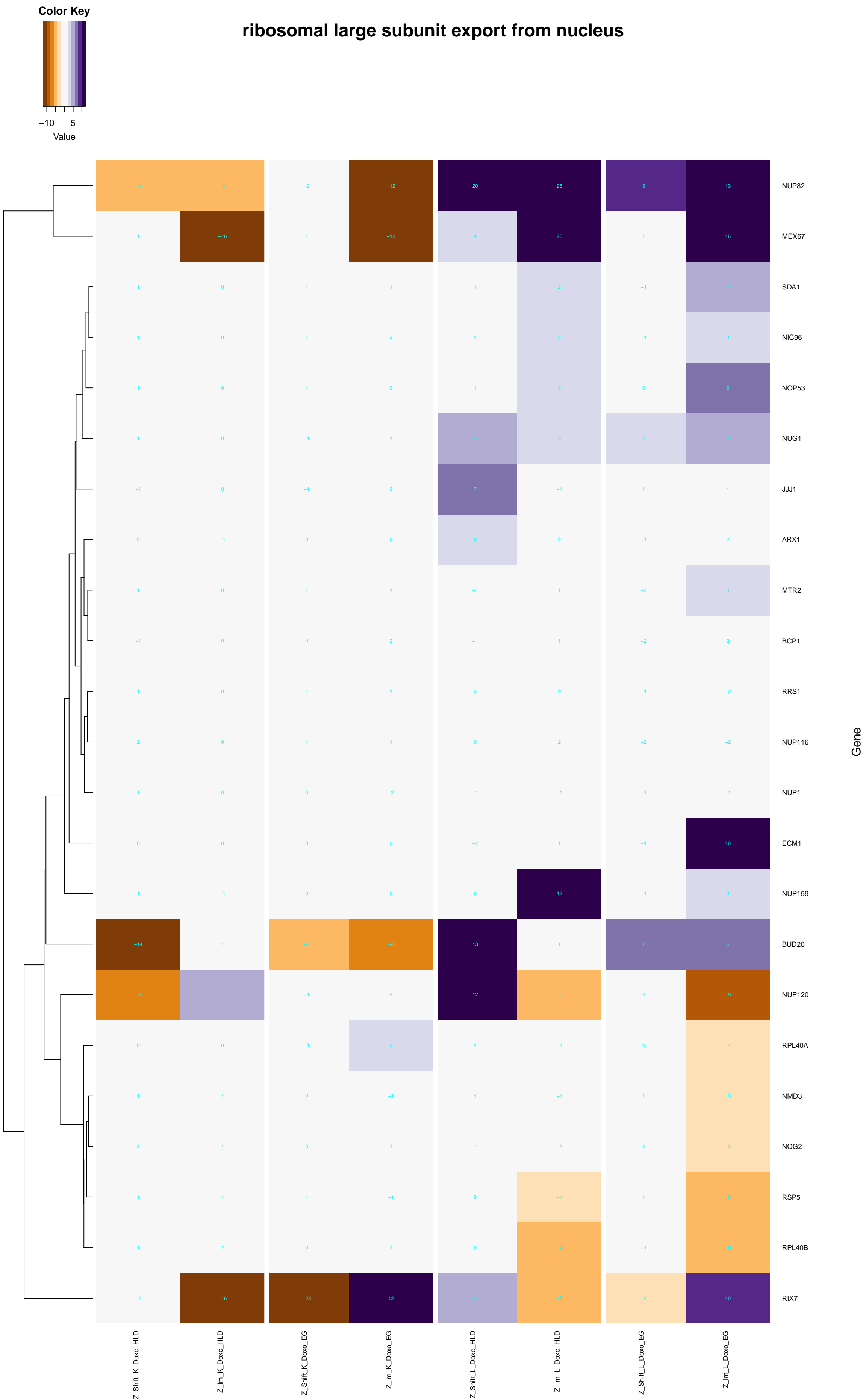

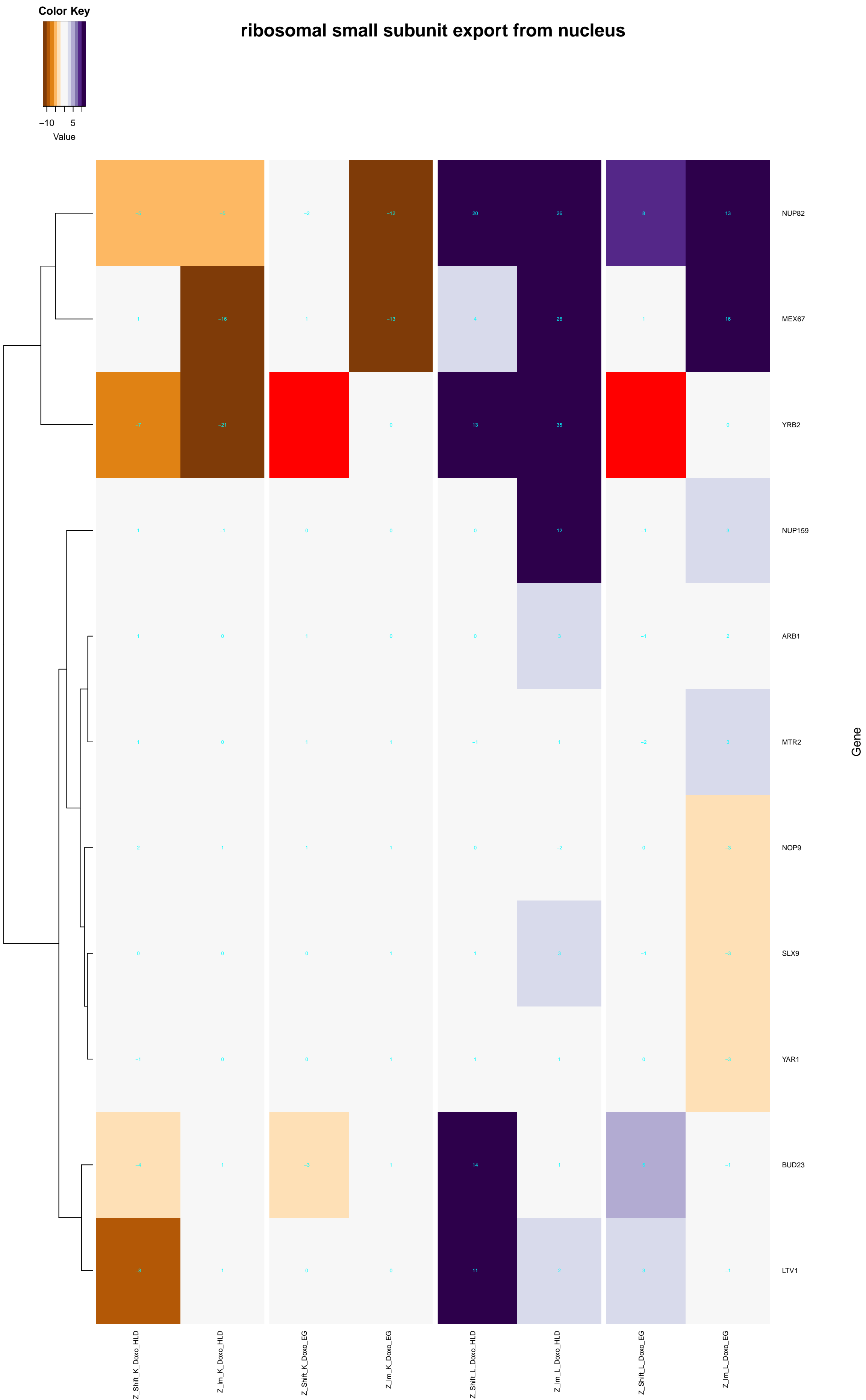

Supplement: Supplementary file 9 — Additional file 9. GO term-specific heatmaps for REMc/GTF-enriched clusters. GO term-specific heatmaps for significant GO process terms were generated as described in methods and Figs. 3 and 4. Any related child terms are presented in subsequent pages of the parent file name. GO terms with more than 100 children, with 2 or fewer genes annotated to the term, or a file size over 300KB are not shown. All heatmaps are generated with the same layout (see Figs. 3 and 4). [file 40170_2019_201_MOESM9_ESM.bz2 › Additional_File9_GOTermHeatmaps/Additional_File9_GOTermHeatmap/nuclear_export.pdf]

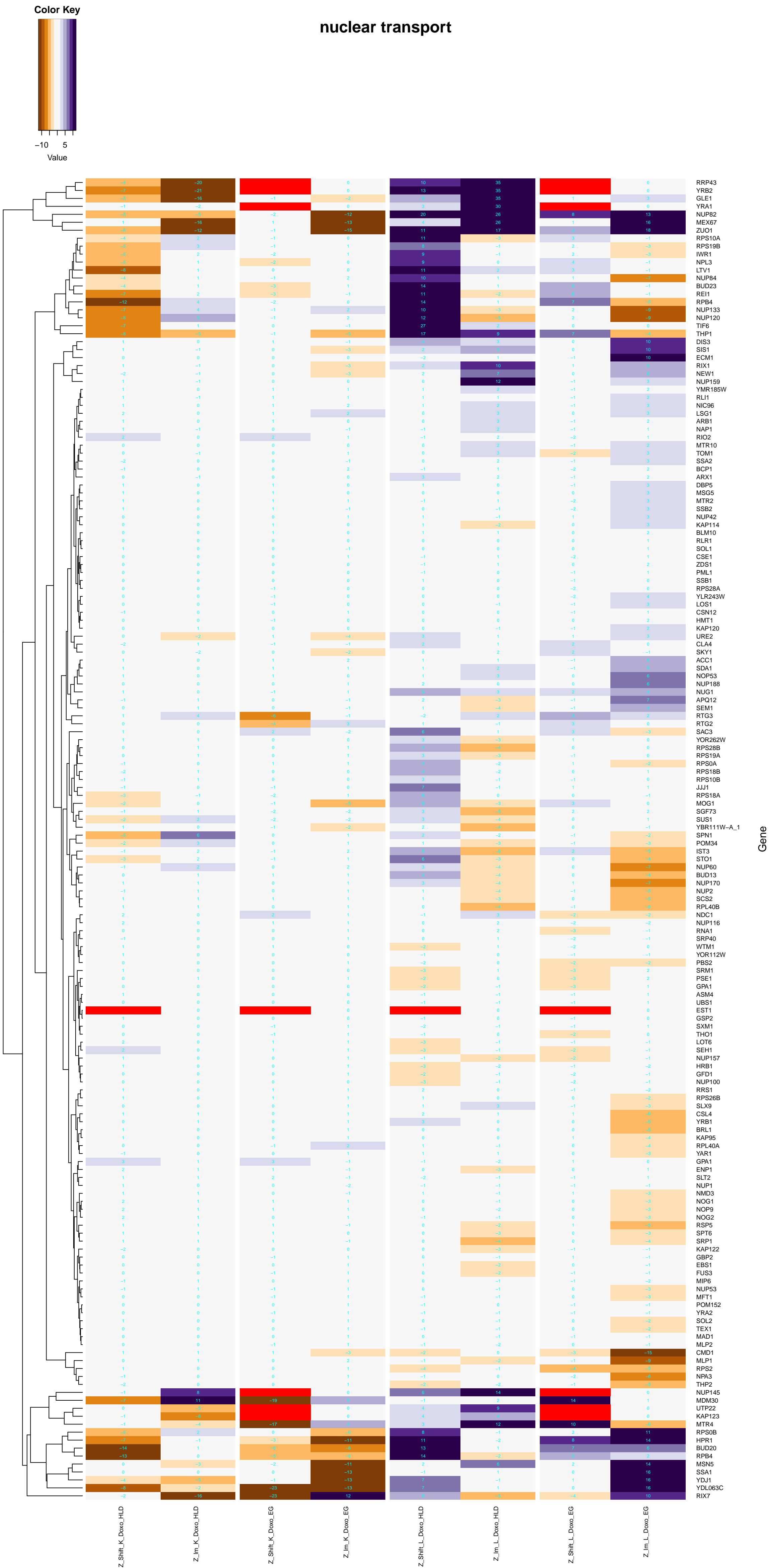

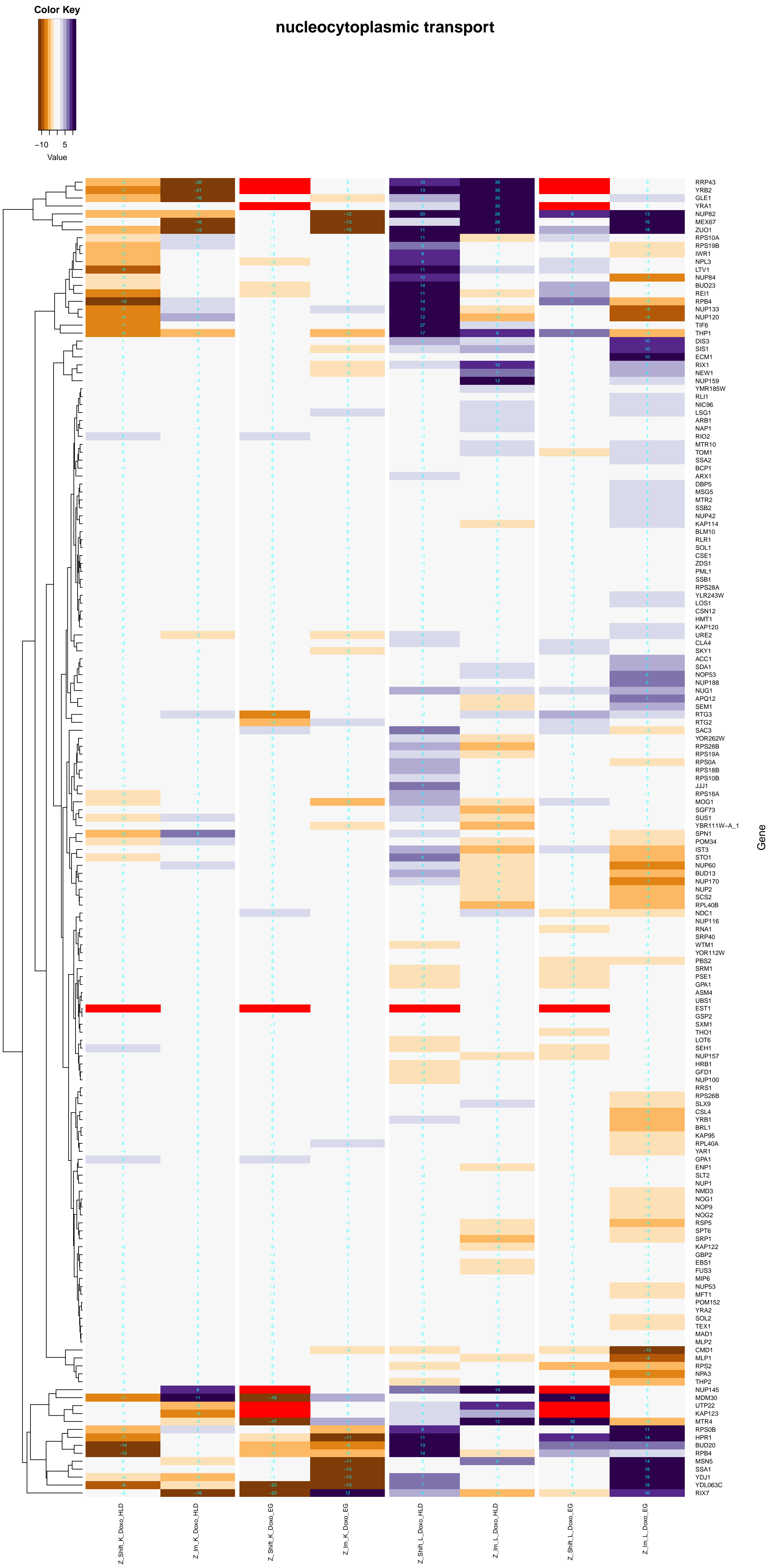

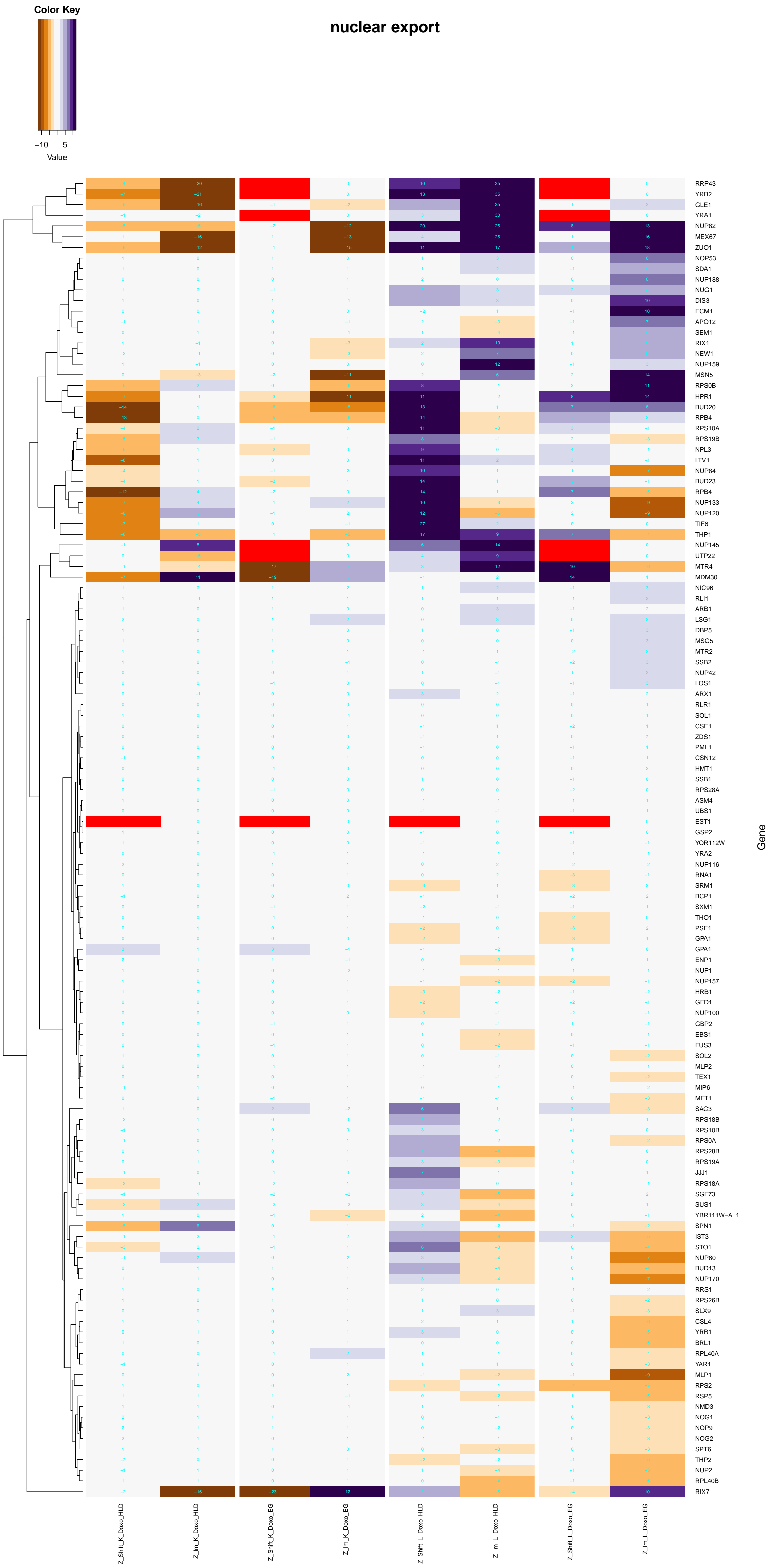

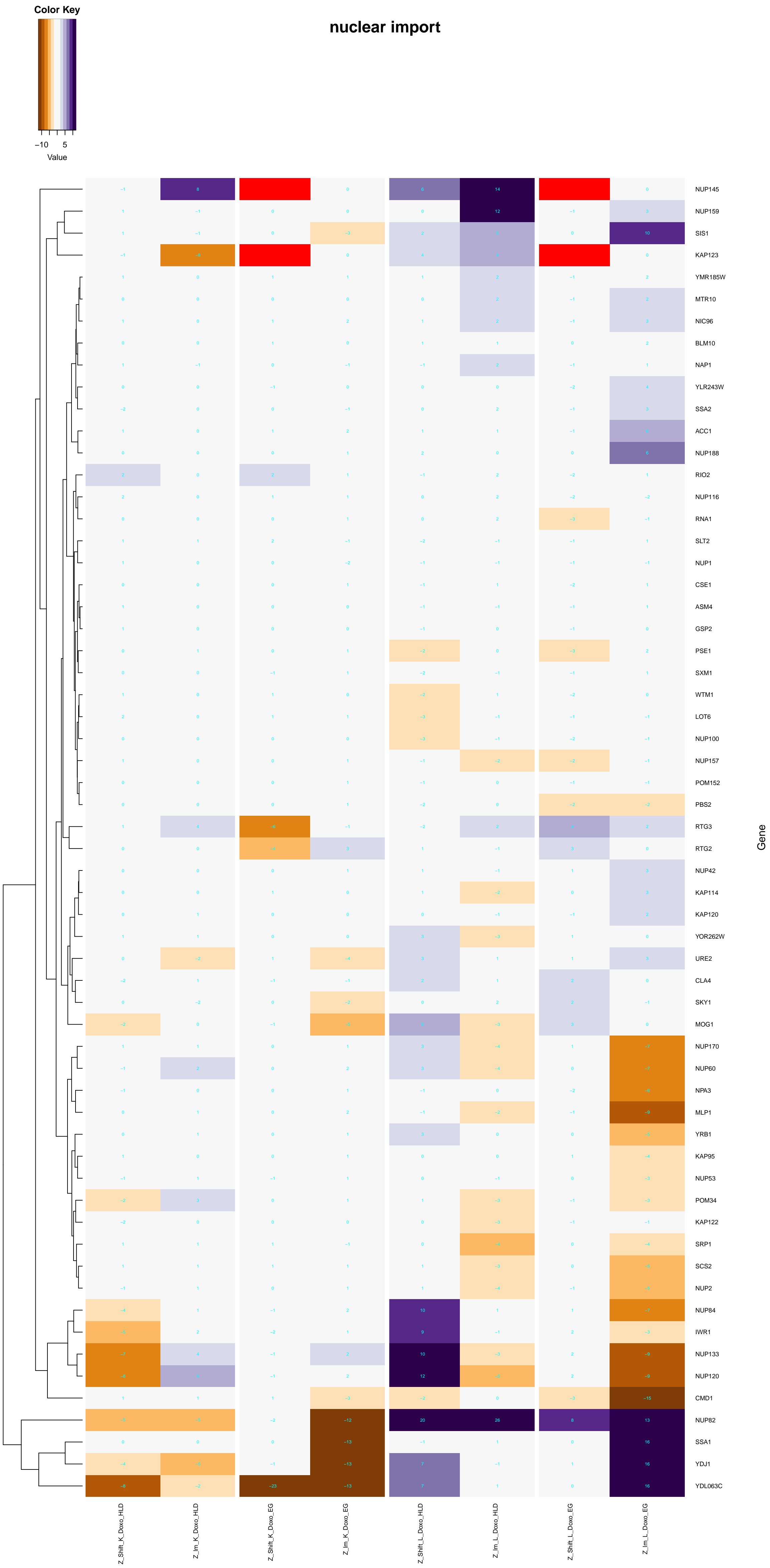

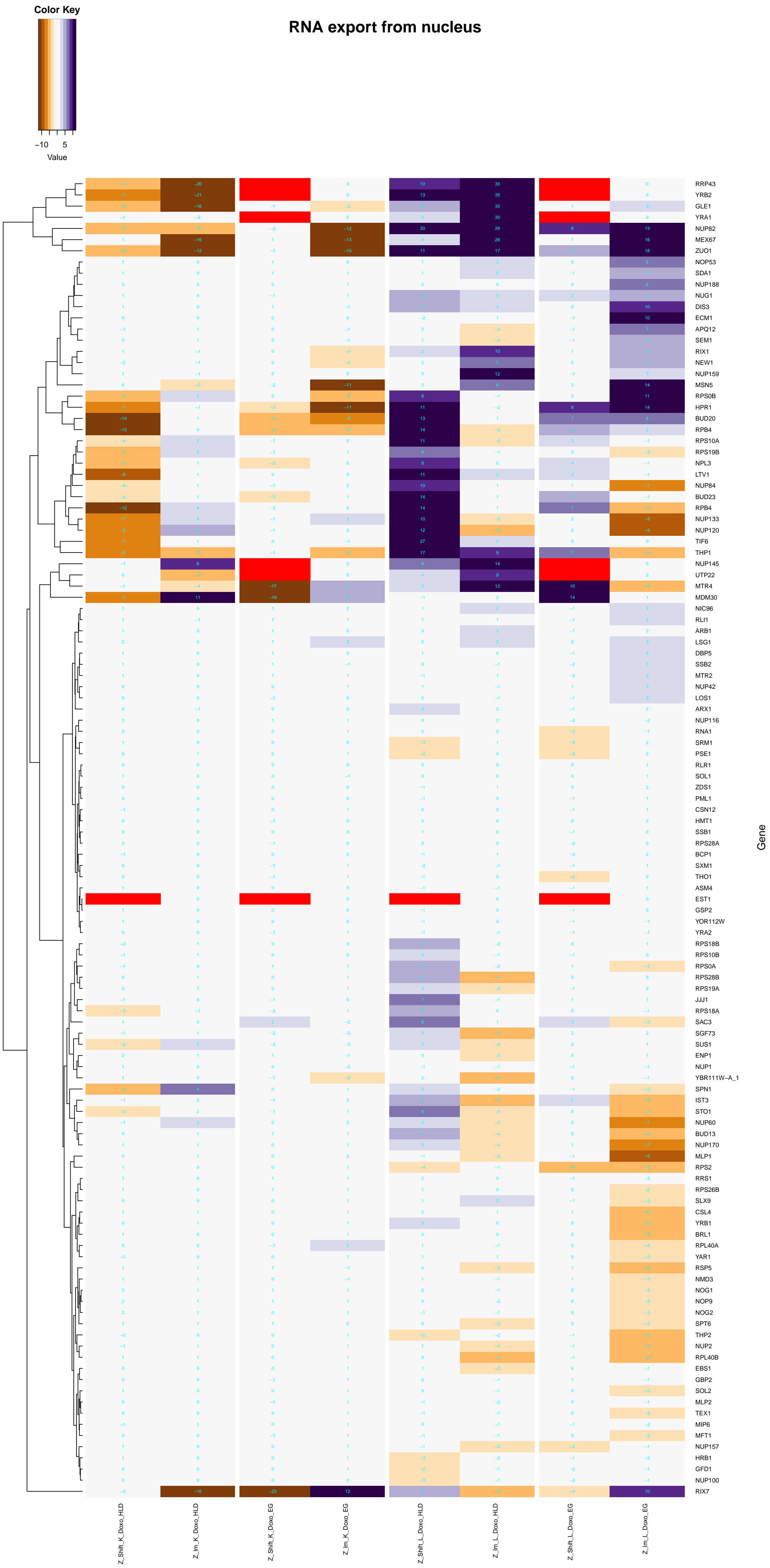



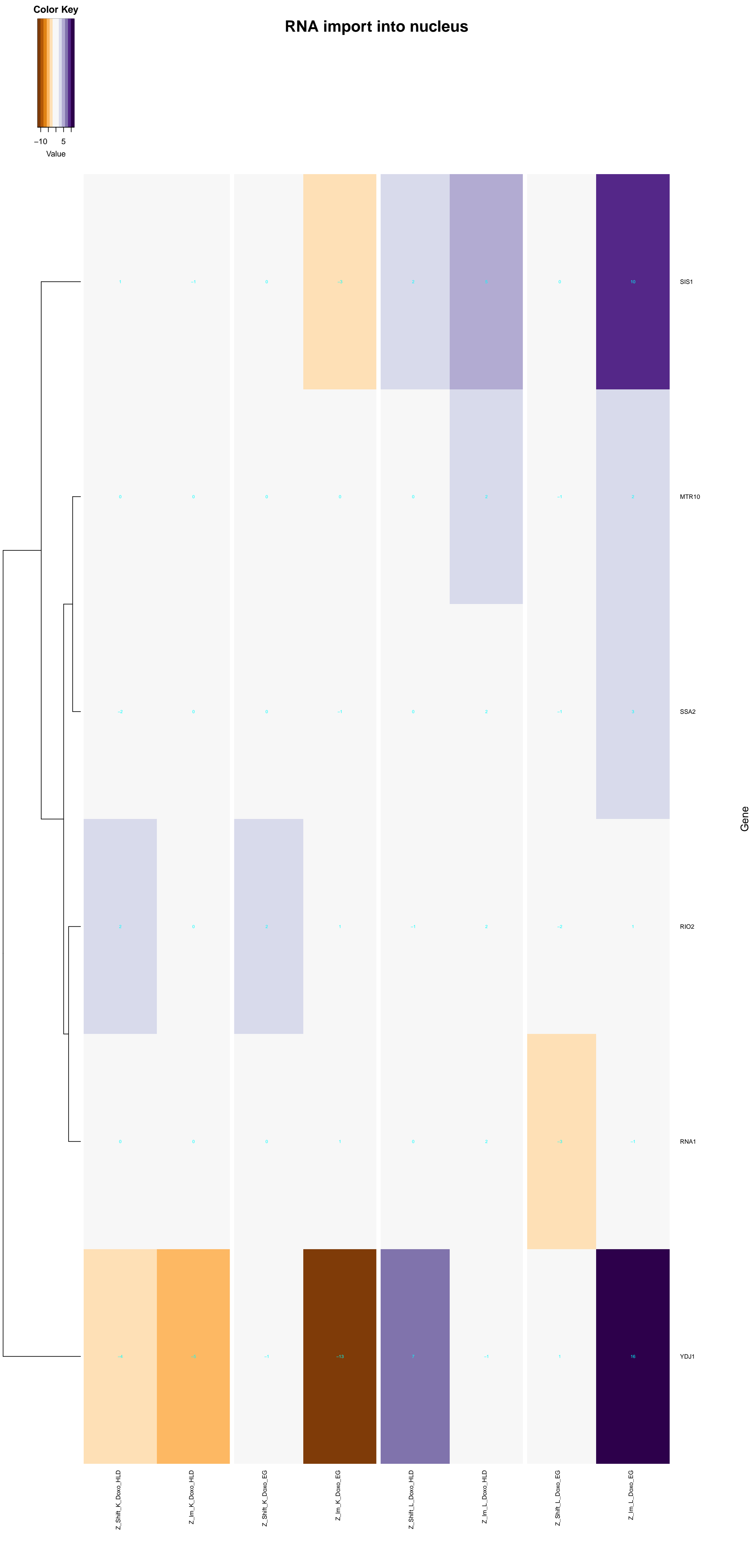

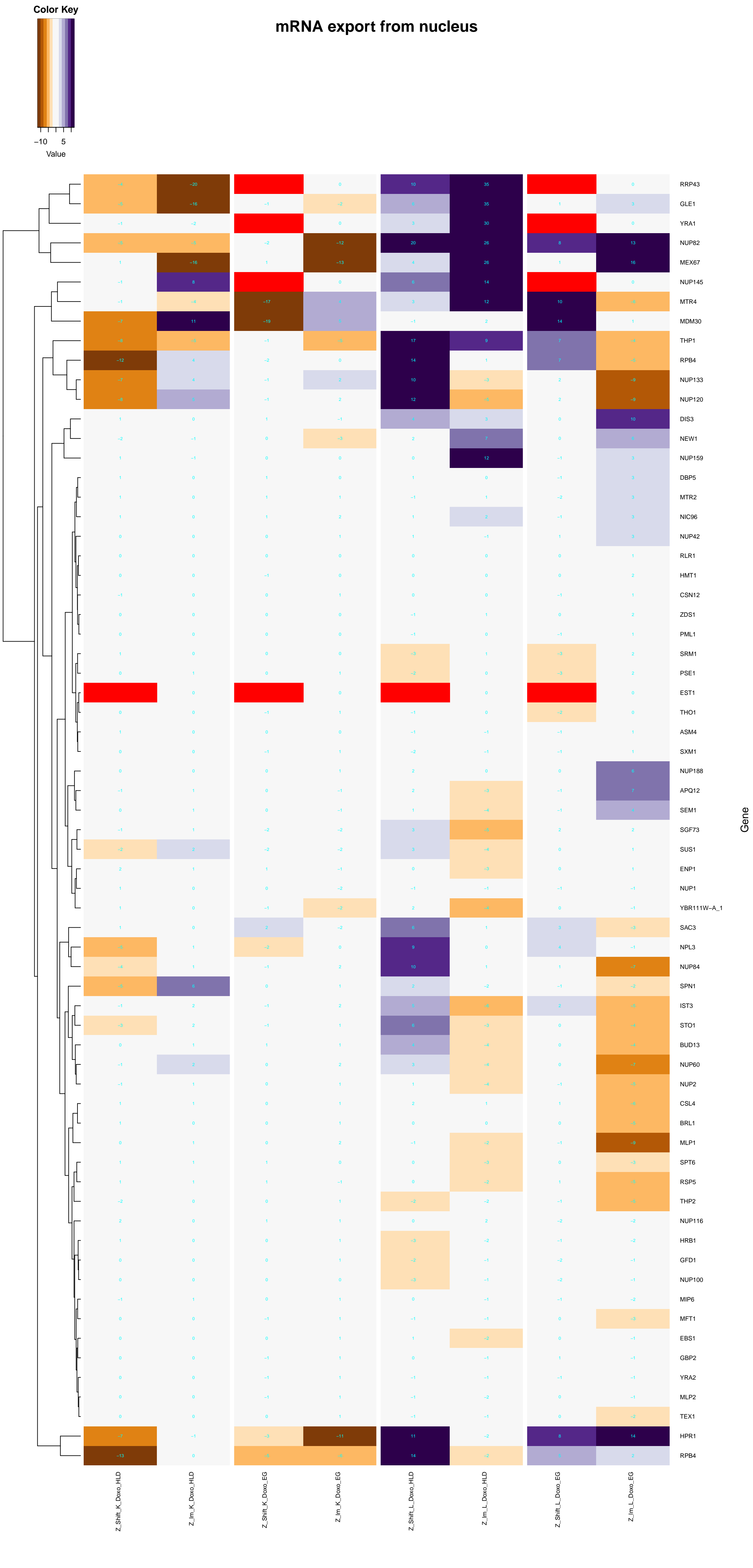

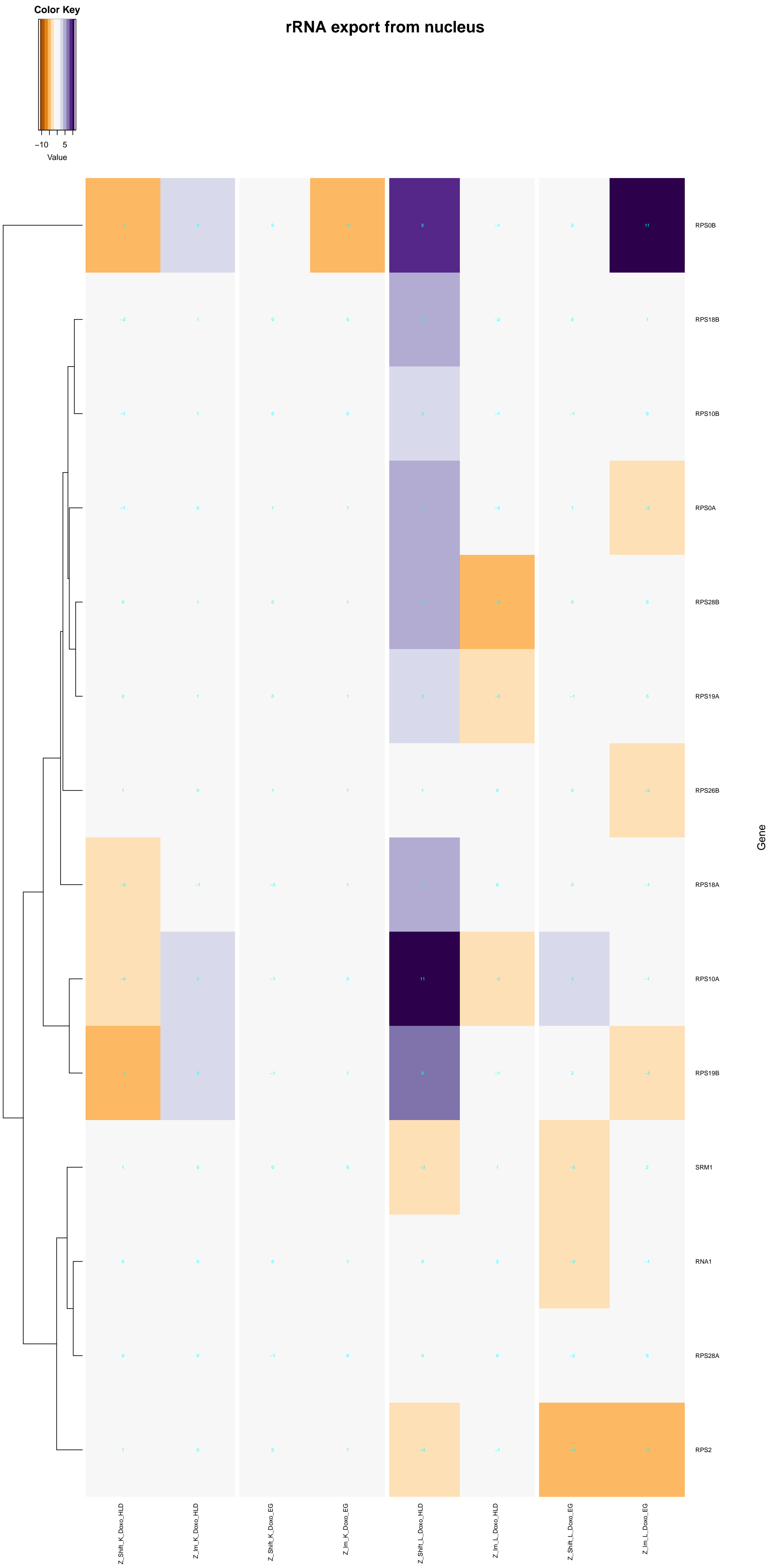

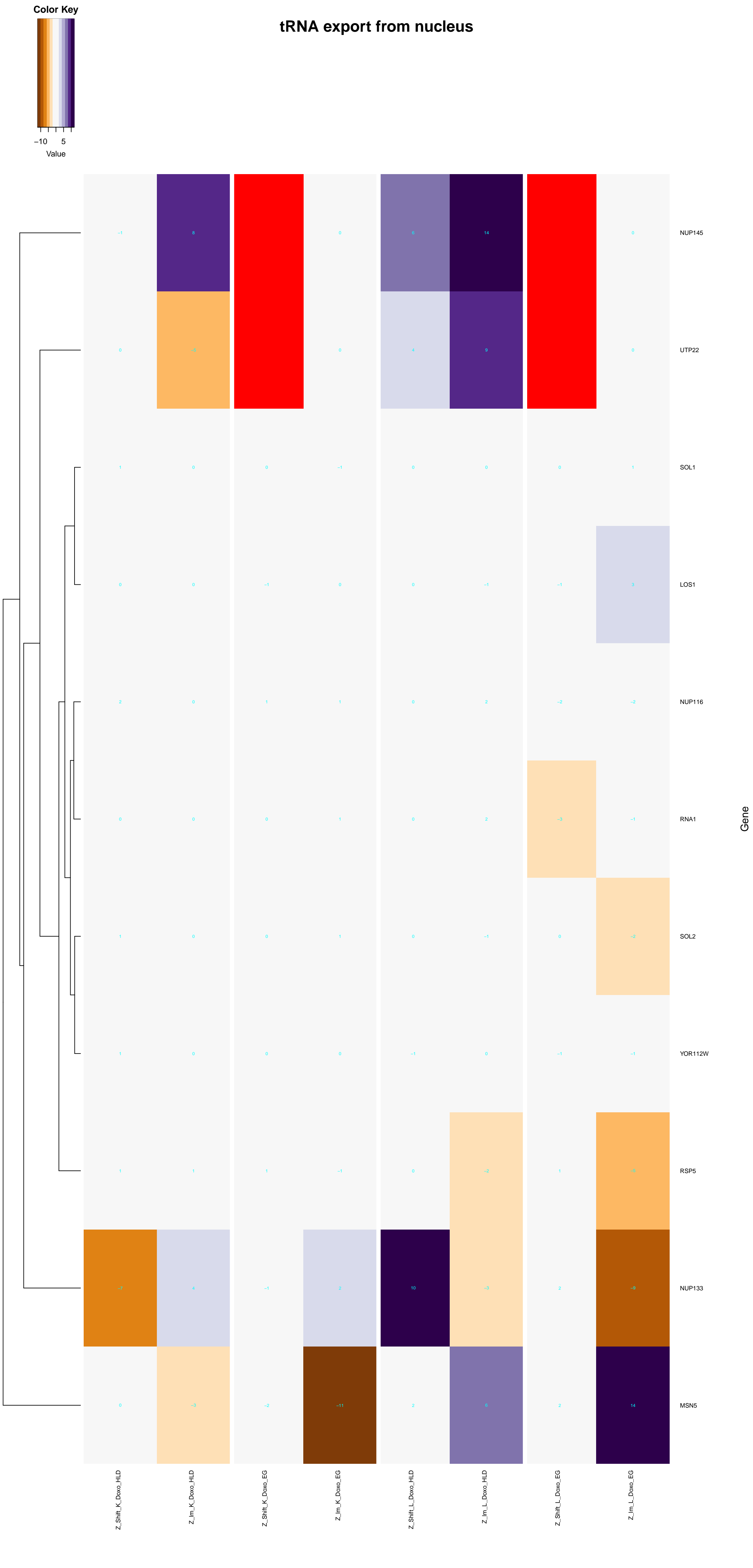



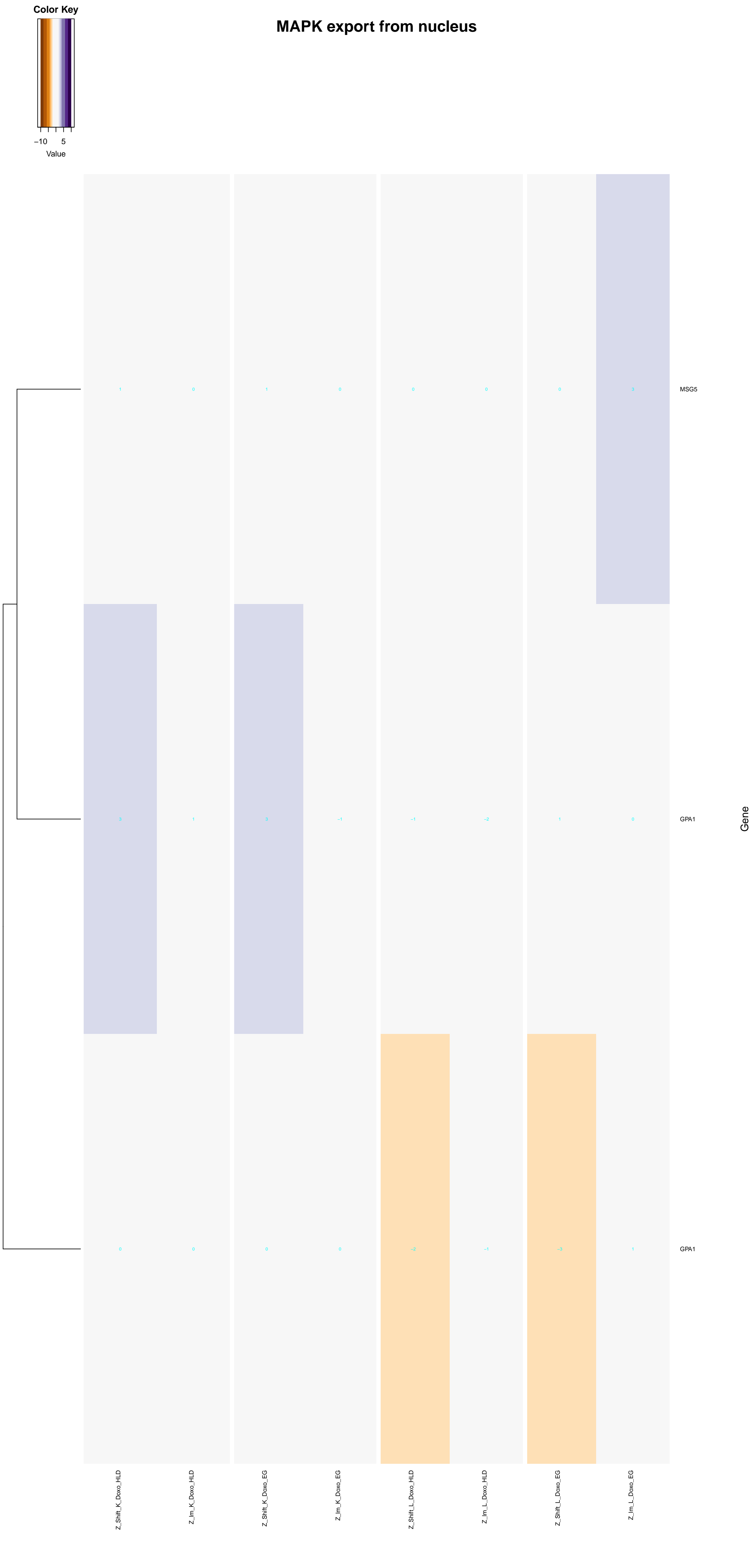

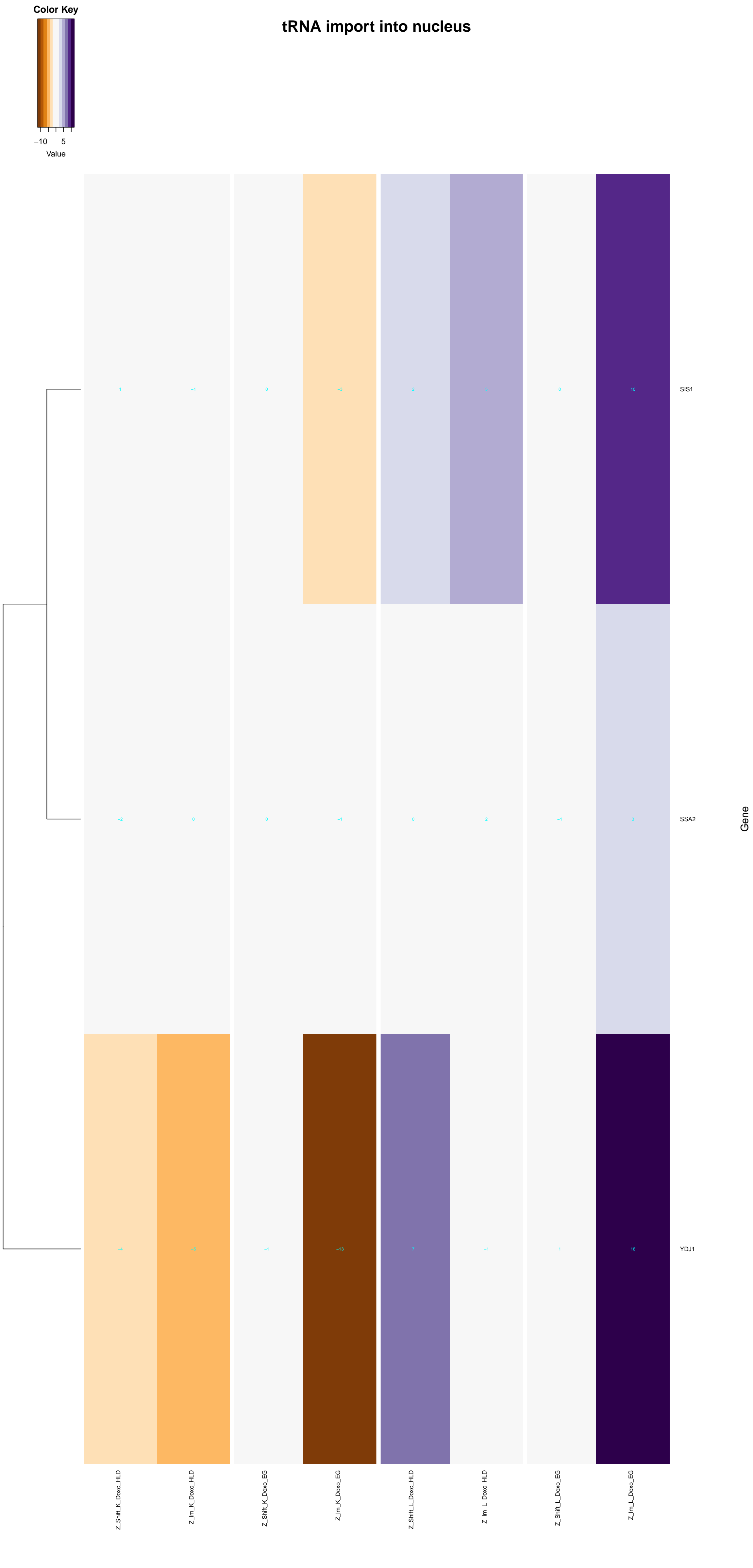

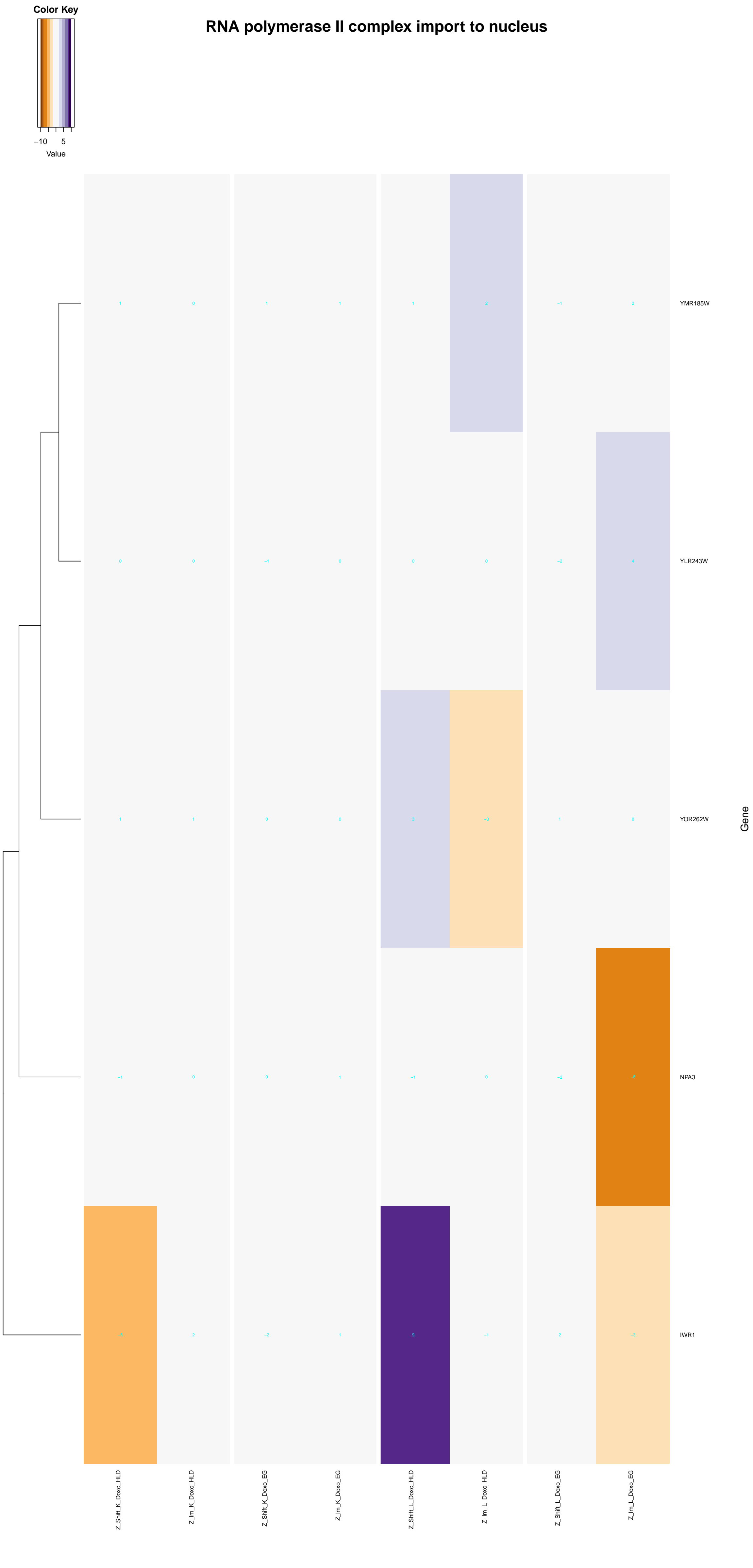

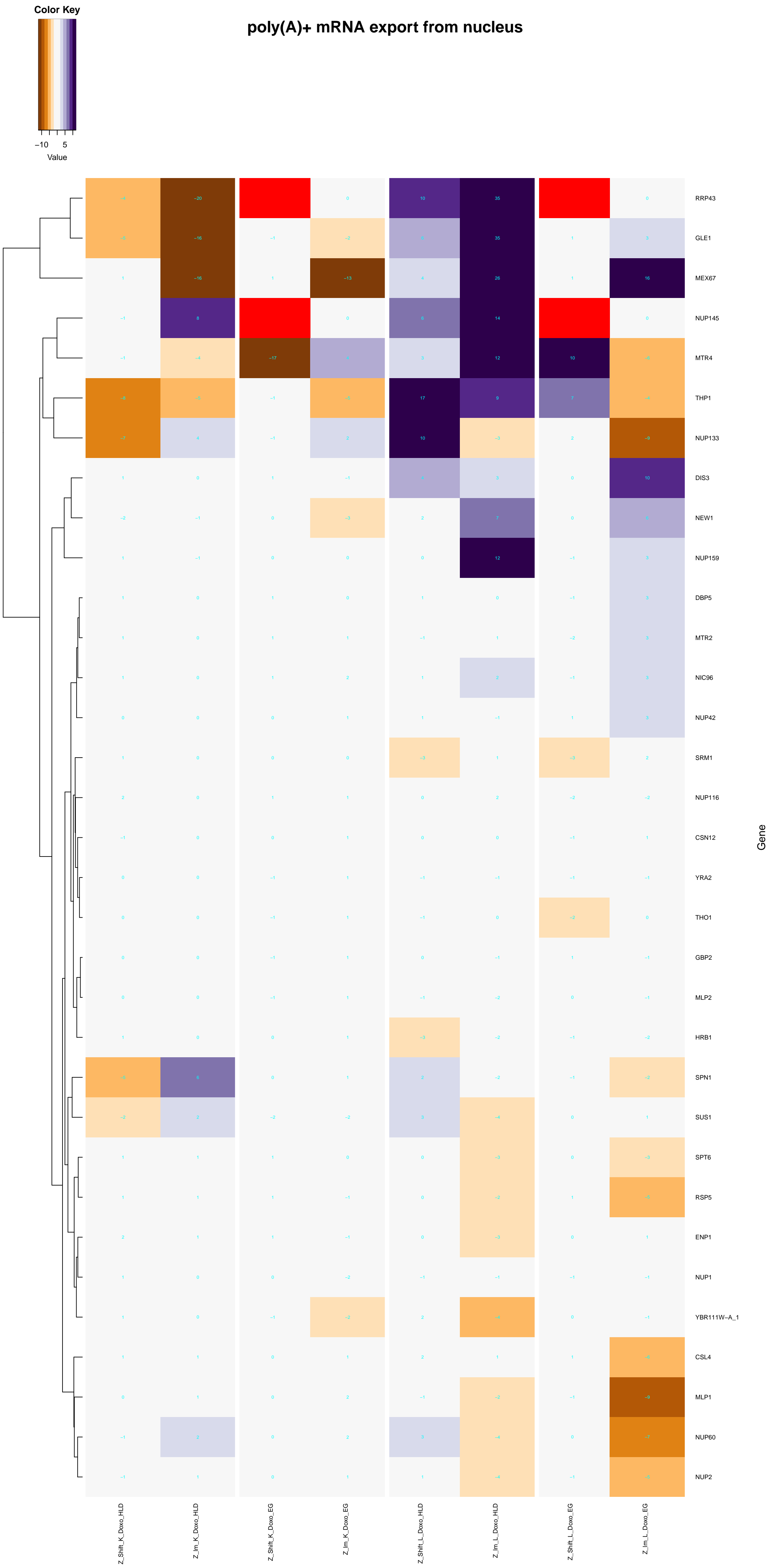

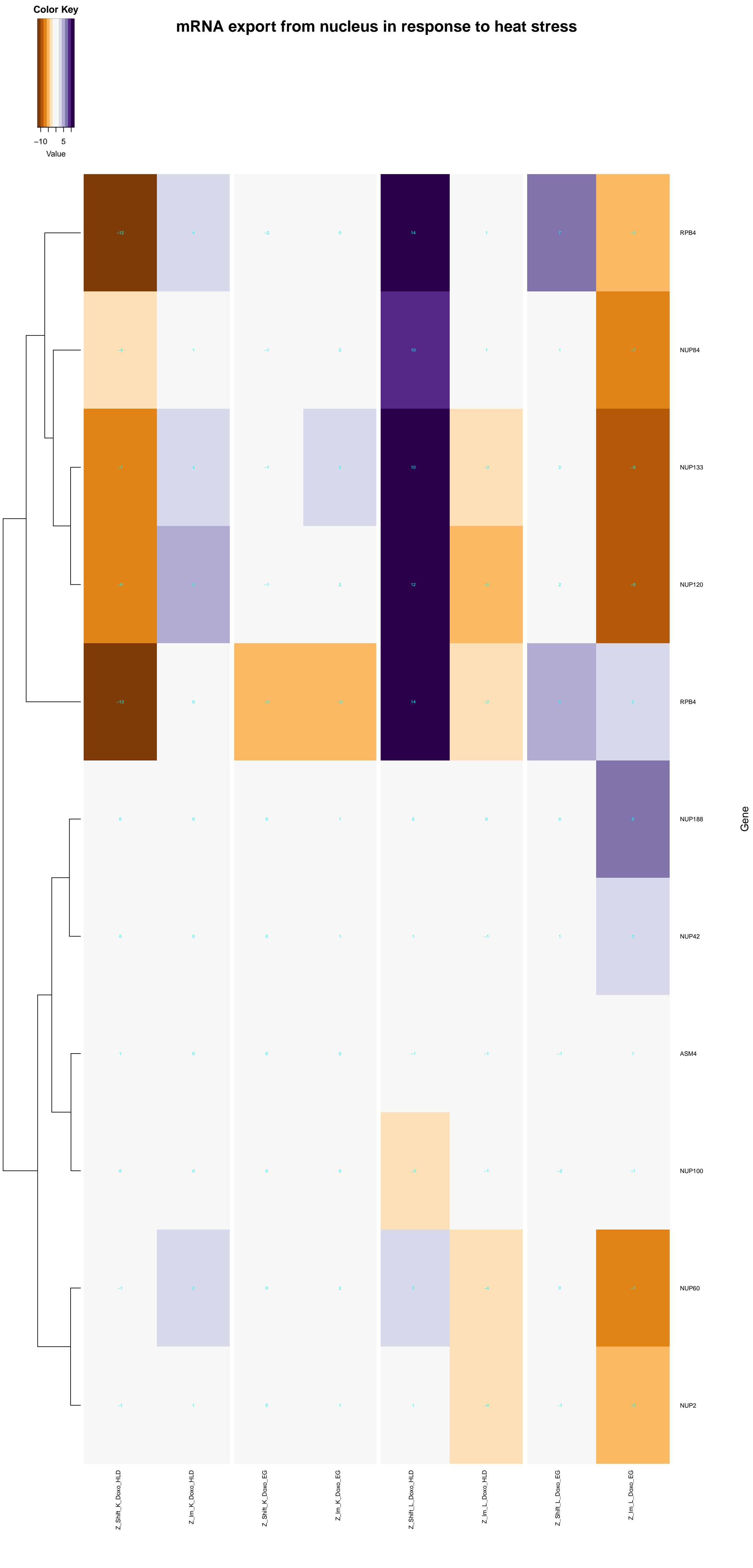

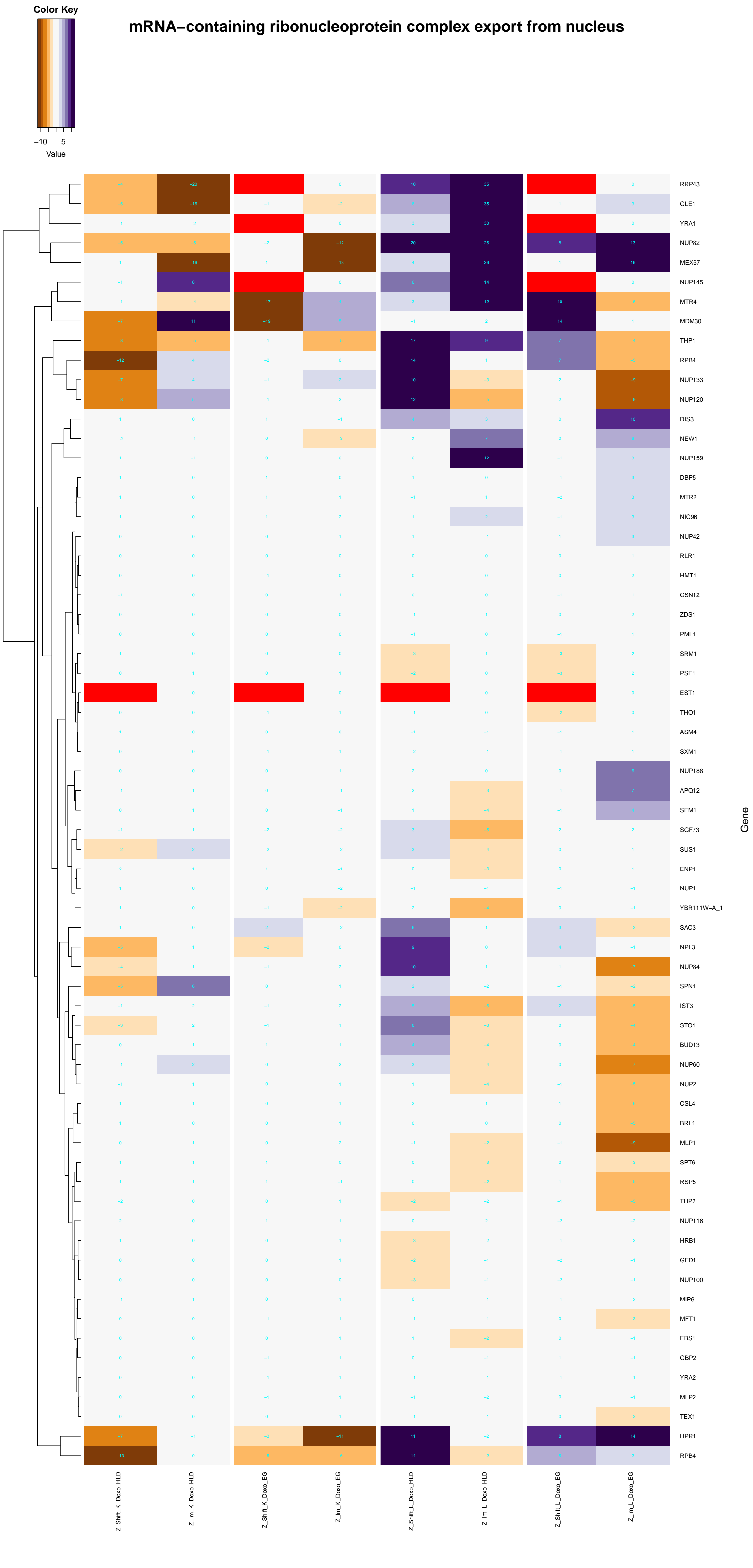

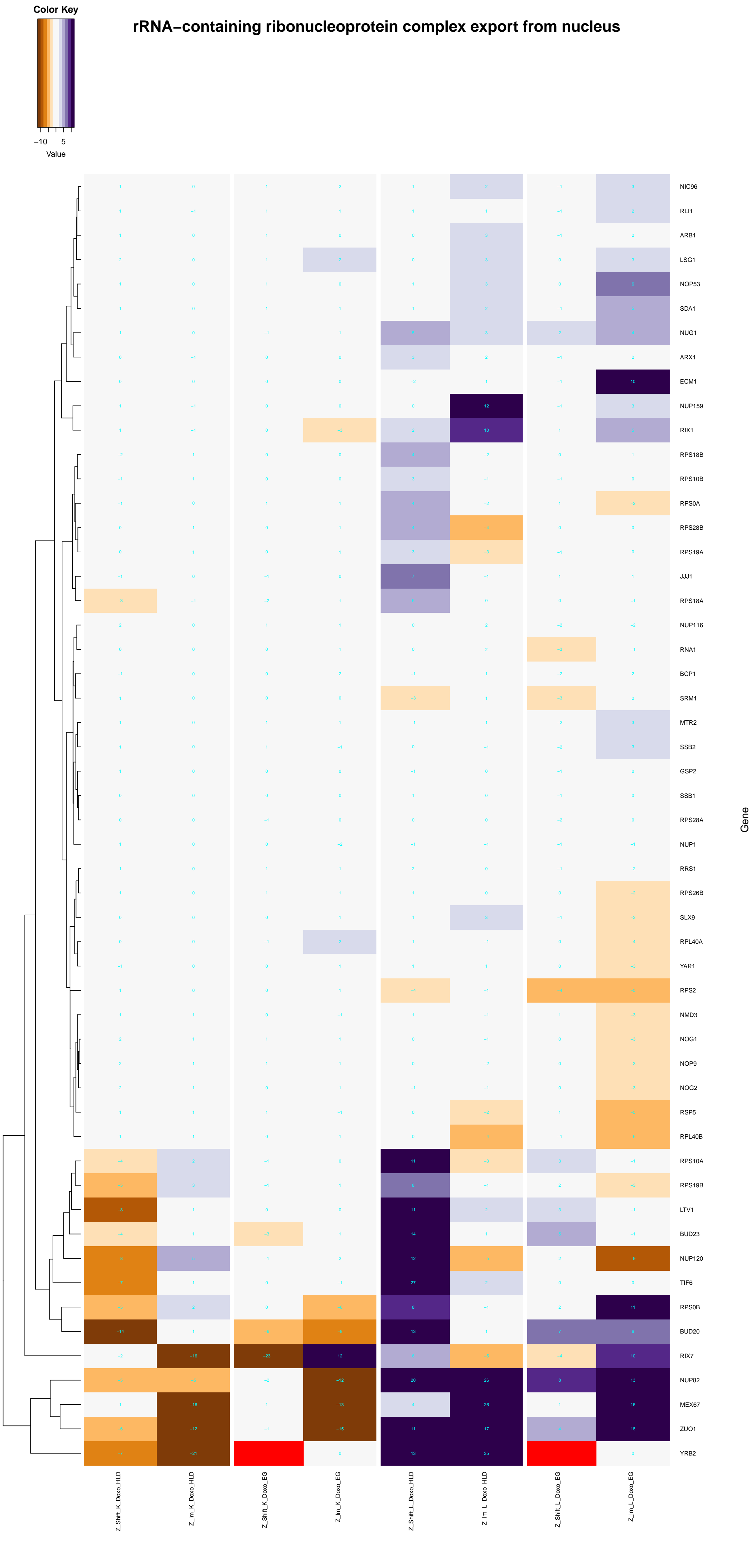

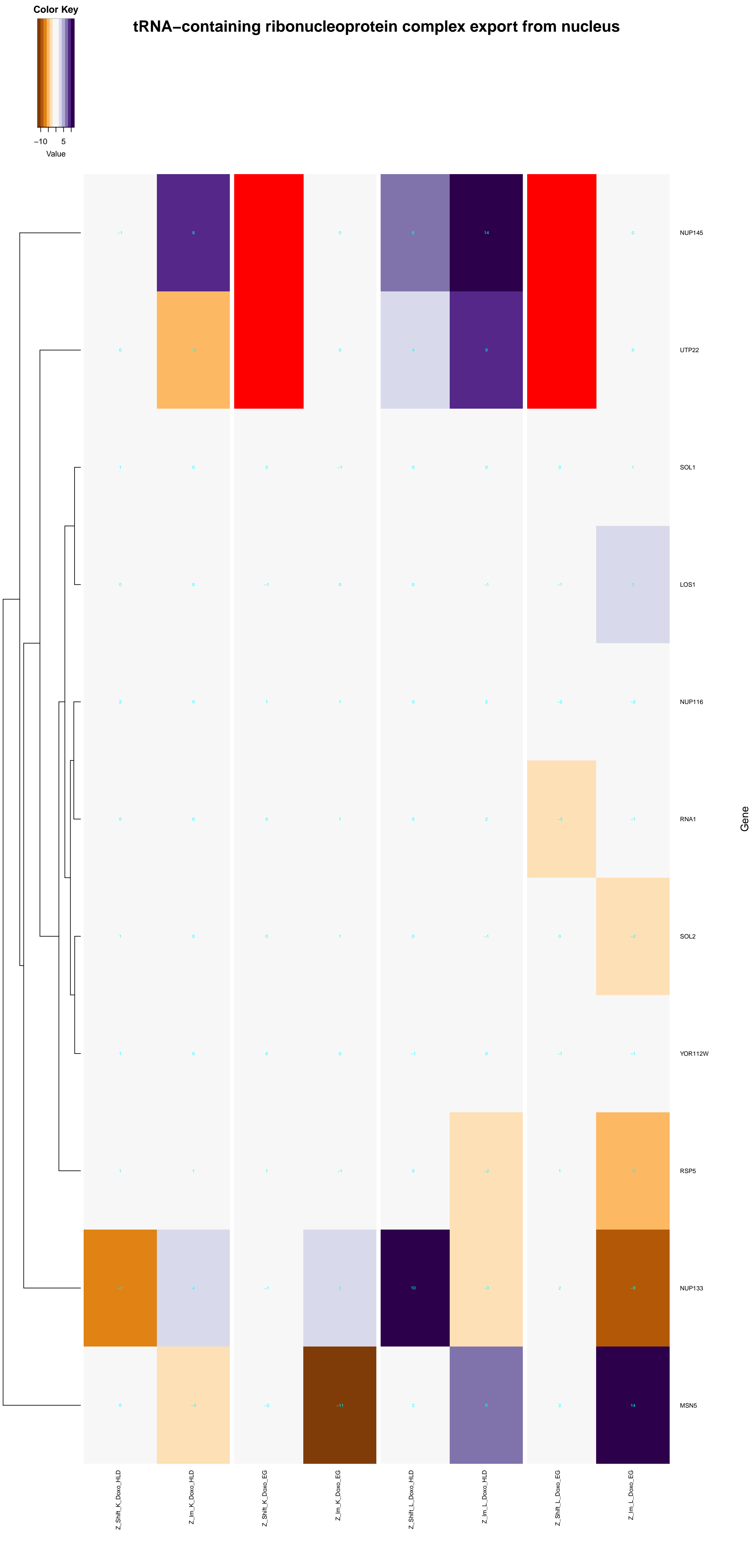

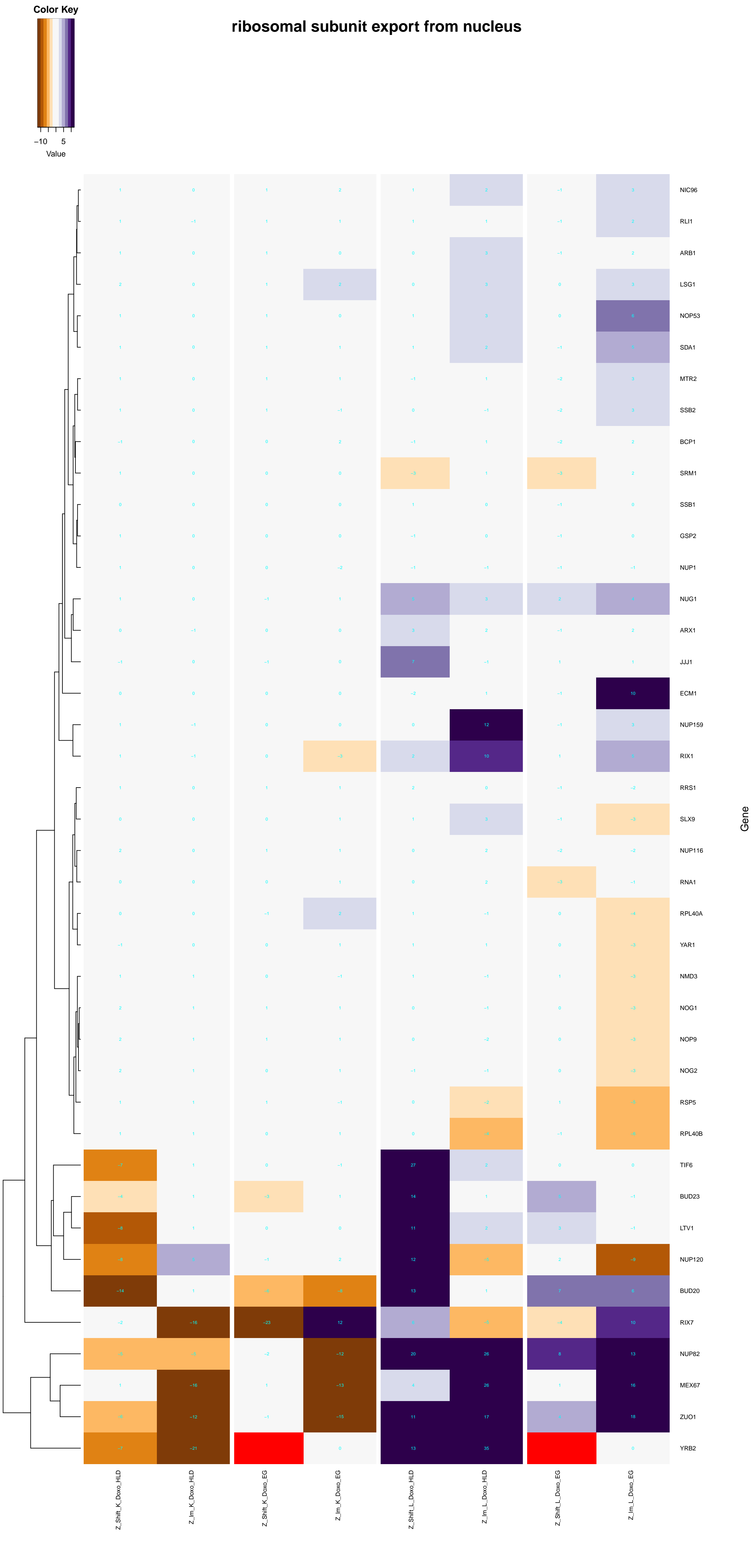

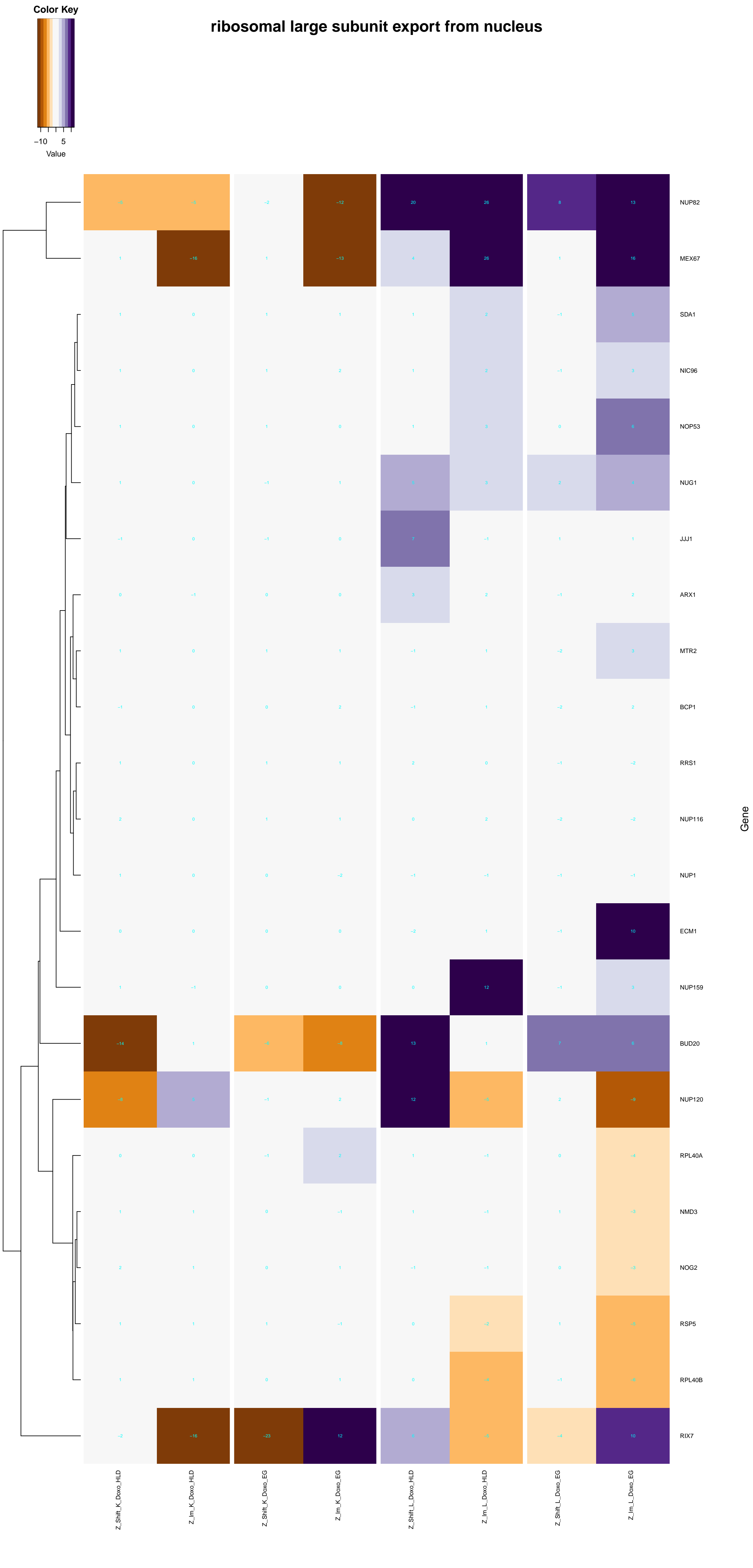

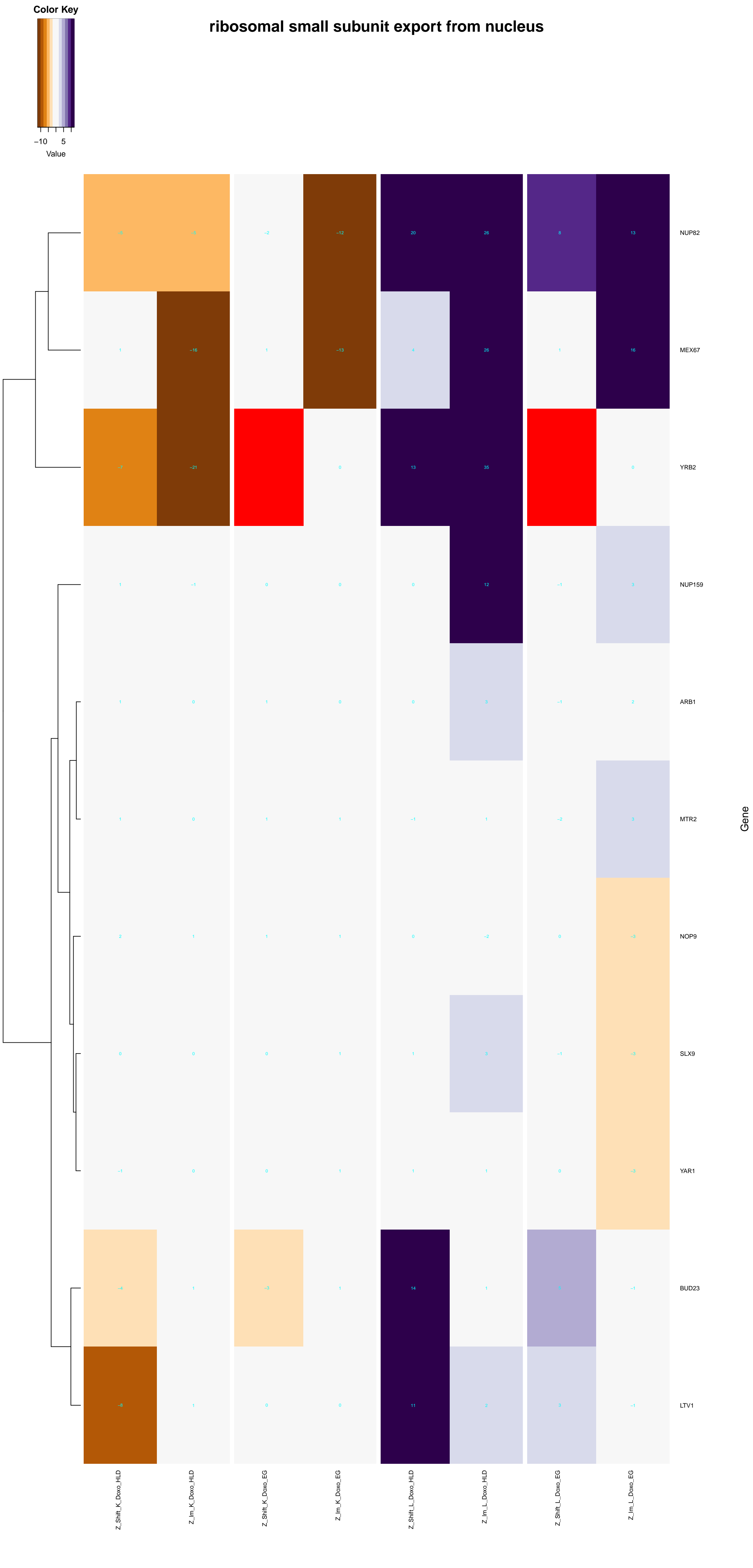

Supplement: Supplementary file 9 — Additional file 9. GO term-specific heatmaps for REMc/GTF-enriched clusters. GO term-specific heatmaps for significant GO process terms were generated as described in methods and Figs. 3 and 4. Any related child terms are presented in subsequent pages of the parent file name. GO terms with more than 100 children, with 2 or fewer genes annotated to the term, or a file size over 300KB are not shown. All heatmaps are generated with the same layout (see Figs. 3 and 4). [file 40170_2019_201_MOESM9_ESM.bz2 › Additional_File9_GOTermHeatmaps/Additional_File9_GOTermHeatmap/nuclear_transport.pdf]

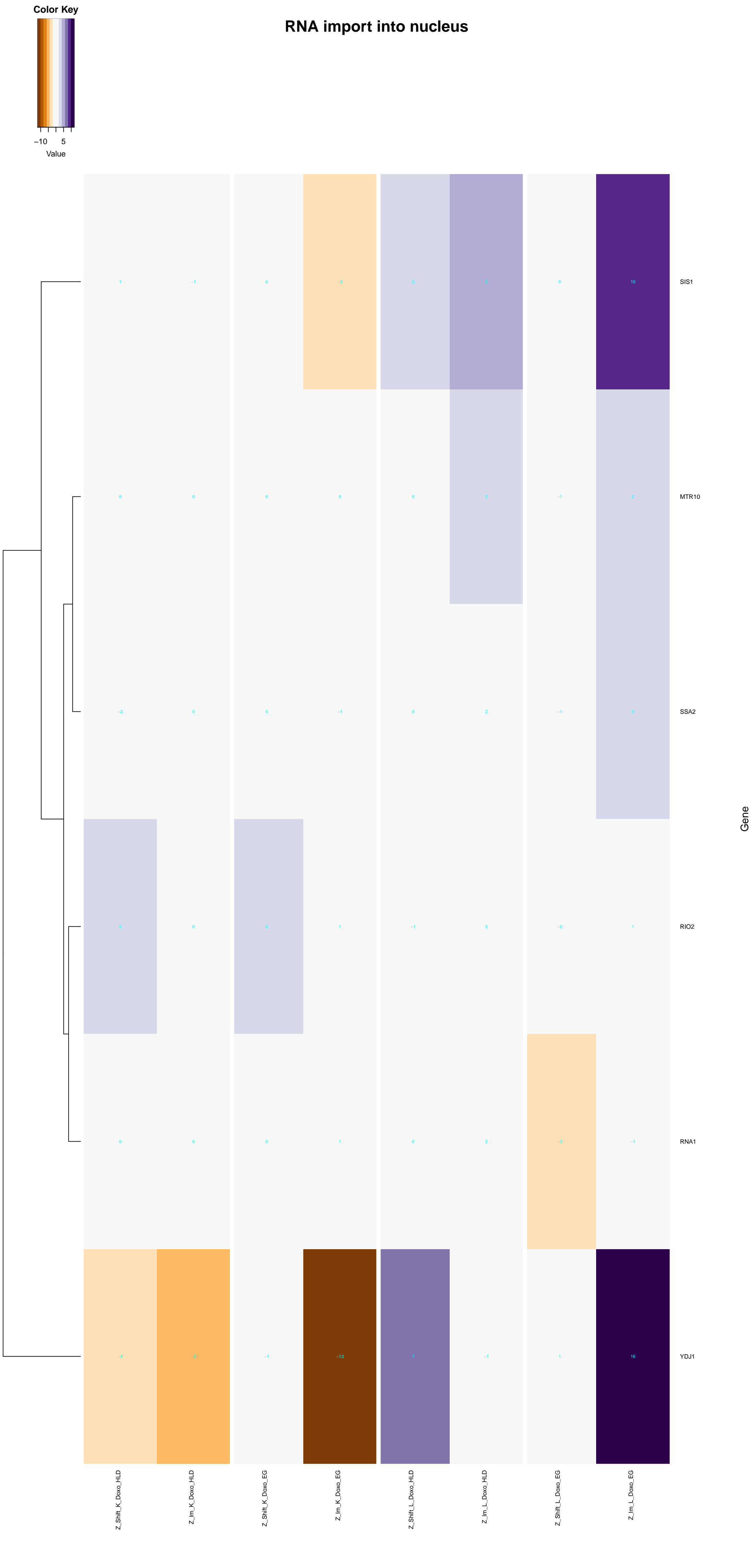

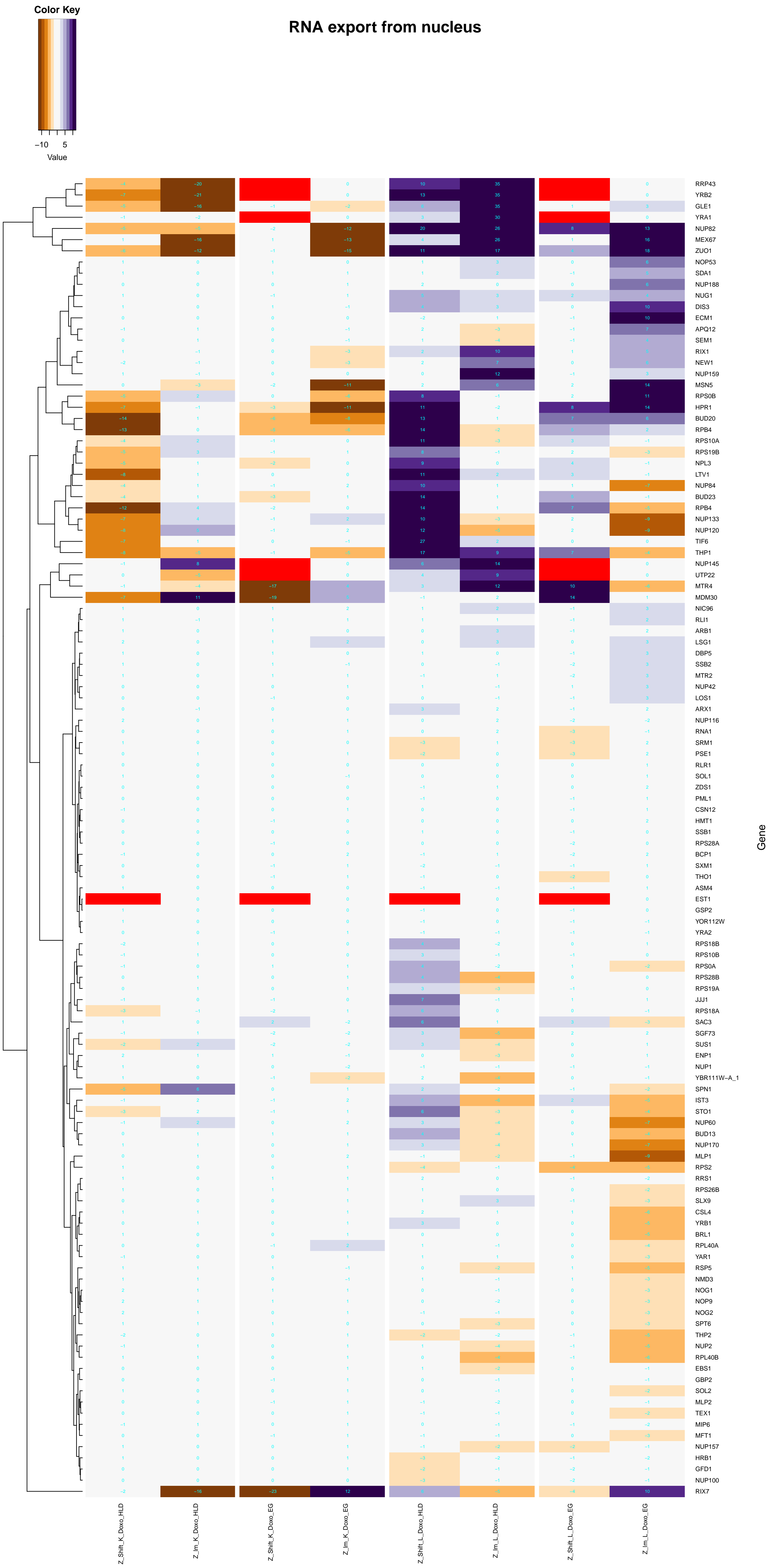

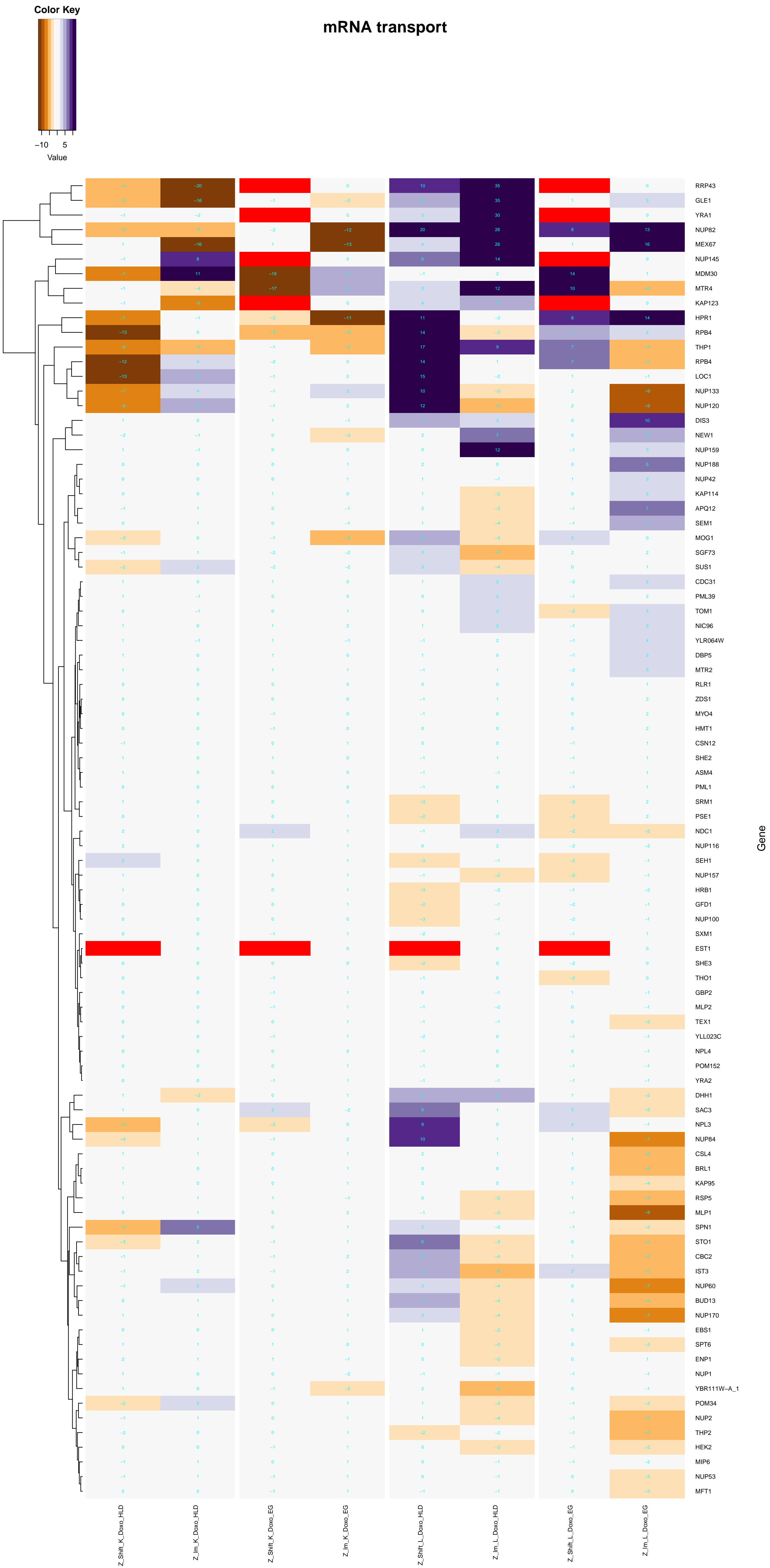

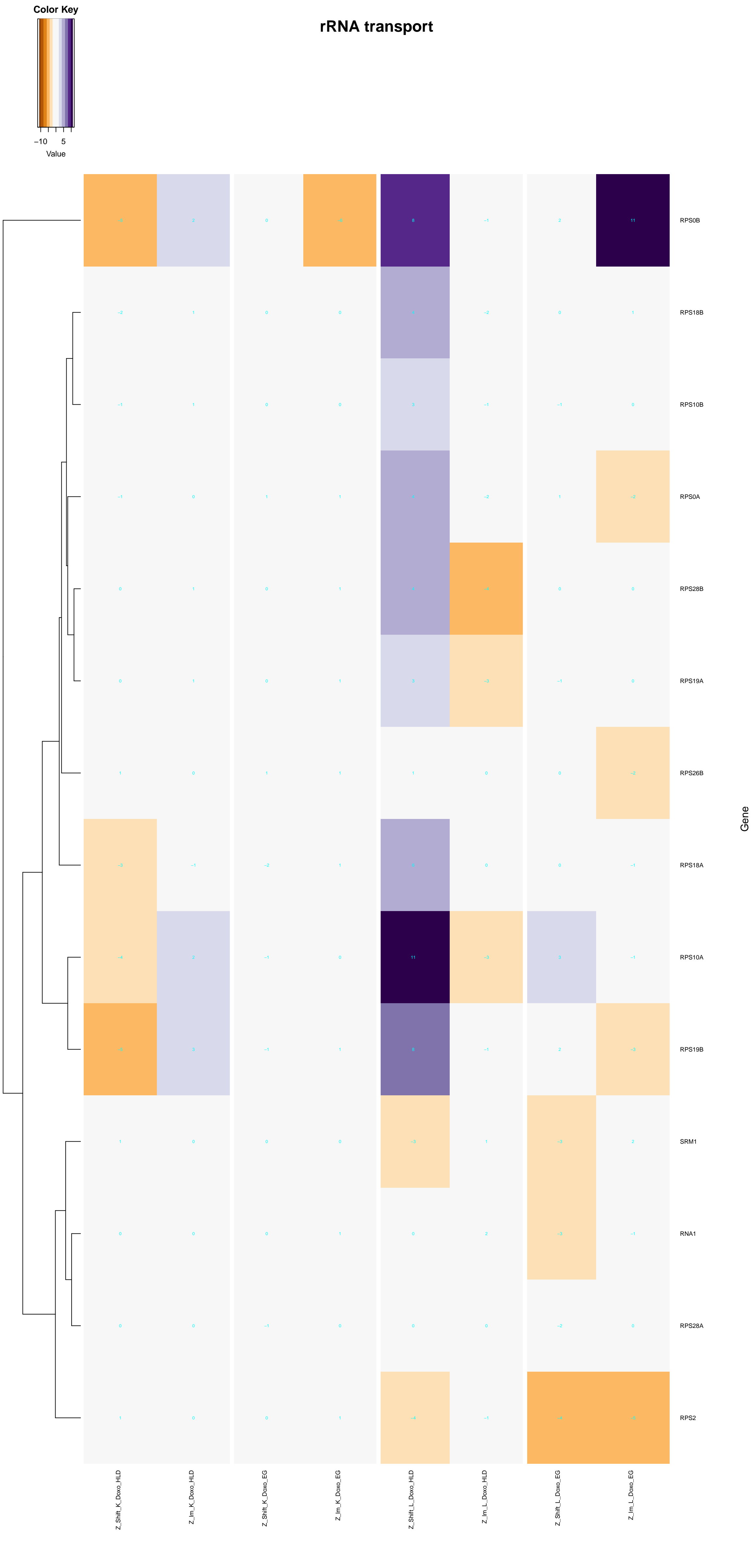

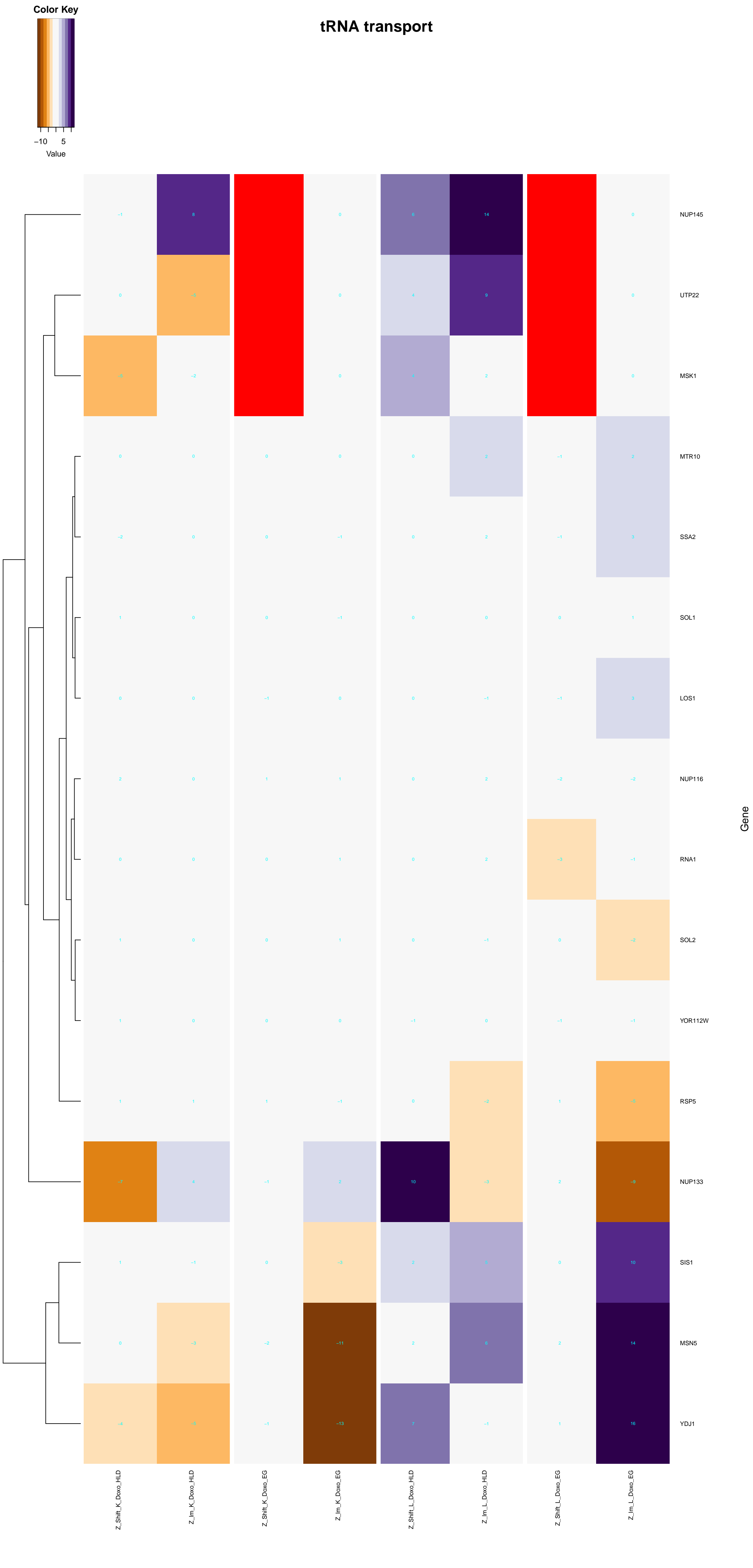

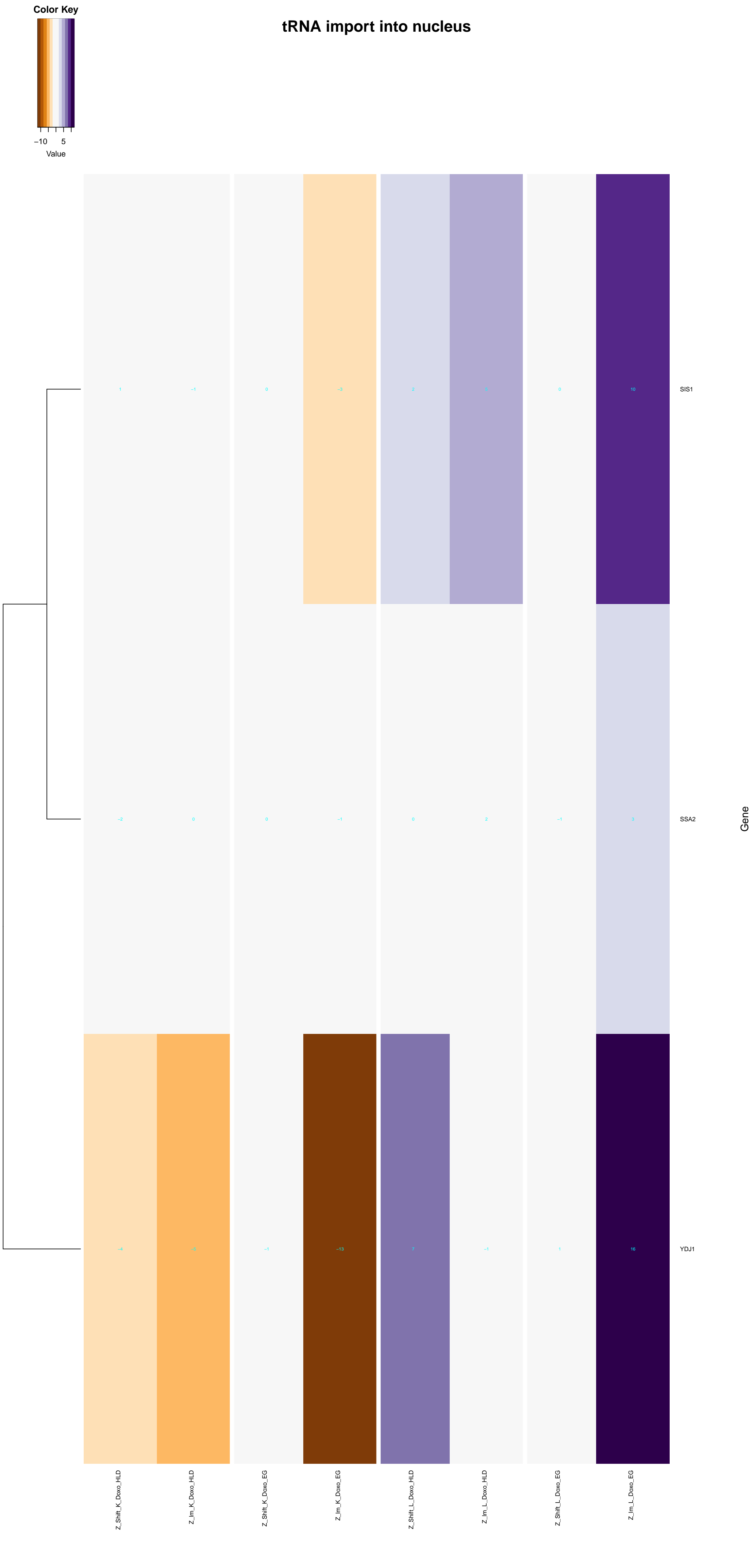

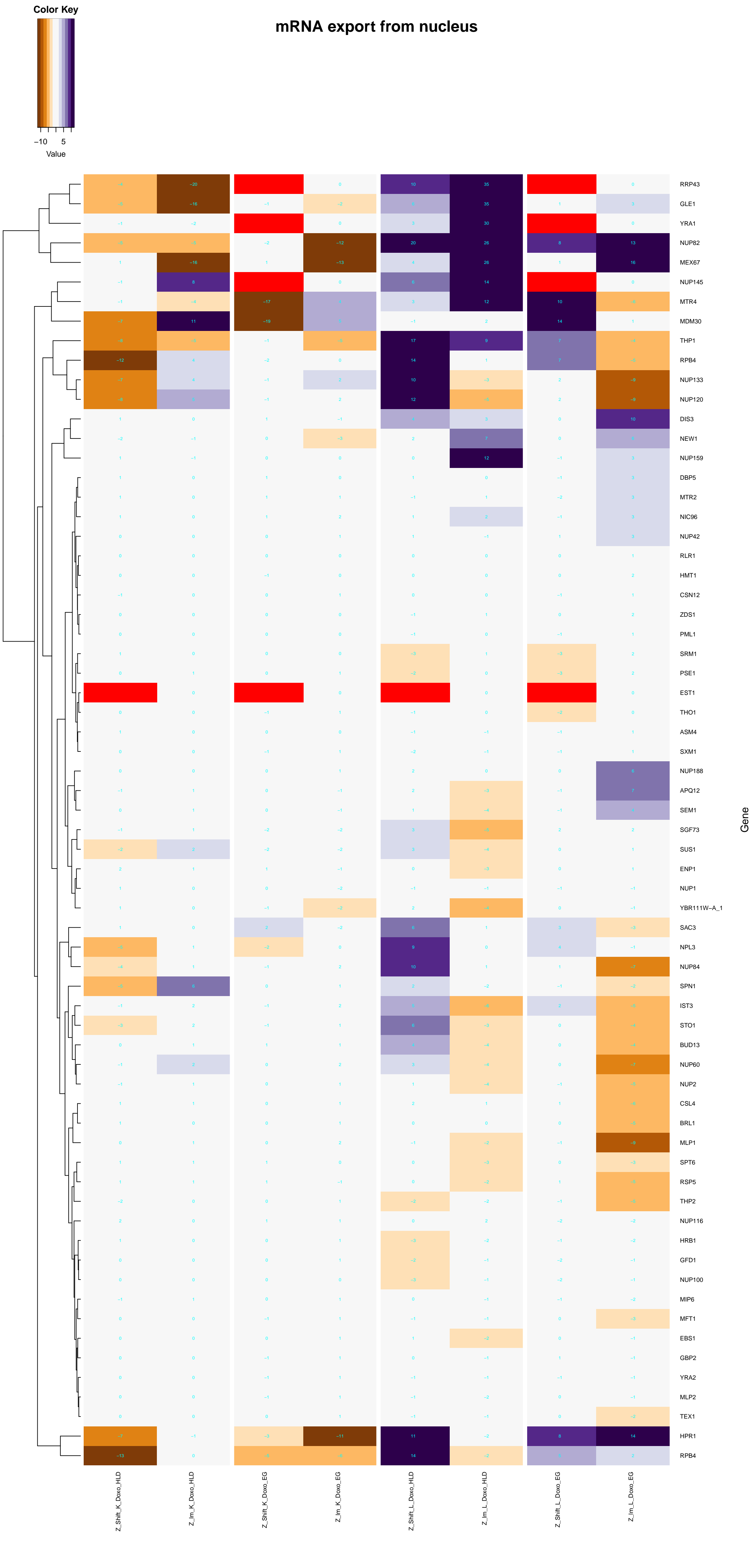

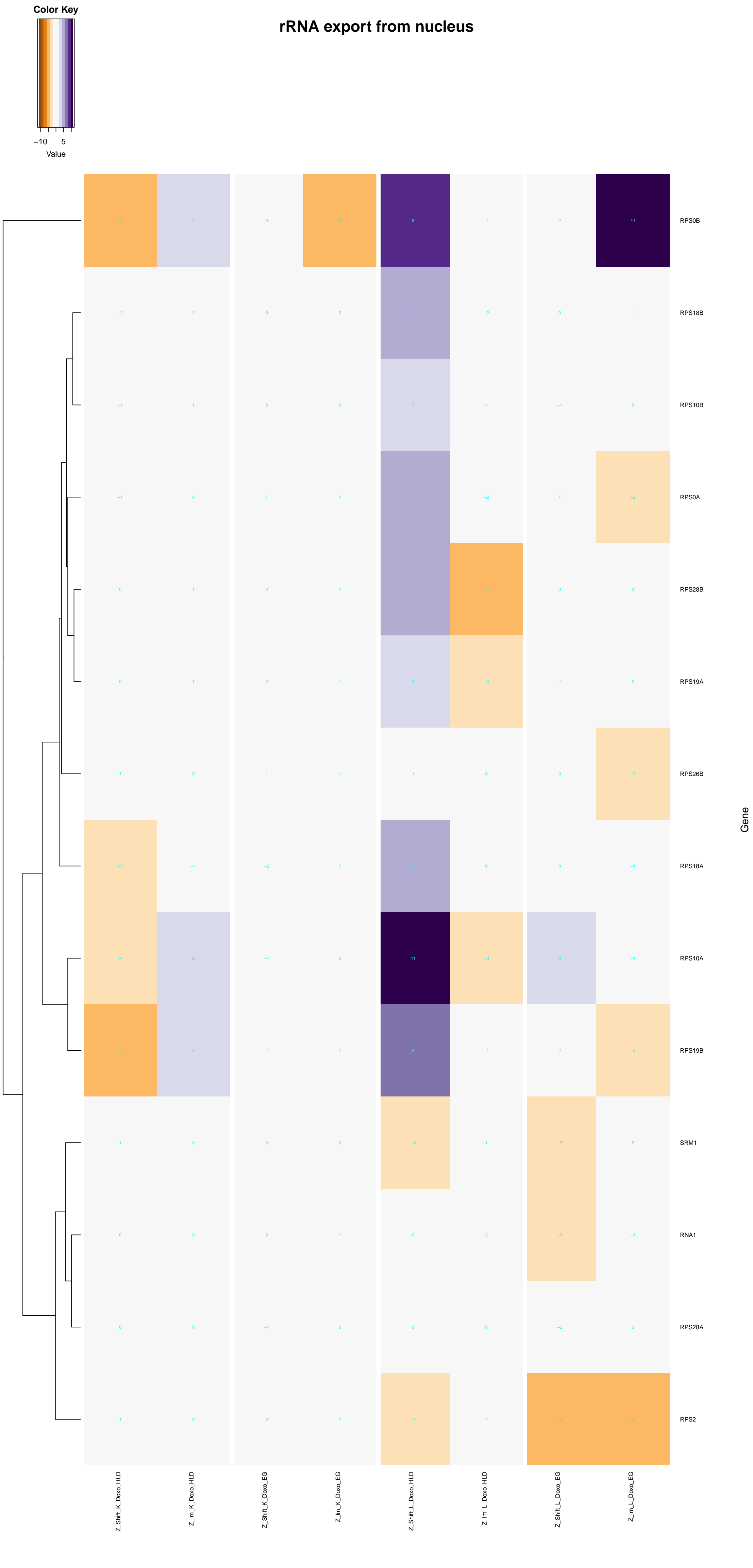

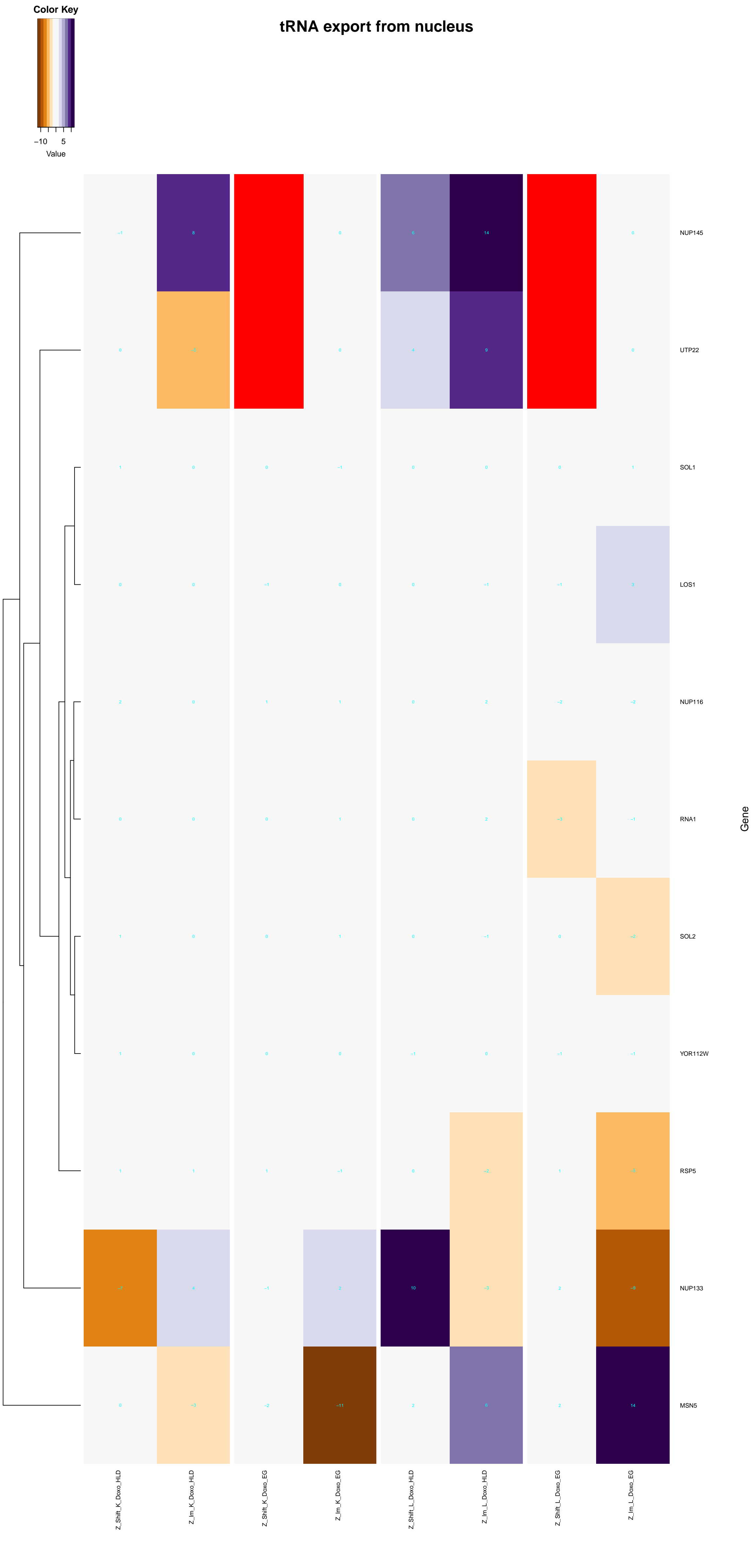

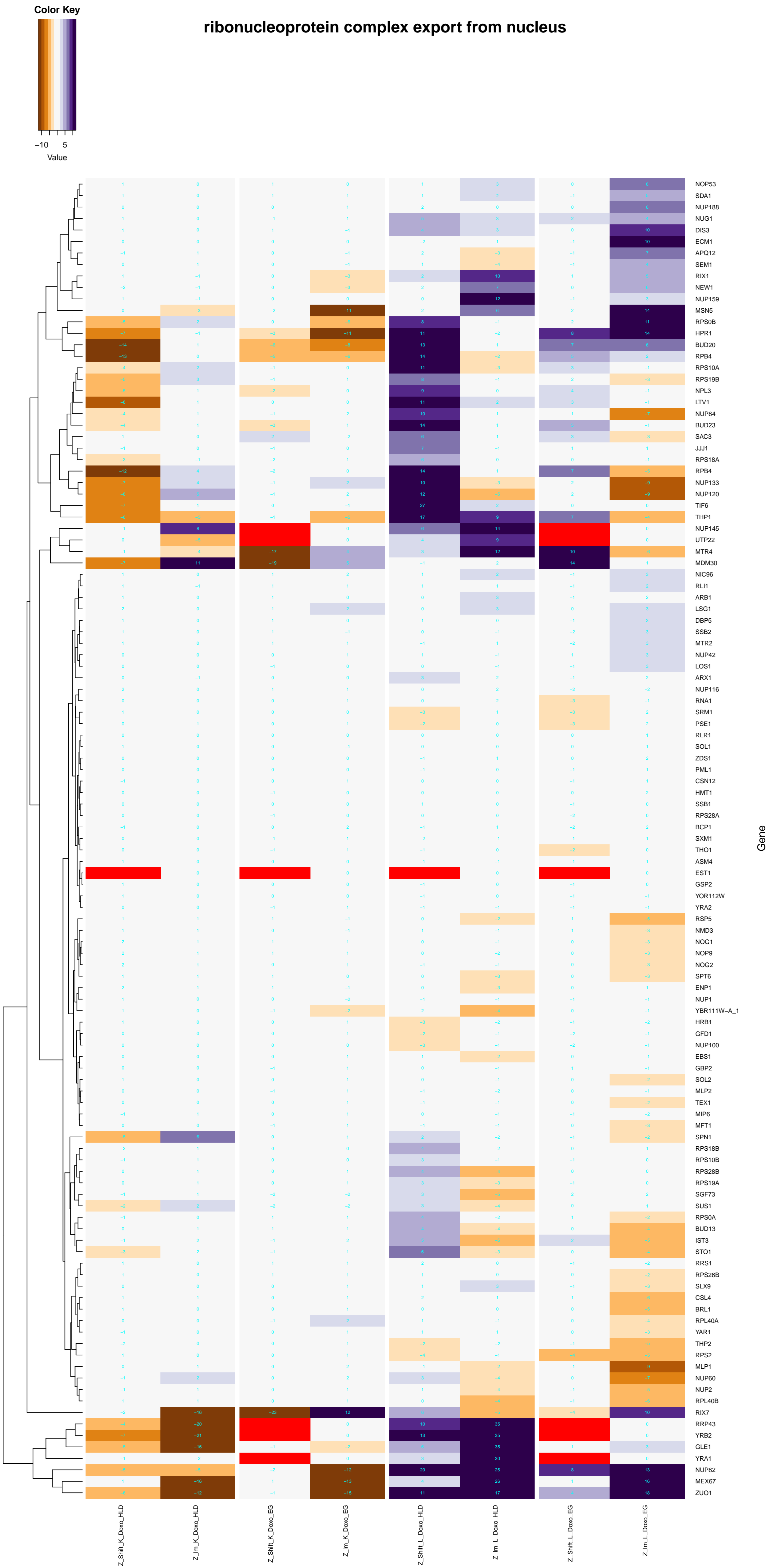

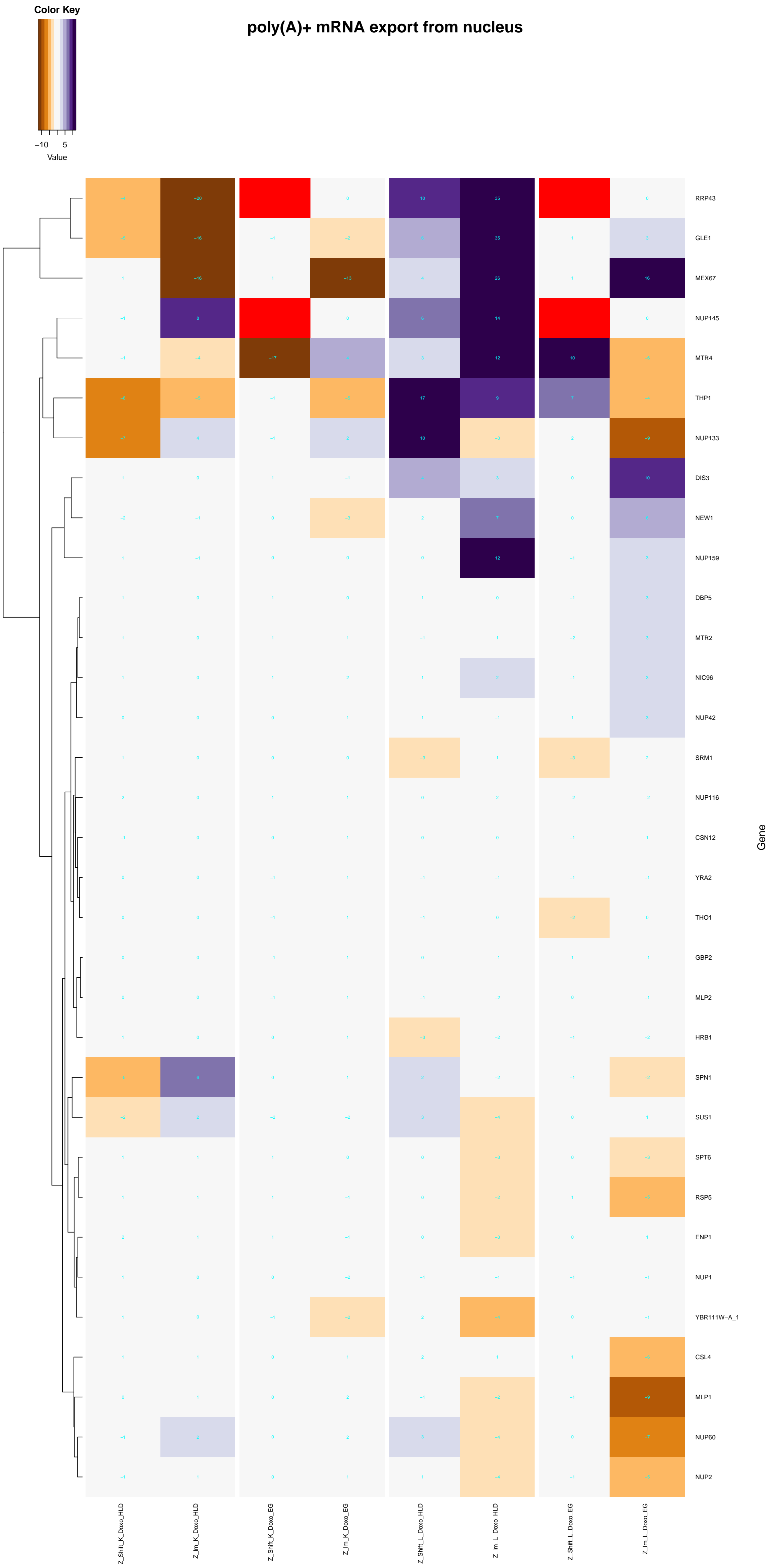

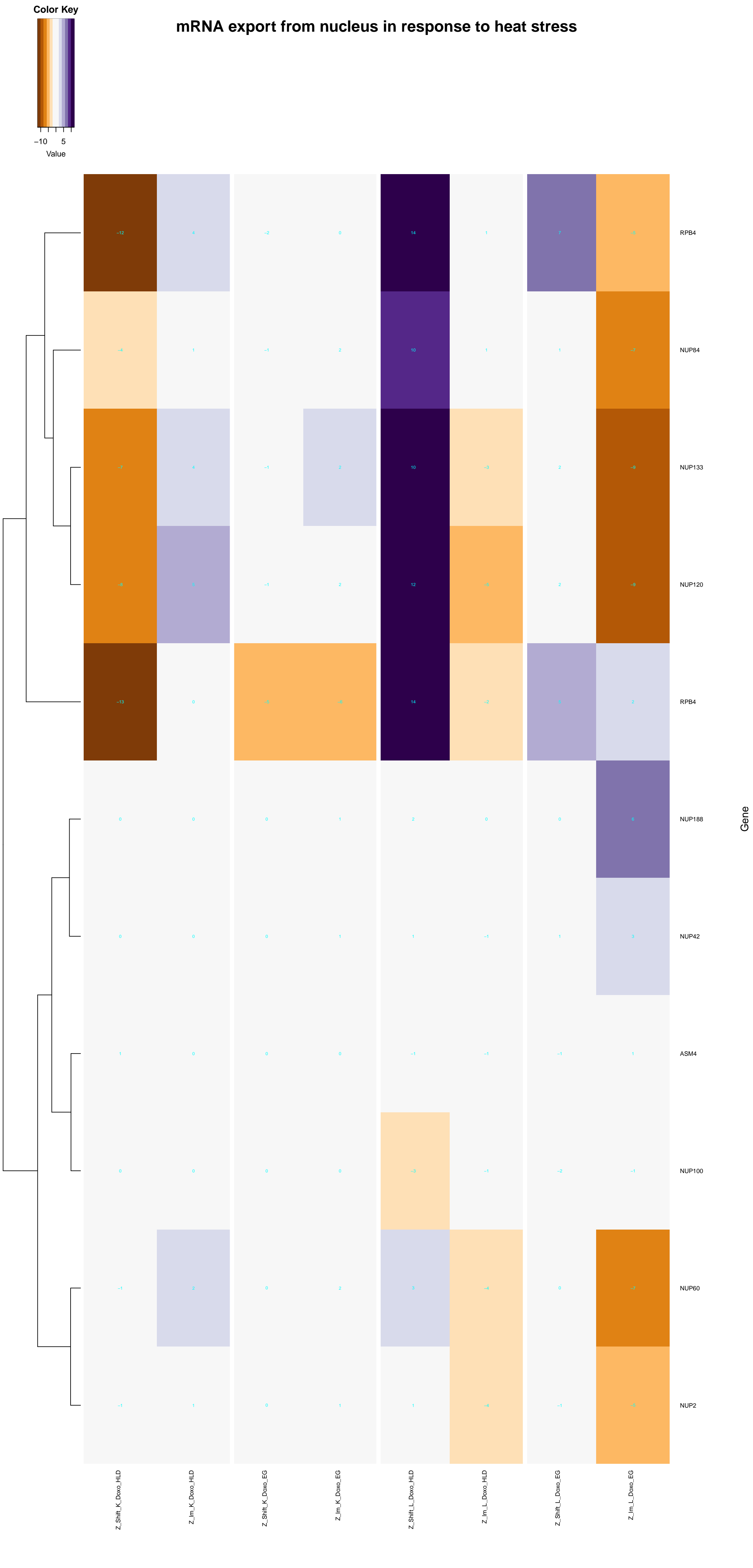

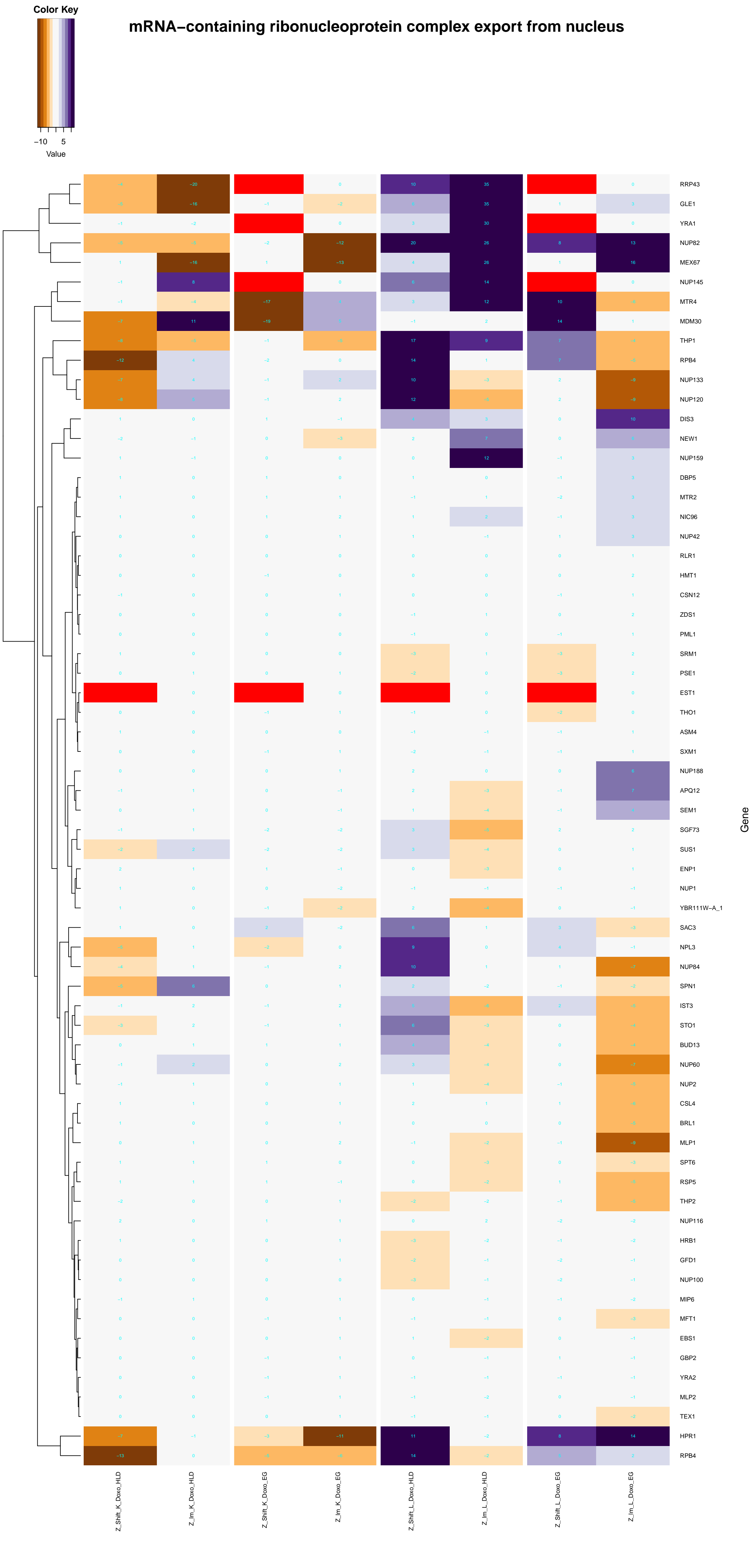

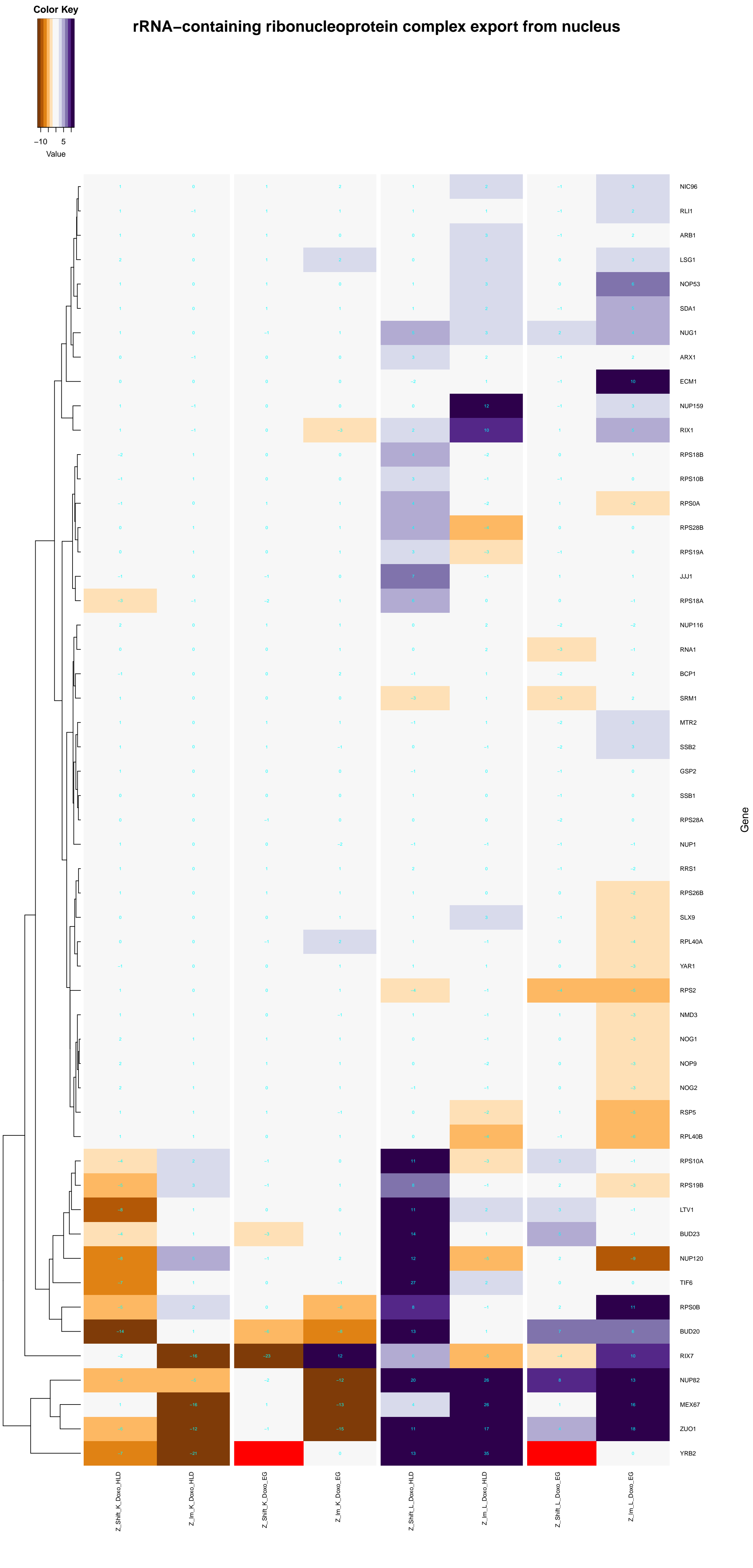

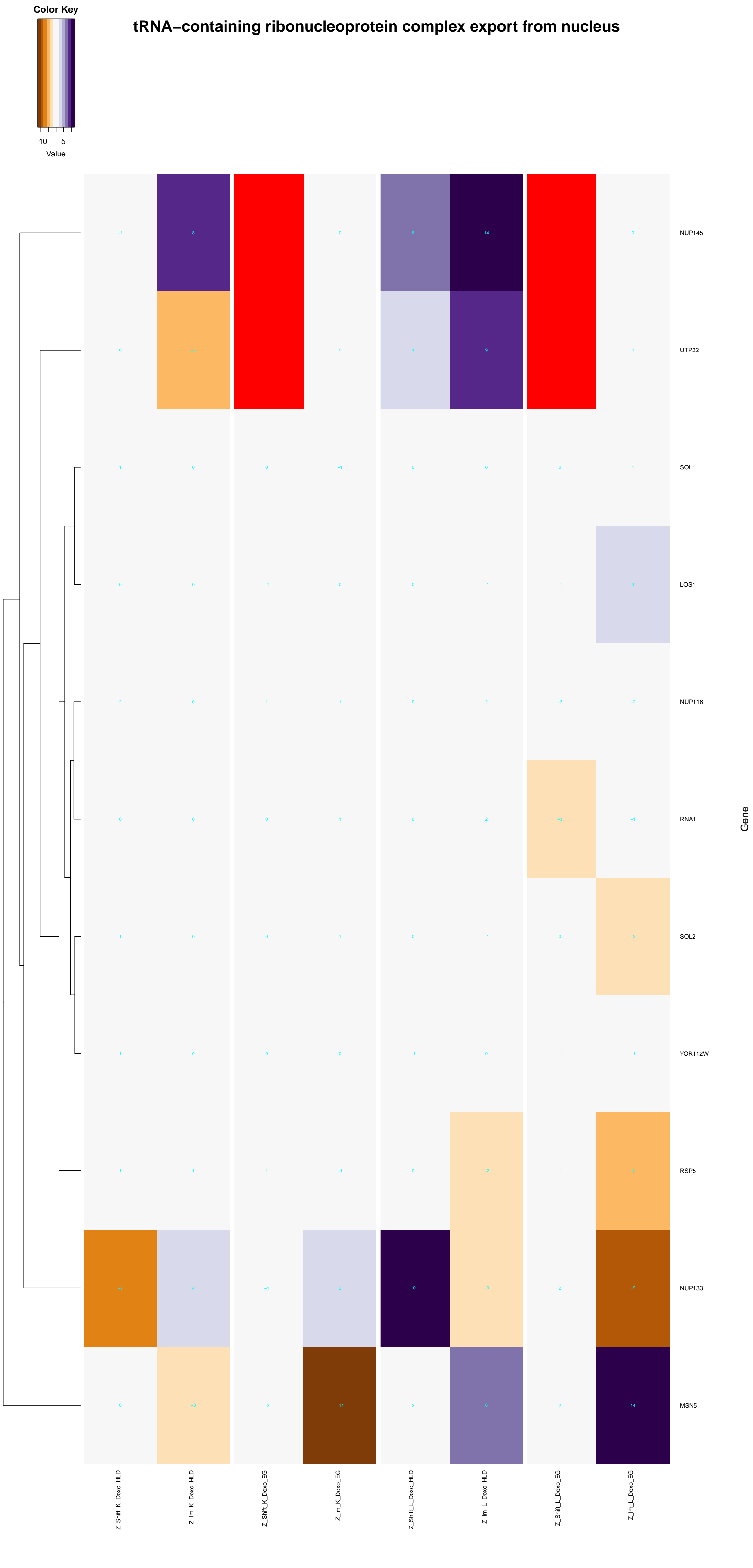

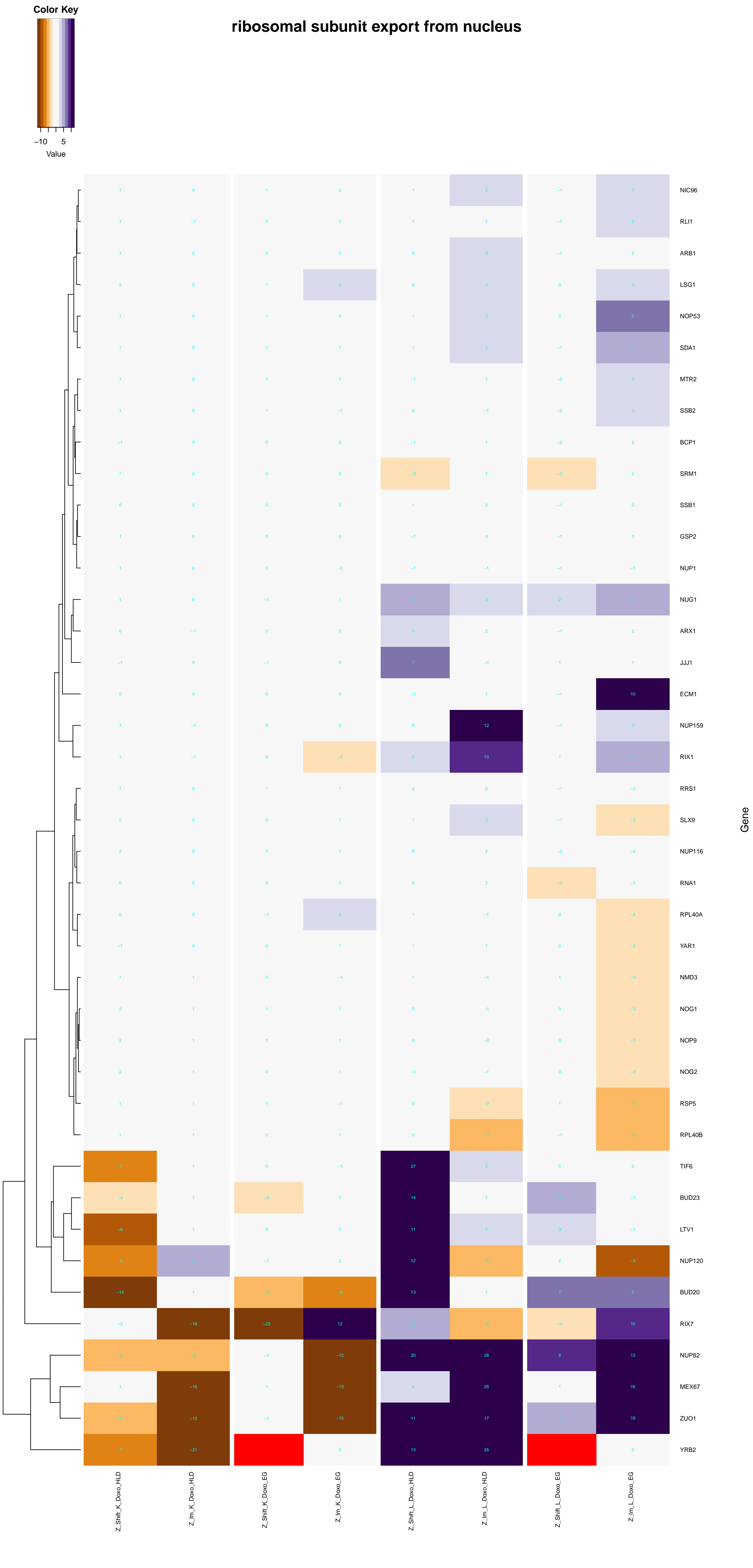

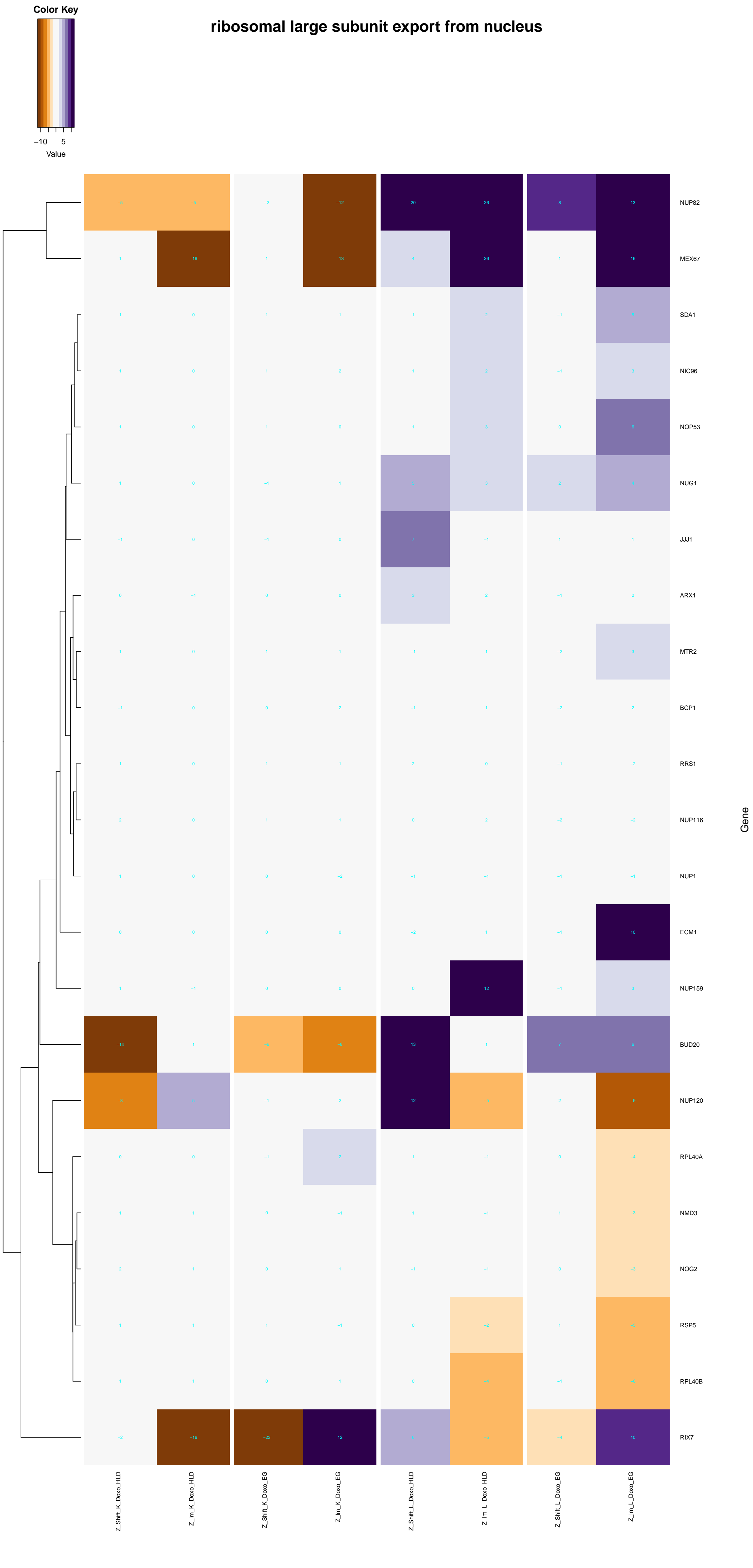

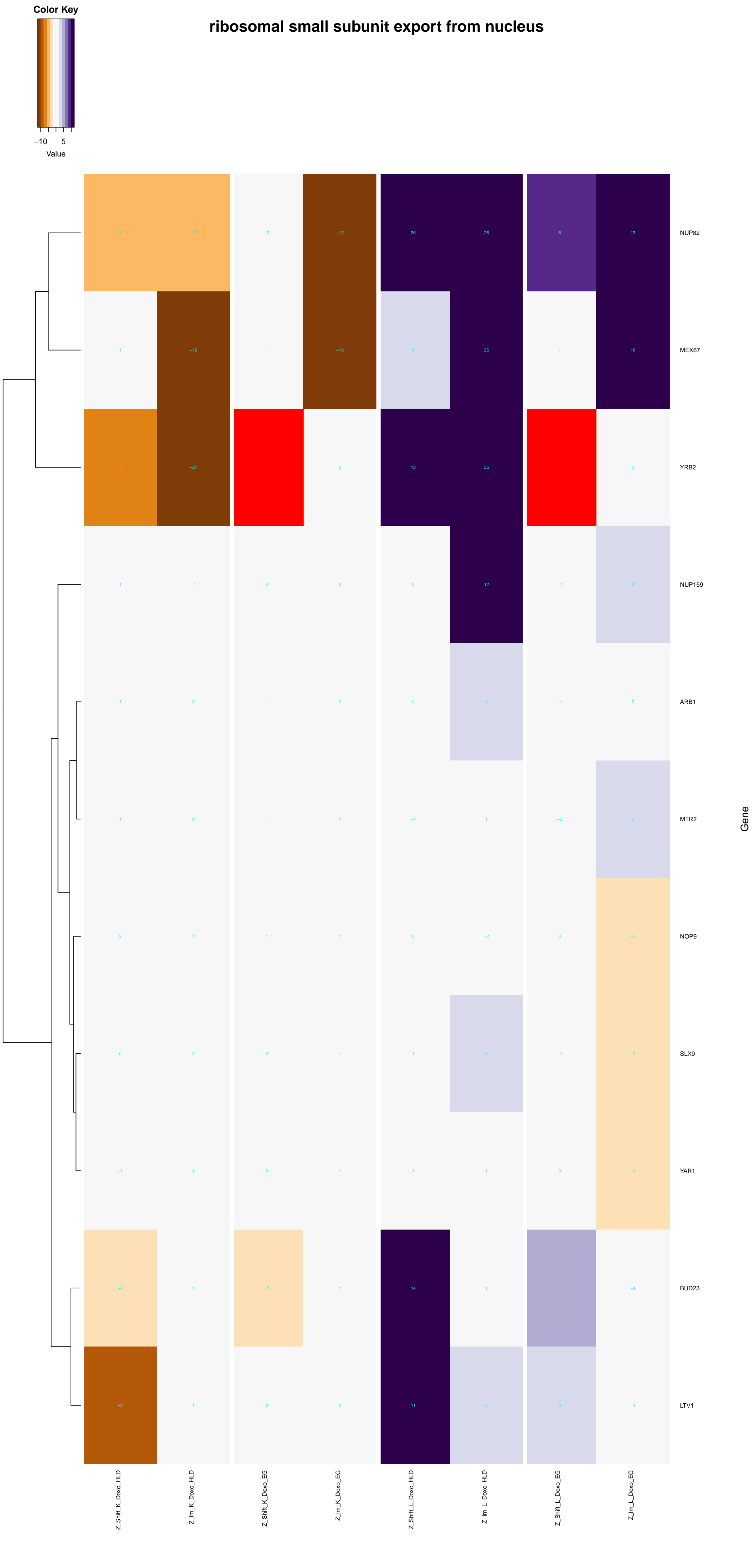

Supplement: Supplementary file 9 — Additional file 9. GO term-specific heatmaps for REMc/GTF-enriched clusters. GO term-specific heatmaps for significant GO process terms were generated as described in methods and Figs. 3 and 4. Any related child terms are presented in subsequent pages of the parent file name. GO terms with more than 100 children, with 2 or fewer genes annotated to the term, or a file size over 300KB are not shown. All heatmaps are generated with the same layout (see Figs. 3 and 4). [file 40170_2019_201_MOESM9_ESM.bz2 › Additional_File9_GOTermHeatmaps/Additional_File9_GOTermHeatmap/nucleic_acid_transport.pdf]

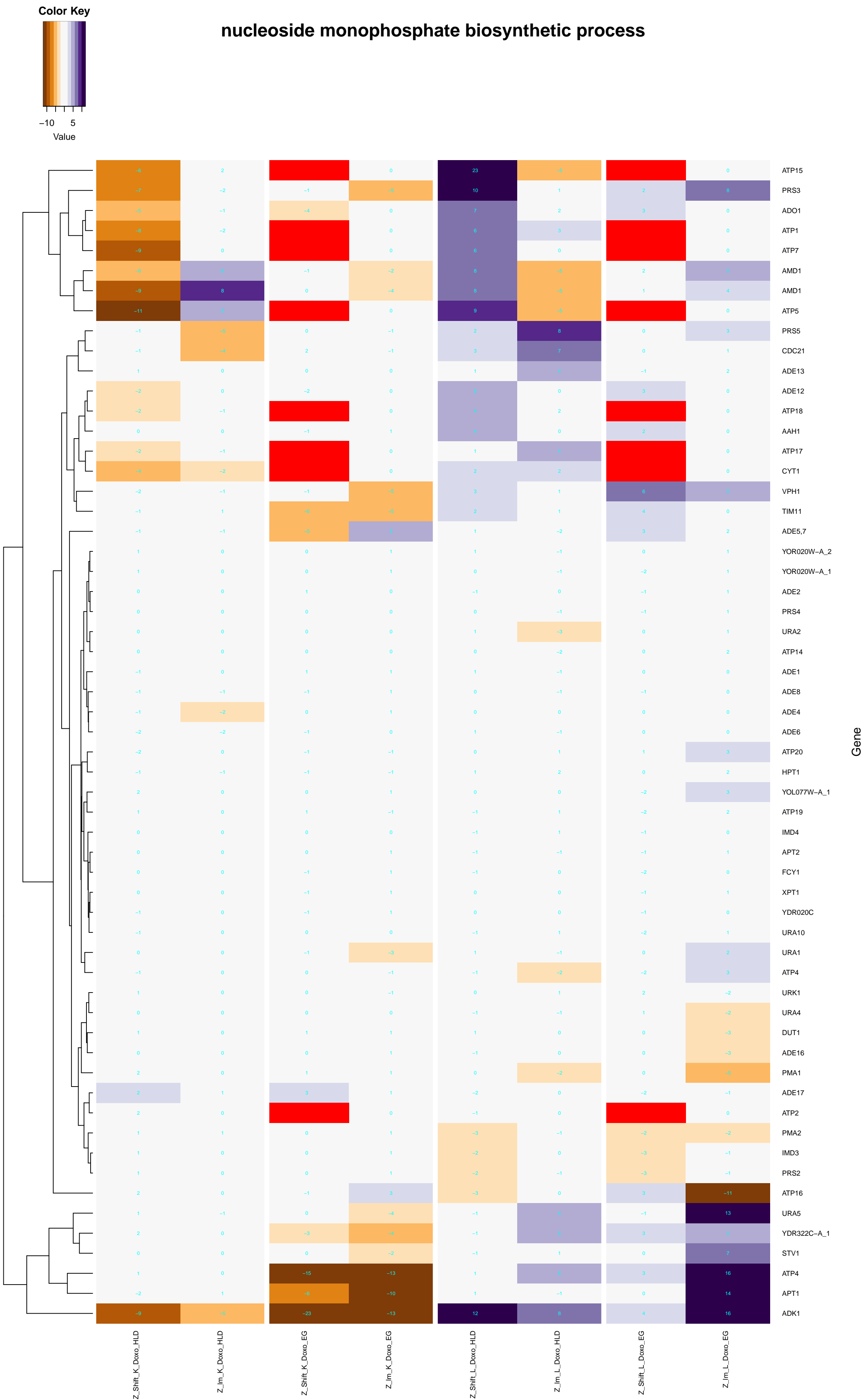

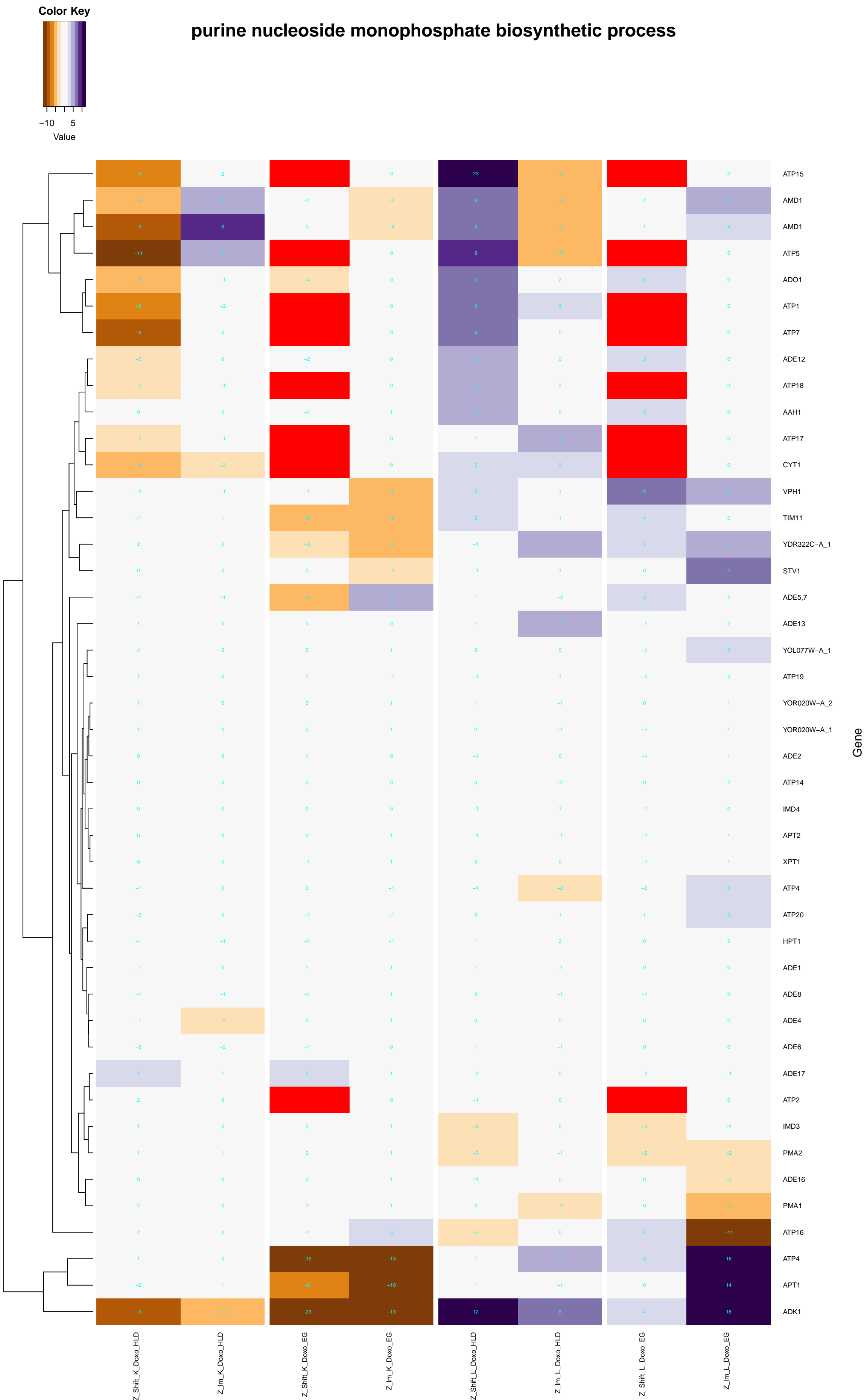

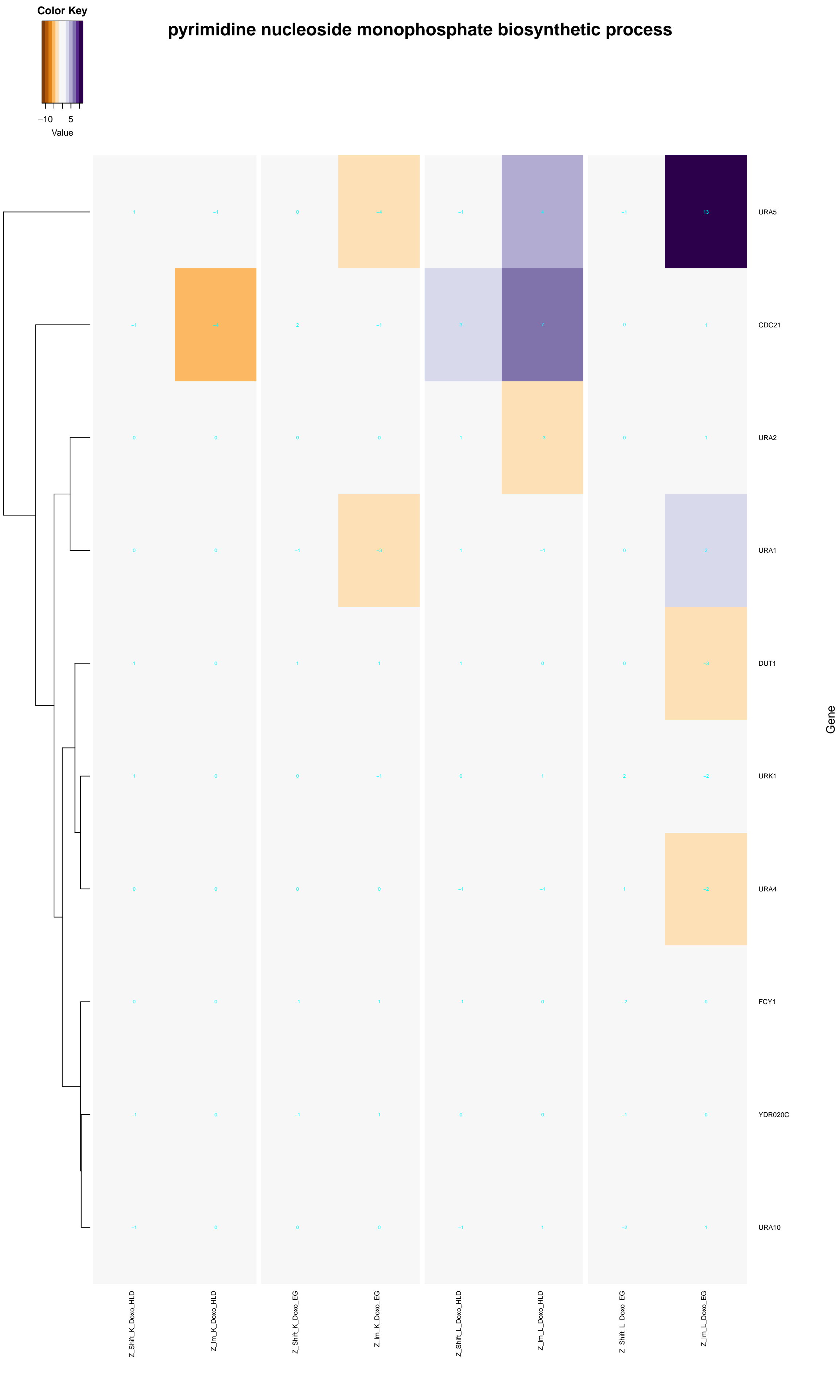

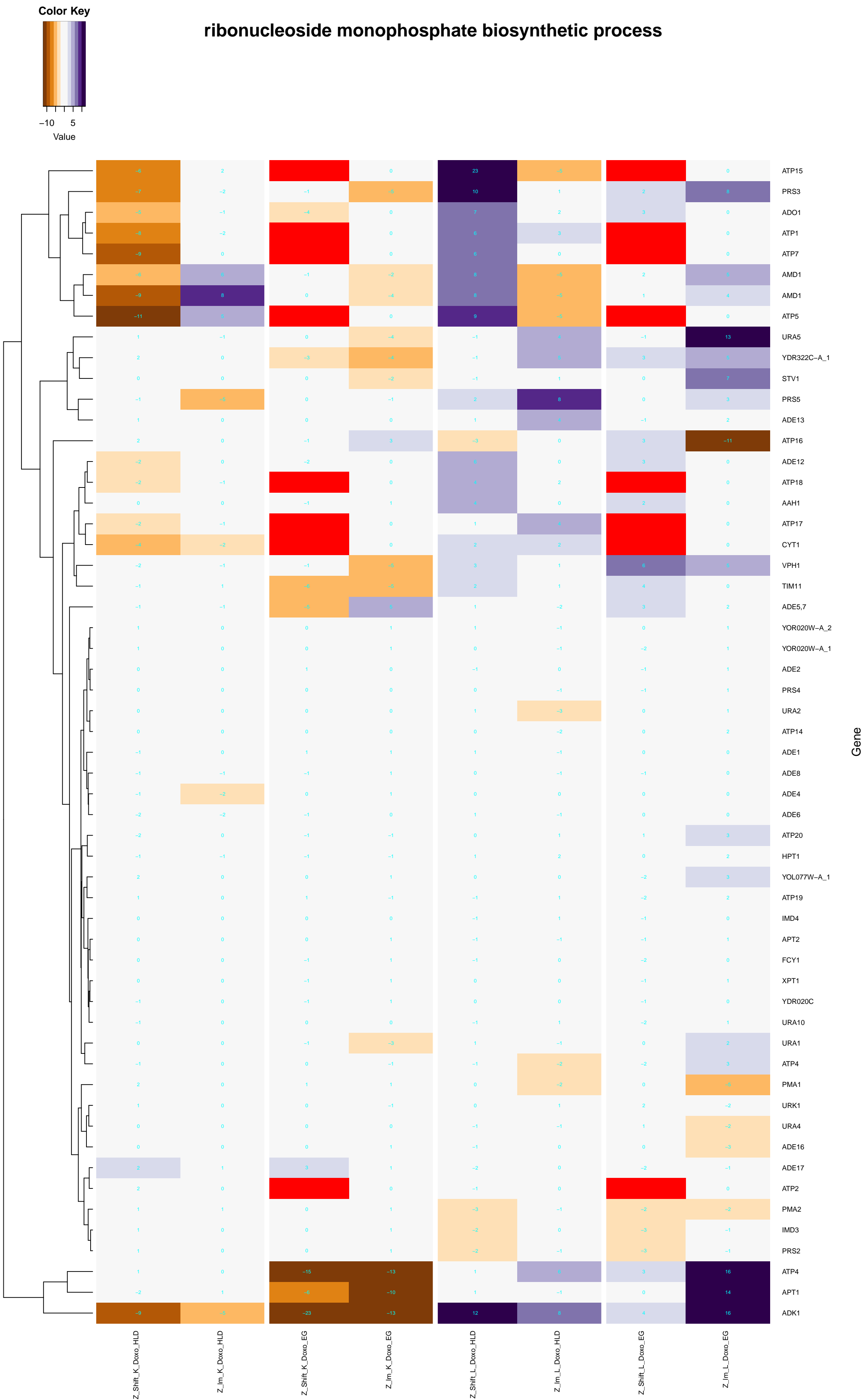

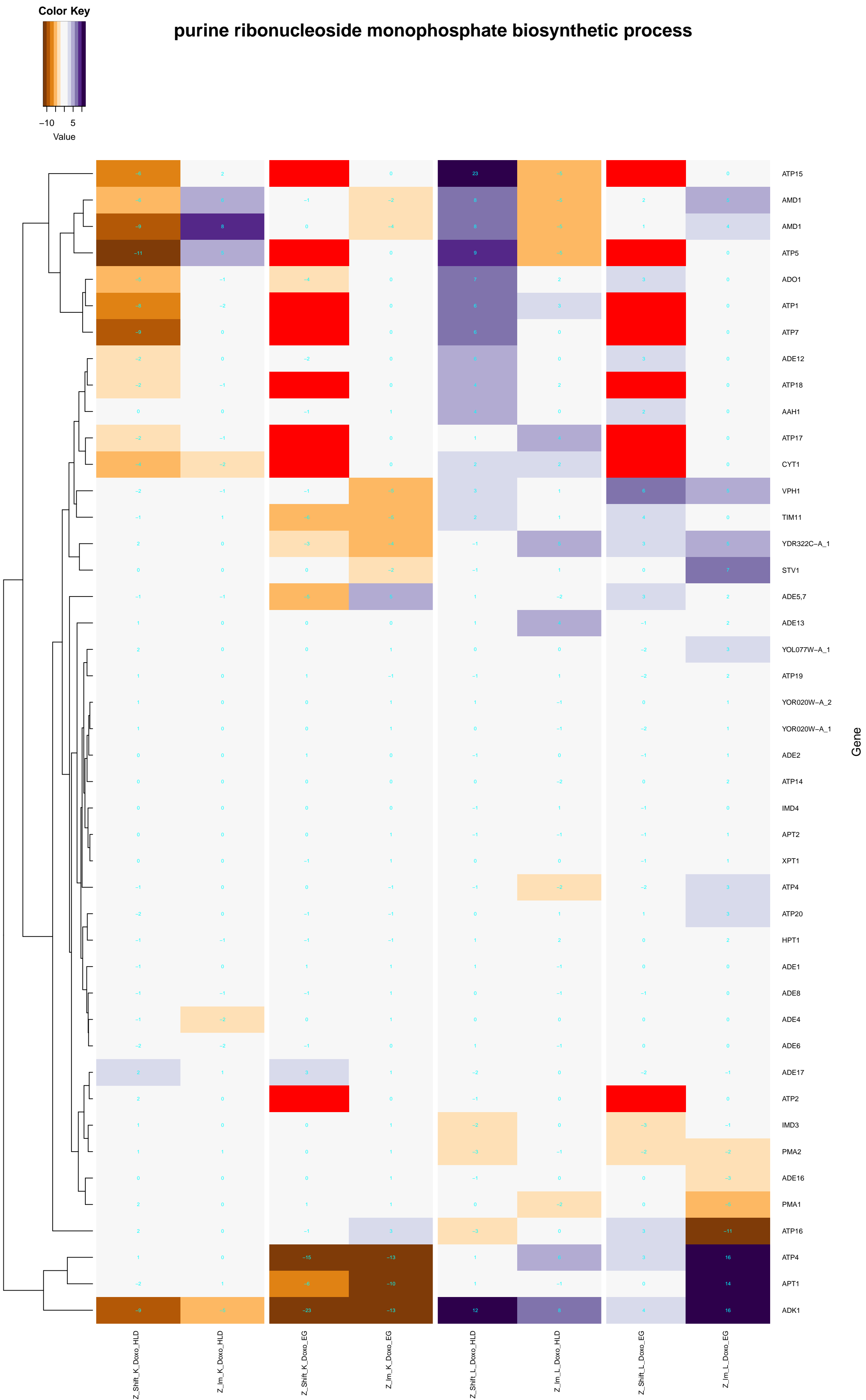

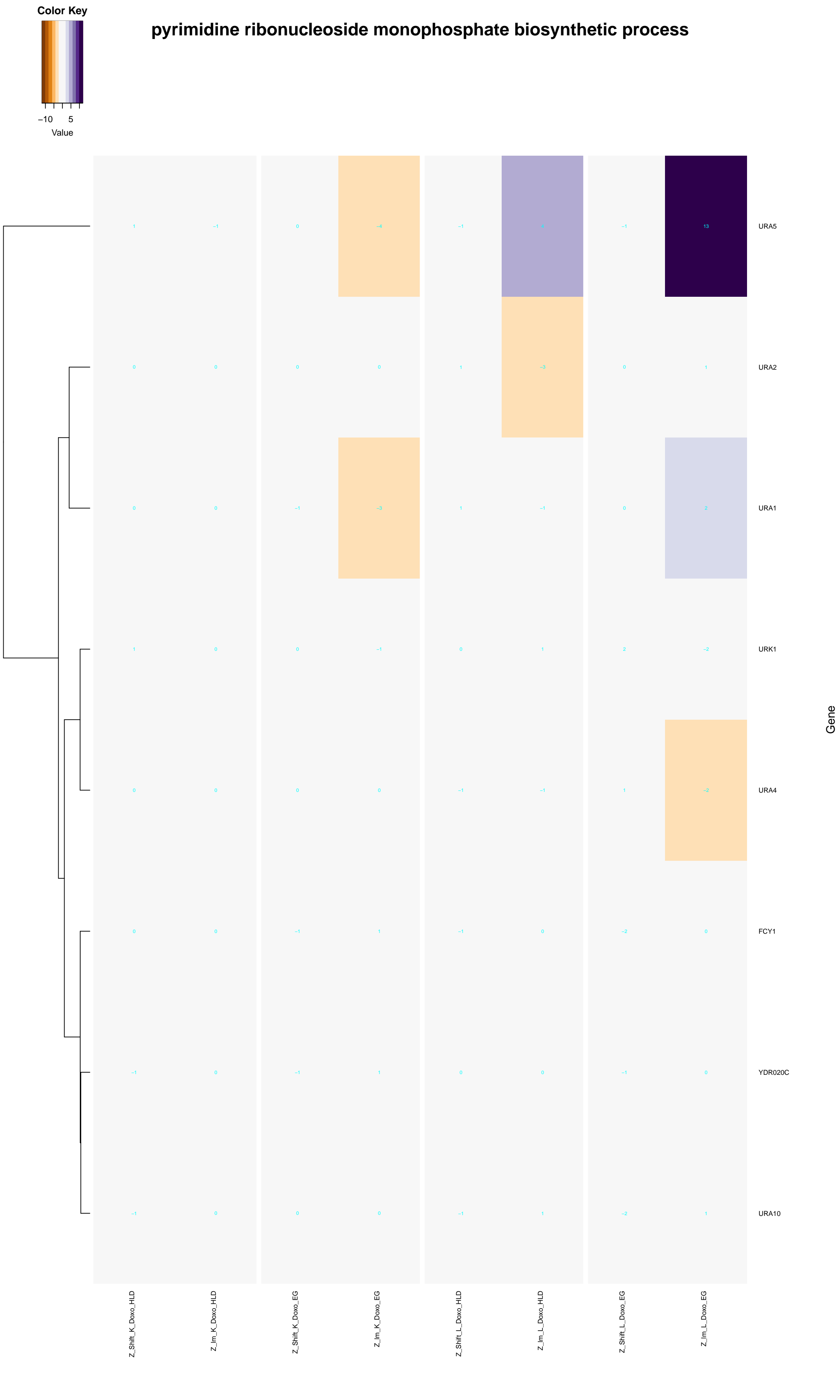

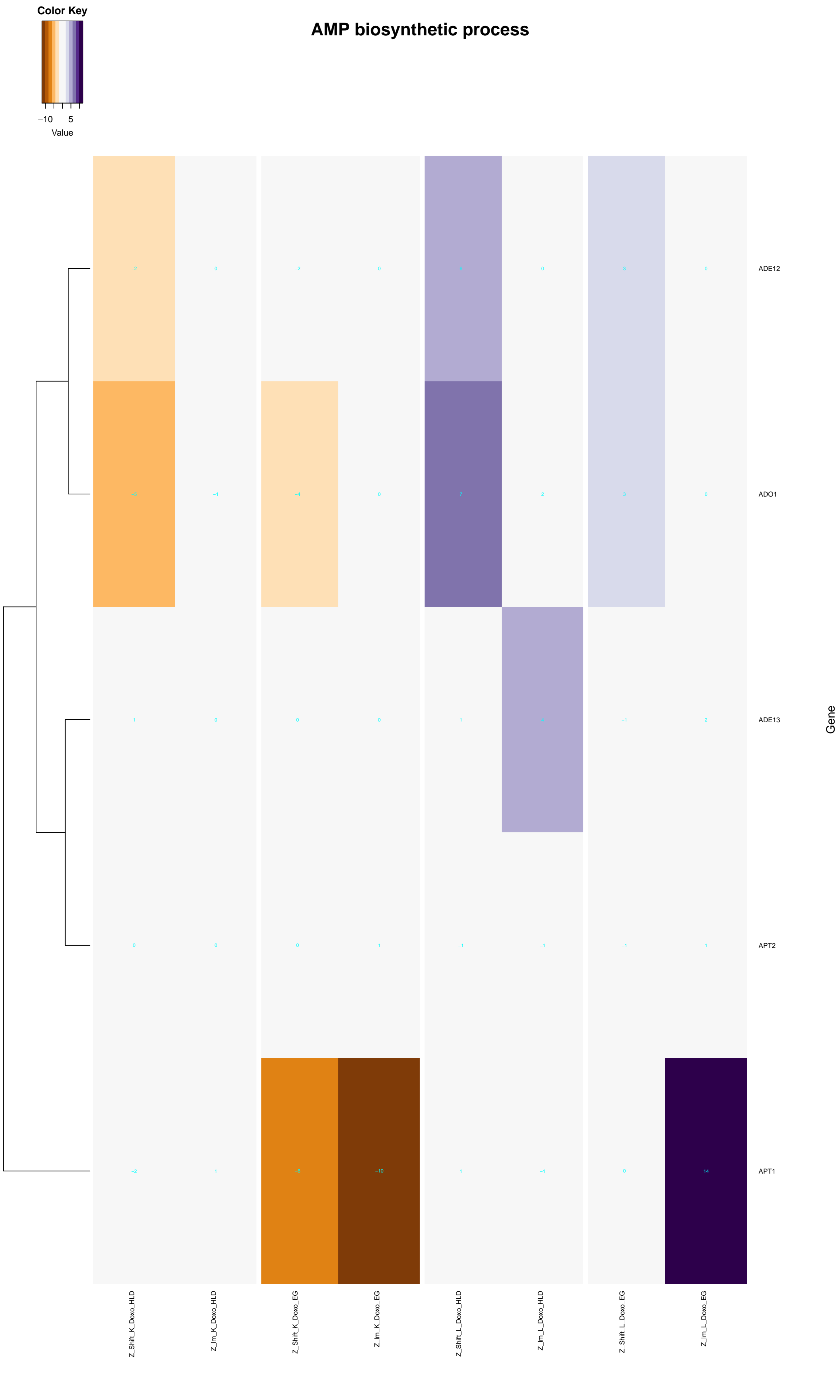

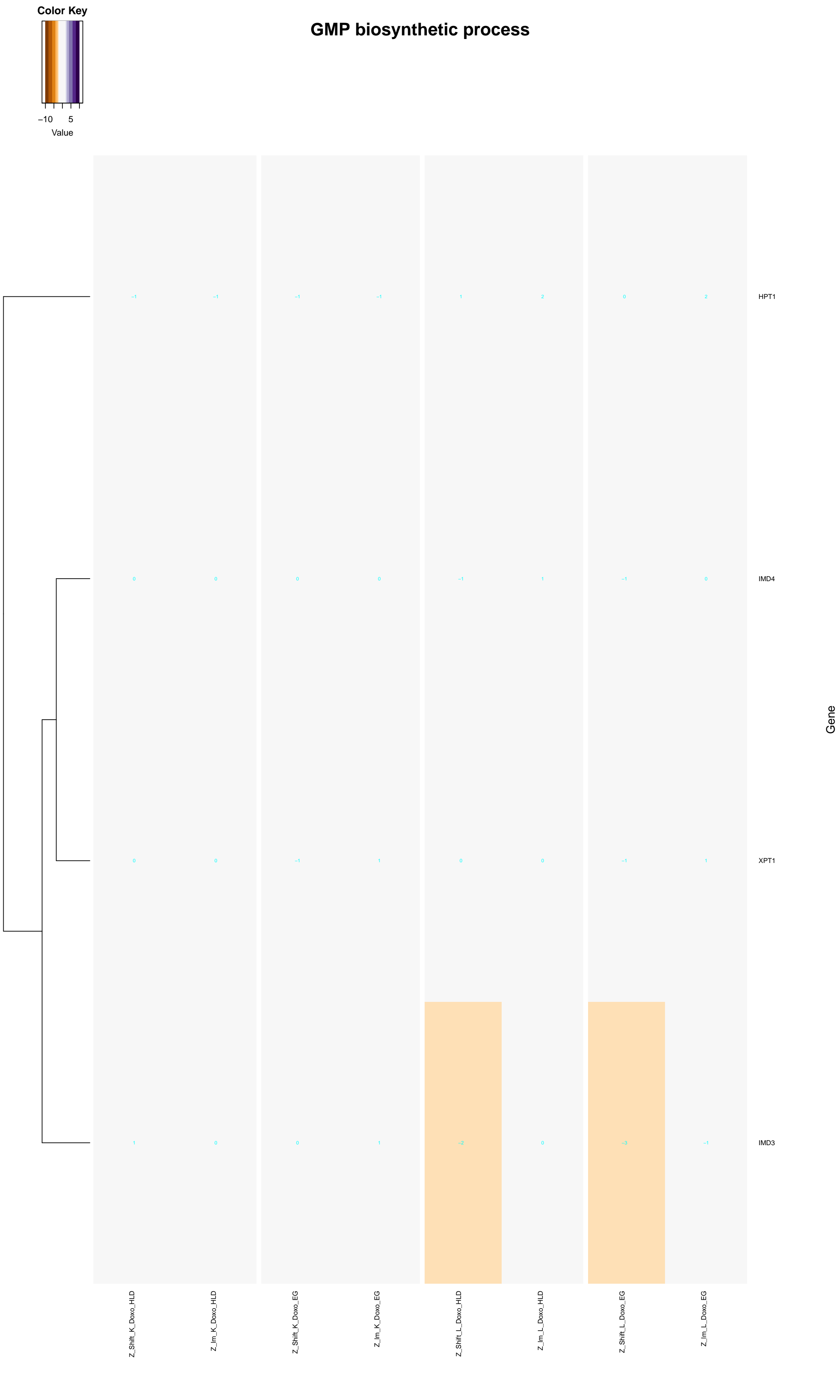

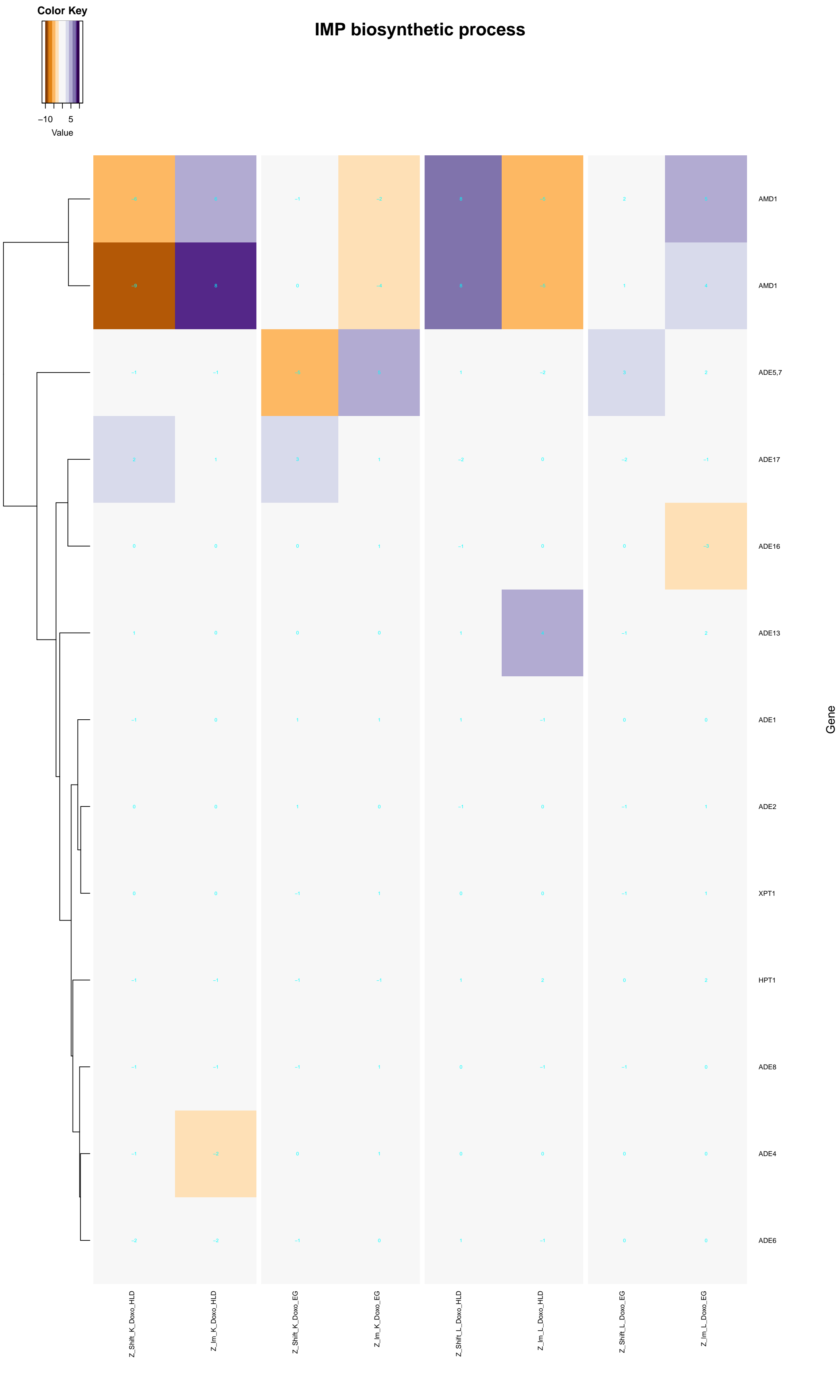

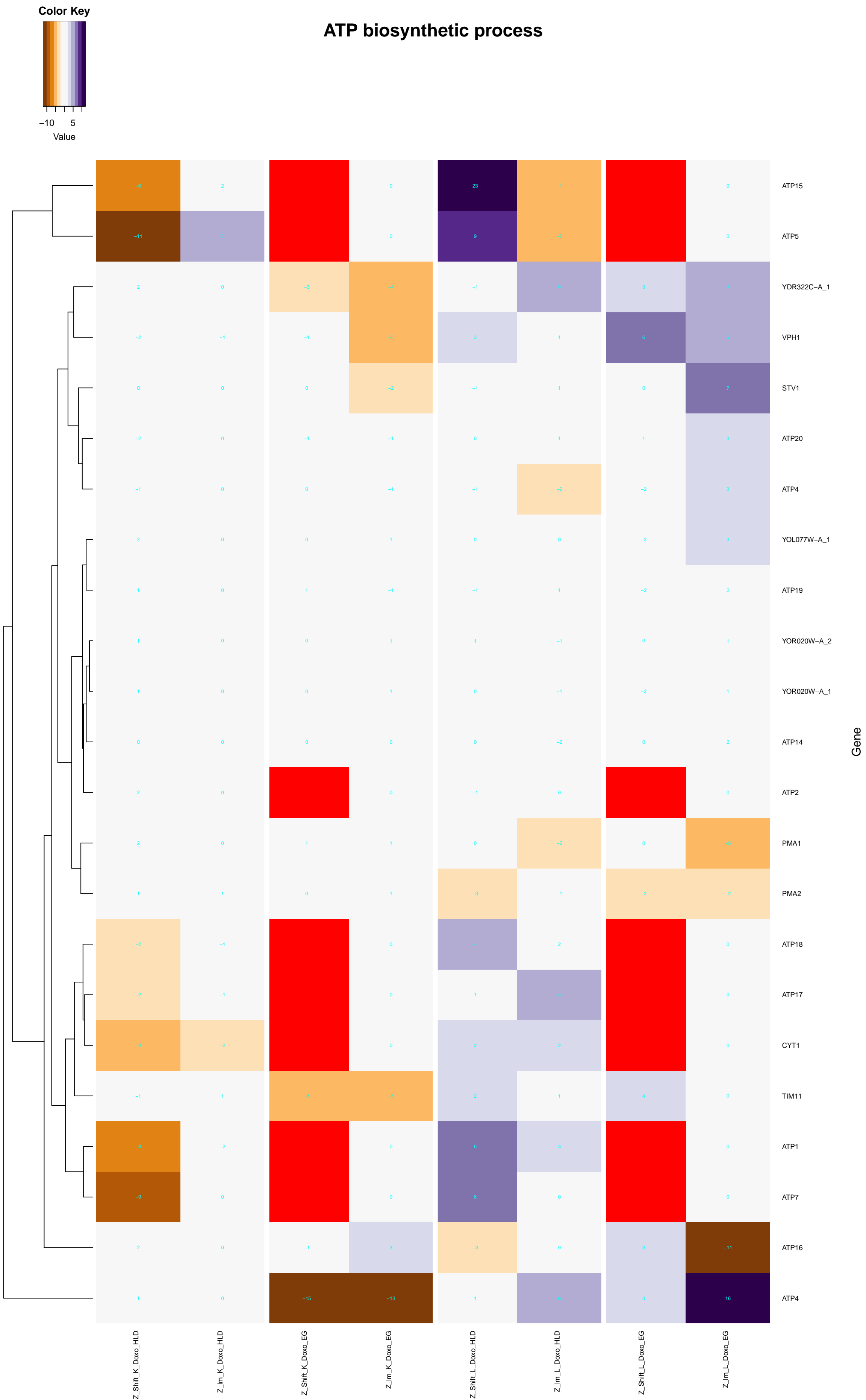

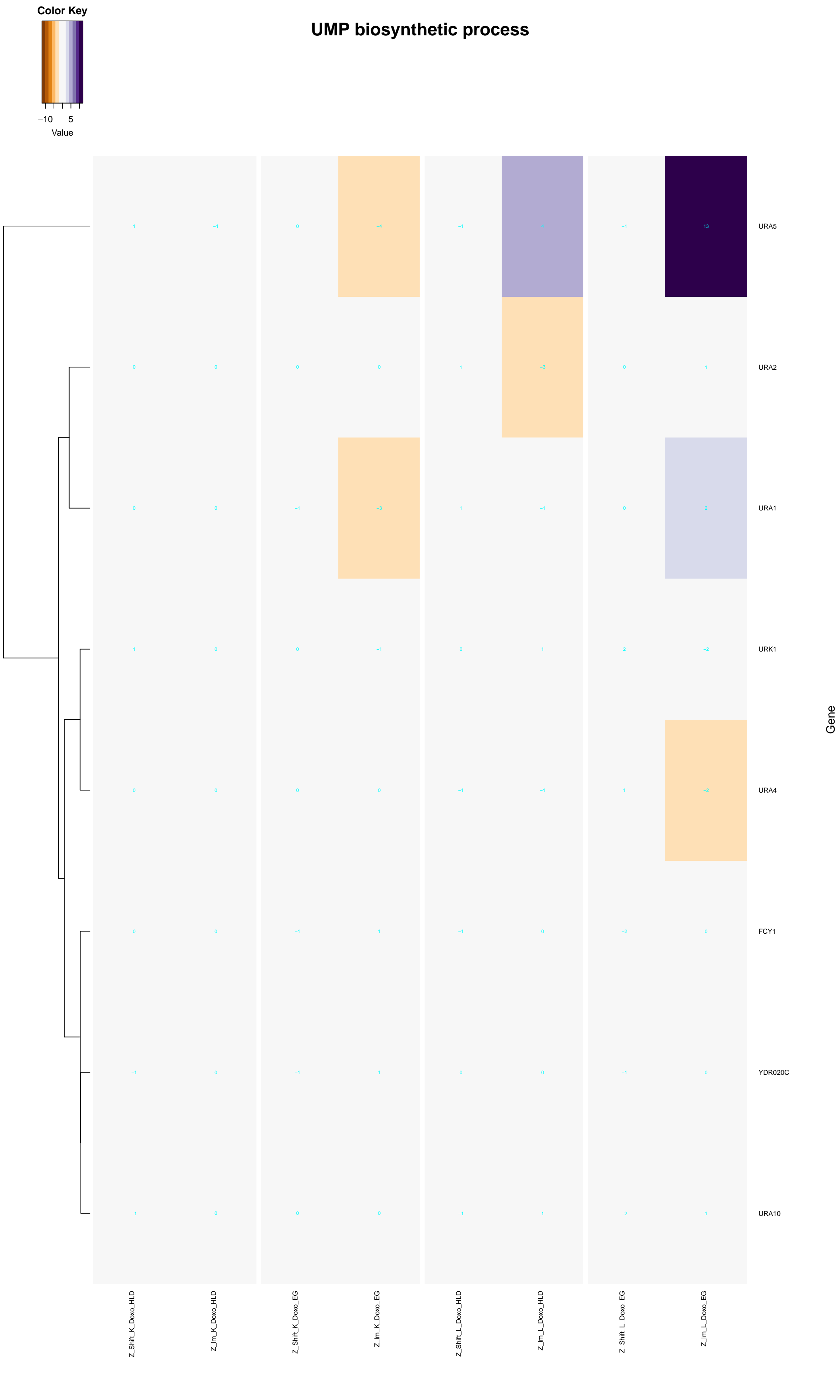

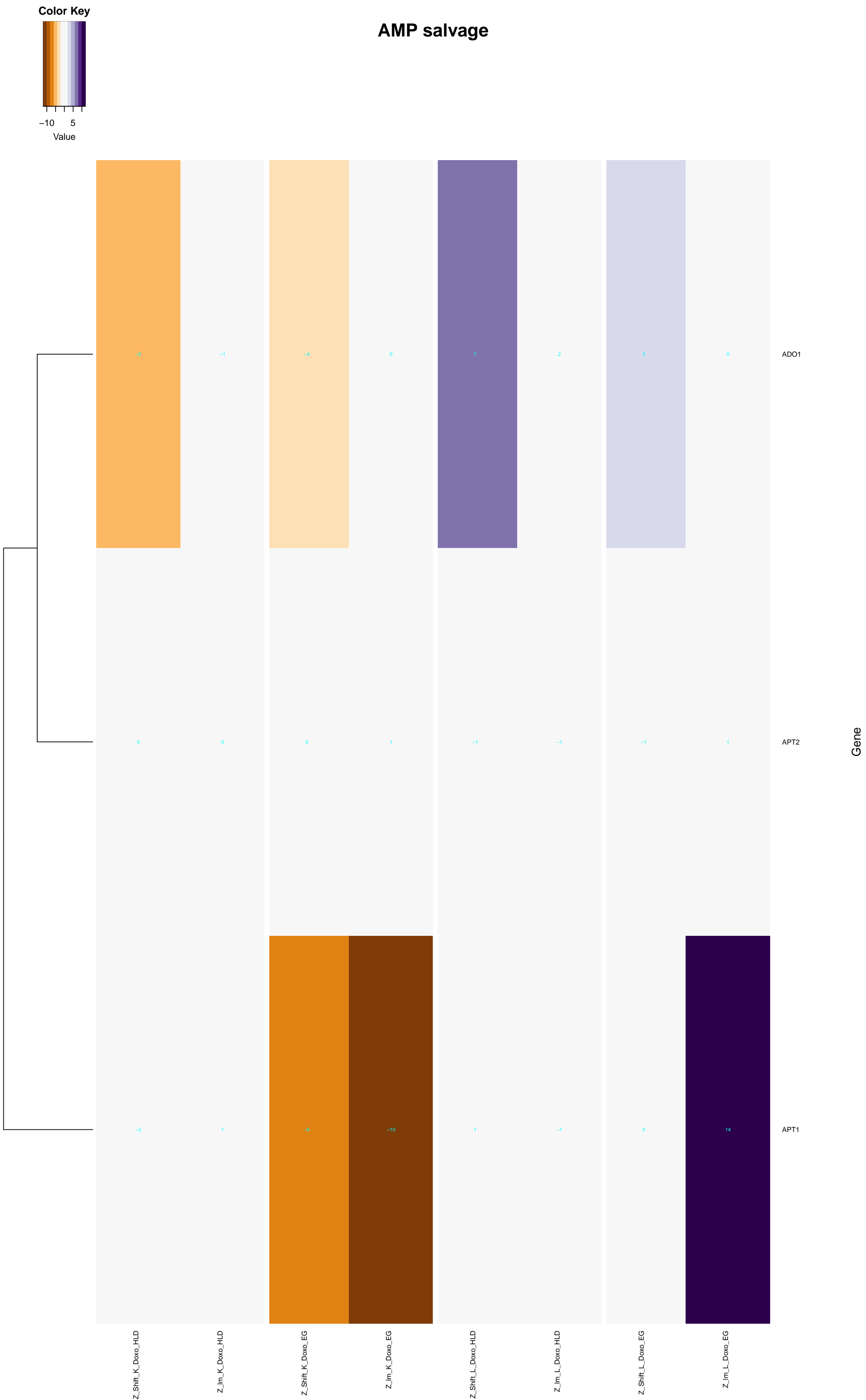

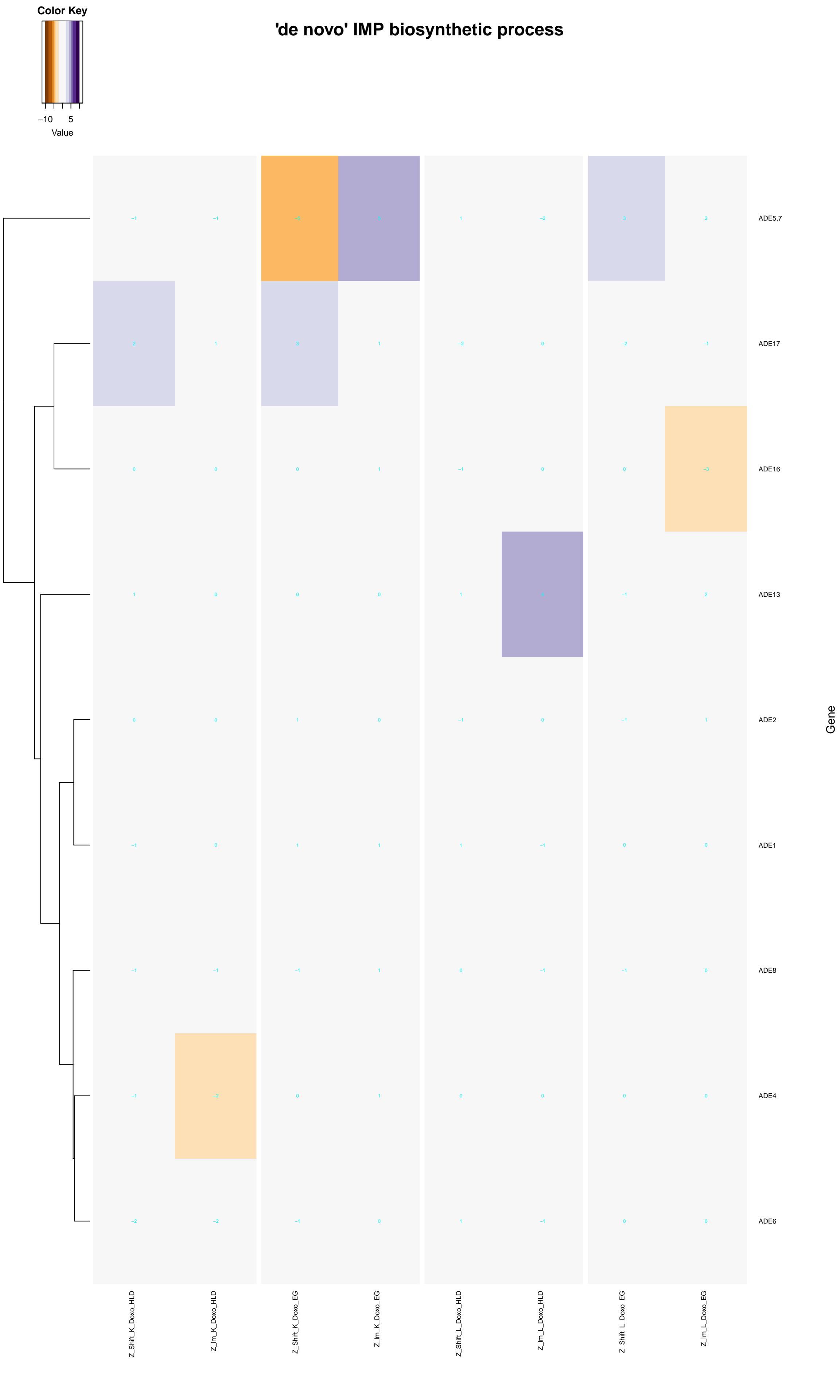

IMP salvage

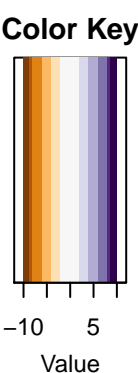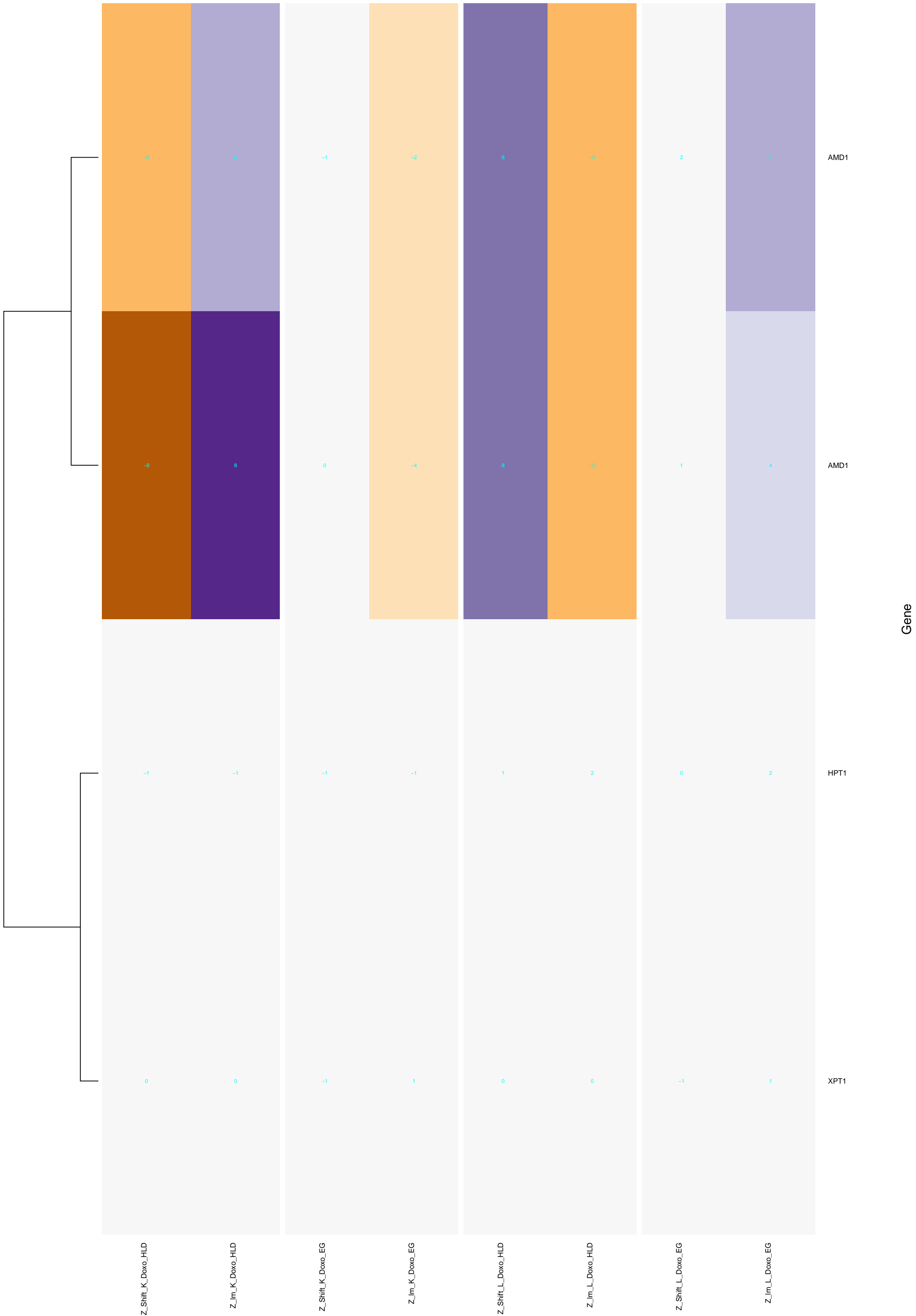

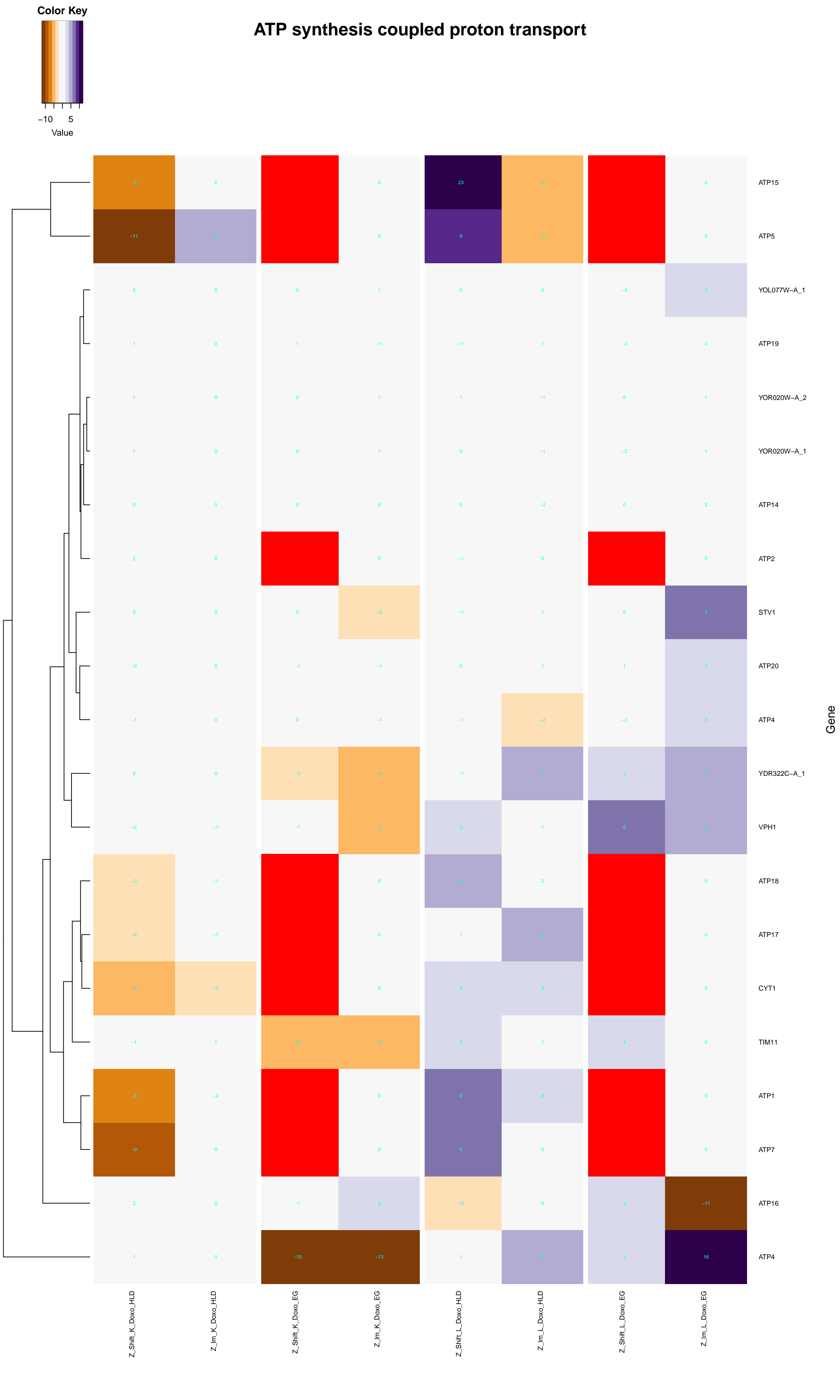

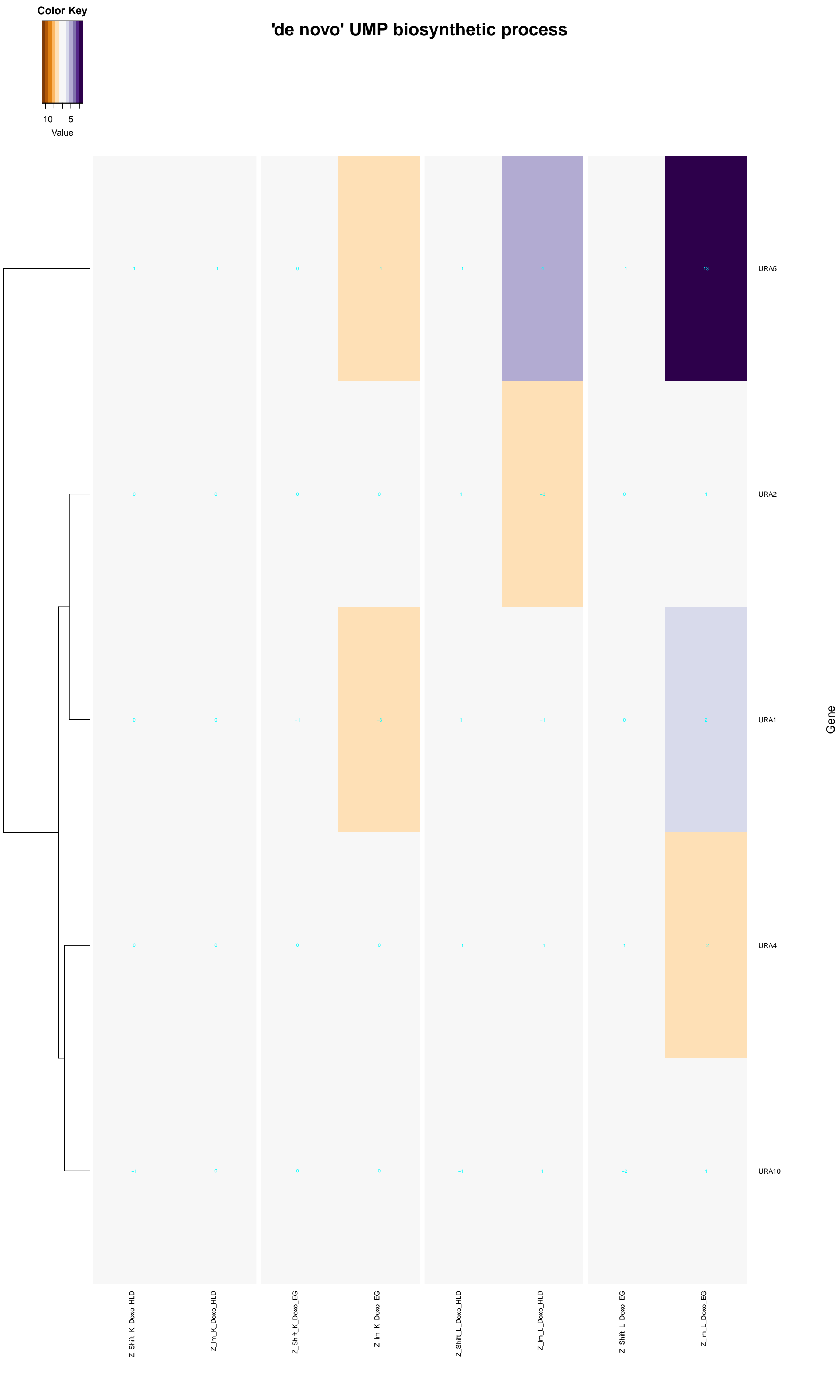

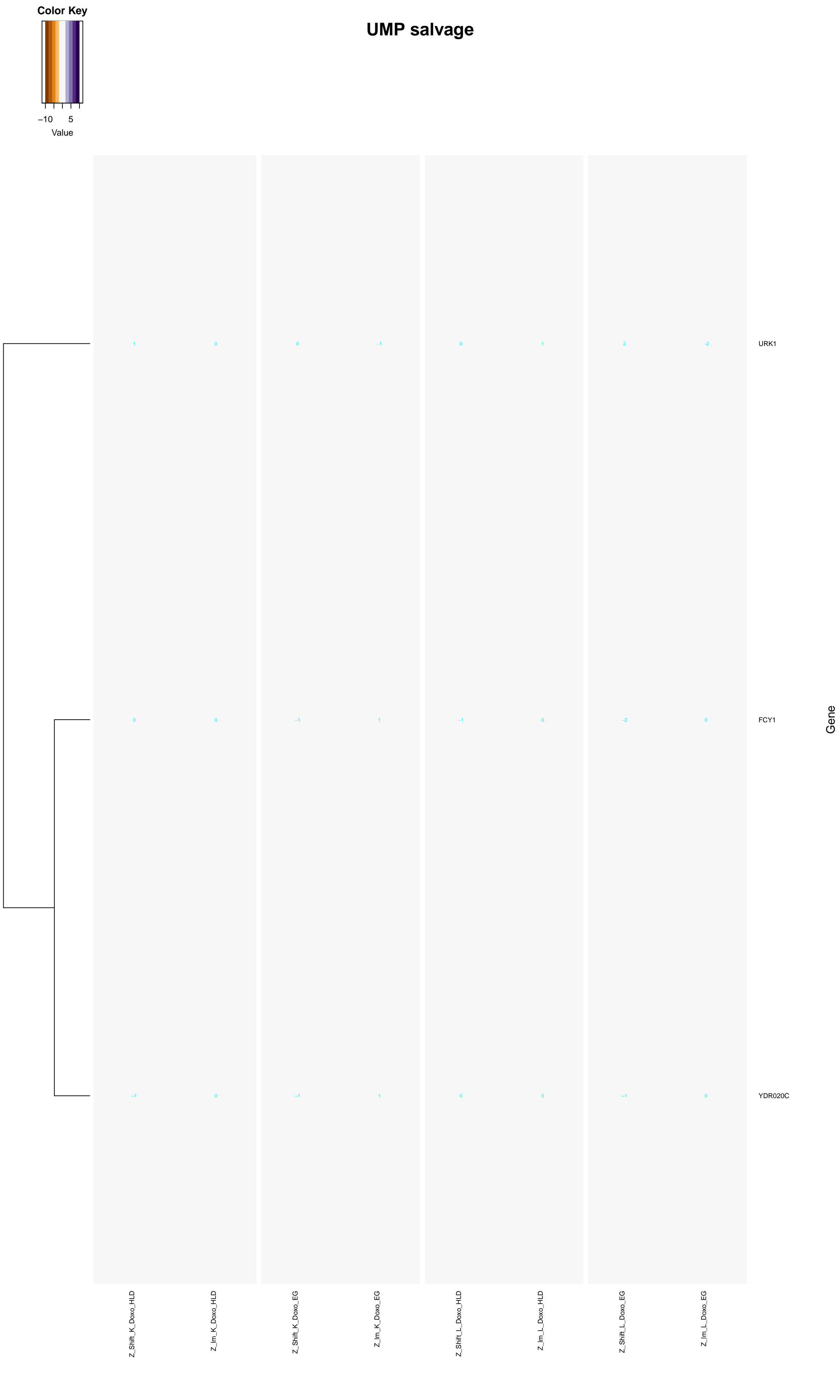

Supplement: Supplementary file 9 — Additional file 9. GO term-specific heatmaps for REMc/GTF-enriched clusters. GO term-specific heatmaps for significant GO process terms were generated as described in methods and Figs. 3 and 4. Any related child terms are presented in subsequent pages of the parent file name. GO terms with more than 100 children, with 2 or fewer genes annotated to the term, or a file size over 300KB are not shown. All heatmaps are generated with the same layout (see Figs. 3 and 4). [file 40170_2019_201_MOESM9_ESM.bz2 › Additional_File9_GOTermHeatmaps/Additional_File9_GOTermHeatmap/nucleoside_monophosphate_biosynthetic_process.pdf]

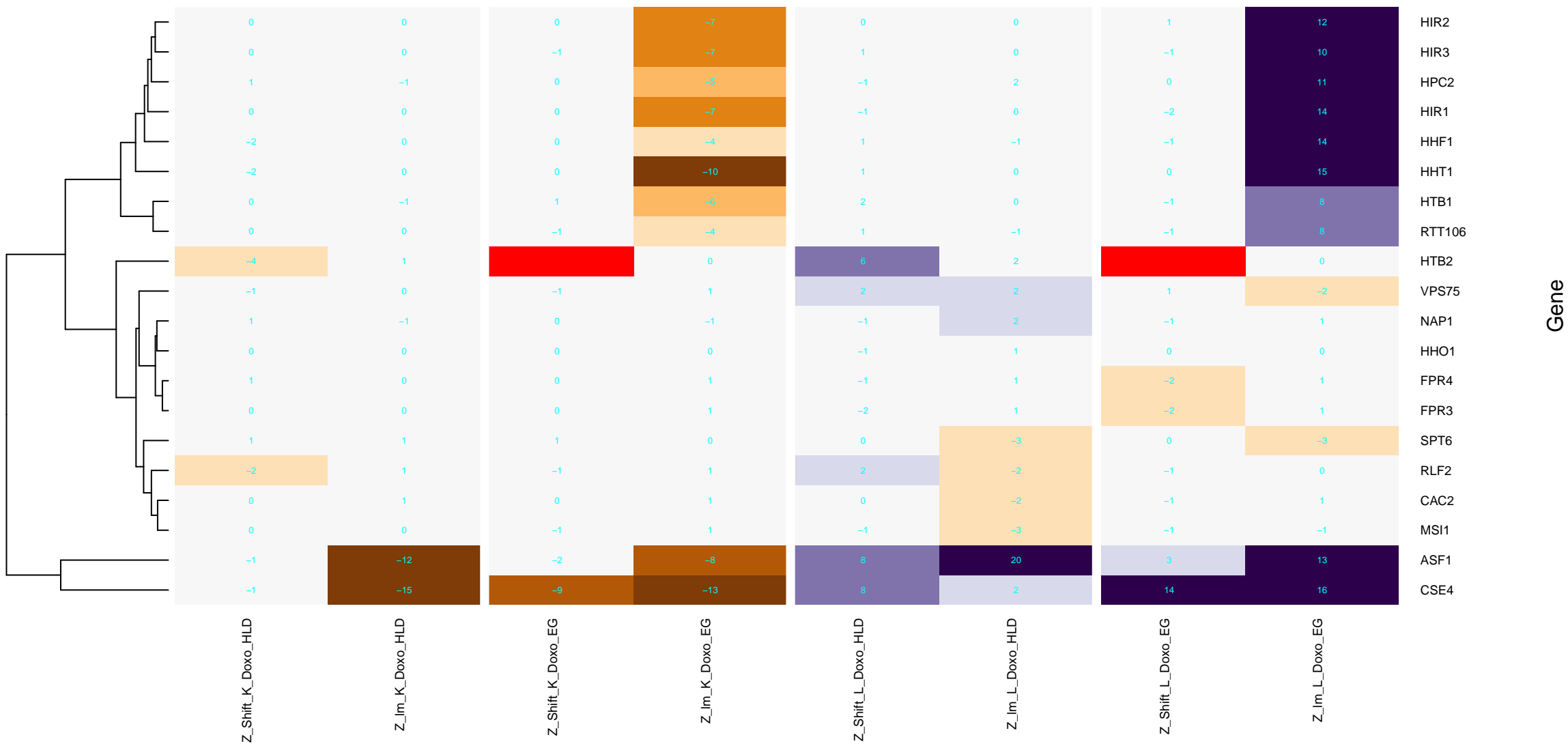

Supplement: Supplementary file 9 — Additional file 9. GO term-specific heatmaps for REMc/GTF-enriched clusters. GO term-specific heatmaps for significant GO process terms were generated as described in methods and Figs. 3 and 4. Any related child terms are presented in subsequent pages of the parent file name. GO terms with more than 100 children, with 2 or fewer genes annotated to the term, or a file size over 300KB are not shown. All heatmaps are generated with the same layout (see Figs. 3 and 4). [file 40170_2019_201_MOESM9_ESM.bz2 › Additional_File9_GOTermHeatmaps/Additional_File9_GOTermHeatmap/nucleosome_assembly.pdf]

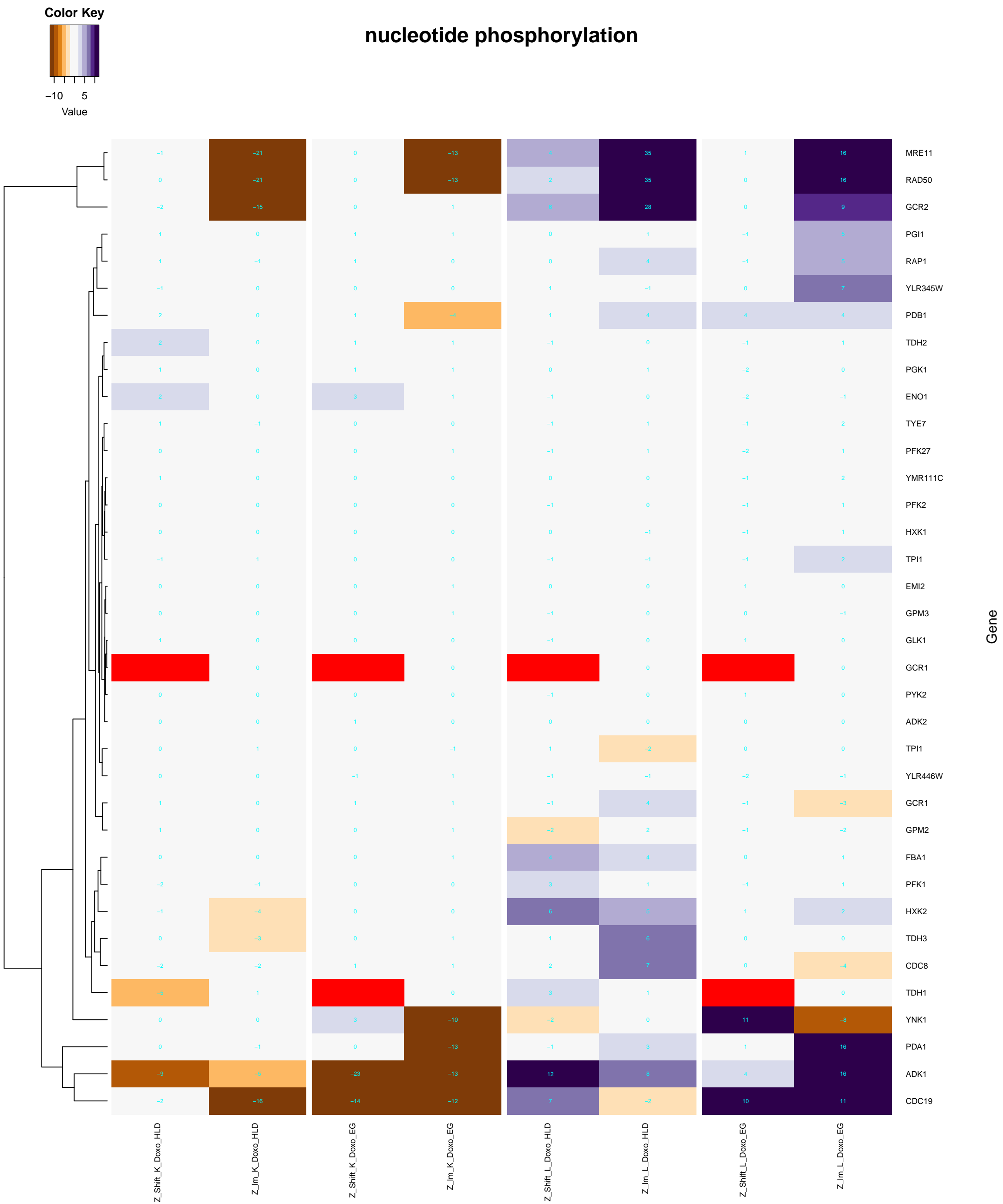

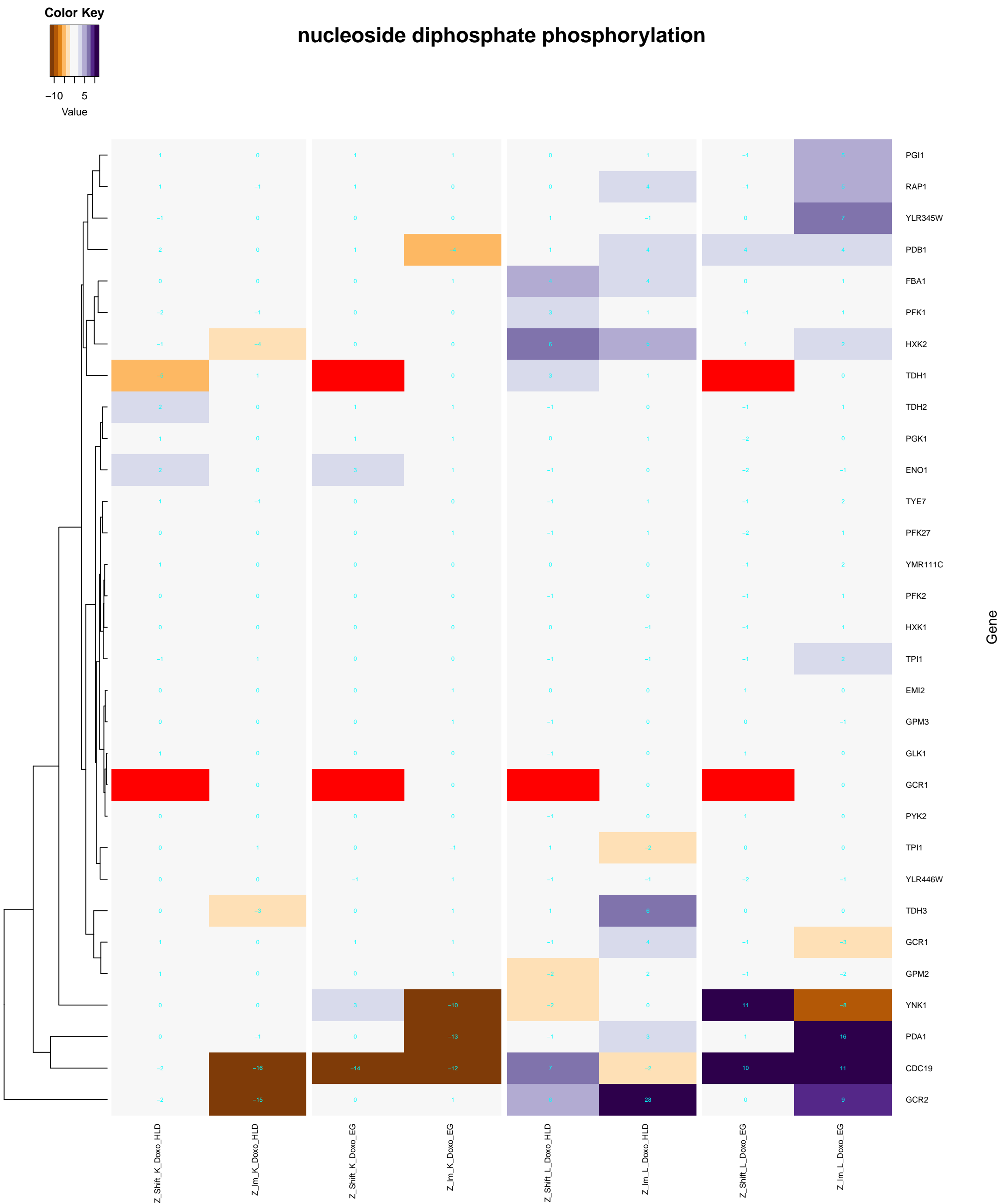

Color Key

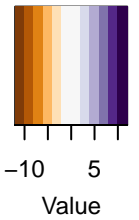

ATP generation from ADP

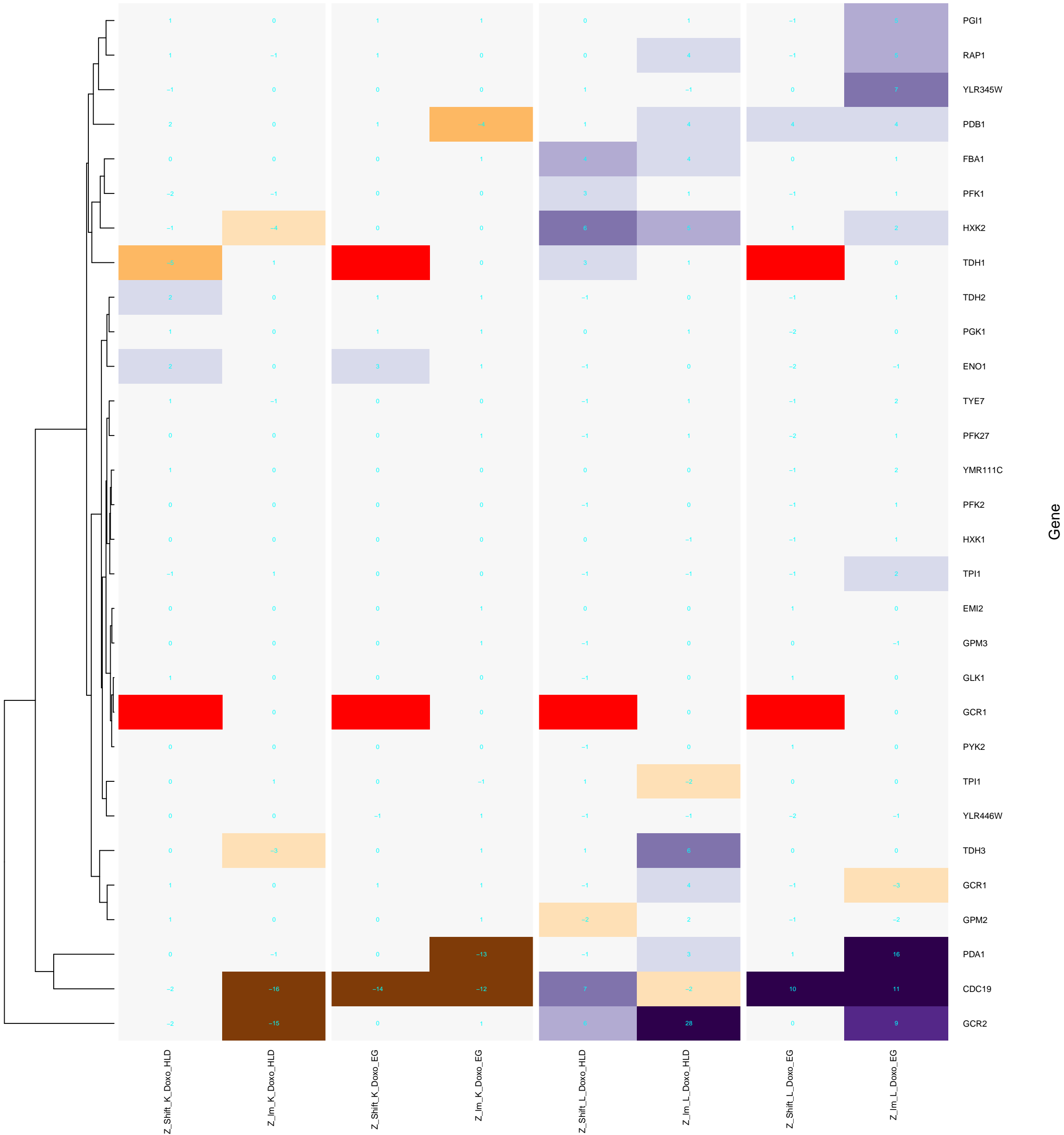

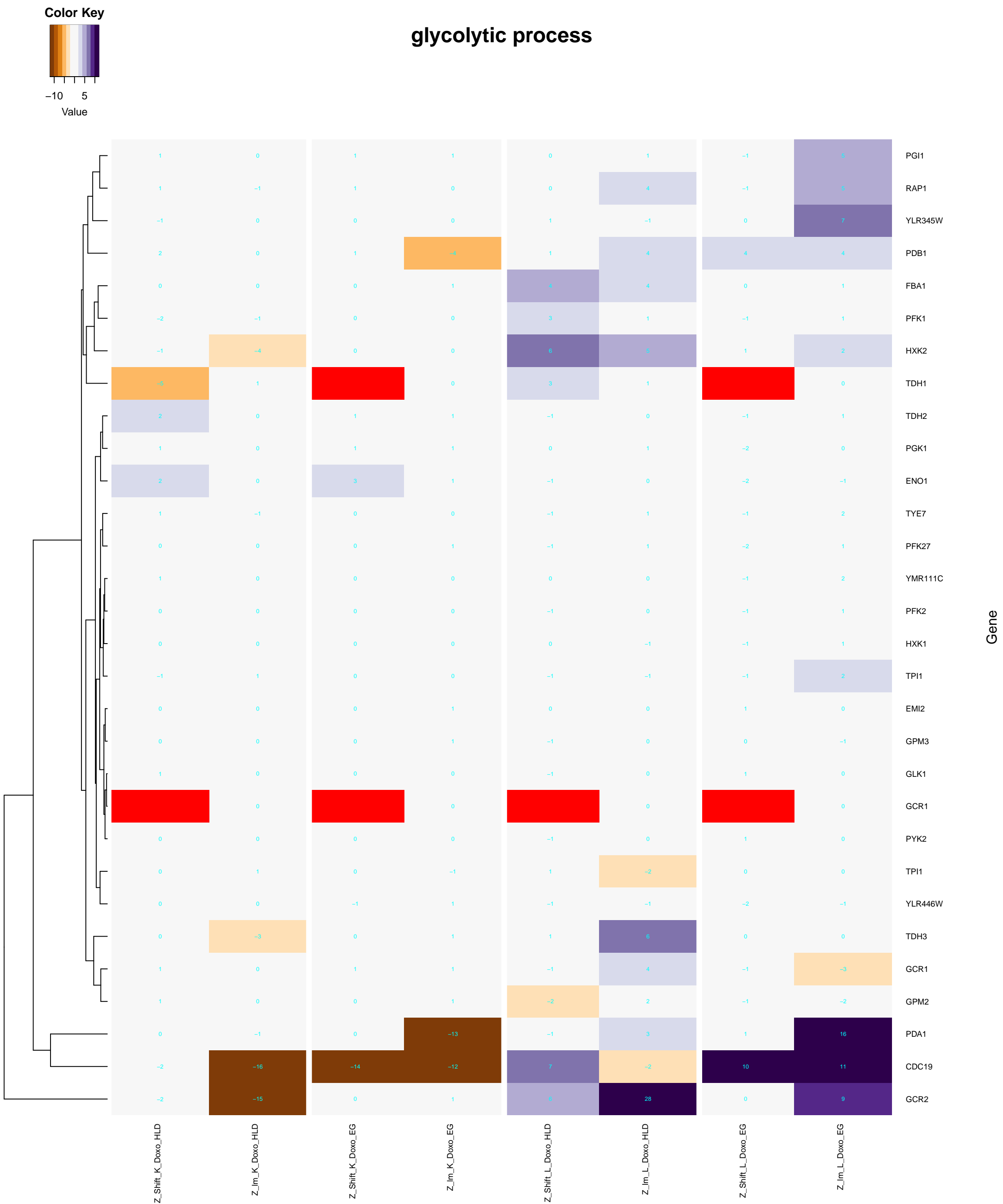

Supplement: Supplementary file 9 — Additional file 9. GO term-specific heatmaps for REMc/GTF-enriched clusters. GO term-specific heatmaps for significant GO process terms were generated as described in methods and Figs. 3 and 4. Any related child terms are presented in subsequent pages of the parent file name. GO terms with more than 100 children, with 2 or fewer genes annotated to the term, or a file size over 300KB are not shown. All heatmaps are generated with the same layout (see Figs. 3 and 4). [file 40170_2019_201_MOESM9_ESM.bz2 › Additional_File9_GOTermHeatmaps/Additional_File9_GOTermHeatmap/nucleotide_phosphorylation.pdf]

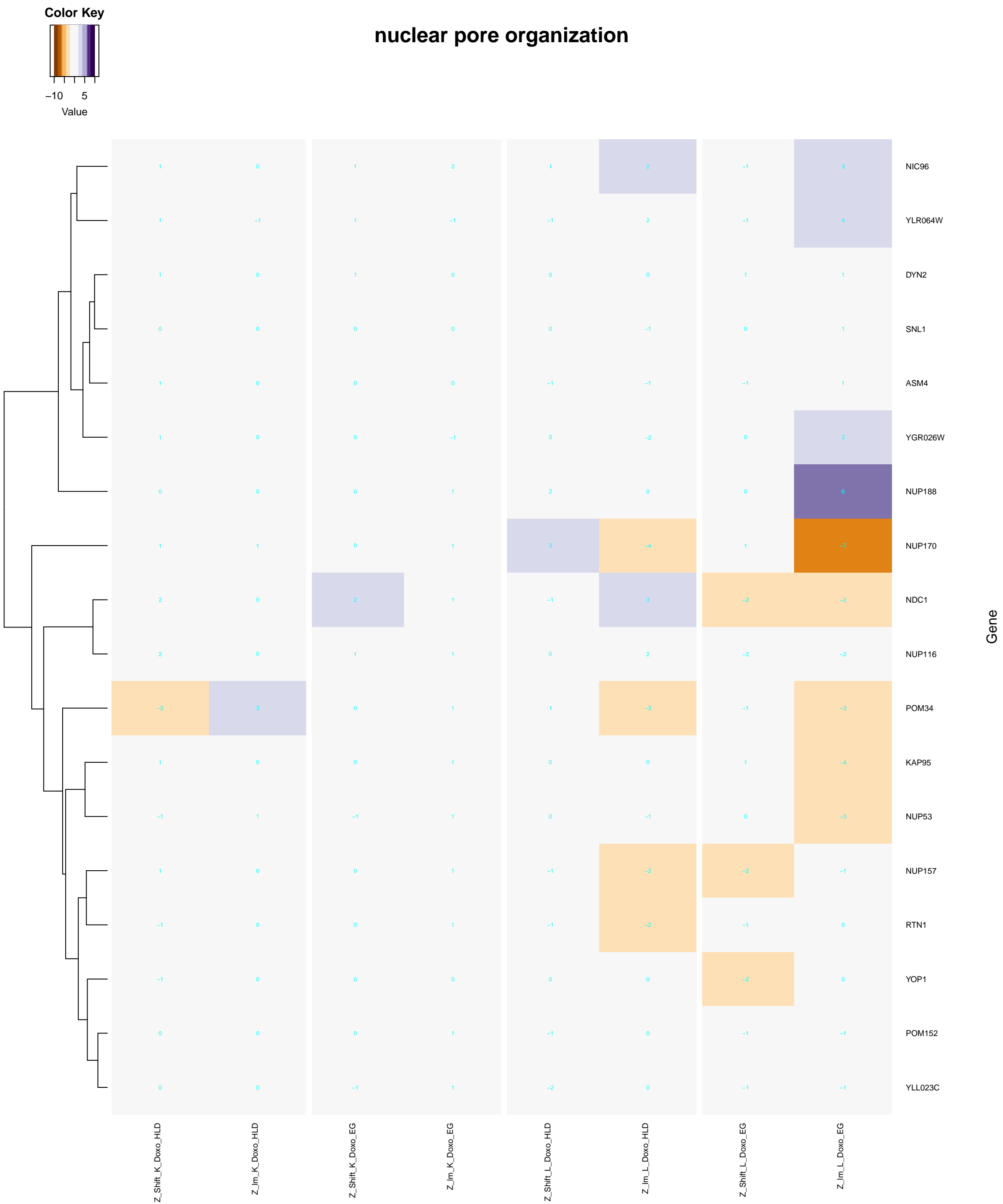

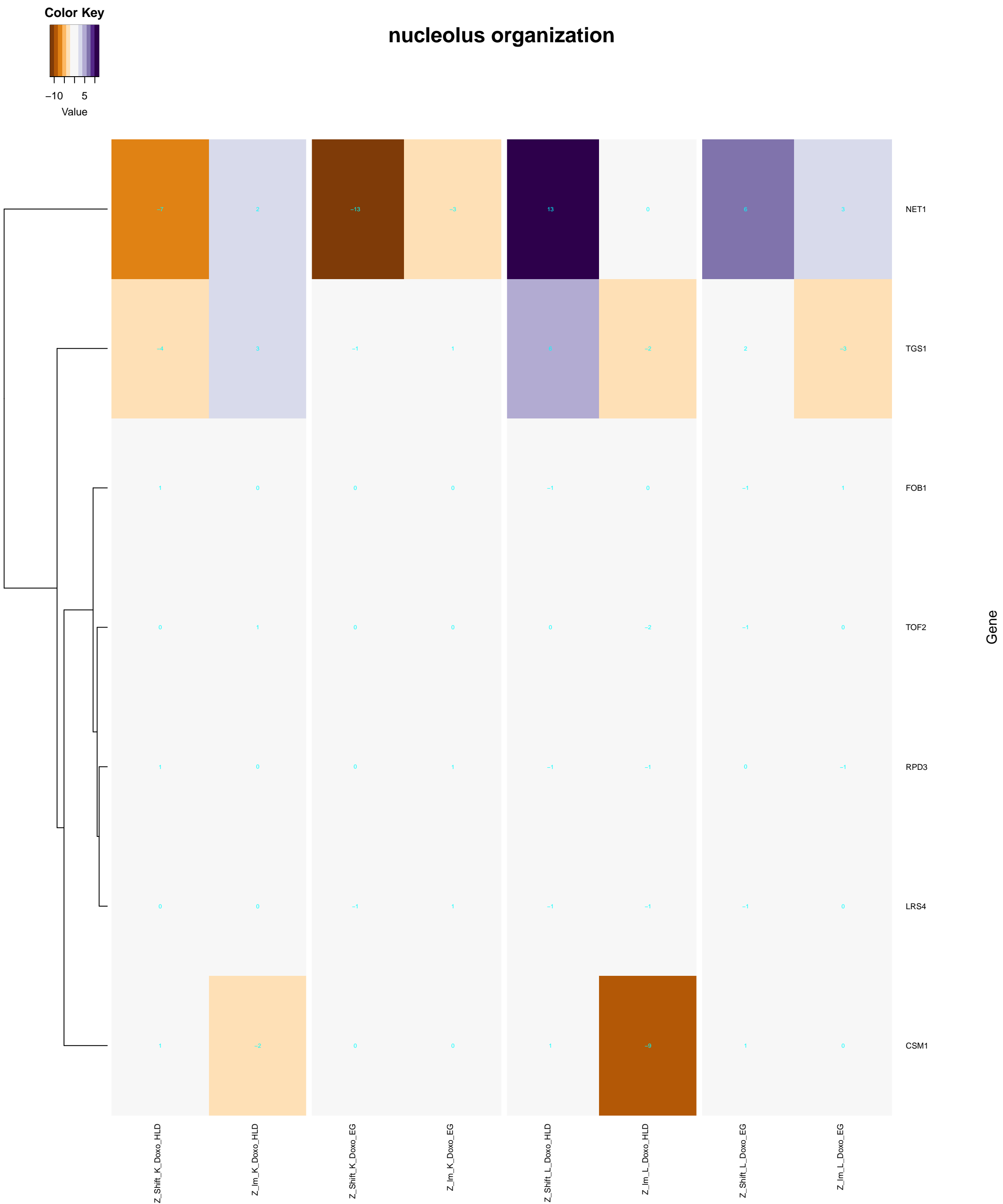

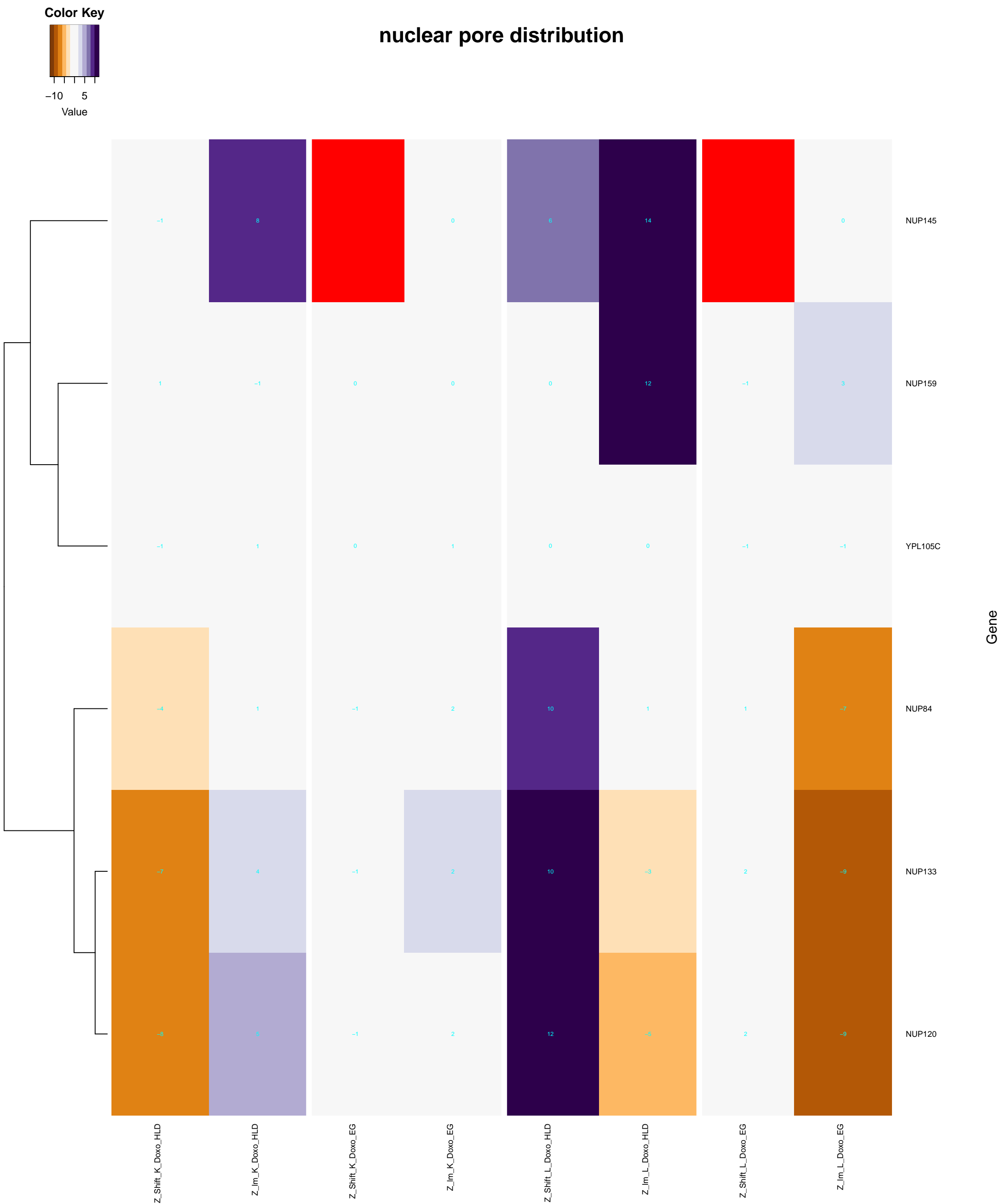

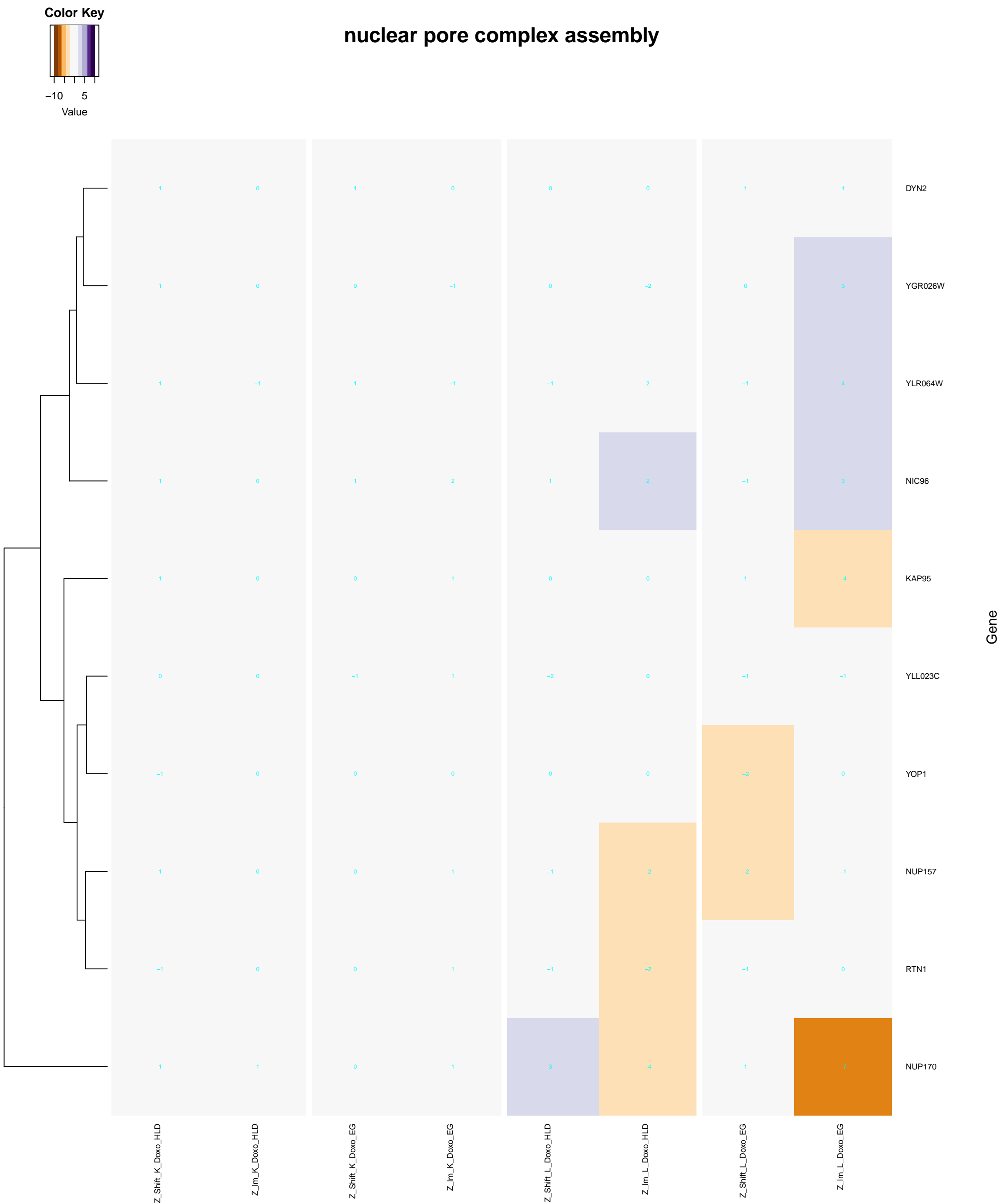

Supplement: Supplementary file 9 — Additional file 9. GO term-specific heatmaps for REMc/GTF-enriched clusters. GO term-specific heatmaps for significant GO process terms were generated as described in methods and Figs. 3 and 4. Any related child terms are presented in subsequent pages of the parent file name. GO terms with more than 100 children, with 2 or fewer genes annotated to the term, or a file size over 300KB are not shown. All heatmaps are generated with the same layout (see Figs. 3 and 4). [file 40170_2019_201_MOESM9_ESM.bz2 › Additional_File9_GOTermHeatmaps/Additional_File9_GOTermHeatmap/nucleus_organization.pdf]

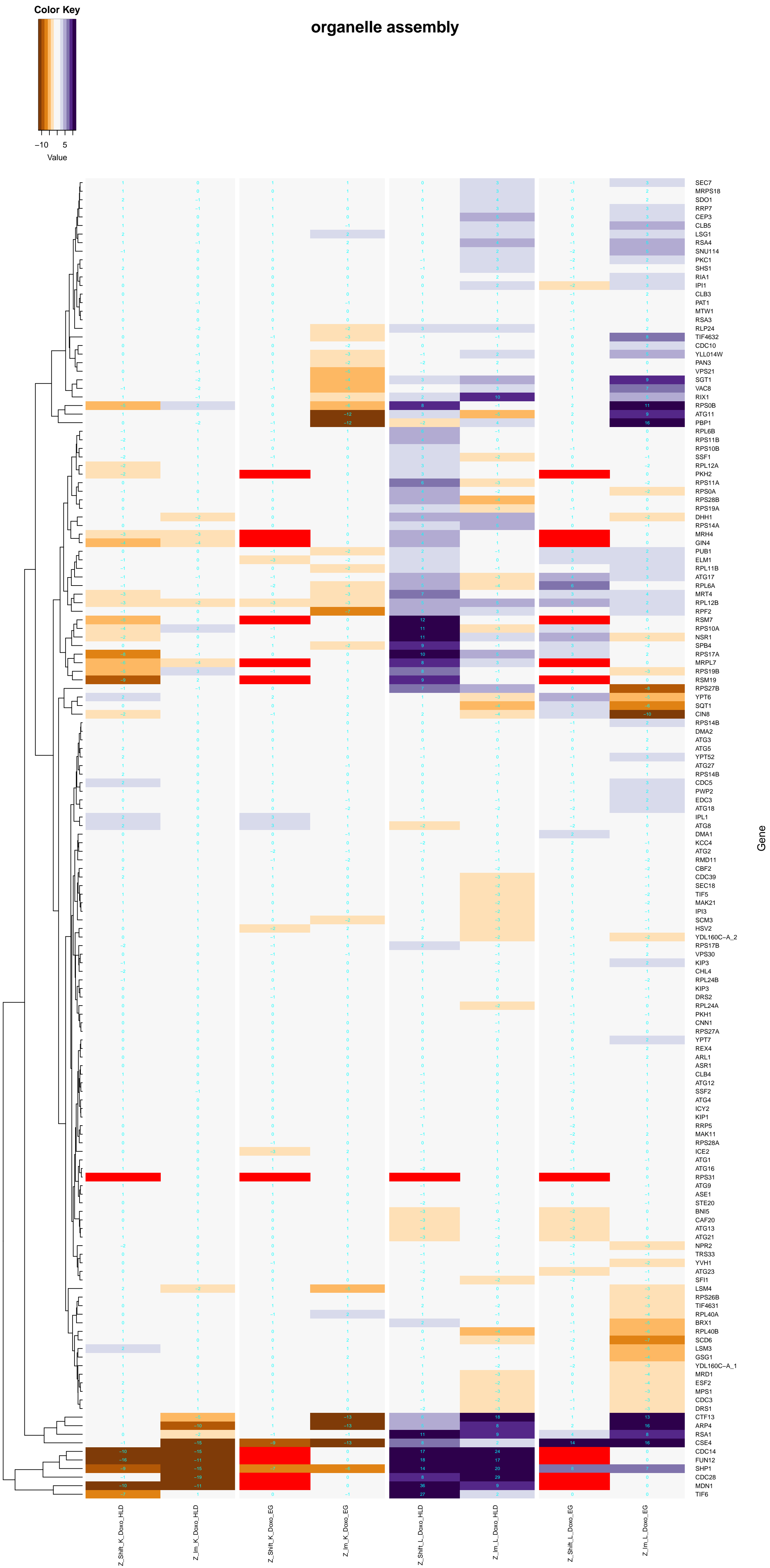

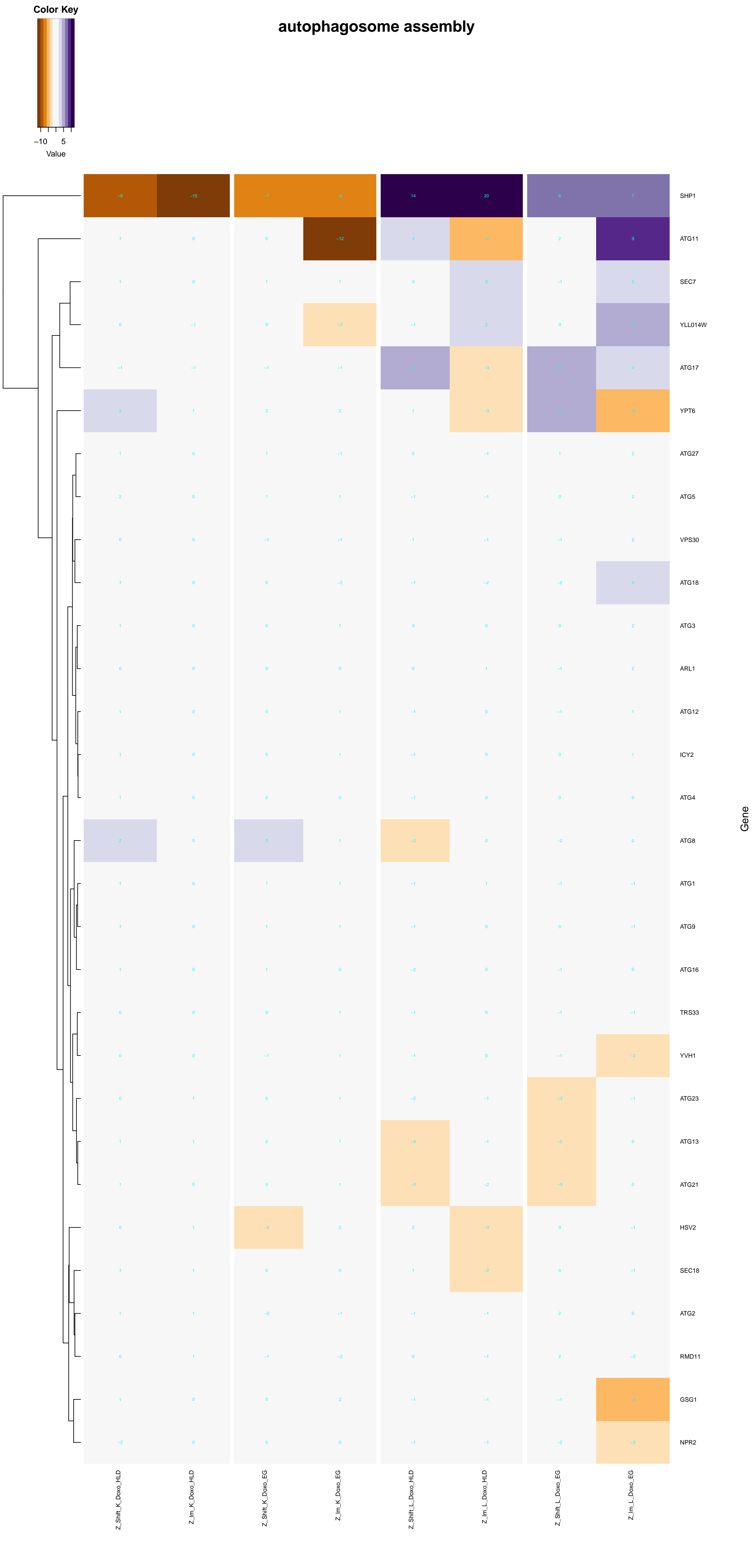

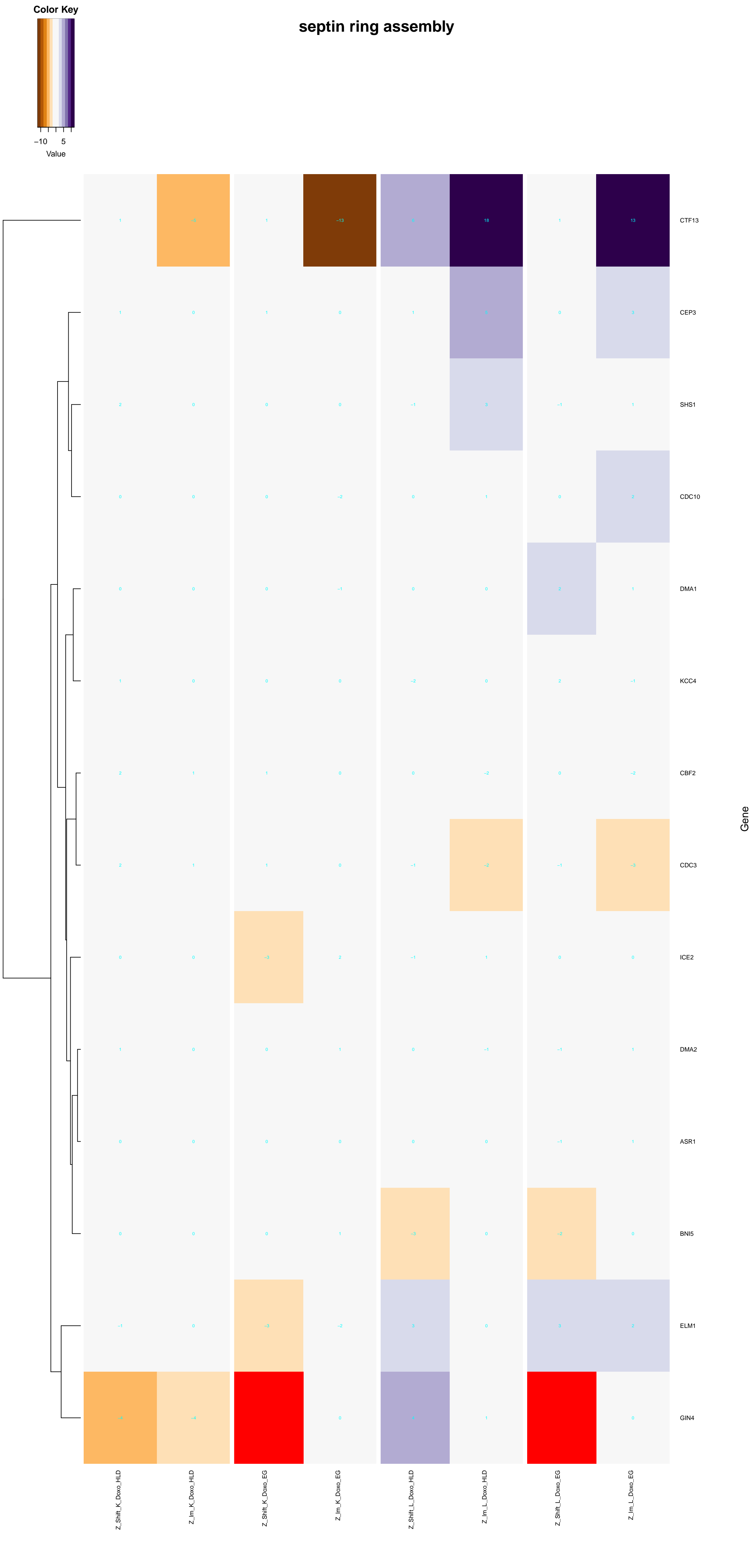

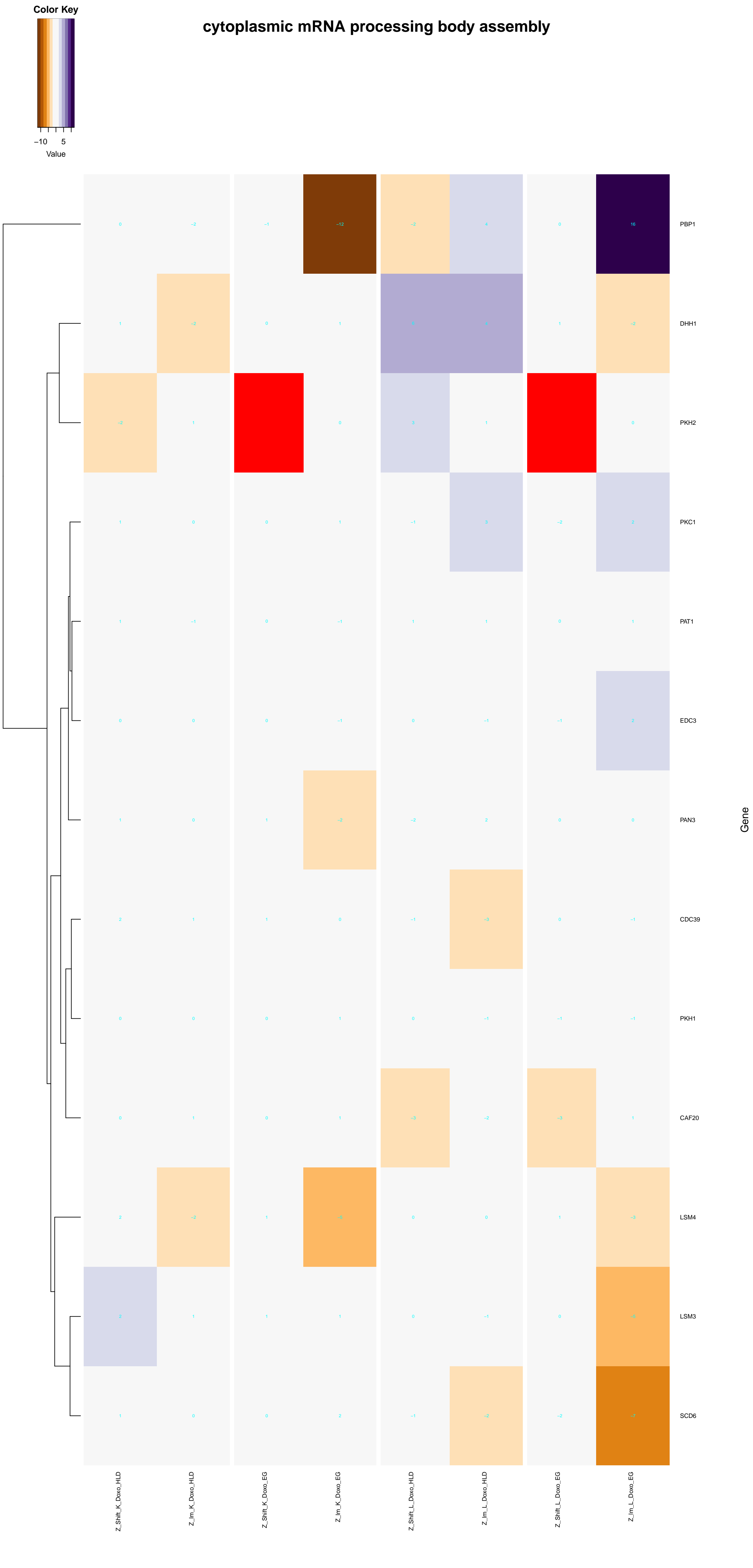

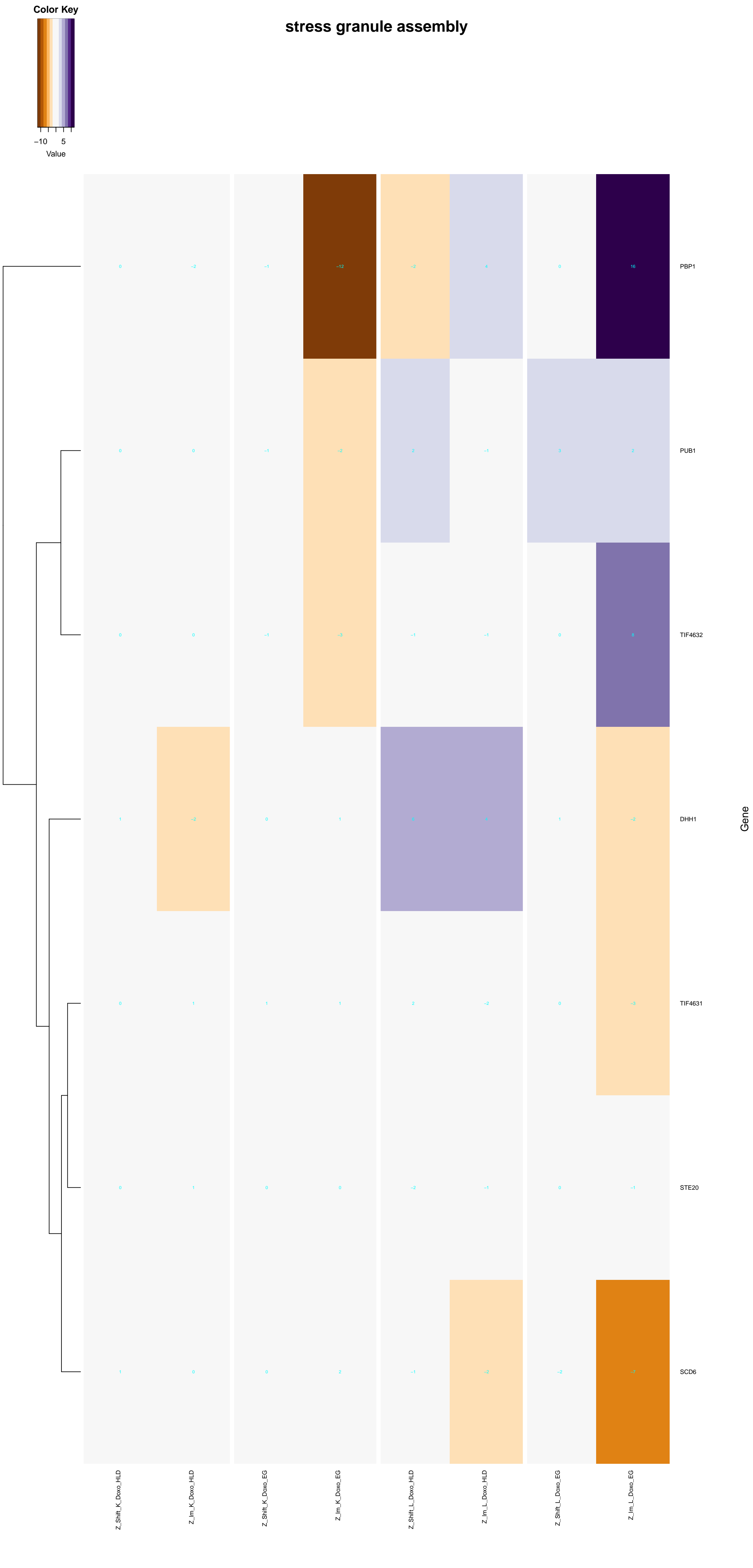



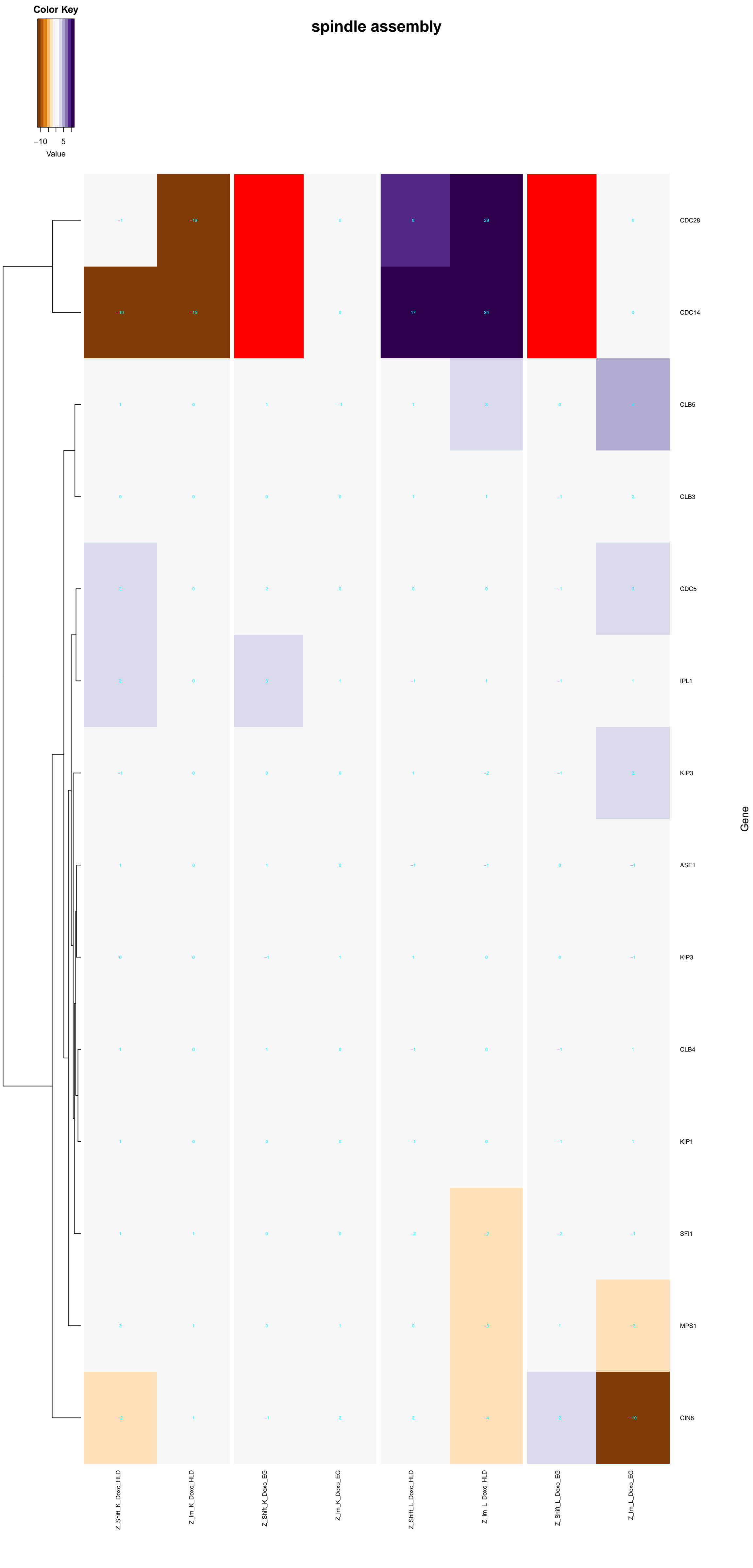

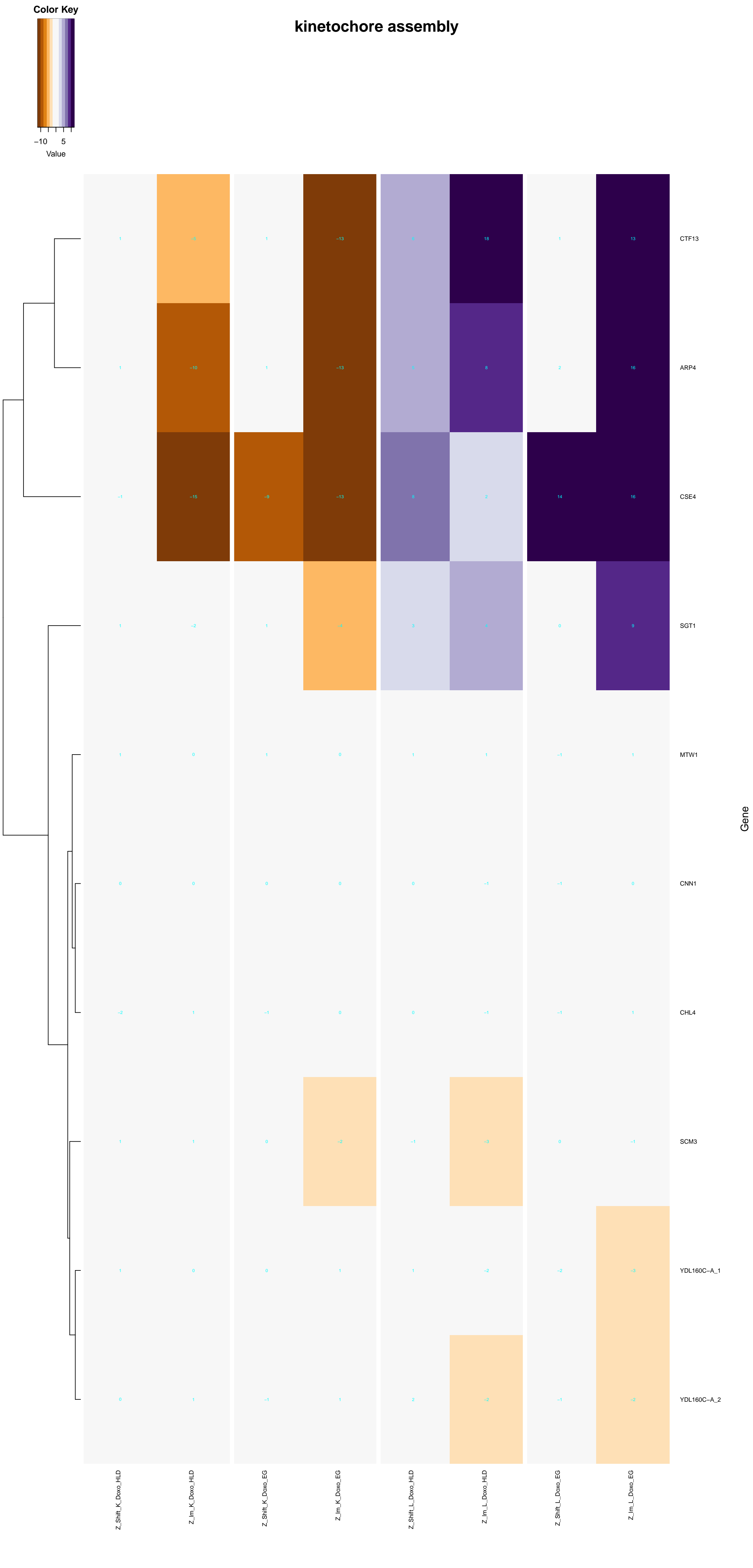

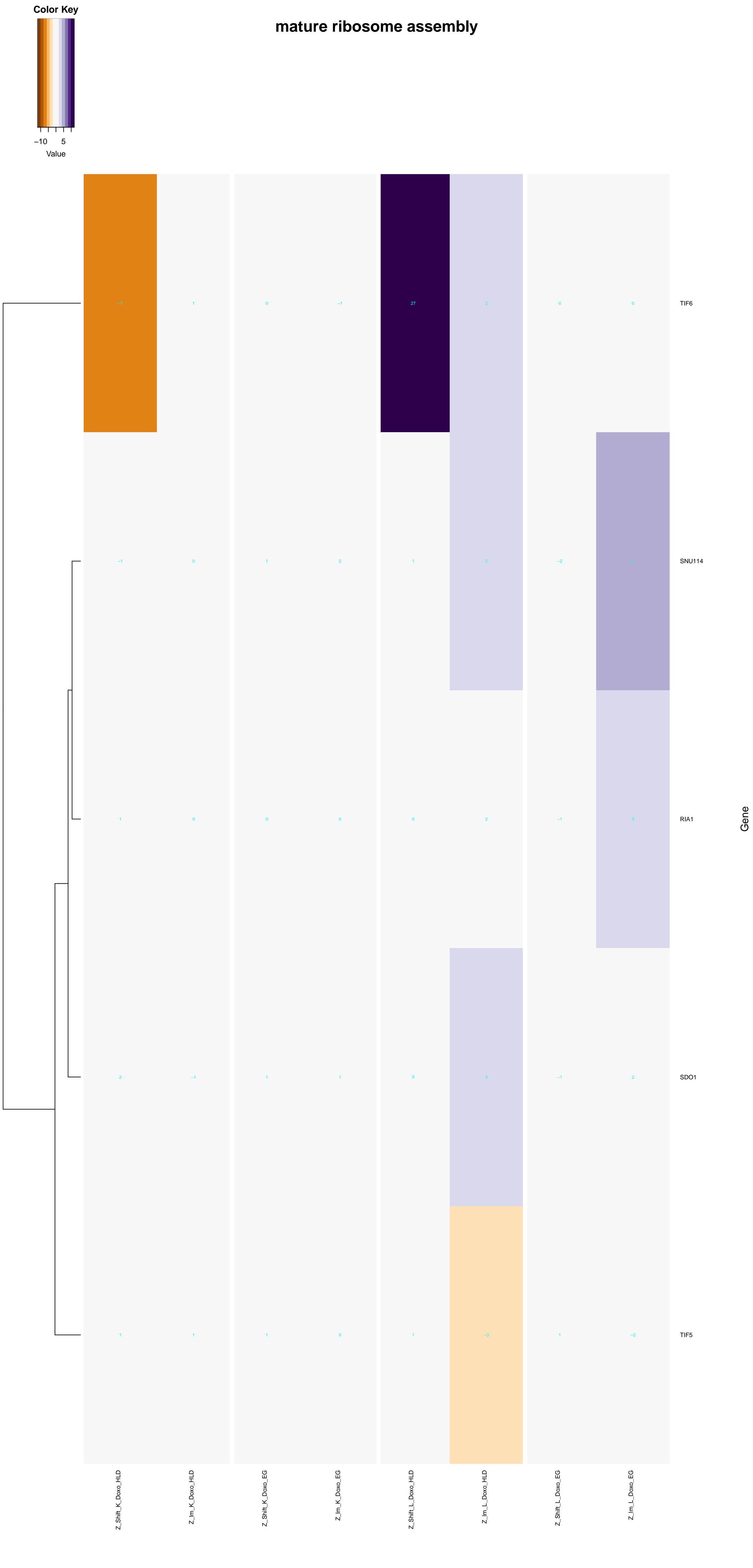

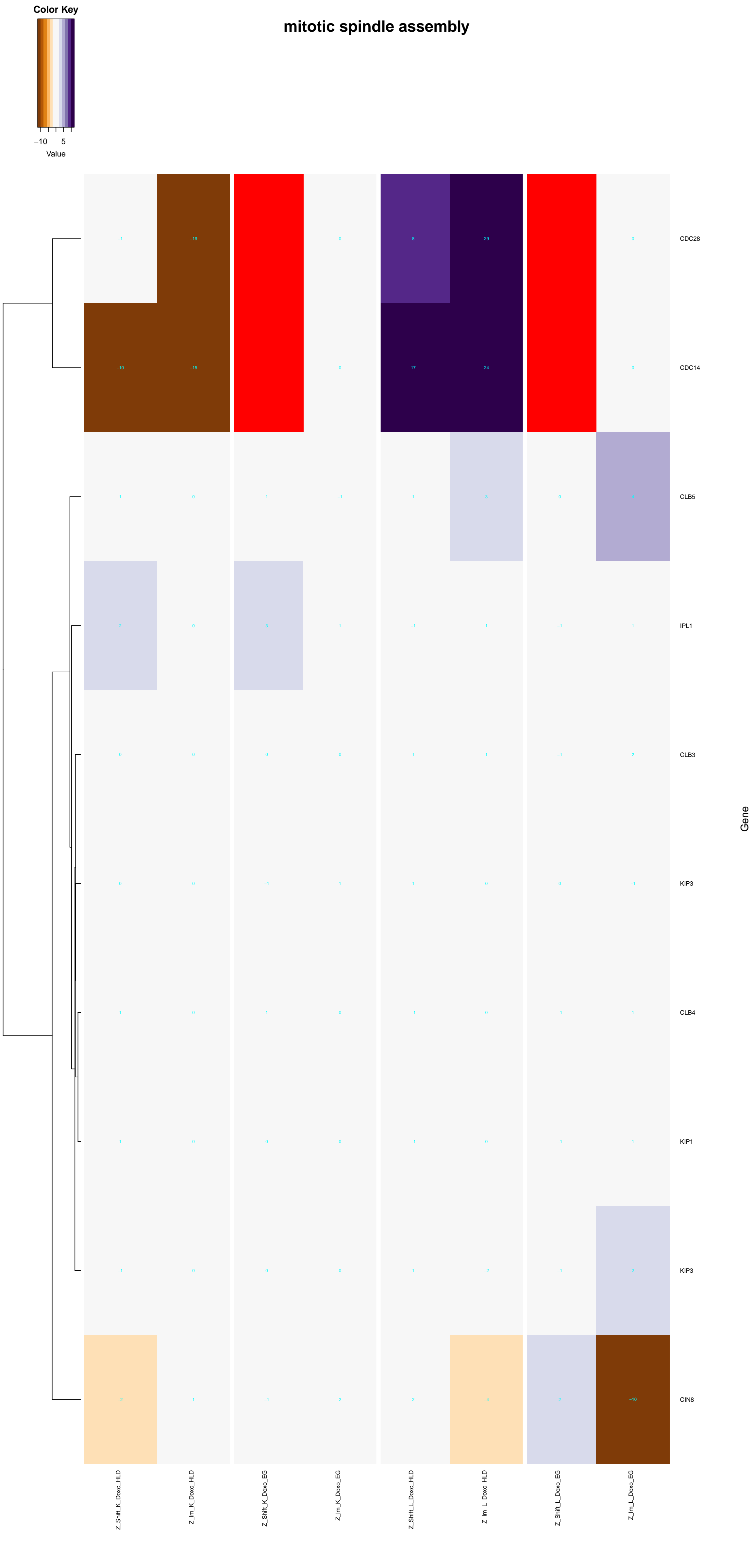

Supplement: Supplementary file 9 — Additional file 9. GO term-specific heatmaps for REMc/GTF-enriched clusters. GO term-specific heatmaps for significant GO process terms were generated as described in methods and Figs. 3 and 4. Any related child terms are presented in subsequent pages of the parent file name. GO terms with more than 100 children, with 2 or fewer genes annotated to the term, or a file size over 300KB are not shown. All heatmaps are generated with the same layout (see Figs. 3 and 4). [file 40170_2019_201_MOESM9_ESM.bz2 › Additional_File9_GOTermHeatmaps/Additional_File9_GOTermHeatmap/organelle_assembly.pdf]

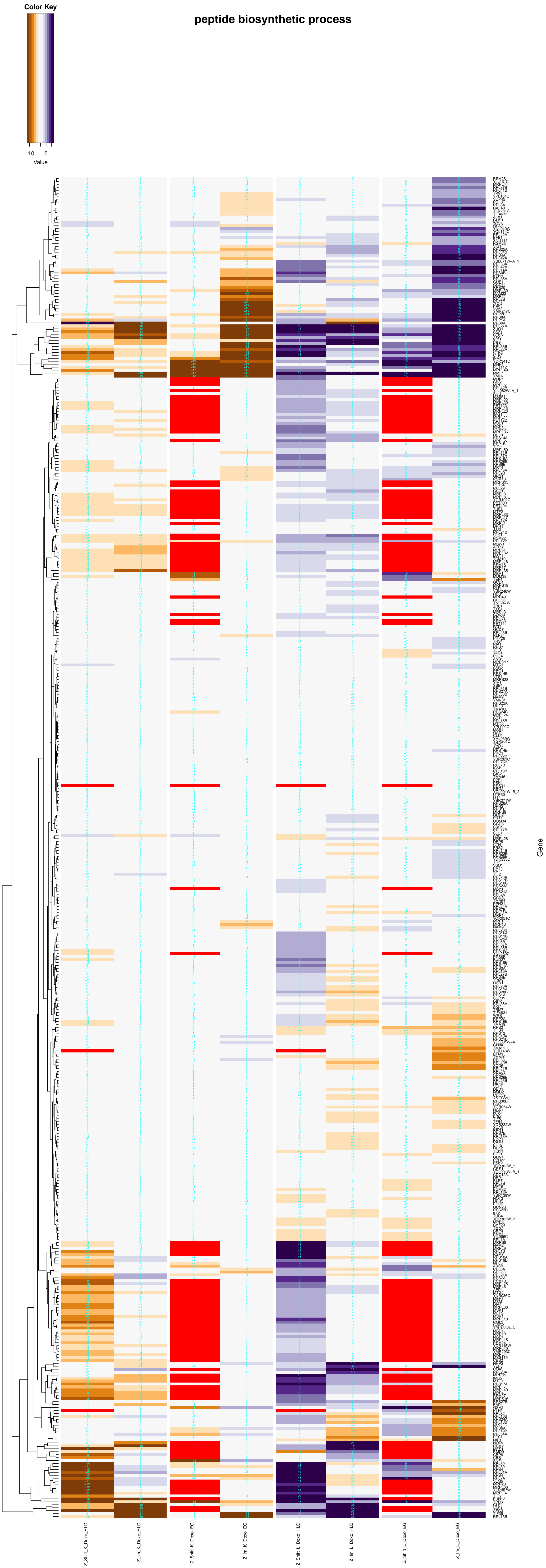

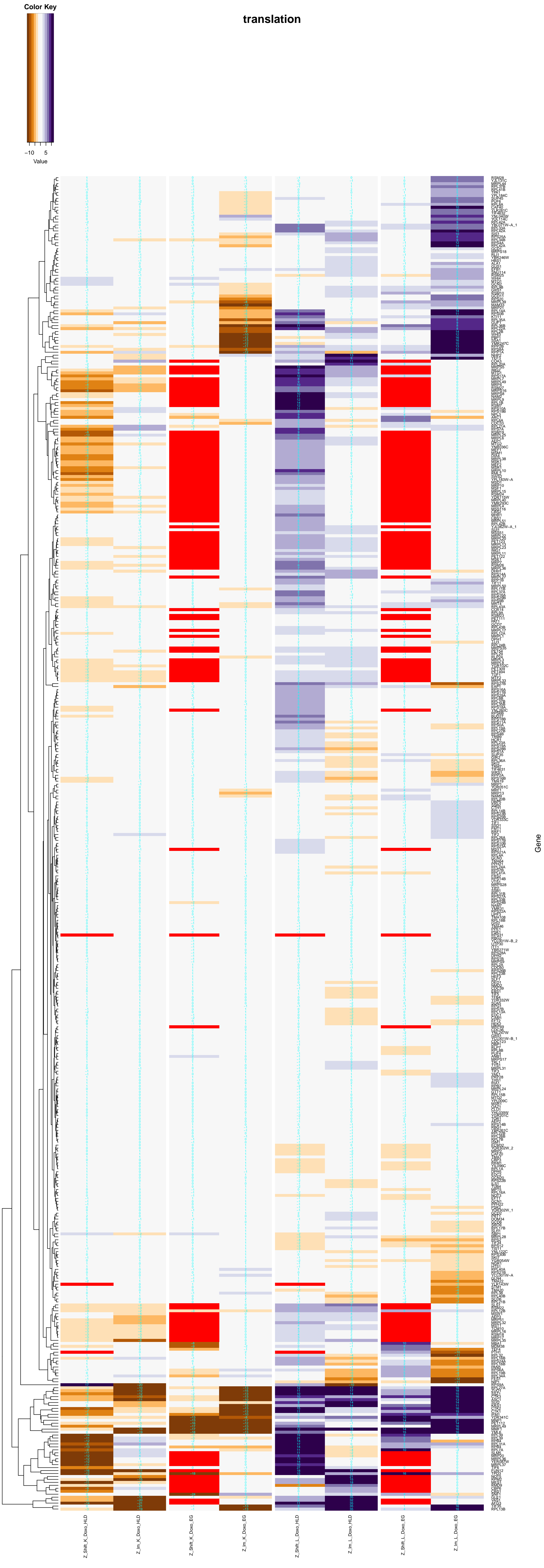



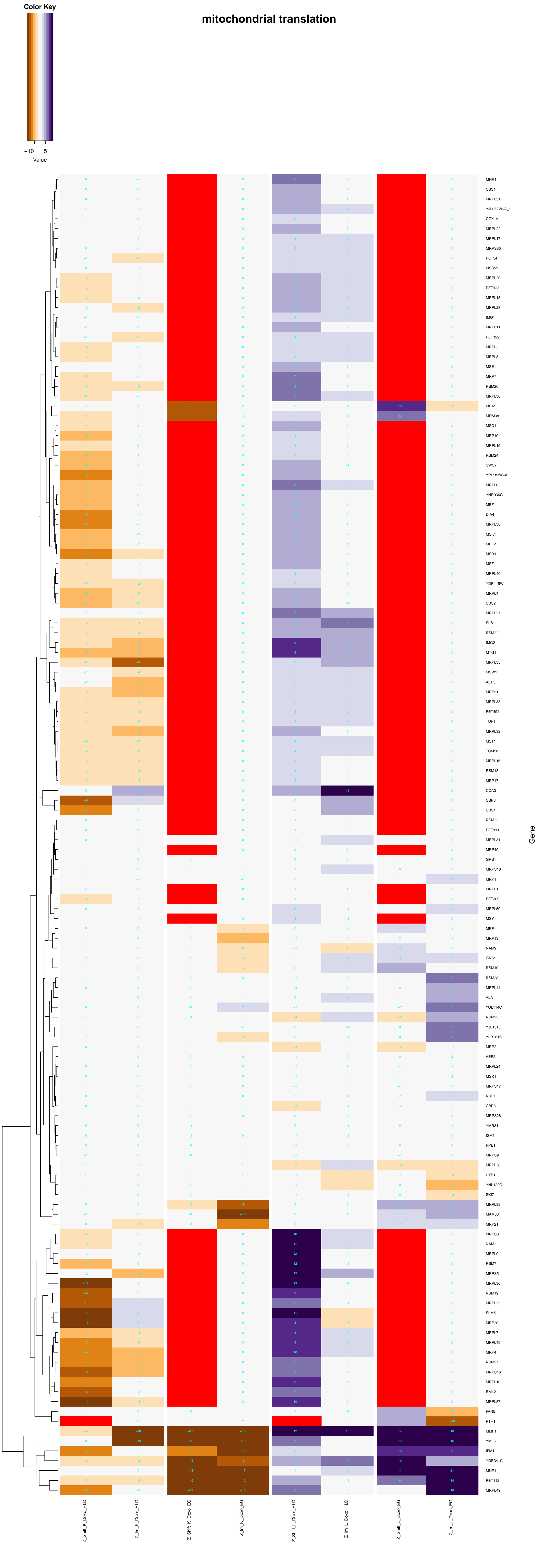

Supplement: Supplementary file 9 — Additional file 9. GO term-specific heatmaps for REMc/GTF-enriched clusters. GO term-specific heatmaps for significant GO process terms were generated as described in methods and Figs. 3 and 4. Any related child terms are presented in subsequent pages of the parent file name. GO terms with more than 100 children, with 2 or fewer genes annotated to the term, or a file size over 300KB are not shown. All heatmaps are generated with the same layout (see Figs. 3 and 4). [file 40170_2019_201_MOESM9_ESM.bz2 › Additional_File9_GOTermHeatmaps/Additional_File9_GOTermHeatmap/peptide_biosynthetic_process.pdf]

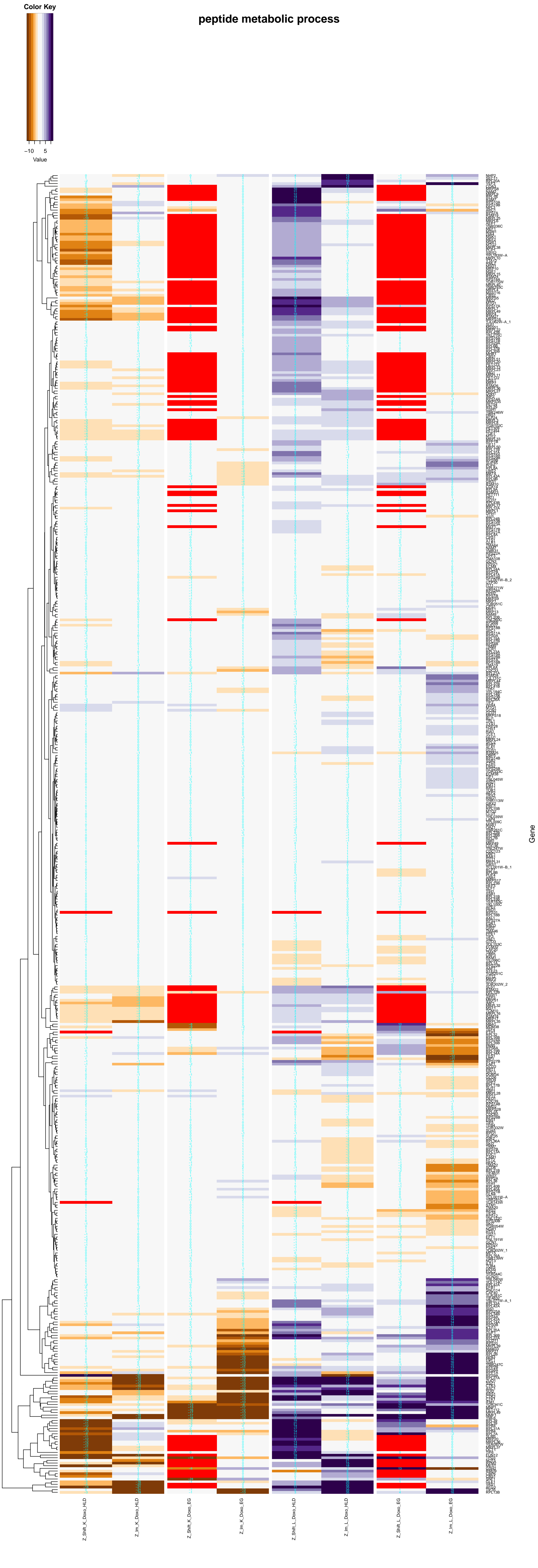

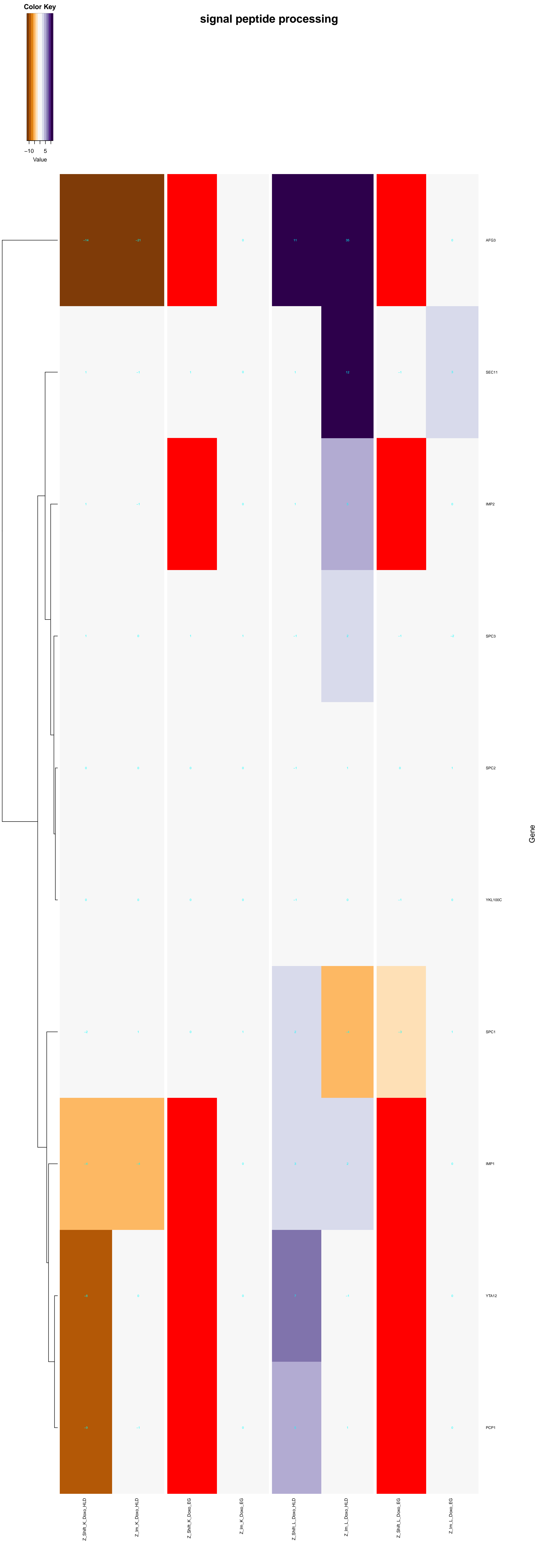

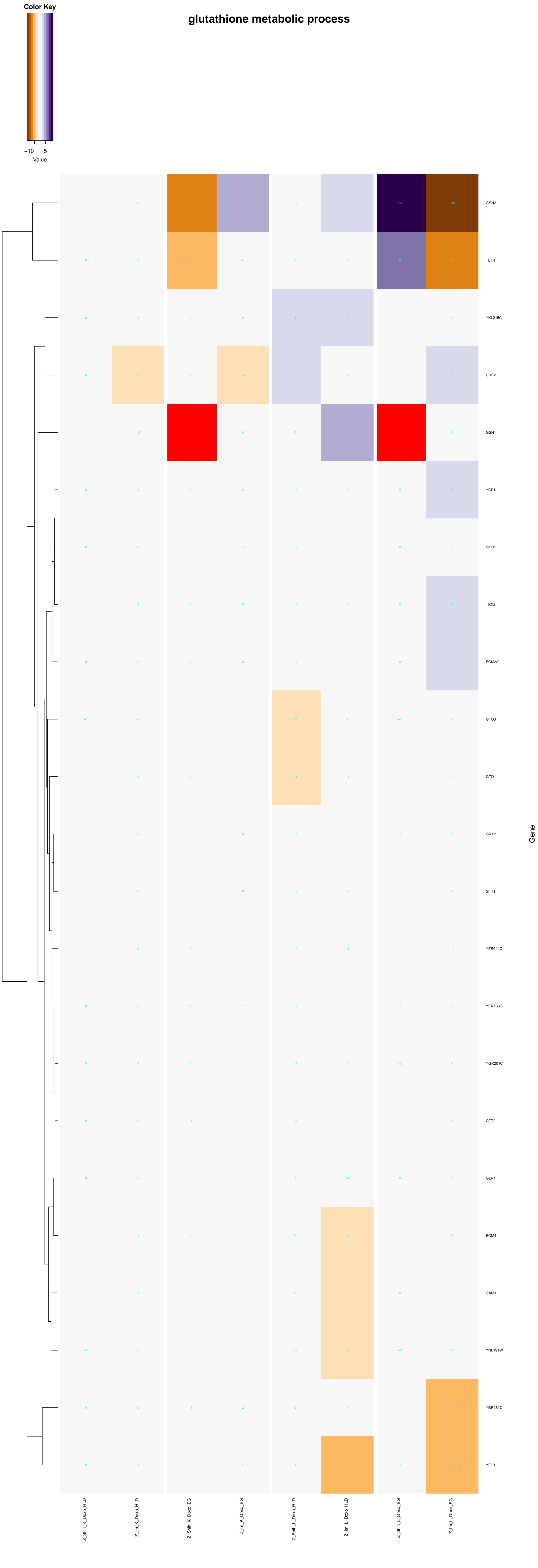

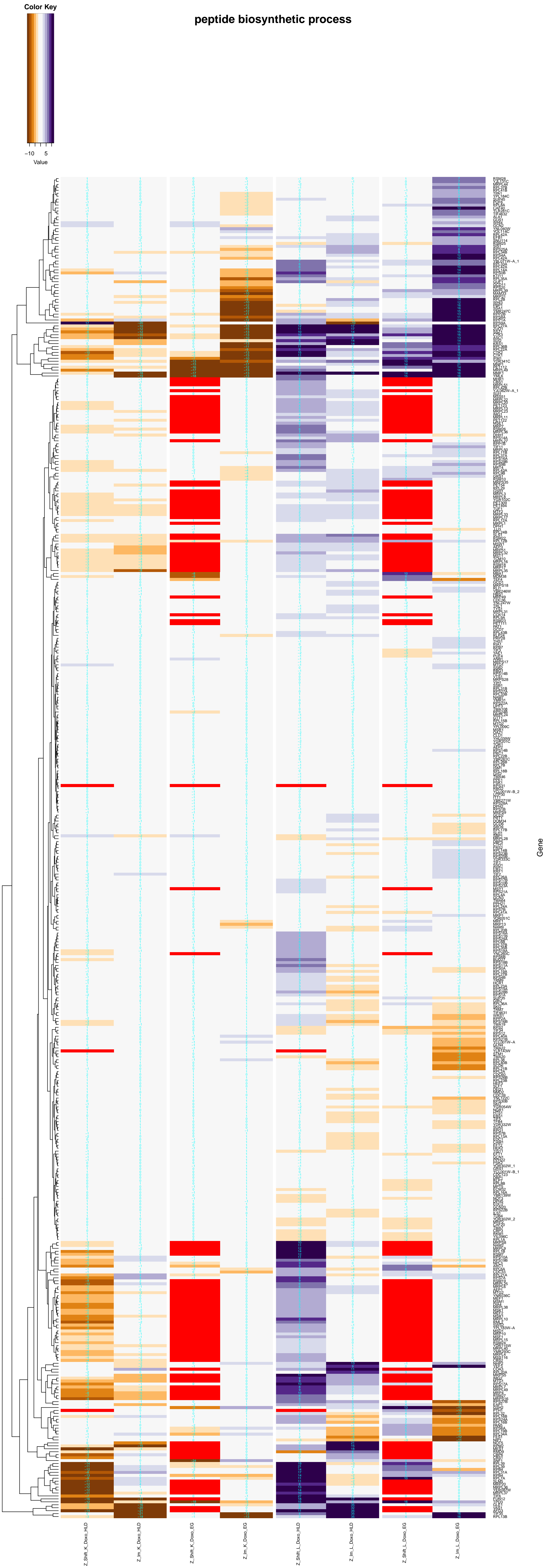

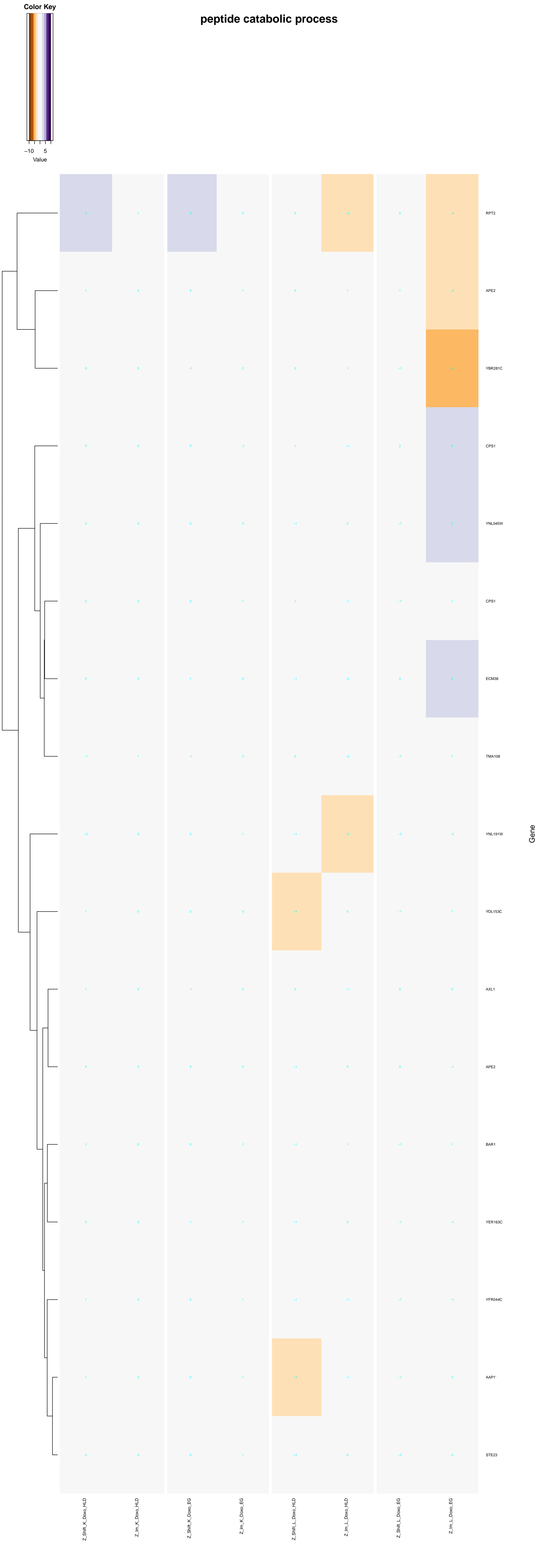

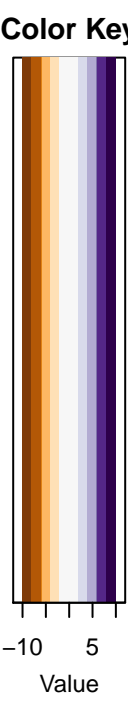

glutathione catabolic process

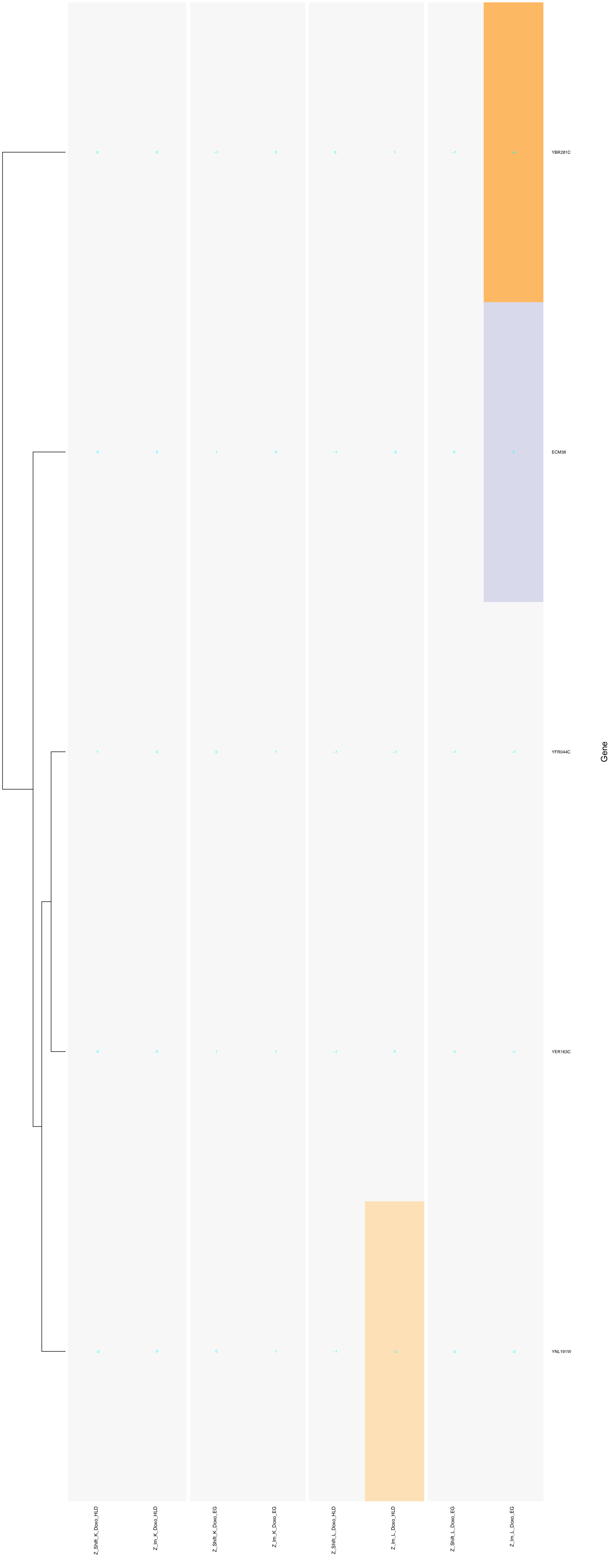

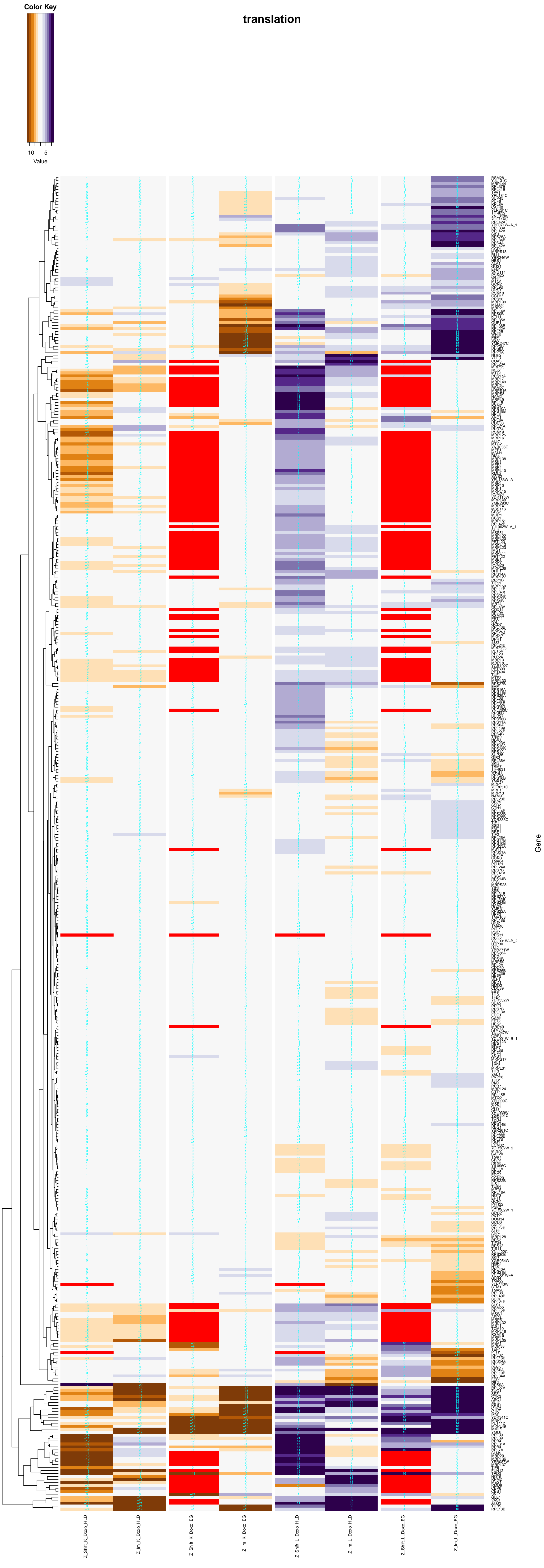

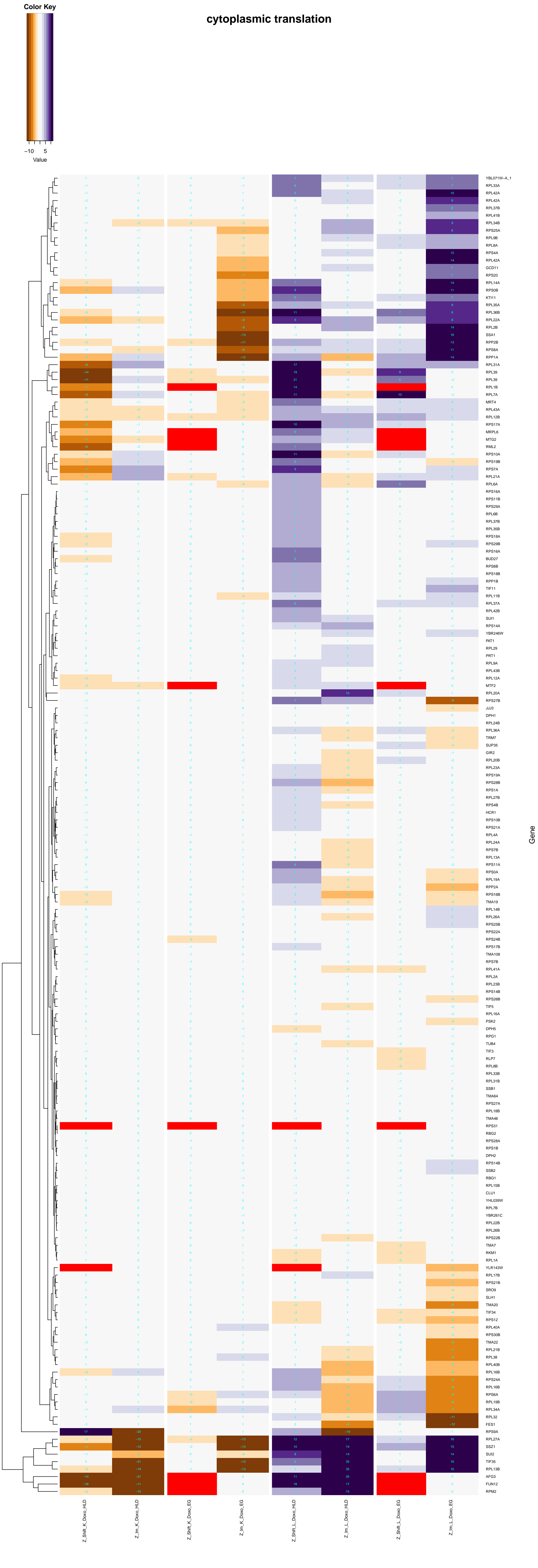

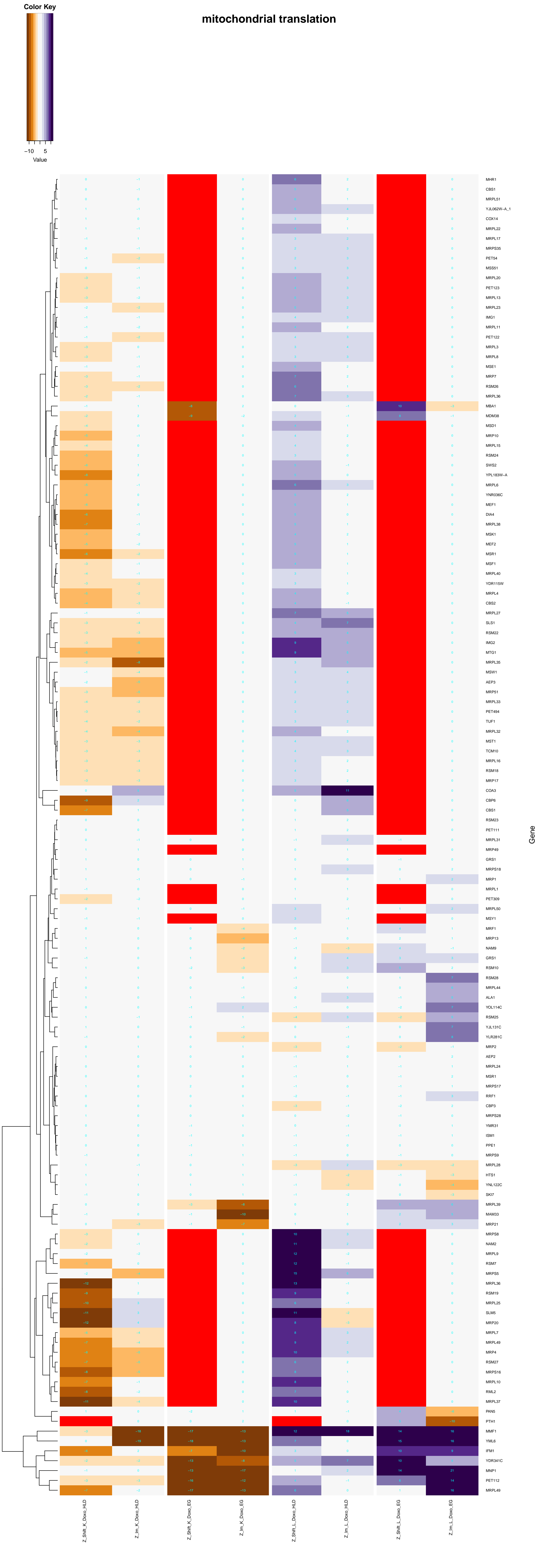

Supplement: Supplementary file 9 — Additional file 9. GO term-specific heatmaps for REMc/GTF-enriched clusters. GO term-specific heatmaps for significant GO process terms were generated as described in methods and Figs. 3 and 4. Any related child terms are presented in subsequent pages of the parent file name. GO terms with more than 100 children, with 2 or fewer genes annotated to the term, or a file size over 300KB are not shown. All heatmaps are generated with the same layout (see Figs. 3 and 4). [file 40170_2019_201_MOESM9_ESM.bz2 › Additional_File9_GOTermHeatmaps/Additional_File9_GOTermHeatmap/peptide_metabolic_process.pdf]

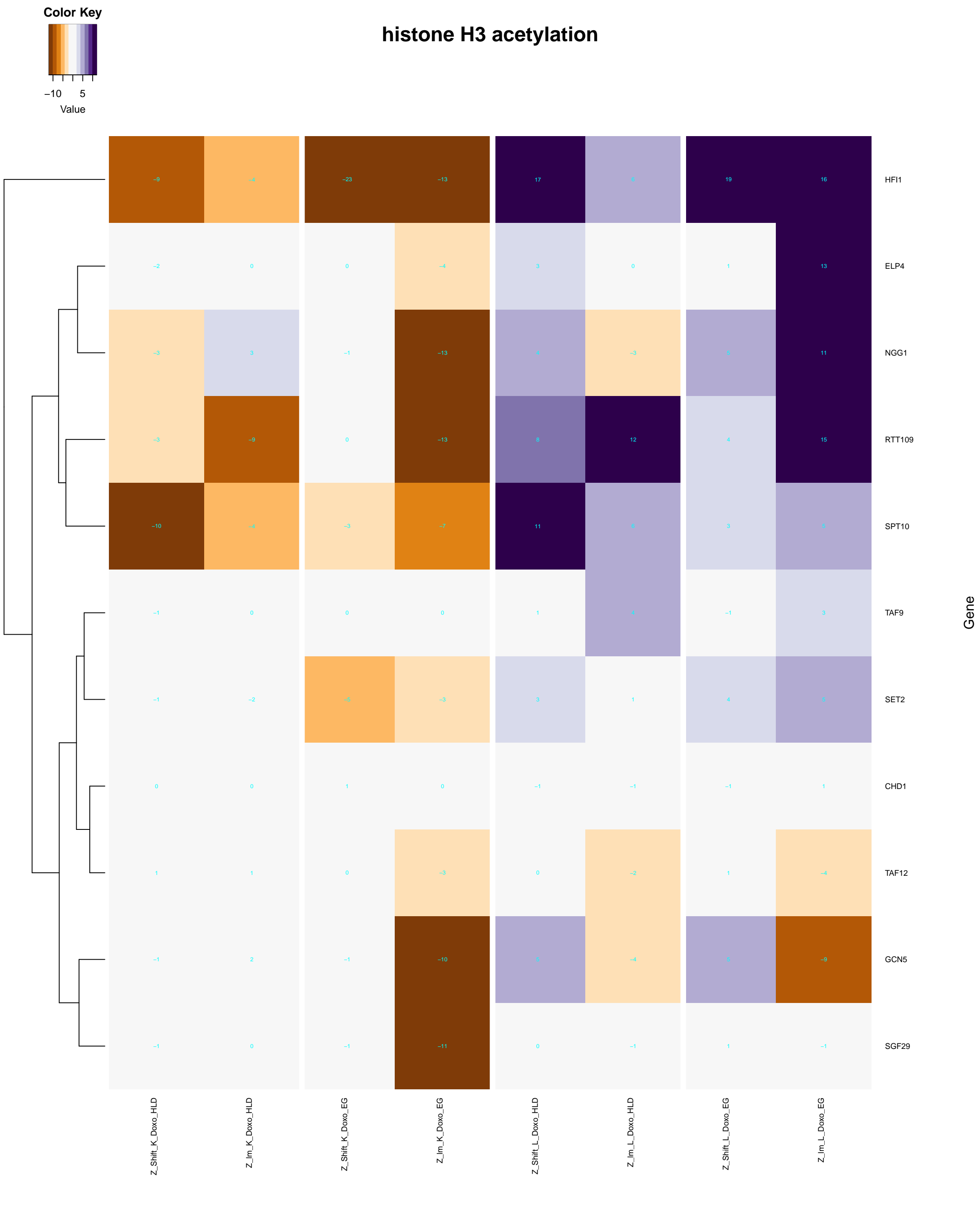

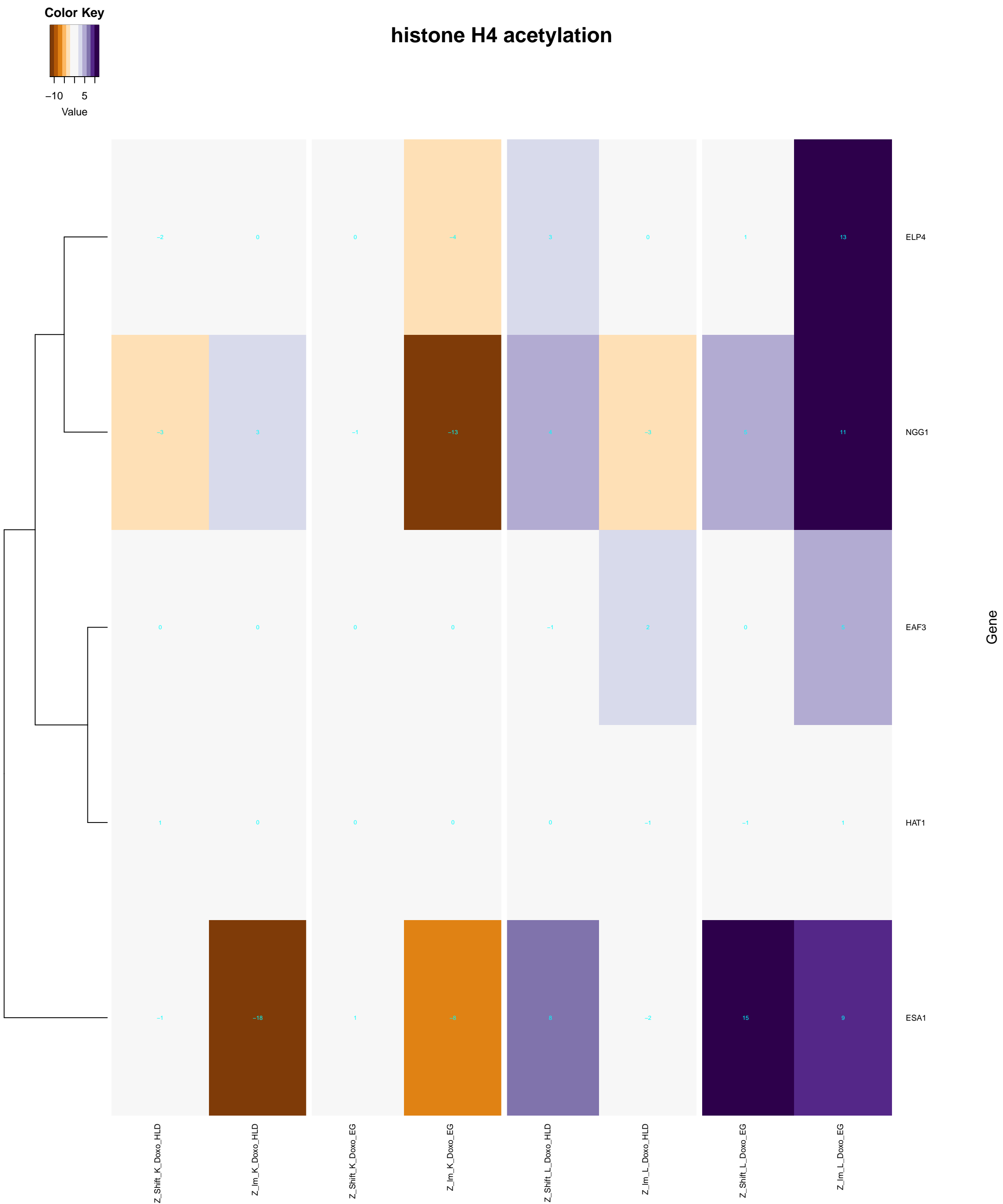

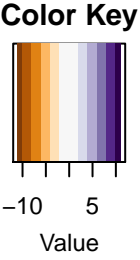

histone H3-K9 acetylation

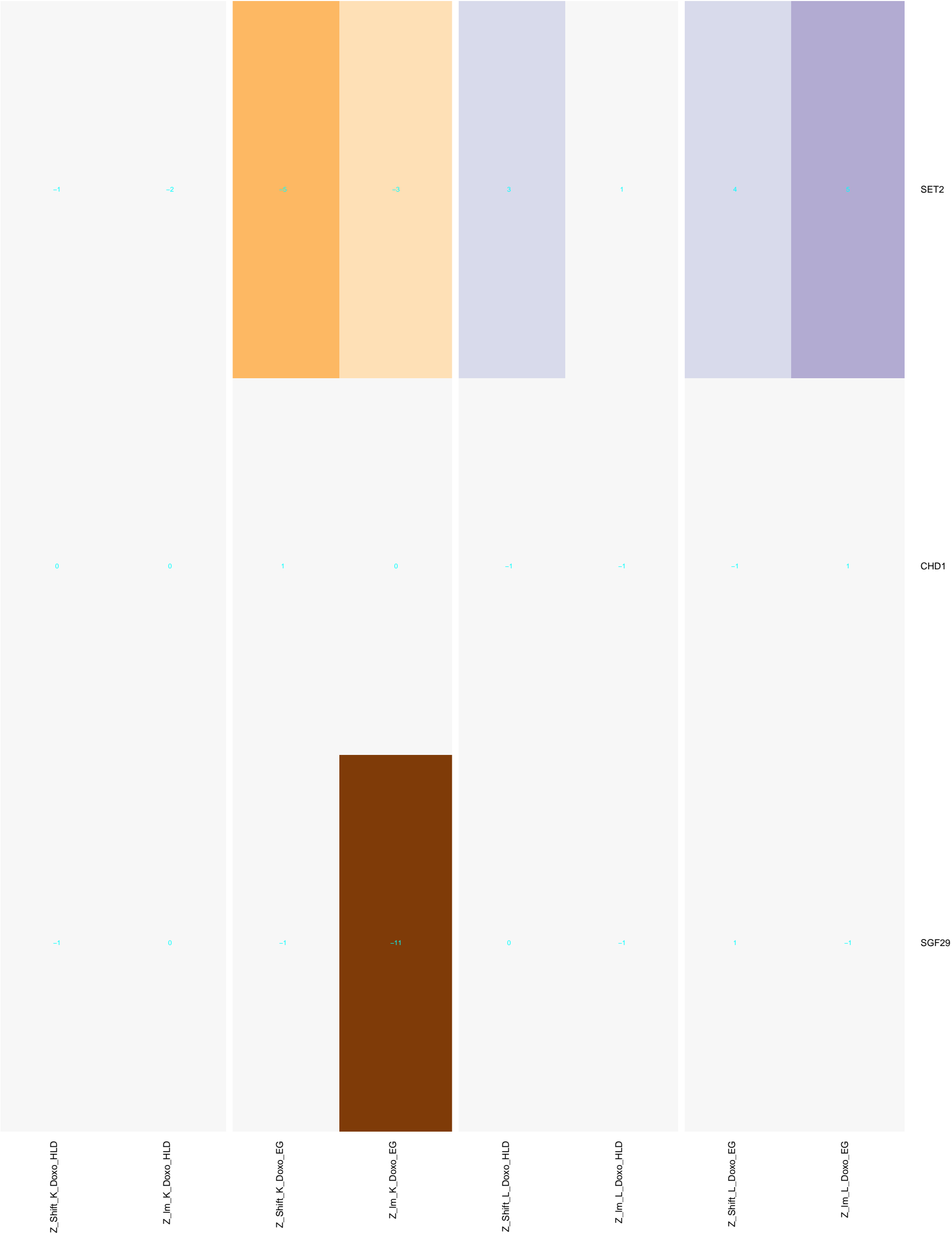

Gene

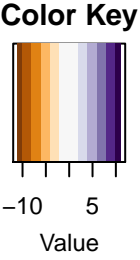

histone H3-K14 acetylation

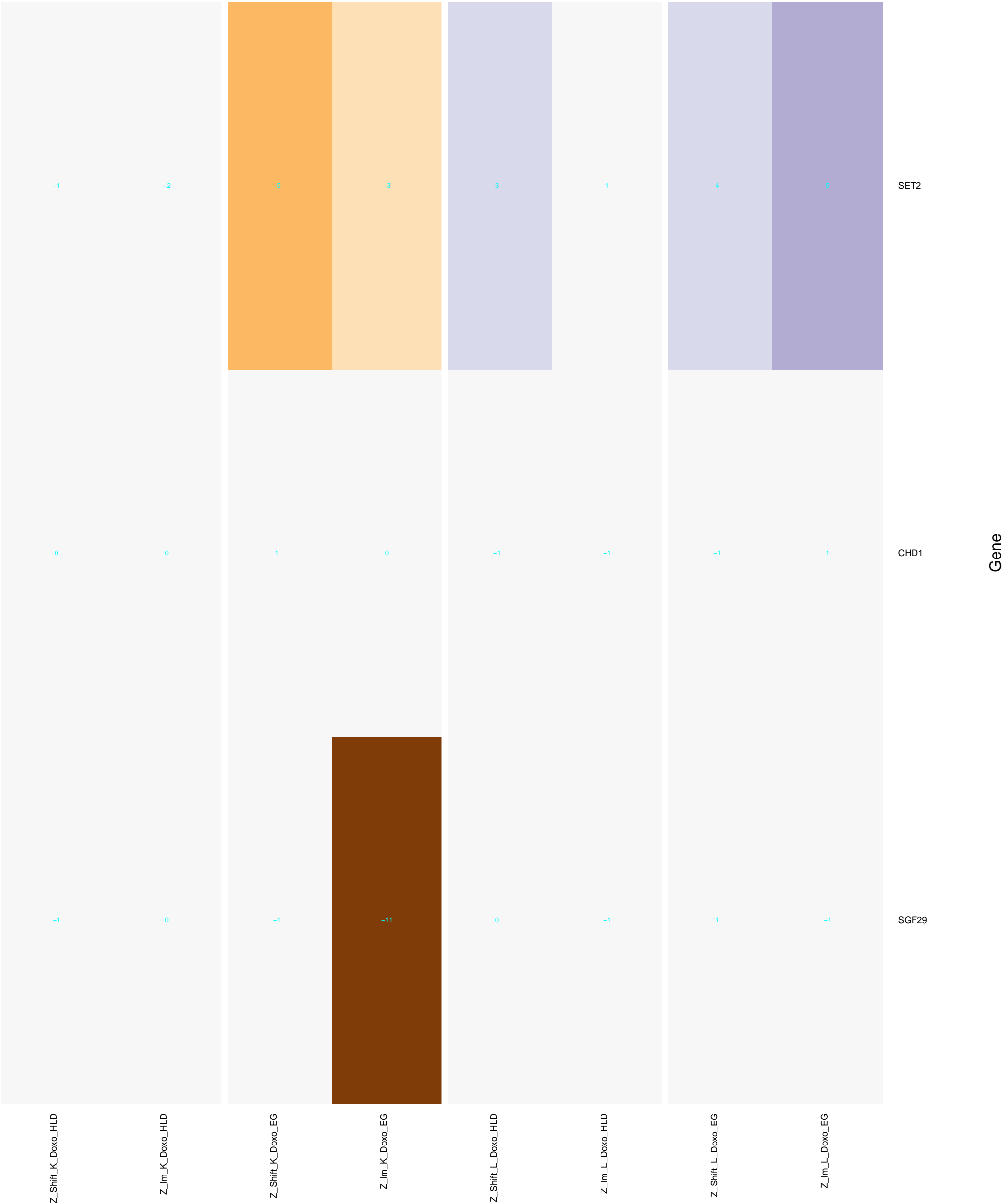

Supplement: Supplementary file 9 — Additional file 9. GO term-specific heatmaps for REMc/GTF-enriched clusters. GO term-specific heatmaps for significant GO process terms were generated as described in methods and Figs. 3 and 4. Any related child terms are presented in subsequent pages of the parent file name. GO terms with more than 100 children, with 2 or fewer genes annotated to the term, or a file size over 300KB are not shown. All heatmaps are generated with the same layout (see Figs. 3 and 4). [file 40170_2019_201_MOESM9_ESM.bz2 › Additional_File9_GOTermHeatmaps/Additional_File9_GOTermHeatmap/peptidyl-lysine_acetylation.pdf]

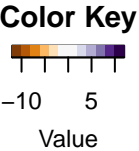

peptidyl-serine modification

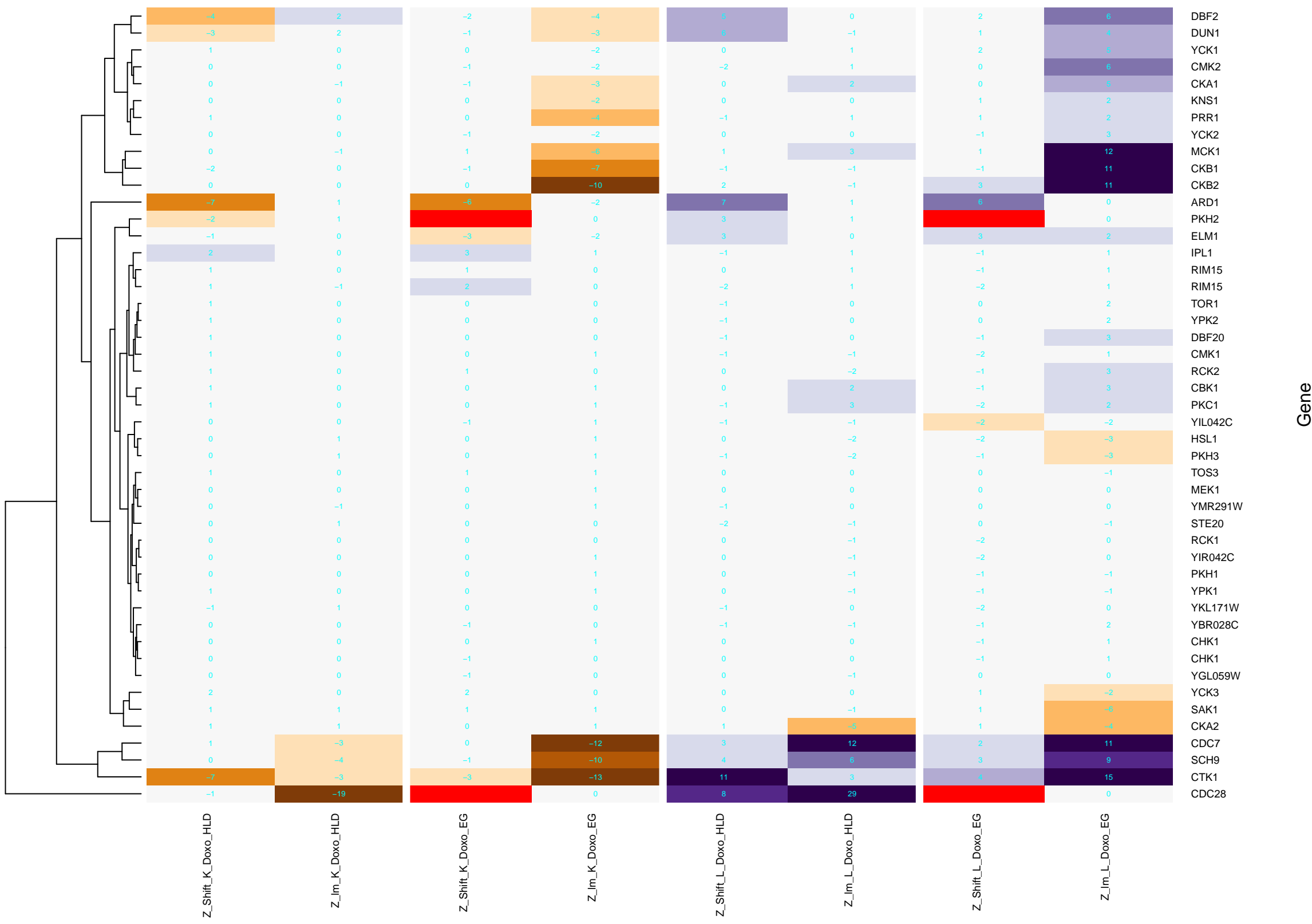

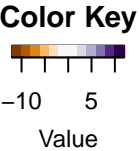

# peptidyl-serine phosphorylation

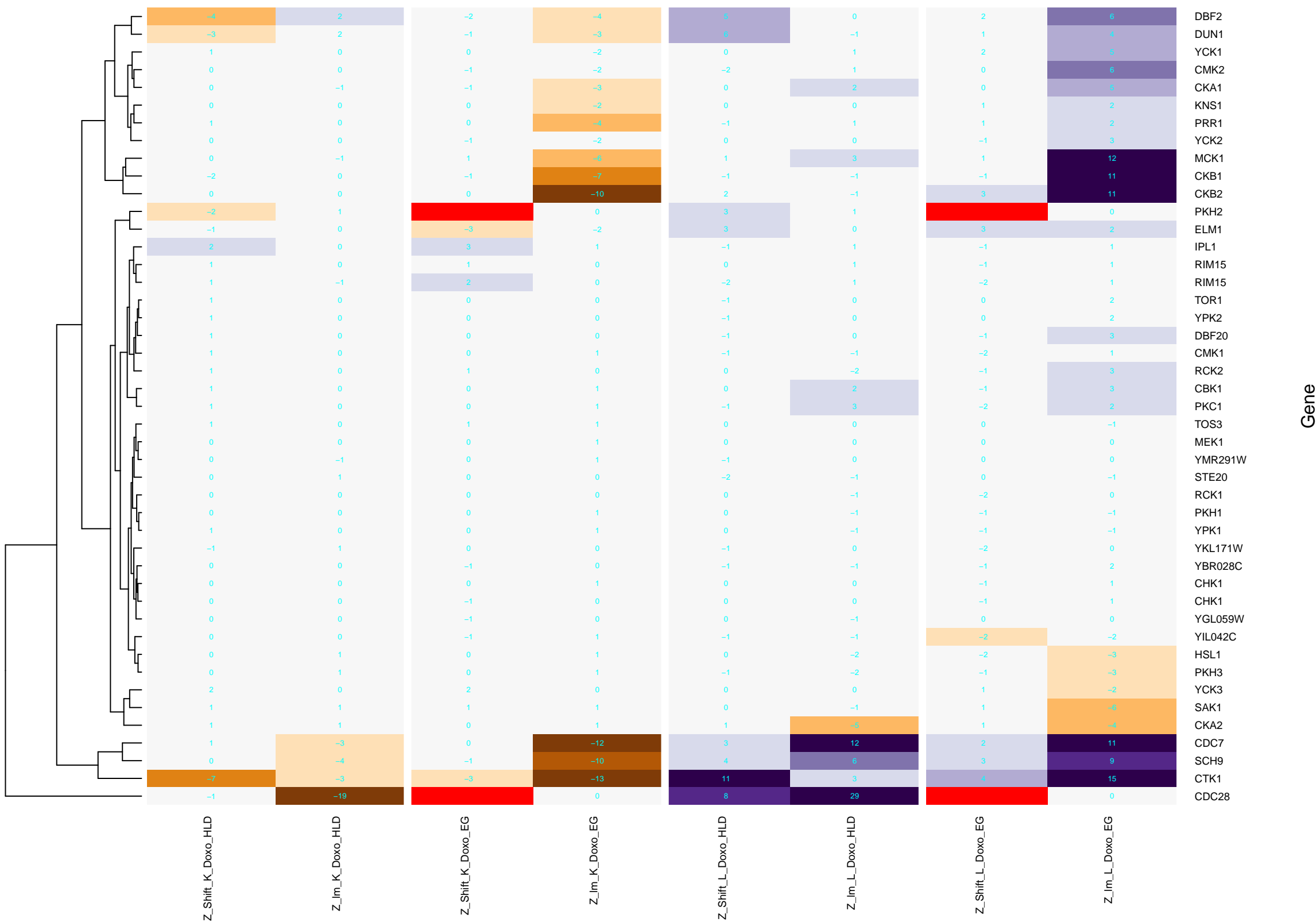

Supplement: Supplementary file 9 — Additional file 9. GO term-specific heatmaps for REMc/GTF-enriched clusters. GO term-specific heatmaps for significant GO process terms were generated as described in methods and Figs. 3 and 4. Any related child terms are presented in subsequent pages of the parent file name. GO terms with more than 100 children, with 2 or fewer genes annotated to the term, or a file size over 300KB are not shown. All heatmaps are generated with the same layout (see Figs. 3 and 4). [file 40170_2019_201_MOESM9_ESM.bz2 › Additional_File9_GOTermHeatmaps/Additional_File9_GOTermHeatmap/peptidyl-serine_modification.pdf]

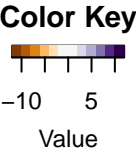

peptidyl-serine phosphorylation

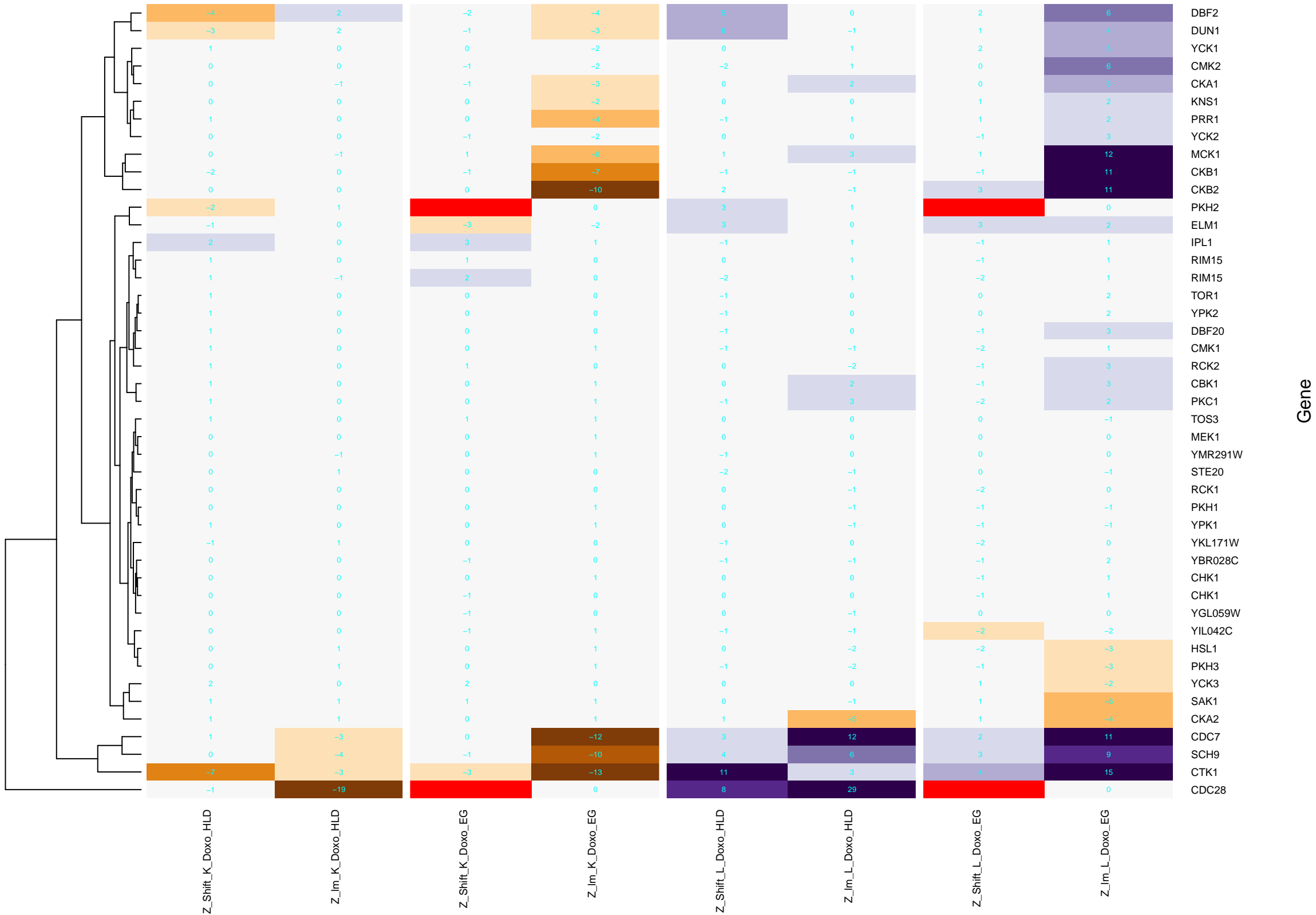

Supplement: Supplementary file 9 — Additional file 9. GO term-specific heatmaps for REMc/GTF-enriched clusters. GO term-specific heatmaps for significant GO process terms were generated as described in methods and Figs. 3 and 4. Any related child terms are presented in subsequent pages of the parent file name. GO terms with more than 100 children, with 2 or fewer genes annotated to the term, or a file size over 300KB are not shown. All heatmaps are generated with the same layout (see Figs. 3 and 4). [file 40170_2019_201_MOESM9_ESM.bz2 › Additional_File9_GOTermHeatmaps/Additional_File9_GOTermHeatmap/peptidyl-serine_phosphorylation.pdf]

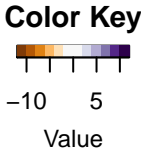

**pH reduction**

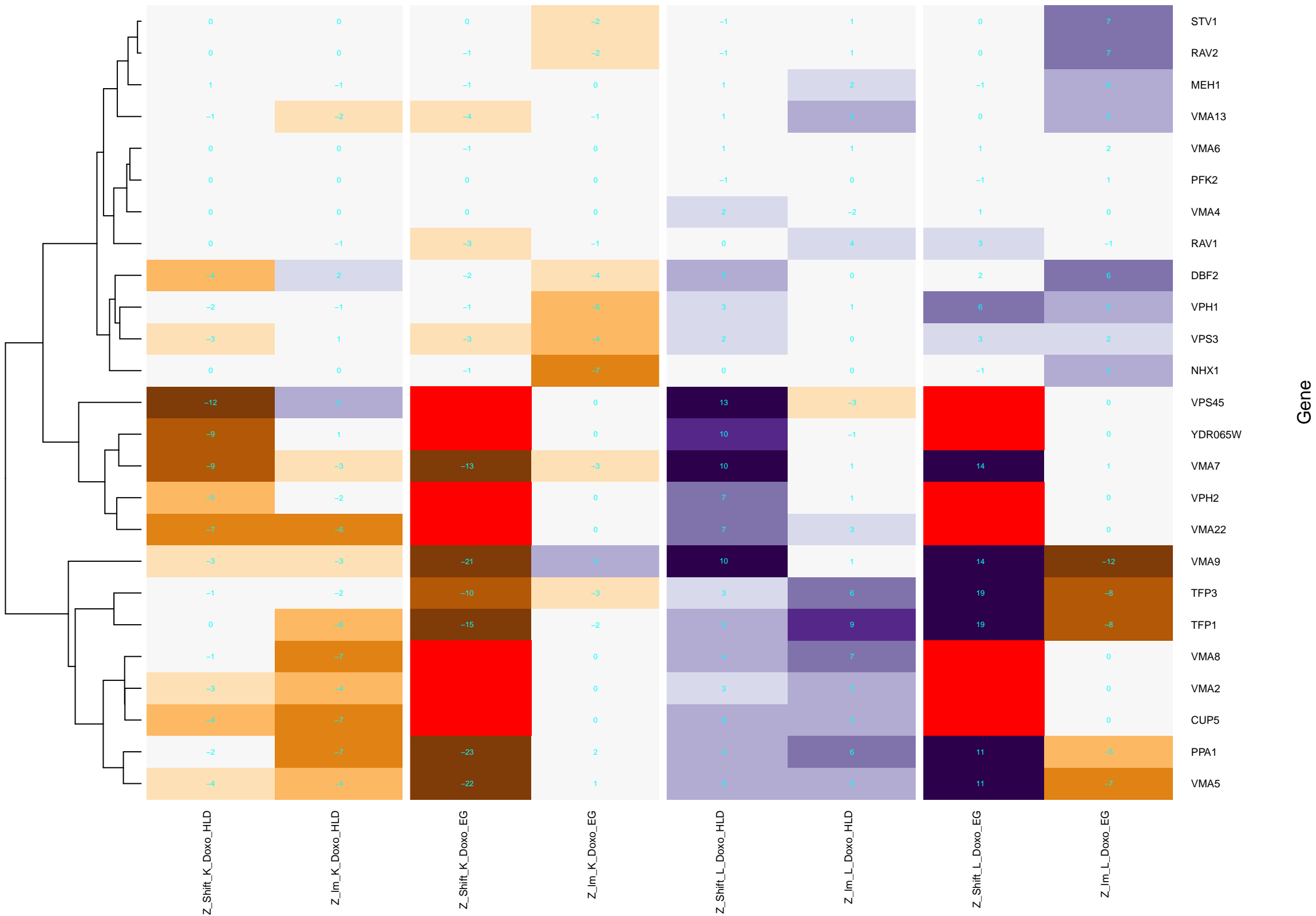

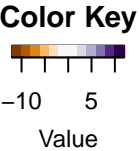

intracellular pH reduction

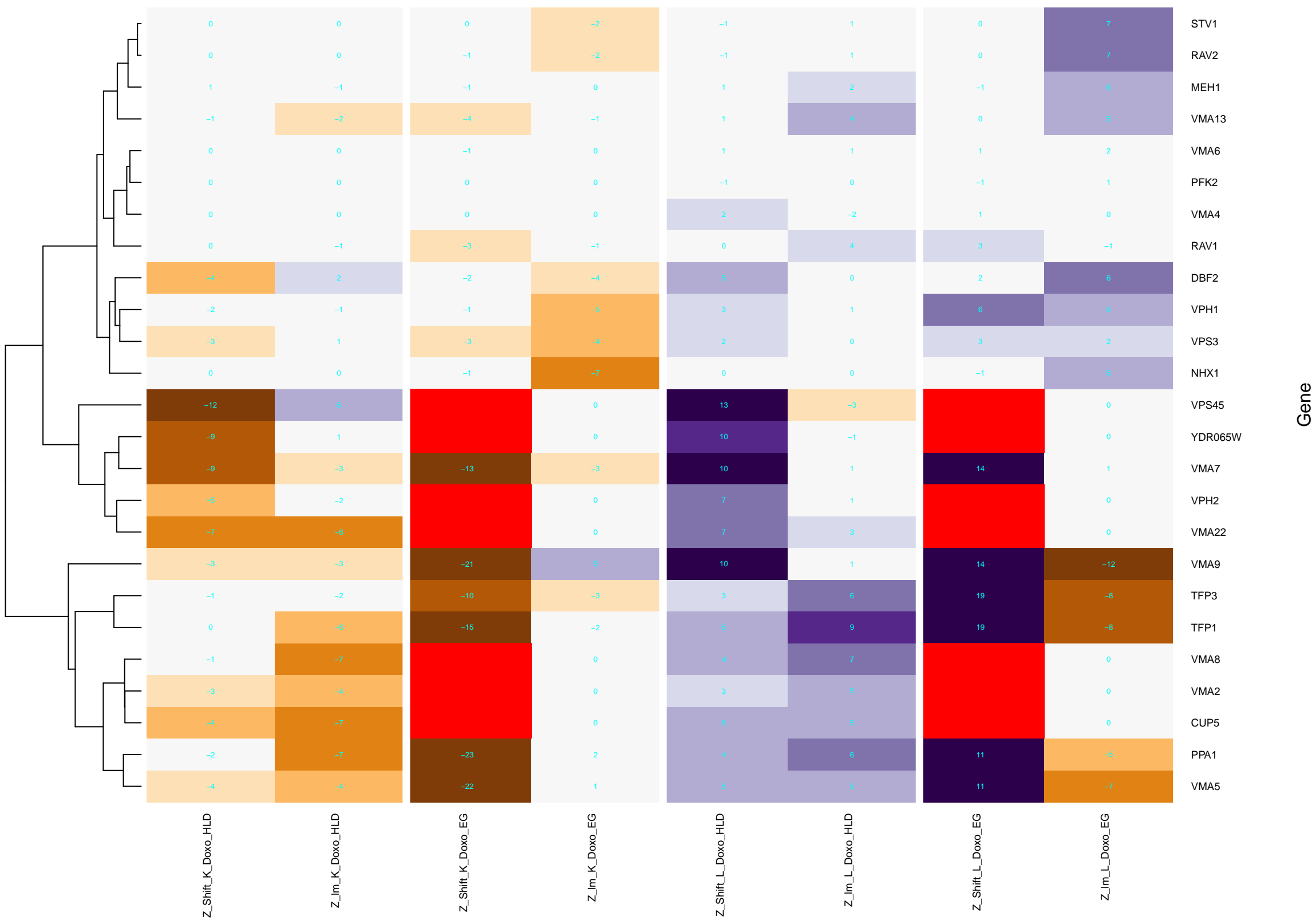

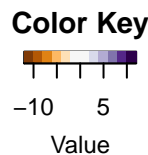

**vacuolar acidification**

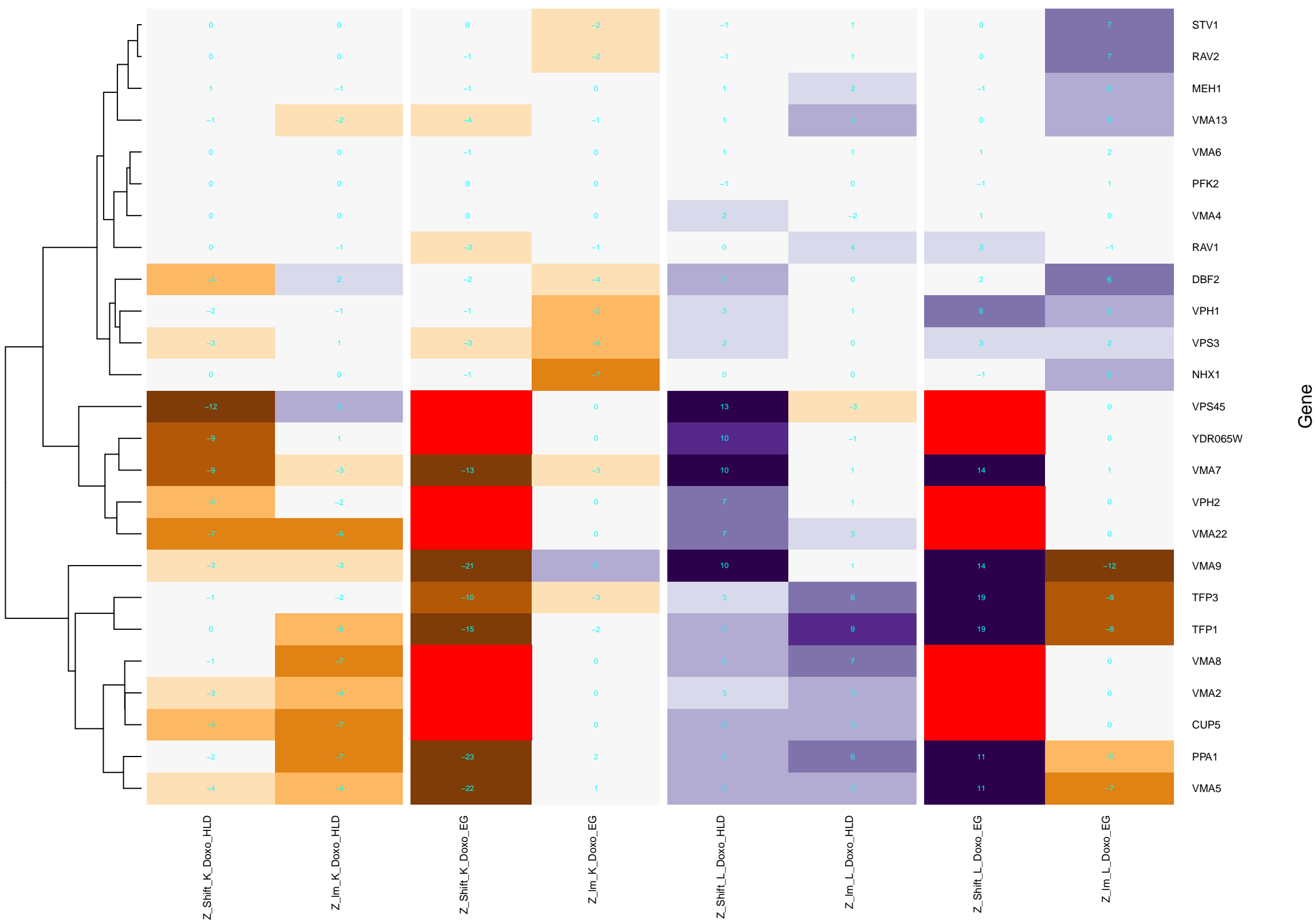

Supplement: Supplementary file 9 — Additional file 9. GO term-specific heatmaps for REMc/GTF-enriched clusters. GO term-specific heatmaps for significant GO process terms were generated as described in methods and Figs. 3 and 4. Any related child terms are presented in subsequent pages of the parent file name. GO terms with more than 100 children, with 2 or fewer genes annotated to the term, or a file size over 300KB are not shown. All heatmaps are generated with the same layout (see Figs. 3 and 4). [file 40170_2019_201_MOESM9_ESM.bz2 › Additional_File9_GOTermHeatmaps/Additional_File9_GOTermHeatmap/pH_reduction.pdf]

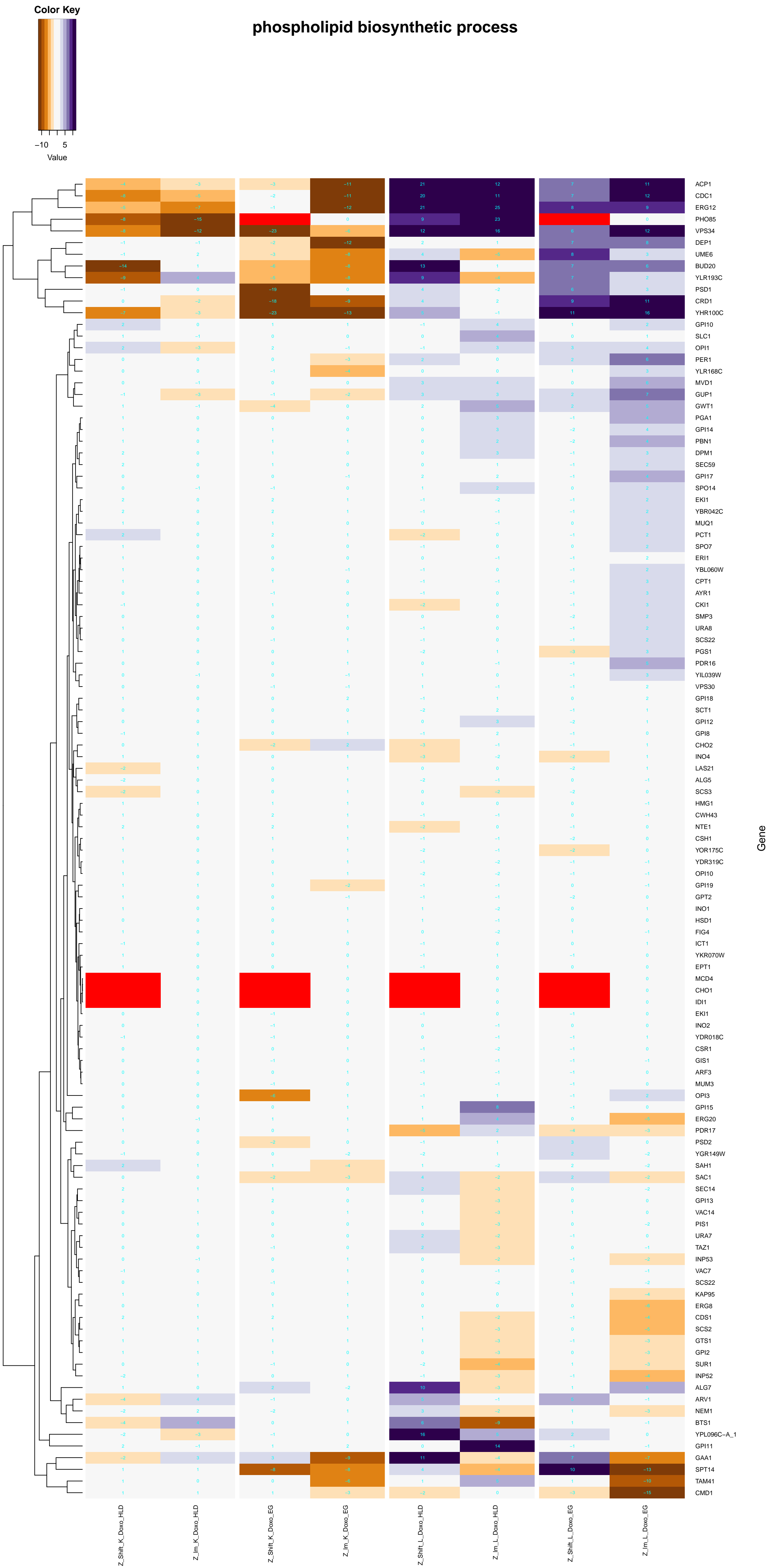

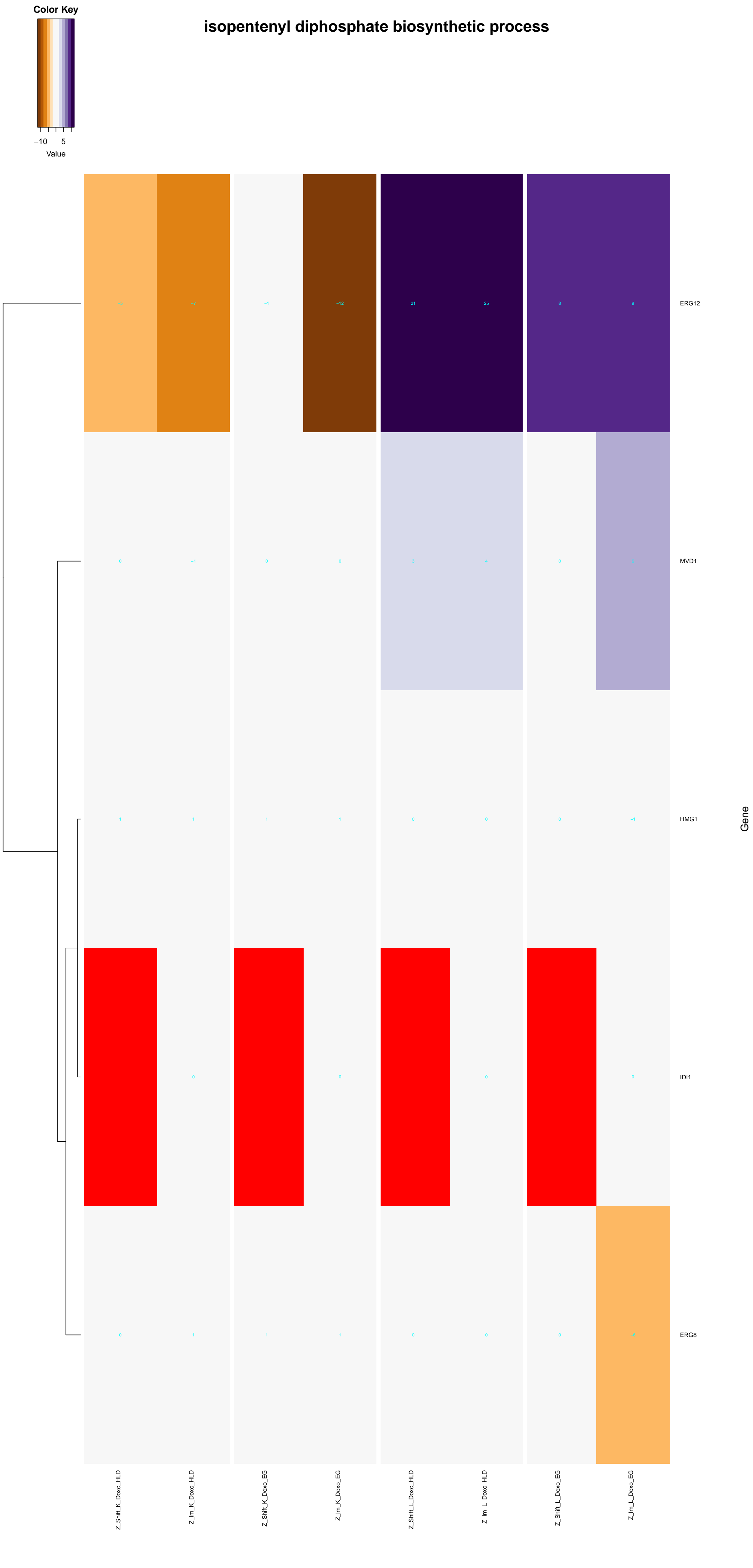

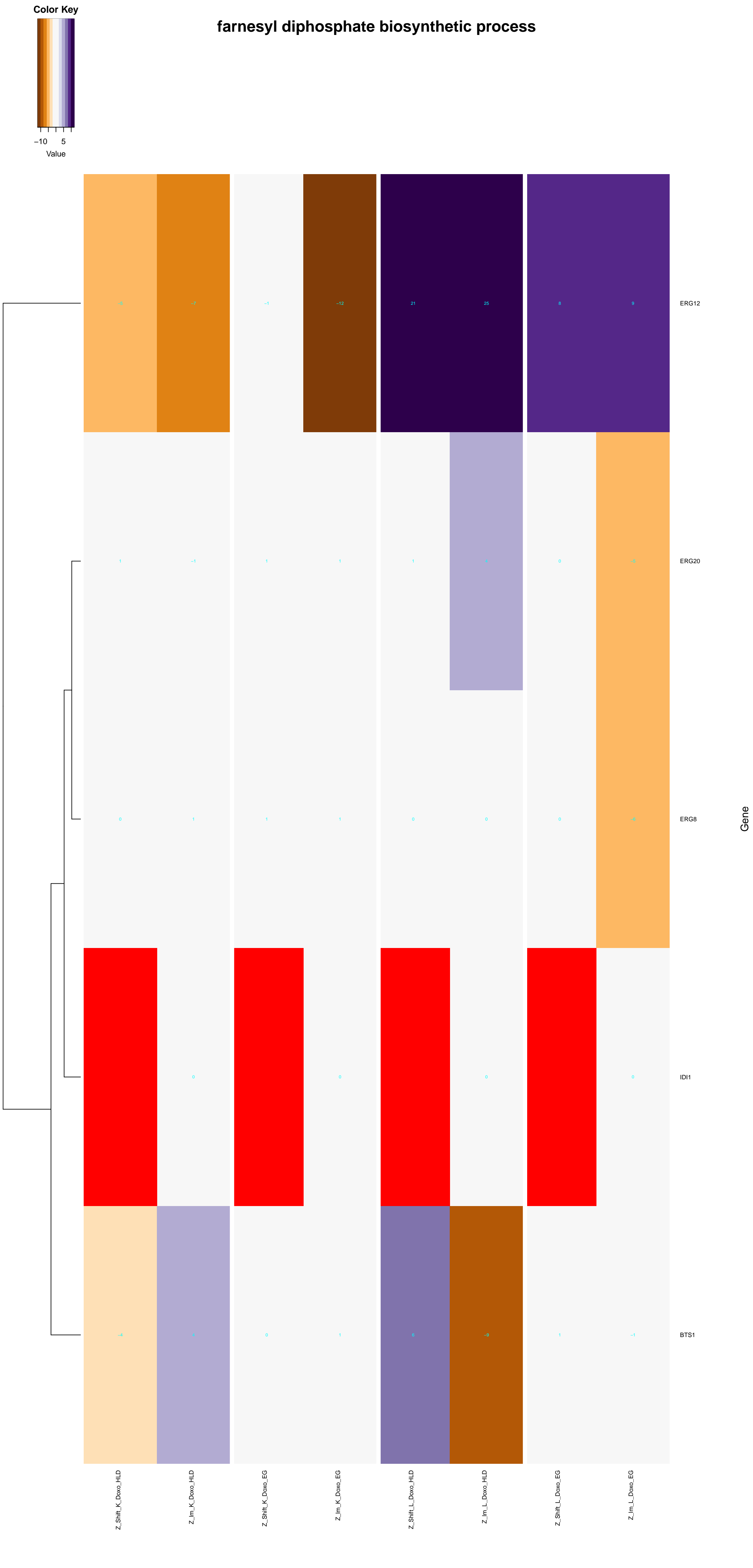

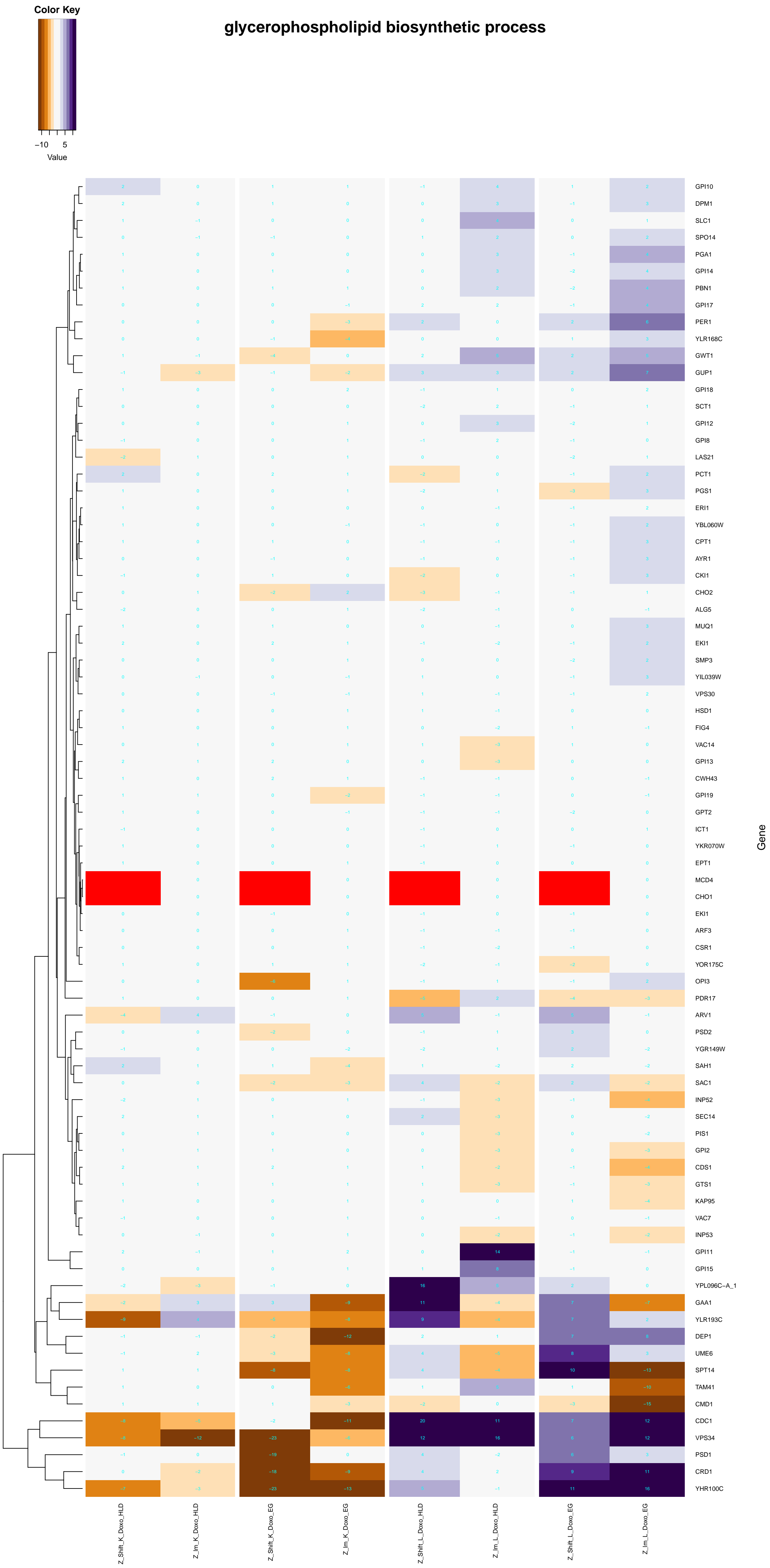

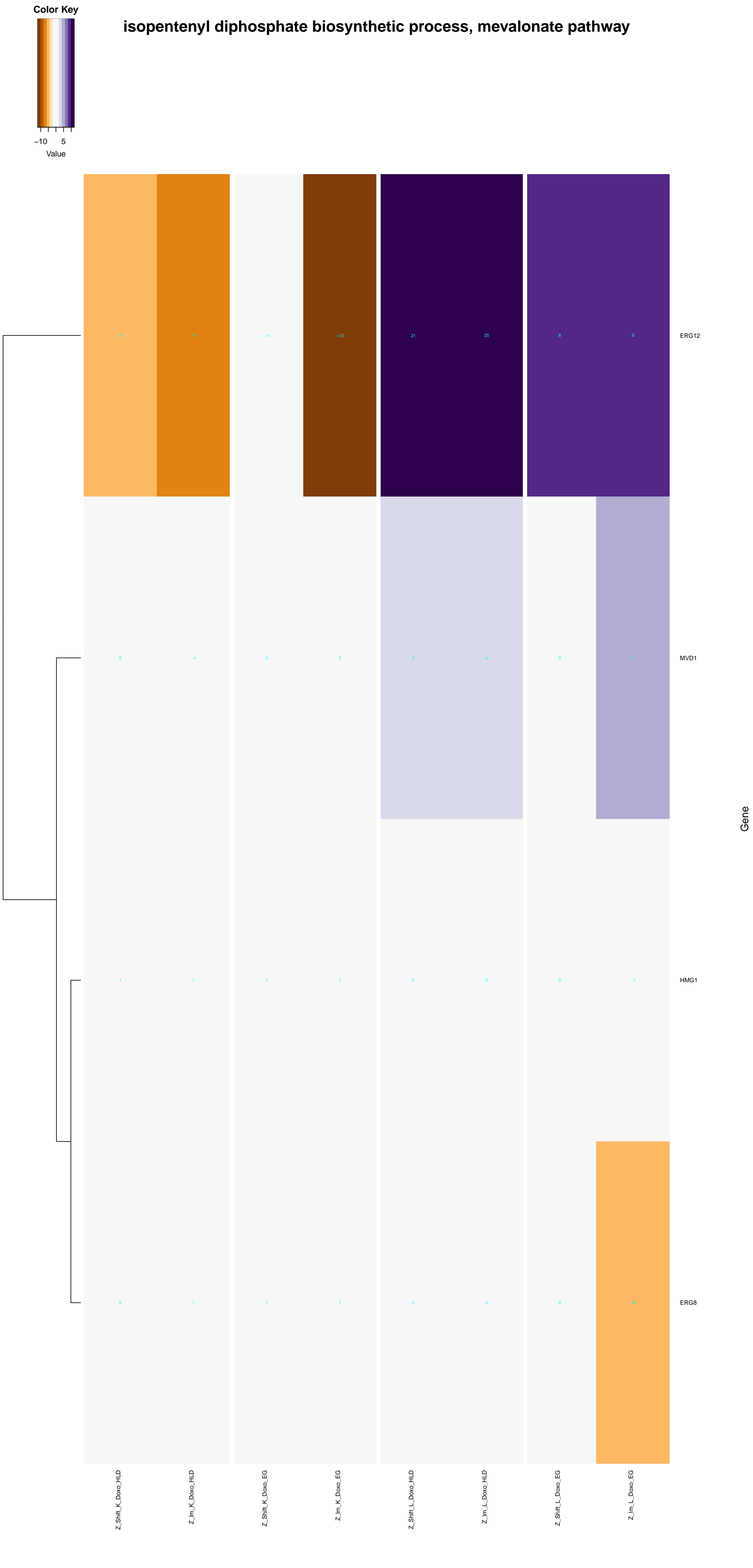

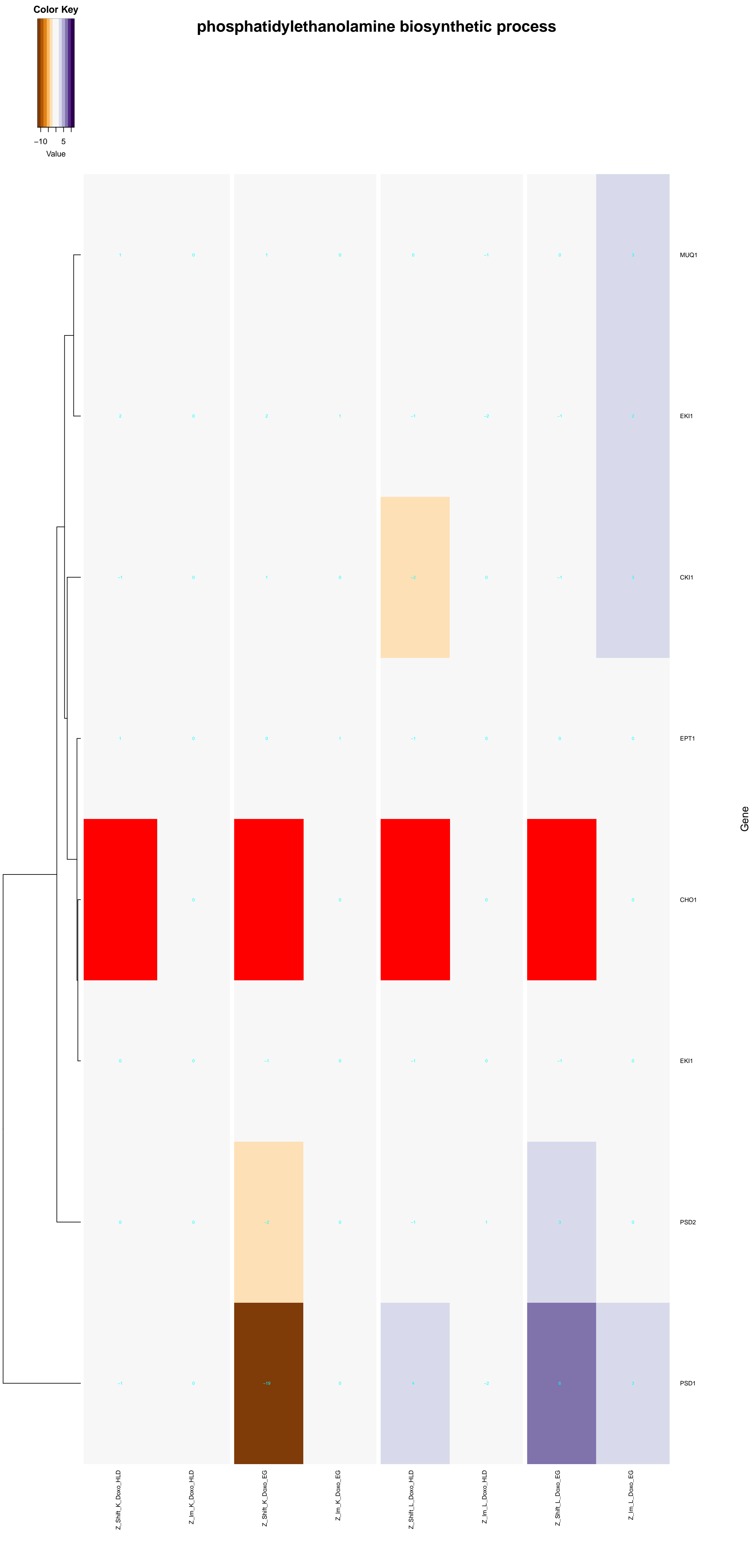

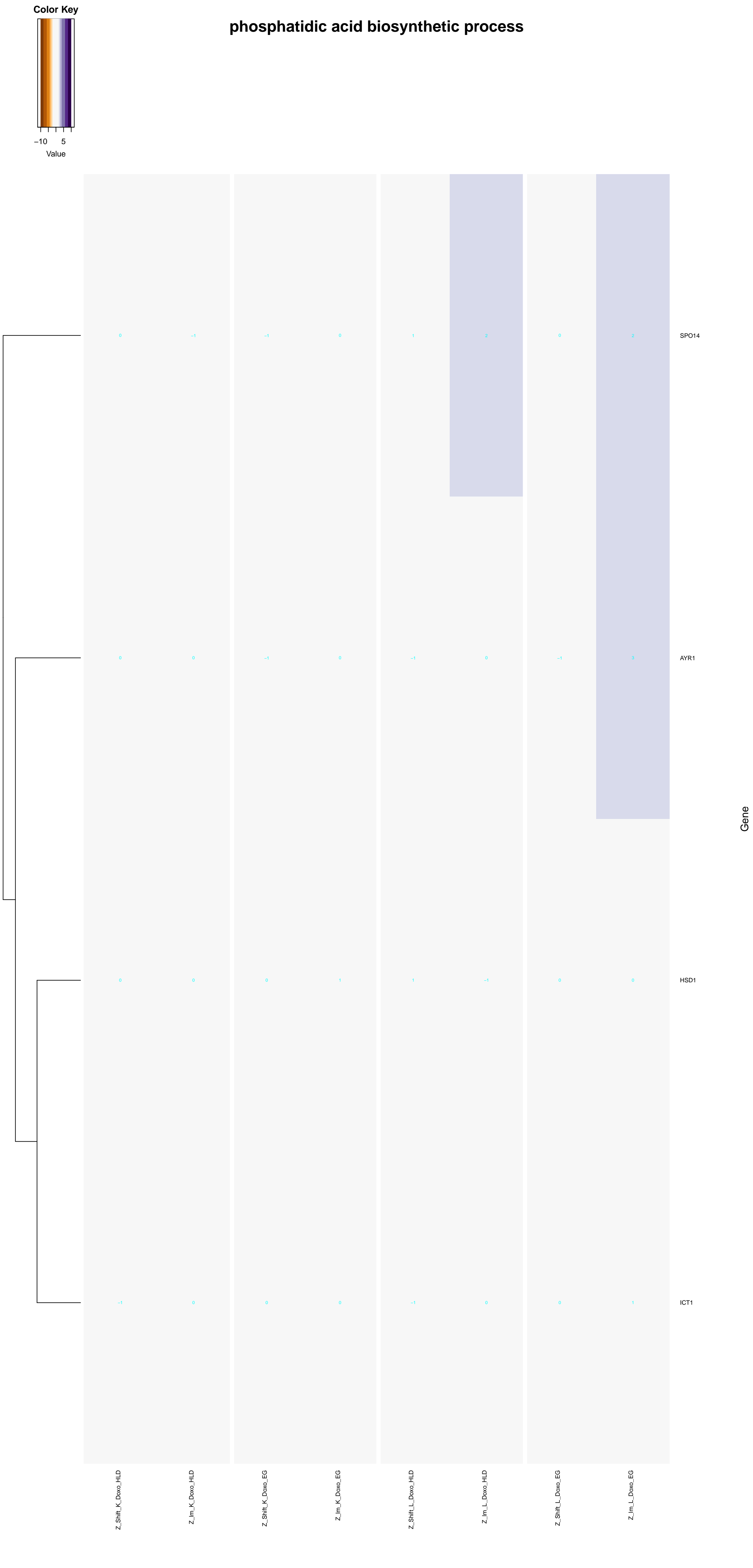

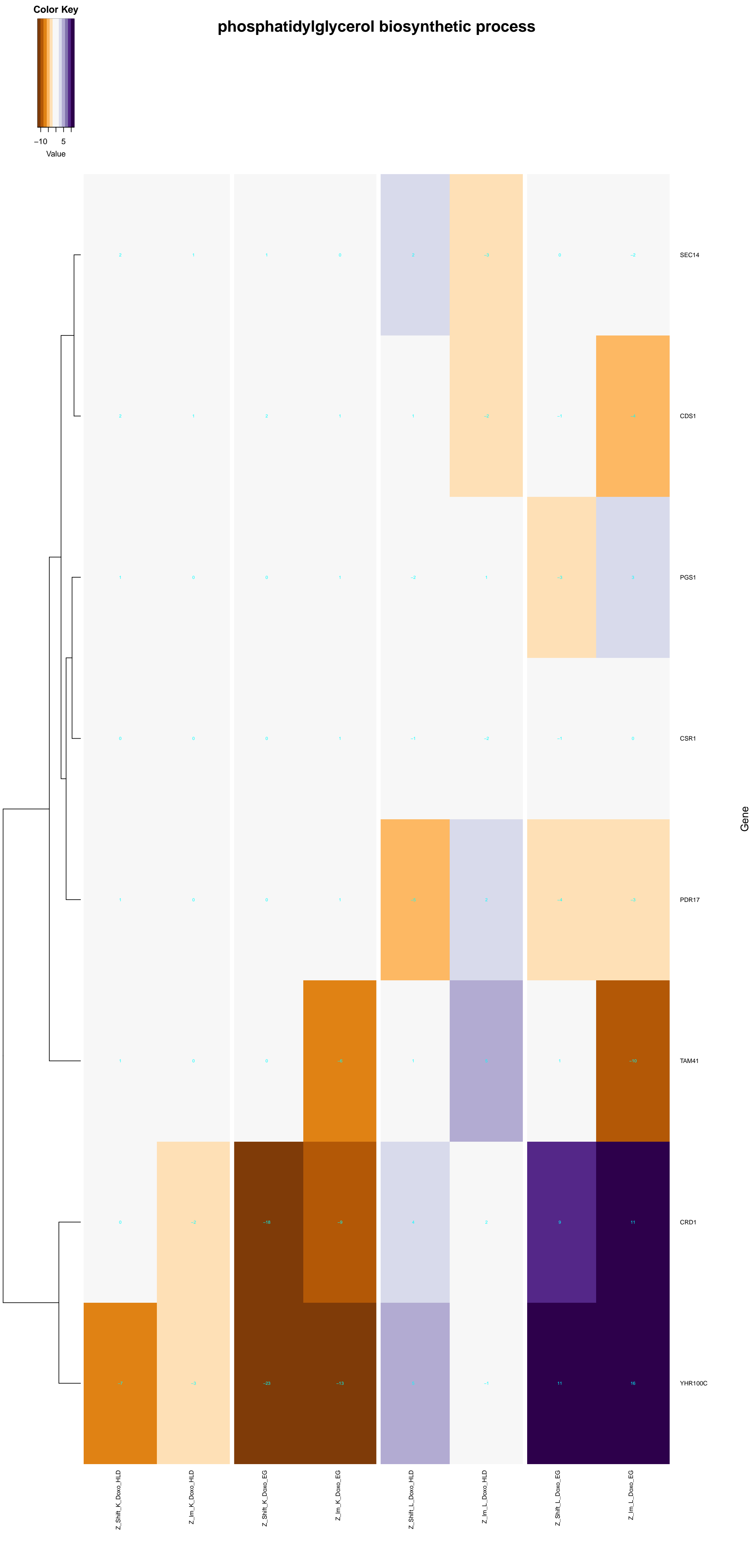

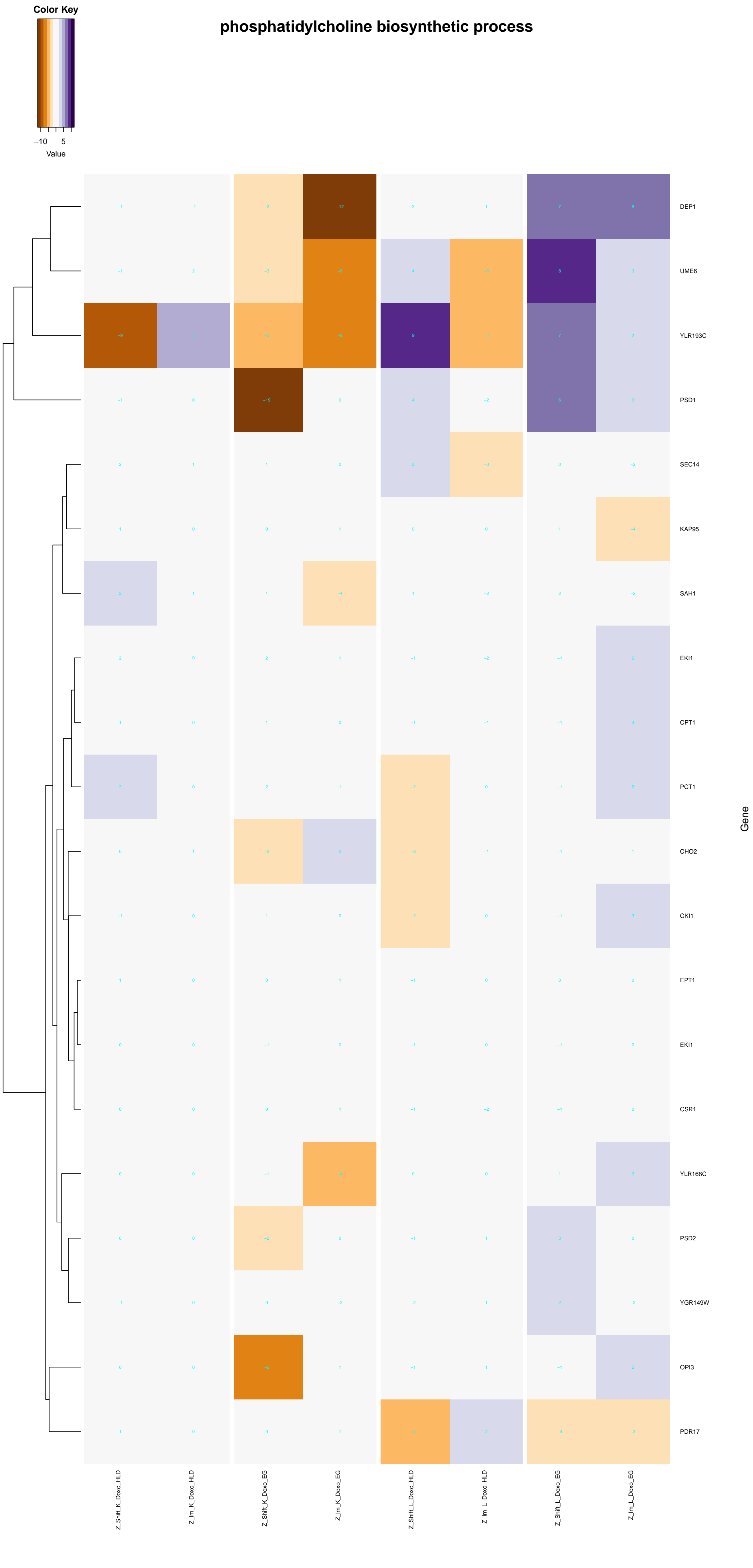

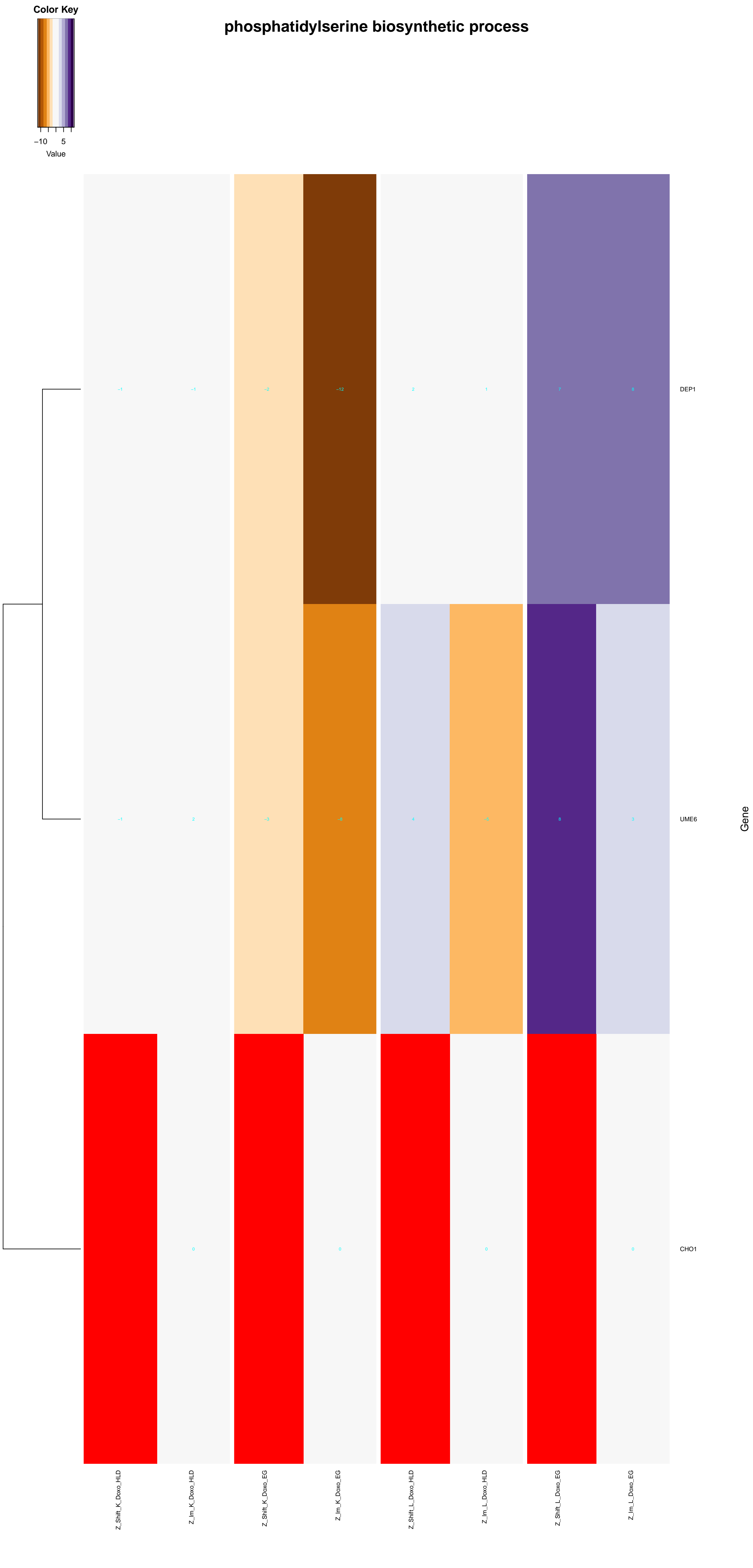

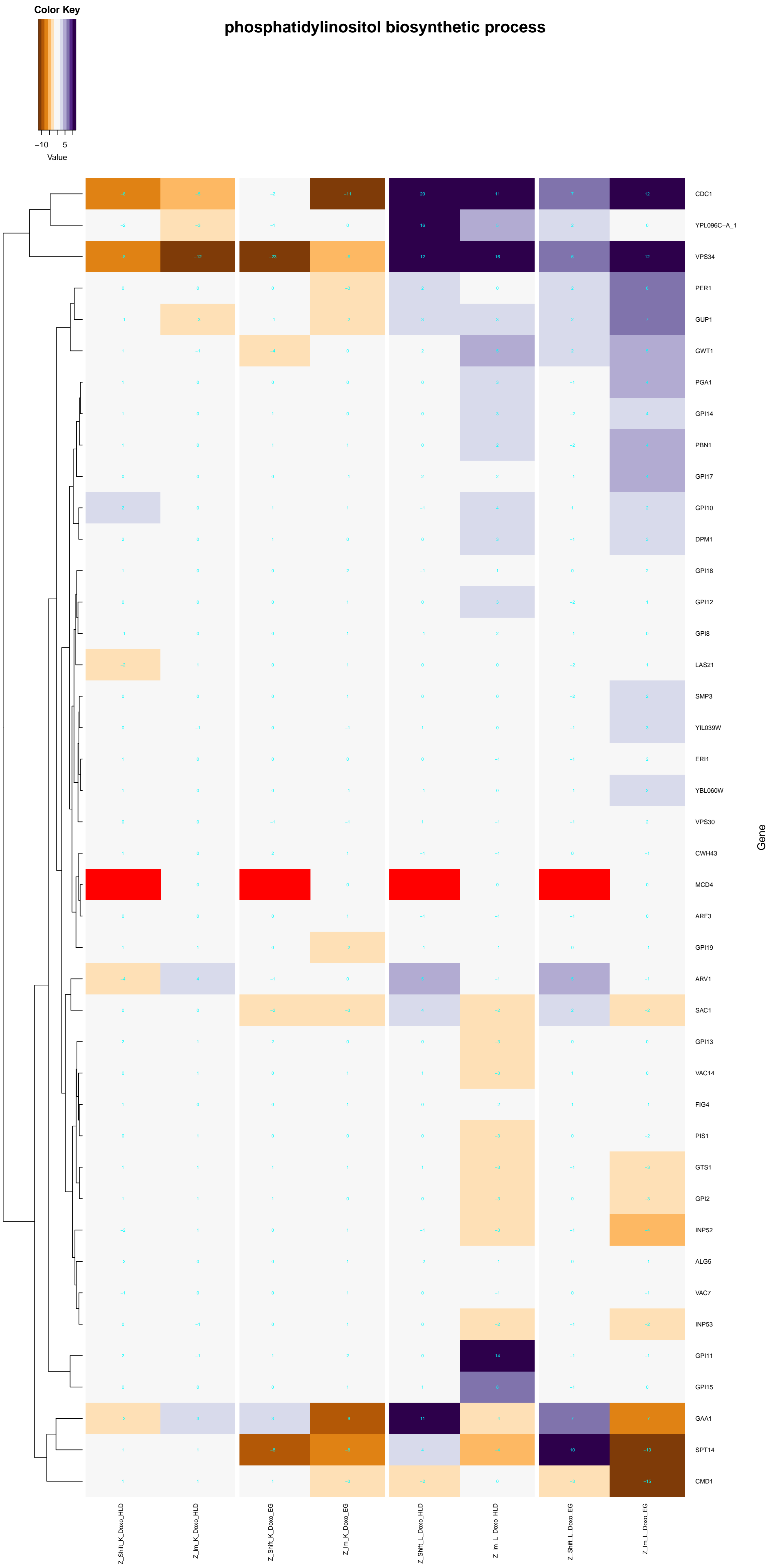

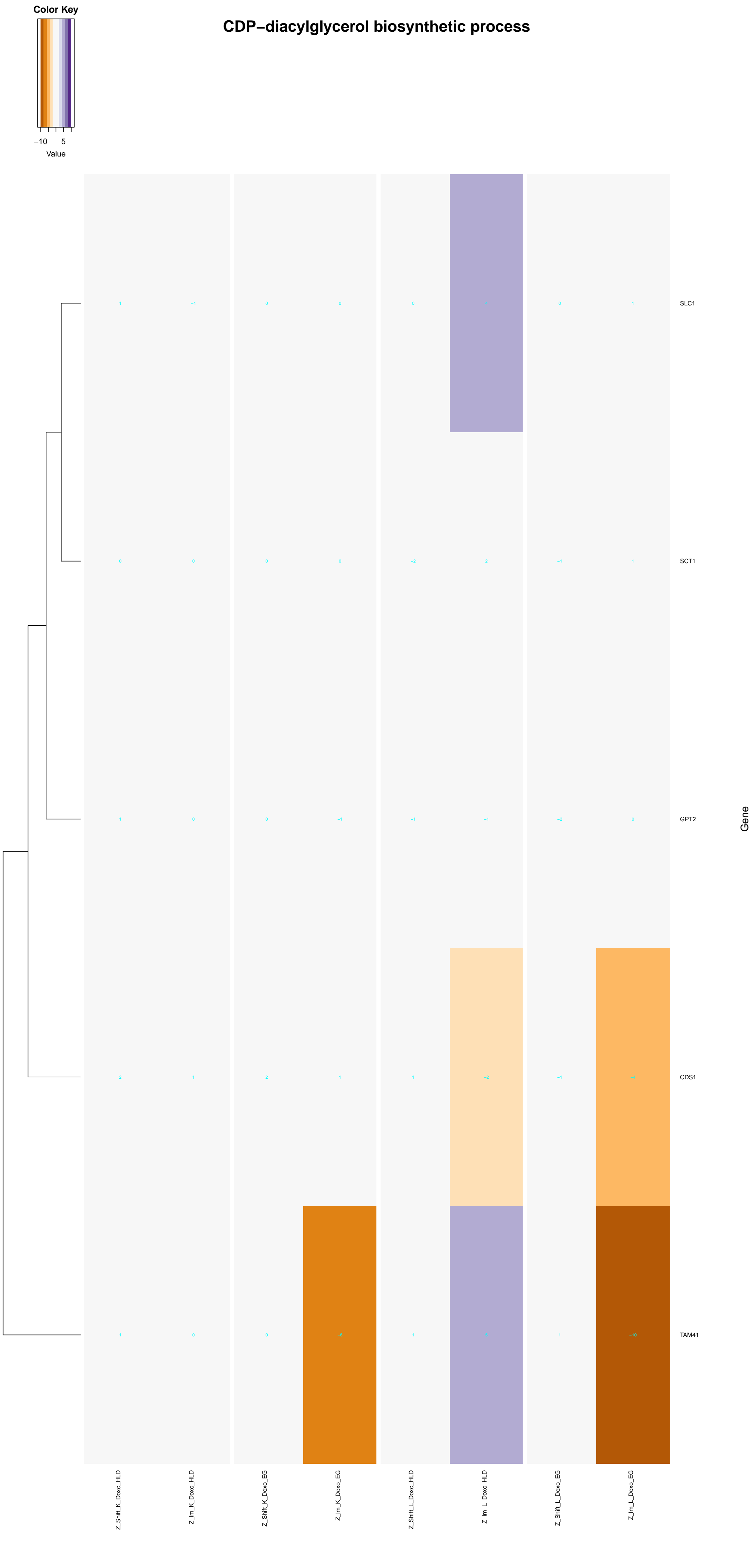

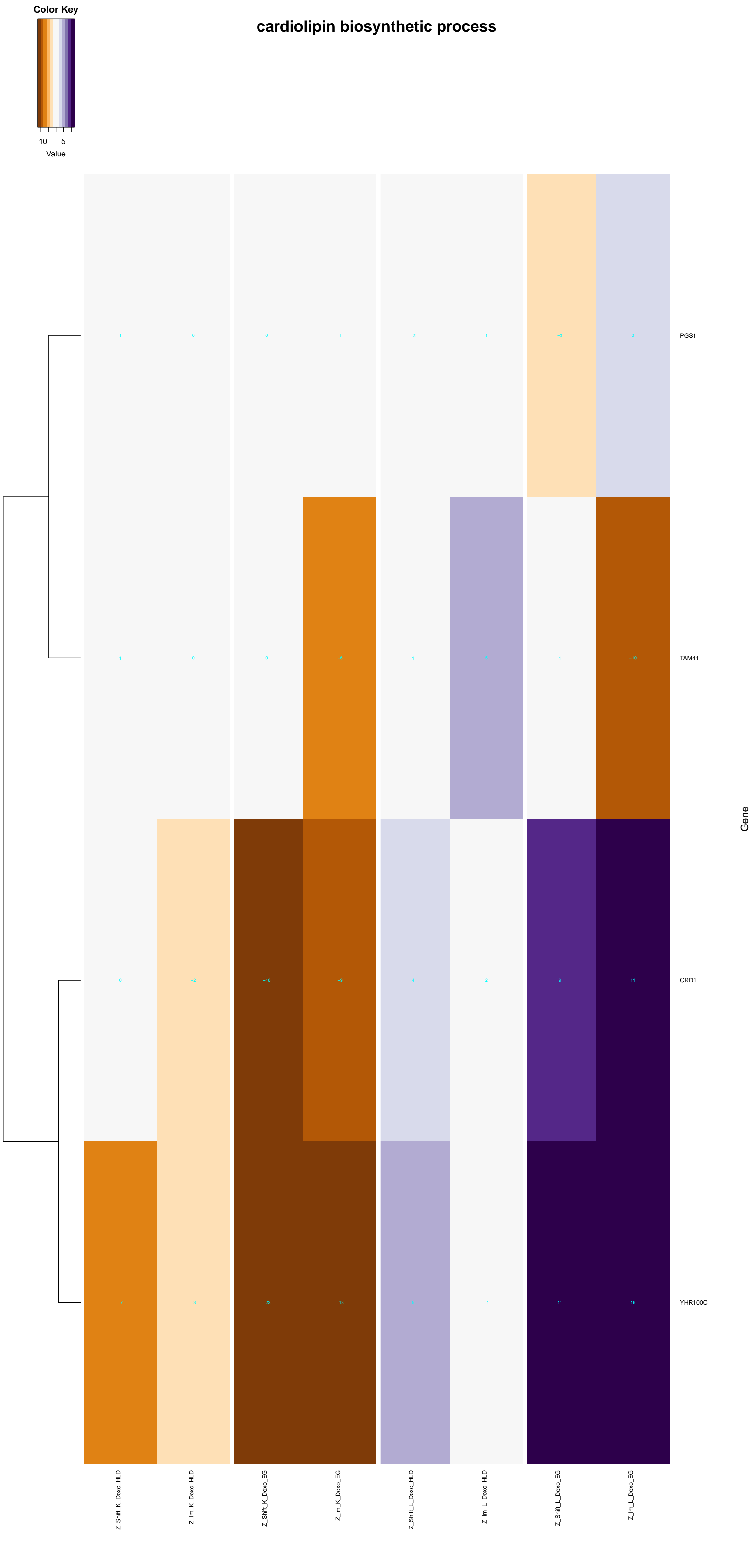

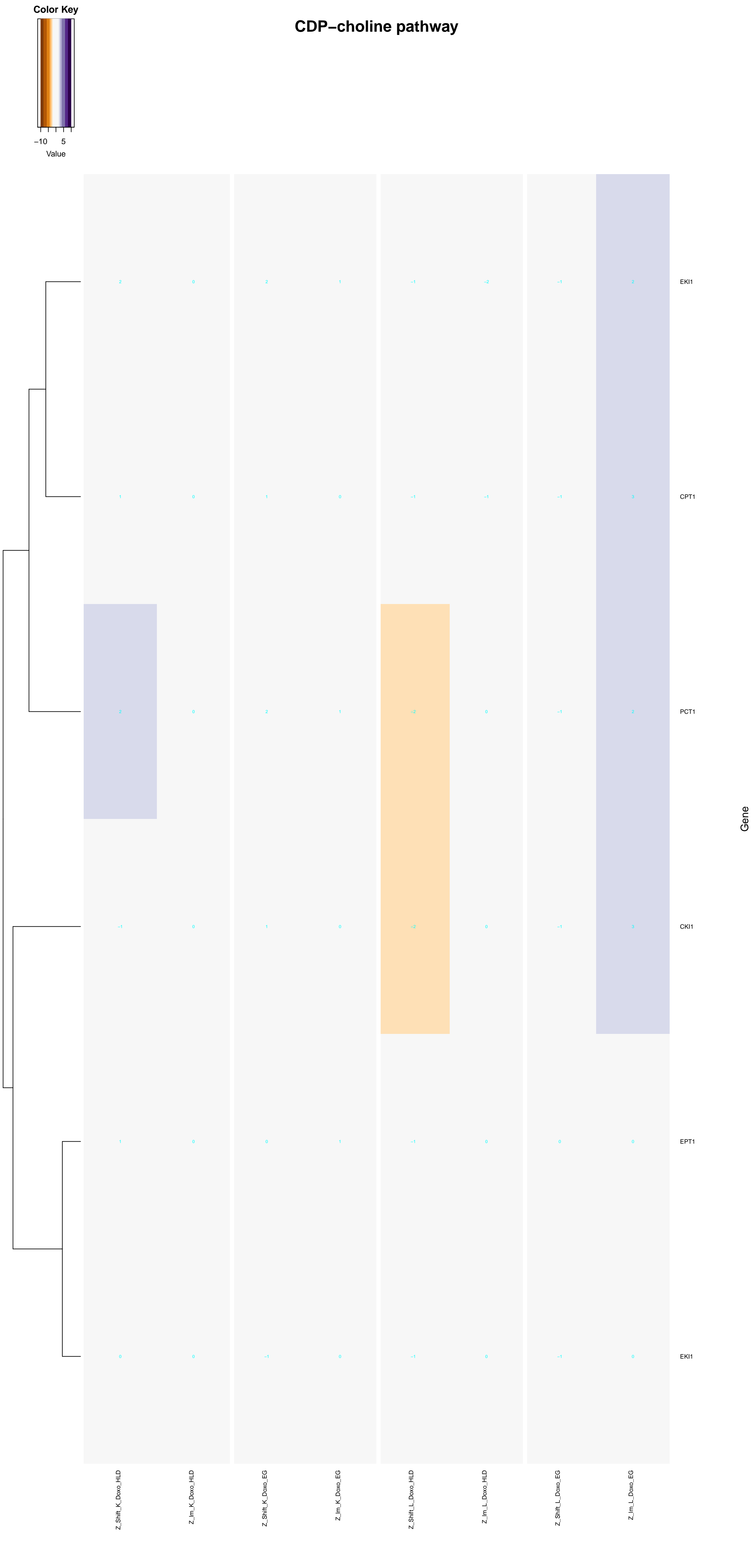

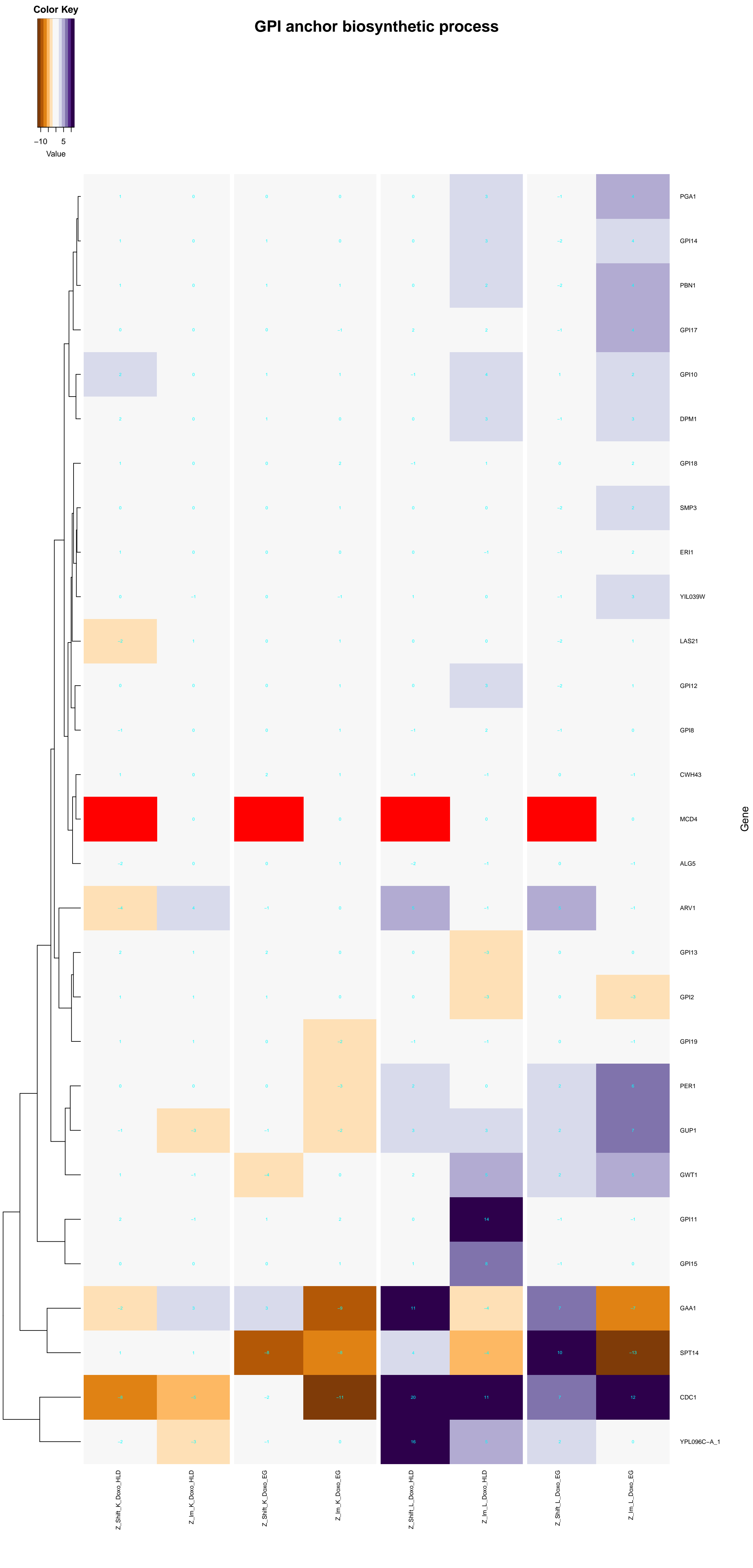

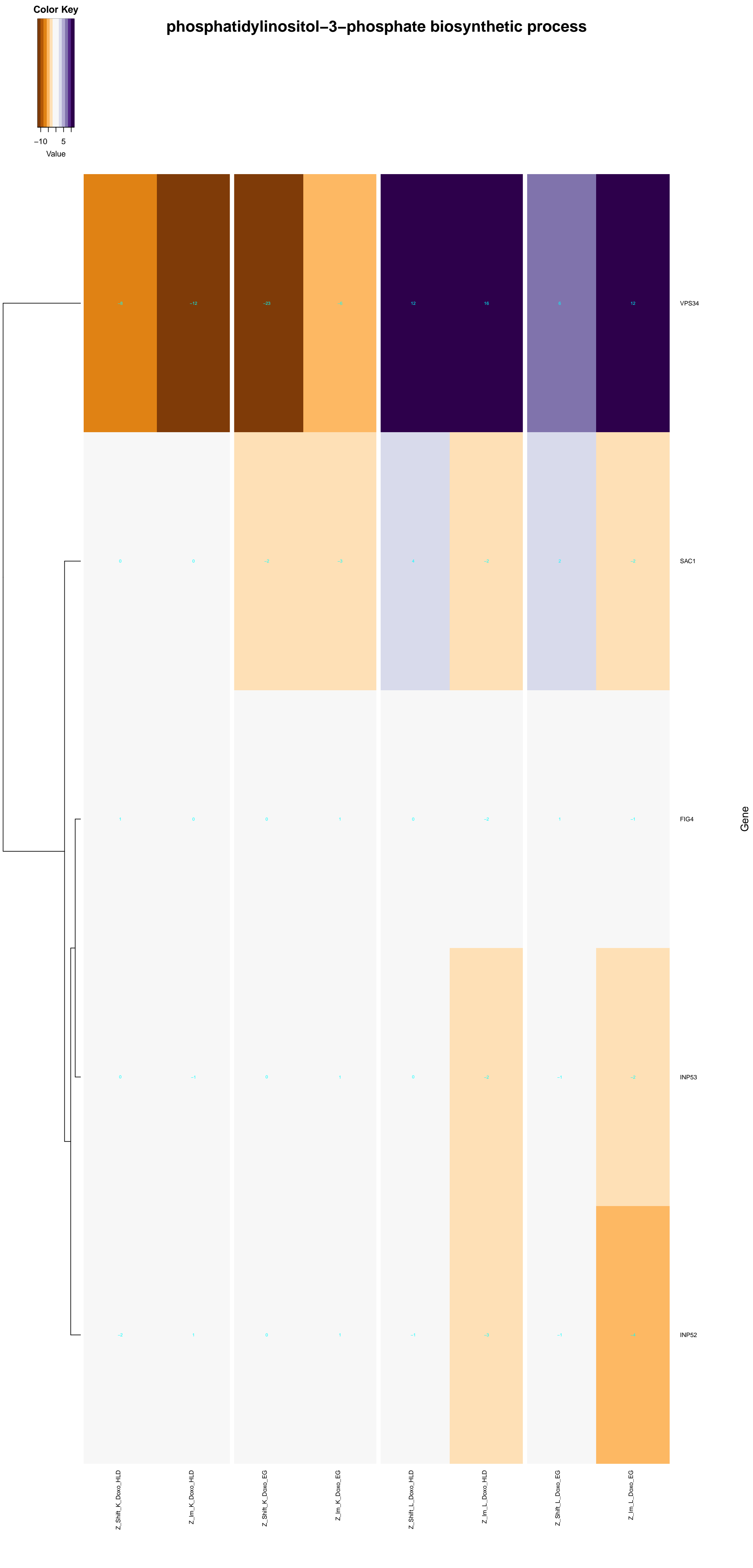

Supplement: Supplementary file 9 — Additional file 9. GO term-specific heatmaps for REMc/GTF-enriched clusters. GO term-specific heatmaps for significant GO process terms were generated as described in methods and Figs. 3 and 4. Any related child terms are presented in subsequent pages of the parent file name. GO terms with more than 100 children, with 2 or fewer genes annotated to the term, or a file size over 300KB are not shown. All heatmaps are generated with the same layout (see Figs. 3 and 4). [file 40170_2019_201_MOESM9_ESM.bz2 › Additional_File9_GOTermHeatmaps/Additional_File9_GOTermHeatmap/phospholipid_biosynthetic_process.pdf]

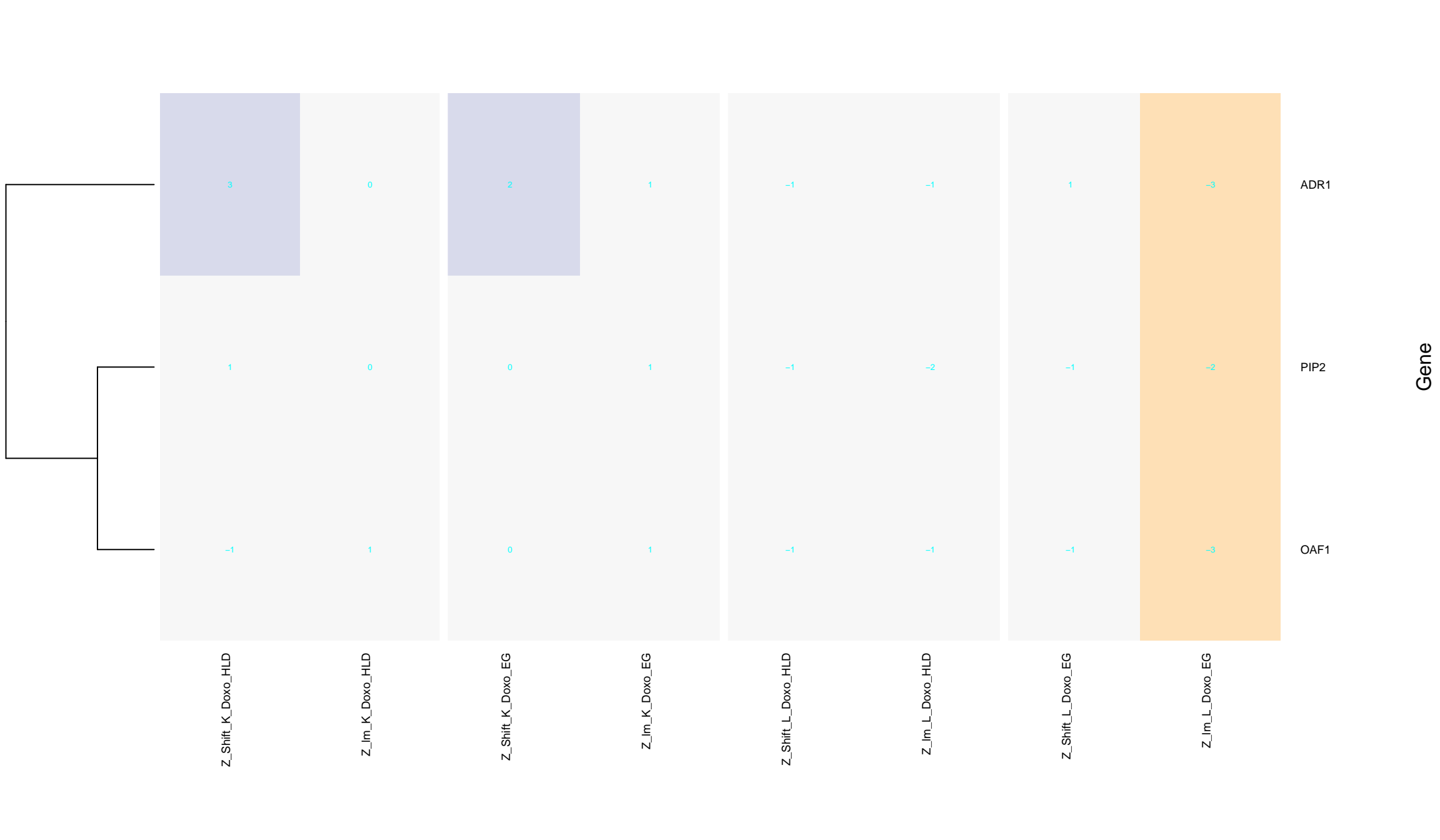

Supplement: Supplementary file 9 — Additional file 9. GO term-specific heatmaps for REMc/GTF-enriched clusters. GO term-specific heatmaps for significant GO process terms were generated as described in methods and Figs. 3 and 4. Any related child terms are presented in subsequent pages of the parent file name. GO terms with more than 100 children, with 2 or fewer genes annotated to the term, or a file size over 300KB are not shown. All heatmaps are generated with the same layout (see Figs. 3 and 4). [file 40170_2019_201_MOESM9_ESM.bz2 › Additional_File9_GOTermHeatmaps/Additional_File9_GOTermHeatmap/positive_regulation_of_fatty_acid_beta-oxidation.pdf]

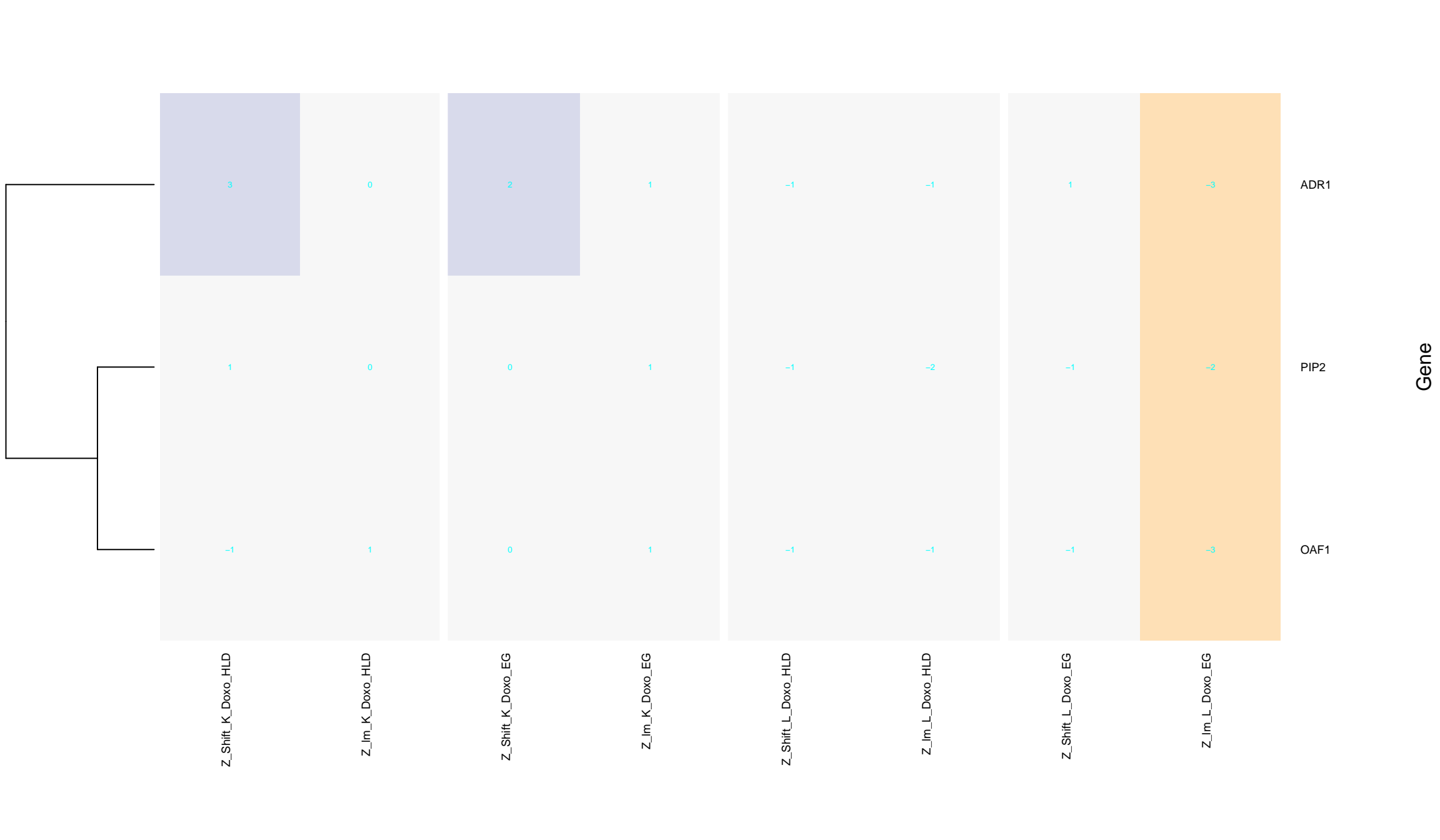

Supplement: Supplementary file 9 — Additional file 9. GO term-specific heatmaps for REMc/GTF-enriched clusters. GO term-specific heatmaps for significant GO process terms were generated as described in methods and Figs. 3 and 4. Any related child terms are presented in subsequent pages of the parent file name. GO terms with more than 100 children, with 2 or fewer genes annotated to the term, or a file size over 300KB are not shown. All heatmaps are generated with the same layout (see Figs. 3 and 4). [file 40170_2019_201_MOESM9_ESM.bz2 › Additional_File9_GOTermHeatmaps/Additional_File9_GOTermHeatmap/positive_regulation_of_fatty_acid_oxidation.pdf]

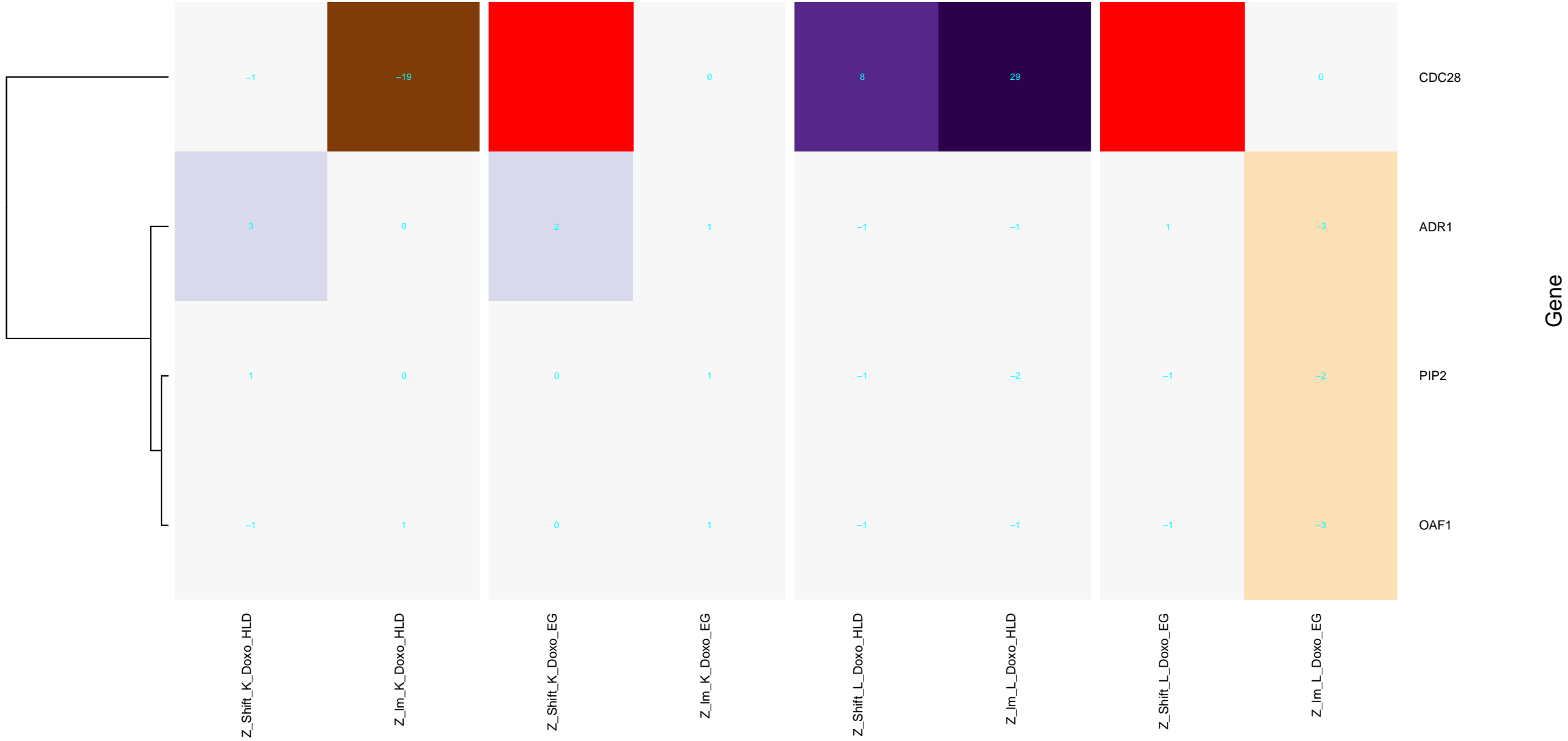

Supplement: Supplementary file 9 — Additional file 9. GO term-specific heatmaps for REMc/GTF-enriched clusters. GO term-specific heatmaps for significant GO process terms were generated as described in methods and Figs. 3 and 4. Any related child terms are presented in subsequent pages of the parent file name. GO terms with more than 100 children, with 2 or fewer genes annotated to the term, or a file size over 300KB are not shown. All heatmaps are generated with the same layout (see Figs. 3 and 4). [file 40170_2019_201_MOESM9_ESM.bz2 › Additional_File9_GOTermHeatmaps/Additional_File9_GOTermHeatmap/positive_regulation_of_lipid_catabolic_process.pdf]

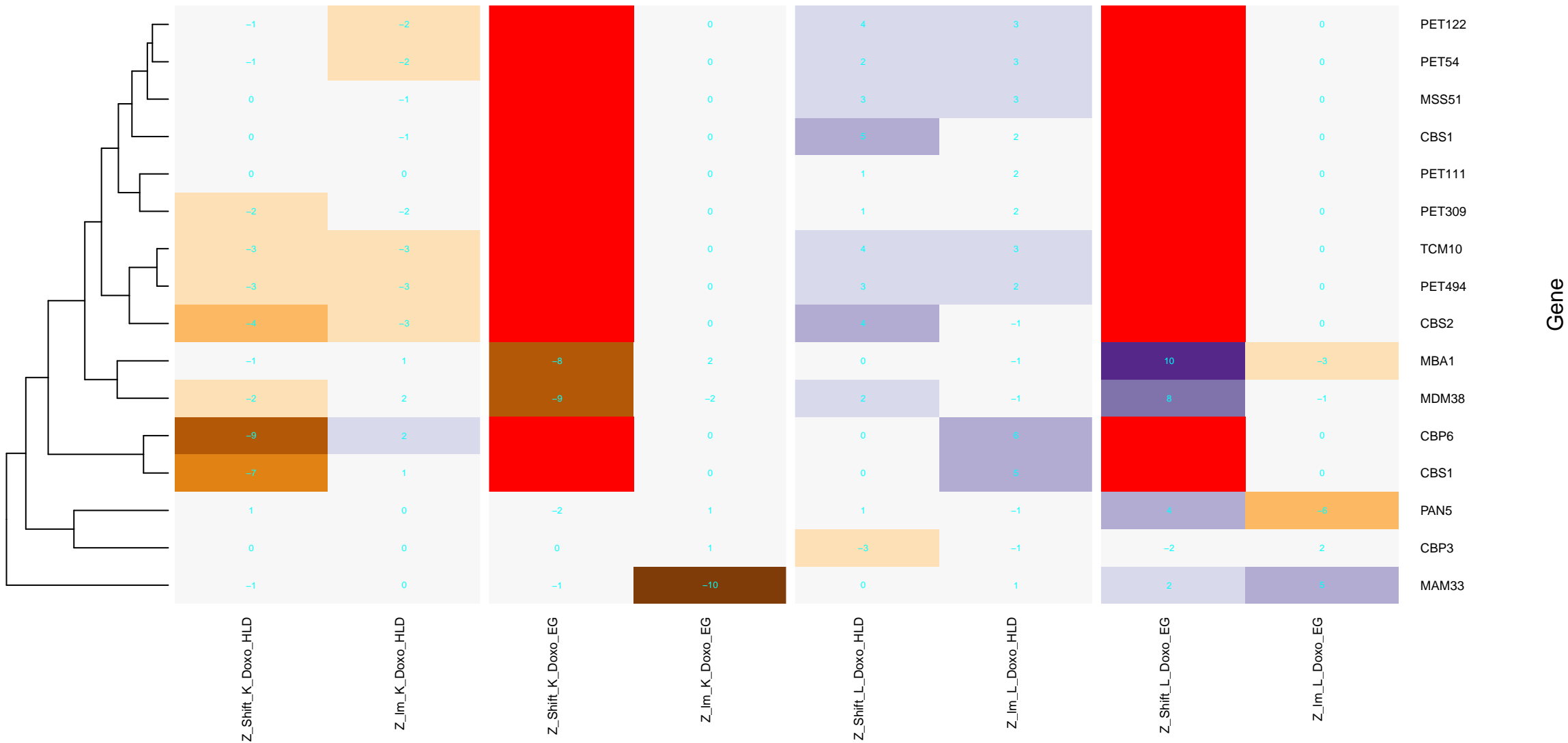

Supplement: Supplementary file 9 — Additional file 9. GO term-specific heatmaps for REMc/GTF-enriched clusters. GO term-specific heatmaps for significant GO process terms were generated as described in methods and Figs. 3 and 4. Any related child terms are presented in subsequent pages of the parent file name. GO terms with more than 100 children, with 2 or fewer genes annotated to the term, or a file size over 300KB are not shown. All heatmaps are generated with the same layout (see Figs. 3 and 4). [file 40170_2019_201_MOESM9_ESM.bz2 › Additional_File9_GOTermHeatmaps/Additional_File9_GOTermHeatmap/positive_regulation_of_mitochondrial_translation.pdf]

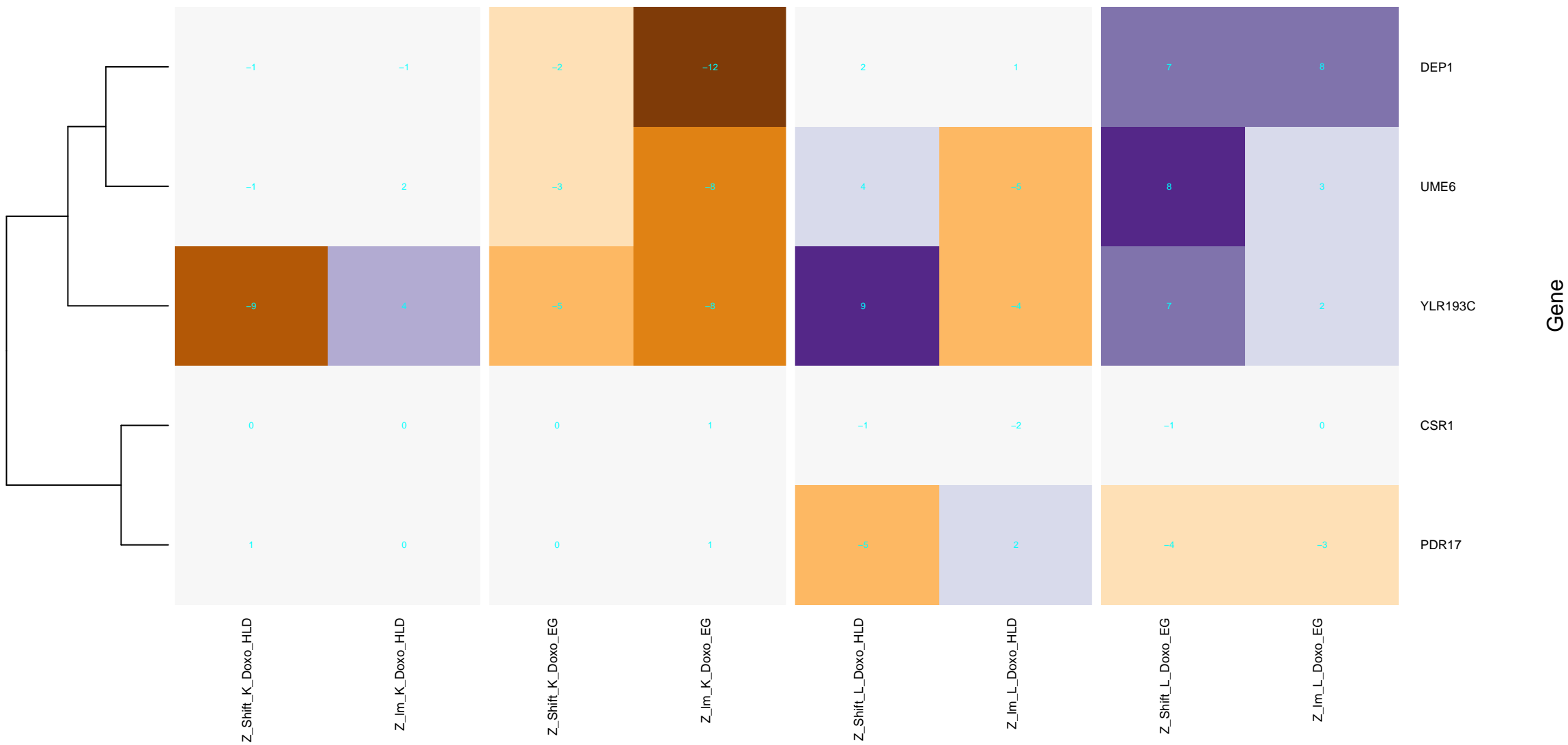

Supplement: Supplementary file 9 — Additional file 9. GO term-specific heatmaps for REMc/GTF-enriched clusters. GO term-specific heatmaps for significant GO process terms were generated as described in methods and Figs. 3 and 4. Any related child terms are presented in subsequent pages of the parent file name. GO terms with more than 100 children, with 2 or fewer genes annotated to the term, or a file size over 300KB are not shown. All heatmaps are generated with the same layout (see Figs. 3 and 4). [file 40170_2019_201_MOESM9_ESM.bz2 › Additional_File9_GOTermHeatmaps/Additional_File9_GOTermHeatmap/positive_regulation_of_phosphatidylcholine_biosynthetic_process.pdf]

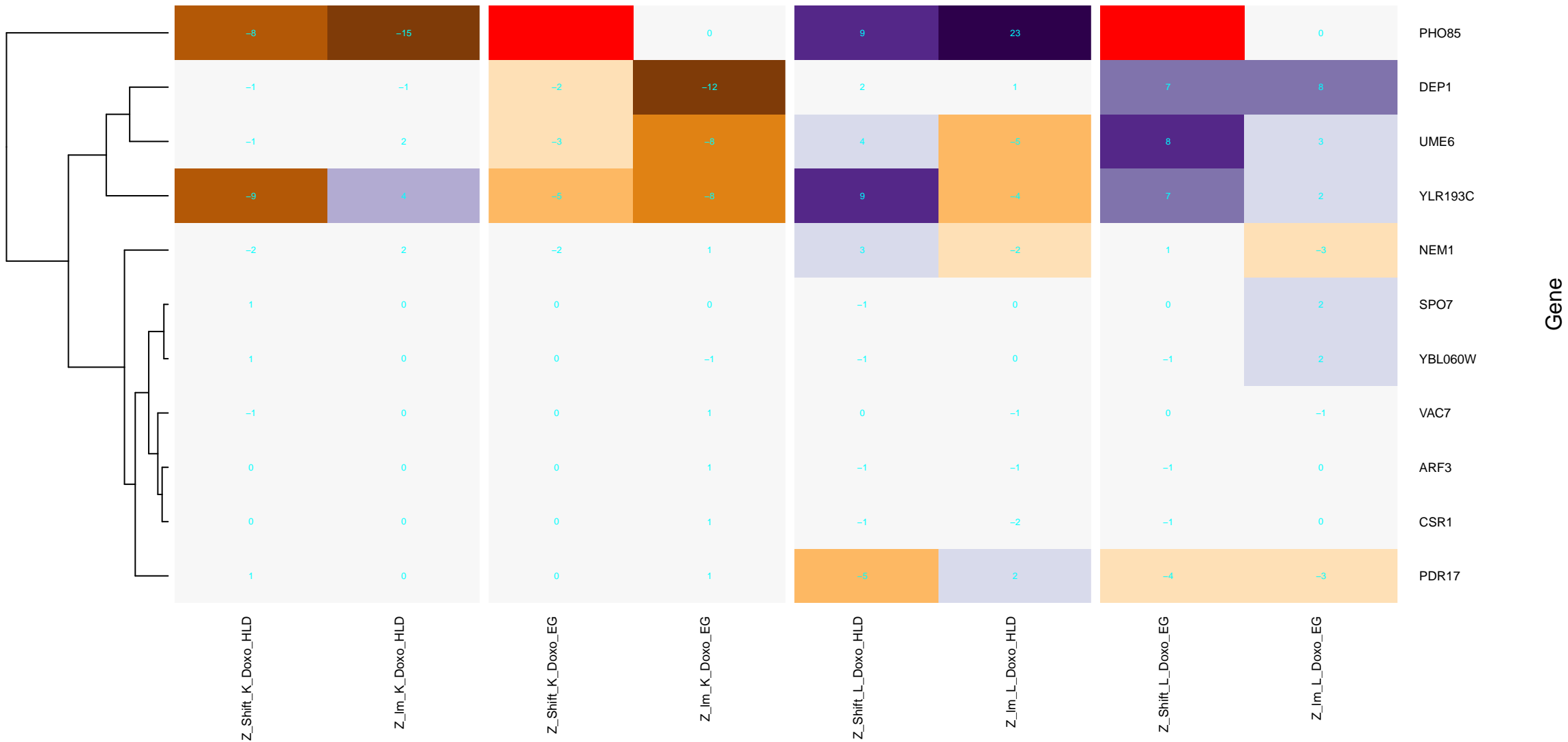

Supplement: Supplementary file 9 — Additional file 9. GO term-specific heatmaps for REMc/GTF-enriched clusters. GO term-specific heatmaps for significant GO process terms were generated as described in methods and Figs. 3 and 4. Any related child terms are presented in subsequent pages of the parent file name. GO terms with more than 100 children, with 2 or fewer genes annotated to the term, or a file size over 300KB are not shown. All heatmaps are generated with the same layout (see Figs. 3 and 4). [file 40170_2019_201_MOESM9_ESM.bz2 › Additional_File9_GOTermHeatmaps/Additional_File9_GOTermHeatmap/positive_regulation_of_phospholipid_biosynthetic_process.pdf]

UES GDSC1000 vs UES gCSI

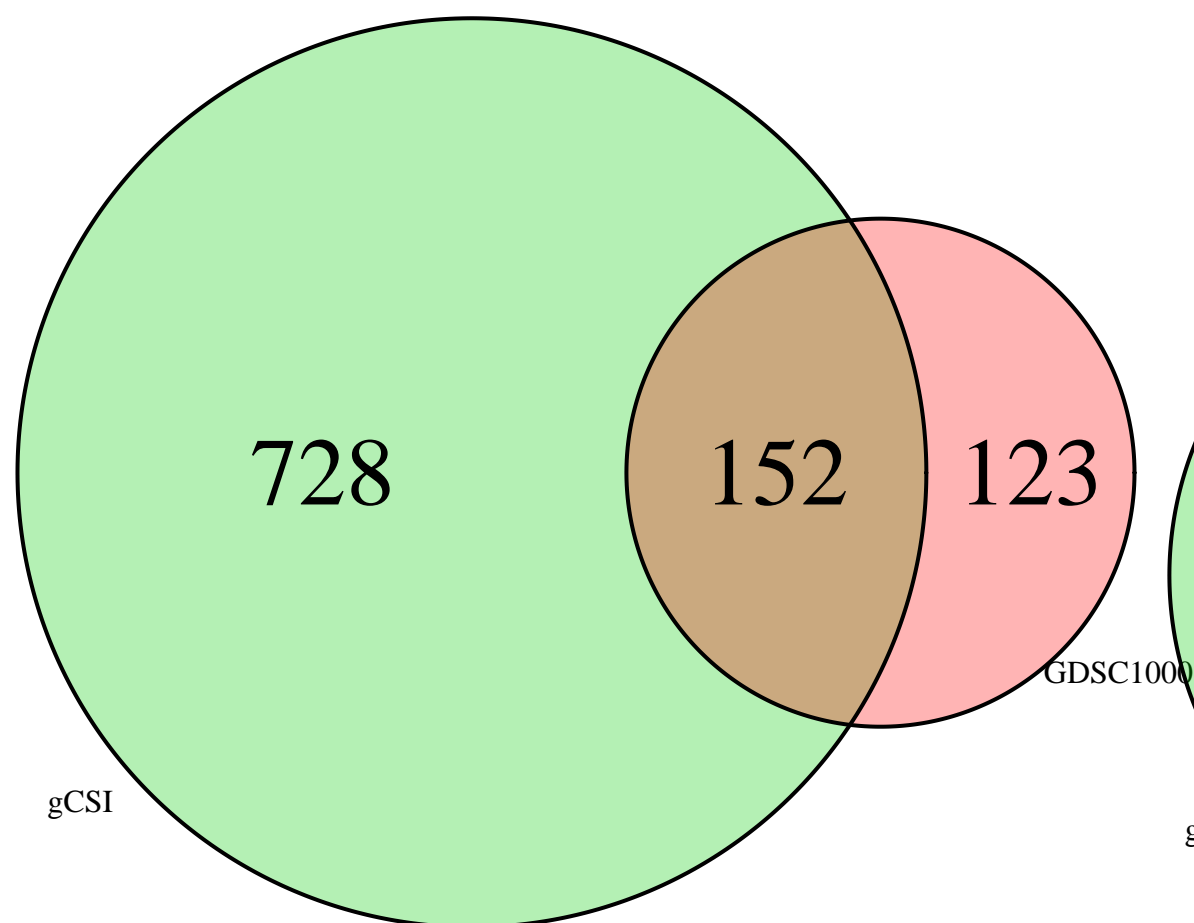

UES GDSC1000 vs OES gCSI

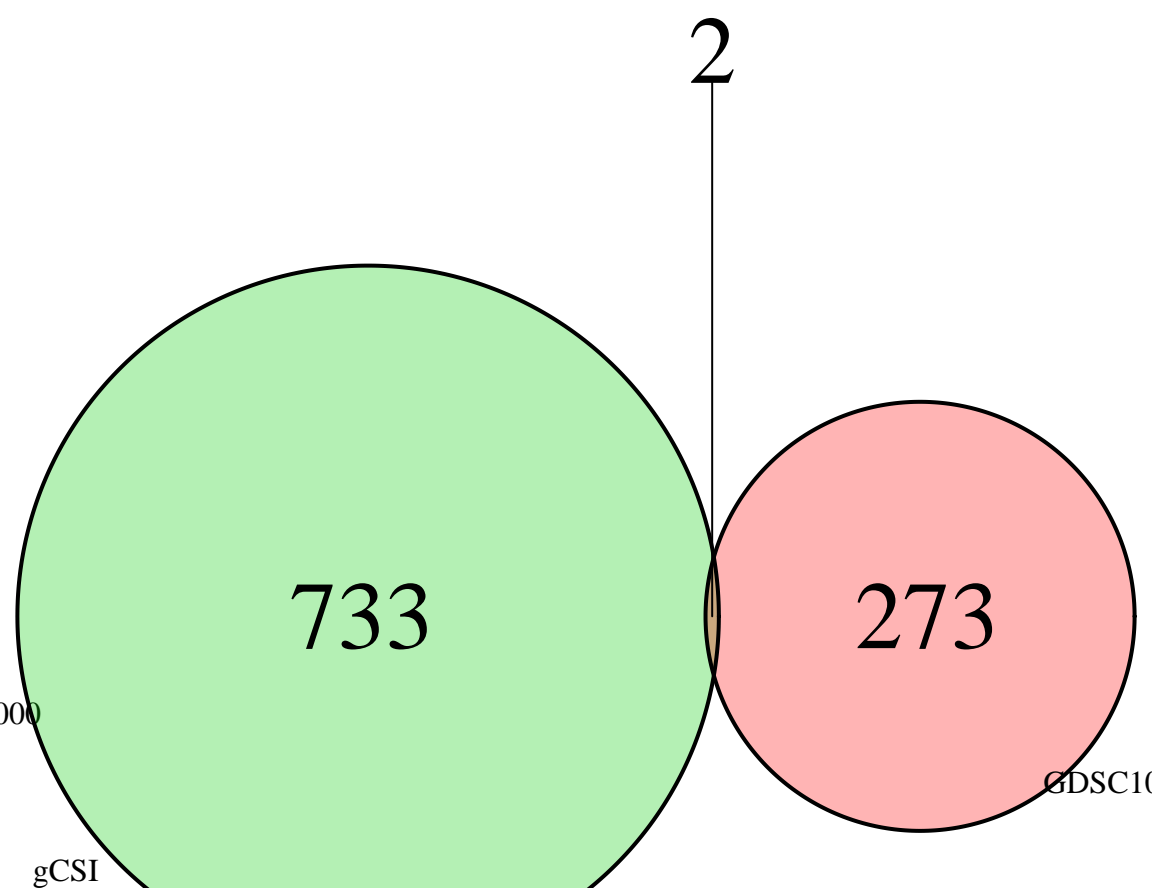

OES GDSC1000 vs OES gCSI

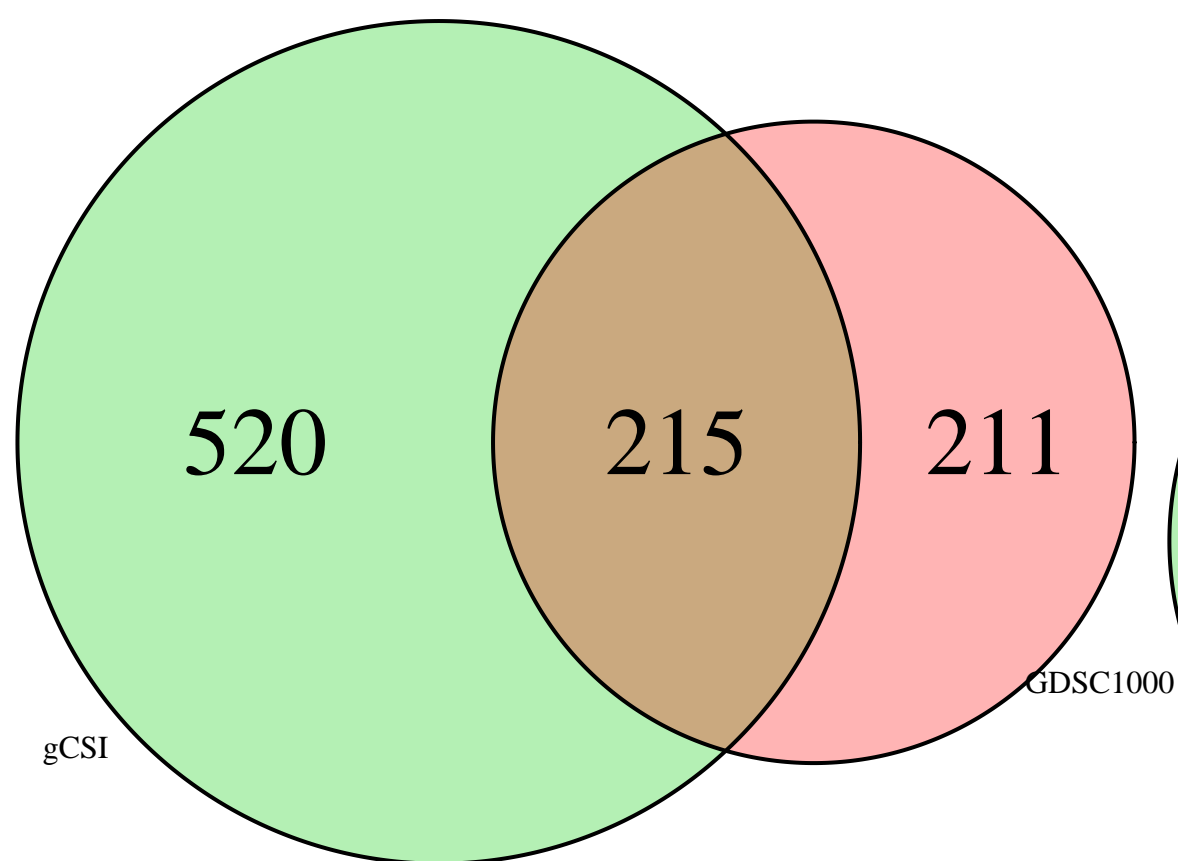

OES GDSC1000 vs UES gCSI

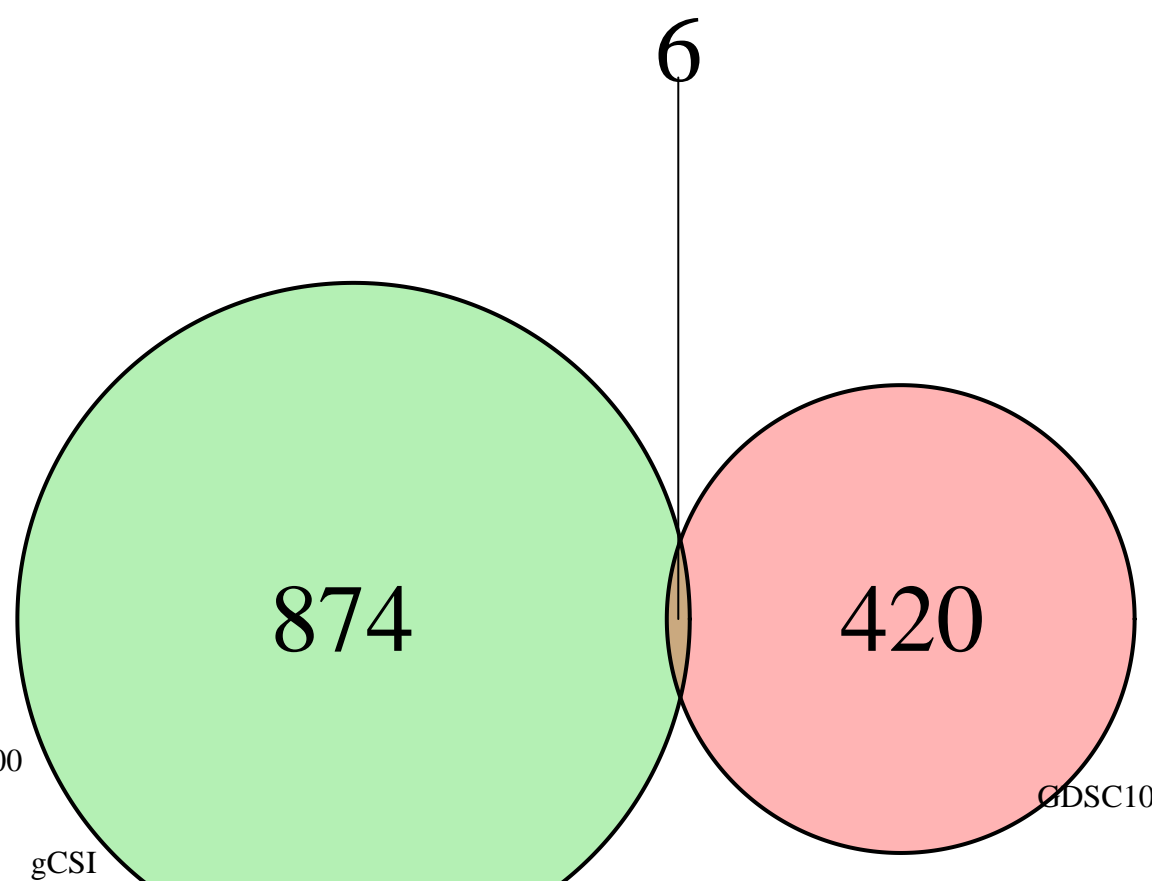

Supplement: Supplementary file 11 — Additional file 11. Integration of yeast phenomic and cancer cell line pharmacogenomic data to predict human genes that modify doxorubicin toxicity in cancer cells. (A) Tables of UES and OES human genes and whether their yeast homologs were found to be deletion enhancing or deletion suppressing, respectively. (B-C) Overlap between the gCSI and GDSC1000 databases with regard to UES and OES human genes (B) across all tissues or (C) for individual tissues. Note: the intersection of UES with OES between gCSI and GDSC was used as a negative control for assessing UES and OES overlap. (D-E) Yeast phenomic doxorubicin-gene interaction profiles for homologs of human UES or OES genes, sub-classified according to interaction type (deletion enhancing or suppressing) and Warburg-dependence of the interaction, for the (D) gCSI or (E) GDSC1000 databases. Similar to Fig. 11, yeast-human homology relationships are shown to the left of heatmaps (blue - one to one; green - one to many; red - many to many). (F-I) Interactive plots for yeast-human homologs, comparing the p-value of human genes to L interaction scores for yeast counterparts in (F, G) HLD or (H, I) HLEG from (F, H) gCSI or (G, I) GDSC1000. For the standardized coefficient (‘estimate’; color gradient), a negative value (purple) indicates UES, while a positive value (orange) indicates OES. Thus, the model would predict causality for a human gene if its yeast homolog has a positive L interaction (deletion enhancing) and is colored purple (UES), or a negative L interaction (deletion suppressing) and colored orange (OES). Genes are only plotted if the human homolog was significant (p-value < 0.05). [file 40170_2019_201_MOESM11_ESM.bz2 › Additional_File11_Pharmacogenomics/B - AcrossTissue.pdf]

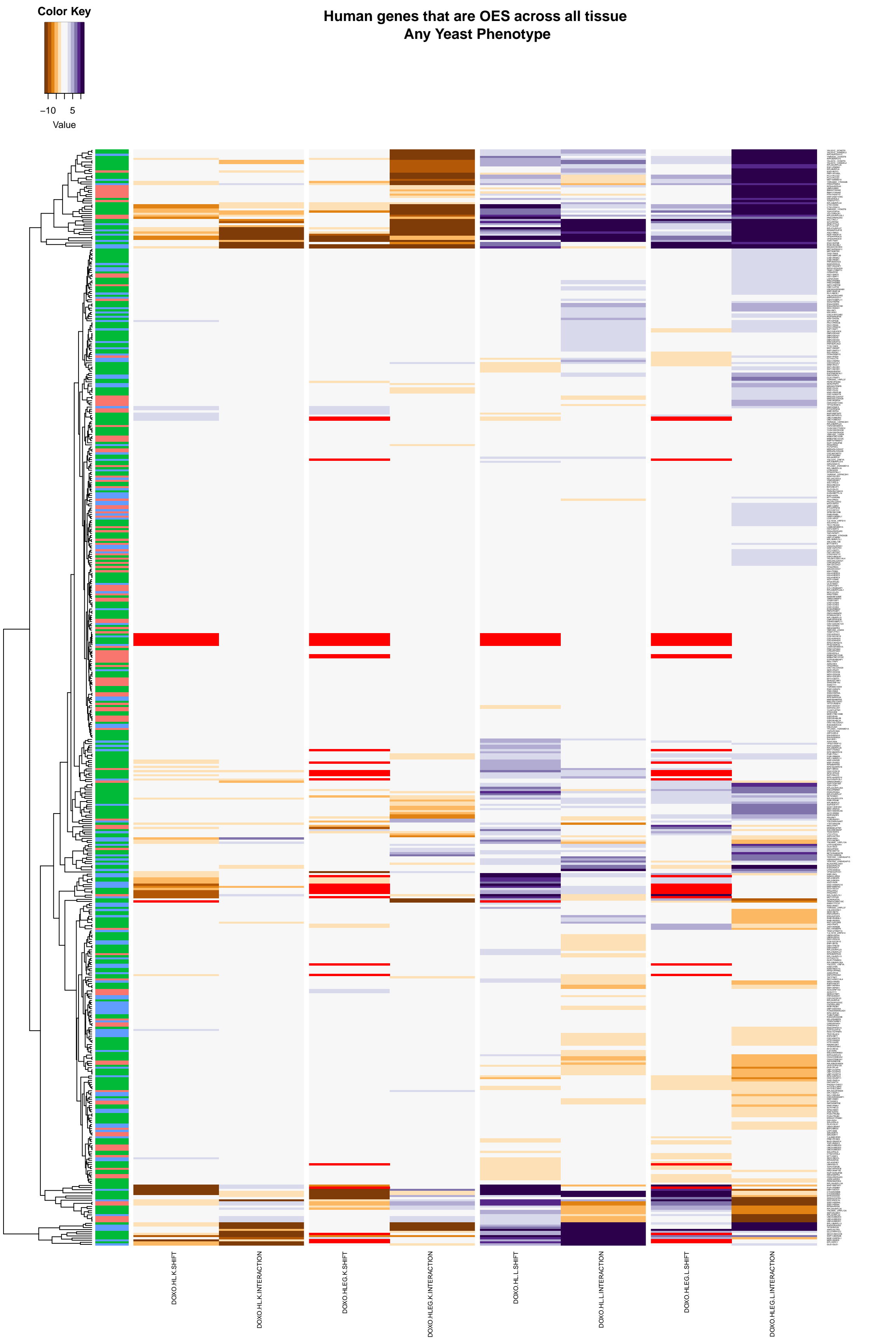

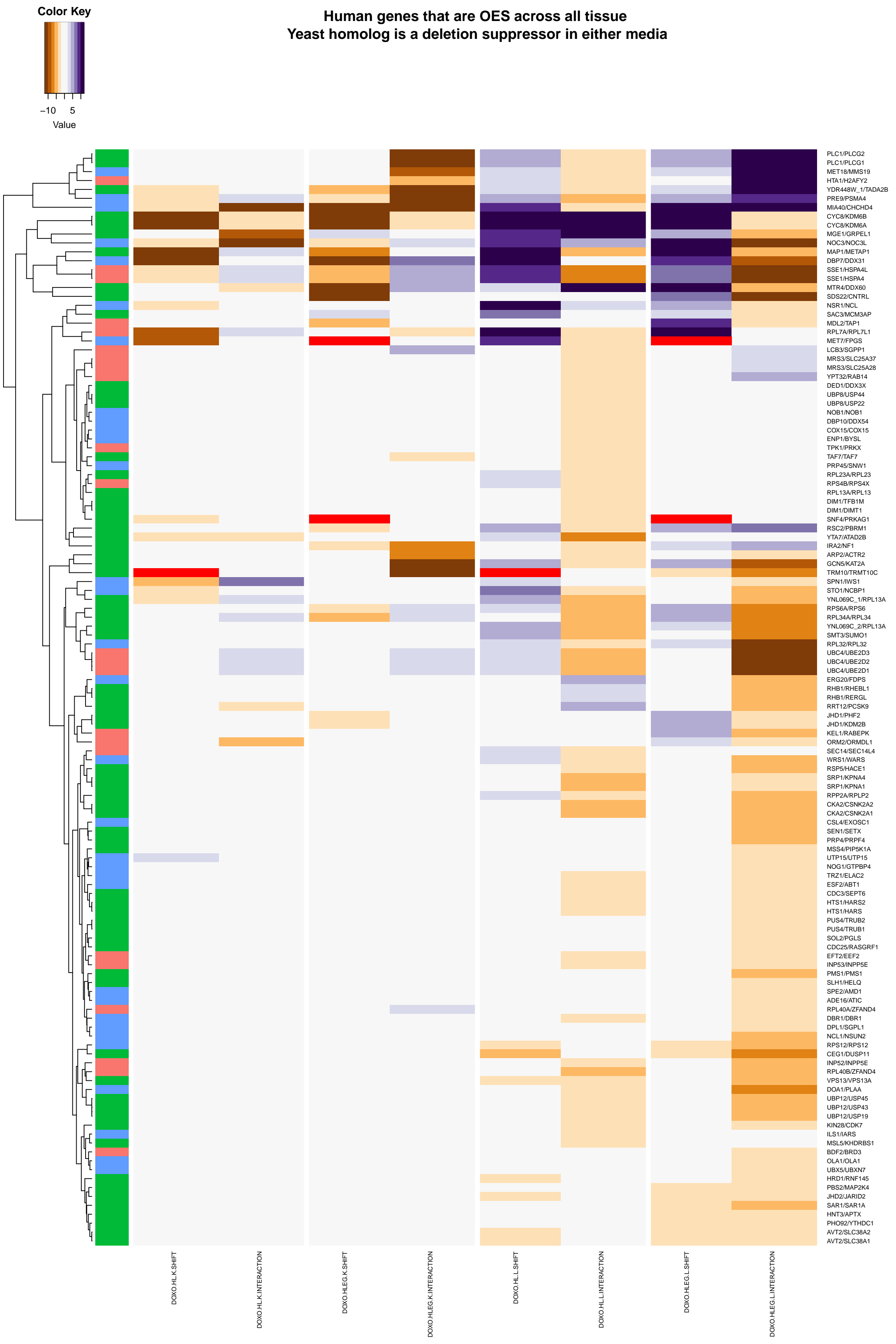

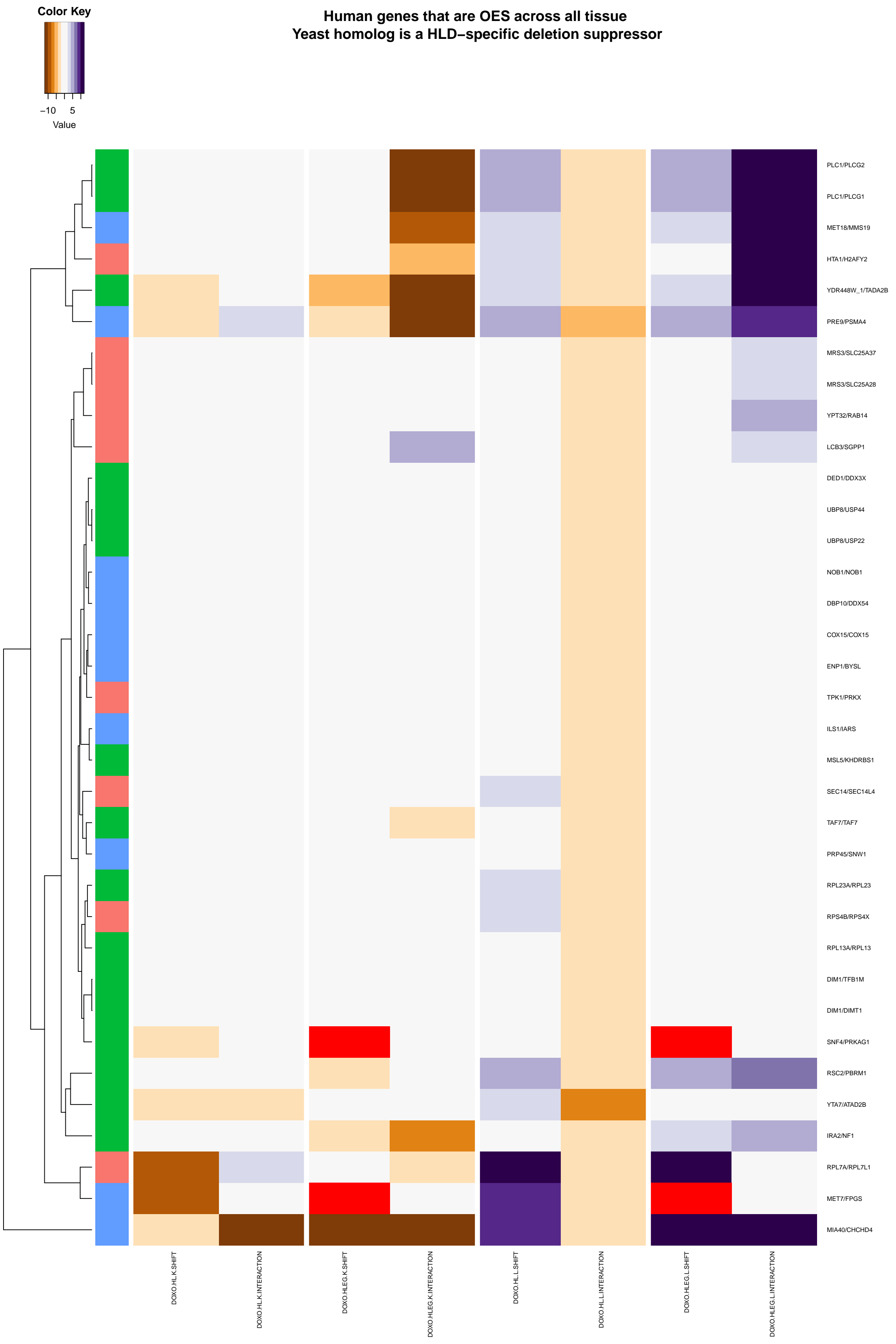

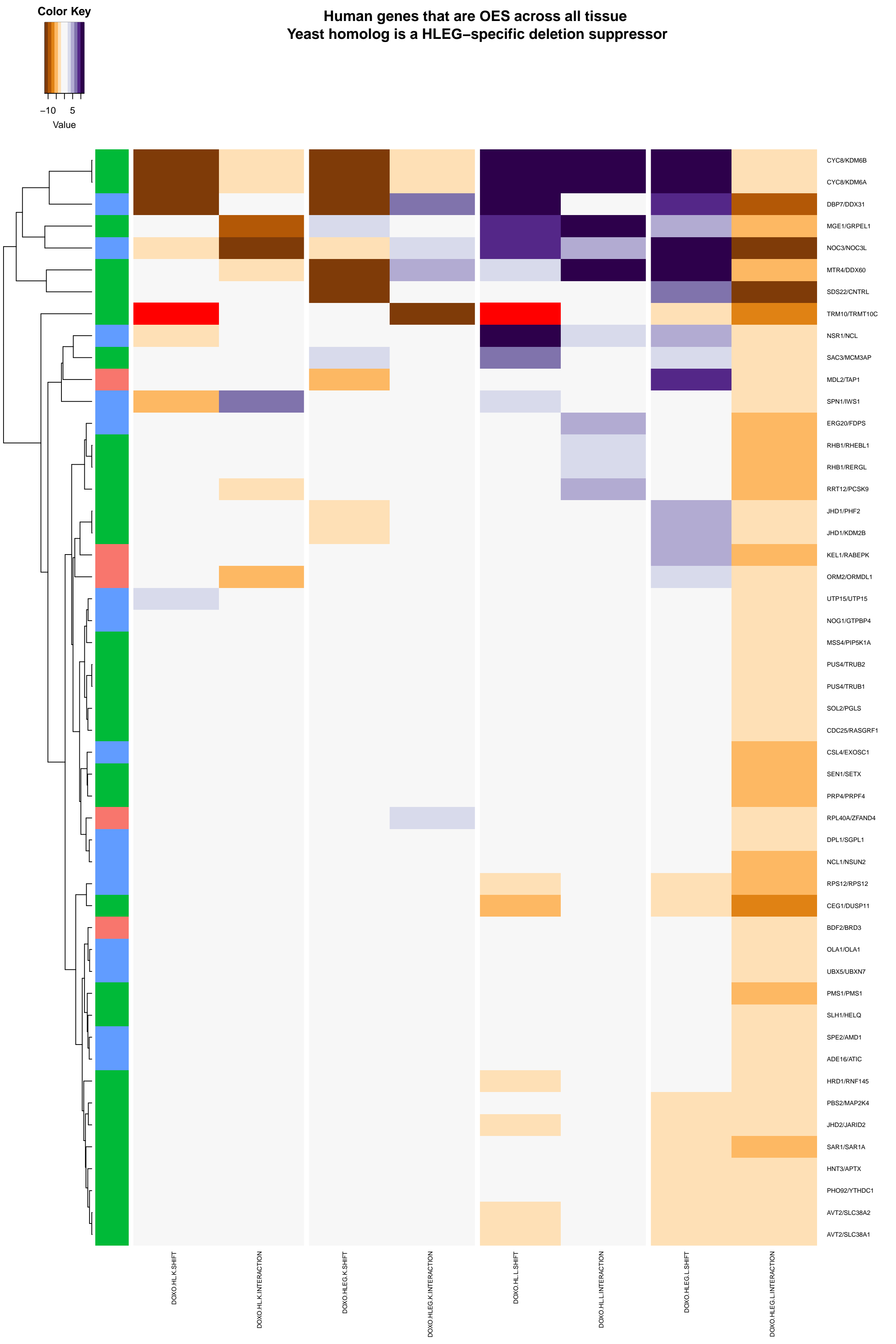

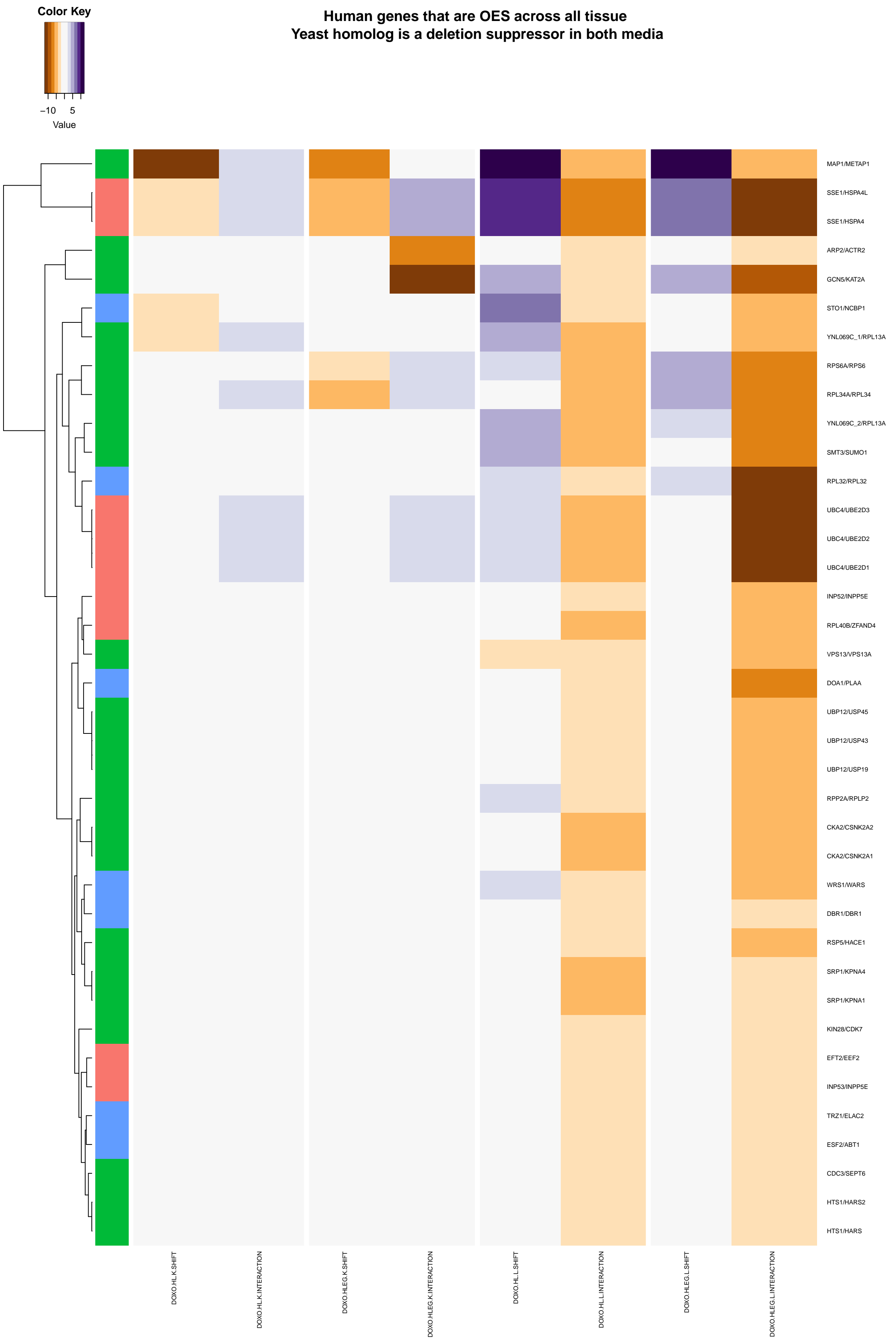

Color Key

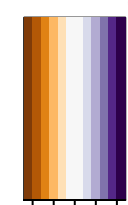

-10 5  
Value

# Human genes that are UES across all tissue Any Yeast Phenotype

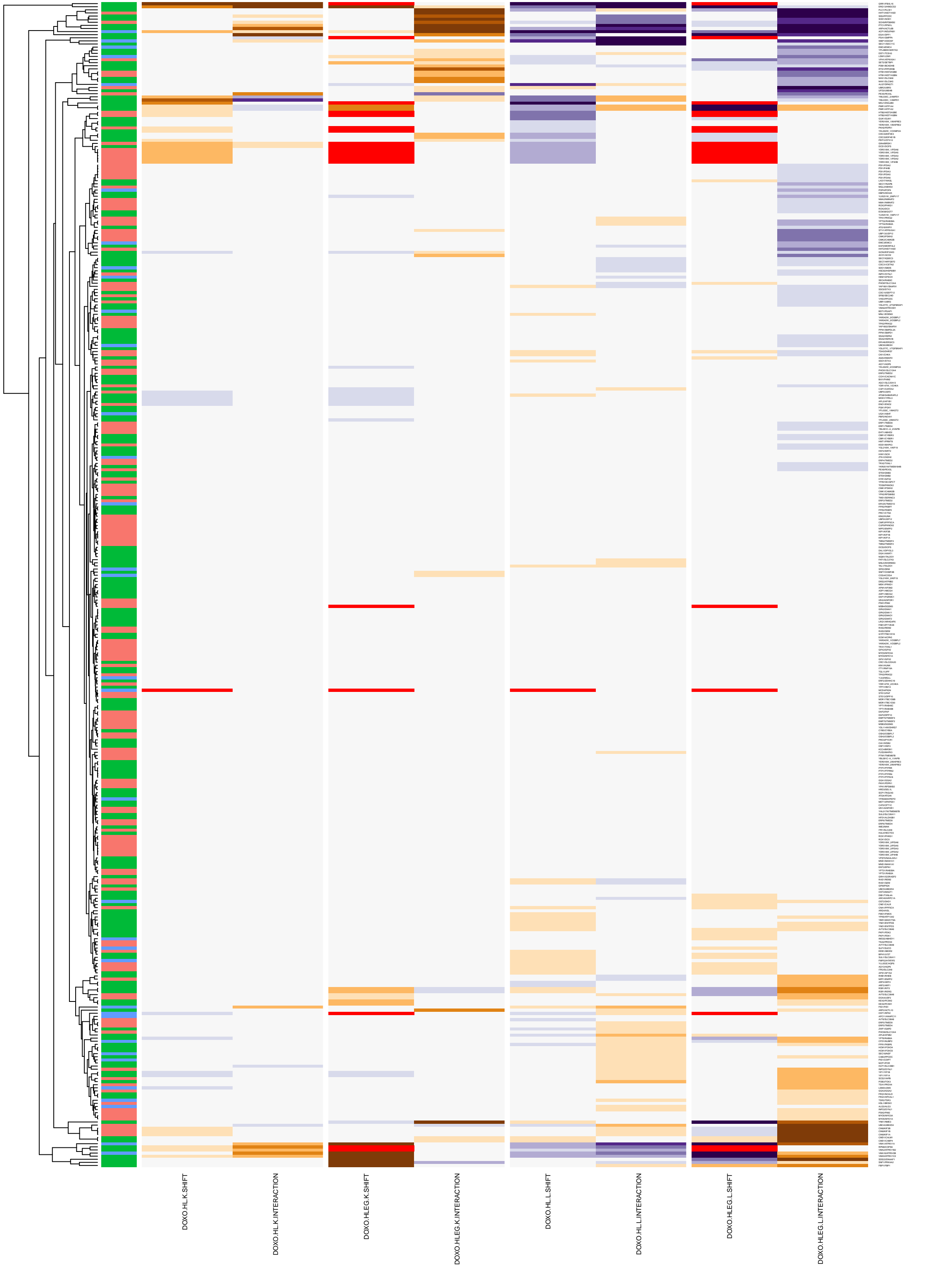

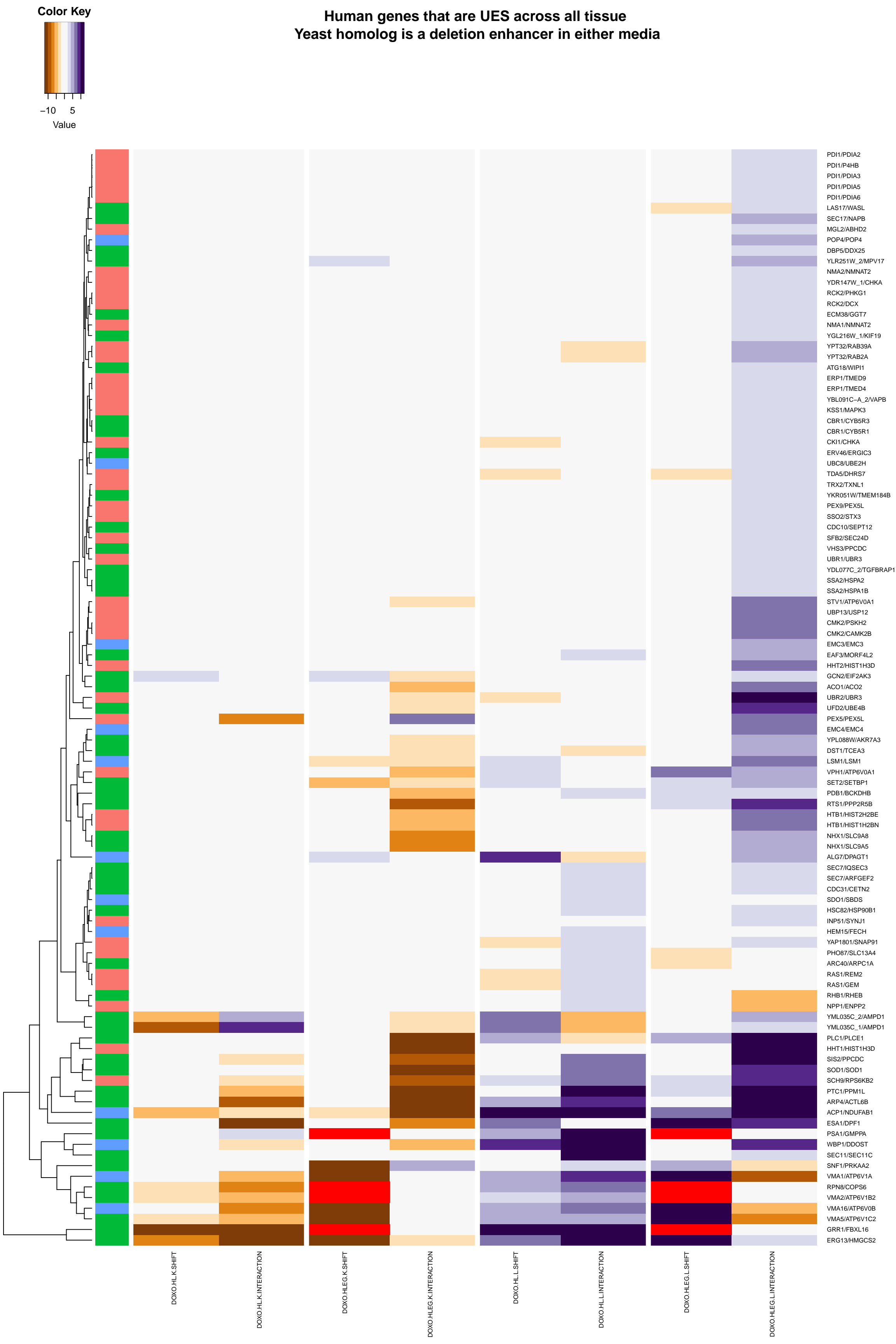

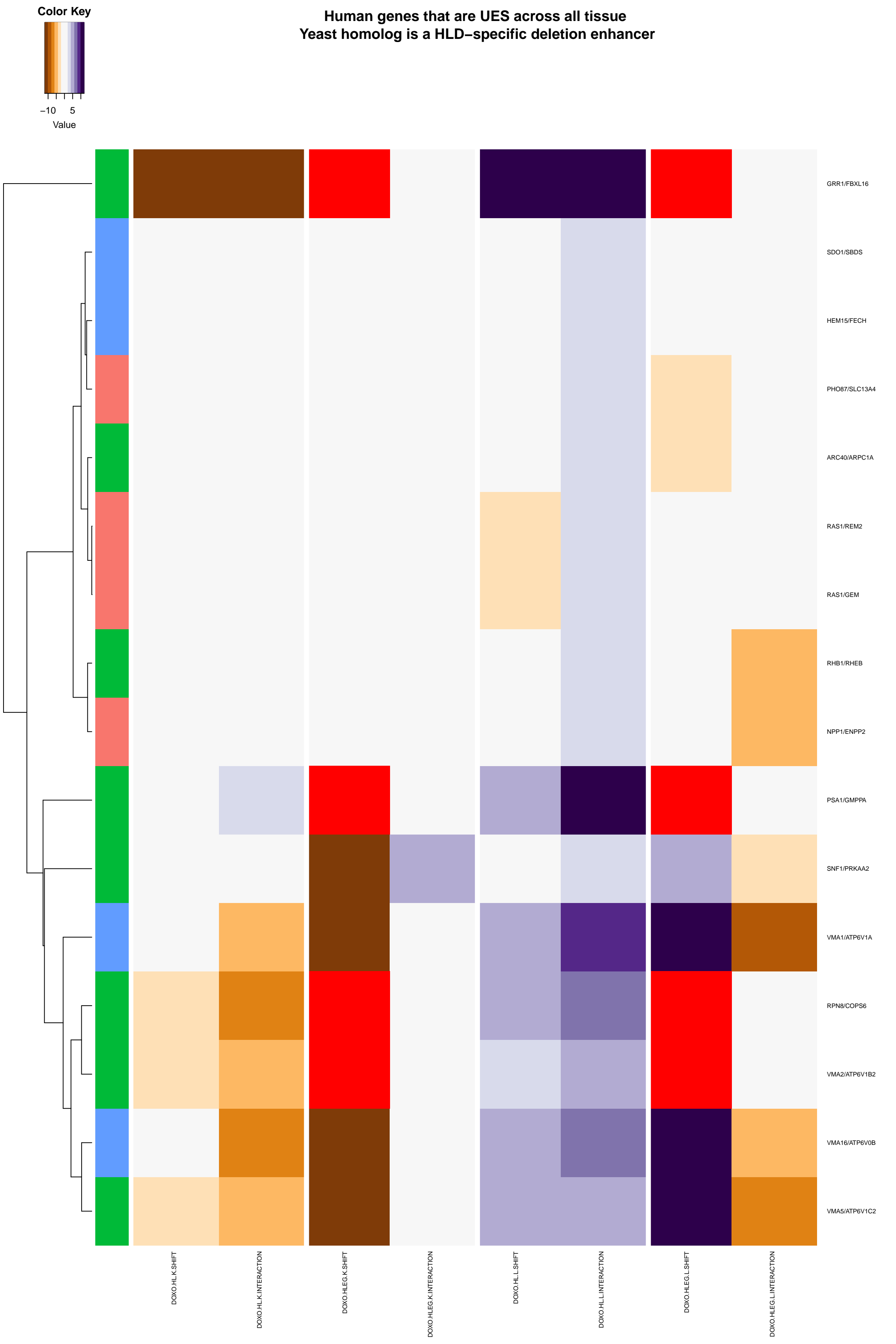

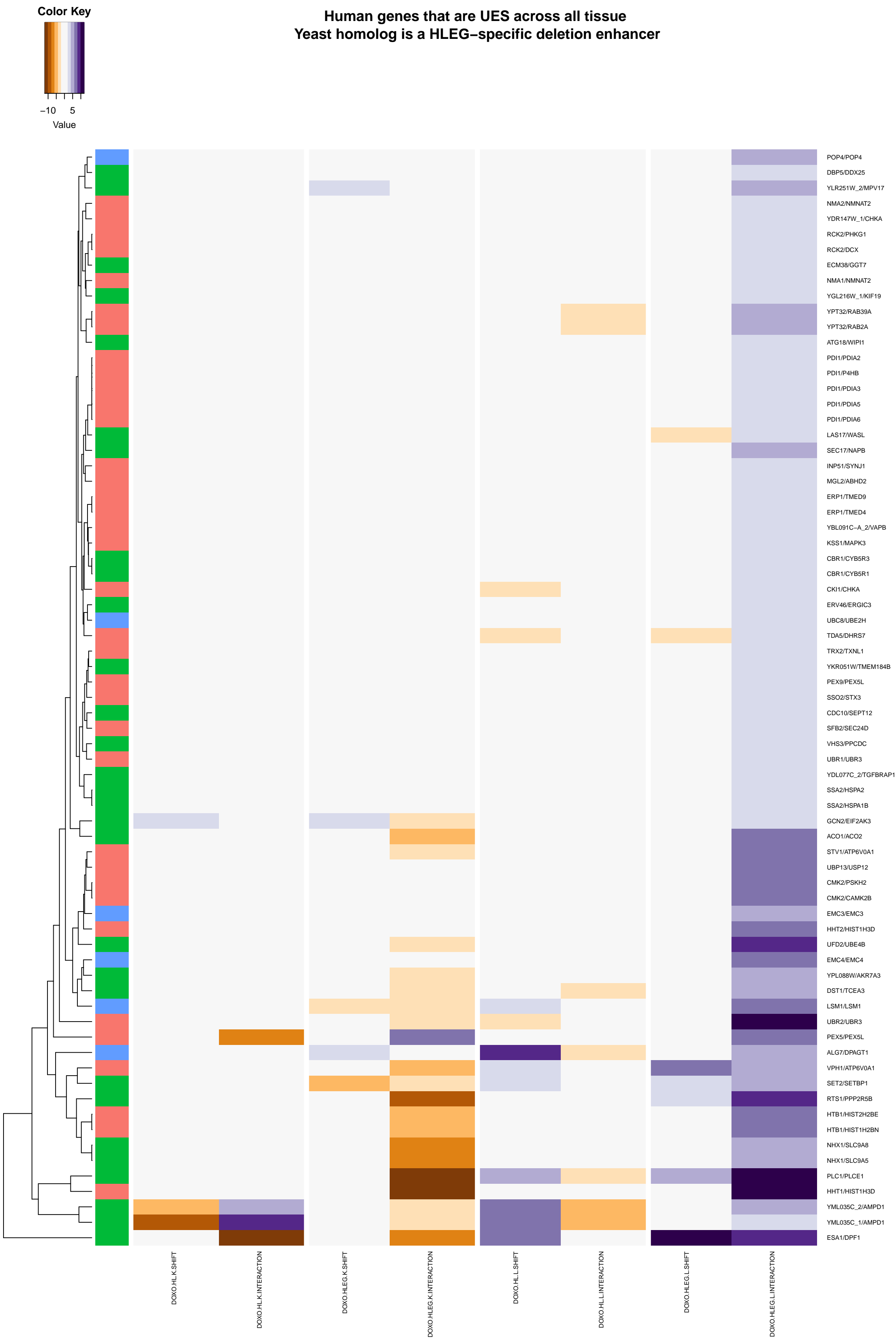

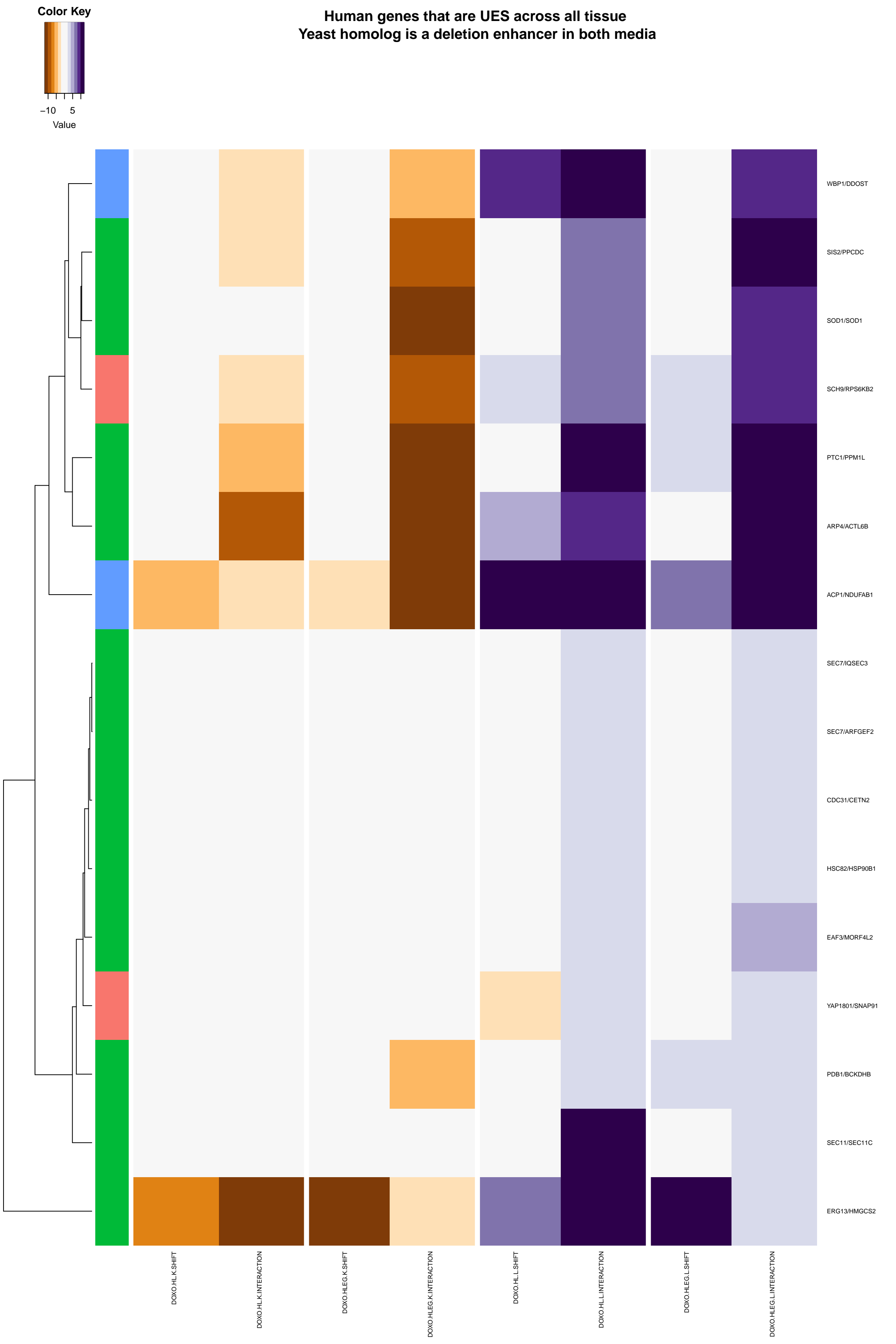

Supplement: Supplementary file 11 — Additional file 11. Integration of yeast phenomic and cancer cell line pharmacogenomic data to predict human genes that modify doxorubicin toxicity in cancer cells. (A) Tables of UES and OES human genes and whether their yeast homologs were found to be deletion enhancing or deletion suppressing, respectively. (B-C) Overlap between the gCSI and GDSC1000 databases with regard to UES and OES human genes (B) across all tissues or (C) for individual tissues. Note: the intersection of UES with OES between gCSI and GDSC was used as a negative control for assessing UES and OES overlap. (D-E) Yeast phenomic doxorubicin-gene interaction profiles for homologs of human UES or OES genes, sub-classified according to interaction type (deletion enhancing or suppressing) and Warburg-dependence of the interaction, for the (D) gCSI or (E) GDSC1000 databases. Similar to Fig. 11, yeast-human homology relationships are shown to the left of heatmaps (blue - one to one; green - one to many; red - many to many). (F-I) Interactive plots for yeast-human homologs, comparing the p-value of human genes to L interaction scores for yeast counterparts in (F, G) HLD or (H, I) HLEG from (F, H) gCSI or (G, I) GDSC1000. For the standardized coefficient (‘estimate’; color gradient), a negative value (purple) indicates UES, while a positive value (orange) indicates OES. Thus, the model would predict causality for a human gene if its yeast homolog has a positive L interaction (deletion enhancing) and is colored purple (UES), or a negative L interaction (deletion suppressing) and colored orange (OES). Genes are only plotted if the human homolog was significant (p-value < 0.05). [file 40170_2019_201_MOESM11_ESM.bz2 › Additional_File11_Pharmacogenomics/E - GDSC1000_Heatmaps.pdf]
