# Supplementary figures and images for: A yeast phenomic model for the influence of Warburg metabolism on genetic buffering of doxorubicin (part 1 of 2)
Source: Cancer Metab. 2019 Oct 23;7:9. doi: 10.1186/s40170-019-0201-3 (PMC6806529; doi:10.1186/s40170-019-0201-3)

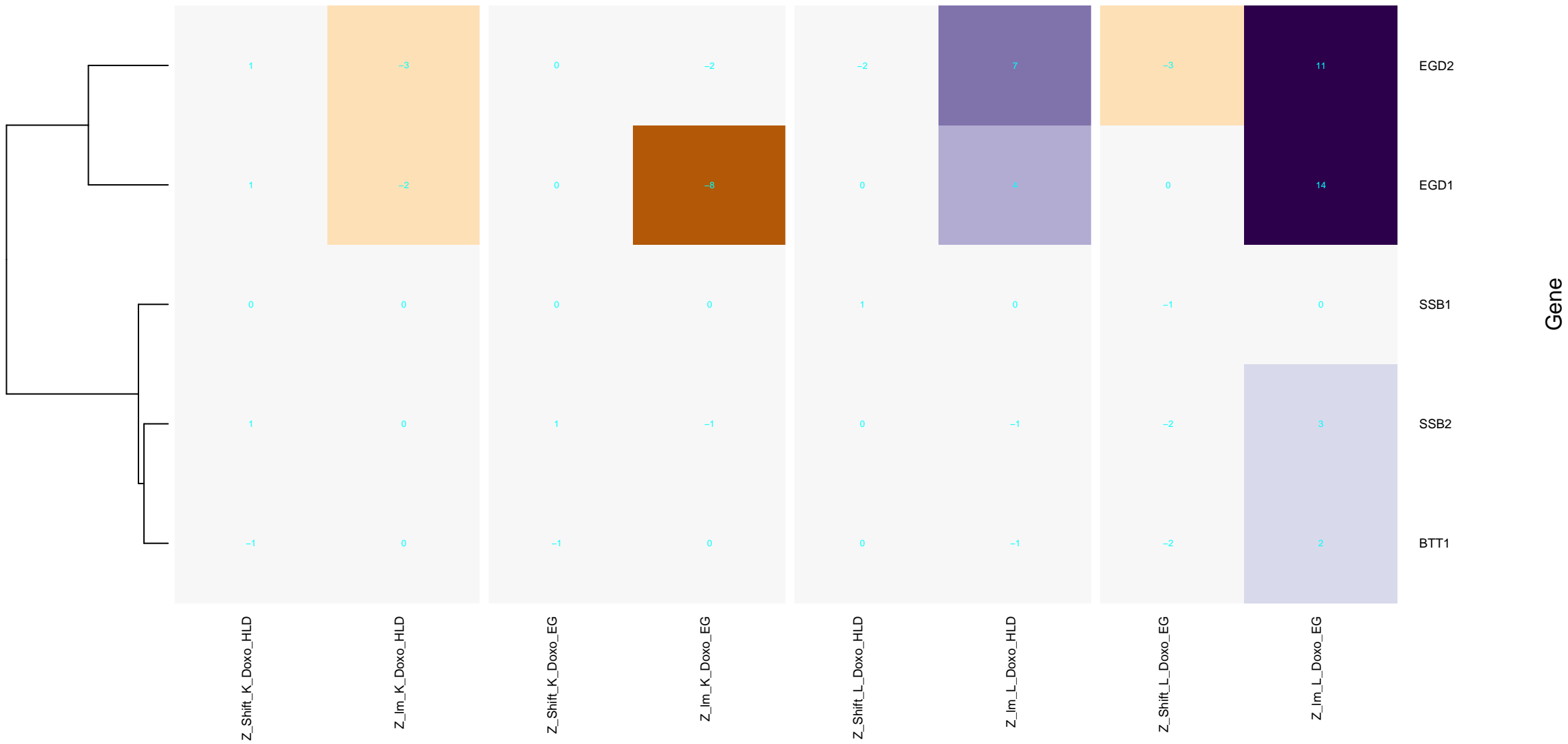

Supplement: Supplementary file 9 — Additional file 9. GO term-specific heatmaps for REMc/GTF-enriched clusters. GO term-specific heatmaps for significant GO process terms were generated as described in methods and Figs. 3 and 4. Any related child terms are presented in subsequent pages of the parent file name. GO terms with more than 100 children, with 2 or fewer genes annotated to the term, or a file size over 300KB are not shown. All heatmaps are generated with the same layout (see Figs. 3 and 4). [file 40170_2019_201_MOESM9_ESM.bz2 › Additional_File9_GOTermHeatmaps/Additional_File9_GOTermHeatmap/'de_novo'_cotranslational_protein_folding.pdf]

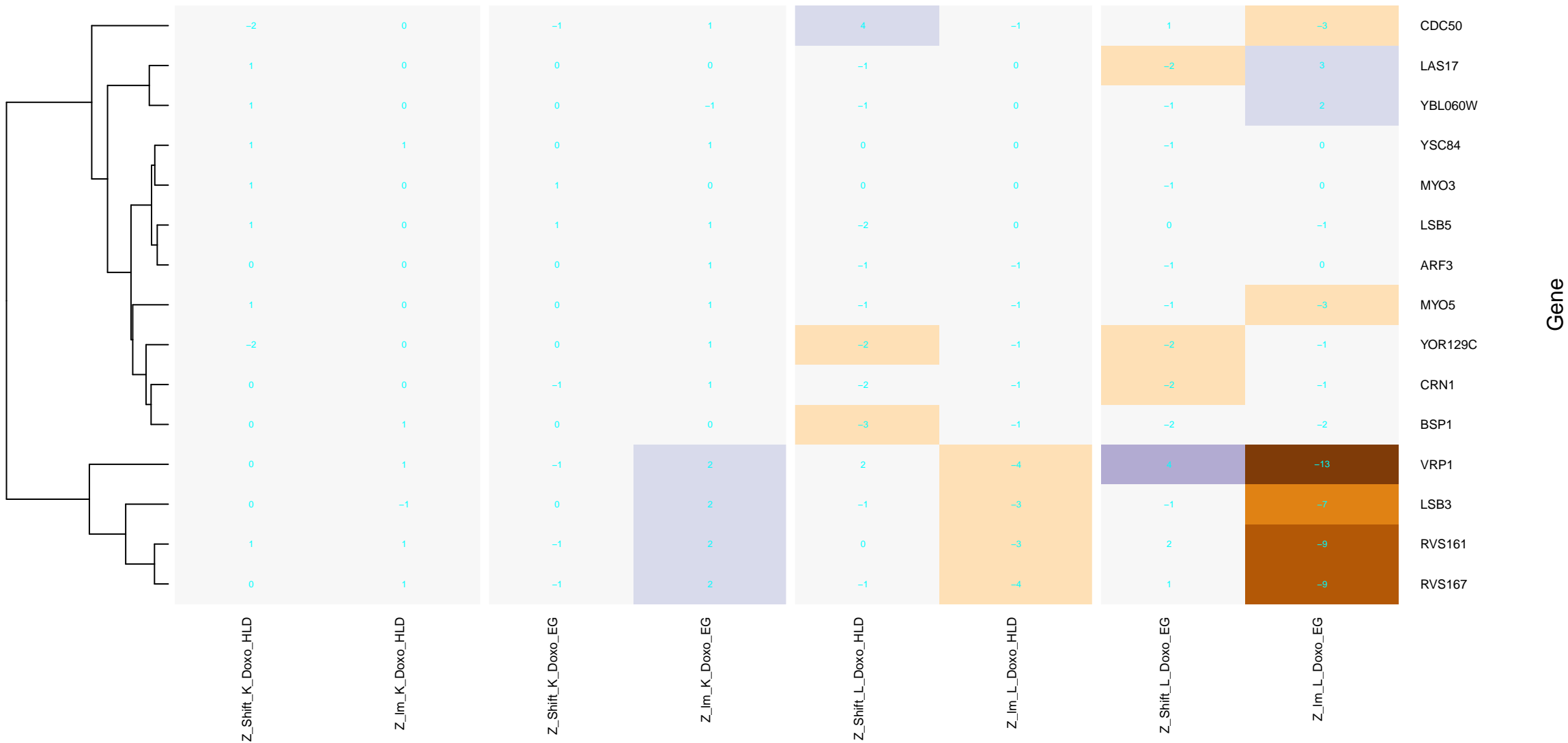

Supplement: Supplementary file 9 — Additional file 9. GO term-specific heatmaps for REMc/GTF-enriched clusters. GO term-specific heatmaps for significant GO process terms were generated as described in methods and Figs. 3 and 4. Any related child terms are presented in subsequent pages of the parent file name. GO terms with more than 100 children, with 2 or fewer genes annotated to the term, or a file size over 300KB are not shown. All heatmaps are generated with the same layout (see Figs. 3 and 4). [file 40170_2019_201_MOESM9_ESM.bz2 › Additional_File9_GOTermHeatmaps/Additional_File9_GOTermHeatmap/actin_cortical_patch_localization.pdf]

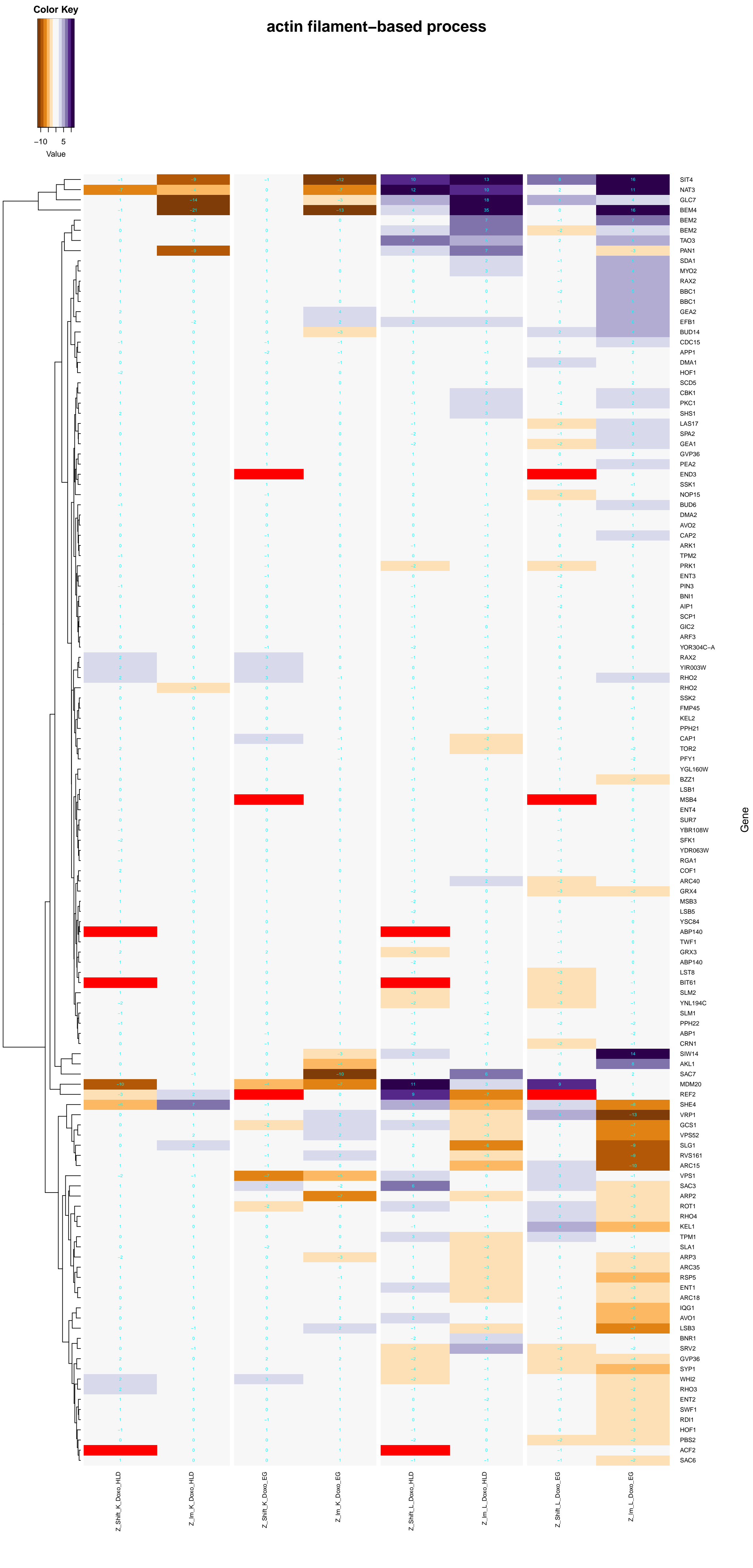



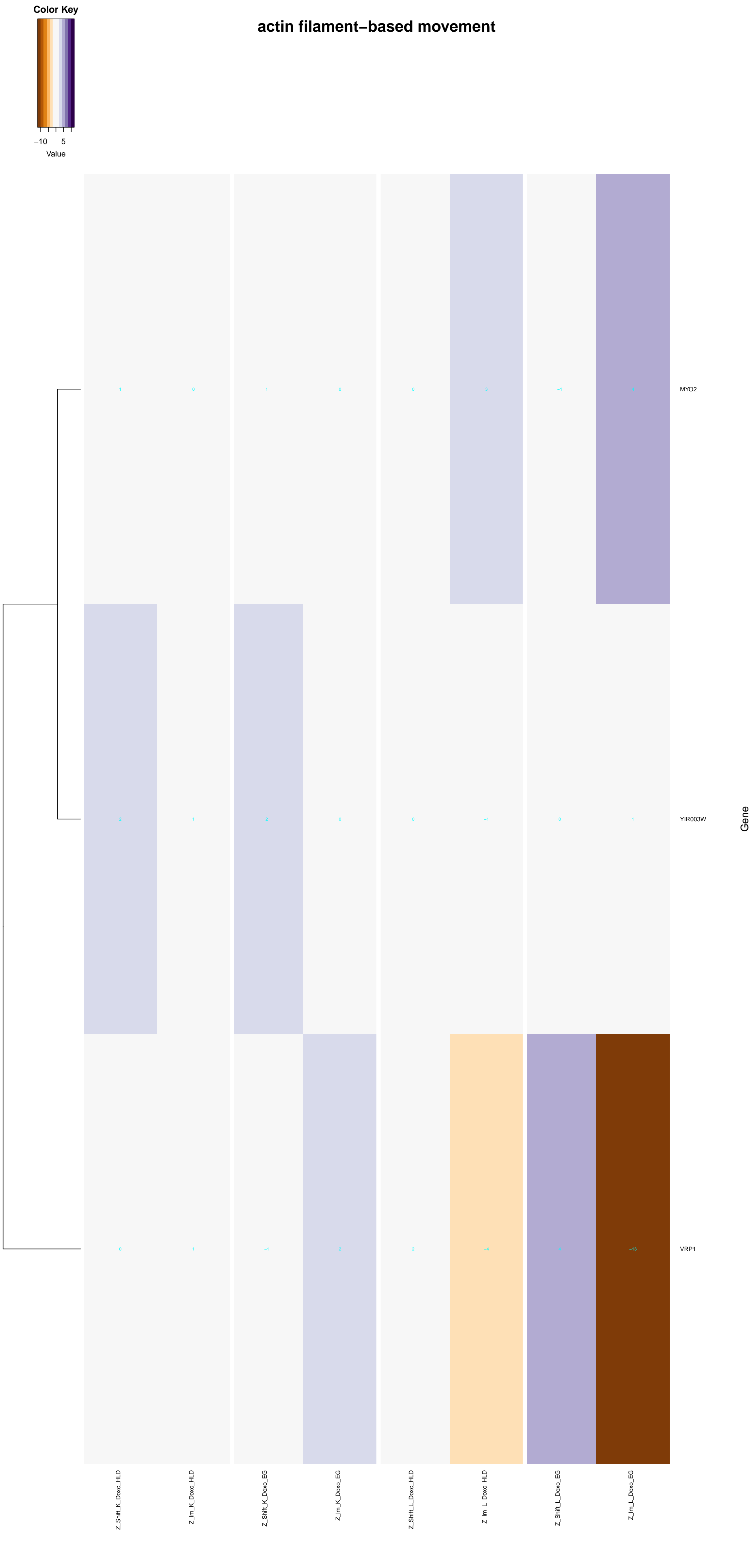

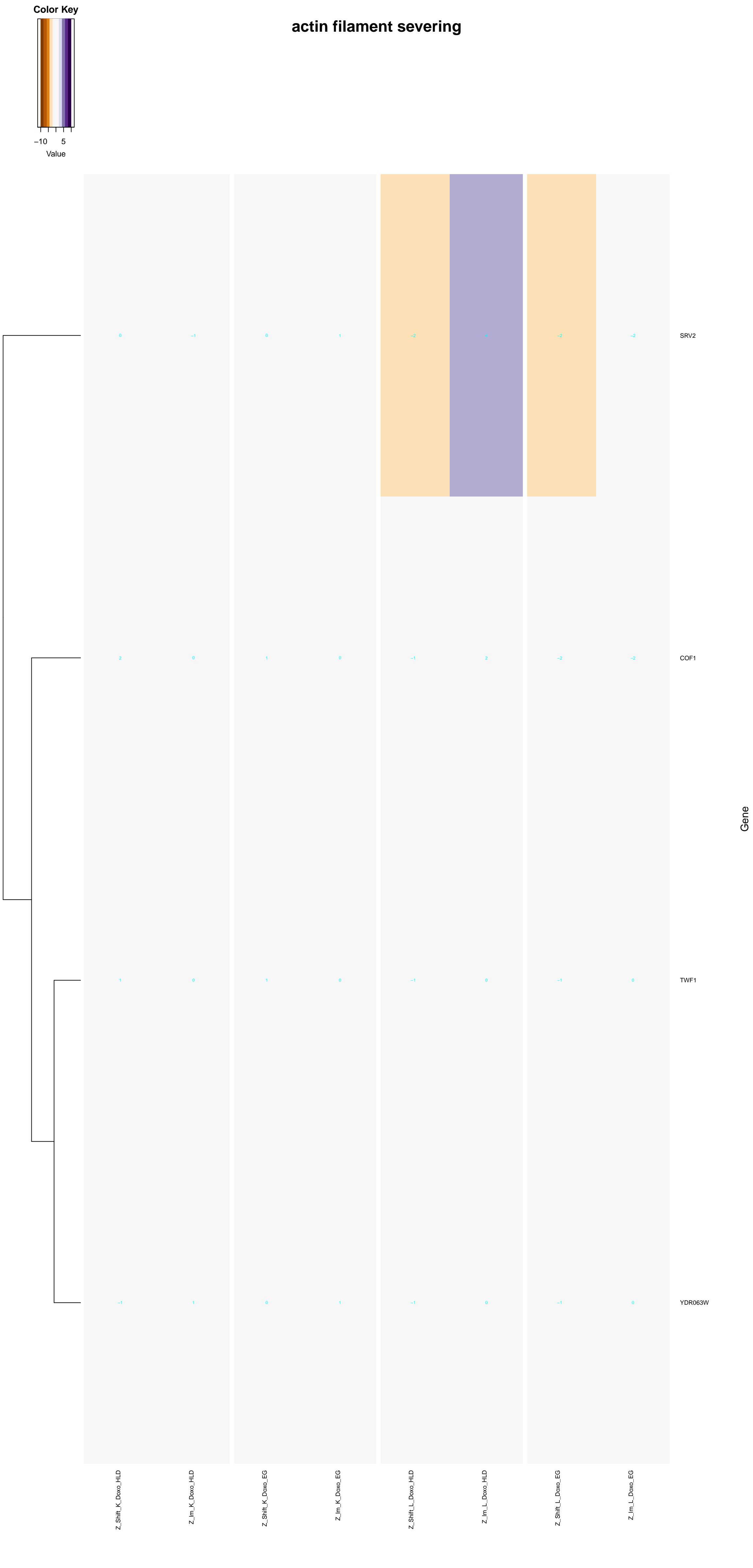

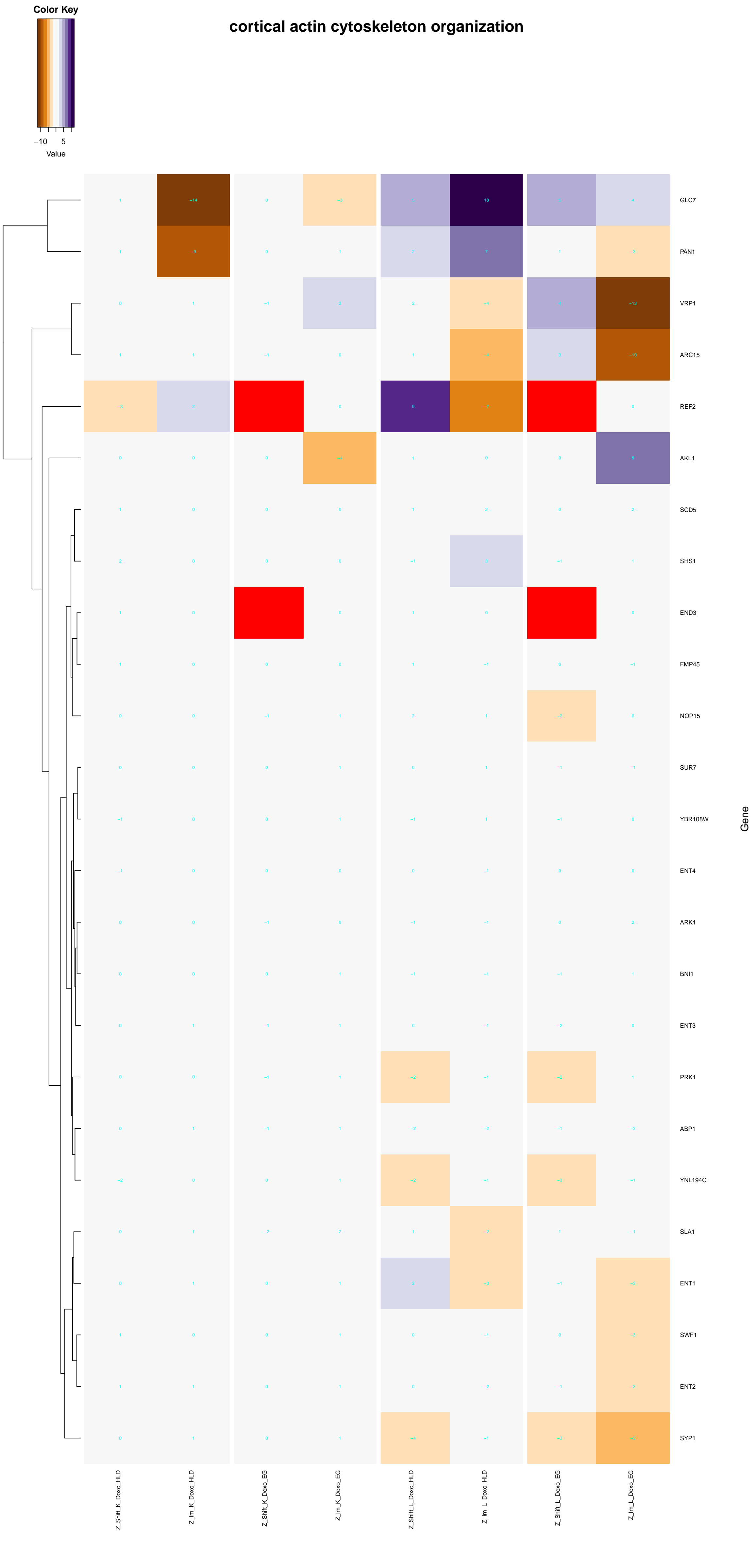

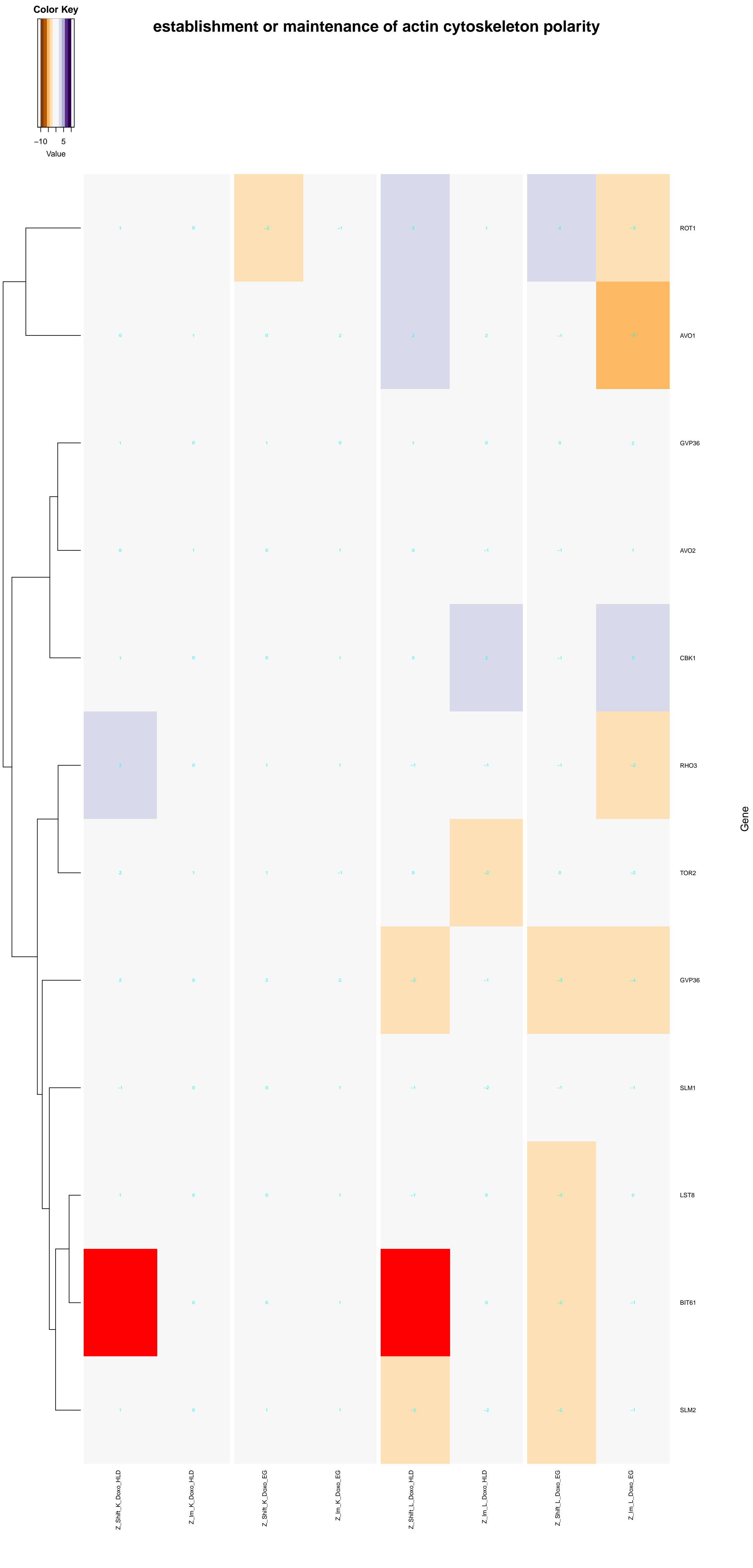

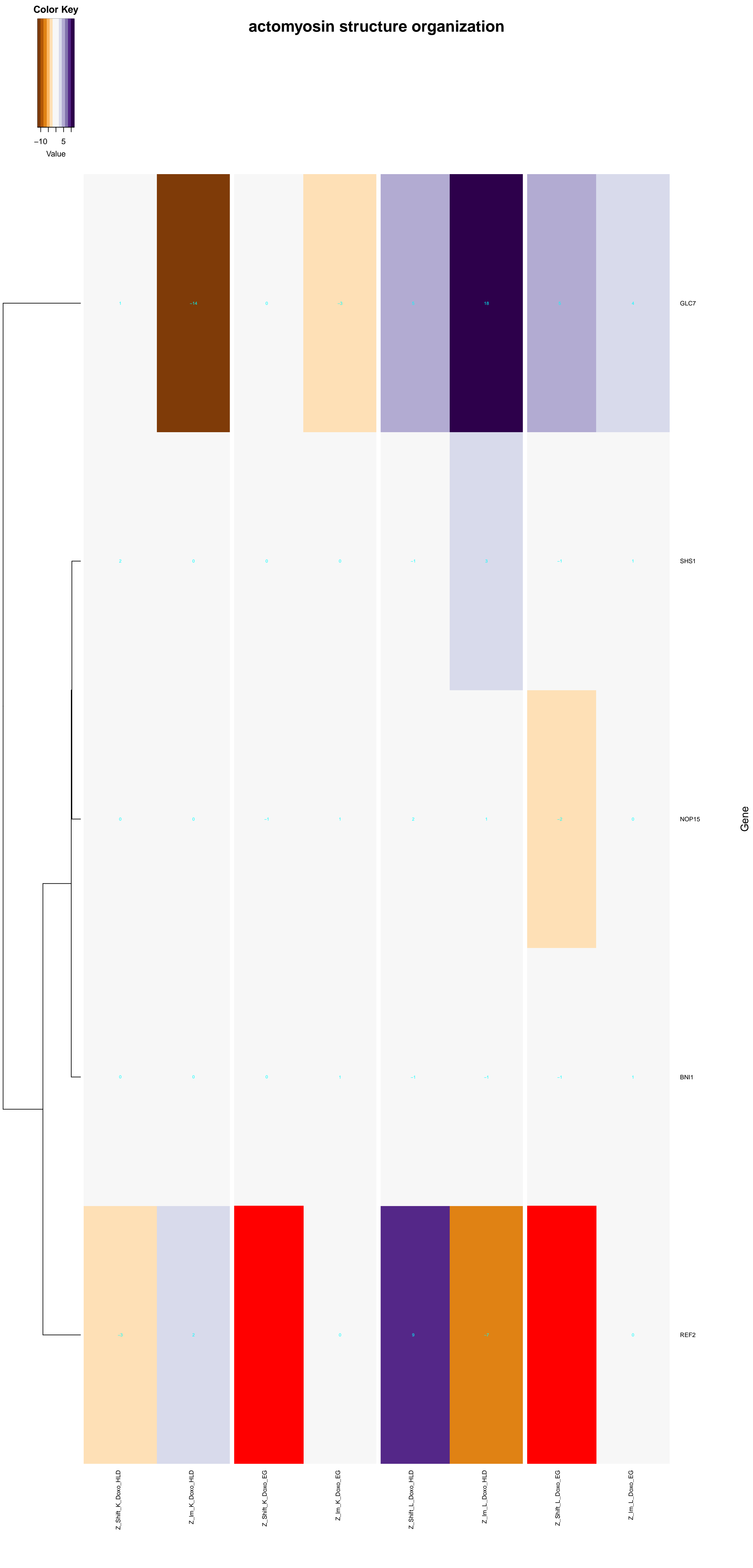

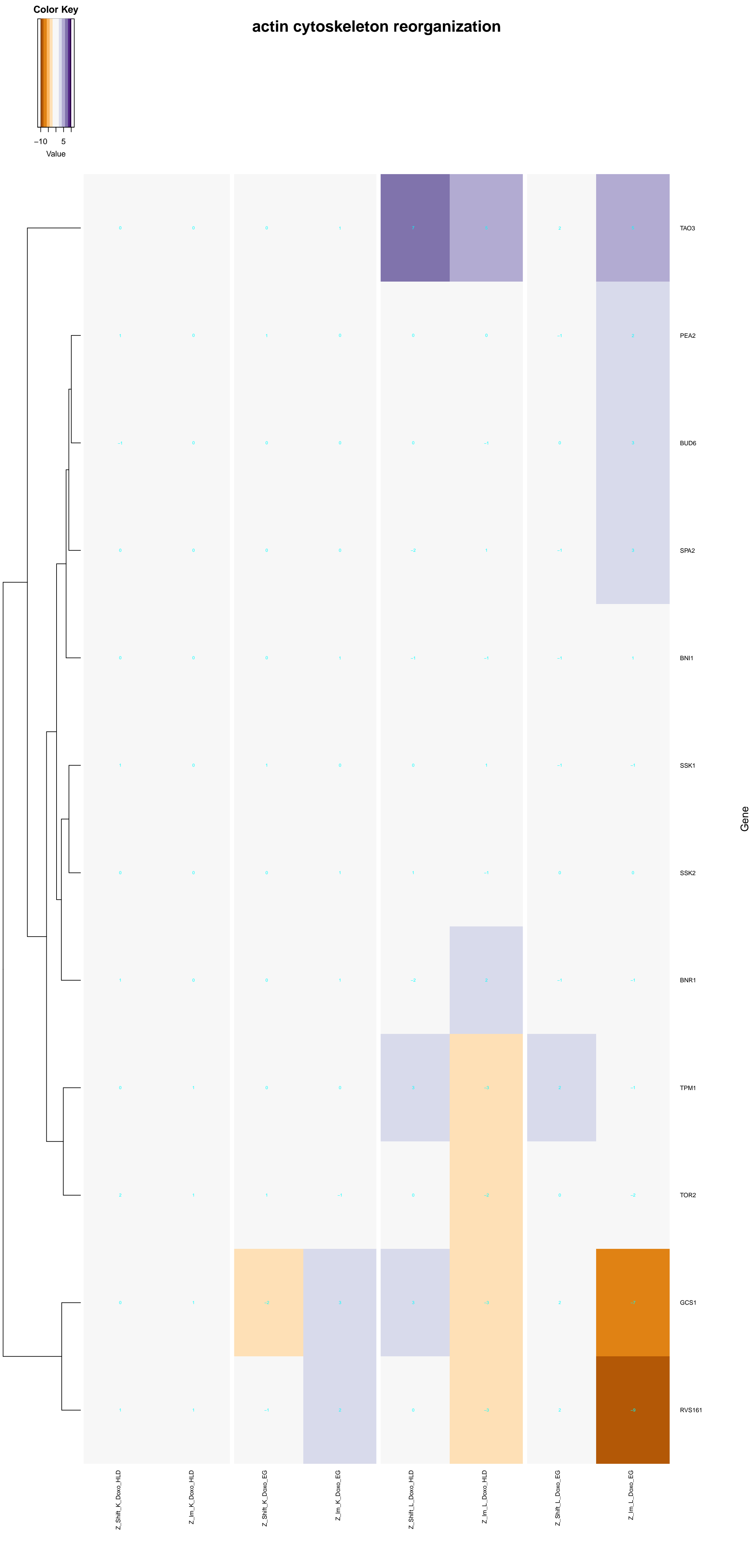

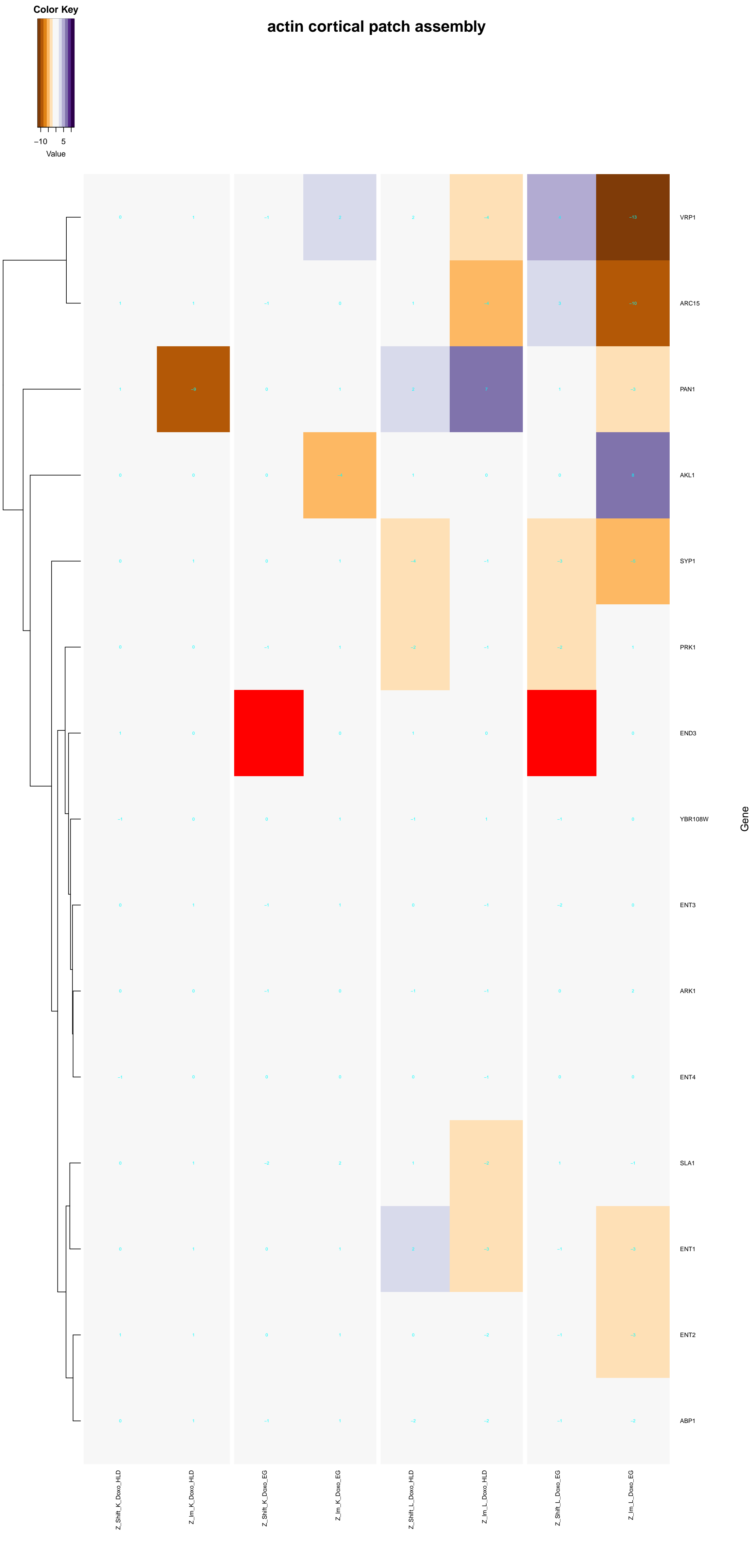

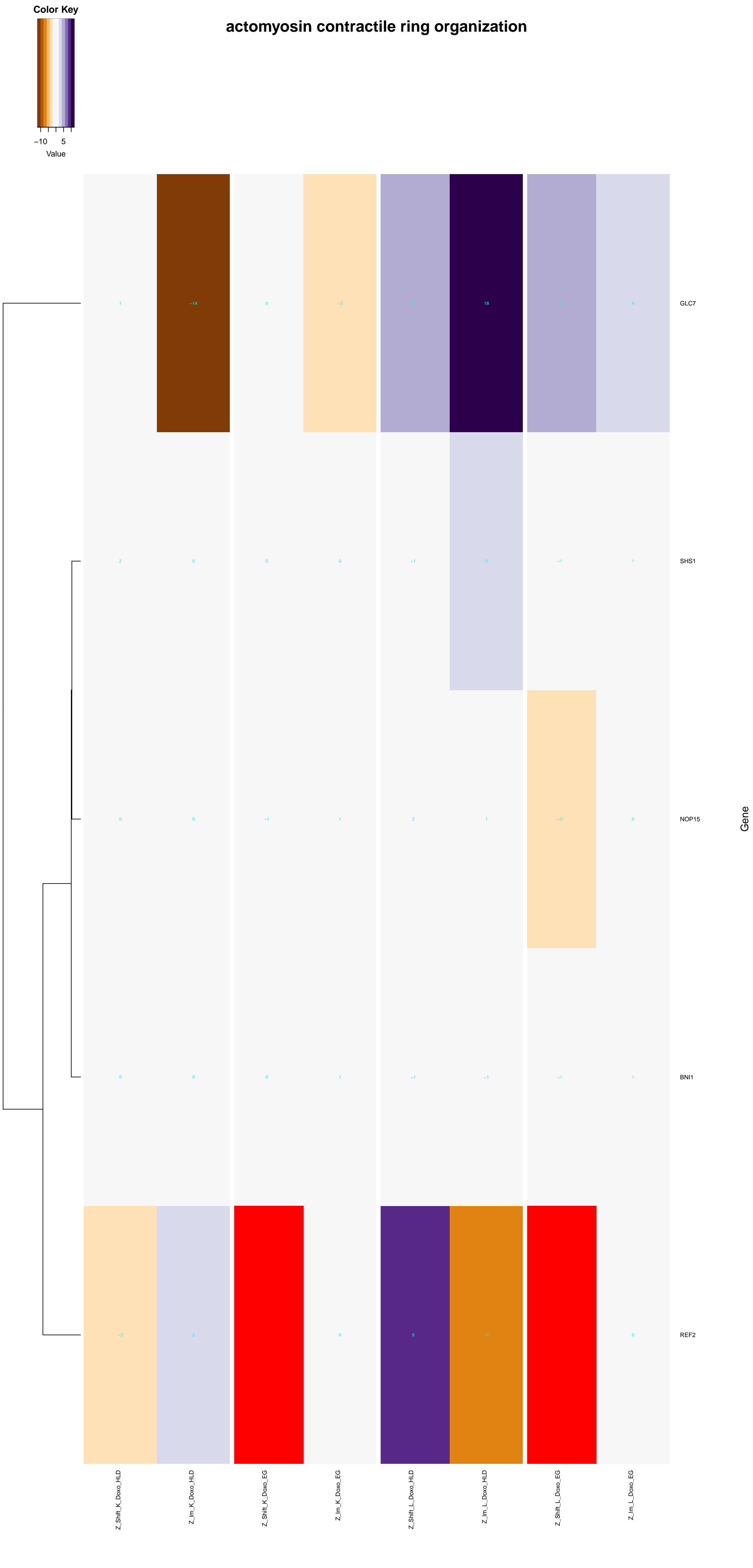

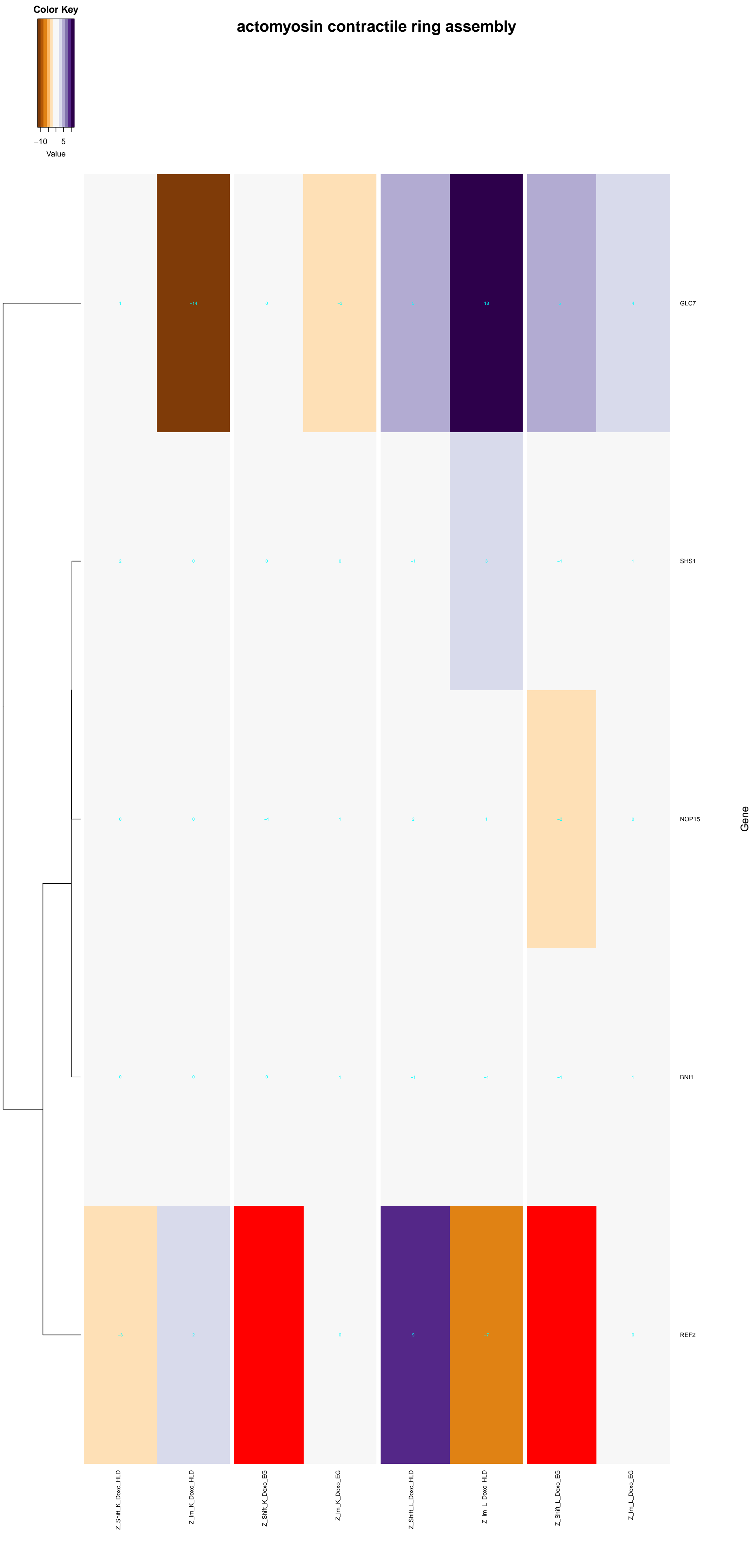

Supplement: Supplementary file 9 — Additional file 9. GO term-specific heatmaps for REMc/GTF-enriched clusters. GO term-specific heatmaps for significant GO process terms were generated as described in methods and Figs. 3 and 4. Any related child terms are presented in subsequent pages of the parent file name. GO terms with more than 100 children, with 2 or fewer genes annotated to the term, or a file size over 300KB are not shown. All heatmaps are generated with the same layout (see Figs. 3 and 4). [file 40170_2019_201_MOESM9_ESM.bz2 › Additional_File9_GOTermHeatmaps/Additional_File9_GOTermHeatmap/actin_filament-based_process.pdf]

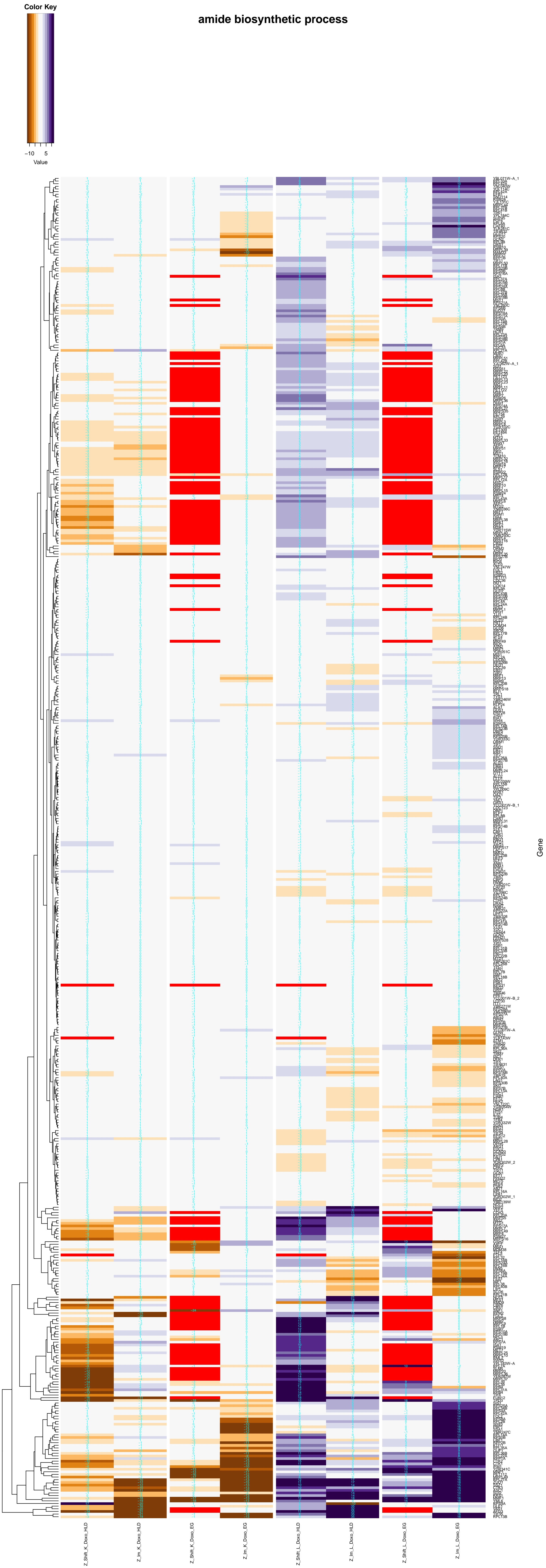

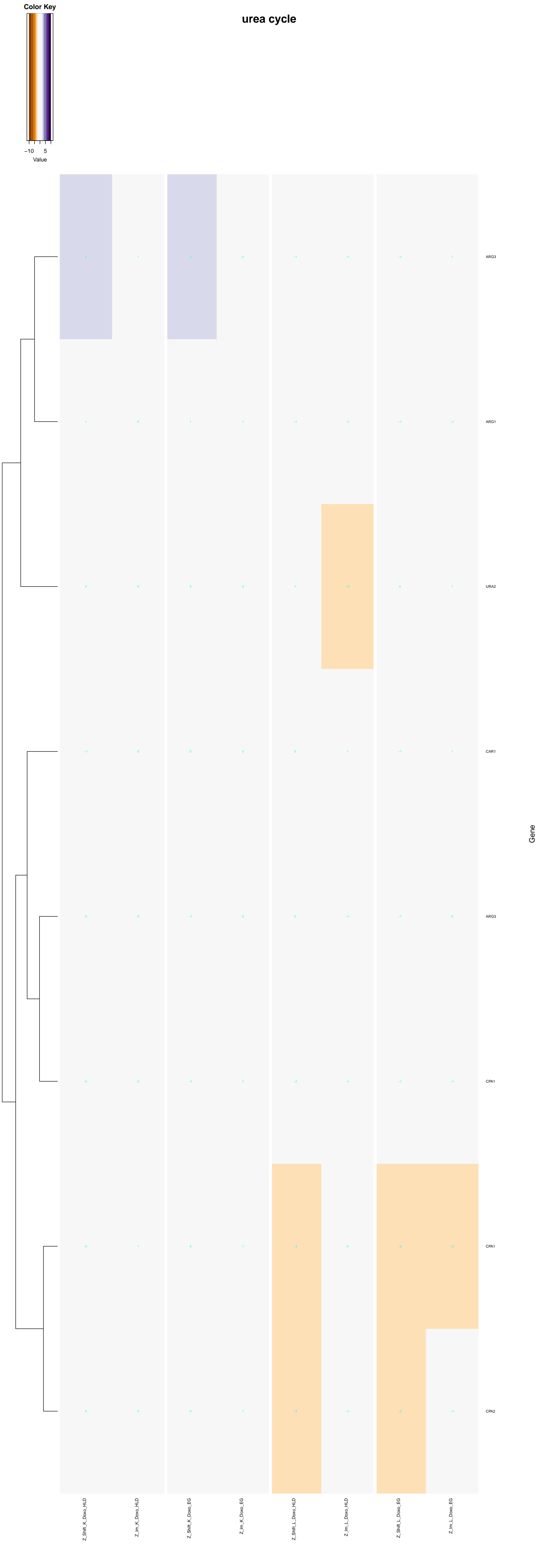

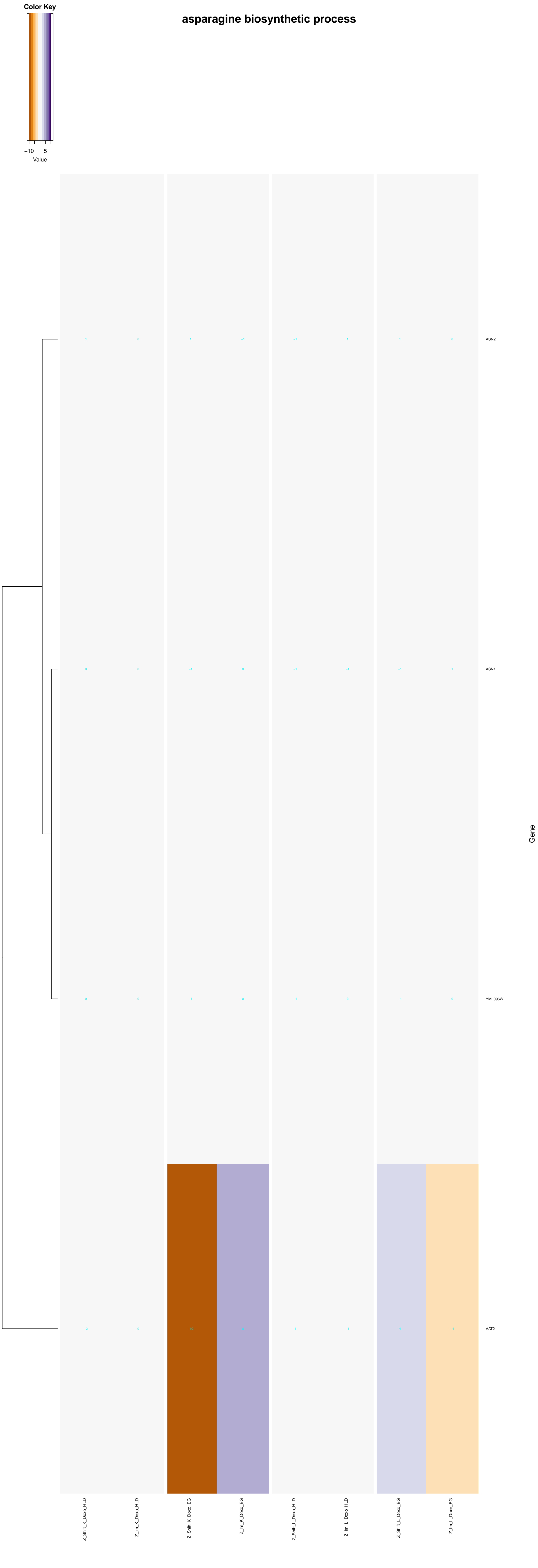

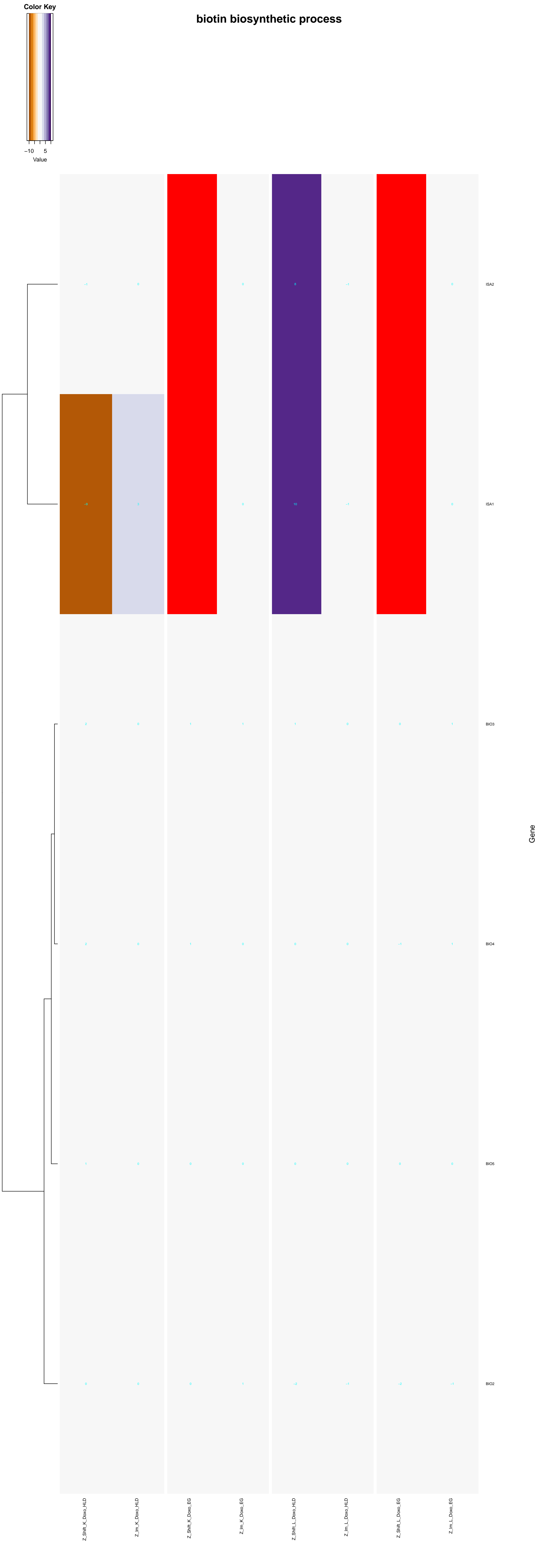

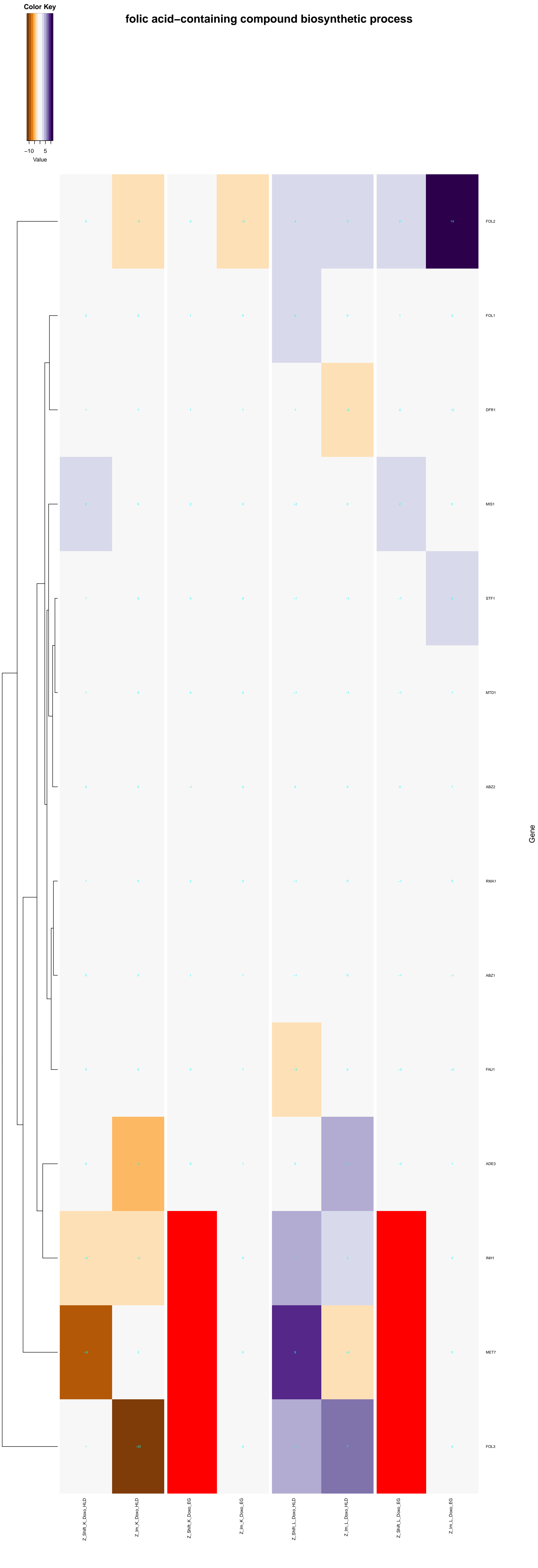

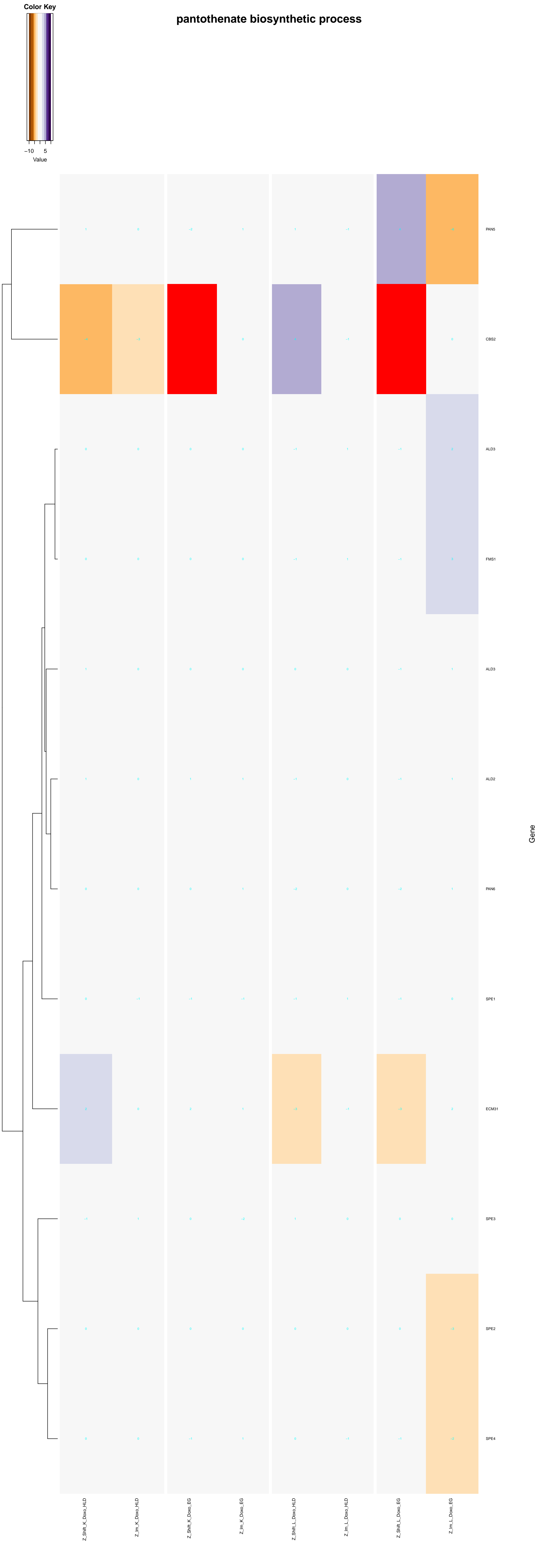

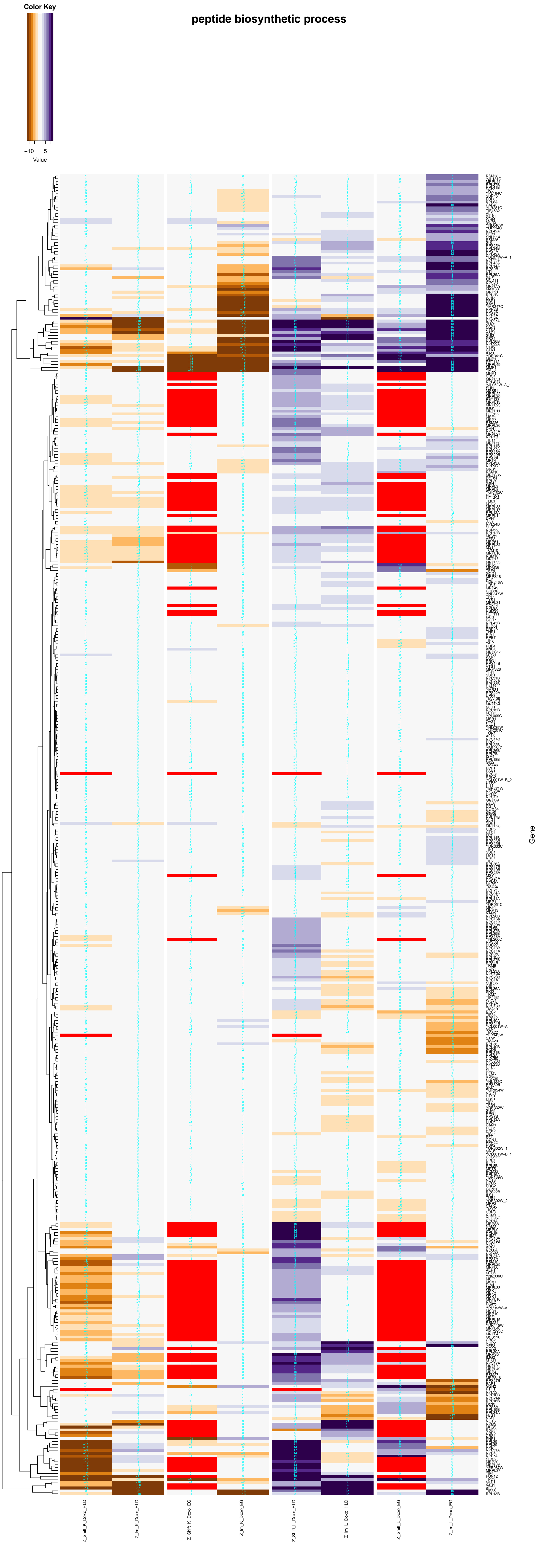

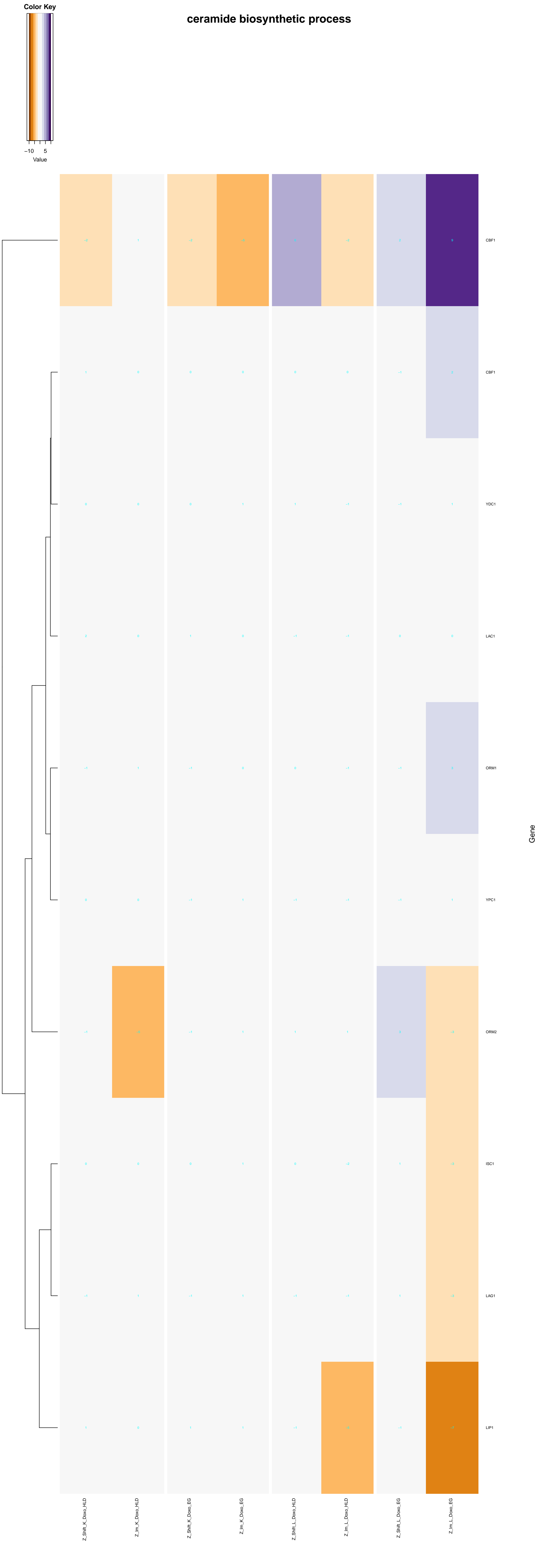

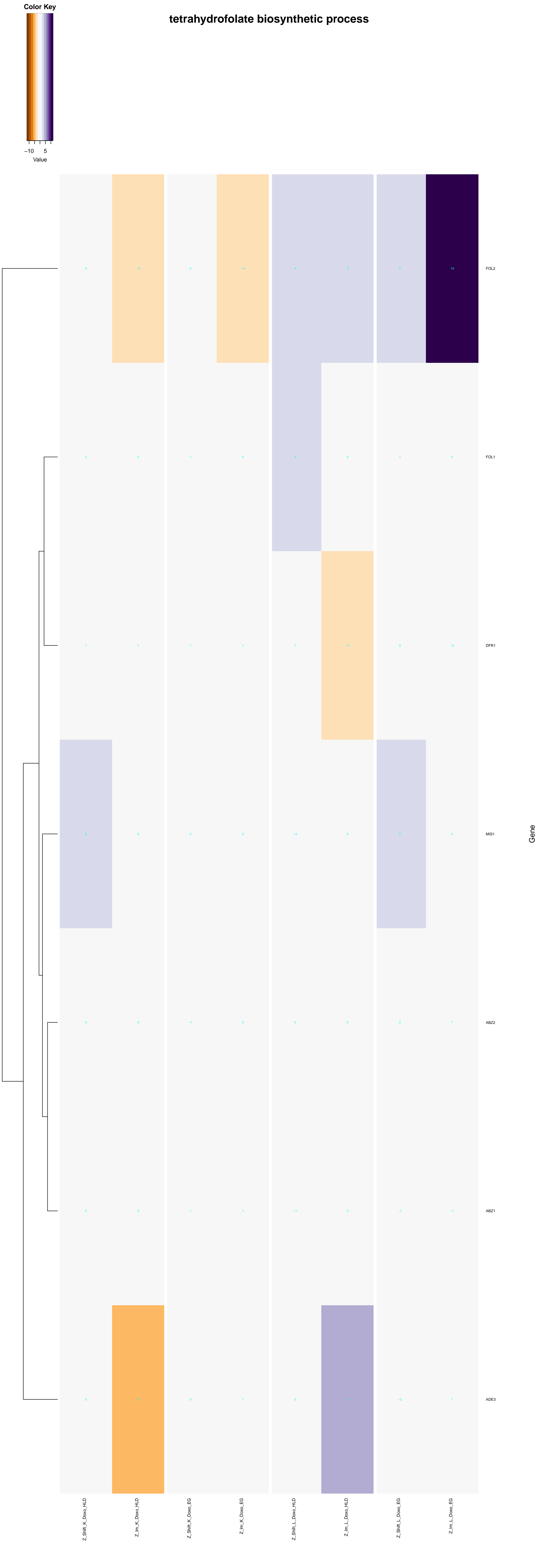

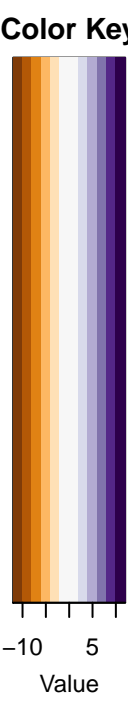

folic acid biosynthetic process

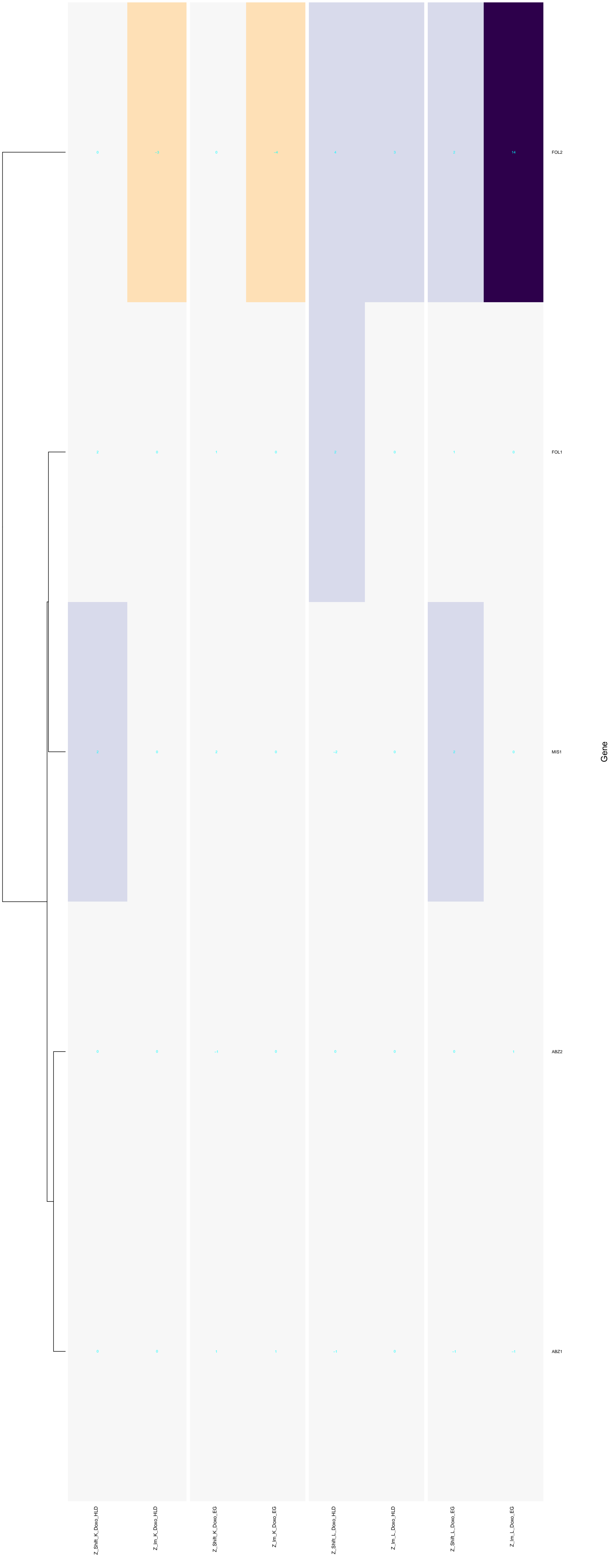

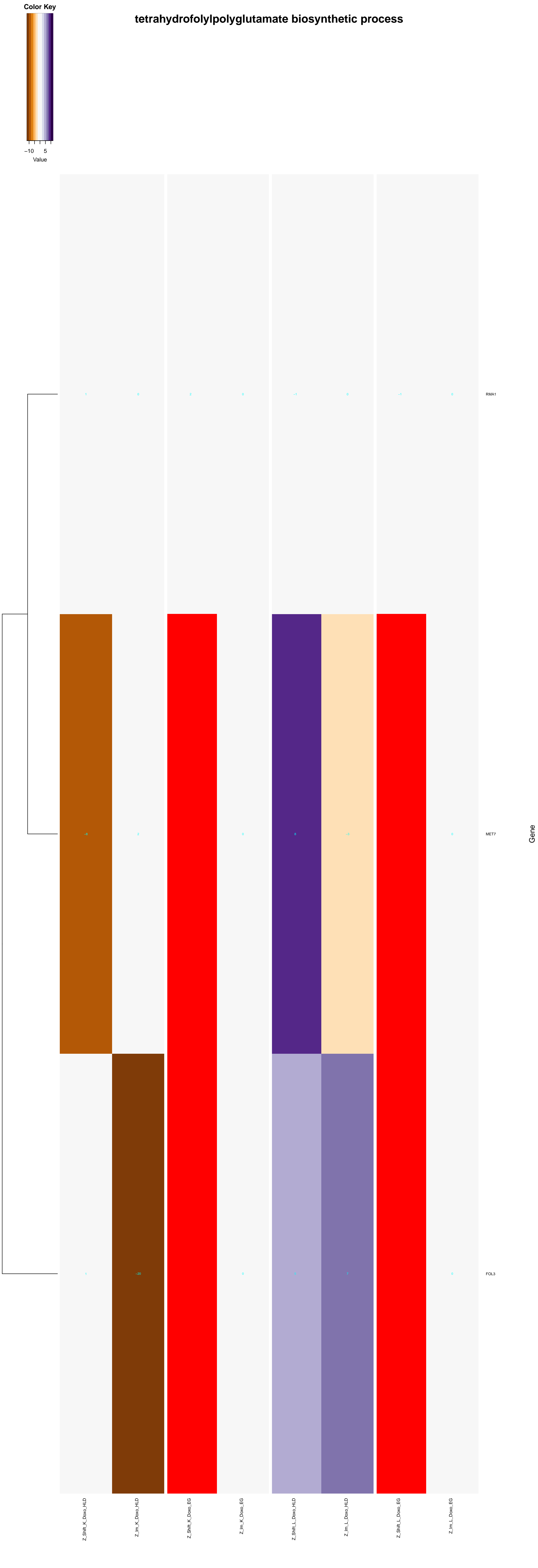

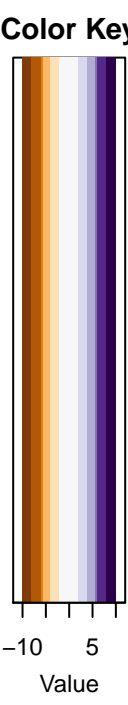

pantothenate biosynthetic process from valine

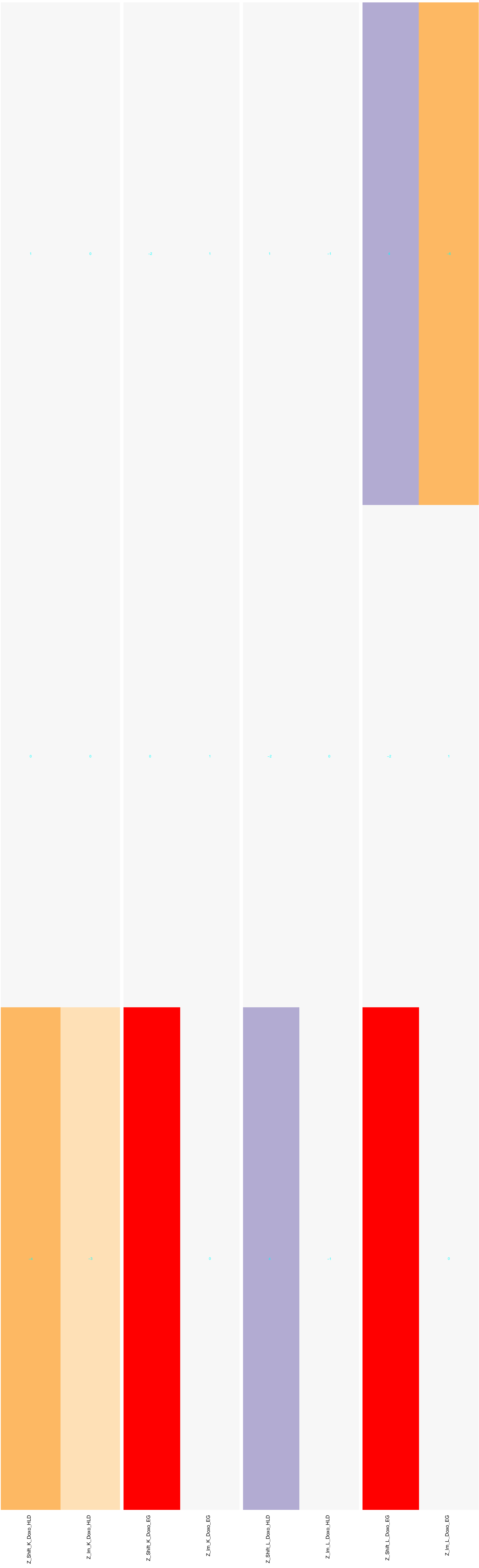

Gene



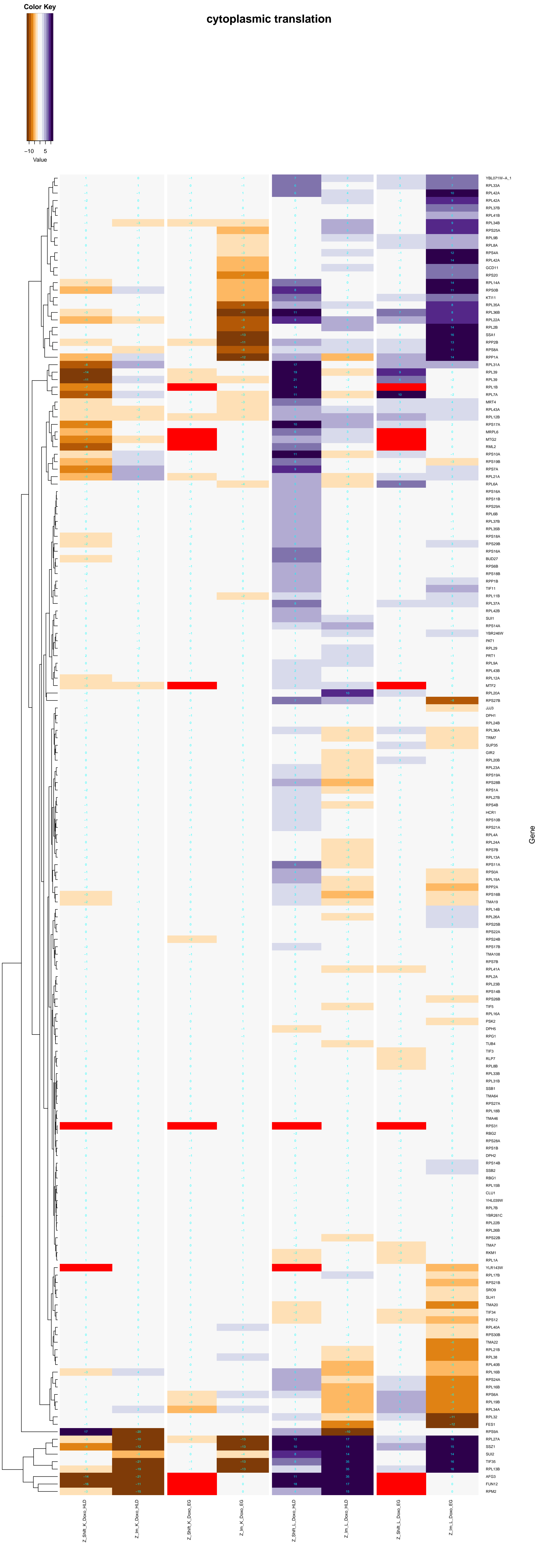

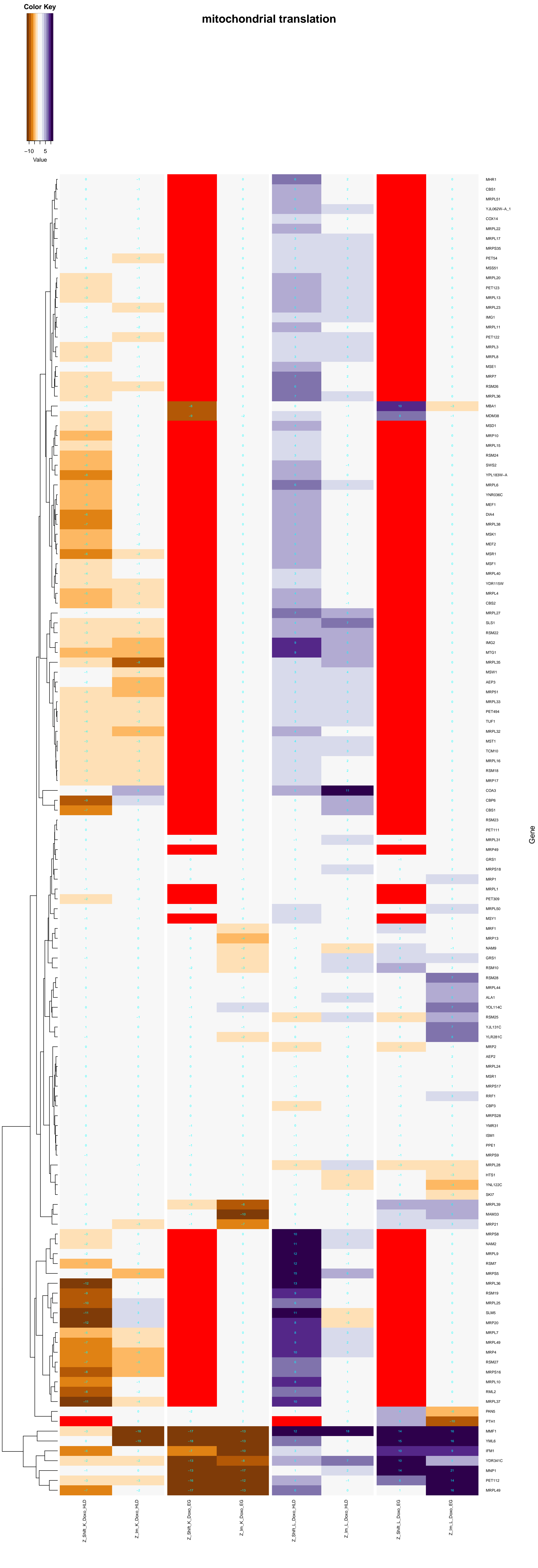

Supplement: Supplementary file 9 — Additional file 9. GO term-specific heatmaps for REMc/GTF-enriched clusters. GO term-specific heatmaps for significant GO process terms were generated as described in methods and Figs. 3 and 4. Any related child terms are presented in subsequent pages of the parent file name. GO terms with more than 100 children, with 2 or fewer genes annotated to the term, or a file size over 300KB are not shown. All heatmaps are generated with the same layout (see Figs. 3 and 4). [file 40170_2019_201_MOESM9_ESM.bz2 › Additional_File9_GOTermHeatmaps/Additional_File9_GOTermHeatmap/amide_biosynthetic_process.pdf]

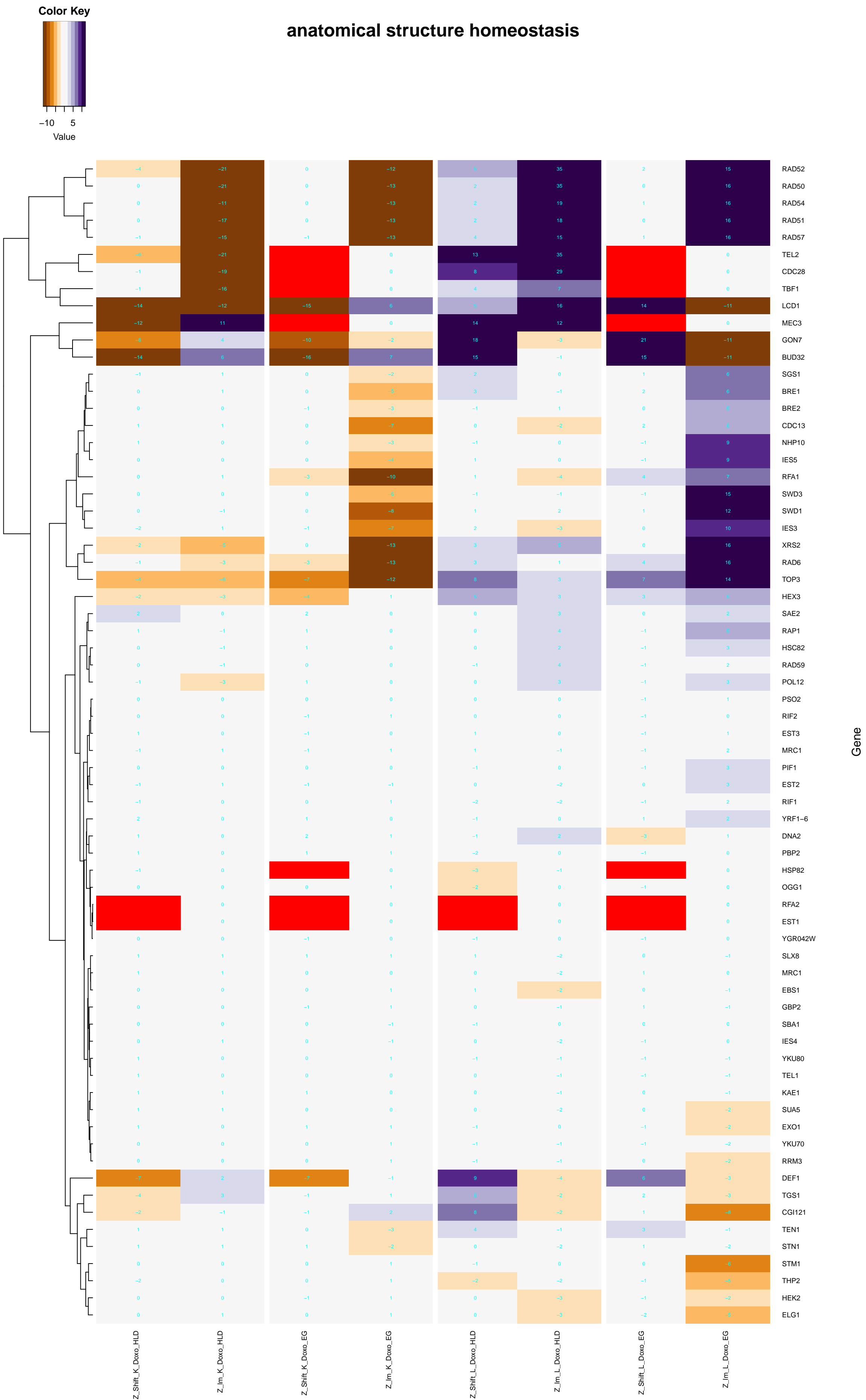

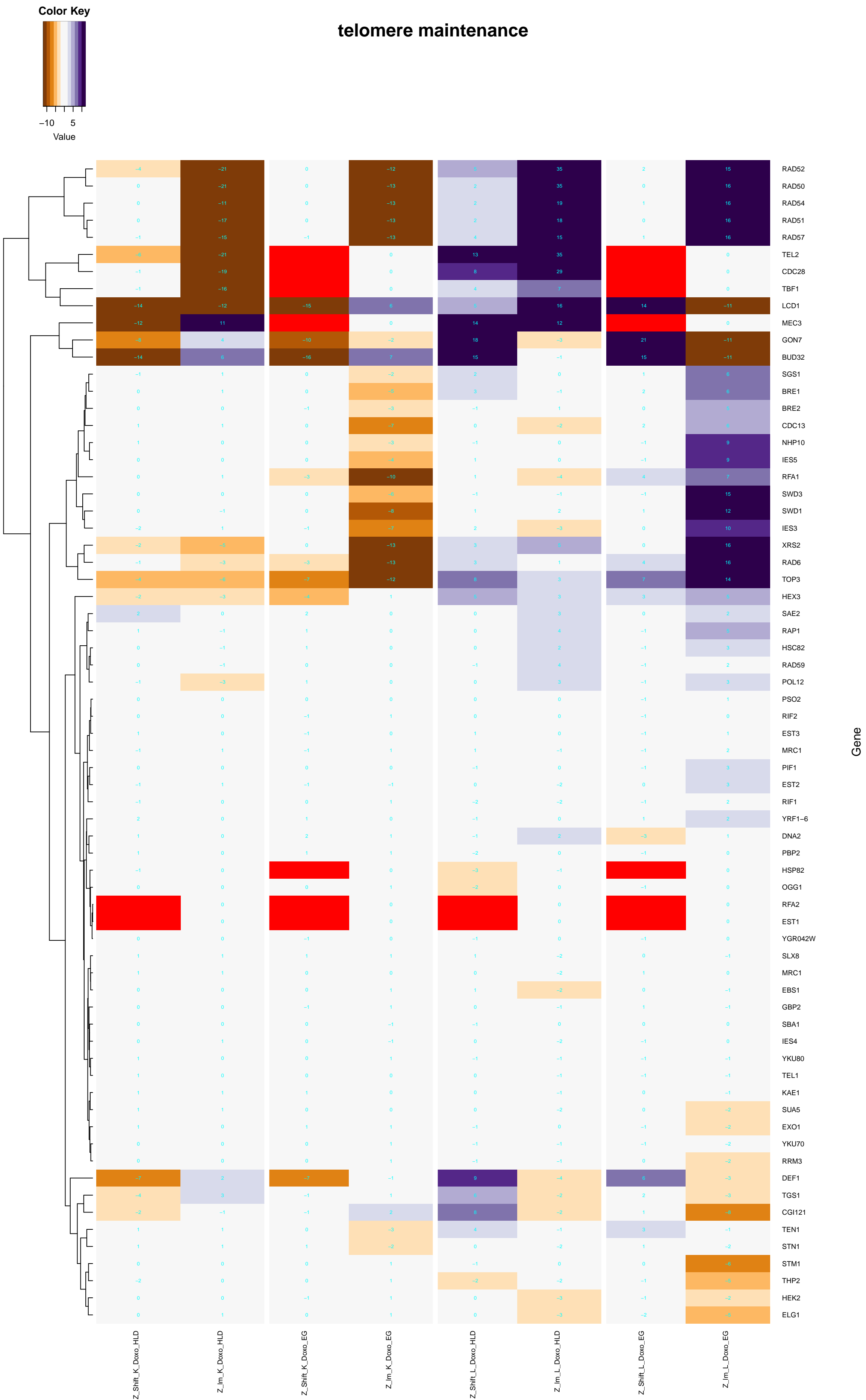

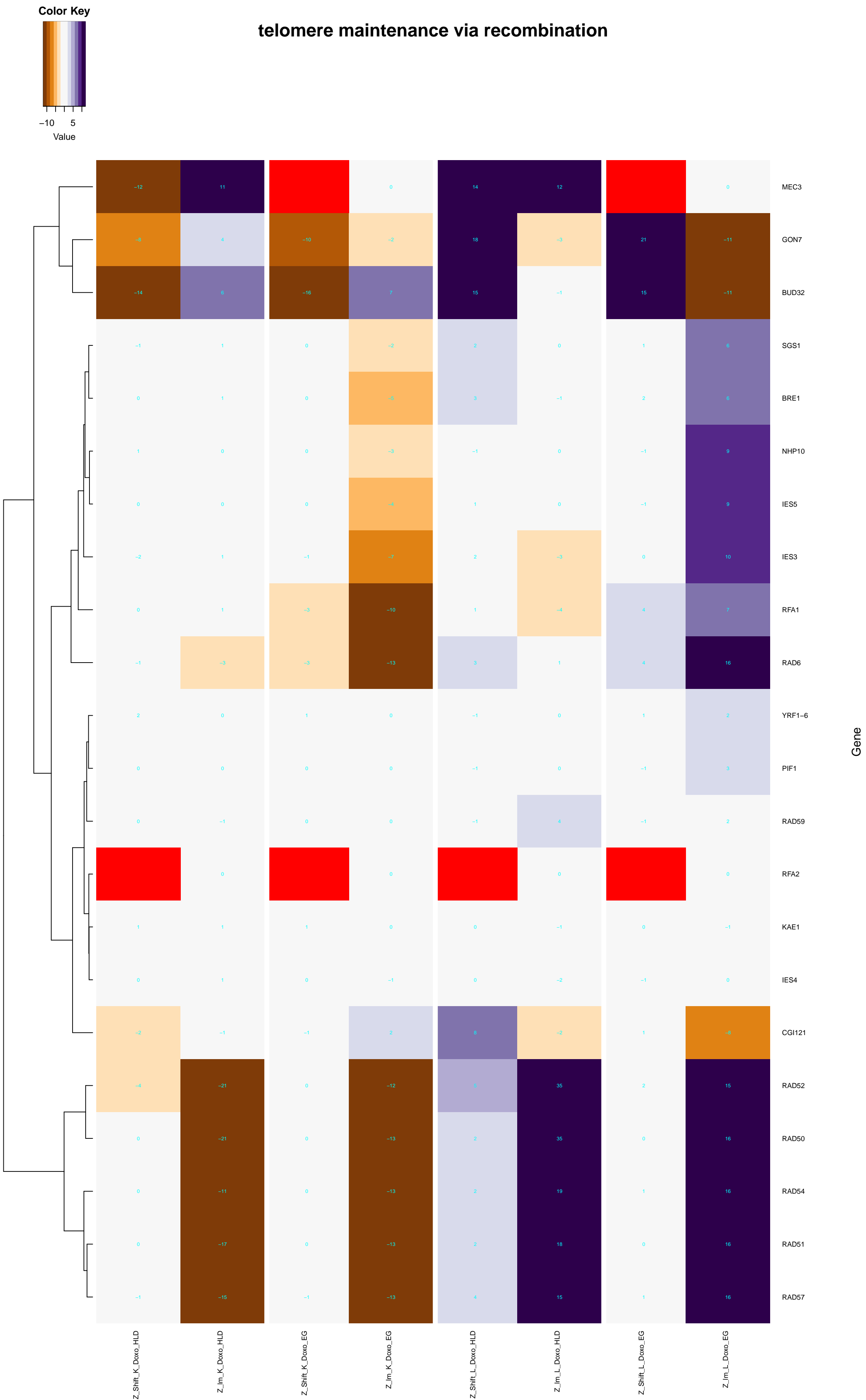

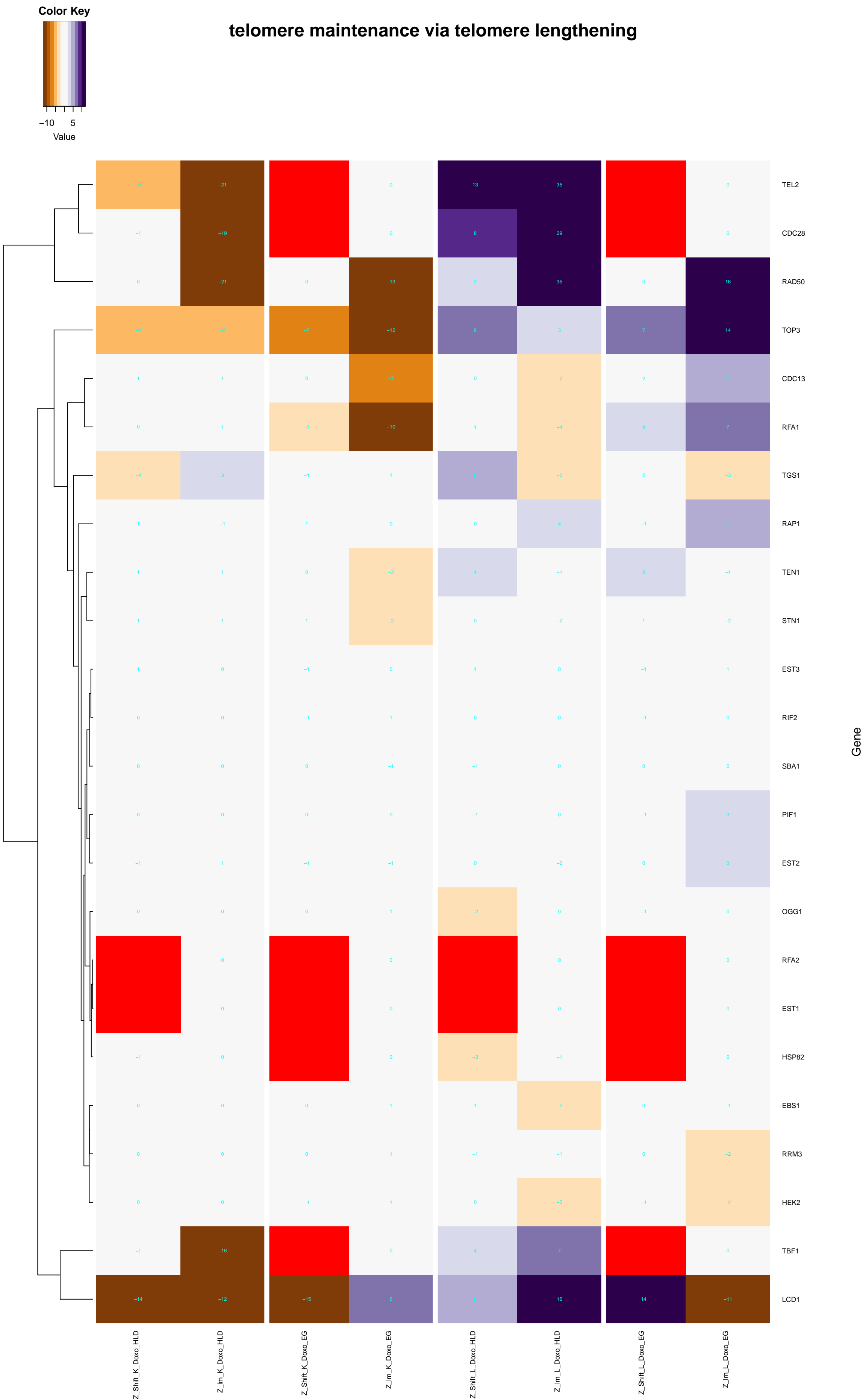

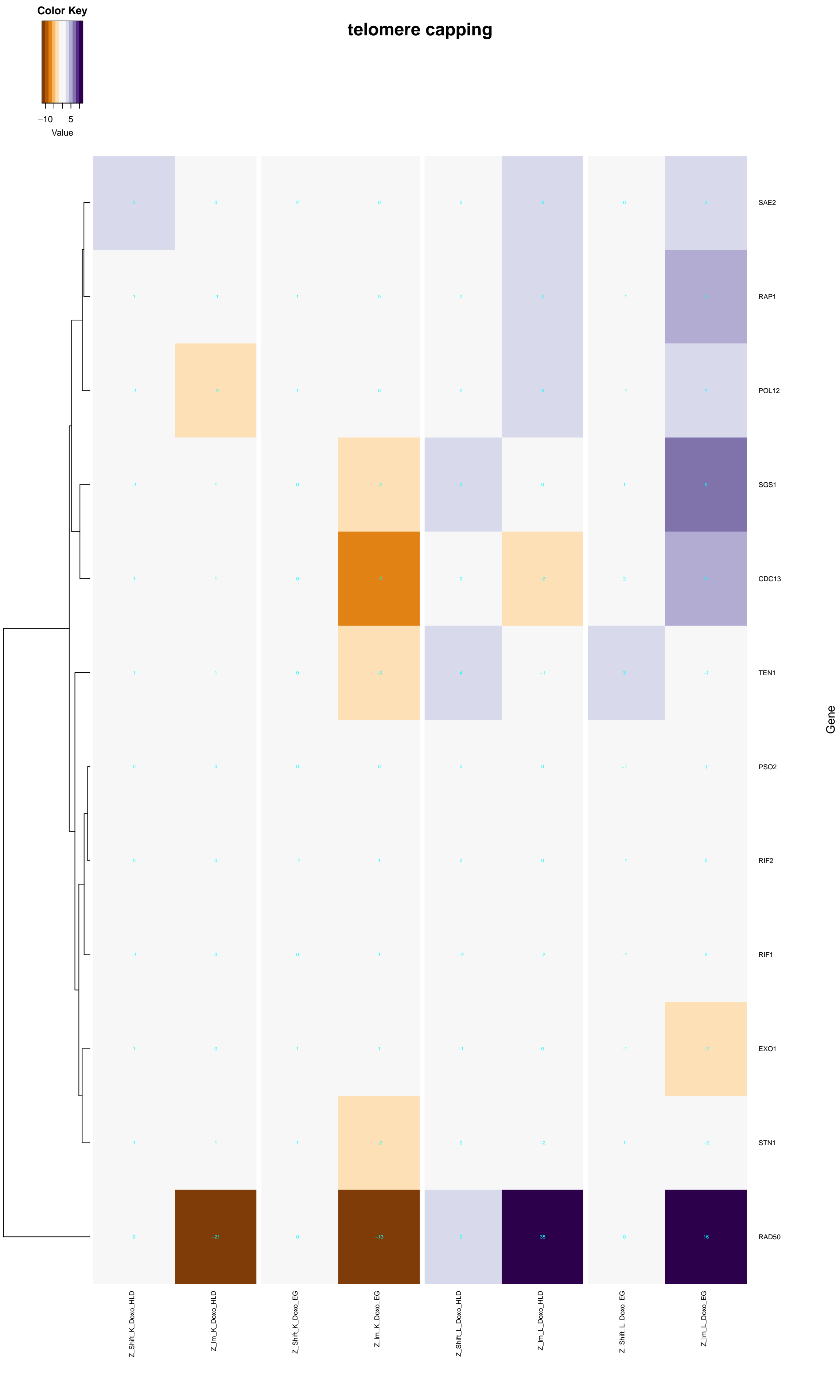

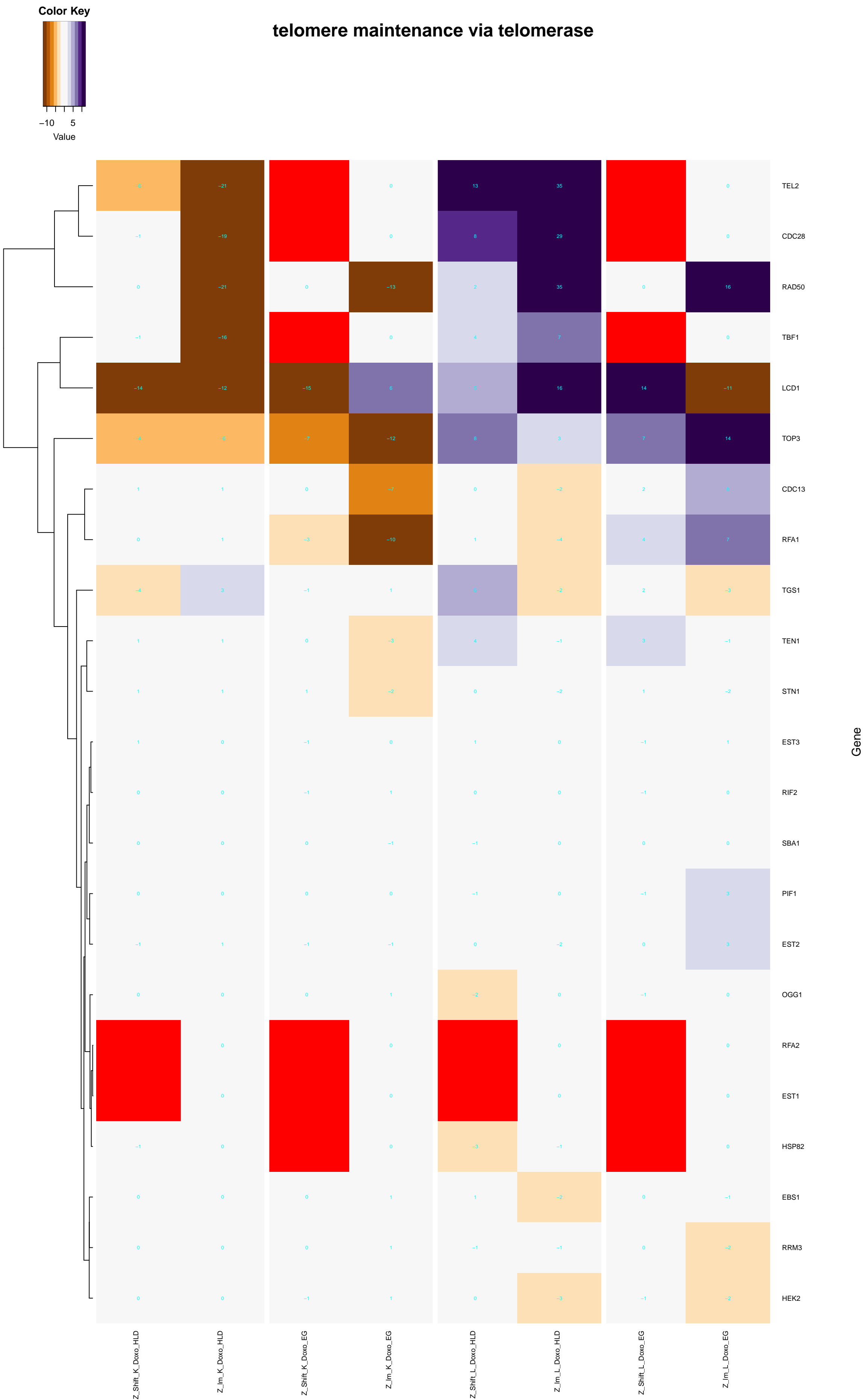

Supplement: Supplementary file 9 — Additional file 9. GO term-specific heatmaps for REMc/GTF-enriched clusters. GO term-specific heatmaps for significant GO process terms were generated as described in methods and Figs. 3 and 4. Any related child terms are presented in subsequent pages of the parent file name. GO terms with more than 100 children, with 2 or fewer genes annotated to the term, or a file size over 300KB are not shown. All heatmaps are generated with the same layout (see Figs. 3 and 4). [file 40170_2019_201_MOESM9_ESM.bz2 › Additional_File9_GOTermHeatmaps/Additional_File9_GOTermHeatmap/anatomical_structure_homeostasis.pdf]

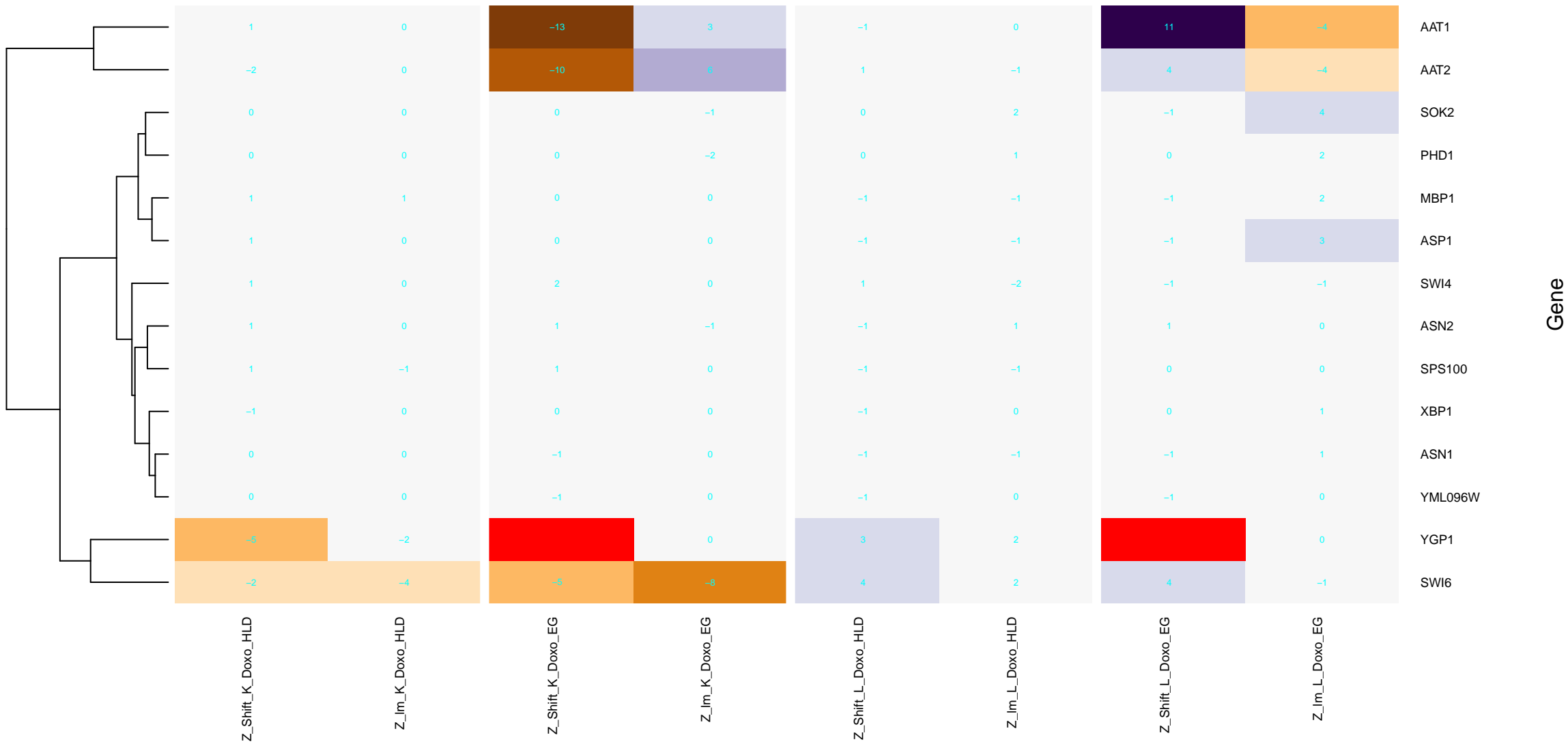

Supplement: Supplementary file 9 — Additional file 9. GO term-specific heatmaps for REMc/GTF-enriched clusters. GO term-specific heatmaps for significant GO process terms were generated as described in methods and Figs. 3 and 4. Any related child terms are presented in subsequent pages of the parent file name. GO terms with more than 100 children, with 2 or fewer genes annotated to the term, or a file size over 300KB are not shown. All heatmaps are generated with the same layout (see Figs. 3 and 4). [file 40170_2019_201_MOESM9_ESM.bz2 › Additional_File9_GOTermHeatmaps/Additional_File9_GOTermHeatmap/asparagine_metabolic_process.pdf]

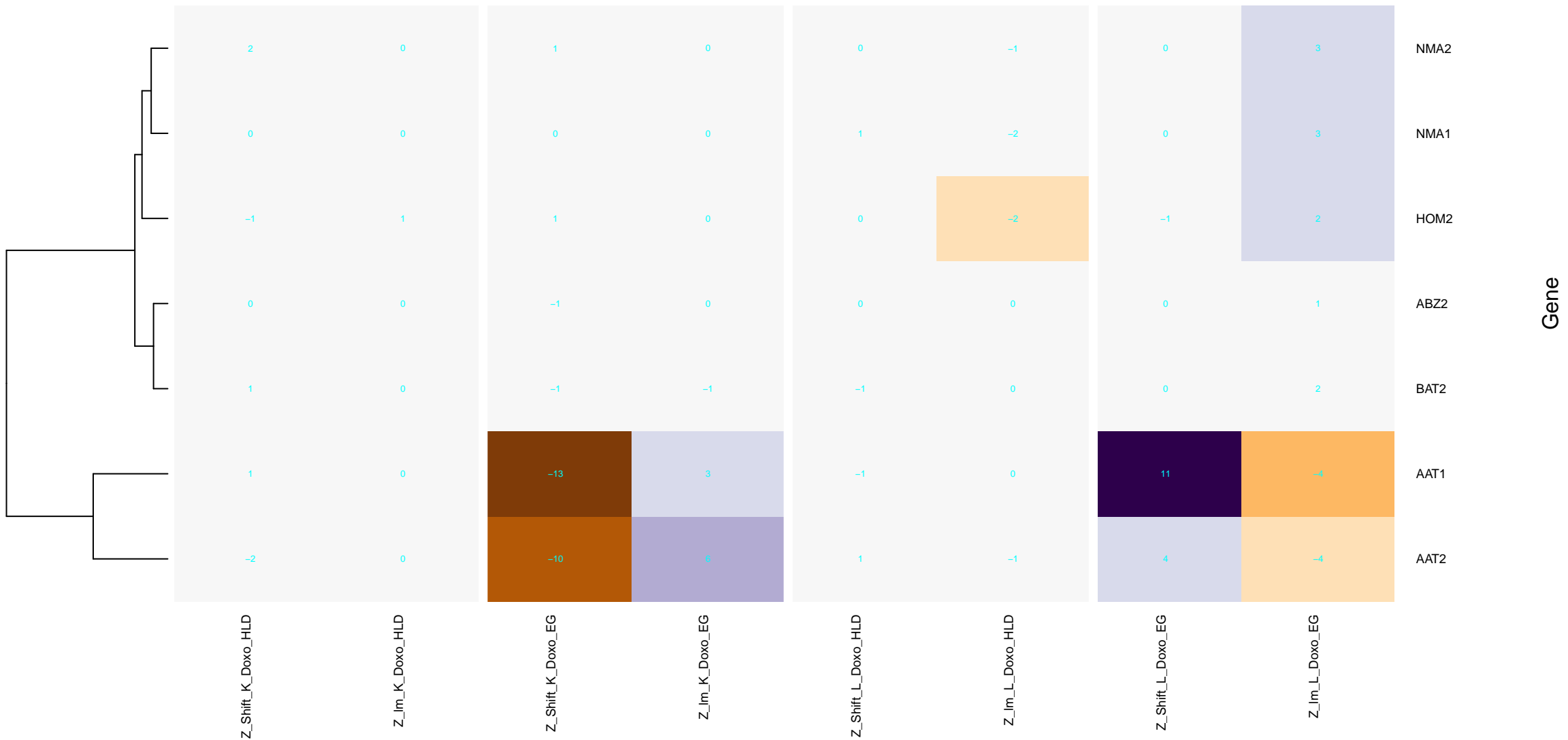

Supplement: Supplementary file 9 — Additional file 9. GO term-specific heatmaps for REMc/GTF-enriched clusters. GO term-specific heatmaps for significant GO process terms were generated as described in methods and Figs. 3 and 4. Any related child terms are presented in subsequent pages of the parent file name. GO terms with more than 100 children, with 2 or fewer genes annotated to the term, or a file size over 300KB are not shown. All heatmaps are generated with the same layout (see Figs. 3 and 4). [file 40170_2019_201_MOESM9_ESM.bz2 › Additional_File9_GOTermHeatmaps/Additional_File9_GOTermHeatmap/aspartate_metabolic_process.pdf]

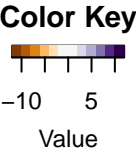

# ATP-dependent chromatin remodeling

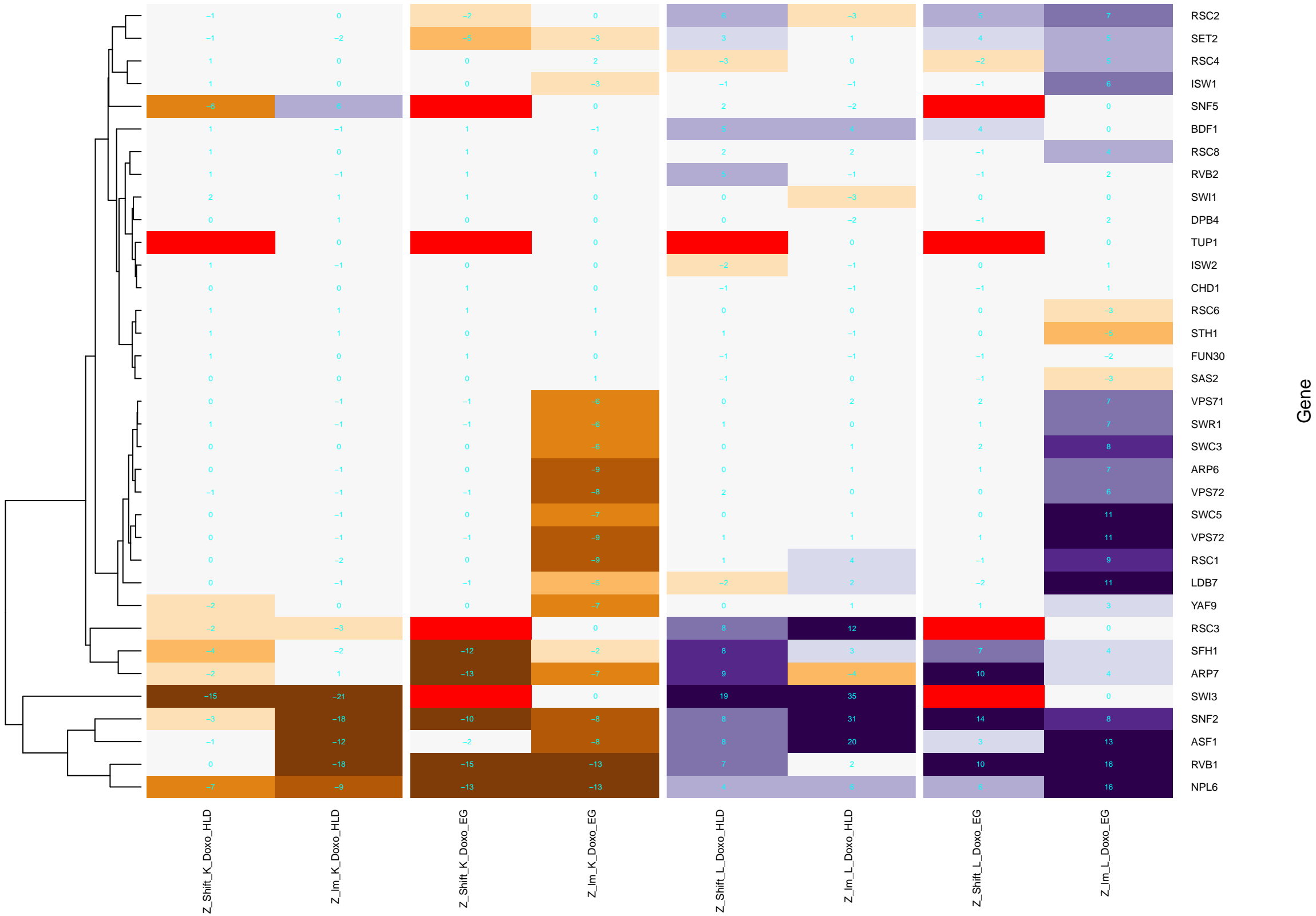

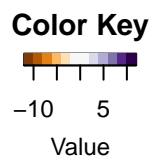

# histone exchange

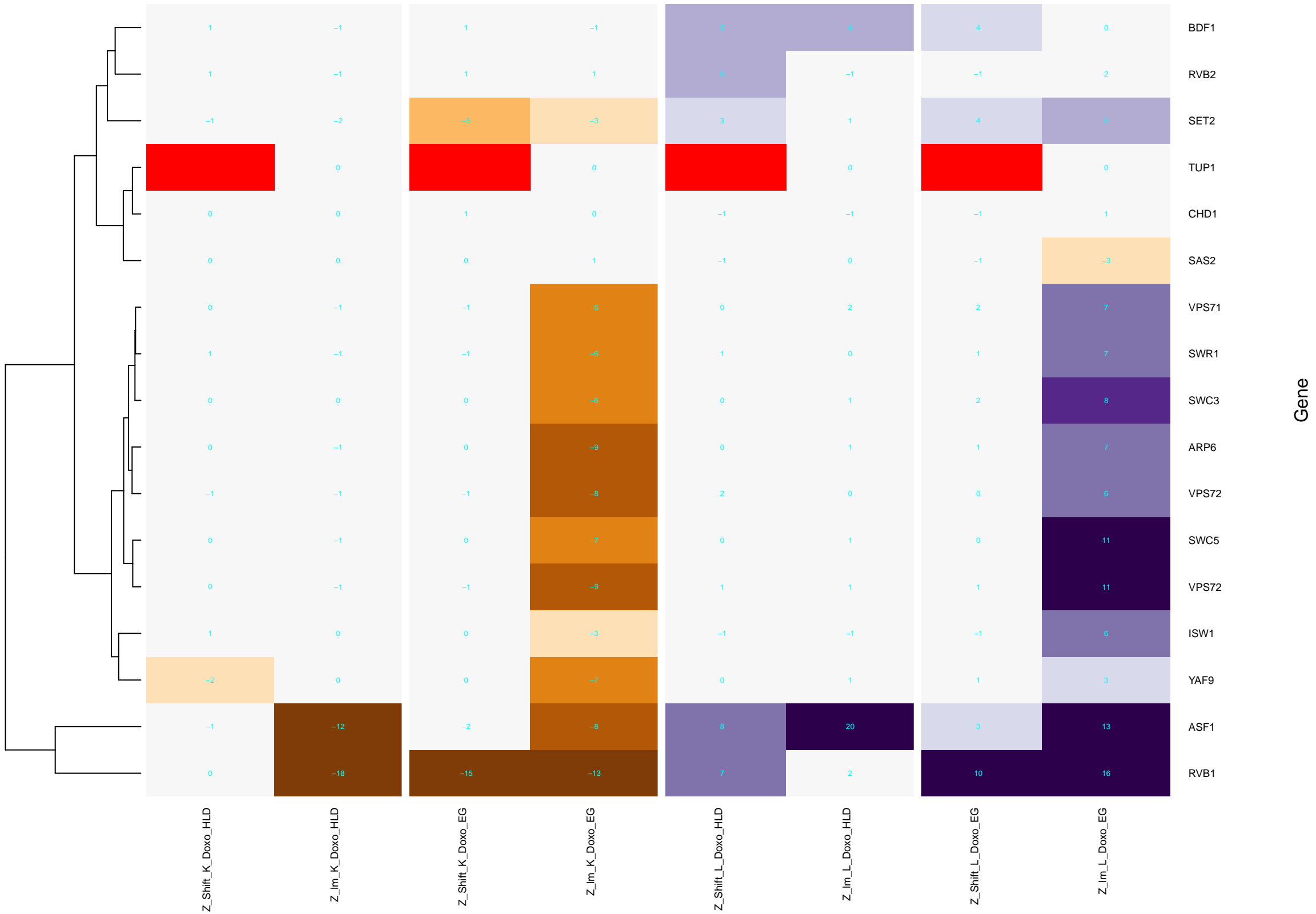

Supplement: Supplementary file 9 — Additional file 9. GO term-specific heatmaps for REMc/GTF-enriched clusters. GO term-specific heatmaps for significant GO process terms were generated as described in methods and Figs. 3 and 4. Any related child terms are presented in subsequent pages of the parent file name. GO terms with more than 100 children, with 2 or fewer genes annotated to the term, or a file size over 300KB are not shown. All heatmaps are generated with the same layout (see Figs. 3 and 4). [file 40170_2019_201_MOESM9_ESM.bz2 › Additional_File9_GOTermHeatmaps/Additional_File9_GOTermHeatmap/ATP-dependent_chromatin_remodeling.pdf]

## ATP synthesis coupled proton transport

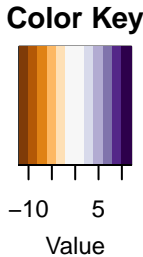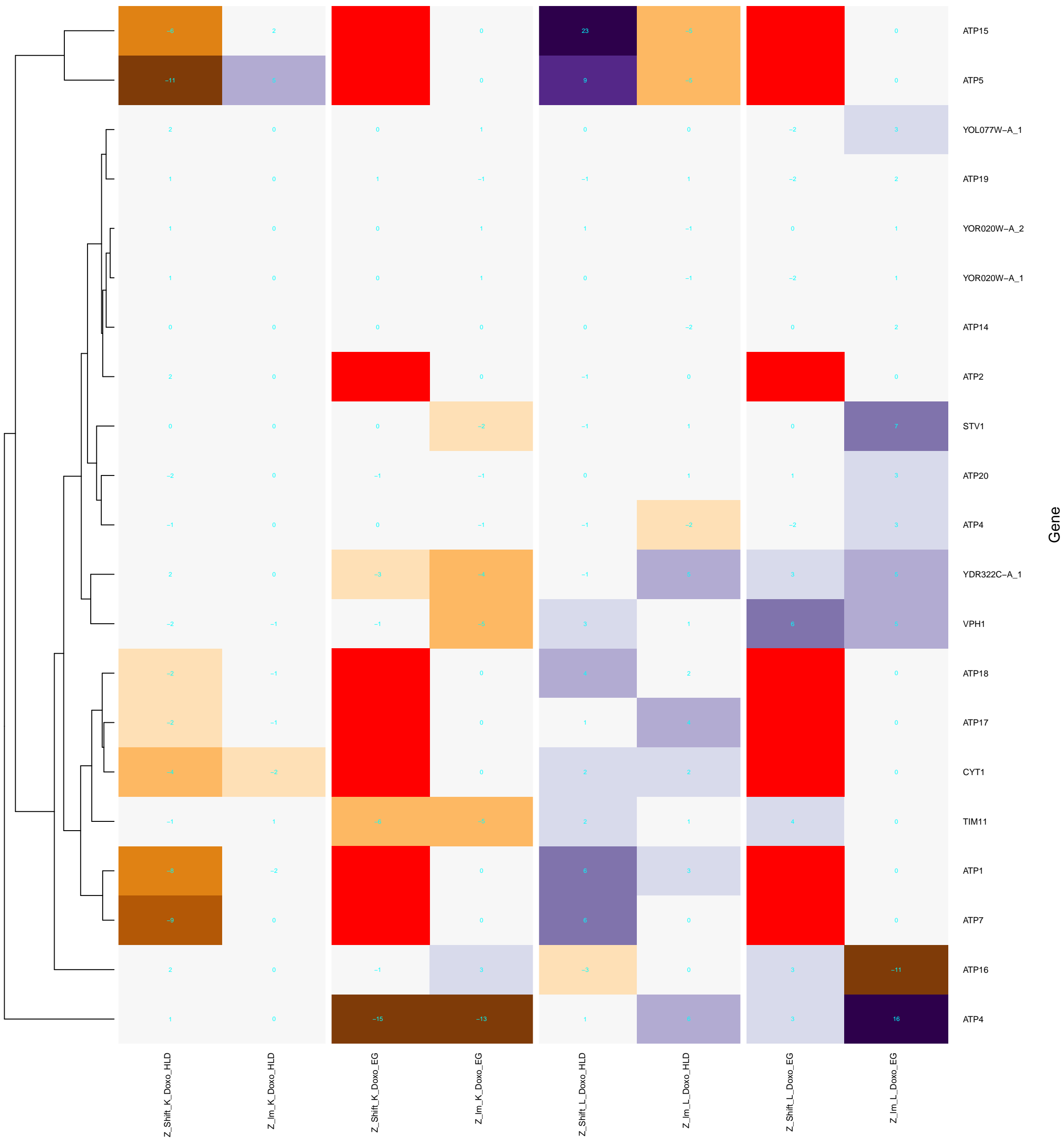

Supplement: Supplementary file 9 — Additional file 9. GO term-specific heatmaps for REMc/GTF-enriched clusters. GO term-specific heatmaps for significant GO process terms were generated as described in methods and Figs. 3 and 4. Any related child terms are presented in subsequent pages of the parent file name. GO terms with more than 100 children, with 2 or fewer genes annotated to the term, or a file size over 300KB are not shown. All heatmaps are generated with the same layout (see Figs. 3 and 4). [file 40170_2019_201_MOESM9_ESM.bz2 › Additional_File9_GOTermHeatmaps/Additional_File9_GOTermHeatmap/ATP_biosynthetic_process.pdf]

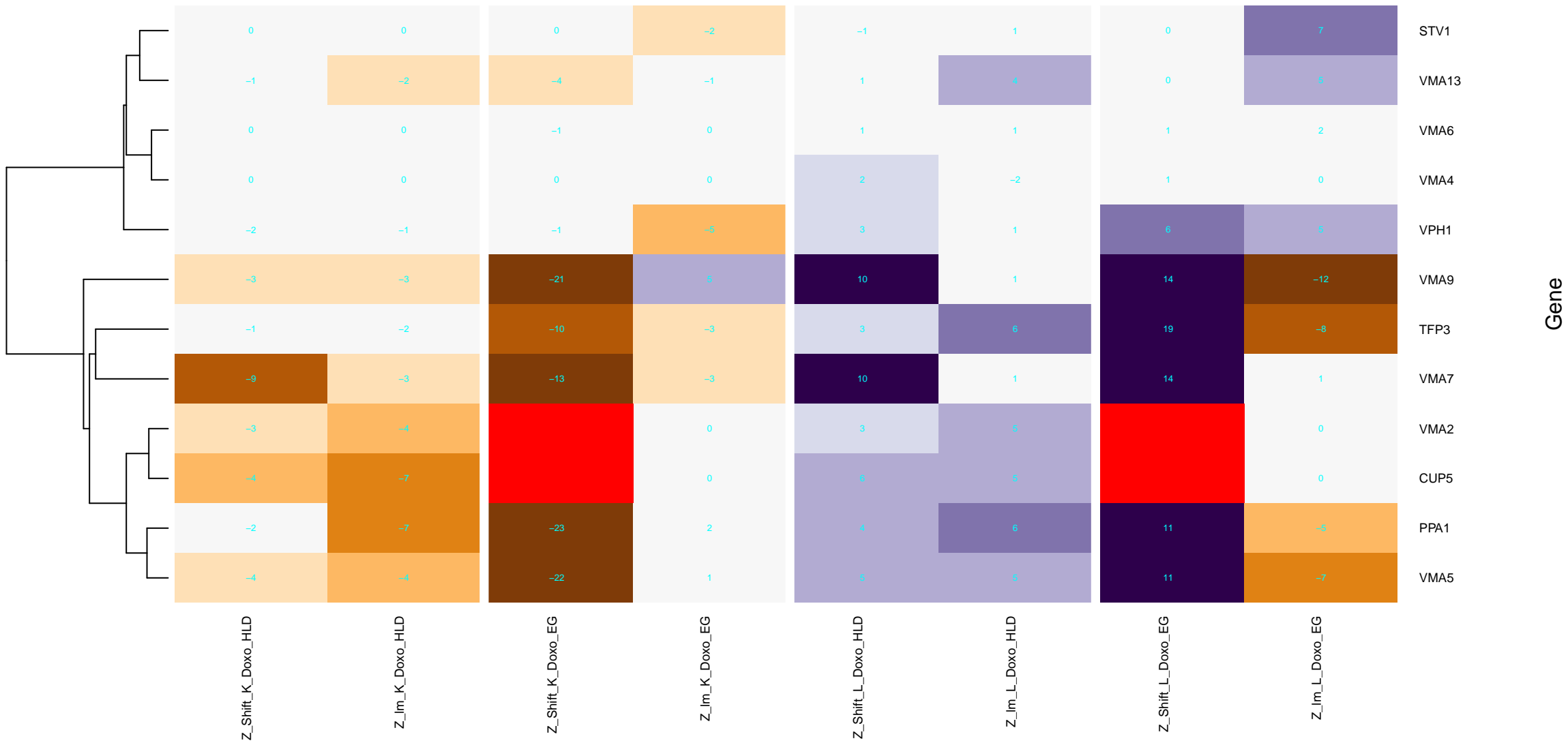

Supplement: Supplementary file 9 — Additional file 9. GO term-specific heatmaps for REMc/GTF-enriched clusters. GO term-specific heatmaps for significant GO process terms were generated as described in methods and Figs. 3 and 4. Any related child terms are presented in subsequent pages of the parent file name. GO terms with more than 100 children, with 2 or fewer genes annotated to the term, or a file size over 300KB are not shown. All heatmaps are generated with the same layout (see Figs. 3 and 4). [file 40170_2019_201_MOESM9_ESM.bz2 › Additional_File9_GOTermHeatmaps/Additional_File9_GOTermHeatmap/ATP_hydrolysis_coupled_proton_transport.pdf]

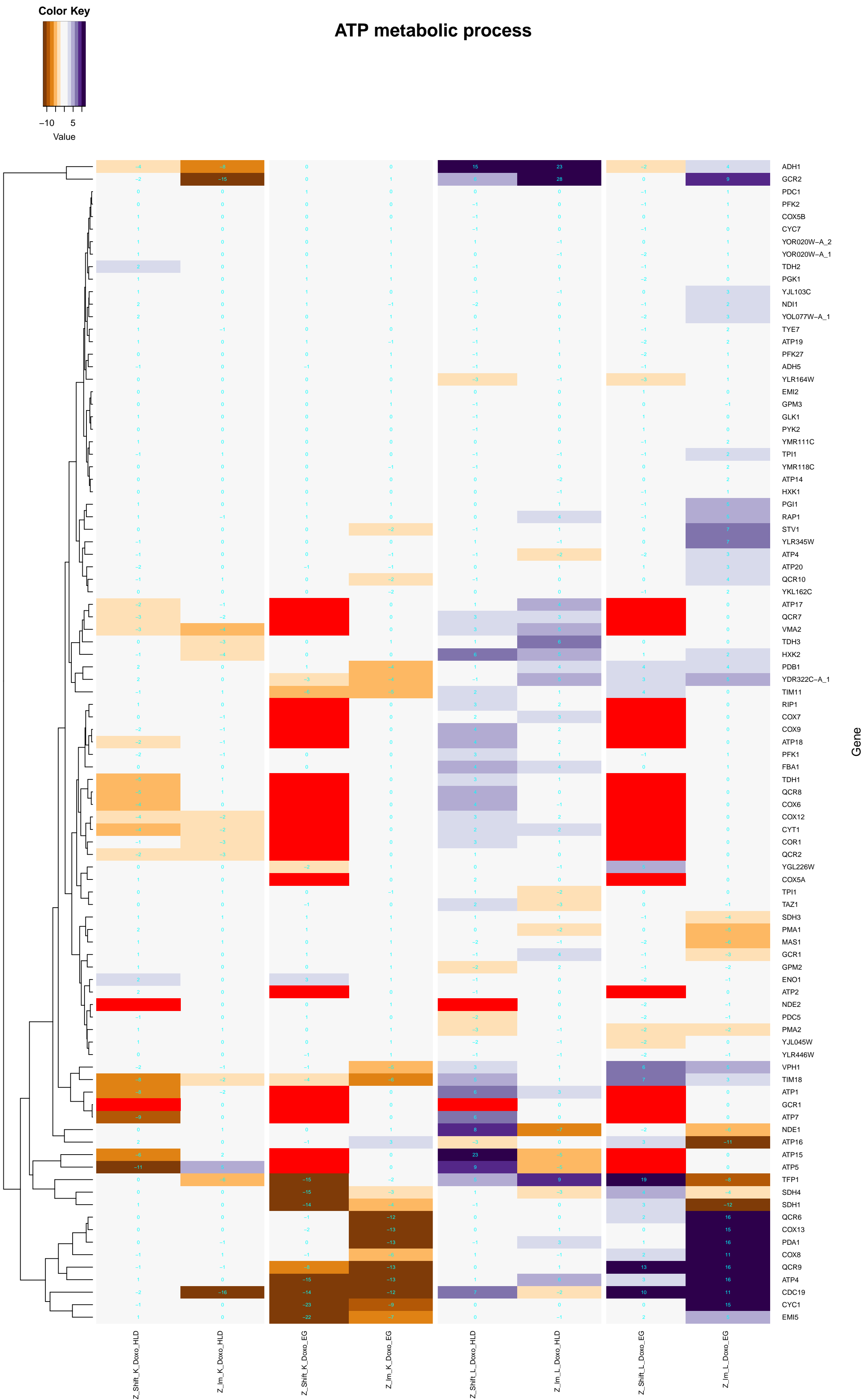

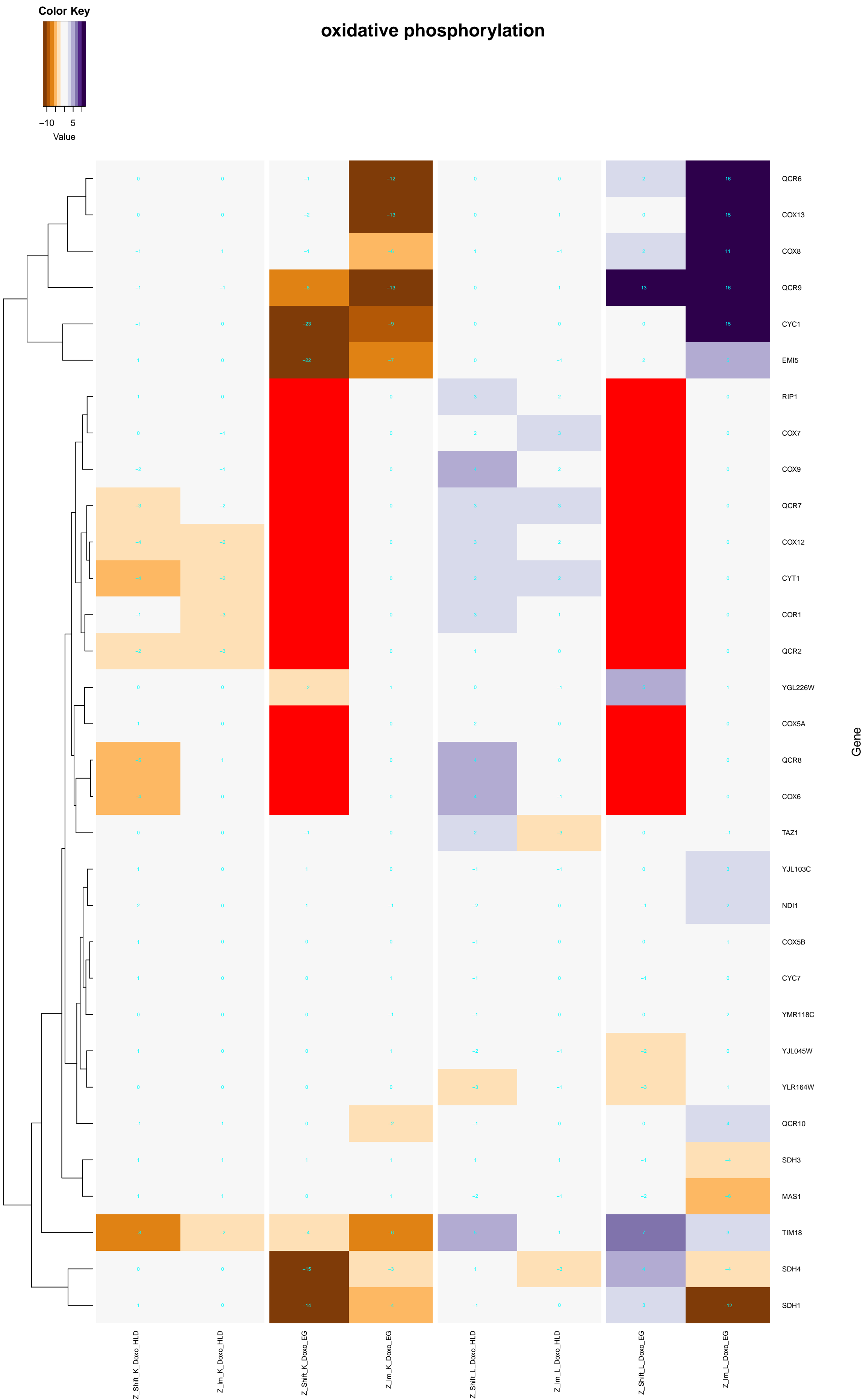

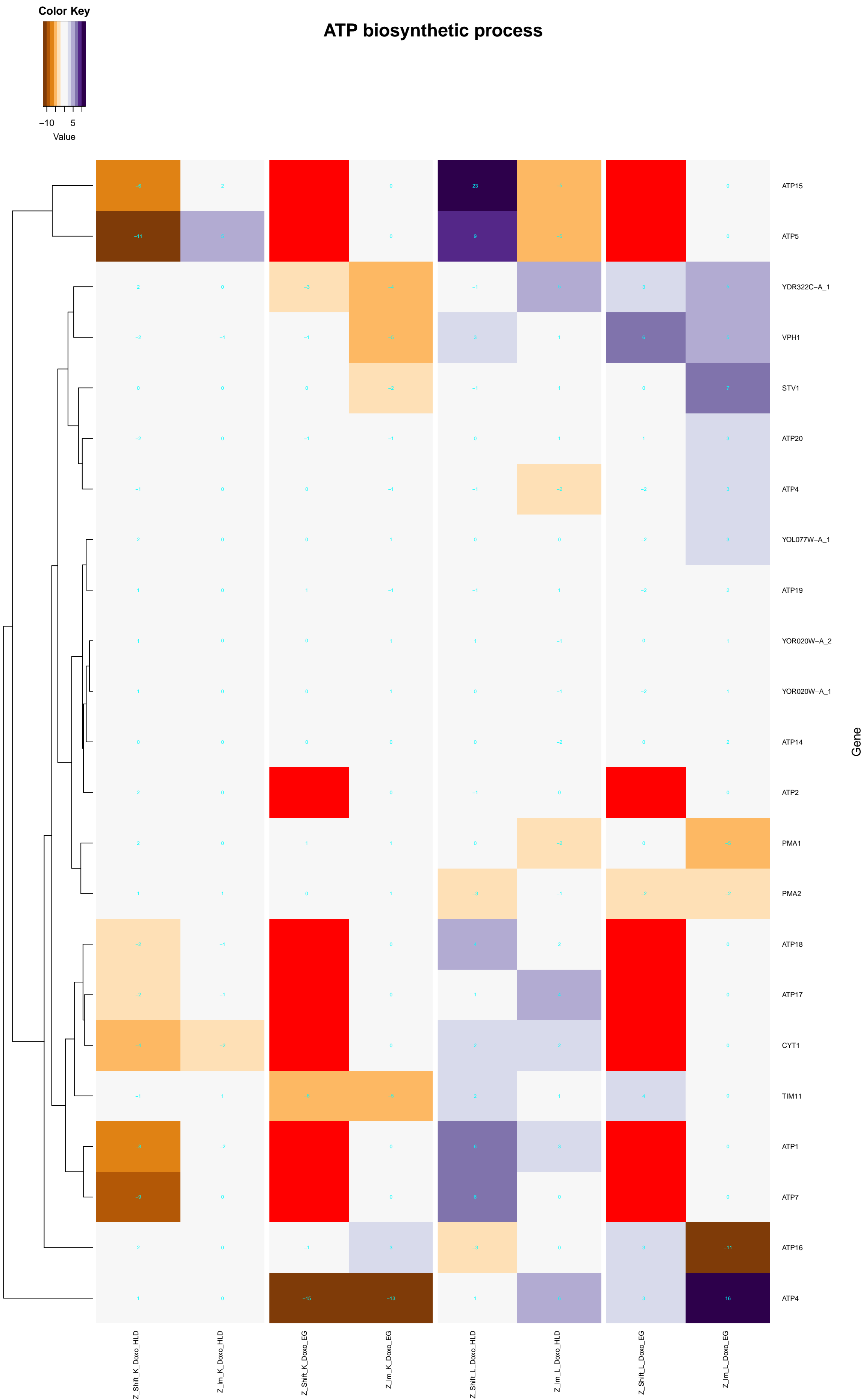

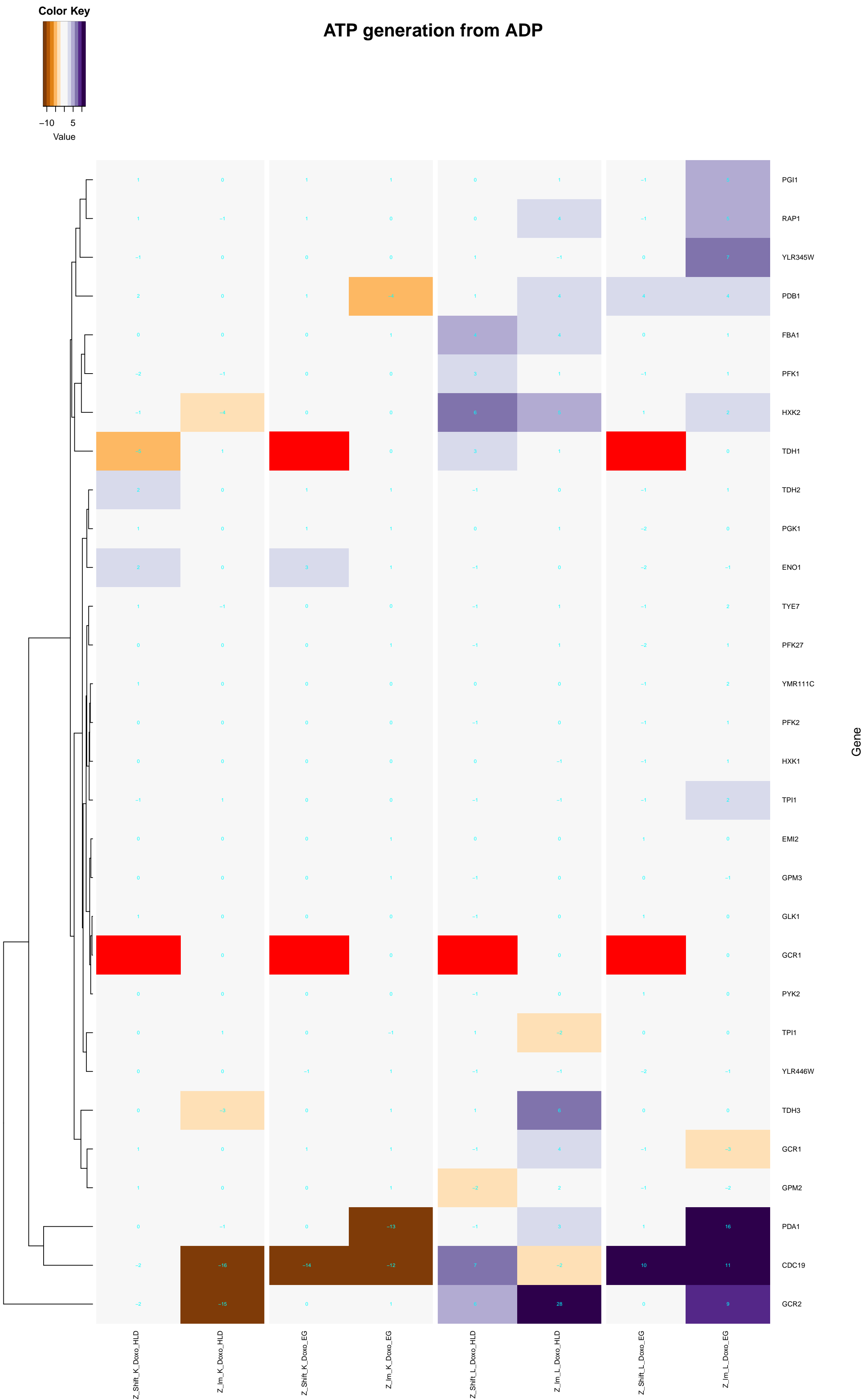

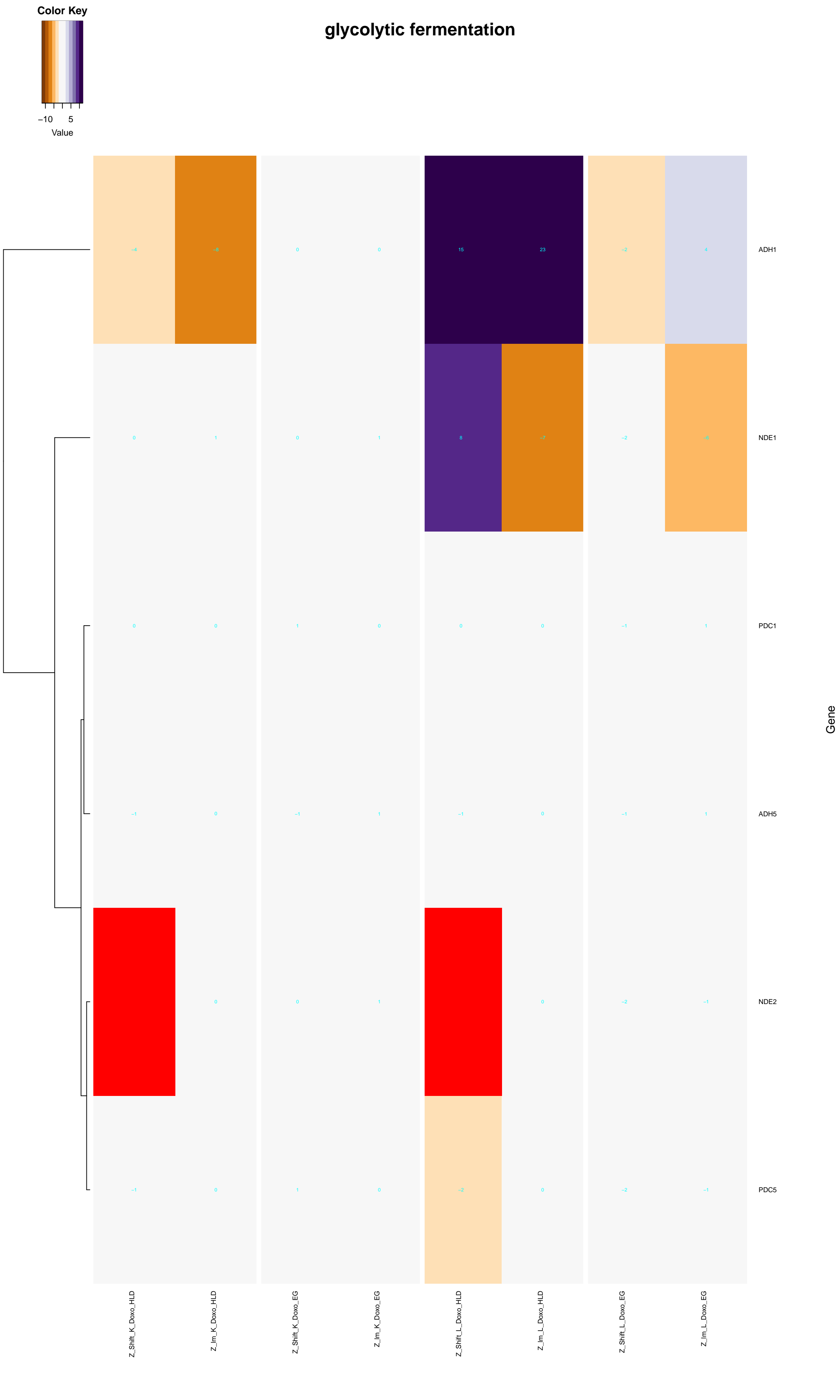

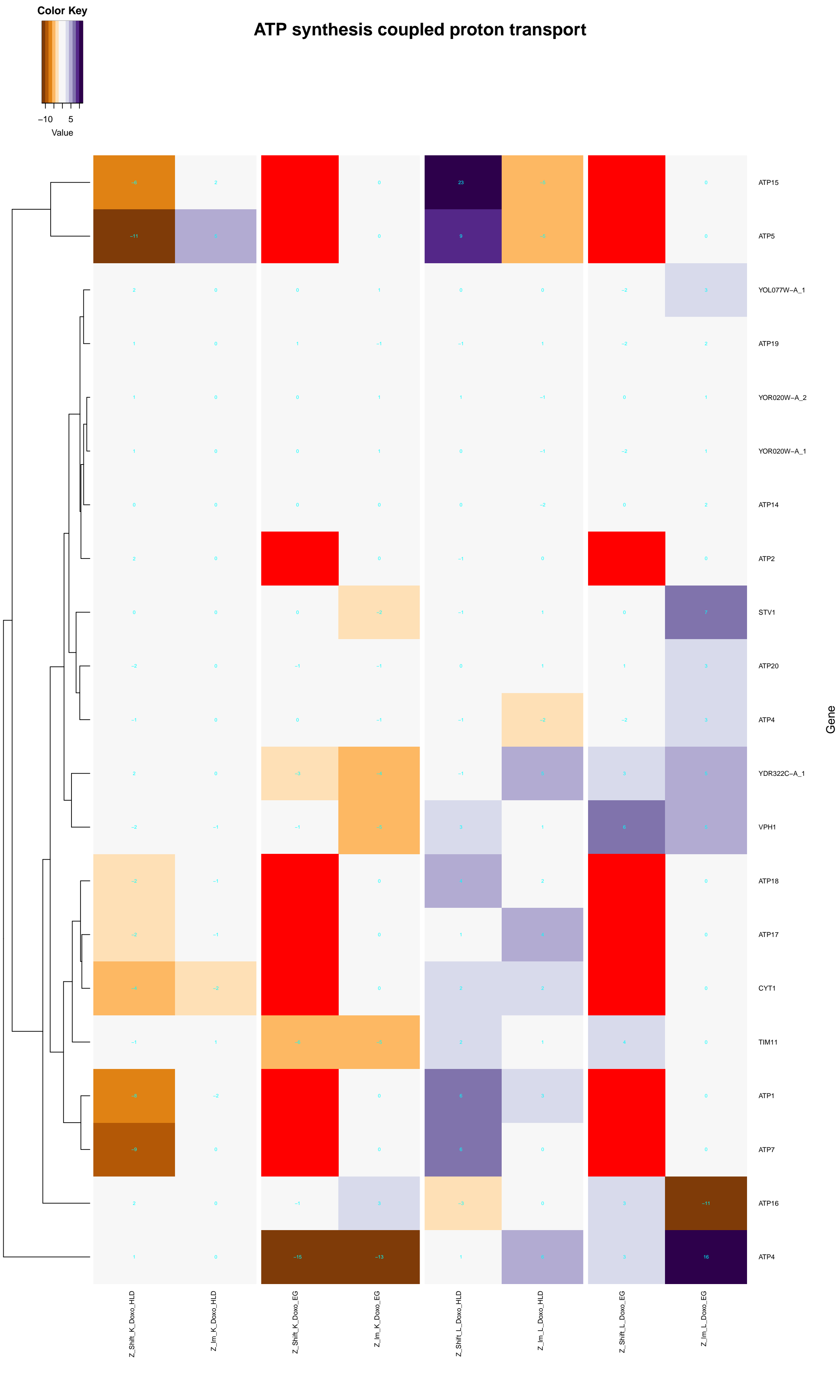

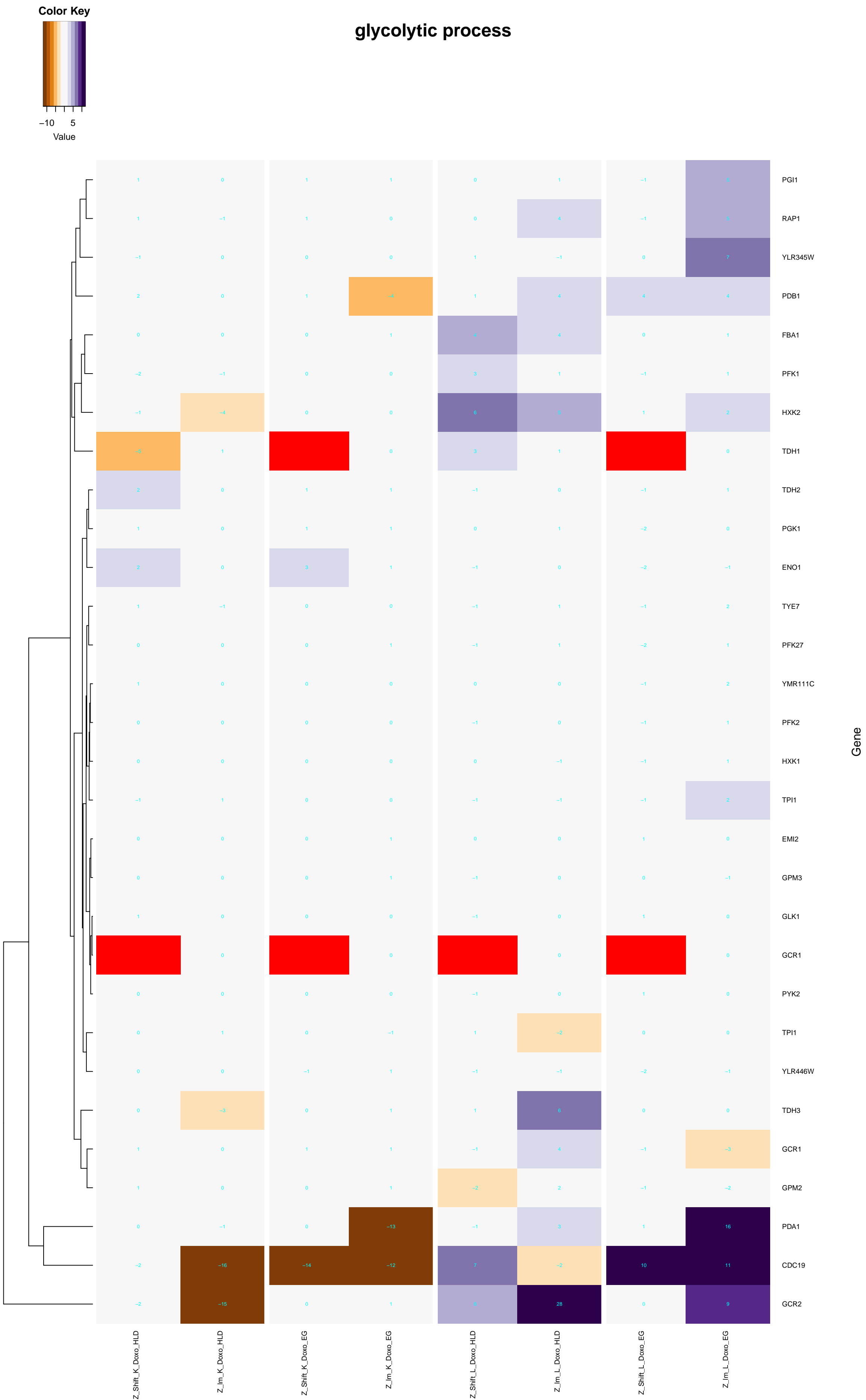

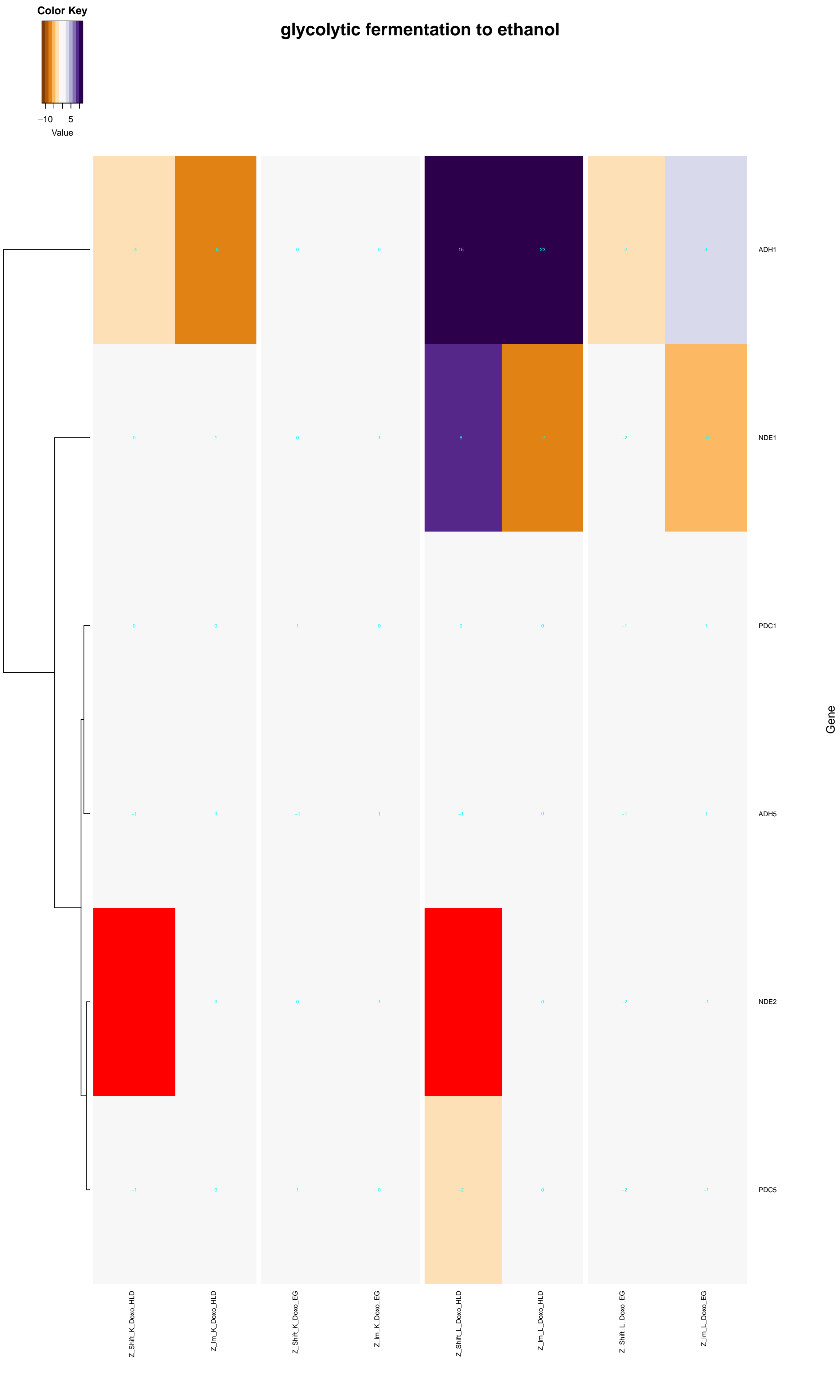

Supplement: Supplementary file 9 — Additional file 9. GO term-specific heatmaps for REMc/GTF-enriched clusters. GO term-specific heatmaps for significant GO process terms were generated as described in methods and Figs. 3 and 4. Any related child terms are presented in subsequent pages of the parent file name. GO terms with more than 100 children, with 2 or fewer genes annotated to the term, or a file size over 300KB are not shown. All heatmaps are generated with the same layout (see Figs. 3 and 4). [file 40170_2019_201_MOESM9_ESM.bz2 › Additional_File9_GOTermHeatmaps/Additional_File9_GOTermHeatmap/ATP_metabolic_process.pdf]

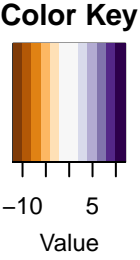

ATP synthesis coupled proton transport

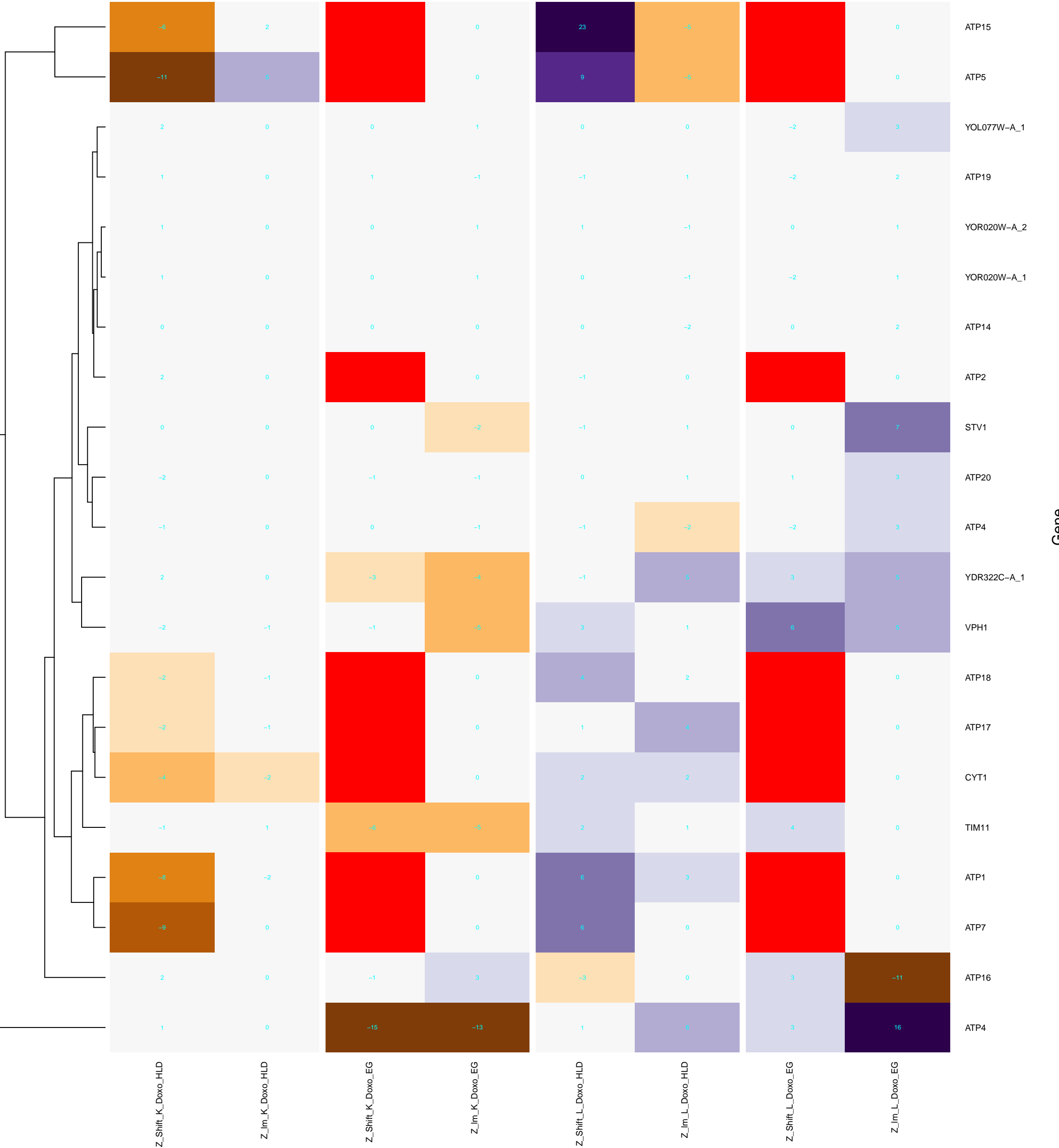

Supplement: Supplementary file 9 — Additional file 9. GO term-specific heatmaps for REMc/GTF-enriched clusters. GO term-specific heatmaps for significant GO process terms were generated as described in methods and Figs. 3 and 4. Any related child terms are presented in subsequent pages of the parent file name. GO terms with more than 100 children, with 2 or fewer genes annotated to the term, or a file size over 300KB are not shown. All heatmaps are generated with the same layout (see Figs. 3 and 4). [file 40170_2019_201_MOESM9_ESM.bz2 › Additional_File9_GOTermHeatmaps/Additional_File9_GOTermHeatmap/ATP_synthesis_coupled_proton_transport.pdf]

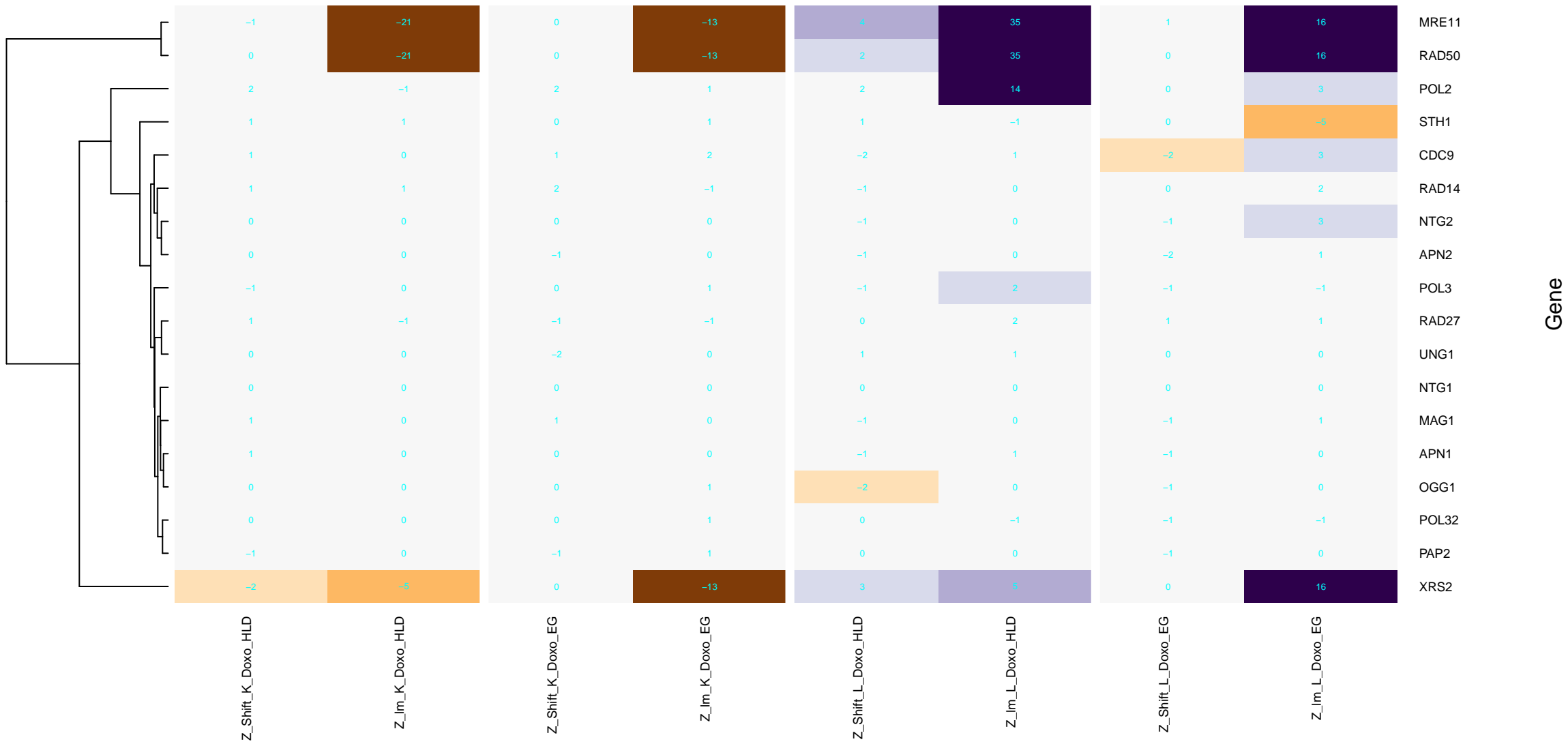

Supplement: Supplementary file 9 — Additional file 9. GO term-specific heatmaps for REMc/GTF-enriched clusters. GO term-specific heatmaps for significant GO process terms were generated as described in methods and Figs. 3 and 4. Any related child terms are presented in subsequent pages of the parent file name. GO terms with more than 100 children, with 2 or fewer genes annotated to the term, or a file size over 300KB are not shown. All heatmaps are generated with the same layout (see Figs. 3 and 4). [file 40170_2019_201_MOESM9_ESM.bz2 › Additional_File9_GOTermHeatmaps/Additional_File9_GOTermHeatmap/base-excision_repair.pdf]

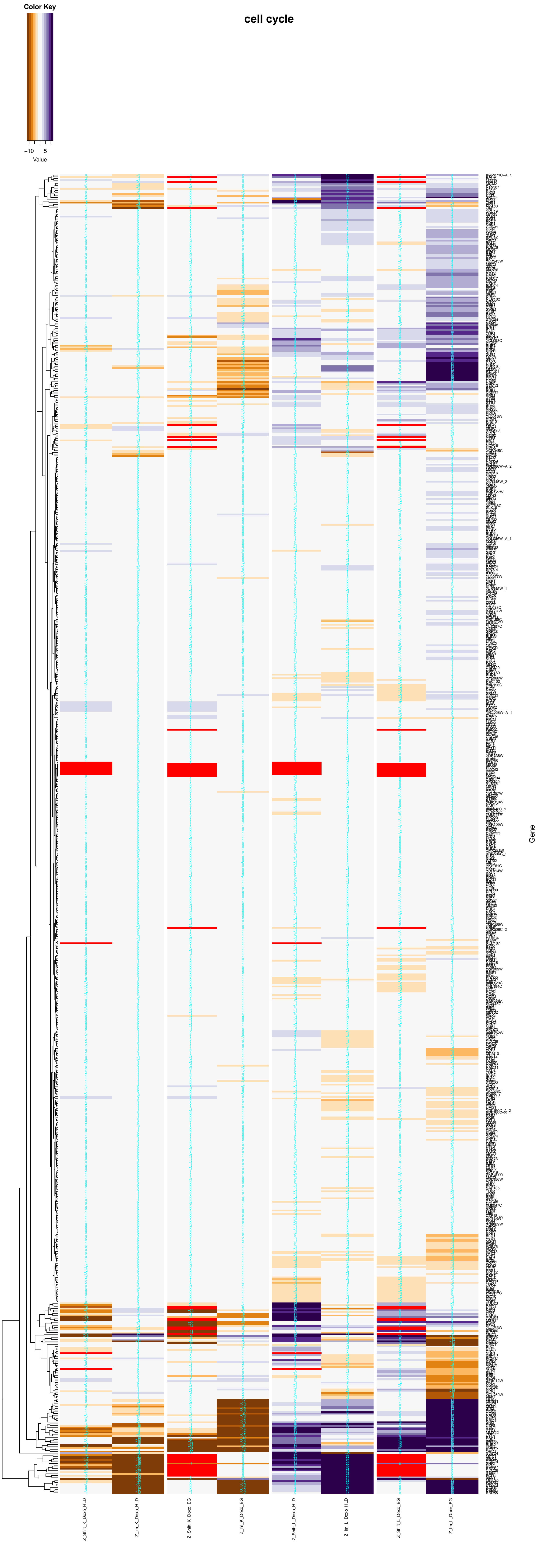

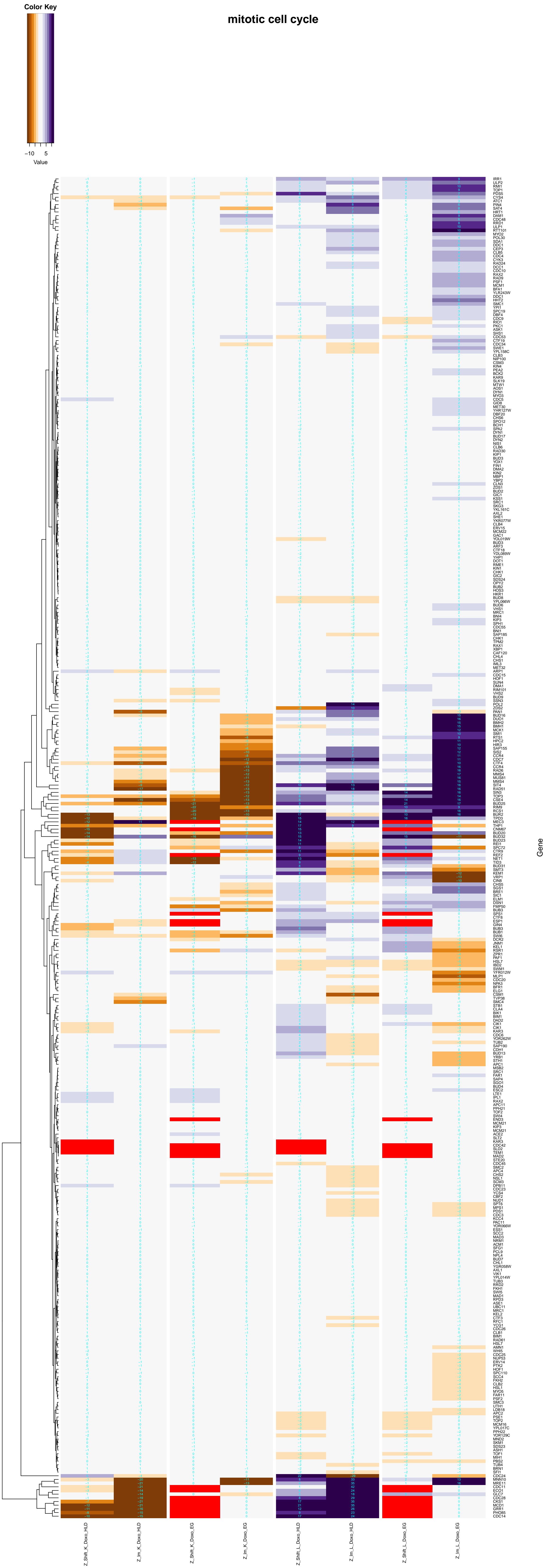

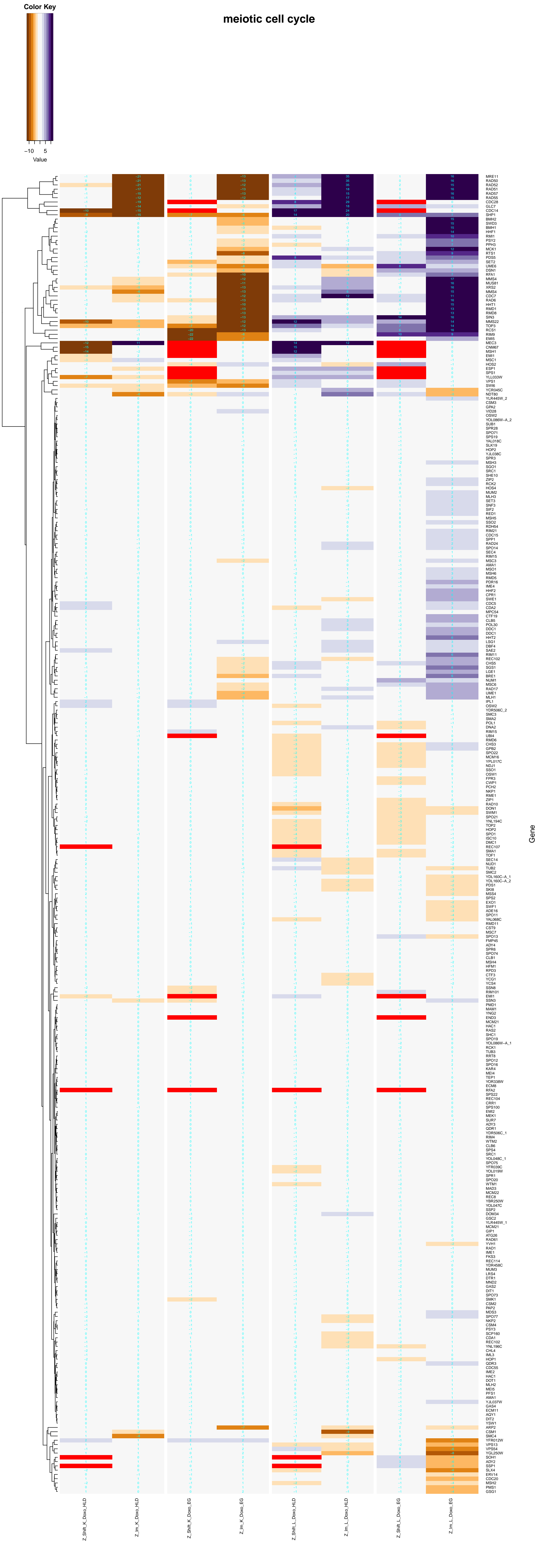

Supplement: Supplementary file 9 — Additional file 9. GO term-specific heatmaps for REMc/GTF-enriched clusters. GO term-specific heatmaps for significant GO process terms were generated as described in methods and Figs. 3 and 4. Any related child terms are presented in subsequent pages of the parent file name. GO terms with more than 100 children, with 2 or fewer genes annotated to the term, or a file size over 300KB are not shown. All heatmaps are generated with the same layout (see Figs. 3 and 4). [file 40170_2019_201_MOESM9_ESM.bz2 › Additional_File9_GOTermHeatmaps/Additional_File9_GOTermHeatmap/cell_cycle.pdf]

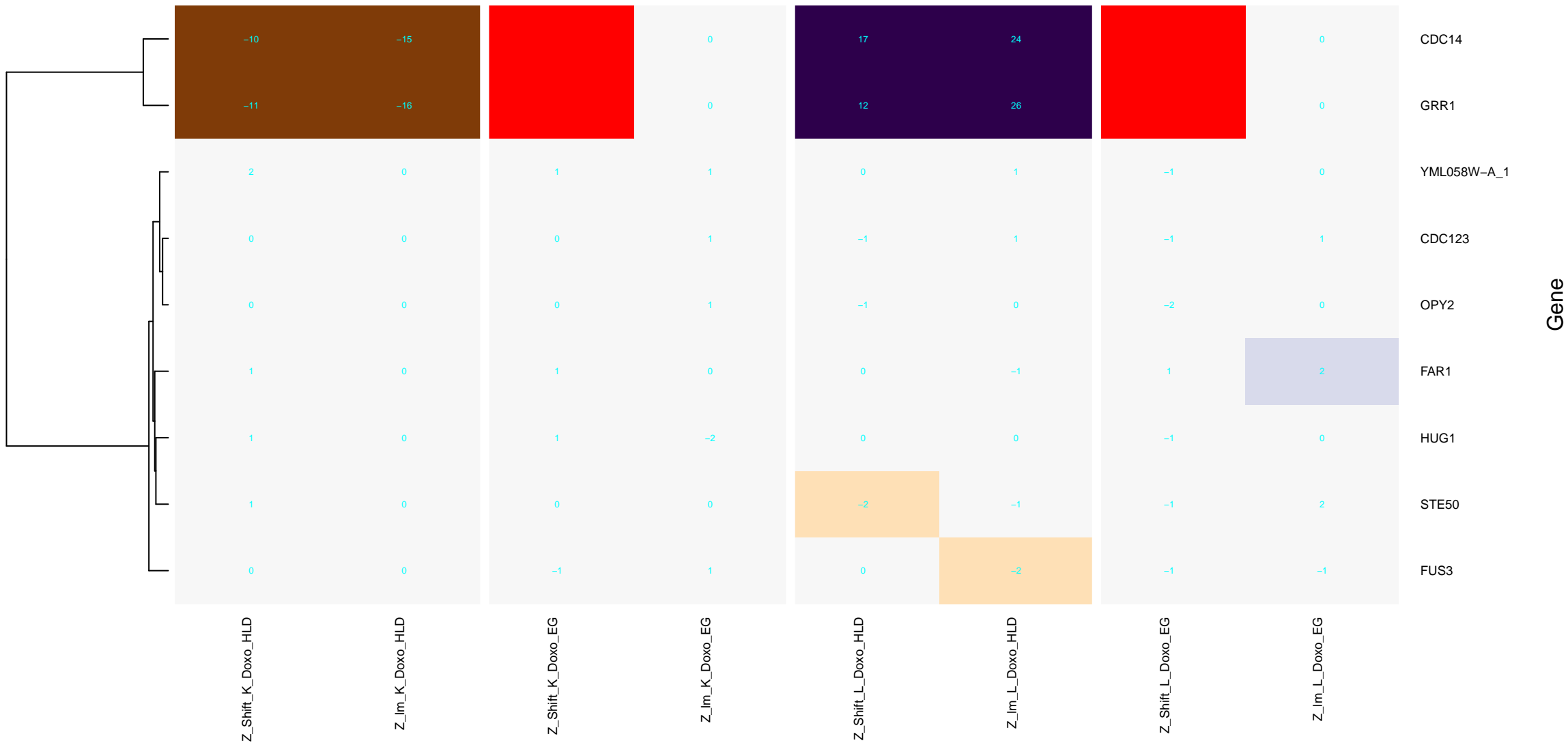

Supplement: Supplementary file 9 — Additional file 9. GO term-specific heatmaps for REMc/GTF-enriched clusters. GO term-specific heatmaps for significant GO process terms were generated as described in methods and Figs. 3 and 4. Any related child terms are presented in subsequent pages of the parent file name. GO terms with more than 100 children, with 2 or fewer genes annotated to the term, or a file size over 300KB are not shown. All heatmaps are generated with the same layout (see Figs. 3 and 4). [file 40170_2019_201_MOESM9_ESM.bz2 › Additional_File9_GOTermHeatmaps/Additional_File9_GOTermHeatmap/cell_cycle_arrest.pdf]

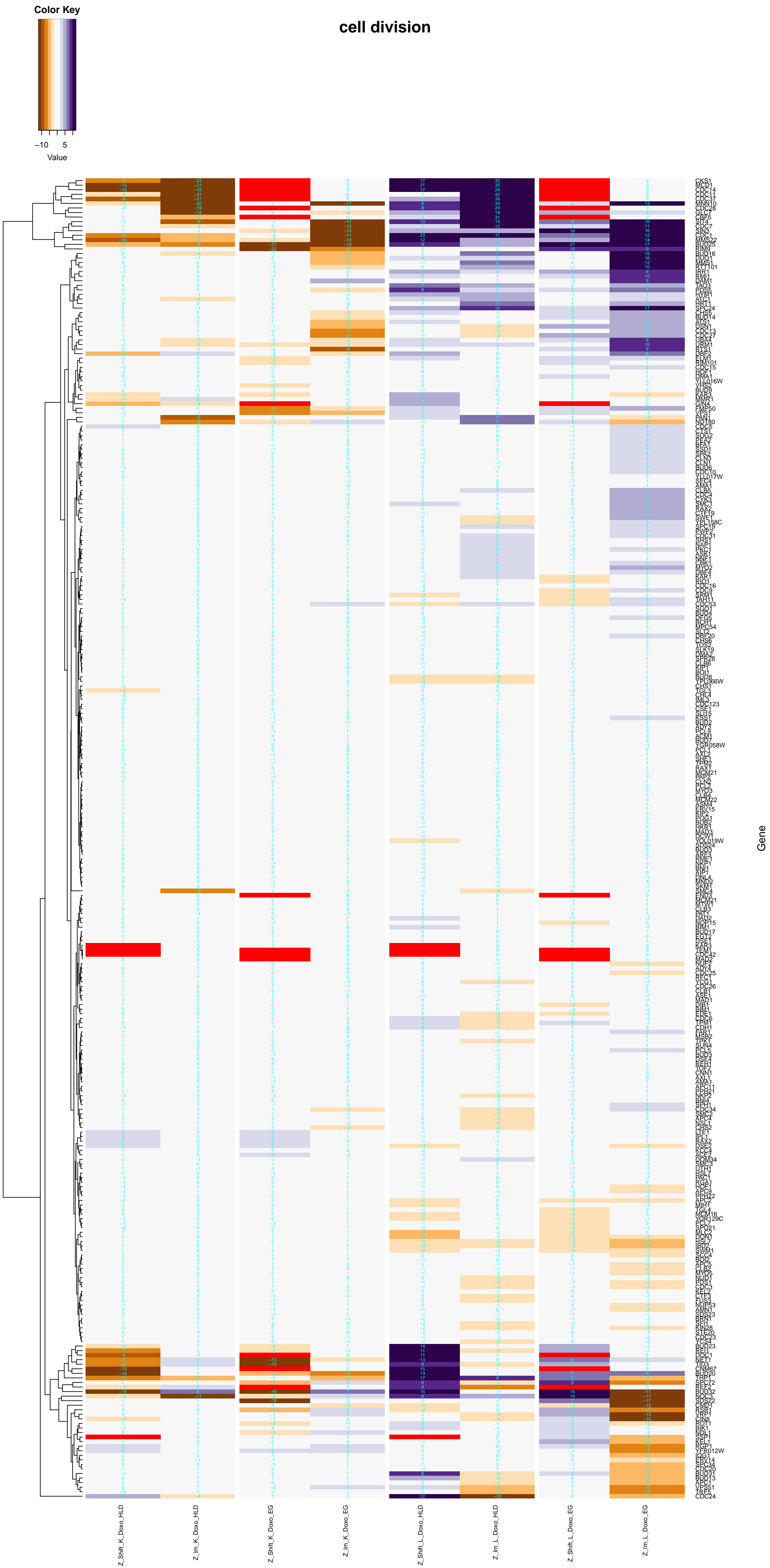

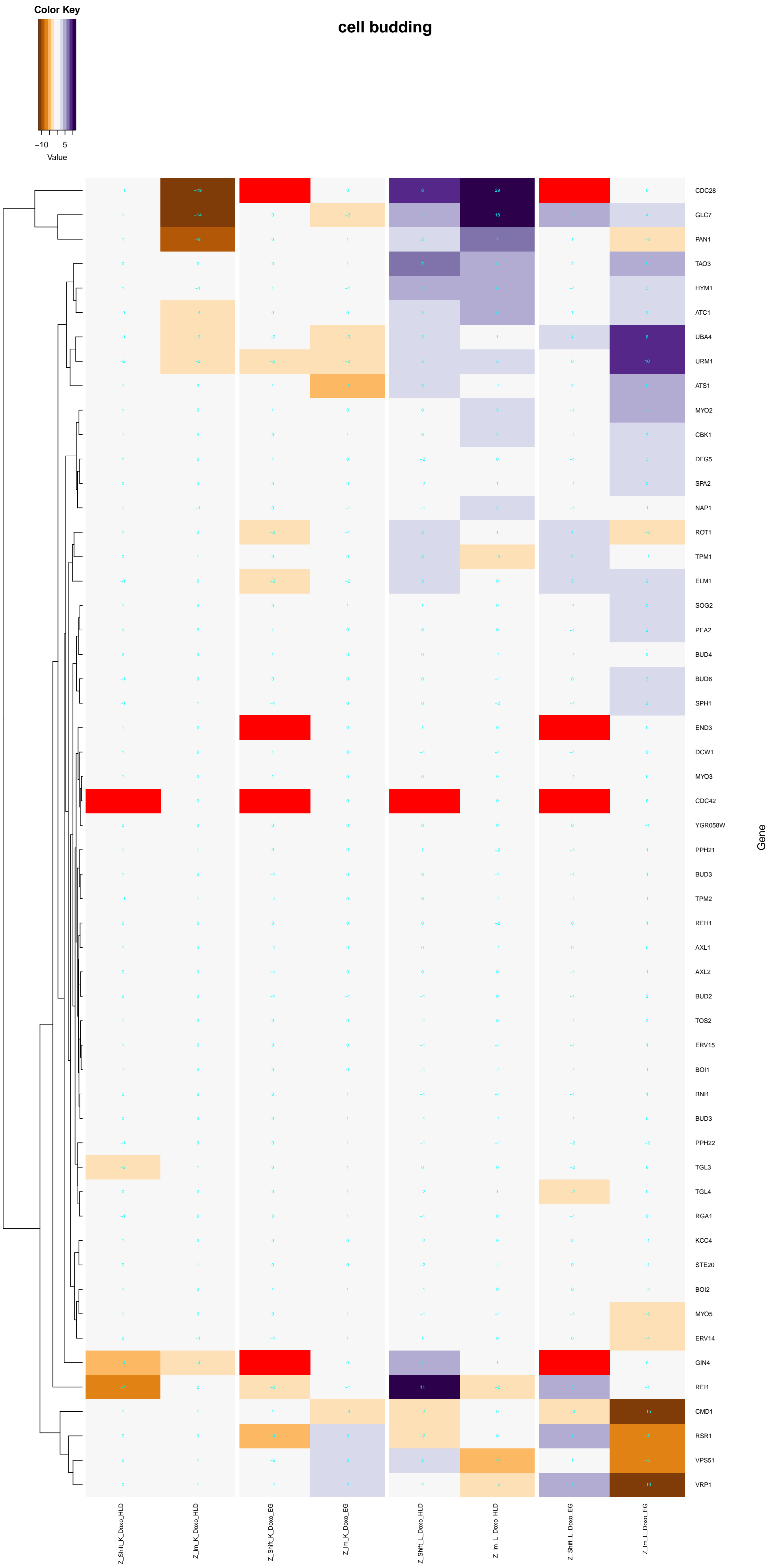

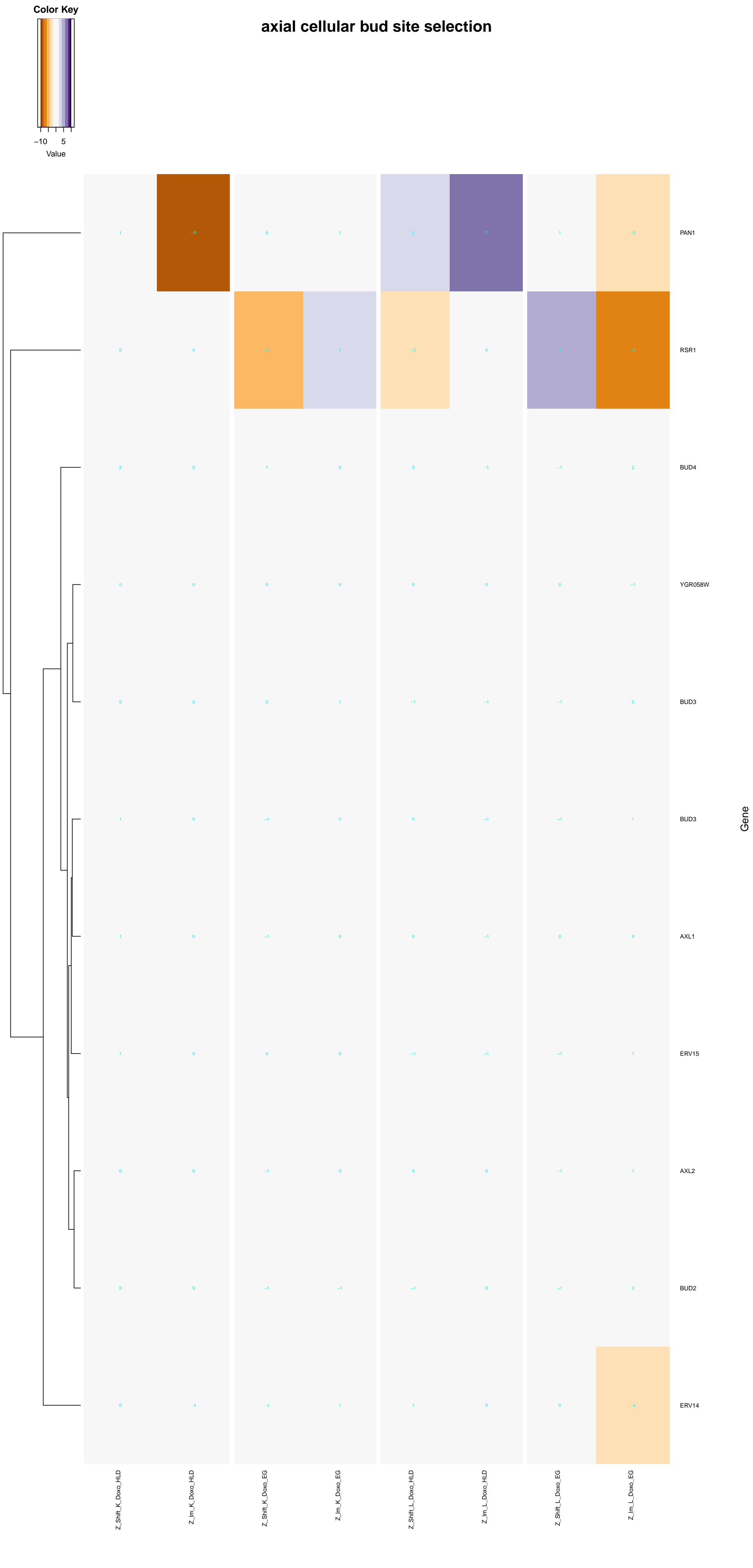

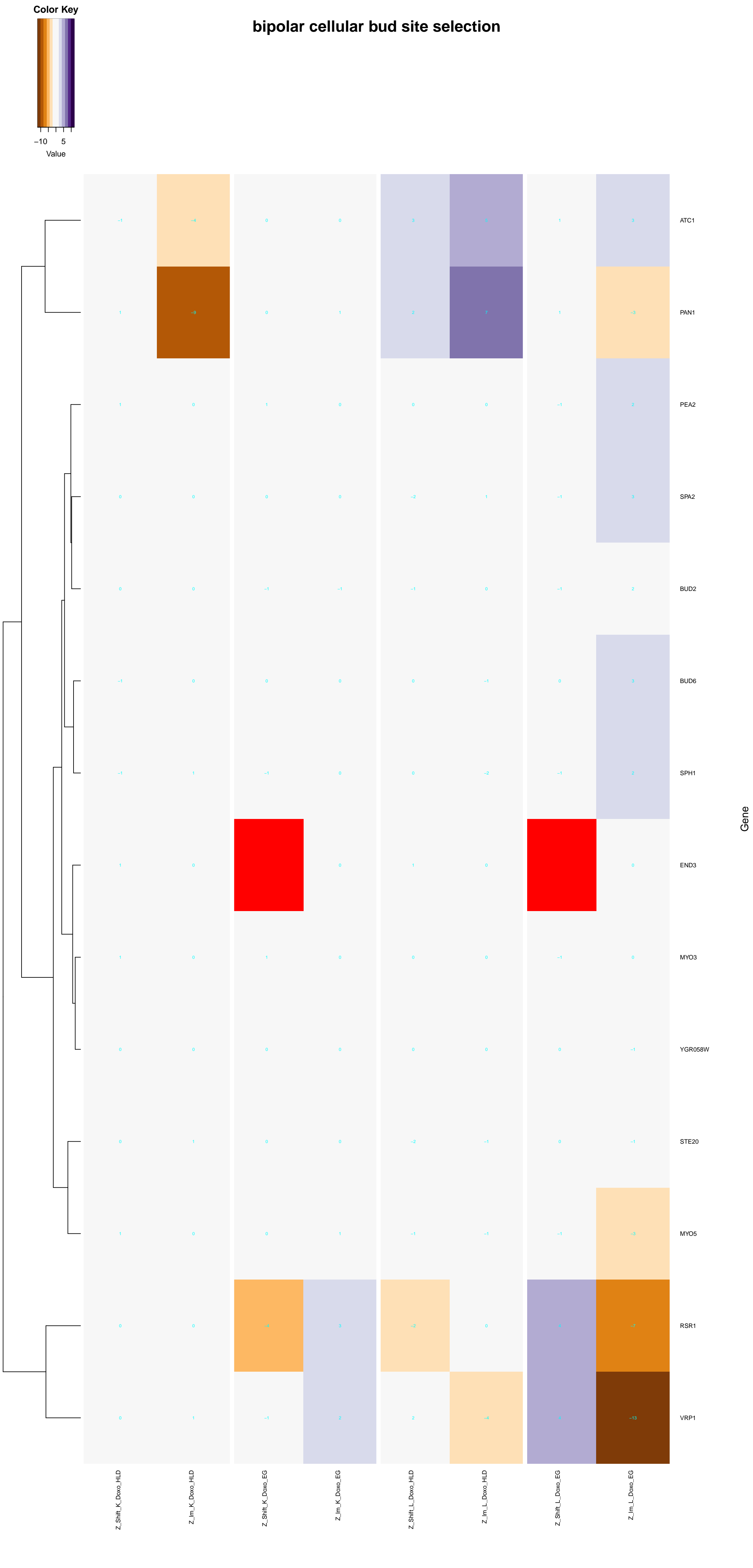

Supplement: Supplementary file 9 — Additional file 9. GO term-specific heatmaps for REMc/GTF-enriched clusters. GO term-specific heatmaps for significant GO process terms were generated as described in methods and Figs. 3 and 4. Any related child terms are presented in subsequent pages of the parent file name. GO terms with more than 100 children, with 2 or fewer genes annotated to the term, or a file size over 300KB are not shown. All heatmaps are generated with the same layout (see Figs. 3 and 4). [file 40170_2019_201_MOESM9_ESM.bz2 › Additional_File9_GOTermHeatmaps/Additional_File9_GOTermHeatmap/cell_division.pdf]

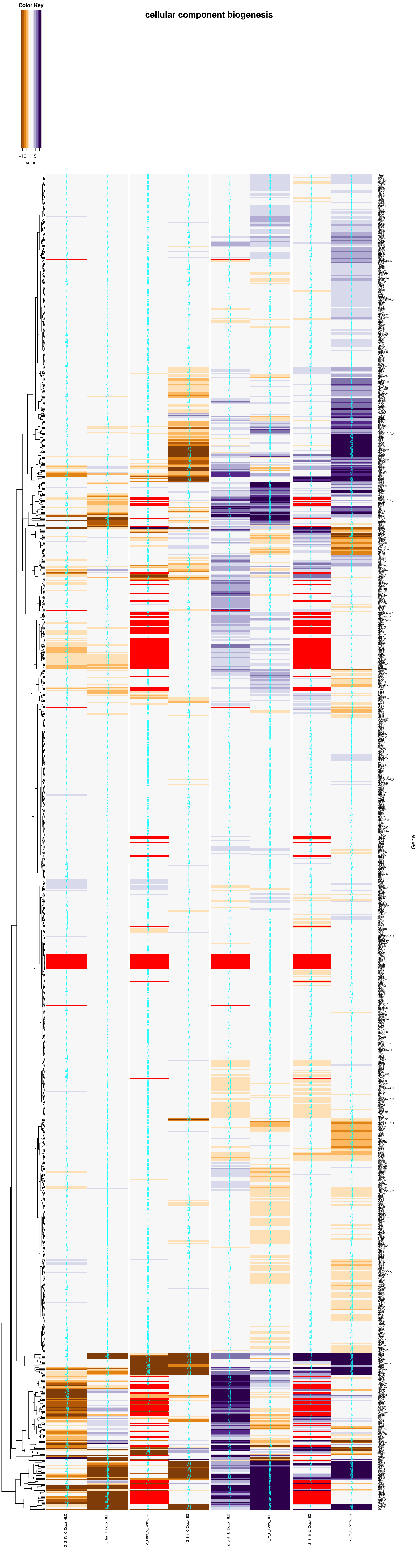

ribonucleoprotein complex biogenesis

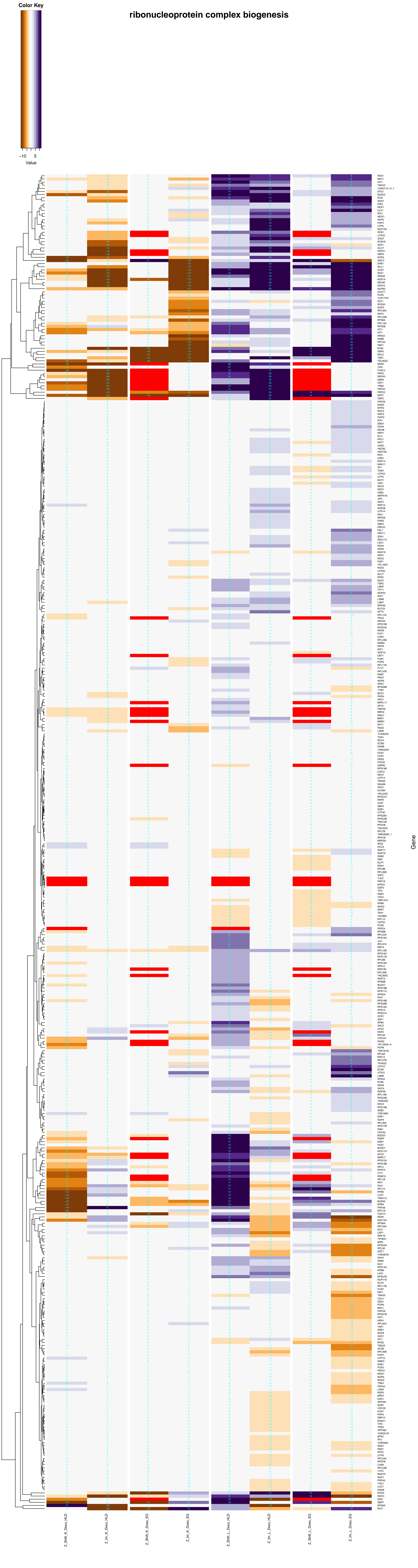

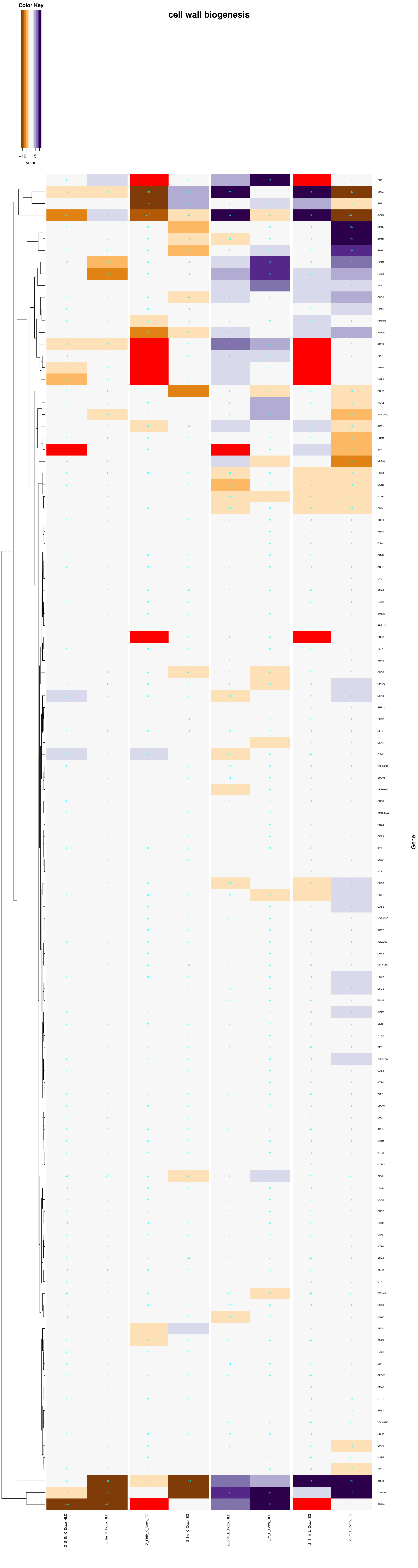

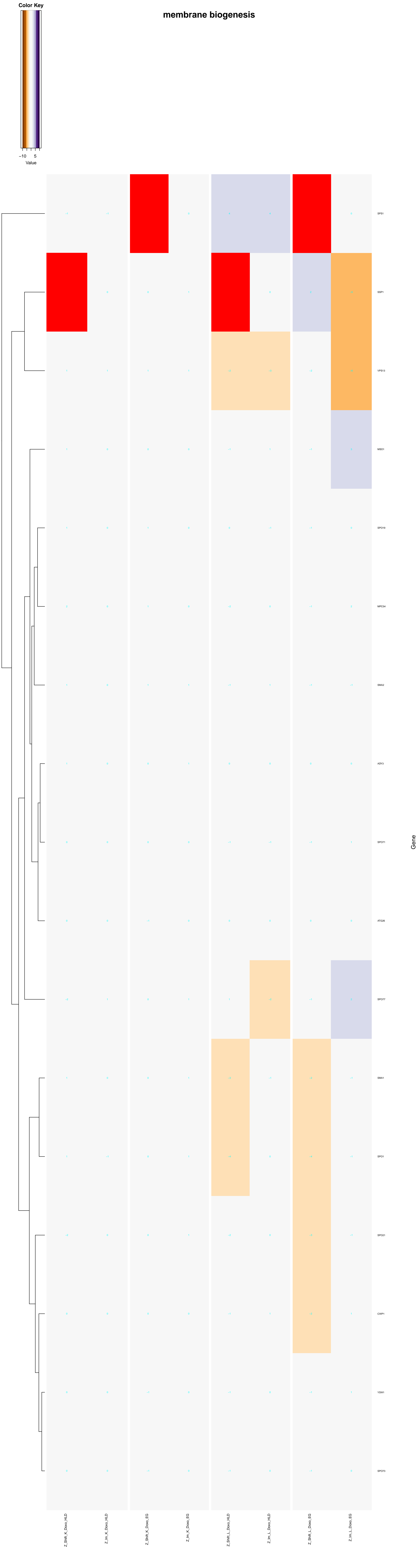

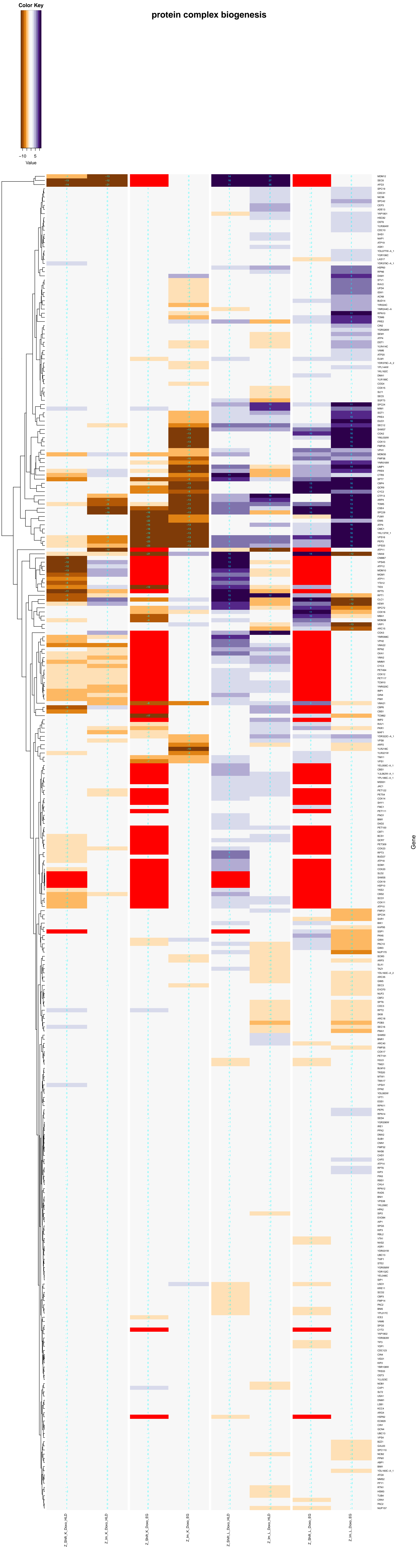

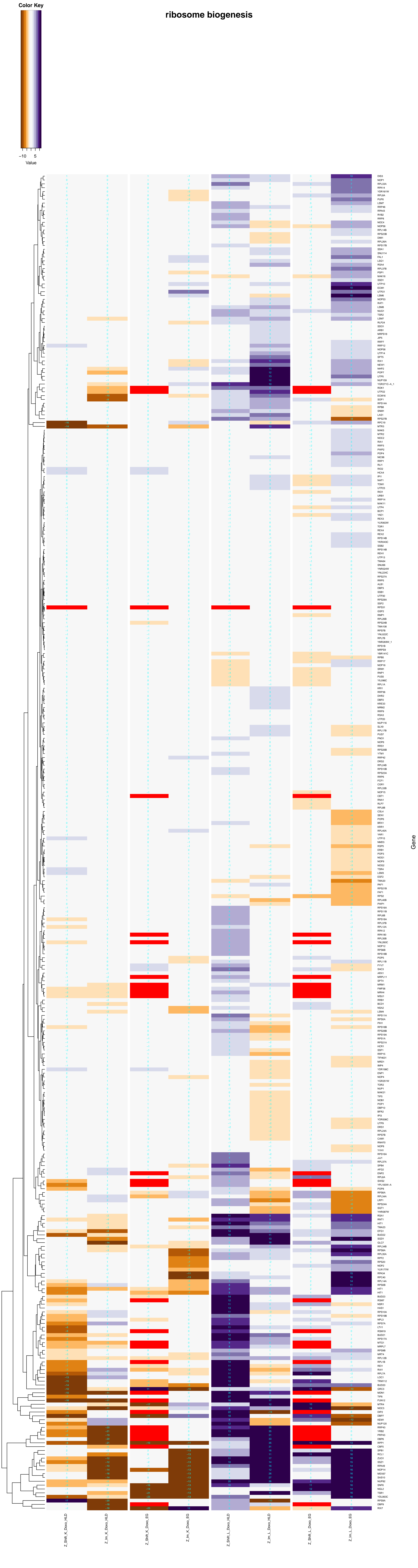

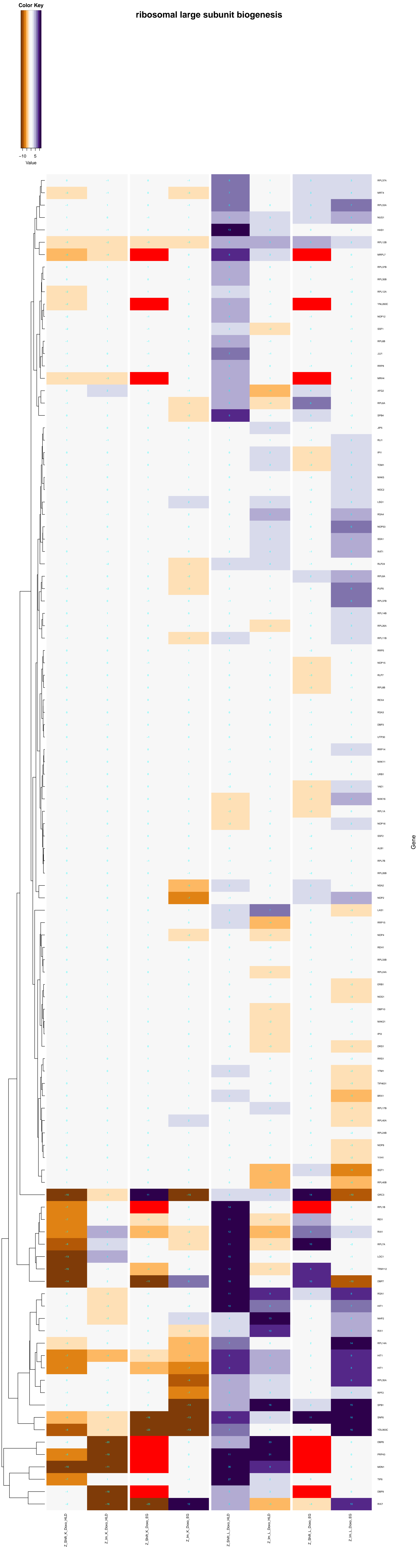

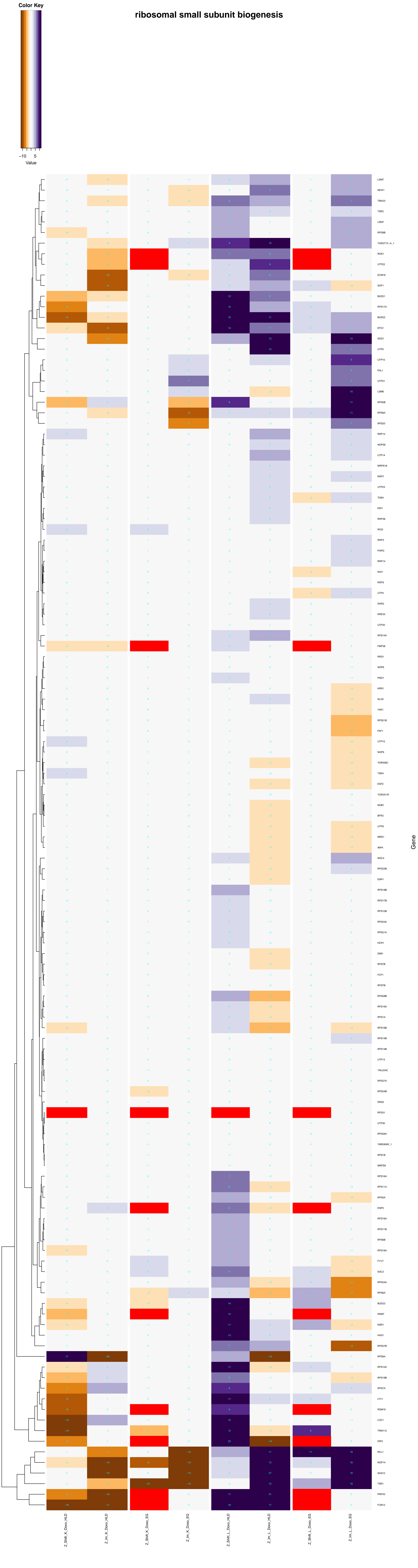

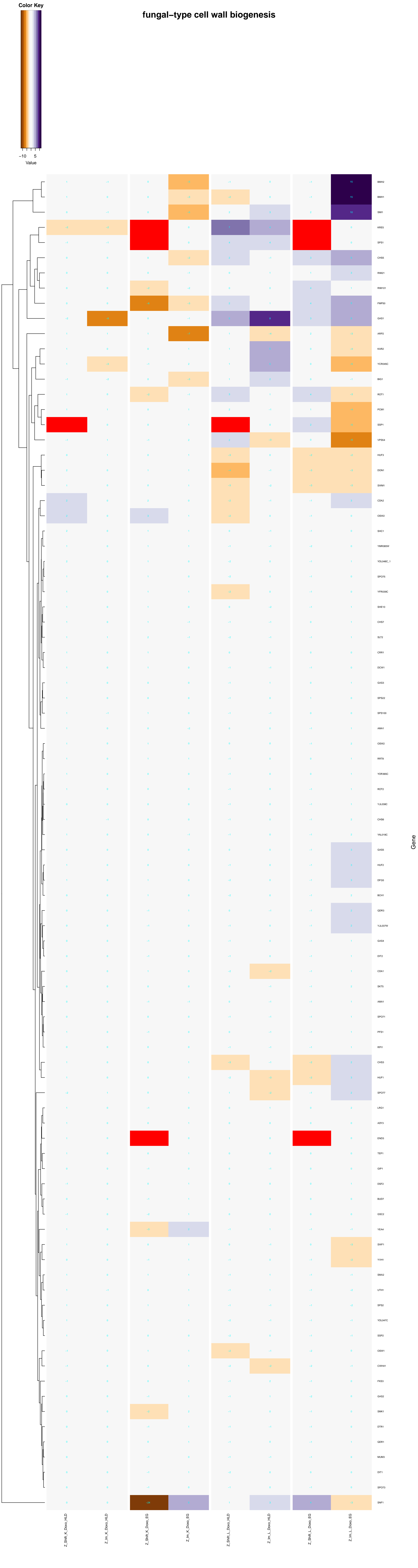

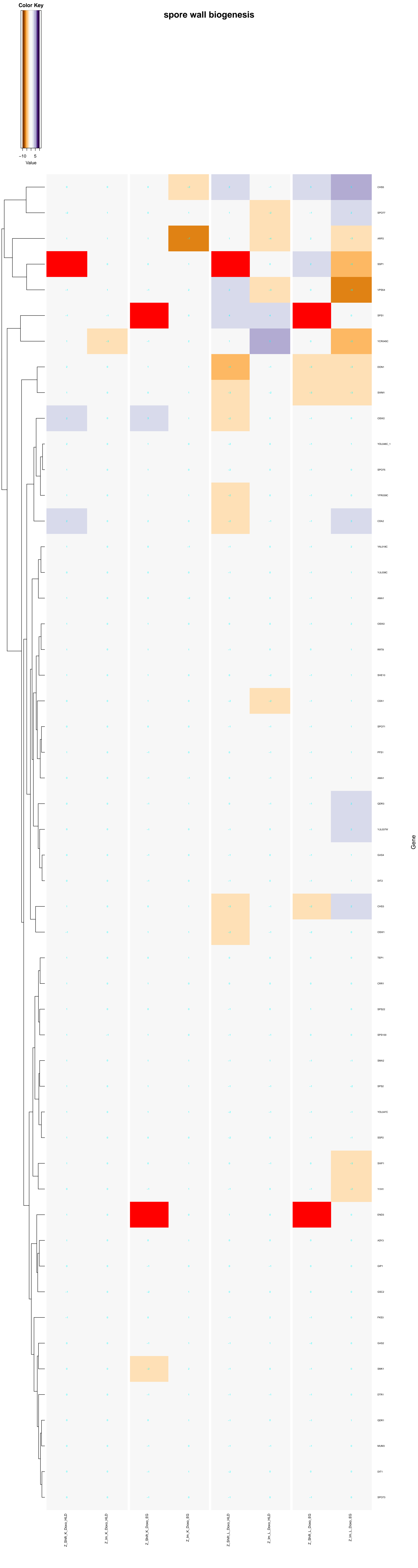

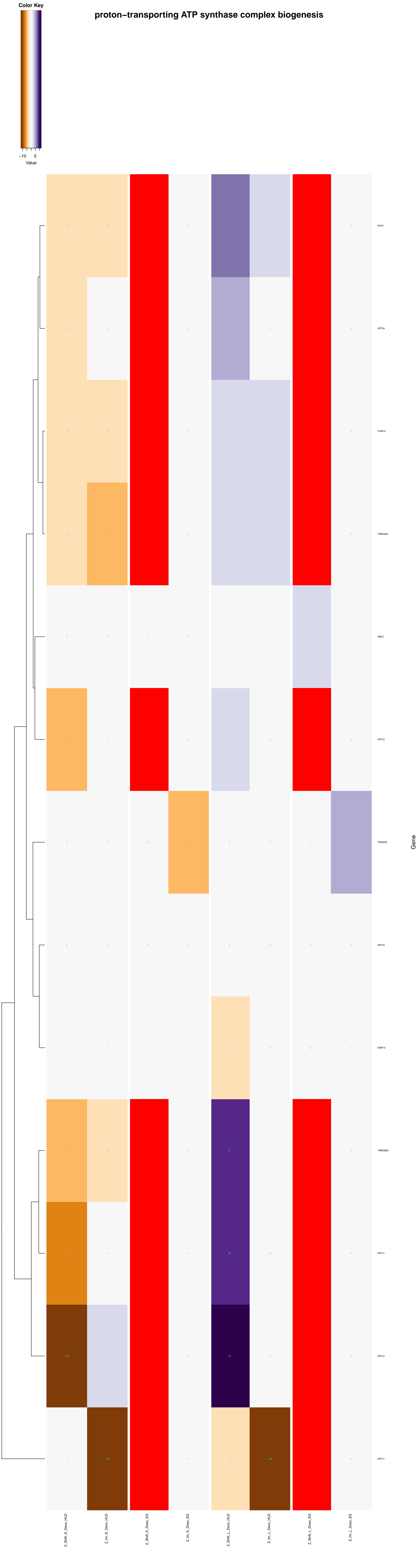

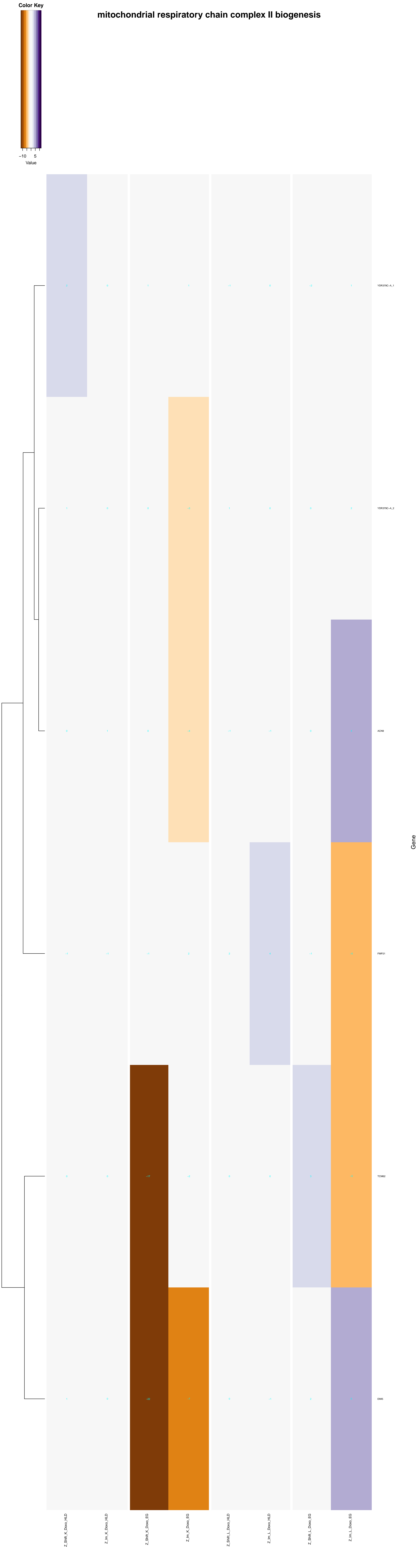

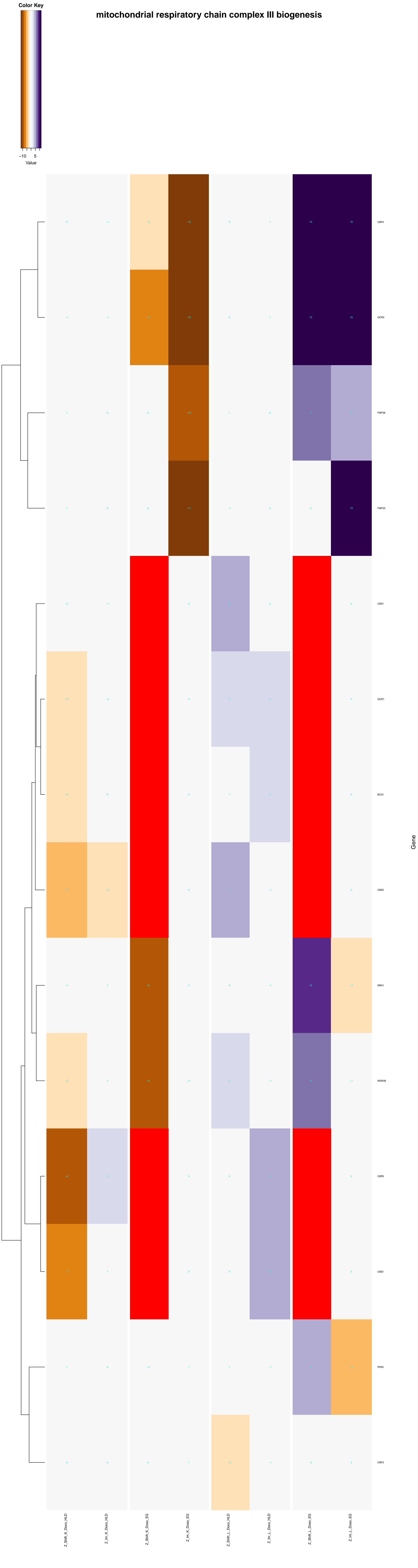

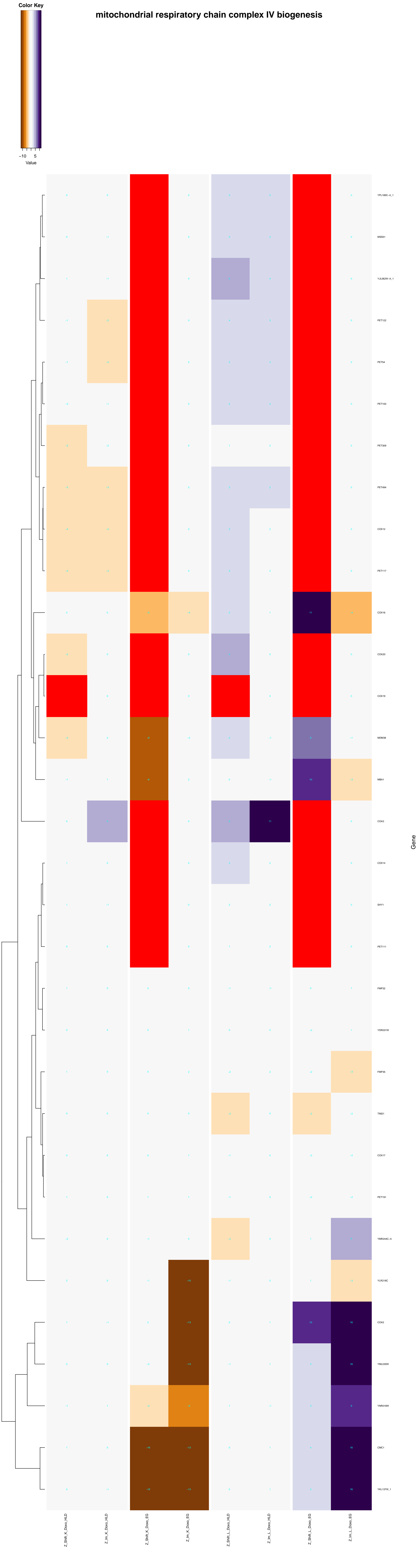

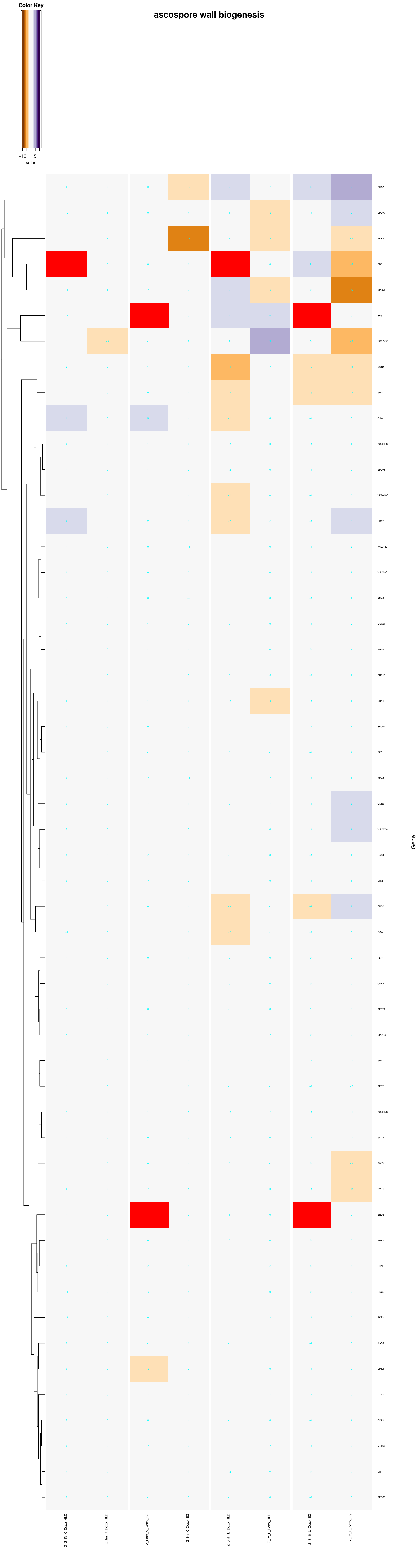

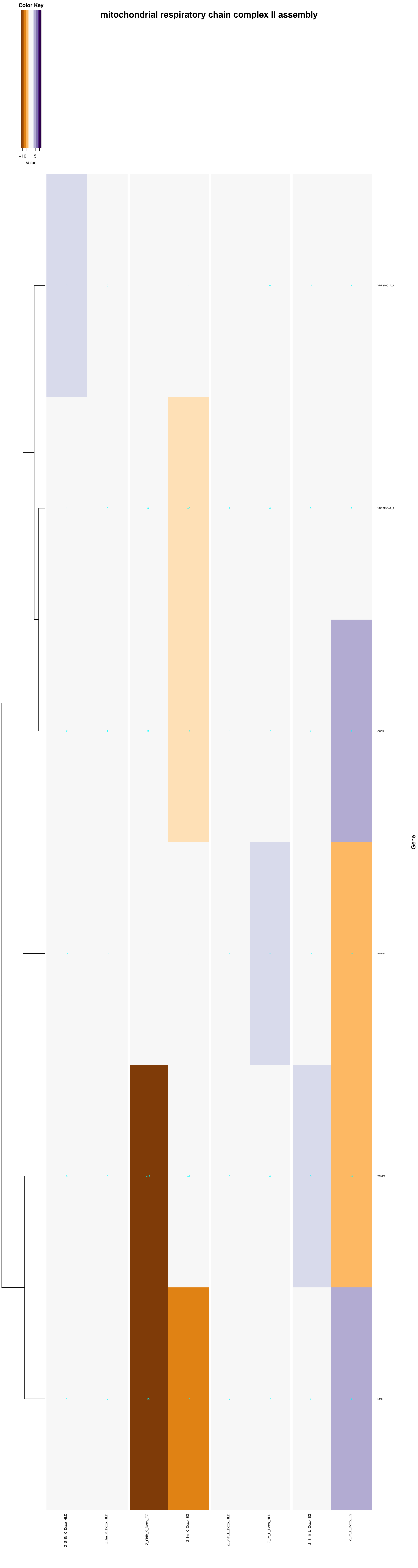

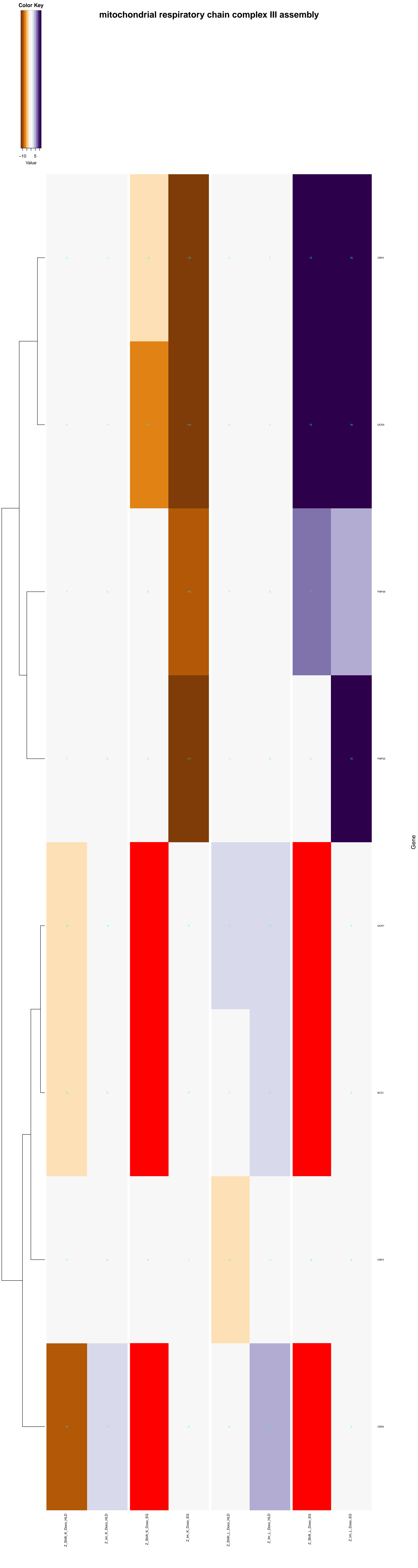

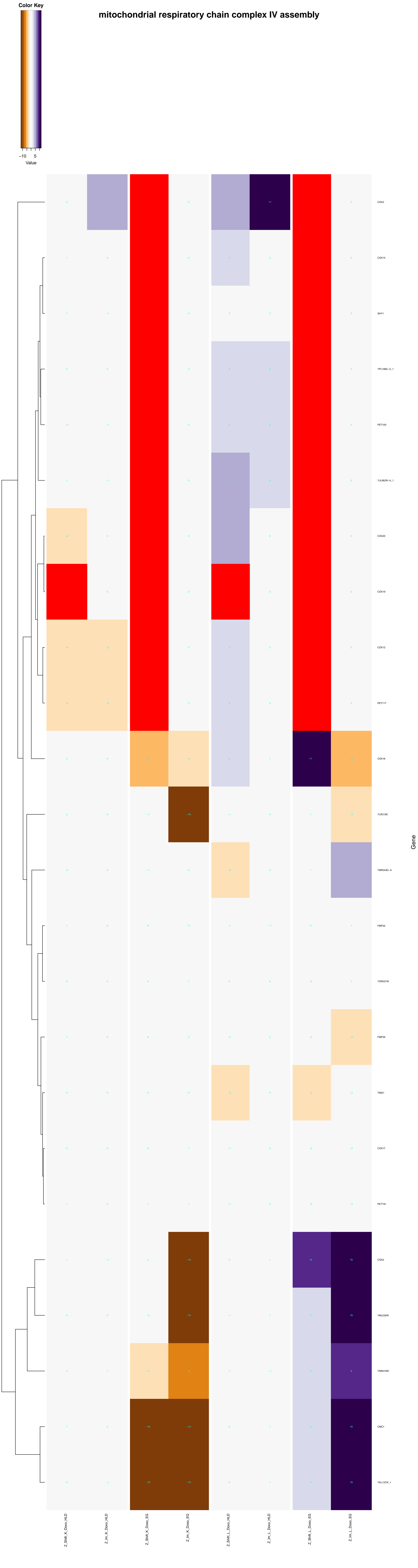

Supplement: Supplementary file 9 — Additional file 9. GO term-specific heatmaps for REMc/GTF-enriched clusters. GO term-specific heatmaps for significant GO process terms were generated as described in methods and Figs. 3 and 4. Any related child terms are presented in subsequent pages of the parent file name. GO terms with more than 100 children, with 2 or fewer genes annotated to the term, or a file size over 300KB are not shown. All heatmaps are generated with the same layout (see Figs. 3 and 4). [file 40170_2019_201_MOESM9_ESM.bz2 › Additional_File9_GOTermHeatmaps/Additional_File9_GOTermHeatmap/cellular_component_biogenesis.pdf]

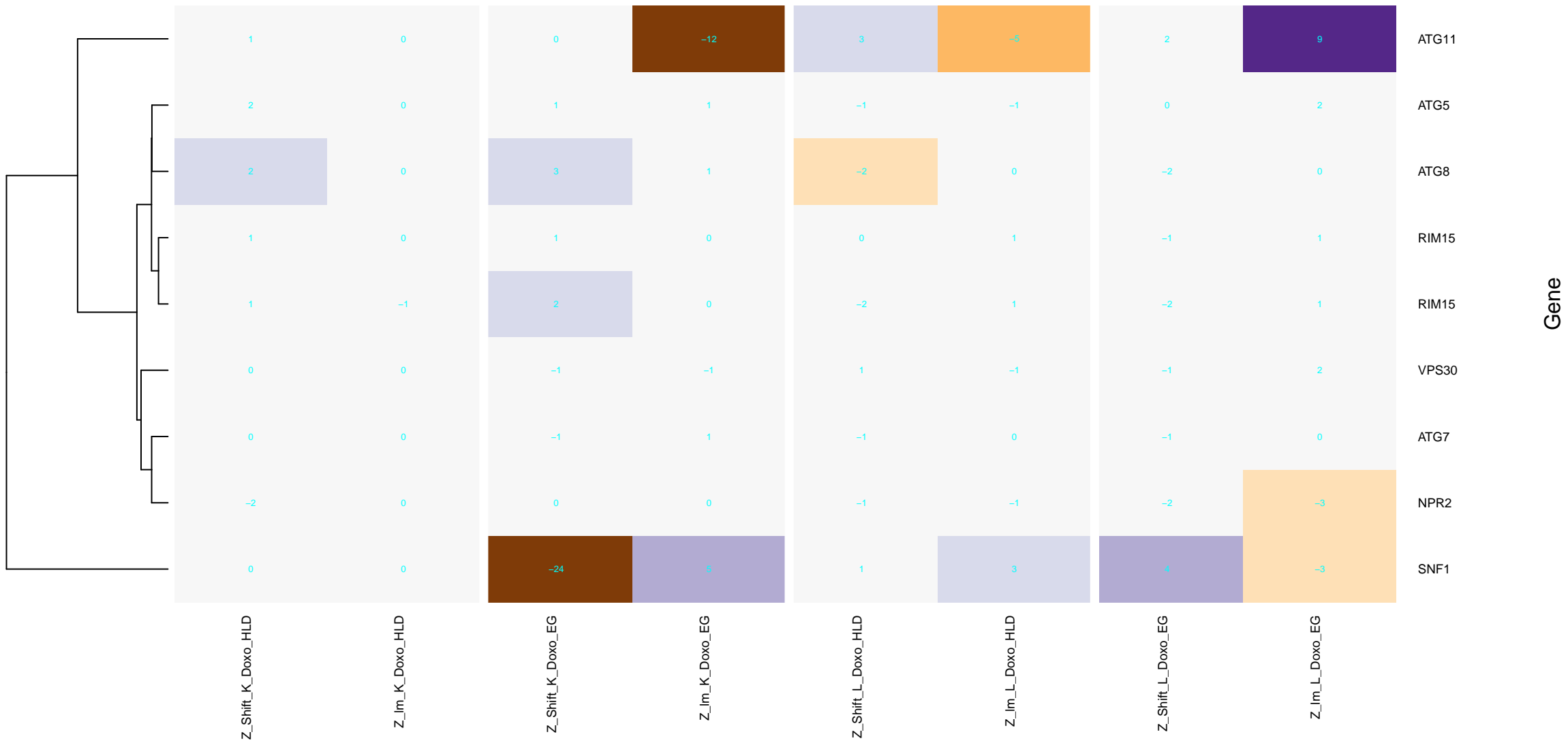

Supplement: Supplementary file 9 — Additional file 9. GO term-specific heatmaps for REMc/GTF-enriched clusters. GO term-specific heatmaps for significant GO process terms were generated as described in methods and Figs. 3 and 4. Any related child terms are presented in subsequent pages of the parent file name. GO terms with more than 100 children, with 2 or fewer genes annotated to the term, or a file size over 300KB are not shown. All heatmaps are generated with the same layout (see Figs. 3 and 4). [file 40170_2019_201_MOESM9_ESM.bz2 › Additional_File9_GOTermHeatmaps/Additional_File9_GOTermHeatmap/cellular_response_to_nitrogen_levels.pdf]

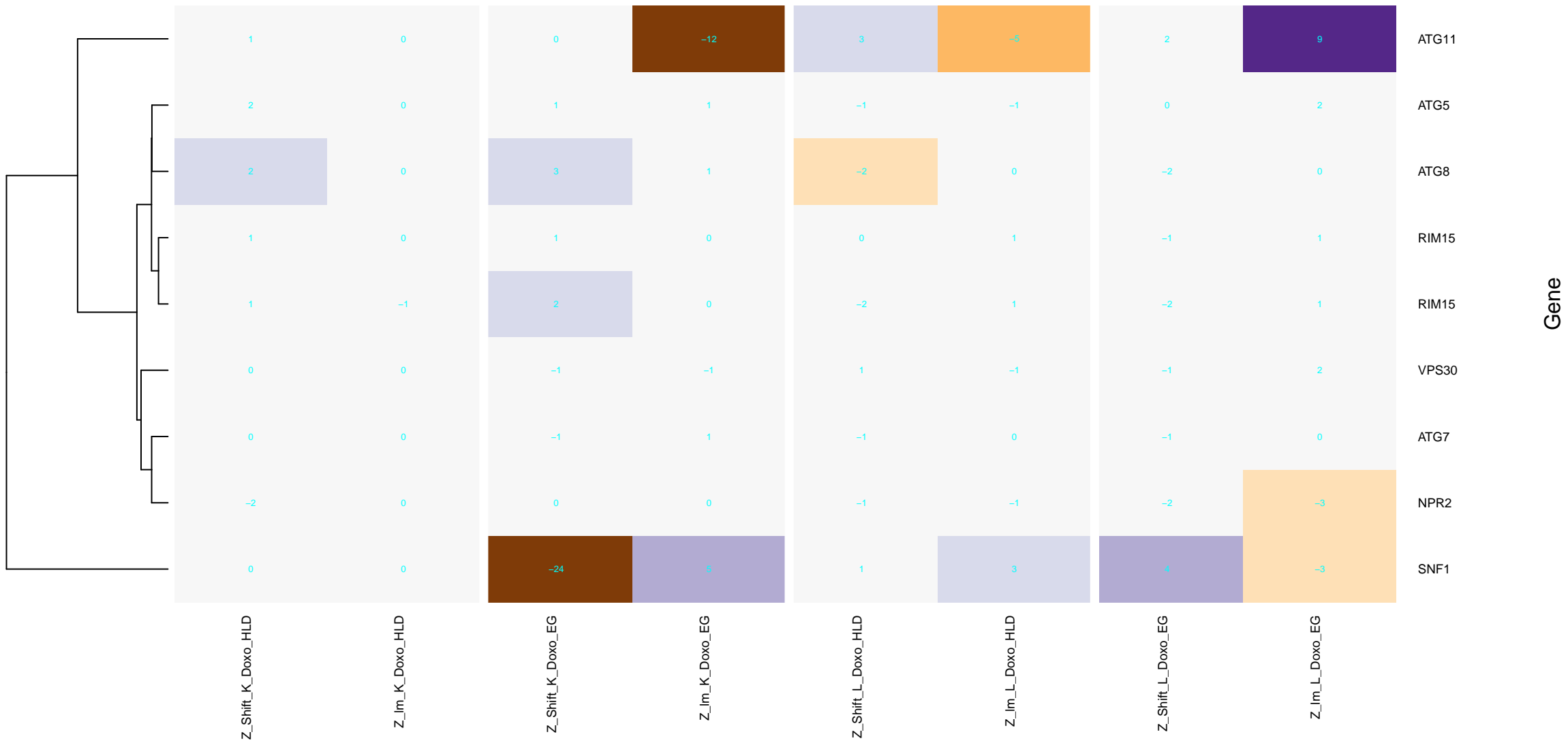

Supplement: Supplementary file 9 — Additional file 9. GO term-specific heatmaps for REMc/GTF-enriched clusters. GO term-specific heatmaps for significant GO process terms were generated as described in methods and Figs. 3 and 4. Any related child terms are presented in subsequent pages of the parent file name. GO terms with more than 100 children, with 2 or fewer genes annotated to the term, or a file size over 300KB are not shown. All heatmaps are generated with the same layout (see Figs. 3 and 4). [file 40170_2019_201_MOESM9_ESM.bz2 › Additional_File9_GOTermHeatmaps/Additional_File9_GOTermHeatmap/cellular_response_to_nitrogen_starvation.pdf]

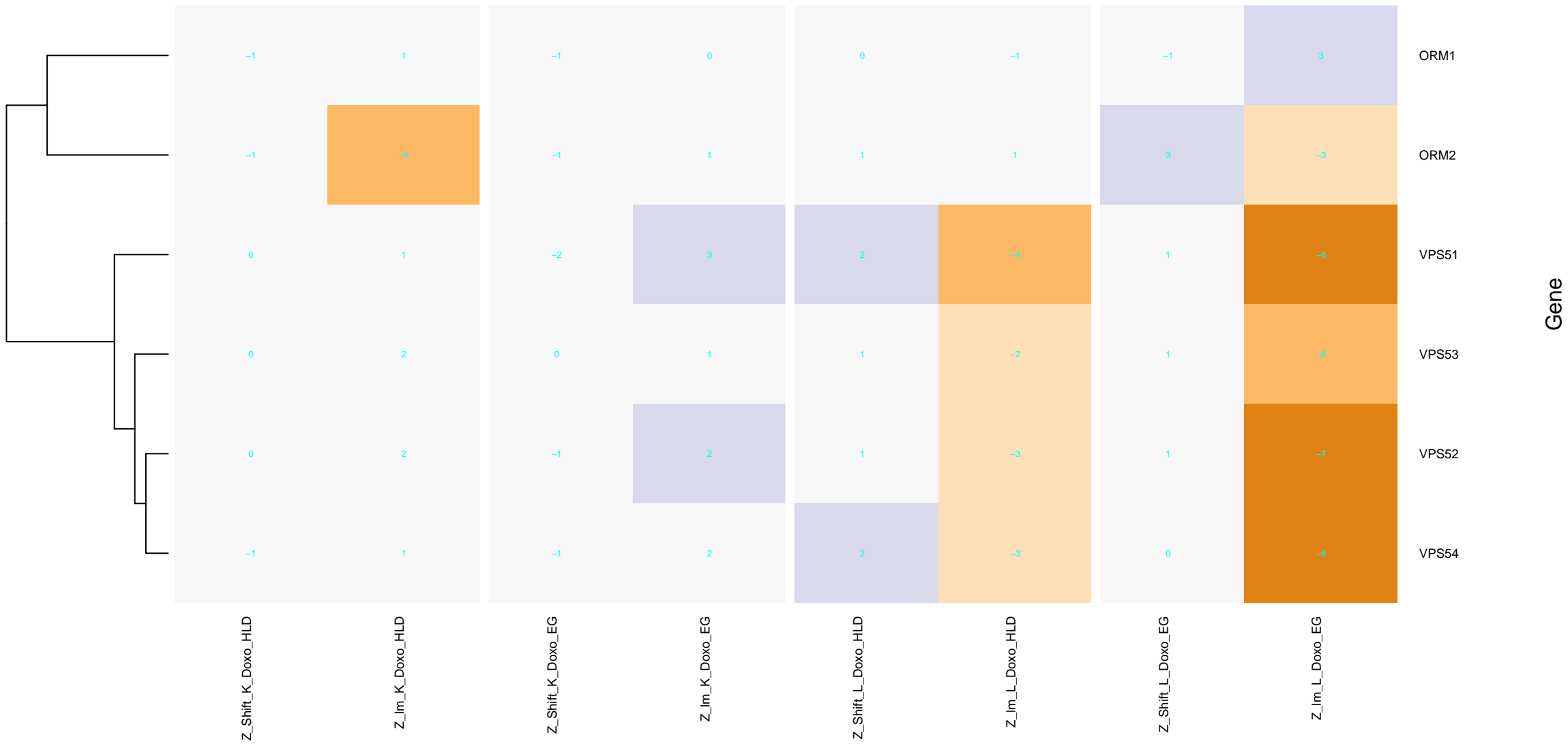

Supplement: Supplementary file 9 — Additional file 9. GO term-specific heatmaps for REMc/GTF-enriched clusters. GO term-specific heatmaps for significant GO process terms were generated as described in methods and Figs. 3 and 4. Any related child terms are presented in subsequent pages of the parent file name. GO terms with more than 100 children, with 2 or fewer genes annotated to the term, or a file size over 300KB are not shown. All heatmaps are generated with the same layout (see Figs. 3 and 4). [file 40170_2019_201_MOESM9_ESM.bz2 › Additional_File9_GOTermHeatmaps/Additional_File9_GOTermHeatmap/cellular_sphingolipid_homeostasis.pdf]

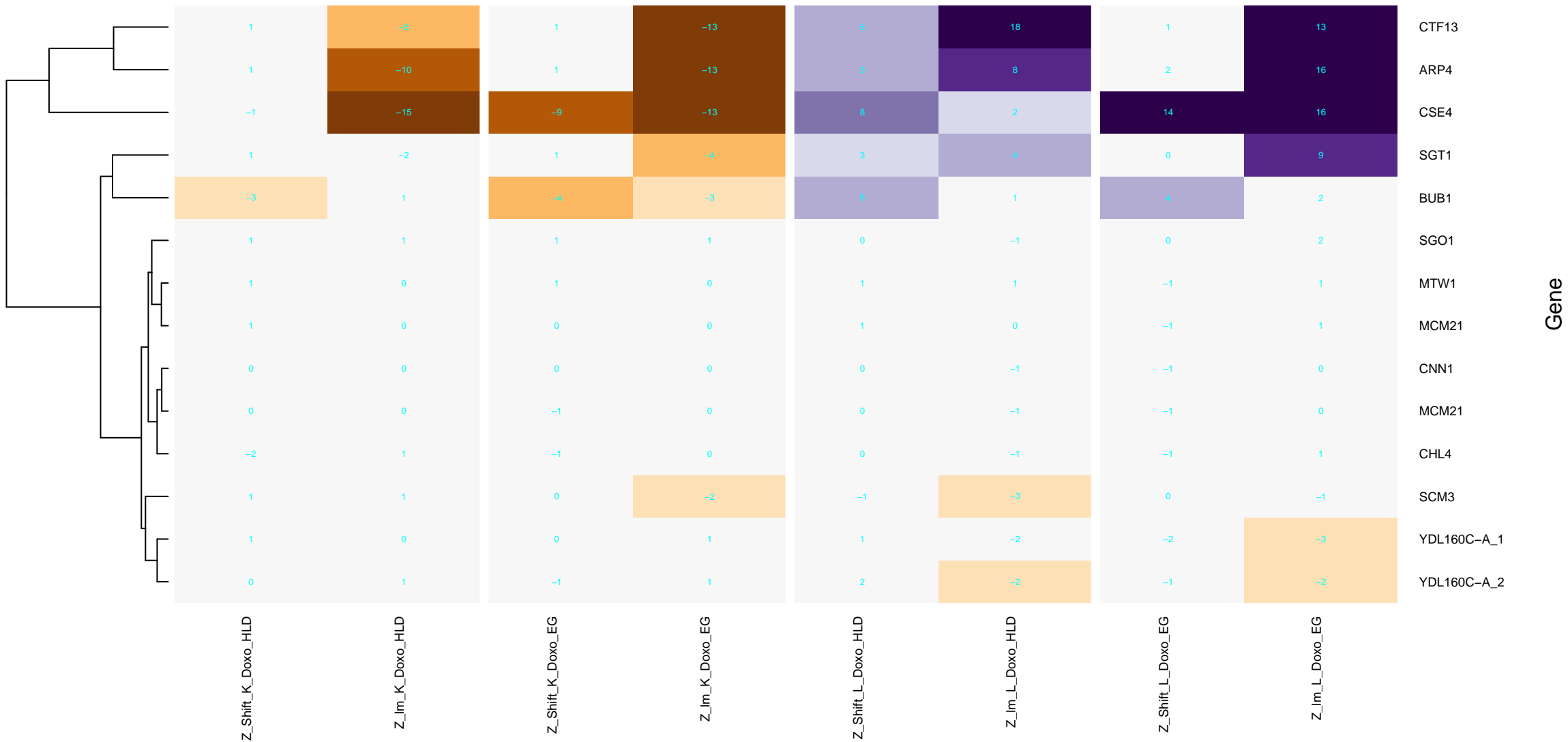

Supplement: Supplementary file 9 — Additional file 9. GO term-specific heatmaps for REMc/GTF-enriched clusters. GO term-specific heatmaps for significant GO process terms were generated as described in methods and Figs. 3 and 4. Any related child terms are presented in subsequent pages of the parent file name. GO terms with more than 100 children, with 2 or fewer genes annotated to the term, or a file size over 300KB are not shown. All heatmaps are generated with the same layout (see Figs. 3 and 4). [file 40170_2019_201_MOESM9_ESM.bz2 › Additional_File9_GOTermHeatmaps/Additional_File9_GOTermHeatmap/centromere_complex_assembly.pdf]

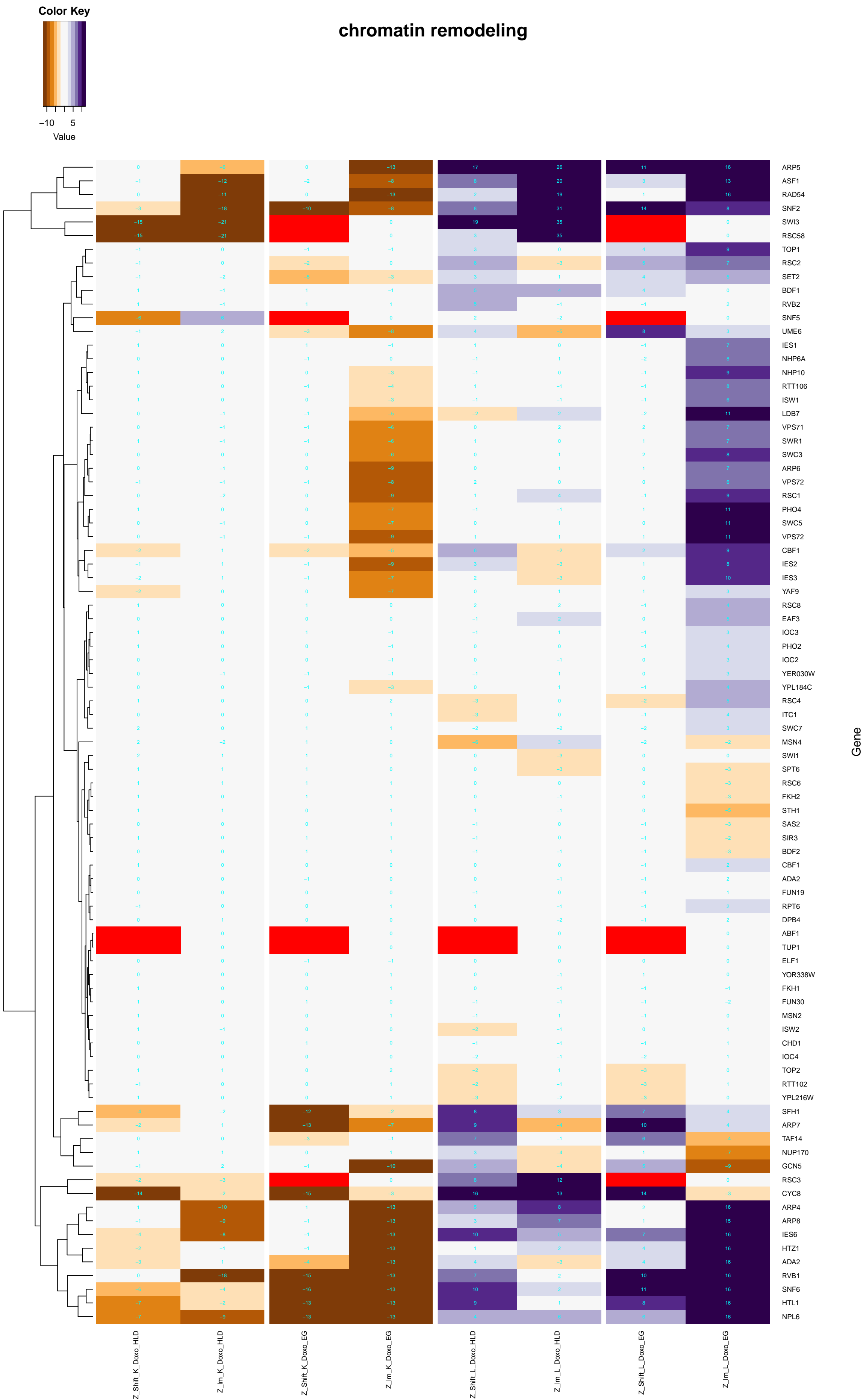

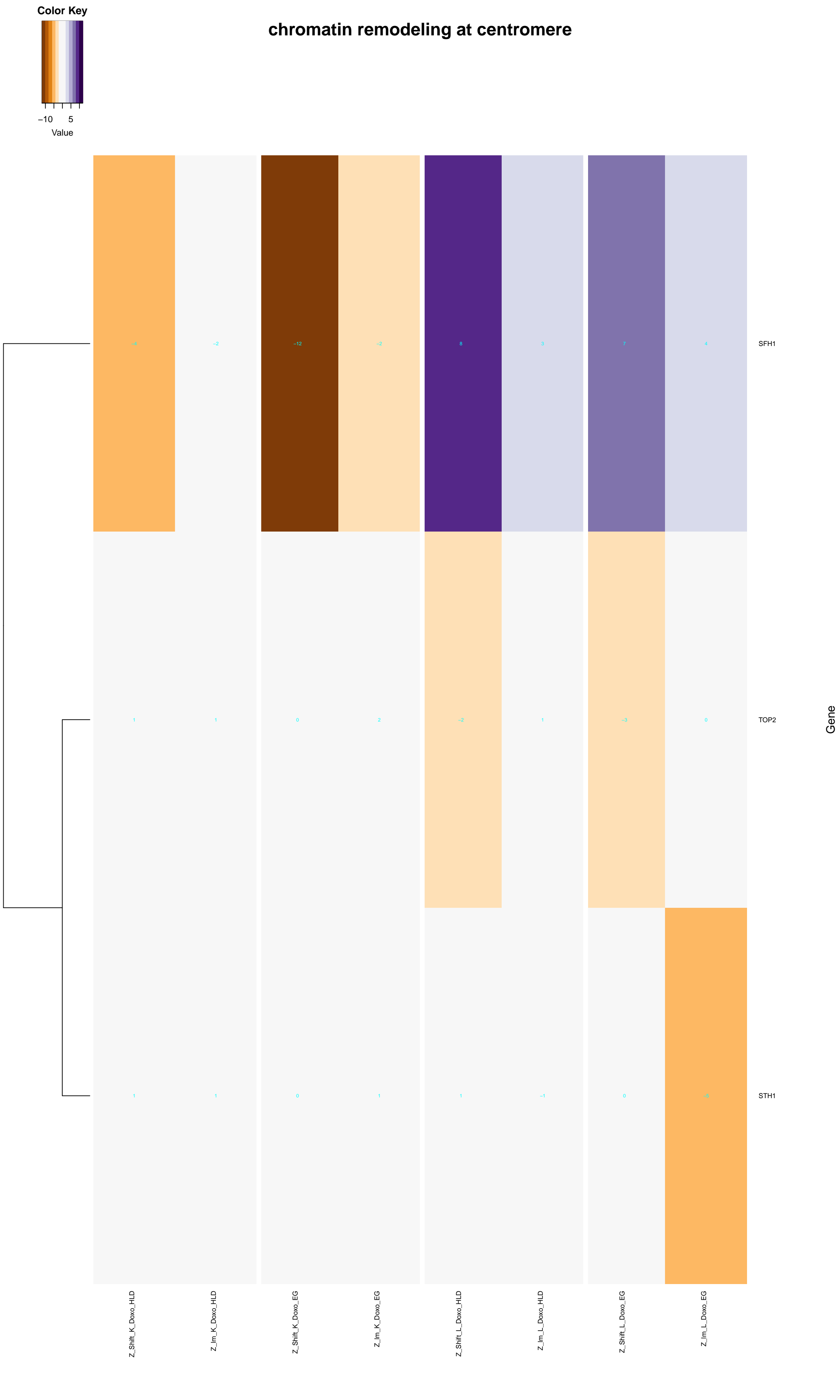

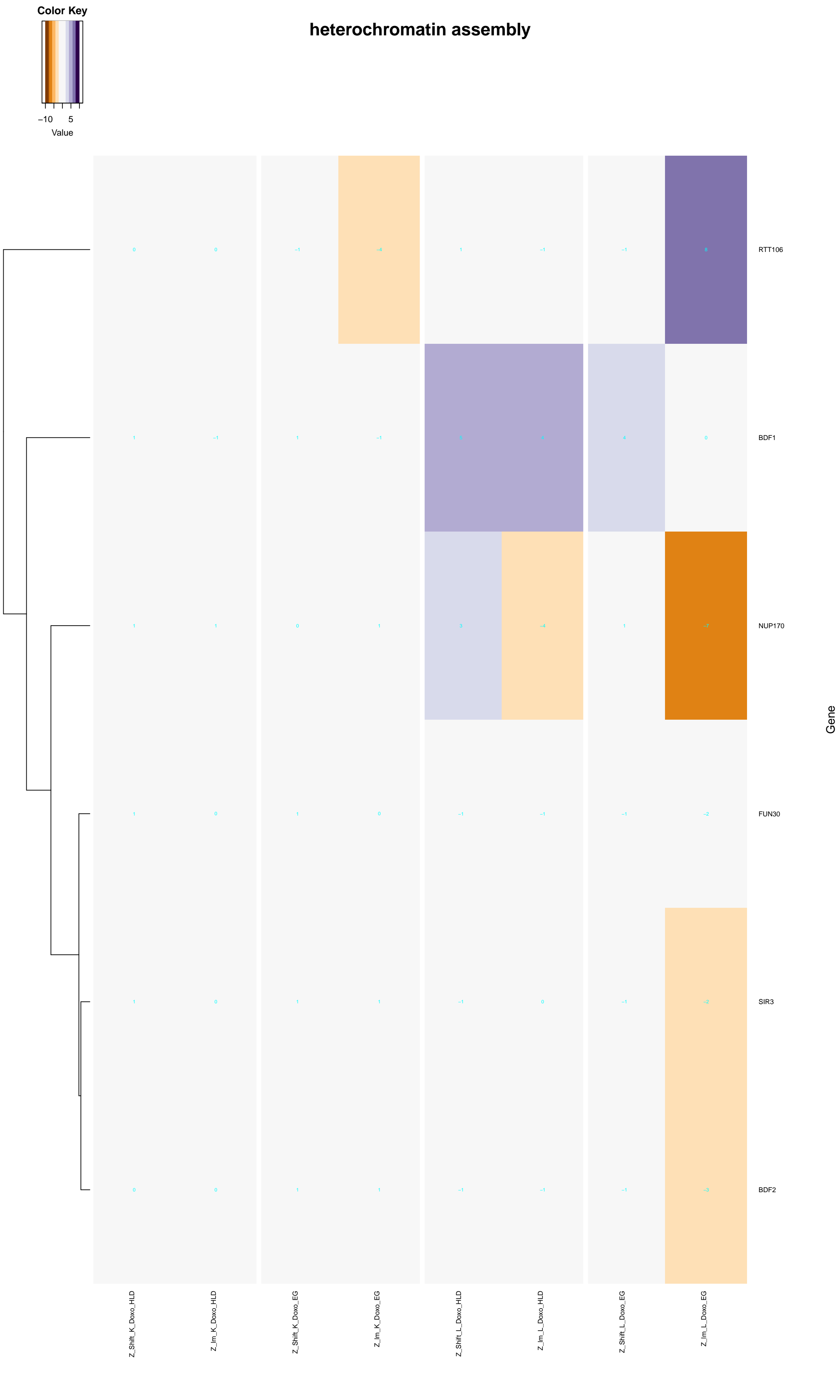

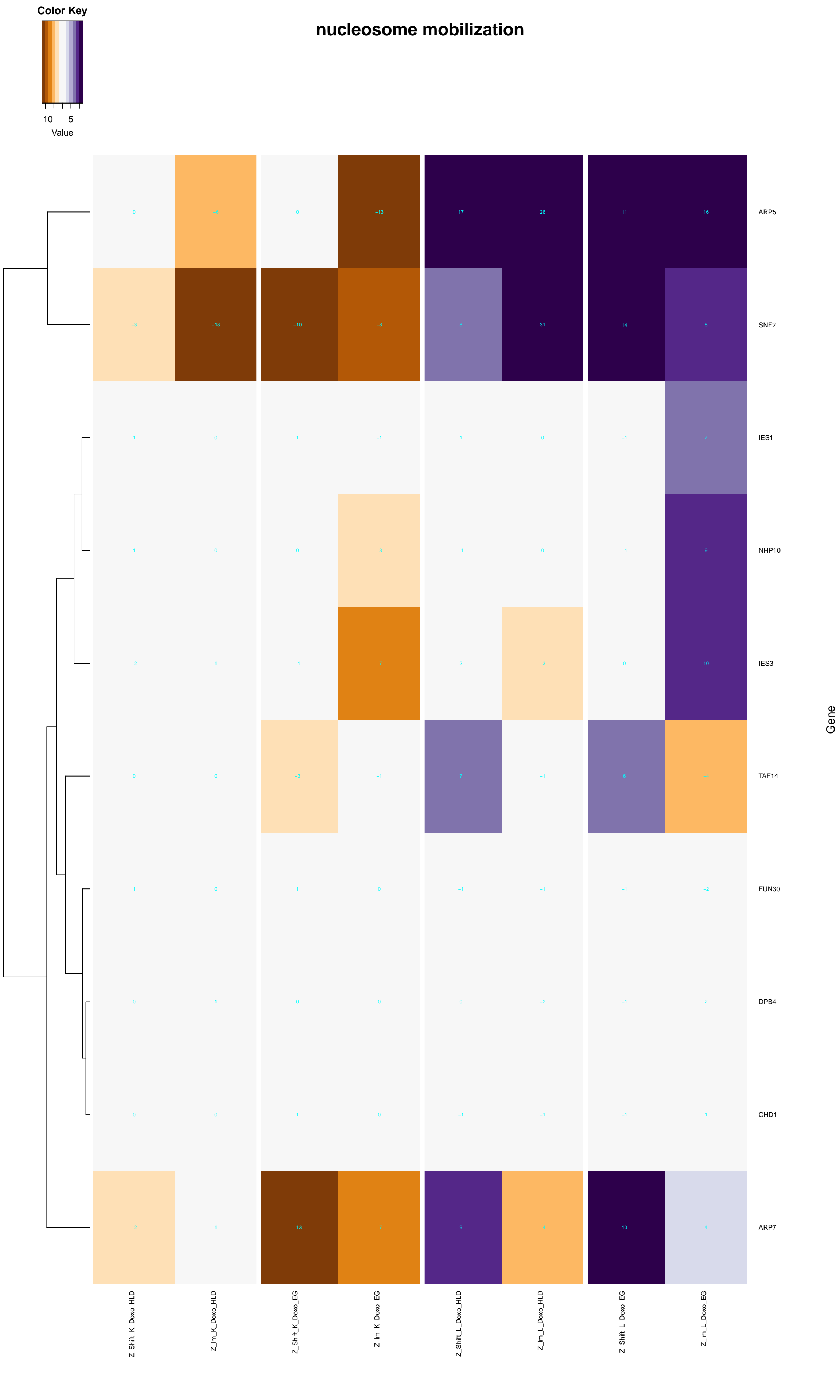

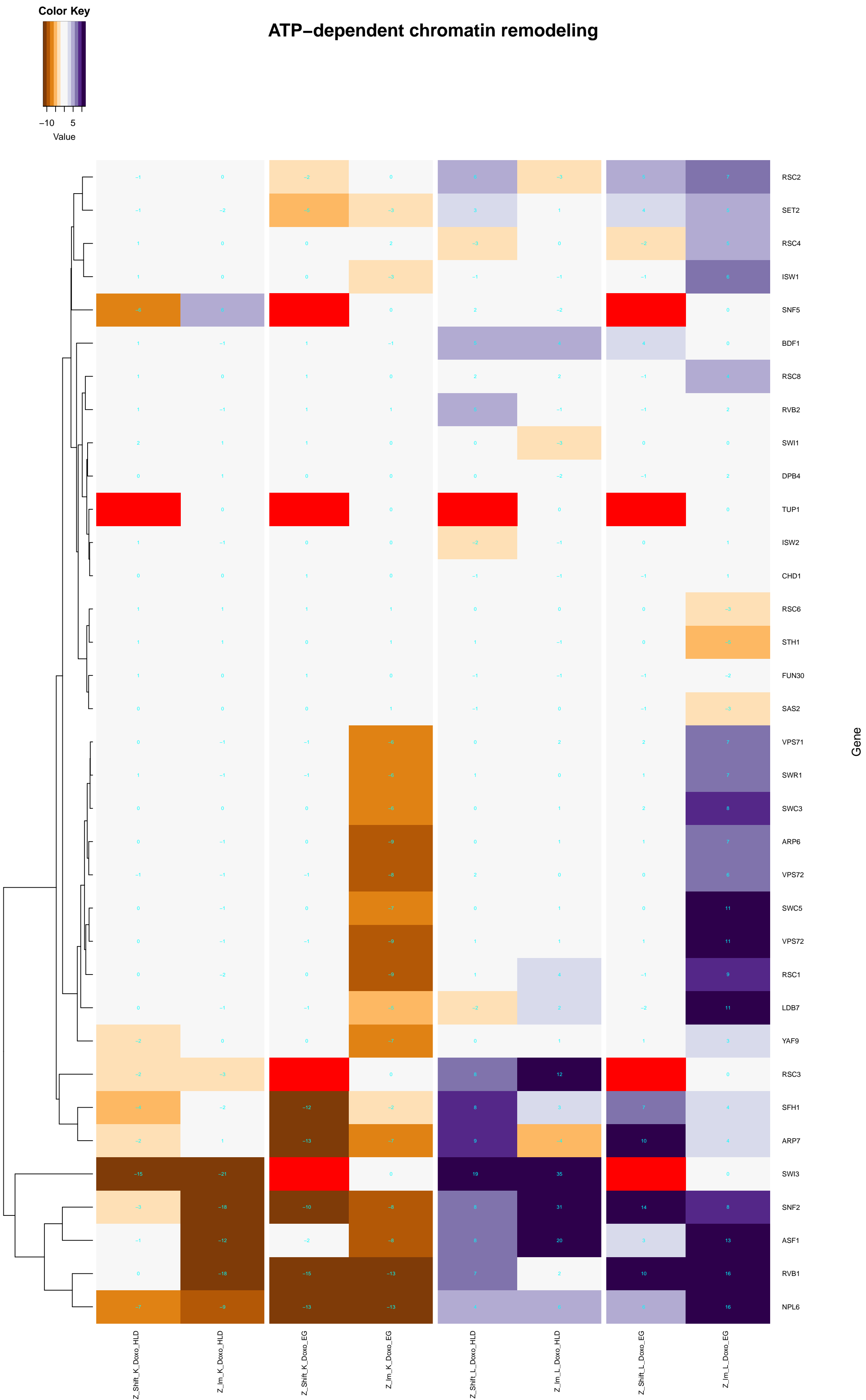

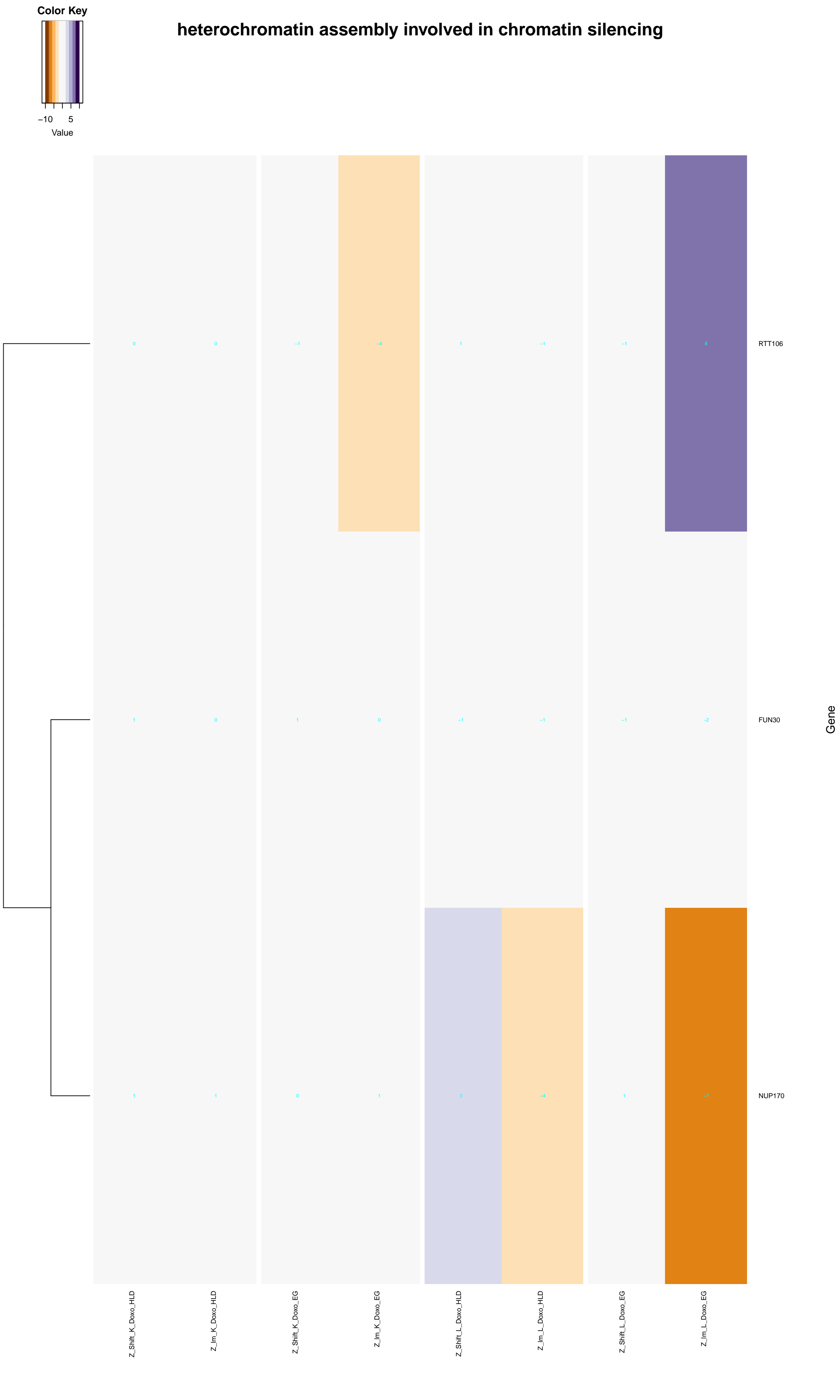

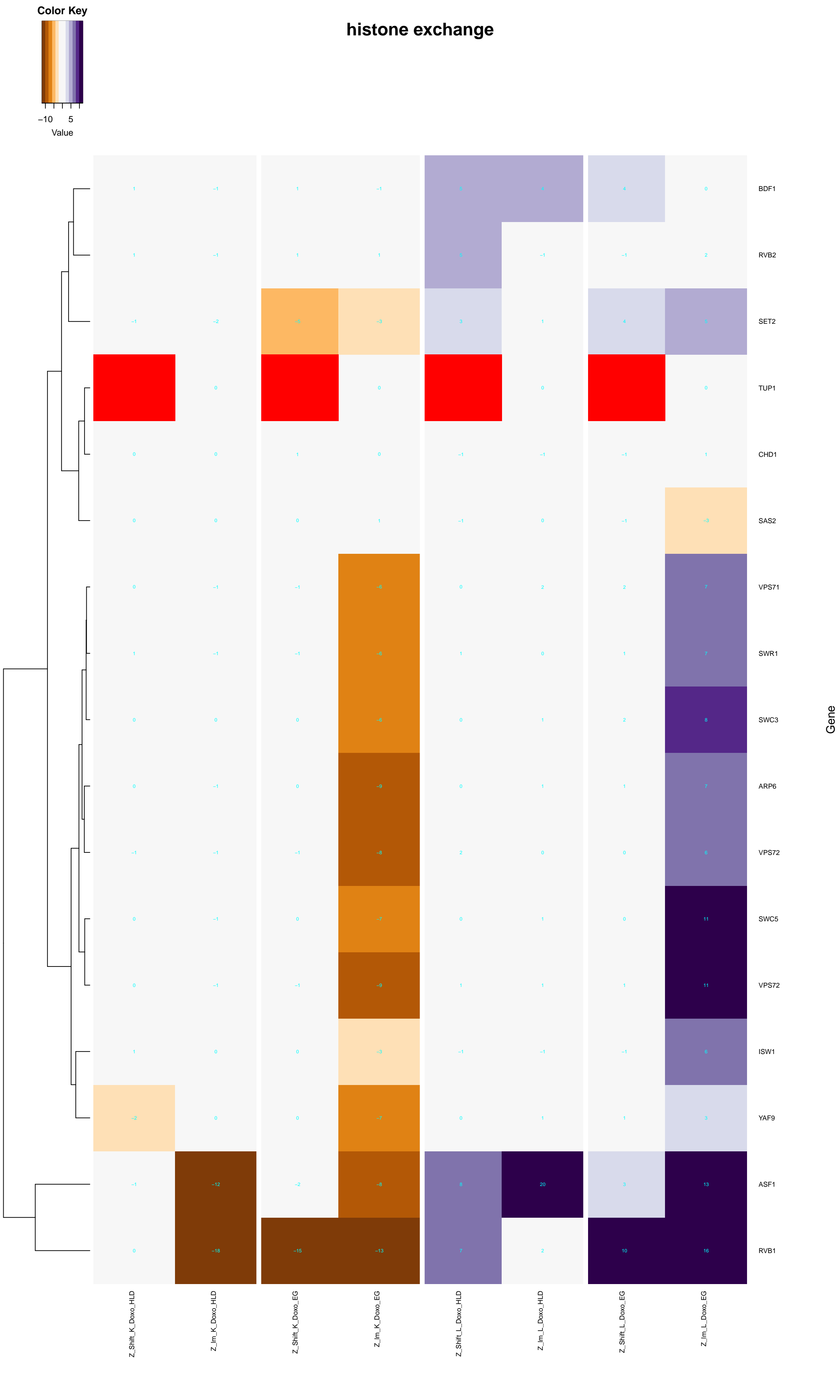

Supplement: Supplementary file 9 — Additional file 9. GO term-specific heatmaps for REMc/GTF-enriched clusters. GO term-specific heatmaps for significant GO process terms were generated as described in methods and Figs. 3 and 4. Any related child terms are presented in subsequent pages of the parent file name. GO terms with more than 100 children, with 2 or fewer genes annotated to the term, or a file size over 300KB are not shown. All heatmaps are generated with the same layout (see Figs. 3 and 4). [file 40170_2019_201_MOESM9_ESM.bz2 › Additional_File9_GOTermHeatmaps/Additional_File9_GOTermHeatmap/chromatin_remodeling.pdf]

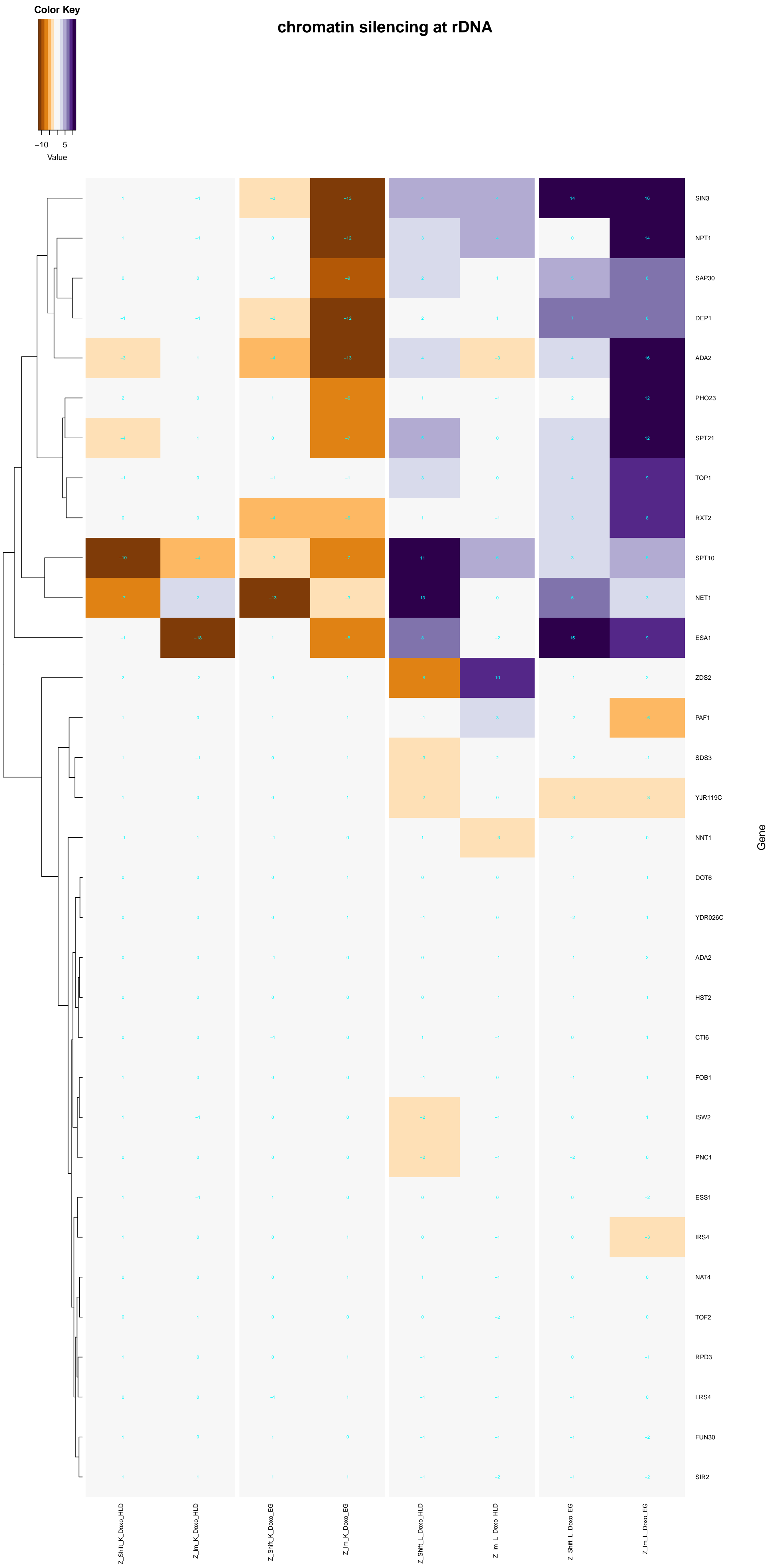



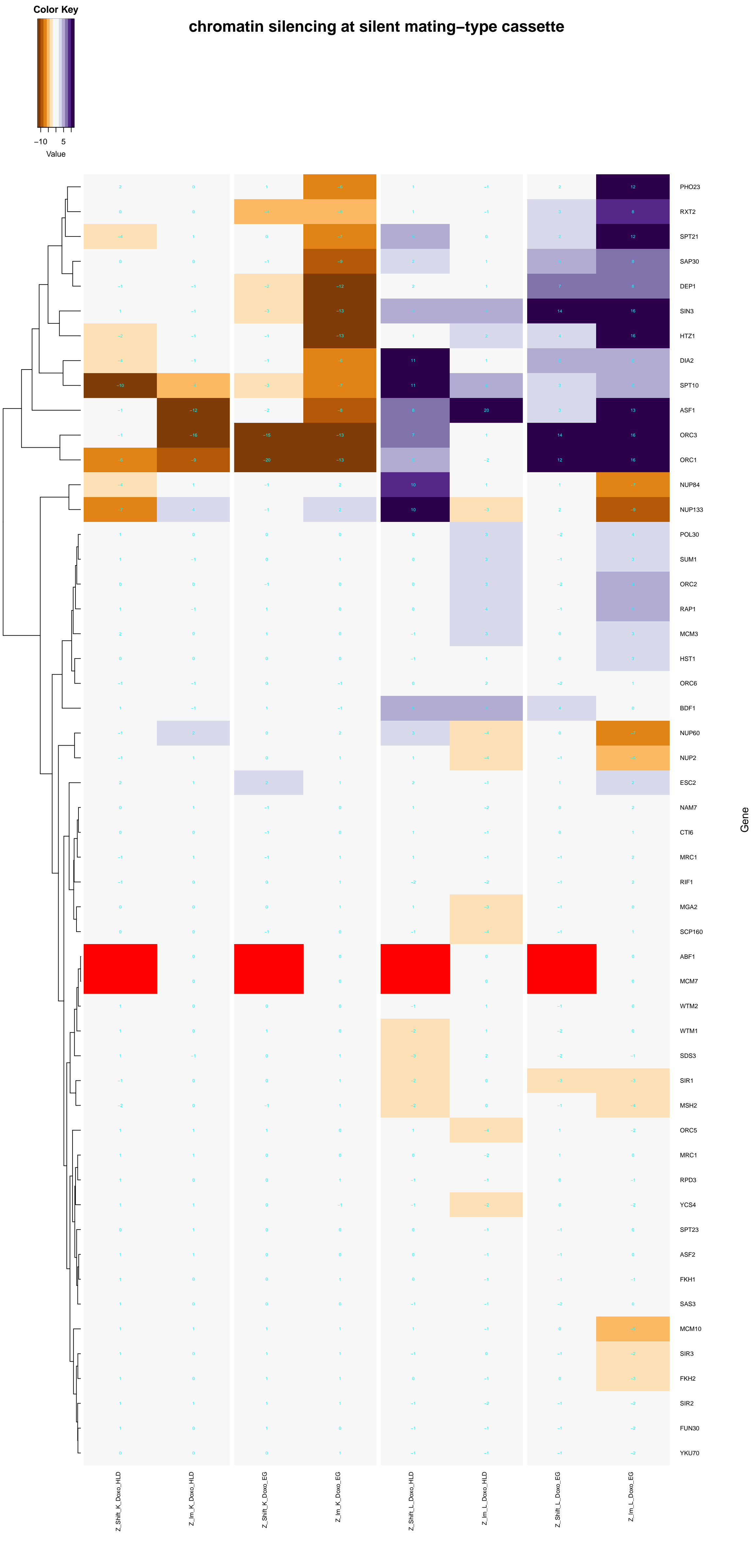

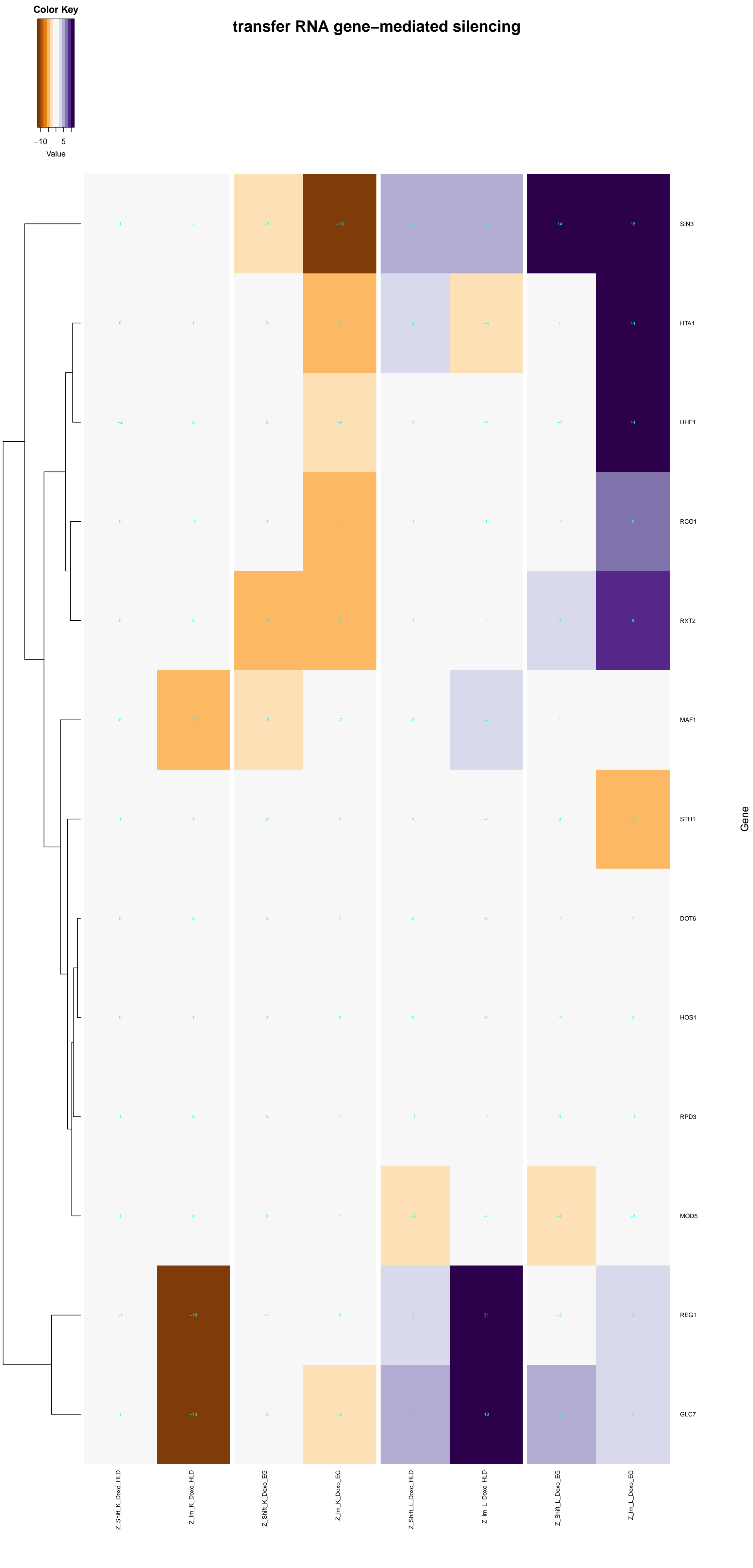

Supplement: Supplementary file 9 — Additional file 9. GO term-specific heatmaps for REMc/GTF-enriched clusters. GO term-specific heatmaps for significant GO process terms were generated as described in methods and Figs. 3 and 4. Any related child terms are presented in subsequent pages of the parent file name. GO terms with more than 100 children, with 2 or fewer genes annotated to the term, or a file size over 300KB are not shown. All heatmaps are generated with the same layout (see Figs. 3 and 4). [file 40170_2019_201_MOESM9_ESM.bz2 › Additional_File9_GOTermHeatmaps/Additional_File9_GOTermHeatmap/chromatin_silencing.pdf]

Color Key

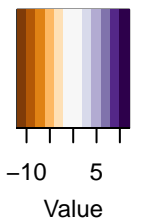

chromatin silencing at telomere

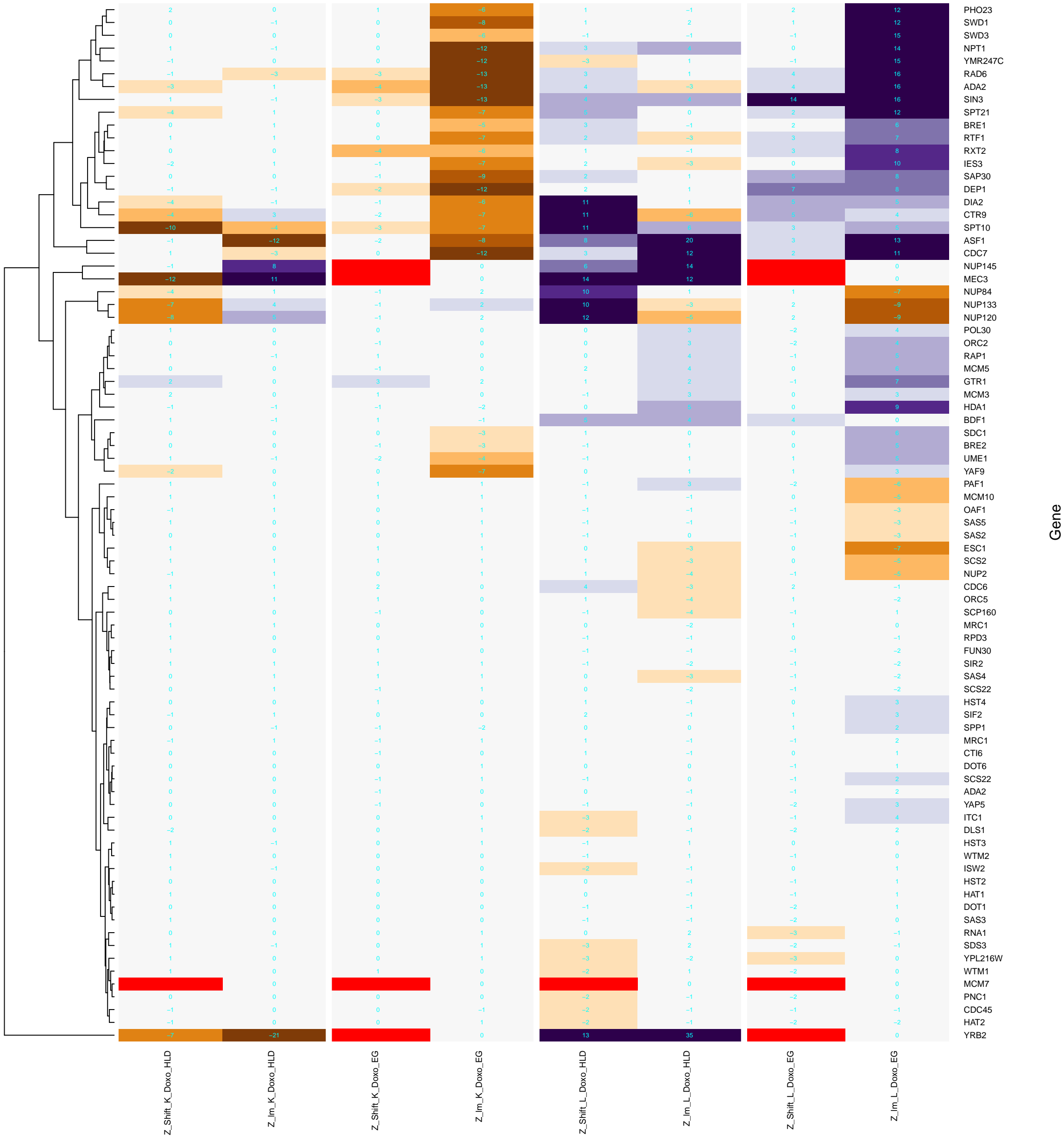

Supplement: Supplementary file 9 — Additional file 9. GO term-specific heatmaps for REMc/GTF-enriched clusters. GO term-specific heatmaps for significant GO process terms were generated as described in methods and Figs. 3 and 4. Any related child terms are presented in subsequent pages of the parent file name. GO terms with more than 100 children, with 2 or fewer genes annotated to the term, or a file size over 300KB are not shown. All heatmaps are generated with the same layout (see Figs. 3 and 4). [file 40170_2019_201_MOESM9_ESM.bz2 › Additional_File9_GOTermHeatmaps/Additional_File9_GOTermHeatmap/chromatin_silencing_at_telomere.pdf]

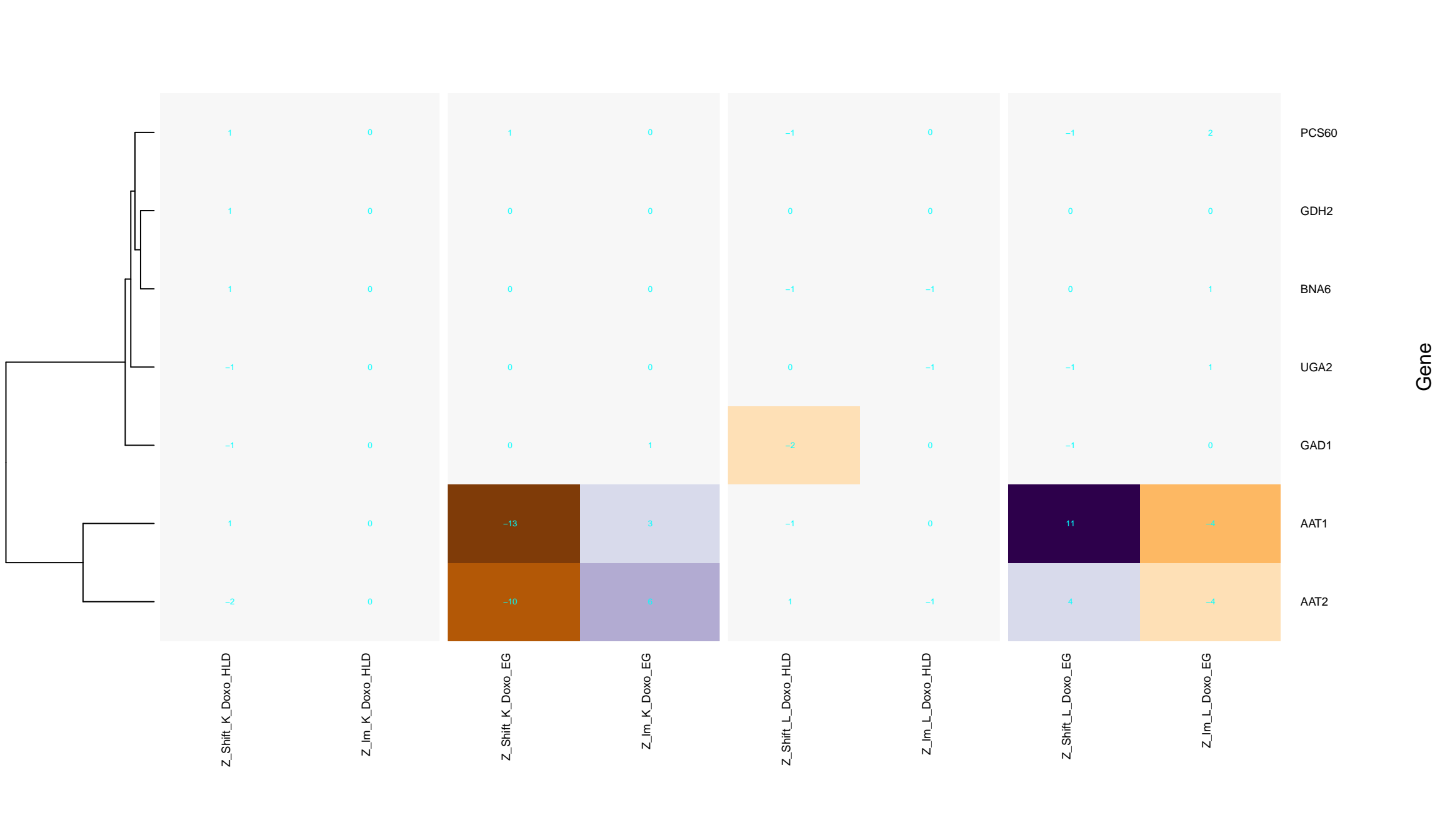

Supplement: Supplementary file 9 — Additional file 9. GO term-specific heatmaps for REMc/GTF-enriched clusters. GO term-specific heatmaps for significant GO process terms were generated as described in methods and Figs. 3 and 4. Any related child terms are presented in subsequent pages of the parent file name. GO terms with more than 100 children, with 2 or fewer genes annotated to the term, or a file size over 300KB are not shown. All heatmaps are generated with the same layout (see Figs. 3 and 4). [file 40170_2019_201_MOESM9_ESM.bz2 › Additional_File9_GOTermHeatmaps/Additional_File9_GOTermHeatmap/dicarboxylic_acid_catabolic_process.pdf]

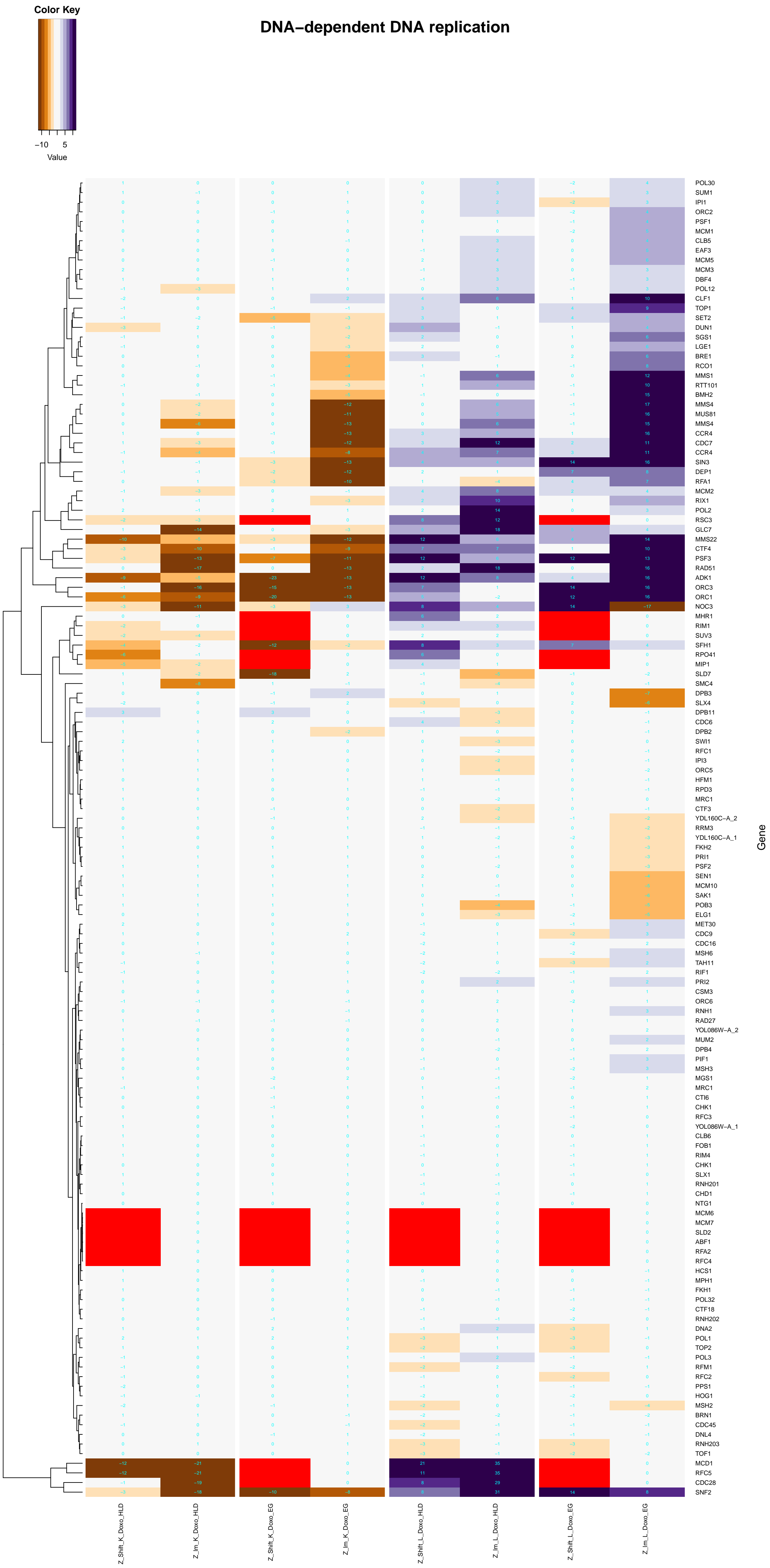

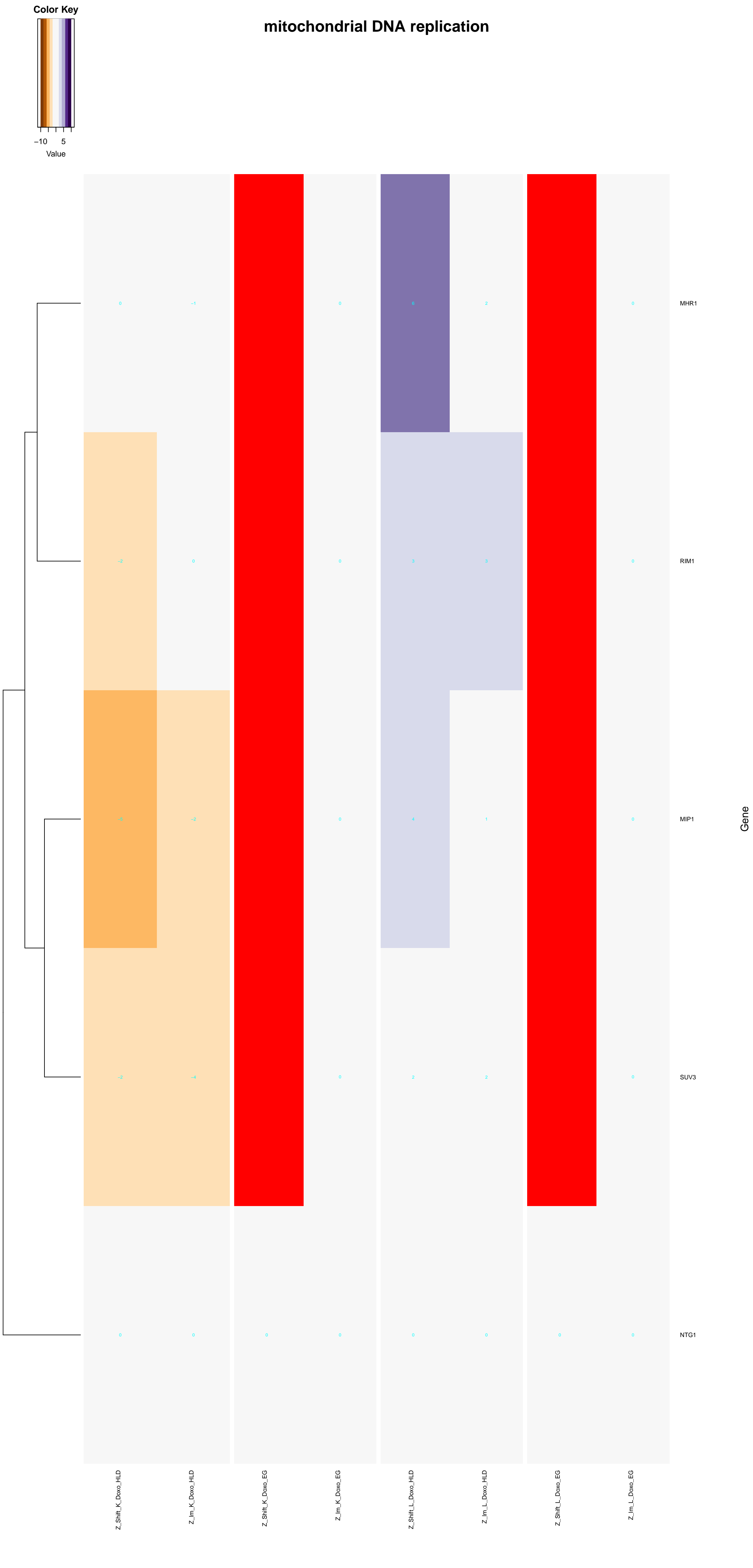

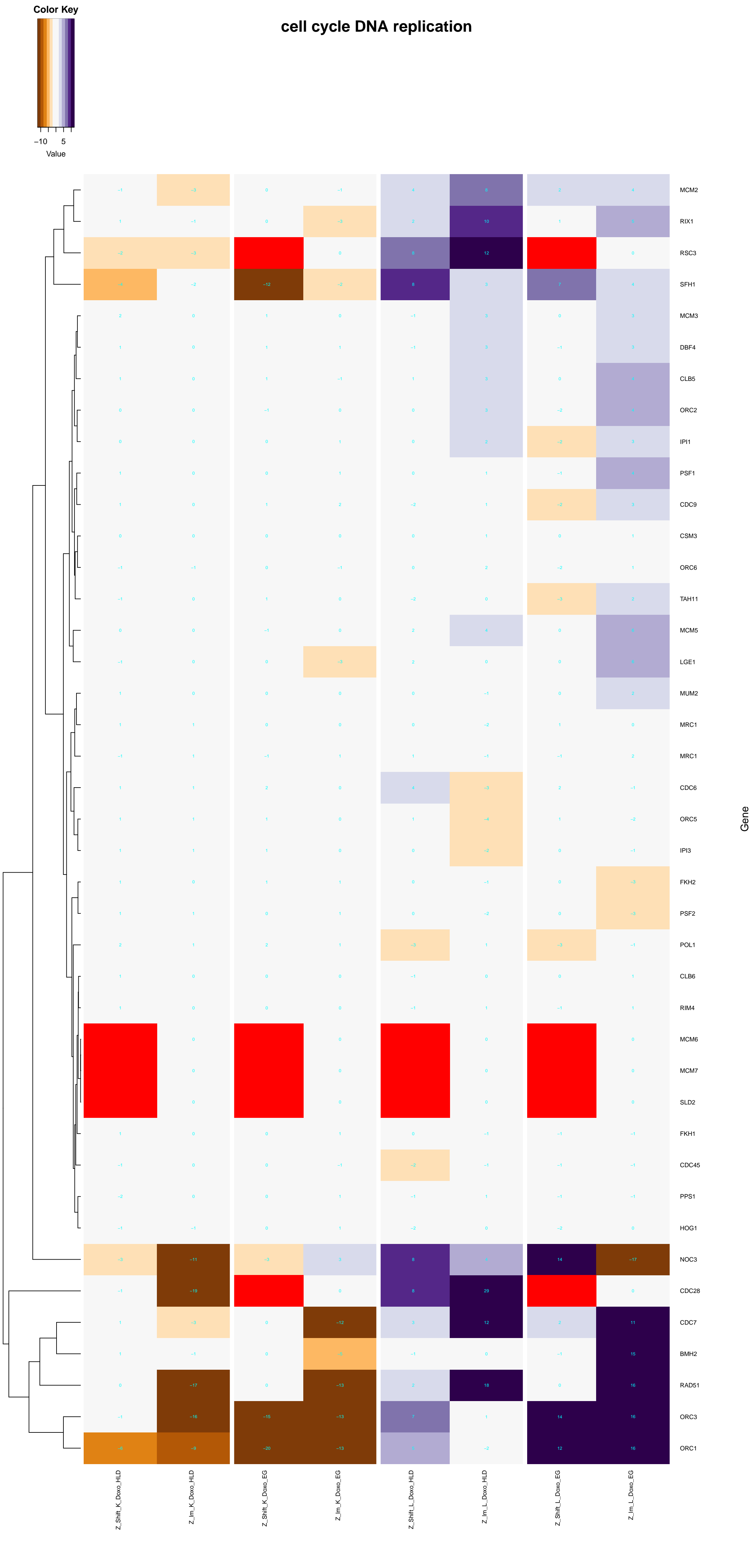

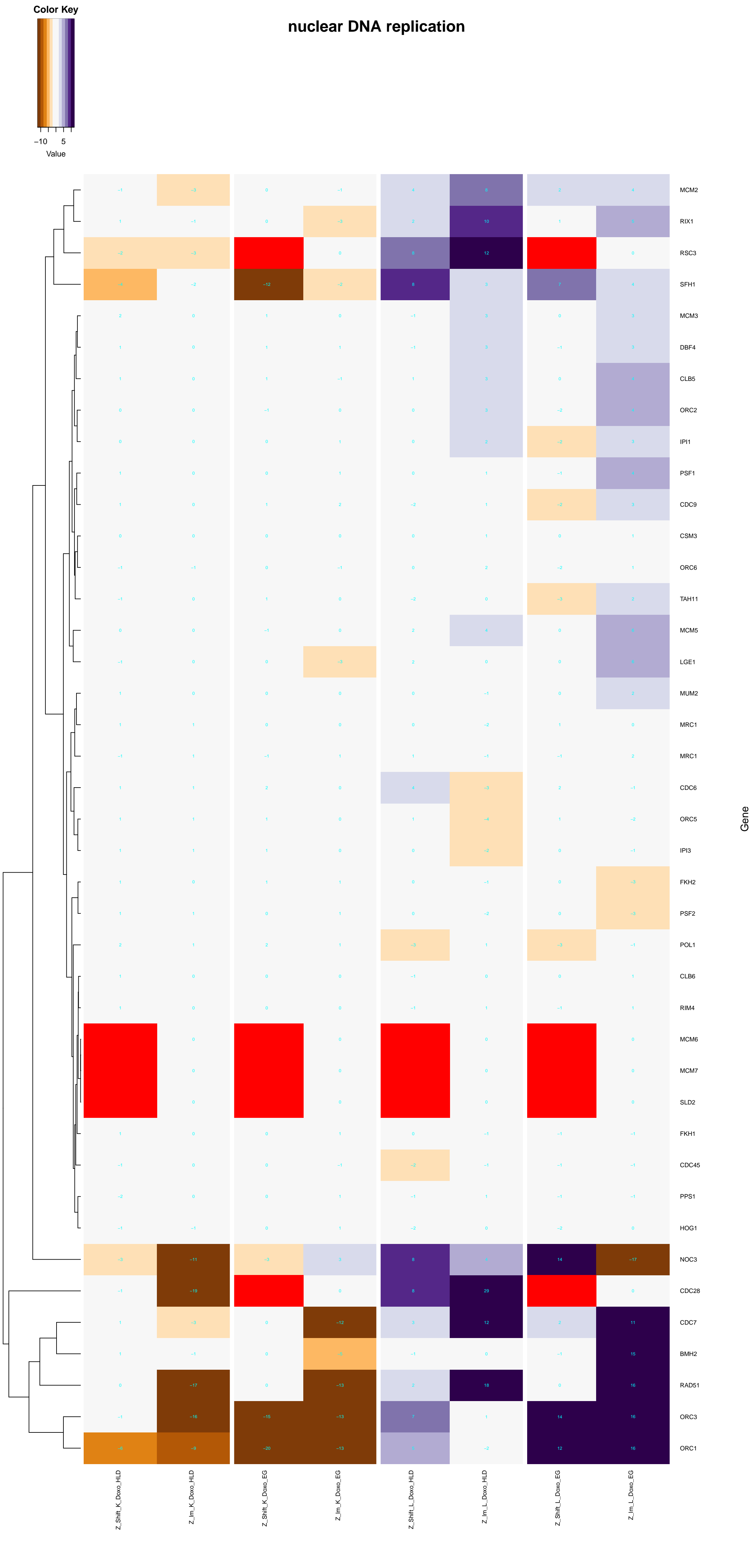

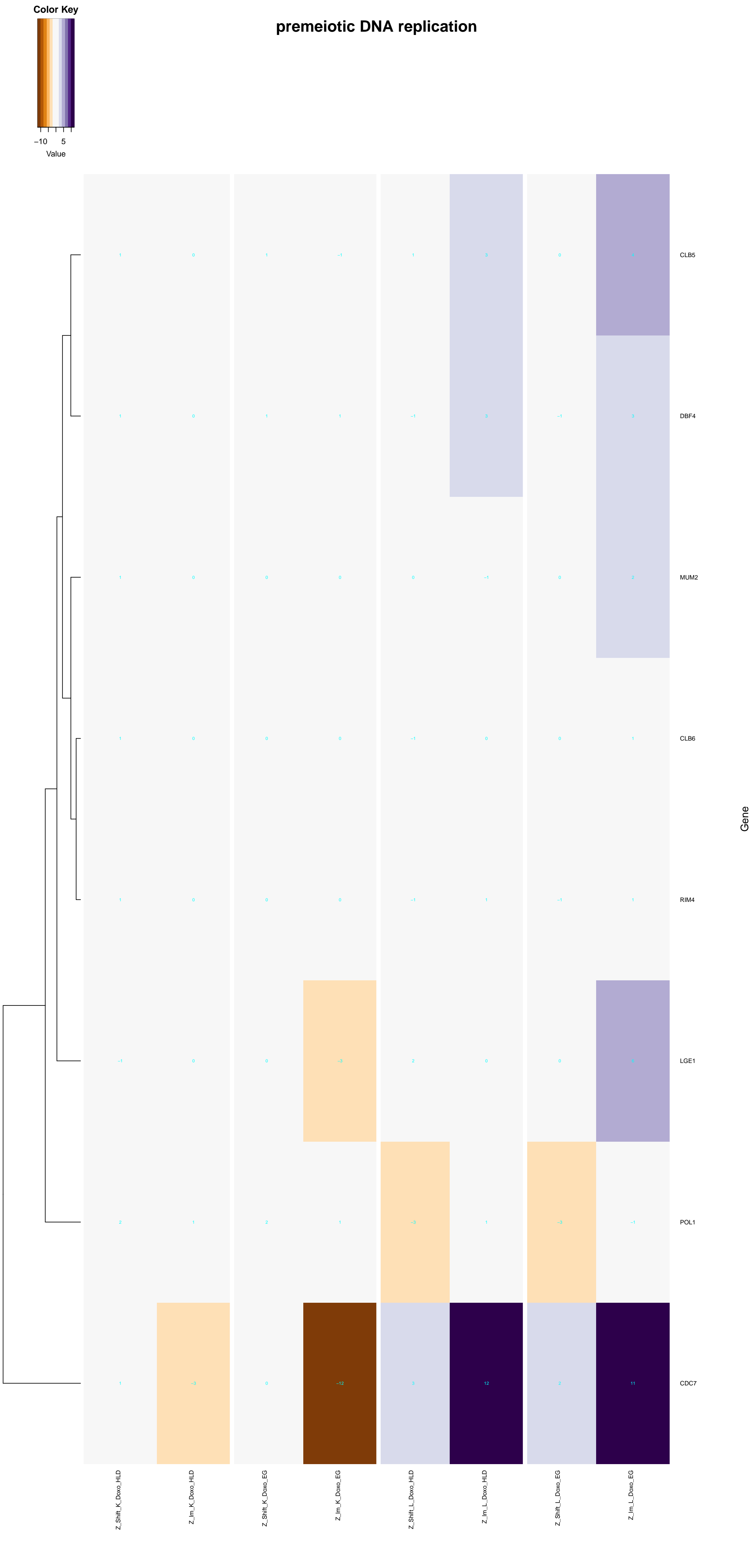

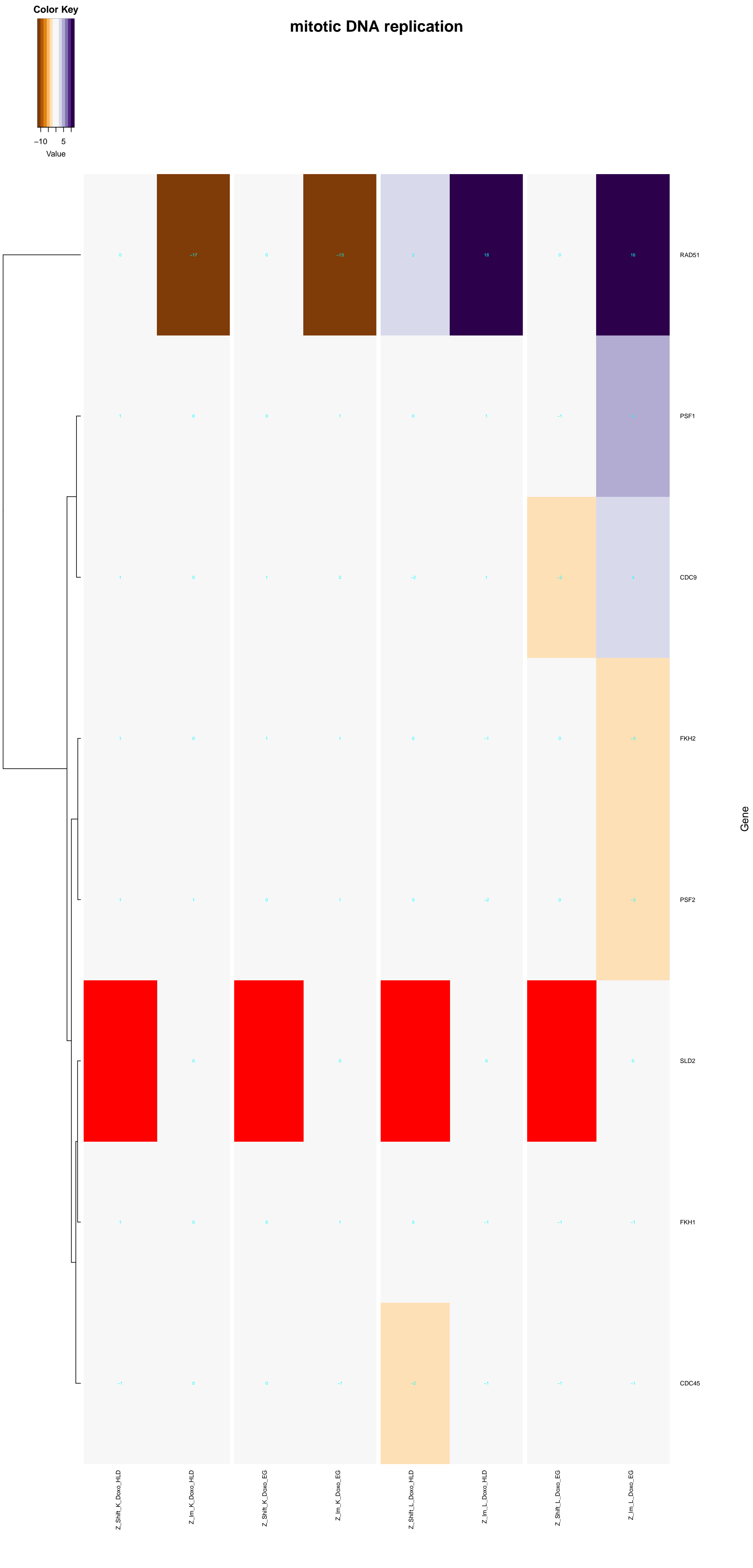

Supplement: Supplementary file 9 — Additional file 9. GO term-specific heatmaps for REMc/GTF-enriched clusters. GO term-specific heatmaps for significant GO process terms were generated as described in methods and Figs. 3 and 4. Any related child terms are presented in subsequent pages of the parent file name. GO terms with more than 100 children, with 2 or fewer genes annotated to the term, or a file size over 300KB are not shown. All heatmaps are generated with the same layout (see Figs. 3 and 4). [file 40170_2019_201_MOESM9_ESM.bz2 › Additional_File9_GOTermHeatmaps/Additional_File9_GOTermHeatmap/DNA-dependent_DNA_replication.pdf]

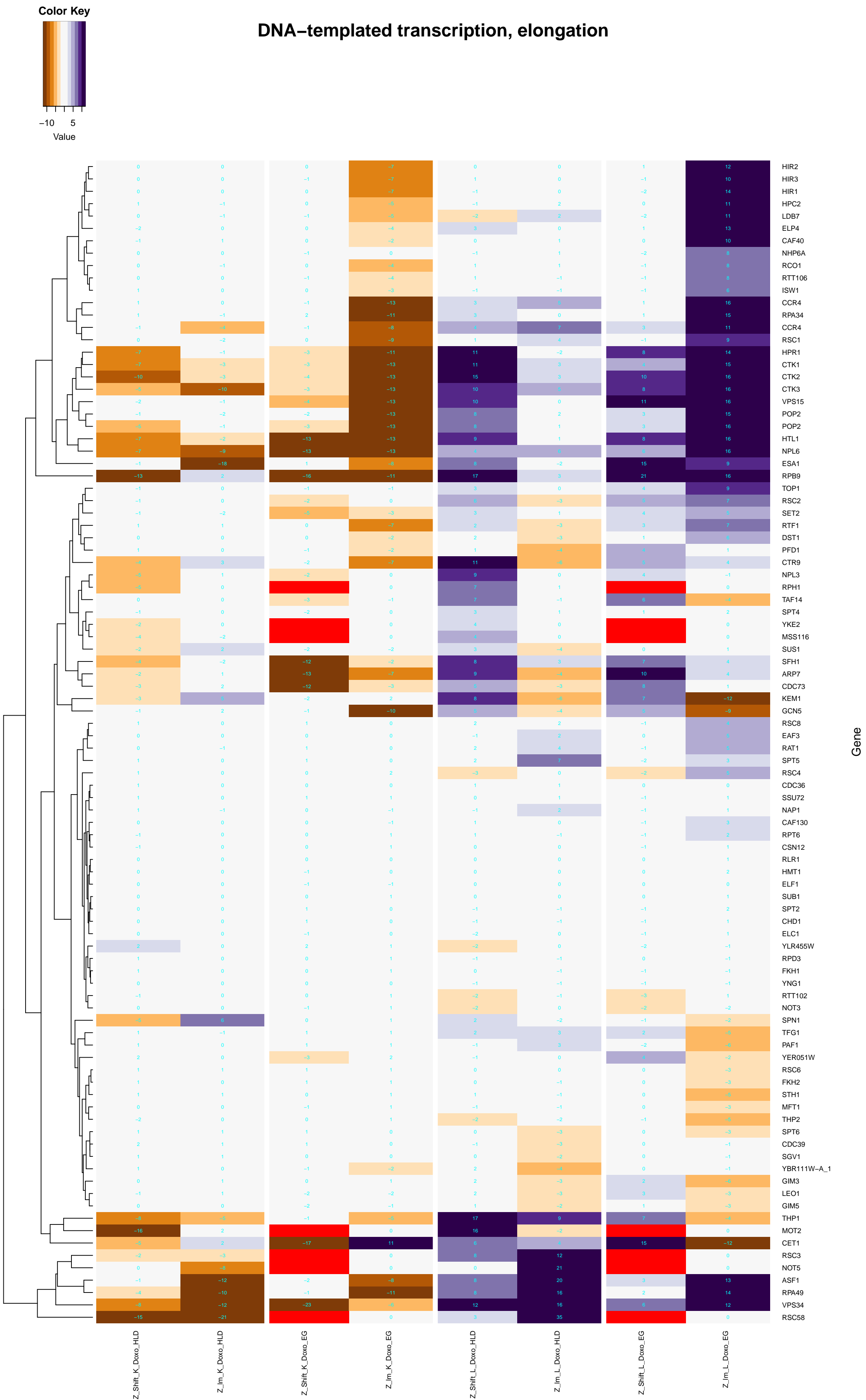

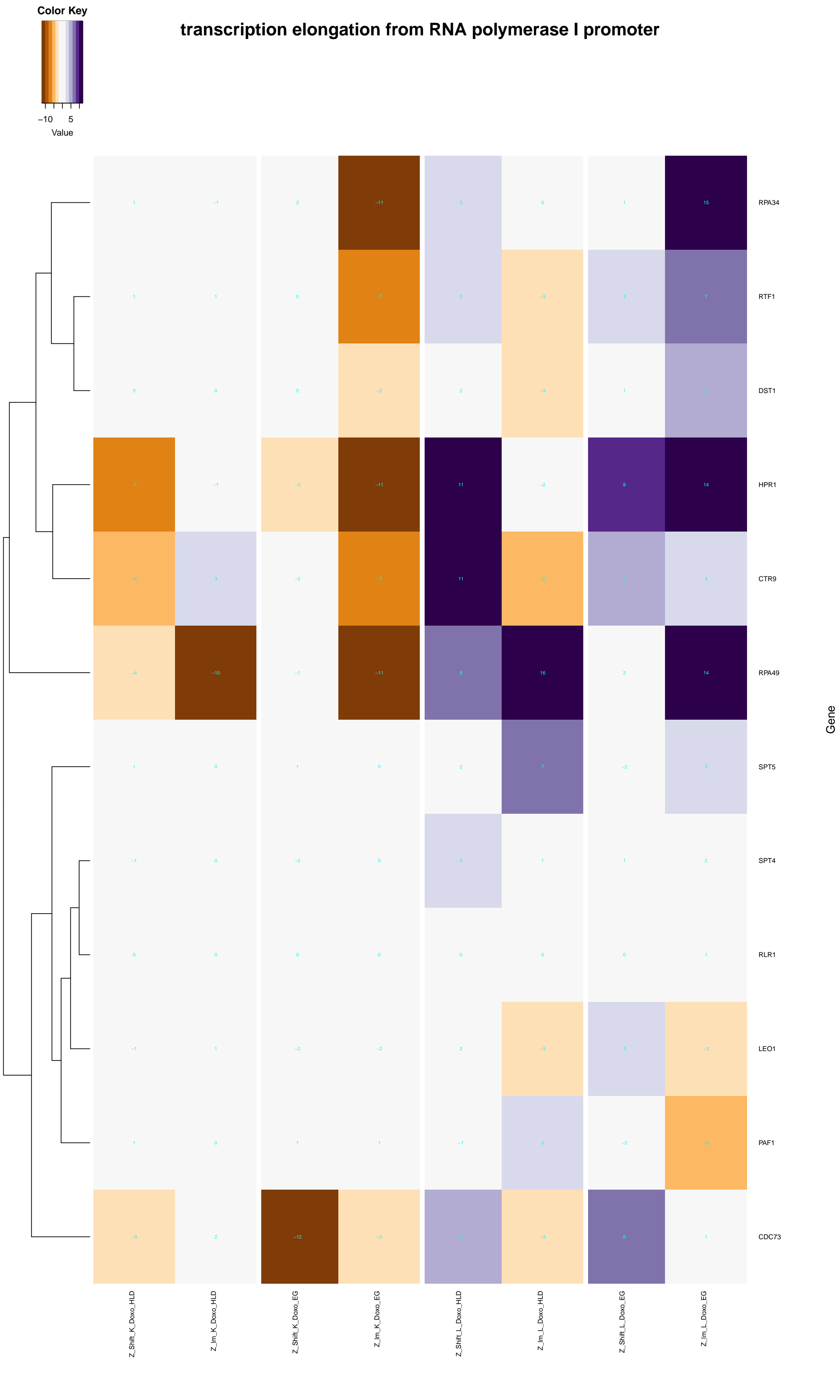

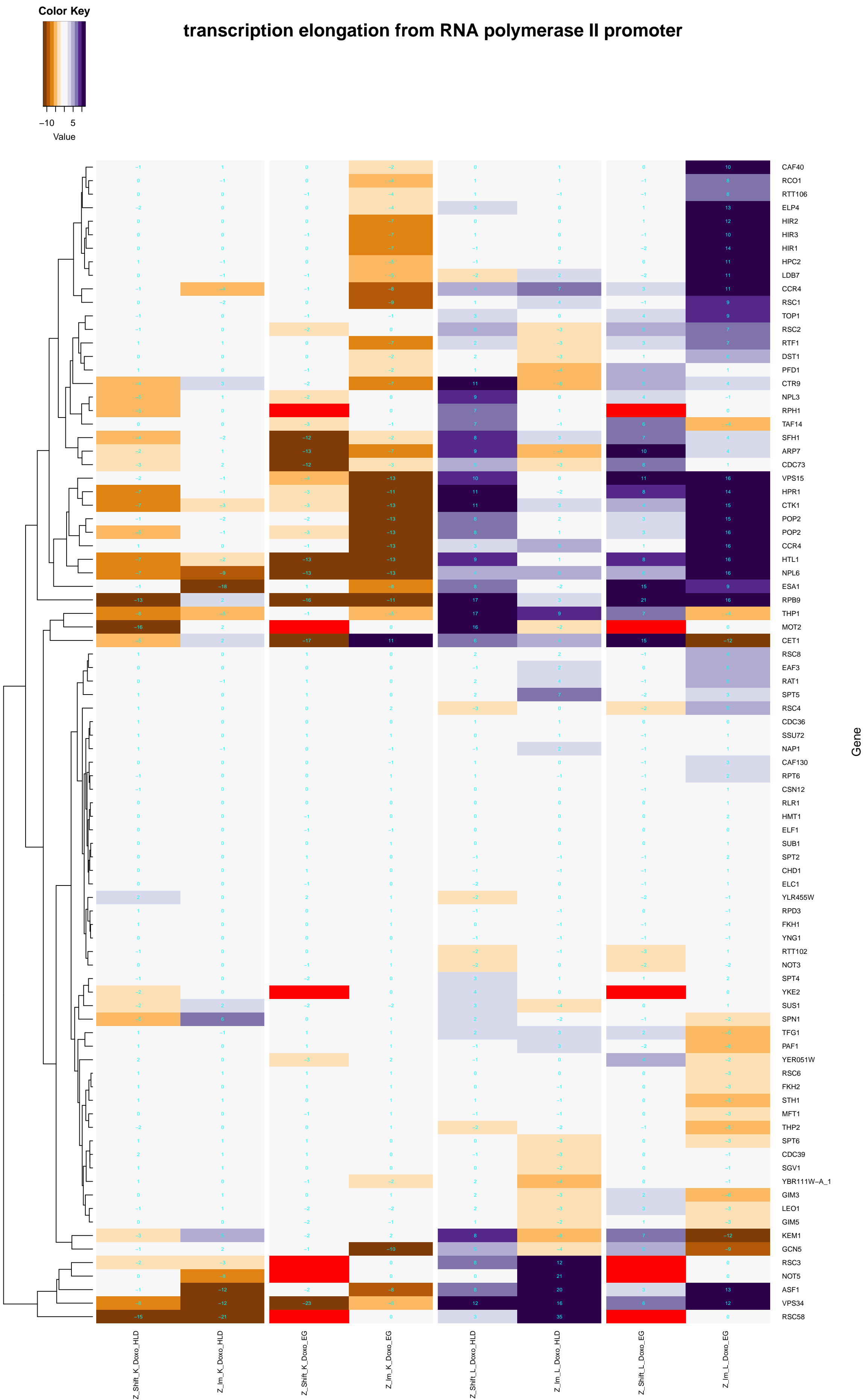

Supplement: Supplementary file 9 — Additional file 9. GO term-specific heatmaps for REMc/GTF-enriched clusters. GO term-specific heatmaps for significant GO process terms were generated as described in methods and Figs. 3 and 4. Any related child terms are presented in subsequent pages of the parent file name. GO terms with more than 100 children, with 2 or fewer genes annotated to the term, or a file size over 300KB are not shown. All heatmaps are generated with the same layout (see Figs. 3 and 4). [file 40170_2019_201_MOESM9_ESM.bz2 › Additional_File9_GOTermHeatmaps/Additional_File9_GOTermHeatmap/DNA-templated_transcription,_elongation.pdf]

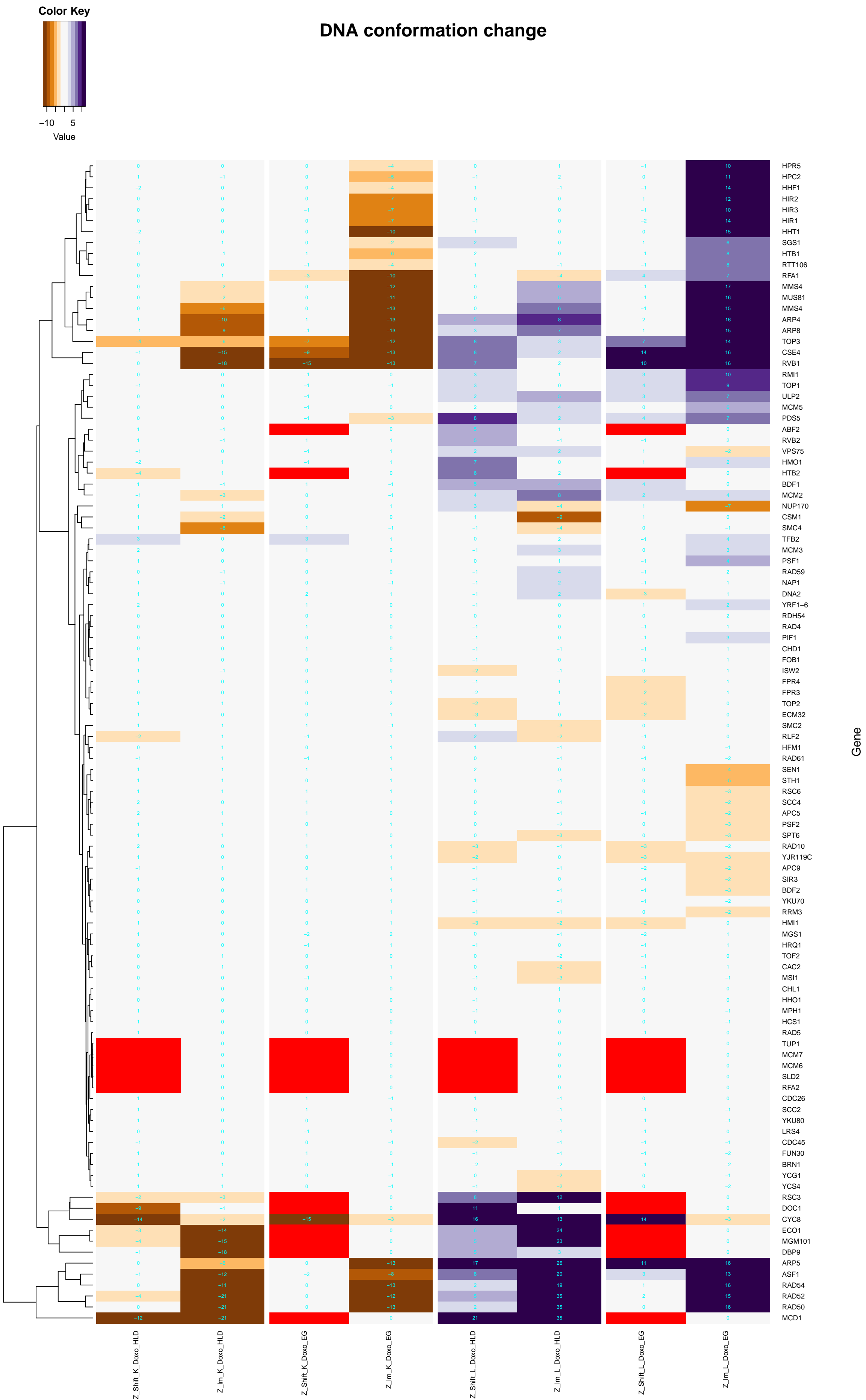

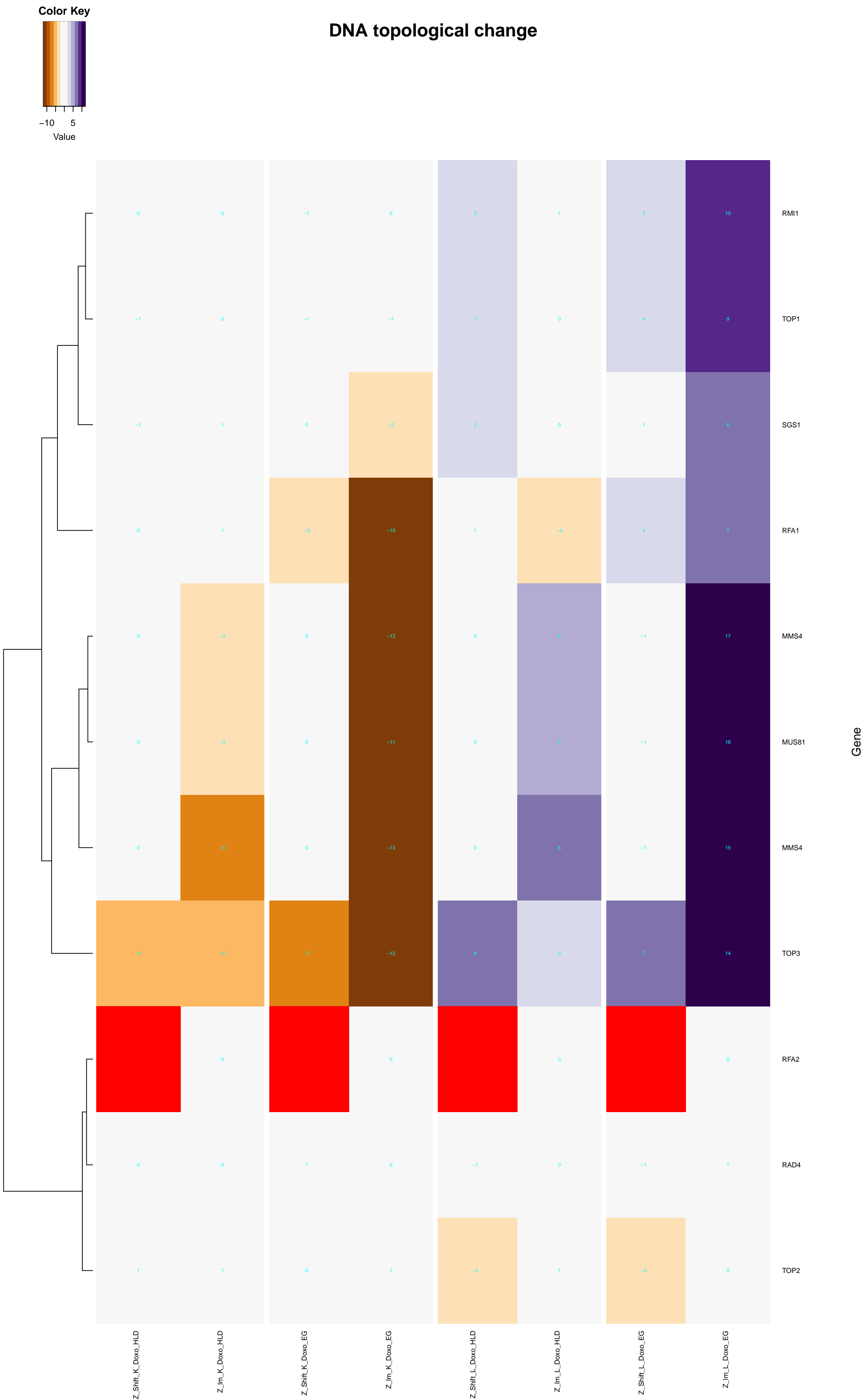

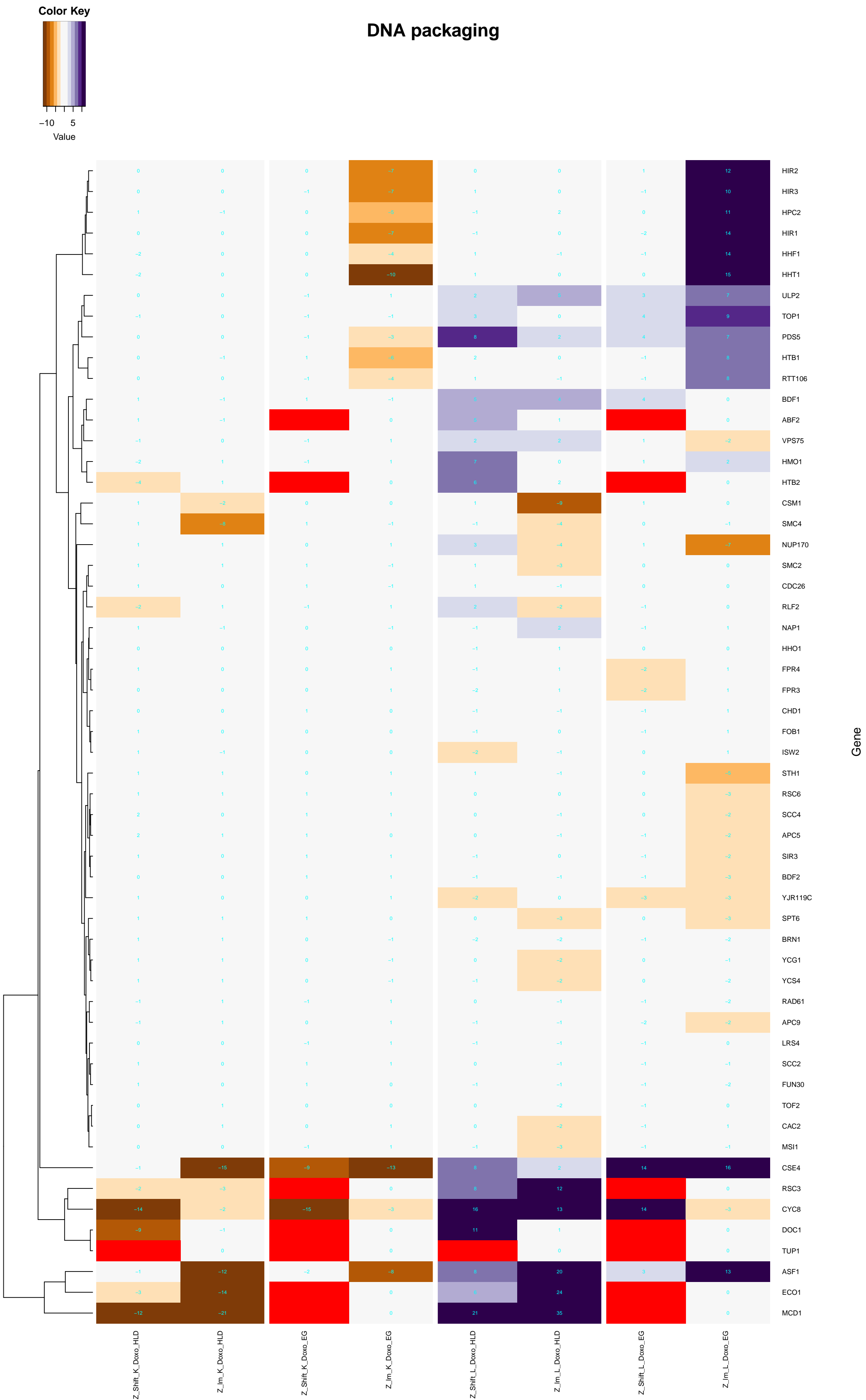

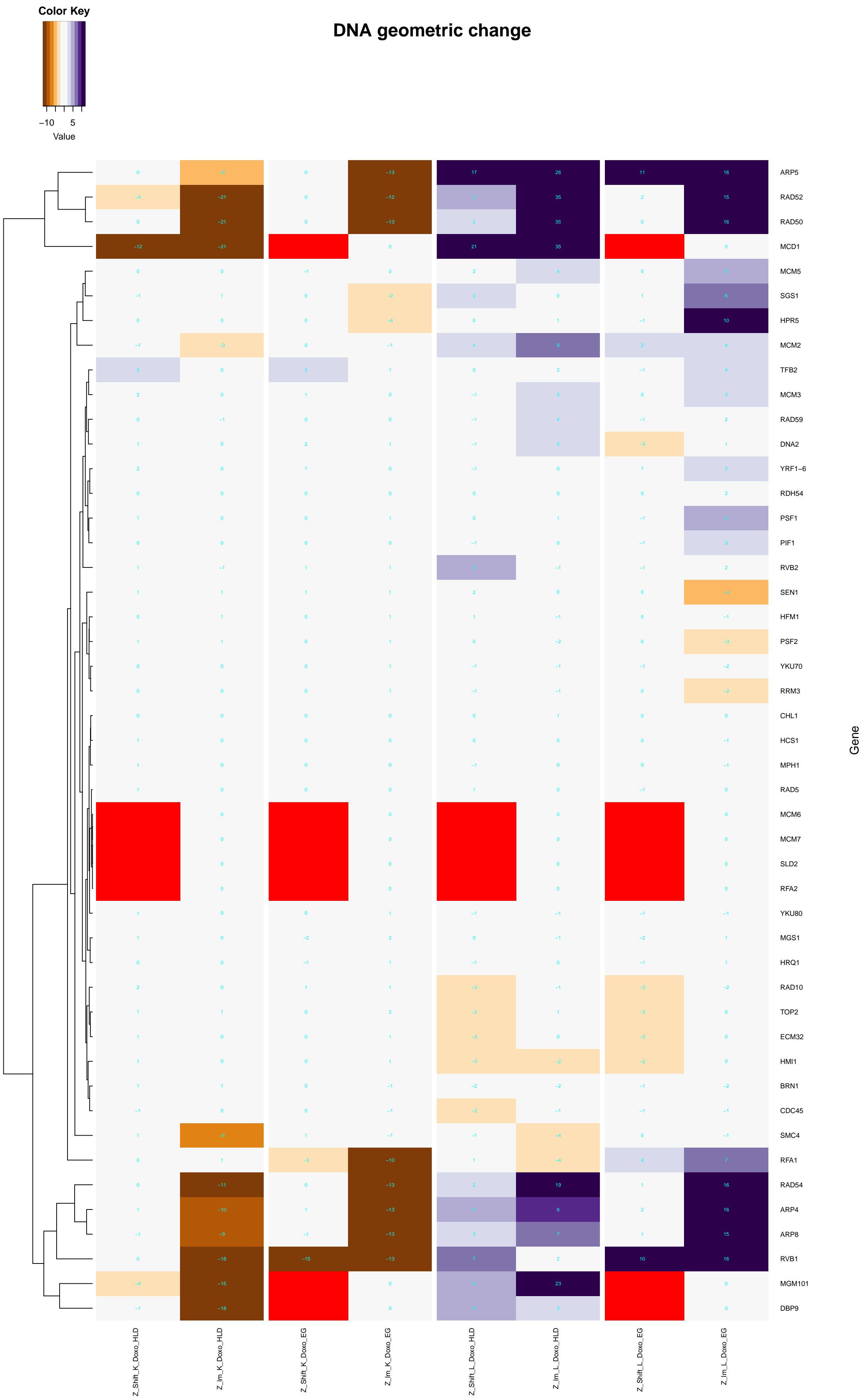

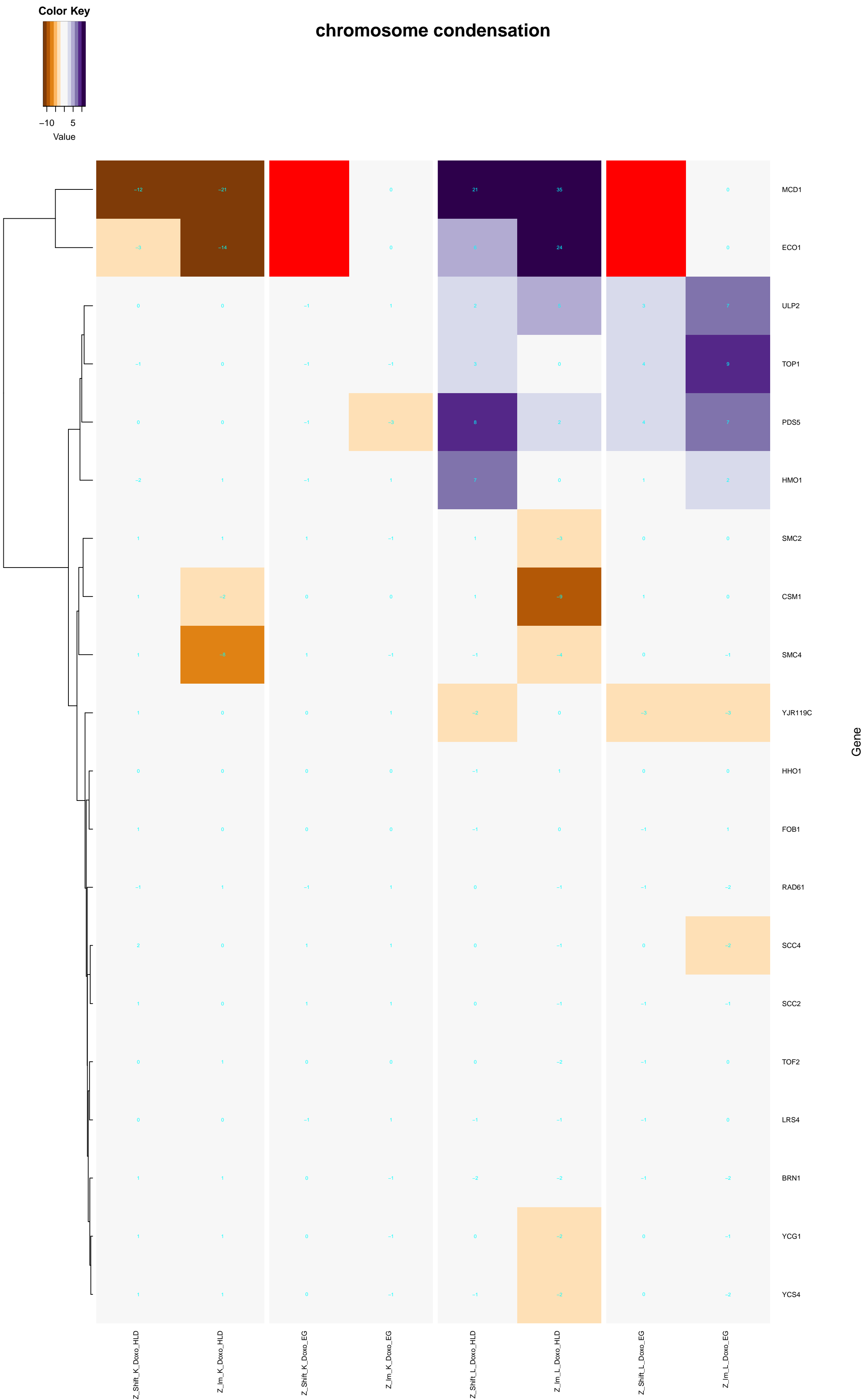

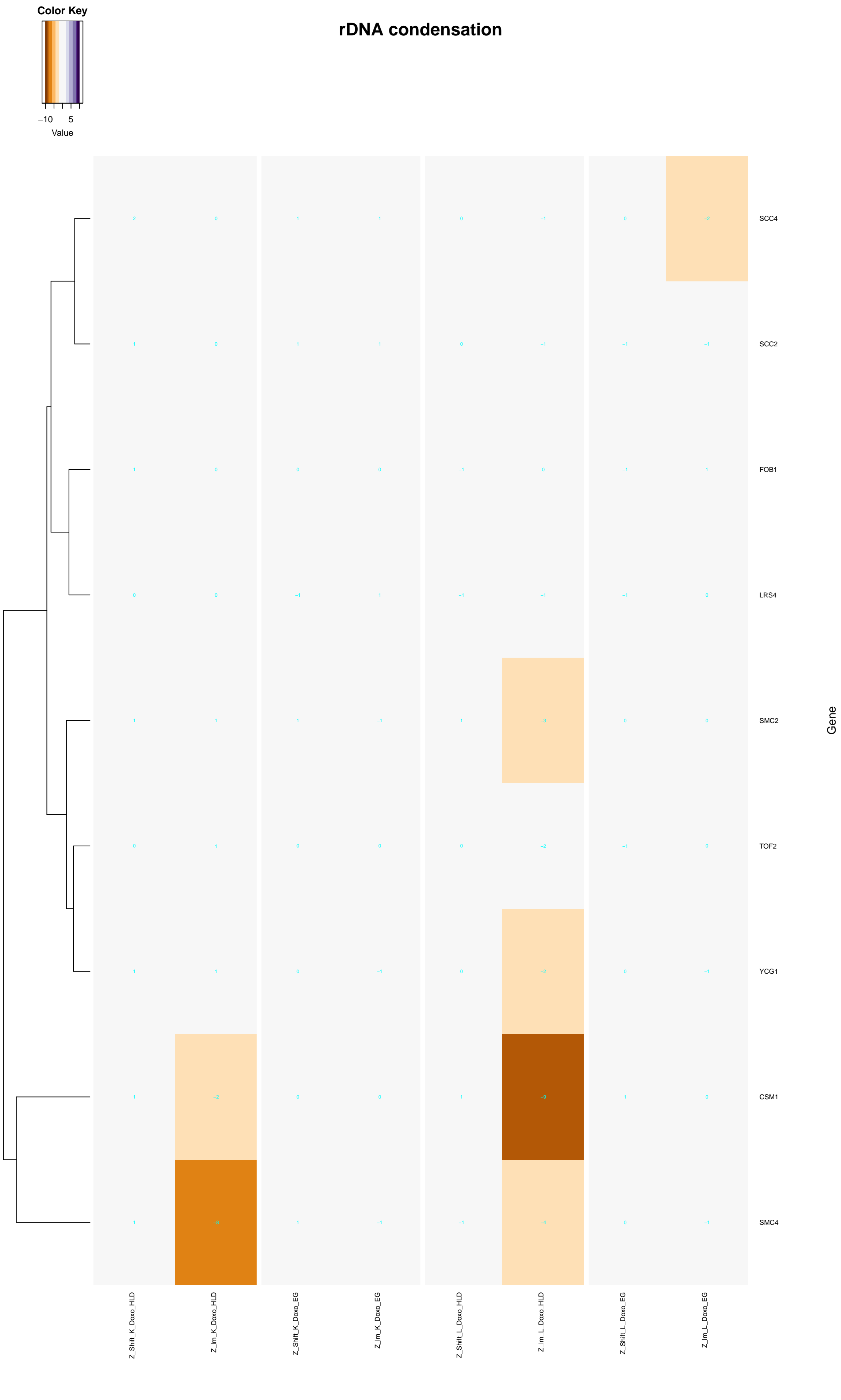

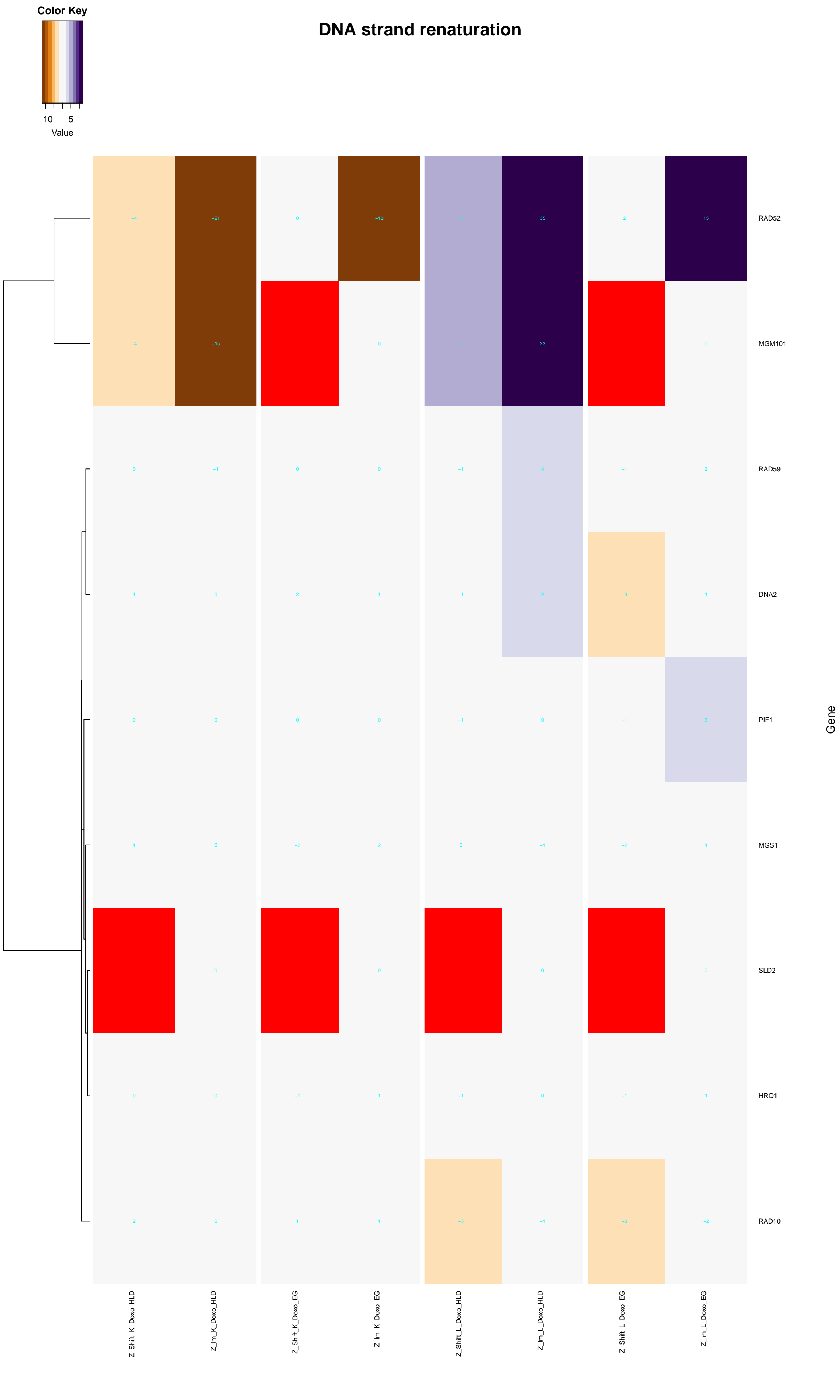

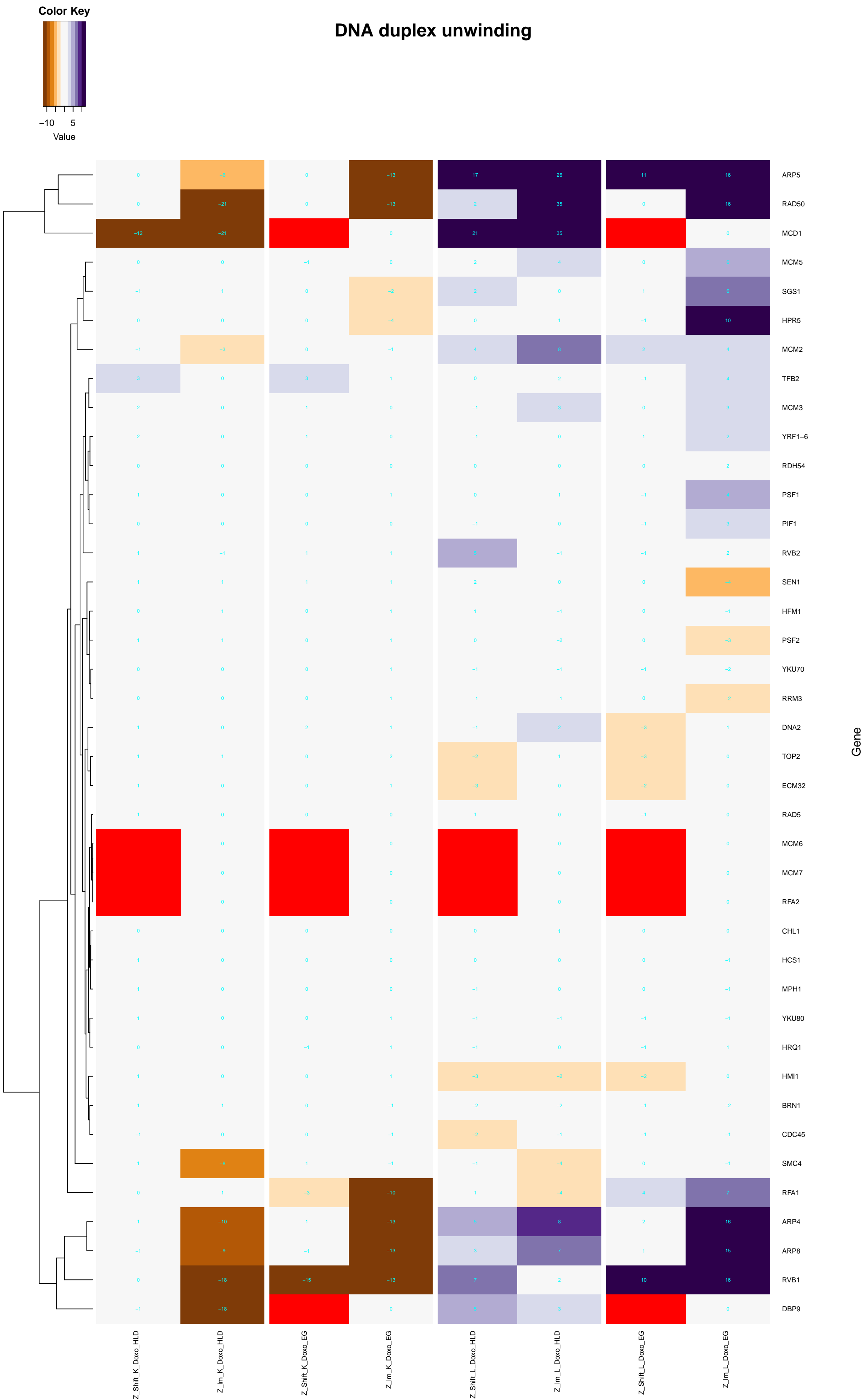

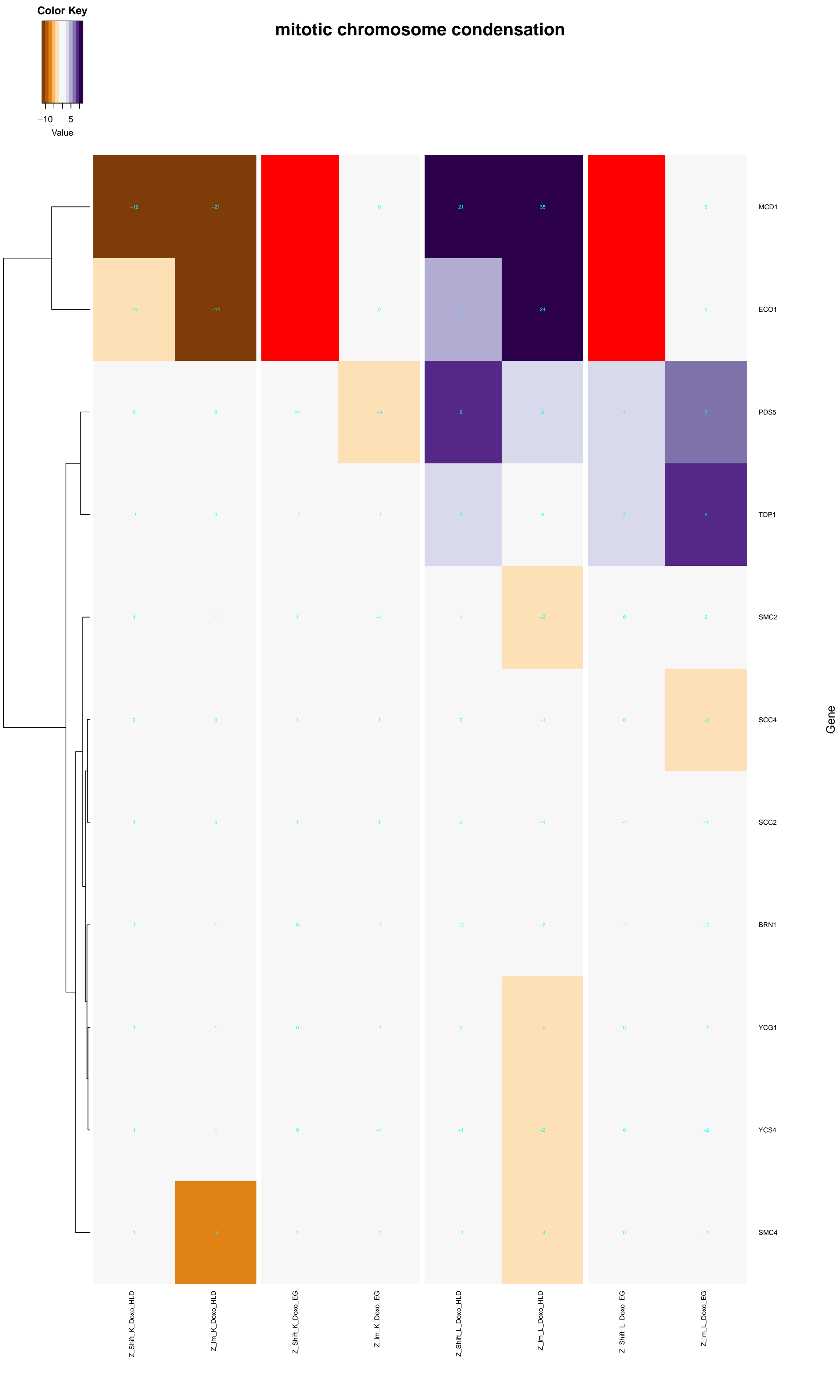

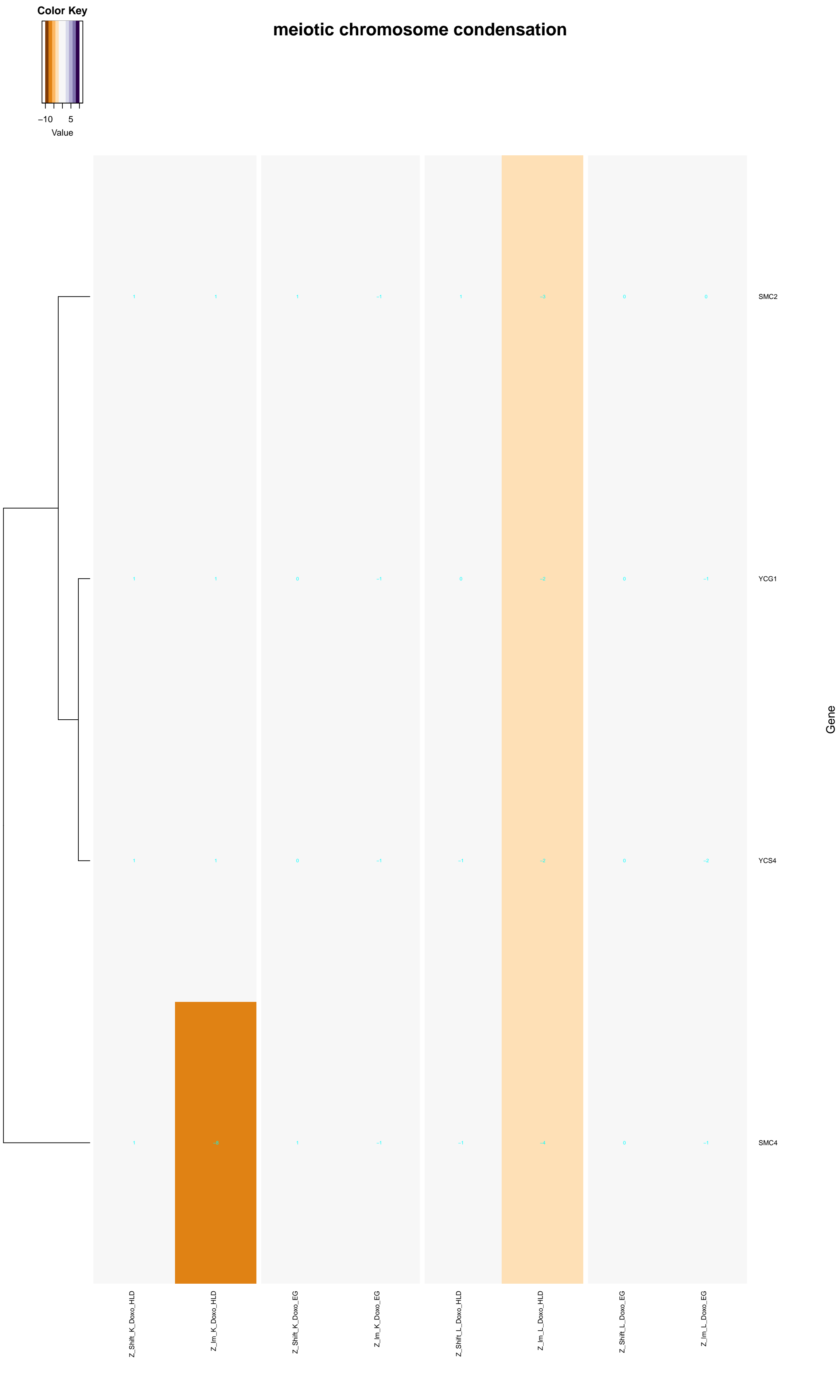

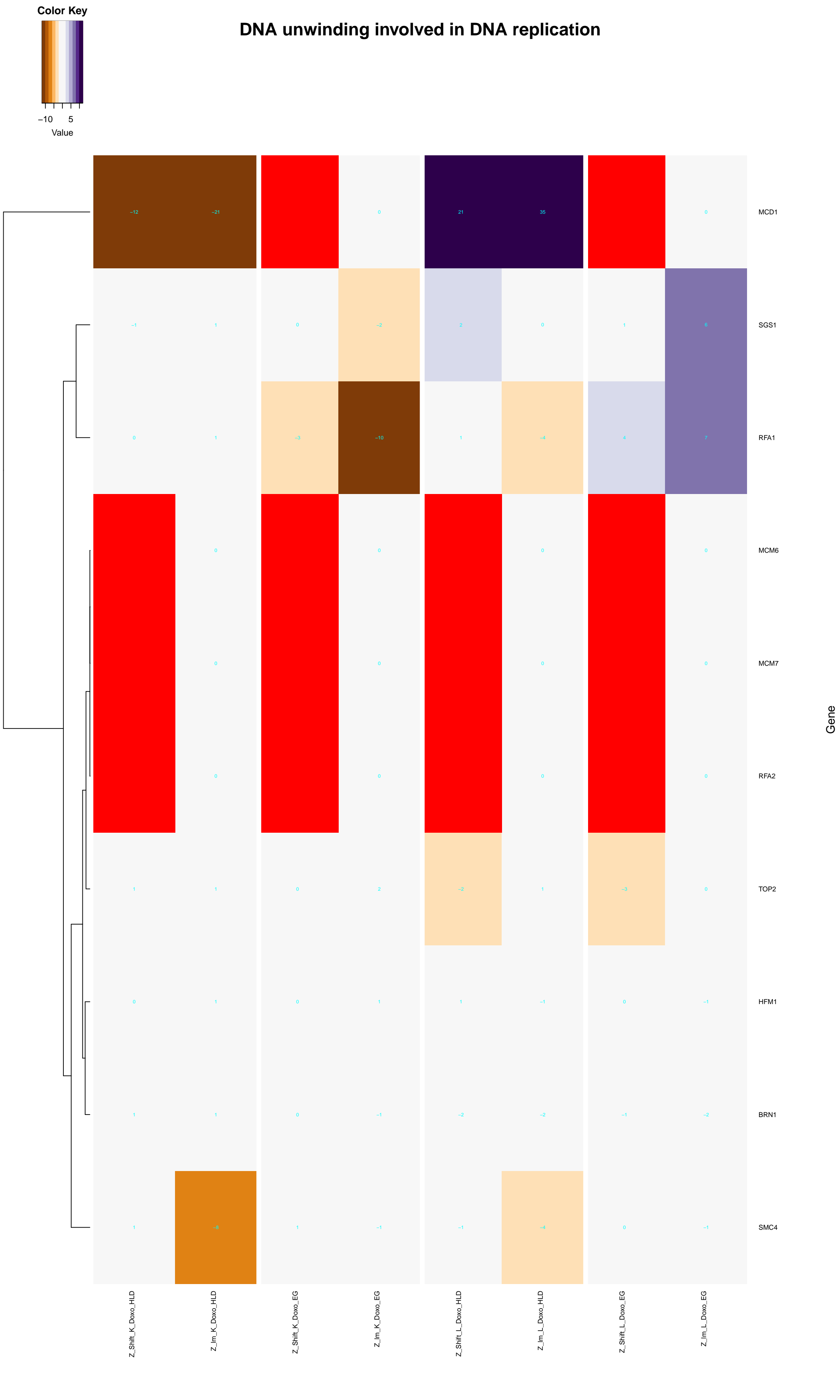

Supplement: Supplementary file 9 — Additional file 9. GO term-specific heatmaps for REMc/GTF-enriched clusters. GO term-specific heatmaps for significant GO process terms were generated as described in methods and Figs. 3 and 4. Any related child terms are presented in subsequent pages of the parent file name. GO terms with more than 100 children, with 2 or fewer genes annotated to the term, or a file size over 300KB are not shown. All heatmaps are generated with the same layout (see Figs. 3 and 4). [file 40170_2019_201_MOESM9_ESM.bz2 › Additional_File9_GOTermHeatmaps/Additional_File9_GOTermHeatmap/DNA_conformation_change.pdf]

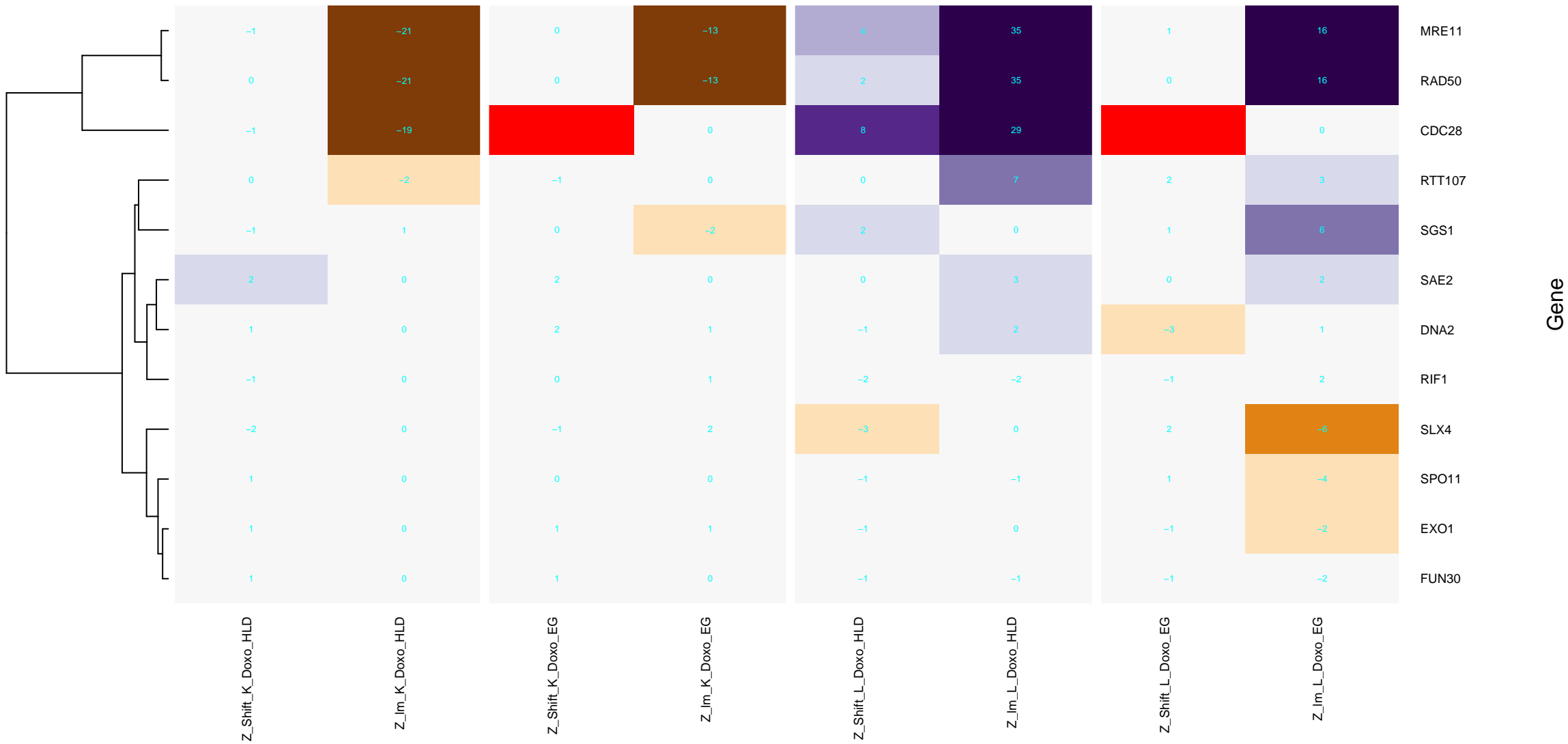

Supplement: Supplementary file 9 — Additional file 9. GO term-specific heatmaps for REMc/GTF-enriched clusters. GO term-specific heatmaps for significant GO process terms were generated as described in methods and Figs. 3 and 4. Any related child terms are presented in subsequent pages of the parent file name. GO terms with more than 100 children, with 2 or fewer genes annotated to the term, or a file size over 300KB are not shown. All heatmaps are generated with the same layout (see Figs. 3 and 4). [file 40170_2019_201_MOESM9_ESM.bz2 › Additional_File9_GOTermHeatmaps/Additional_File9_GOTermHeatmap/DNA_double-strand_break_processing.pdf]

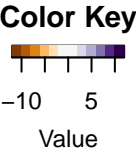

# DNA duplex unwinding

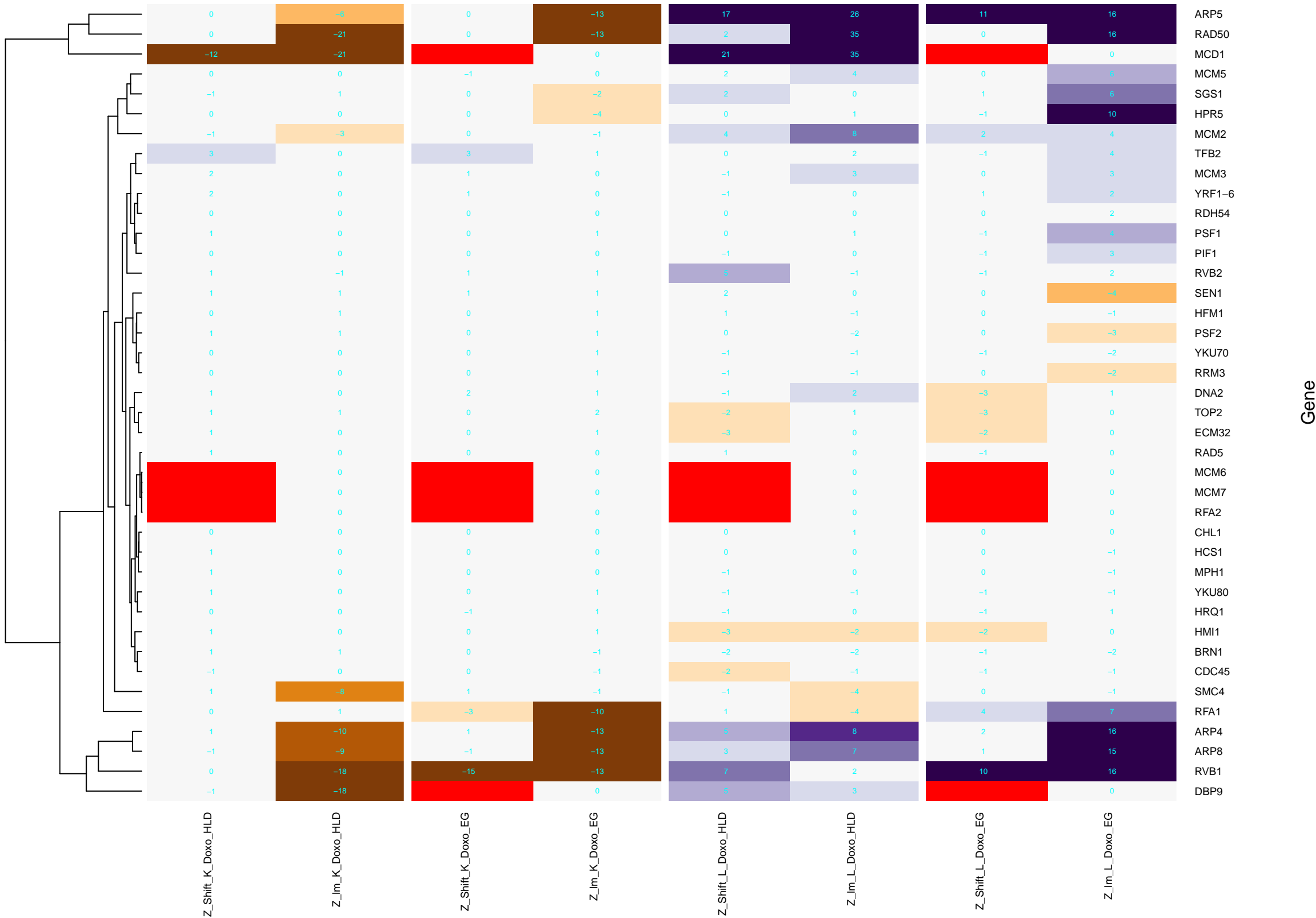

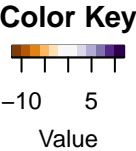

DNA unwinding involved in DNA replication

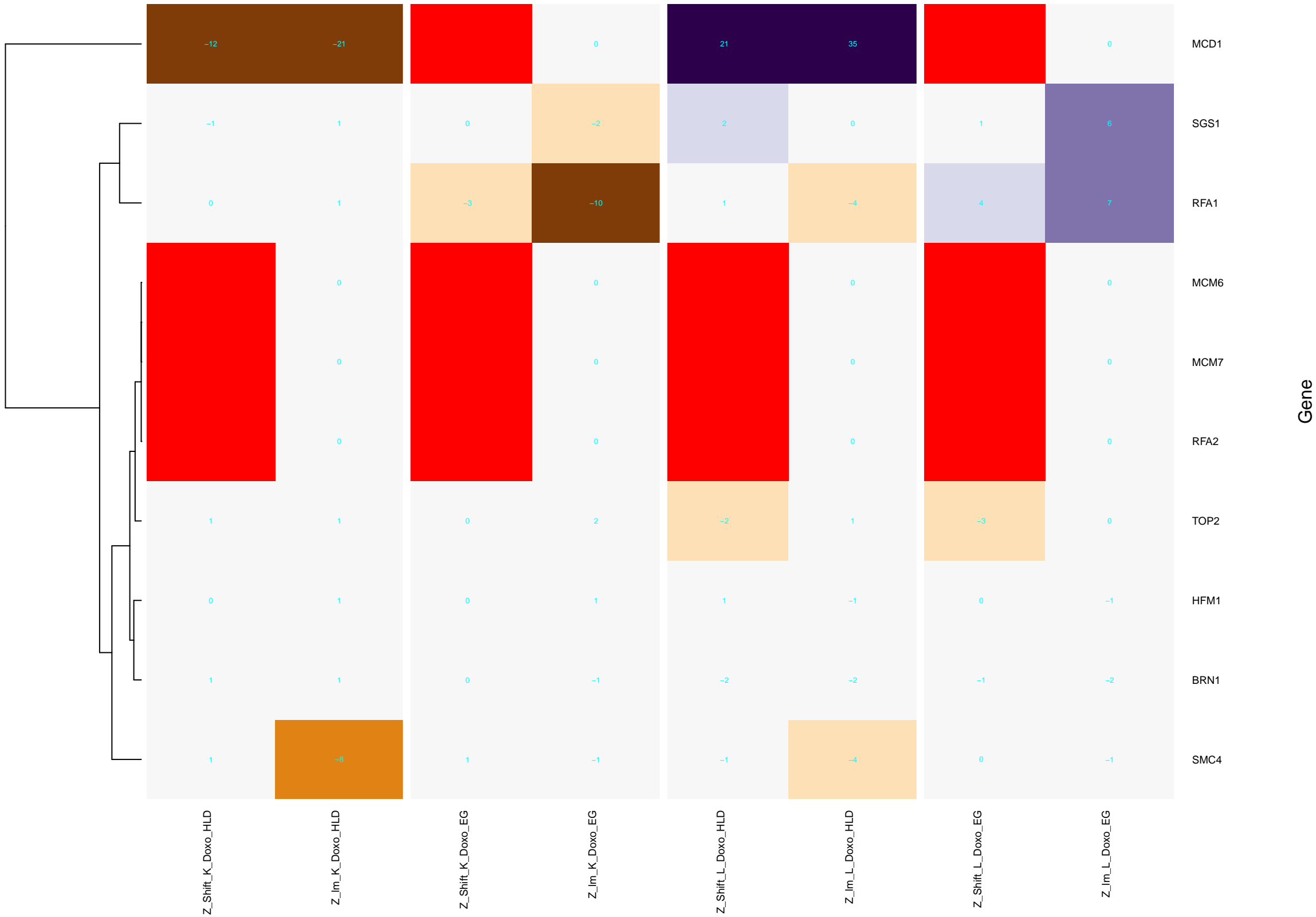

Supplement: Supplementary file 9 — Additional file 9. GO term-specific heatmaps for REMc/GTF-enriched clusters. GO term-specific heatmaps for significant GO process terms were generated as described in methods and Figs. 3 and 4. Any related child terms are presented in subsequent pages of the parent file name. GO terms with more than 100 children, with 2 or fewer genes annotated to the term, or a file size over 300KB are not shown. All heatmaps are generated with the same layout (see Figs. 3 and 4). [file 40170_2019_201_MOESM9_ESM.bz2 › Additional_File9_GOTermHeatmaps/Additional_File9_GOTermHeatmap/DNA_duplex_unwinding.pdf]

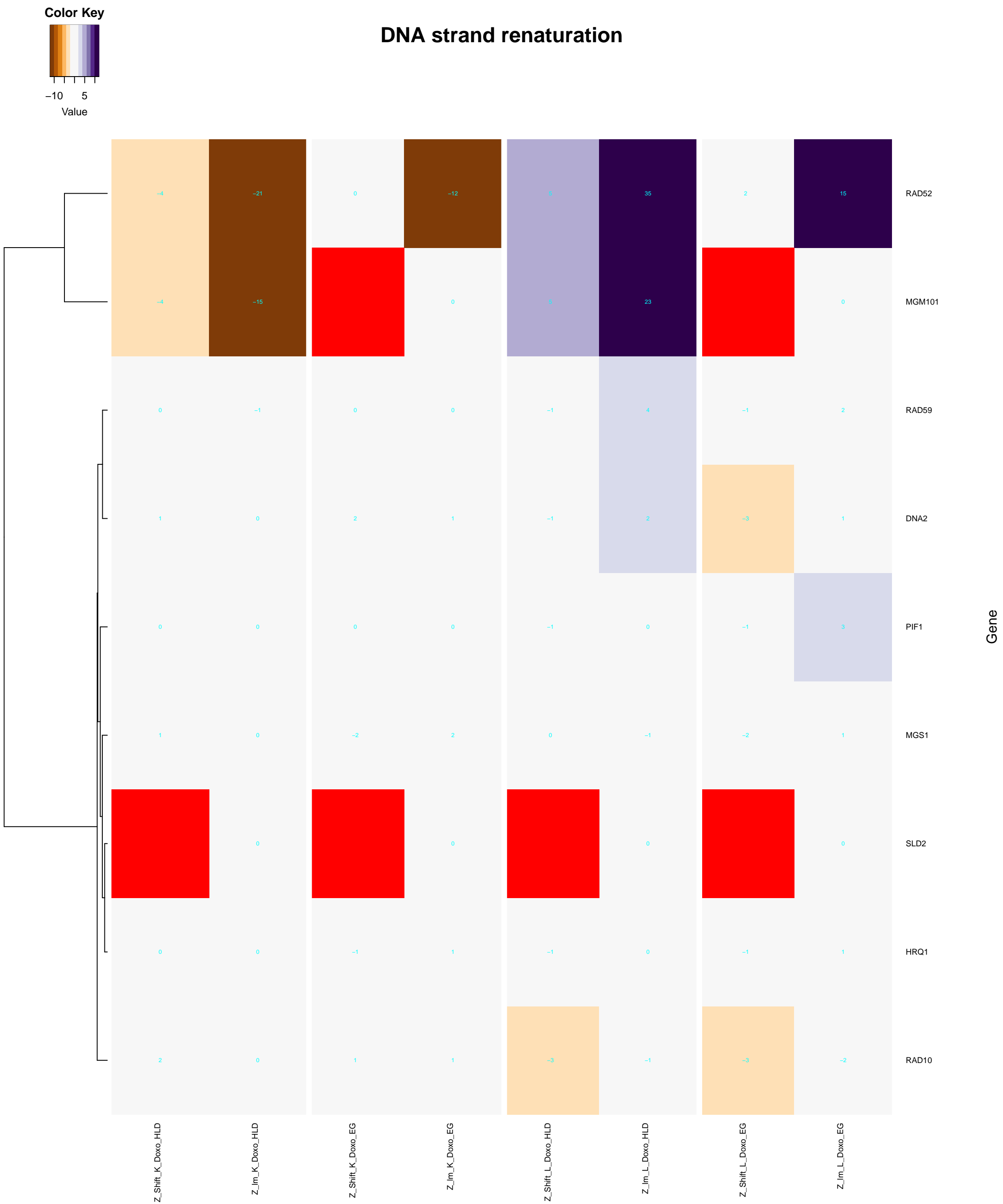

## DNA duplex unwinding

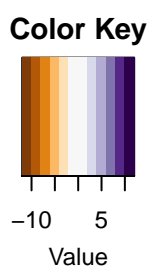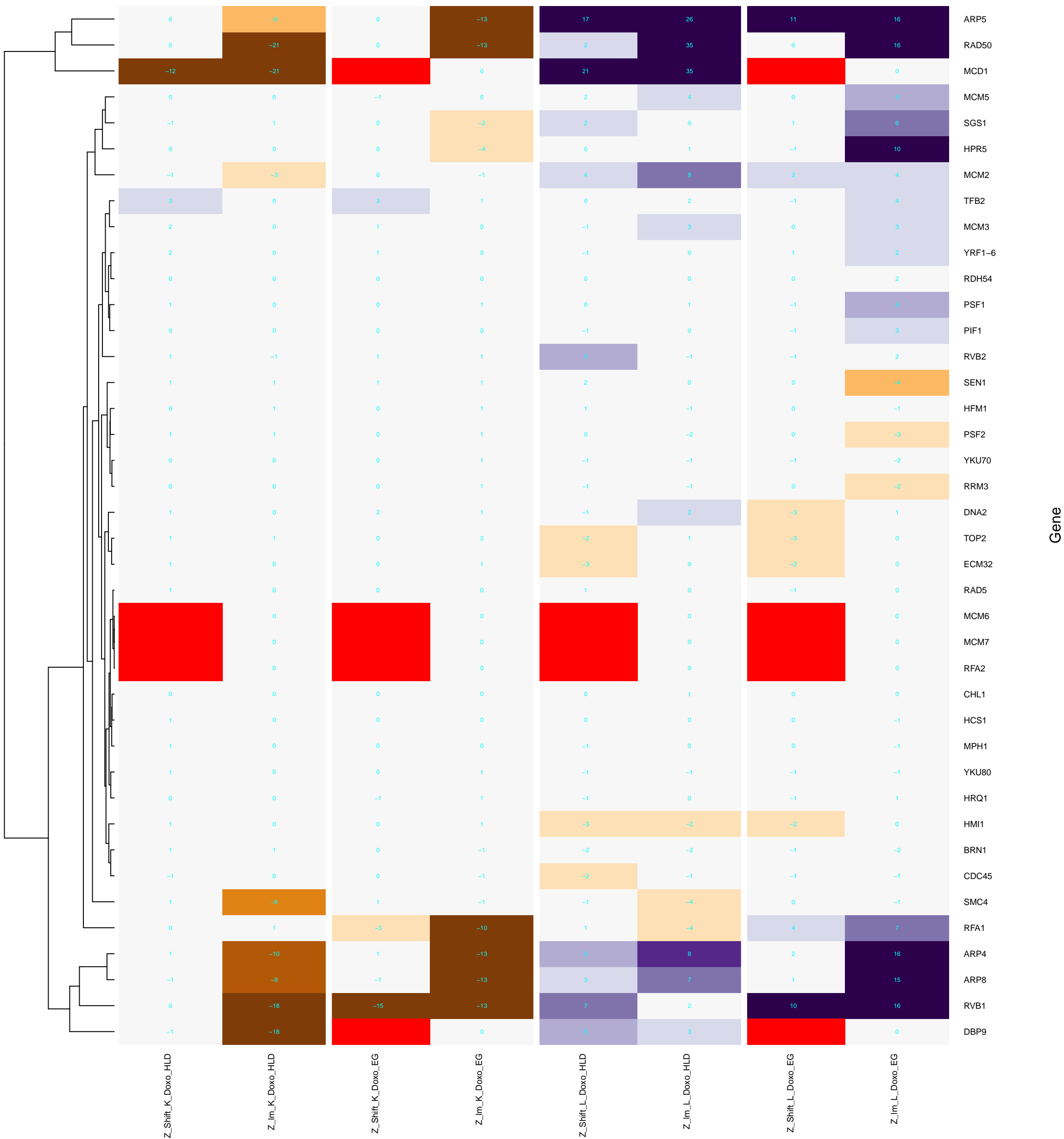

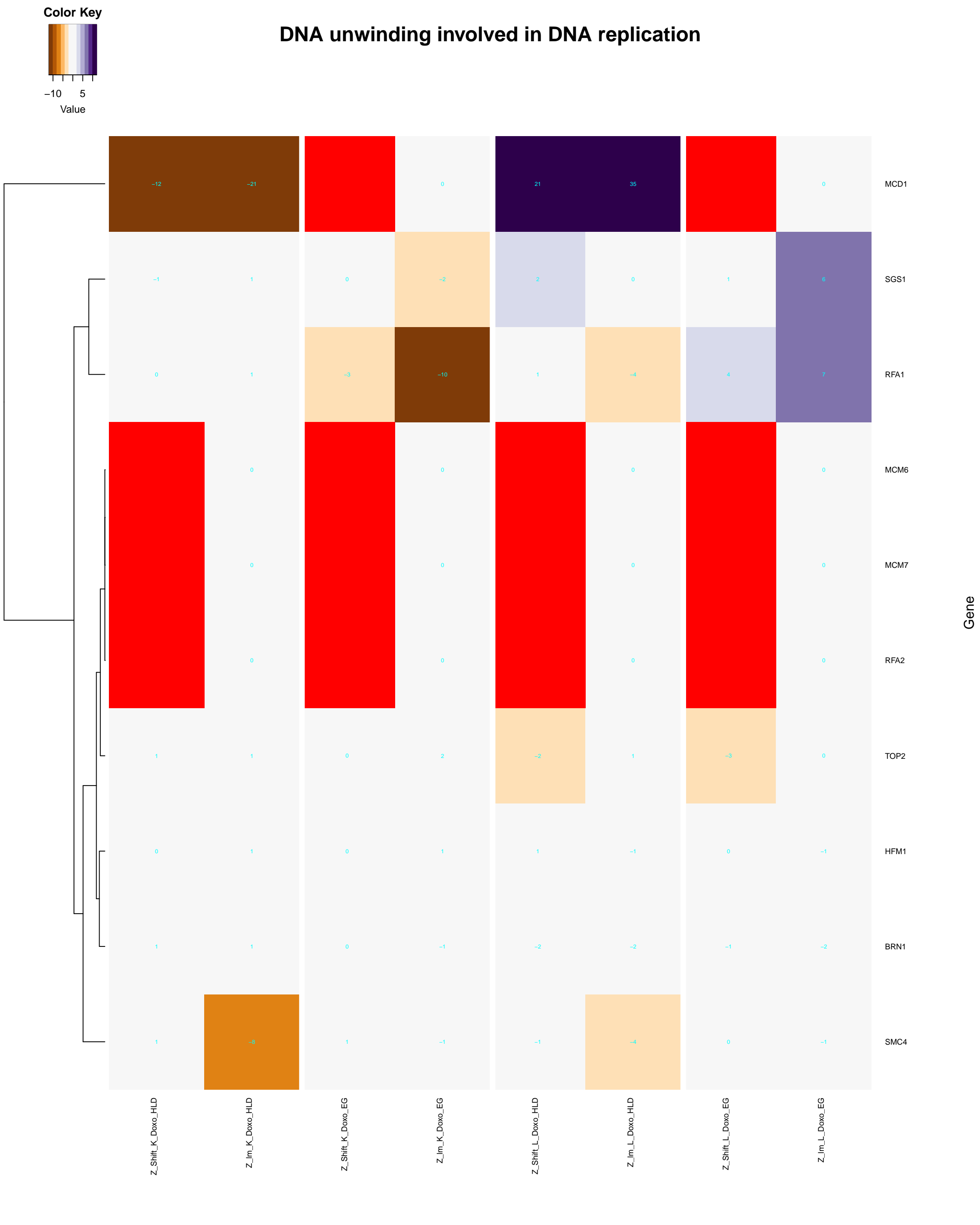

Supplement: Supplementary file 9 — Additional file 9. GO term-specific heatmaps for REMc/GTF-enriched clusters. GO term-specific heatmaps for significant GO process terms were generated as described in methods and Figs. 3 and 4. Any related child terms are presented in subsequent pages of the parent file name. GO terms with more than 100 children, with 2 or fewer genes annotated to the term, or a file size over 300KB are not shown. All heatmaps are generated with the same layout (see Figs. 3 and 4). [file 40170_2019_201_MOESM9_ESM.bz2 › Additional_File9_GOTermHeatmaps/Additional_File9_GOTermHeatmap/DNA_geometric_change.pdf]

DNA packaging

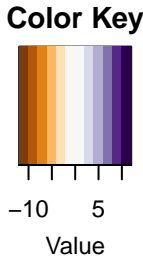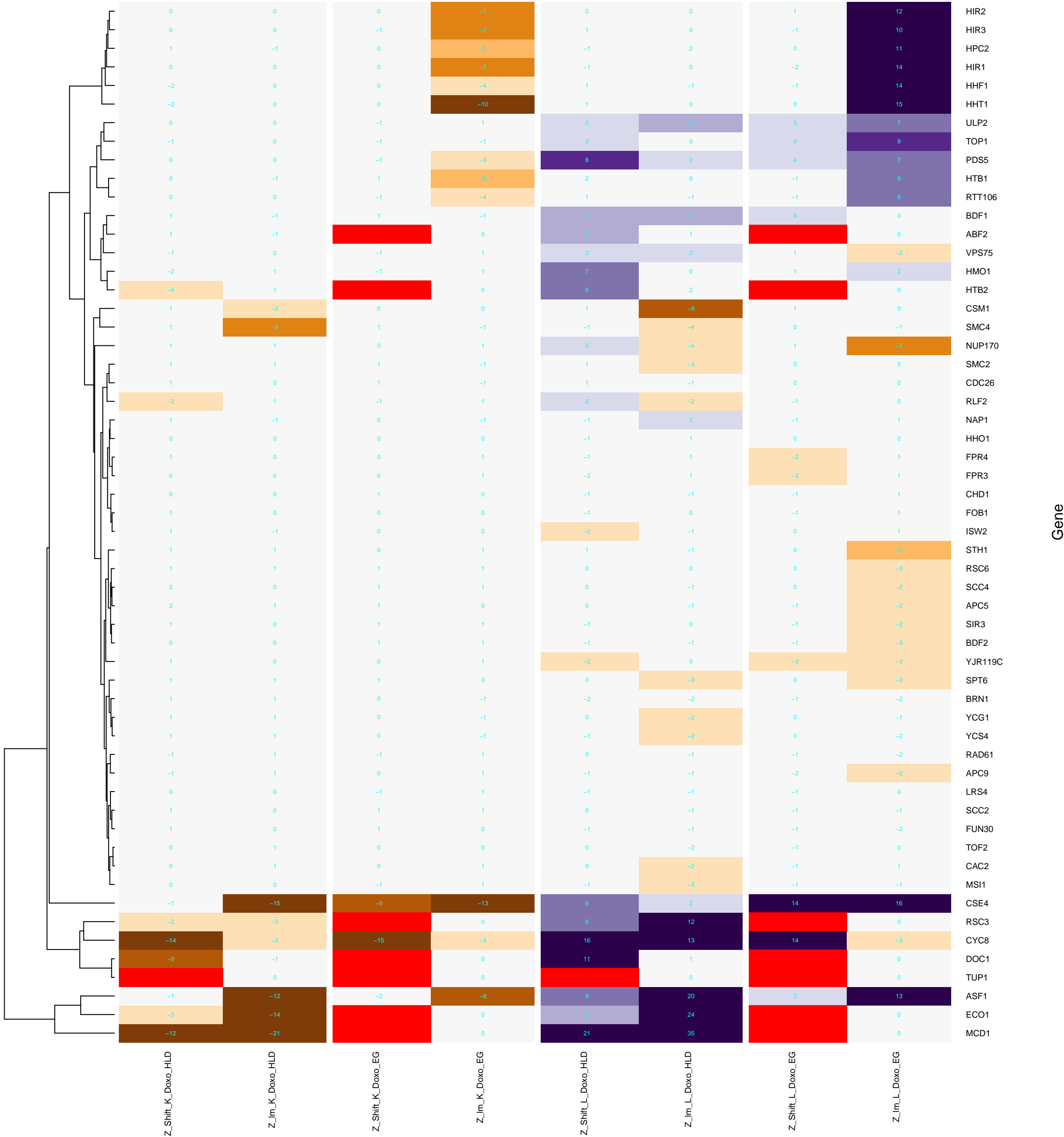

### Color Key

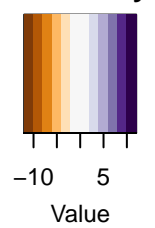

## chromosome condensation

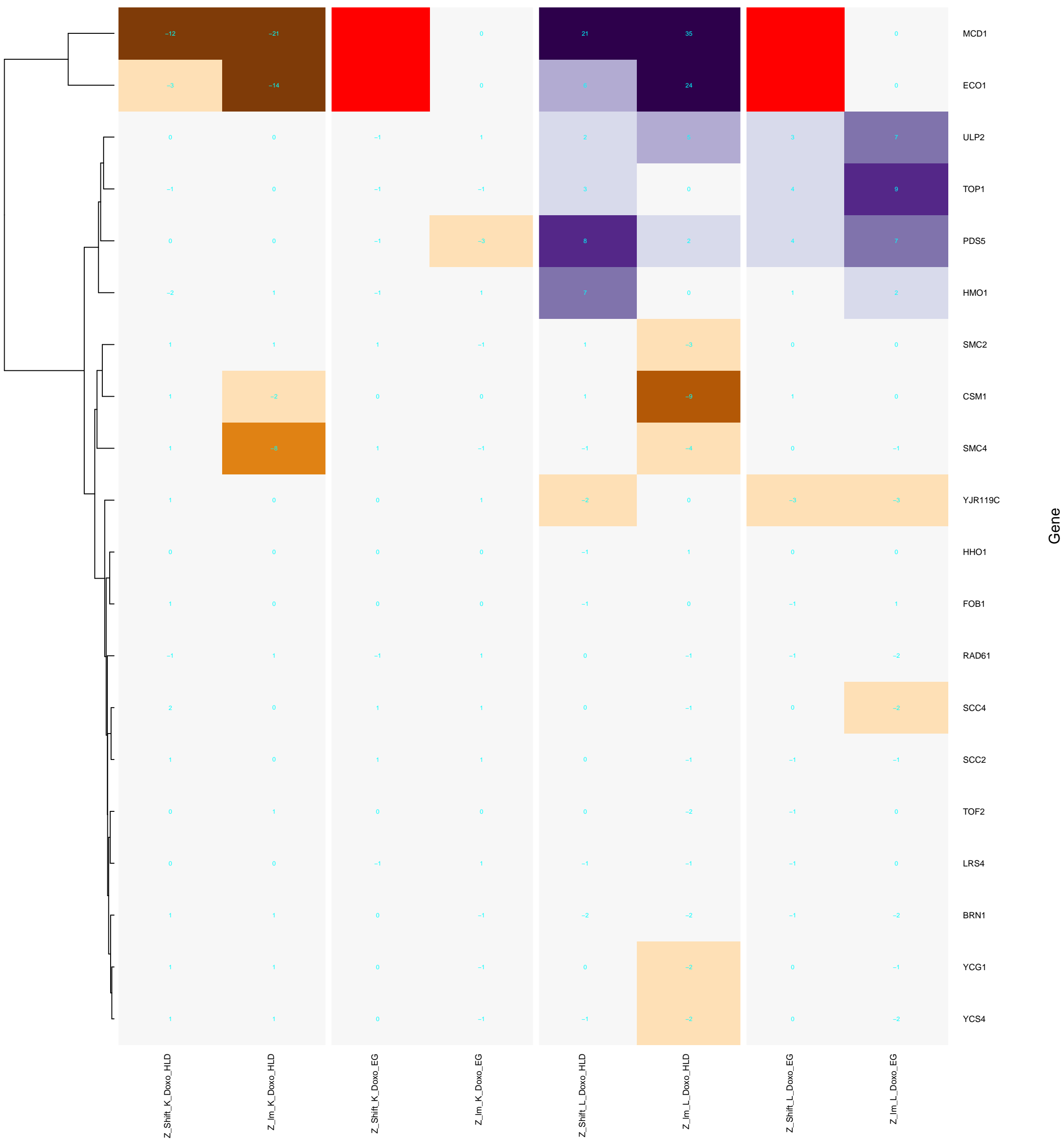

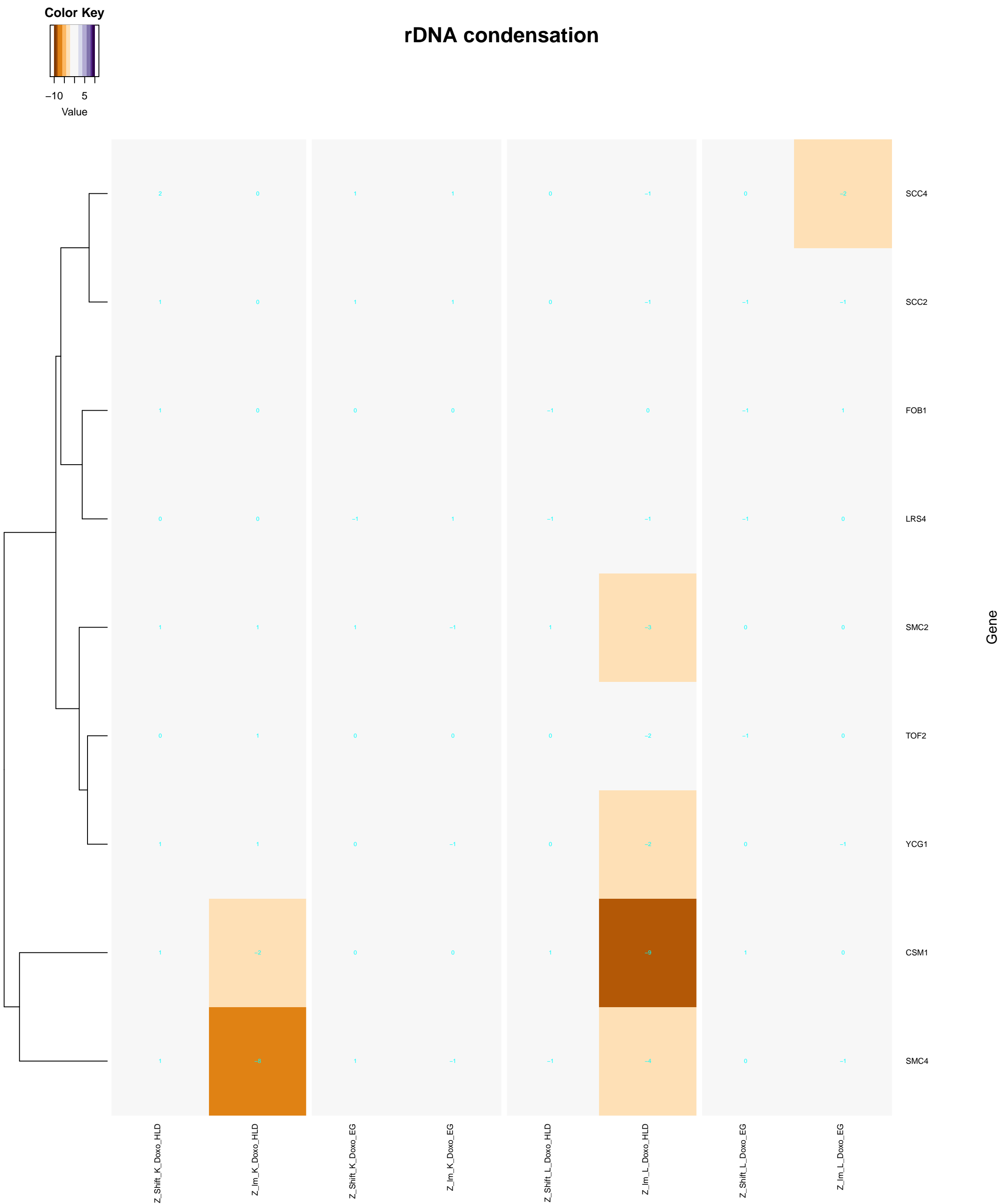

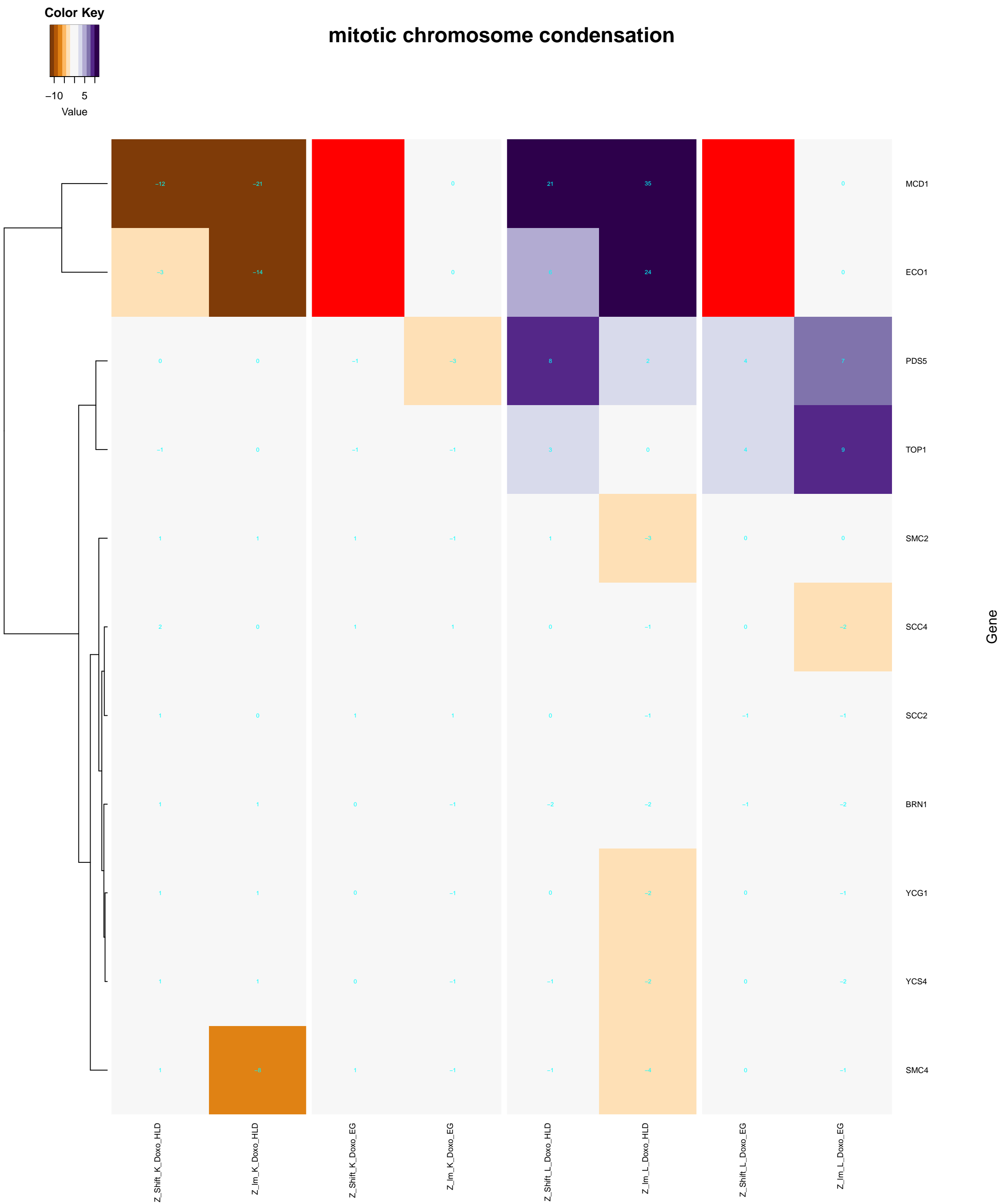

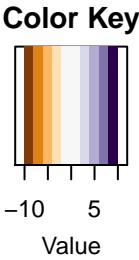

meiotic chromosome condensation

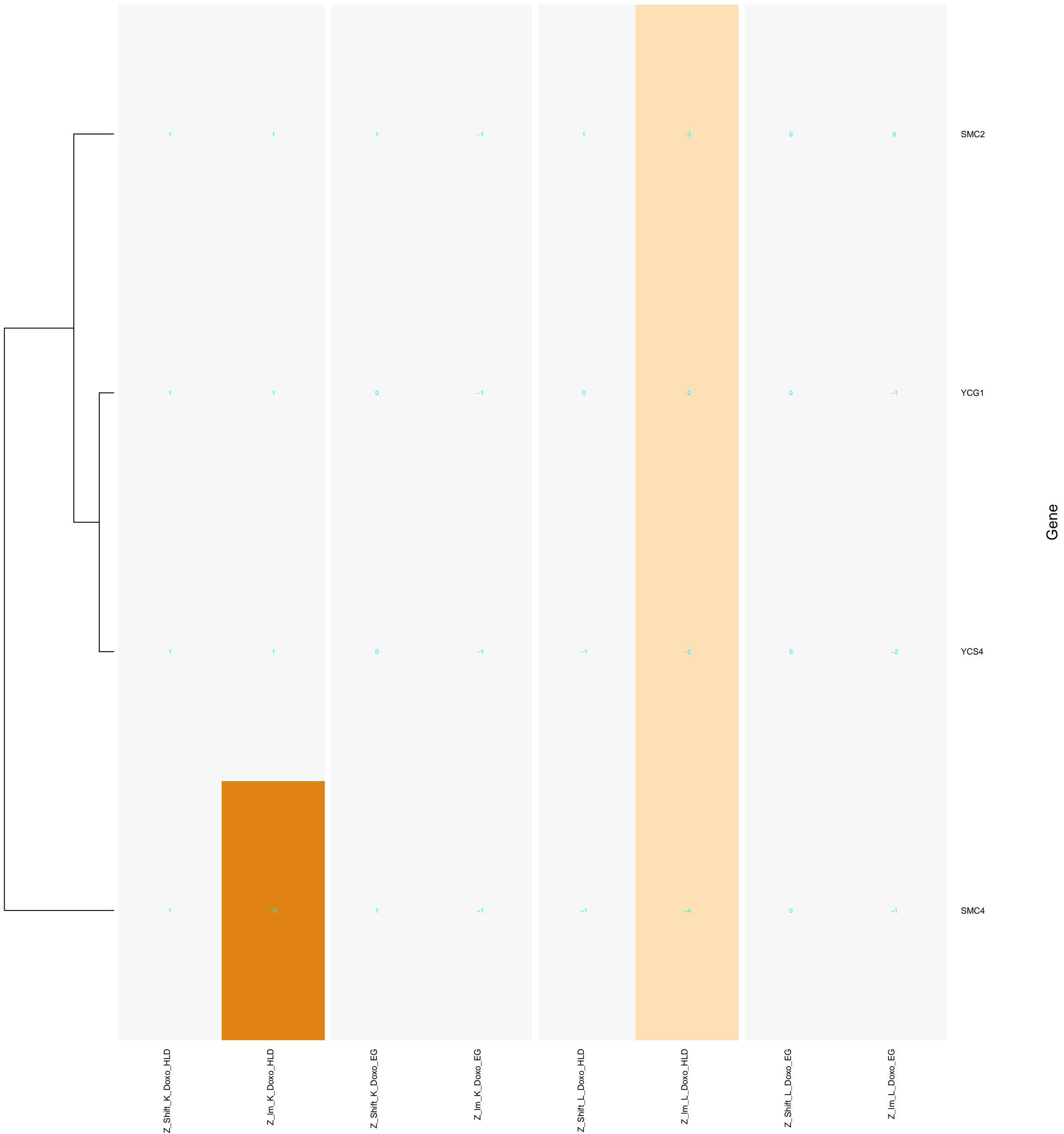

Supplement: Supplementary file 9 — Additional file 9. GO term-specific heatmaps for REMc/GTF-enriched clusters. GO term-specific heatmaps for significant GO process terms were generated as described in methods and Figs. 3 and 4. Any related child terms are presented in subsequent pages of the parent file name. GO terms with more than 100 children, with 2 or fewer genes annotated to the term, or a file size over 300KB are not shown. All heatmaps are generated with the same layout (see Figs. 3 and 4). [file 40170_2019_201_MOESM9_ESM.bz2 › Additional_File9_GOTermHeatmaps/Additional_File9_GOTermHeatmap/DNA_packaging.pdf]

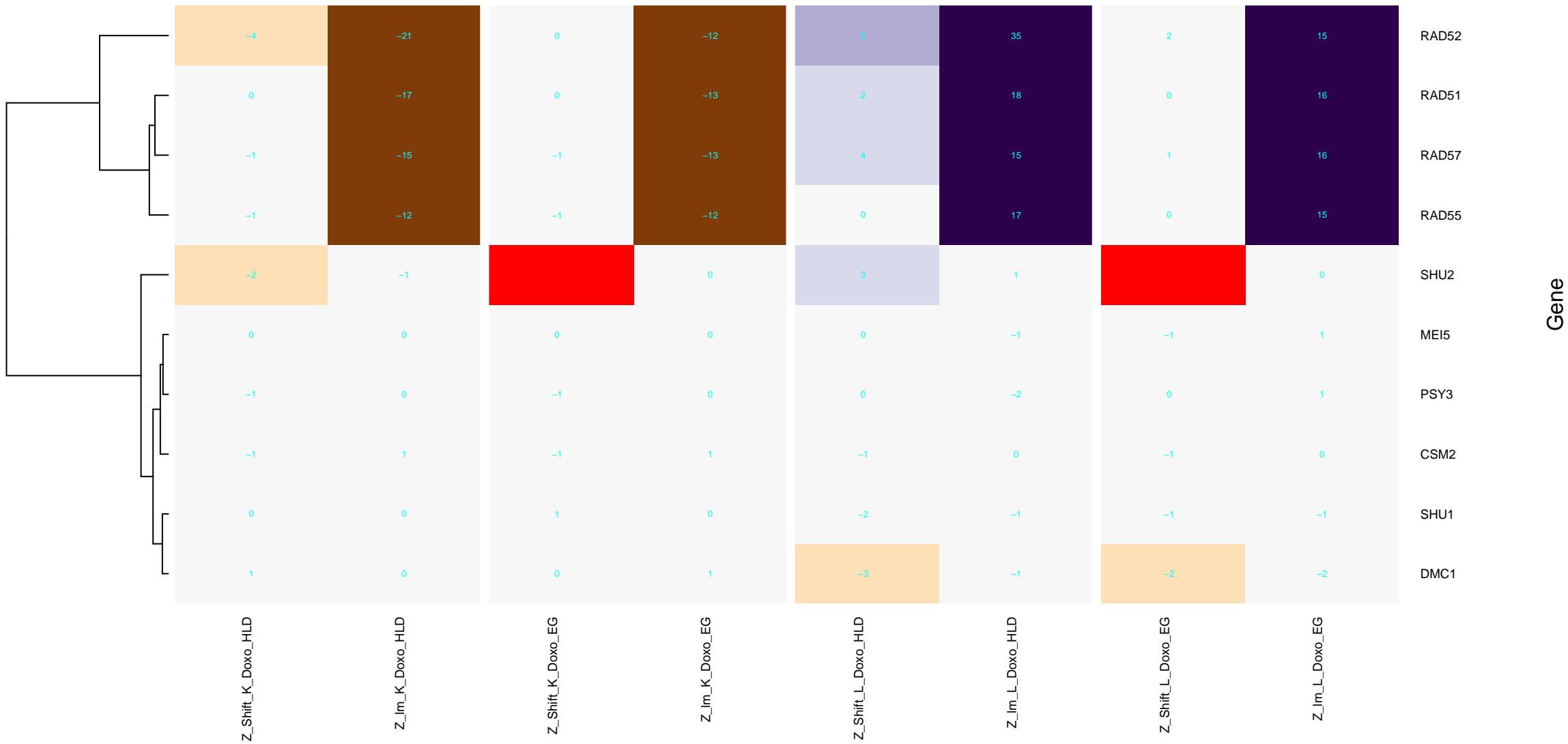

Supplement: Supplementary file 9 — Additional file 9. GO term-specific heatmaps for REMc/GTF-enriched clusters. GO term-specific heatmaps for significant GO process terms were generated as described in methods and Figs. 3 and 4. Any related child terms are presented in subsequent pages of the parent file name. GO terms with more than 100 children, with 2 or fewer genes annotated to the term, or a file size over 300KB are not shown. All heatmaps are generated with the same layout (see Figs. 3 and 4). [file 40170_2019_201_MOESM9_ESM.bz2 › Additional_File9_GOTermHeatmaps/Additional_File9_GOTermHeatmap/DNA_recombinase_assembly.pdf]

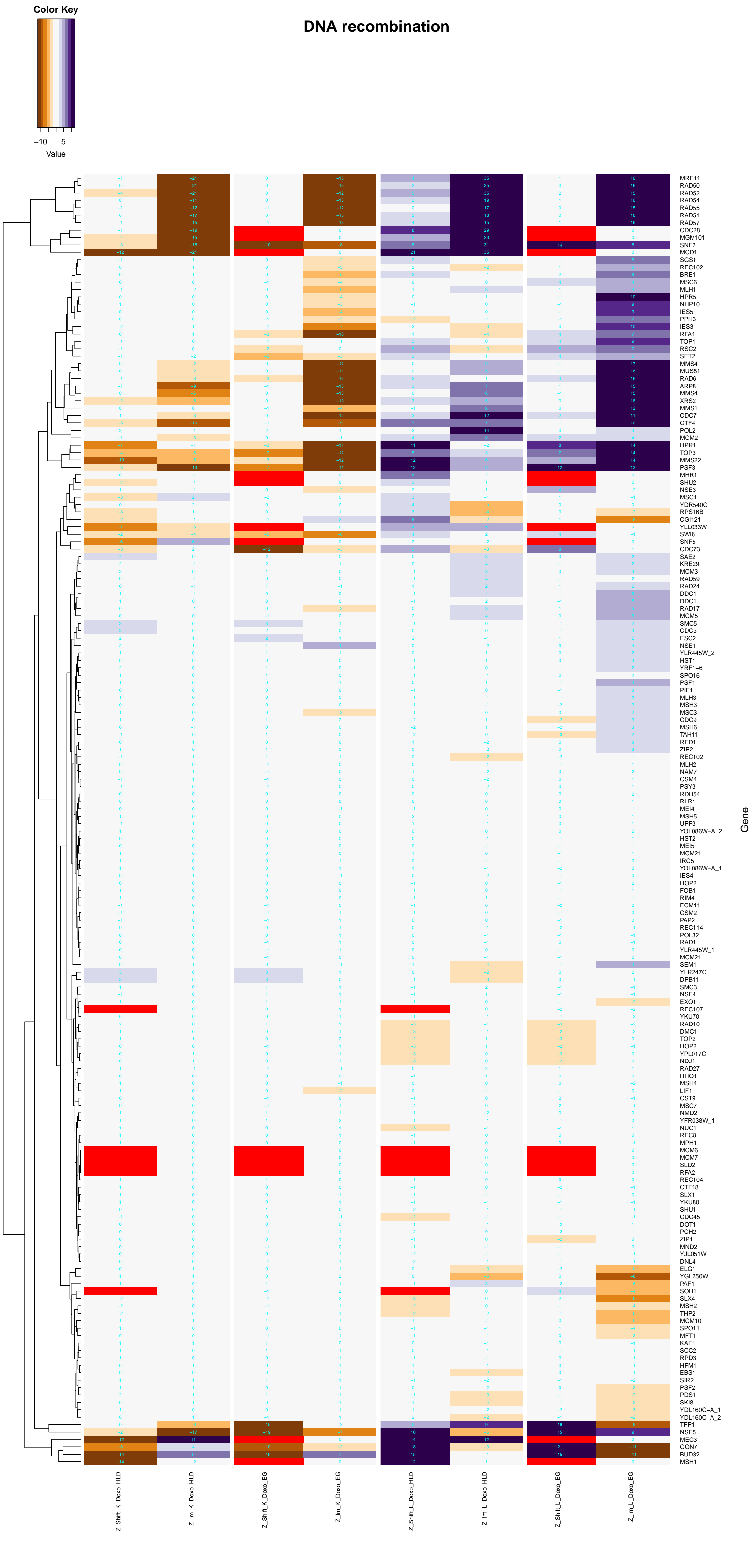

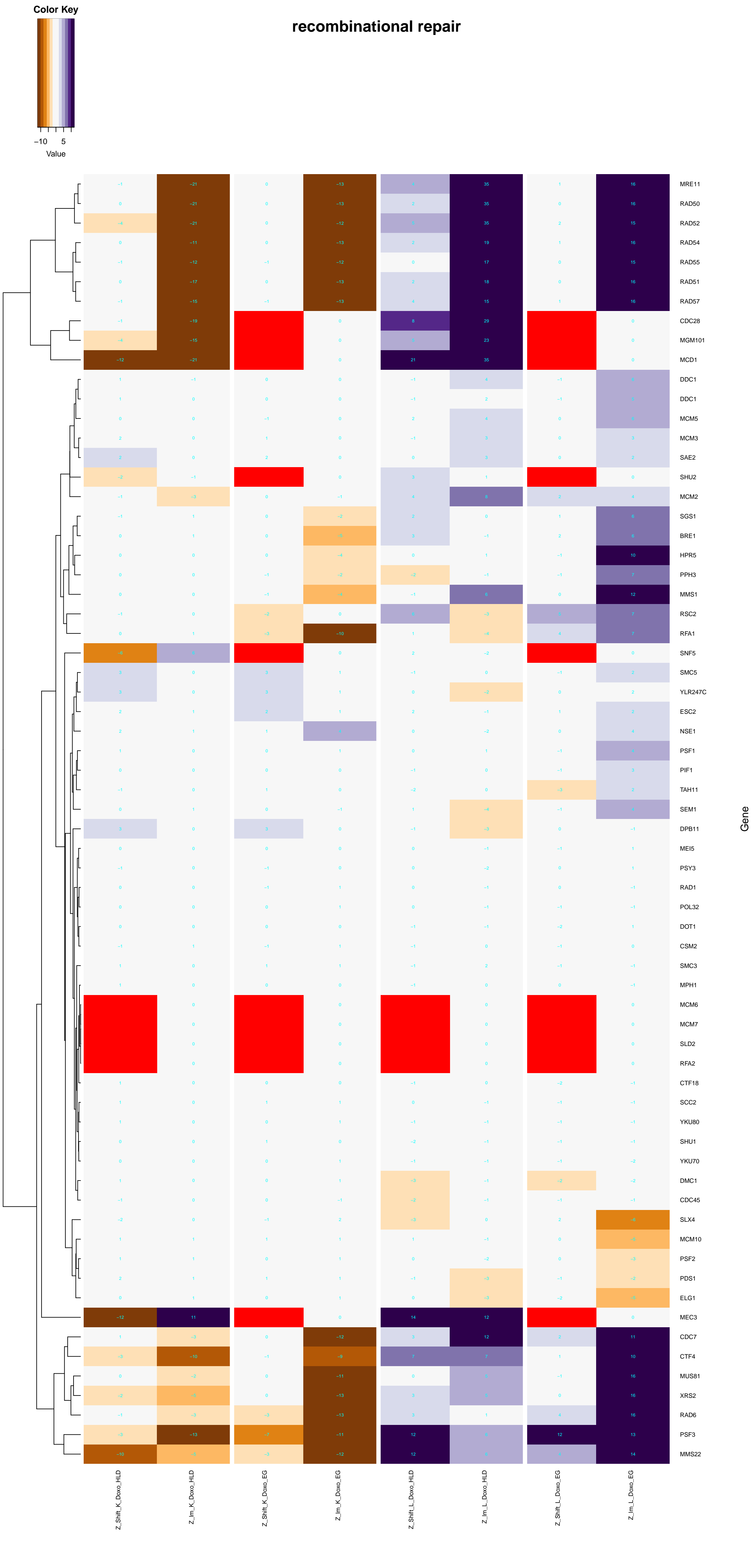

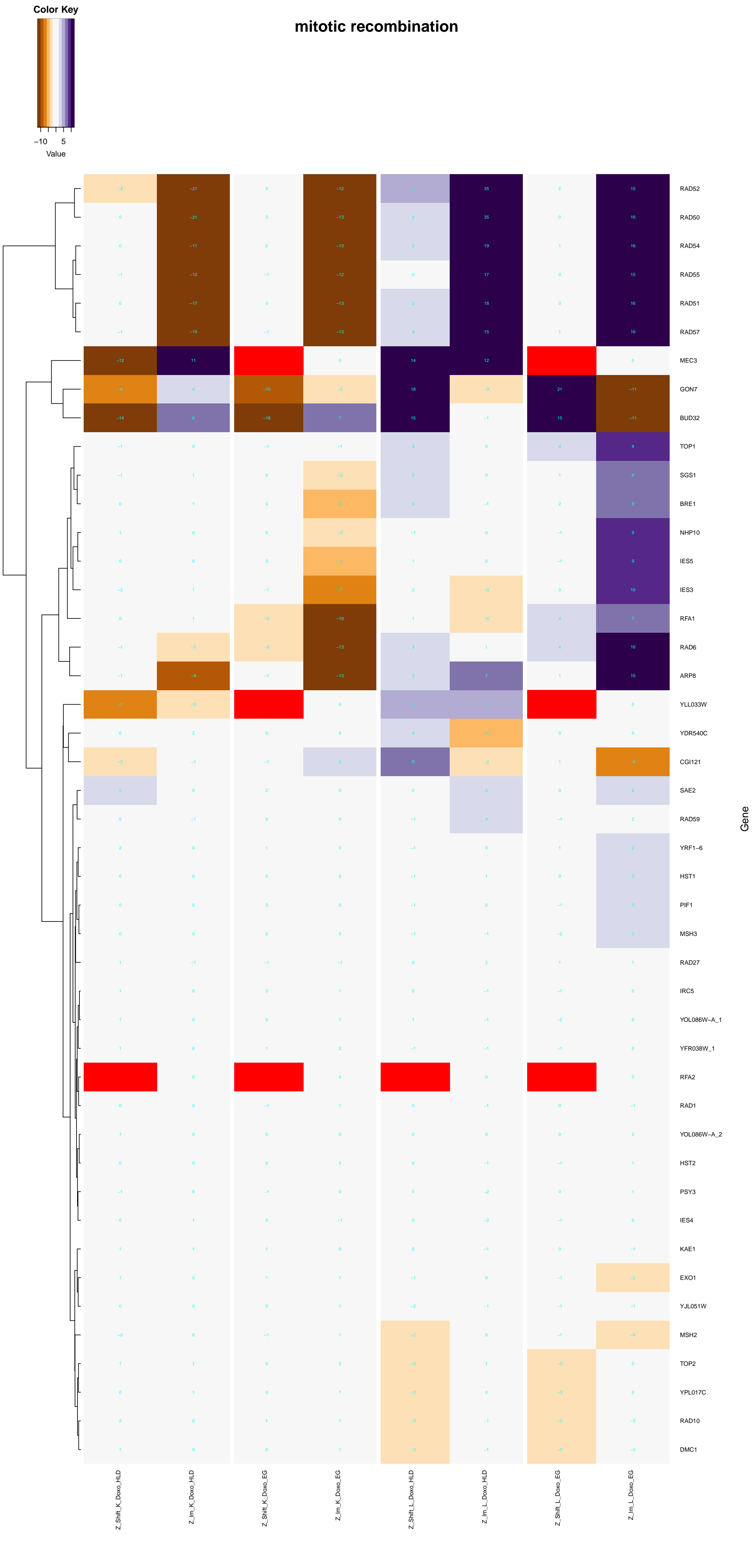

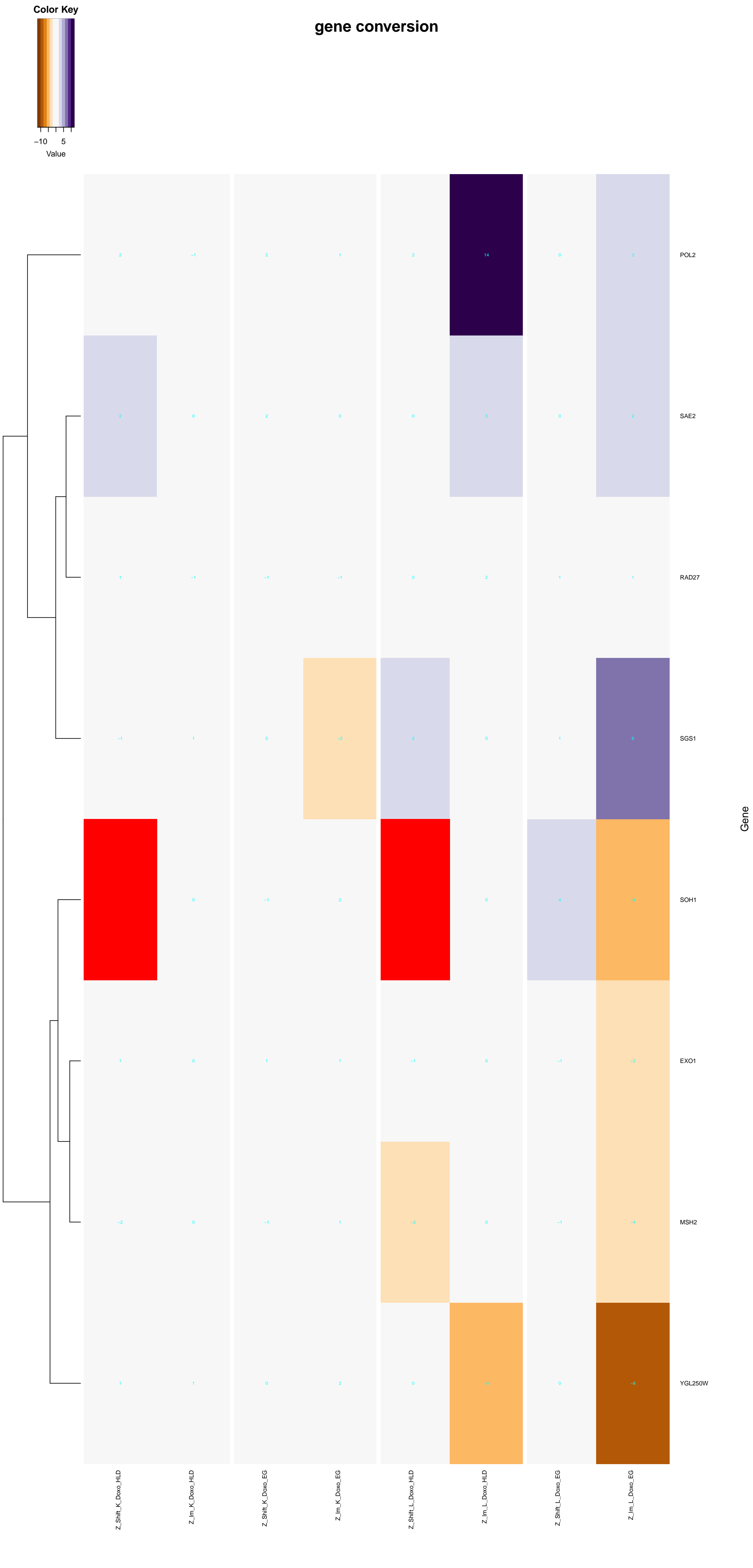

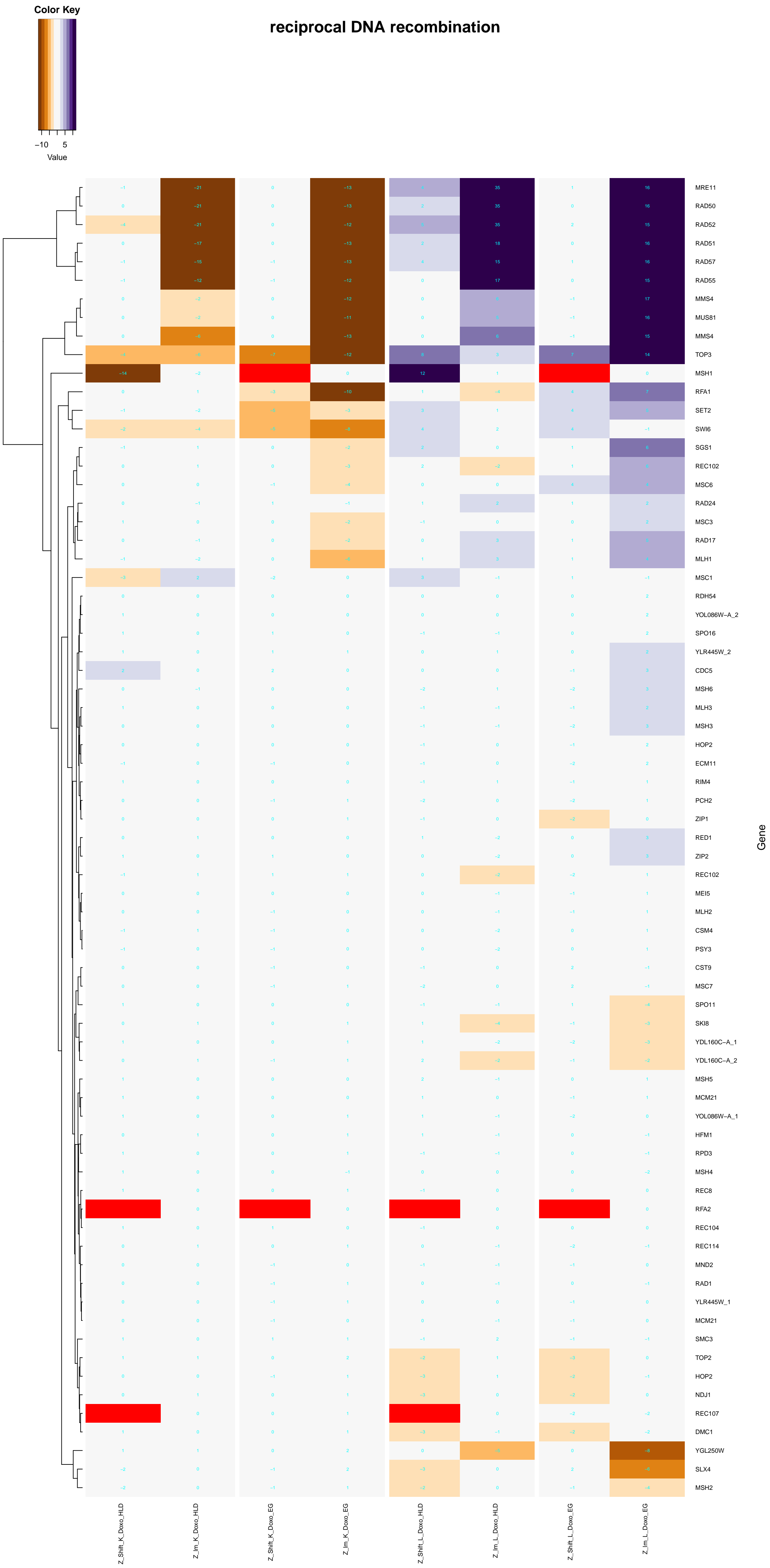

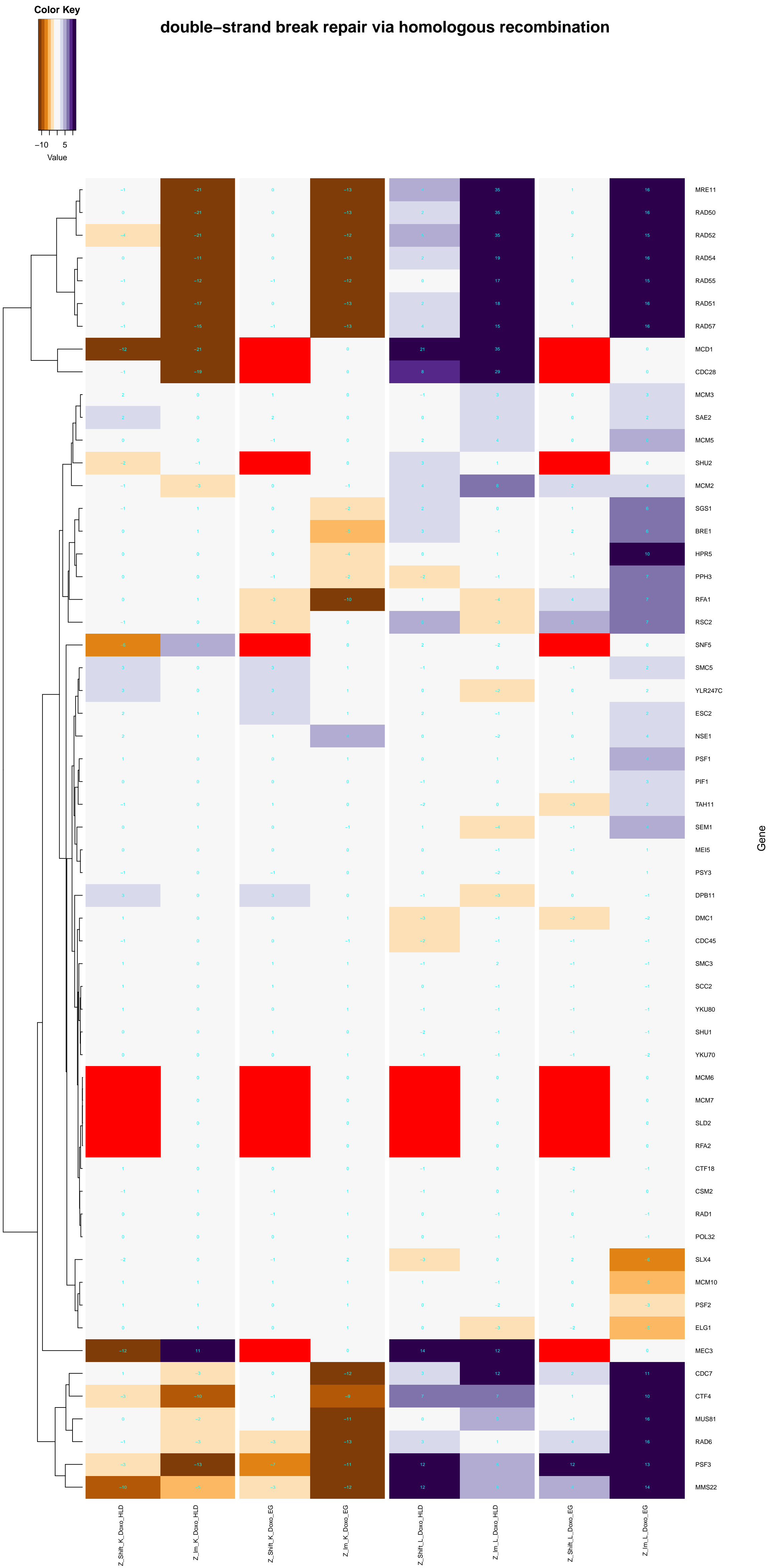

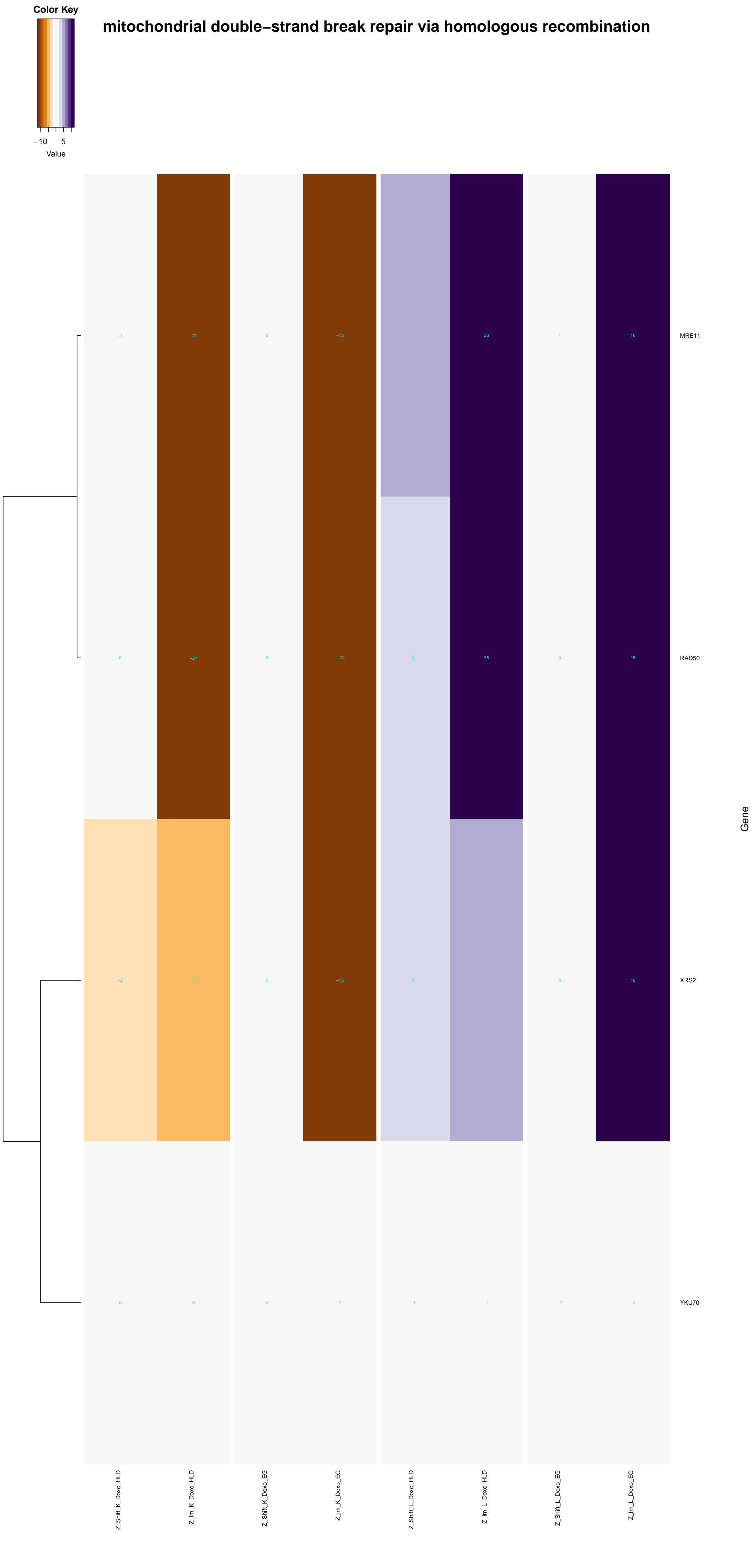

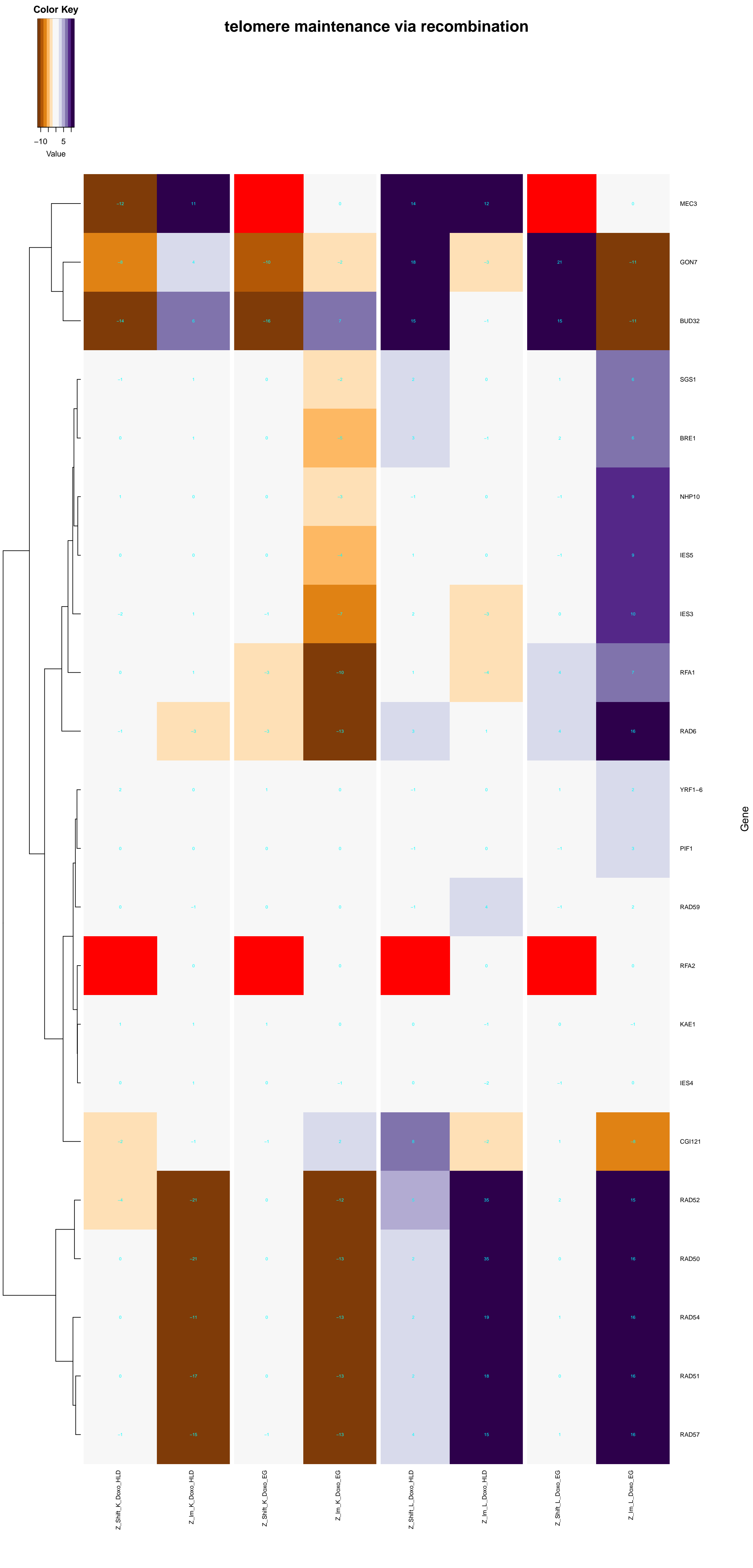

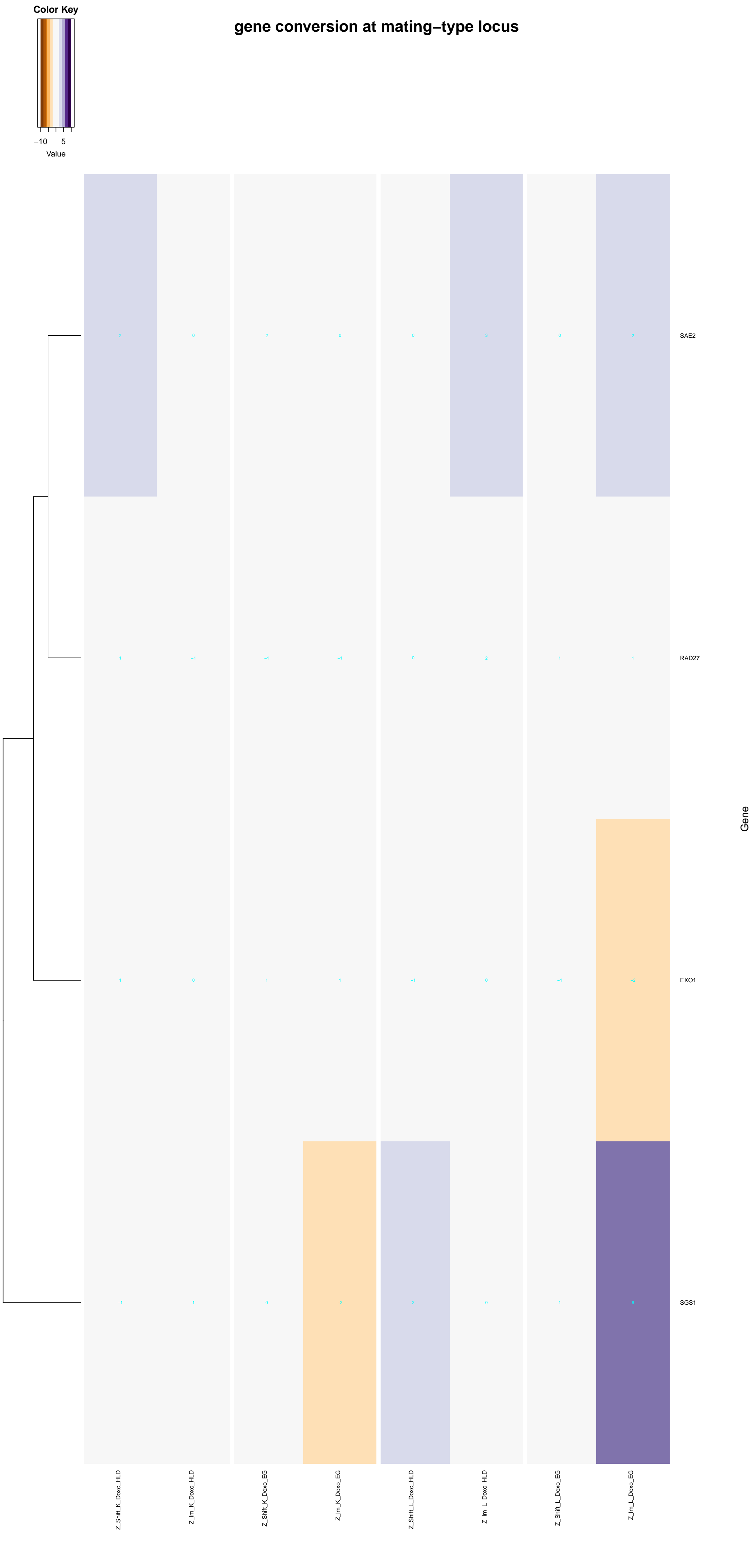

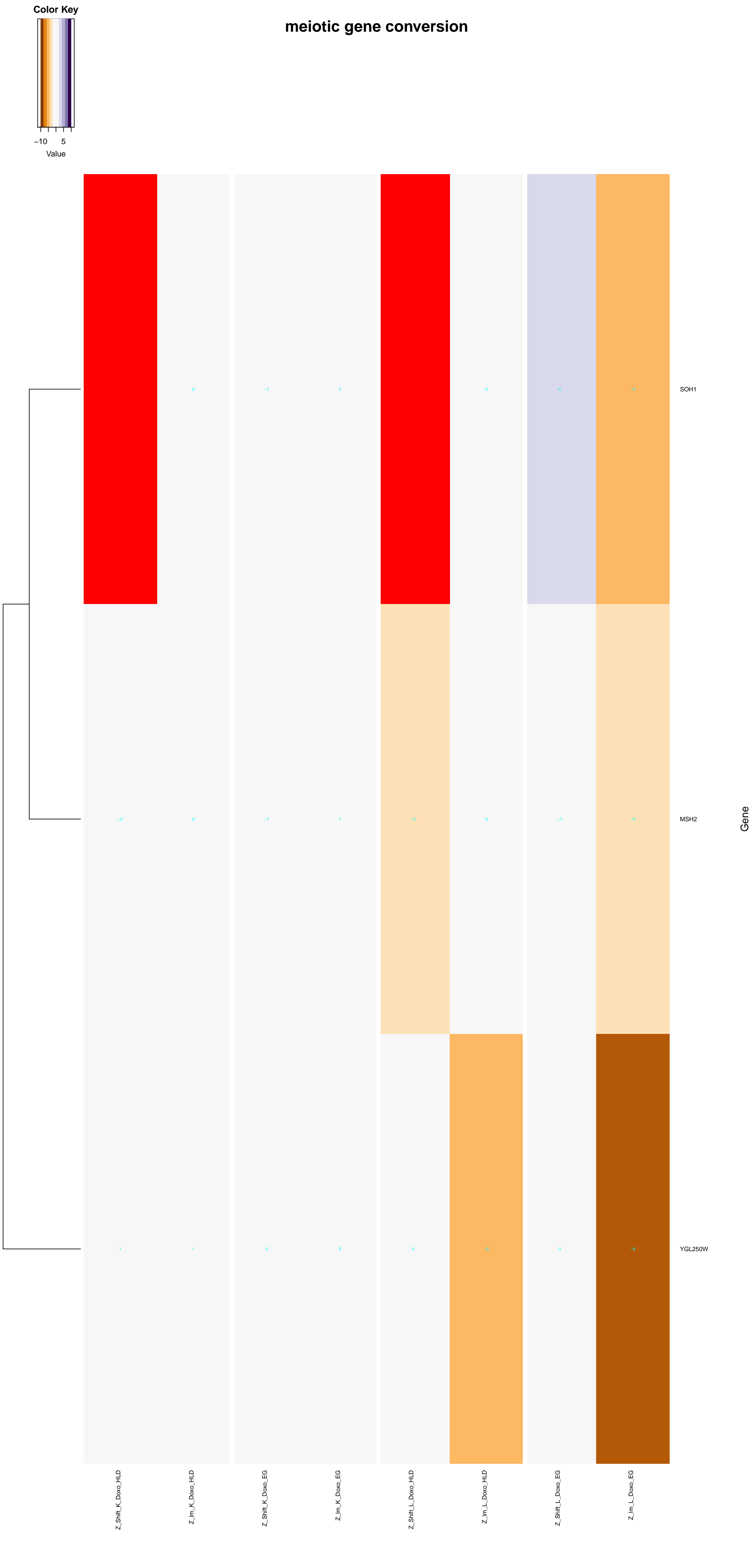

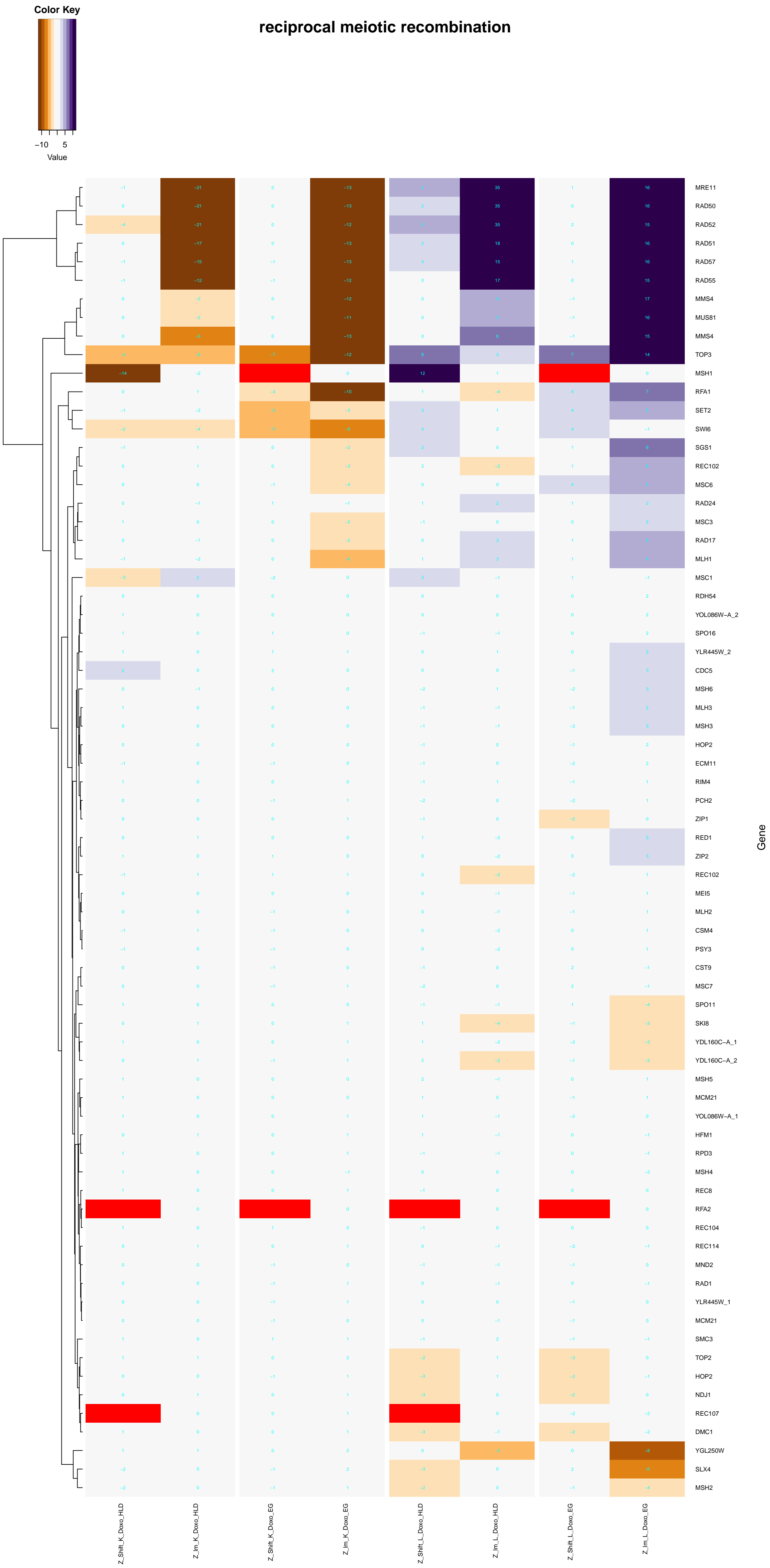

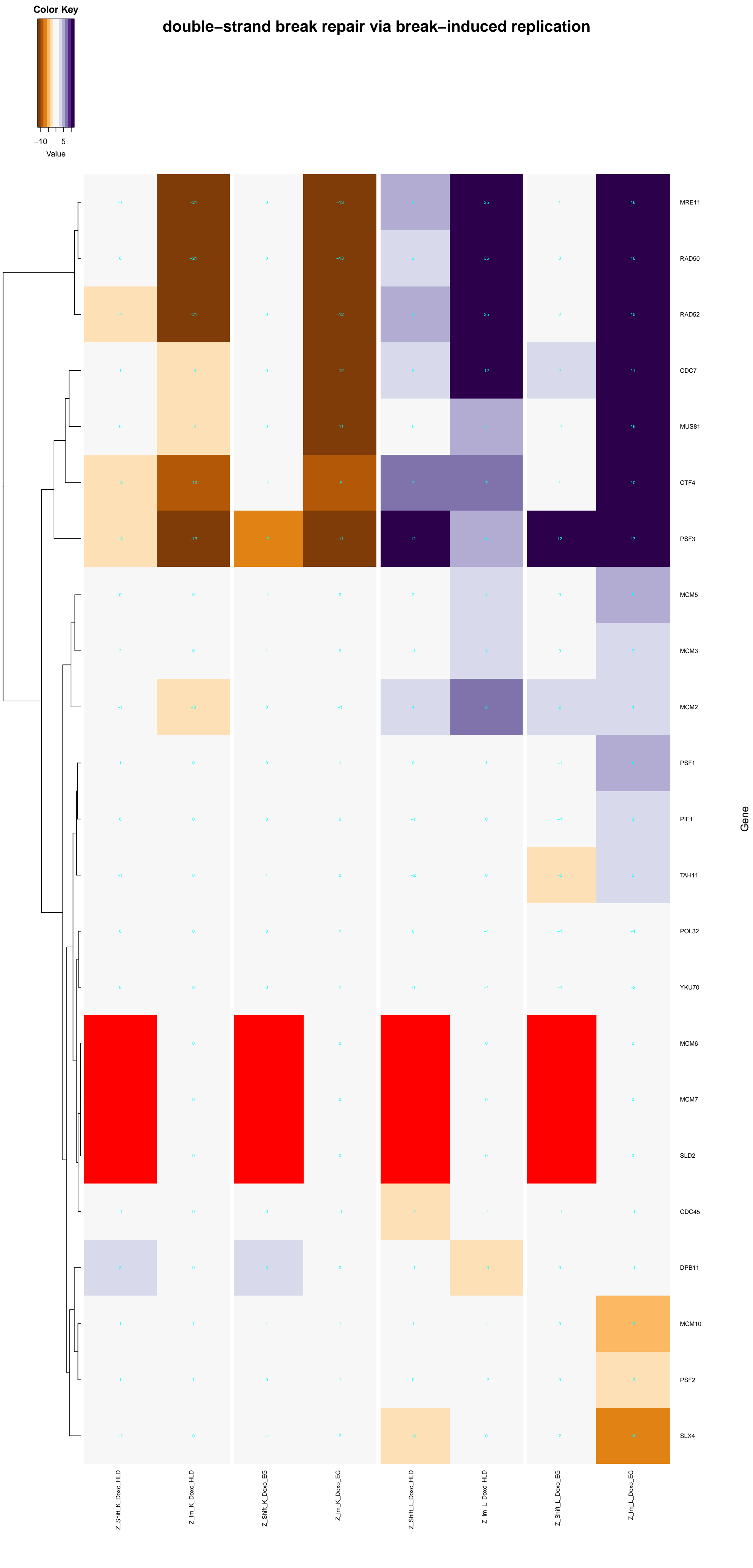

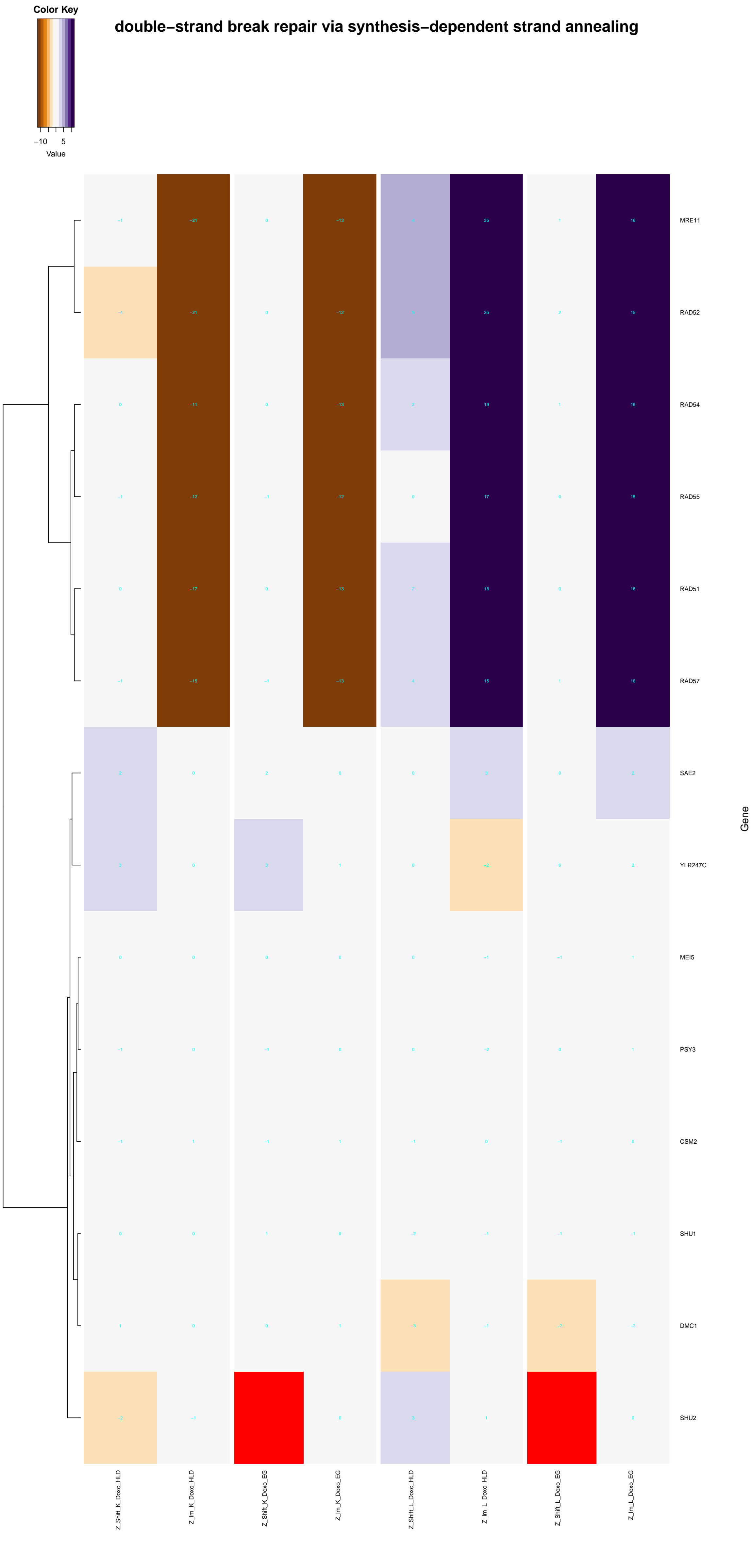

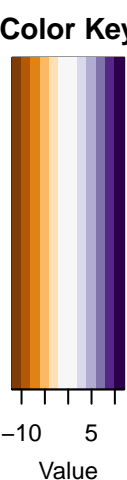

replication–born double–strand break repair via sister chromatid exchange

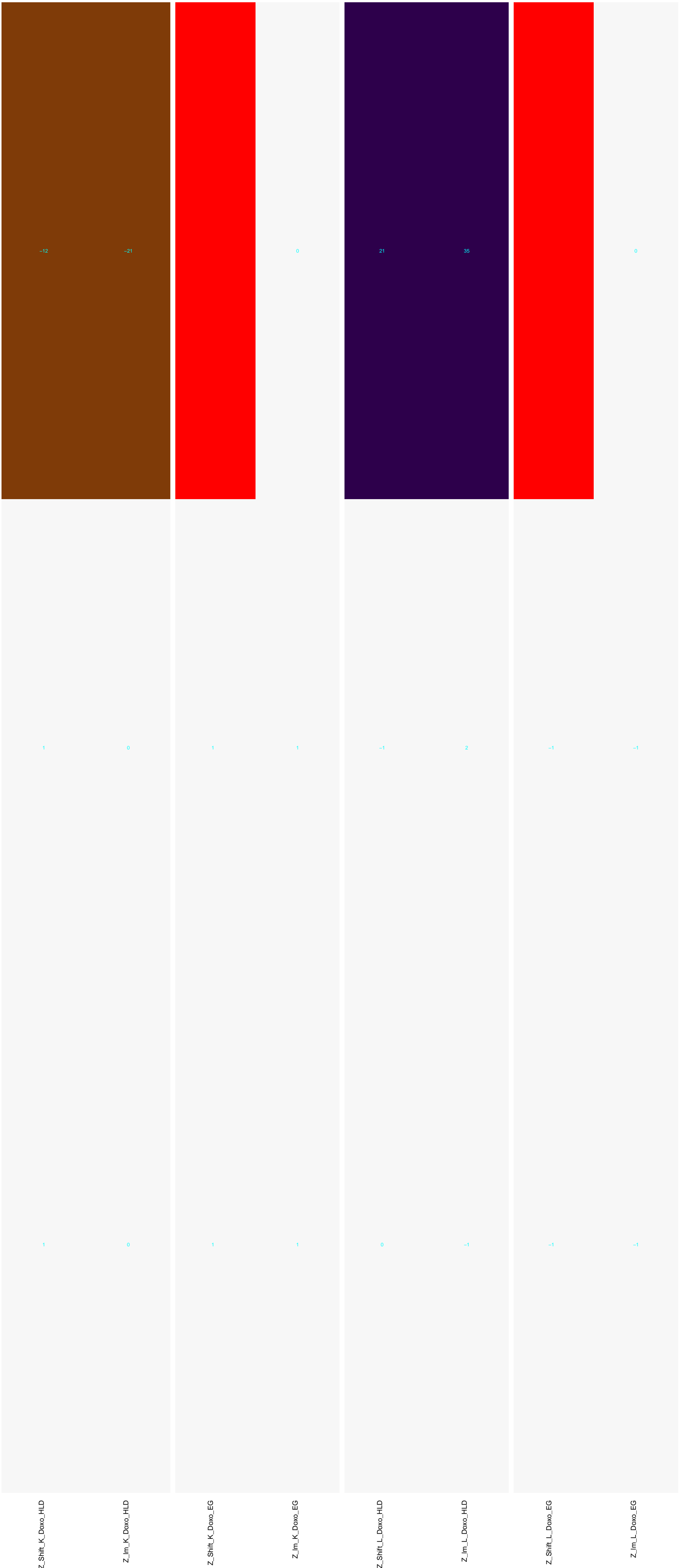

Gene

Supplement: Supplementary file 9 — Additional file 9. GO term-specific heatmaps for REMc/GTF-enriched clusters. GO term-specific heatmaps for significant GO process terms were generated as described in methods and Figs. 3 and 4. Any related child terms are presented in subsequent pages of the parent file name. GO terms with more than 100 children, with 2 or fewer genes annotated to the term, or a file size over 300KB are not shown. All heatmaps are generated with the same layout (see Figs. 3 and 4). [file 40170_2019_201_MOESM9_ESM.bz2 › Additional_File9_GOTermHeatmaps/Additional_File9_GOTermHeatmap/DNA_recombination.pdf]

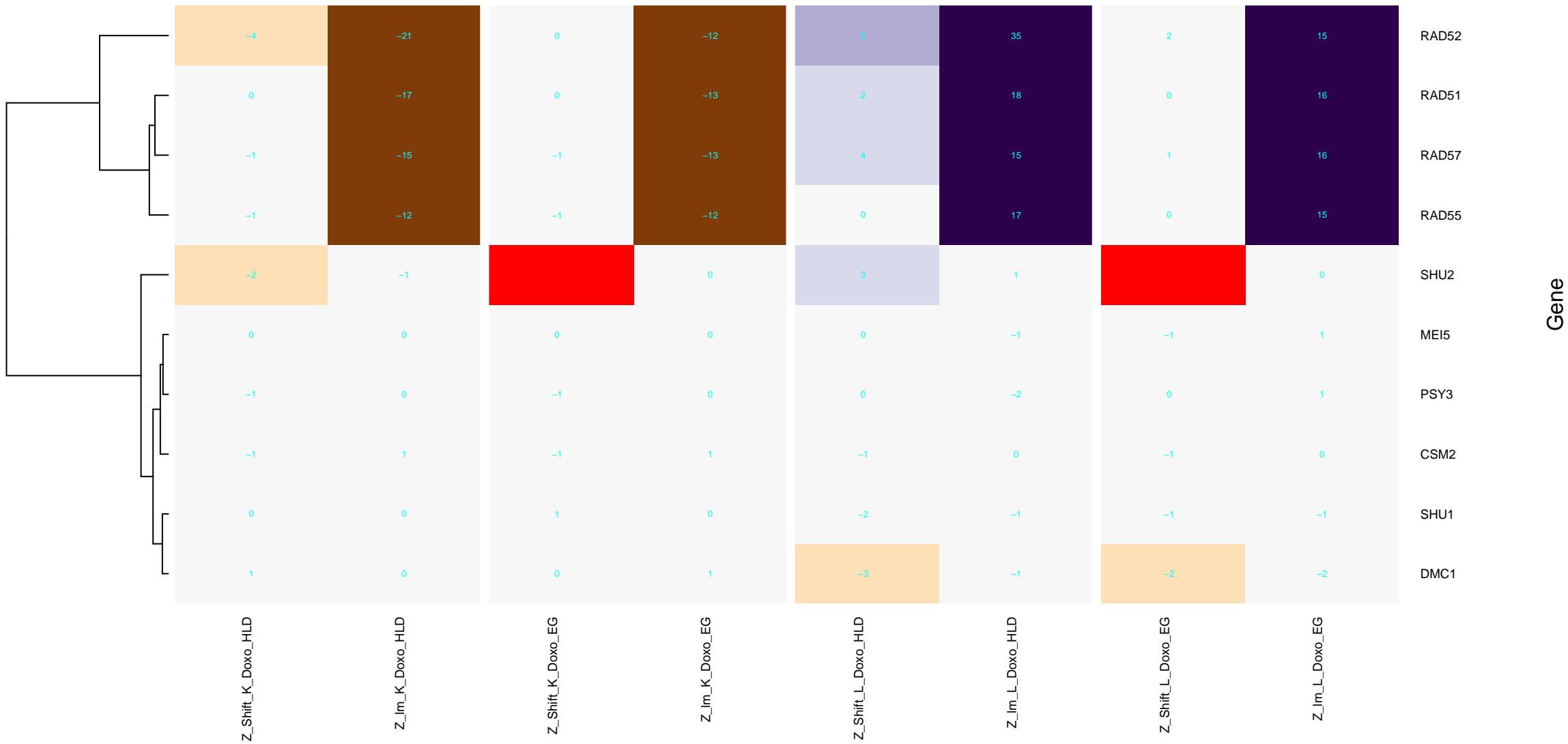

Supplement: Supplementary file 9 — Additional file 9. GO term-specific heatmaps for REMc/GTF-enriched clusters. GO term-specific heatmaps for significant GO process terms were generated as described in methods and Figs. 3 and 4. Any related child terms are presented in subsequent pages of the parent file name. GO terms with more than 100 children, with 2 or fewer genes annotated to the term, or a file size over 300KB are not shown. All heatmaps are generated with the same layout (see Figs. 3 and 4). [file 40170_2019_201_MOESM9_ESM.bz2 › Additional_File9_GOTermHeatmaps/Additional_File9_GOTermHeatmap/DNA_repair_complex_assembly.pdf]

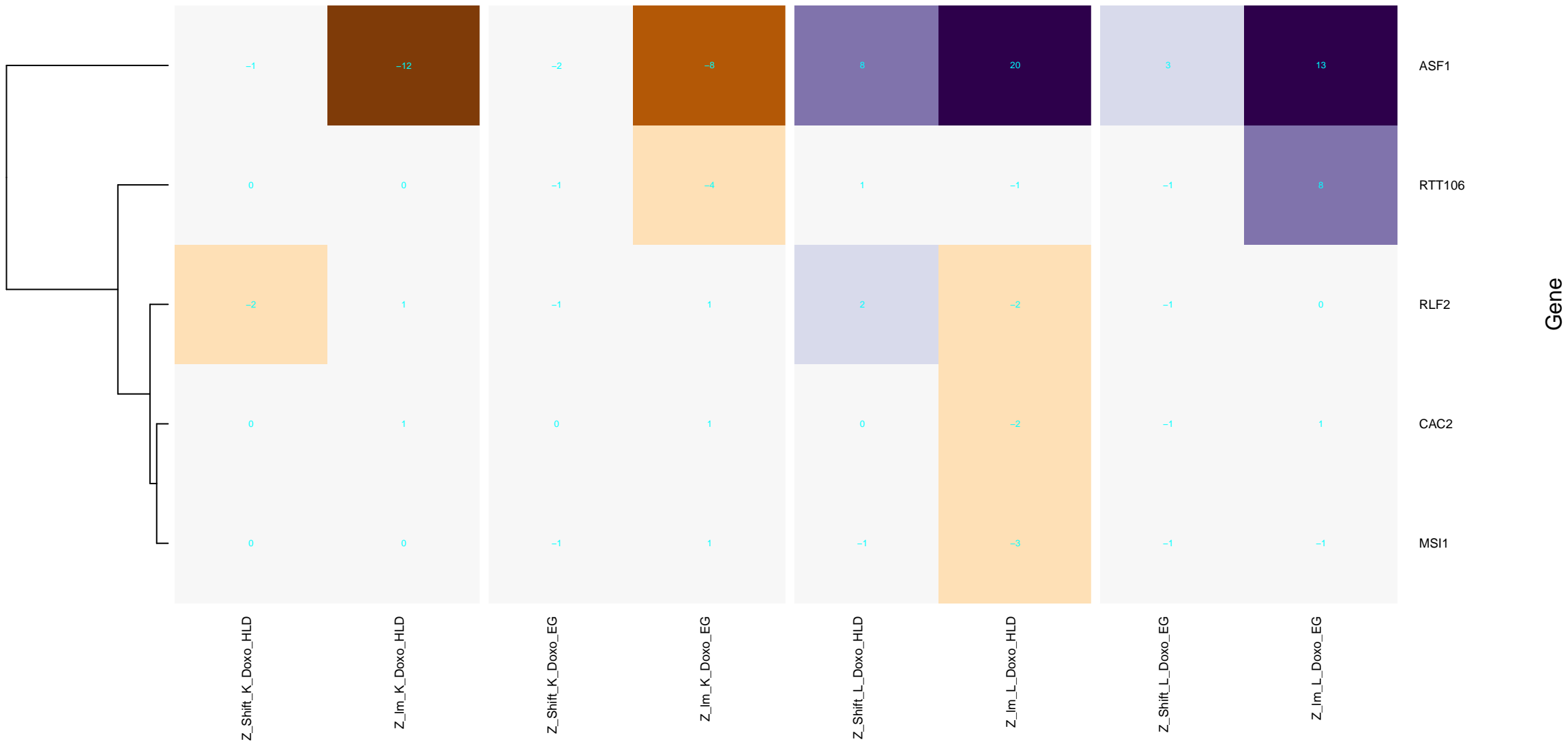

Supplement: Supplementary file 9 — Additional file 9. GO term-specific heatmaps for REMc/GTF-enriched clusters. GO term-specific heatmaps for significant GO process terms were generated as described in methods and Figs. 3 and 4. Any related child terms are presented in subsequent pages of the parent file name. GO terms with more than 100 children, with 2 or fewer genes annotated to the term, or a file size over 300KB are not shown. All heatmaps are generated with the same layout (see Figs. 3 and 4). [file 40170_2019_201_MOESM9_ESM.bz2 › Additional_File9_GOTermHeatmaps/Additional_File9_GOTermHeatmap/DNA_replication-dependent_nucleosome_assembly.pdf]

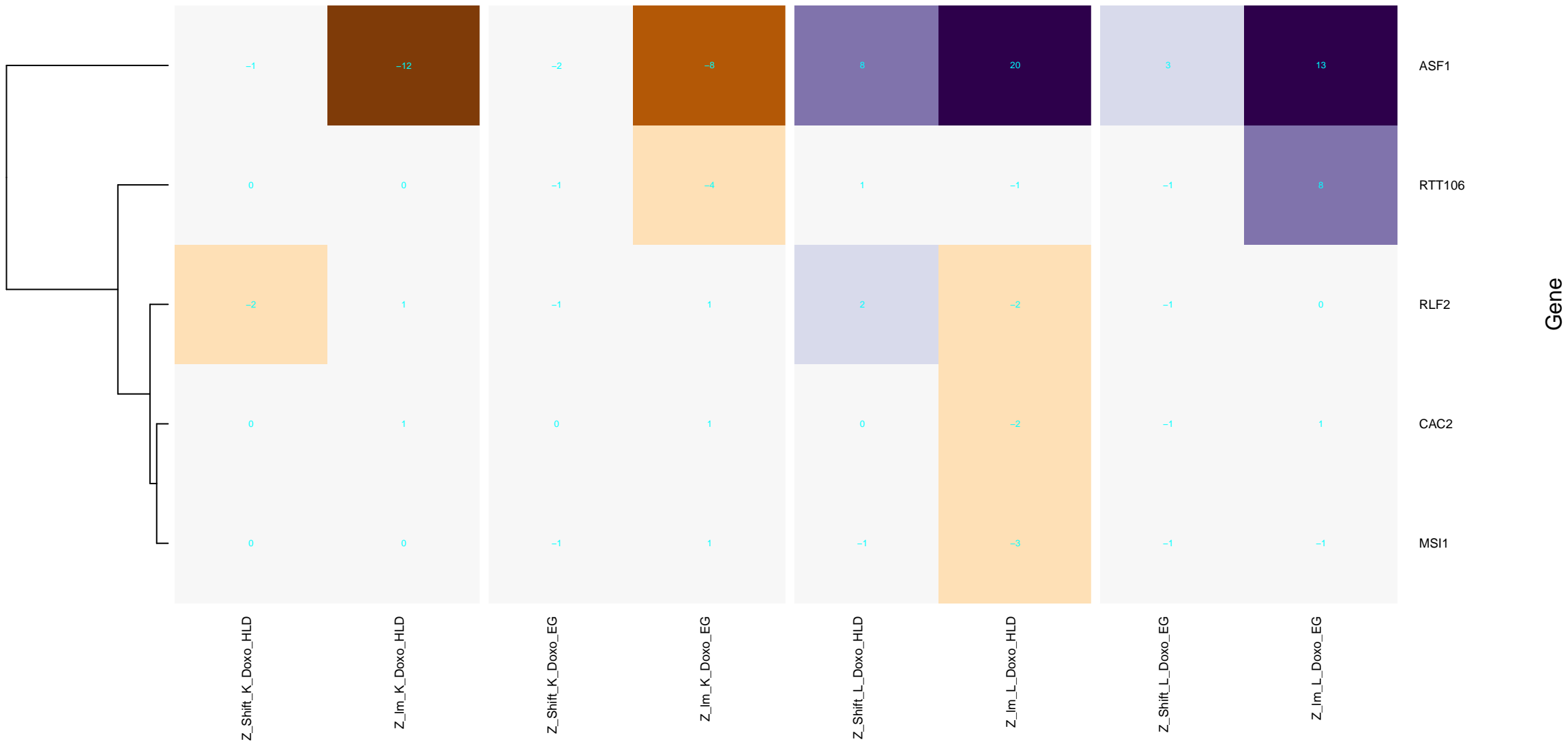

Supplement: Supplementary file 9 — Additional file 9. GO term-specific heatmaps for REMc/GTF-enriched clusters. GO term-specific heatmaps for significant GO process terms were generated as described in methods and Figs. 3 and 4. Any related child terms are presented in subsequent pages of the parent file name. GO terms with more than 100 children, with 2 or fewer genes annotated to the term, or a file size over 300KB are not shown. All heatmaps are generated with the same layout (see Figs. 3 and 4). [file 40170_2019_201_MOESM9_ESM.bz2 › Additional_File9_GOTermHeatmaps/Additional_File9_GOTermHeatmap/DNA_replication-dependent_nucleosome_organization.pdf]

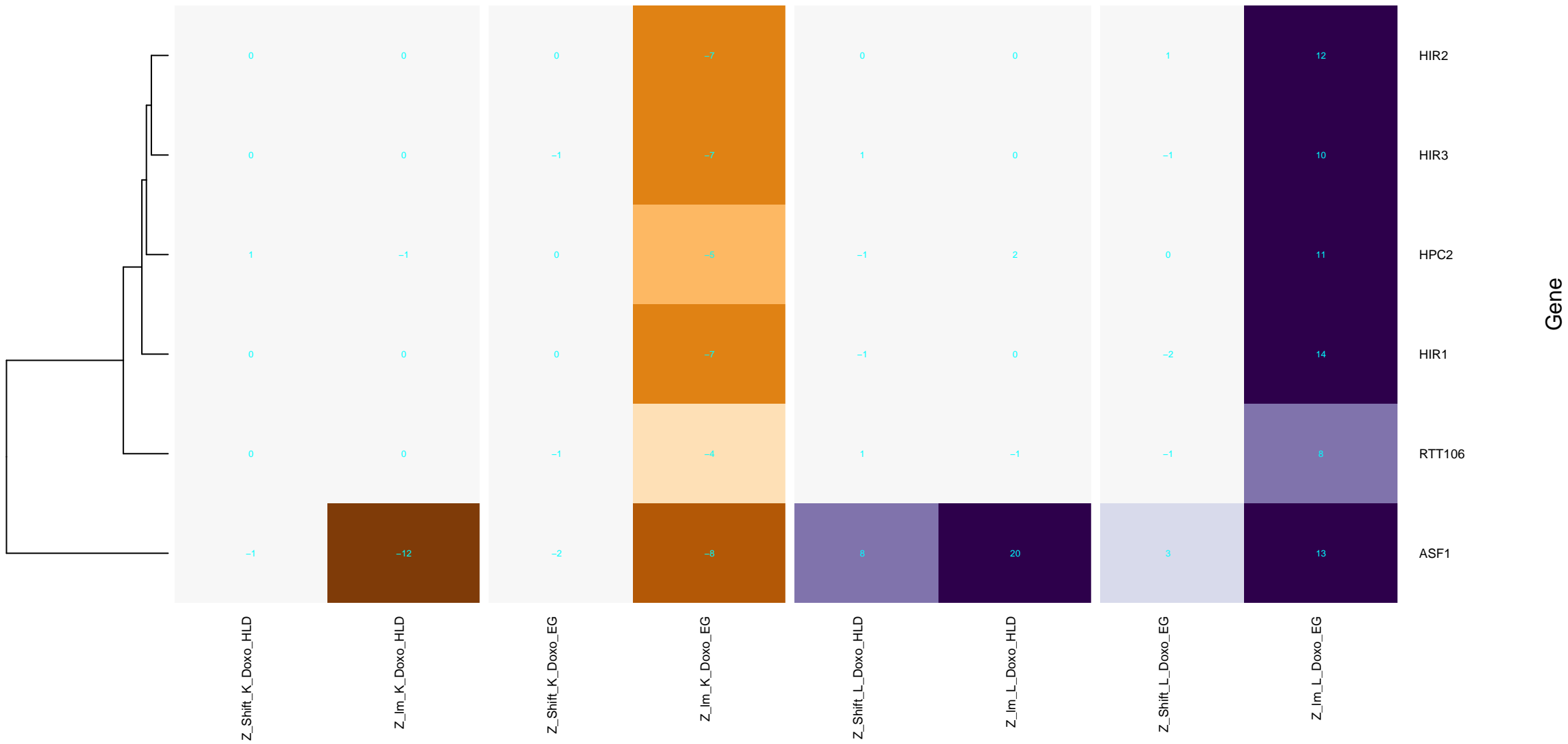

Supplement: Supplementary file 9 — Additional file 9. GO term-specific heatmaps for REMc/GTF-enriched clusters. GO term-specific heatmaps for significant GO process terms were generated as described in methods and Figs. 3 and 4. Any related child terms are presented in subsequent pages of the parent file name. GO terms with more than 100 children, with 2 or fewer genes annotated to the term, or a file size over 300KB are not shown. All heatmaps are generated with the same layout (see Figs. 3 and 4). [file 40170_2019_201_MOESM9_ESM.bz2 › Additional_File9_GOTermHeatmaps/Additional_File9_GOTermHeatmap/DNA_replication-independent_nucleosome_assembly.pdf]

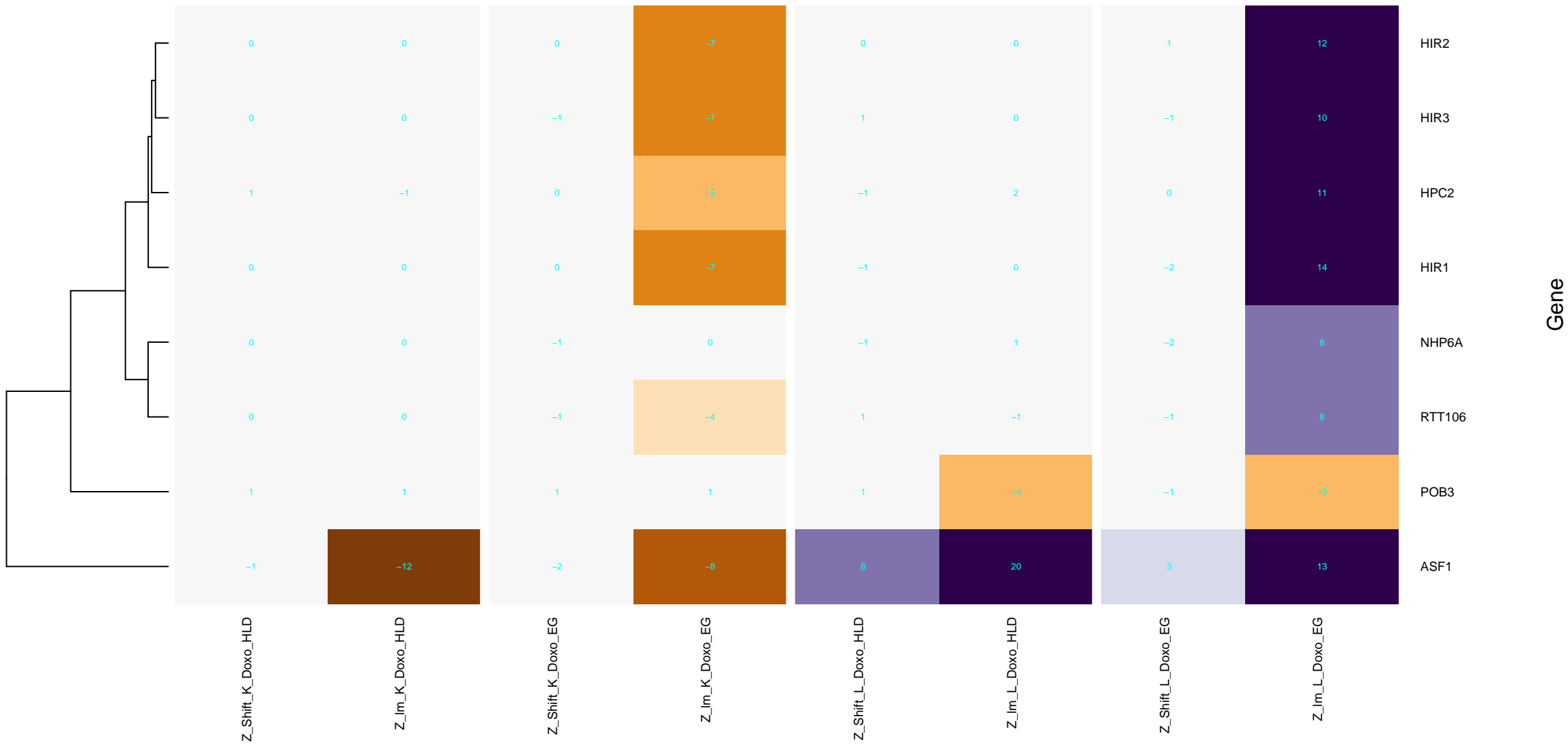

Supplement: Supplementary file 9 — Additional file 9. GO term-specific heatmaps for REMc/GTF-enriched clusters. GO term-specific heatmaps for significant GO process terms were generated as described in methods and Figs. 3 and 4. Any related child terms are presented in subsequent pages of the parent file name. GO terms with more than 100 children, with 2 or fewer genes annotated to the term, or a file size over 300KB are not shown. All heatmaps are generated with the same layout (see Figs. 3 and 4). [file 40170_2019_201_MOESM9_ESM.bz2 › Additional_File9_GOTermHeatmaps/Additional_File9_GOTermHeatmap/DNA_replication-independent_nucleosome_organization.pdf]

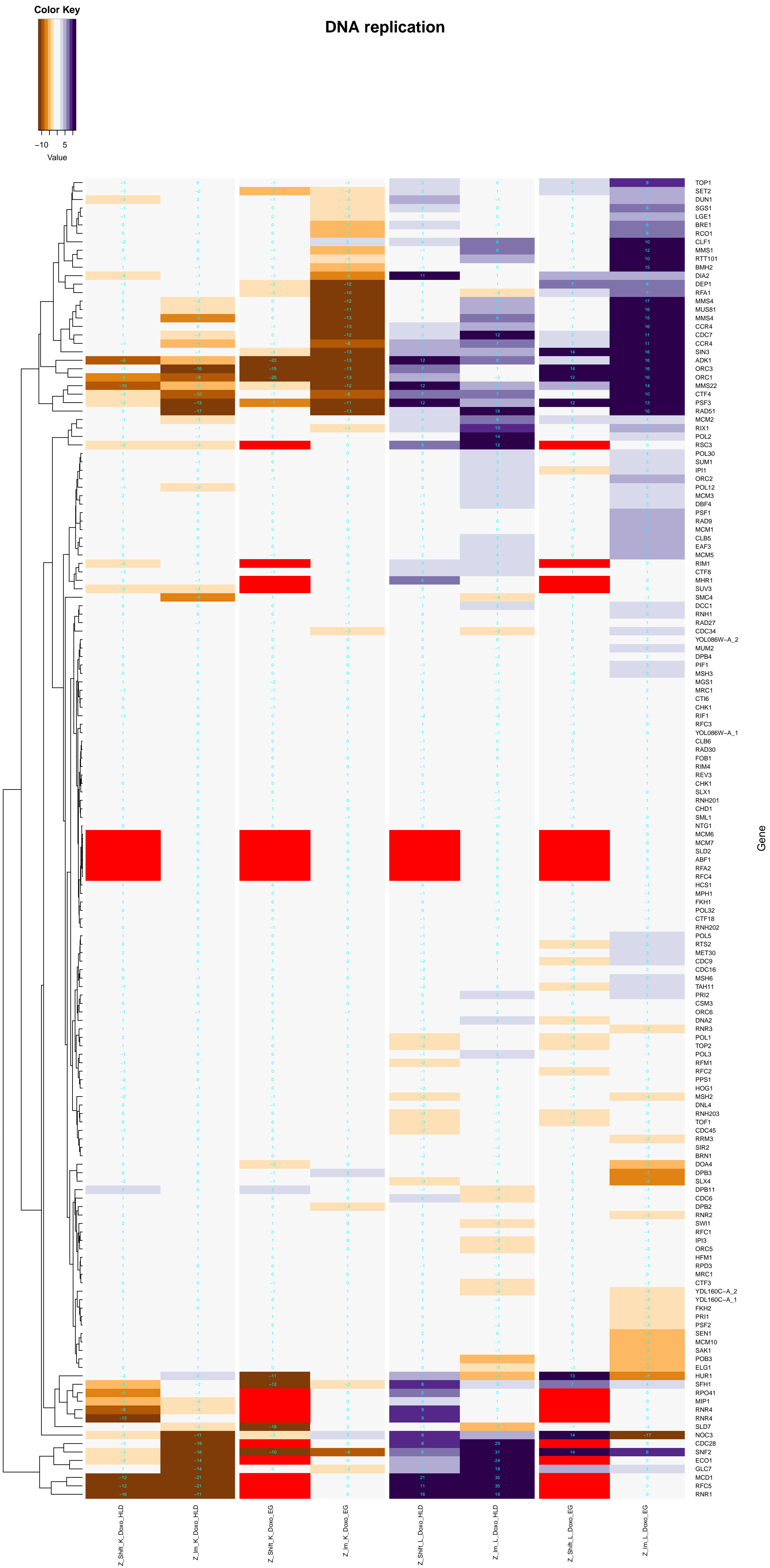



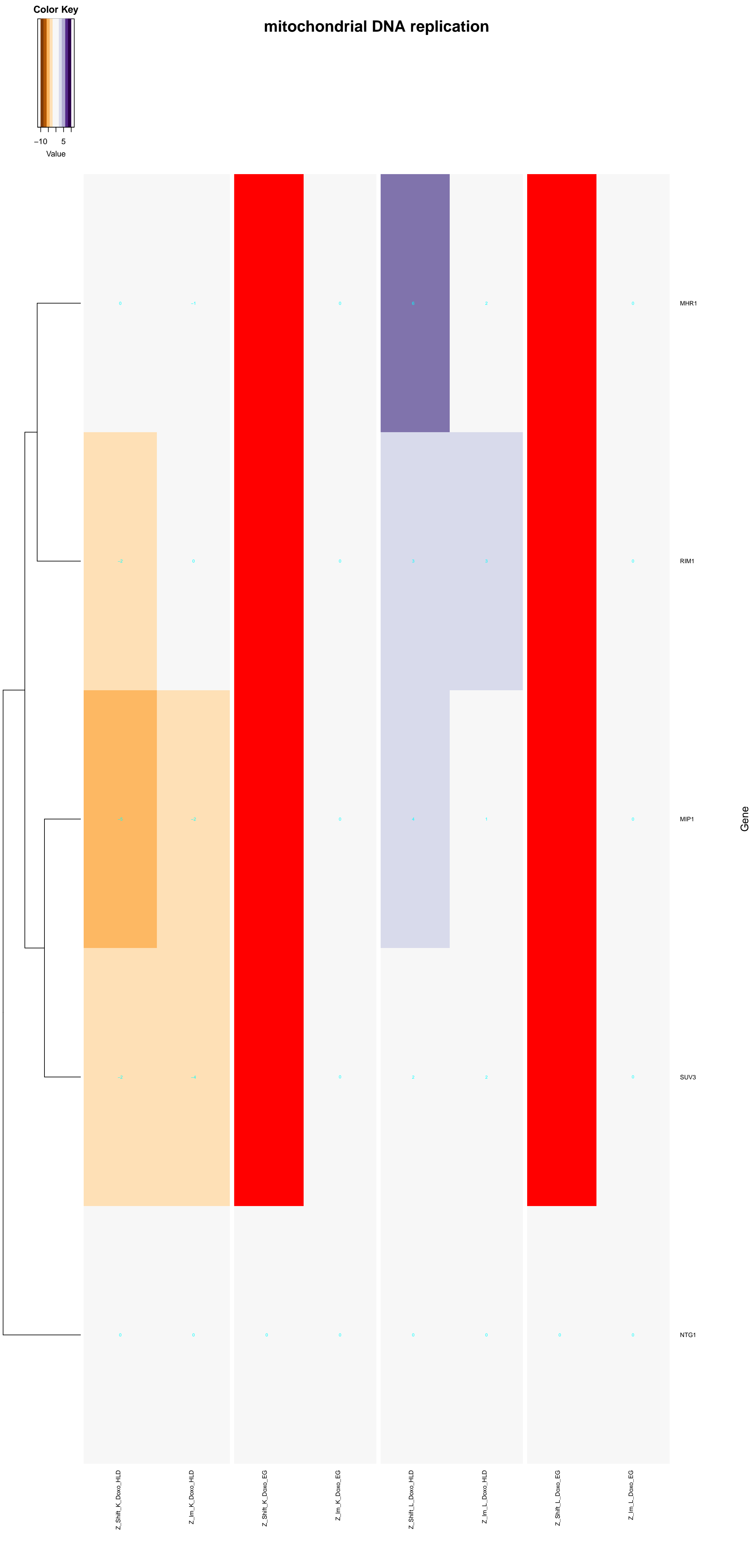

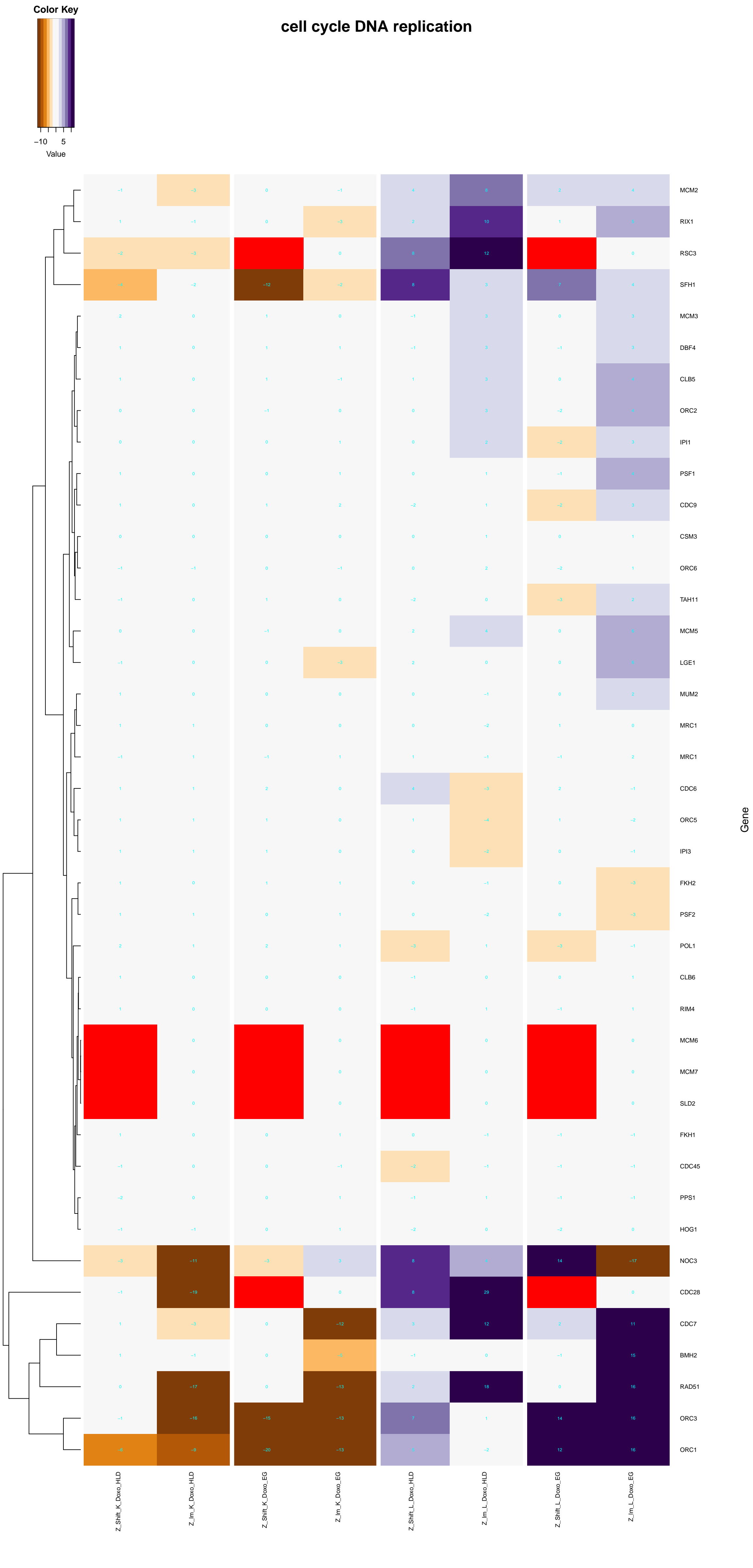

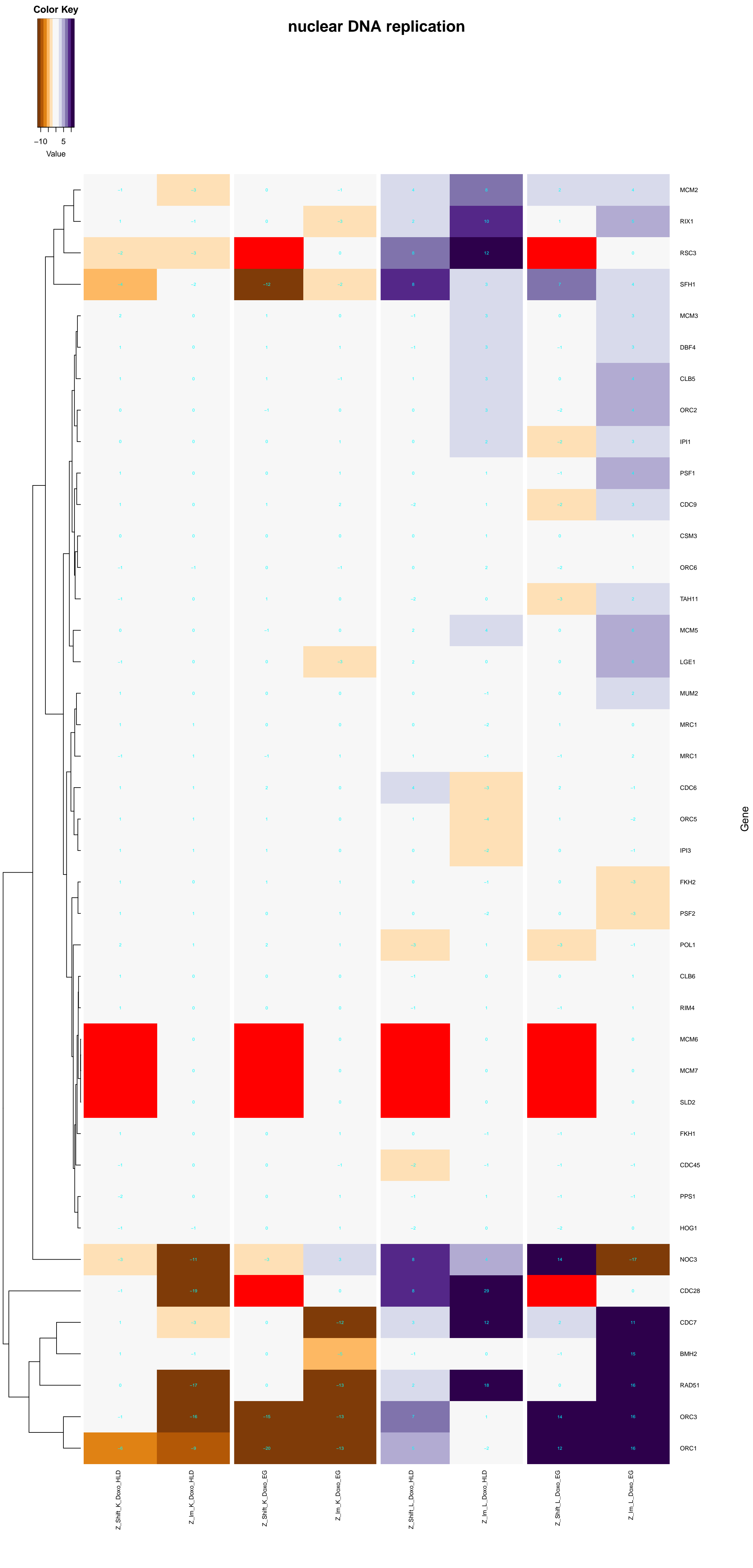

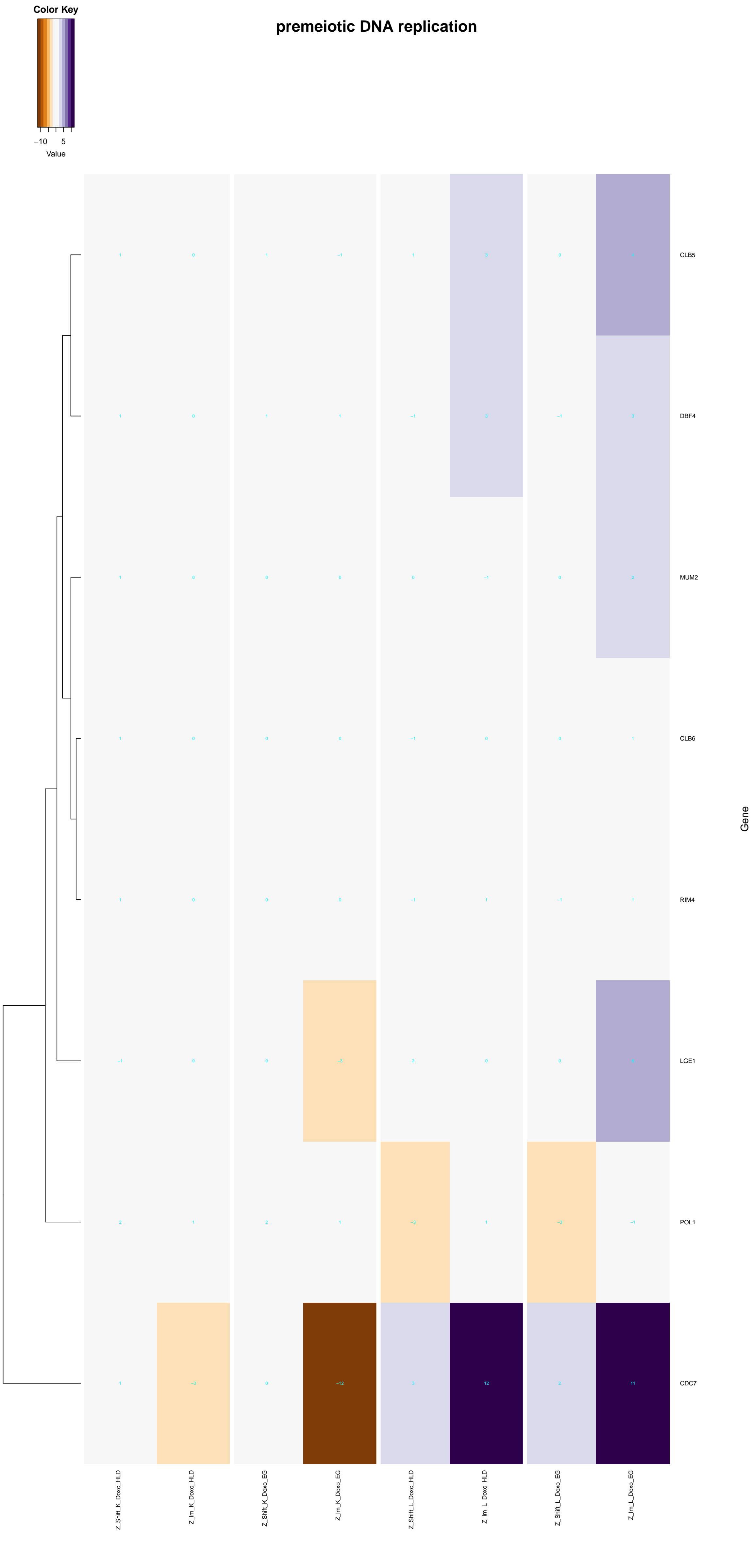

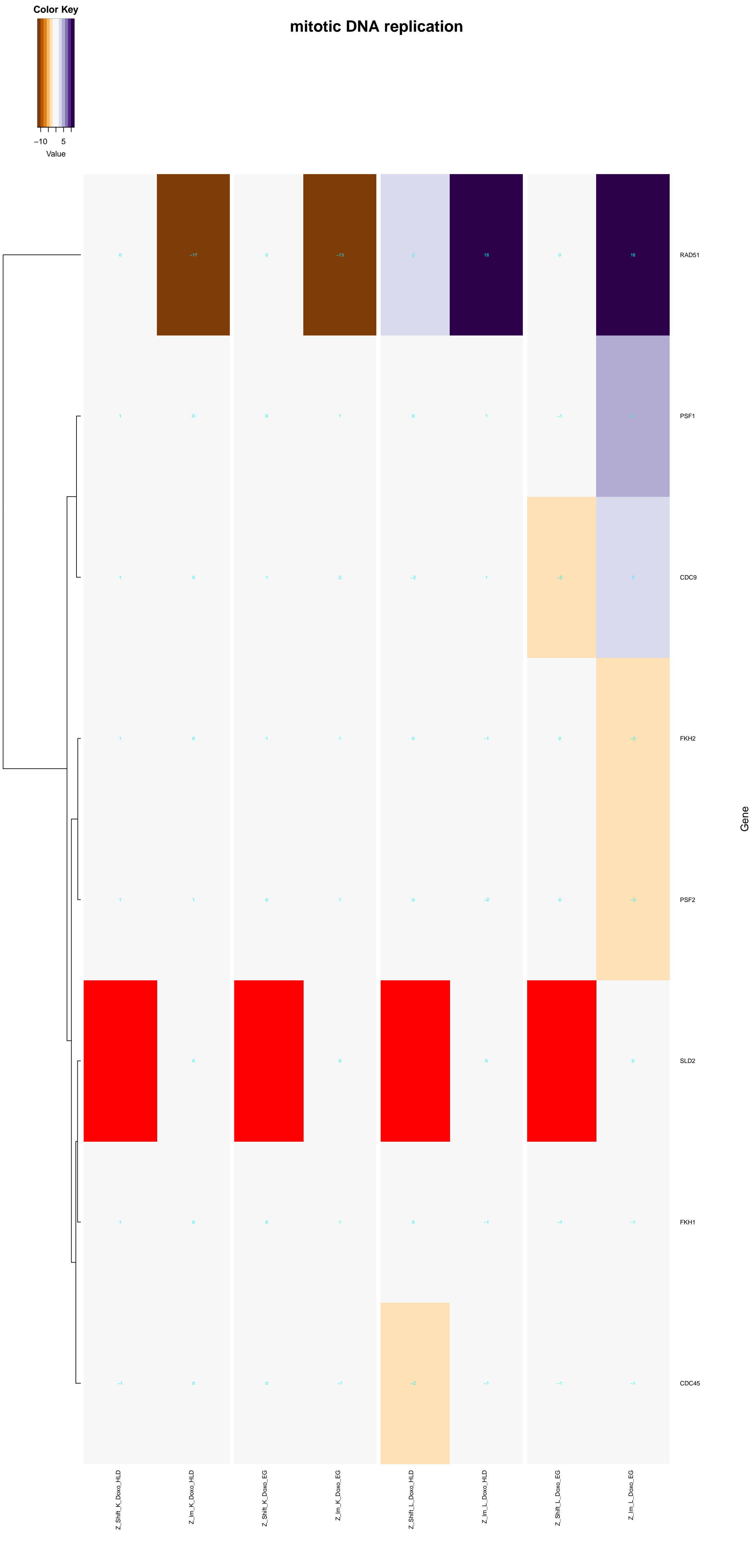

Supplement: Supplementary file 9 — Additional file 9. GO term-specific heatmaps for REMc/GTF-enriched clusters. GO term-specific heatmaps for significant GO process terms were generated as described in methods and Figs. 3 and 4. Any related child terms are presented in subsequent pages of the parent file name. GO terms with more than 100 children, with 2 or fewer genes annotated to the term, or a file size over 300KB are not shown. All heatmaps are generated with the same layout (see Figs. 3 and 4). [file 40170_2019_201_MOESM9_ESM.bz2 › Additional_File9_GOTermHeatmaps/Additional_File9_GOTermHeatmap/DNA_replication.pdf]

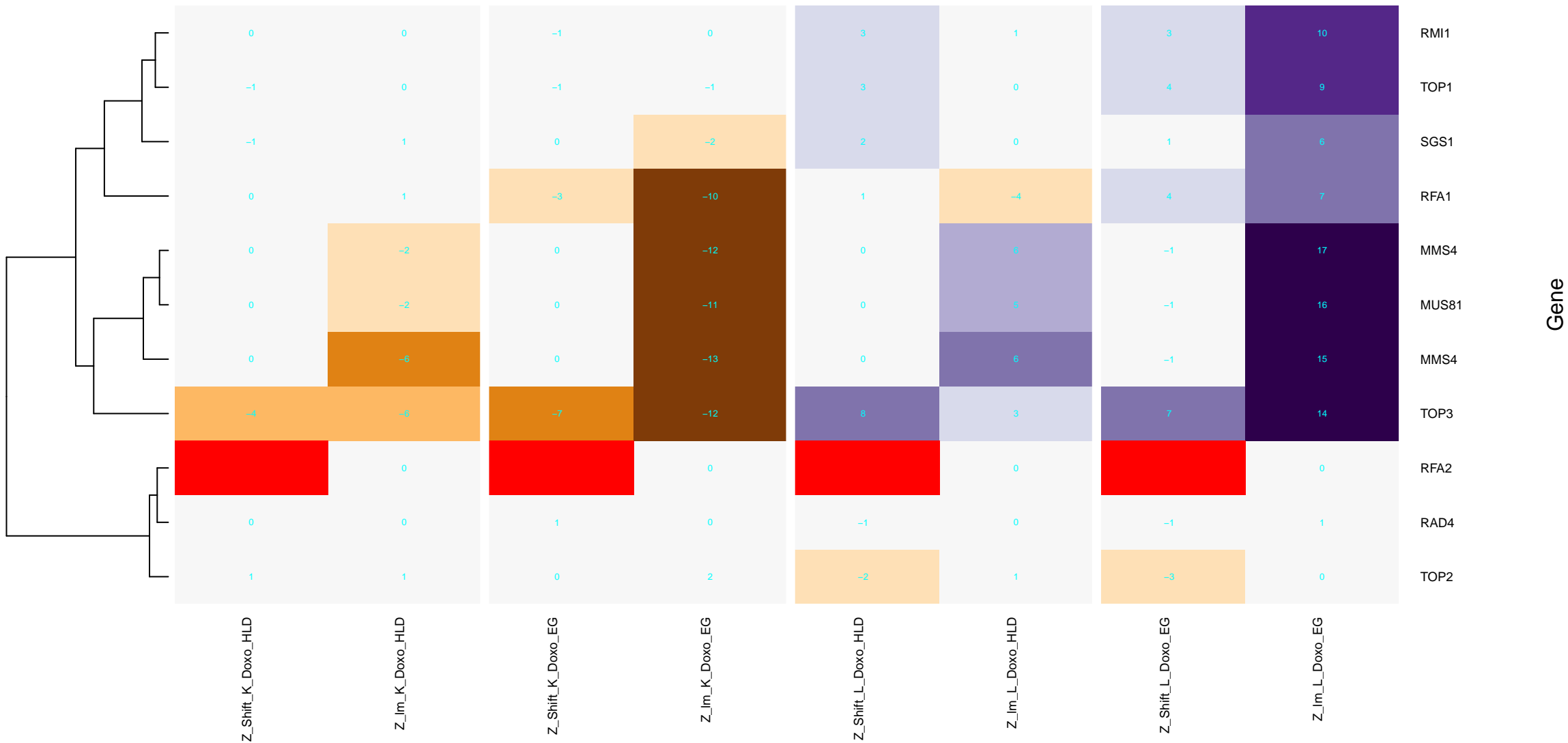

Supplement: Supplementary file 9 — Additional file 9. GO term-specific heatmaps for REMc/GTF-enriched clusters. GO term-specific heatmaps for significant GO process terms were generated as described in methods and Figs. 3 and 4. Any related child terms are presented in subsequent pages of the parent file name. GO terms with more than 100 children, with 2 or fewer genes annotated to the term, or a file size over 300KB are not shown. All heatmaps are generated with the same layout (see Figs. 3 and 4). [file 40170_2019_201_MOESM9_ESM.bz2 › Additional_File9_GOTermHeatmaps/Additional_File9_GOTermHeatmap/DNA_topological_change.pdf]

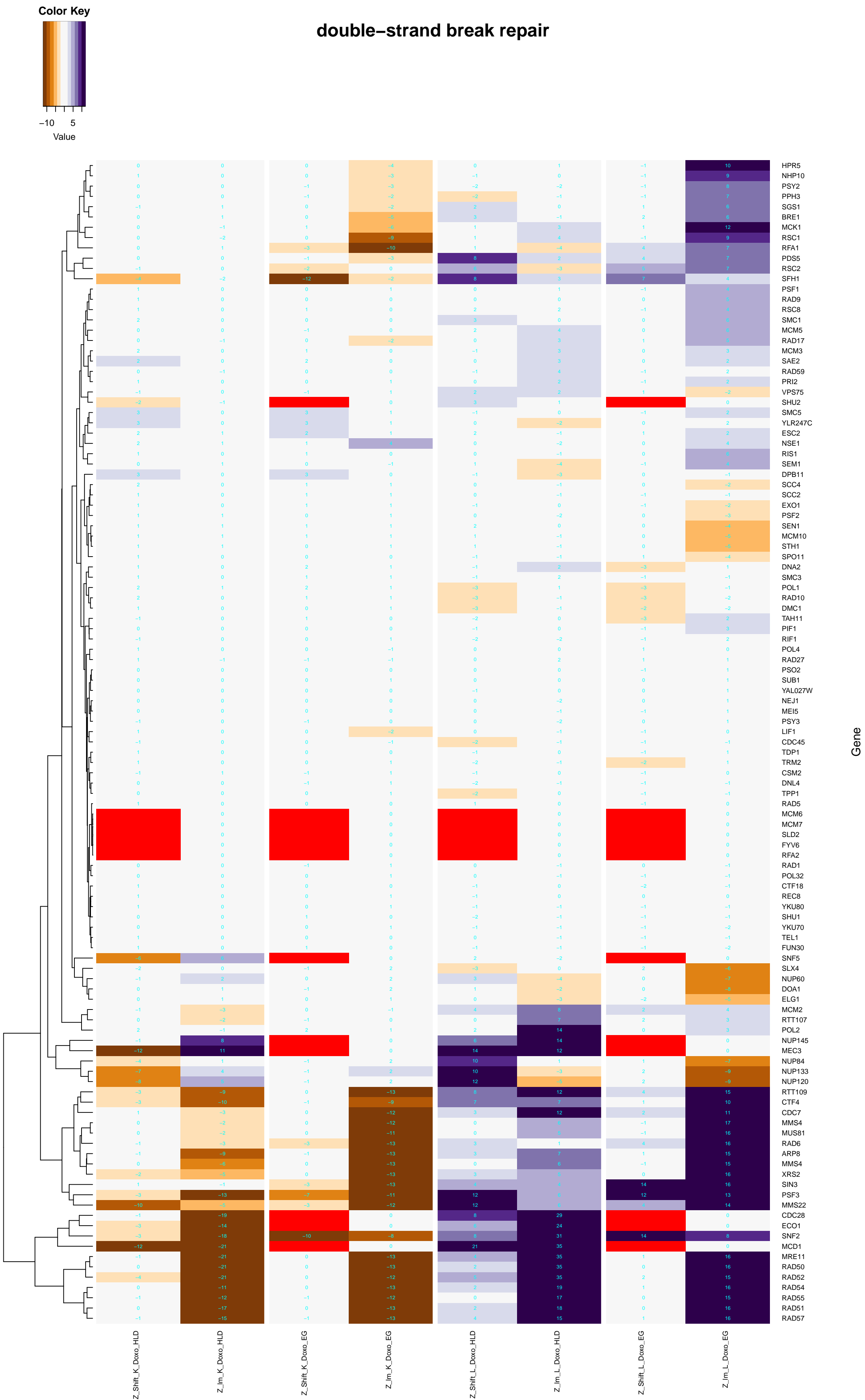

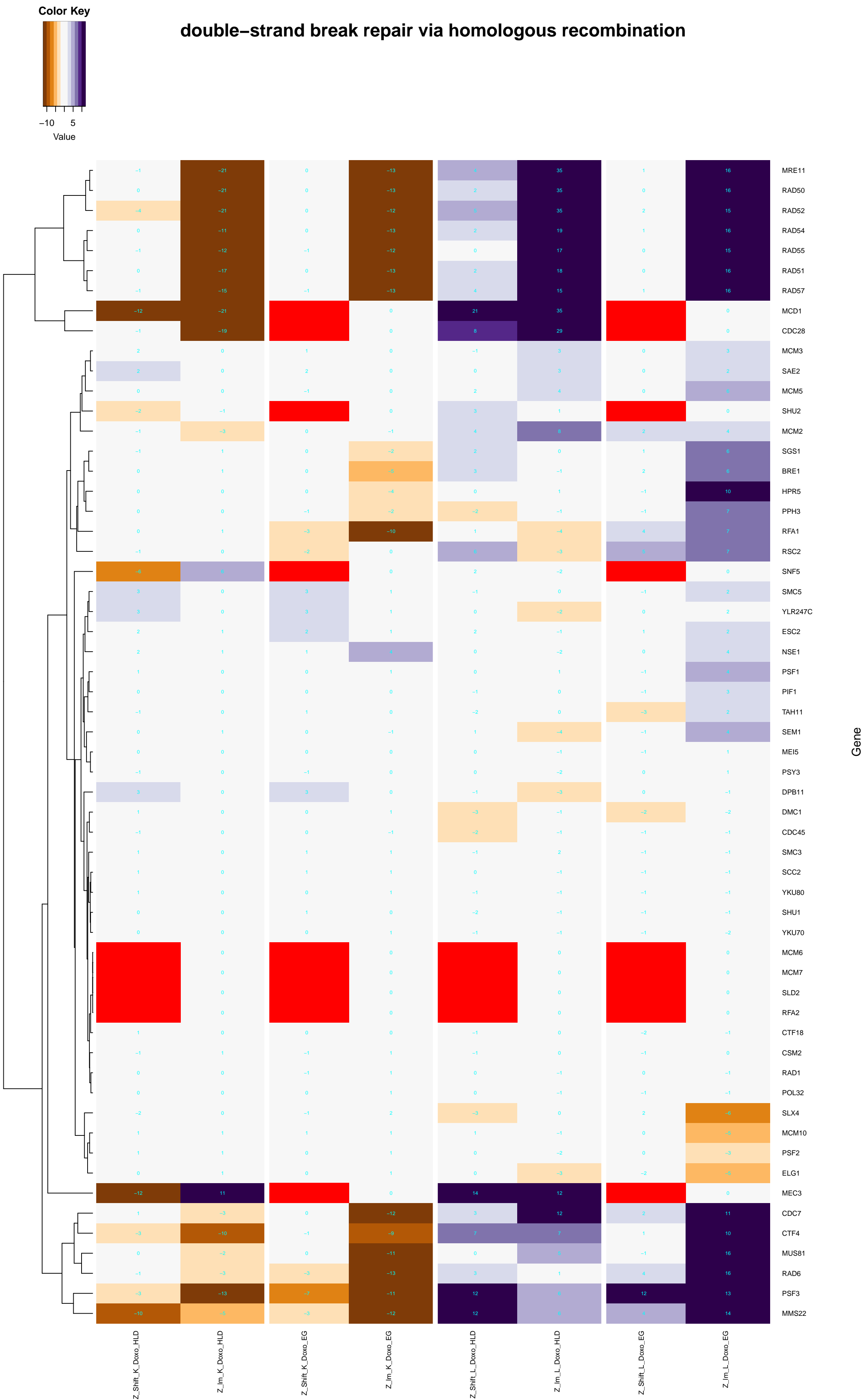

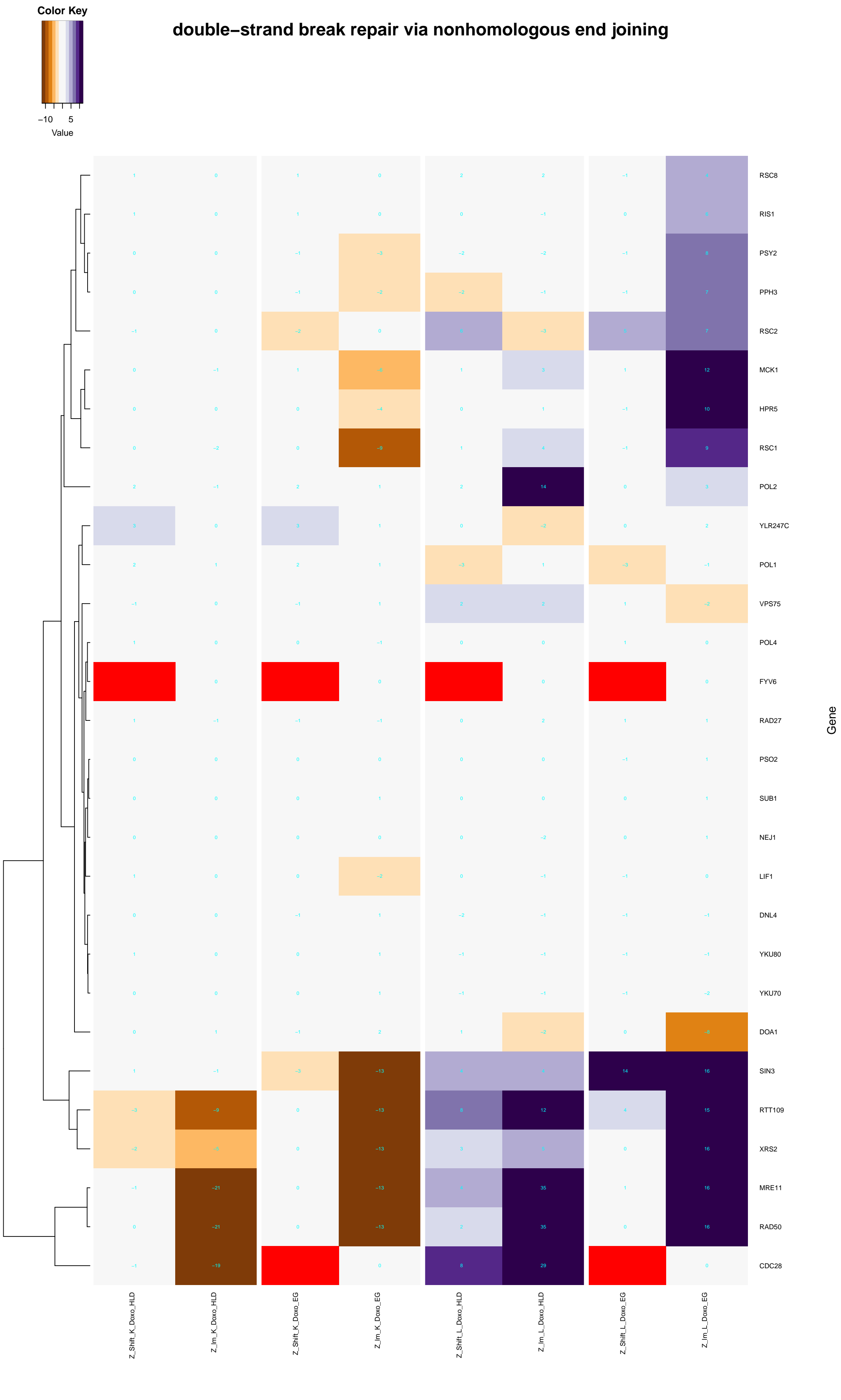

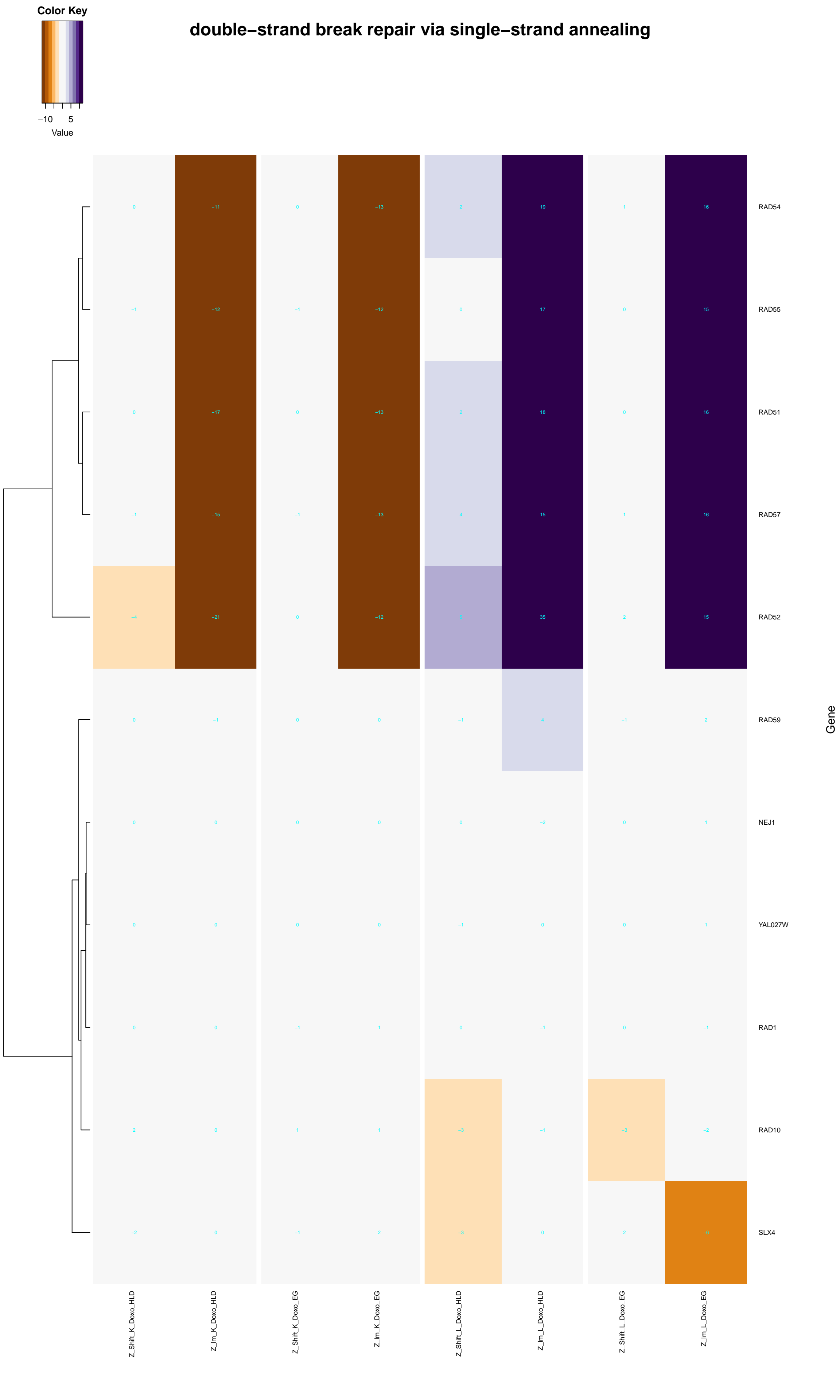

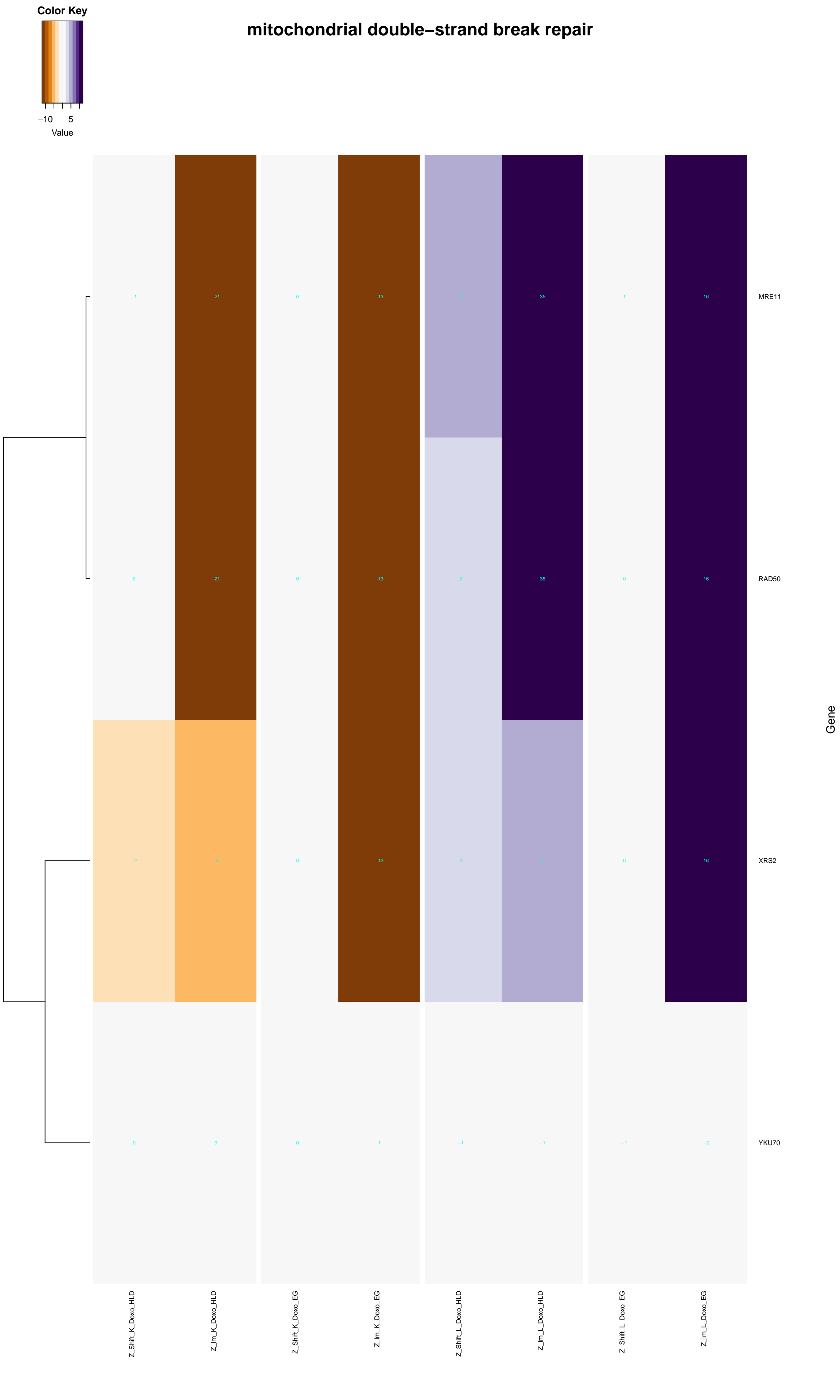

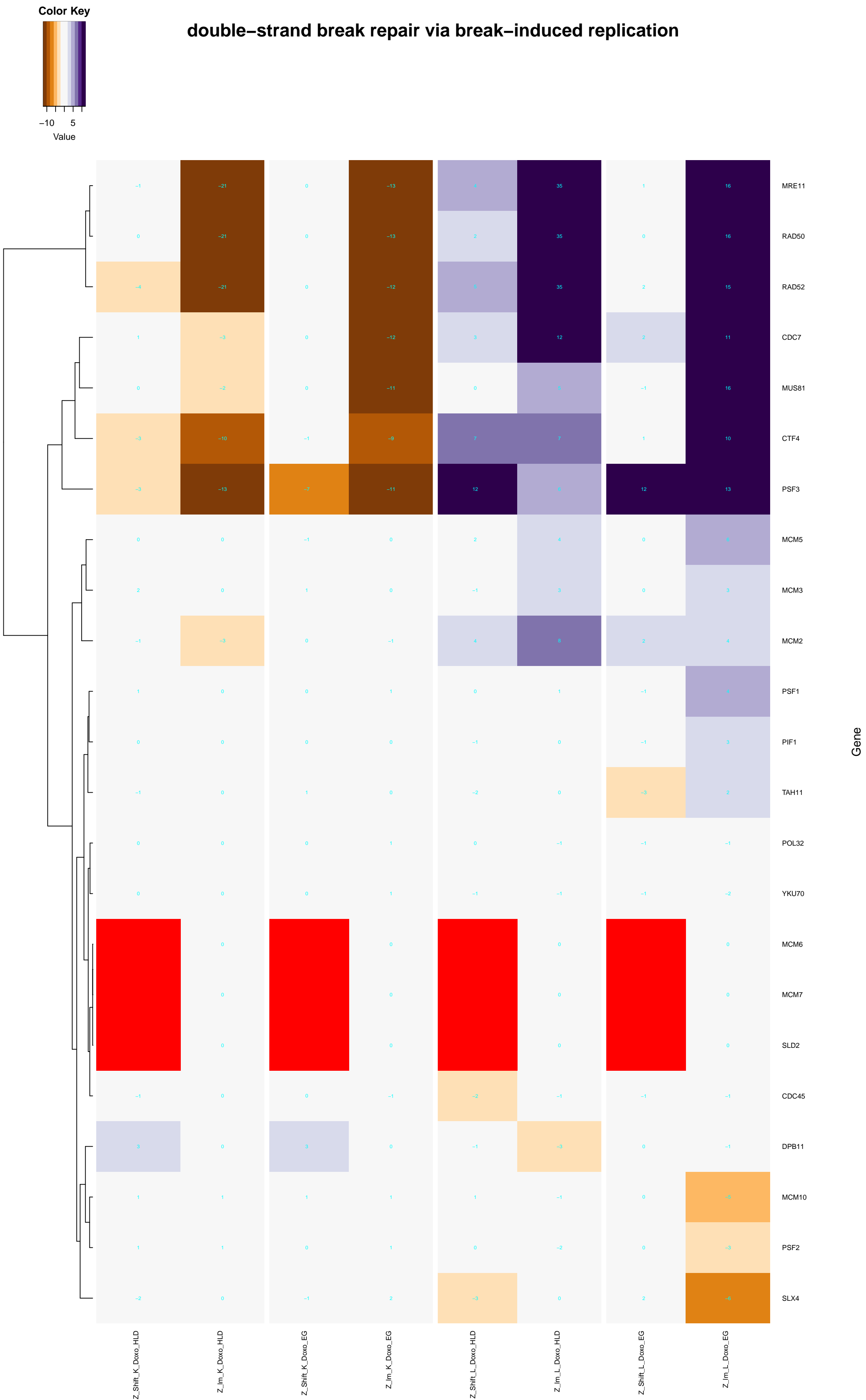

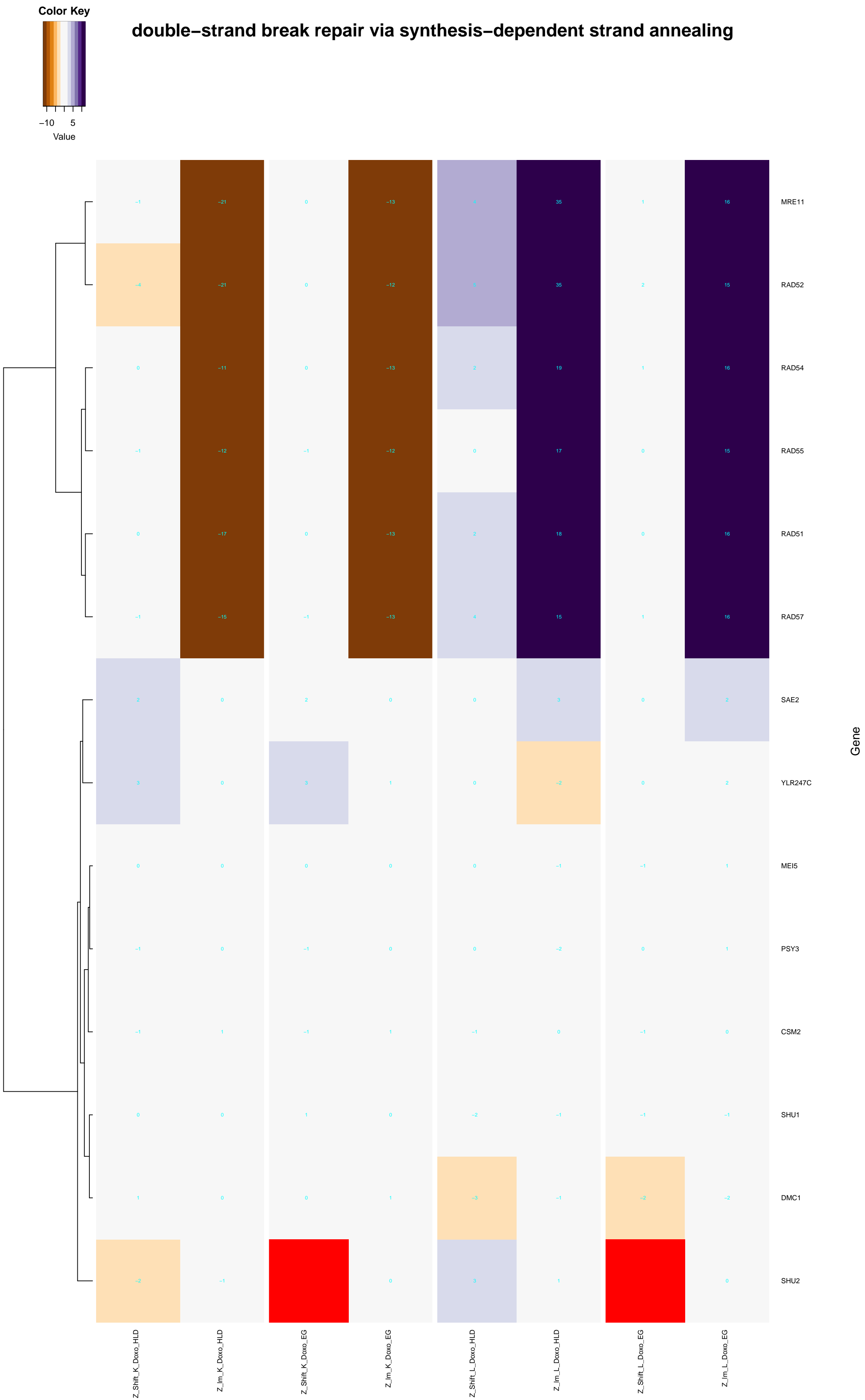

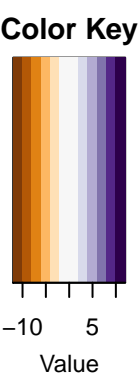

replication-born double-strand break repair via sister chromatid exchange

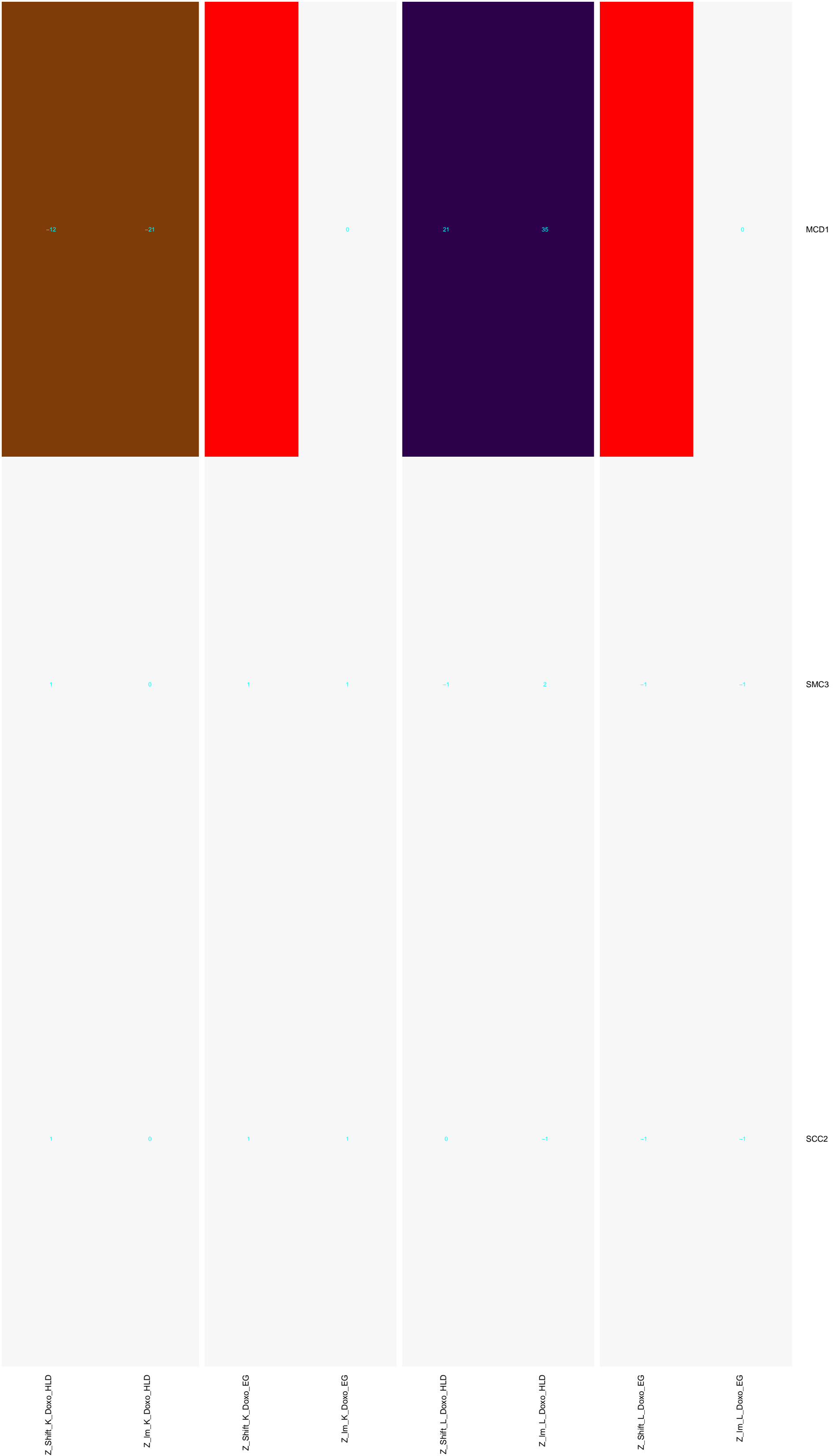

Gene

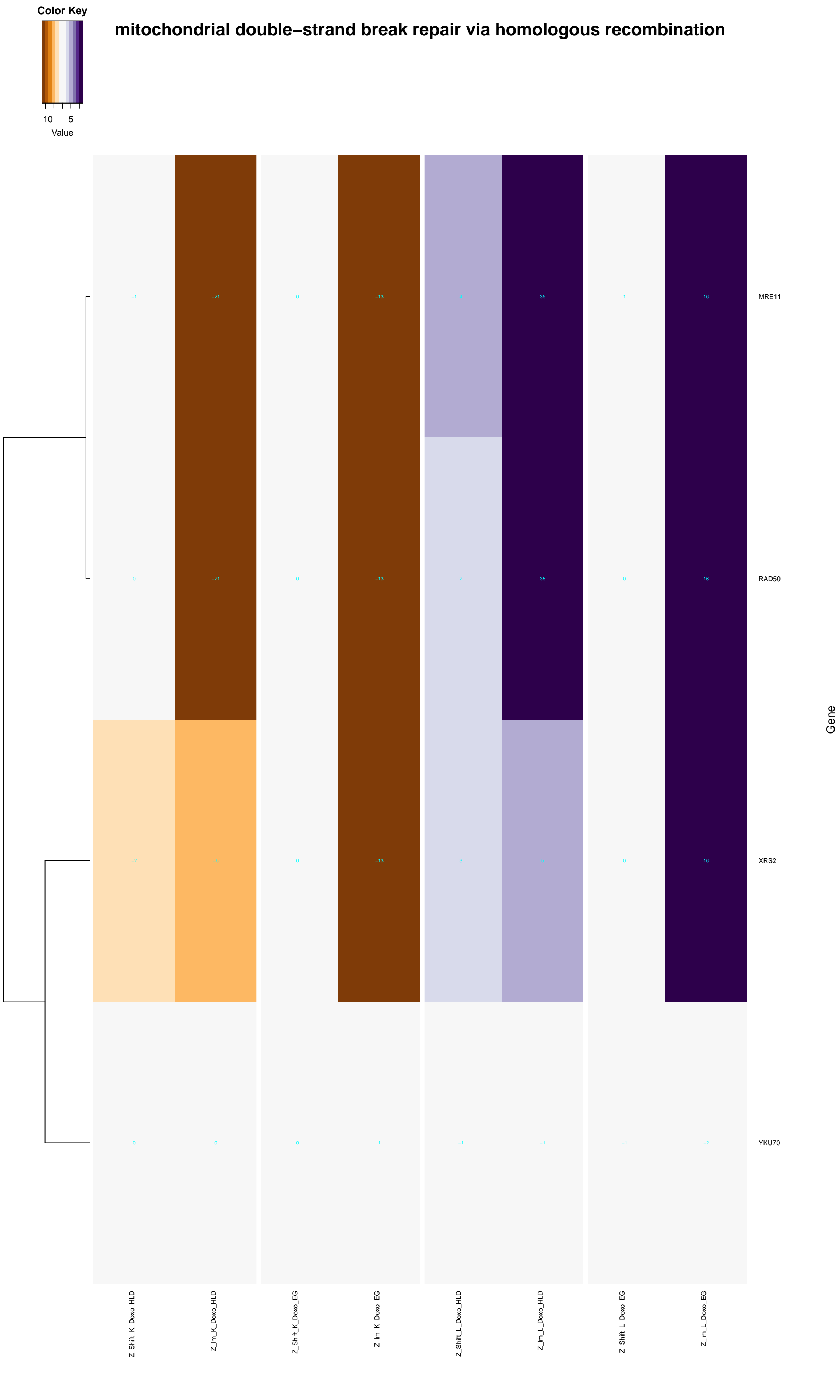

Supplement: Supplementary file 9 — Additional file 9. GO term-specific heatmaps for REMc/GTF-enriched clusters. GO term-specific heatmaps for significant GO process terms were generated as described in methods and Figs. 3 and 4. Any related child terms are presented in subsequent pages of the parent file name. GO terms with more than 100 children, with 2 or fewer genes annotated to the term, or a file size over 300KB are not shown. All heatmaps are generated with the same layout (see Figs. 3 and 4). [file 40170_2019_201_MOESM9_ESM.bz2 › Additional_File9_GOTermHeatmaps/Additional_File9_GOTermHeatmap/double-strand_break_repair.pdf]

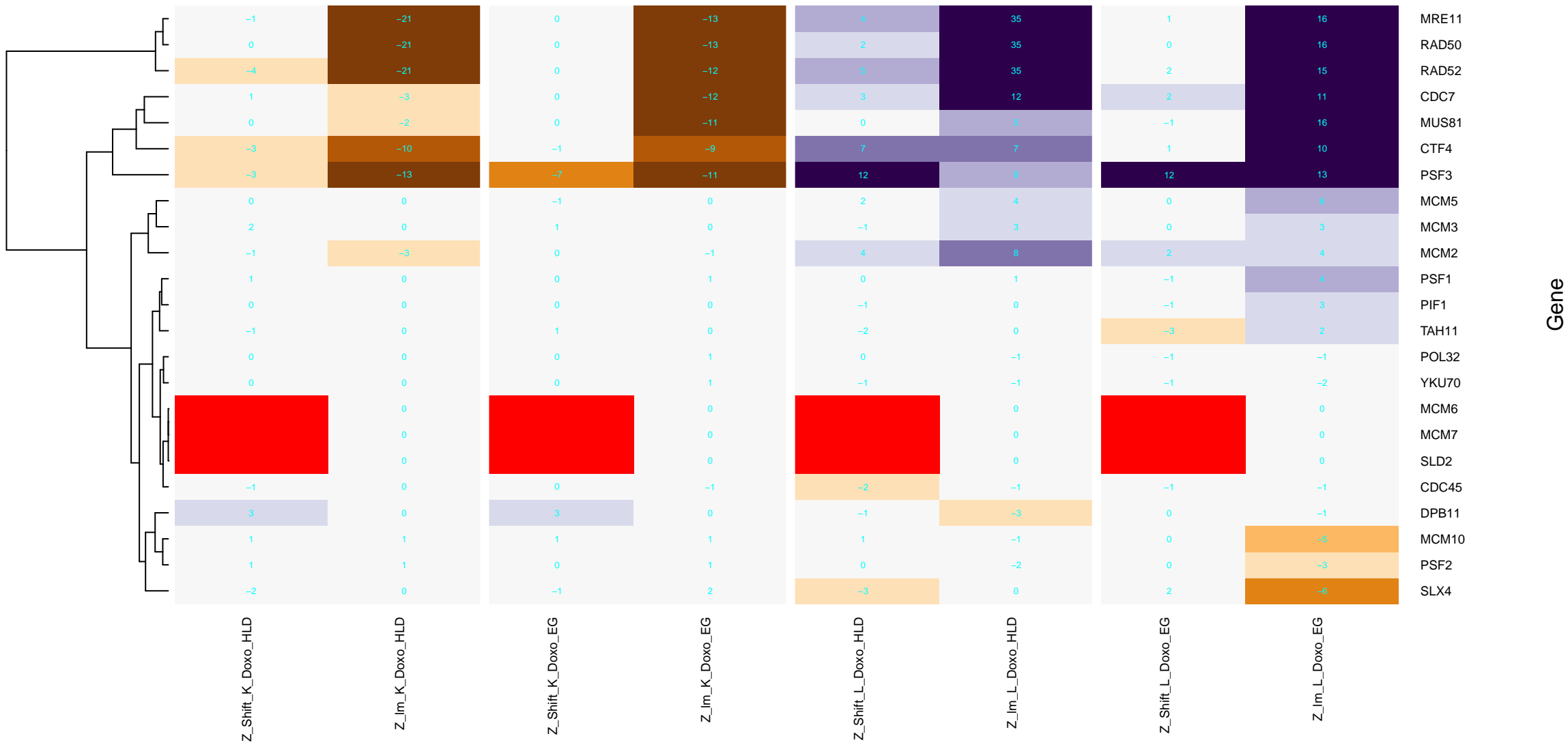

Supplement: Supplementary file 9 — Additional file 9. GO term-specific heatmaps for REMc/GTF-enriched clusters. GO term-specific heatmaps for significant GO process terms were generated as described in methods and Figs. 3 and 4. Any related child terms are presented in subsequent pages of the parent file name. GO terms with more than 100 children, with 2 or fewer genes annotated to the term, or a file size over 300KB are not shown. All heatmaps are generated with the same layout (see Figs. 3 and 4). [file 40170_2019_201_MOESM9_ESM.bz2 › Additional_File9_GOTermHeatmaps/Additional_File9_GOTermHeatmap/double-strand_break_repair_via_break-induced_replication.pdf]

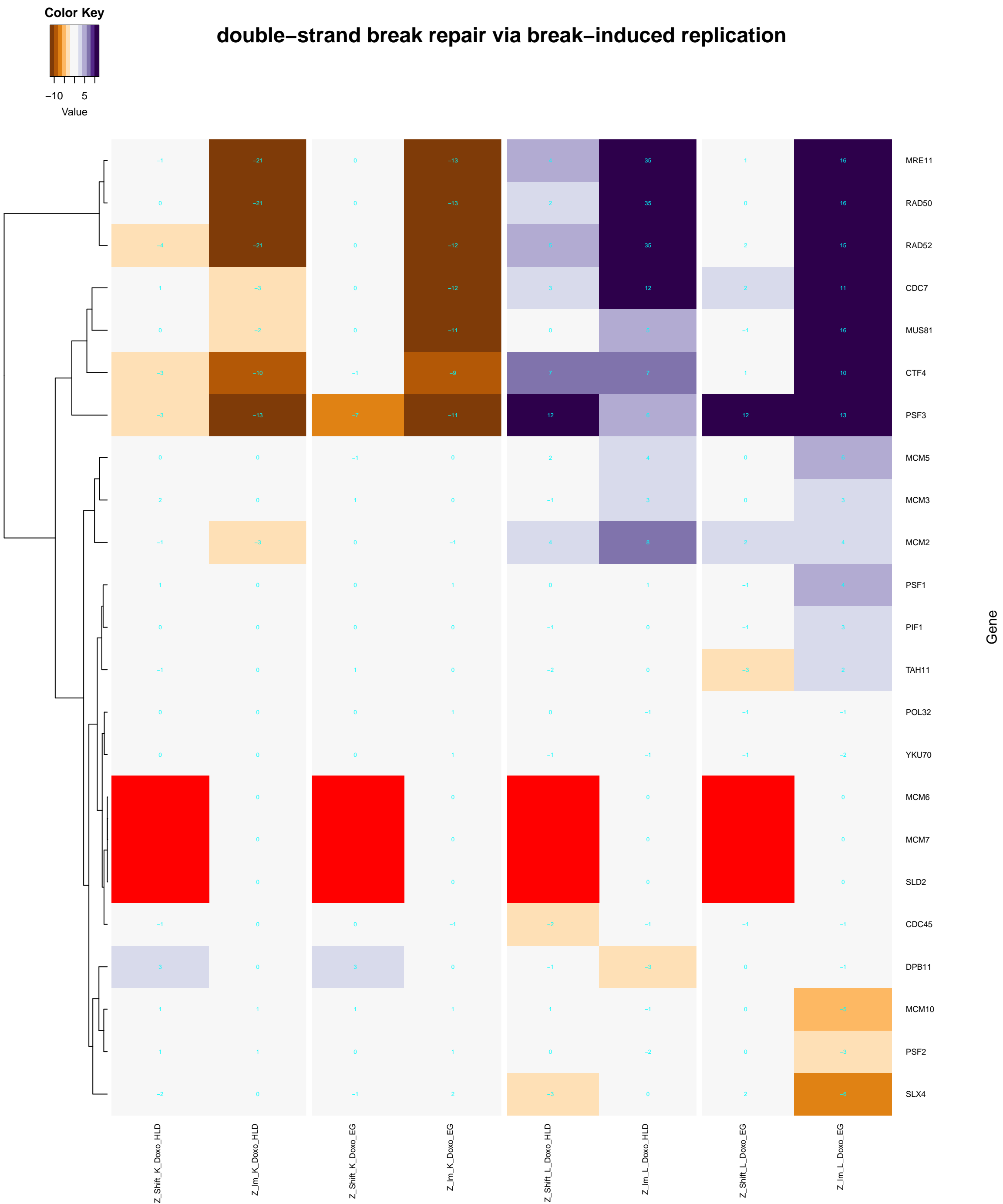

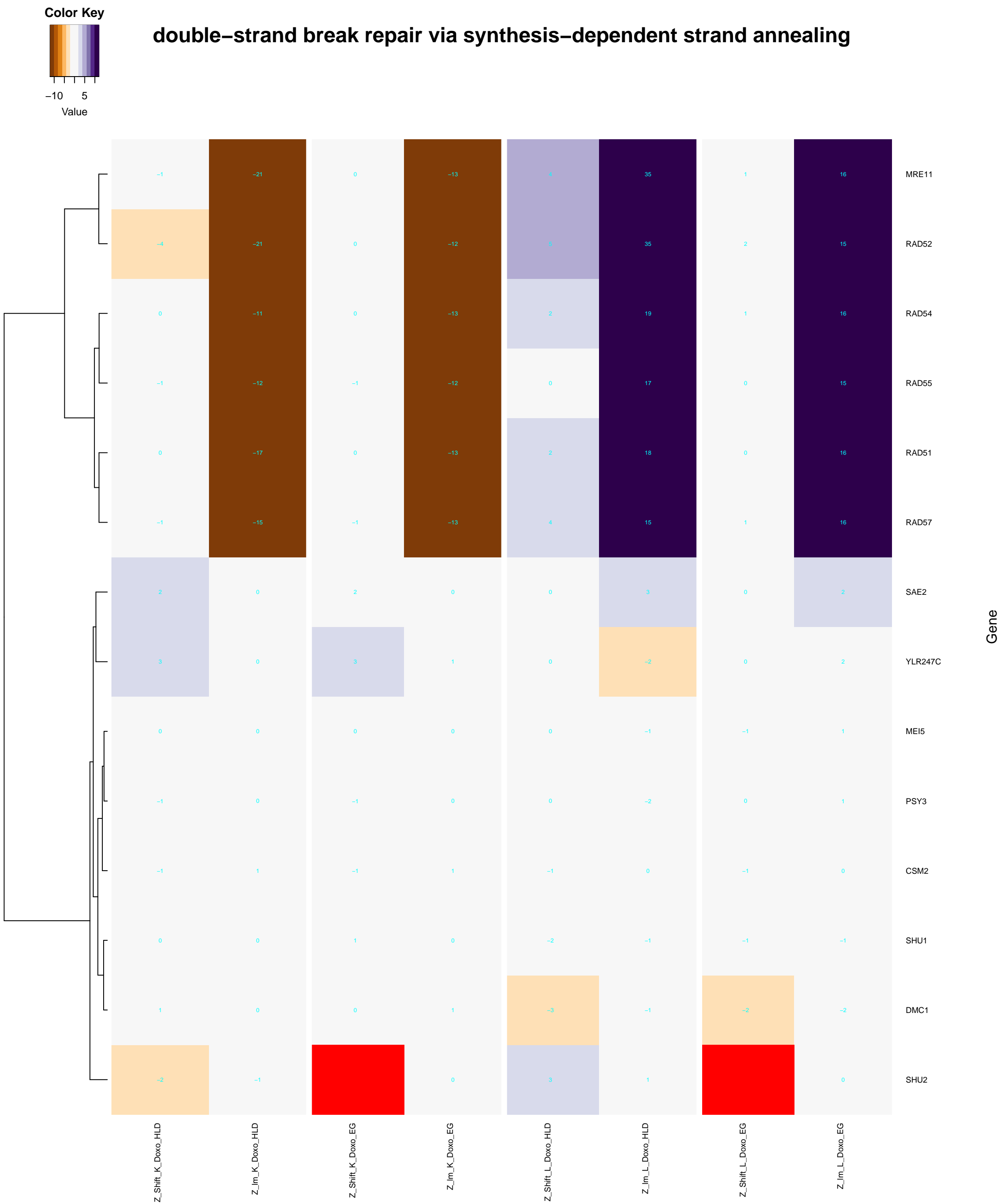

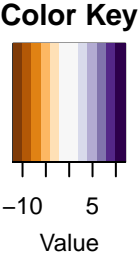

replication-born double-strand break repair via sister chromatid exchange

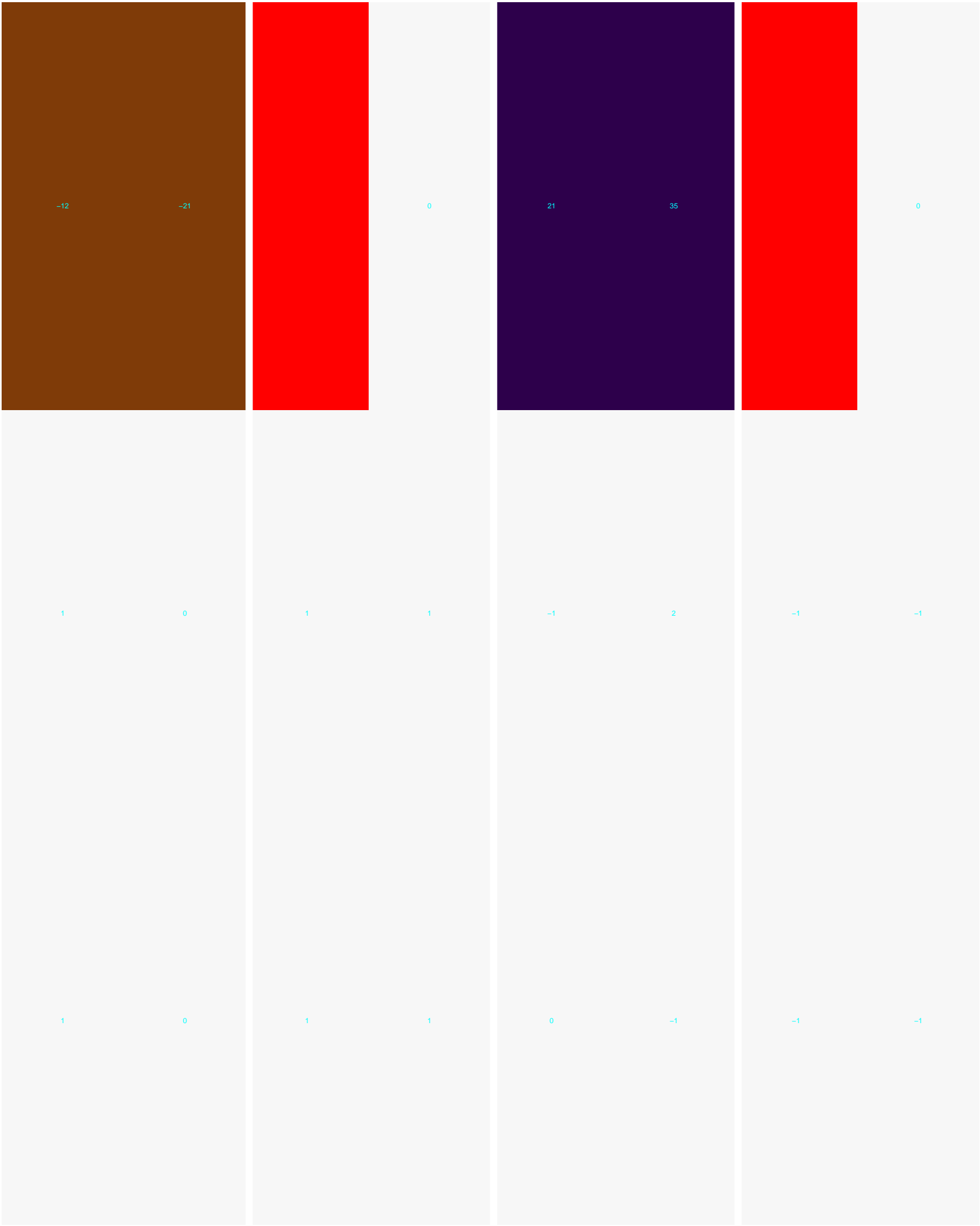

Gene

Supplement: Supplementary file 9 — Additional file 9. GO term-specific heatmaps for REMc/GTF-enriched clusters. GO term-specific heatmaps for significant GO process terms were generated as described in methods and Figs. 3 and 4. Any related child terms are presented in subsequent pages of the parent file name. GO terms with more than 100 children, with 2 or fewer genes annotated to the term, or a file size over 300KB are not shown. All heatmaps are generated with the same layout (see Figs. 3 and 4). [file 40170_2019_201_MOESM9_ESM.bz2 › Additional_File9_GOTermHeatmaps/Additional_File9_GOTermHeatmap/double-strand_break_repair_via_homologous_recombination.pdf]

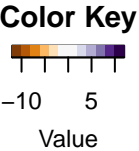

**double-strand break repair via nonhomologous end joining**

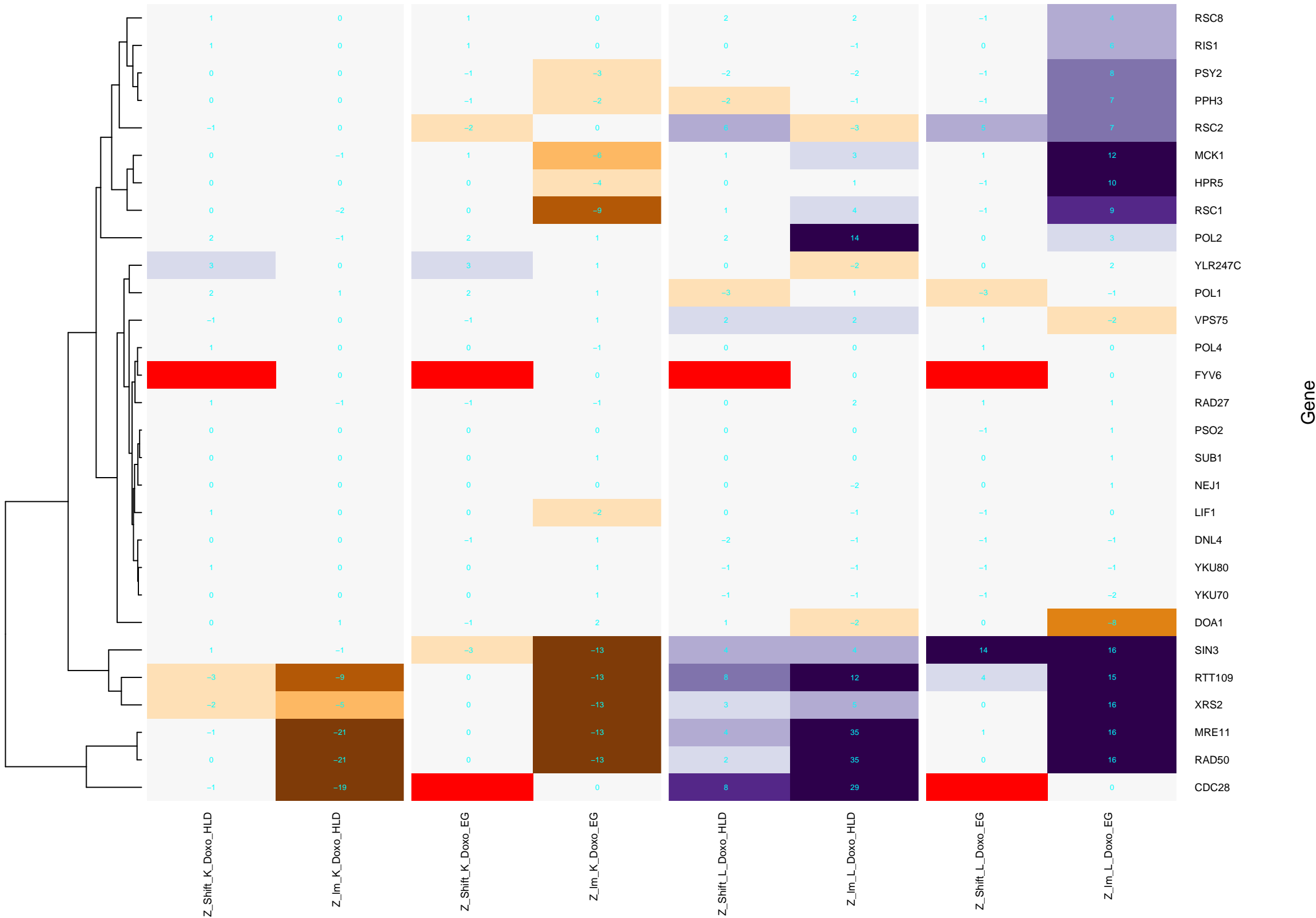

Supplement: Supplementary file 9 — Additional file 9. GO term-specific heatmaps for REMc/GTF-enriched clusters. GO term-specific heatmaps for significant GO process terms were generated as described in methods and Figs. 3 and 4. Any related child terms are presented in subsequent pages of the parent file name. GO terms with more than 100 children, with 2 or fewer genes annotated to the term, or a file size over 300KB are not shown. All heatmaps are generated with the same layout (see Figs. 3 and 4). [file 40170_2019_201_MOESM9_ESM.bz2 › Additional_File9_GOTermHeatmaps/Additional_File9_GOTermHeatmap/double-strand_break_repair_via_nonhomologous_end_joining.pdf]

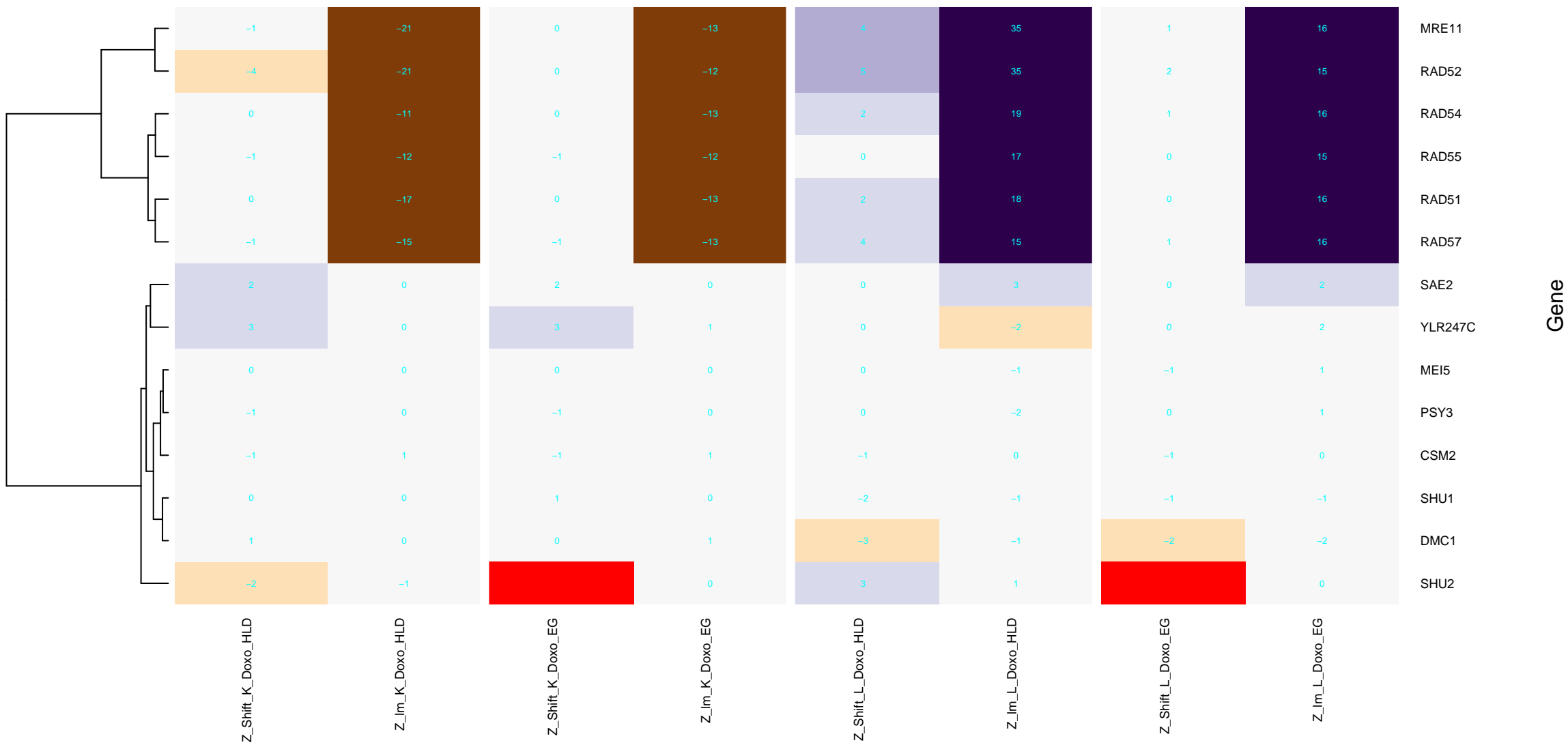

Supplement: Supplementary file 9 — Additional file 9. GO term-specific heatmaps for REMc/GTF-enriched clusters. GO term-specific heatmaps for significant GO process terms were generated as described in methods and Figs. 3 and 4. Any related child terms are presented in subsequent pages of the parent file name. GO terms with more than 100 children, with 2 or fewer genes annotated to the term, or a file size over 300KB are not shown. All heatmaps are generated with the same layout (see Figs. 3 and 4). [file 40170_2019_201_MOESM9_ESM.bz2 › Additional_File9_GOTermHeatmaps/Additional_File9_GOTermHeatmap/double-strand_break_repair_via_synthesis-dependent_strand_annealing.pdf]

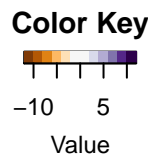

# lytic cleavage in 5'-ETS of tricistronic rRNA transcript (SSU-rRNA, 5.8S rRNA, LSU-rRNA)

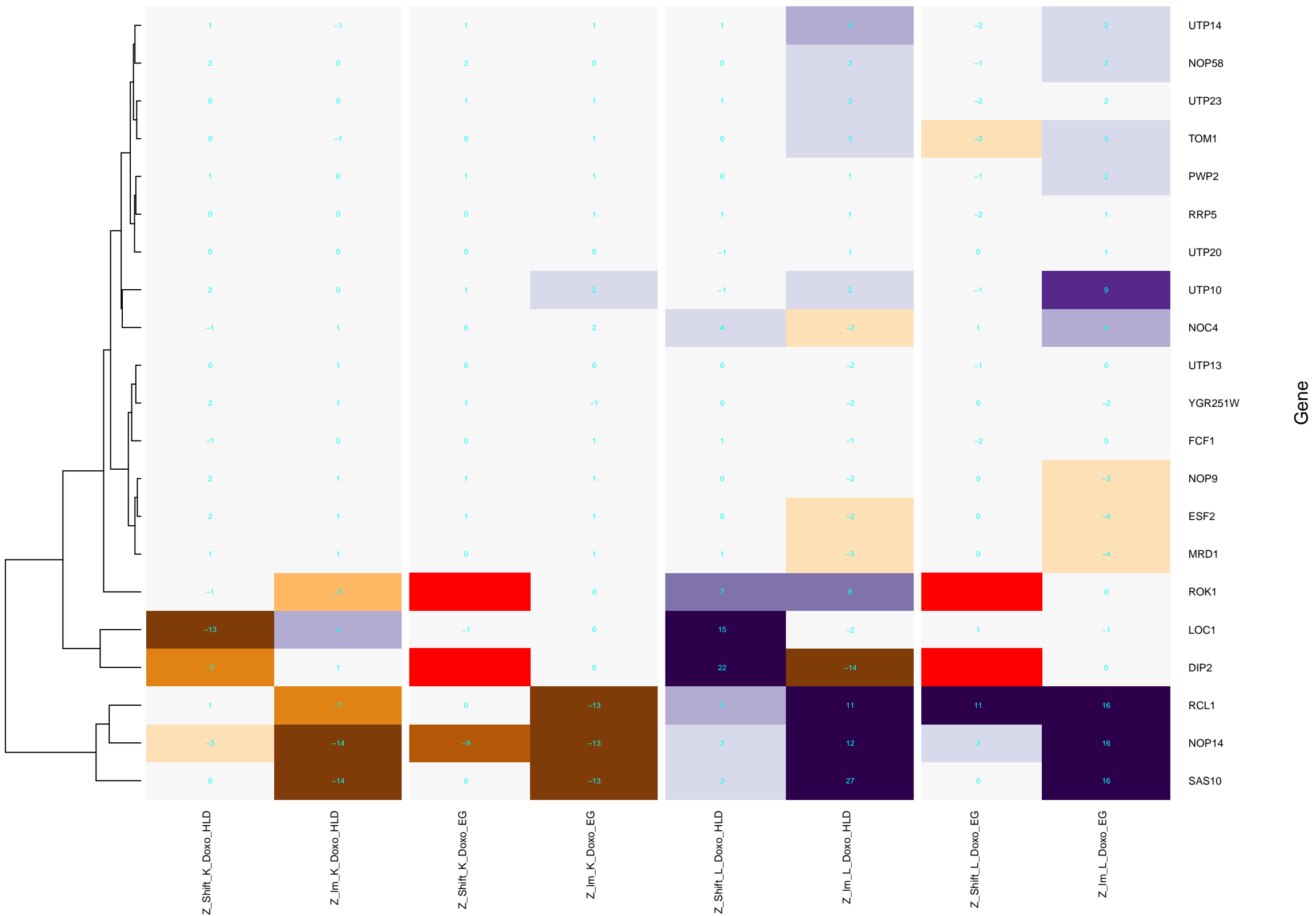

Supplement: Supplementary file 9 — Additional file 9. GO term-specific heatmaps for REMc/GTF-enriched clusters. GO term-specific heatmaps for significant GO process terms were generated as described in methods and Figs. 3 and 4. Any related child terms are presented in subsequent pages of the parent file name. GO terms with more than 100 children, with 2 or fewer genes annotated to the term, or a file size over 300KB are not shown. All heatmaps are generated with the same layout (see Figs. 3 and 4). [file 40170_2019_201_MOESM9_ESM.bz2 › Additional_File9_GOTermHeatmaps/Additional_File9_GOTermHeatmap/endonucleolytic_cleavage_in_5'-ETS_of_tricistronic_rRNA_transcript_(SSU-rRNA,_5.8S_rRNA,_LSU-rRNA).pdf]

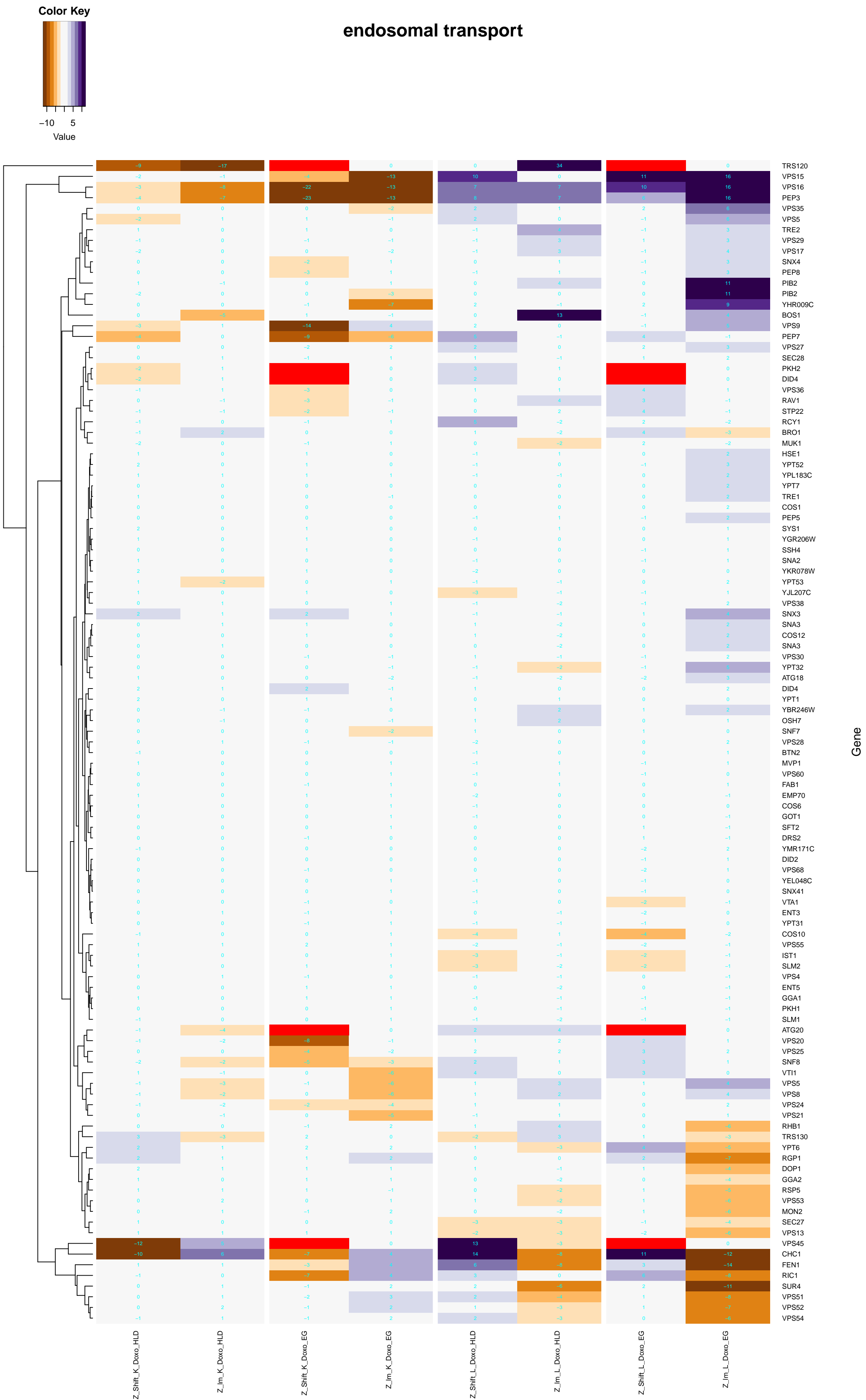

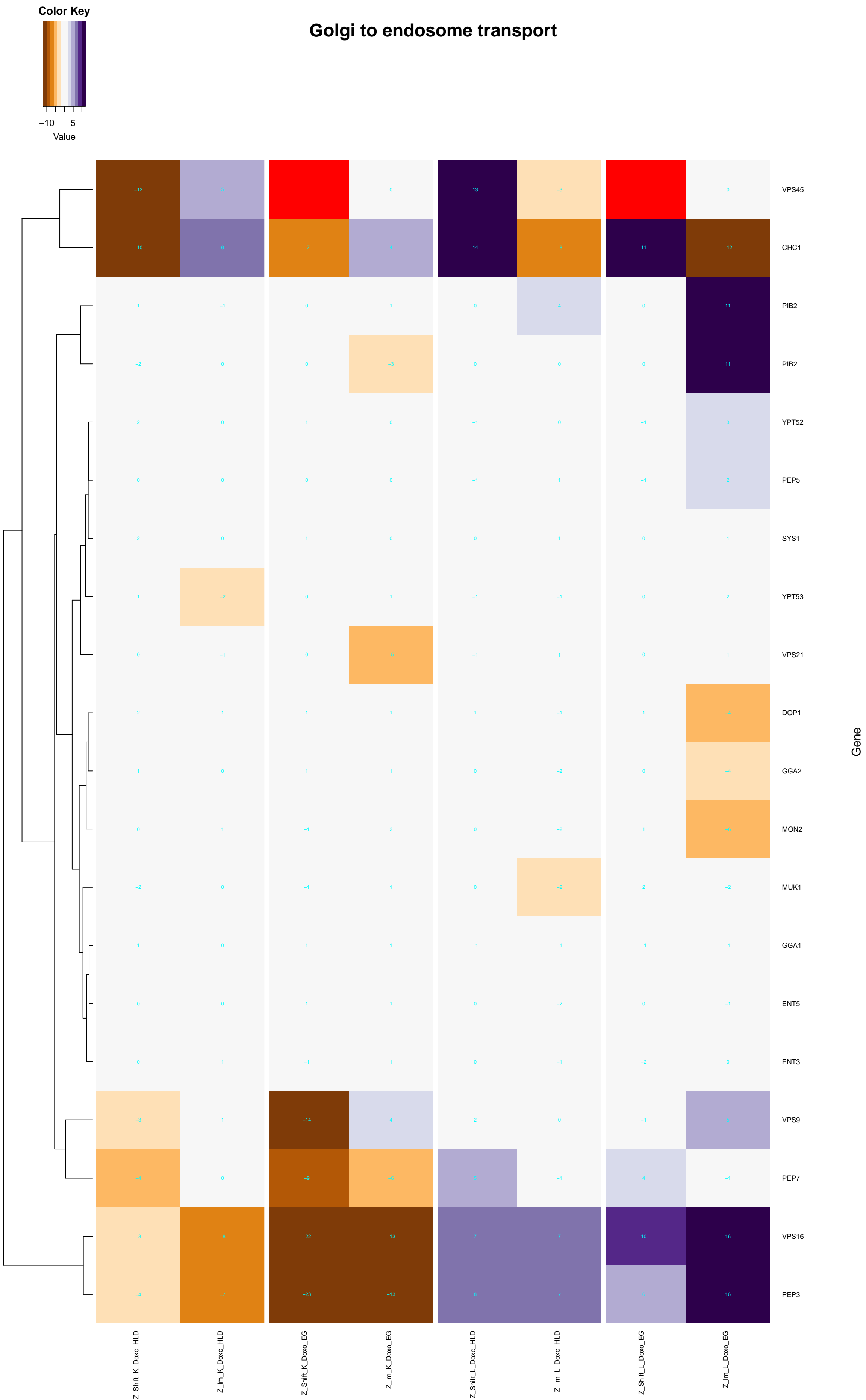

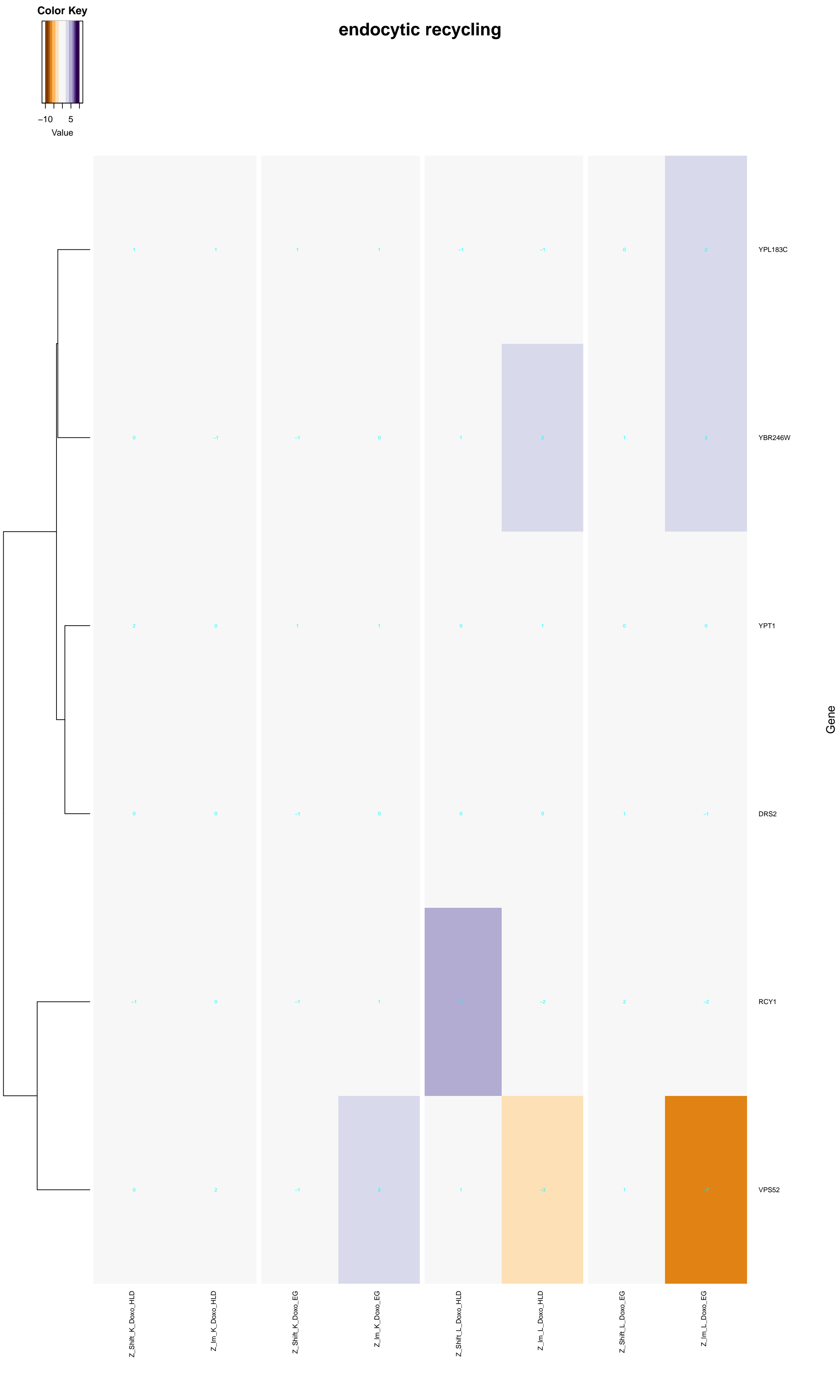

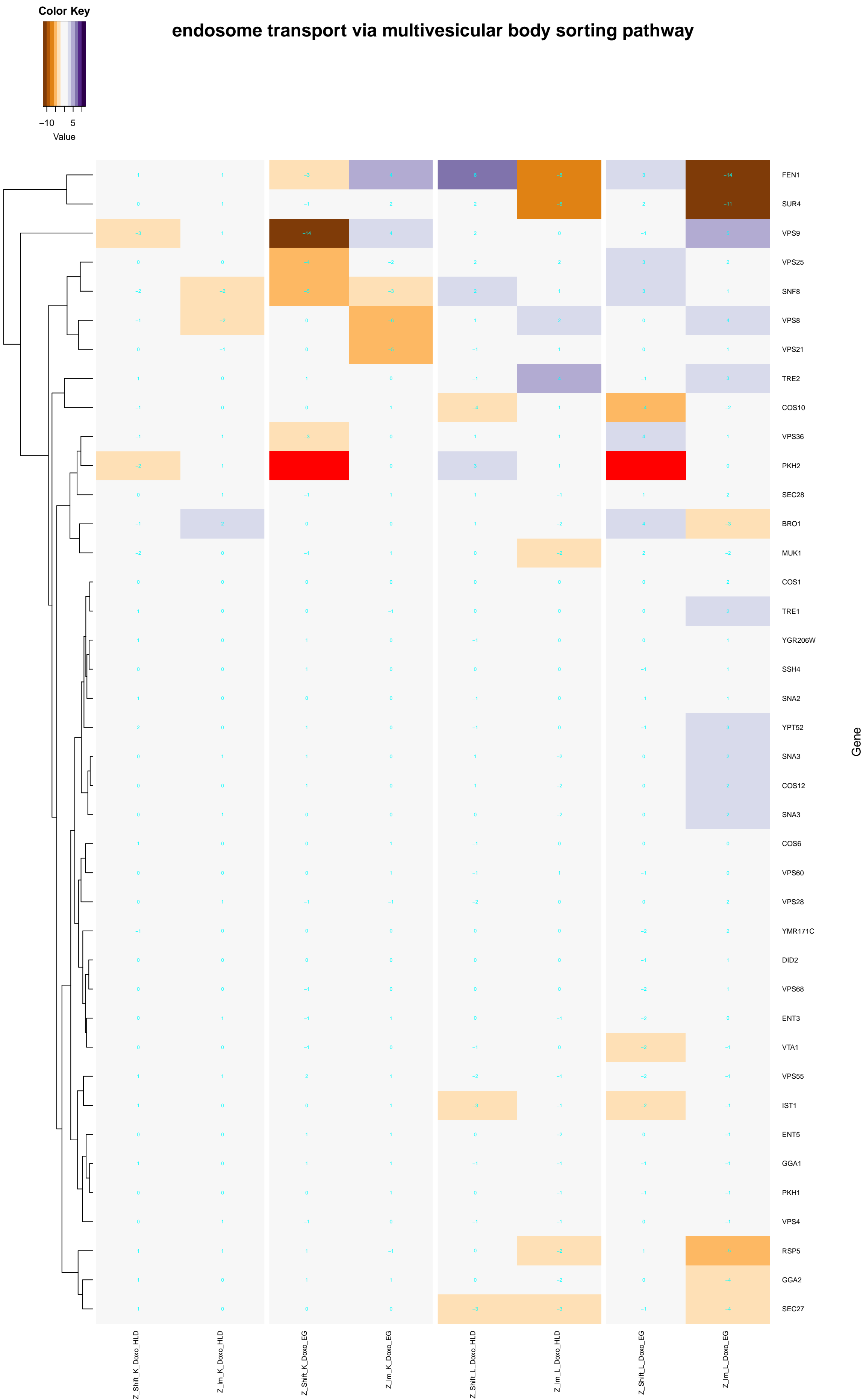

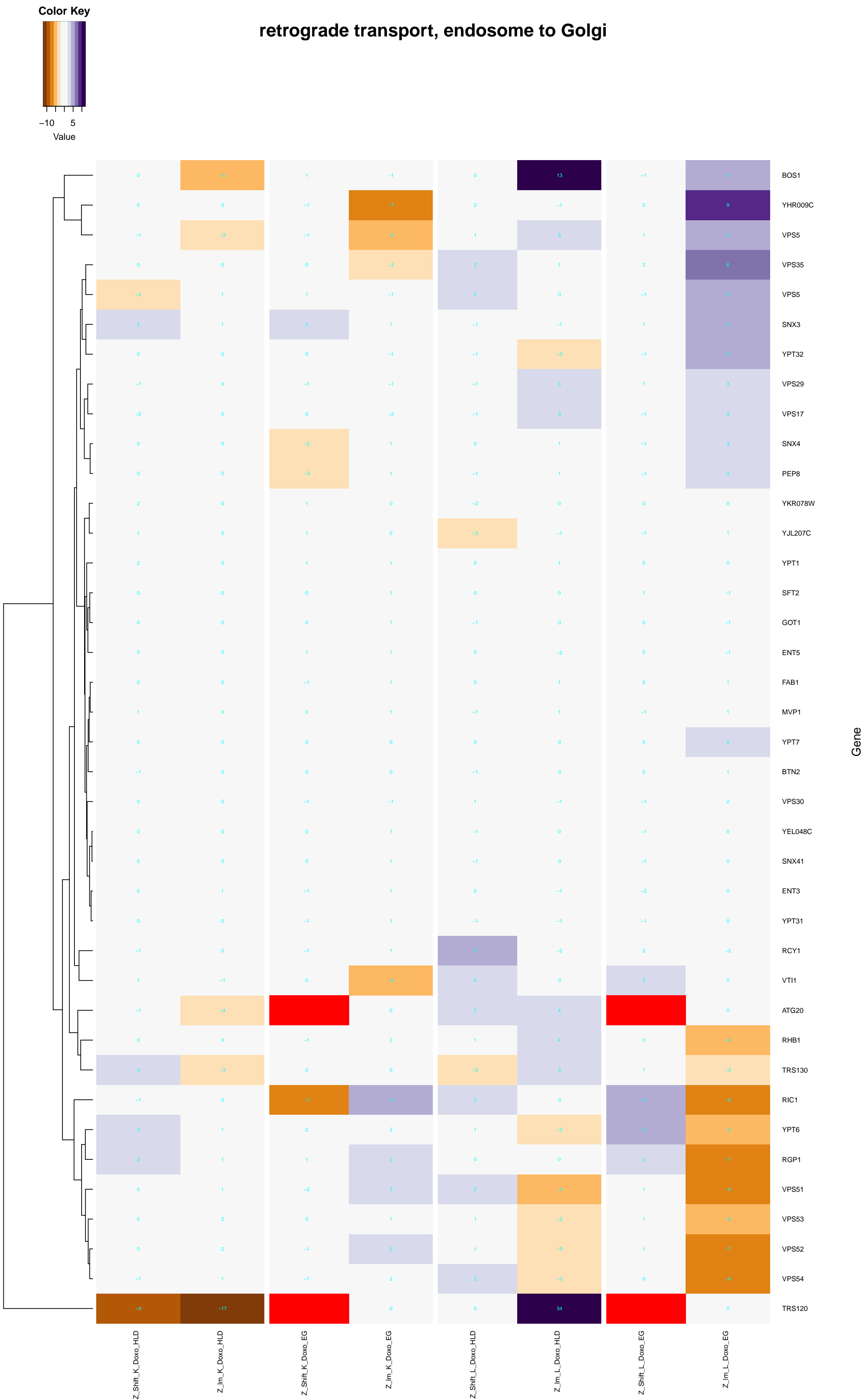

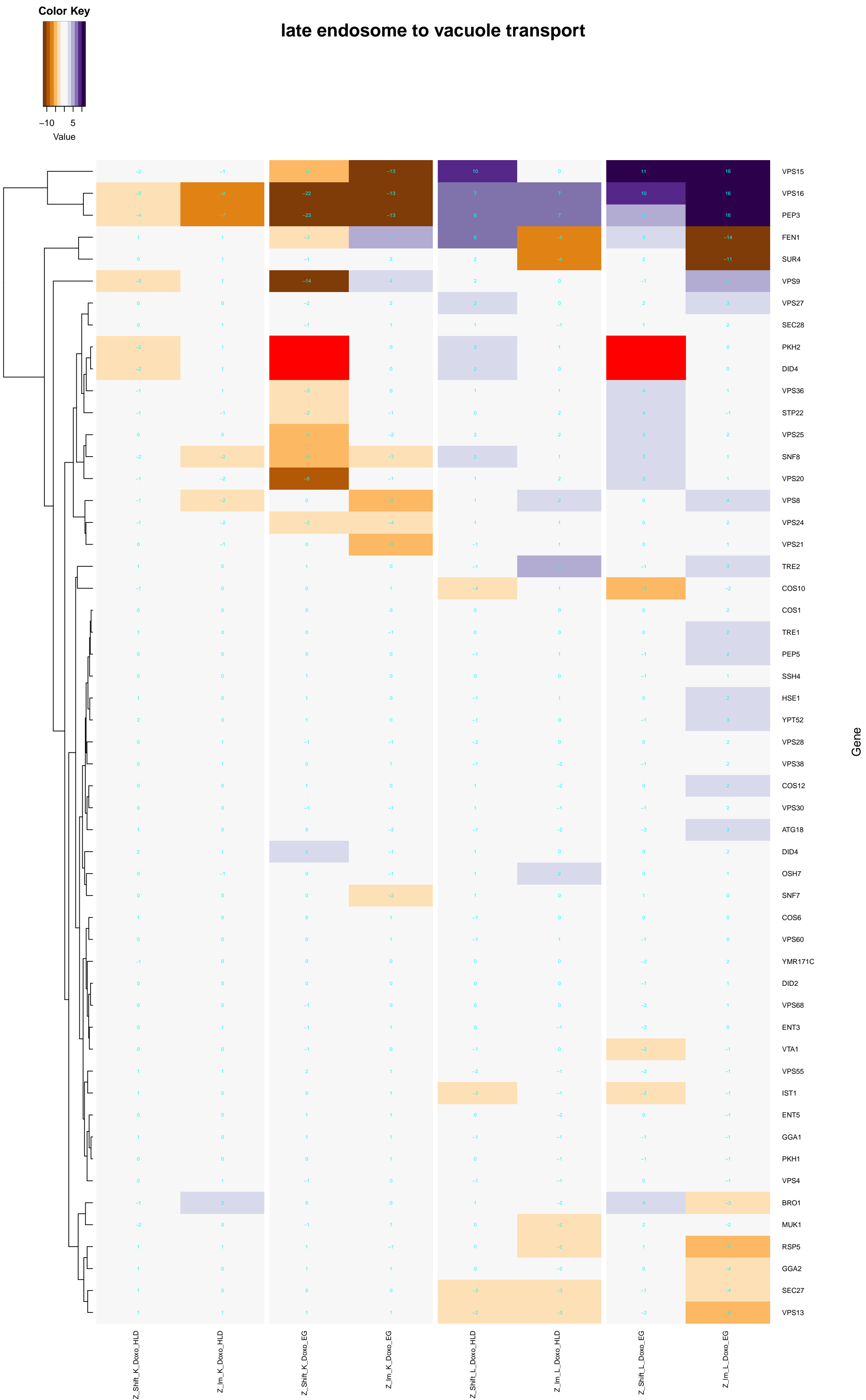

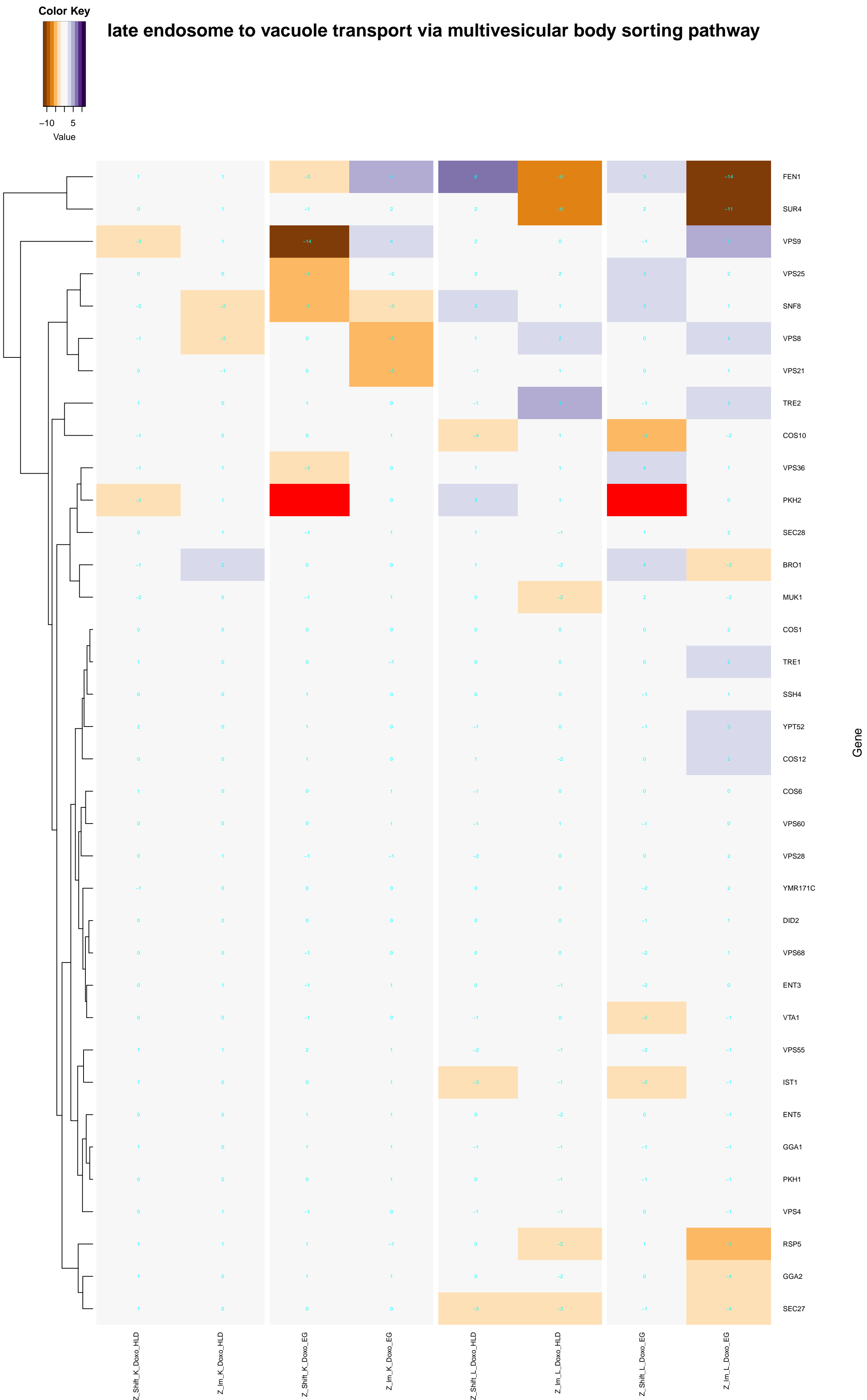

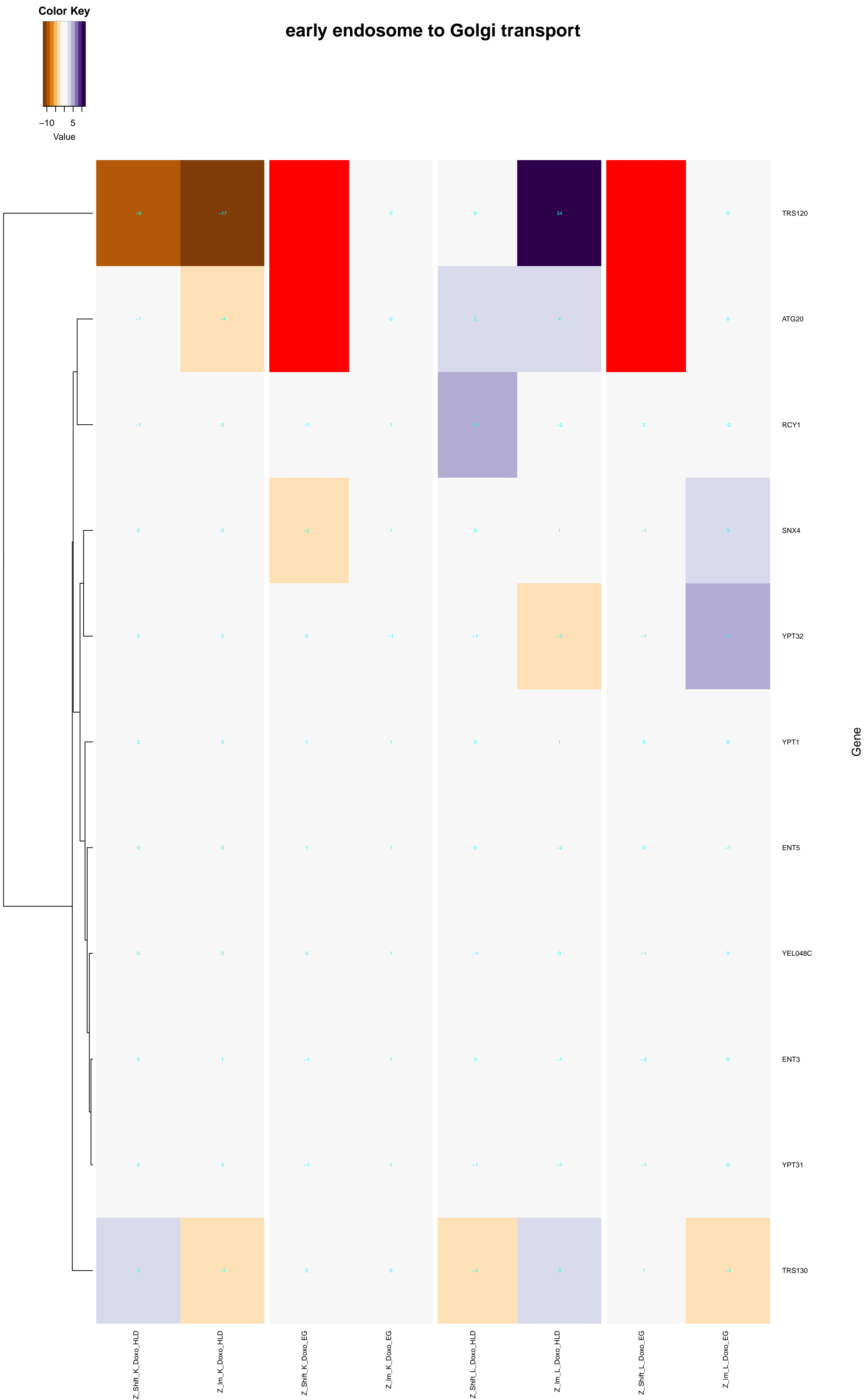

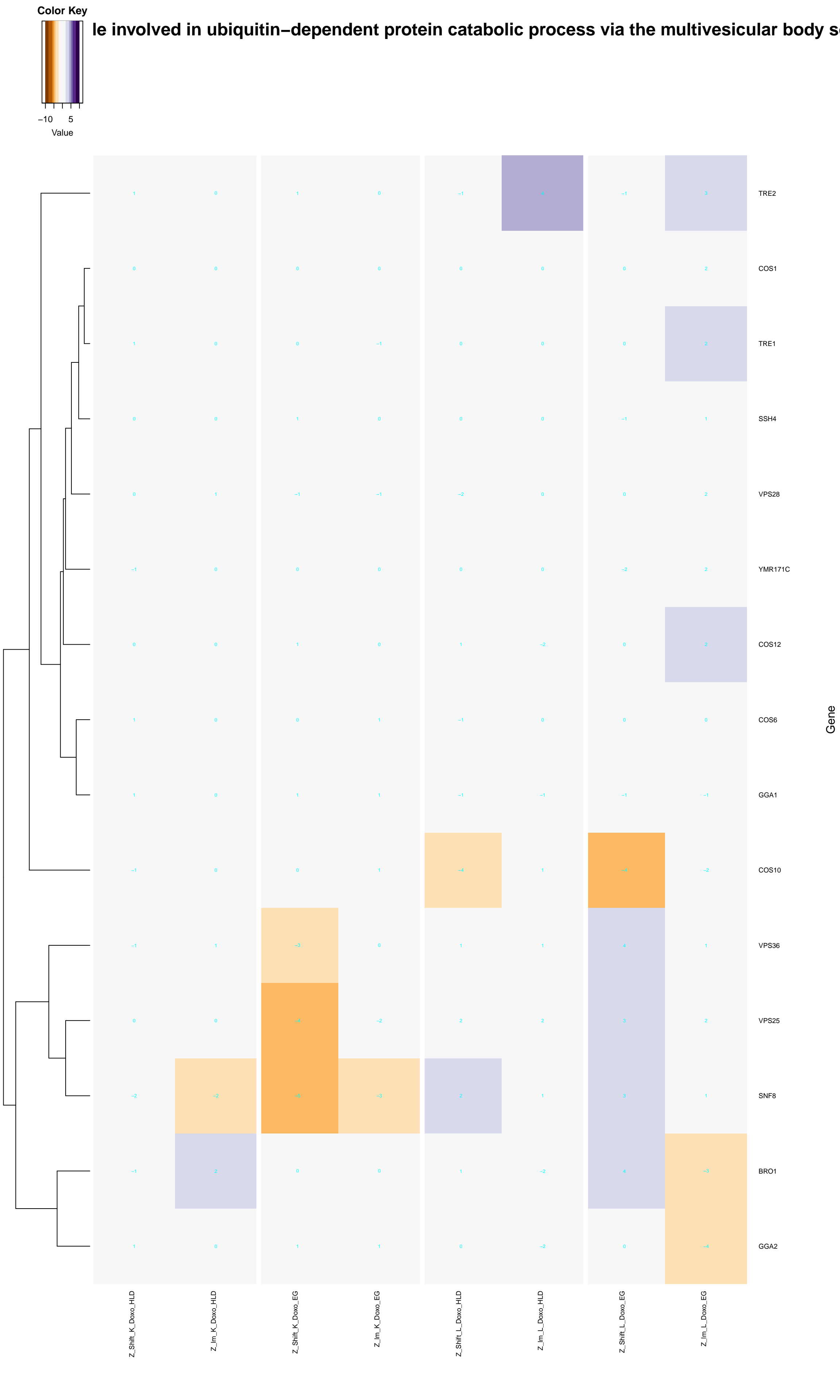

Supplement: Supplementary file 9 — Additional file 9. GO term-specific heatmaps for REMc/GTF-enriched clusters. GO term-specific heatmaps for significant GO process terms were generated as described in methods and Figs. 3 and 4. Any related child terms are presented in subsequent pages of the parent file name. GO terms with more than 100 children, with 2 or fewer genes annotated to the term, or a file size over 300KB are not shown. All heatmaps are generated with the same layout (see Figs. 3 and 4). [file 40170_2019_201_MOESM9_ESM.bz2 › Additional_File9_GOTermHeatmaps/Additional_File9_GOTermHeatmap/endosomal_transport.pdf]

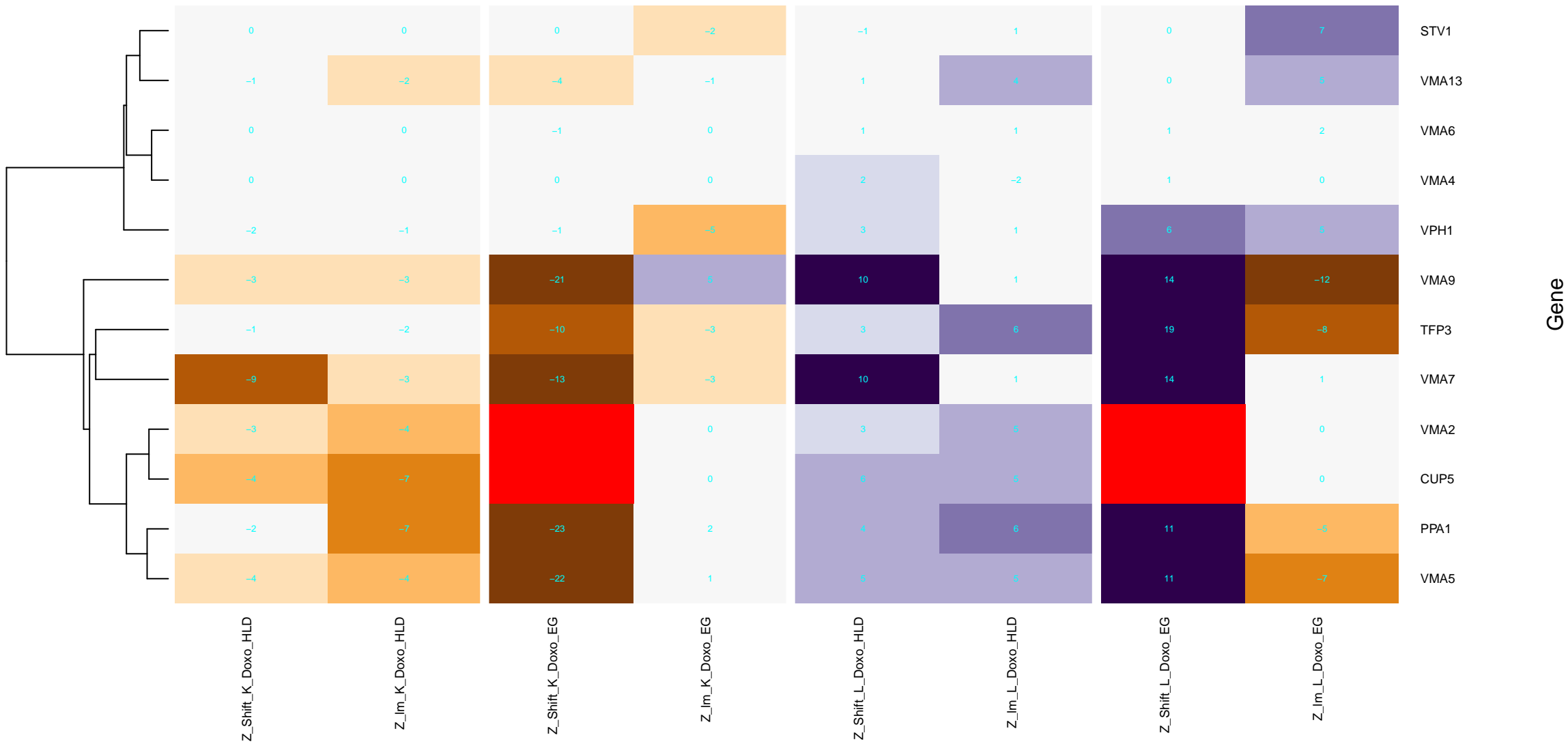

Supplement: Supplementary file 9 — Additional file 9. GO term-specific heatmaps for REMc/GTF-enriched clusters. GO term-specific heatmaps for significant GO process terms were generated as described in methods and Figs. 3 and 4. Any related child terms are presented in subsequent pages of the parent file name. GO terms with more than 100 children, with 2 or fewer genes annotated to the term, or a file size over 300KB are not shown. All heatmaps are generated with the same layout (see Figs. 3 and 4). [file 40170_2019_201_MOESM9_ESM.bz2 › Additional_File9_GOTermHeatmaps/Additional_File9_GOTermHeatmap/energy_coupled_proton_transmembrane_transport,_against_electrochemical_gradient.pdf]

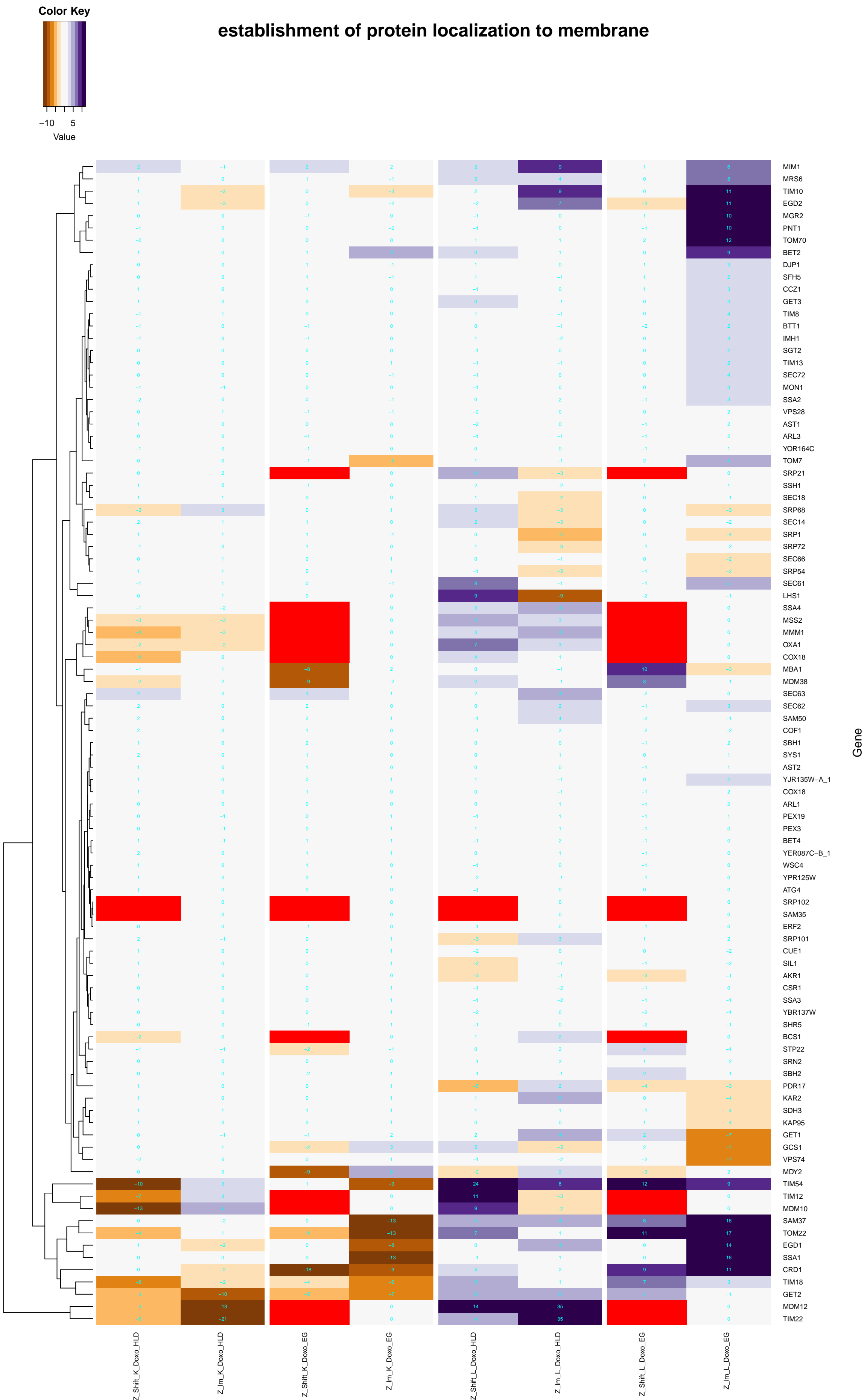

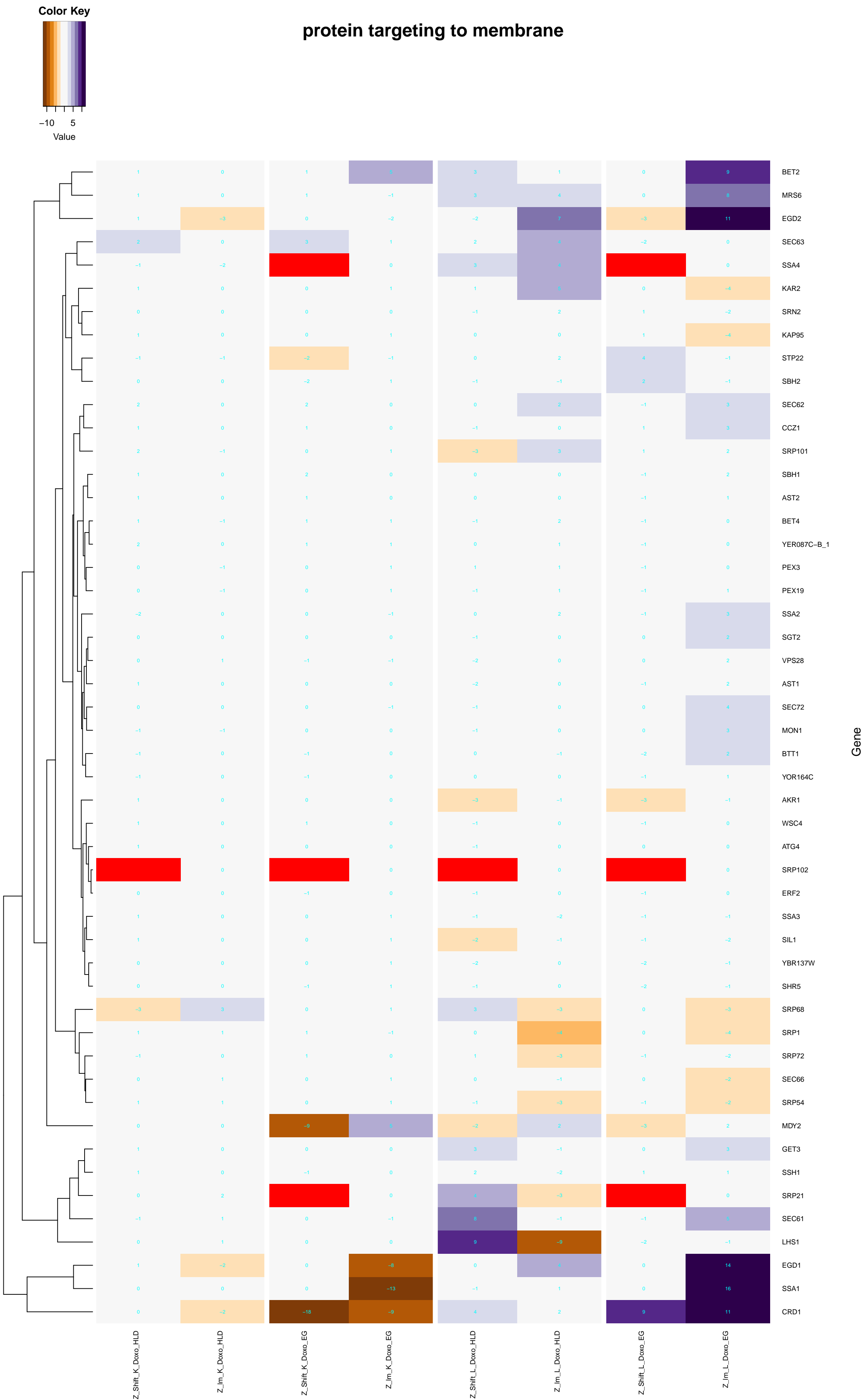

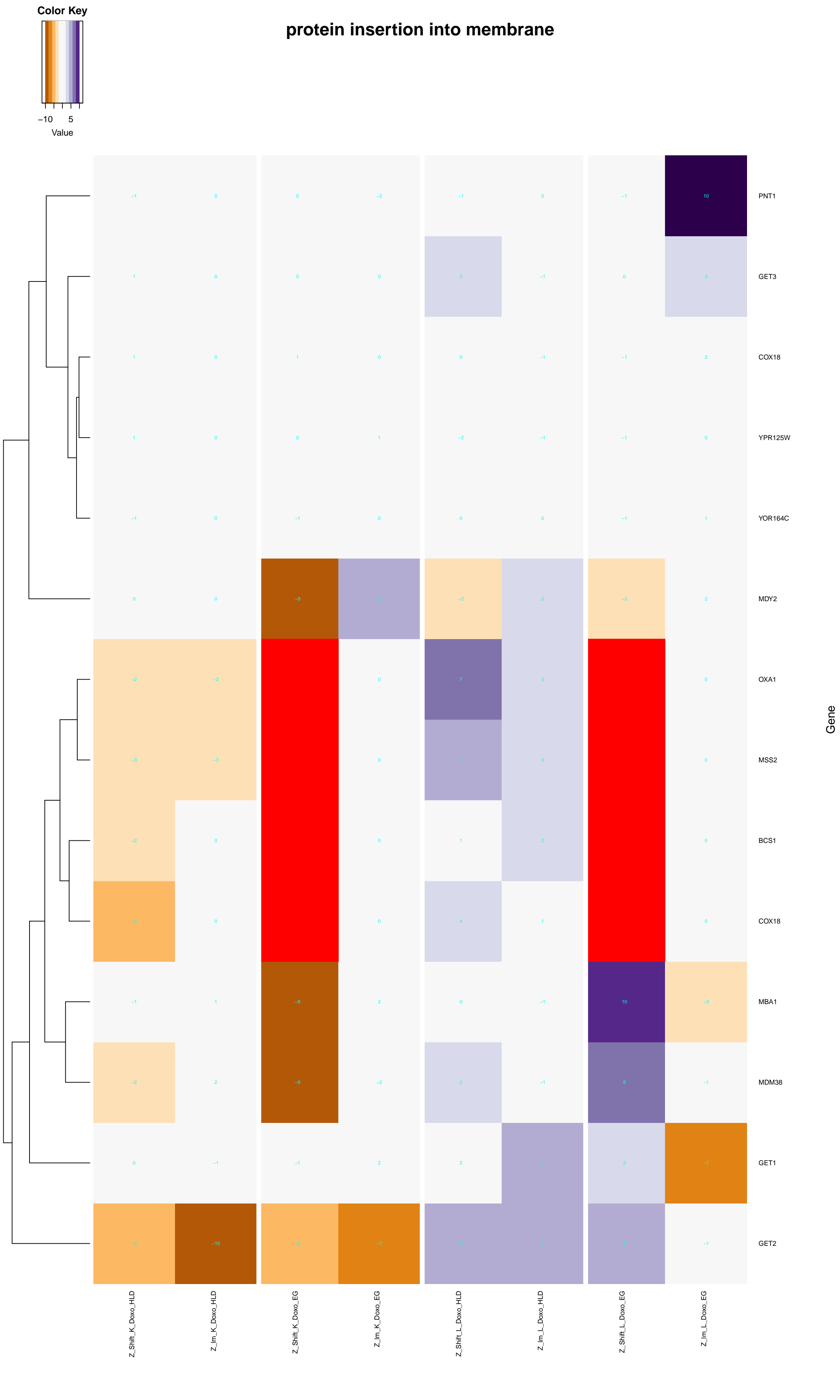

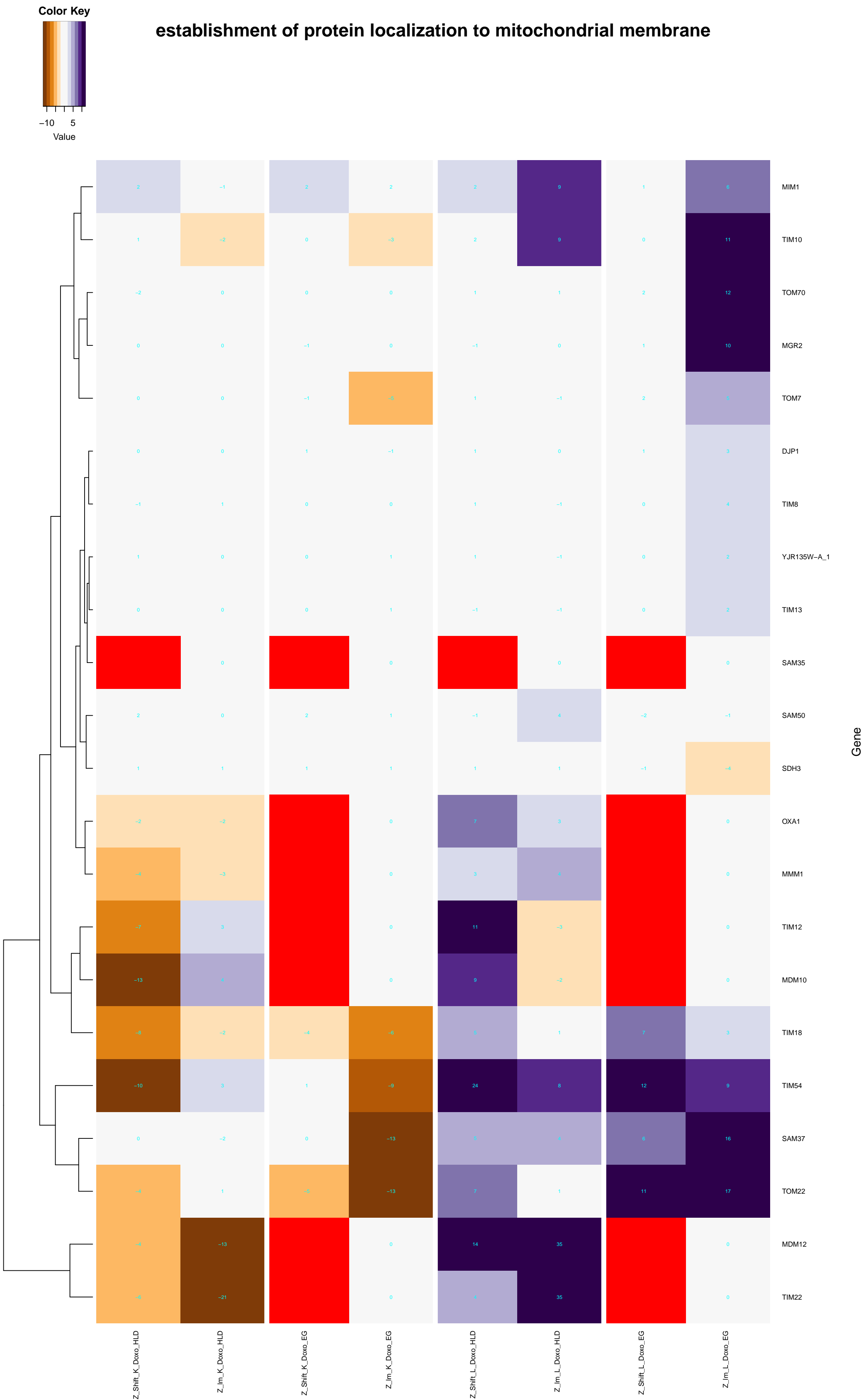

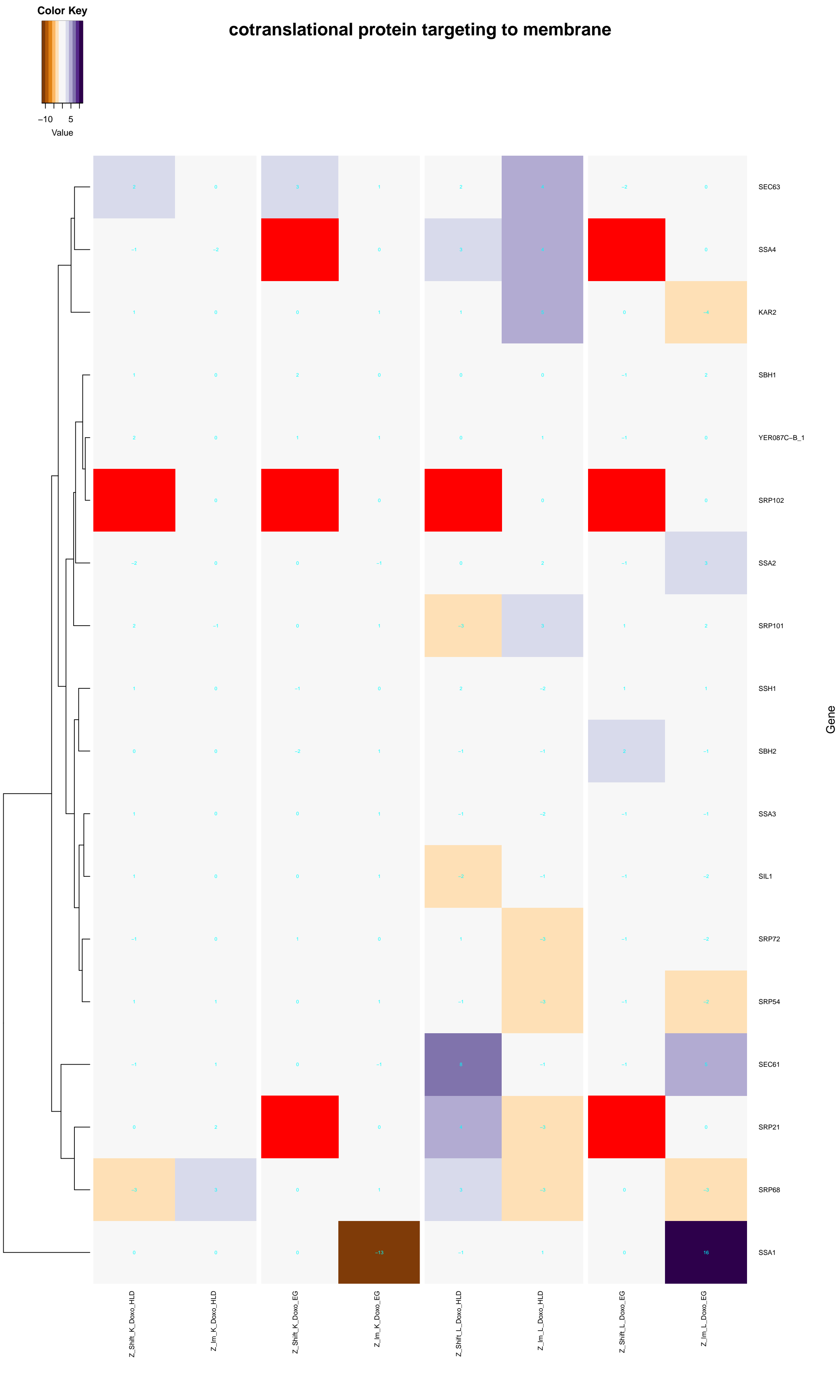

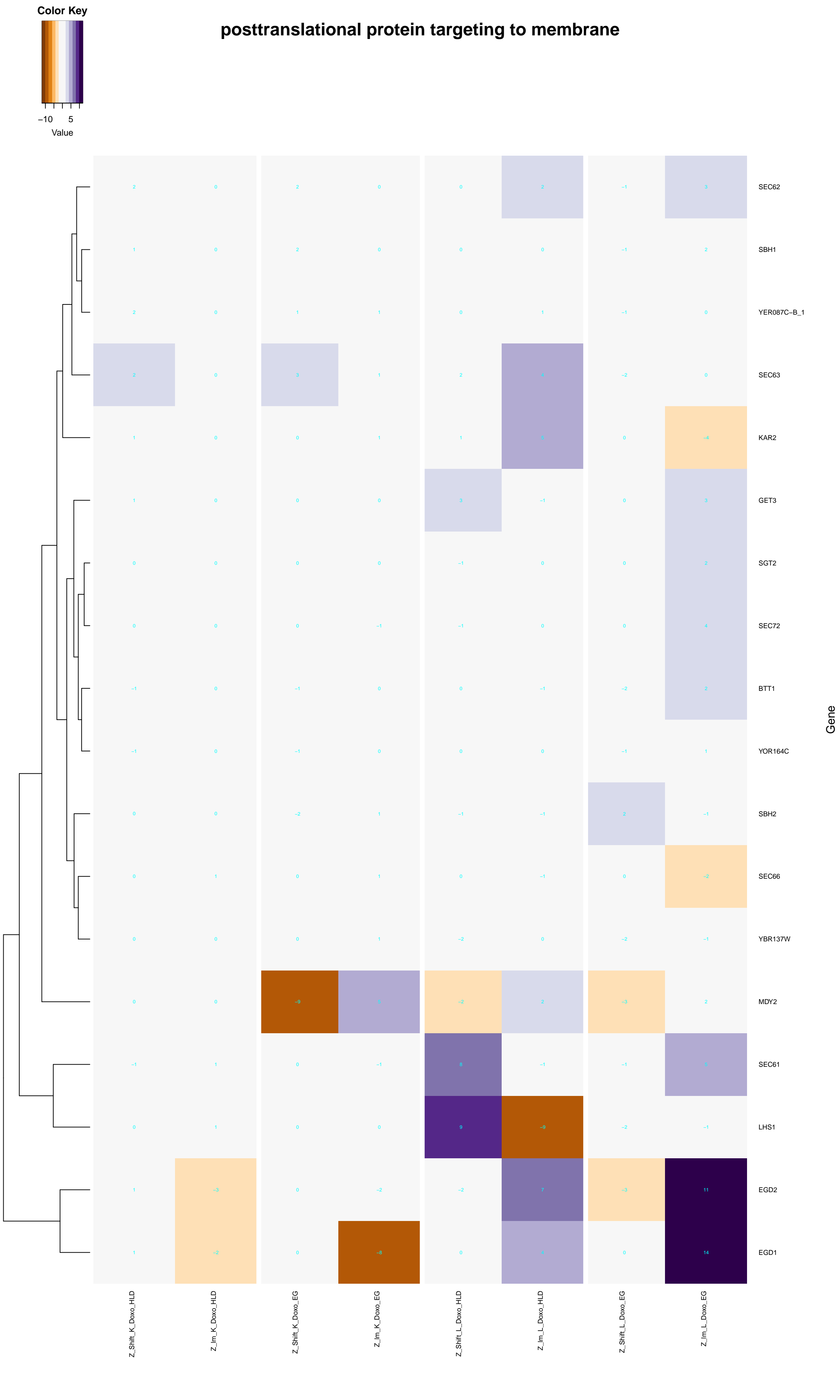

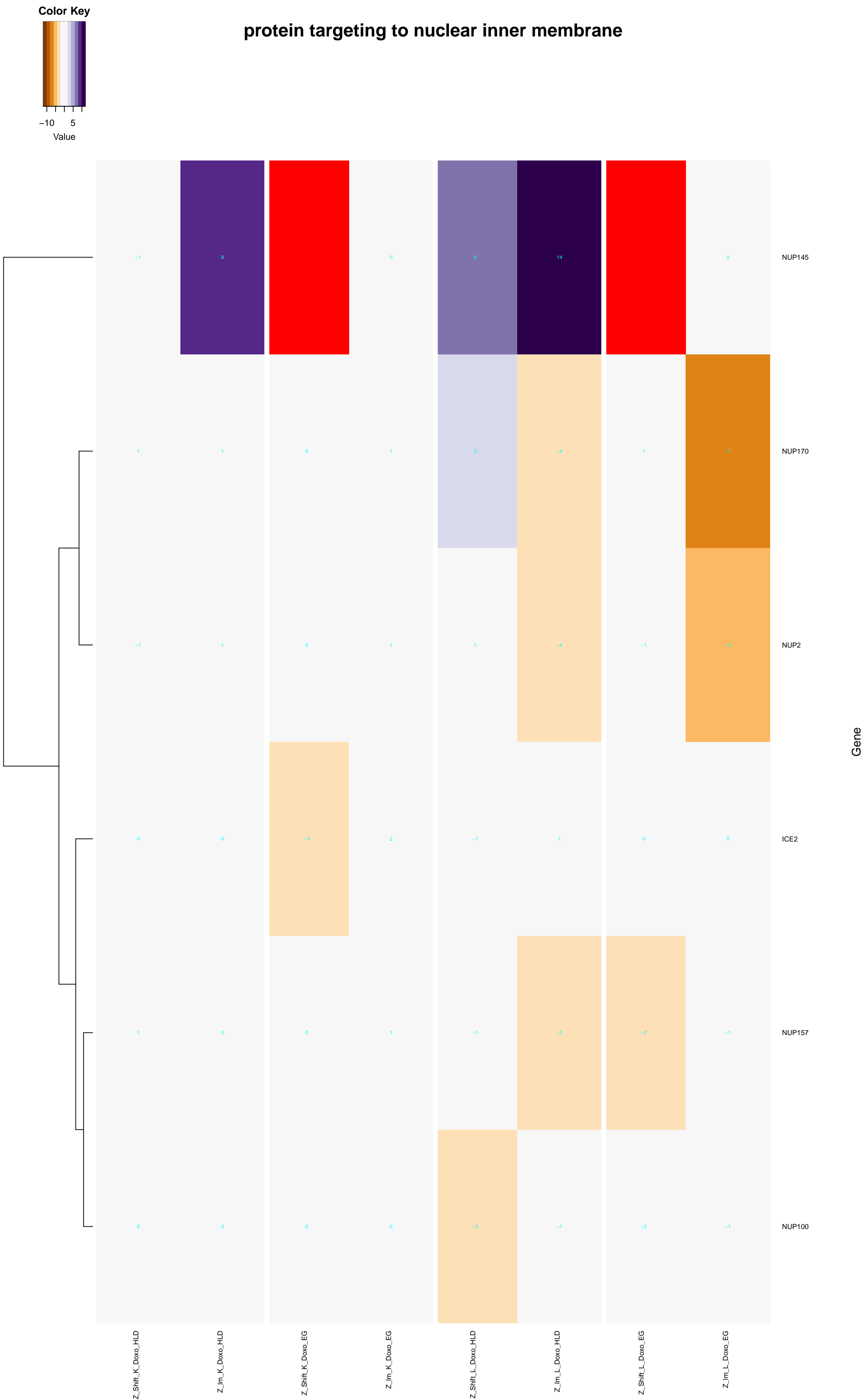

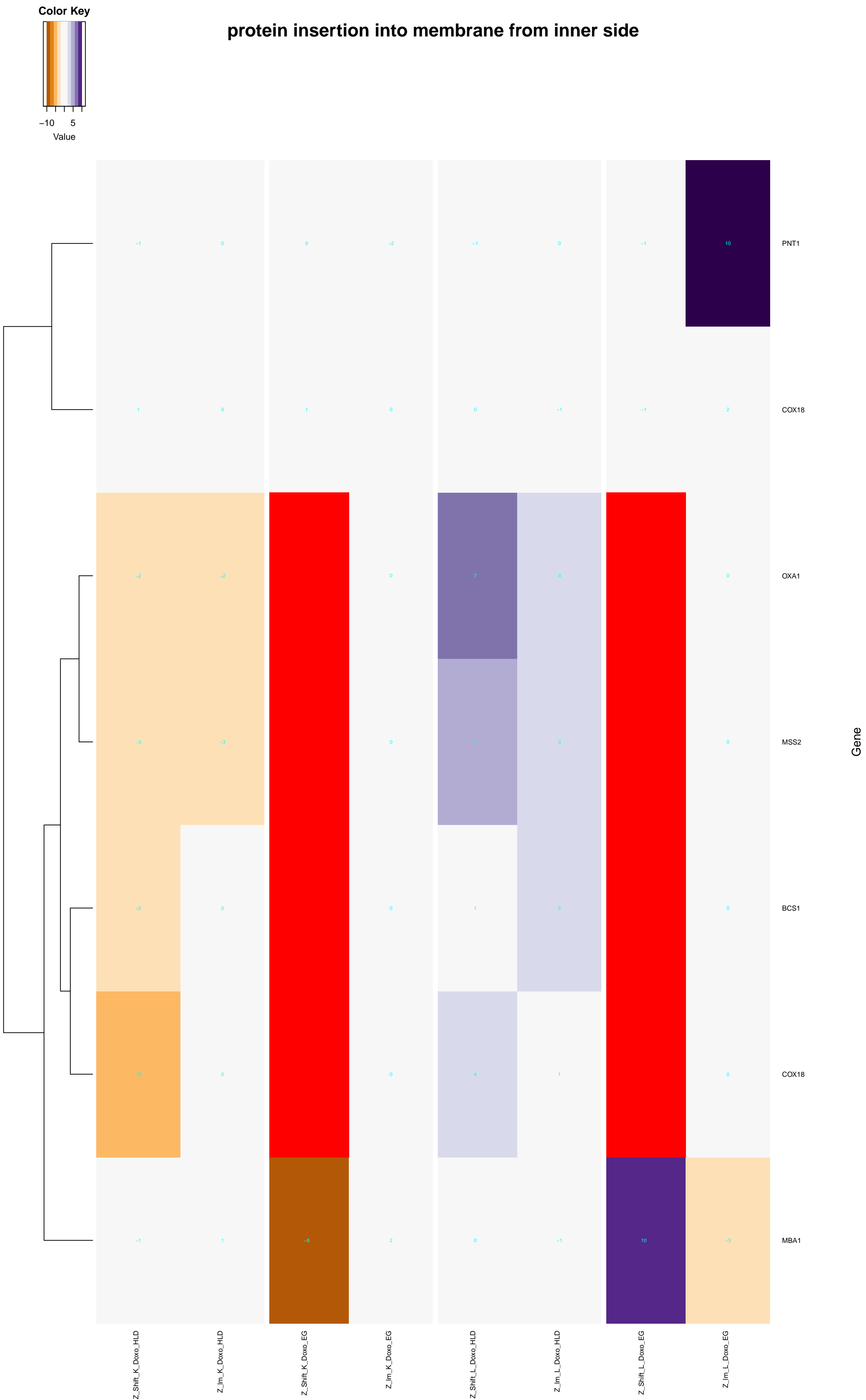

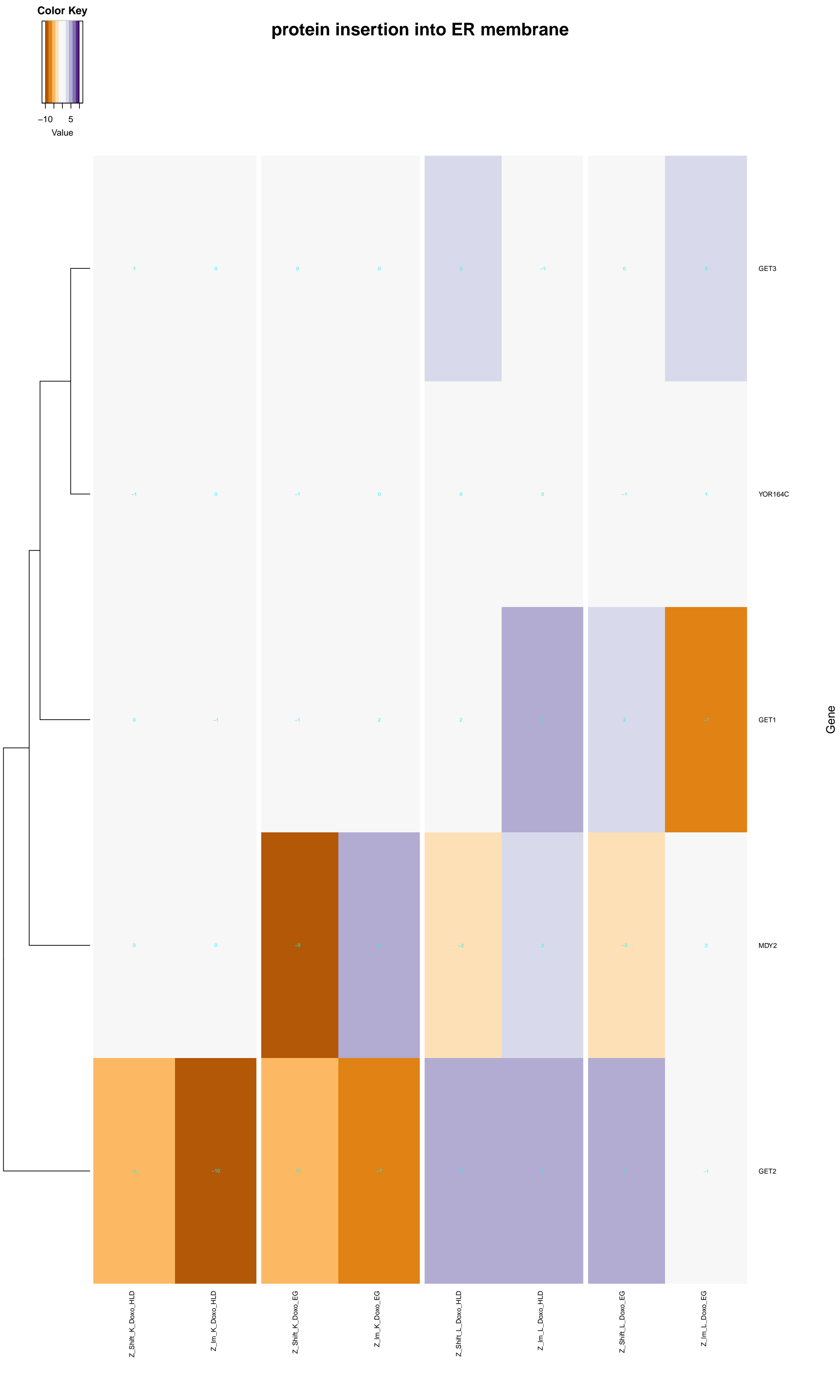

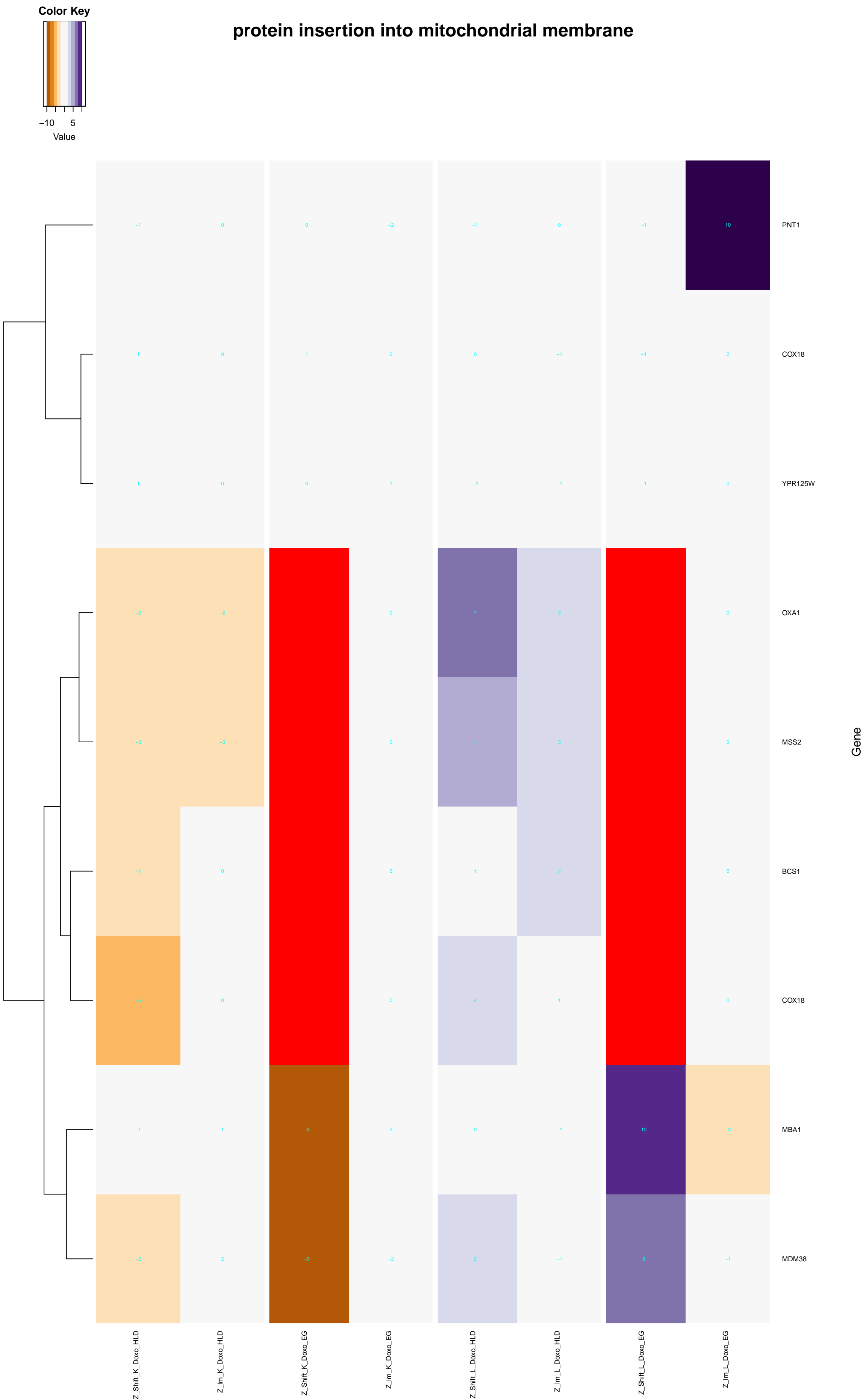

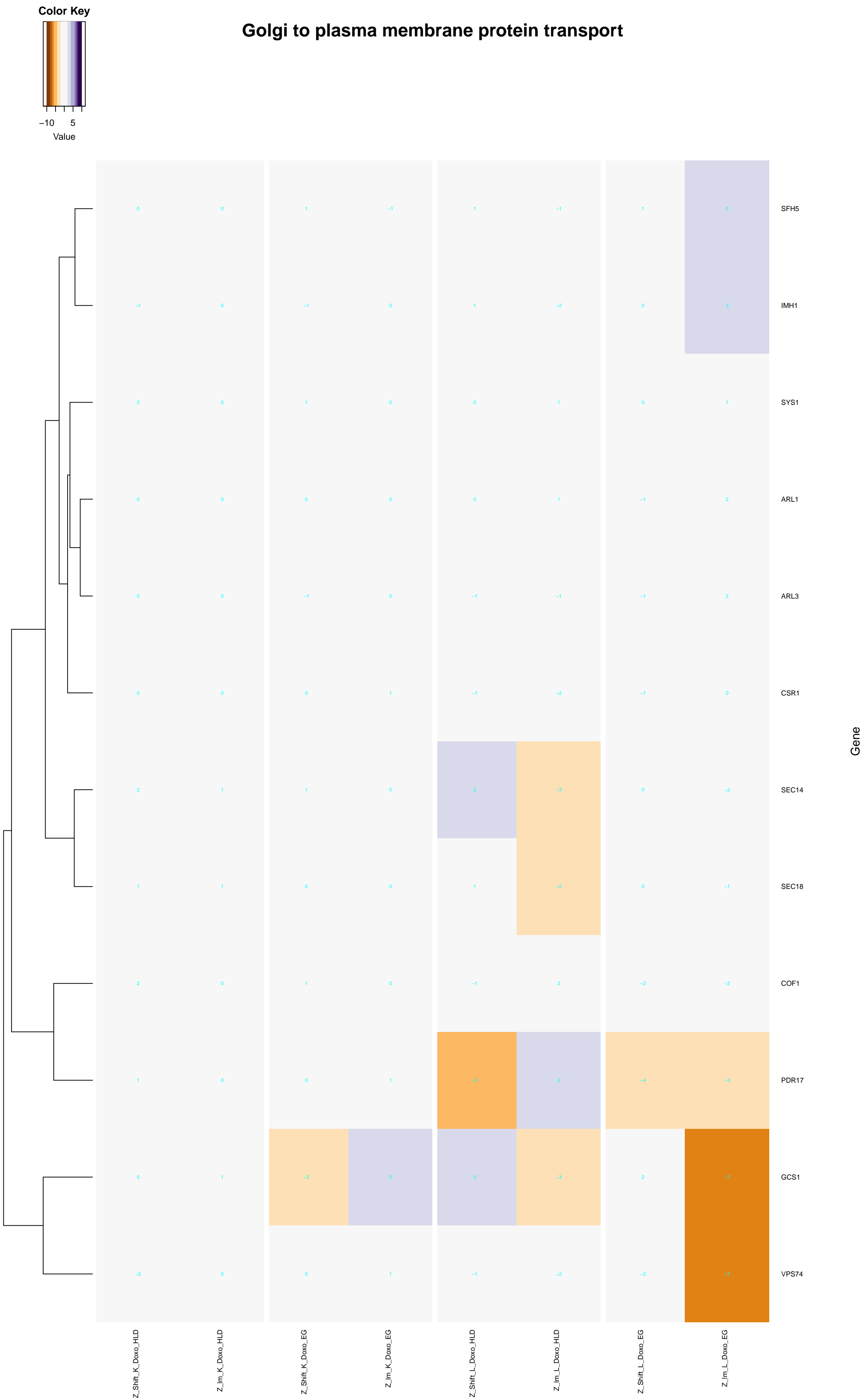

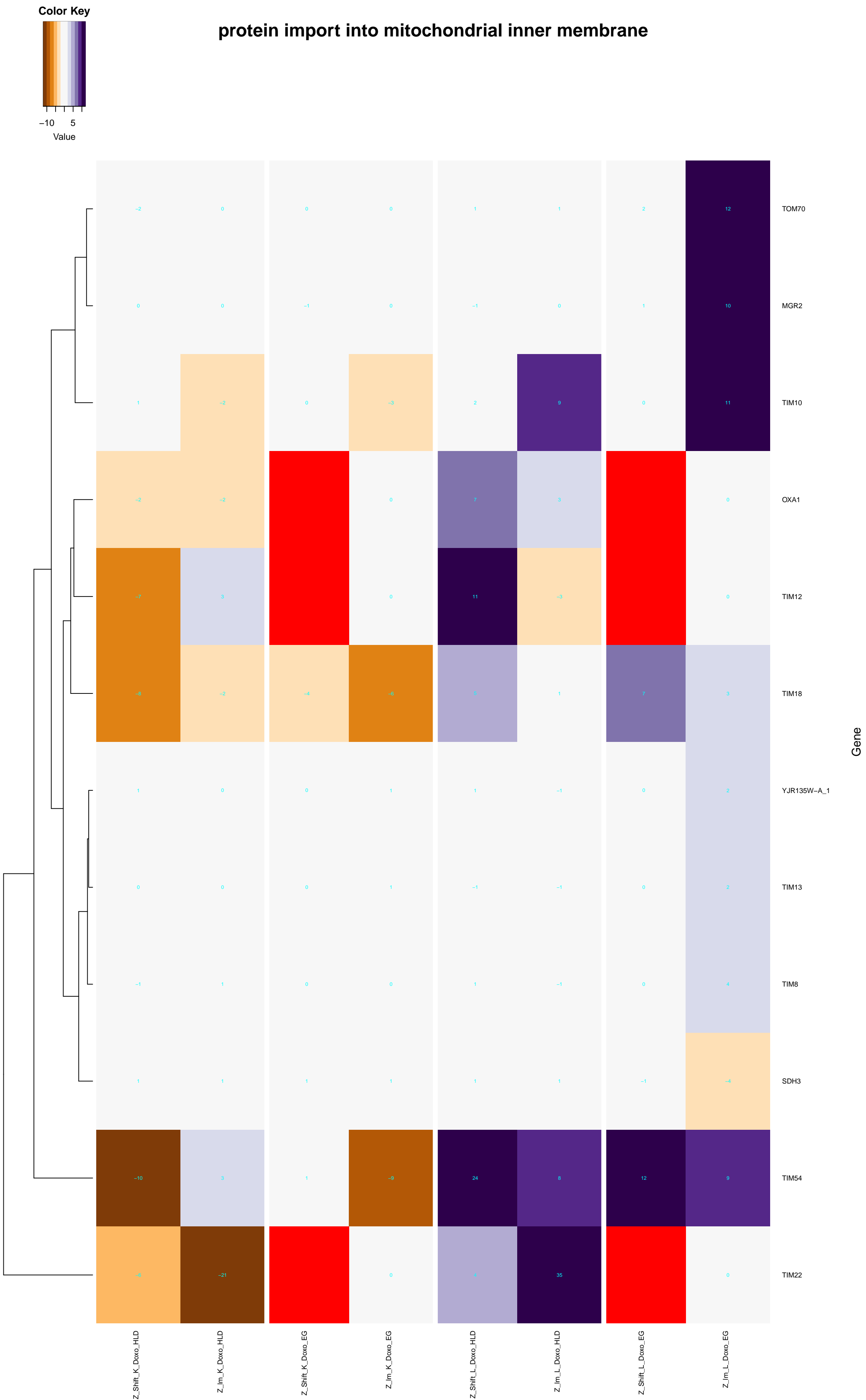

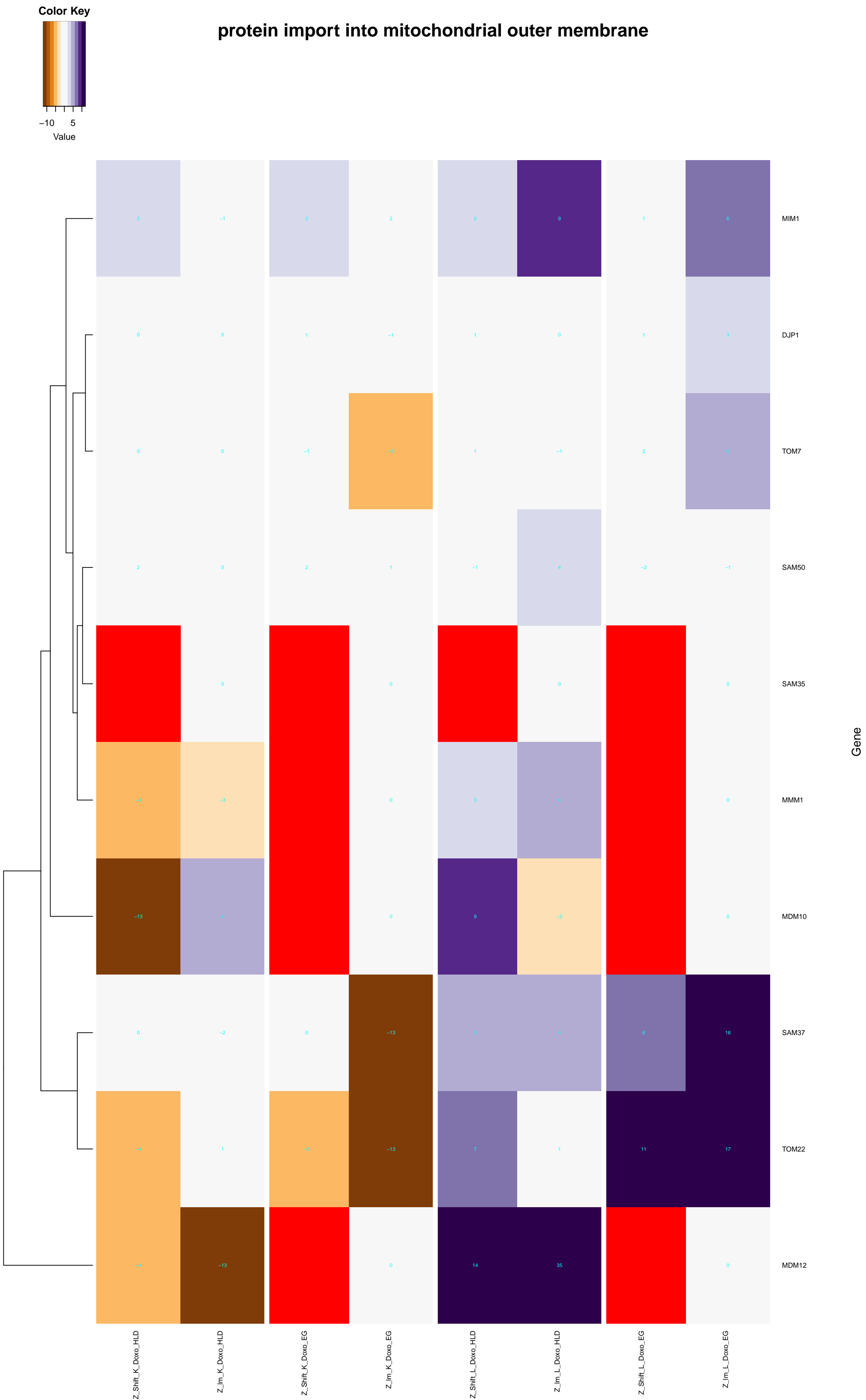

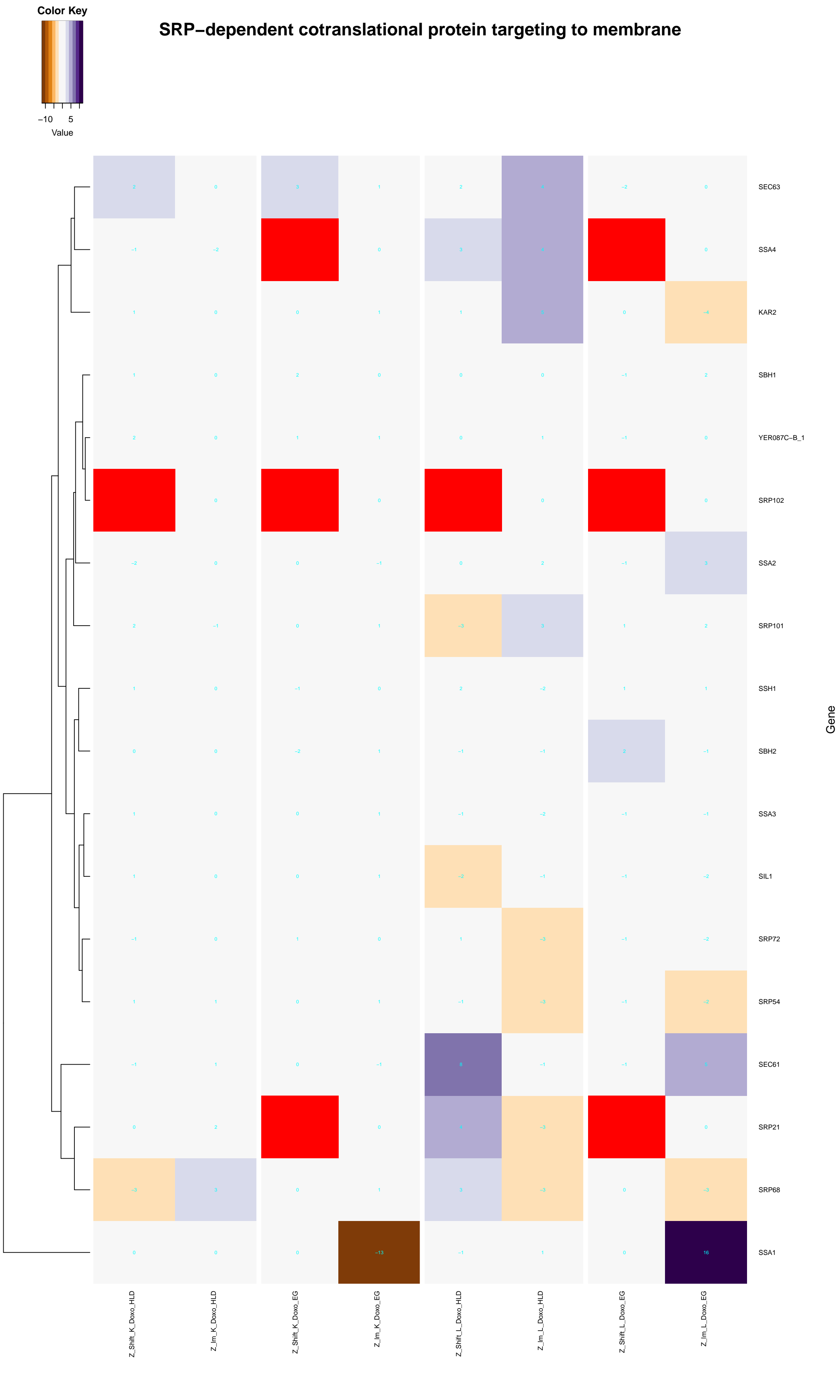

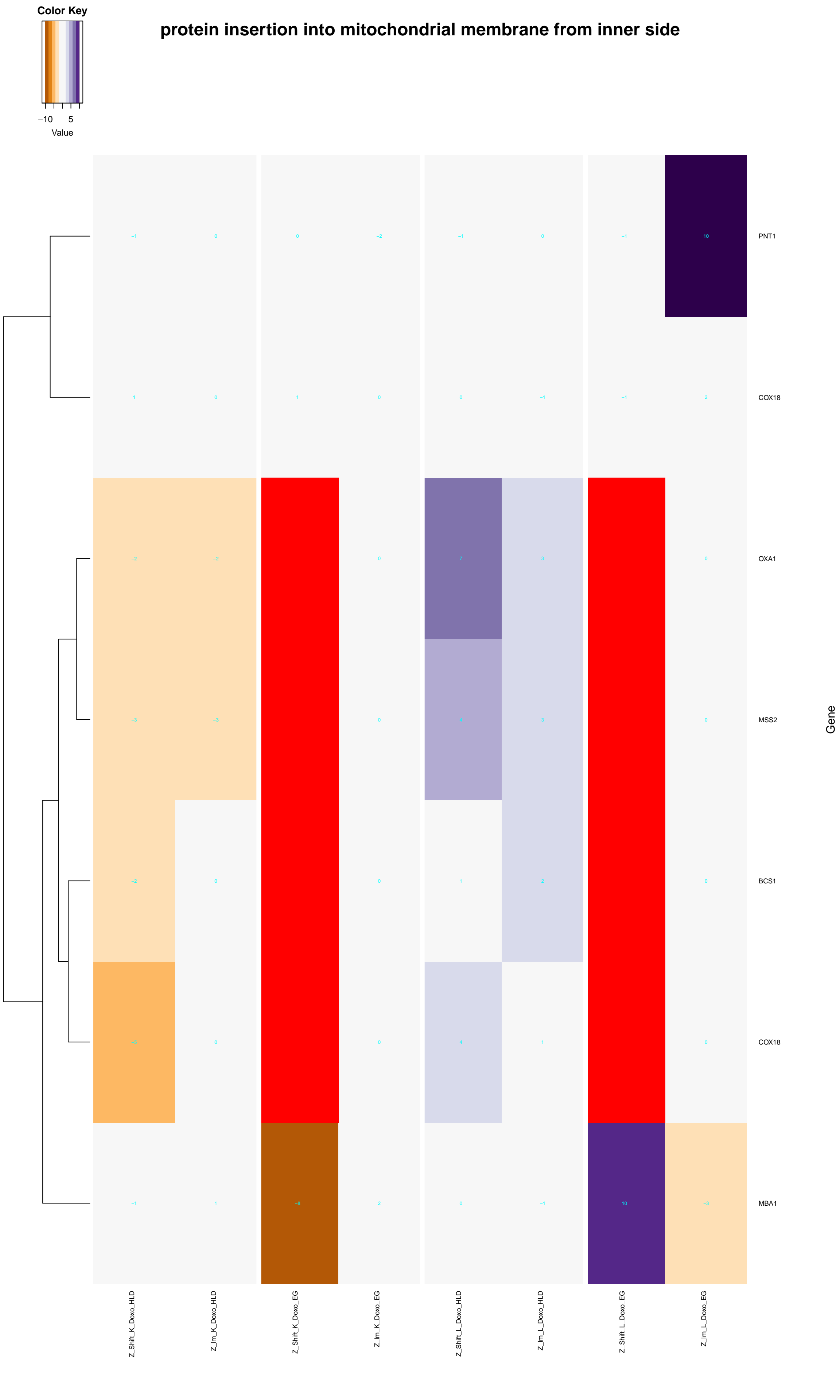

Supplement: Supplementary file 9 — Additional file 9. GO term-specific heatmaps for REMc/GTF-enriched clusters. GO term-specific heatmaps for significant GO process terms were generated as described in methods and Figs. 3 and 4. Any related child terms are presented in subsequent pages of the parent file name. GO terms with more than 100 children, with 2 or fewer genes annotated to the term, or a file size over 300KB are not shown. All heatmaps are generated with the same layout (see Figs. 3 and 4). [file 40170_2019_201_MOESM9_ESM.bz2 › Additional_File9_GOTermHeatmaps/Additional_File9_GOTermHeatmap/establishment_of_protein_localization_to_membrane.pdf]

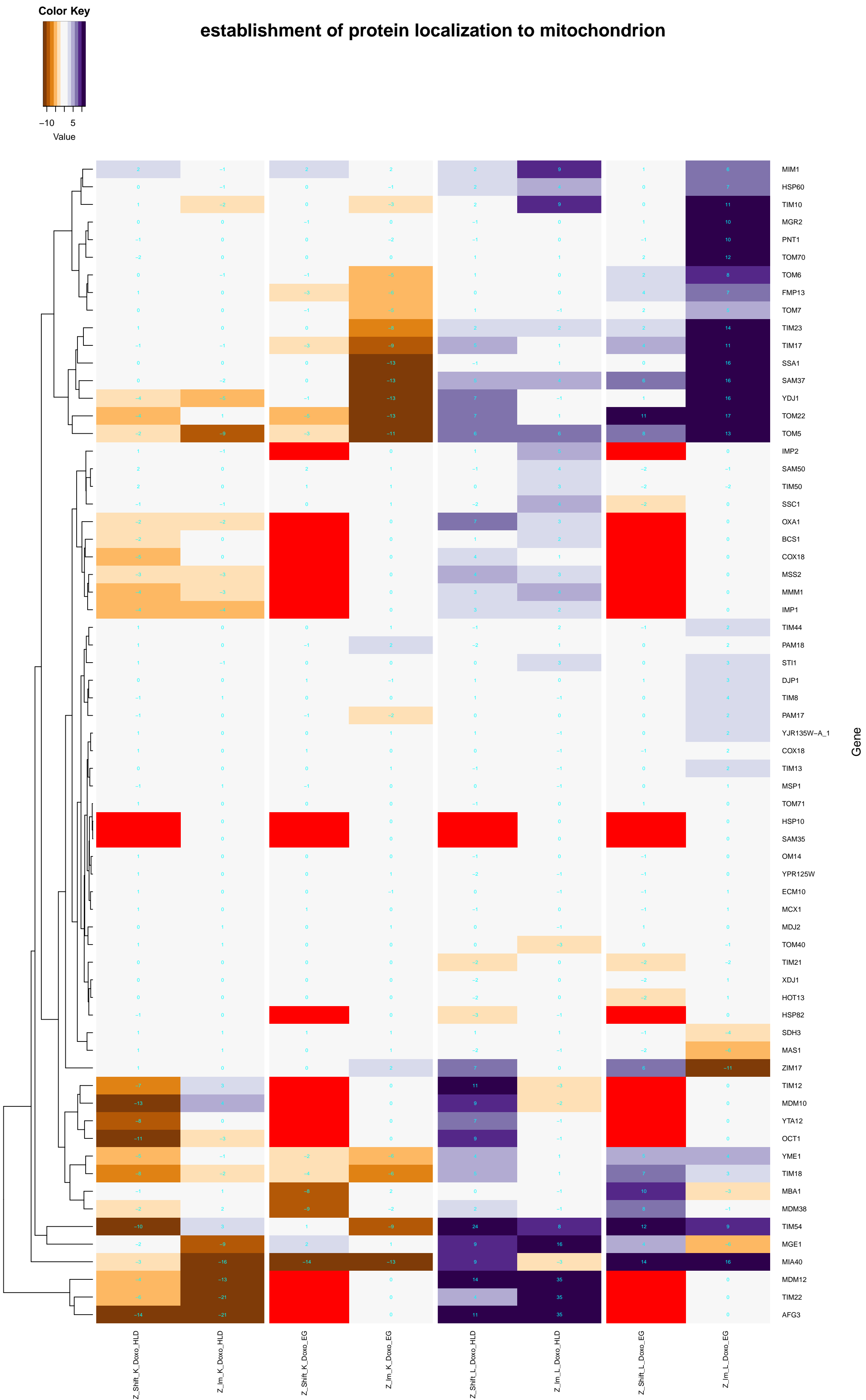

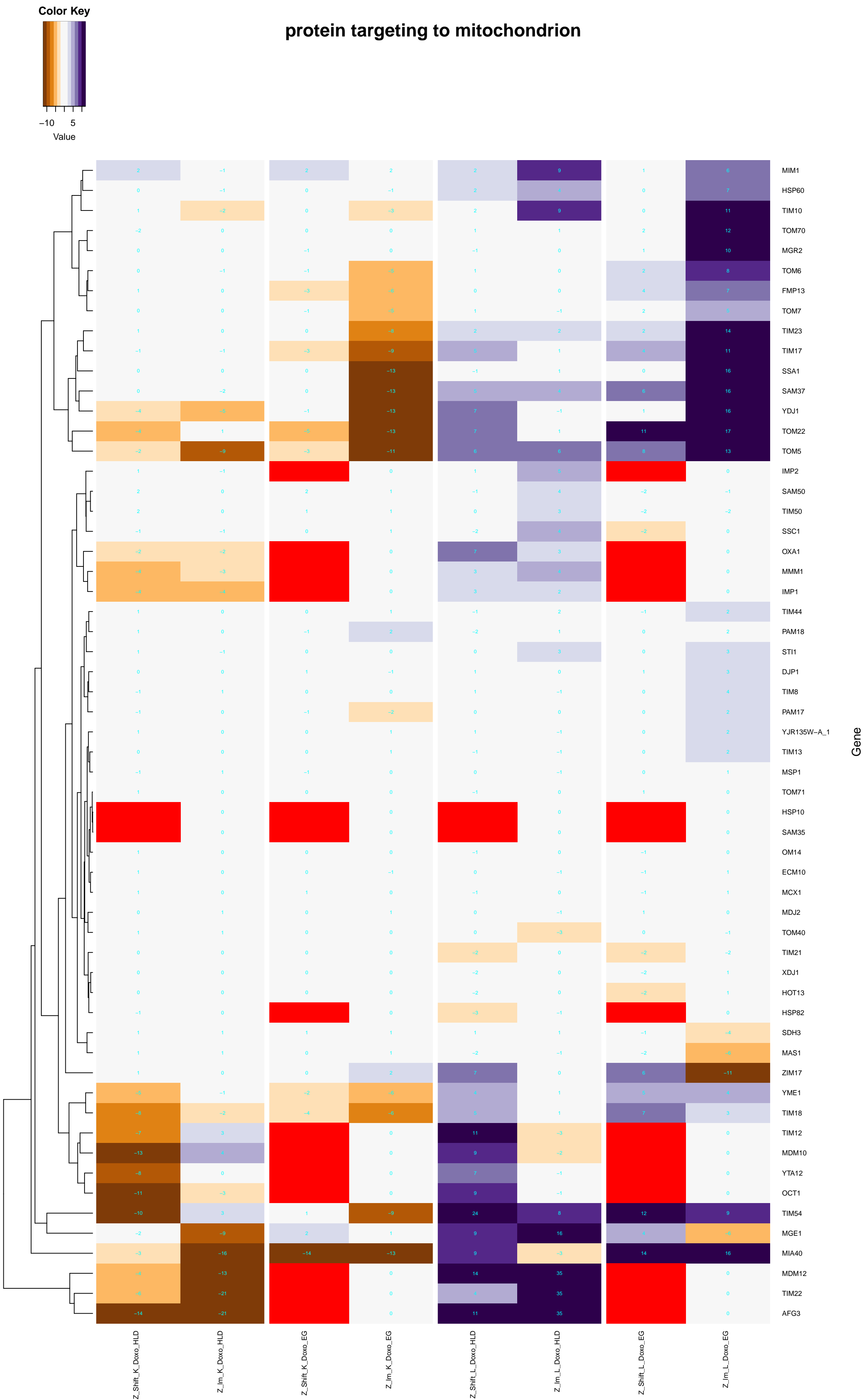

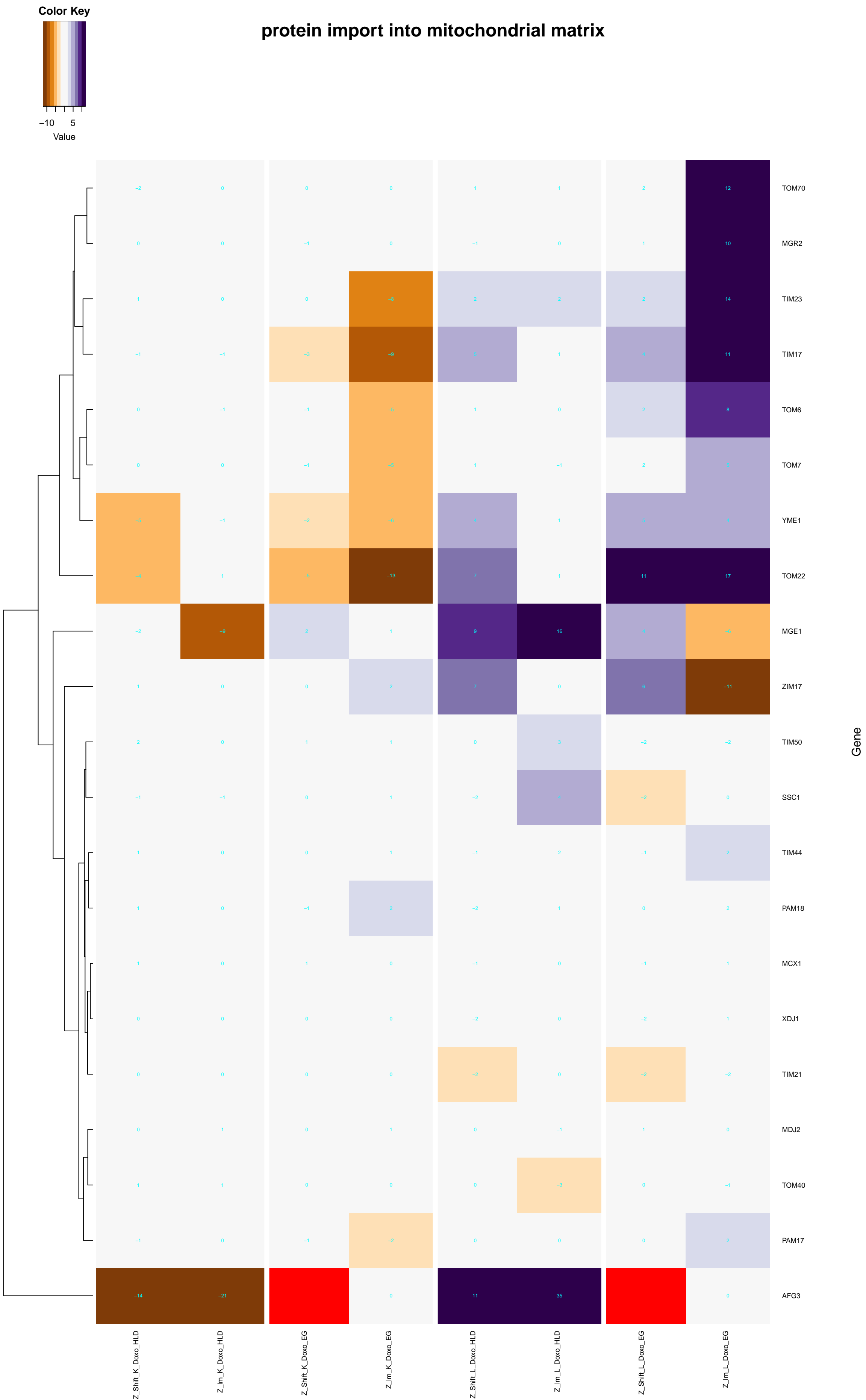

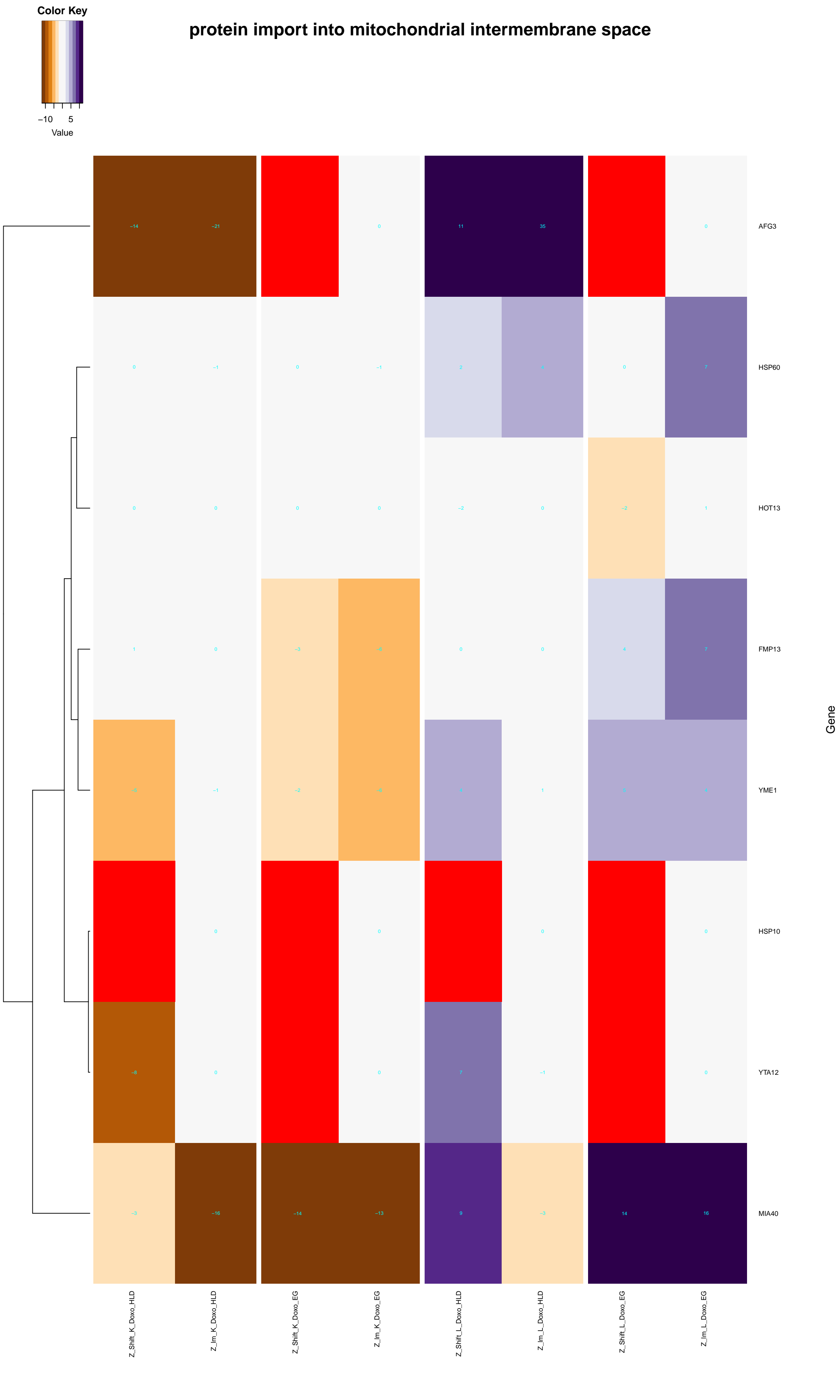

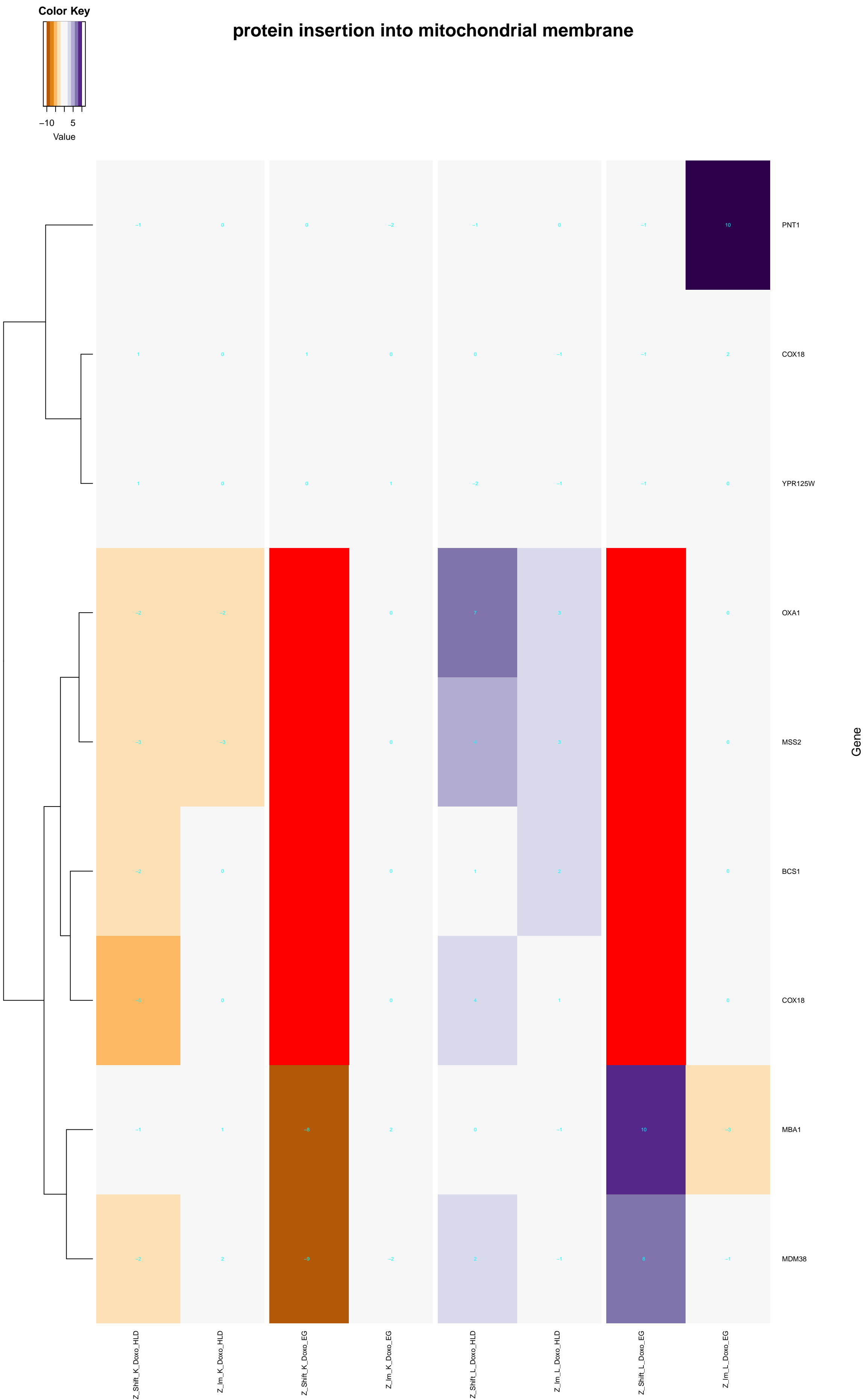

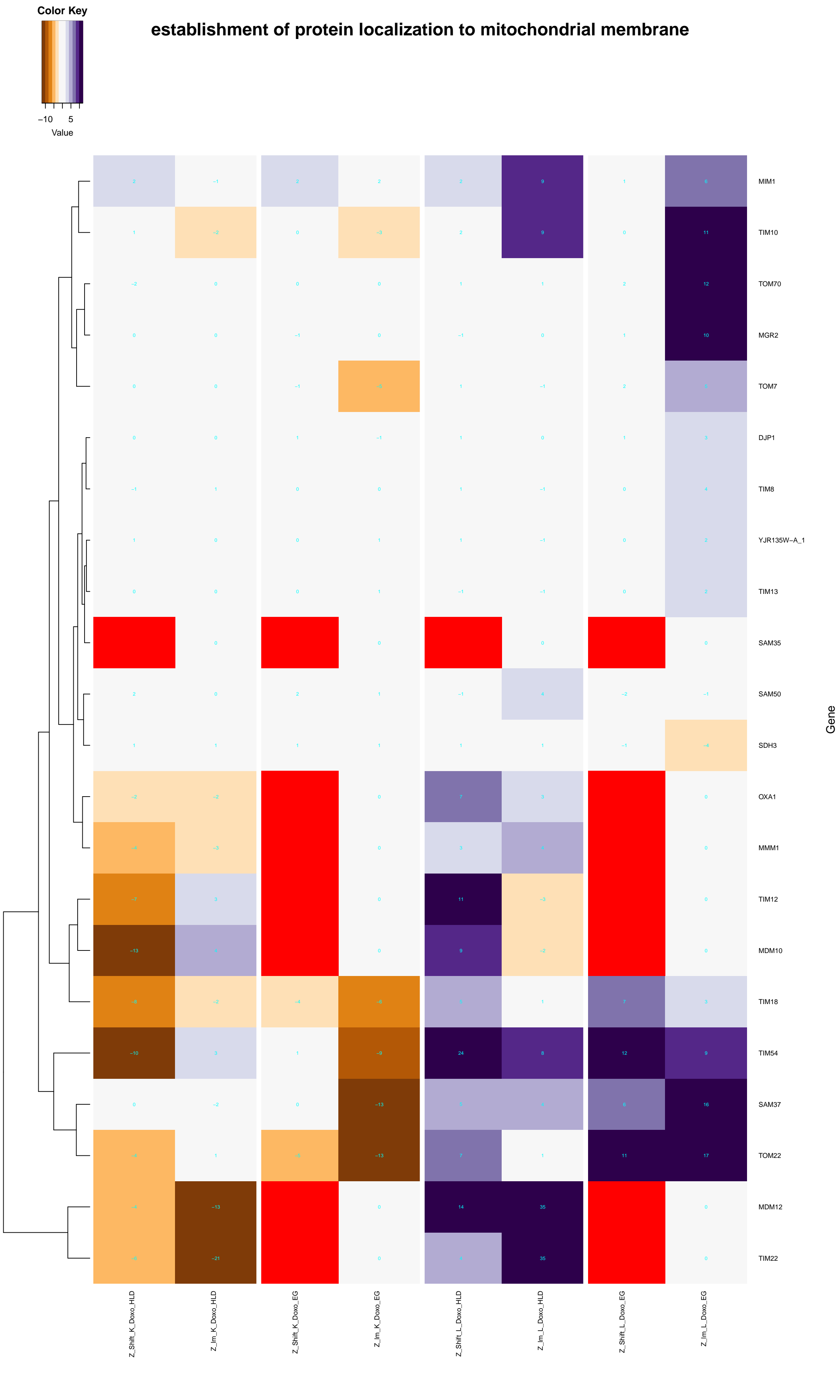

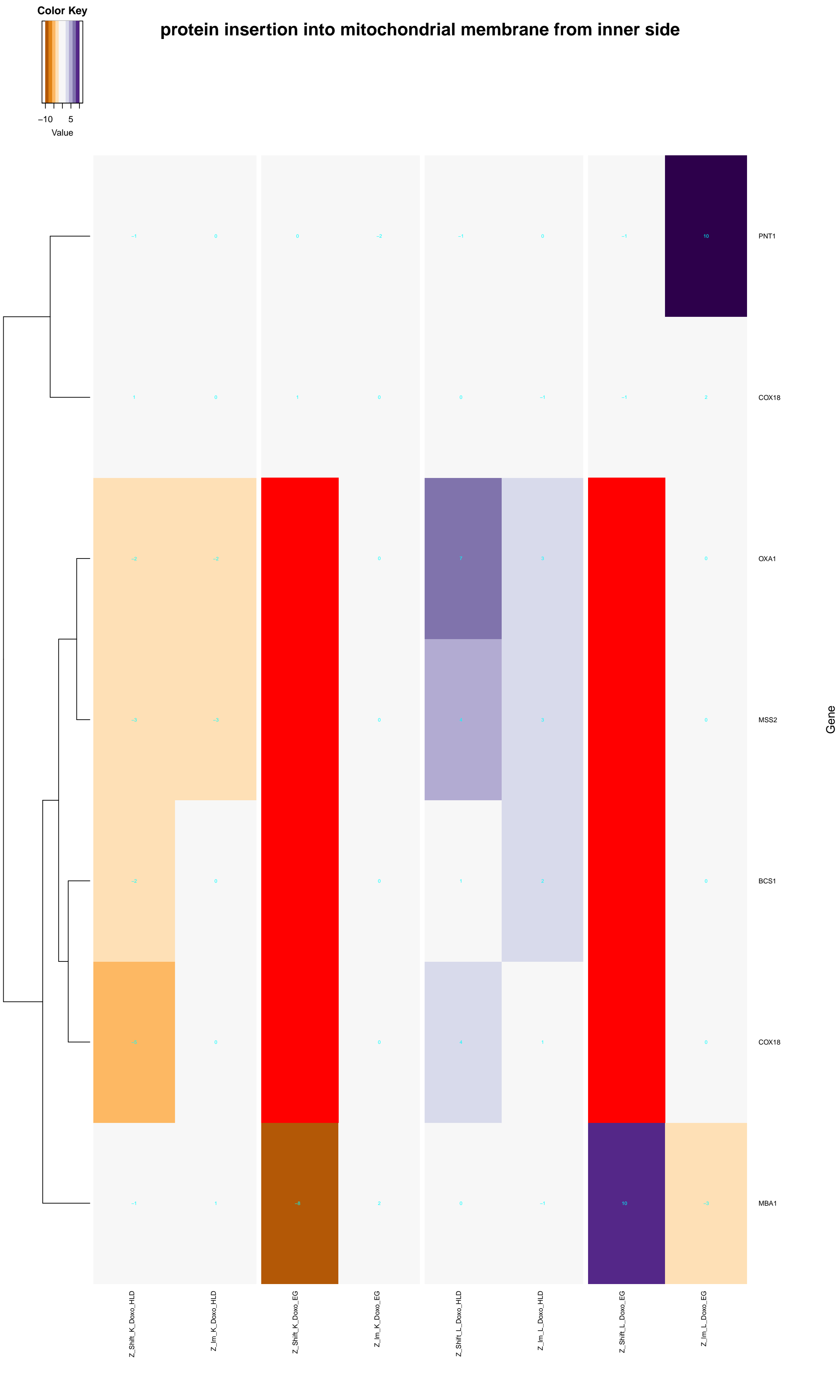

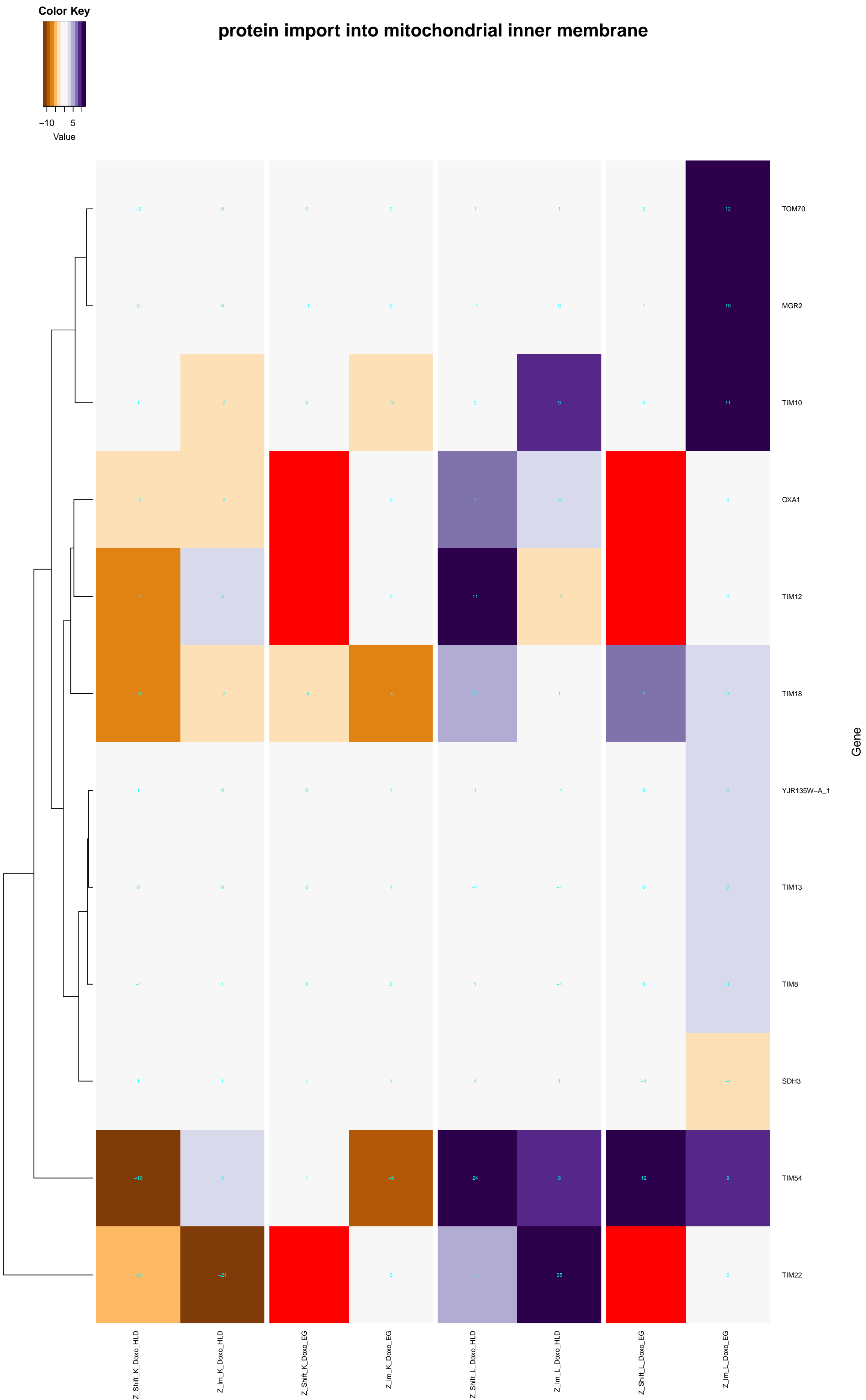

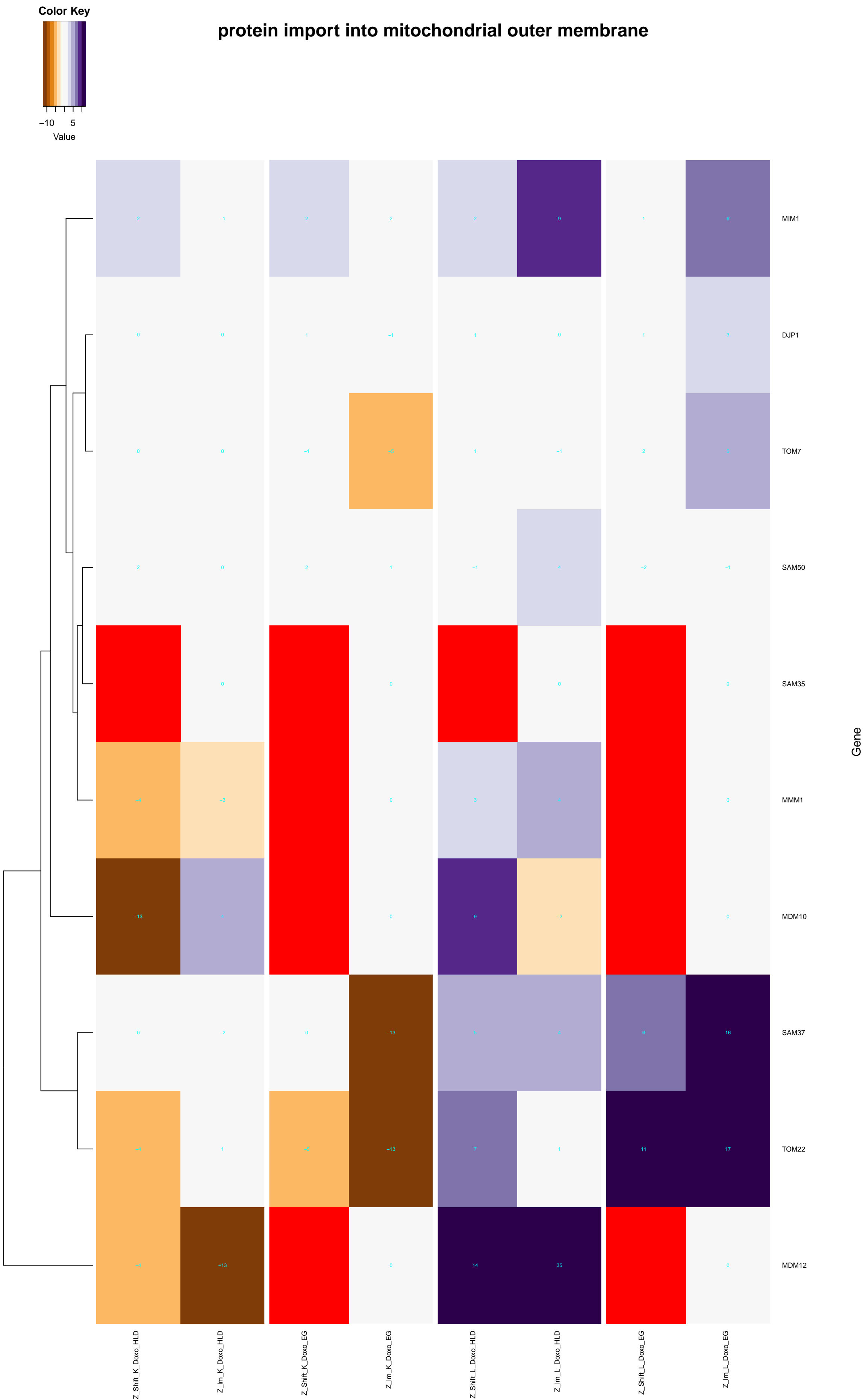

Supplement: Supplementary file 9 — Additional file 9. GO term-specific heatmaps for REMc/GTF-enriched clusters. GO term-specific heatmaps for significant GO process terms were generated as described in methods and Figs. 3 and 4. Any related child terms are presented in subsequent pages of the parent file name. GO terms with more than 100 children, with 2 or fewer genes annotated to the term, or a file size over 300KB are not shown. All heatmaps are generated with the same layout (see Figs. 3 and 4). [file 40170_2019_201_MOESM9_ESM.bz2 › Additional_File9_GOTermHeatmaps/Additional_File9_GOTermHeatmap/establishment_of_protein_localization_to_mitochondrion.pdf]

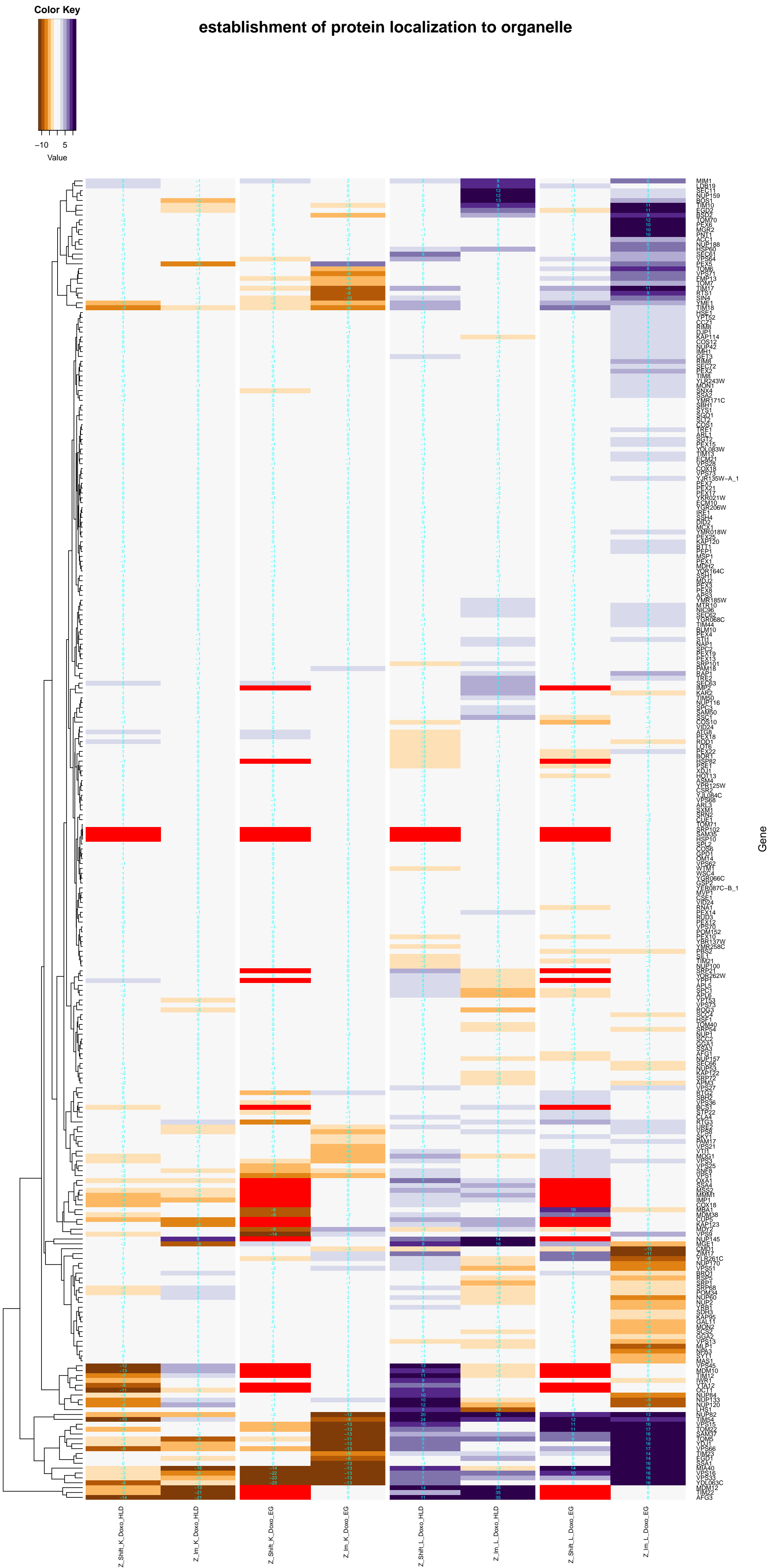

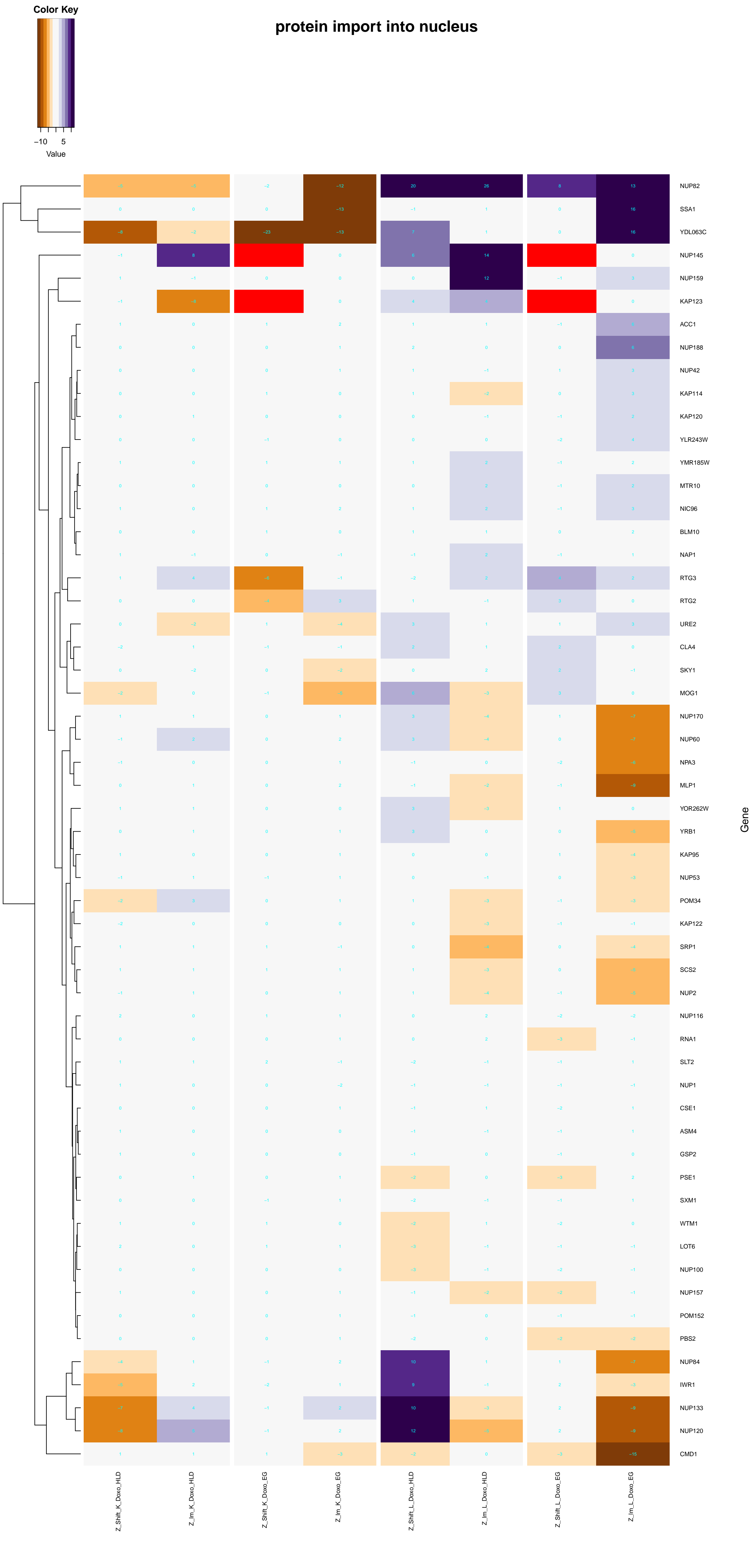

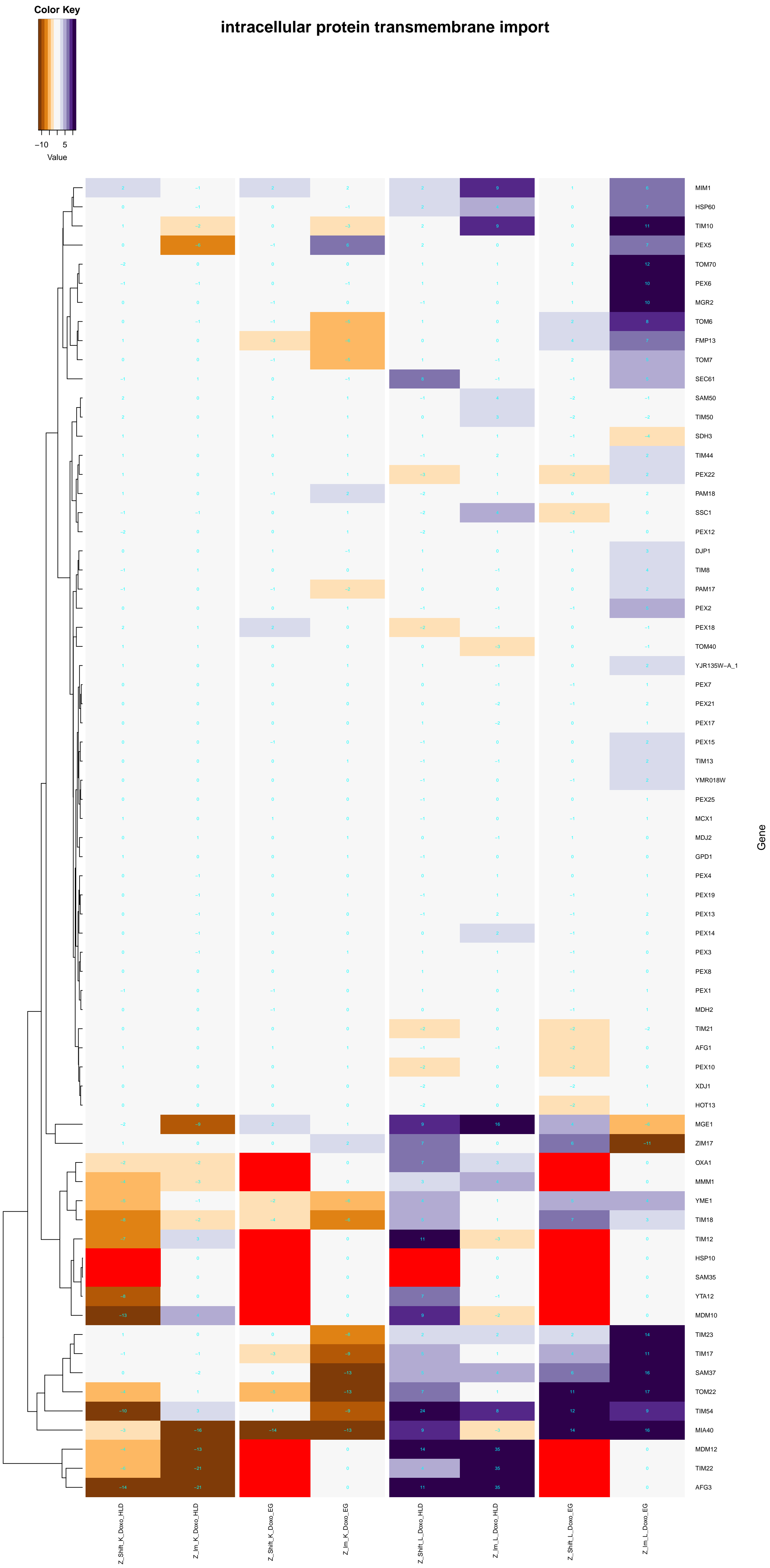

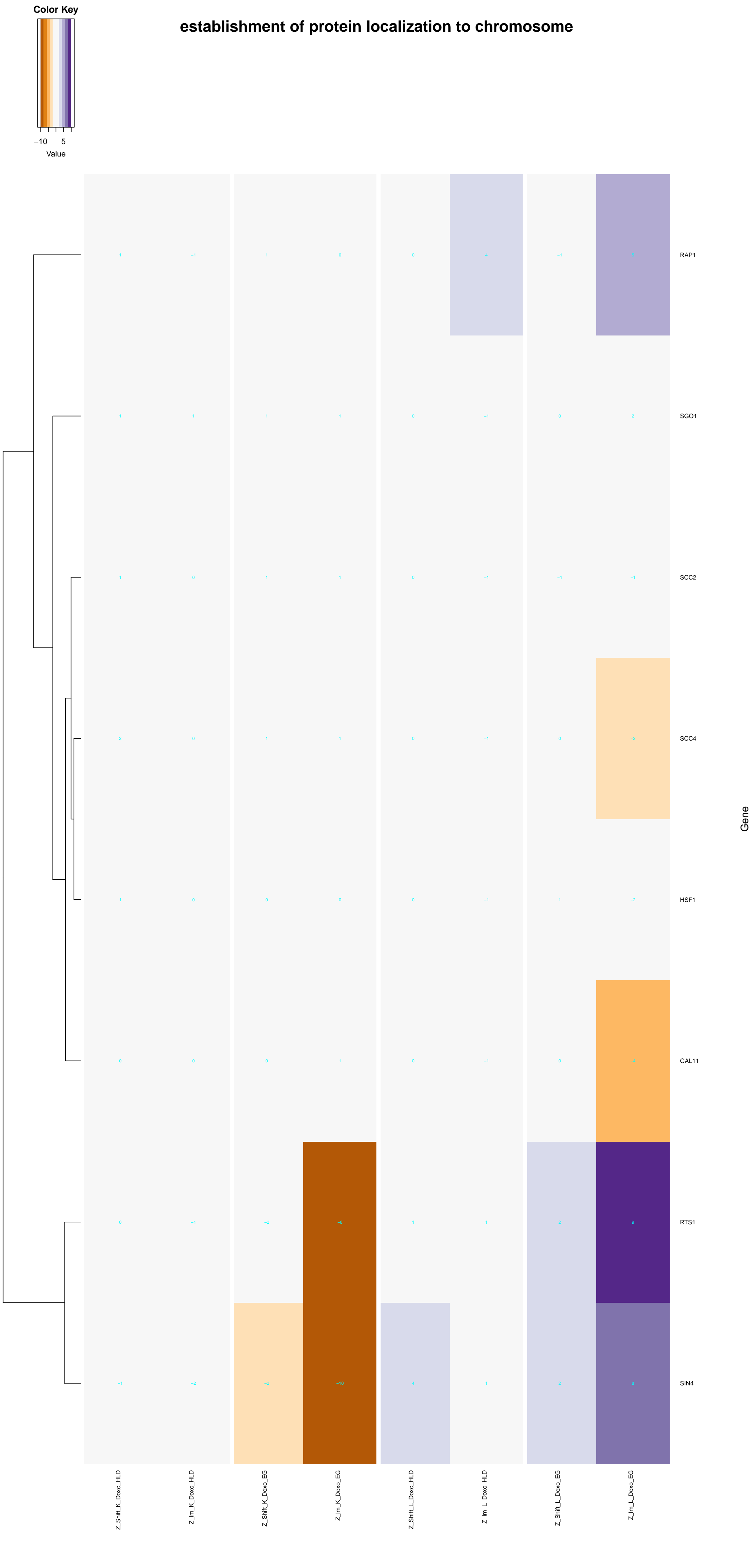

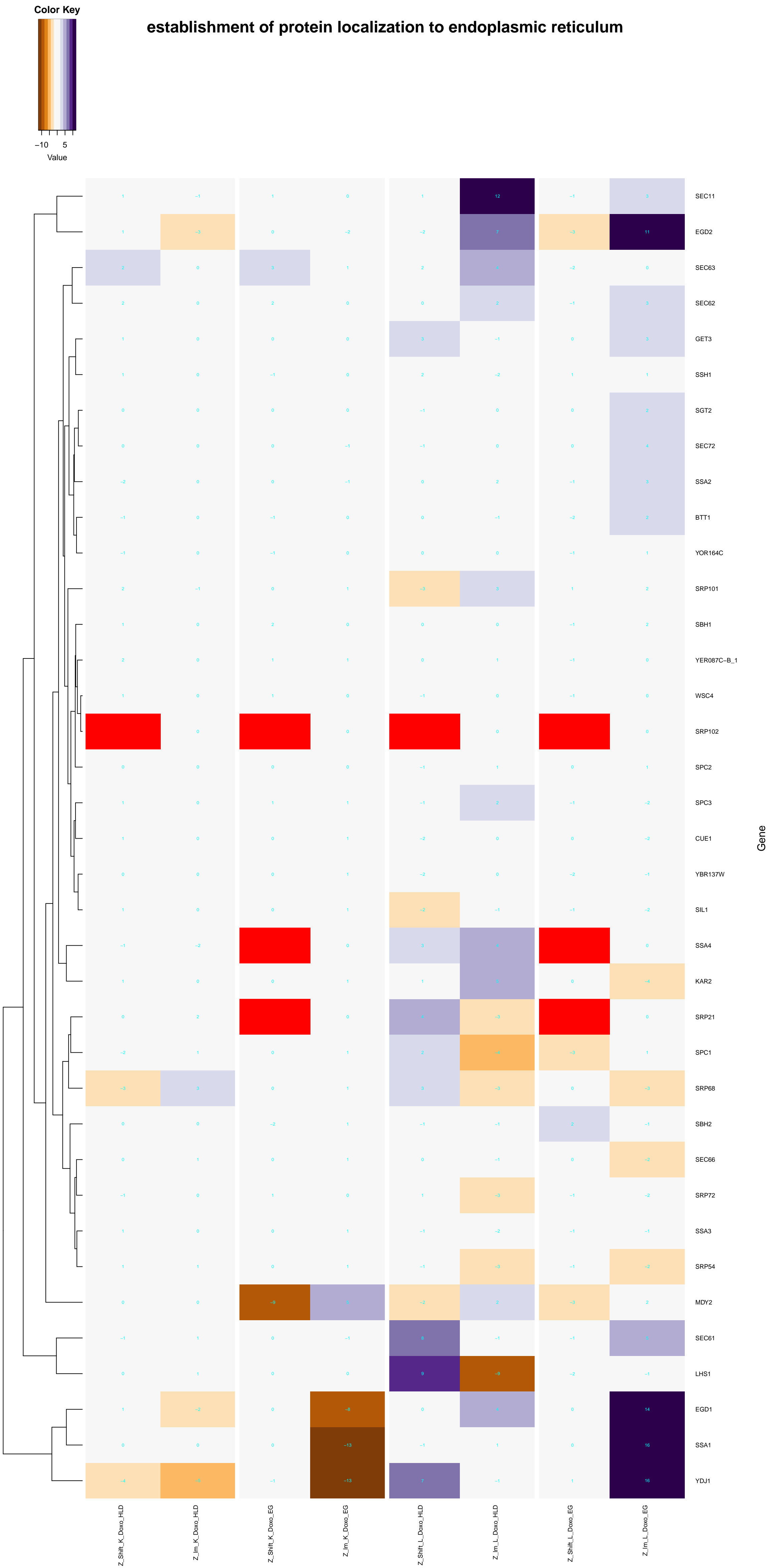

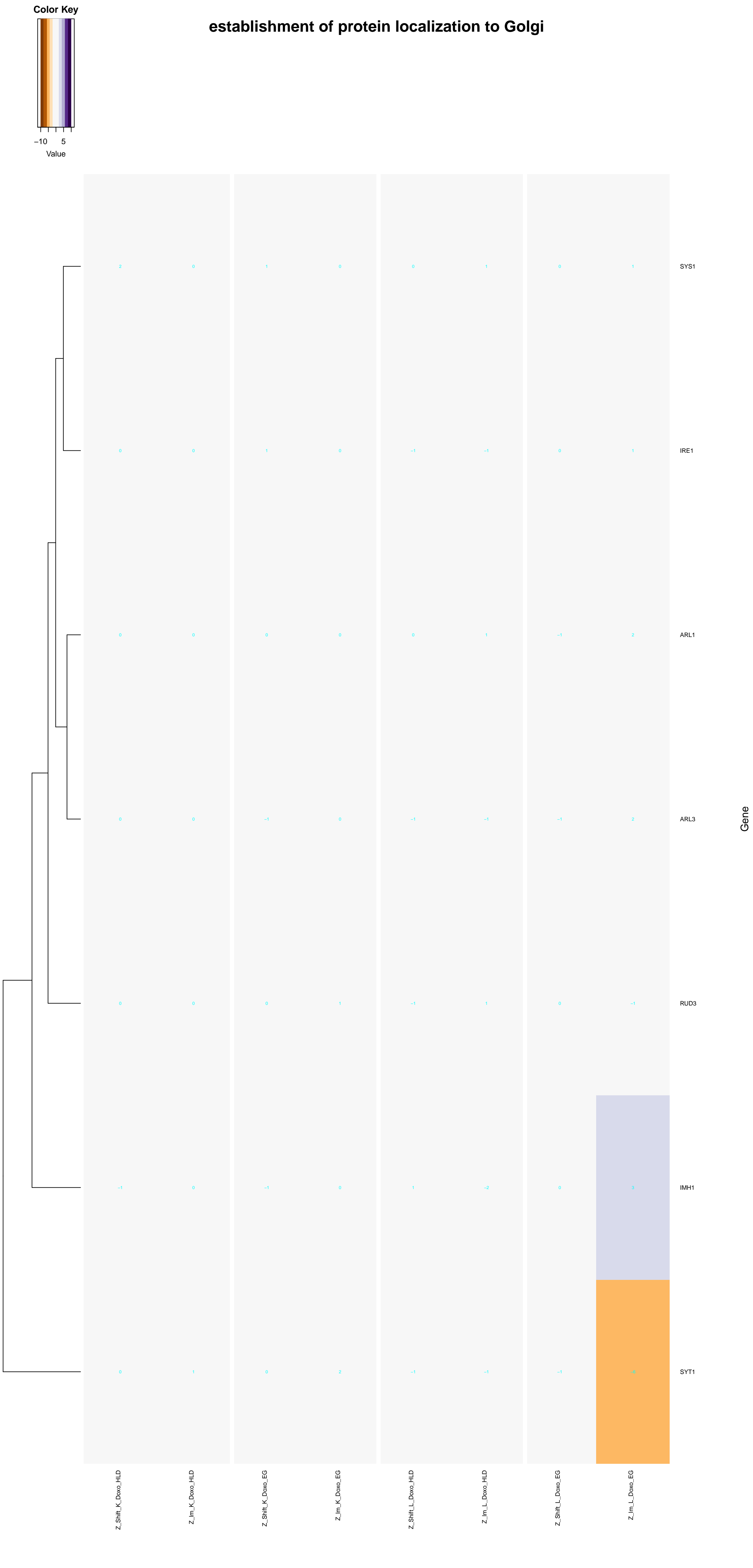

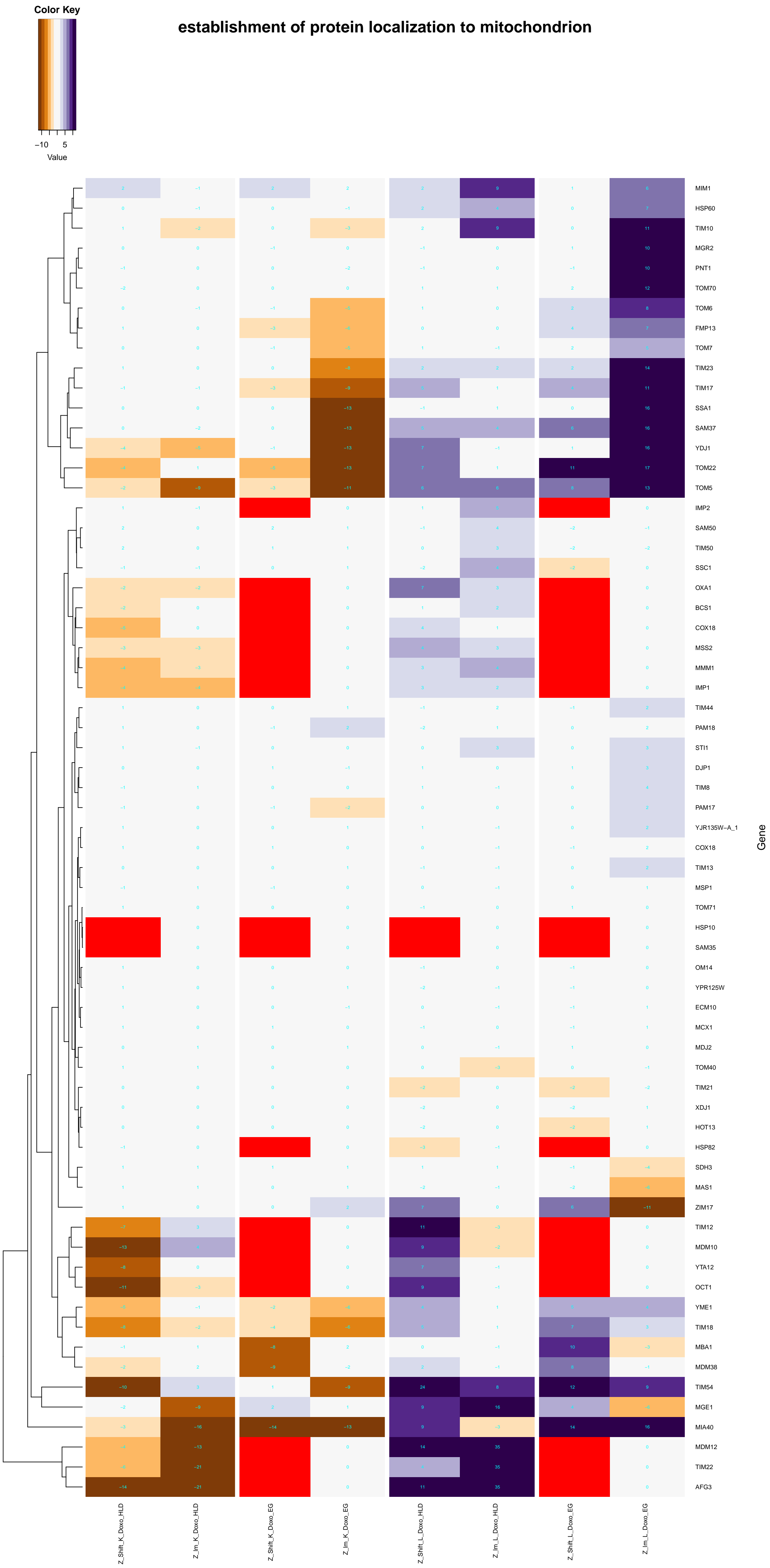

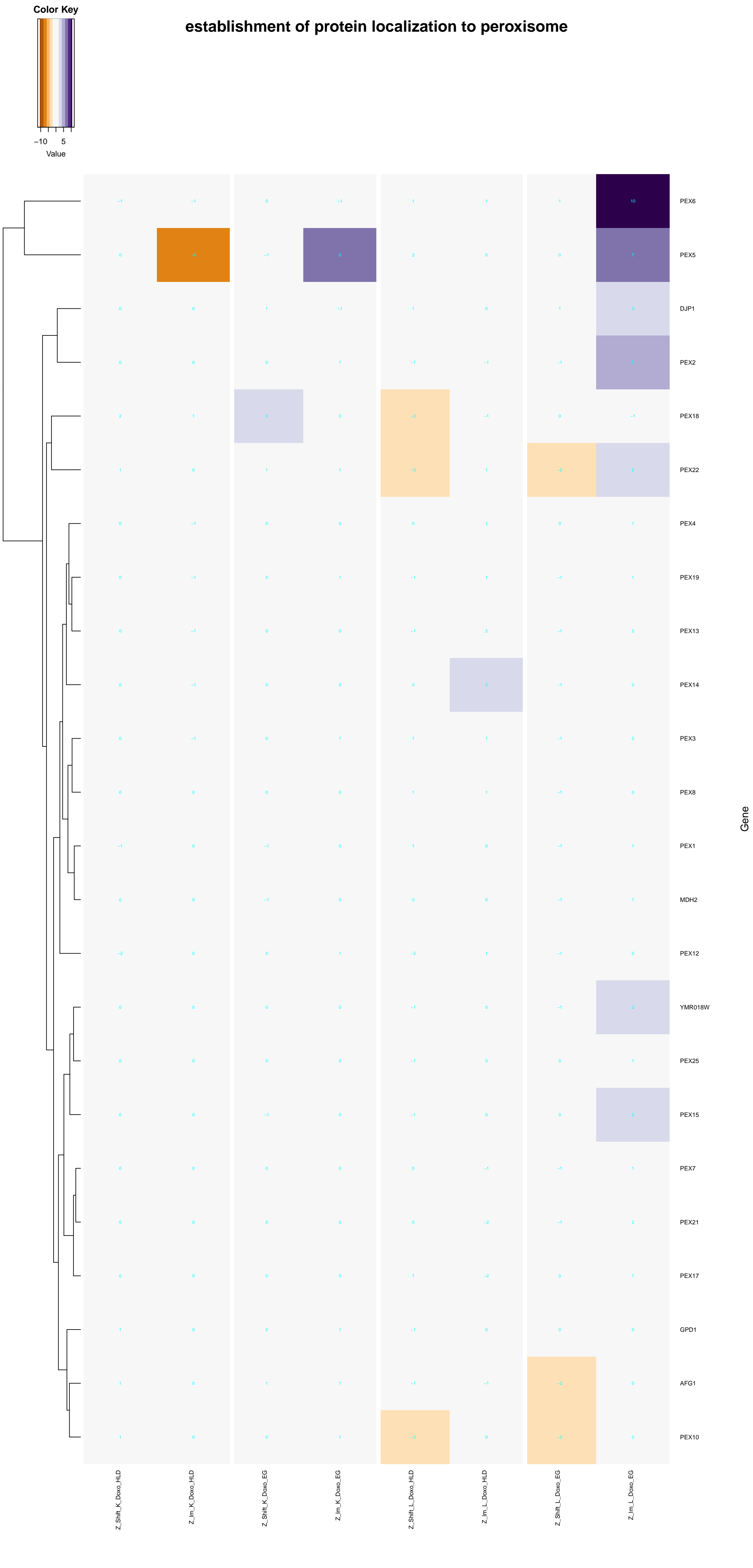

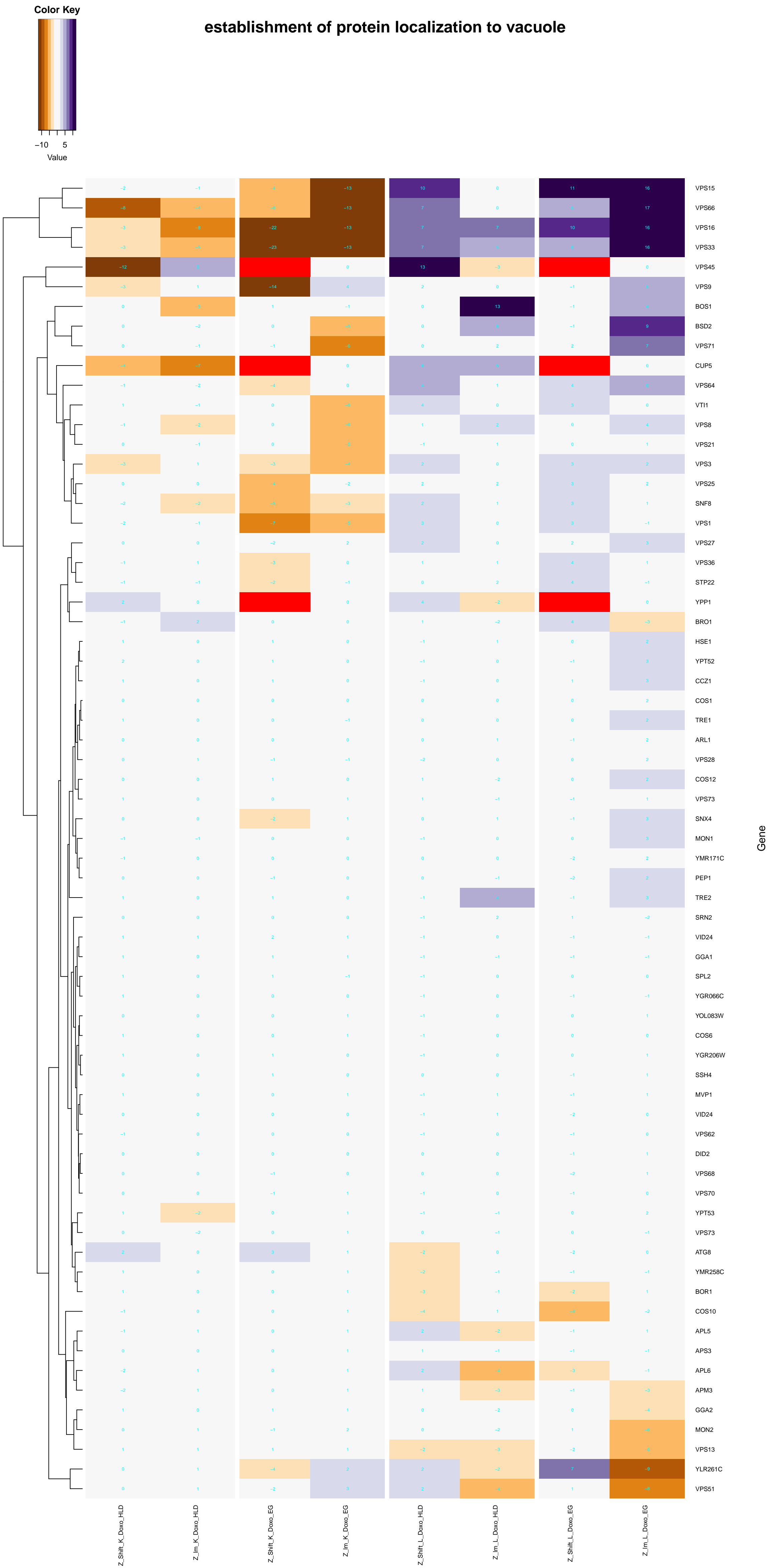

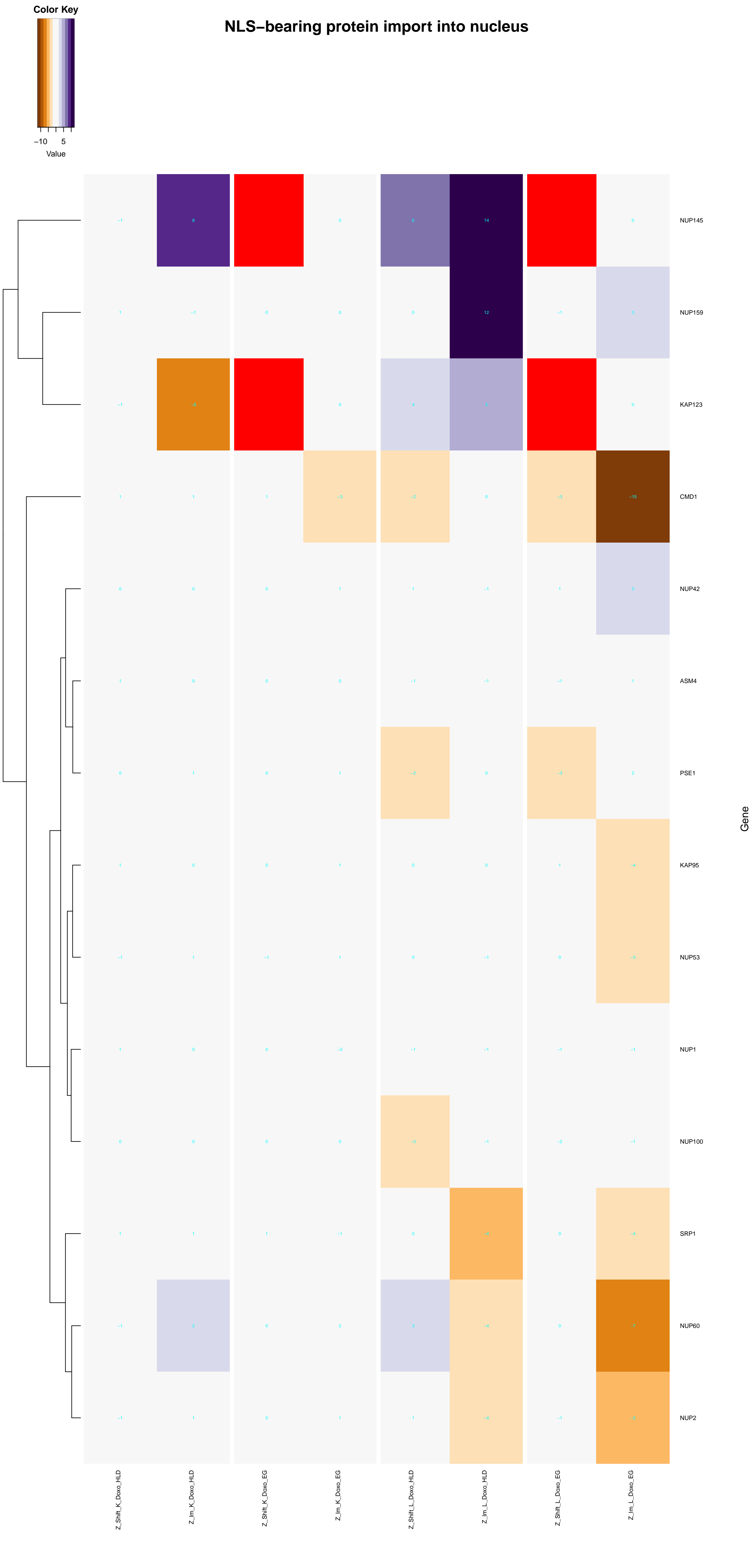

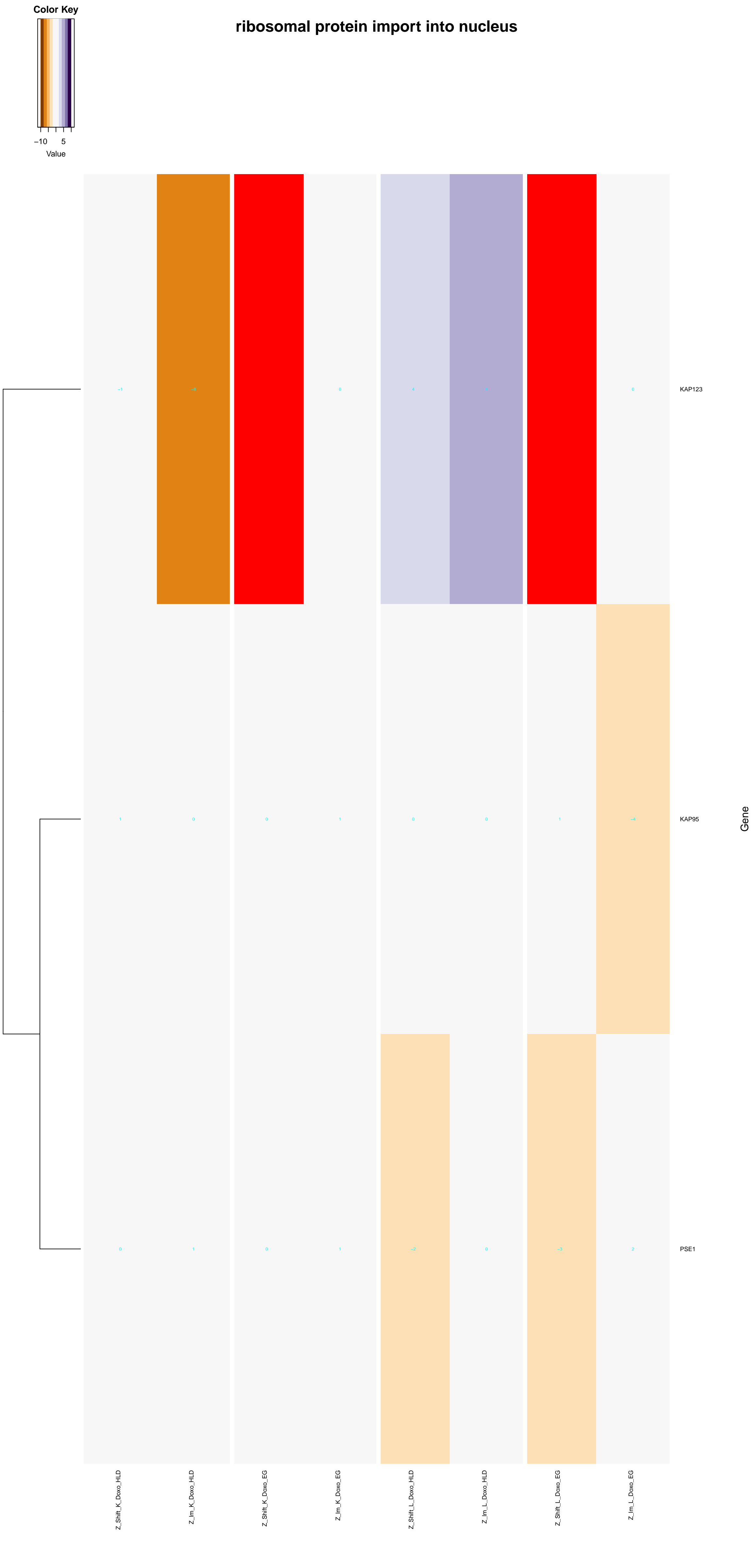

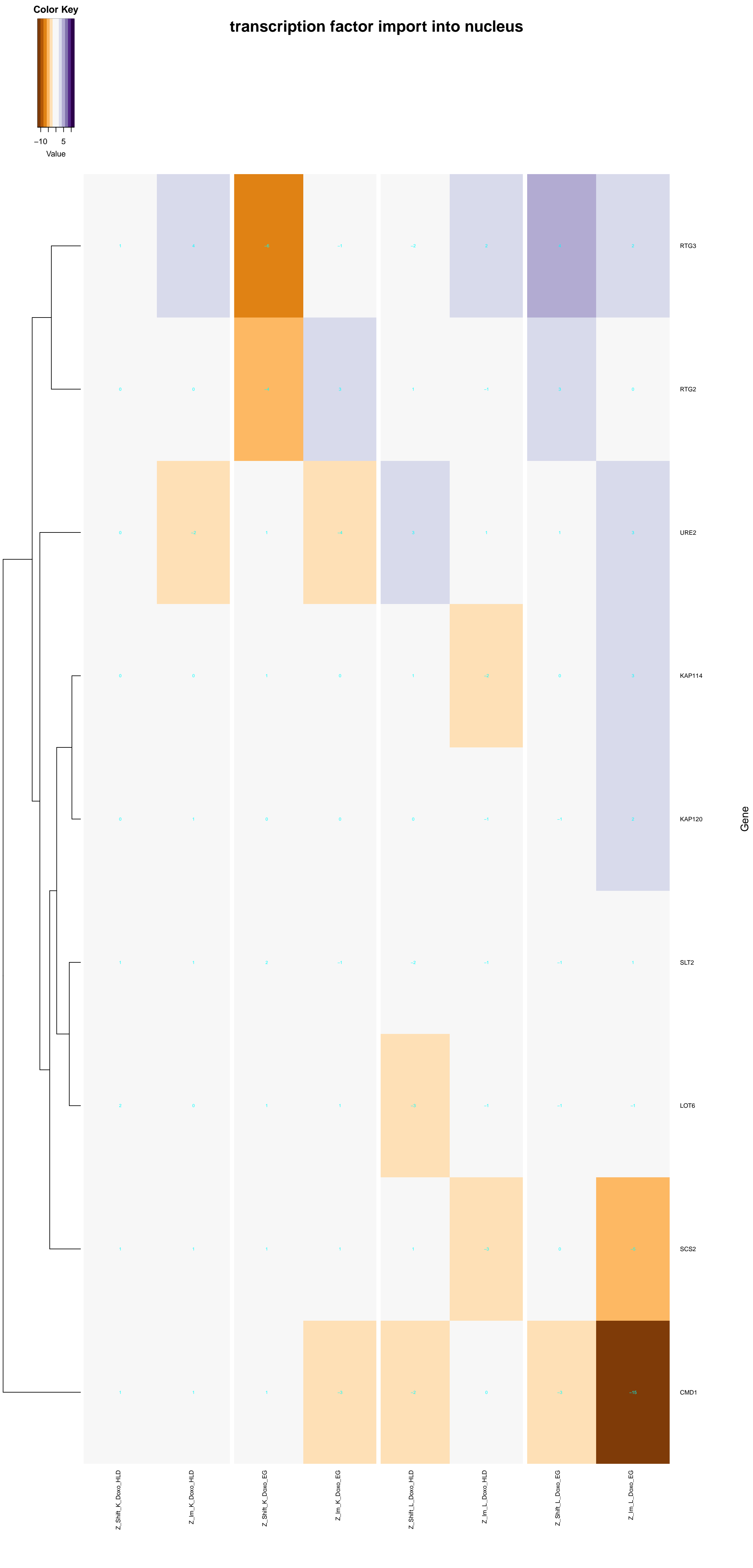

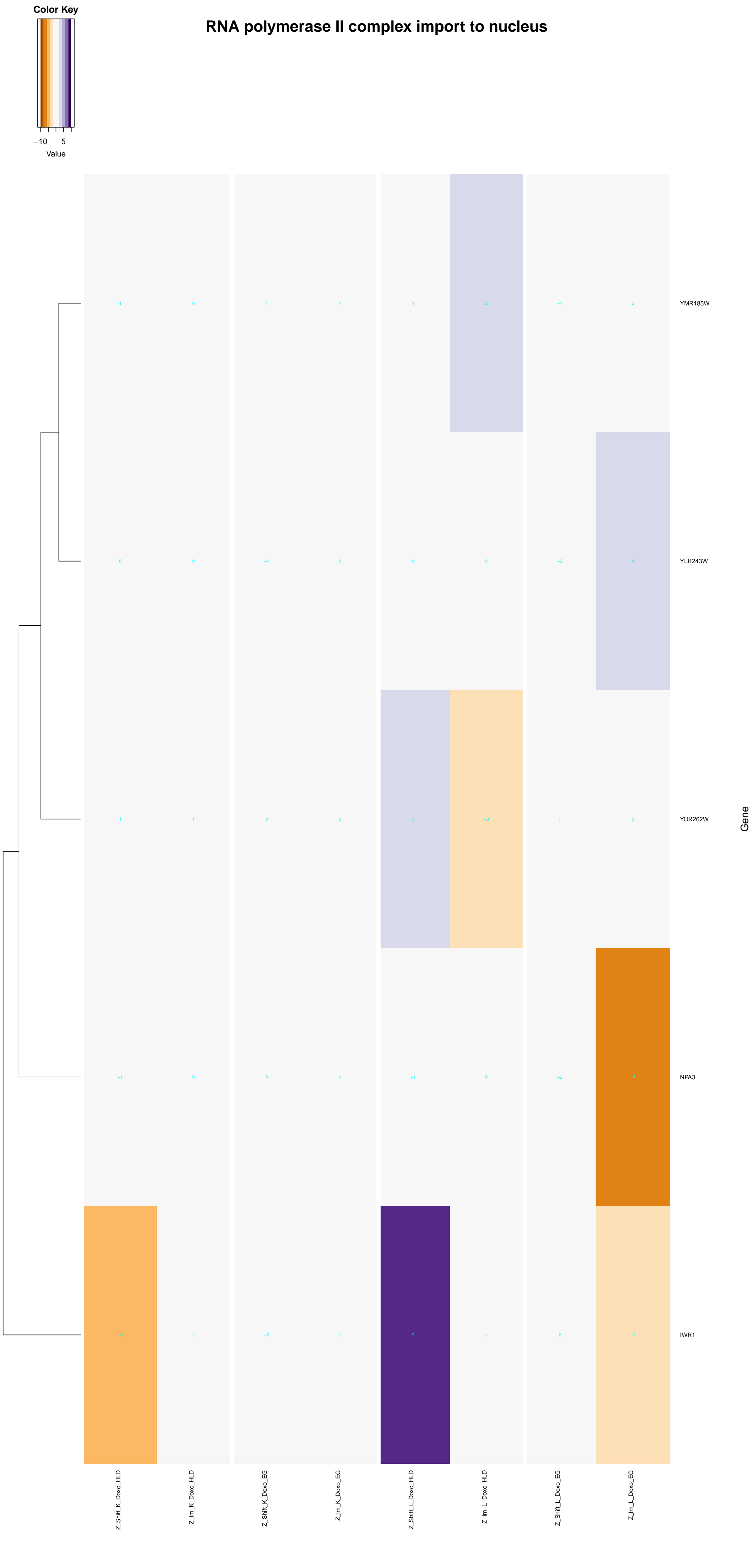

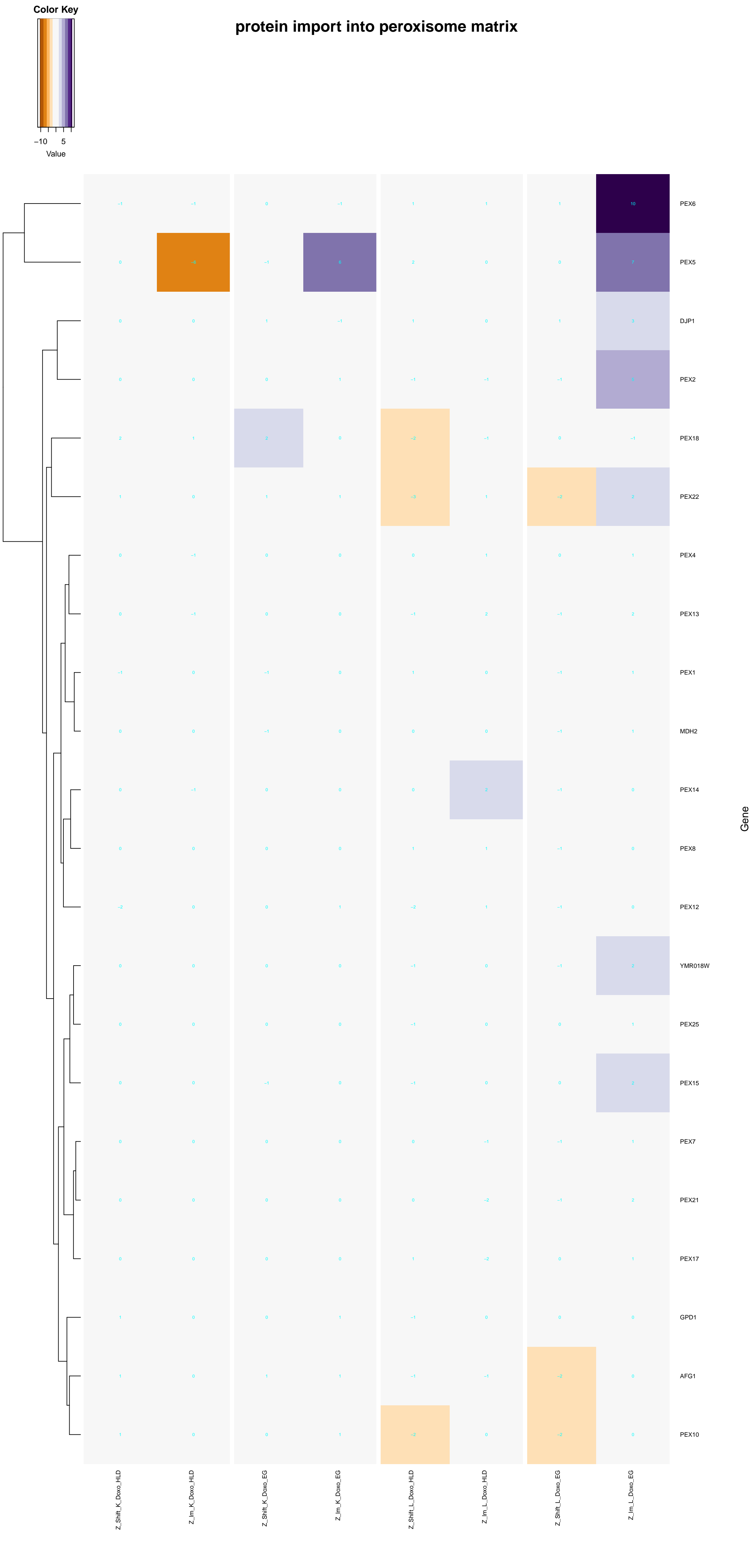

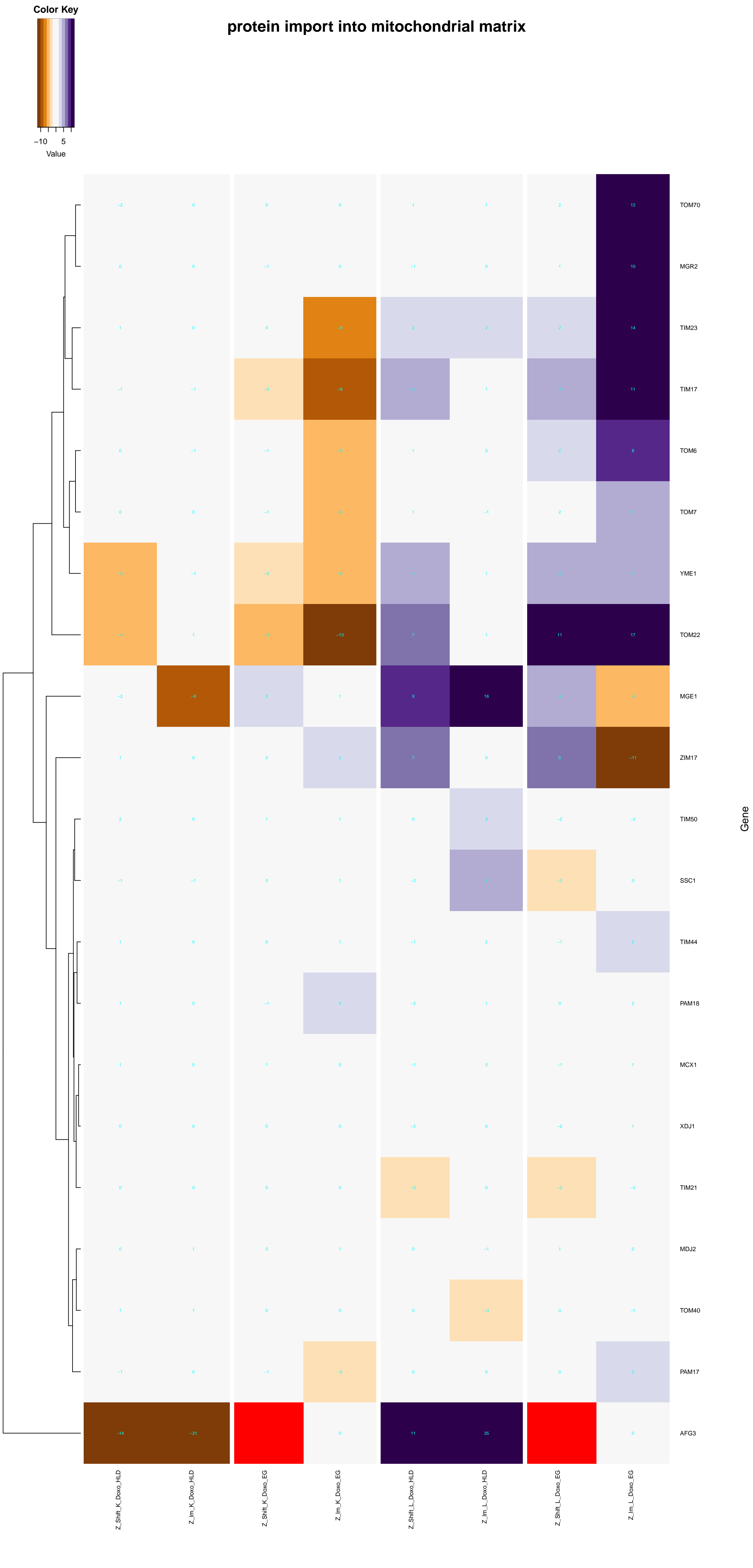

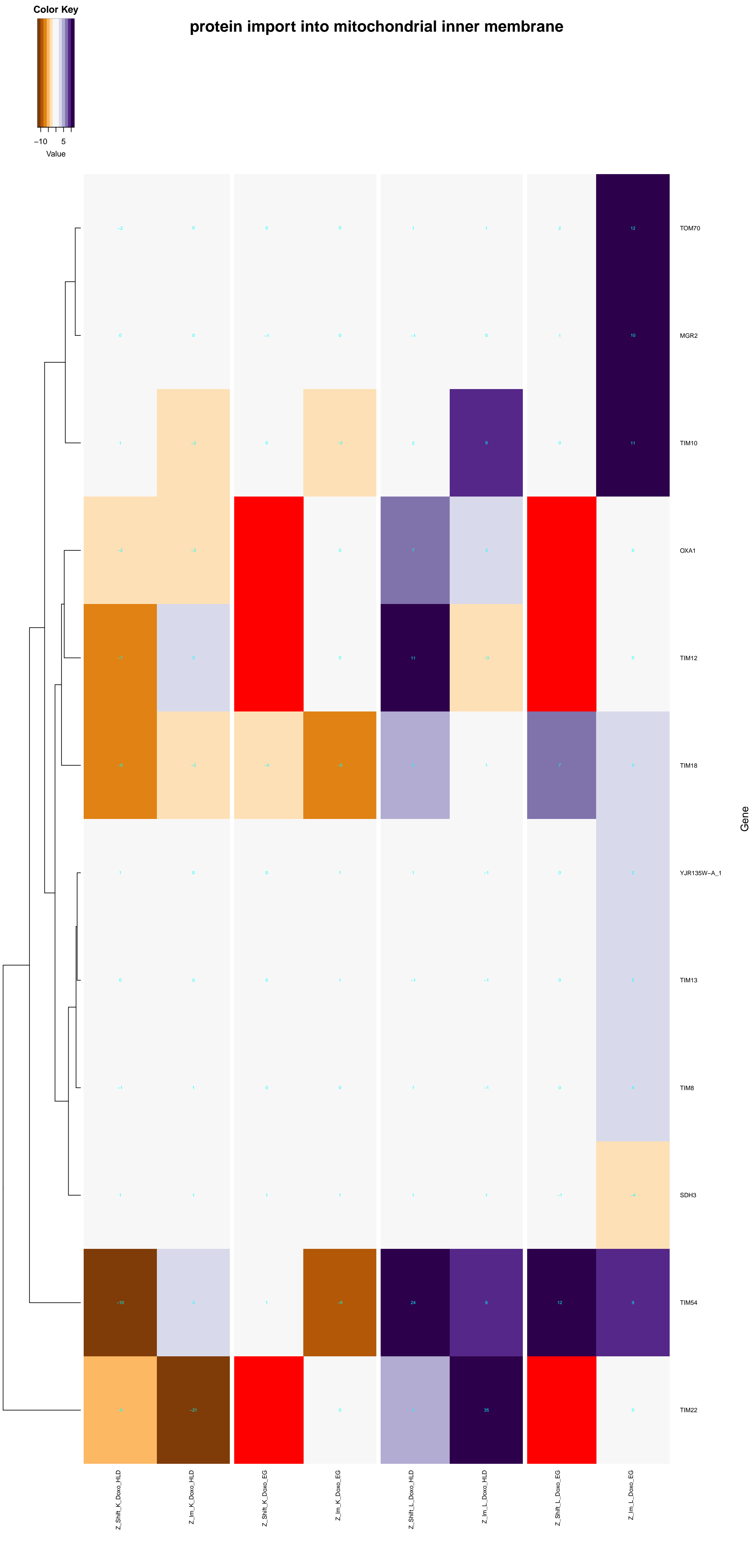

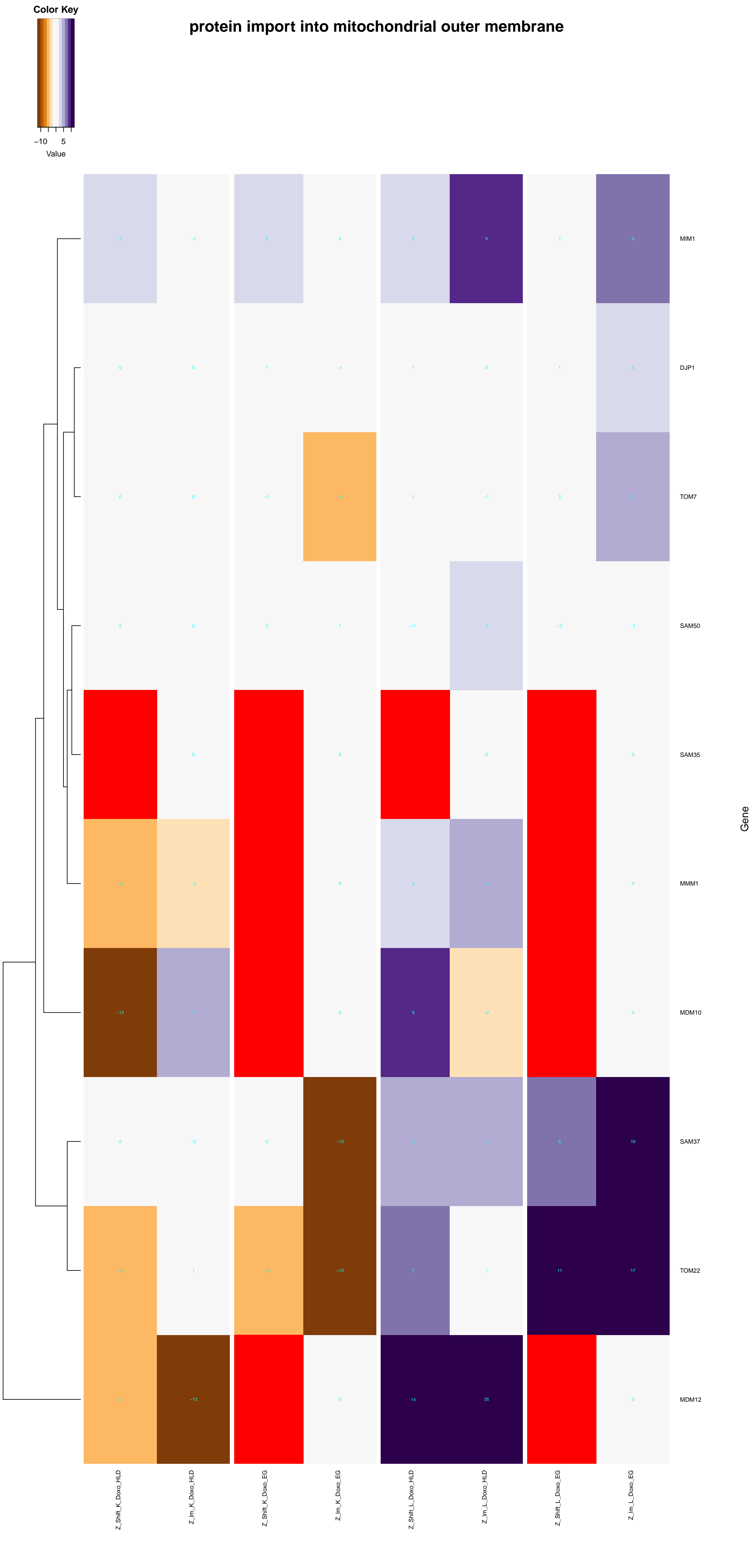

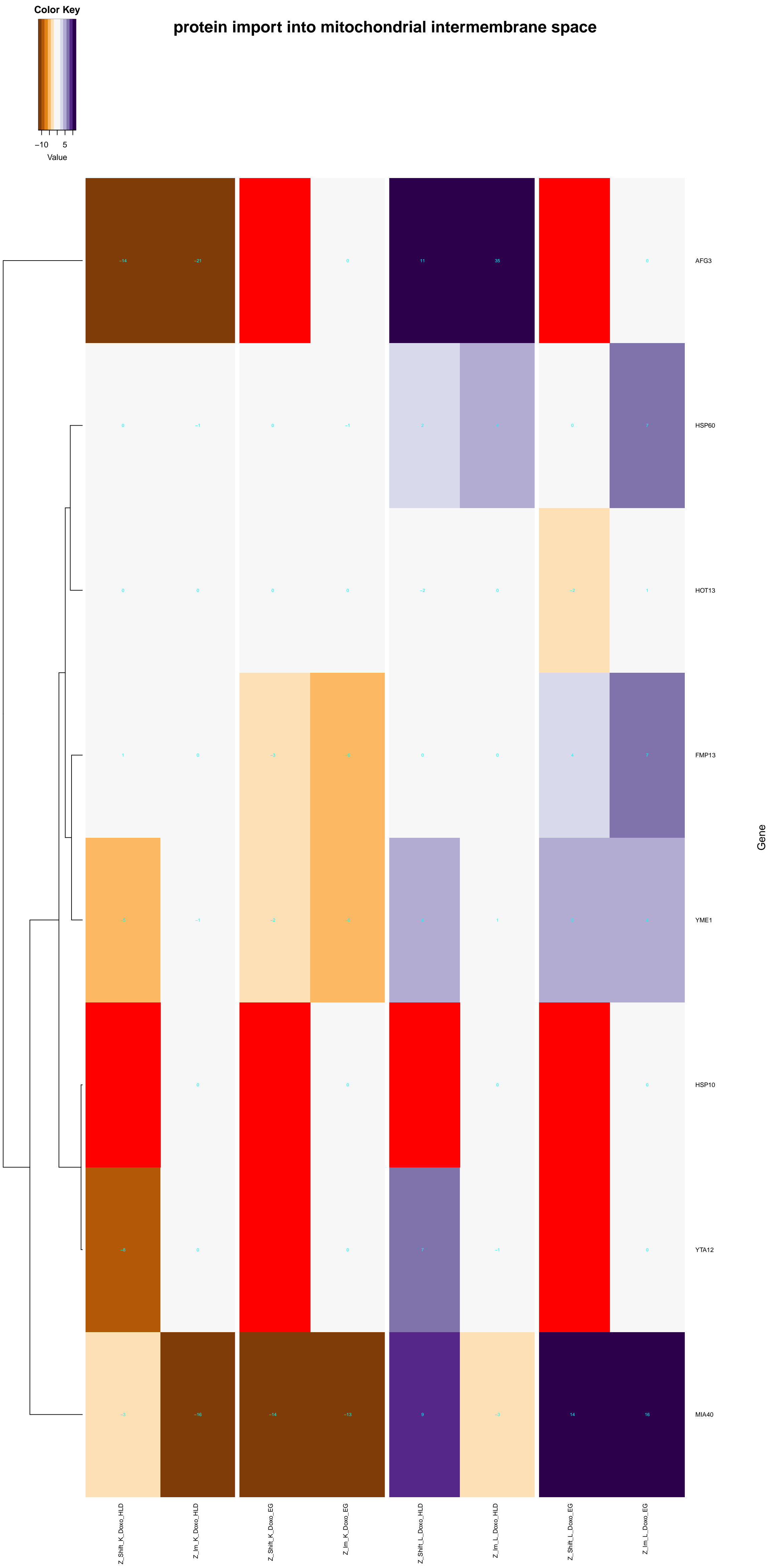

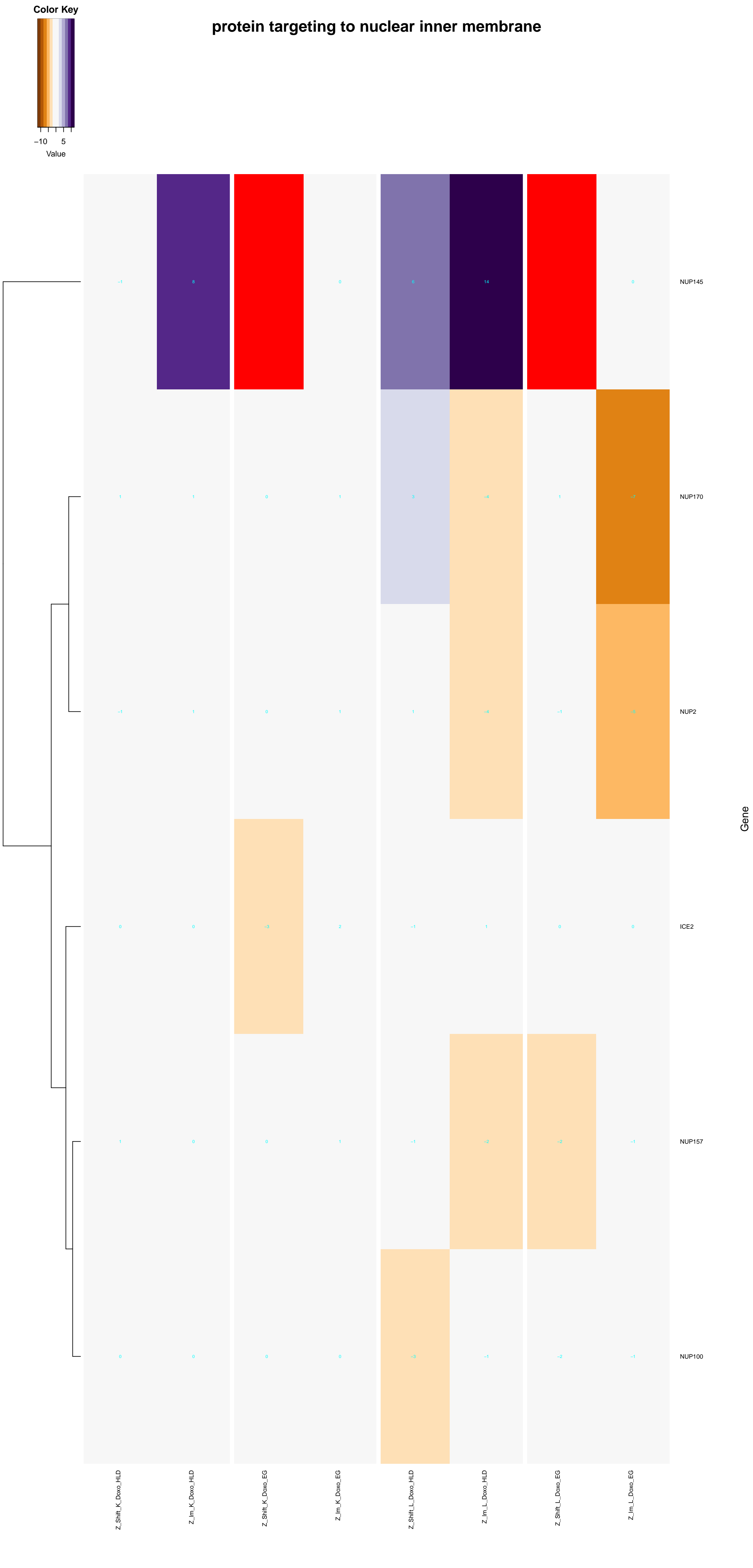

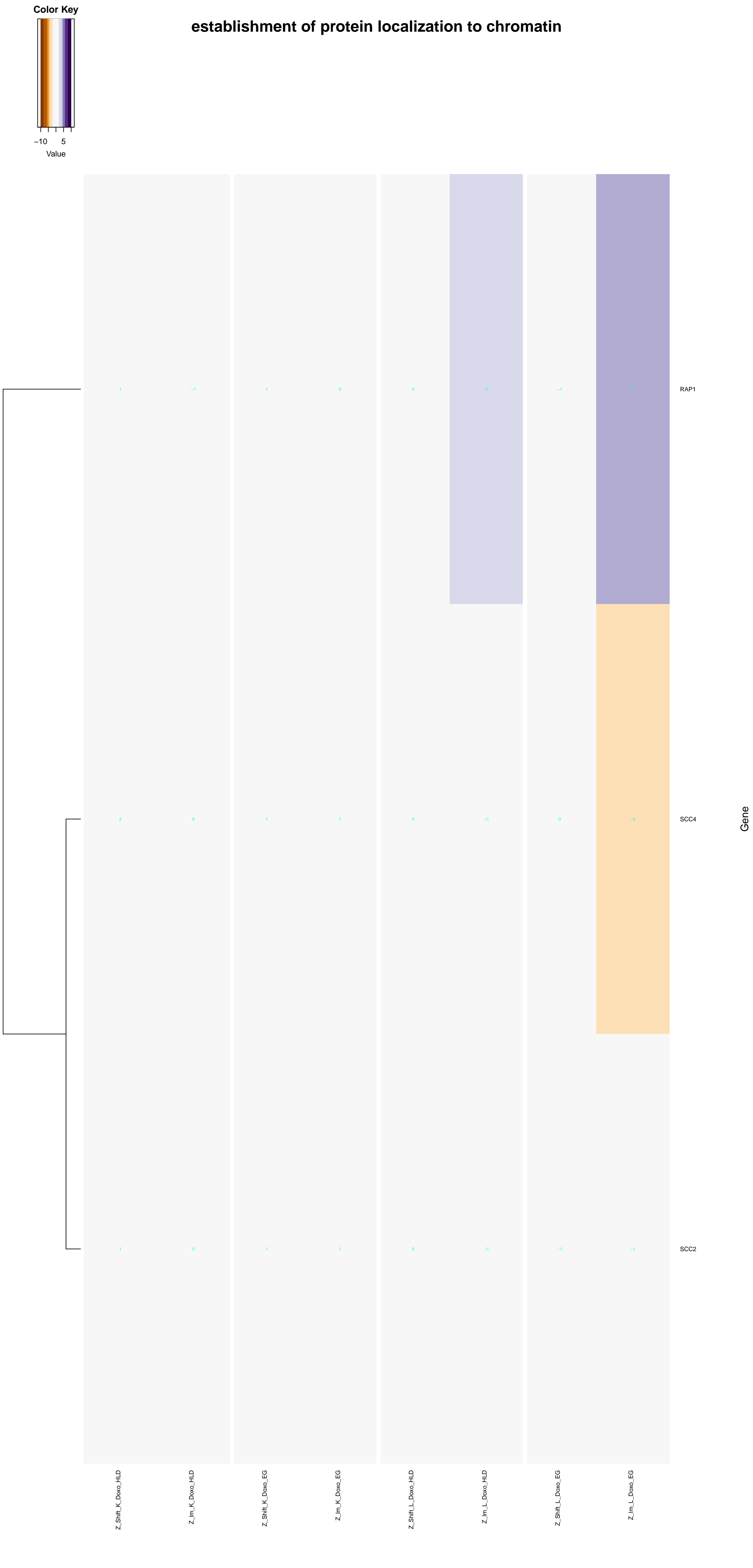

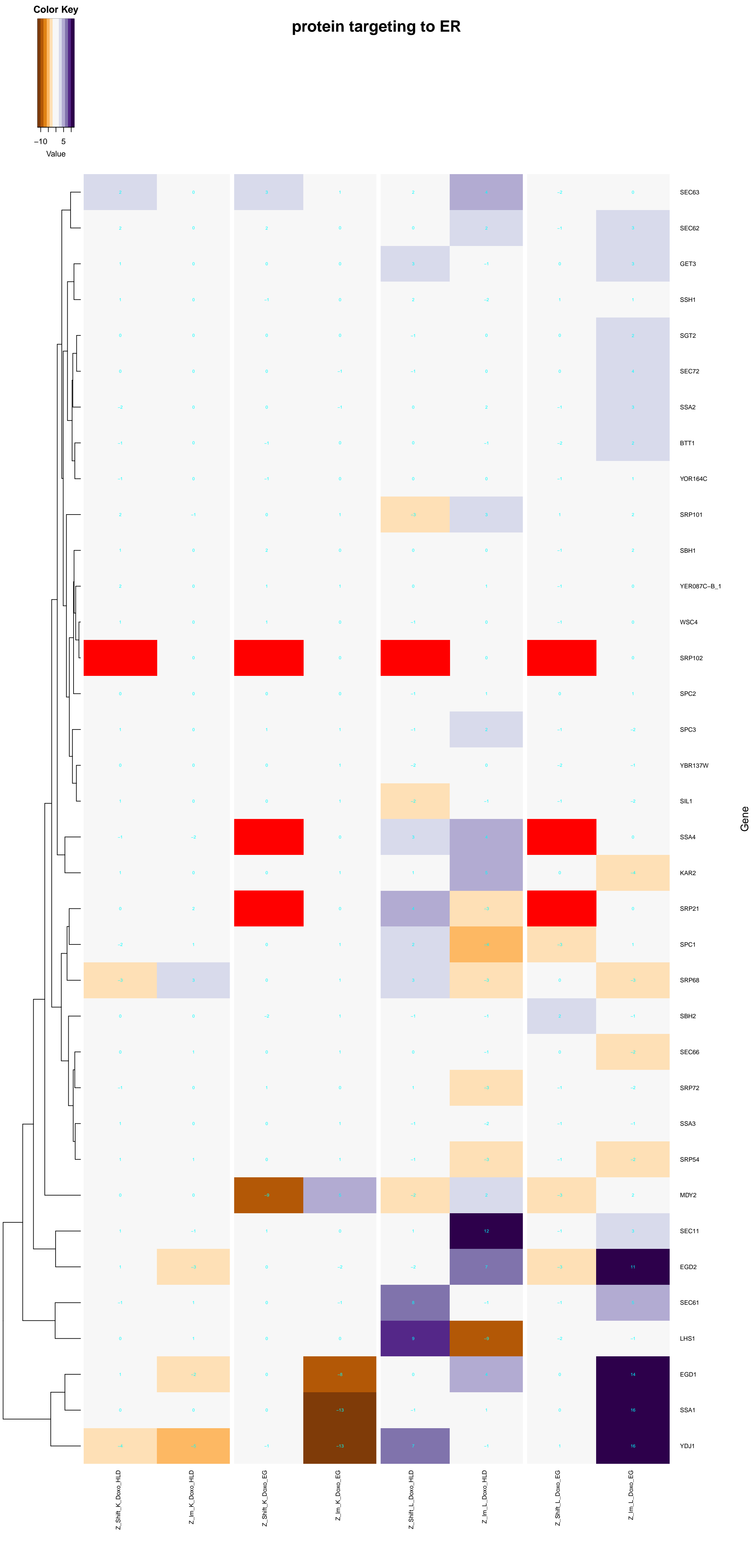

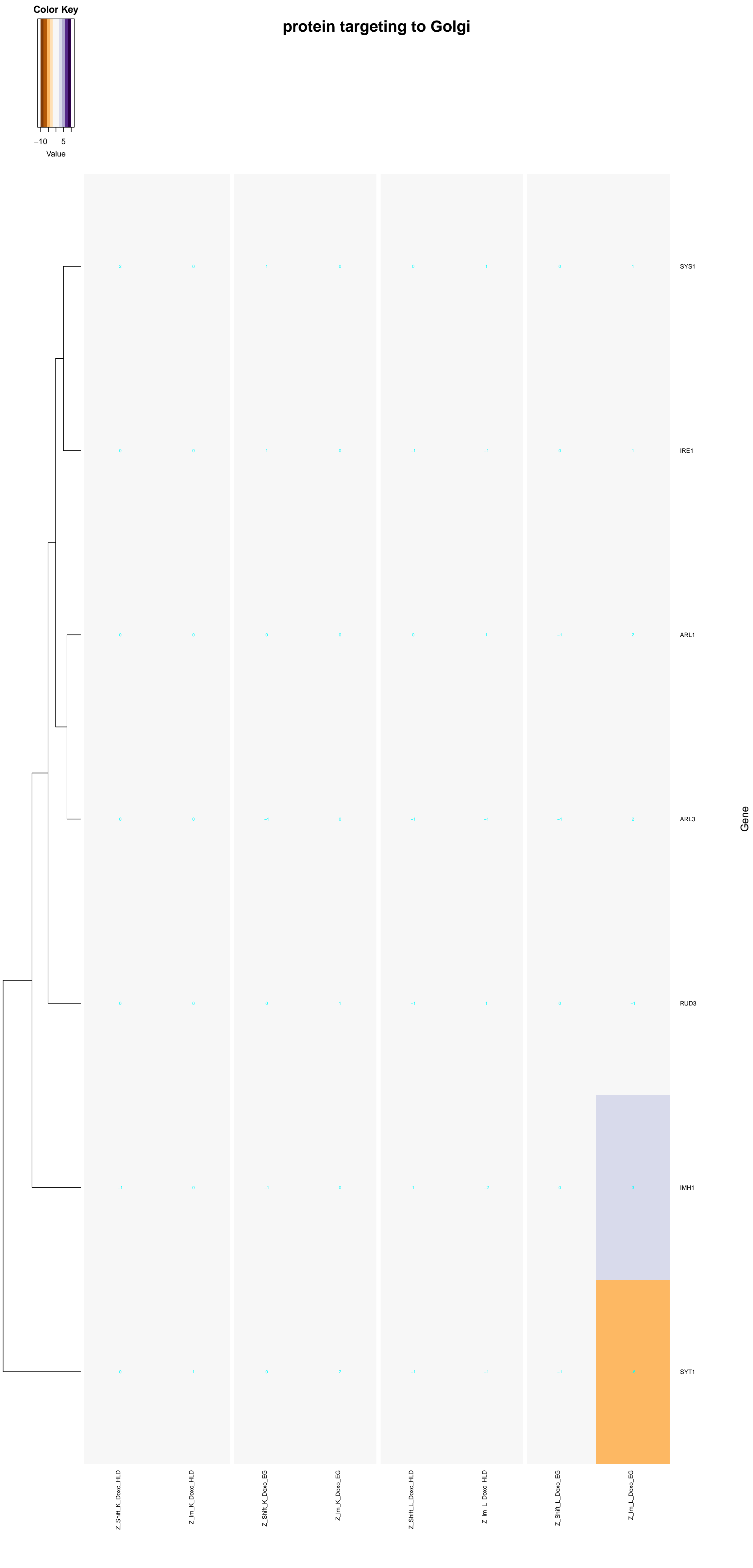

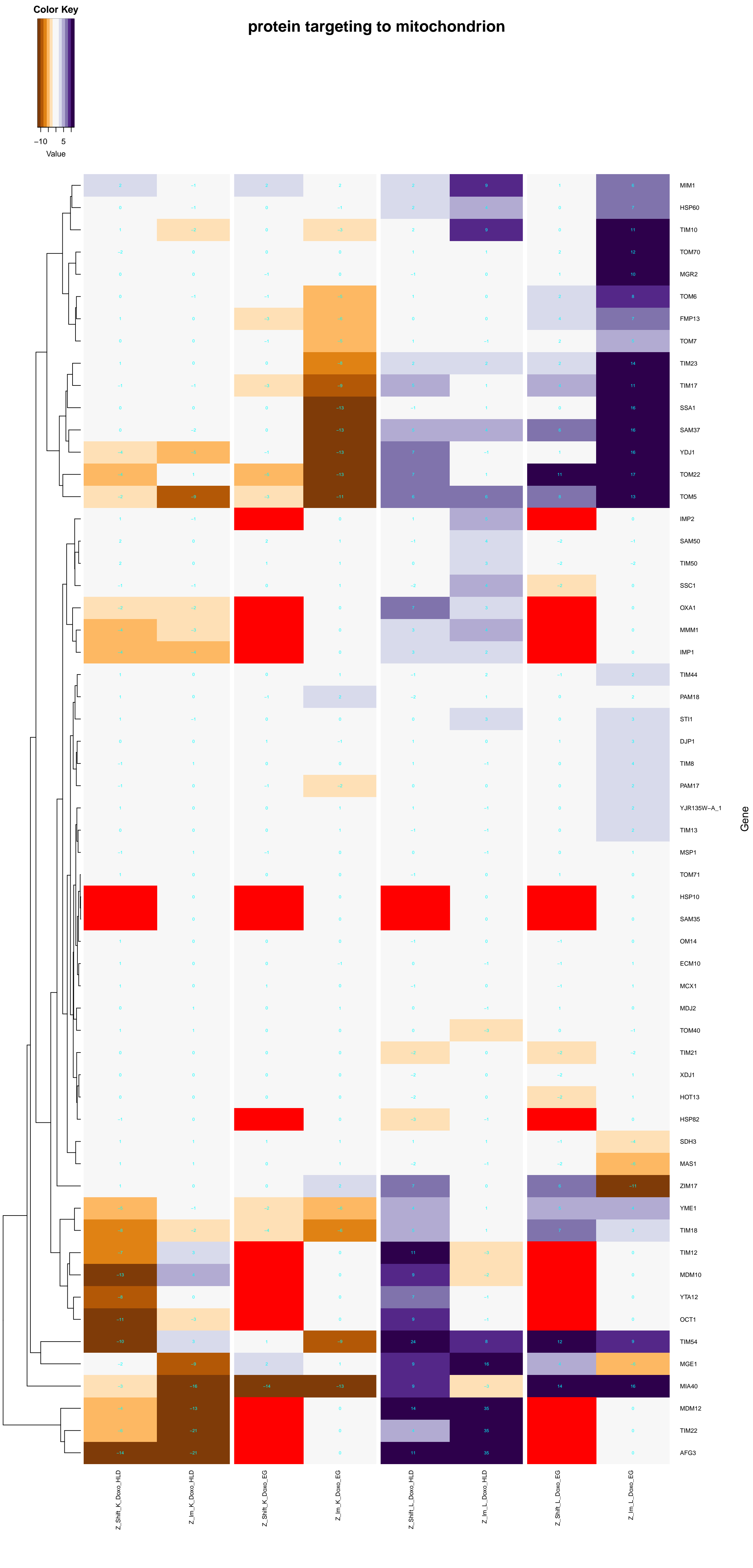

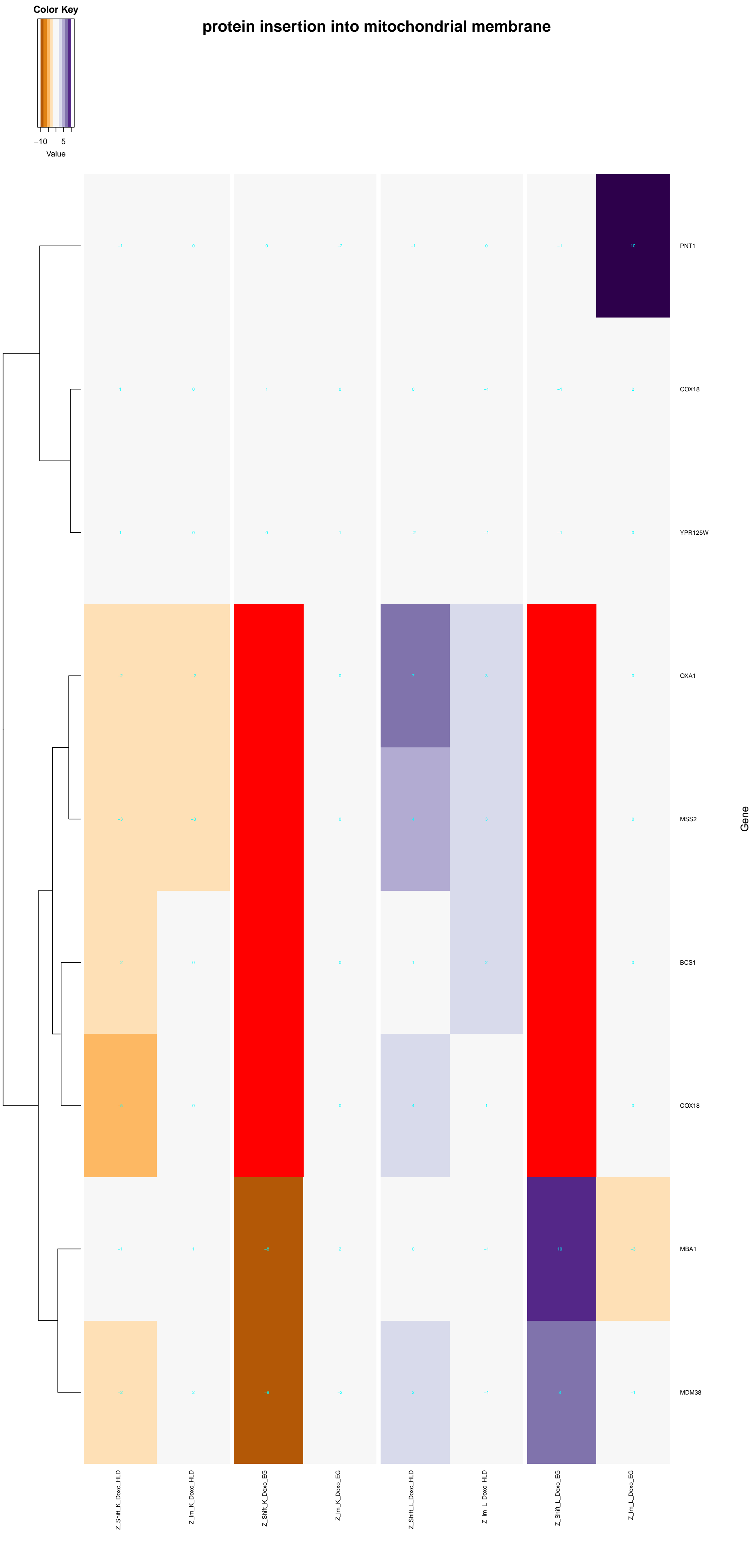

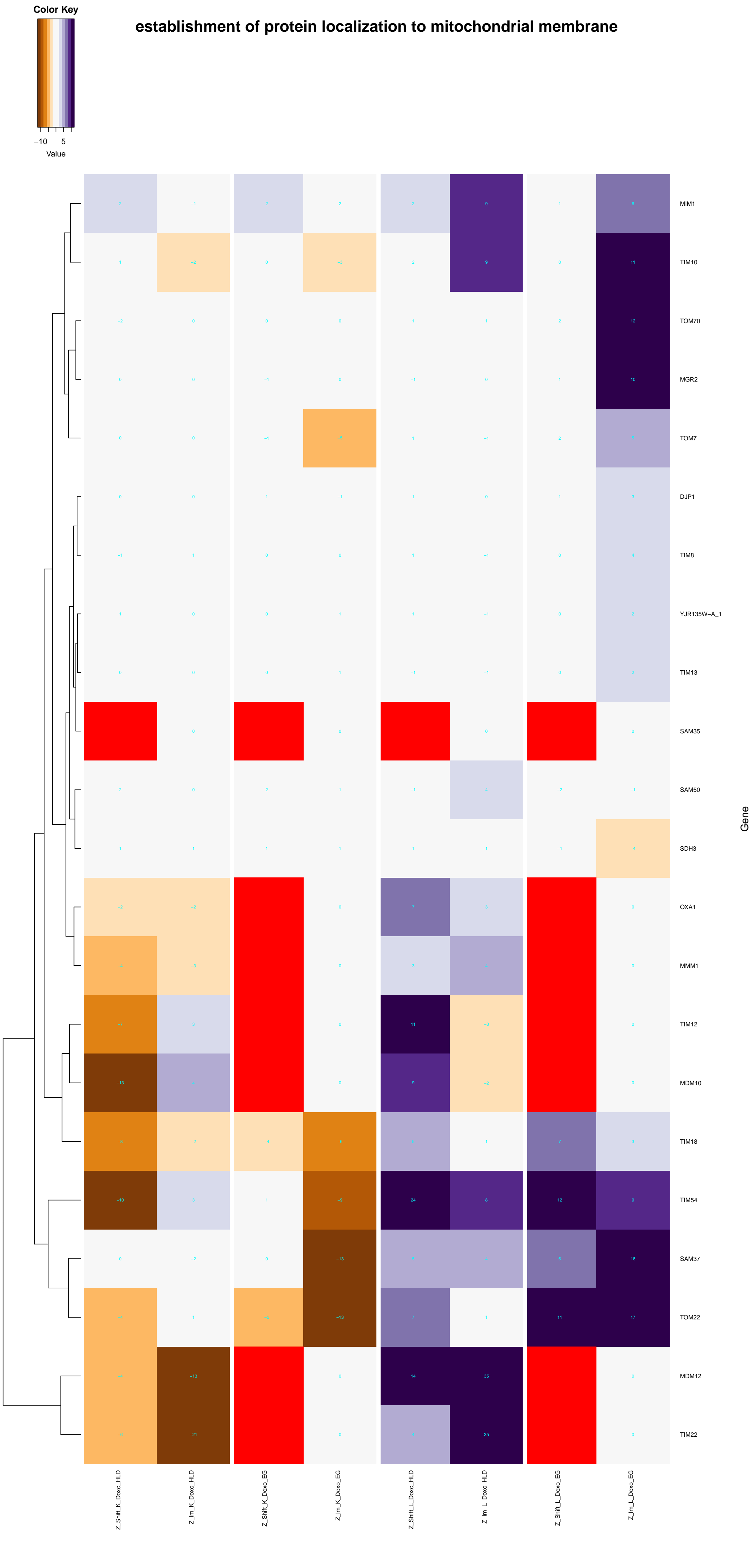

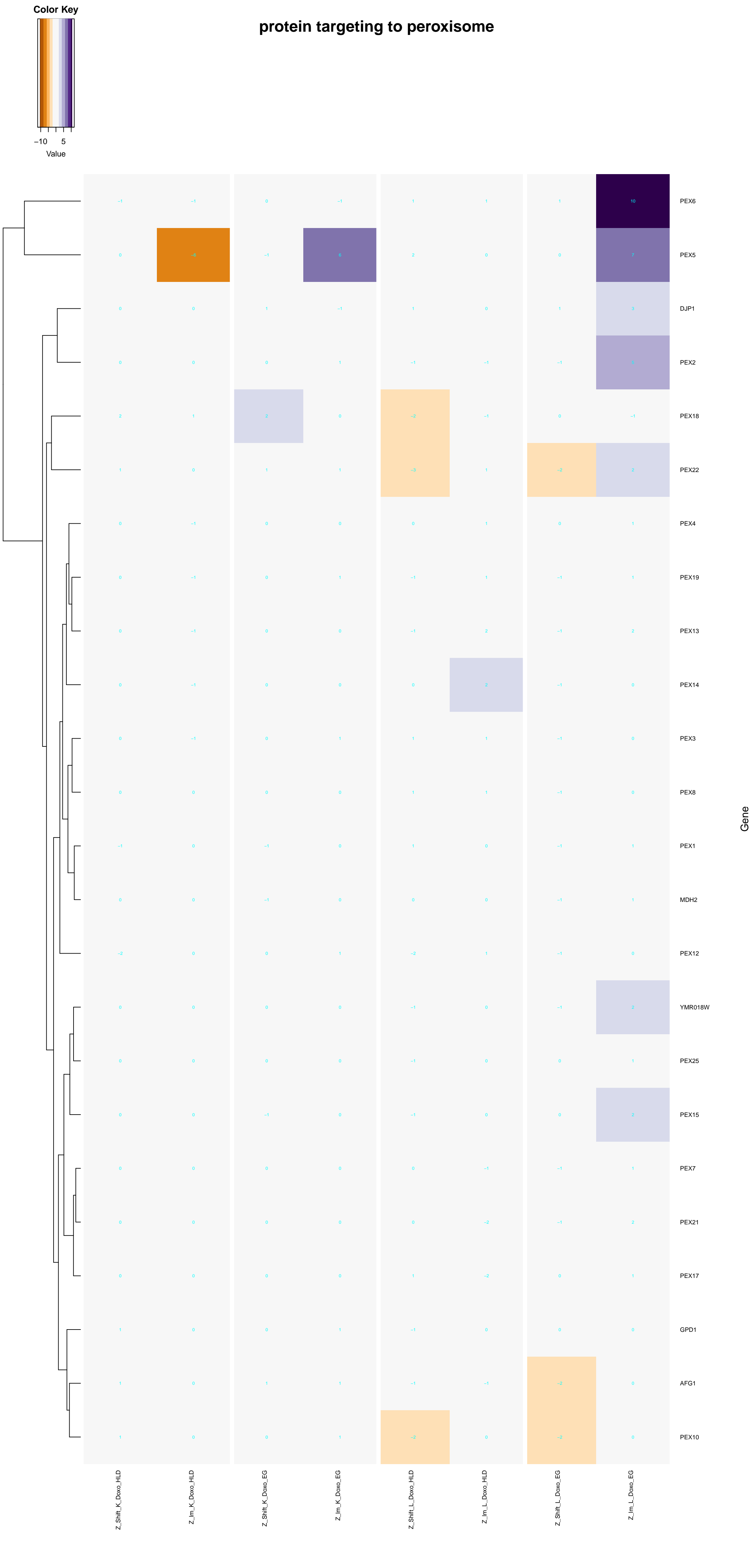

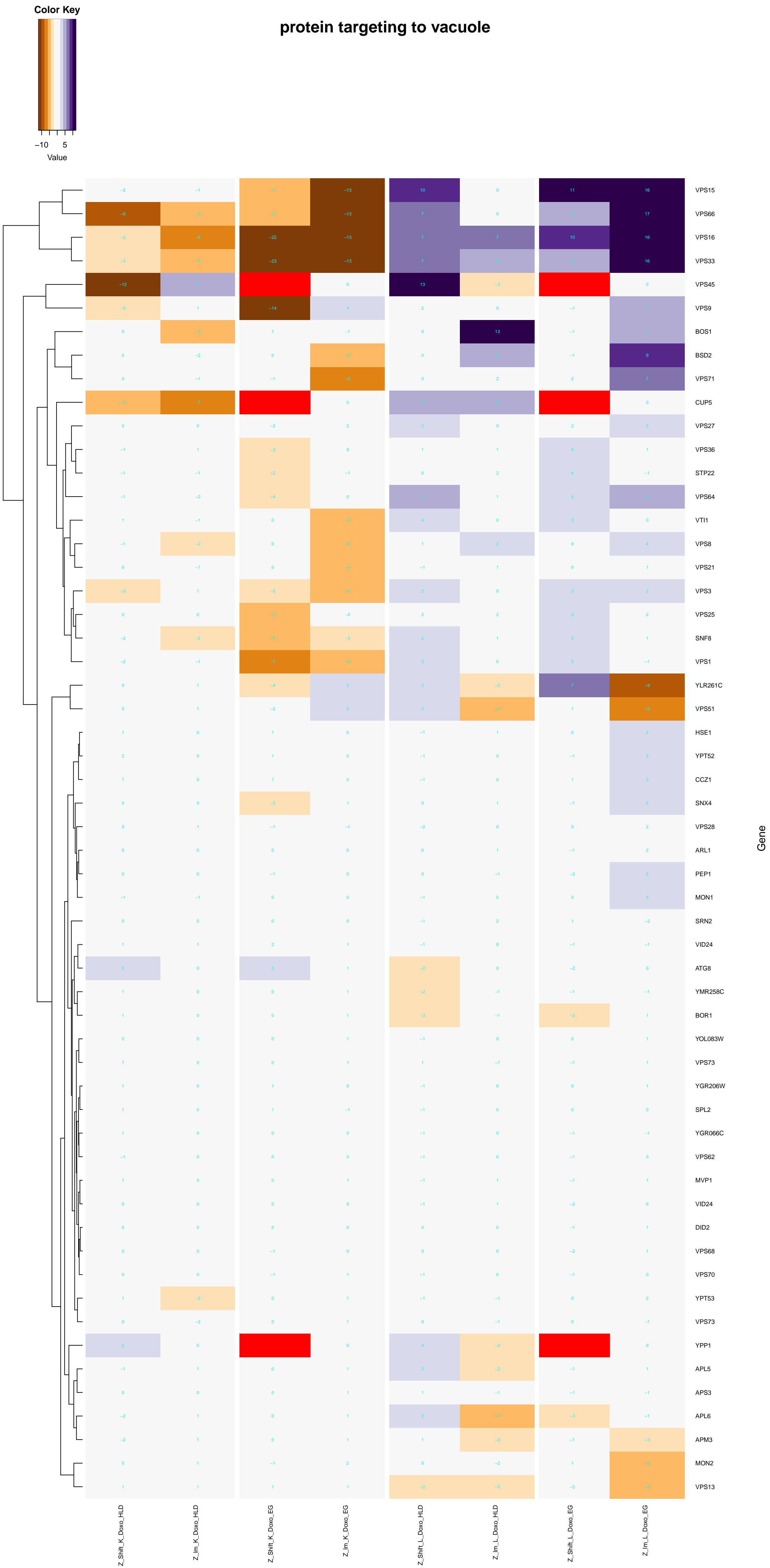

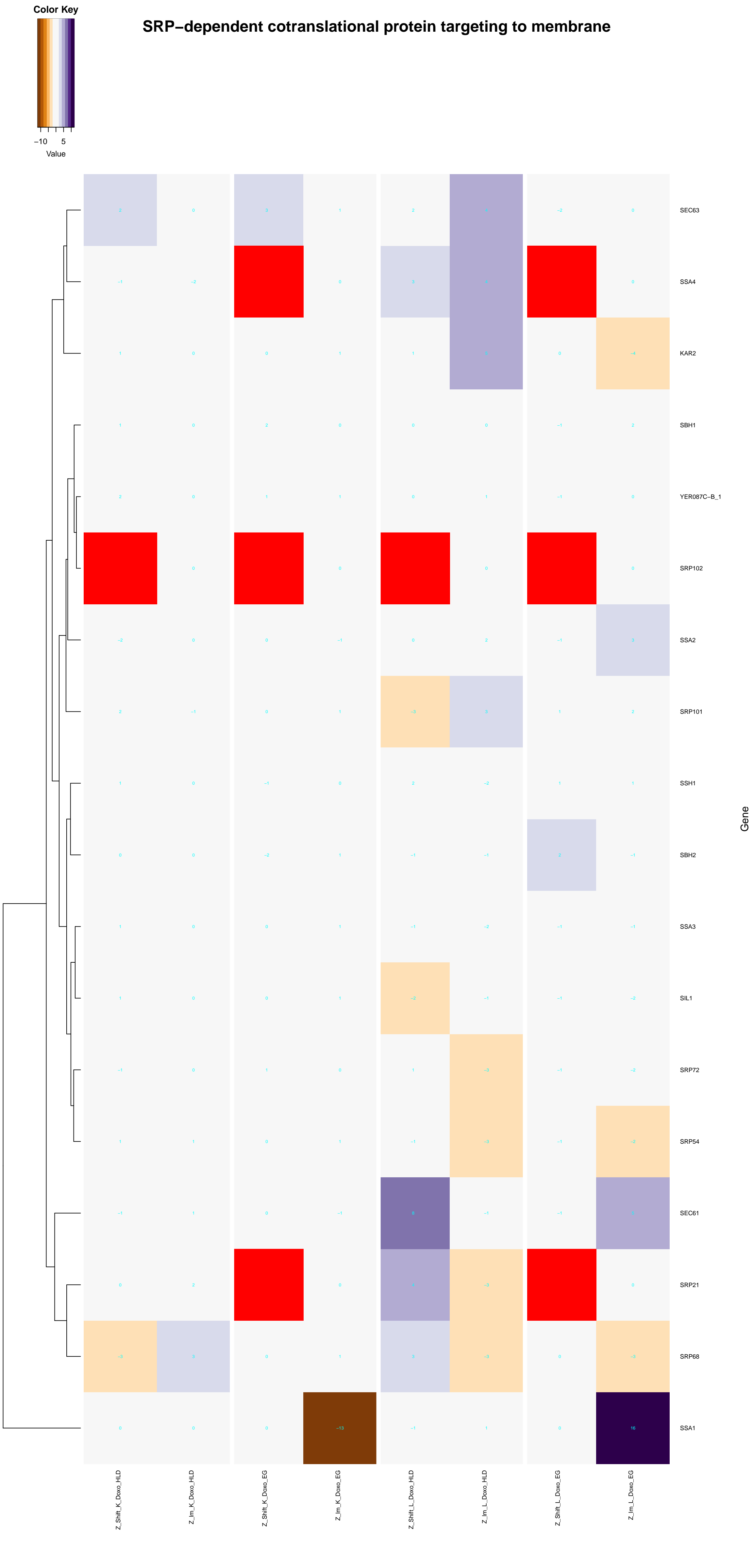

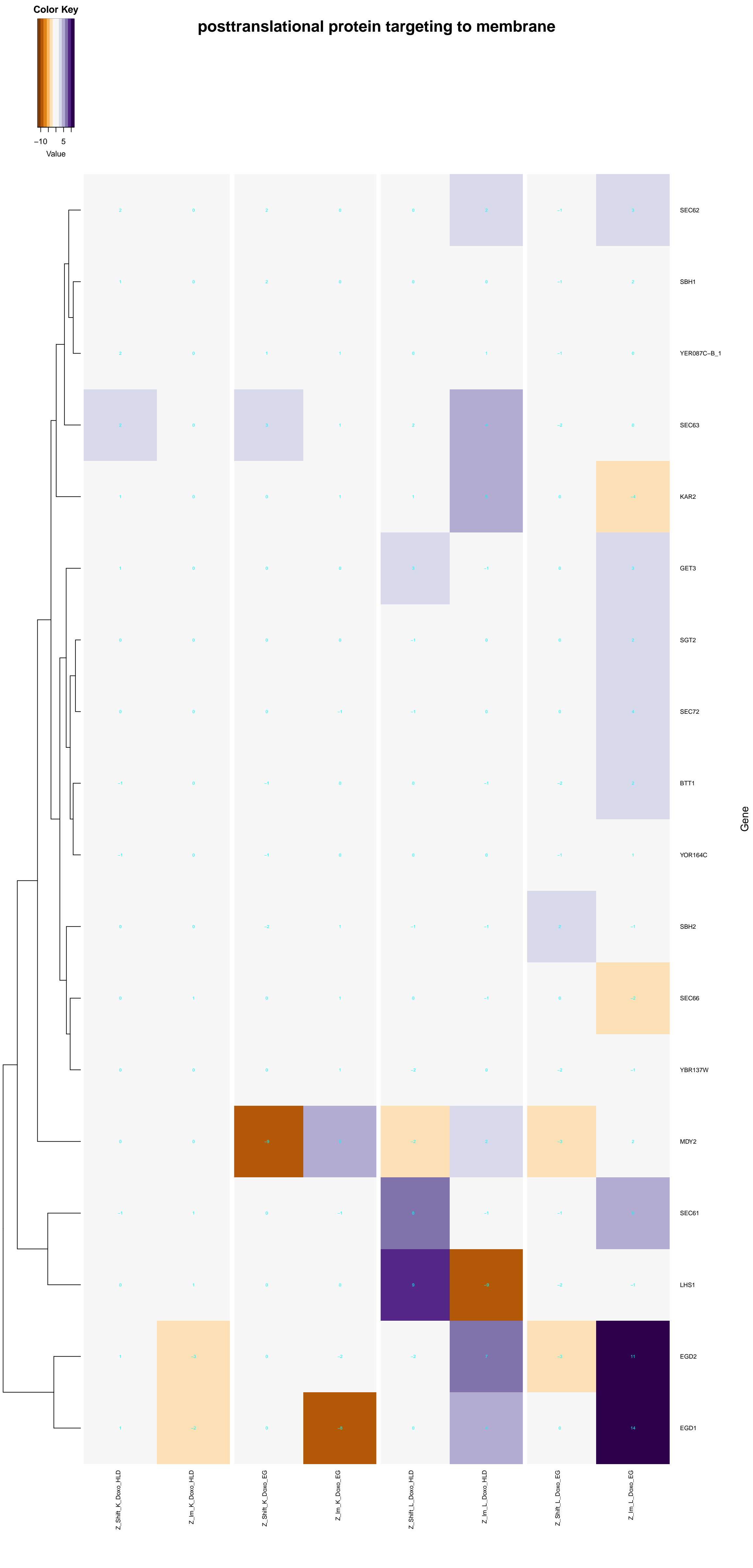

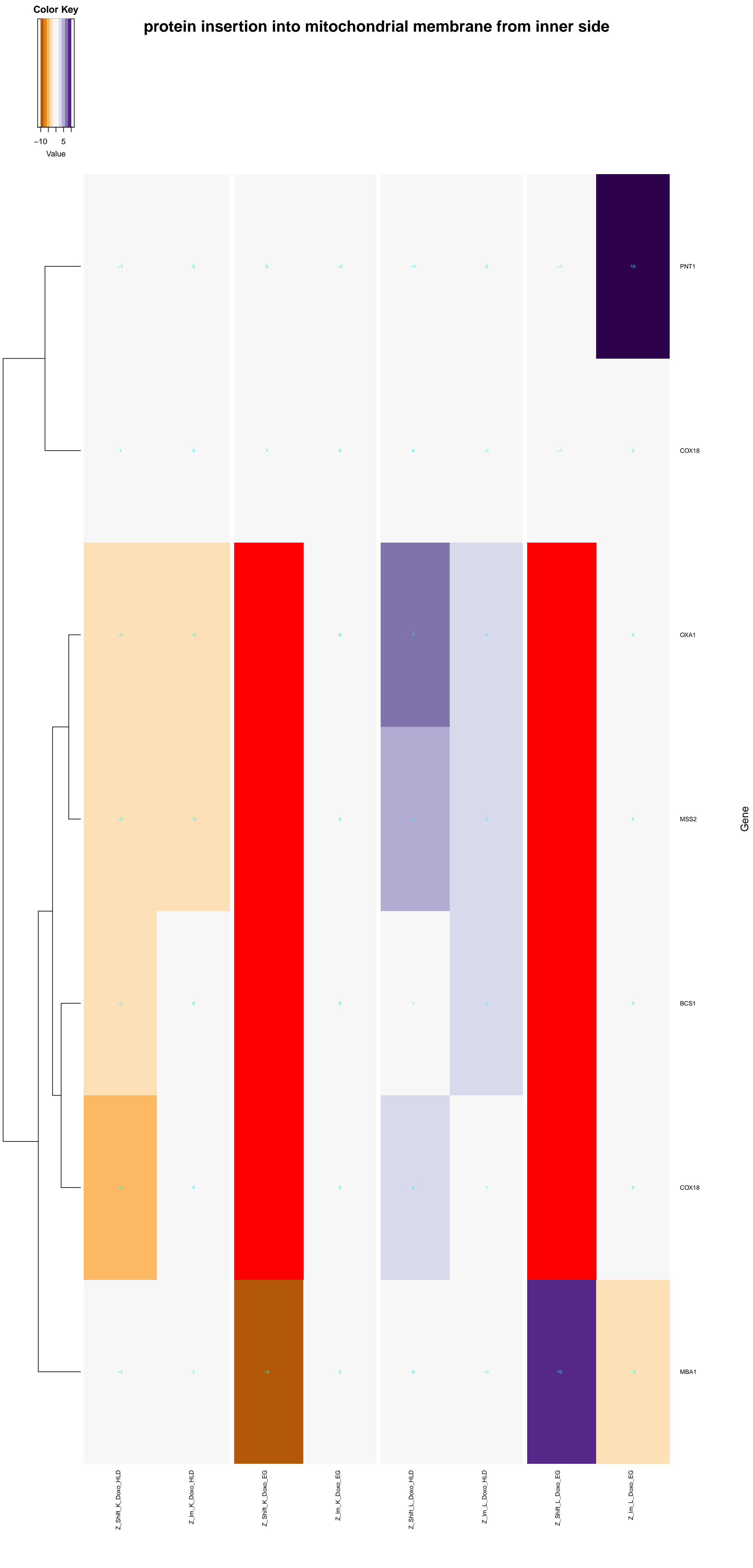



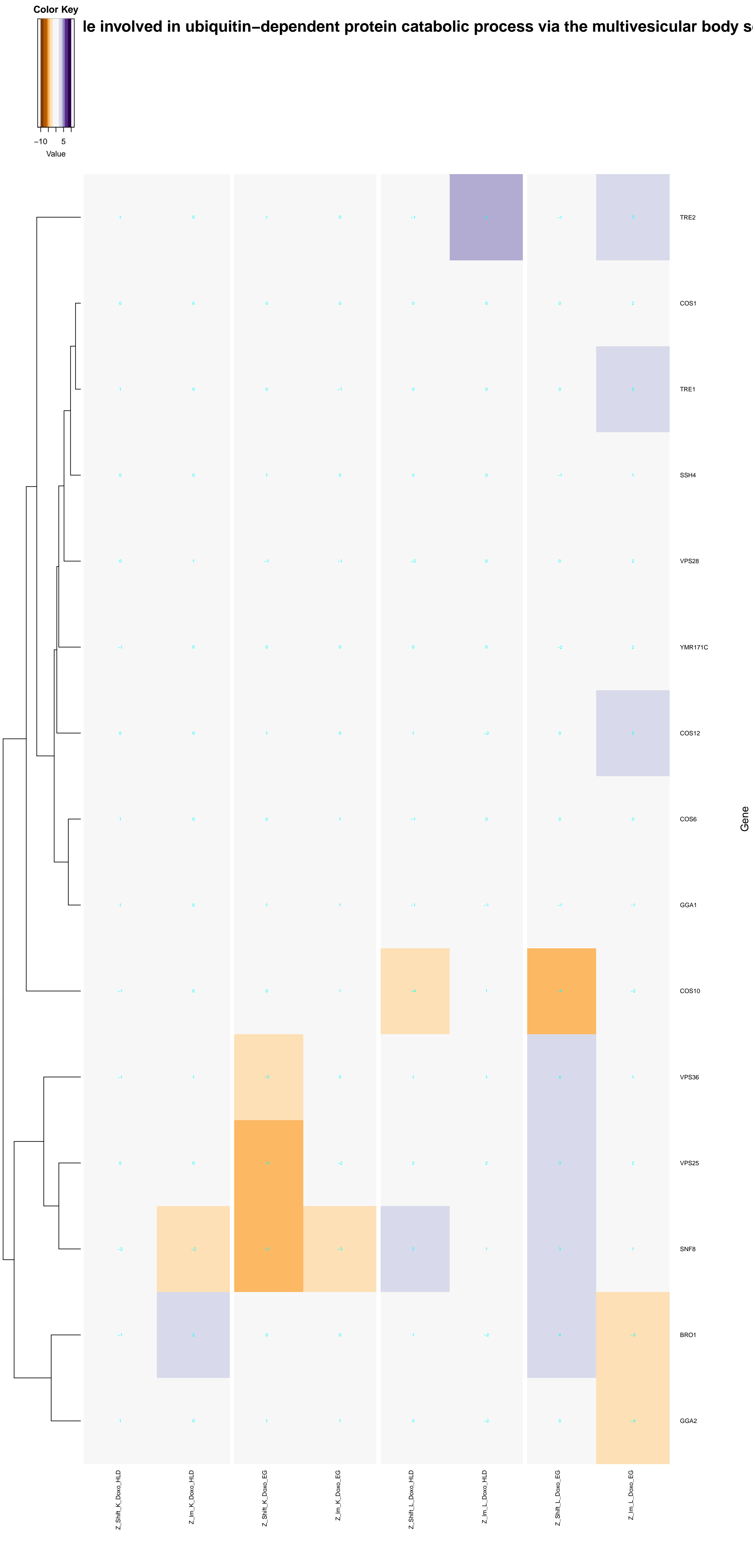

Supplement: Supplementary file 9 — Additional file 9. GO term-specific heatmaps for REMc/GTF-enriched clusters. GO term-specific heatmaps for significant GO process terms were generated as described in methods and Figs. 3 and 4. Any related child terms are presented in subsequent pages of the parent file name. GO terms with more than 100 children, with 2 or fewer genes annotated to the term, or a file size over 300KB are not shown. All heatmaps are generated with the same layout (see Figs. 3 and 4). [file 40170_2019_201_MOESM9_ESM.bz2 › Additional_File9_GOTermHeatmaps/Additional_File9_GOTermHeatmap/establishment_of_protein_localization_to_organelle.pdf]

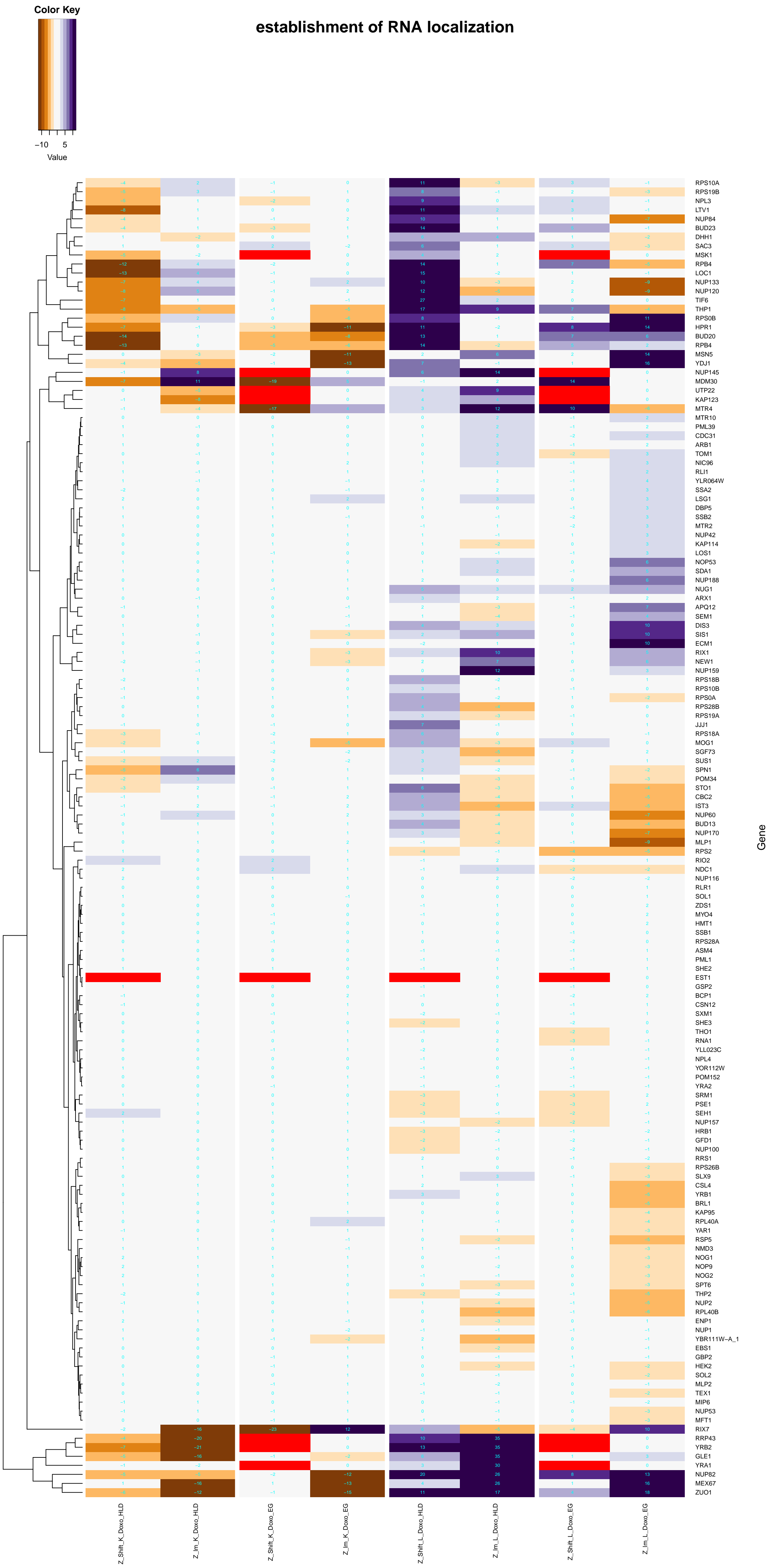



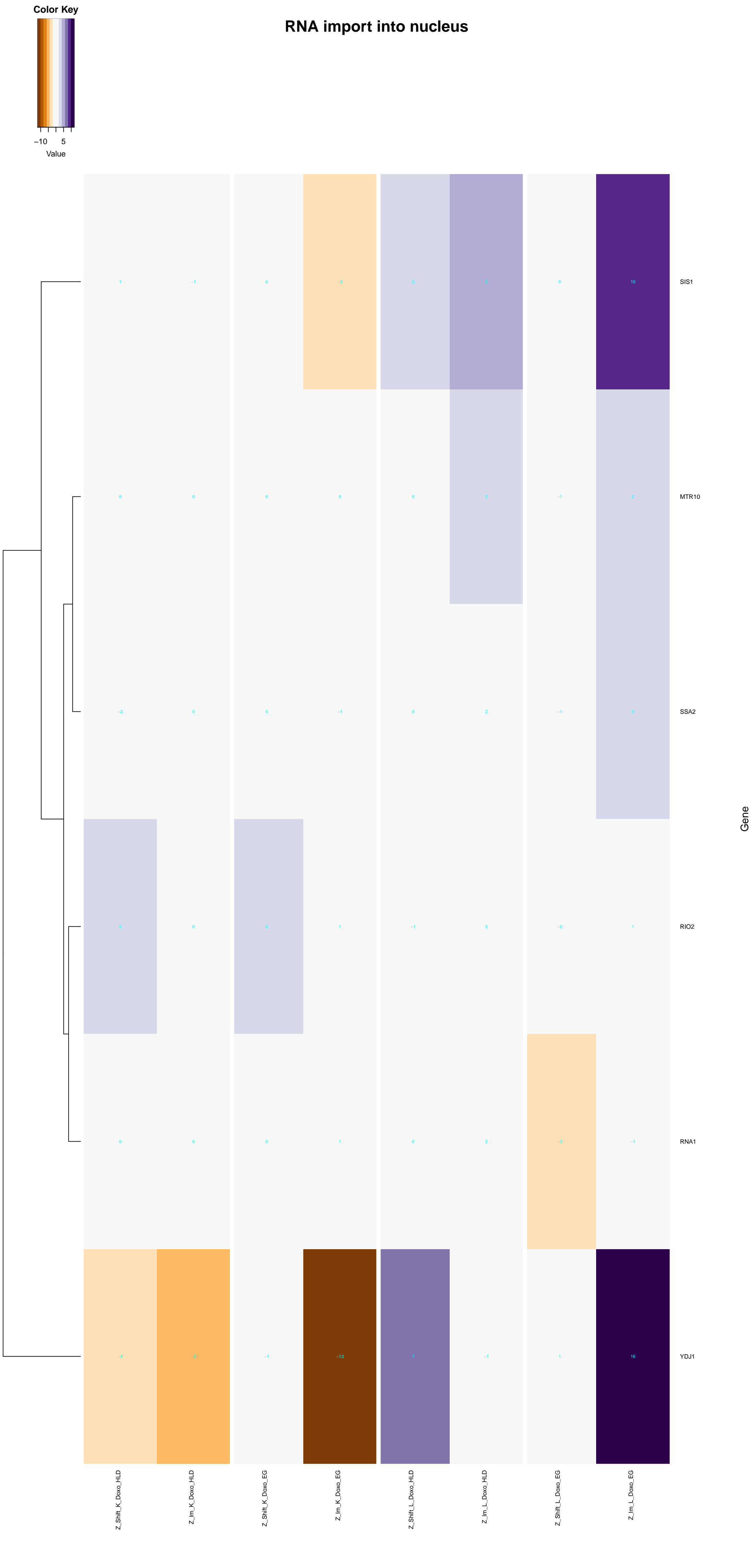

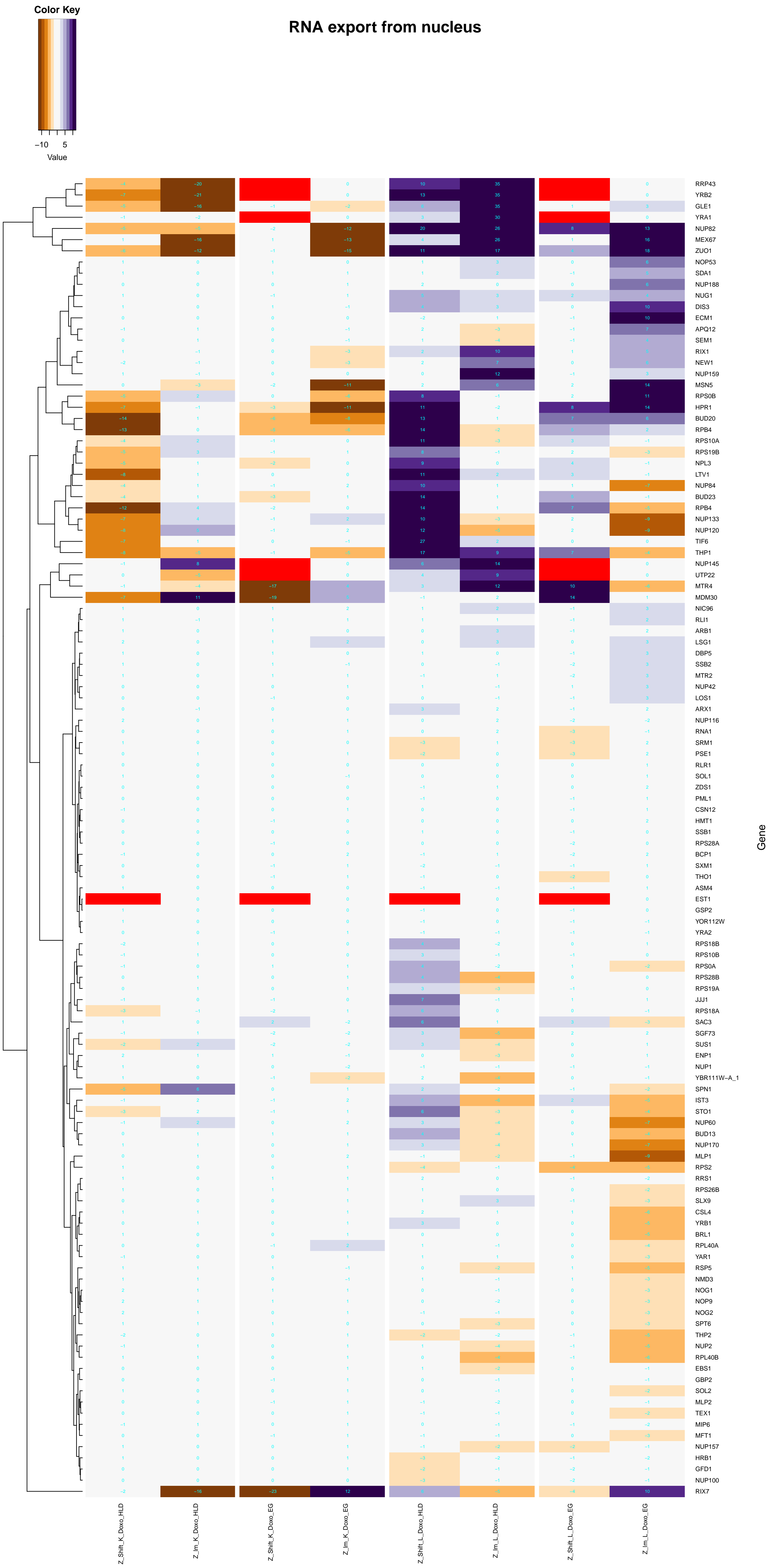



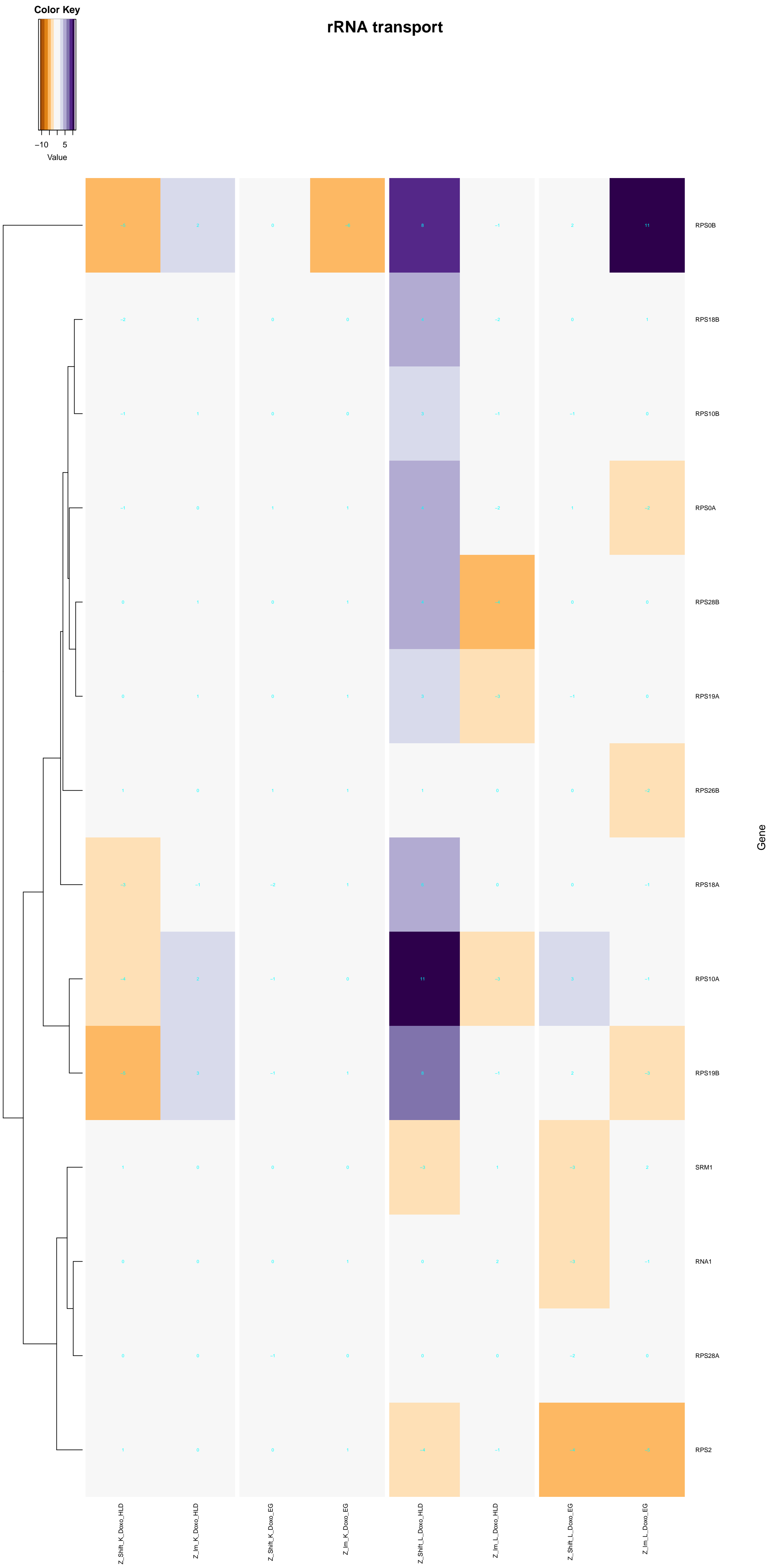

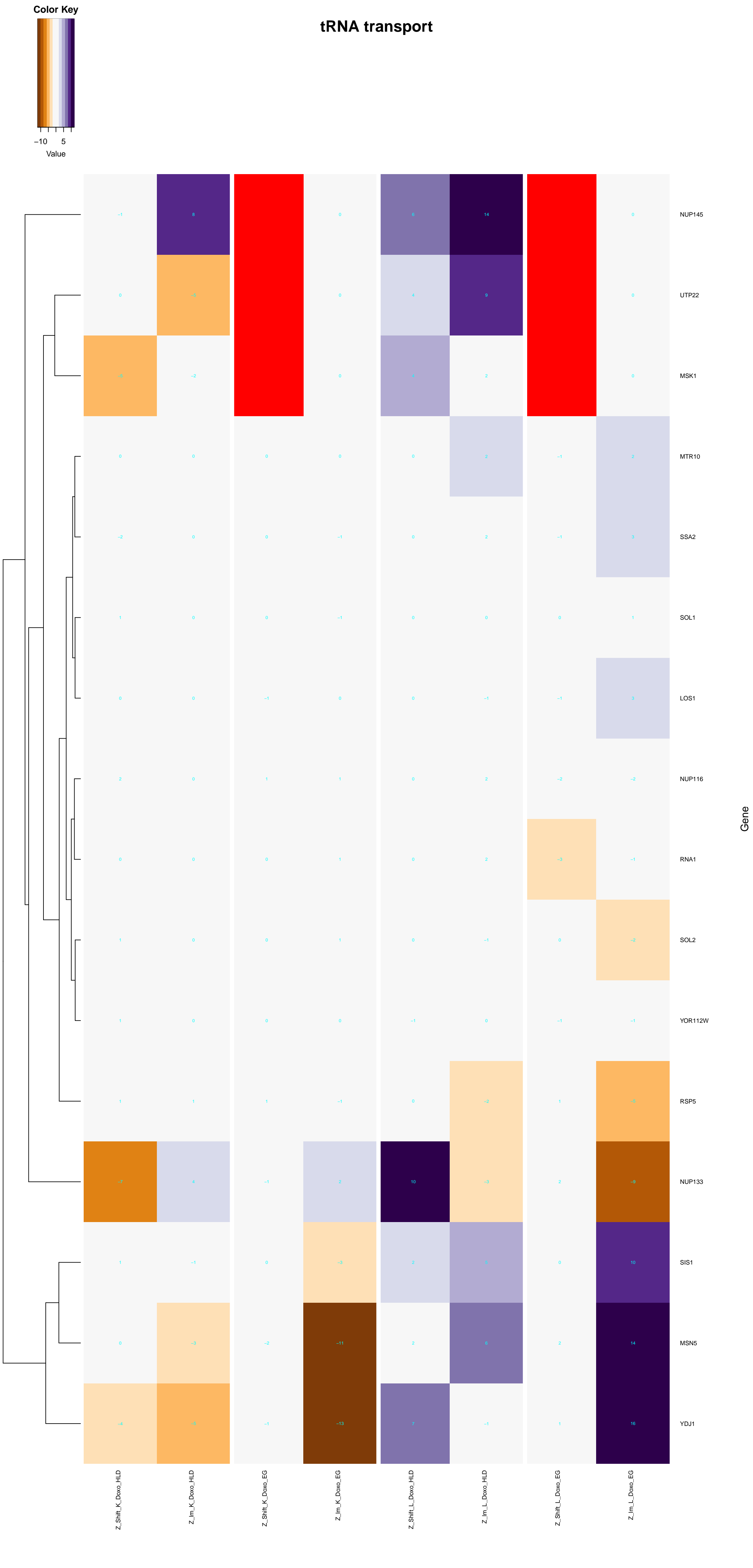

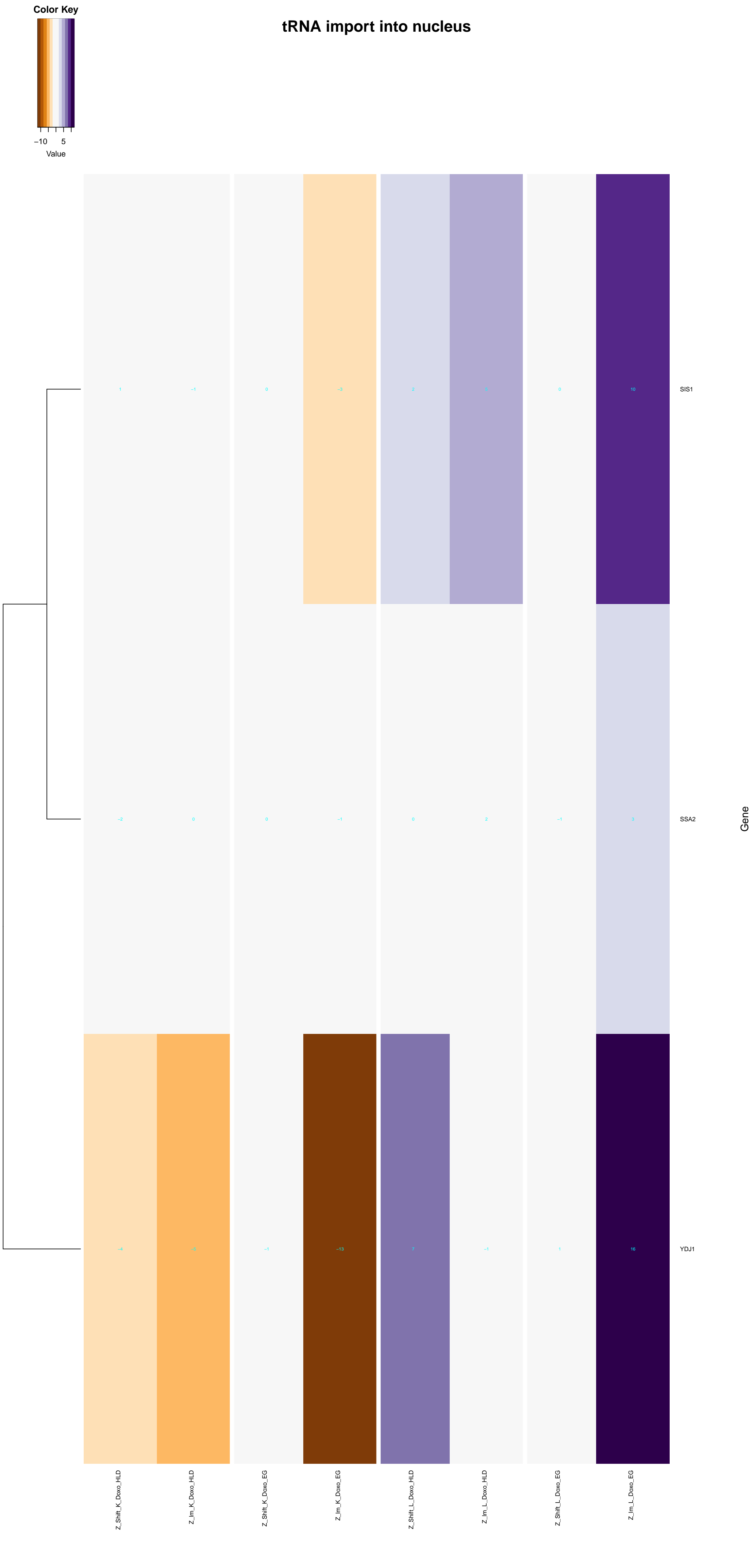

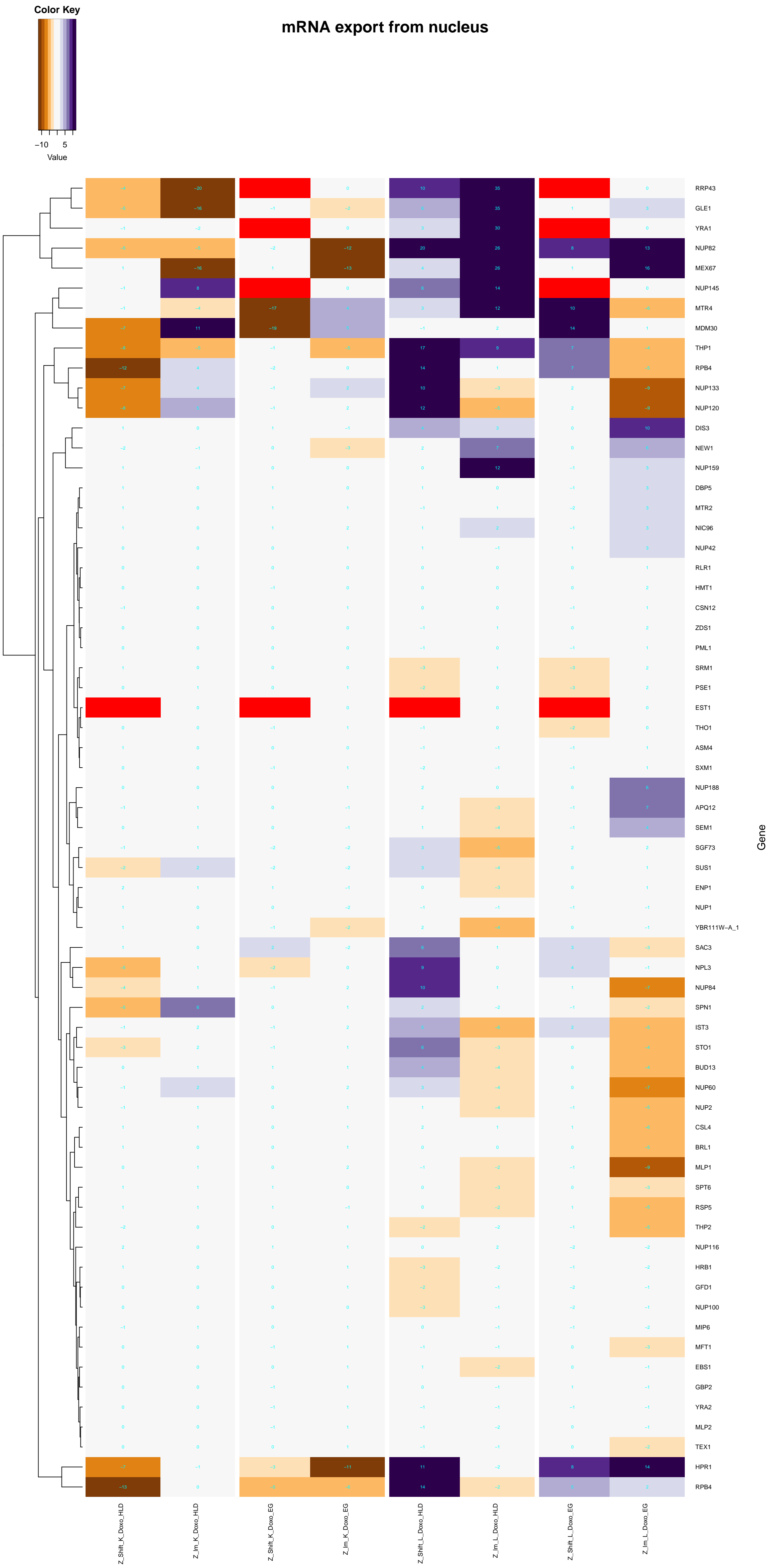

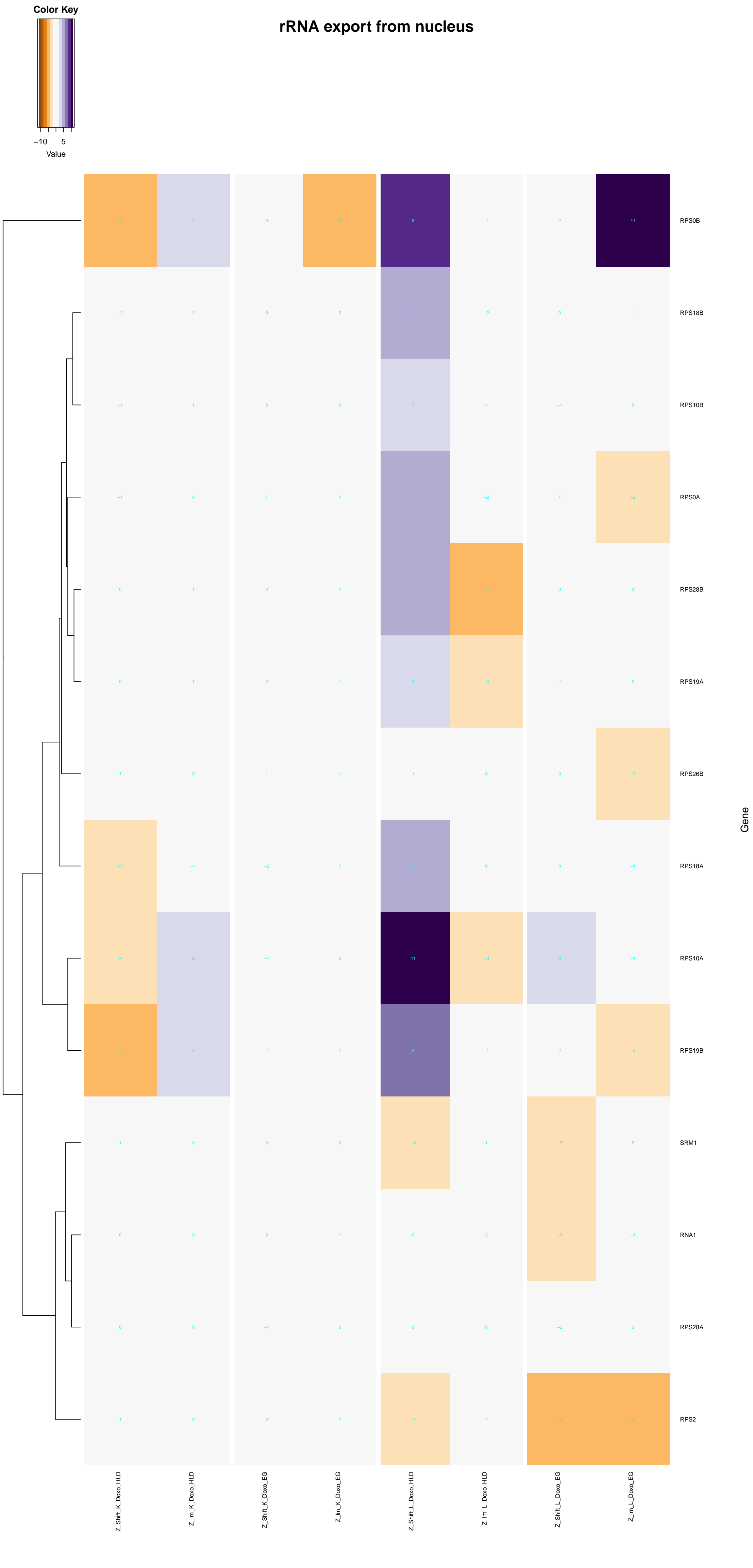

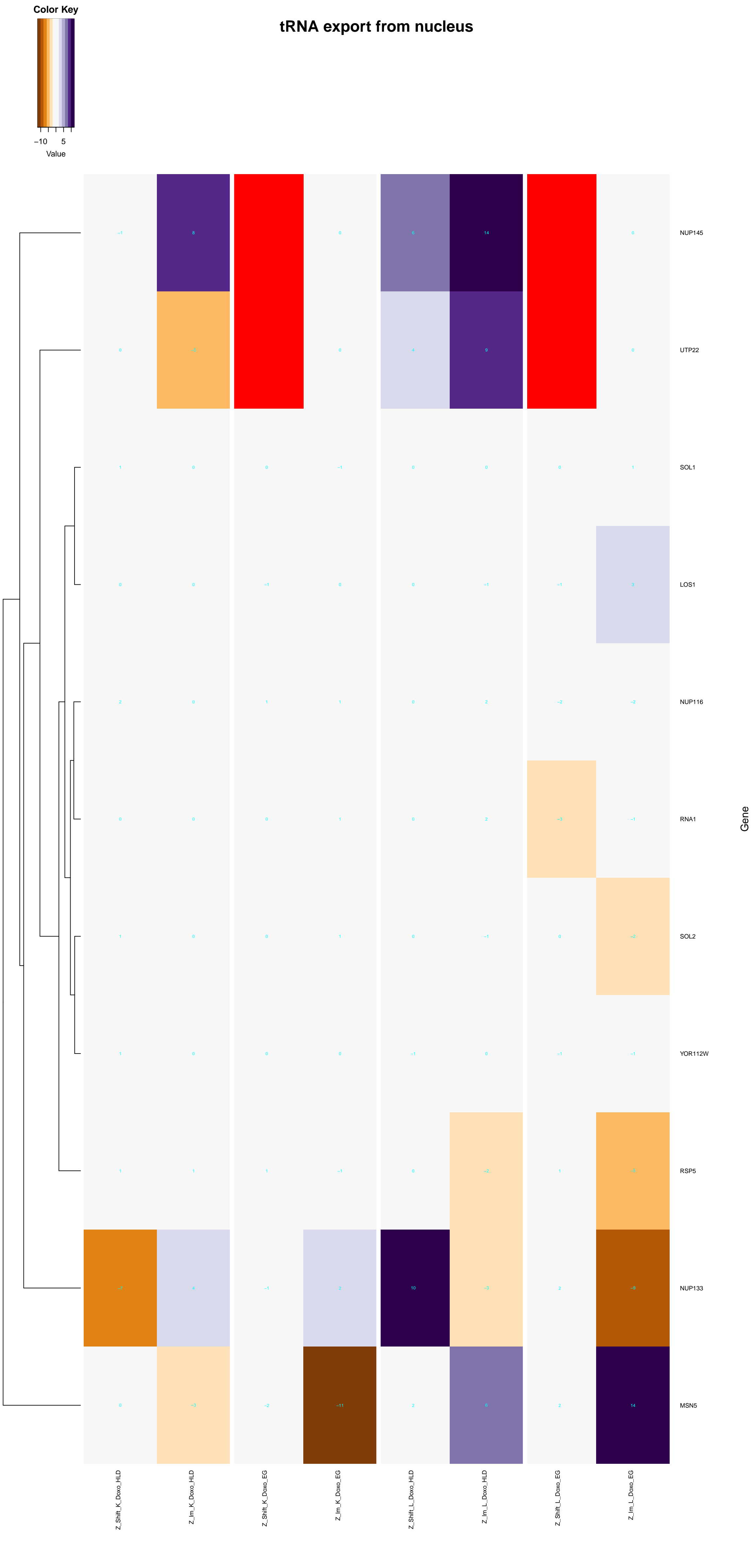

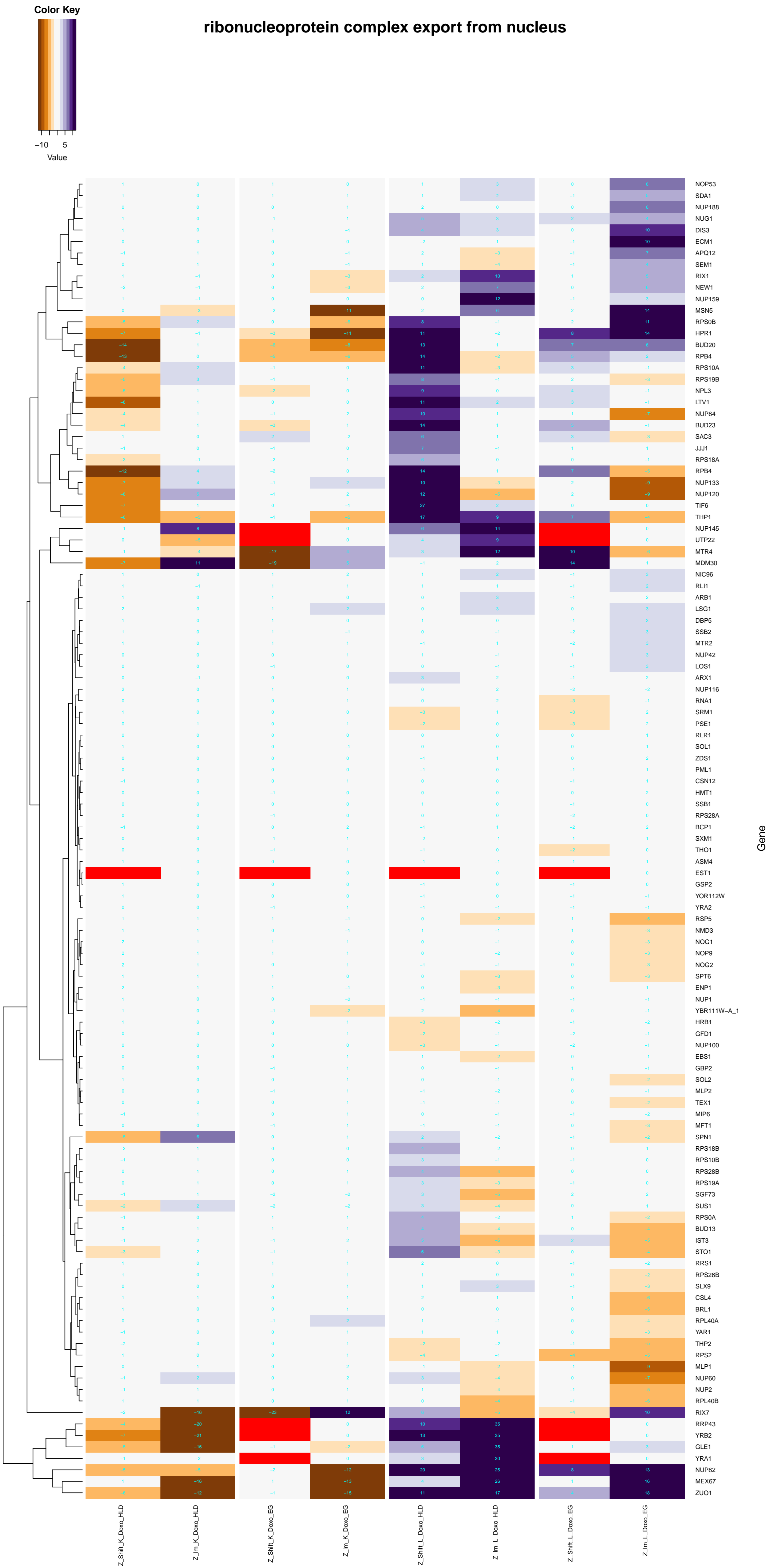

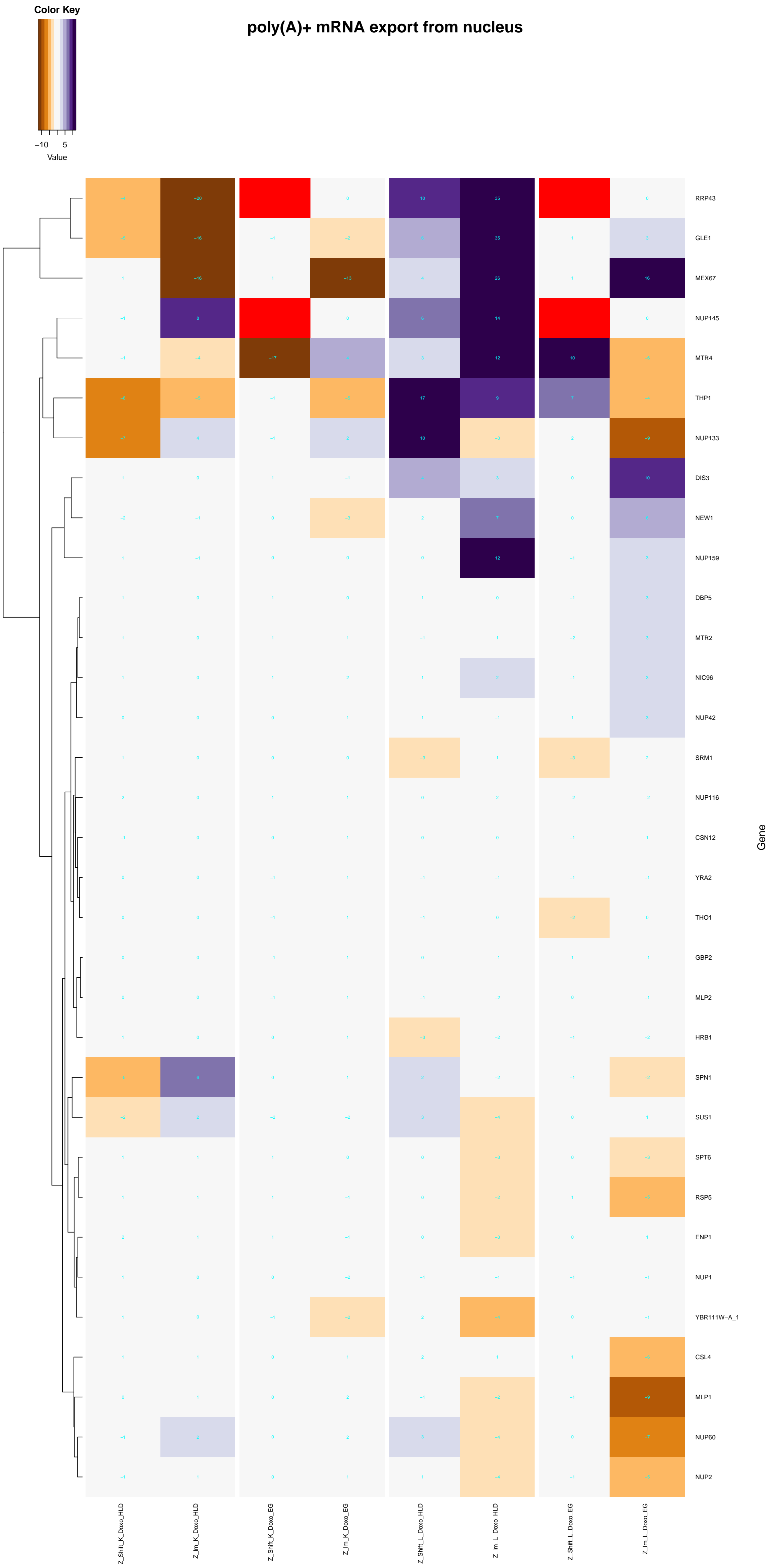

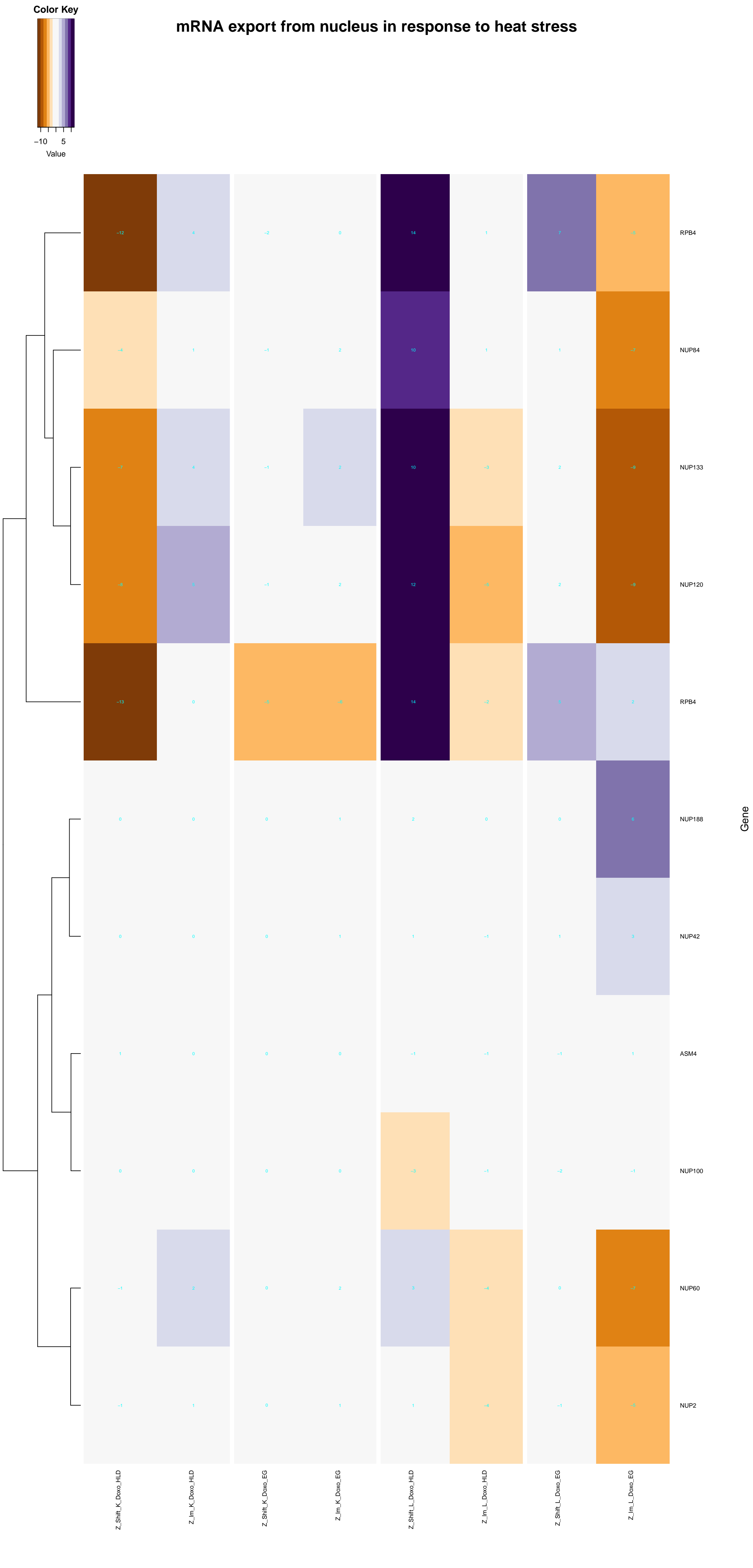

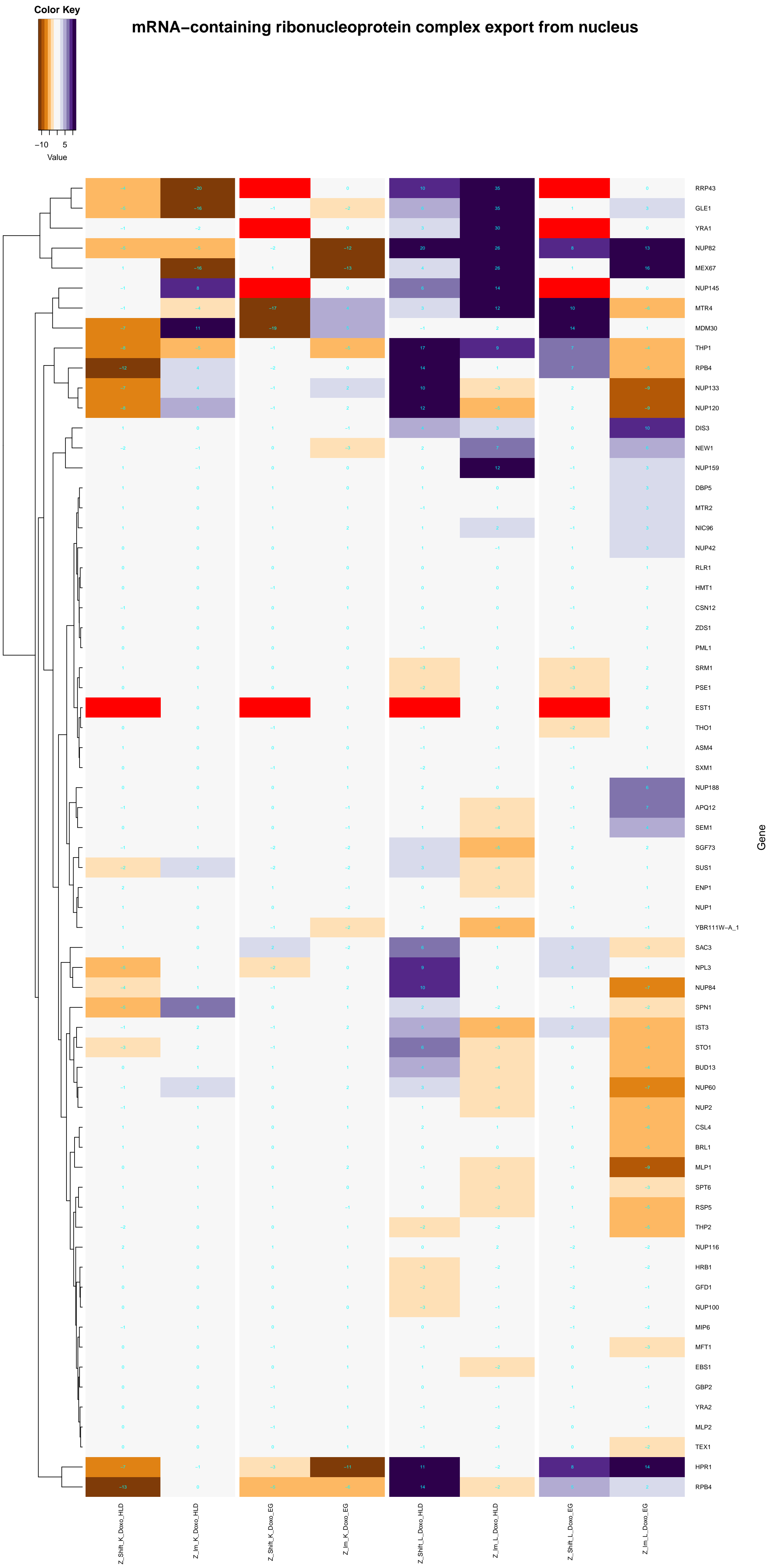

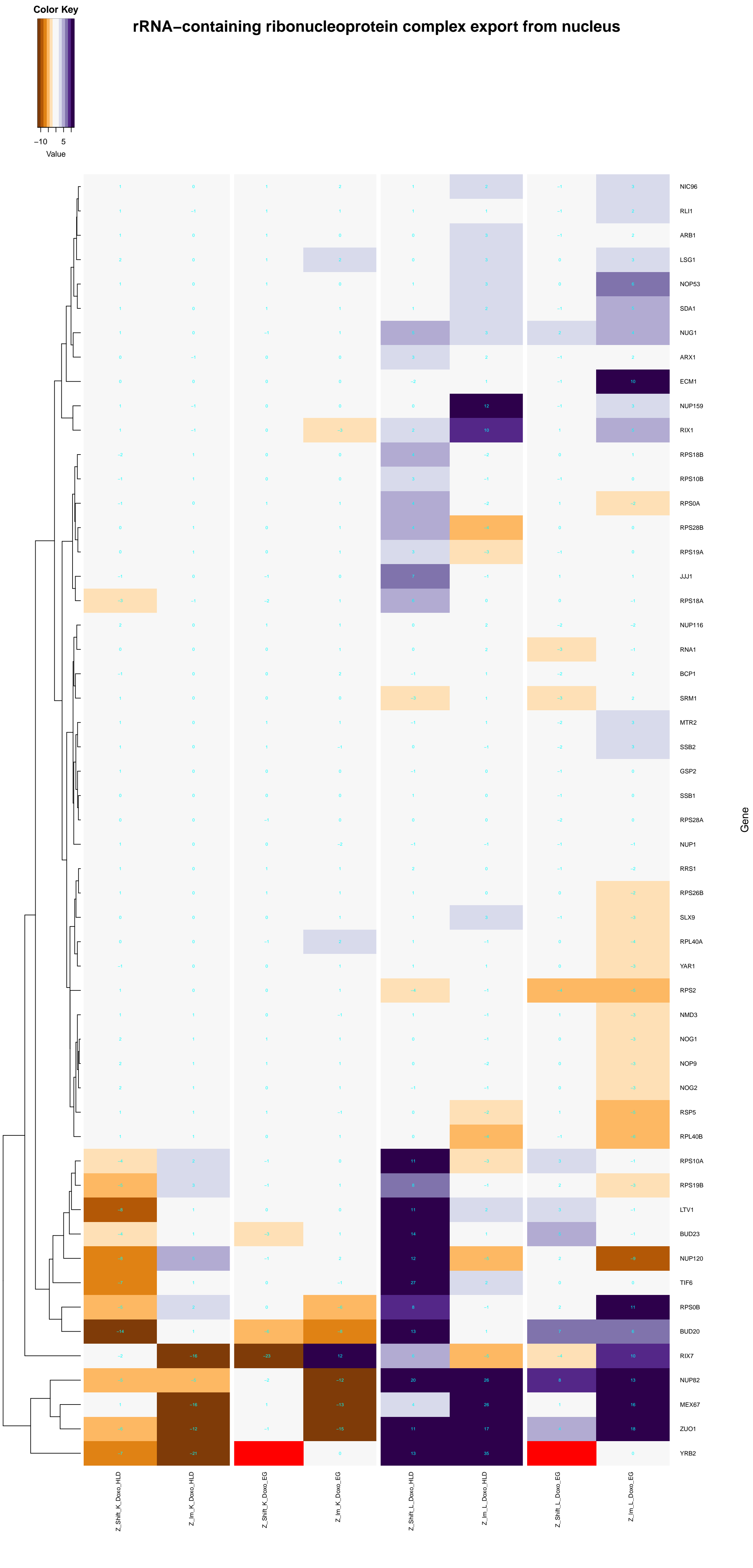

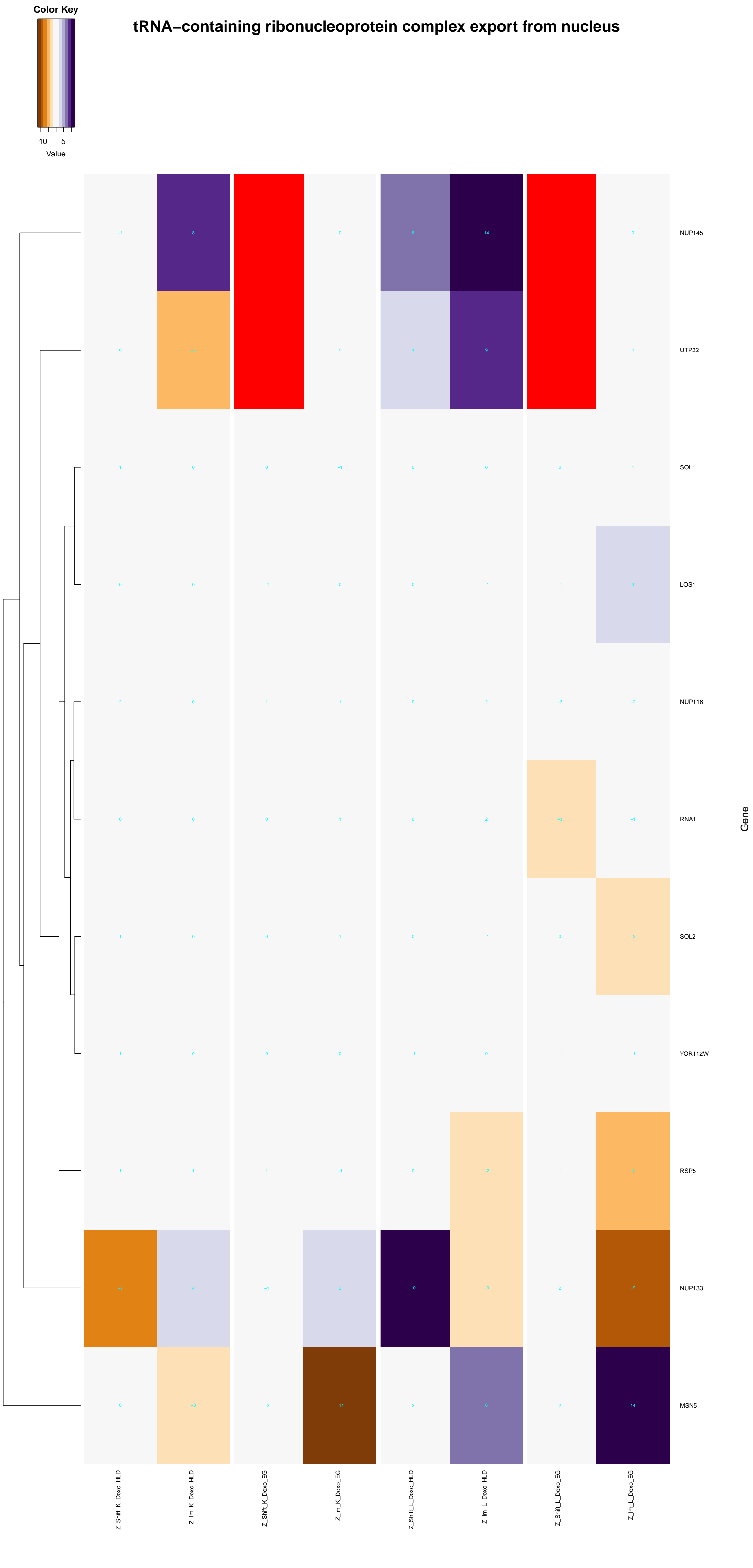

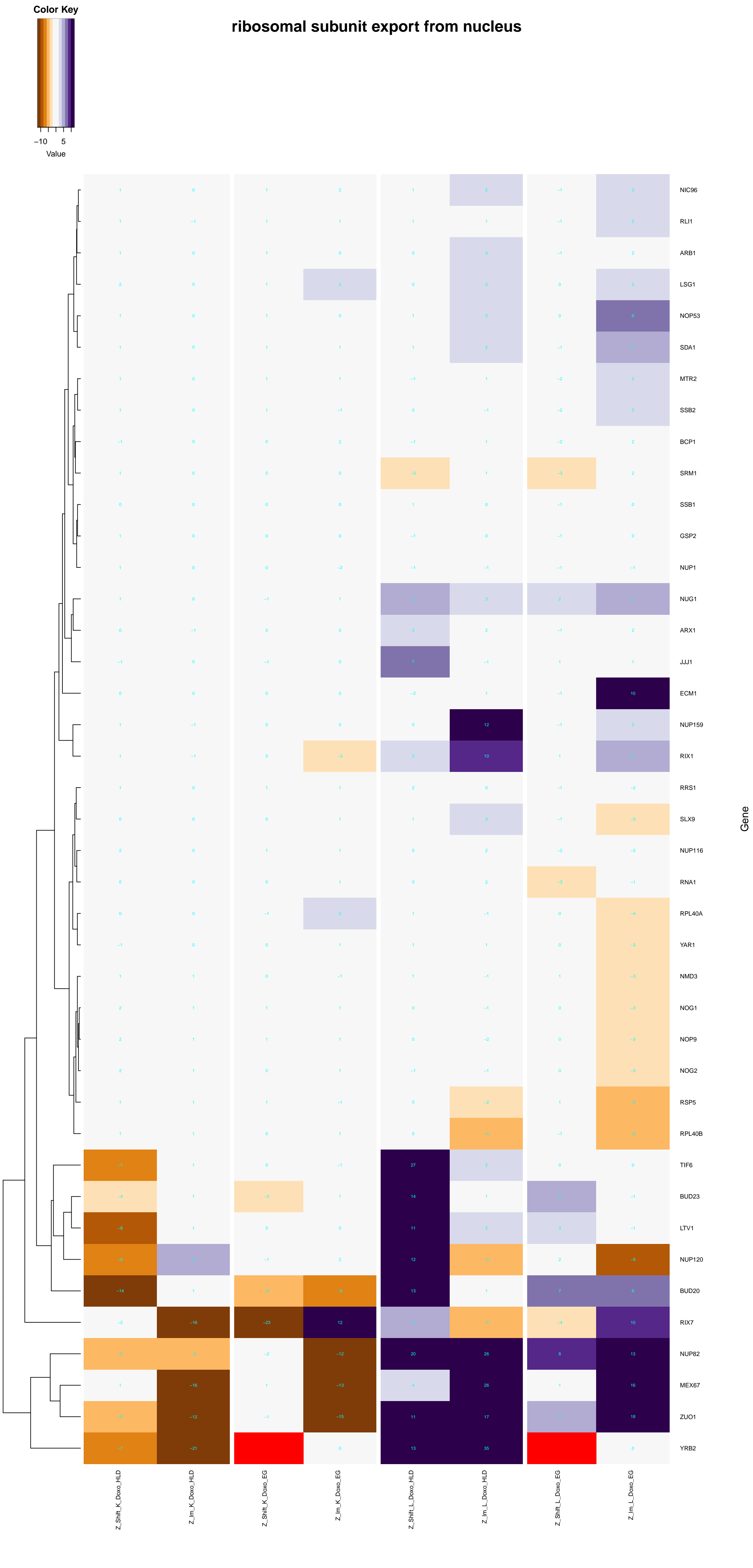

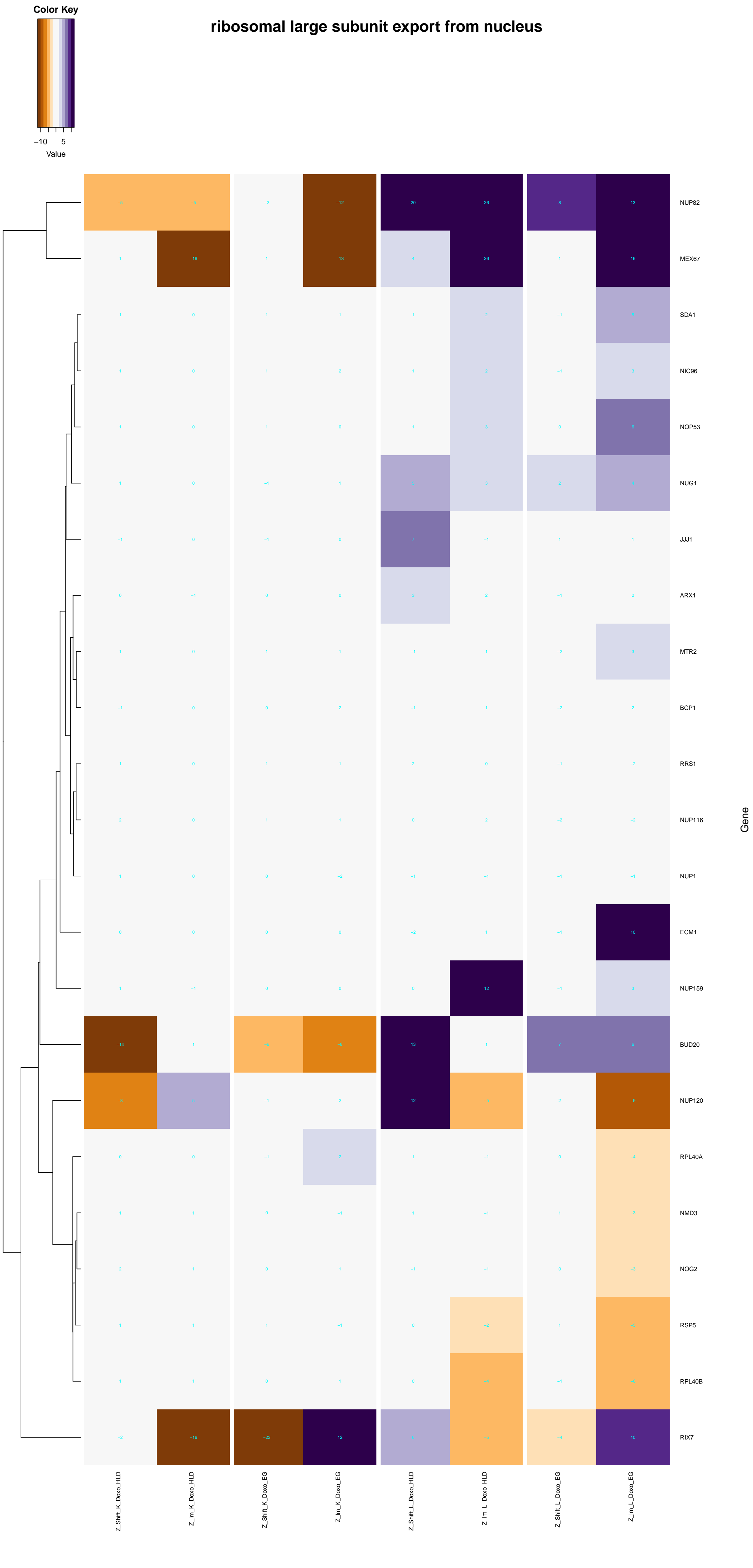

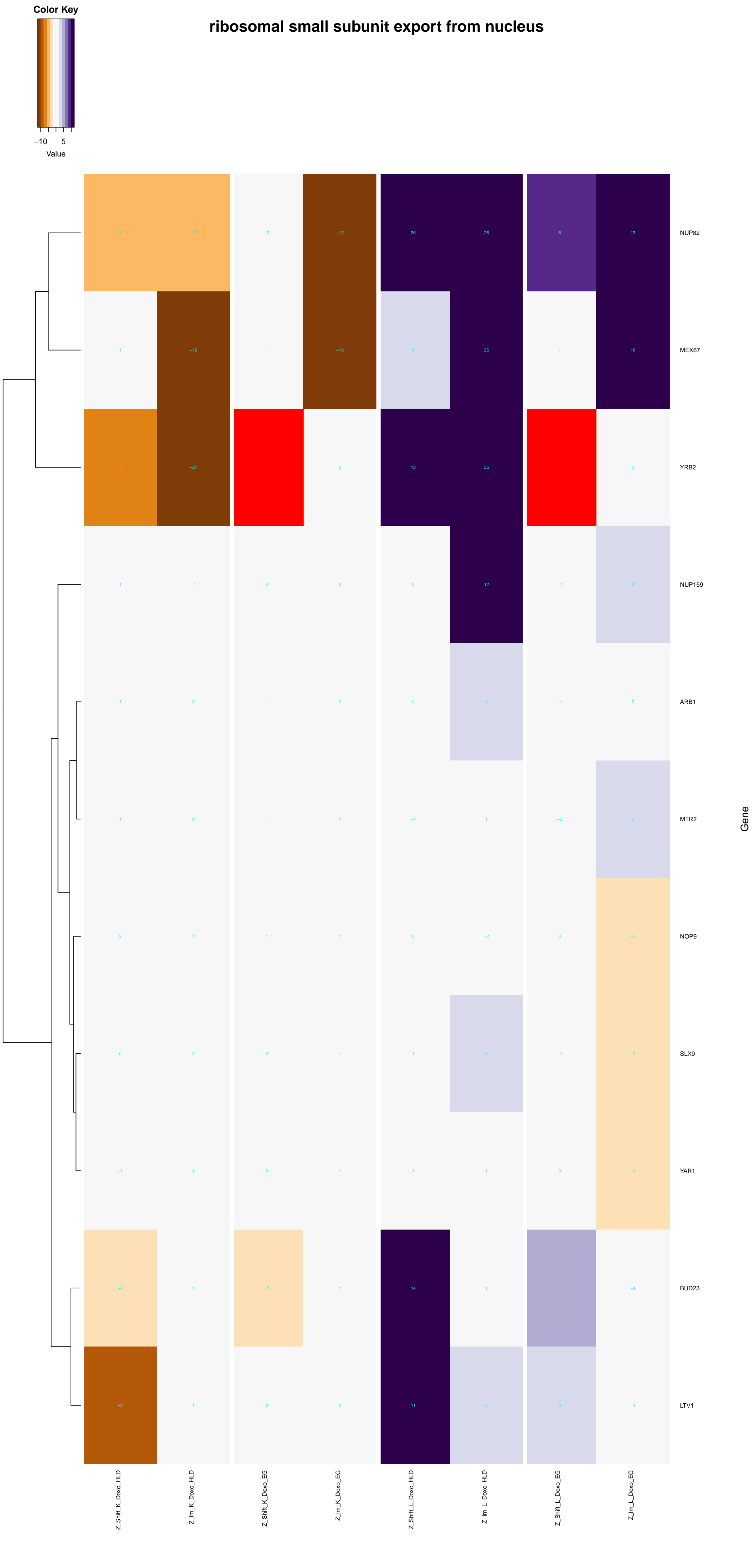

Supplement: Supplementary file 9 — Additional file 9. GO term-specific heatmaps for REMc/GTF-enriched clusters. GO term-specific heatmaps for significant GO process terms were generated as described in methods and Figs. 3 and 4. Any related child terms are presented in subsequent pages of the parent file name. GO terms with more than 100 children, with 2 or fewer genes annotated to the term, or a file size over 300KB are not shown. All heatmaps are generated with the same layout (see Figs. 3 and 4). [file 40170_2019_201_MOESM9_ESM.bz2 › Additional_File9_GOTermHeatmaps/Additional_File9_GOTermHeatmap/establishment_of_RNA_localization.pdf]

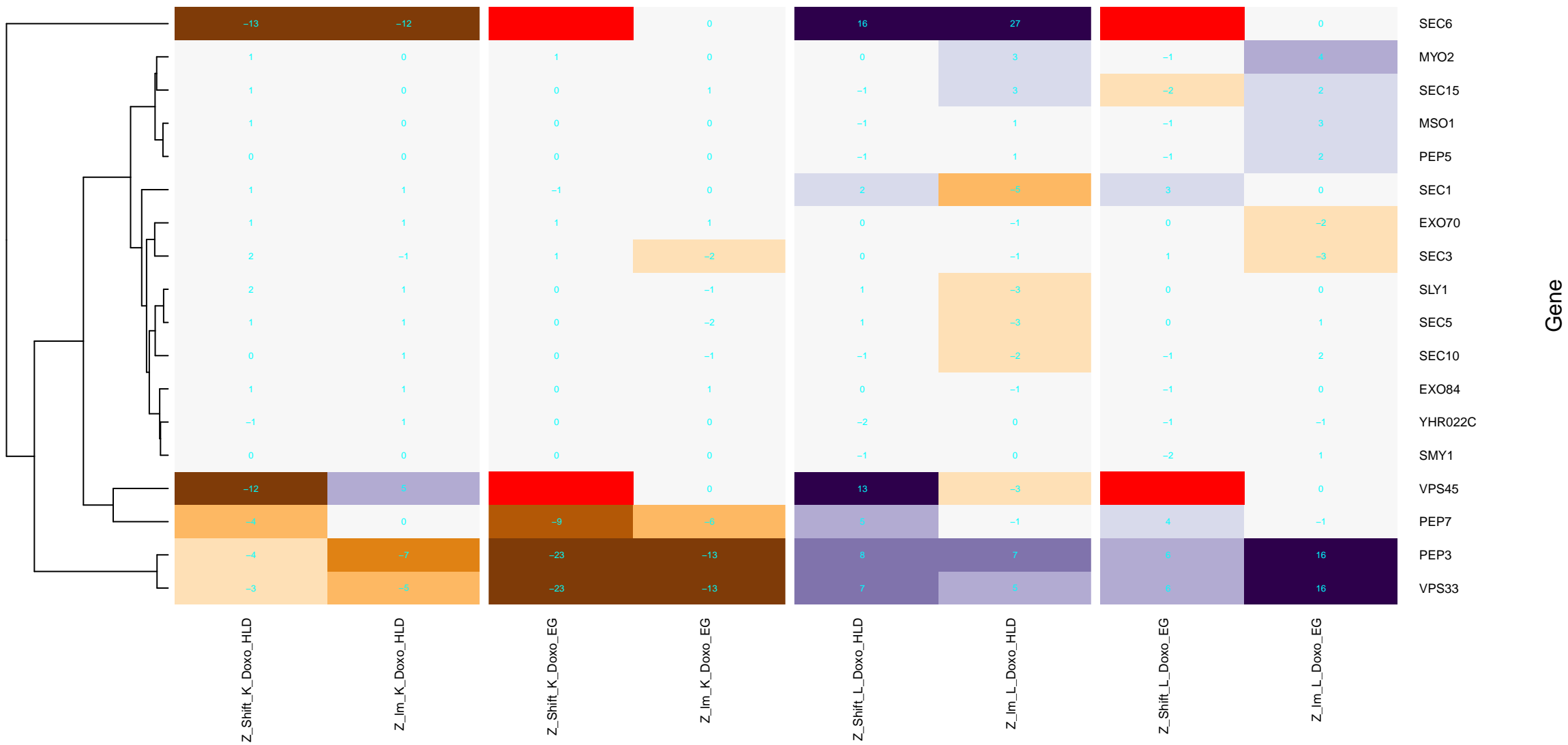

Supplement: Supplementary file 9 — Additional file 9. GO term-specific heatmaps for REMc/GTF-enriched clusters. GO term-specific heatmaps for significant GO process terms were generated as described in methods and Figs. 3 and 4. Any related child terms are presented in subsequent pages of the parent file name. GO terms with more than 100 children, with 2 or fewer genes annotated to the term, or a file size over 300KB are not shown. All heatmaps are generated with the same layout (see Figs. 3 and 4). [file 40170_2019_201_MOESM9_ESM.bz2 › Additional_File9_GOTermHeatmaps/Additional_File9_GOTermHeatmap/exocytic_process.pdf]

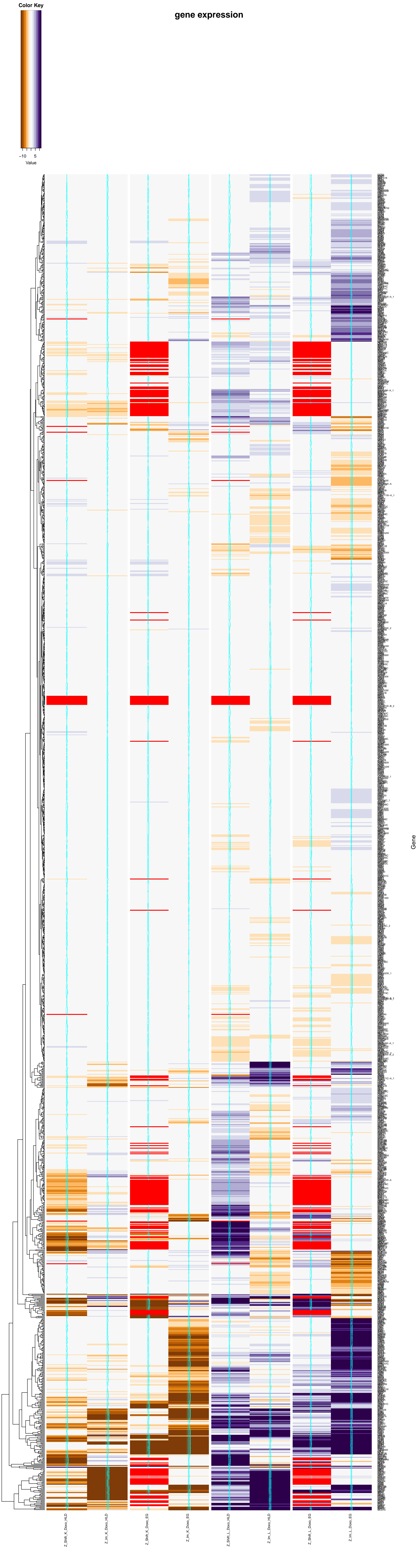

Supplement: Supplementary file 9 — Additional file 9. GO term-specific heatmaps for REMc/GTF-enriched clusters. GO term-specific heatmaps for significant GO process terms were generated as described in methods and Figs. 3 and 4. Any related child terms are presented in subsequent pages of the parent file name. GO terms with more than 100 children, with 2 or fewer genes annotated to the term, or a file size over 300KB are not shown. All heatmaps are generated with the same layout (see Figs. 3 and 4). [file 40170_2019_201_MOESM9_ESM.bz2 › Additional_File9_GOTermHeatmaps/Additional_File9_GOTermHeatmap/gene_expression.pdf]

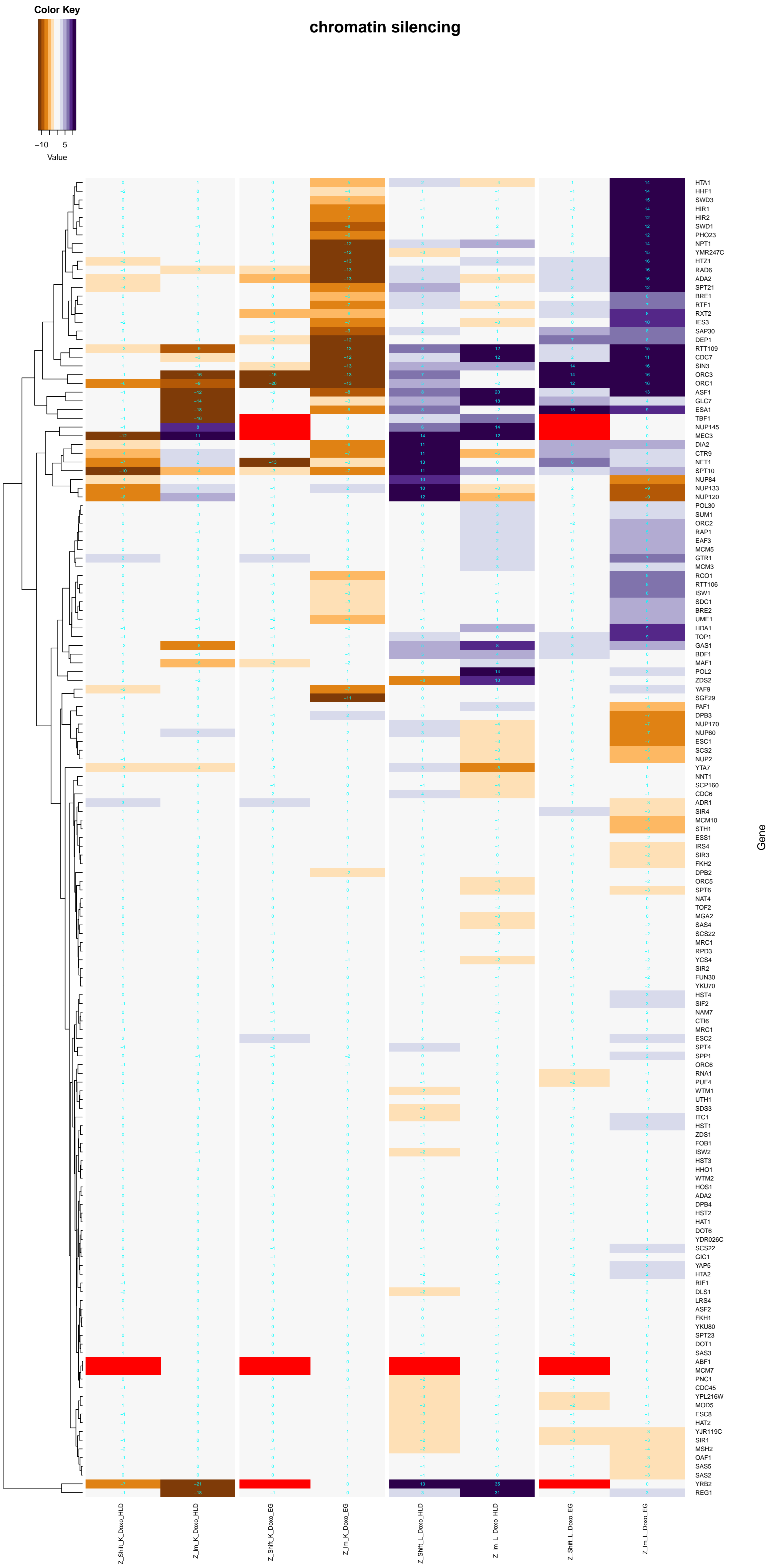

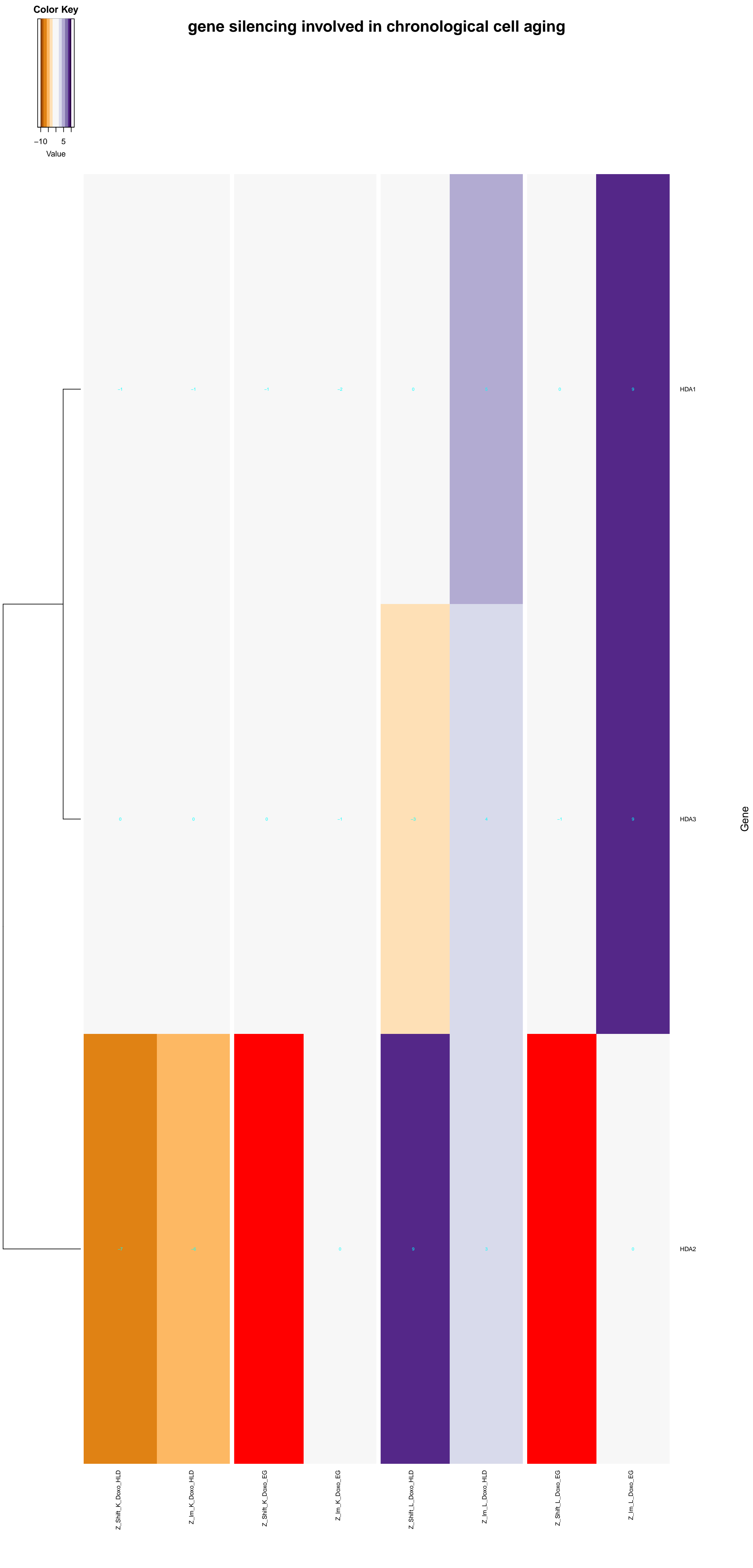

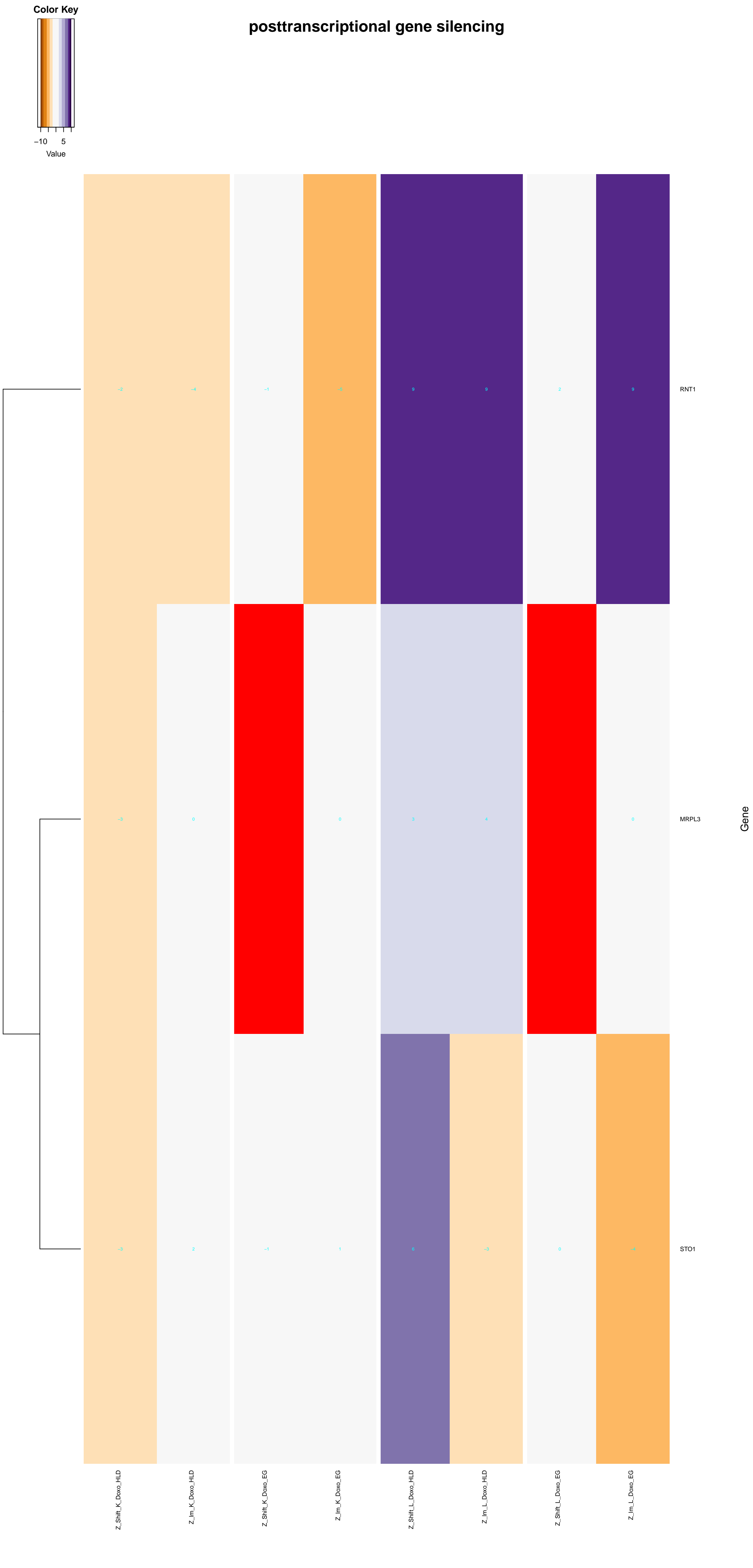

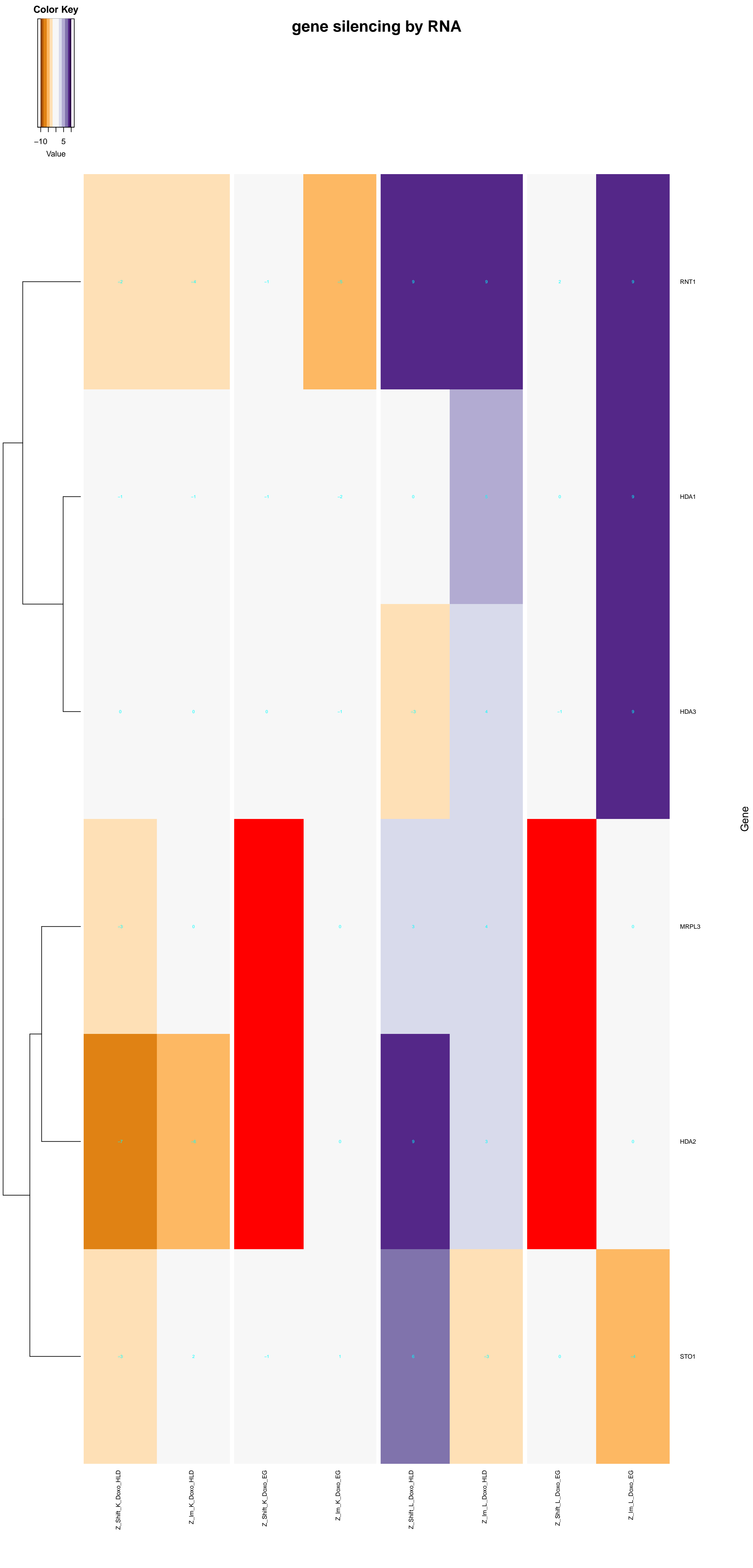



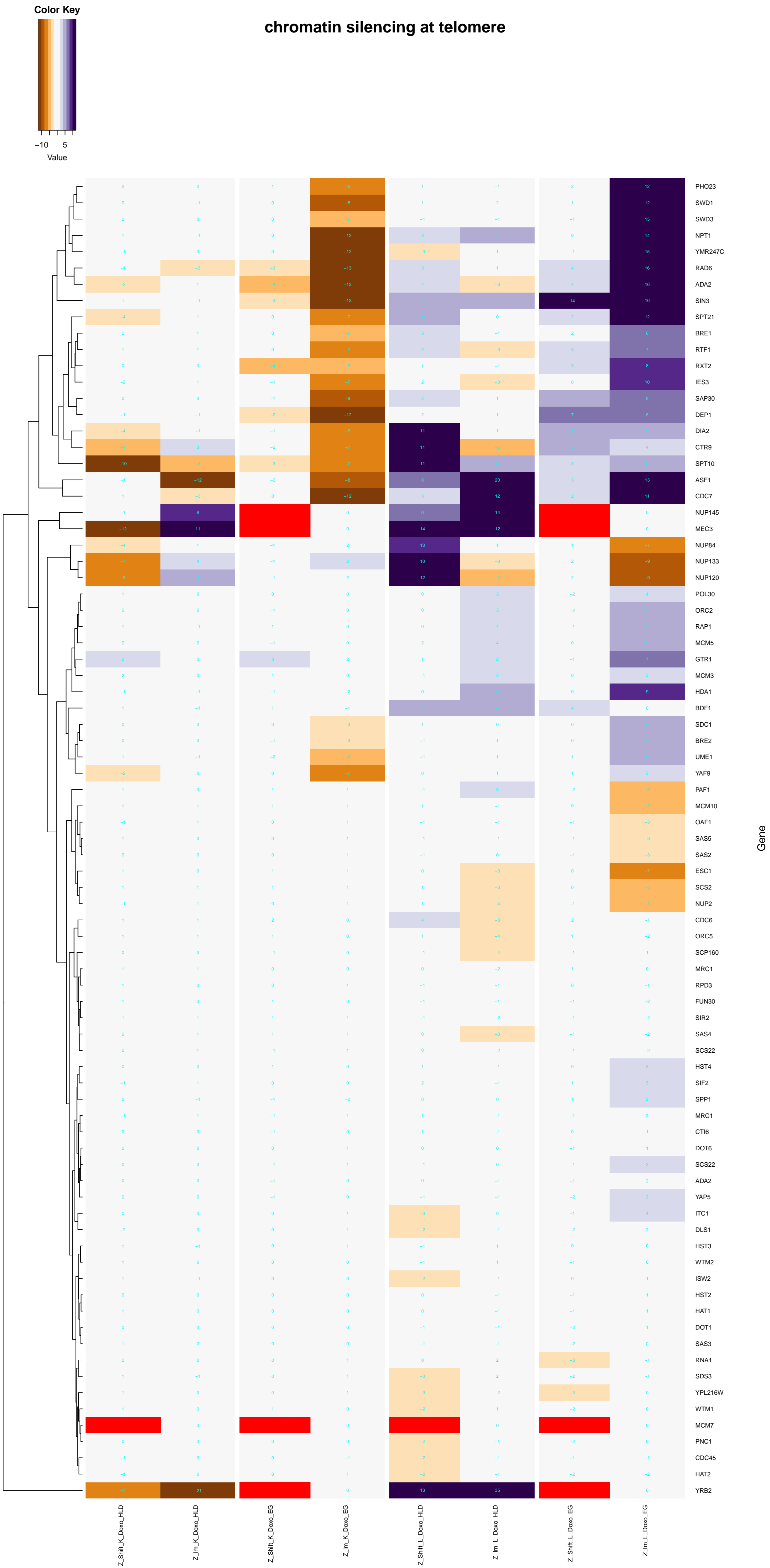

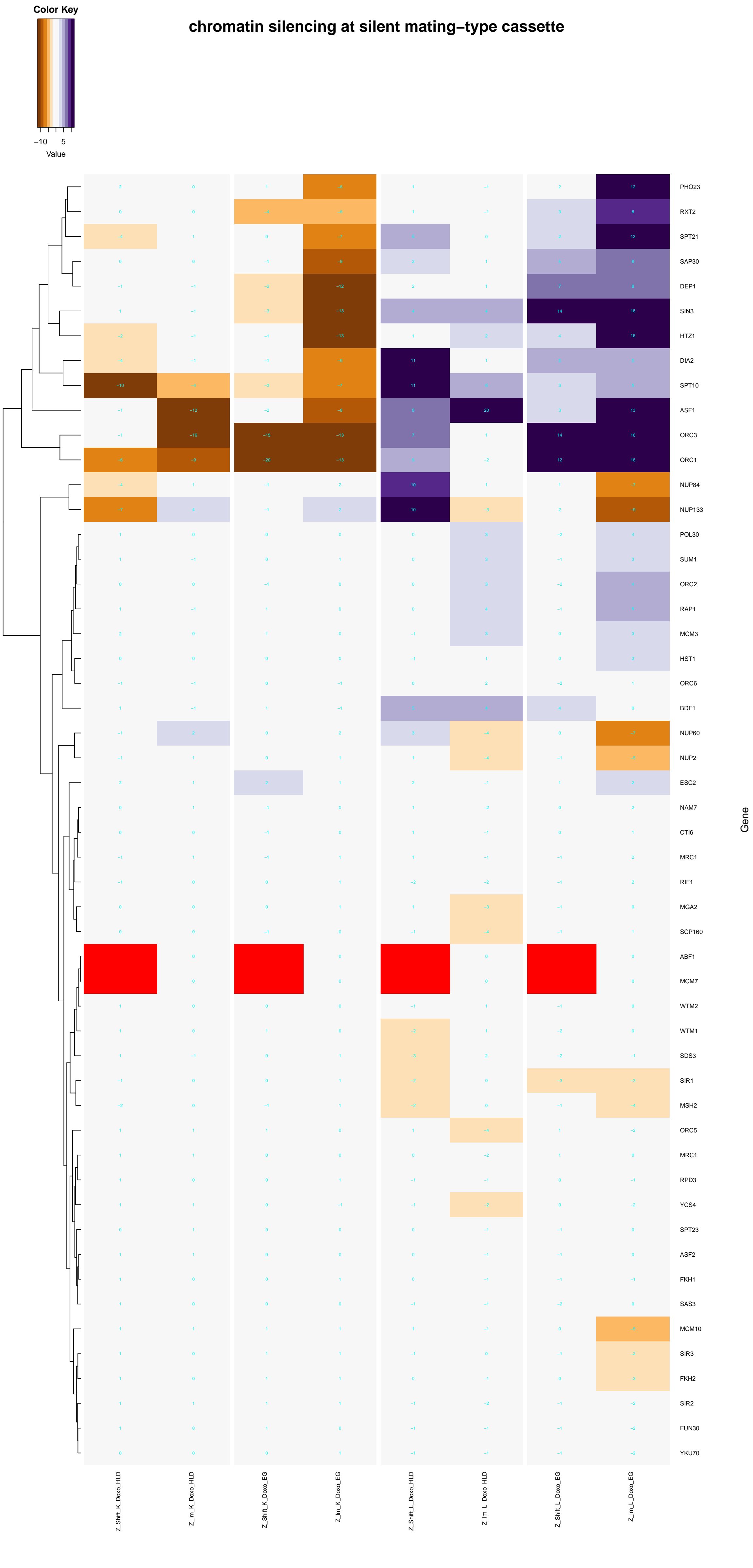

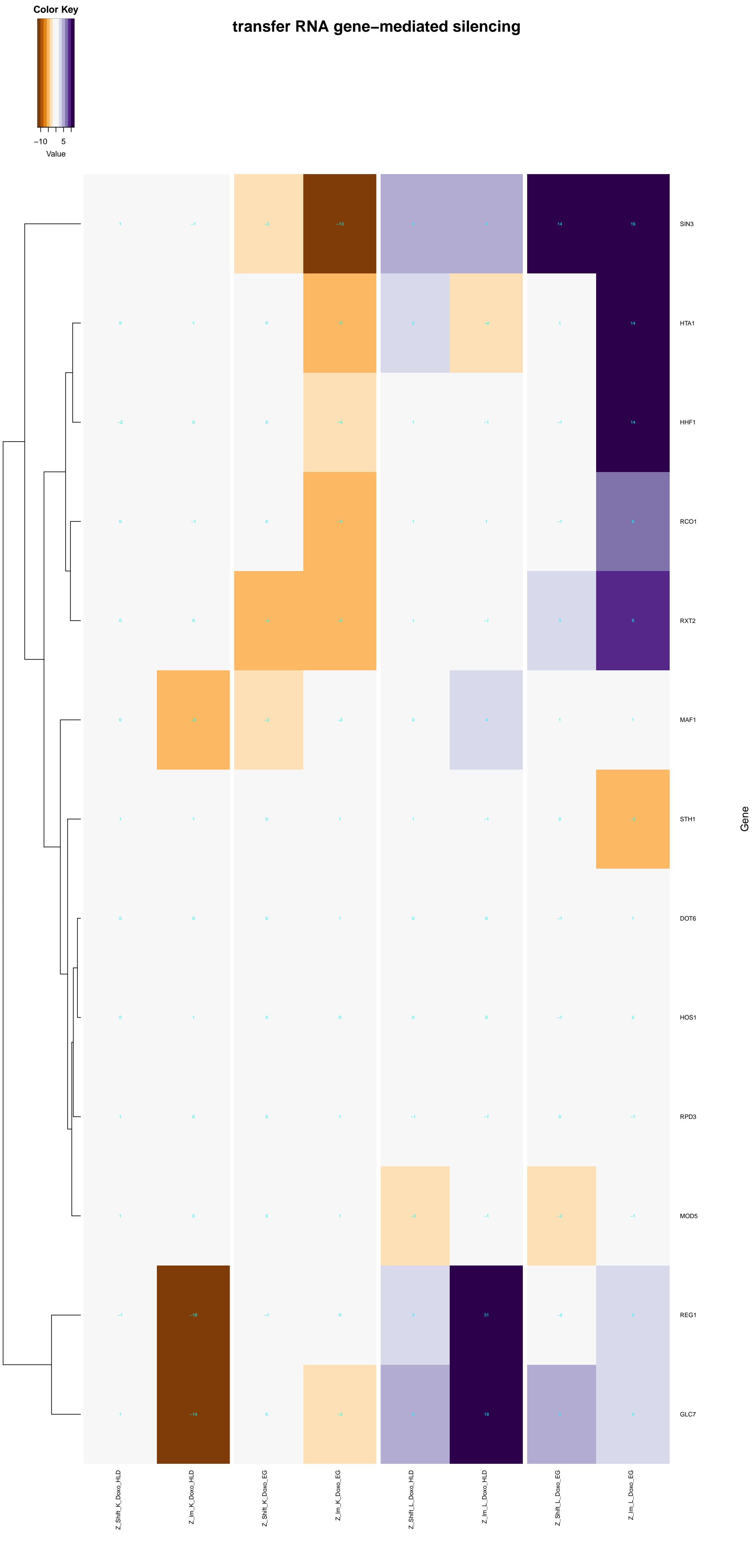

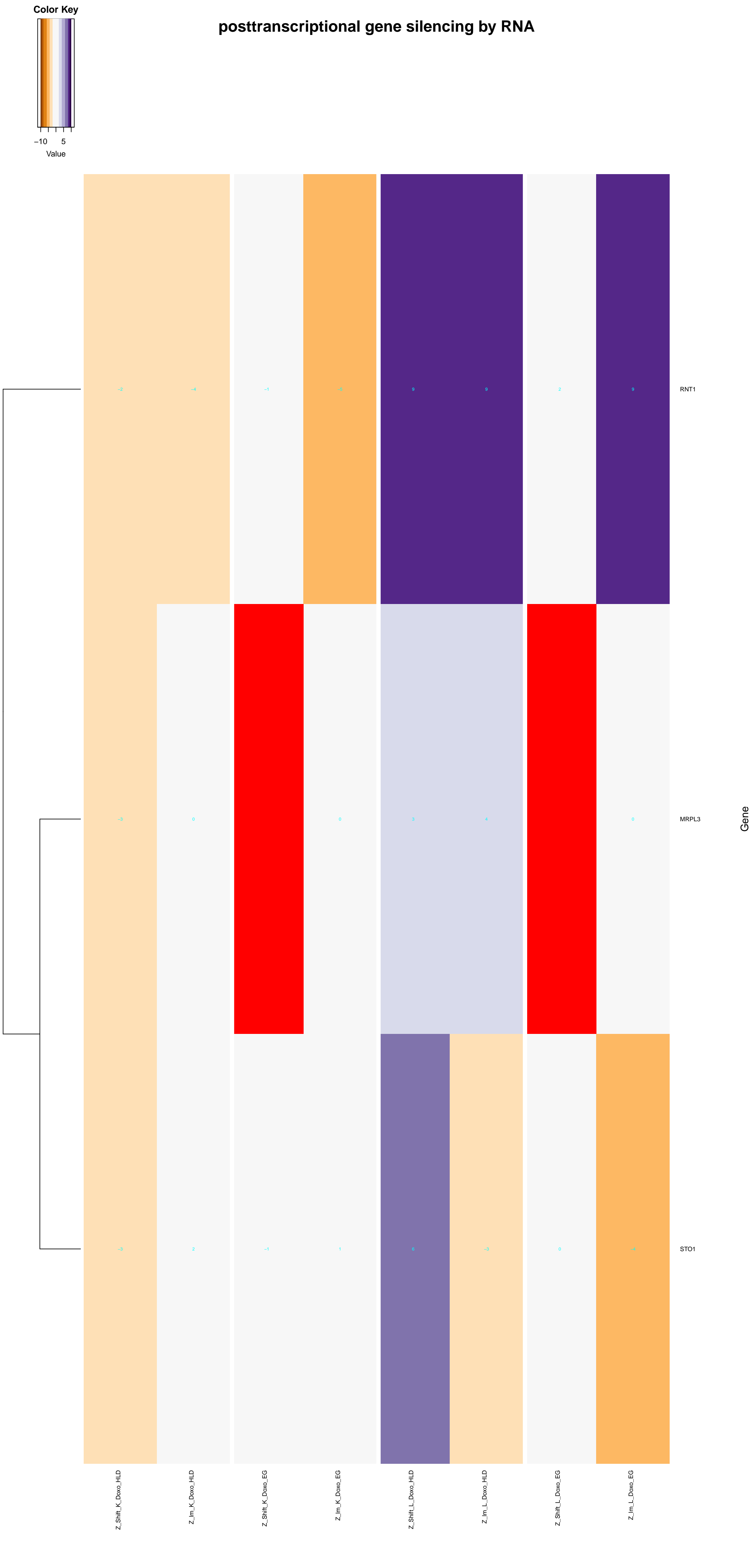

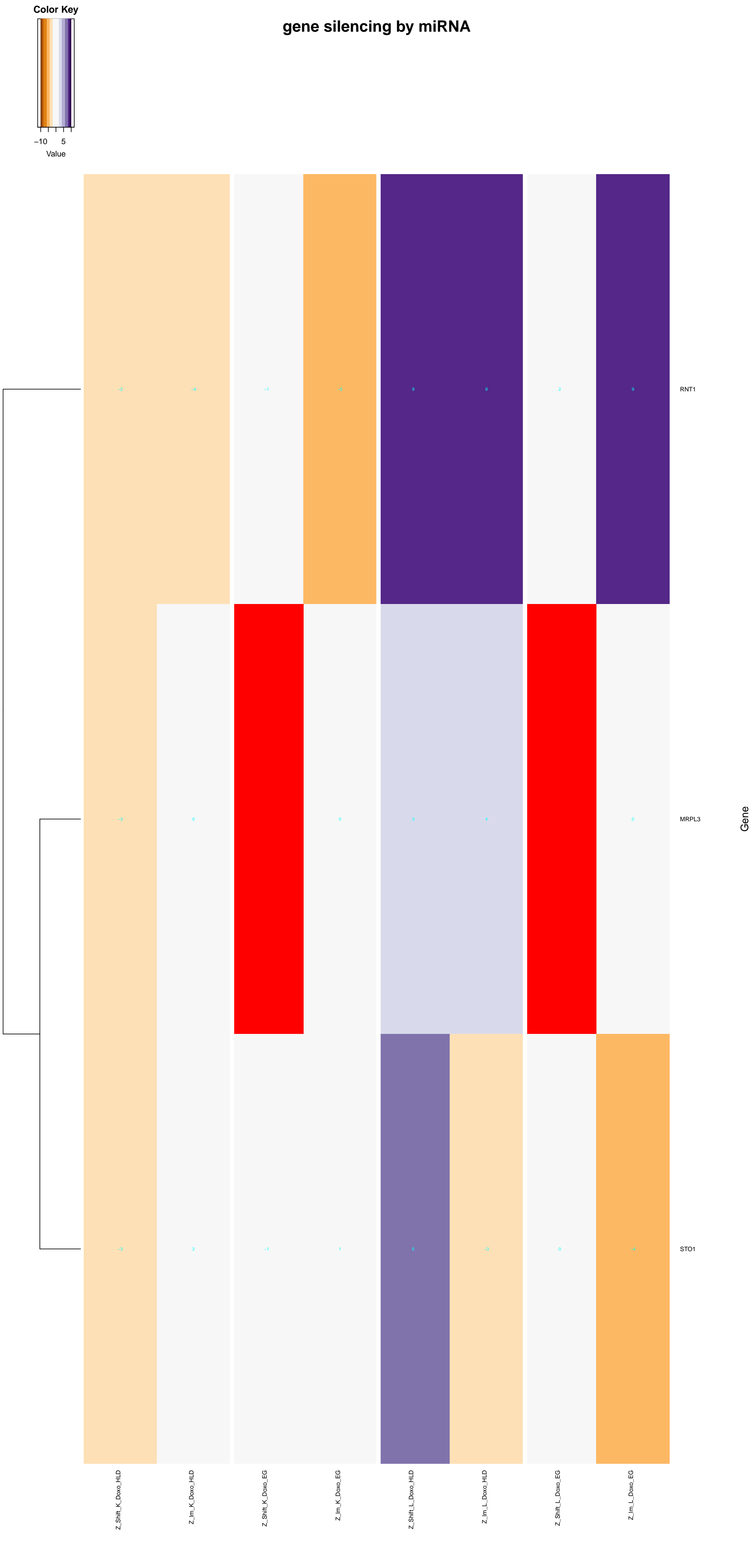

Supplement: Supplementary file 9 — Additional file 9. GO term-specific heatmaps for REMc/GTF-enriched clusters. GO term-specific heatmaps for significant GO process terms were generated as described in methods and Figs. 3 and 4. Any related child terms are presented in subsequent pages of the parent file name. GO terms with more than 100 children, with 2 or fewer genes annotated to the term, or a file size over 300KB are not shown. All heatmaps are generated with the same layout (see Figs. 3 and 4). [file 40170_2019_201_MOESM9_ESM.bz2 › Additional_File9_GOTermHeatmaps/Additional_File9_GOTermHeatmap/gene_silencing.pdf]

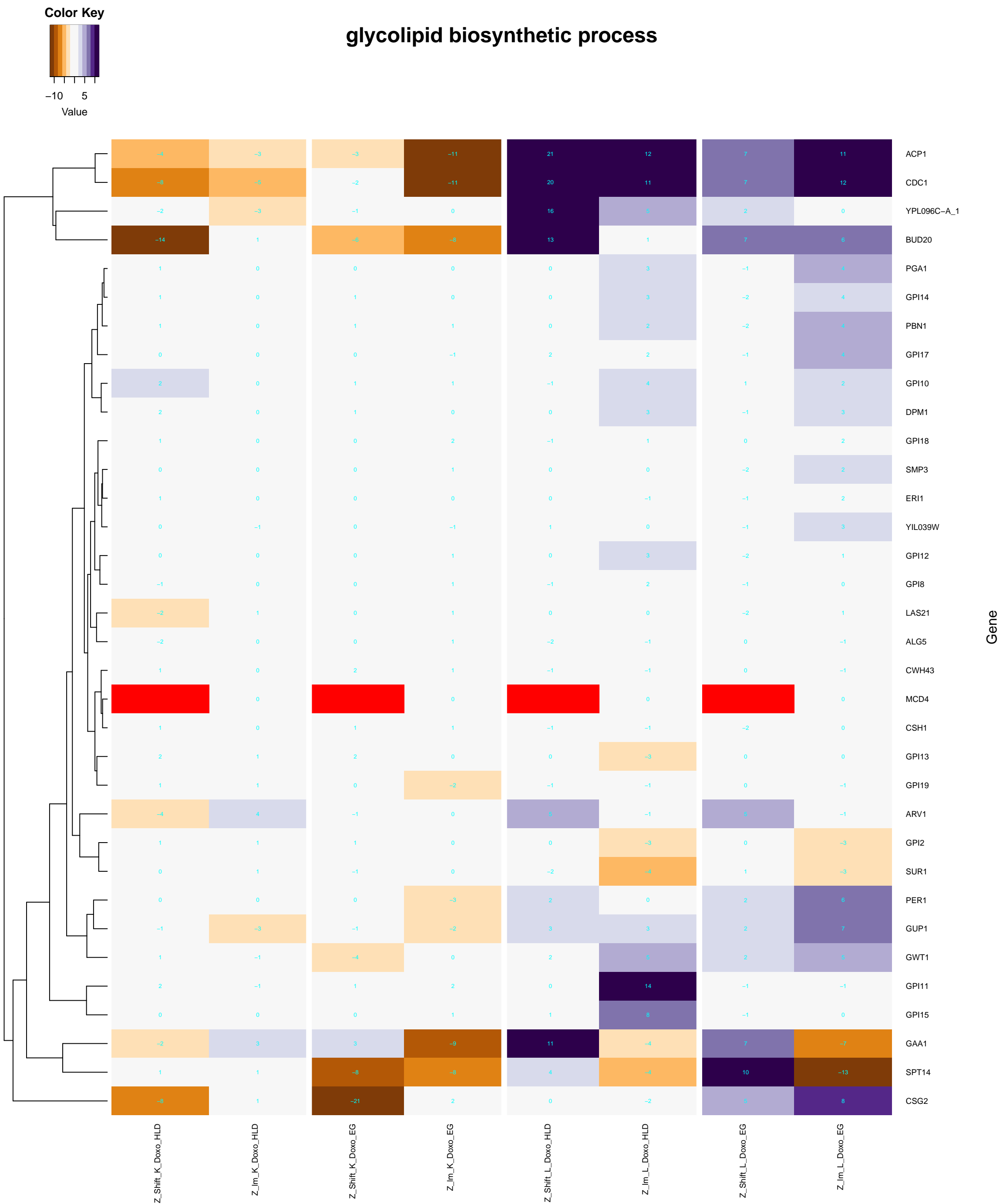

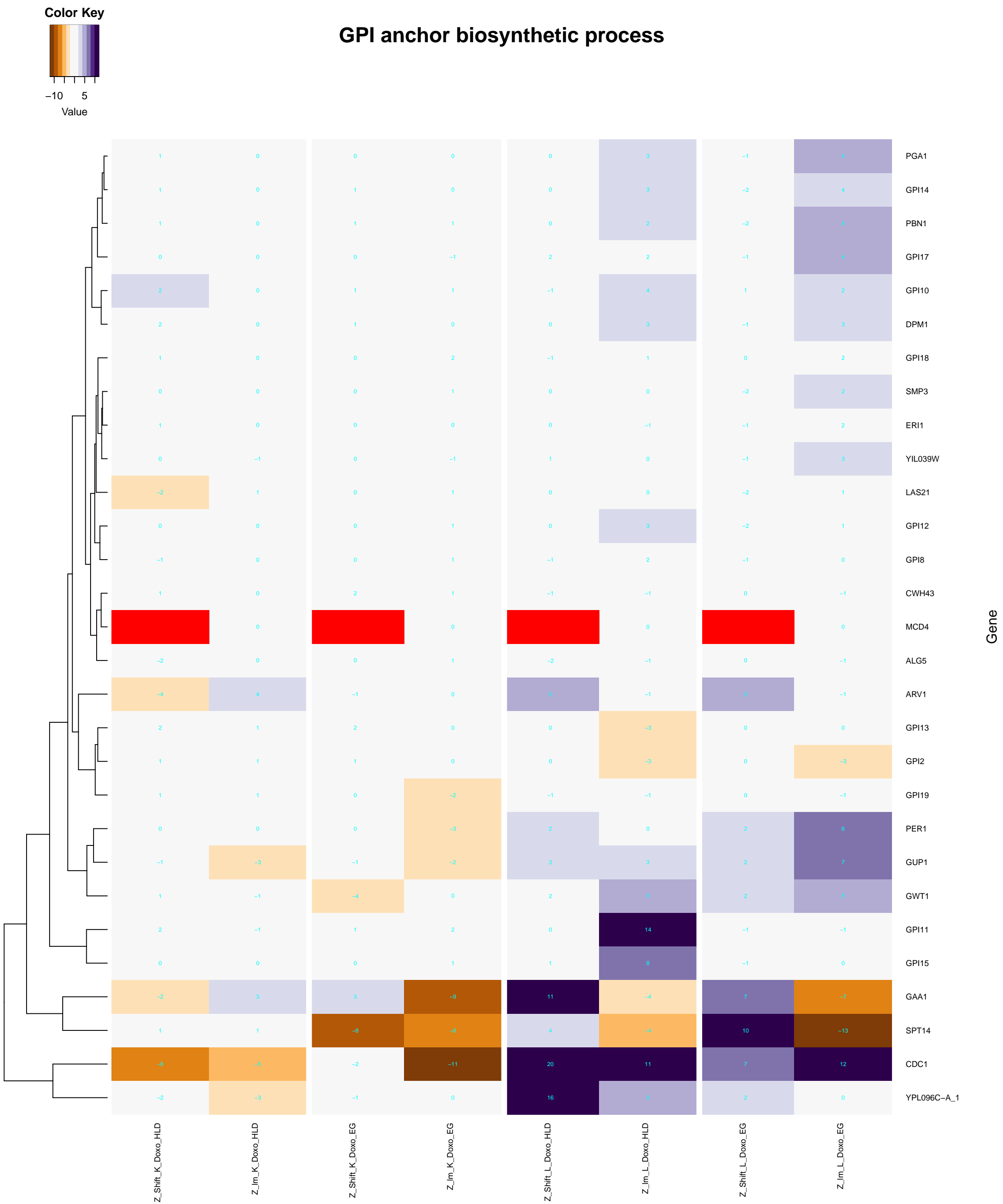

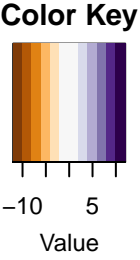

glycosphingolipid biosynthetic process

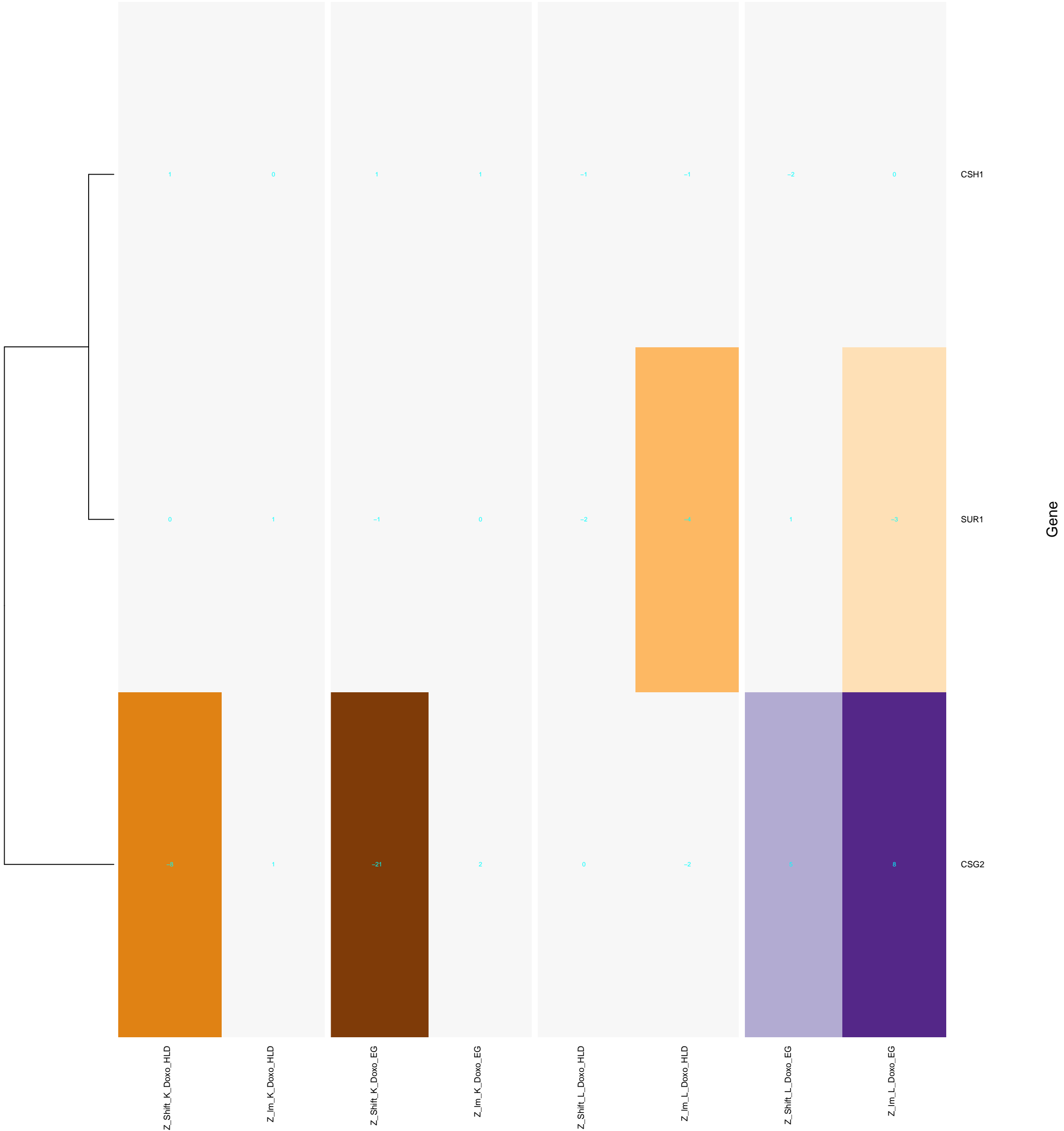

Supplement: Supplementary file 9 — Additional file 9. GO term-specific heatmaps for REMc/GTF-enriched clusters. GO term-specific heatmaps for significant GO process terms were generated as described in methods and Figs. 3 and 4. Any related child terms are presented in subsequent pages of the parent file name. GO terms with more than 100 children, with 2 or fewer genes annotated to the term, or a file size over 300KB are not shown. All heatmaps are generated with the same layout (see Figs. 3 and 4). [file 40170_2019_201_MOESM9_ESM.bz2 › Additional_File9_GOTermHeatmaps/Additional_File9_GOTermHeatmap/glycolipid_biosynthetic_process.pdf]

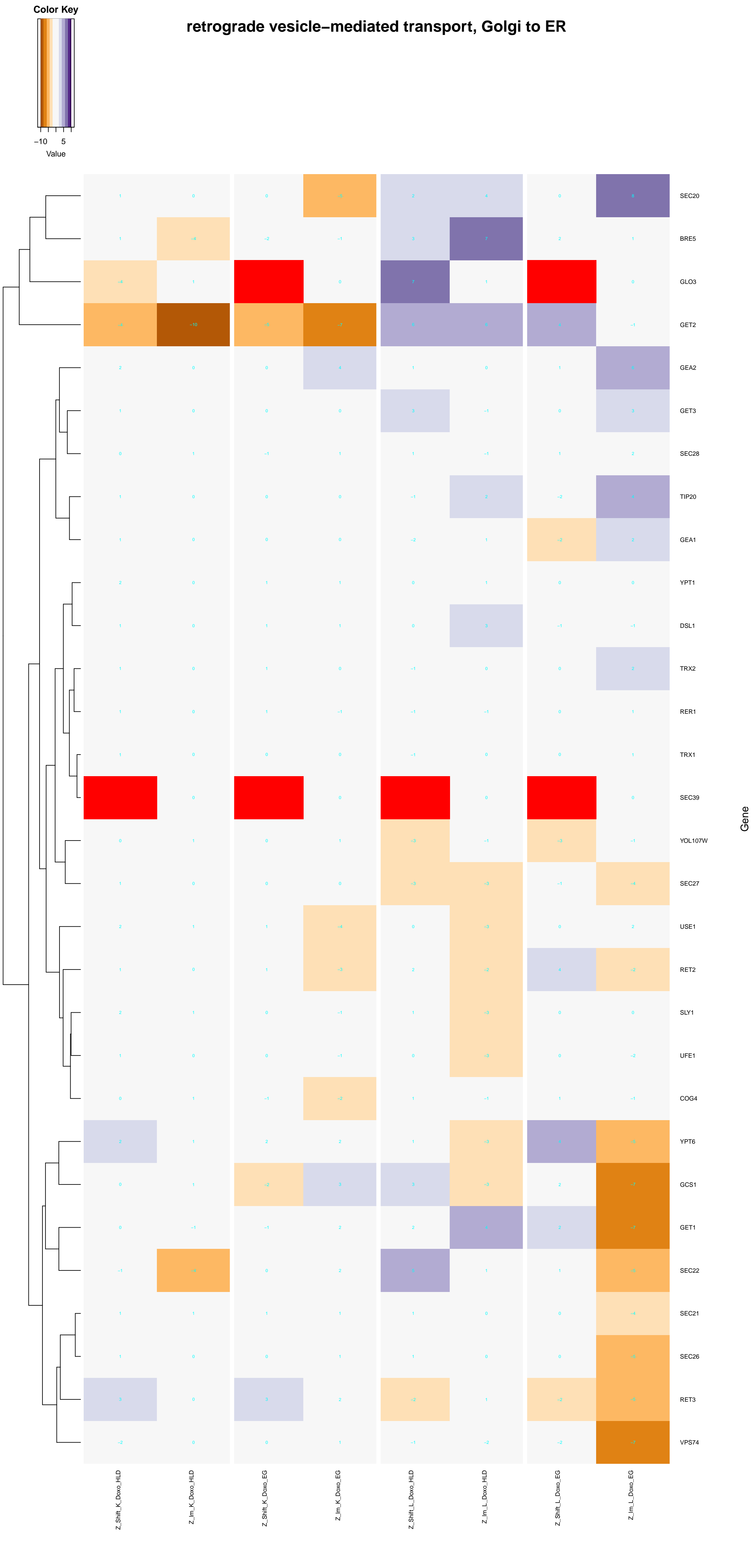

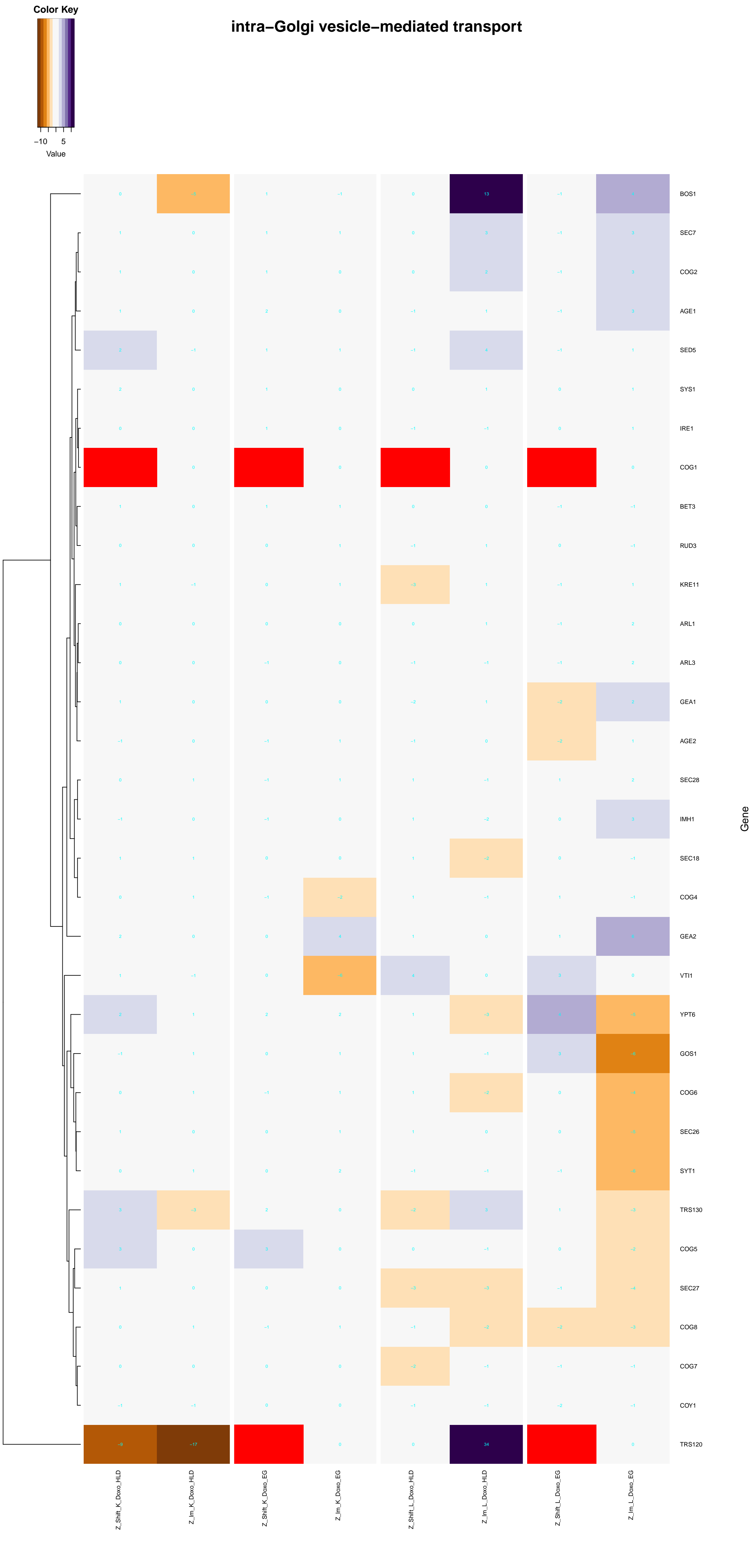

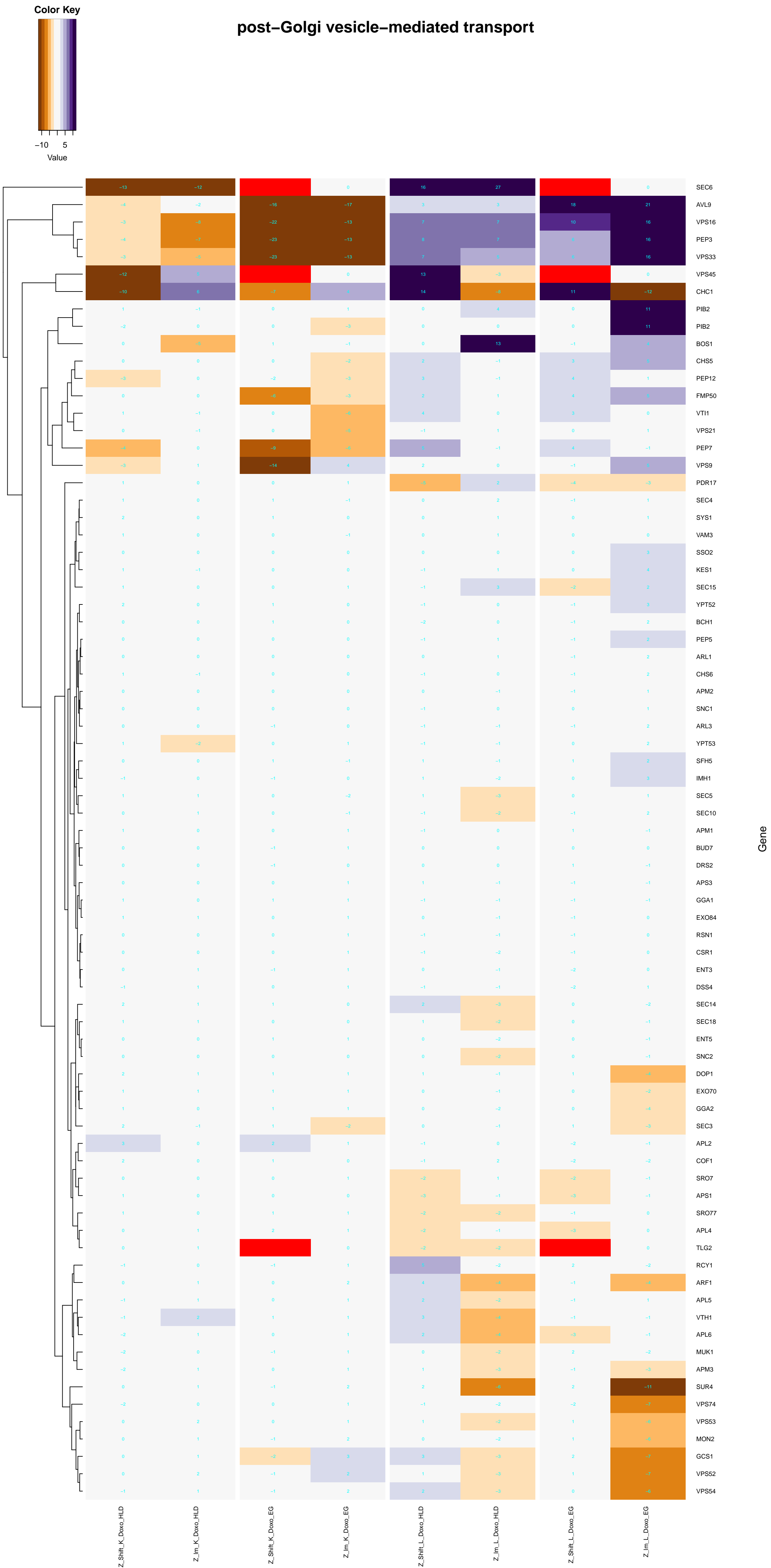

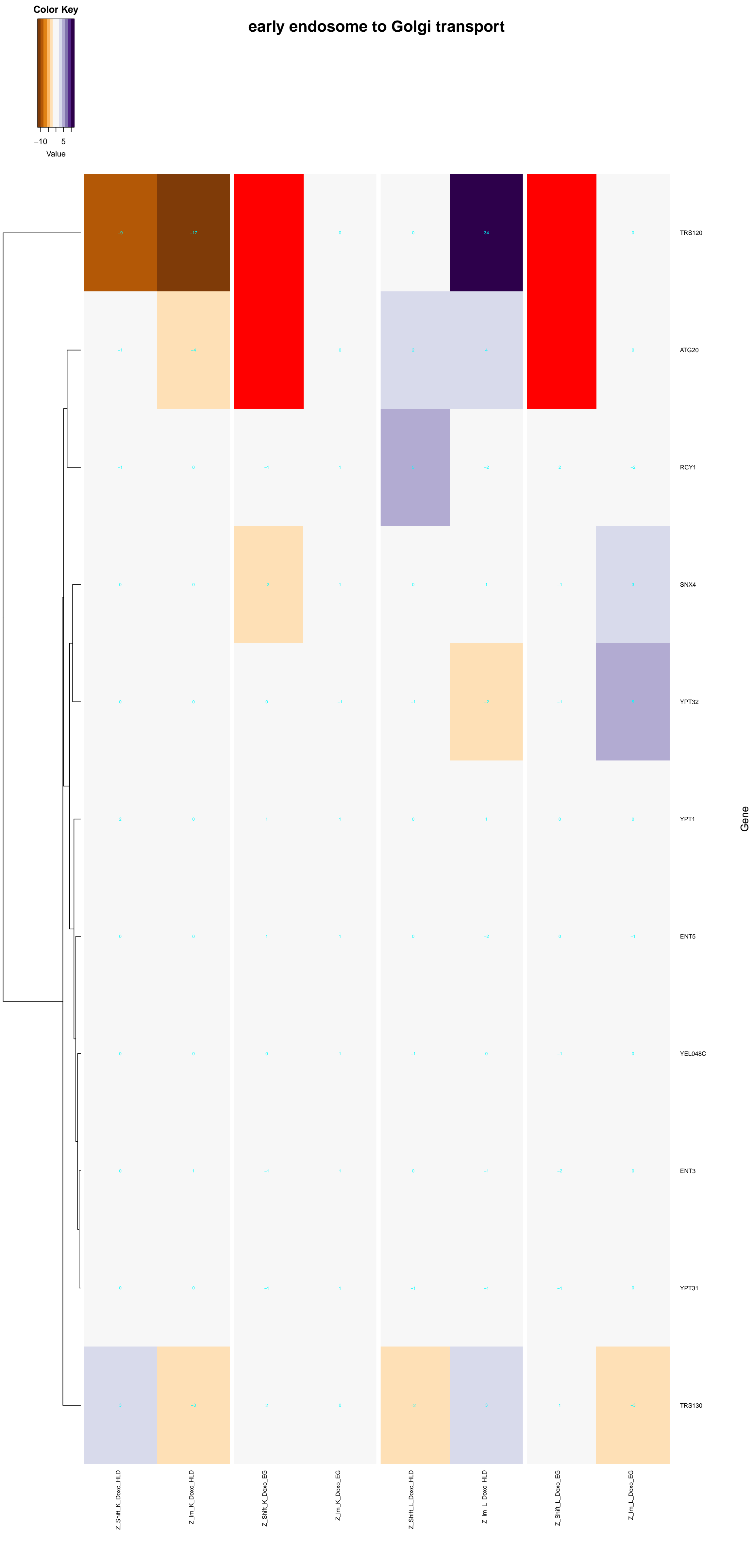

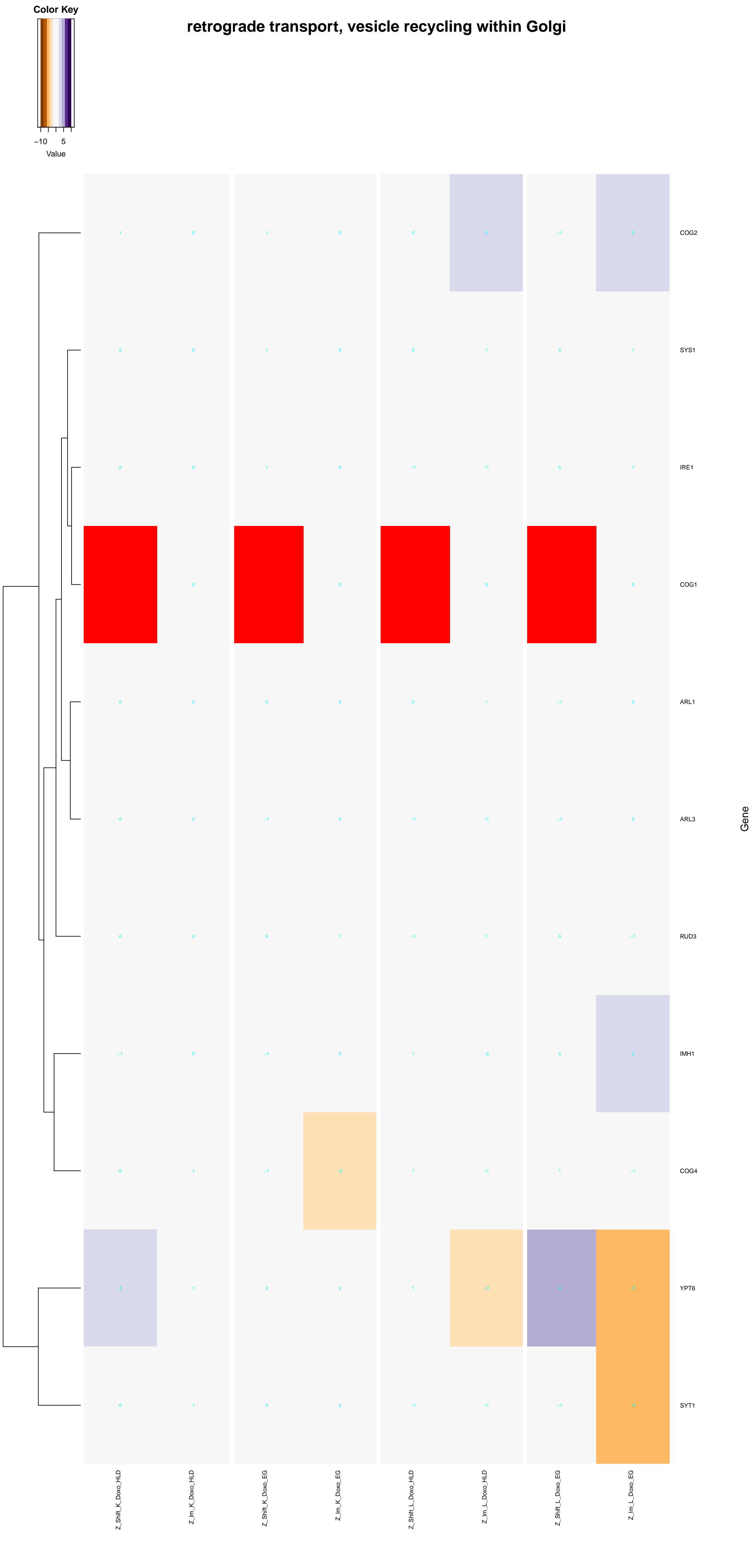

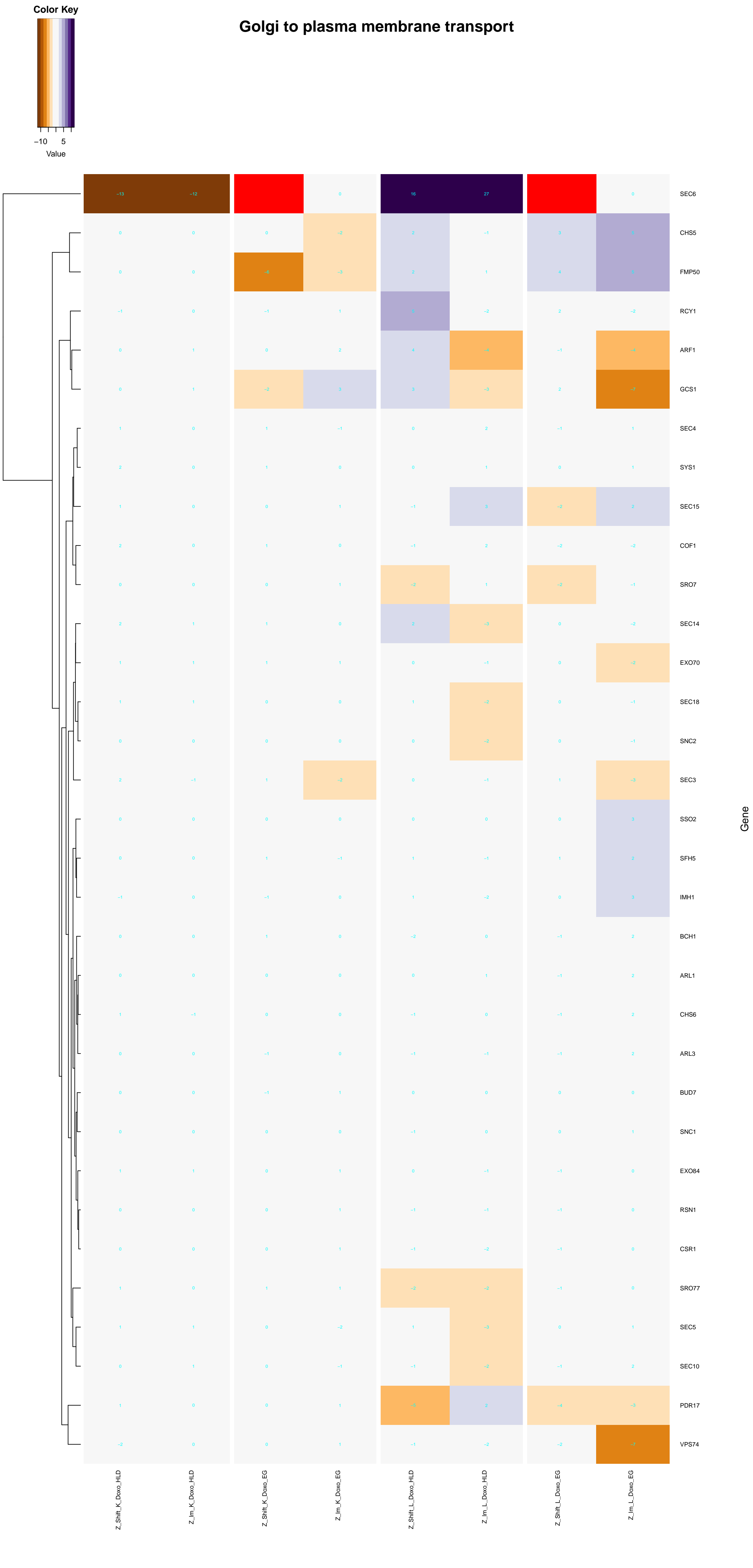

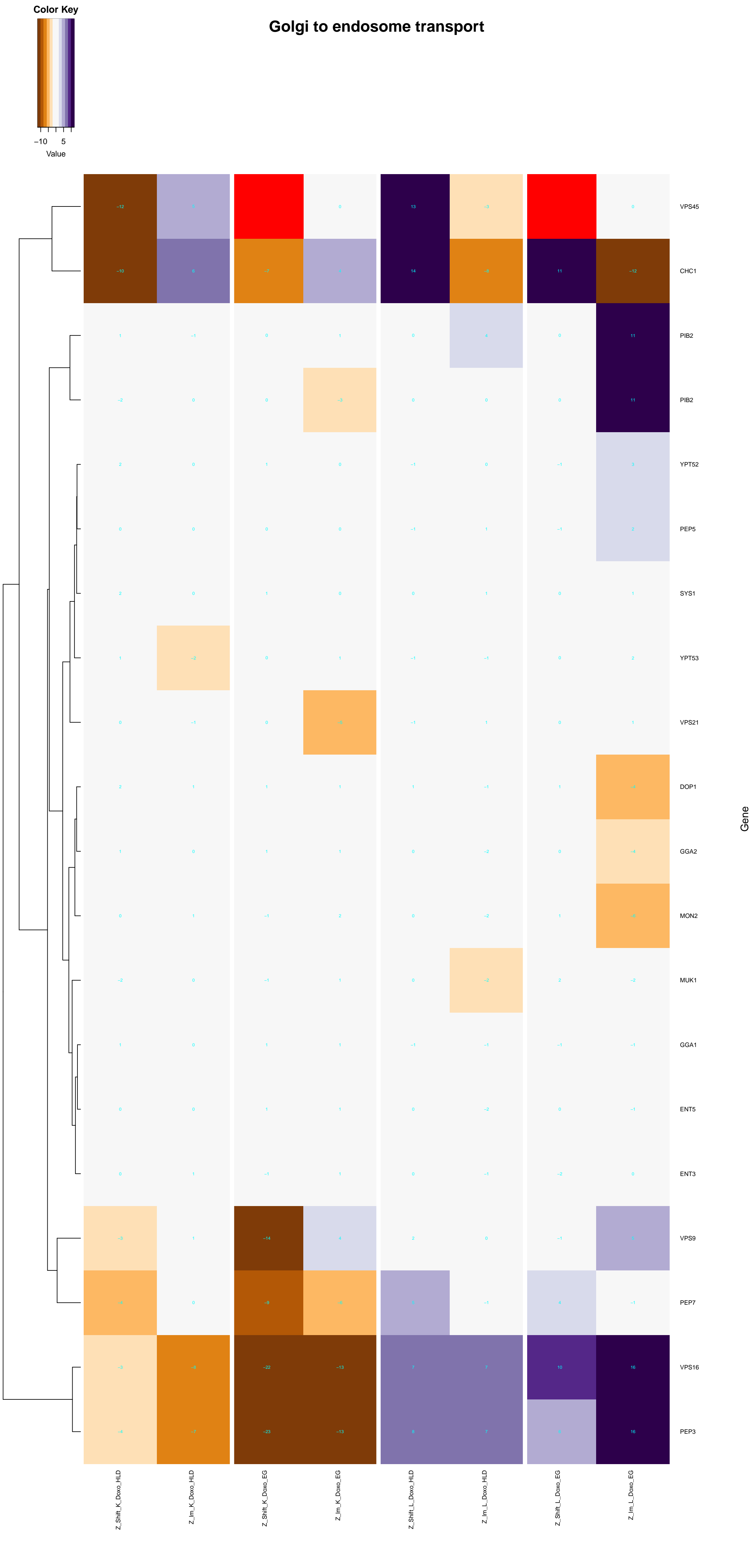

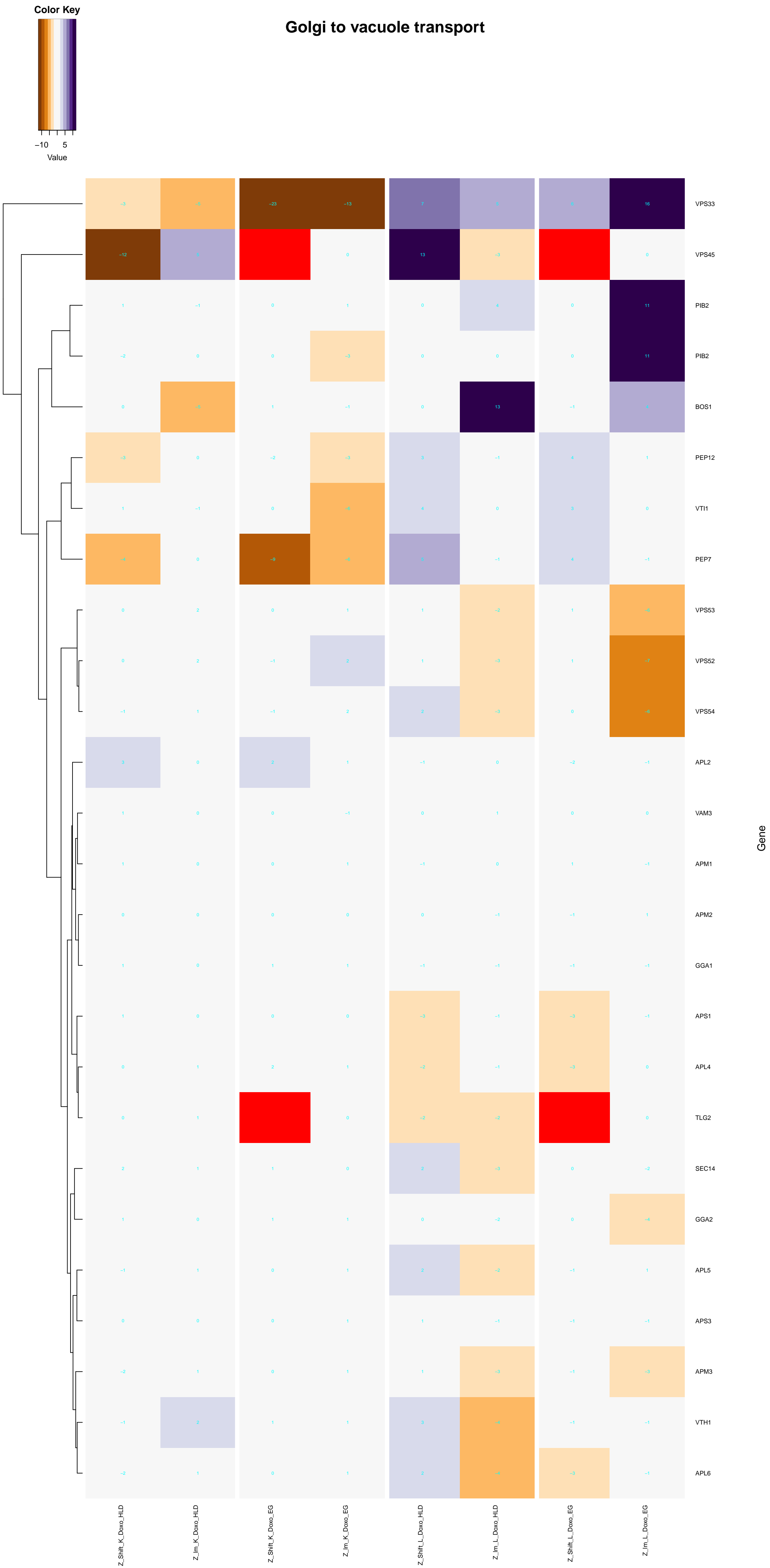

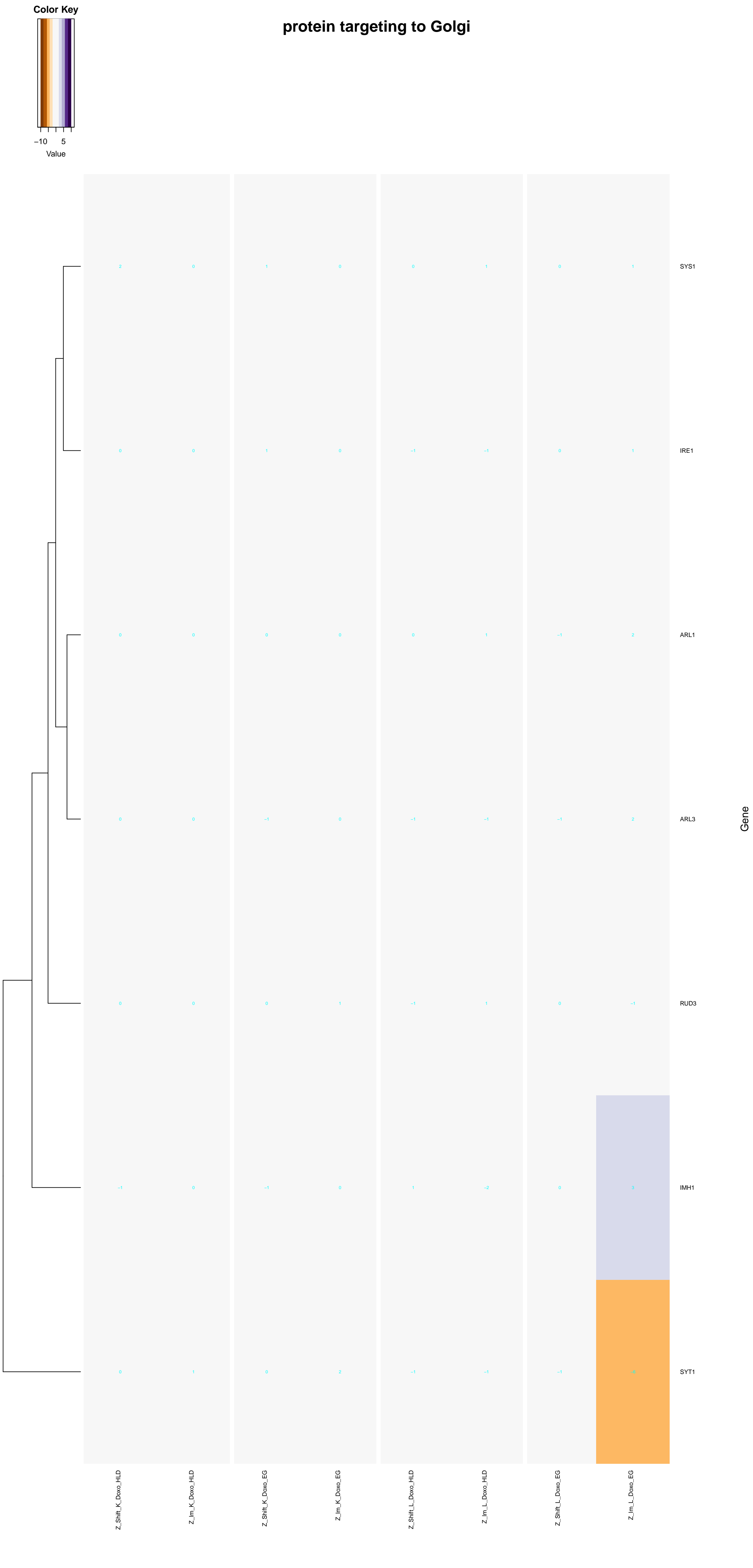

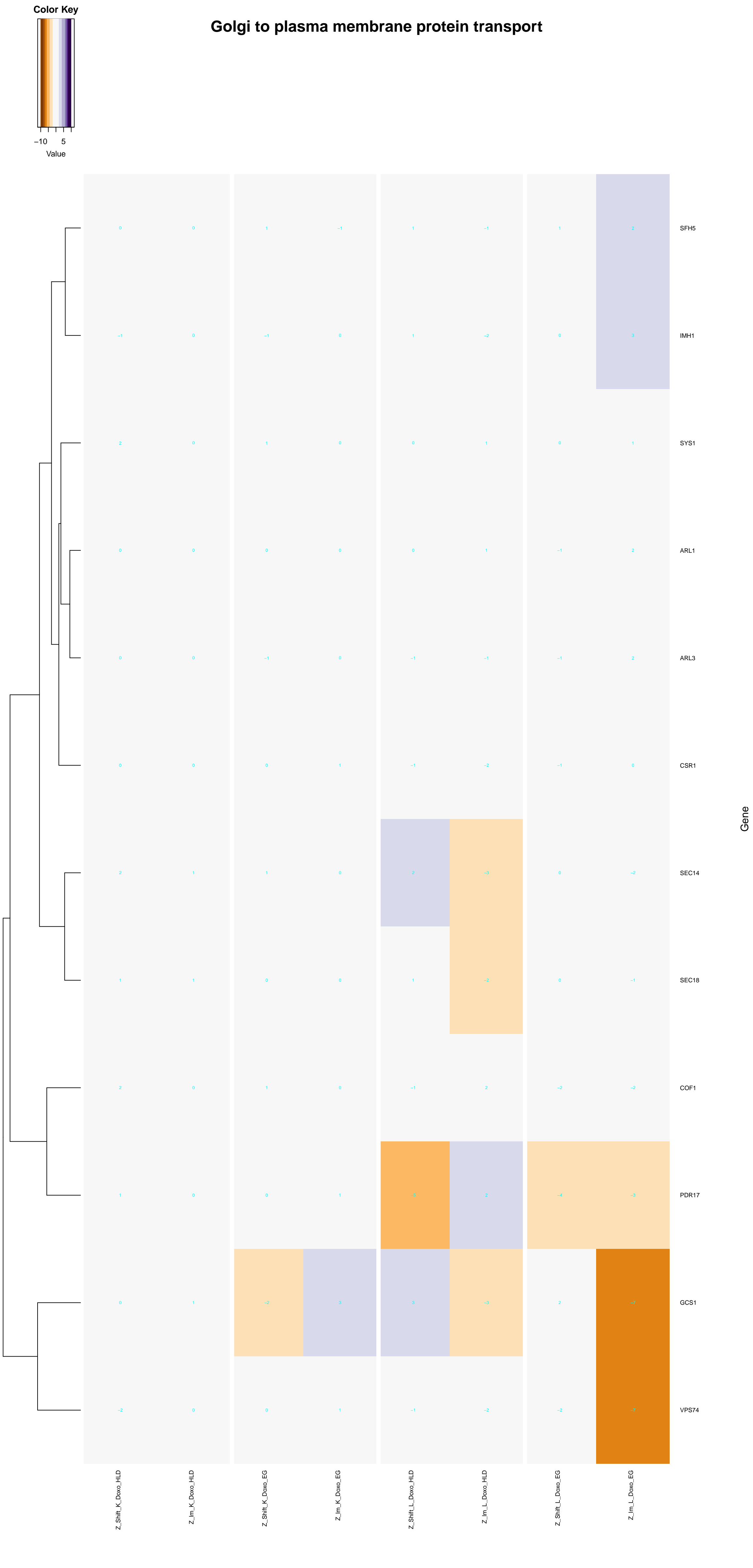

Supplement: Supplementary file 9 — Additional file 9. GO term-specific heatmaps for REMc/GTF-enriched clusters. GO term-specific heatmaps for significant GO process terms were generated as described in methods and Figs. 3 and 4. Any related child terms are presented in subsequent pages of the parent file name. GO terms with more than 100 children, with 2 or fewer genes annotated to the term, or a file size over 300KB are not shown. All heatmaps are generated with the same layout (see Figs. 3 and 4). [file 40170_2019_201_MOESM9_ESM.bz2 › Additional_File9_GOTermHeatmaps/Additional_File9_GOTermHeatmap/Golgi_vesicle_transport.pdf]

## GPI anchor biosynthetic process

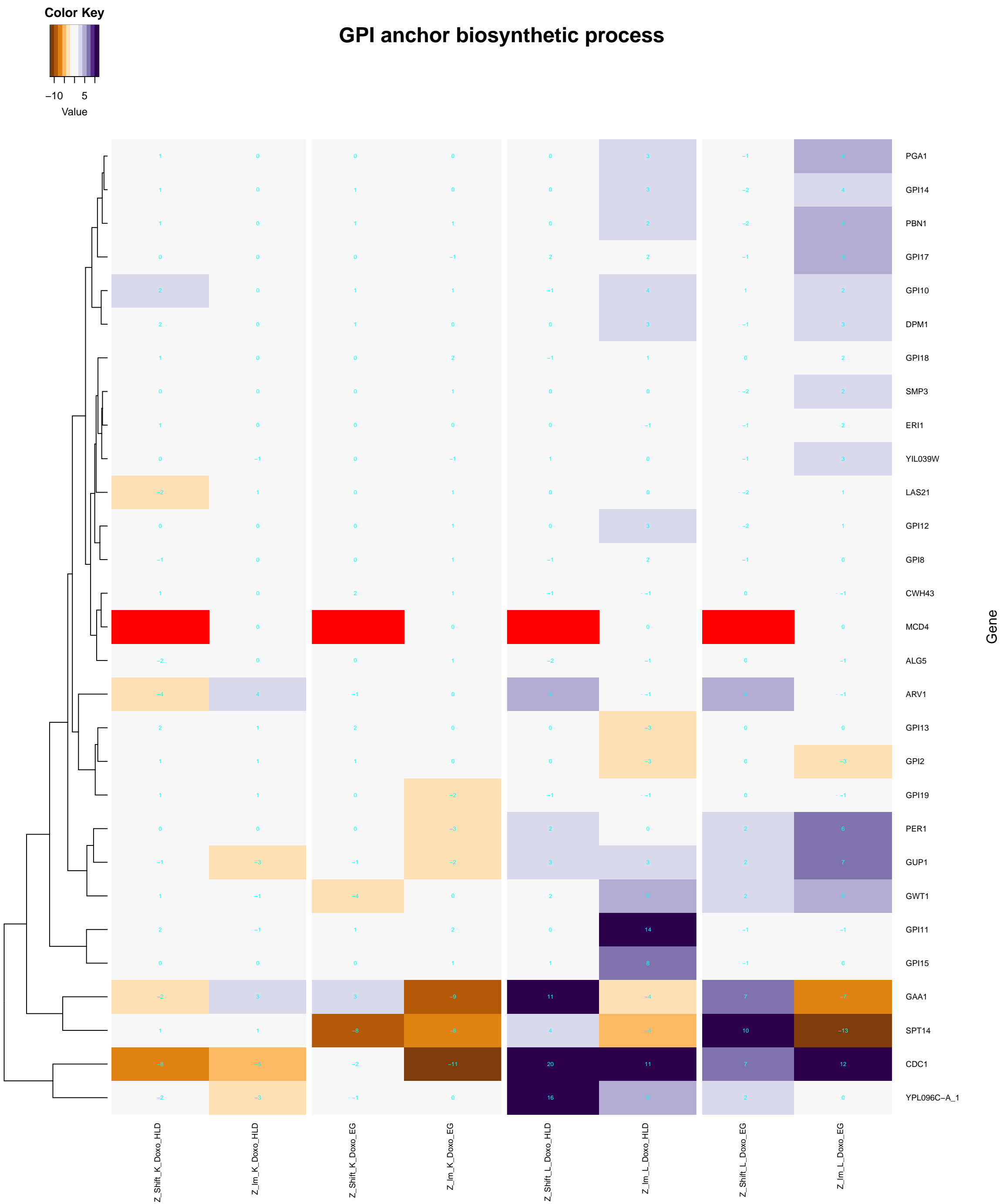

Supplement: Supplementary file 9 — Additional file 9. GO term-specific heatmaps for REMc/GTF-enriched clusters. GO term-specific heatmaps for significant GO process terms were generated as described in methods and Figs. 3 and 4. Any related child terms are presented in subsequent pages of the parent file name. GO terms with more than 100 children, with 2 or fewer genes annotated to the term, or a file size over 300KB are not shown. All heatmaps are generated with the same layout (see Figs. 3 and 4). [file 40170_2019_201_MOESM9_ESM.bz2 › Additional_File9_GOTermHeatmaps/Additional_File9_GOTermHeatmap/GPI_anchor_biosynthetic_process.pdf]

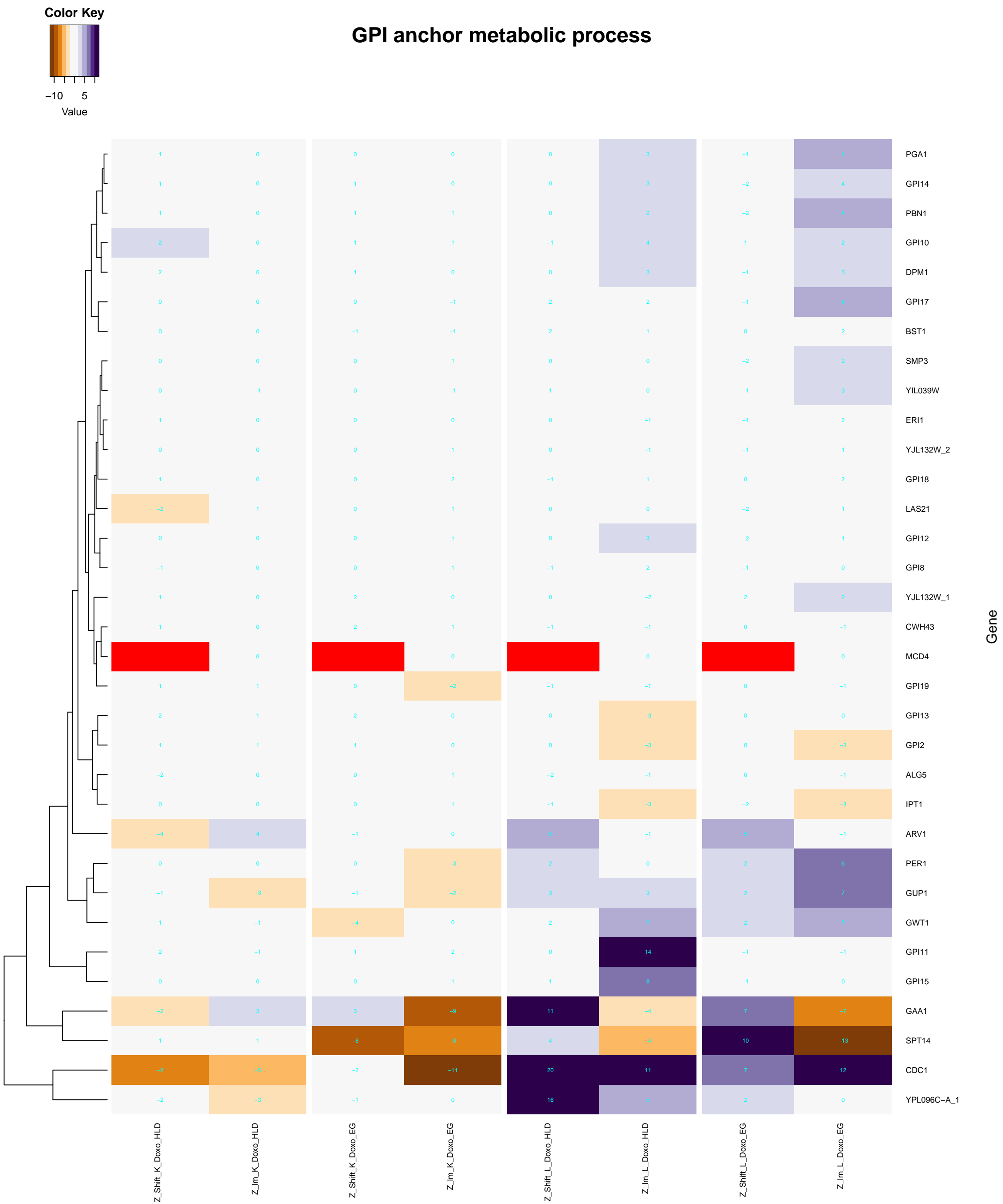

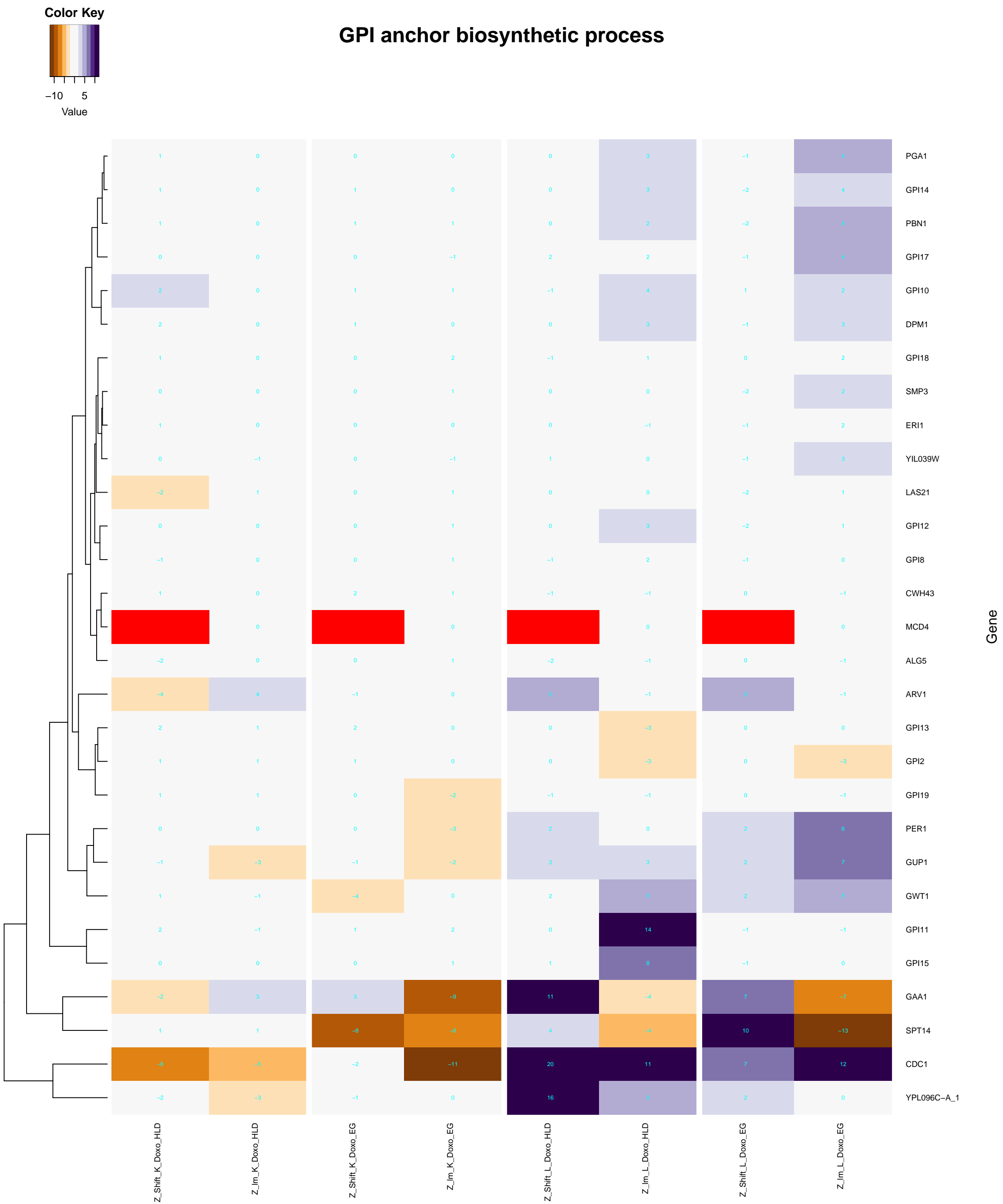

Supplement: Supplementary file 9 — Additional file 9. GO term-specific heatmaps for REMc/GTF-enriched clusters. GO term-specific heatmaps for significant GO process terms were generated as described in methods and Figs. 3 and 4. Any related child terms are presented in subsequent pages of the parent file name. GO terms with more than 100 children, with 2 or fewer genes annotated to the term, or a file size over 300KB are not shown. All heatmaps are generated with the same layout (see Figs. 3 and 4). [file 40170_2019_201_MOESM9_ESM.bz2 › Additional_File9_GOTermHeatmaps/Additional_File9_GOTermHeatmap/GPI_anchor_metabolic_process.pdf]

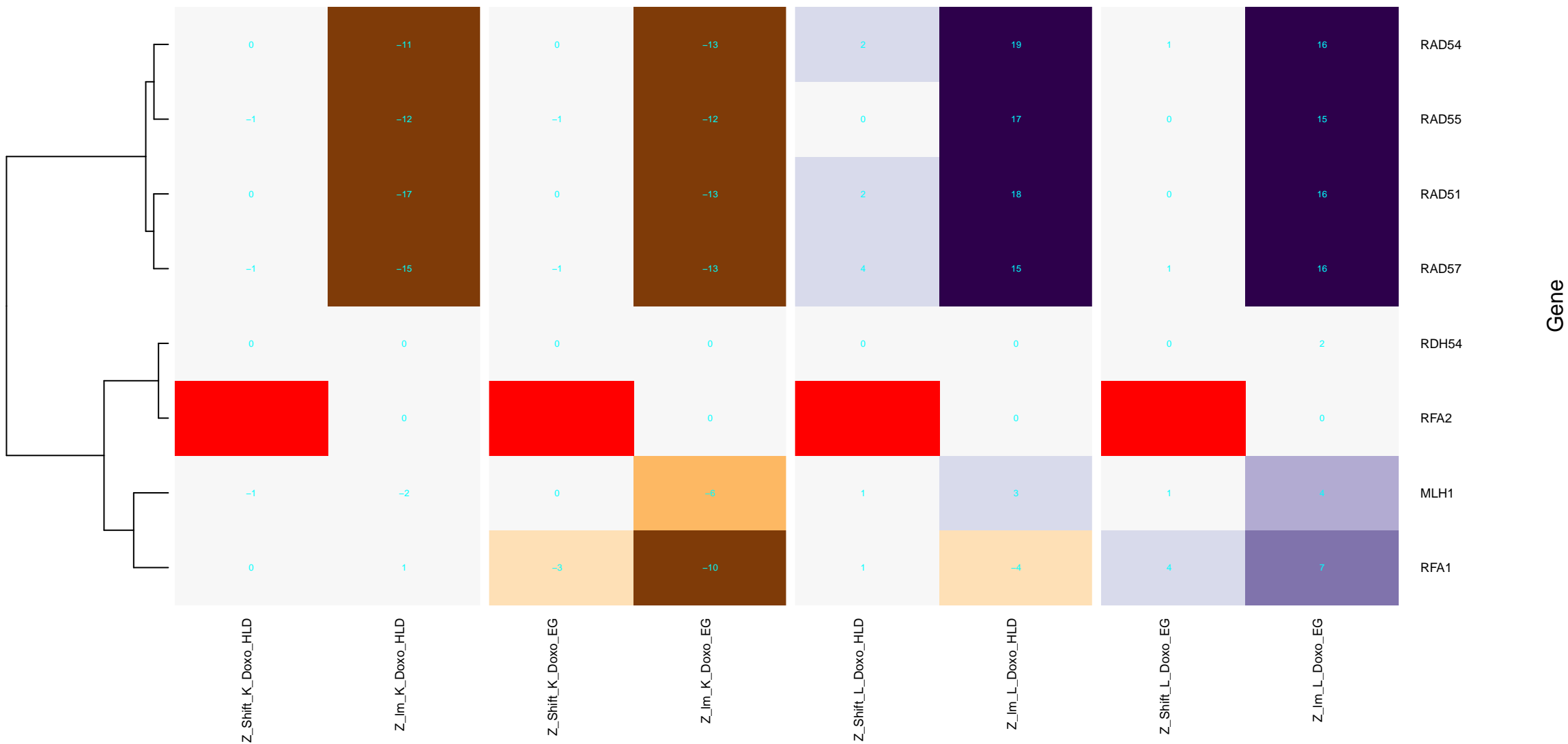

Supplement: Supplementary file 9 — Additional file 9. GO term-specific heatmaps for REMc/GTF-enriched clusters. GO term-specific heatmaps for significant GO process terms were generated as described in methods and Figs. 3 and 4. Any related child terms are presented in subsequent pages of the parent file name. GO terms with more than 100 children, with 2 or fewer genes annotated to the term, or a file size over 300KB are not shown. All heatmaps are generated with the same layout (see Figs. 3 and 4). [file 40170_2019_201_MOESM9_ESM.bz2 › Additional_File9_GOTermHeatmaps/Additional_File9_GOTermHeatmap/heteroduplex_formation.pdf]

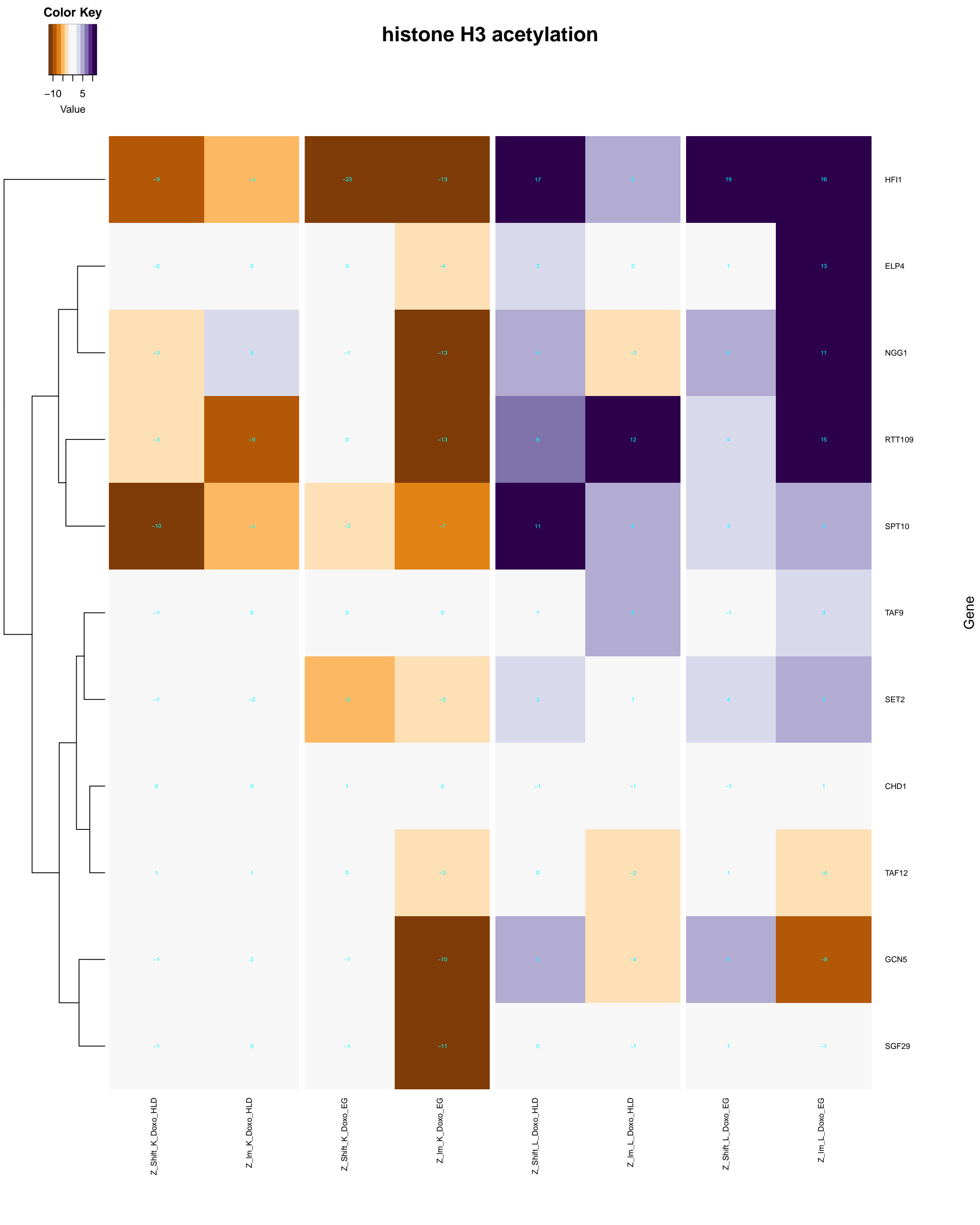

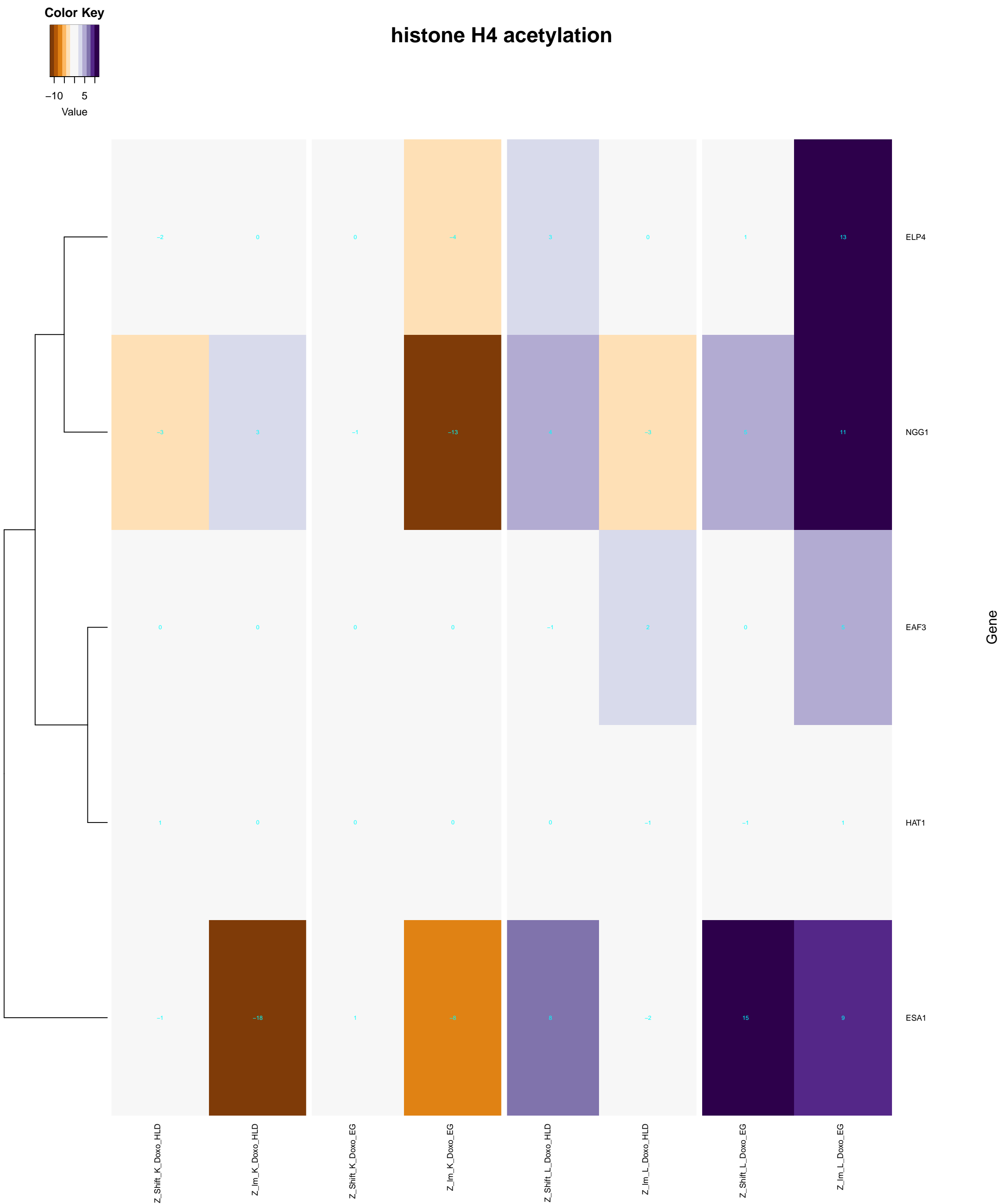

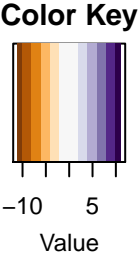

histone H3-K9 acetylation

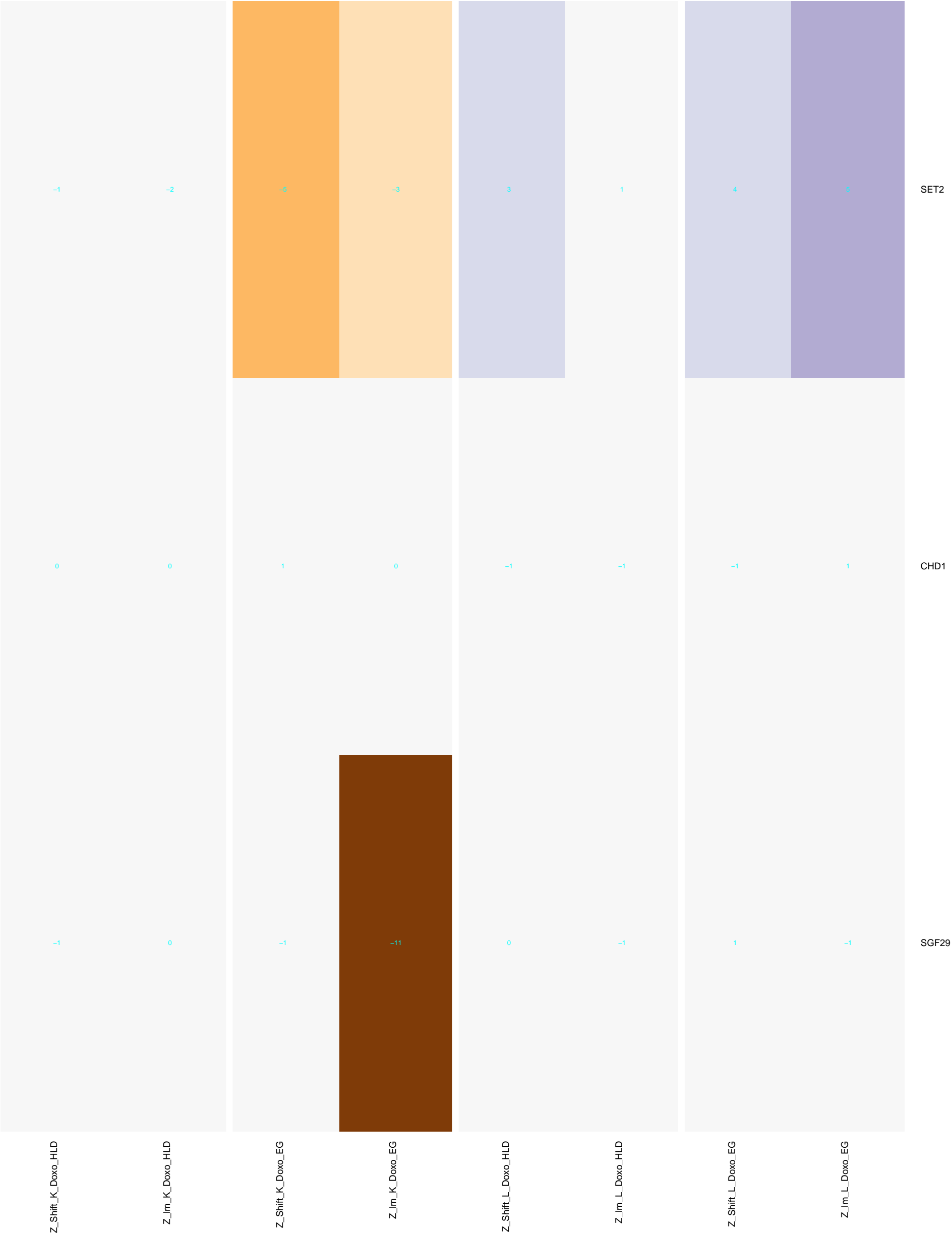

Gene

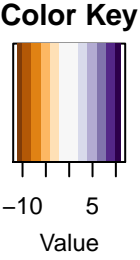

histone H3-K14 acetylation

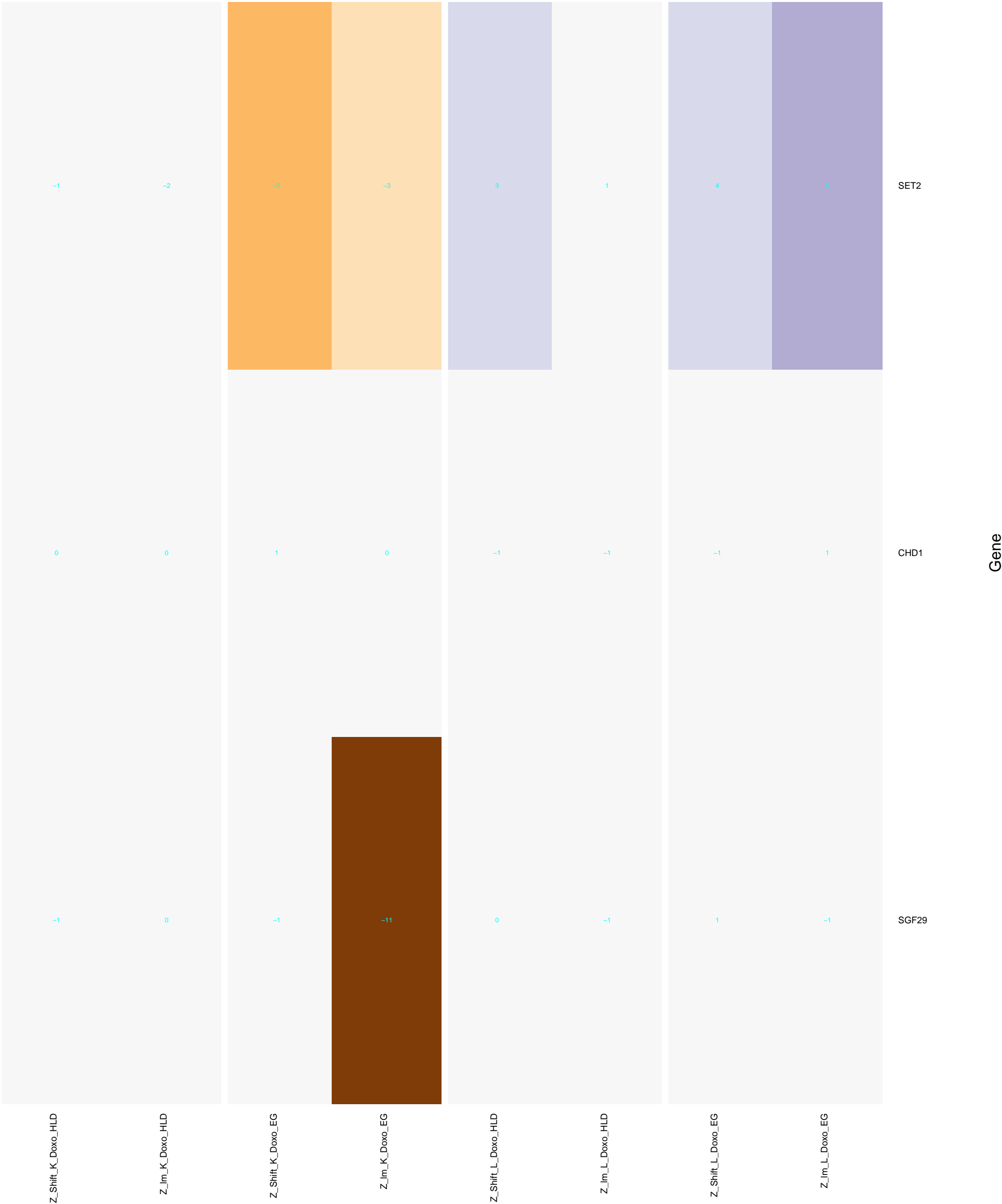

Supplement: Supplementary file 9 — Additional file 9. GO term-specific heatmaps for REMc/GTF-enriched clusters. GO term-specific heatmaps for significant GO process terms were generated as described in methods and Figs. 3 and 4. Any related child terms are presented in subsequent pages of the parent file name. GO terms with more than 100 children, with 2 or fewer genes annotated to the term, or a file size over 300KB are not shown. All heatmaps are generated with the same layout (see Figs. 3 and 4). [file 40170_2019_201_MOESM9_ESM.bz2 › Additional_File9_GOTermHeatmaps/Additional_File9_GOTermHeatmap/histone_acetylation.pdf]

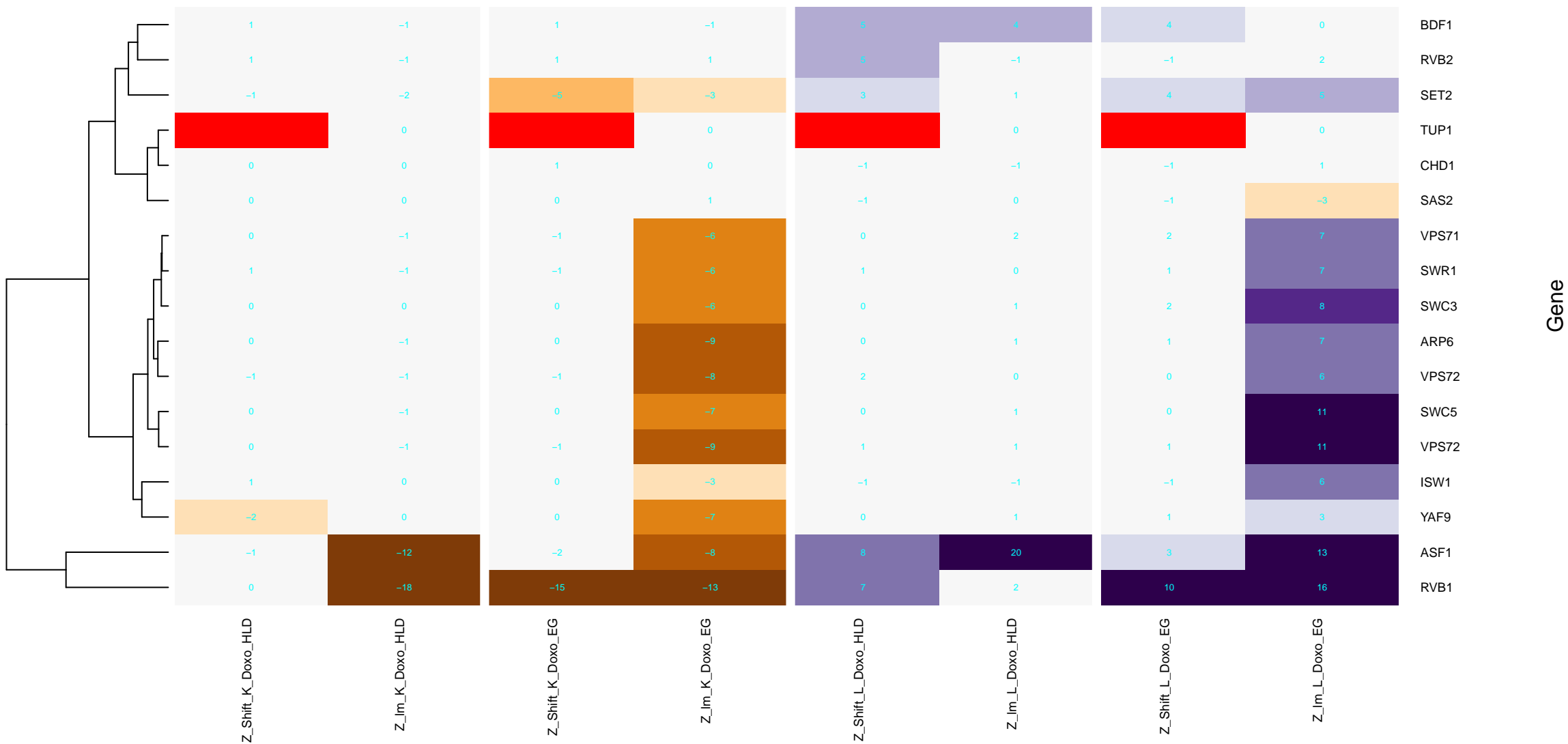

Supplement: Supplementary file 9 — Additional file 9. GO term-specific heatmaps for REMc/GTF-enriched clusters. GO term-specific heatmaps for significant GO process terms were generated as described in methods and Figs. 3 and 4. Any related child terms are presented in subsequent pages of the parent file name. GO terms with more than 100 children, with 2 or fewer genes annotated to the term, or a file size over 300KB are not shown. All heatmaps are generated with the same layout (see Figs. 3 and 4). [file 40170_2019_201_MOESM9_ESM.bz2 › Additional_File9_GOTermHeatmaps/Additional_File9_GOTermHeatmap/histone_exchange.pdf]

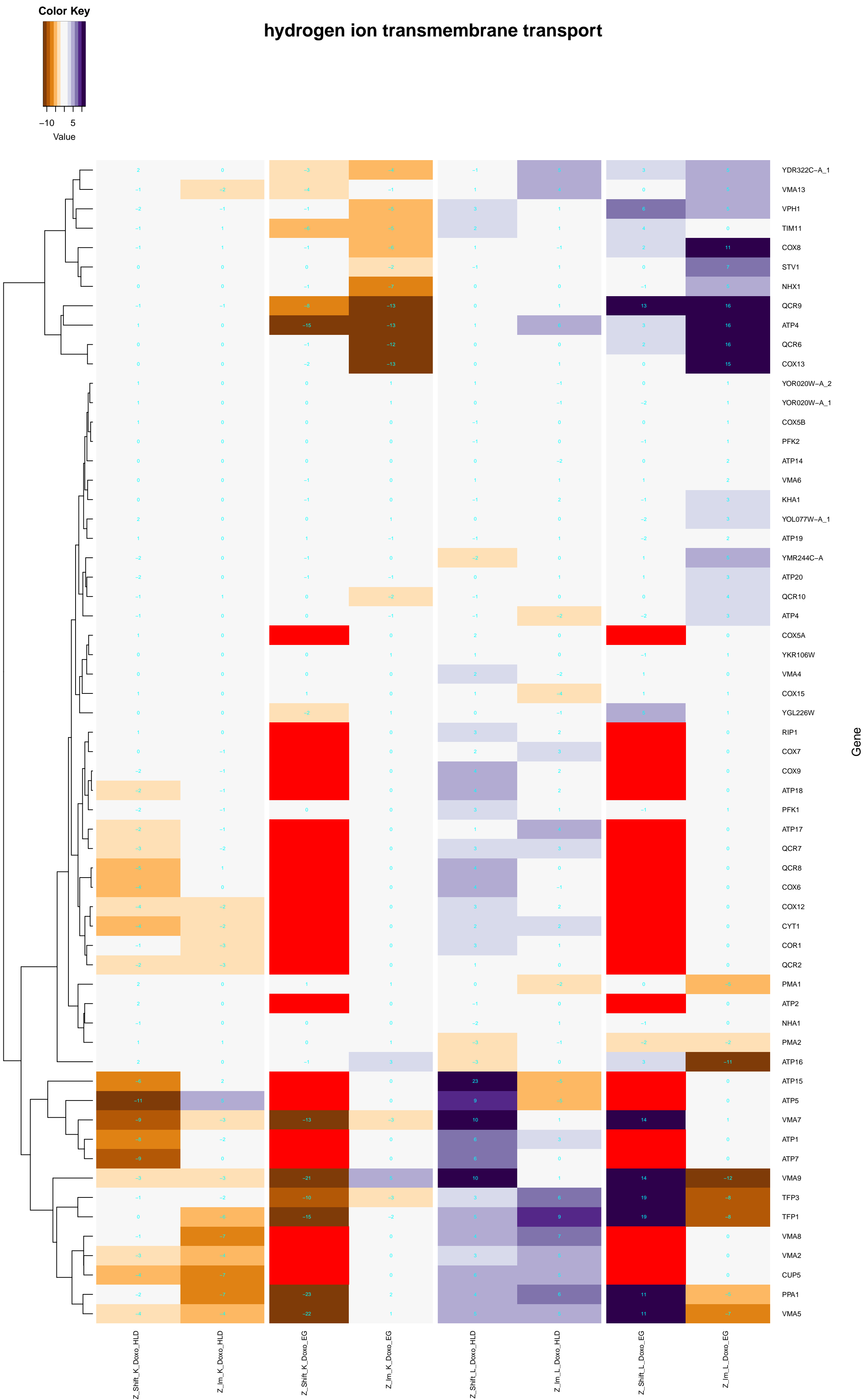

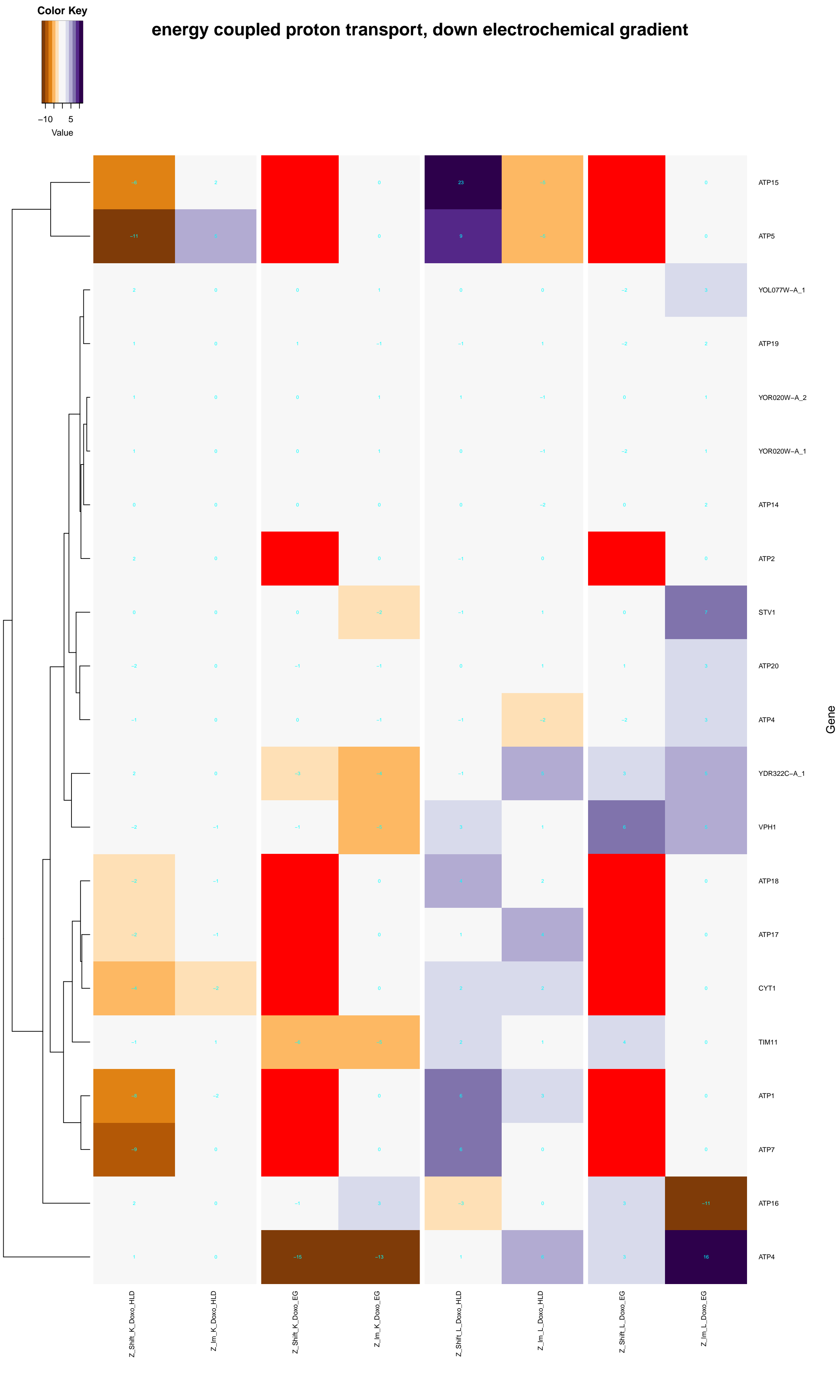

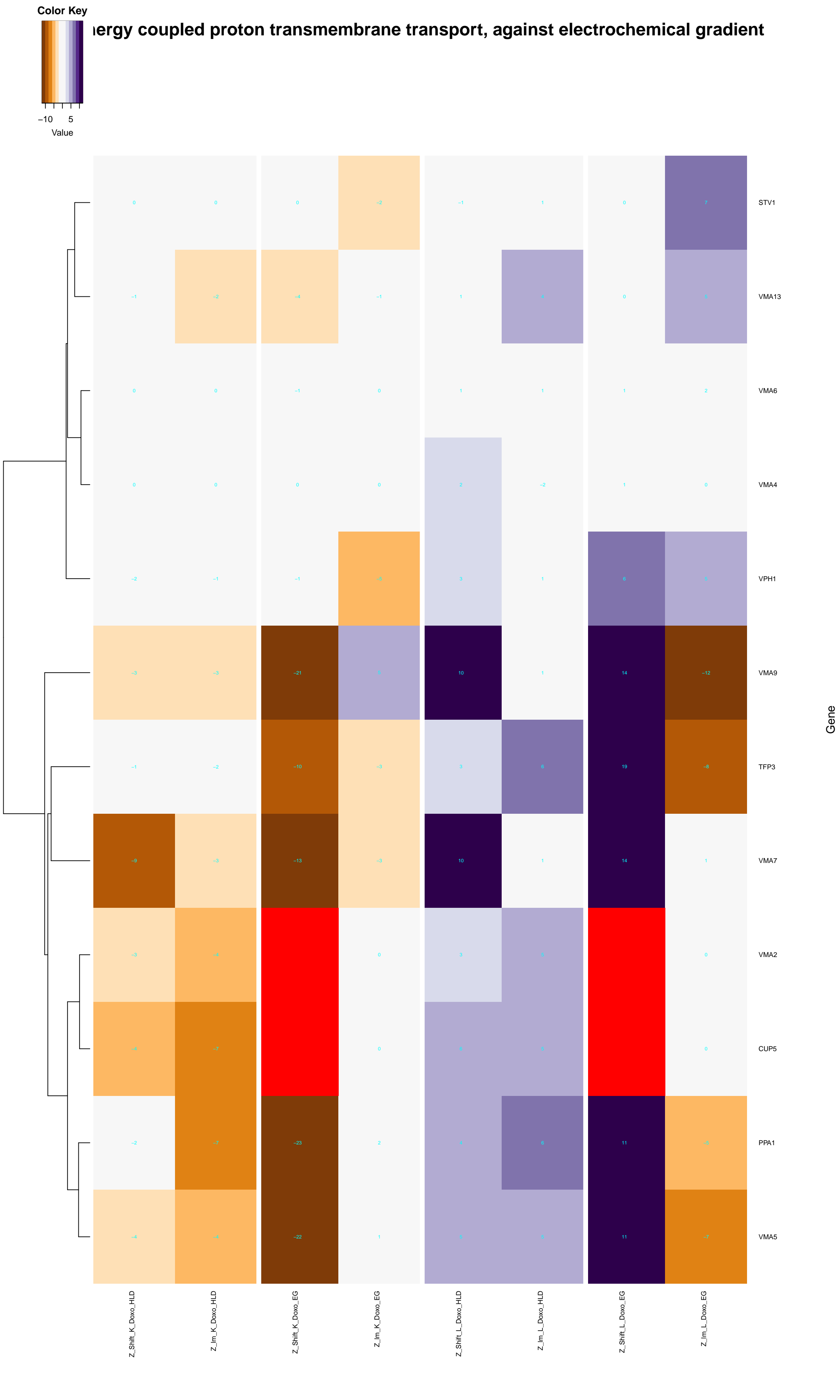

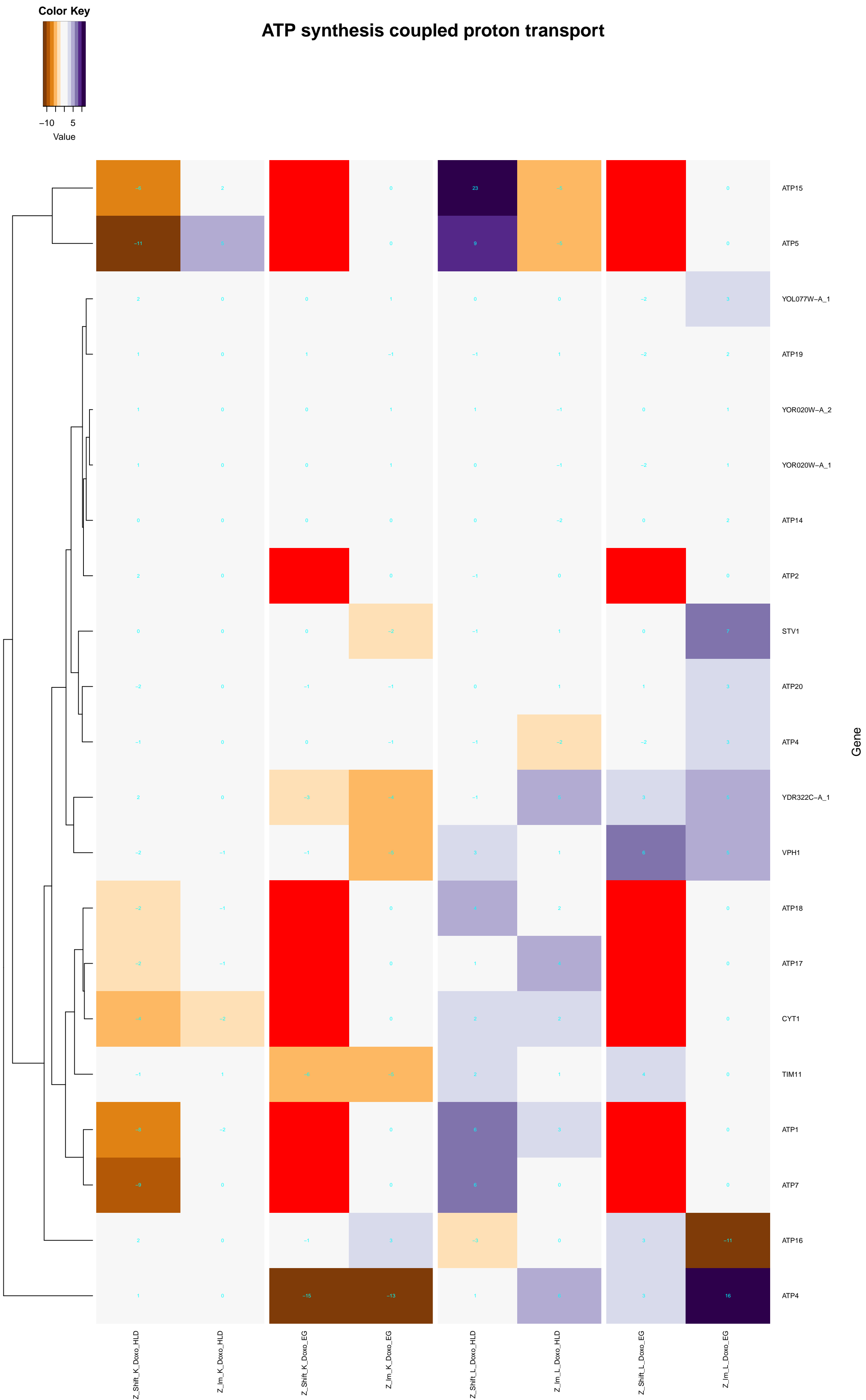

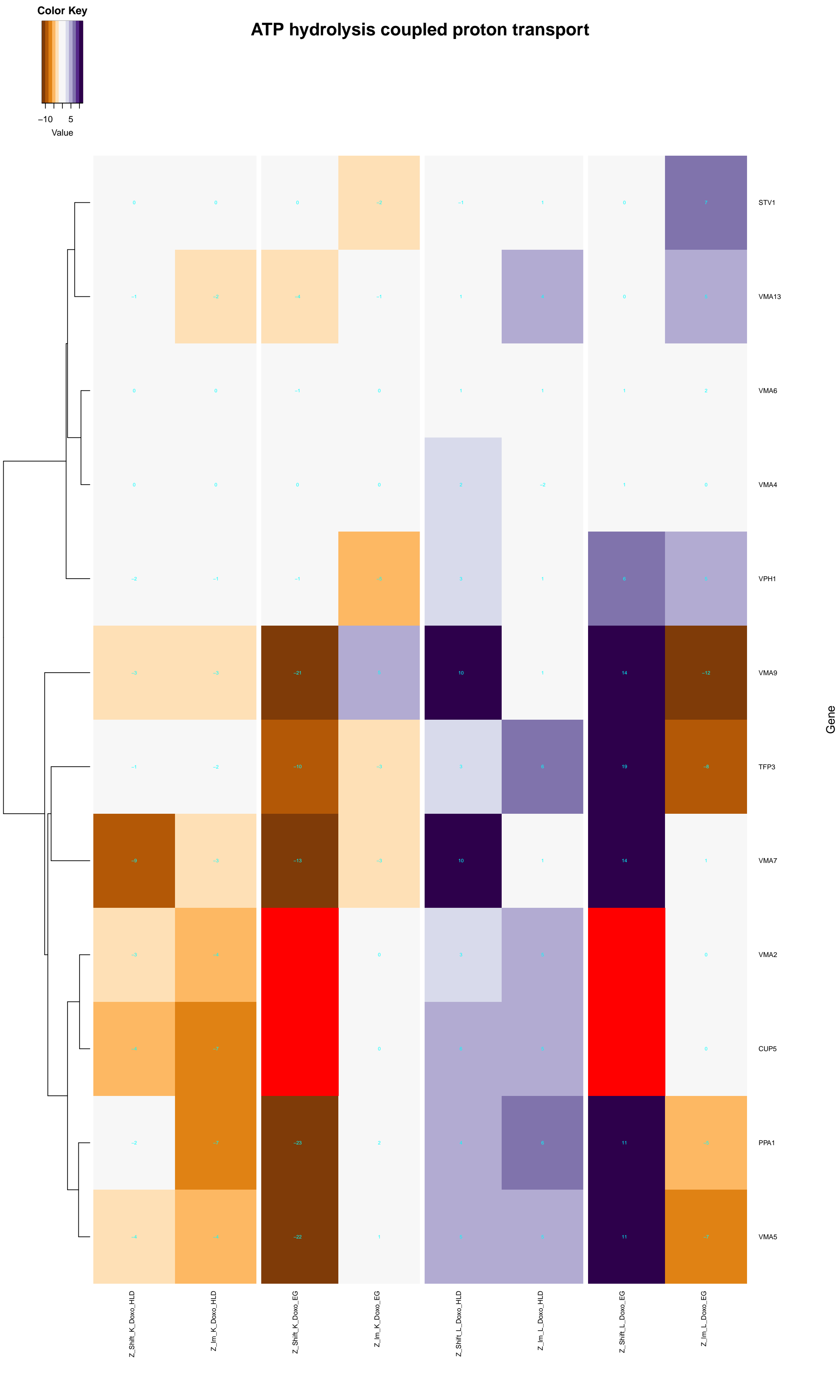

Supplement: Supplementary file 9 — Additional file 9. GO term-specific heatmaps for REMc/GTF-enriched clusters. GO term-specific heatmaps for significant GO process terms were generated as described in methods and Figs. 3 and 4. Any related child terms are presented in subsequent pages of the parent file name. GO terms with more than 100 children, with 2 or fewer genes annotated to the term, or a file size over 300KB are not shown. All heatmaps are generated with the same layout (see Figs. 3 and 4). [file 40170_2019_201_MOESM9_ESM.bz2 › Additional_File9_GOTermHeatmaps/Additional_File9_GOTermHeatmap/hydrogen_ion_transmembrane_transport.pdf]

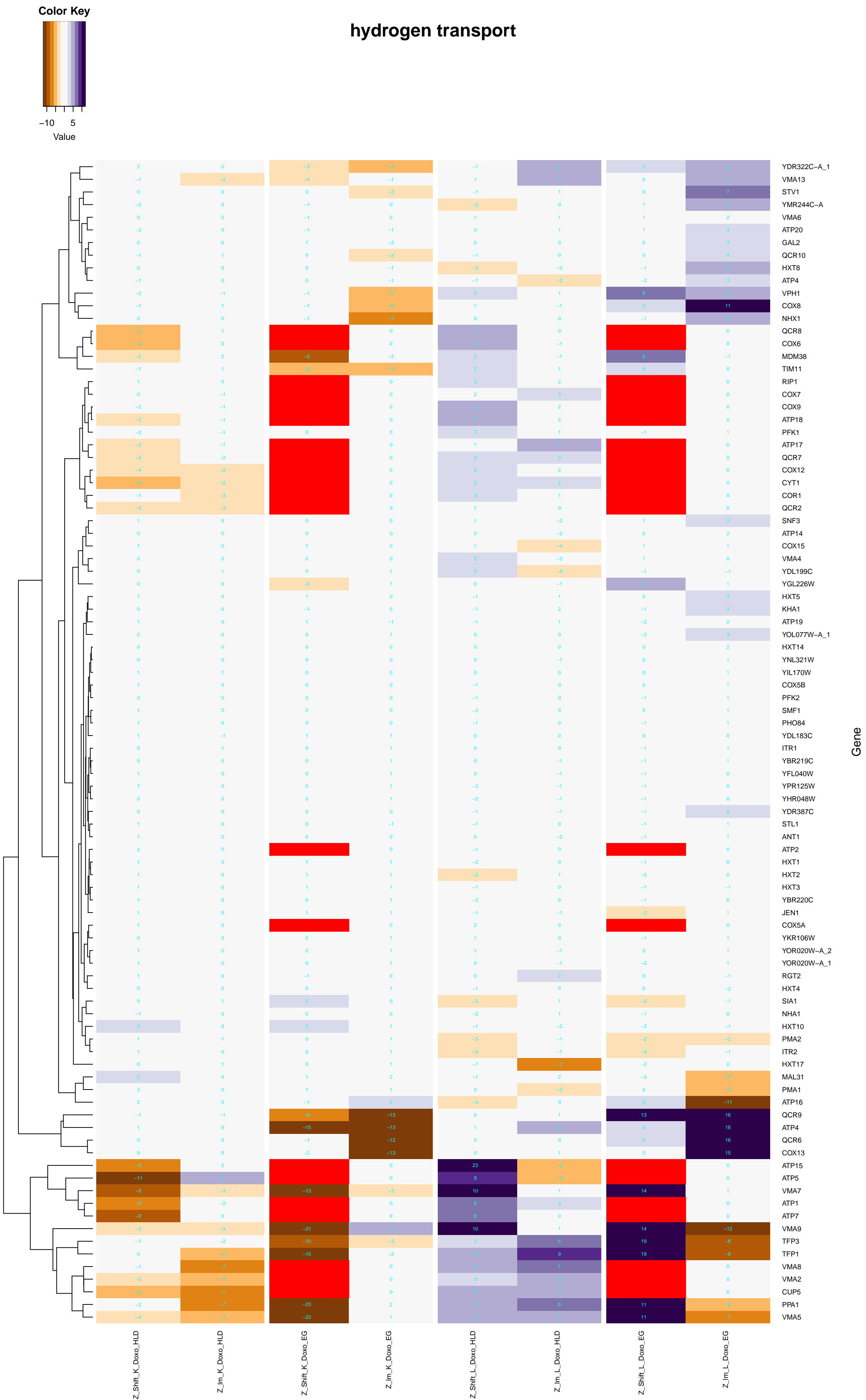

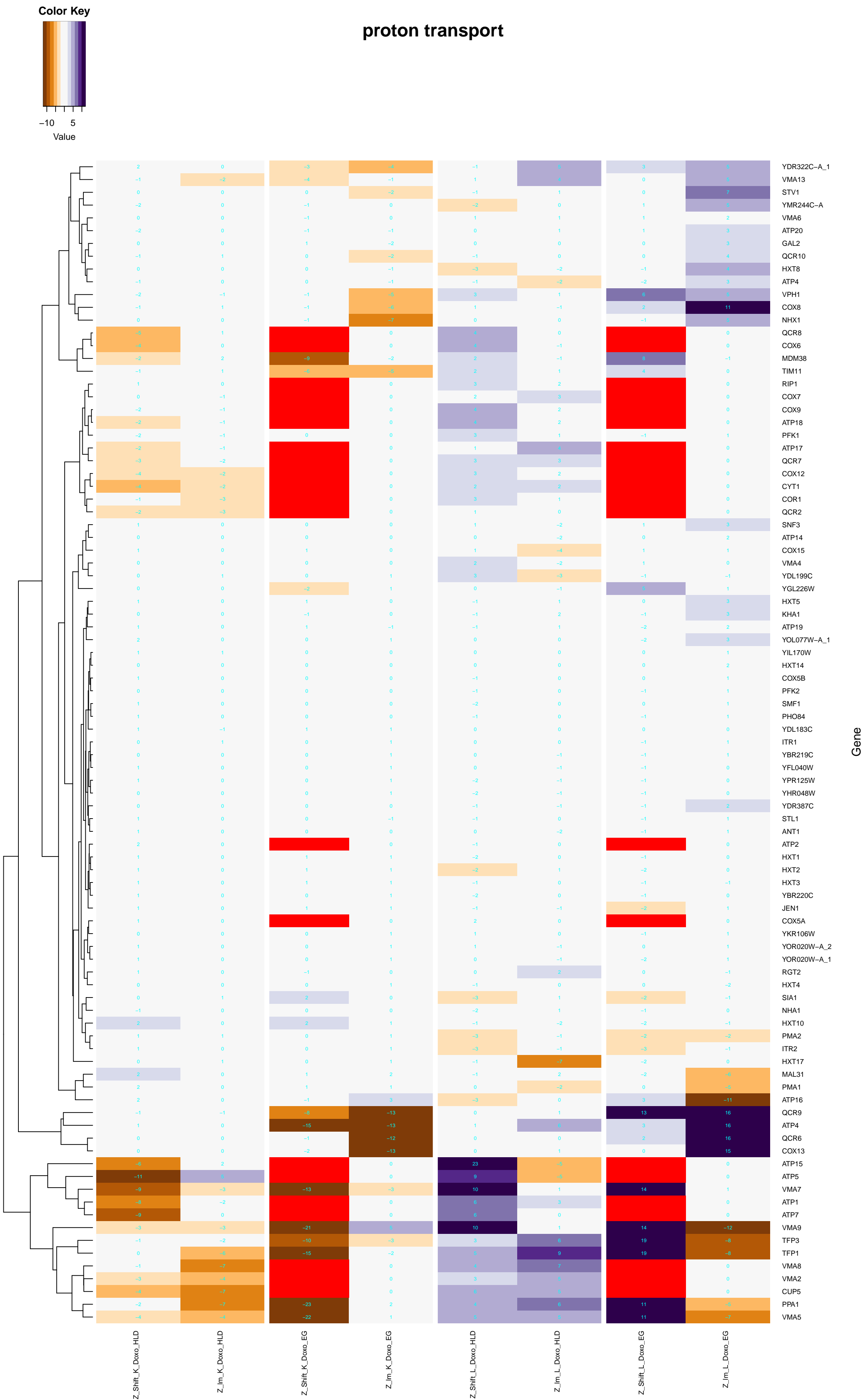

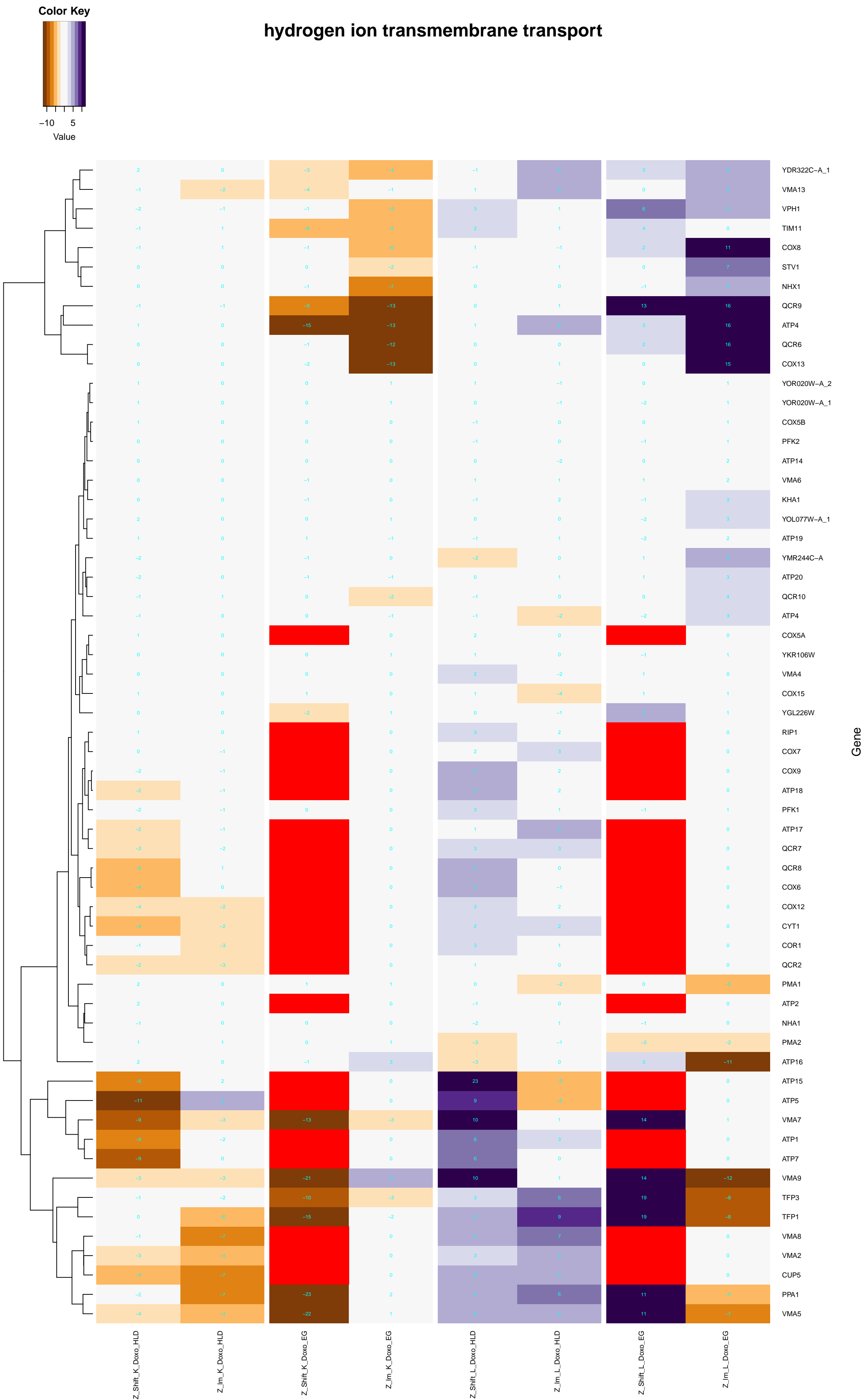

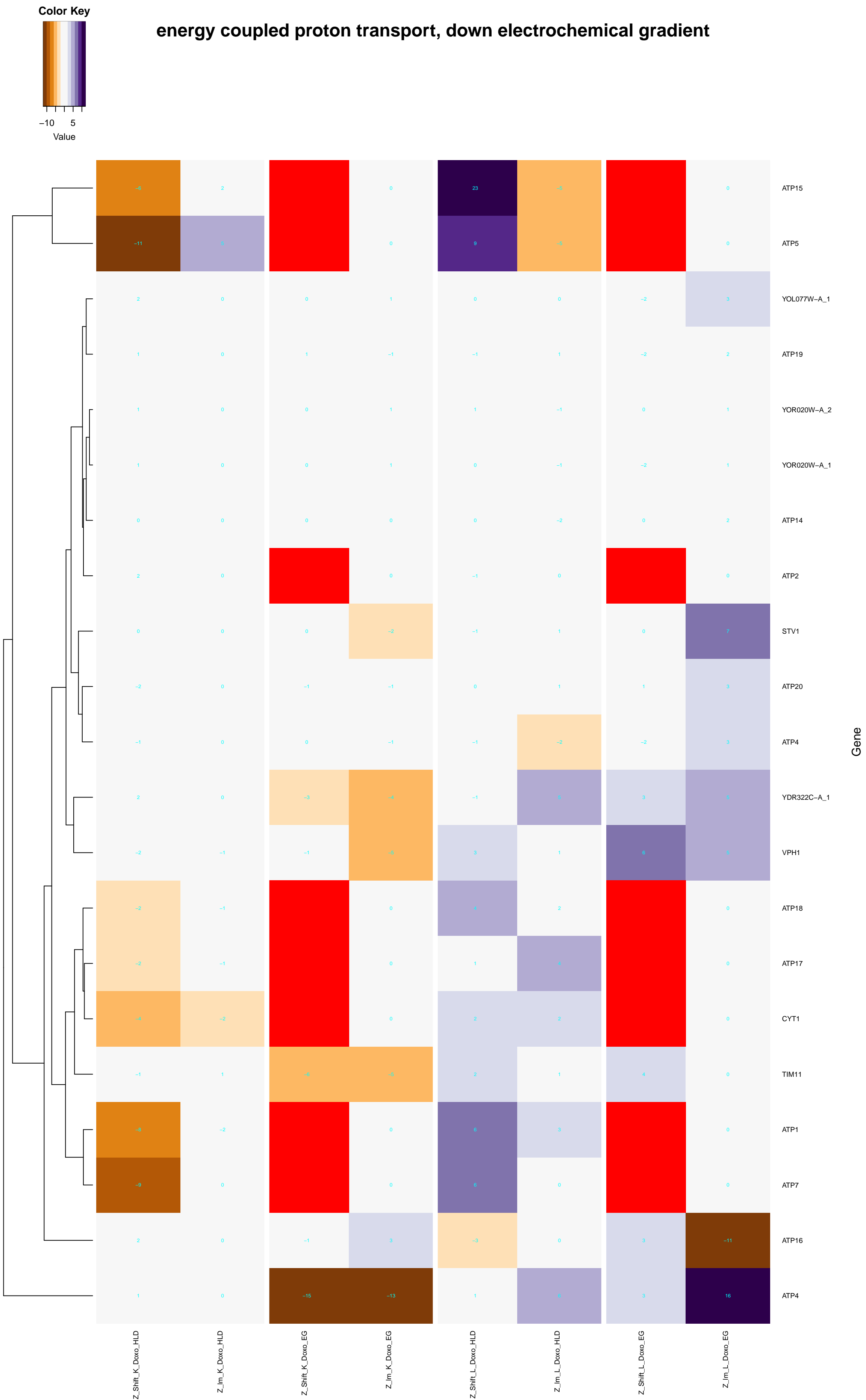

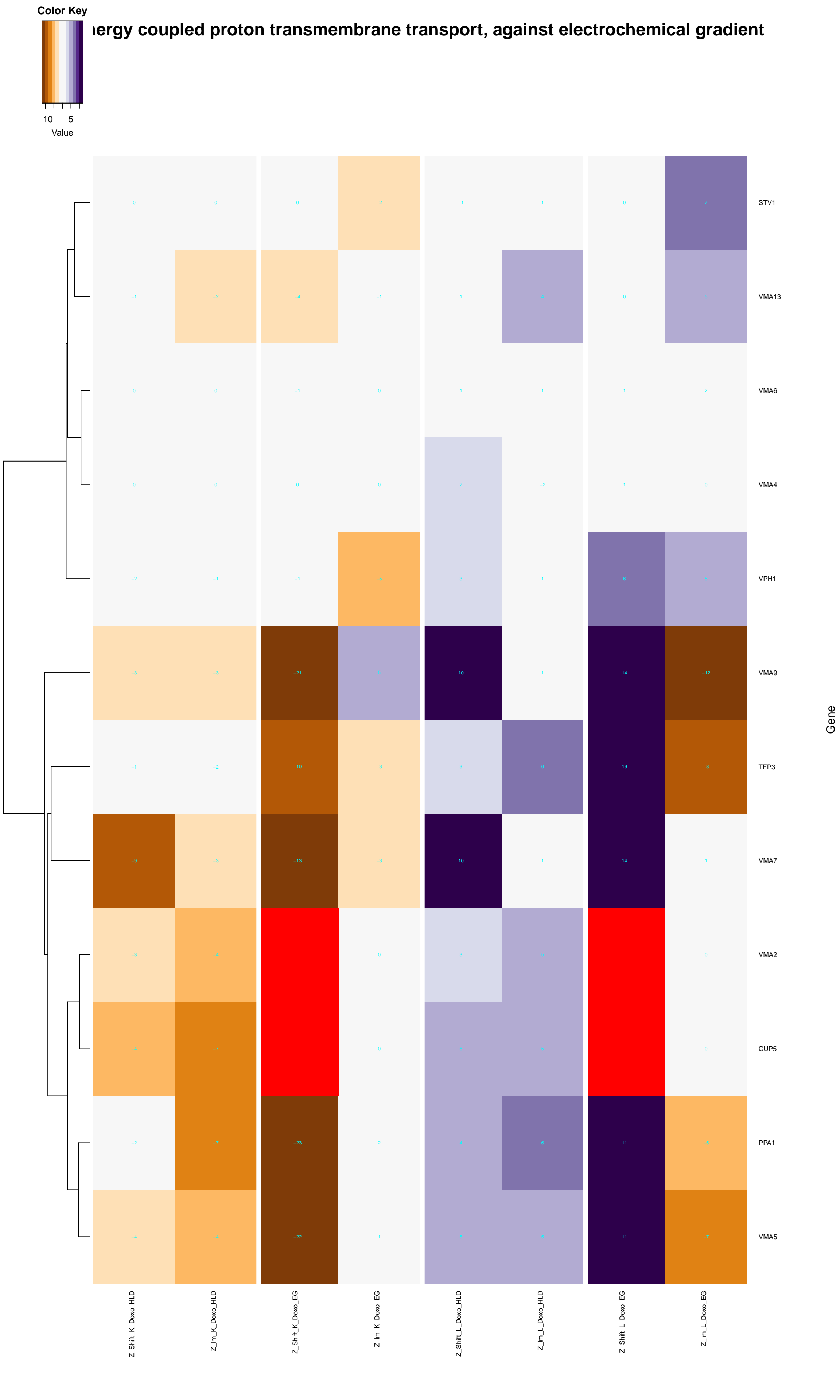

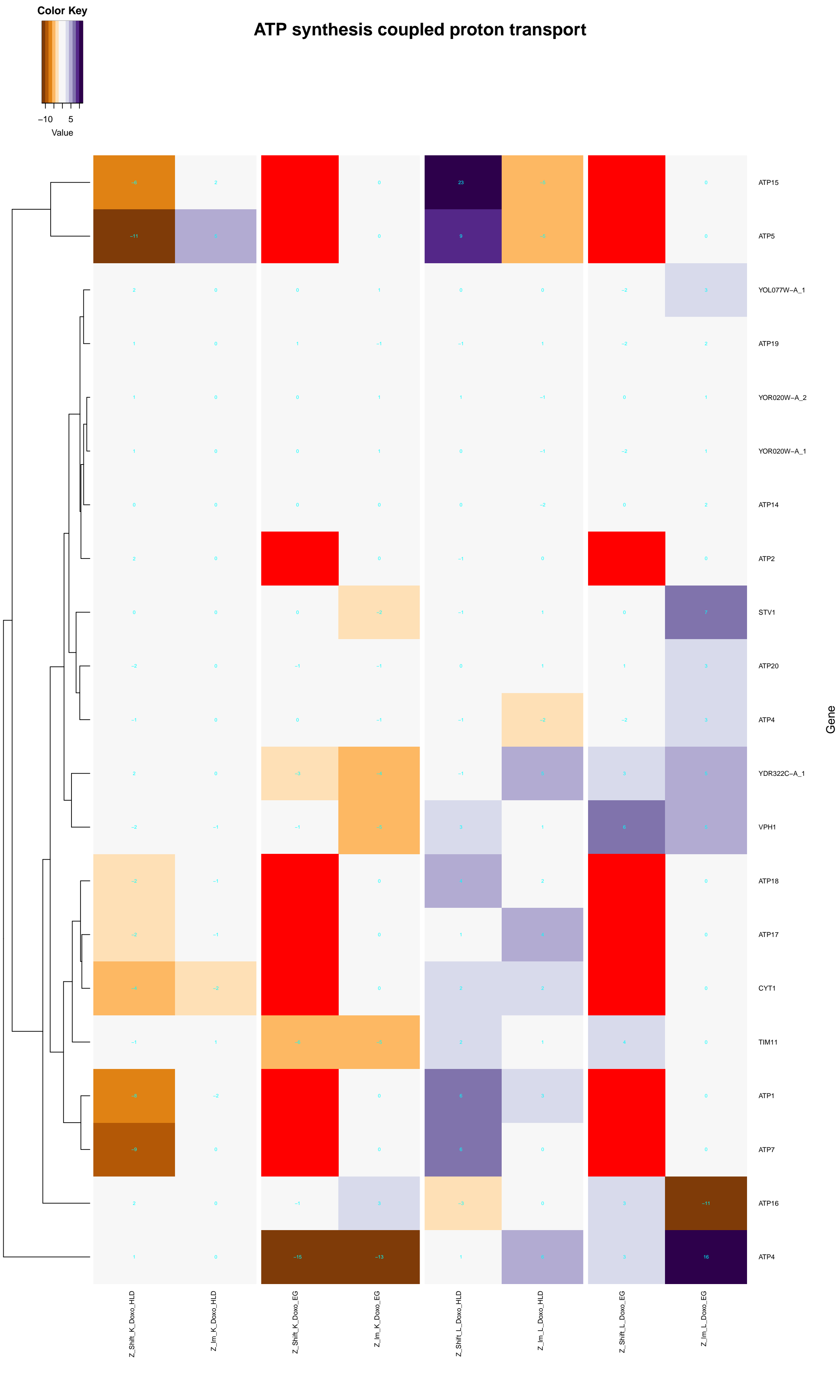

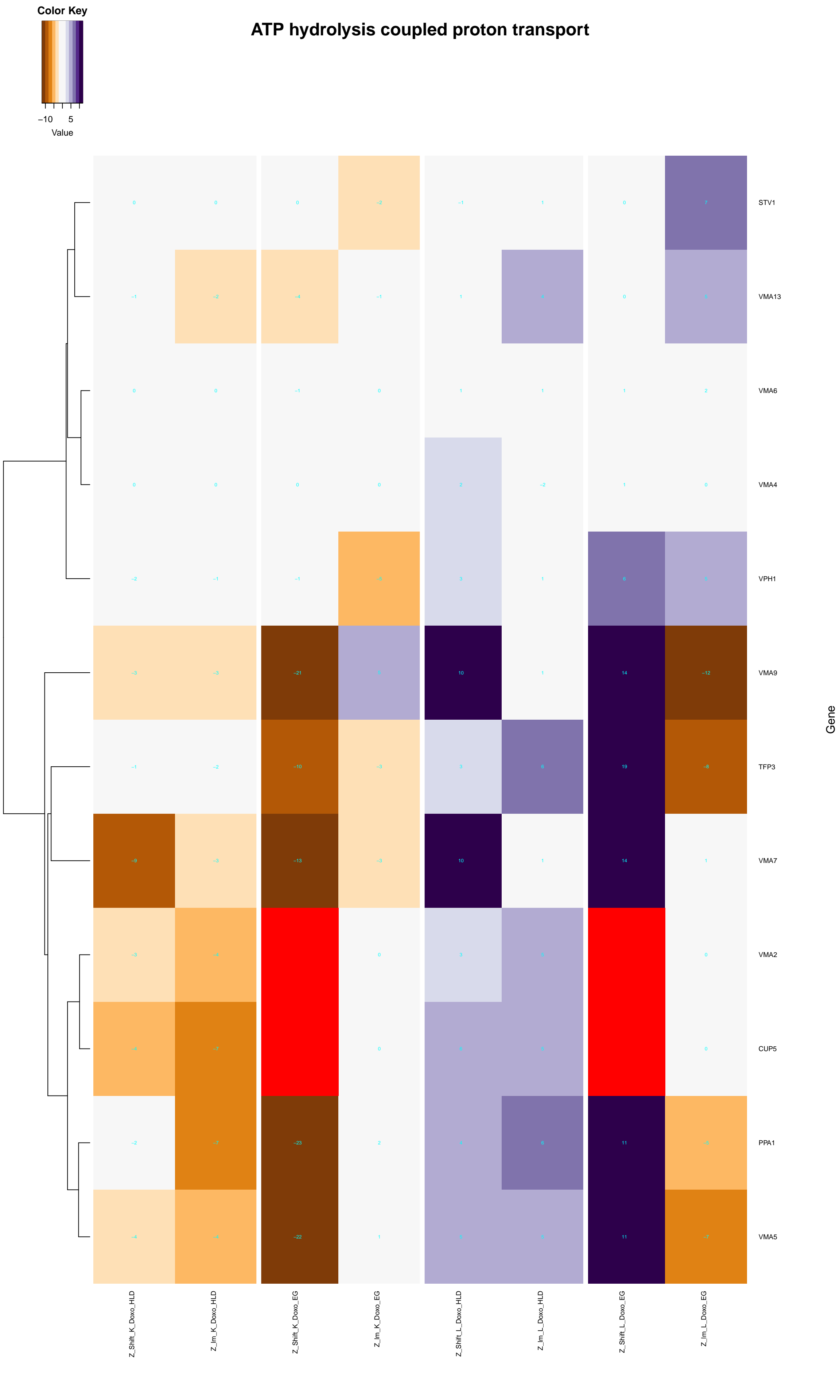

Supplement: Supplementary file 9 — Additional file 9. GO term-specific heatmaps for REMc/GTF-enriched clusters. GO term-specific heatmaps for significant GO process terms were generated as described in methods and Figs. 3 and 4. Any related child terms are presented in subsequent pages of the parent file name. GO terms with more than 100 children, with 2 or fewer genes annotated to the term, or a file size over 300KB are not shown. All heatmaps are generated with the same layout (see Figs. 3 and 4). [file 40170_2019_201_MOESM9_ESM.bz2 › Additional_File9_GOTermHeatmaps/Additional_File9_GOTermHeatmap/hydrogen_transport.pdf]

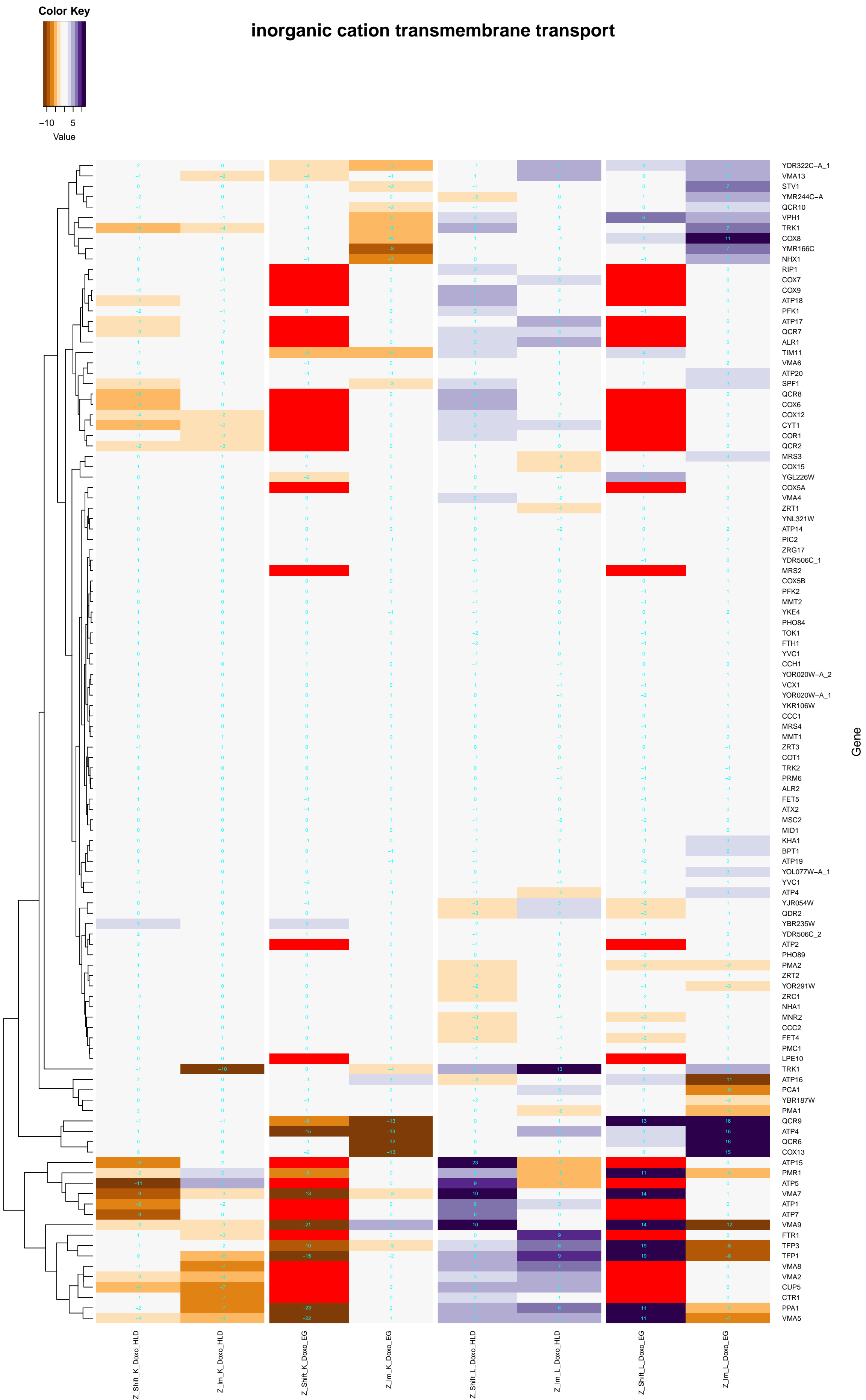

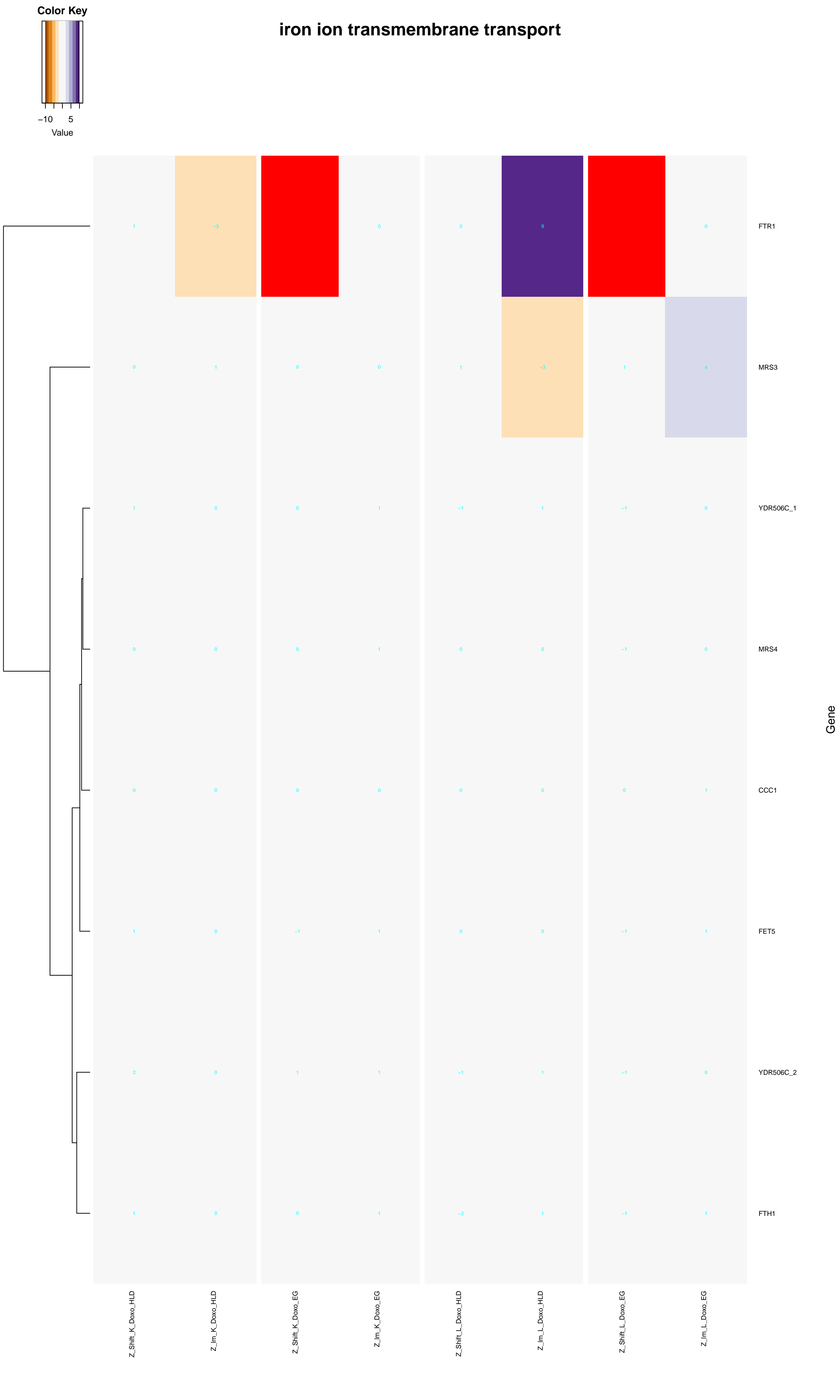

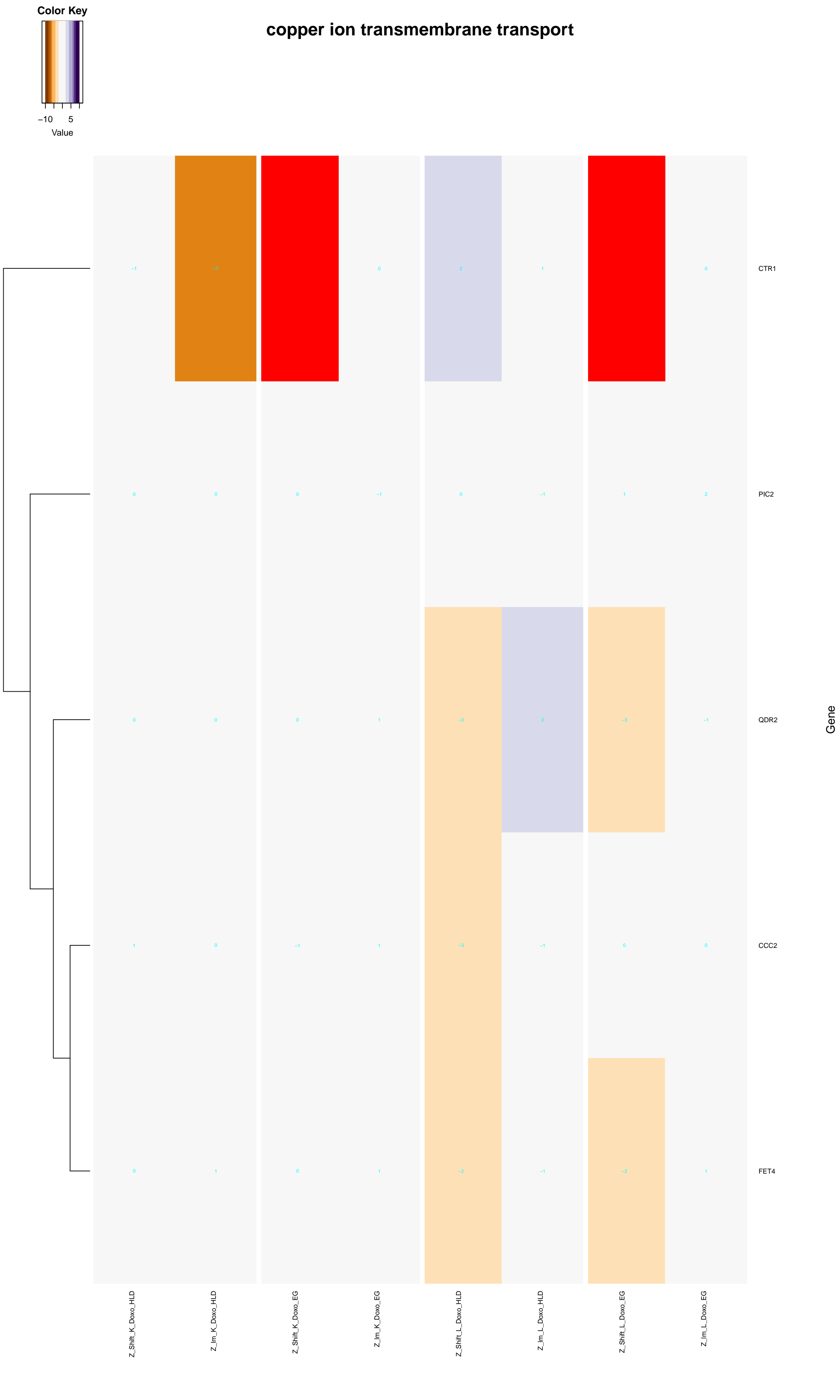

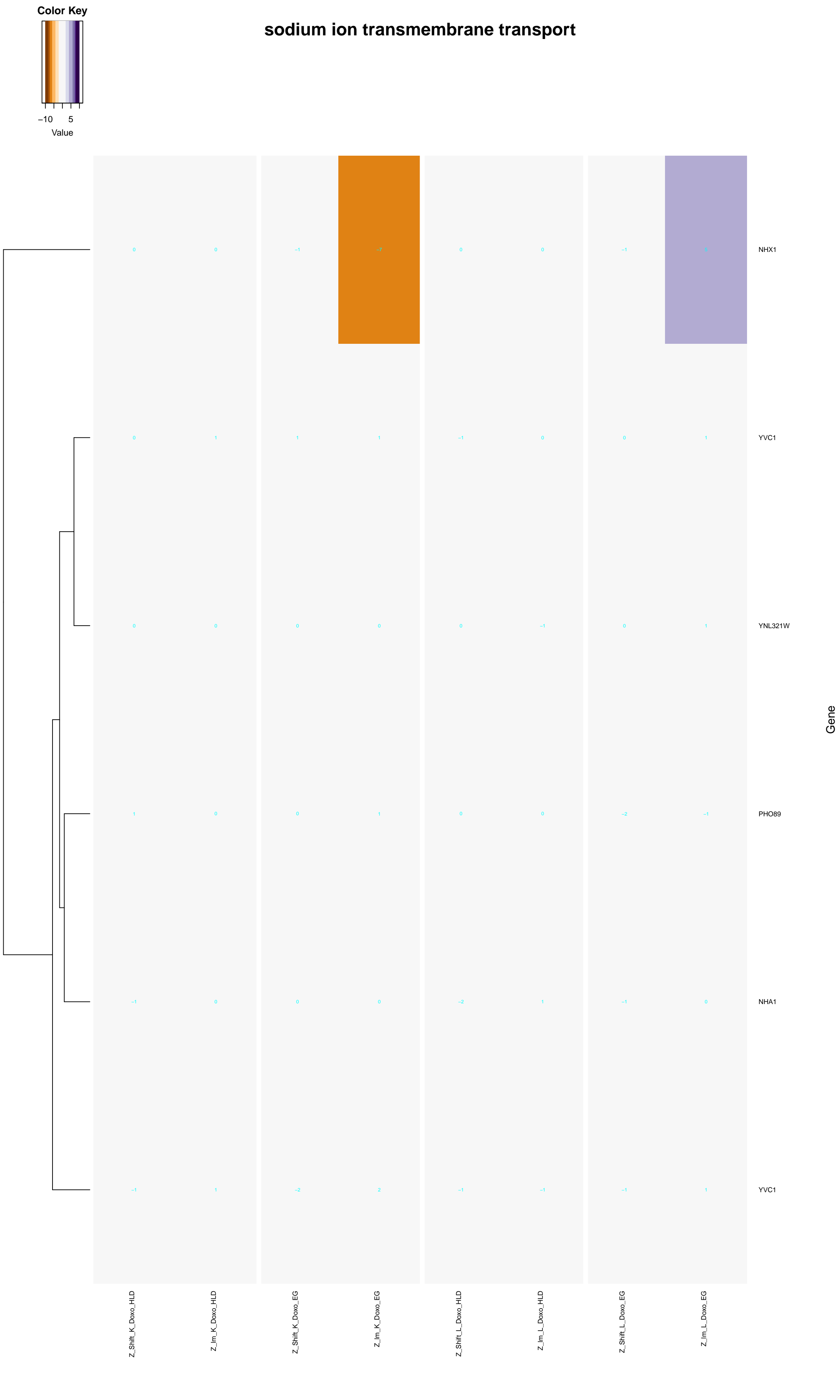

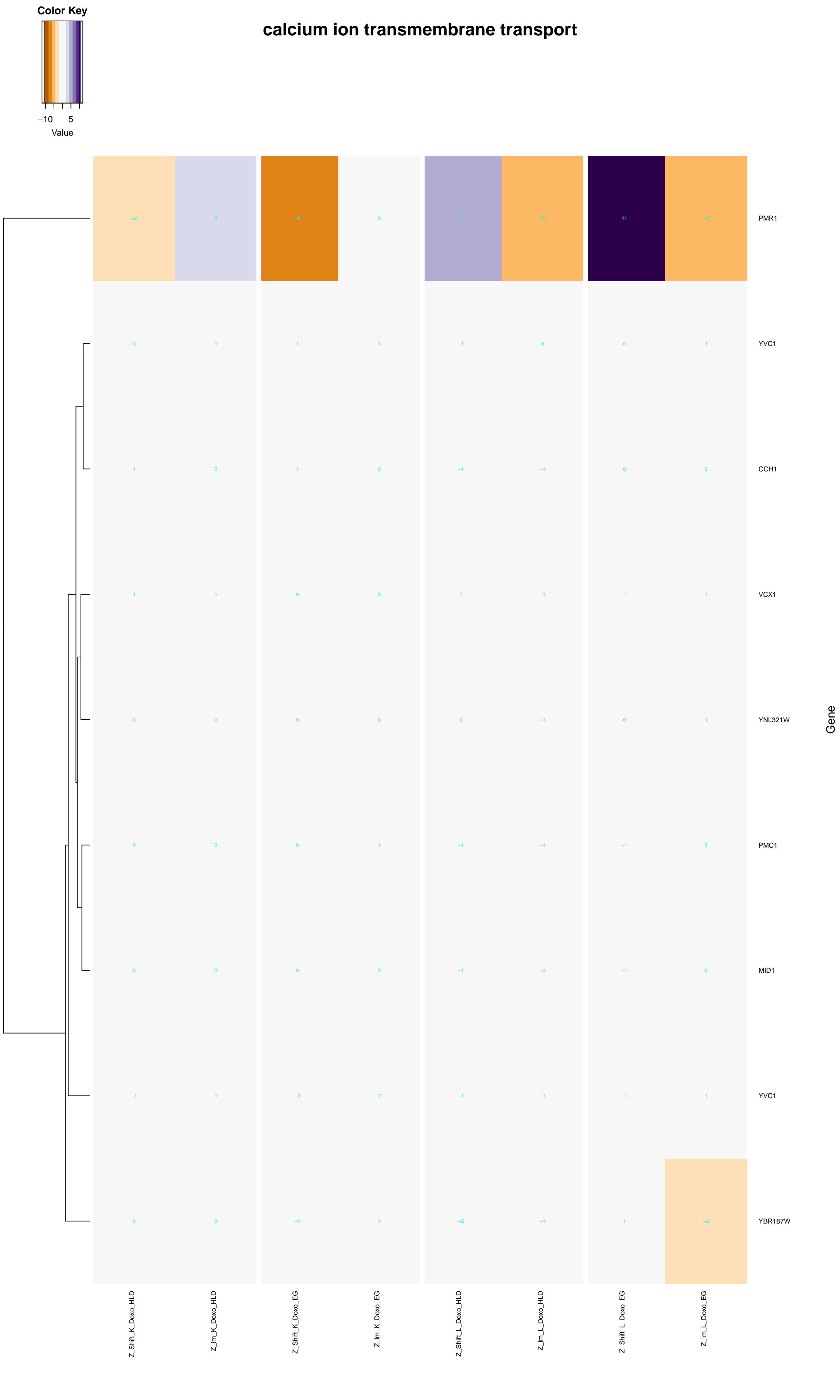

## manganese ion transmembrane transport

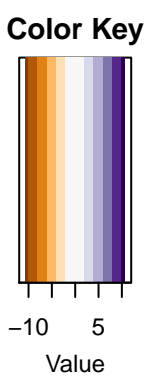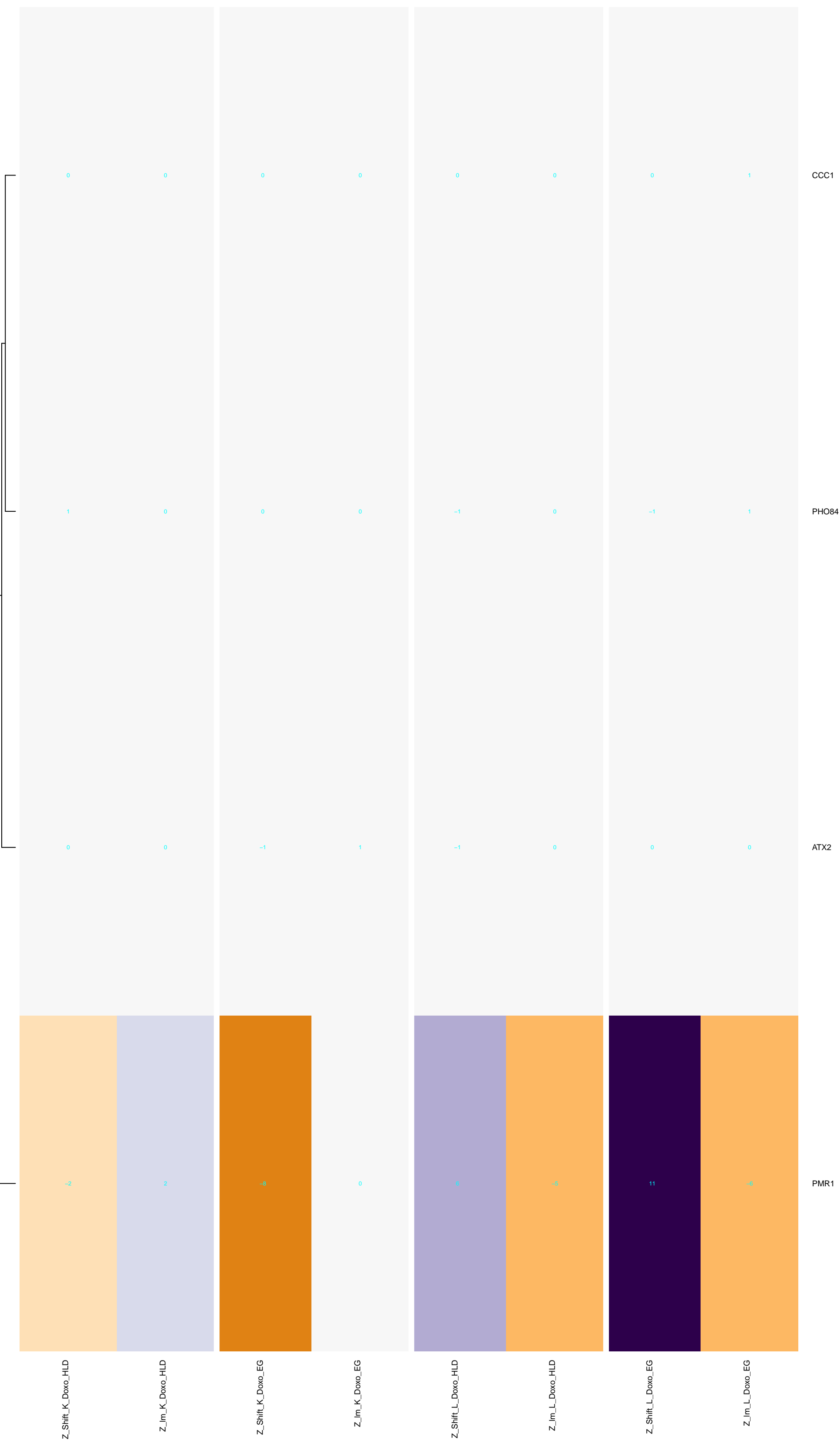

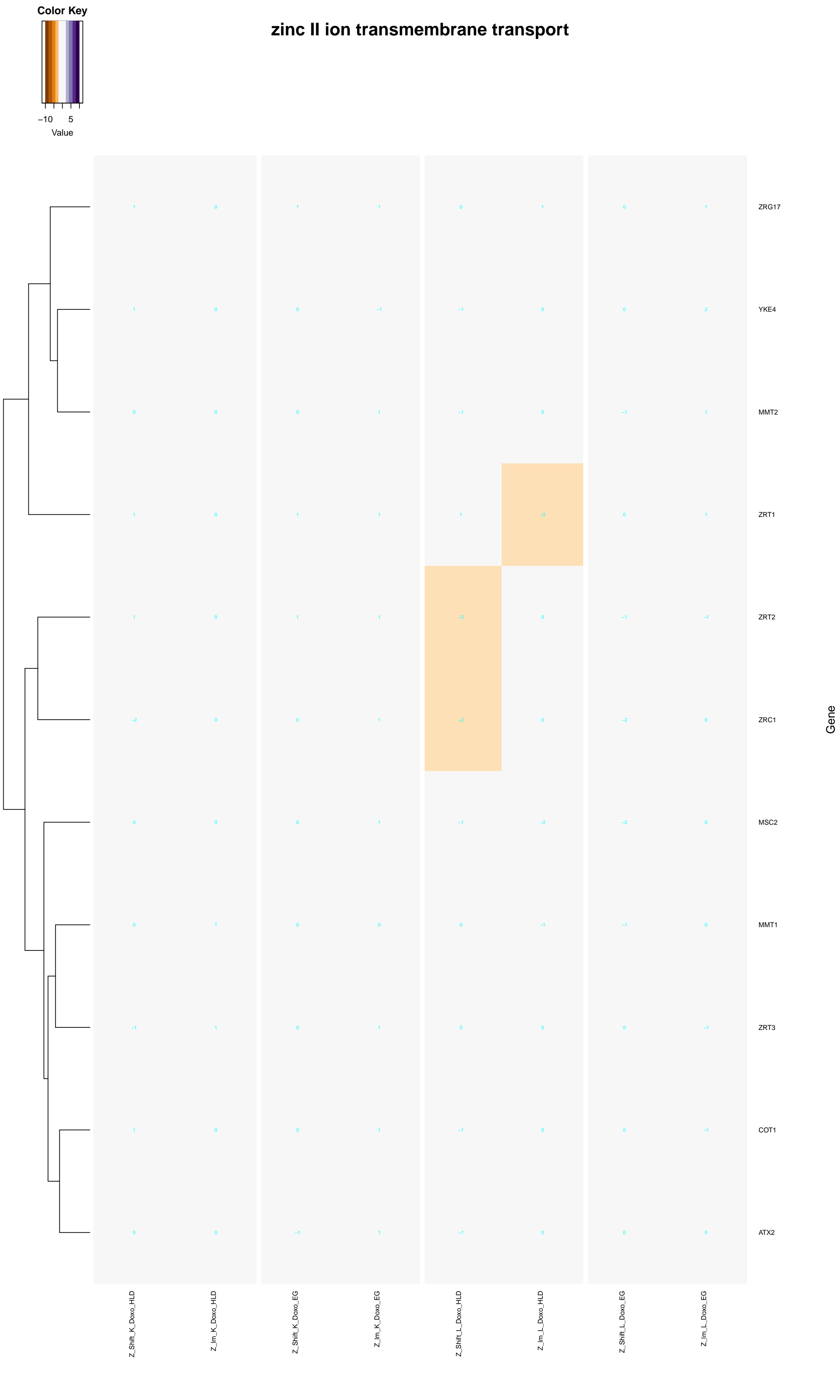

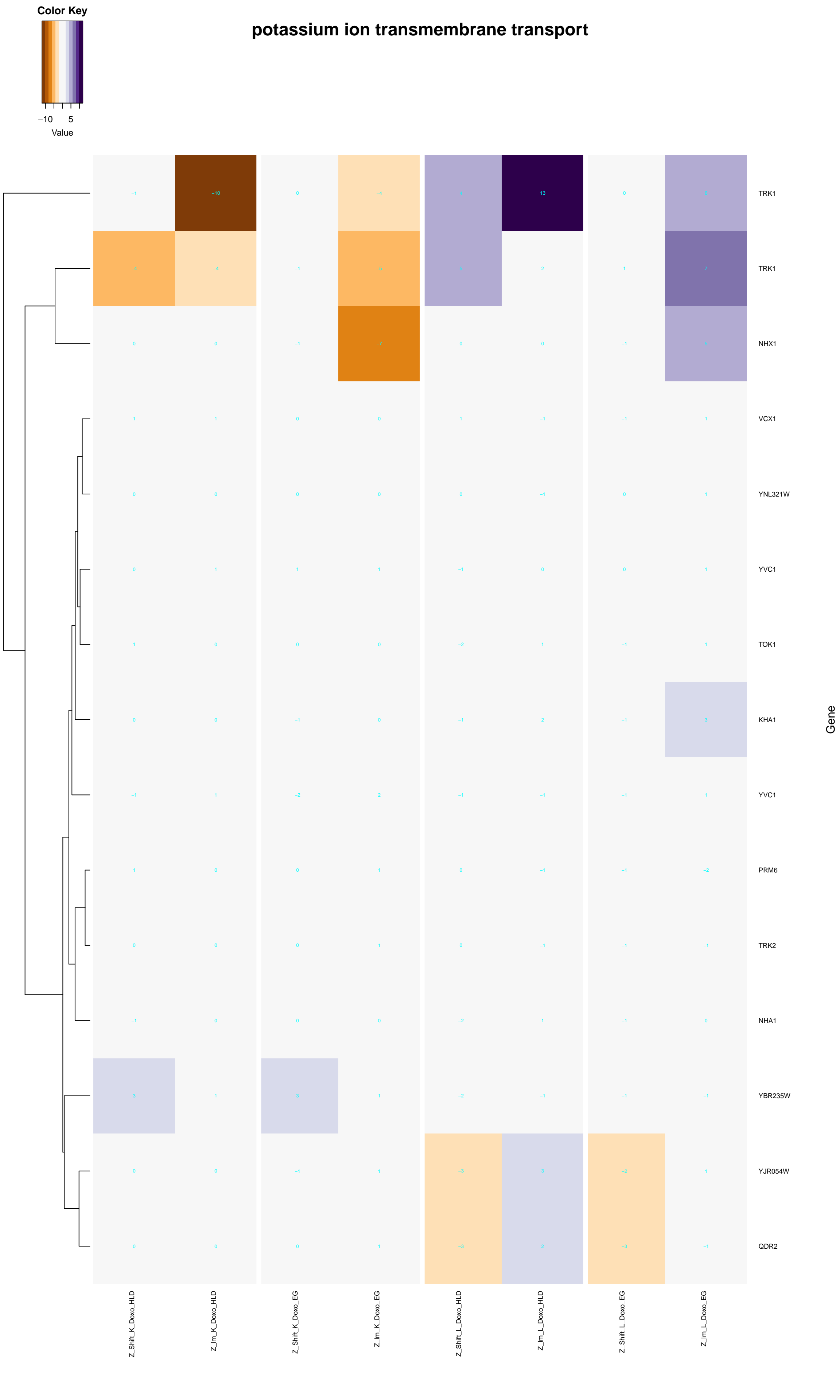

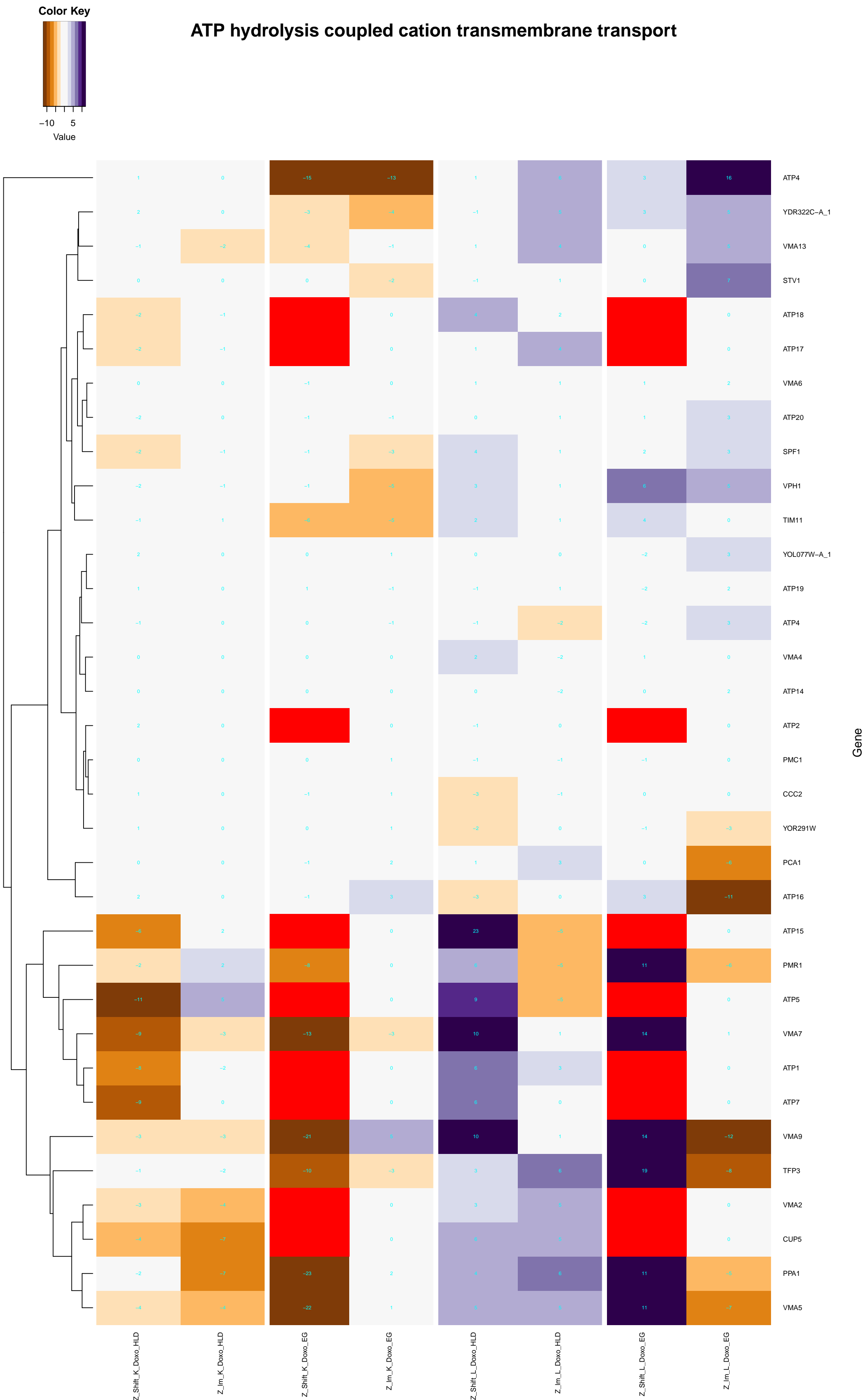

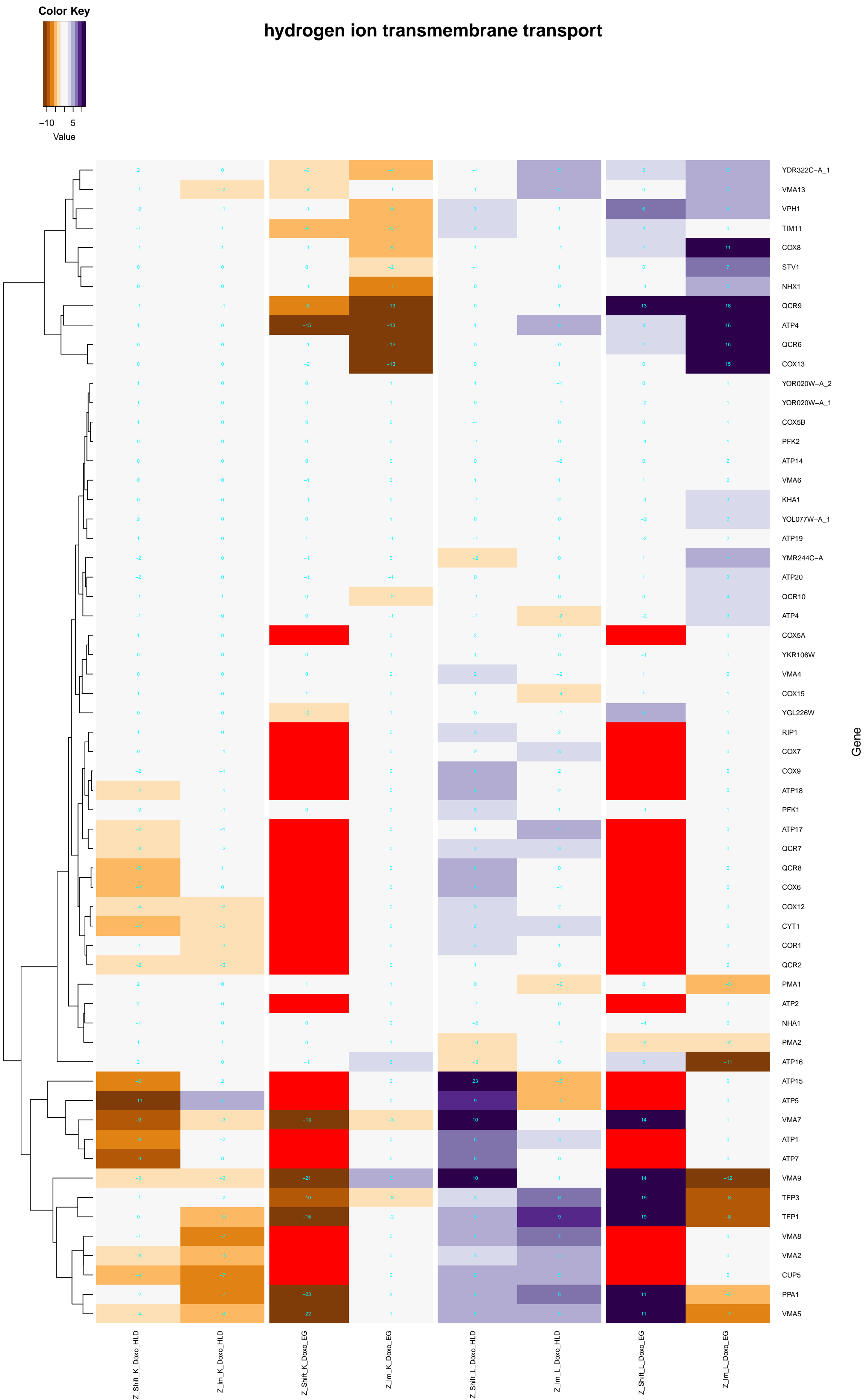

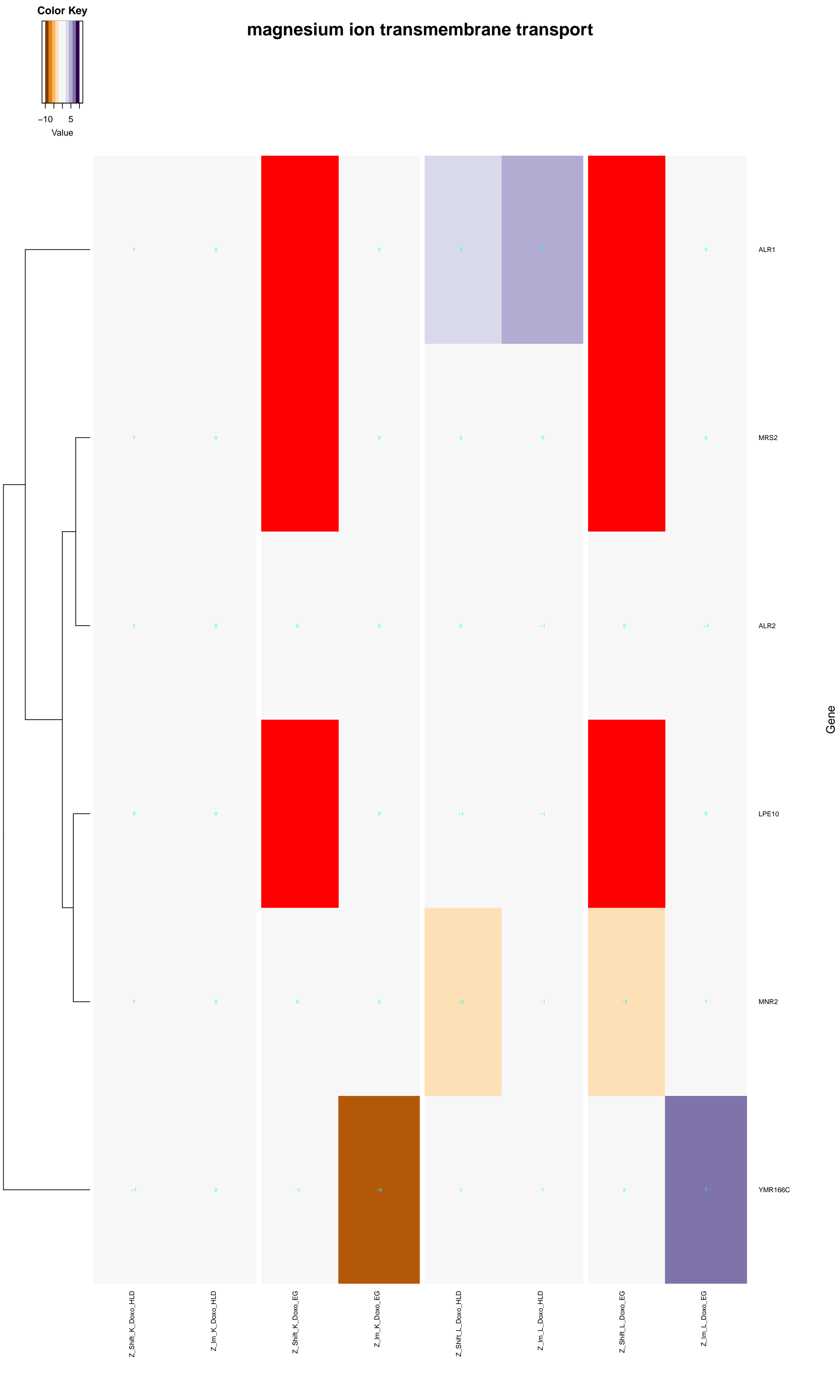

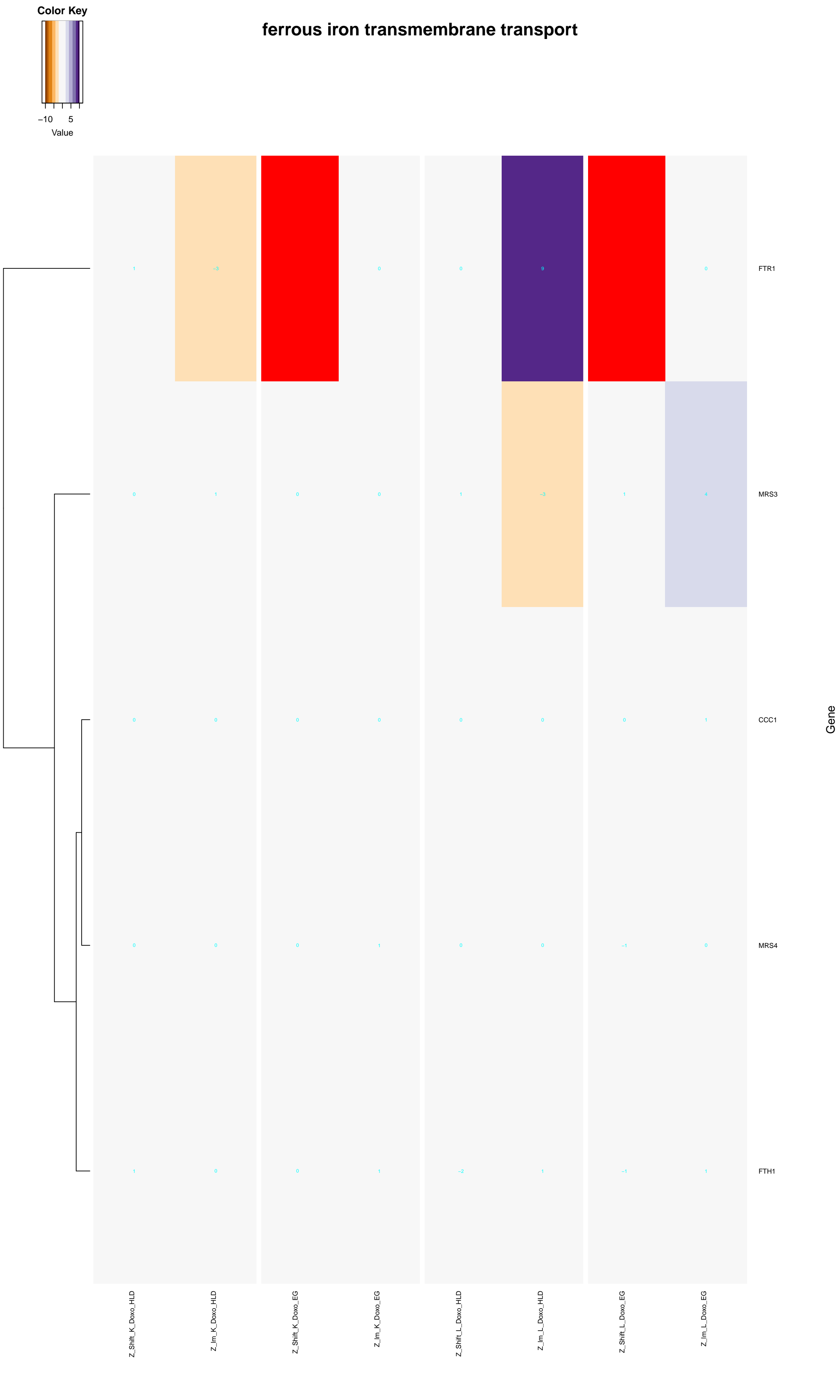

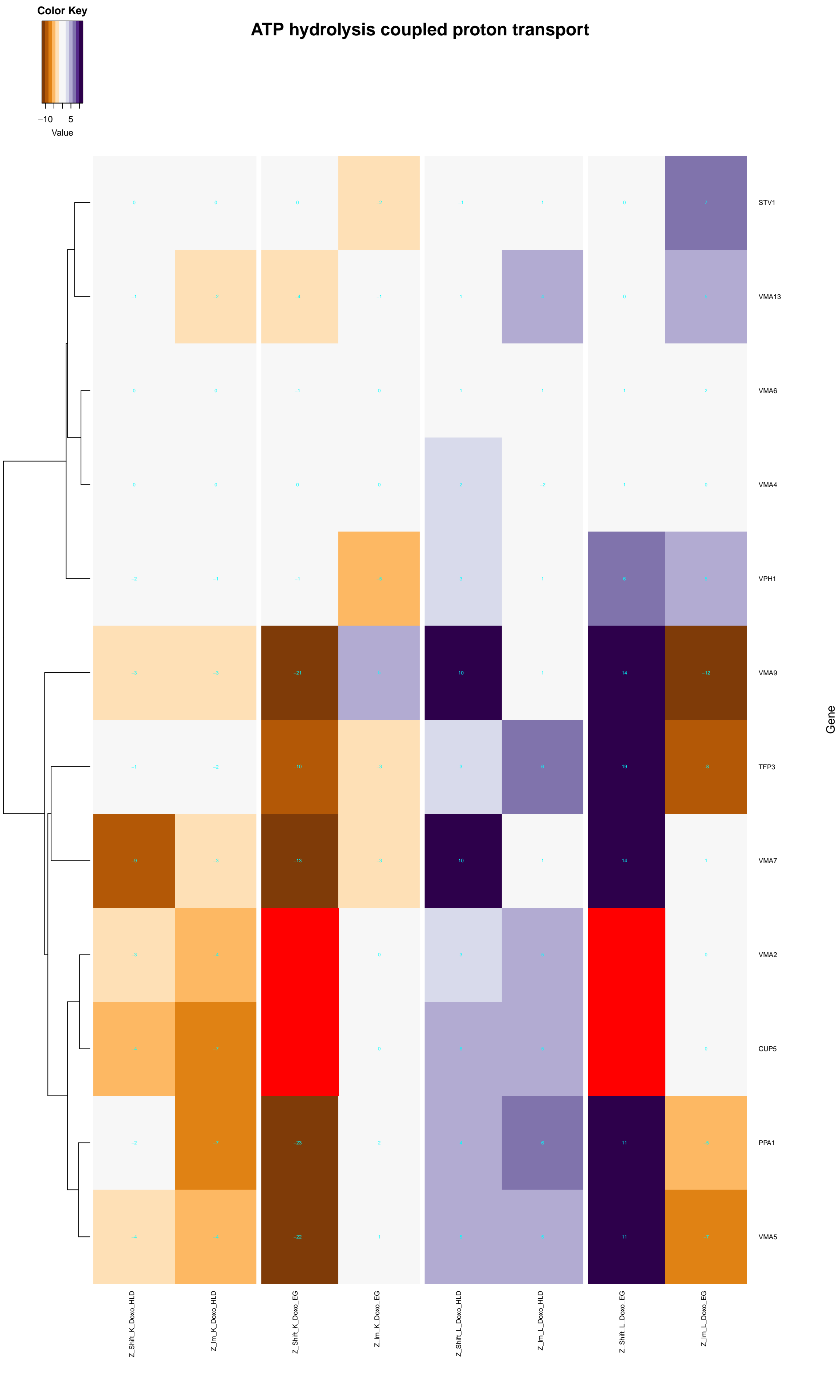

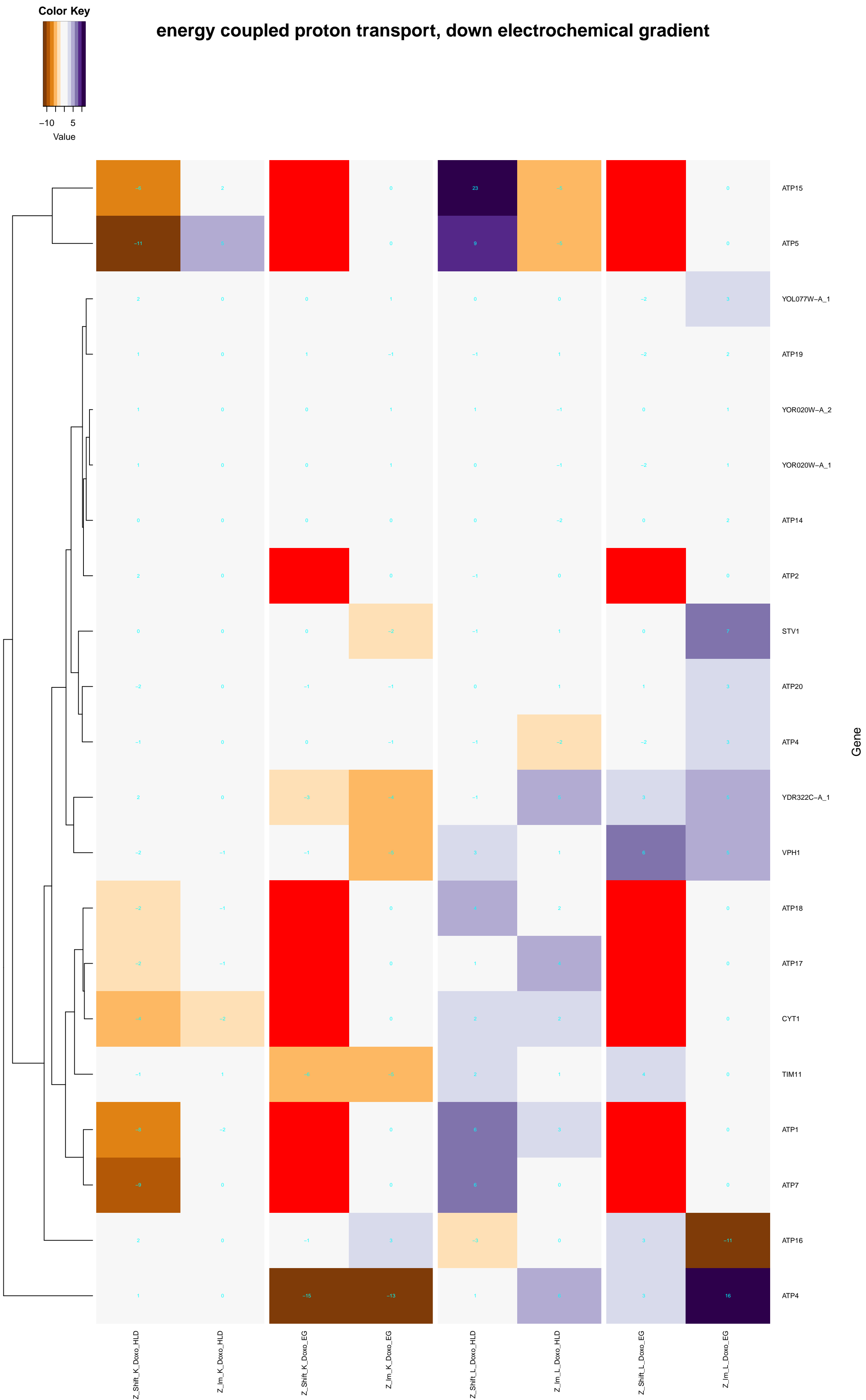

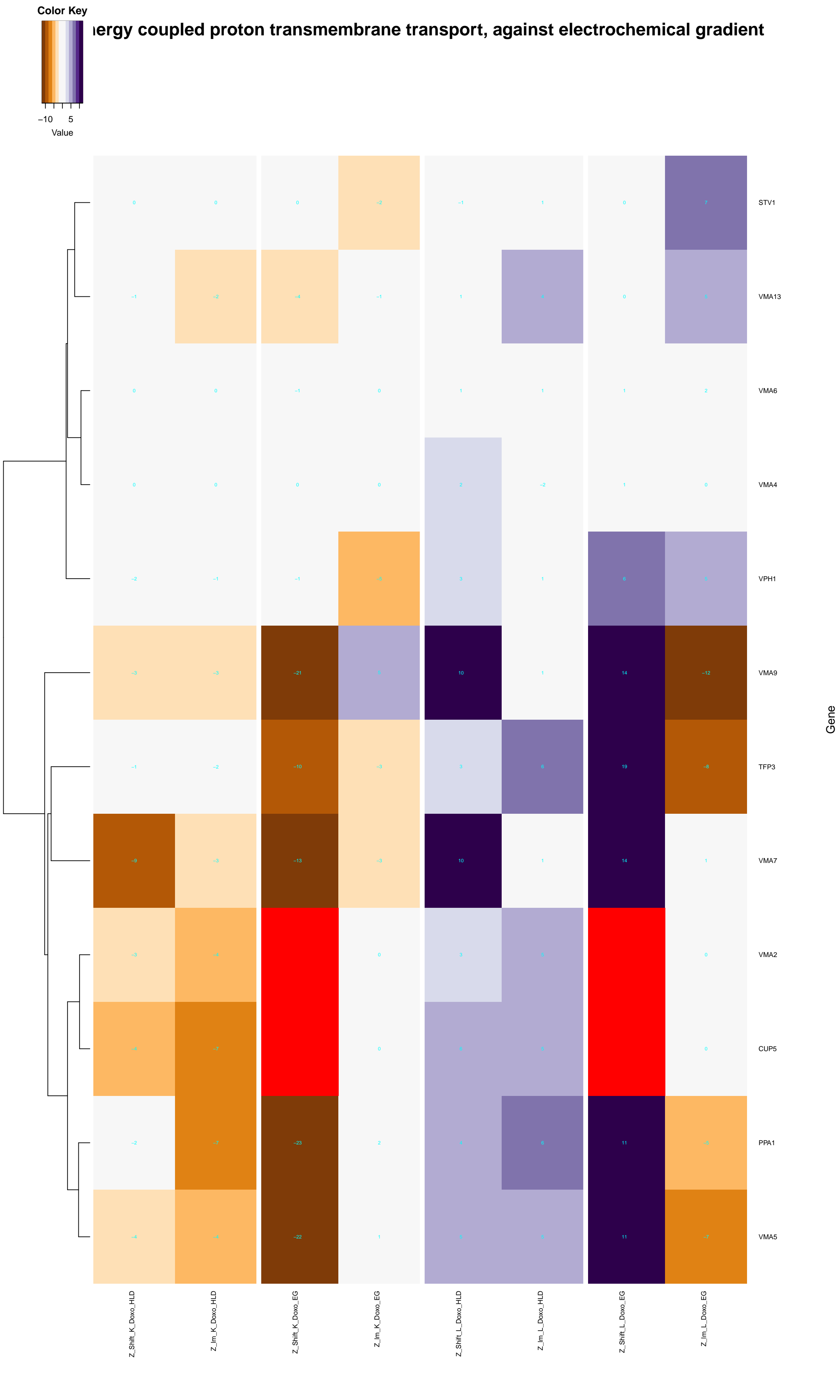

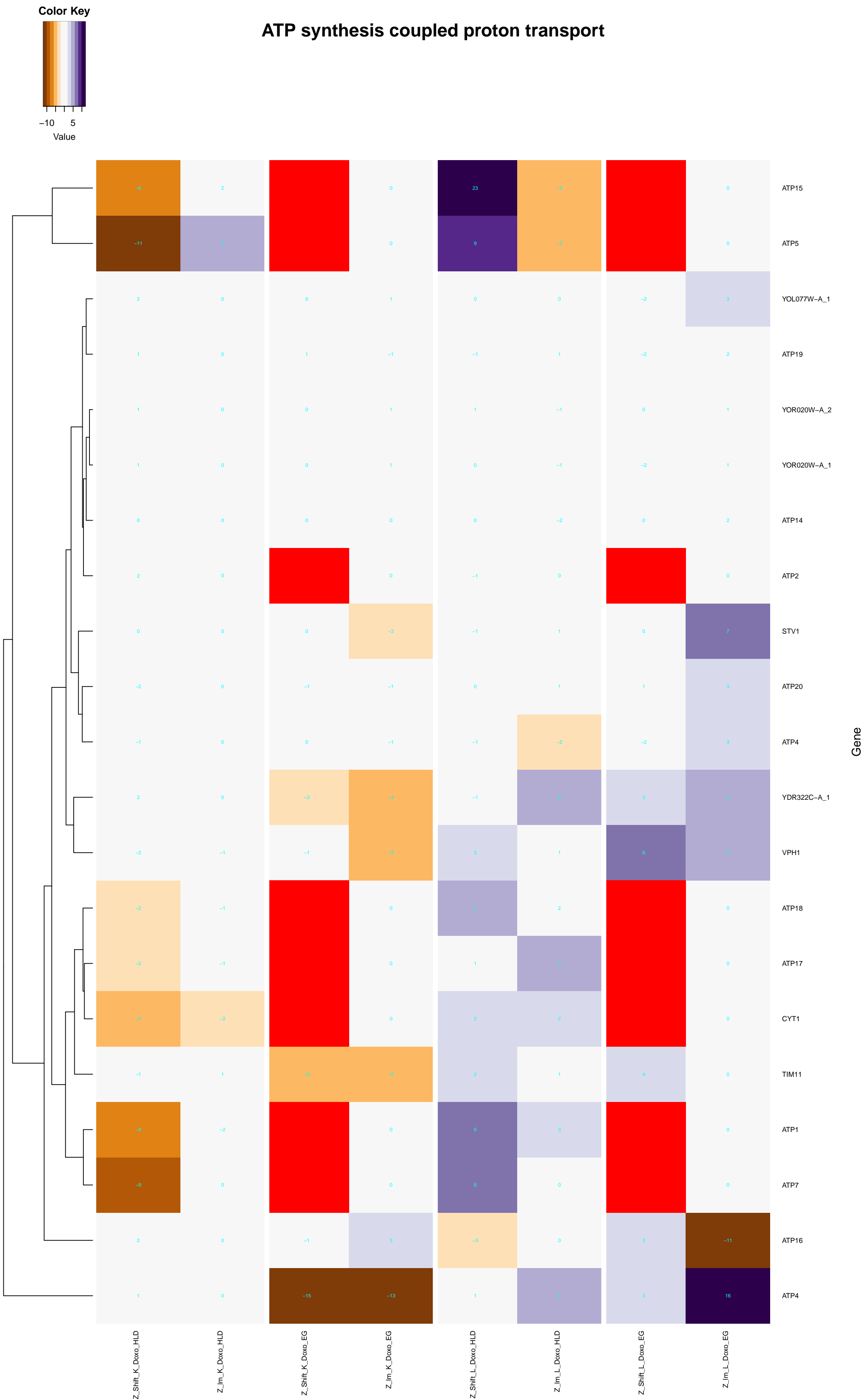

Supplement: Supplementary file 9 — Additional file 9. GO term-specific heatmaps for REMc/GTF-enriched clusters. GO term-specific heatmaps for significant GO process terms were generated as described in methods and Figs. 3 and 4. Any related child terms are presented in subsequent pages of the parent file name. GO terms with more than 100 children, with 2 or fewer genes annotated to the term, or a file size over 300KB are not shown. All heatmaps are generated with the same layout (see Figs. 3 and 4). [file 40170_2019_201_MOESM9_ESM.bz2 › Additional_File9_GOTermHeatmaps/Additional_File9_GOTermHeatmap/inorganic_cation_transmembrane_transport.pdf]

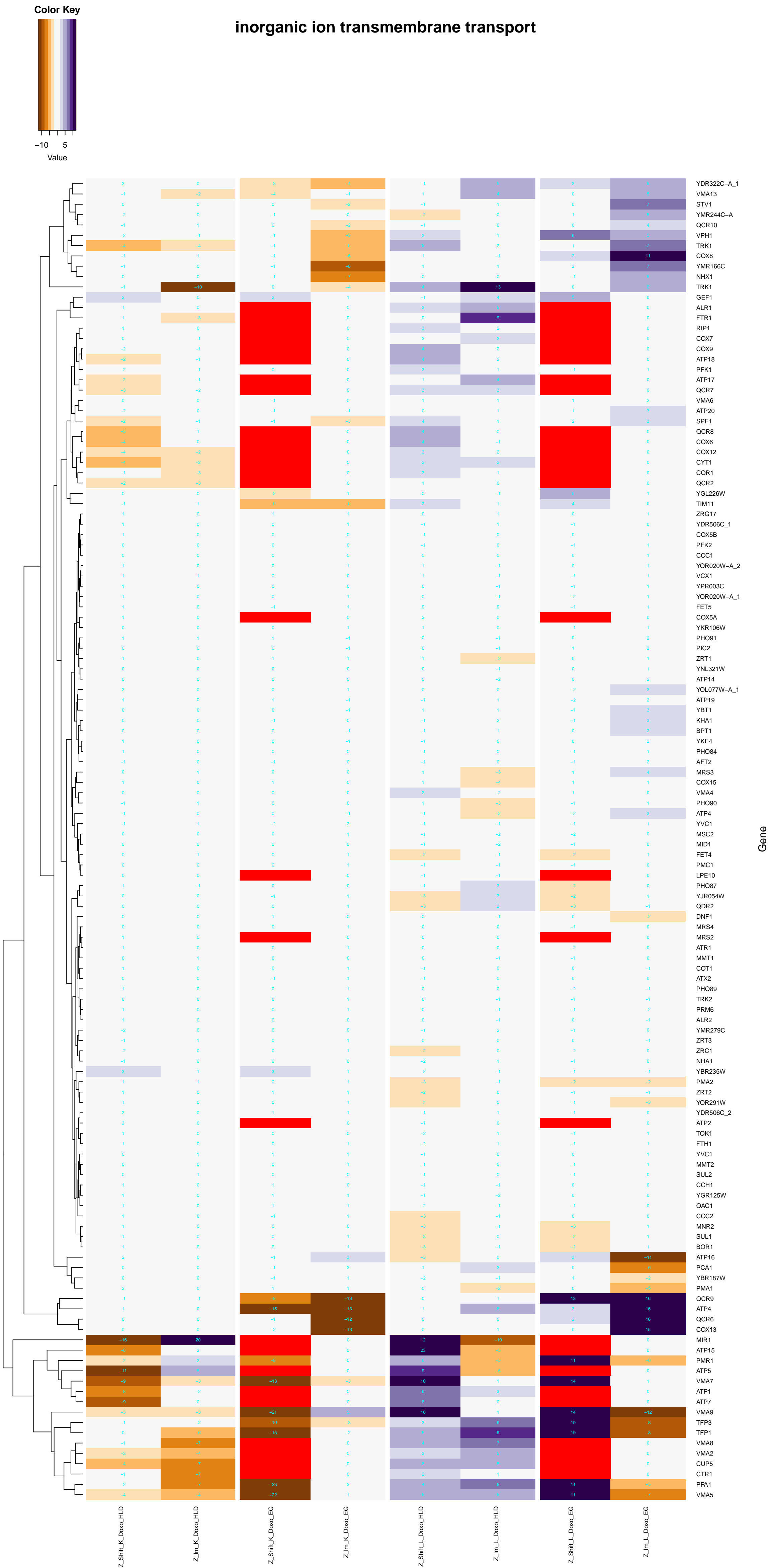

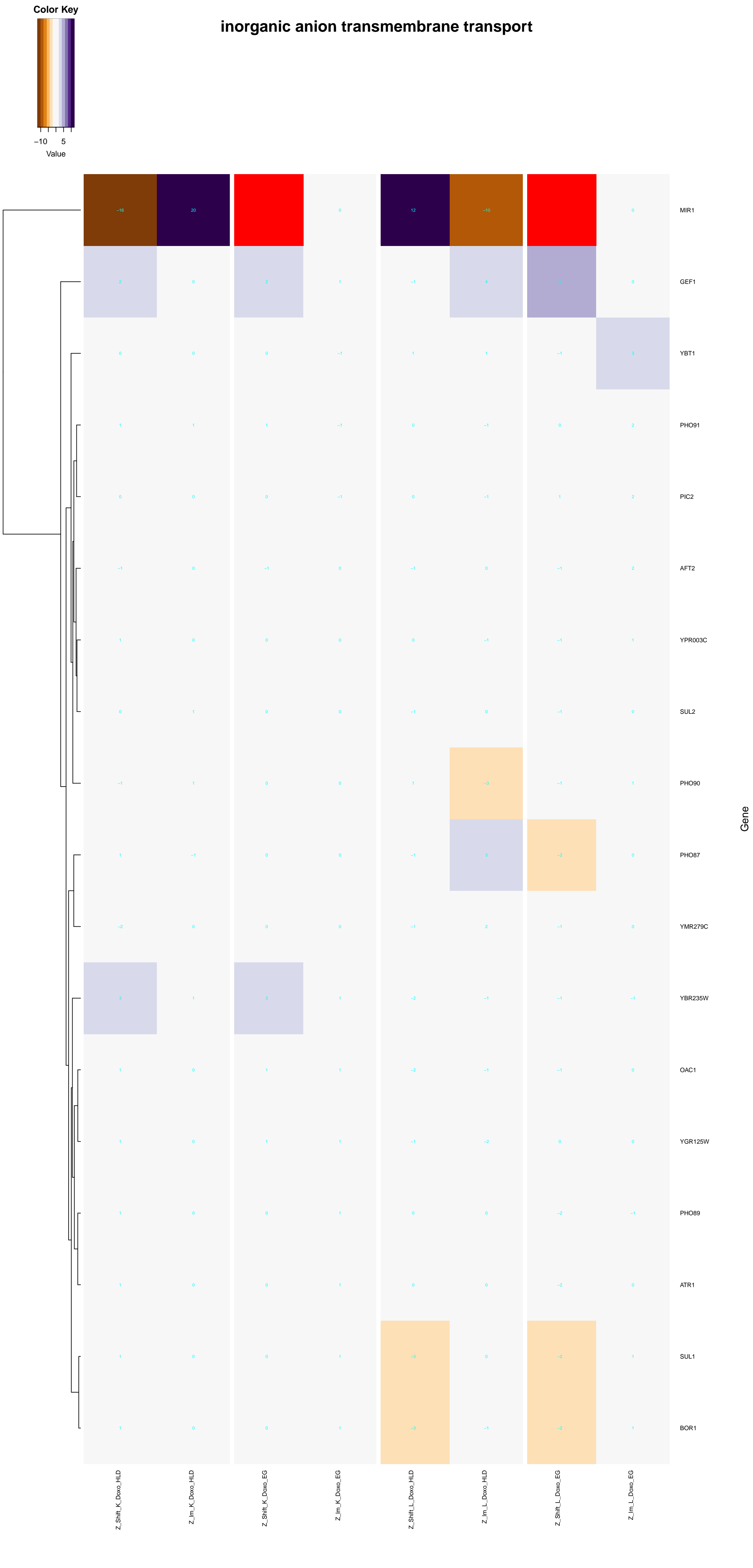

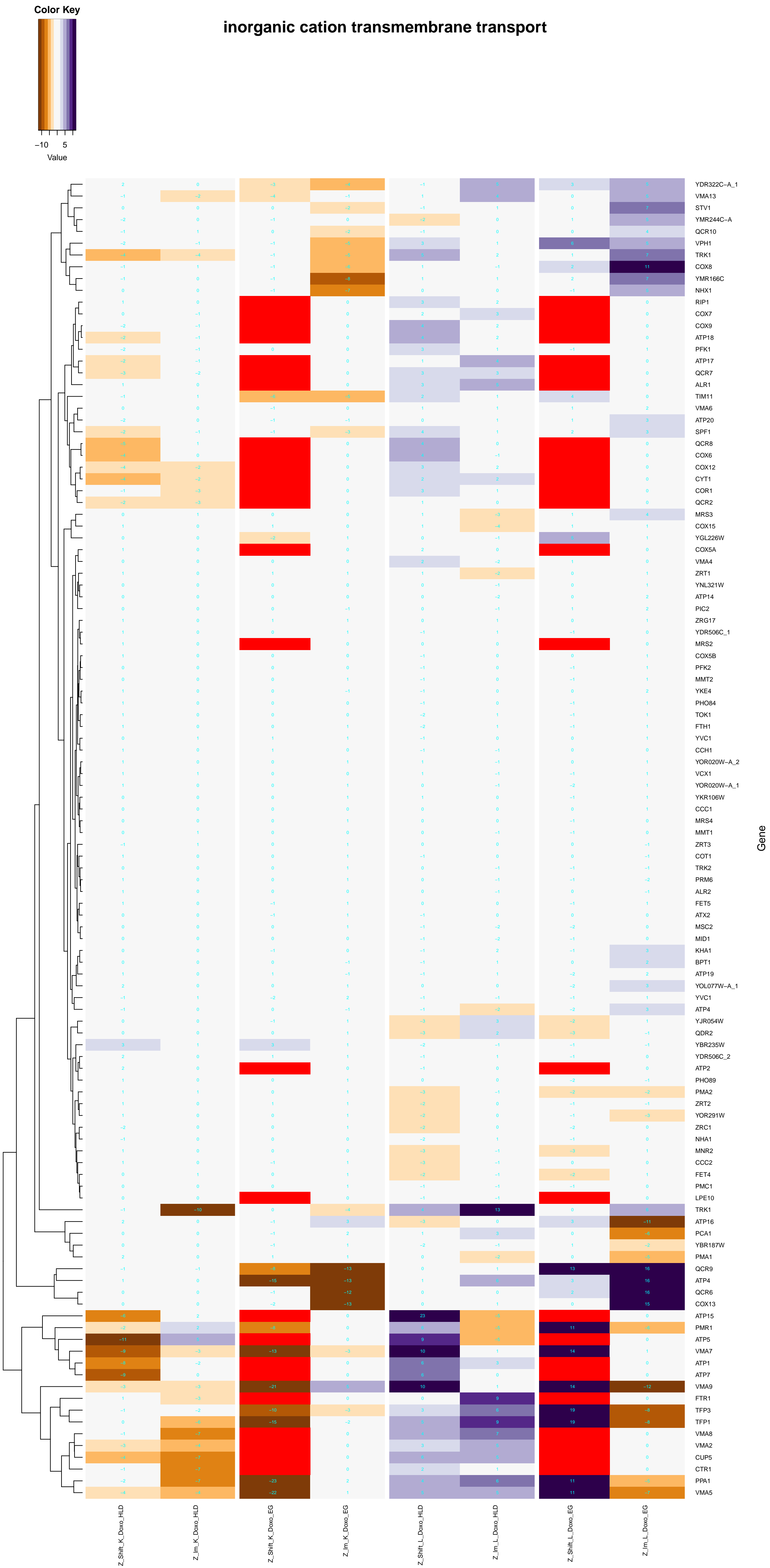

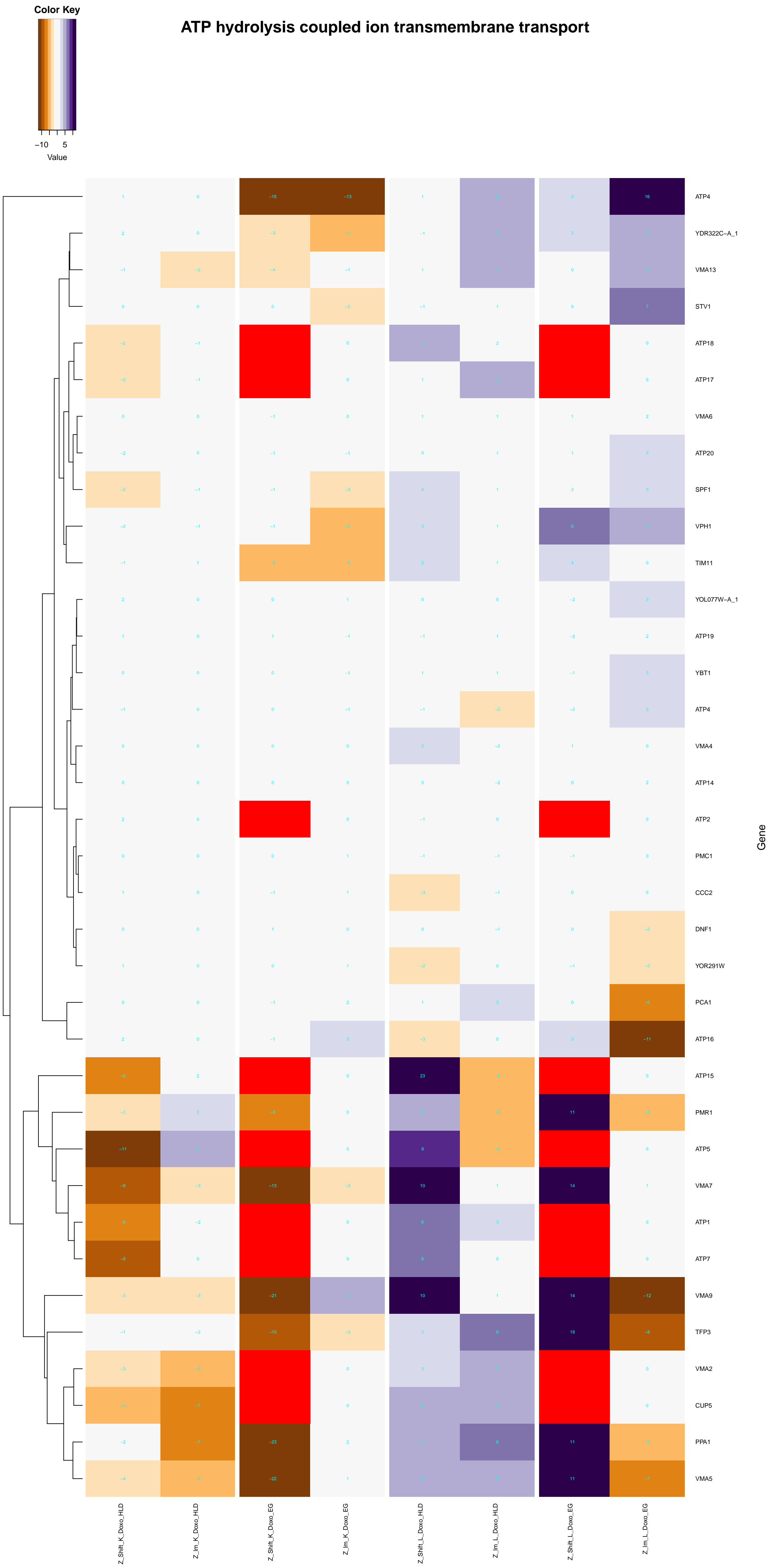

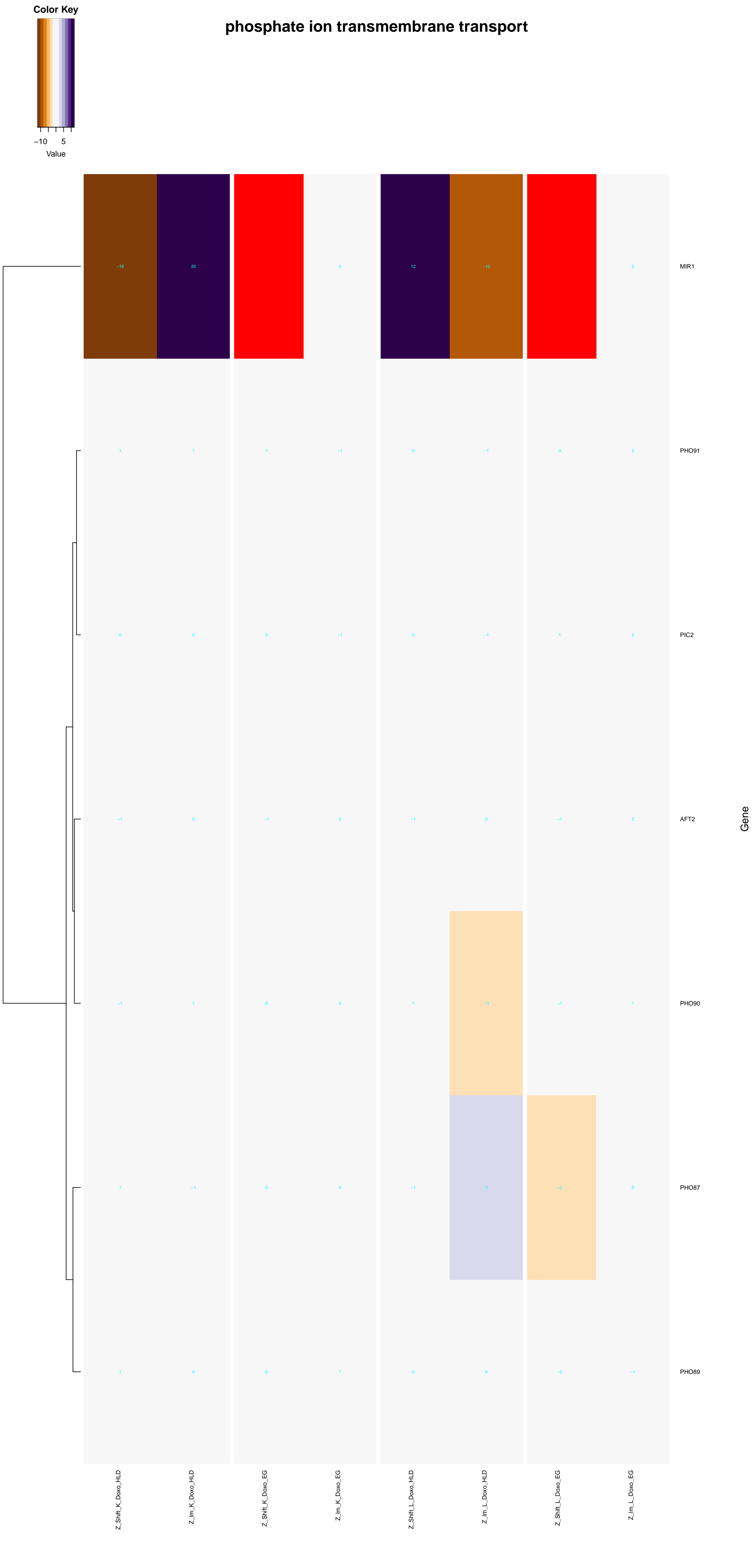

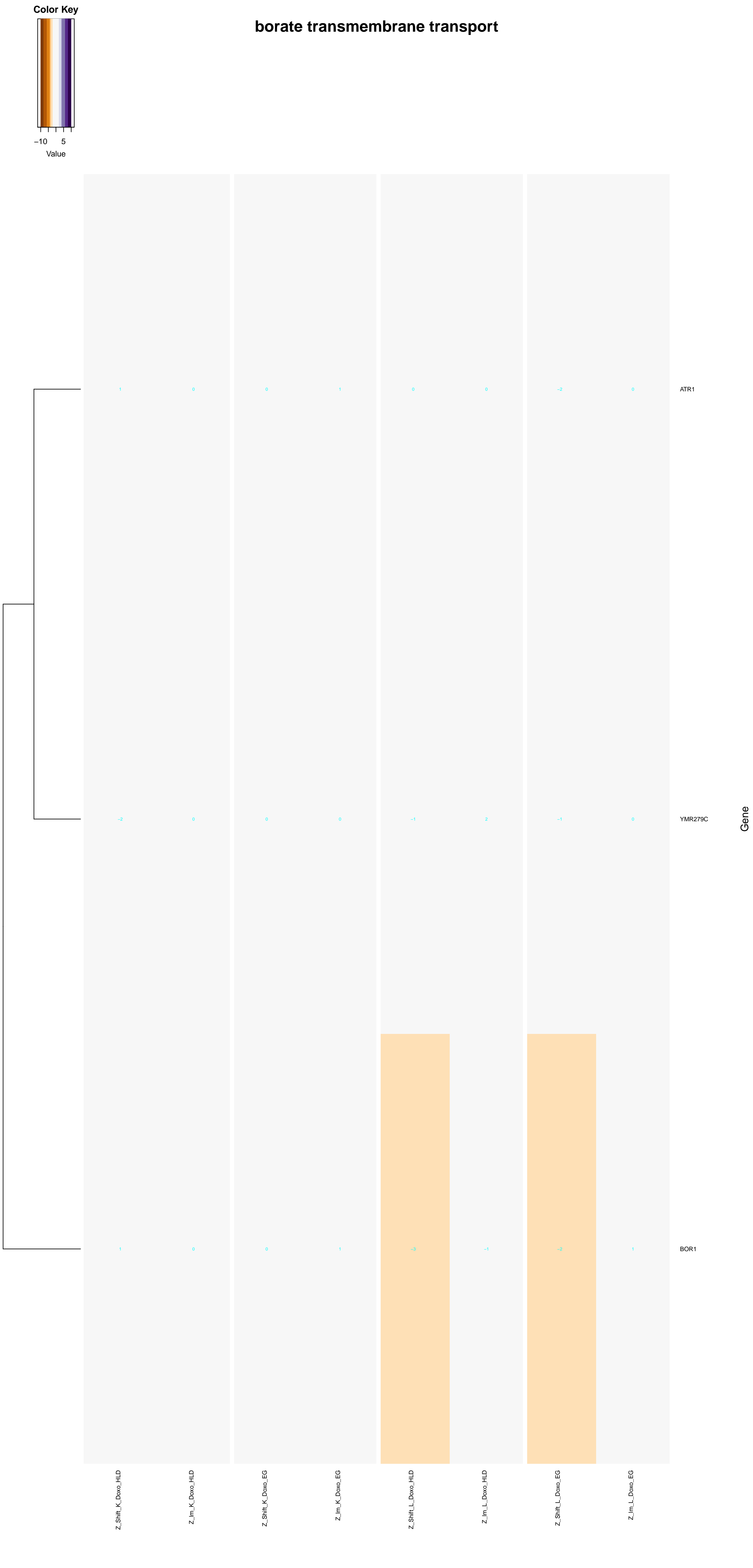

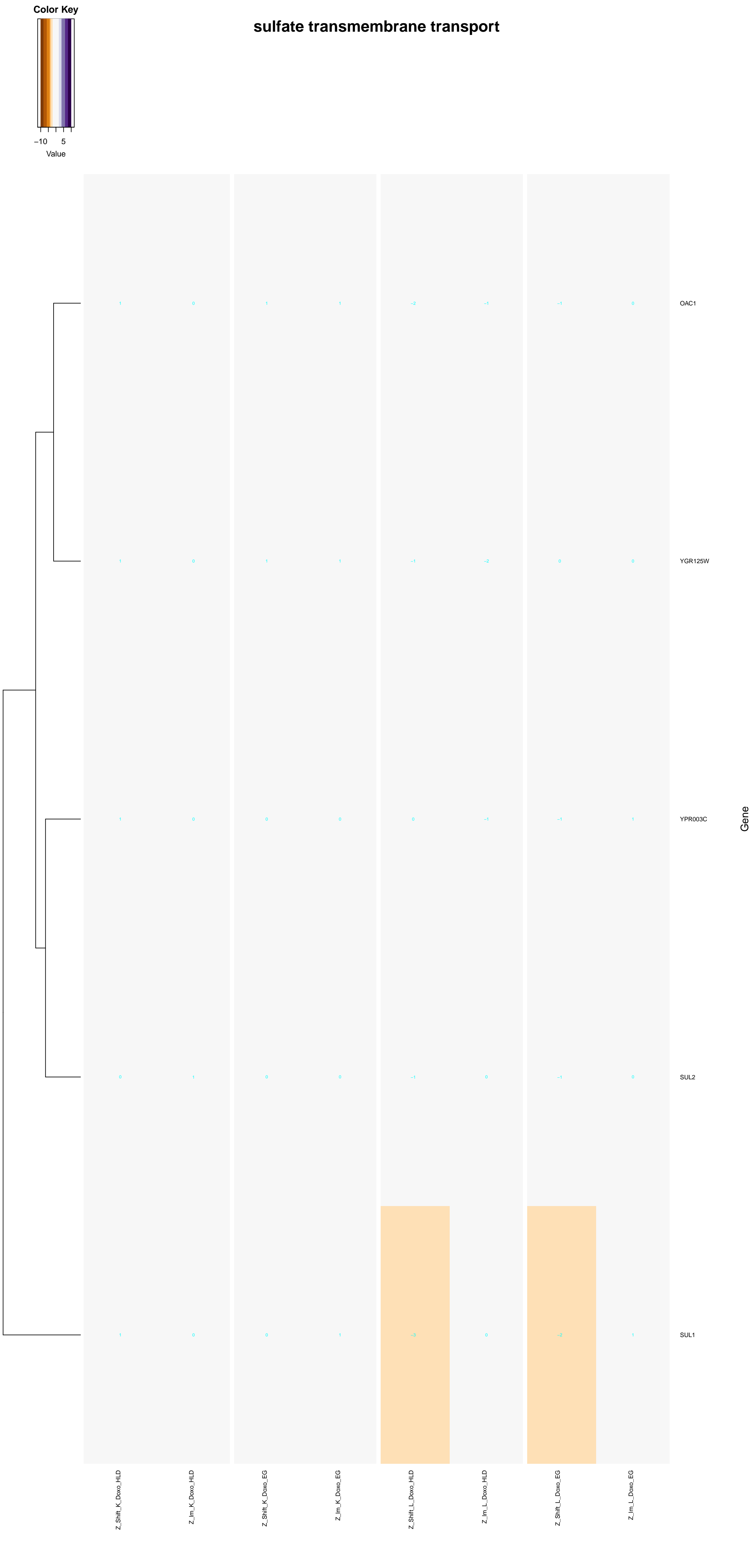

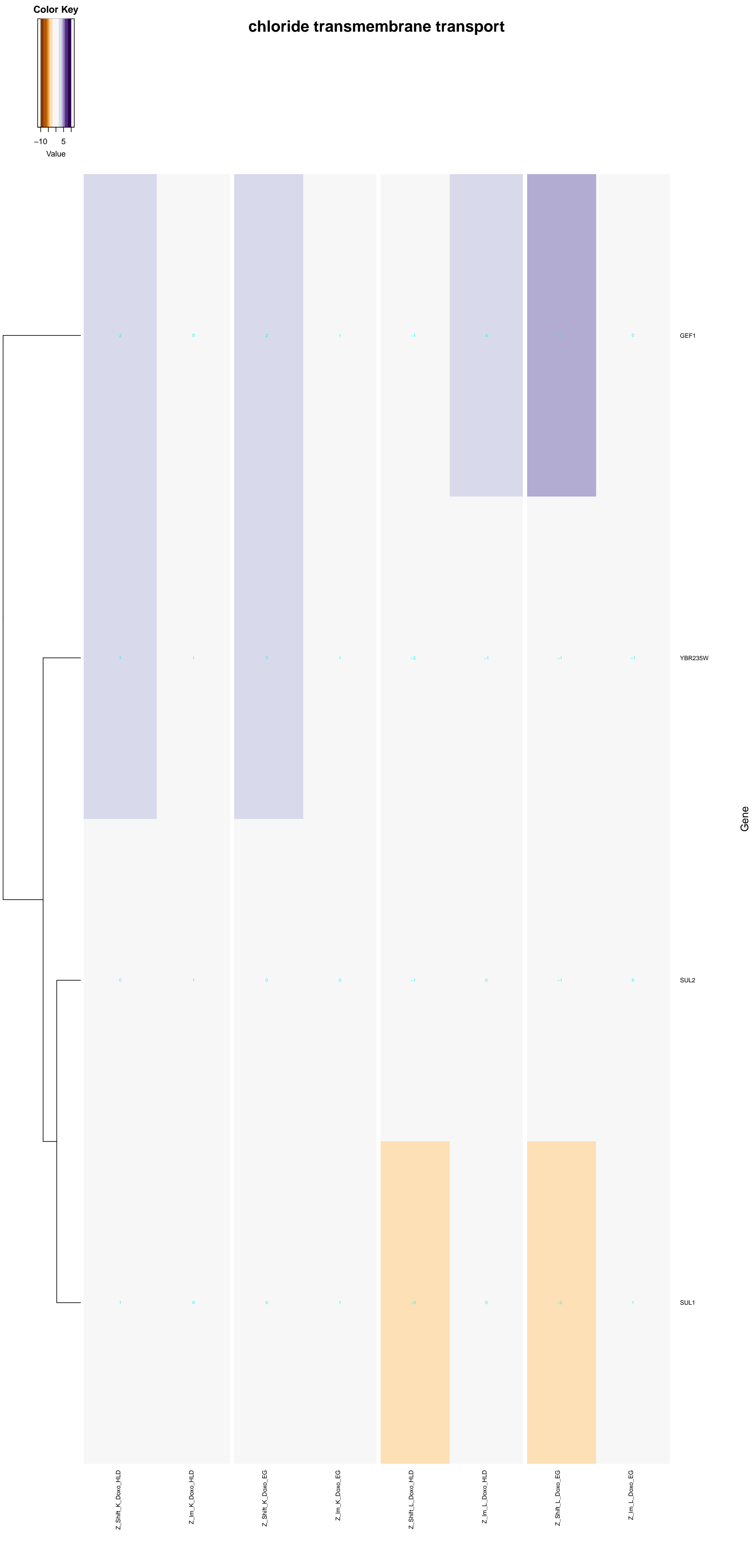

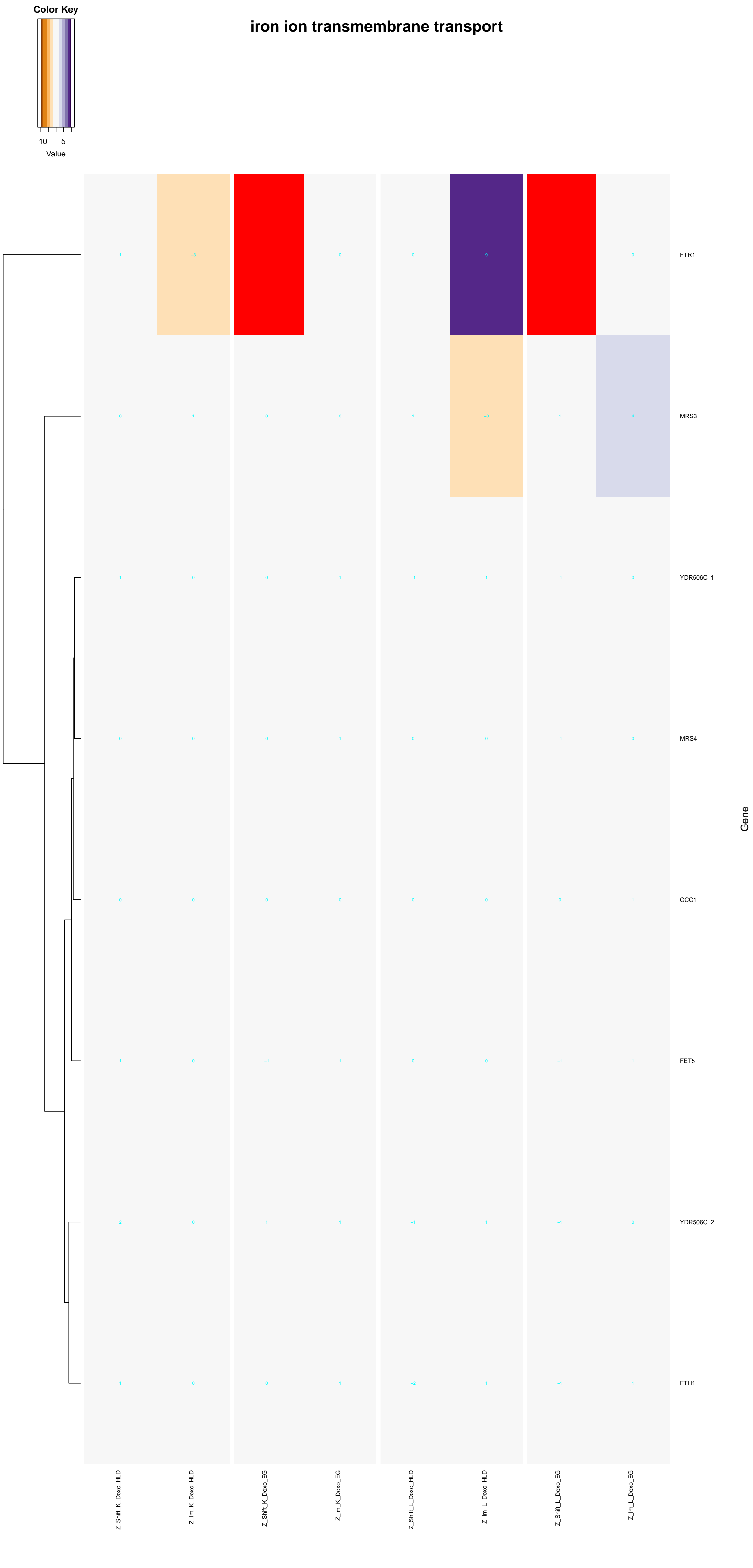

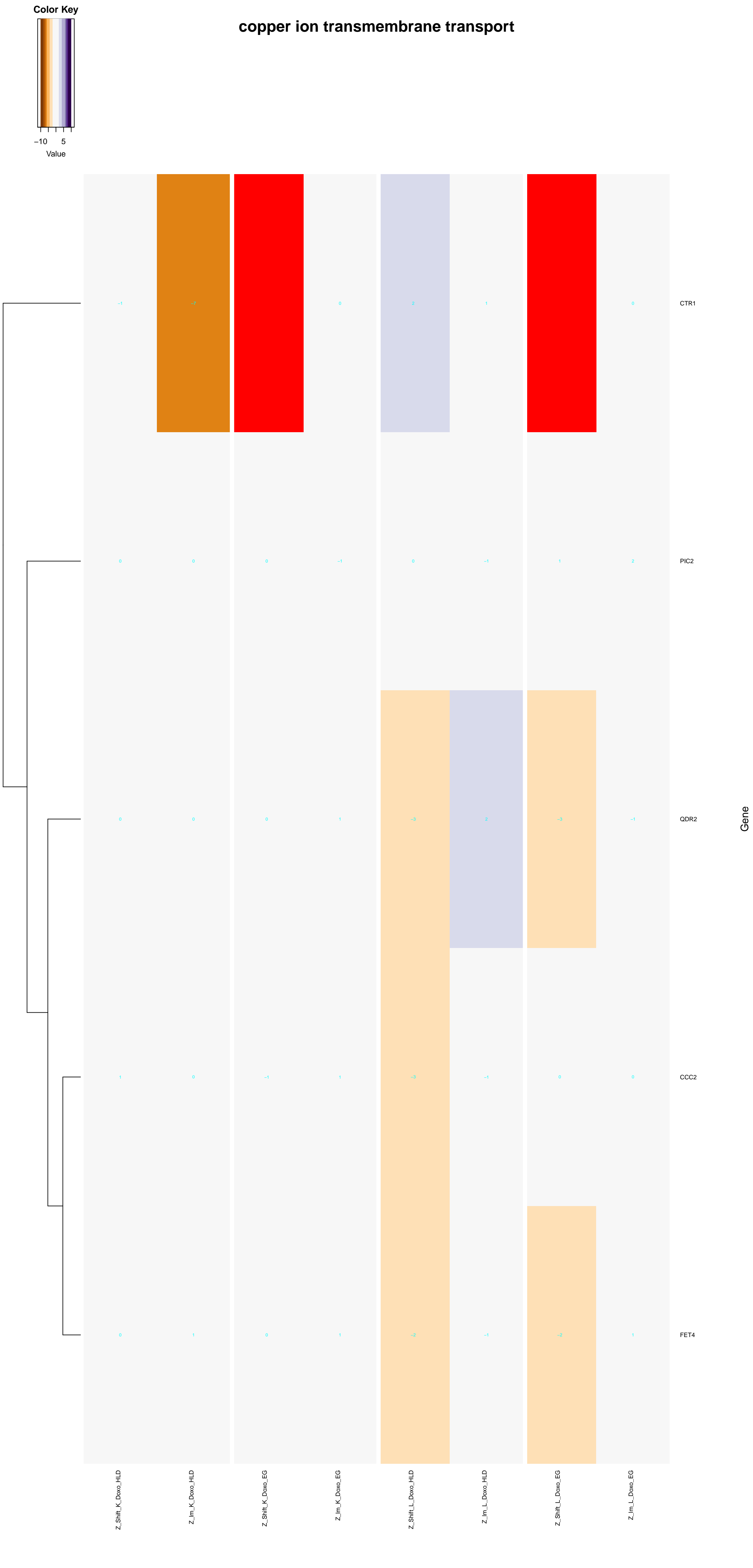

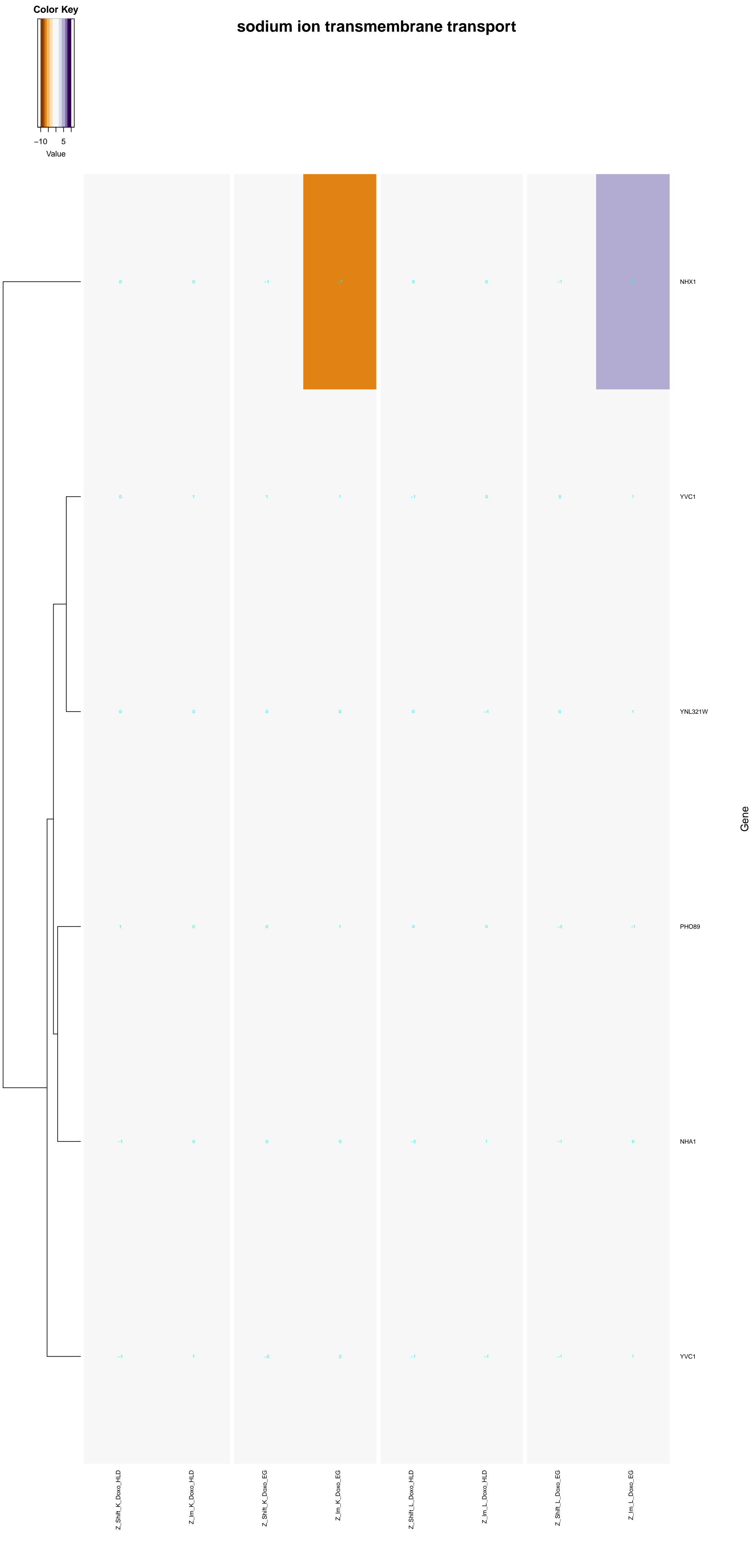

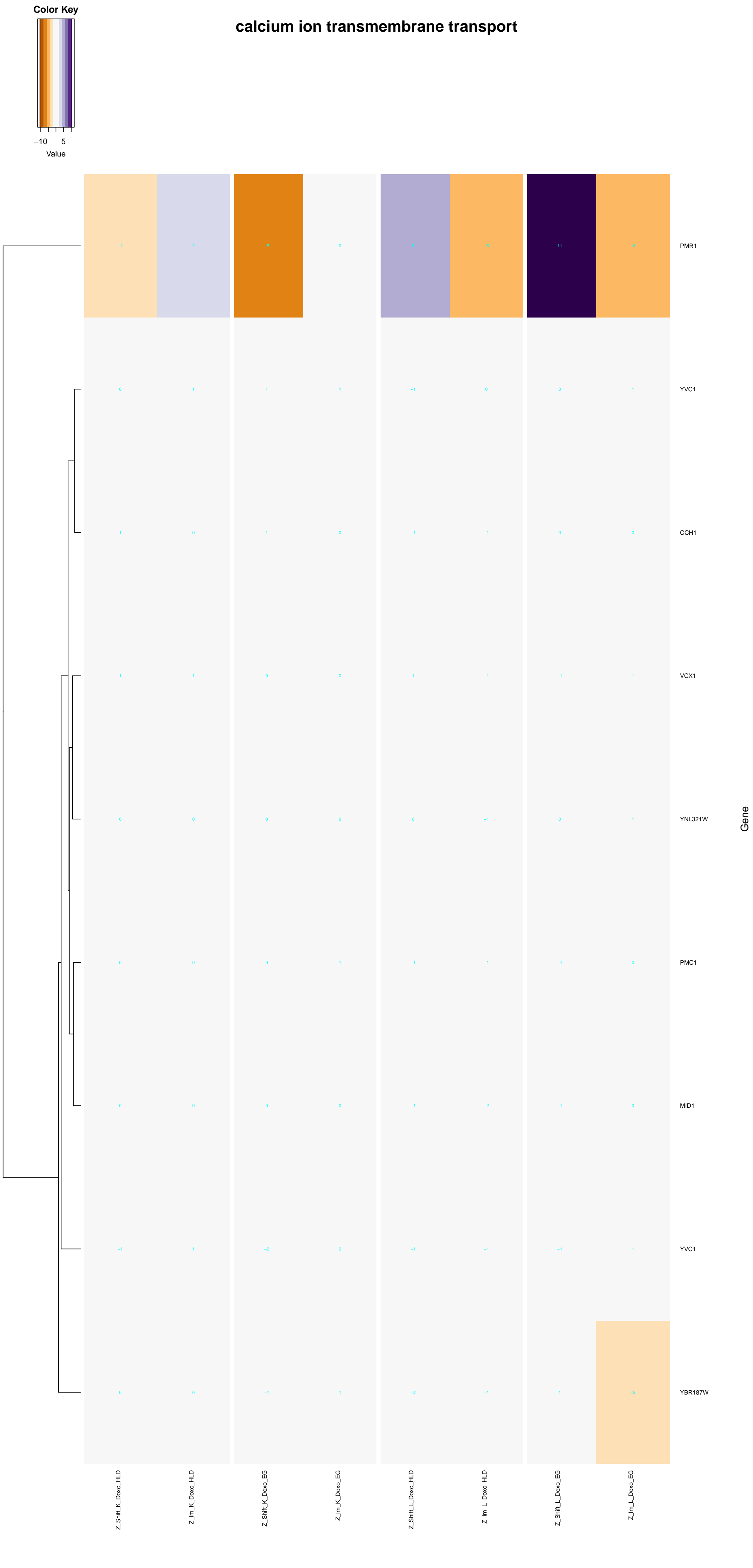

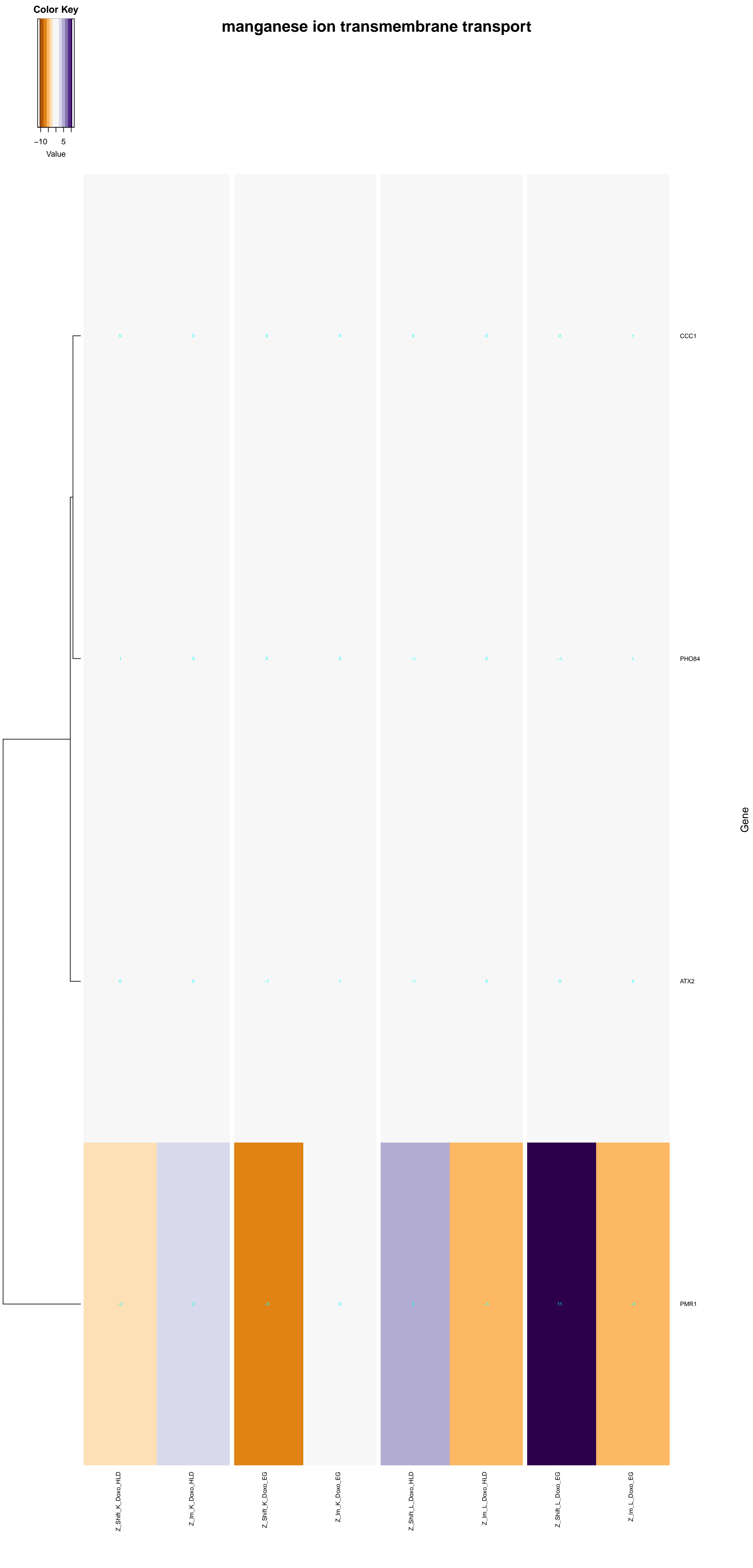

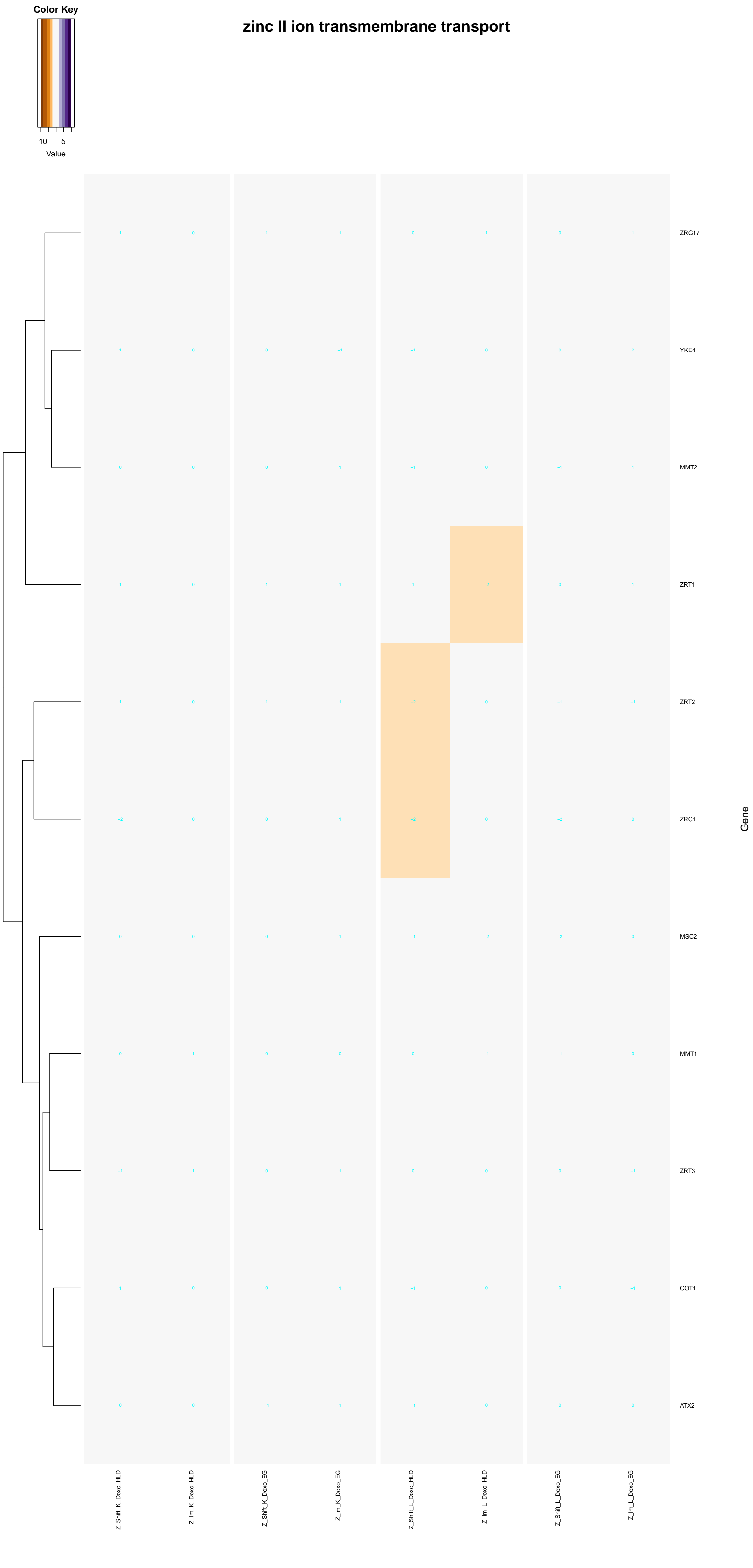

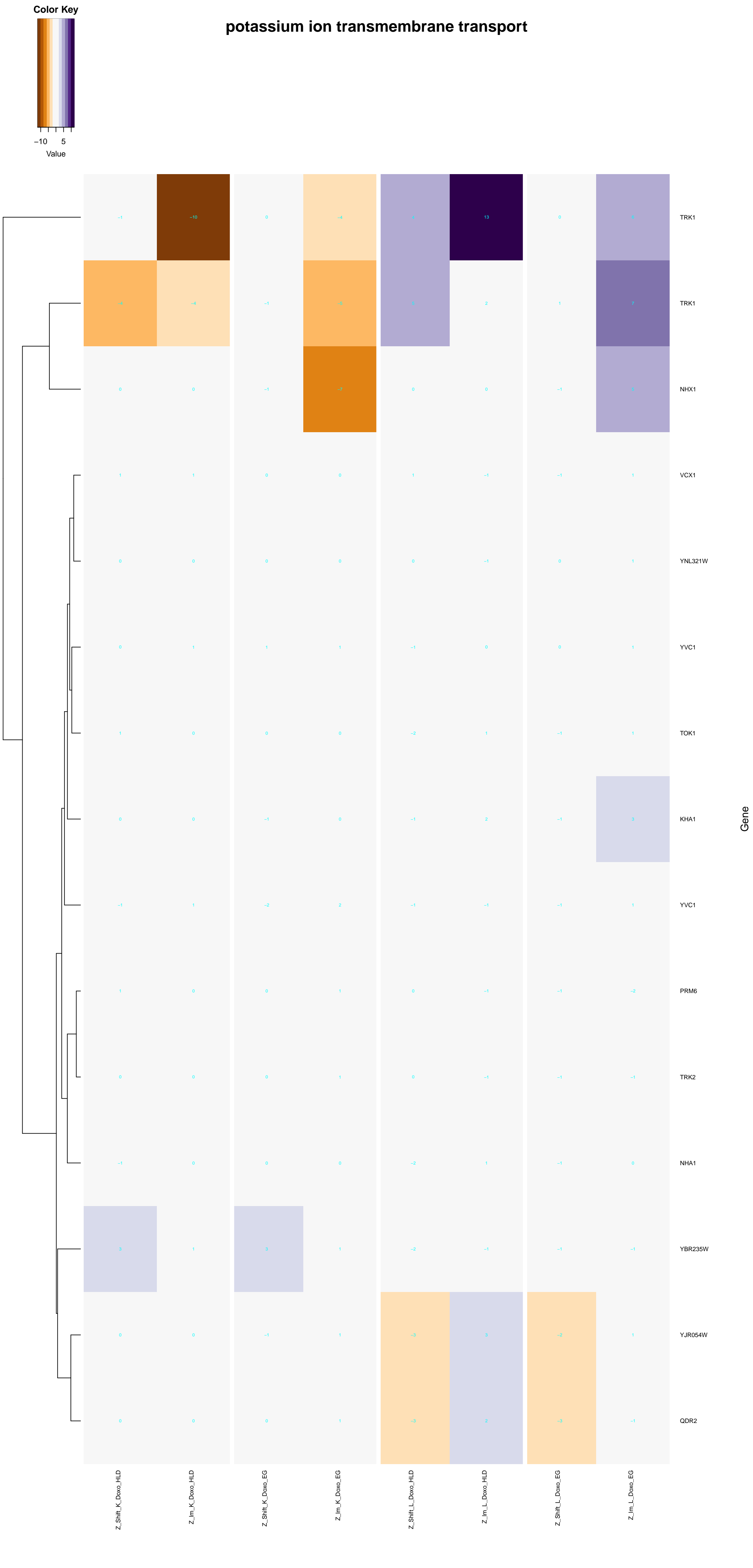

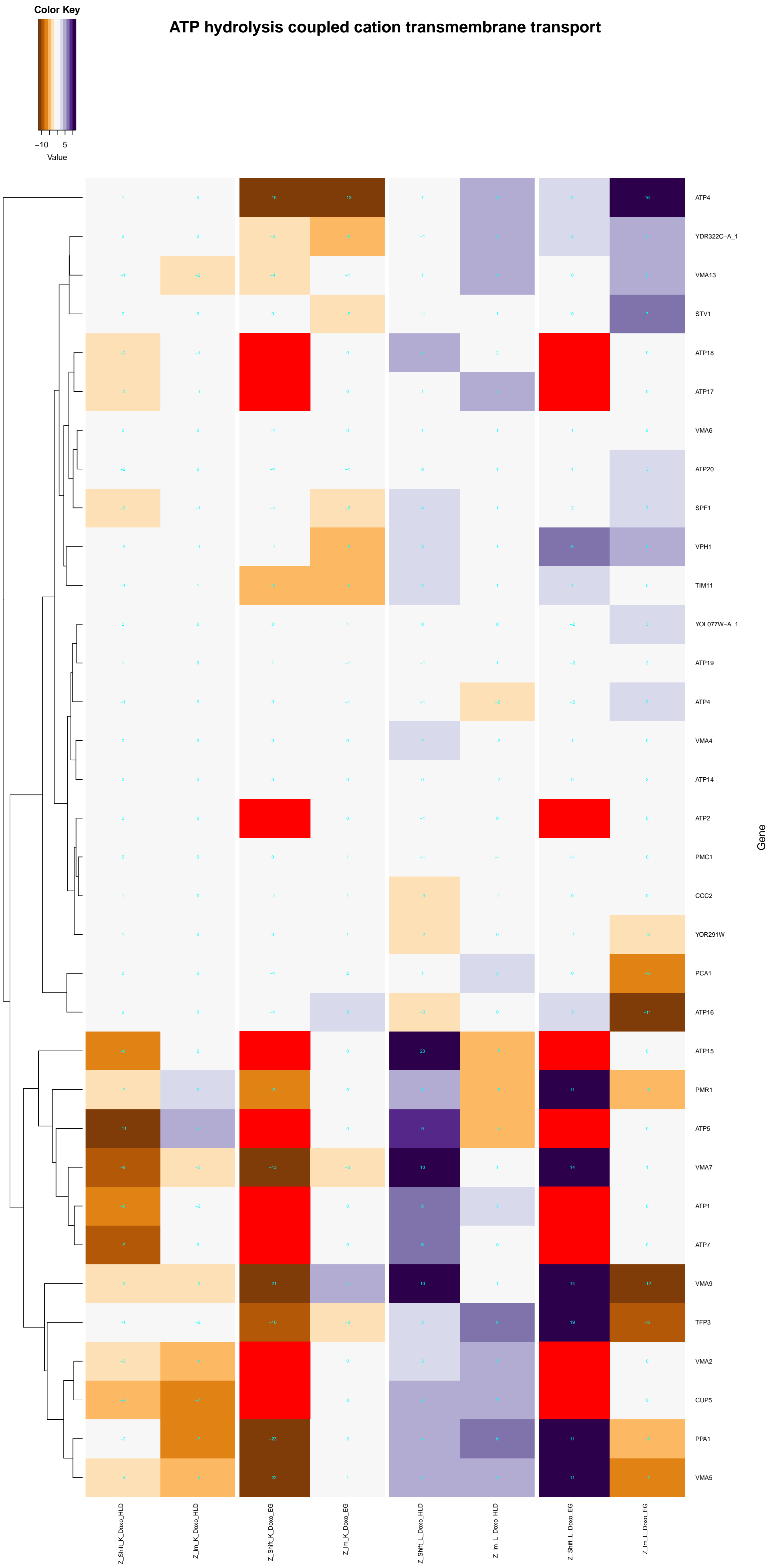

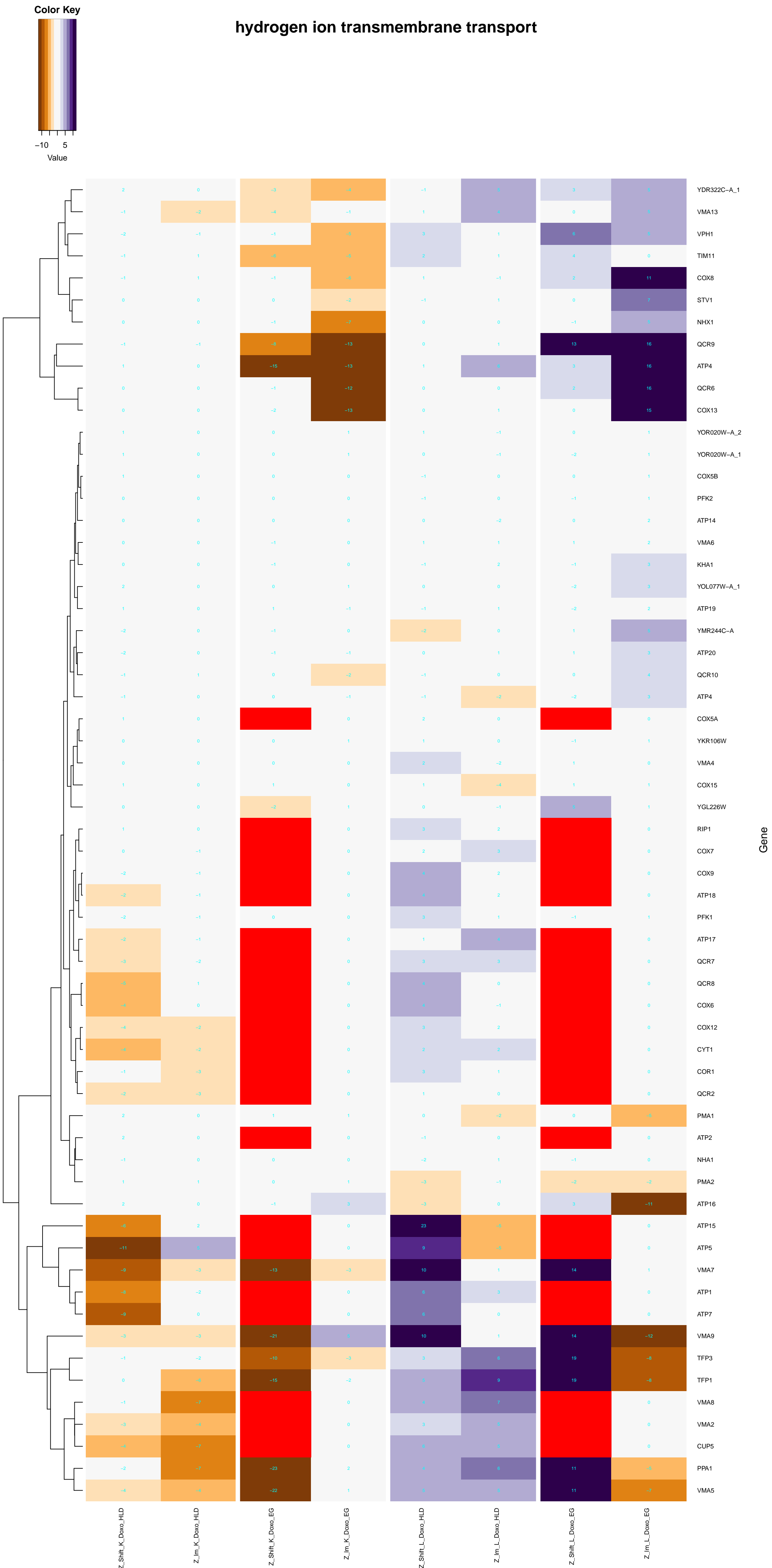

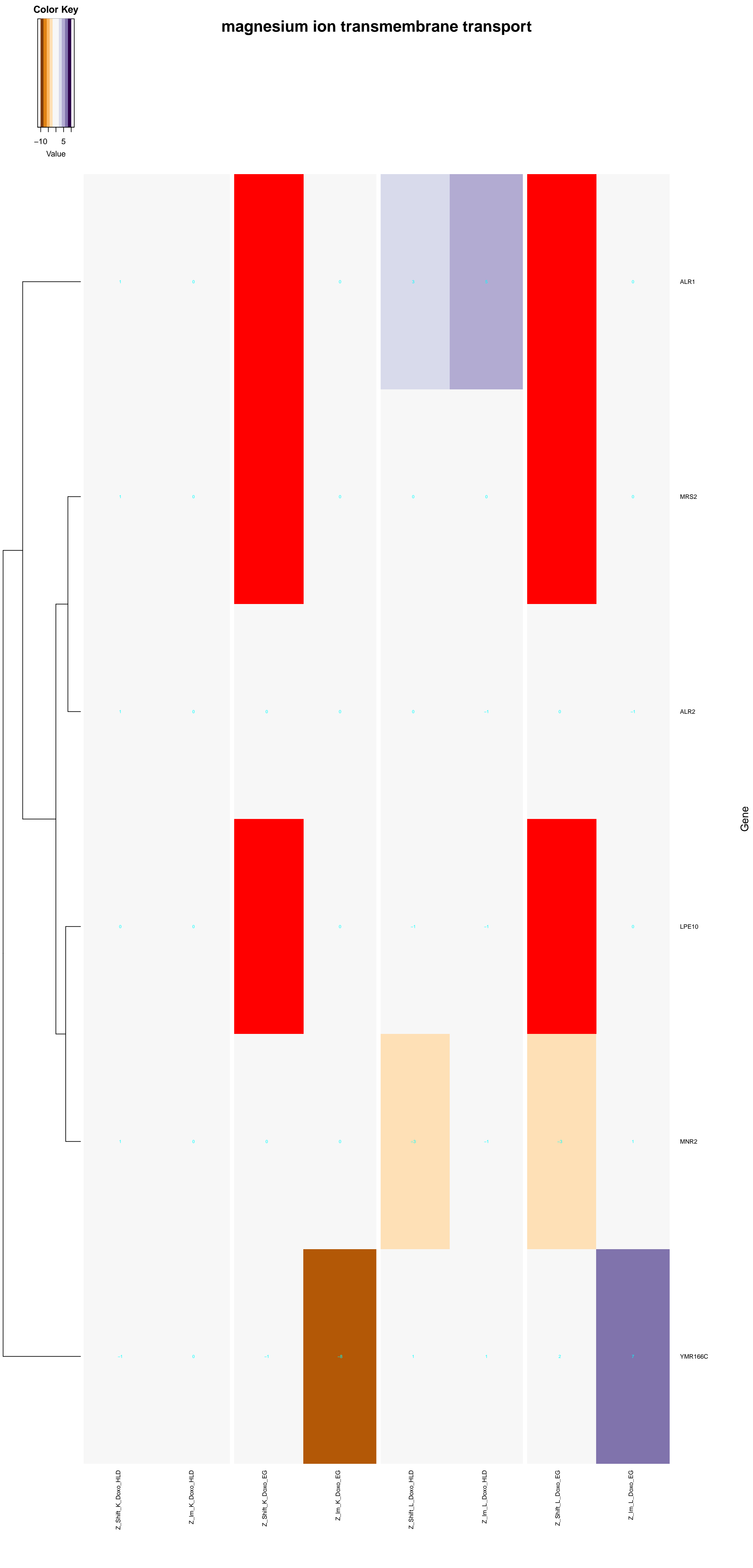

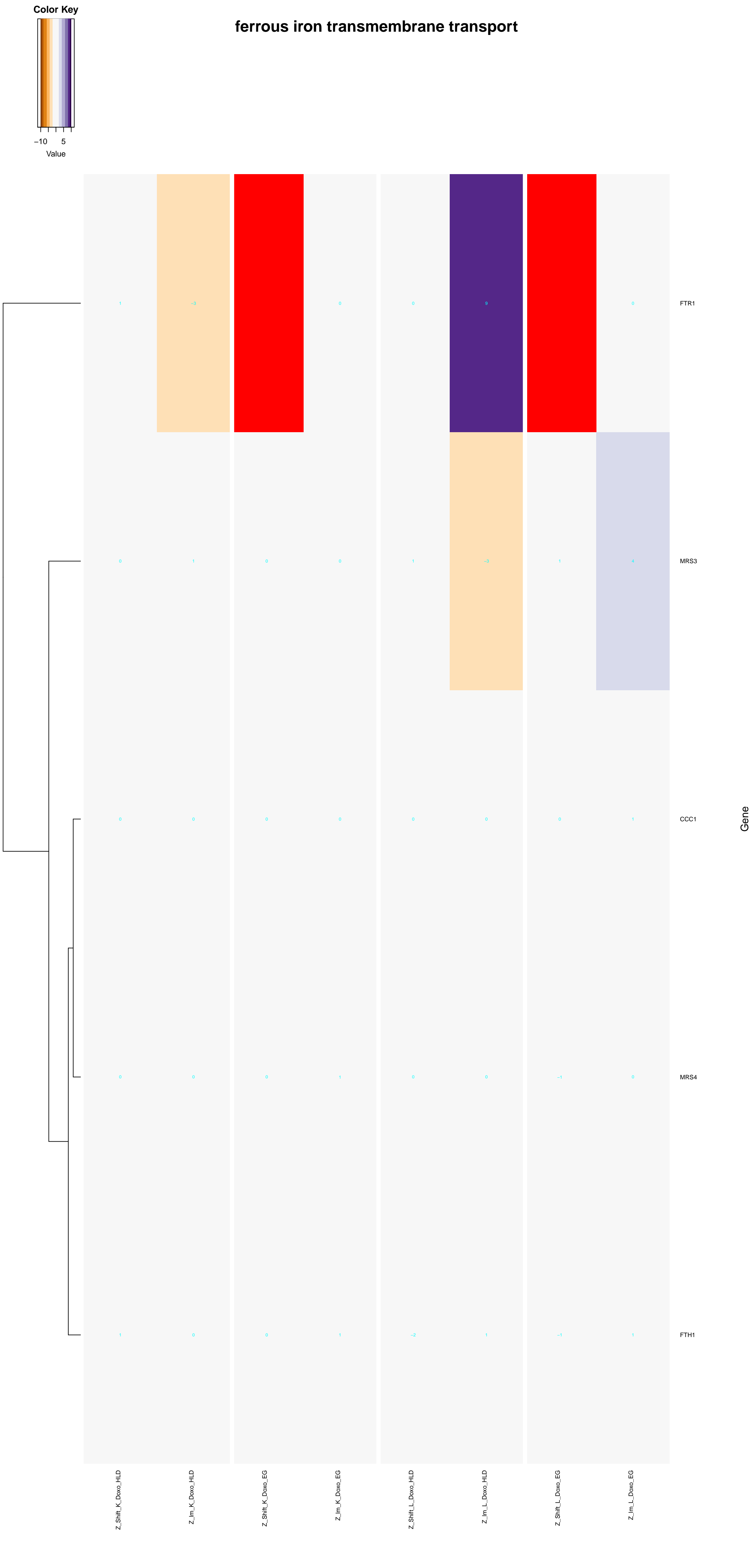

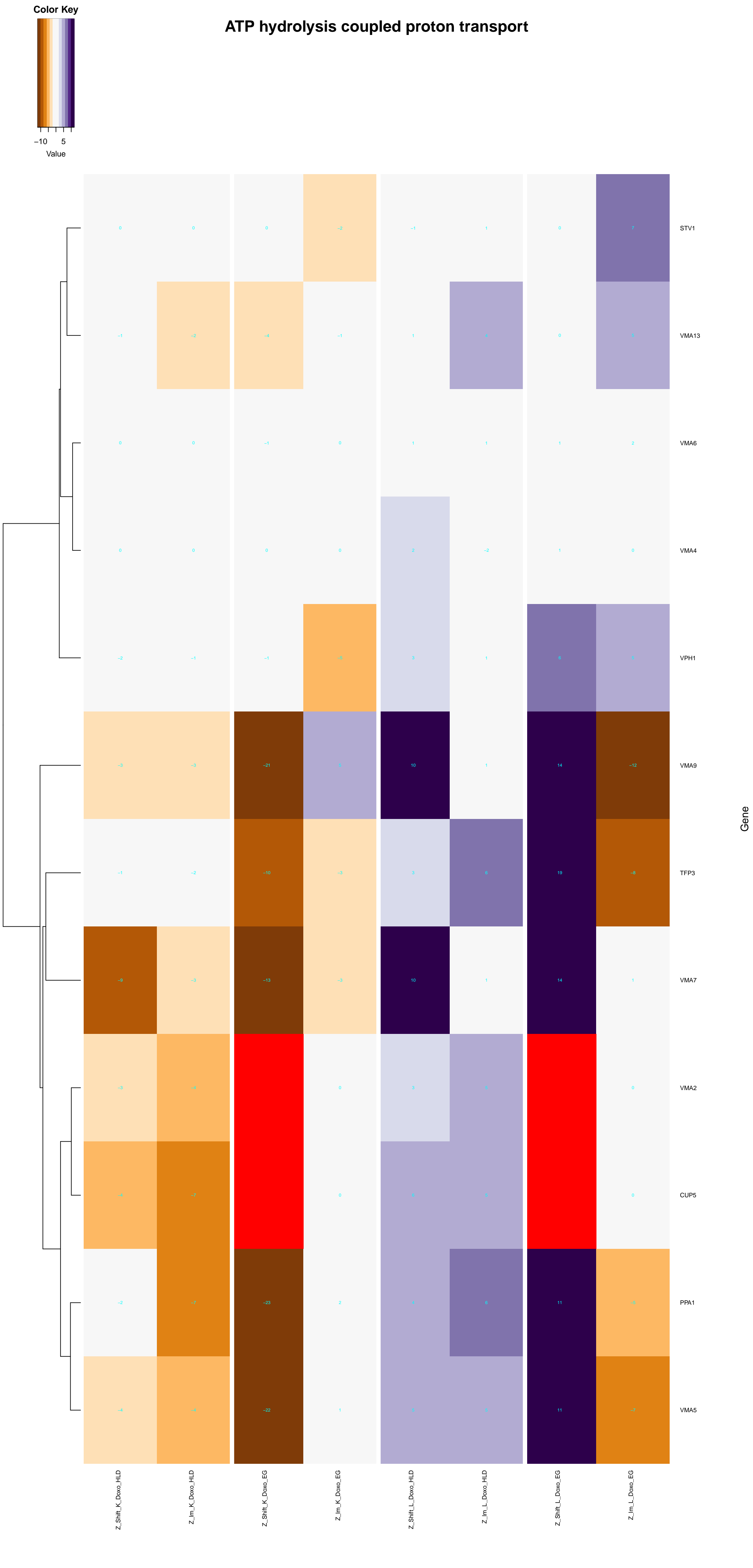

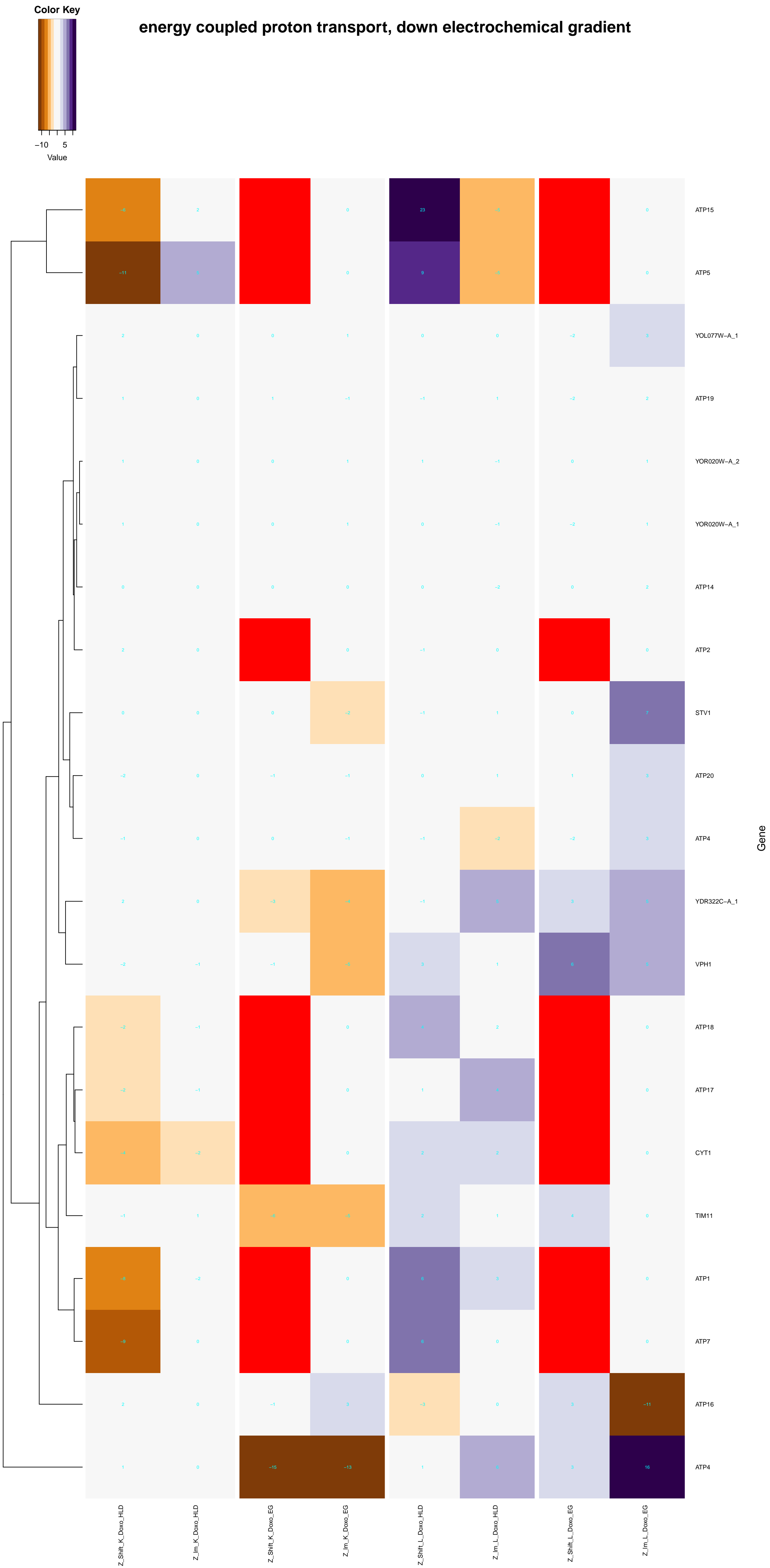

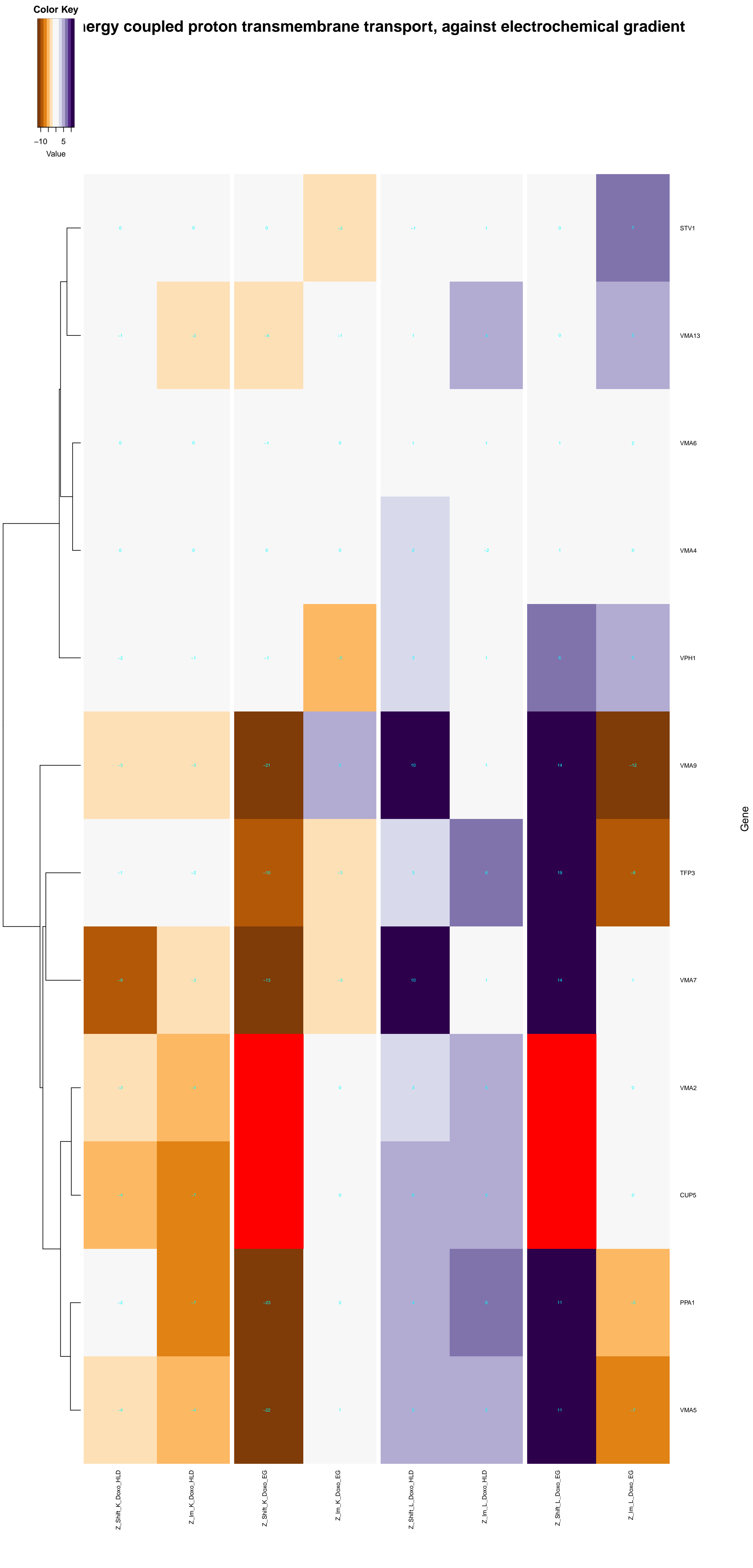

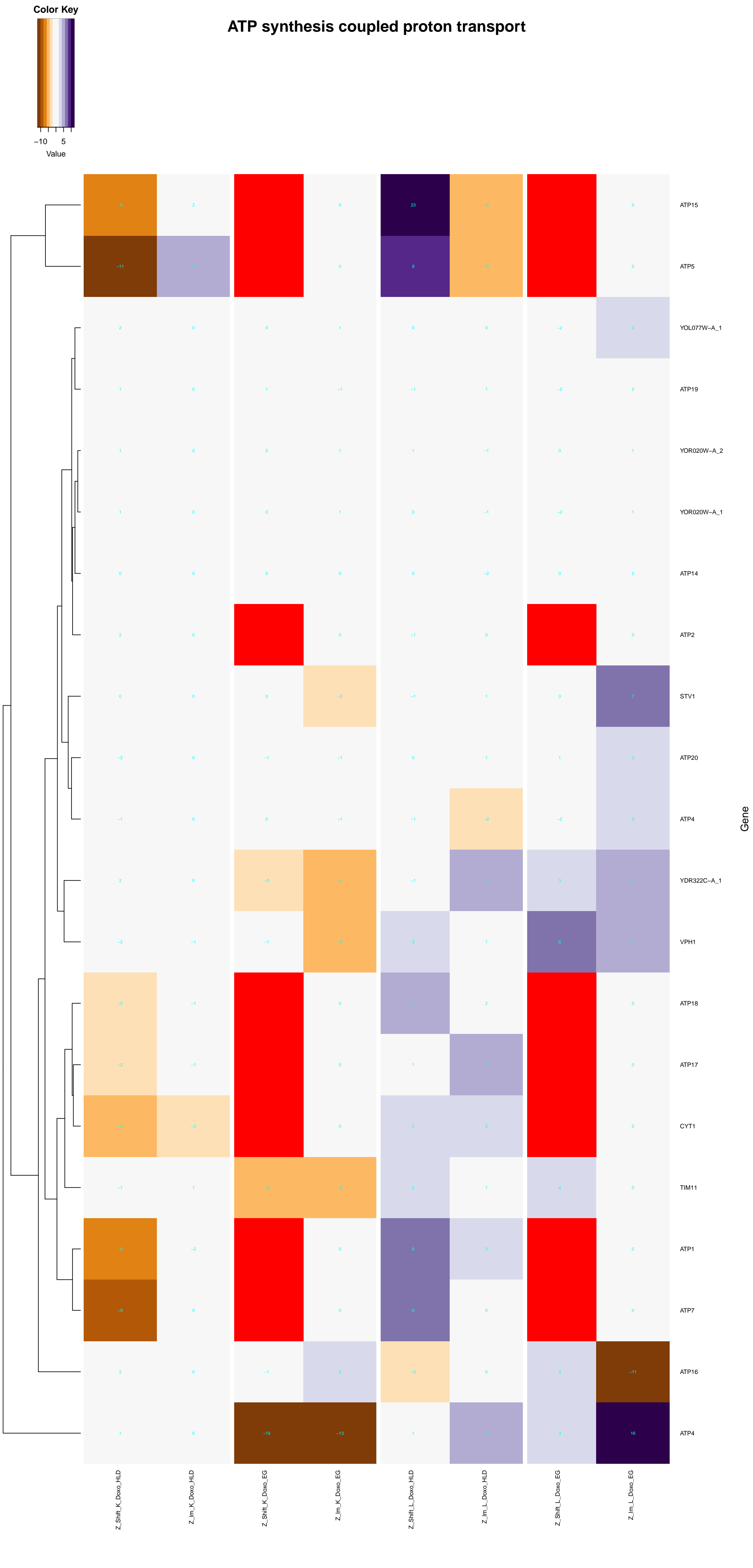

Supplement: Supplementary file 9 — Additional file 9. GO term-specific heatmaps for REMc/GTF-enriched clusters. GO term-specific heatmaps for significant GO process terms were generated as described in methods and Figs. 3 and 4. Any related child terms are presented in subsequent pages of the parent file name. GO terms with more than 100 children, with 2 or fewer genes annotated to the term, or a file size over 300KB are not shown. All heatmaps are generated with the same layout (see Figs. 3 and 4). [file 40170_2019_201_MOESM9_ESM.bz2 › Additional_File9_GOTermHeatmaps/Additional_File9_GOTermHeatmap/inorganic_ion_transmembrane_transport.pdf]

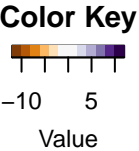

**intracellular mRNA localization**

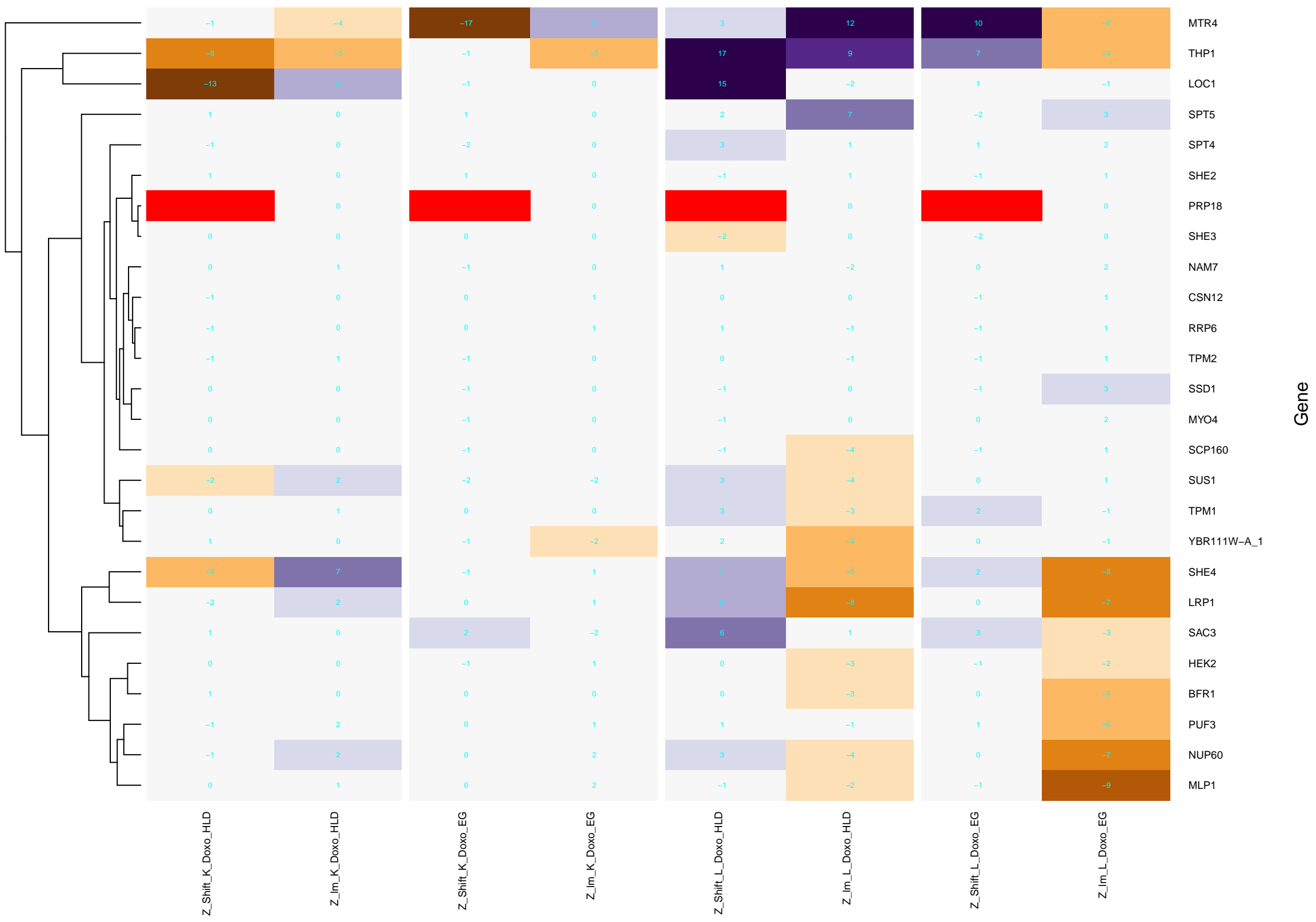

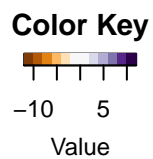

# nuclear retention of pre-mRNA at the site of transcription

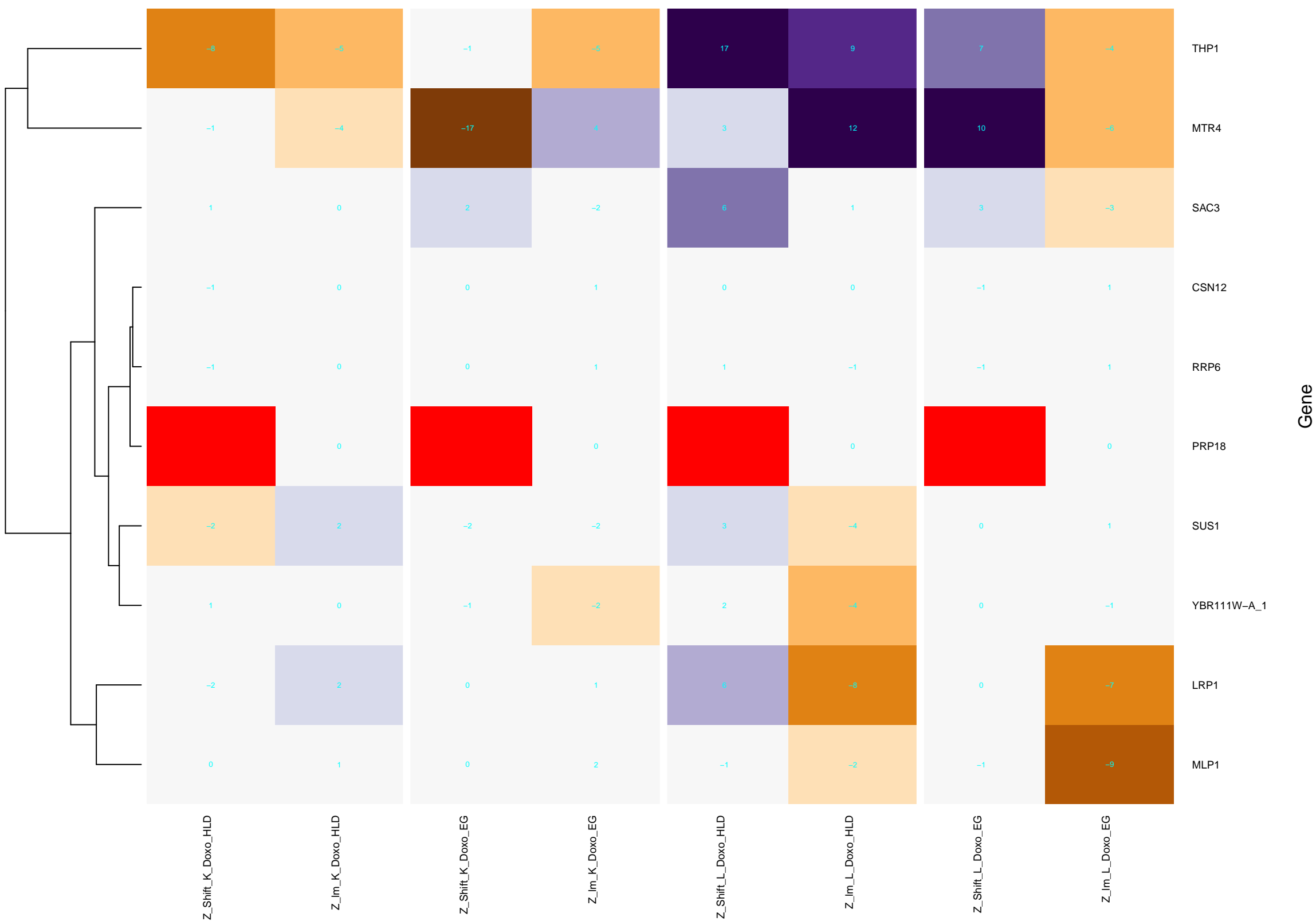

Supplement: Supplementary file 9 — Additional file 9. GO term-specific heatmaps for REMc/GTF-enriched clusters. GO term-specific heatmaps for significant GO process terms were generated as described in methods and Figs. 3 and 4. Any related child terms are presented in subsequent pages of the parent file name. GO terms with more than 100 children, with 2 or fewer genes annotated to the term, or a file size over 300KB are not shown. All heatmaps are generated with the same layout (see Figs. 3 and 4). [file 40170_2019_201_MOESM9_ESM.bz2 › Additional_File9_GOTermHeatmaps/Additional_File9_GOTermHeatmap/intracellular_mRNA_localization.pdf]

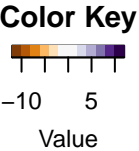

intracellular pH reduction

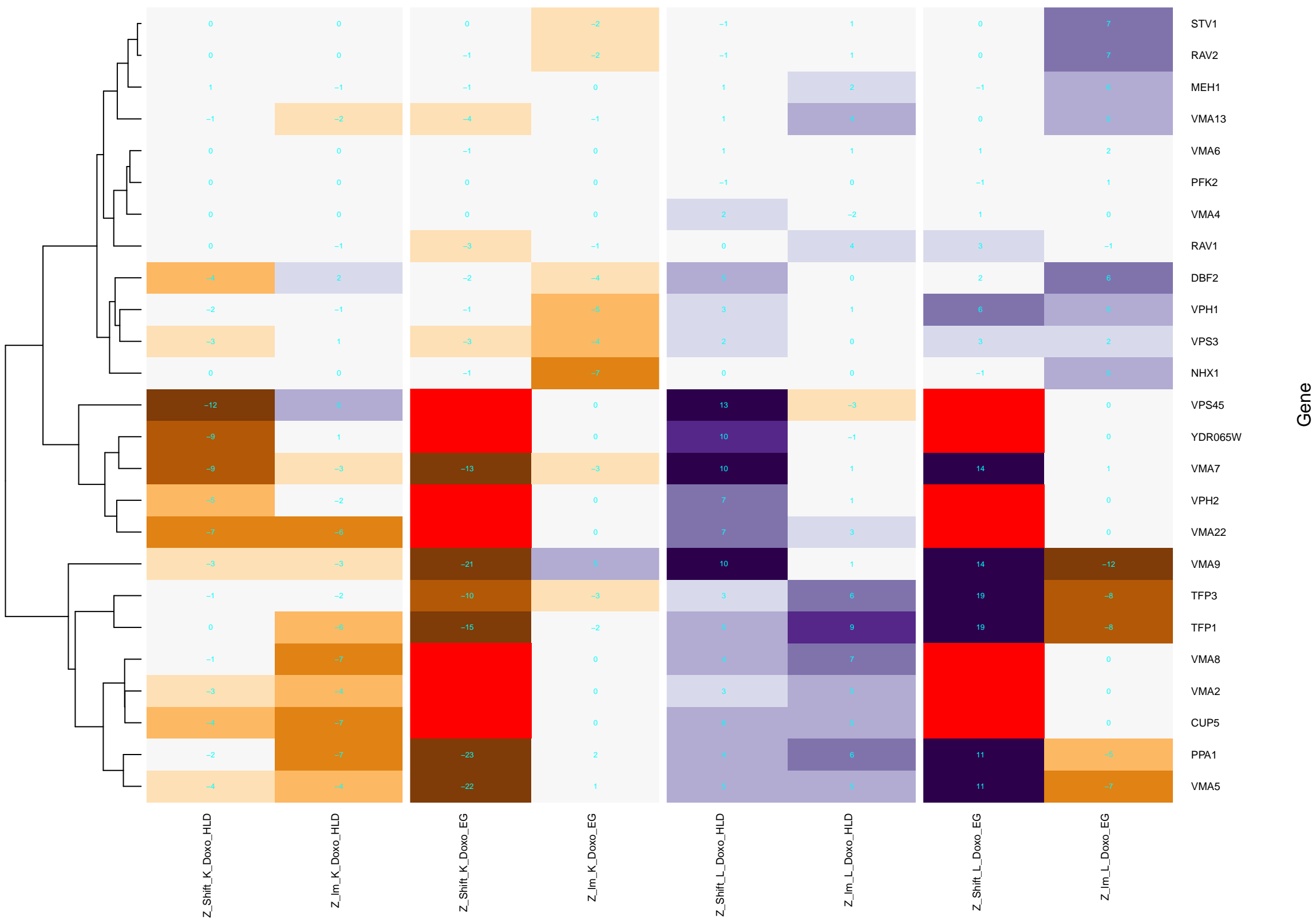

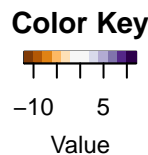

**vacuolar acidification**

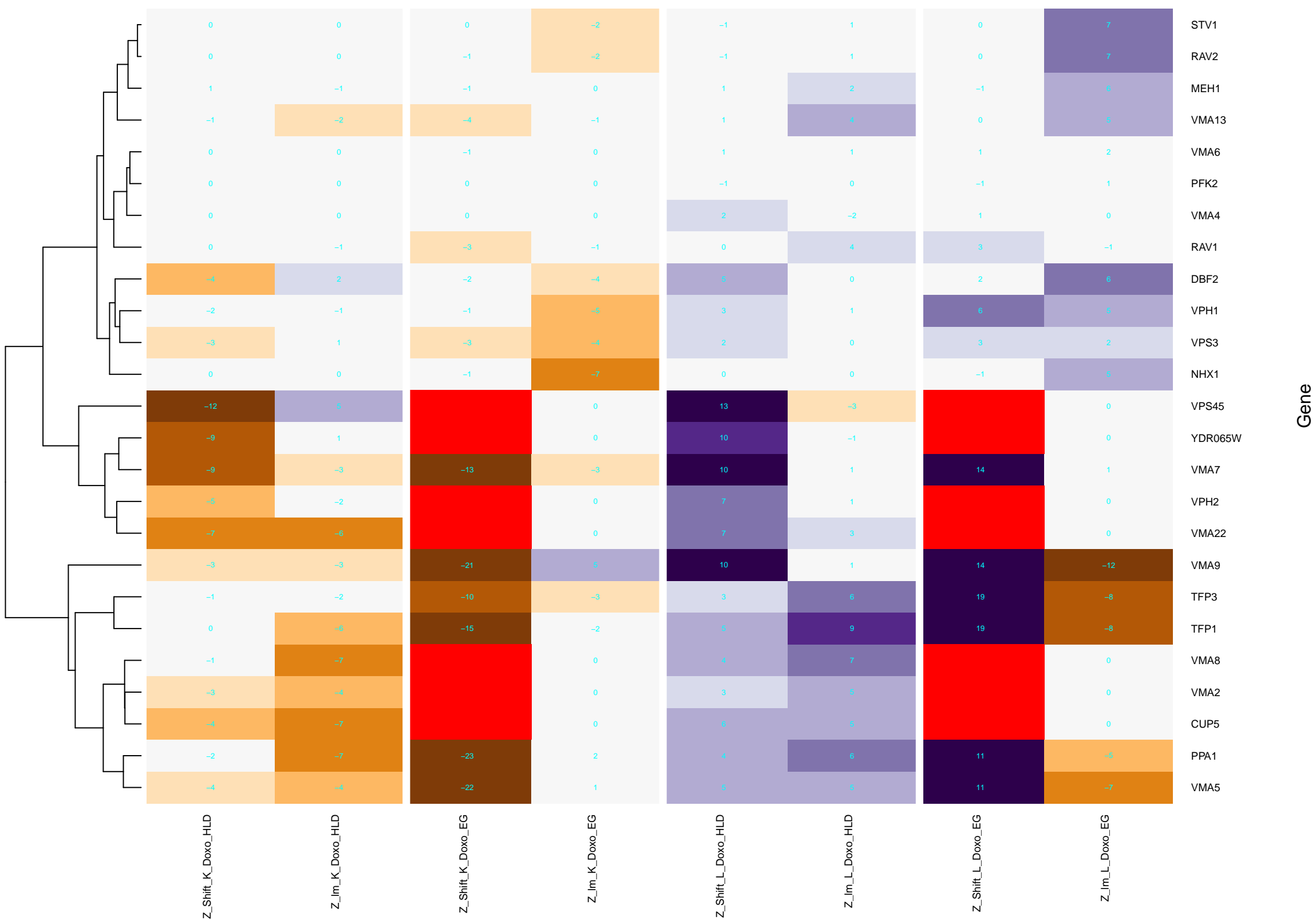

Supplement: Supplementary file 9 — Additional file 9. GO term-specific heatmaps for REMc/GTF-enriched clusters. GO term-specific heatmaps for significant GO process terms were generated as described in methods and Figs. 3 and 4. Any related child terms are presented in subsequent pages of the parent file name. GO terms with more than 100 children, with 2 or fewer genes annotated to the term, or a file size over 300KB are not shown. All heatmaps are generated with the same layout (see Figs. 3 and 4). [file 40170_2019_201_MOESM9_ESM.bz2 › Additional_File9_GOTermHeatmaps/Additional_File9_GOTermHeatmap/intracellular_pH_reduction.pdf]

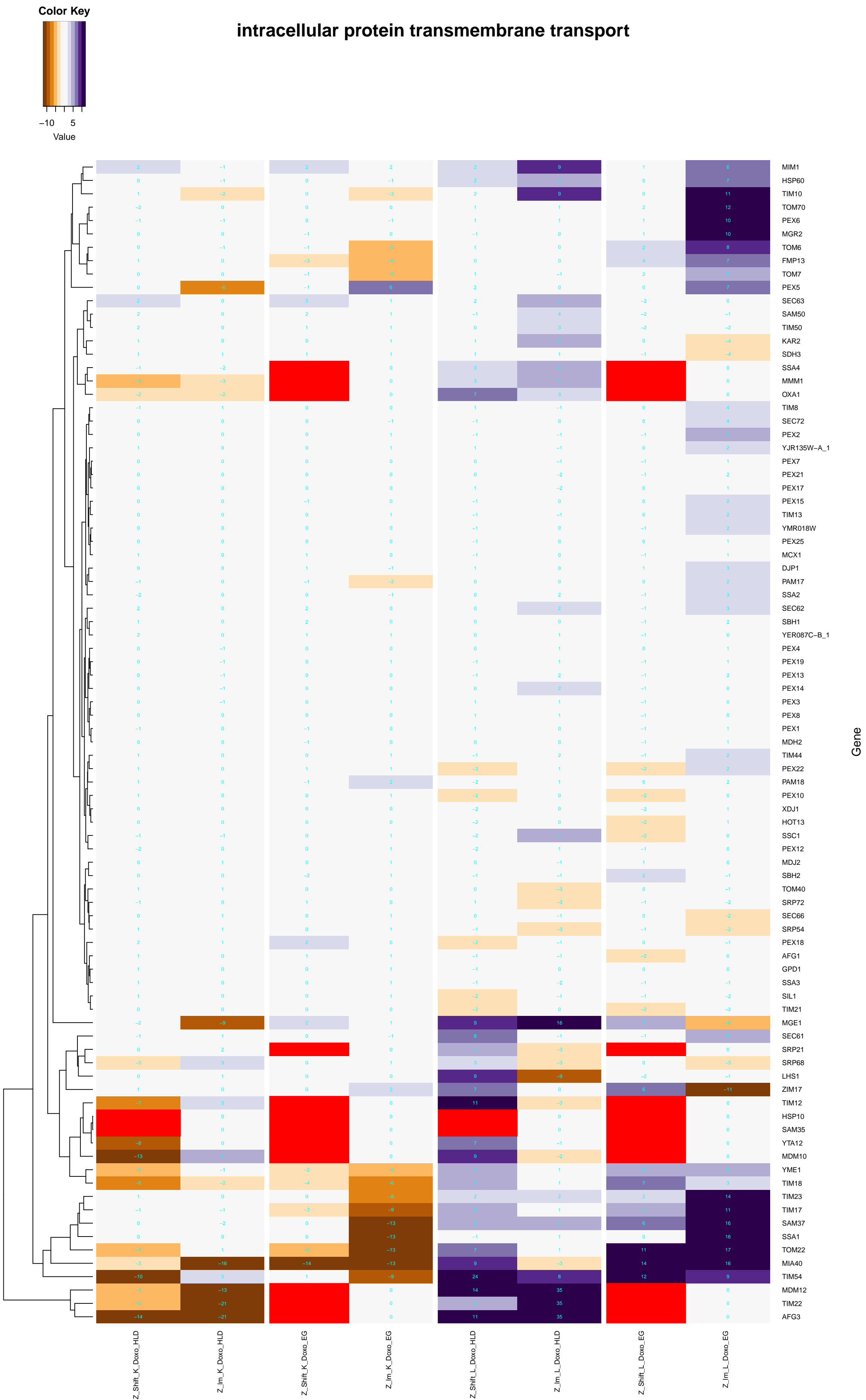

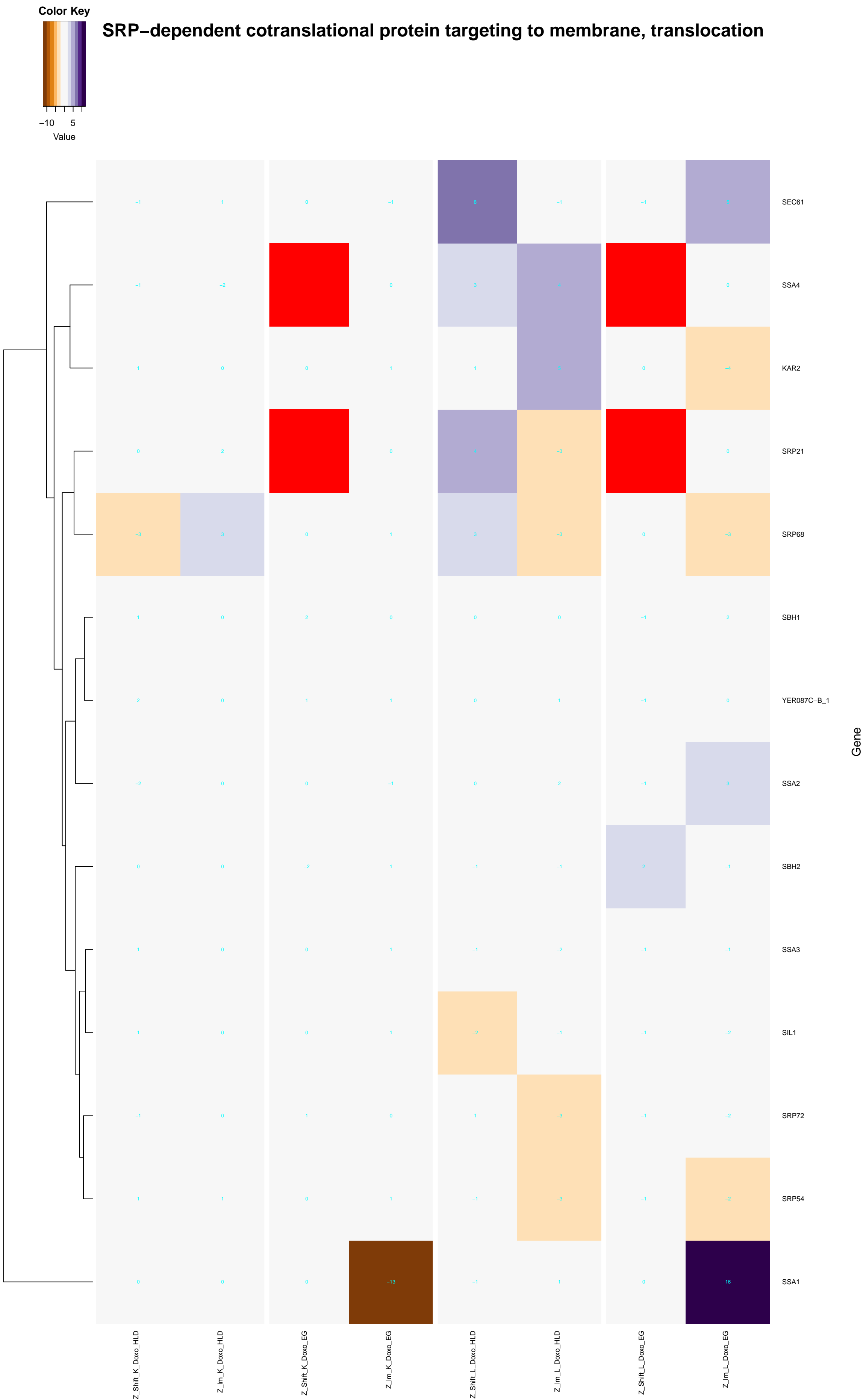

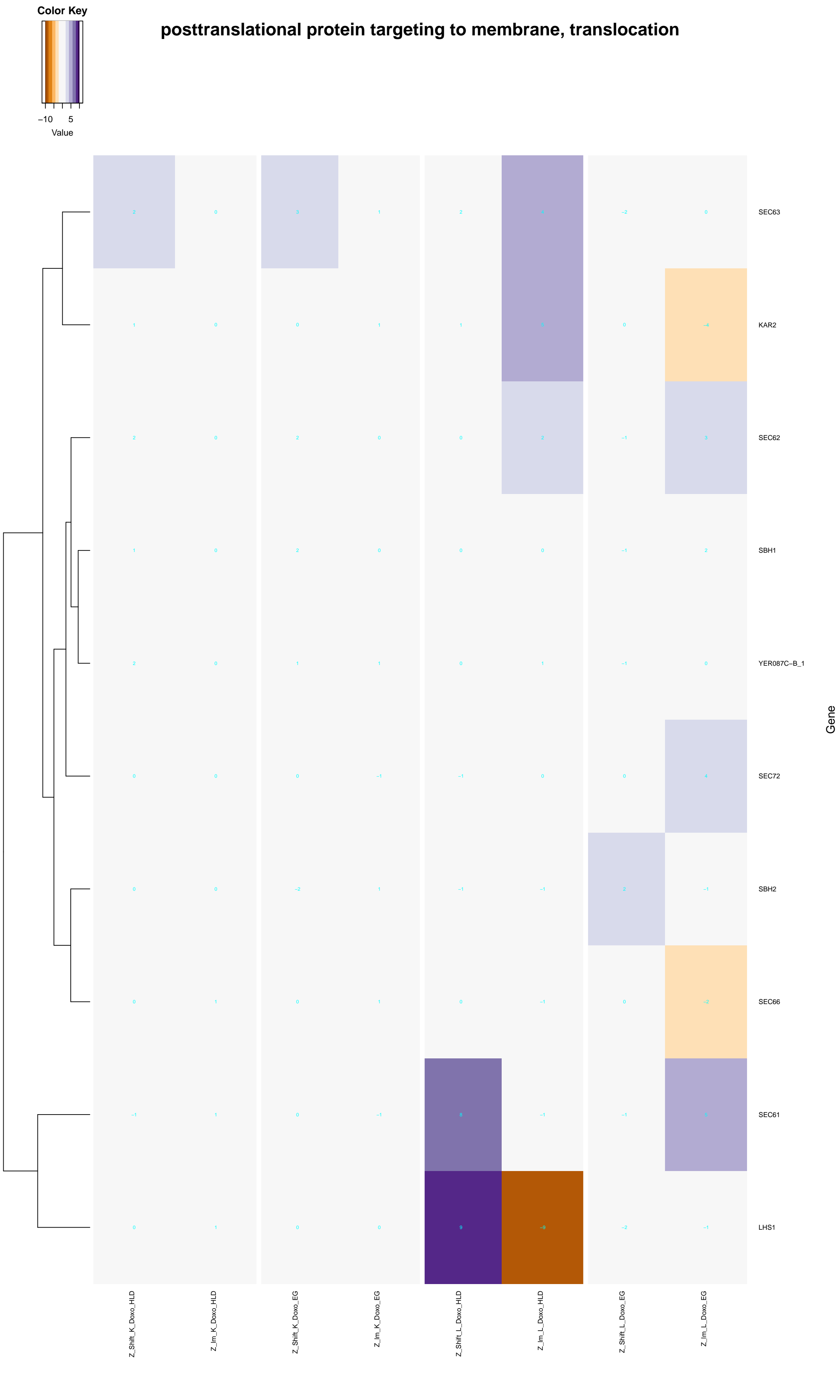

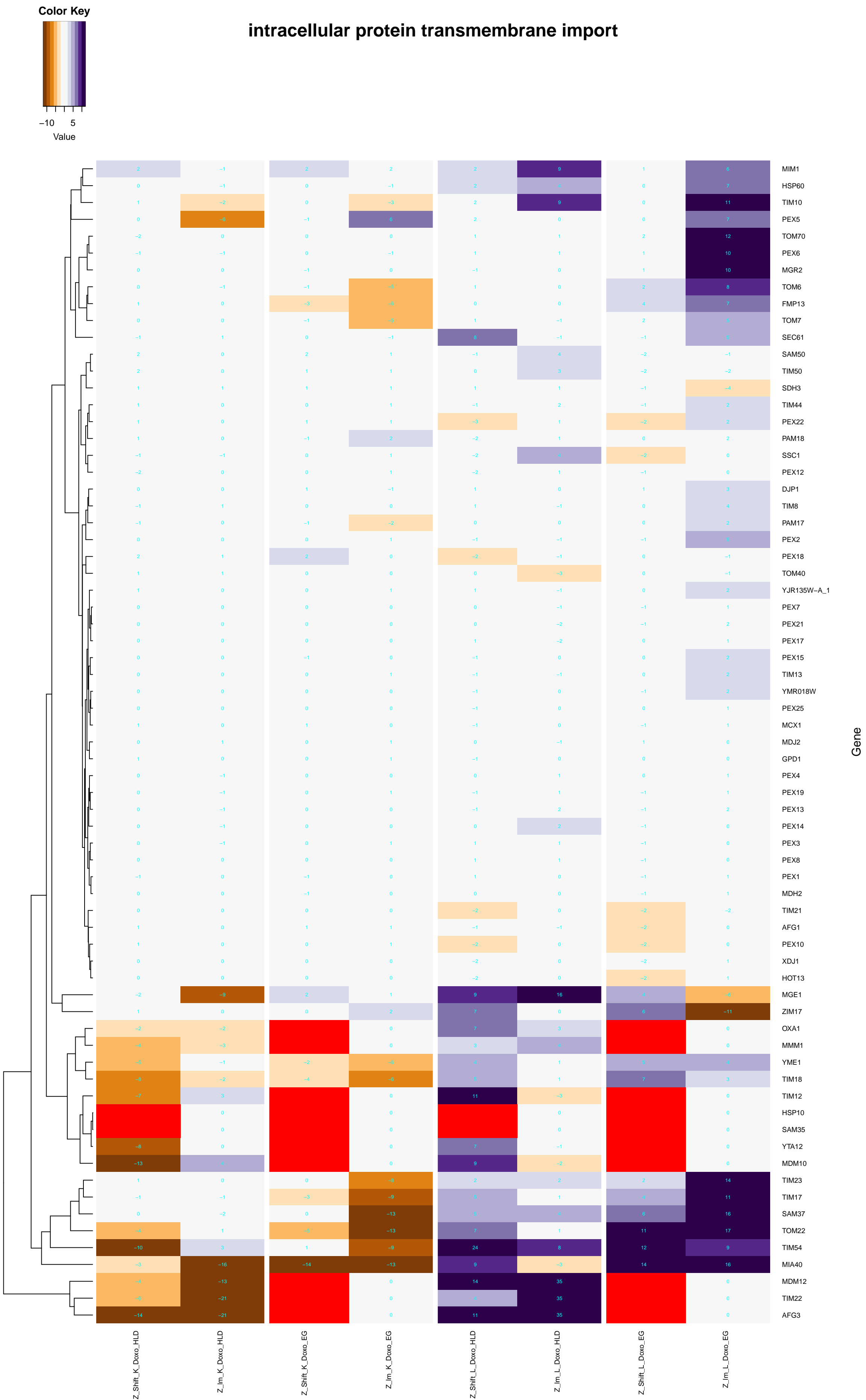

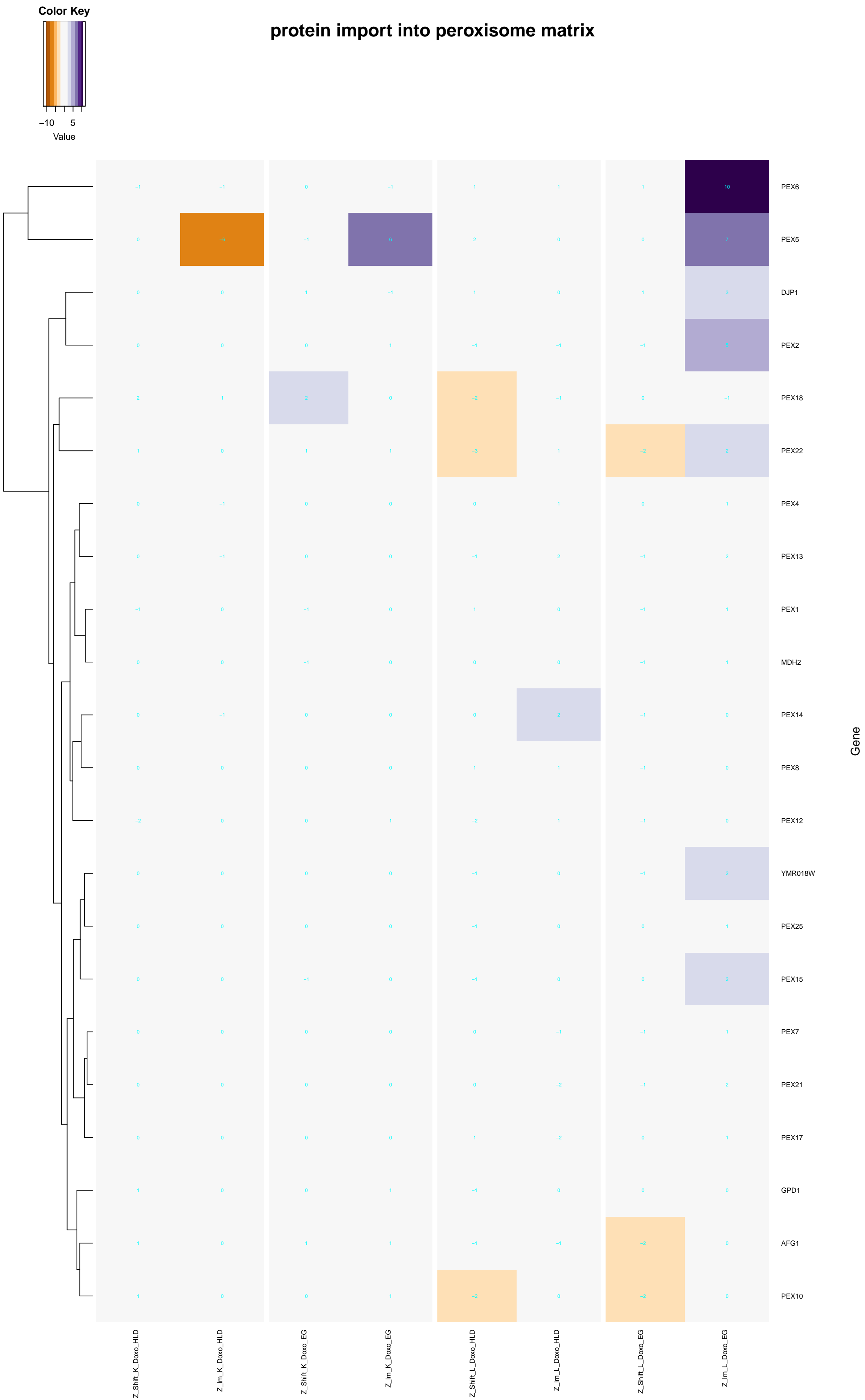

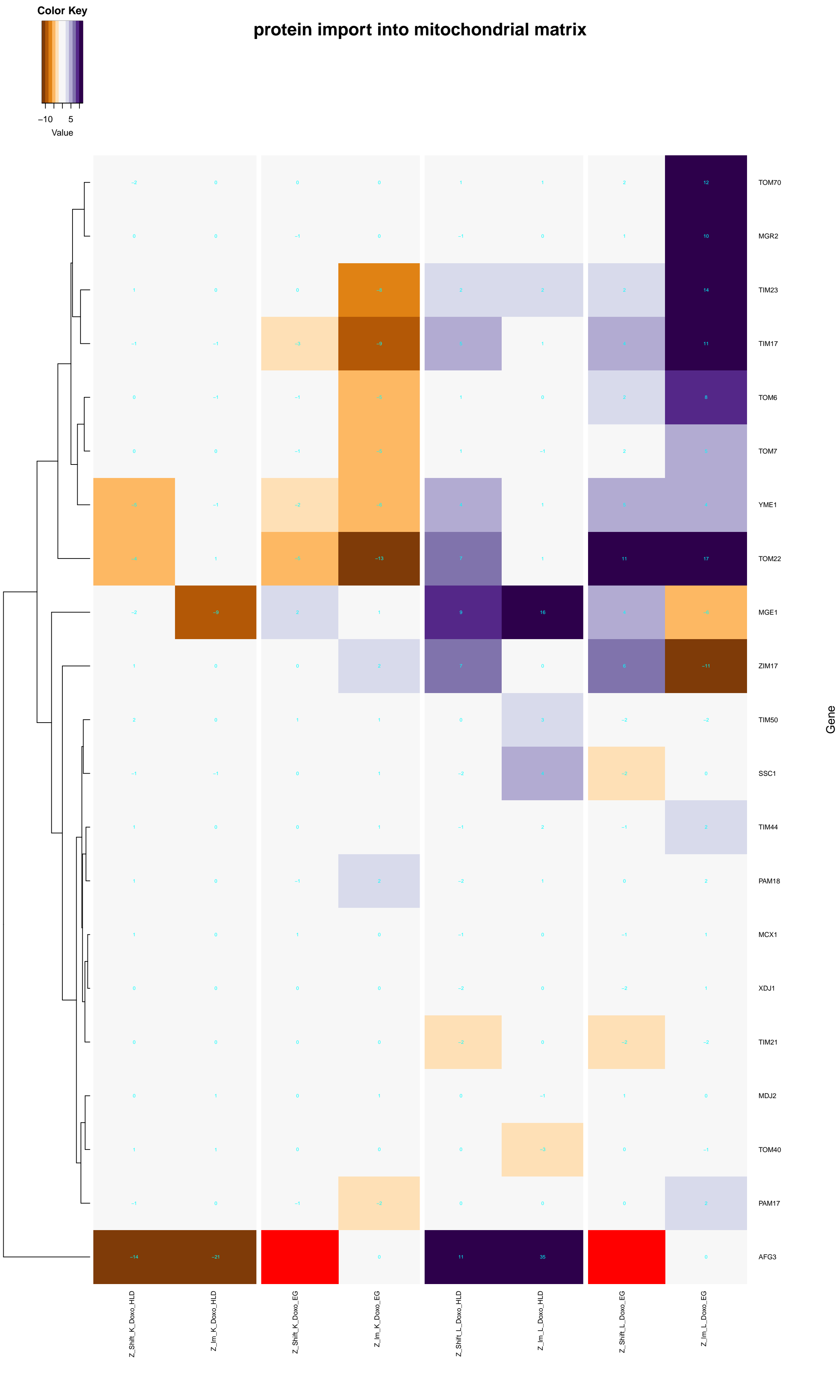

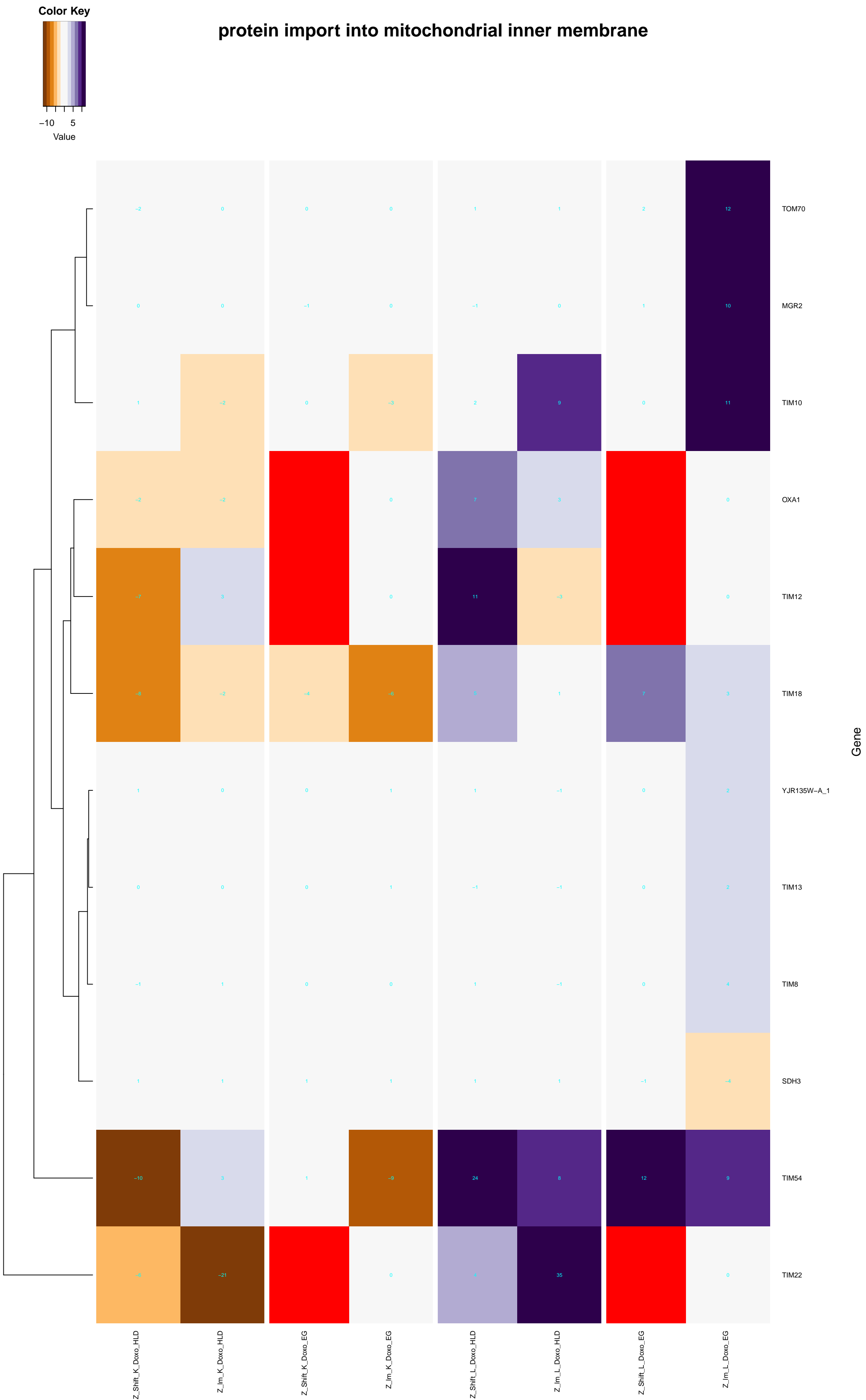

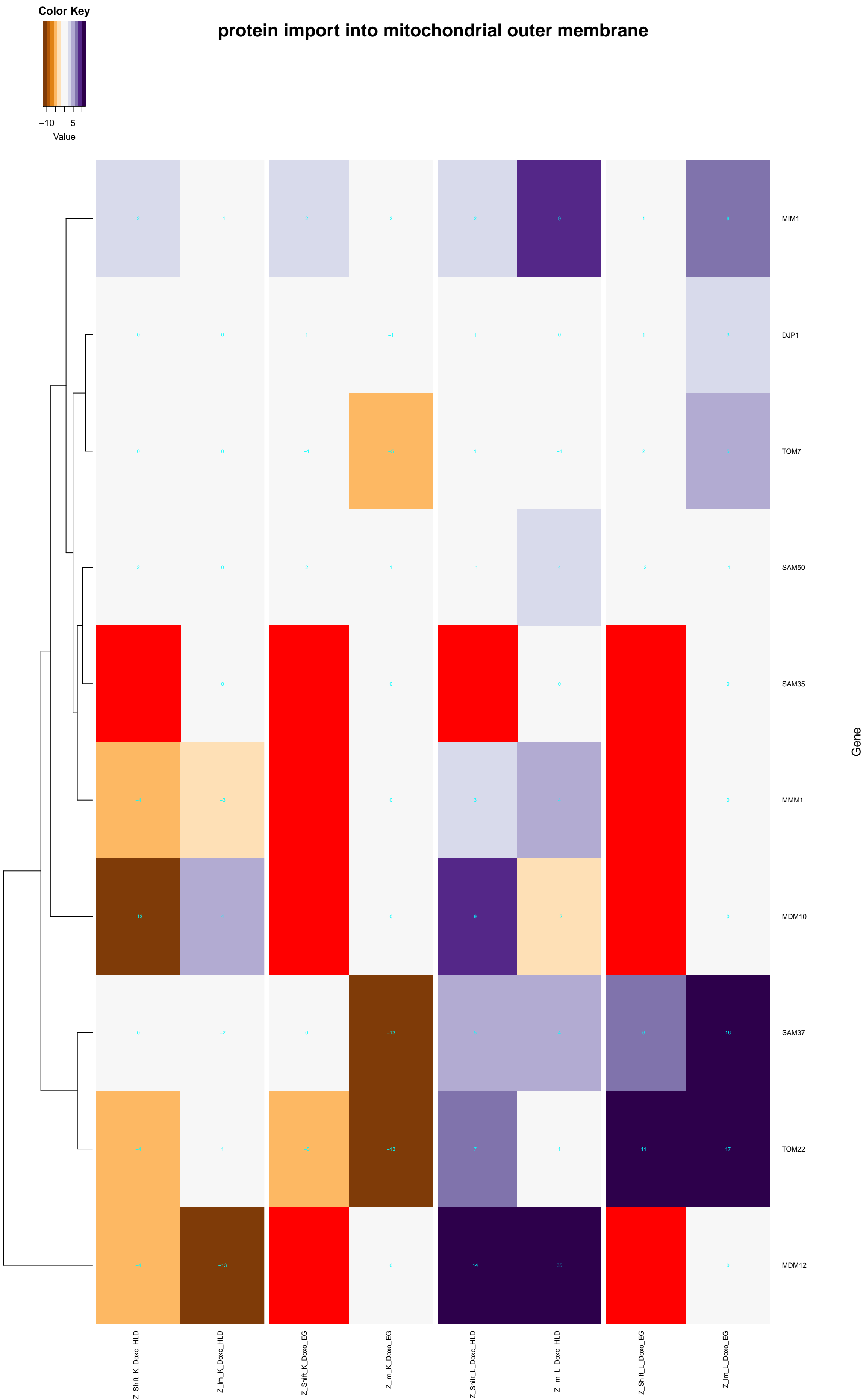

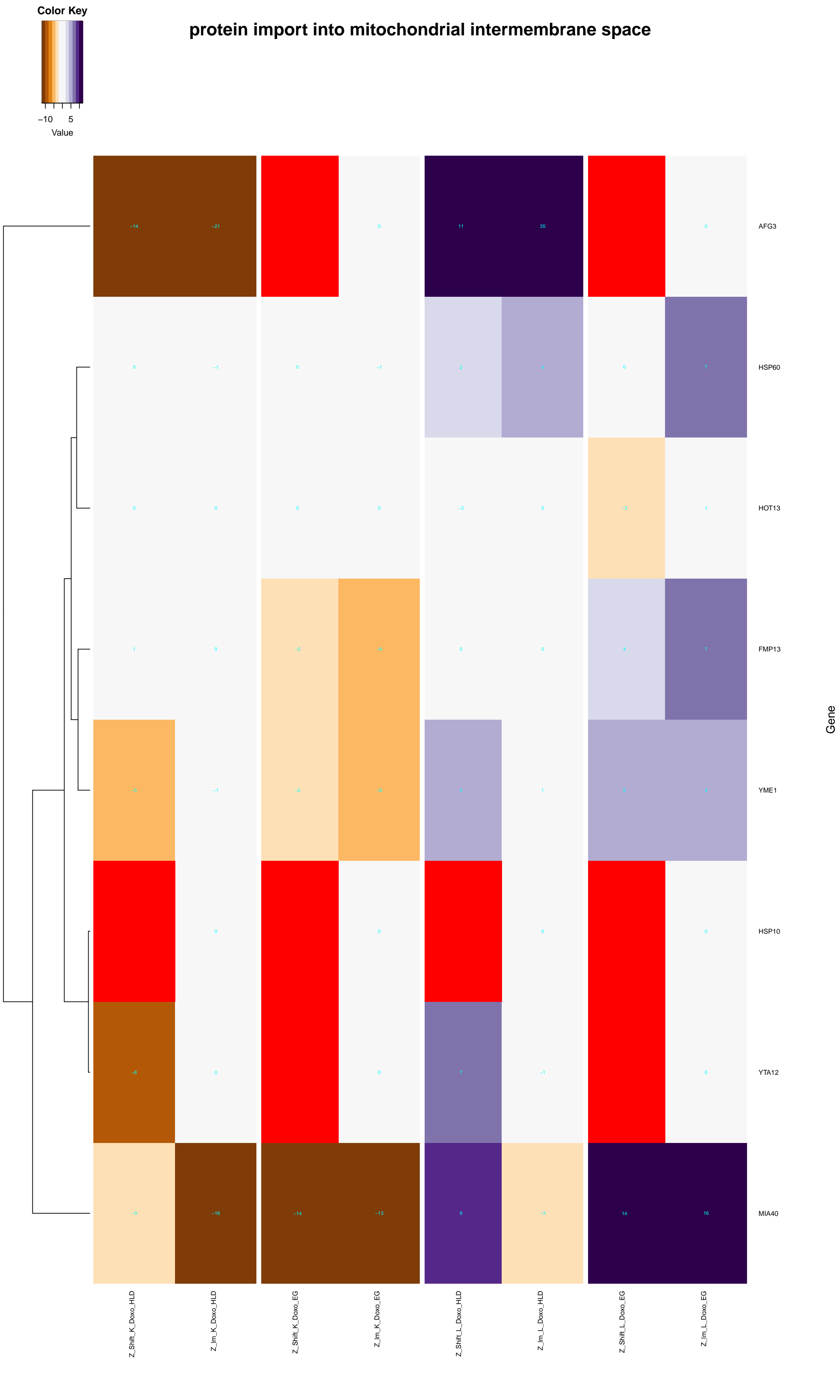

Supplement: Supplementary file 9 — Additional file 9. GO term-specific heatmaps for REMc/GTF-enriched clusters. GO term-specific heatmaps for significant GO process terms were generated as described in methods and Figs. 3 and 4. Any related child terms are presented in subsequent pages of the parent file name. GO terms with more than 100 children, with 2 or fewer genes annotated to the term, or a file size over 300KB are not shown. All heatmaps are generated with the same layout (see Figs. 3 and 4). [file 40170_2019_201_MOESM9_ESM.bz2 › Additional_File9_GOTermHeatmaps/Additional_File9_GOTermHeatmap/intracellular_protein_transmembrane_transport.pdf]

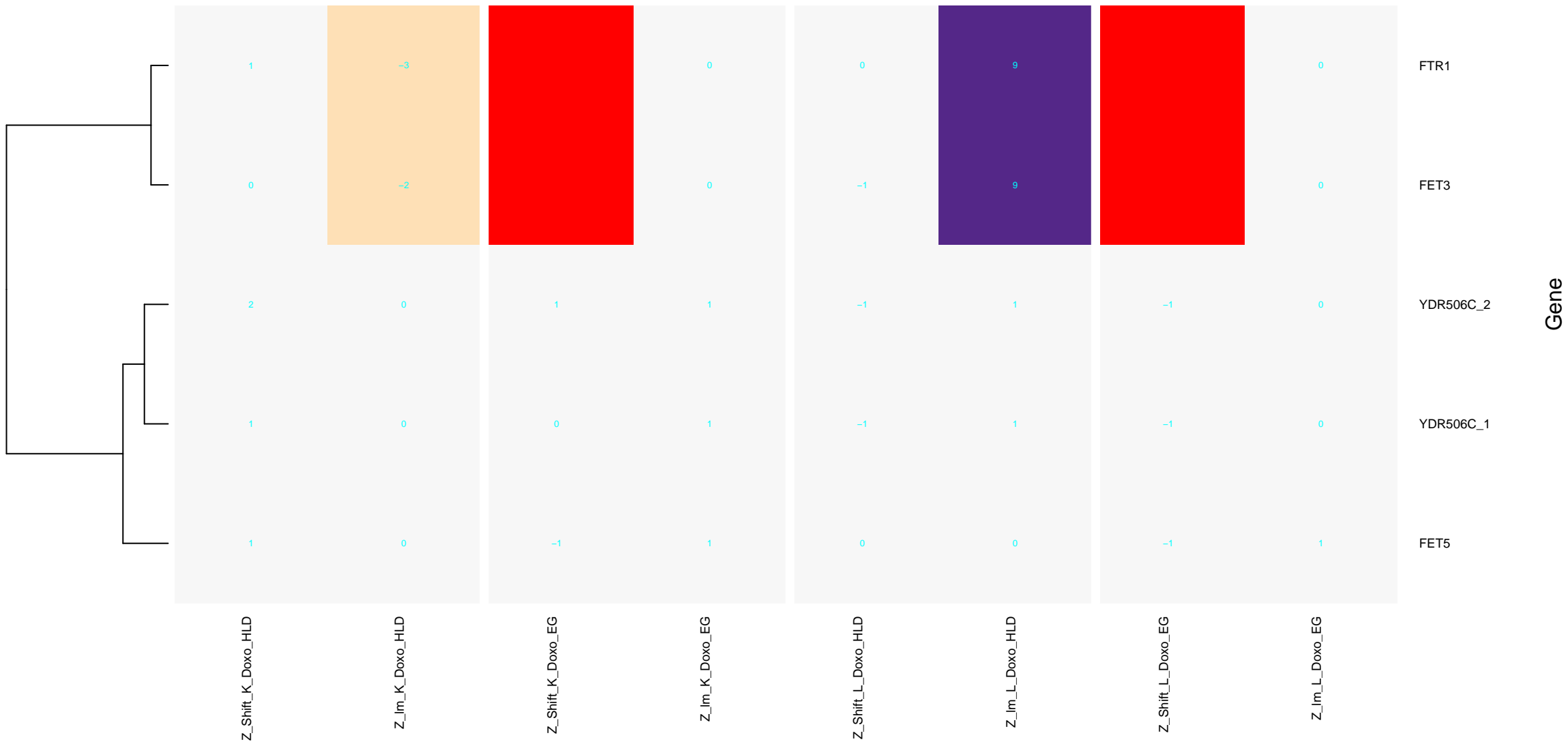

Supplement: Supplementary file 9 — Additional file 9. GO term-specific heatmaps for REMc/GTF-enriched clusters. GO term-specific heatmaps for significant GO process terms were generated as described in methods and Figs. 3 and 4. Any related child terms are presented in subsequent pages of the parent file name. GO terms with more than 100 children, with 2 or fewer genes annotated to the term, or a file size over 300KB are not shown. All heatmaps are generated with the same layout (see Figs. 3 and 4). [file 40170_2019_201_MOESM9_ESM.bz2 › Additional_File9_GOTermHeatmaps/Additional_File9_GOTermHeatmap/iron_assimilation.pdf]

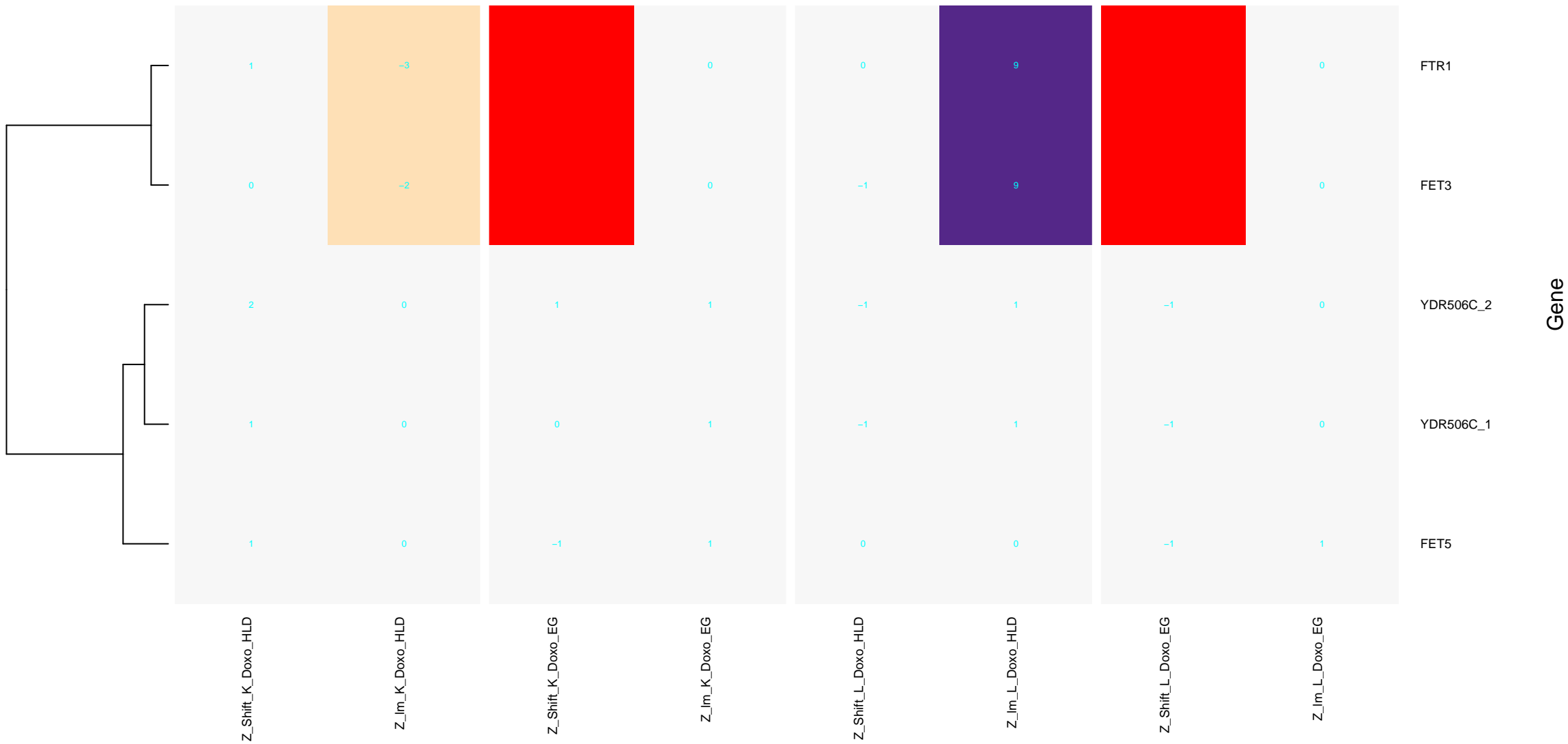

Supplement: Supplementary file 9 — Additional file 9. GO term-specific heatmaps for REMc/GTF-enriched clusters. GO term-specific heatmaps for significant GO process terms were generated as described in methods and Figs. 3 and 4. Any related child terms are presented in subsequent pages of the parent file name. GO terms with more than 100 children, with 2 or fewer genes annotated to the term, or a file size over 300KB are not shown. All heatmaps are generated with the same layout (see Figs. 3 and 4). [file 40170_2019_201_MOESM9_ESM.bz2 › Additional_File9_GOTermHeatmaps/Additional_File9_GOTermHeatmap/iron_assimilation_by_reduction_and_transport.pdf]

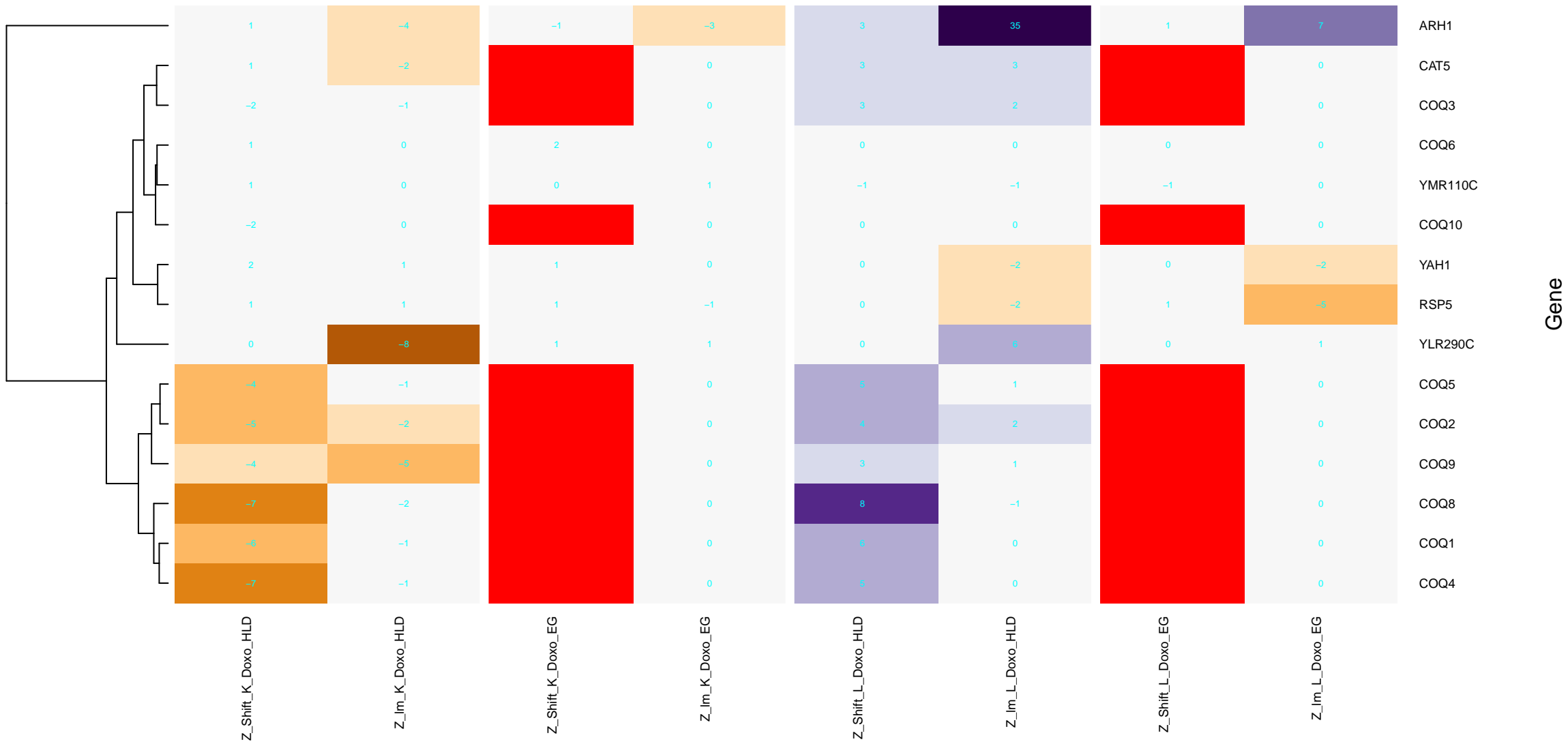

Supplement: Supplementary file 9 — Additional file 9. GO term-specific heatmaps for REMc/GTF-enriched clusters. GO term-specific heatmaps for significant GO process terms were generated as described in methods and Figs. 3 and 4. Any related child terms are presented in subsequent pages of the parent file name. GO terms with more than 100 children, with 2 or fewer genes annotated to the term, or a file size over 300KB are not shown. All heatmaps are generated with the same layout (see Figs. 3 and 4). [file 40170_2019_201_MOESM9_ESM.bz2 › Additional_File9_GOTermHeatmaps/Additional_File9_GOTermHeatmap/ketone_biosynthetic_process.pdf]

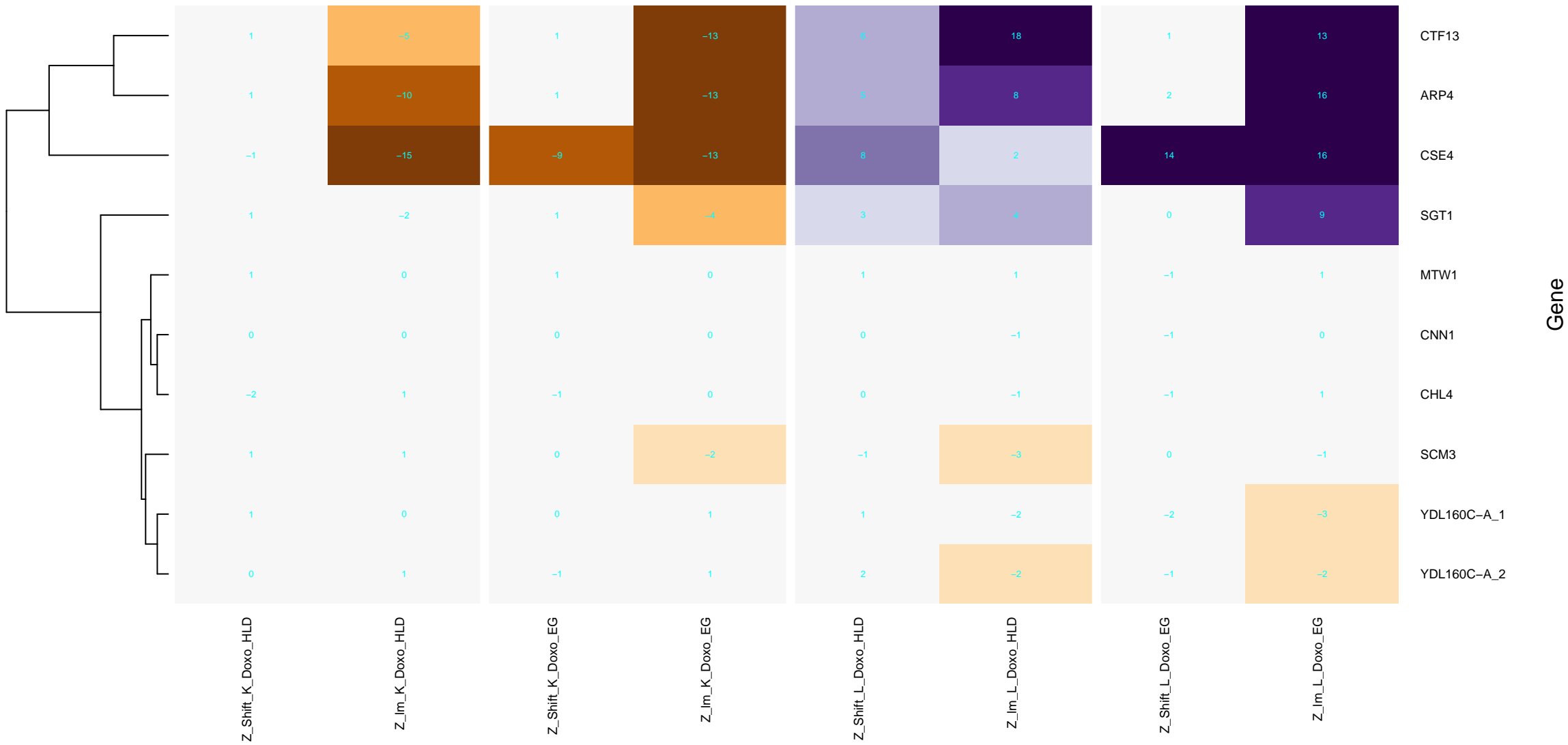

Supplement: Supplementary file 9 — Additional file 9. GO term-specific heatmaps for REMc/GTF-enriched clusters. GO term-specific heatmaps for significant GO process terms were generated as described in methods and Figs. 3 and 4. Any related child terms are presented in subsequent pages of the parent file name. GO terms with more than 100 children, with 2 or fewer genes annotated to the term, or a file size over 300KB are not shown. All heatmaps are generated with the same layout (see Figs. 3 and 4). [file 40170_2019_201_MOESM9_ESM.bz2 › Additional_File9_GOTermHeatmaps/Additional_File9_GOTermHeatmap/kinetochore_assembly.pdf]

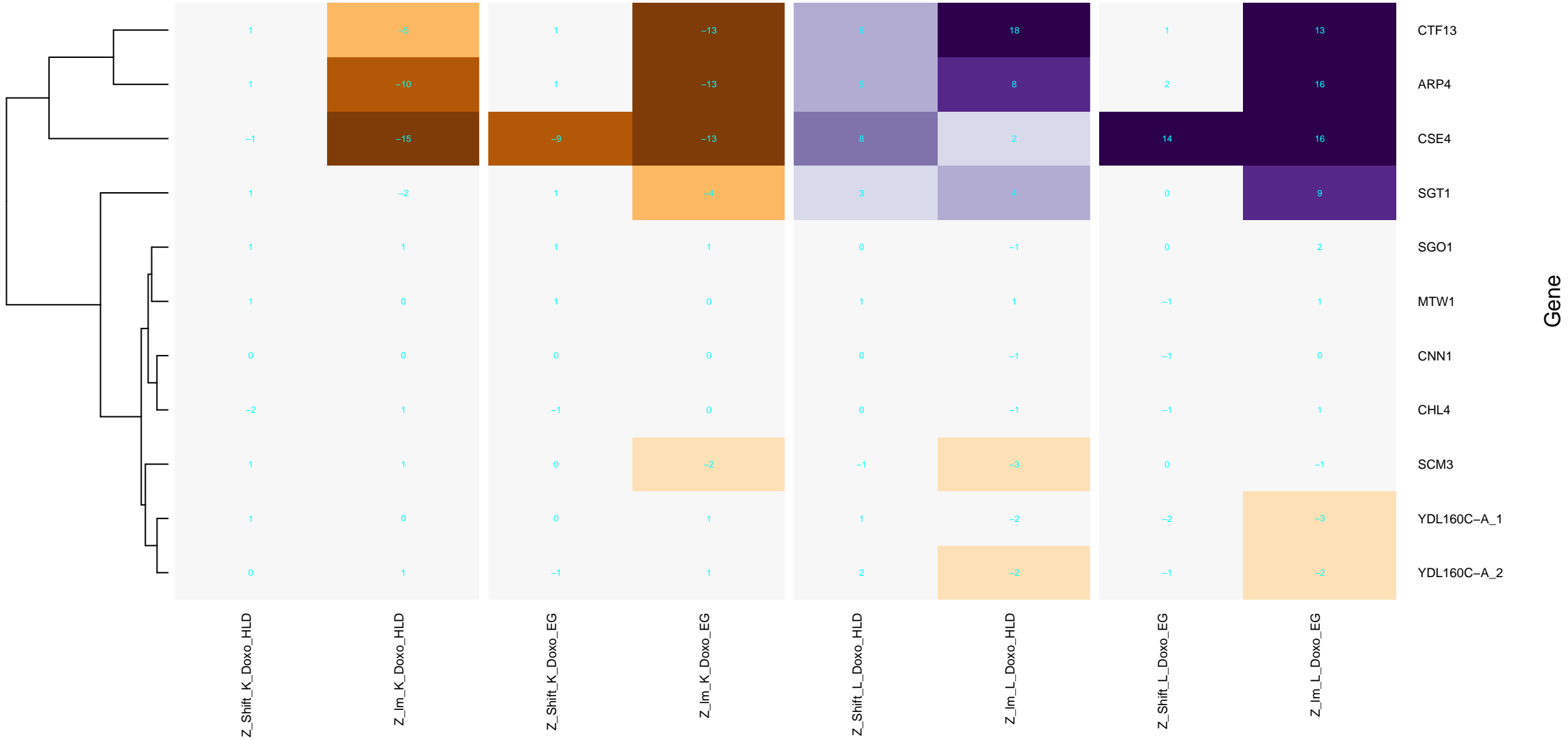

Supplement: Supplementary file 9 — Additional file 9. GO term-specific heatmaps for REMc/GTF-enriched clusters. GO term-specific heatmaps for significant GO process terms were generated as described in methods and Figs. 3 and 4. Any related child terms are presented in subsequent pages of the parent file name. GO terms with more than 100 children, with 2 or fewer genes annotated to the term, or a file size over 300KB are not shown. All heatmaps are generated with the same layout (see Figs. 3 and 4). [file 40170_2019_201_MOESM9_ESM.bz2 › Additional_File9_GOTermHeatmaps/Additional_File9_GOTermHeatmap/kinetochore_organization.pdf]

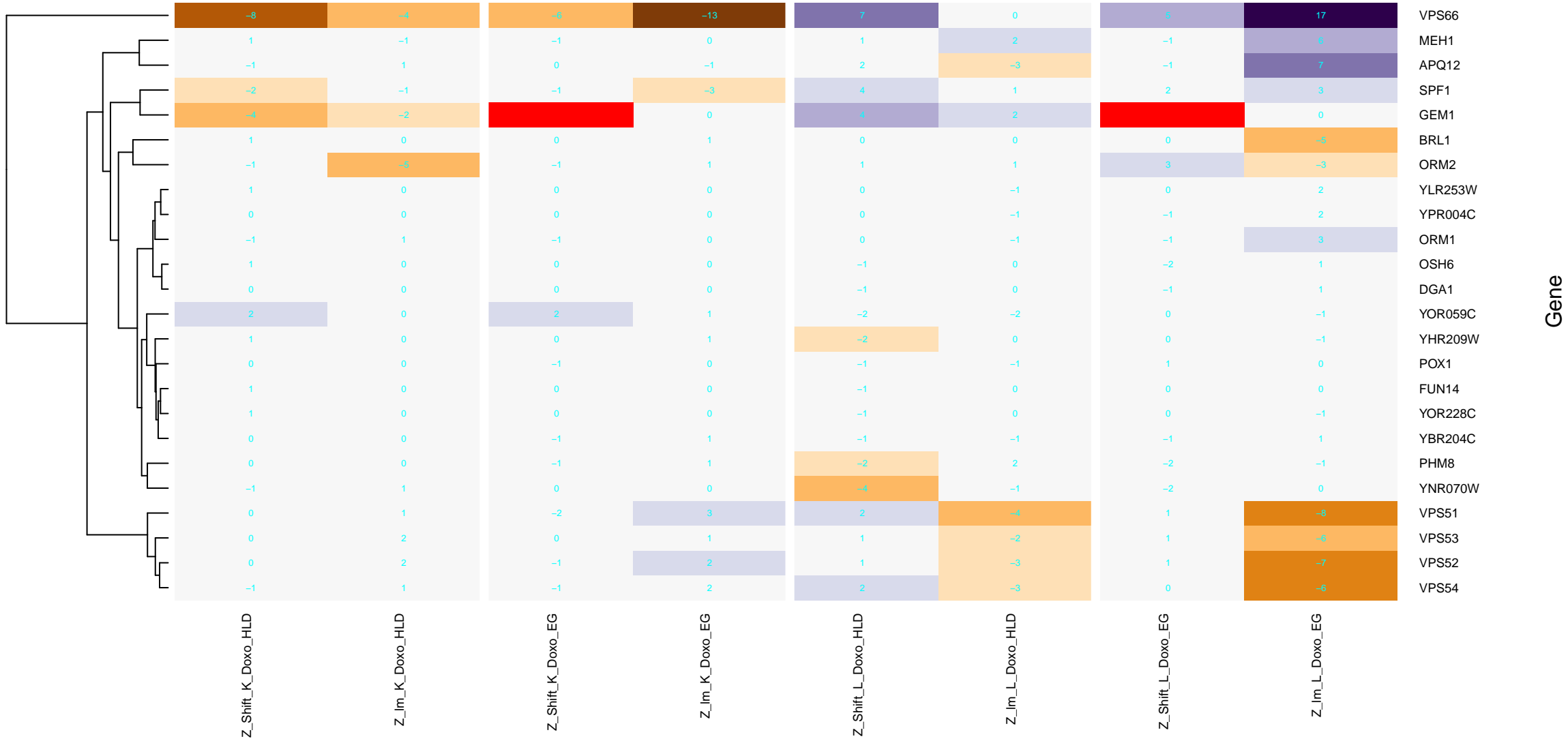

Supplement: Supplementary file 9 — Additional file 9. GO term-specific heatmaps for REMc/GTF-enriched clusters. GO term-specific heatmaps for significant GO process terms were generated as described in methods and Figs. 3 and 4. Any related child terms are presented in subsequent pages of the parent file name. GO terms with more than 100 children, with 2 or fewer genes annotated to the term, or a file size over 300KB are not shown. All heatmaps are generated with the same layout (see Figs. 3 and 4). [file 40170_2019_201_MOESM9_ESM.bz2 › Additional_File9_GOTermHeatmaps/Additional_File9_GOTermHeatmap/lipid_homeostasis.pdf]

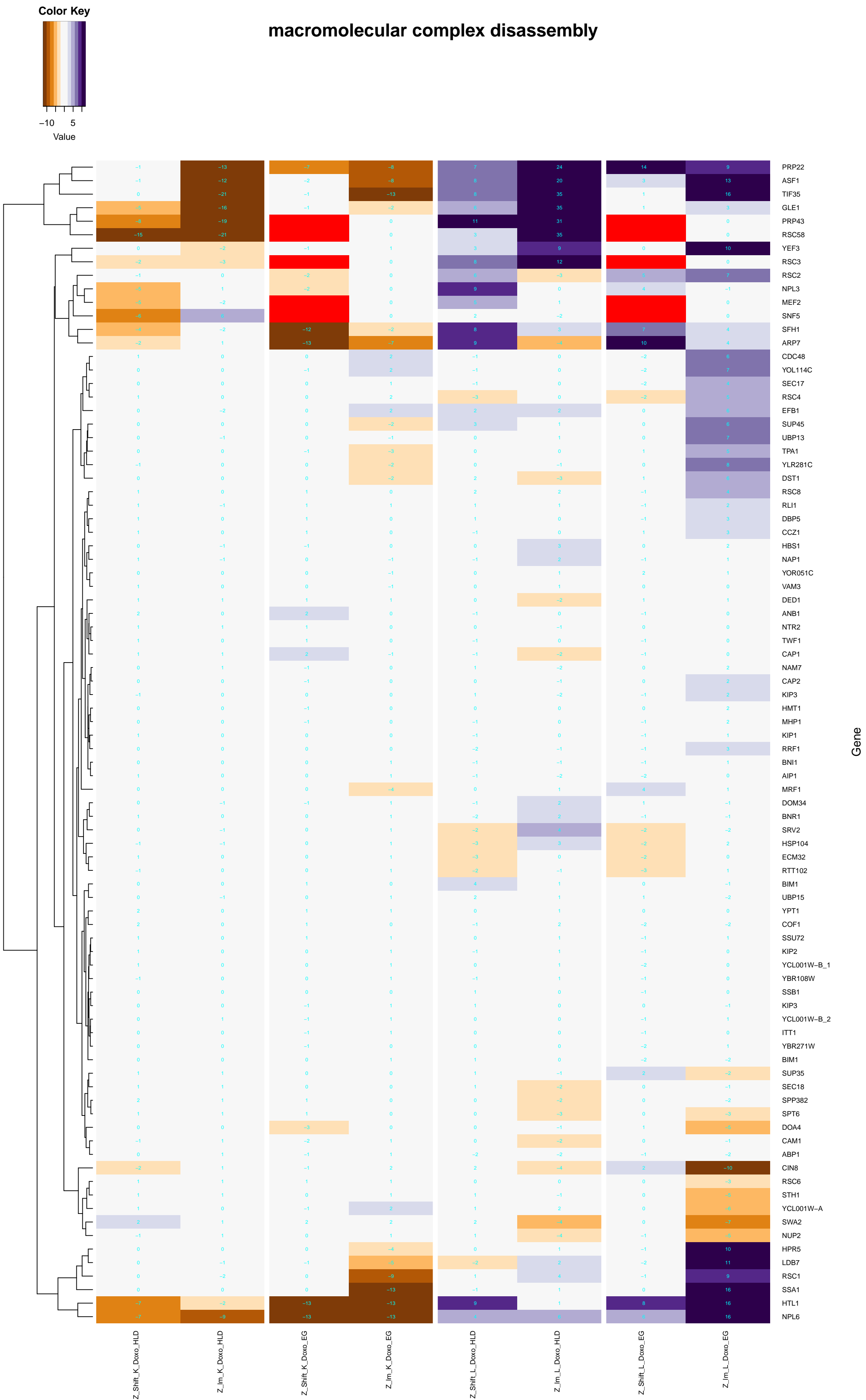

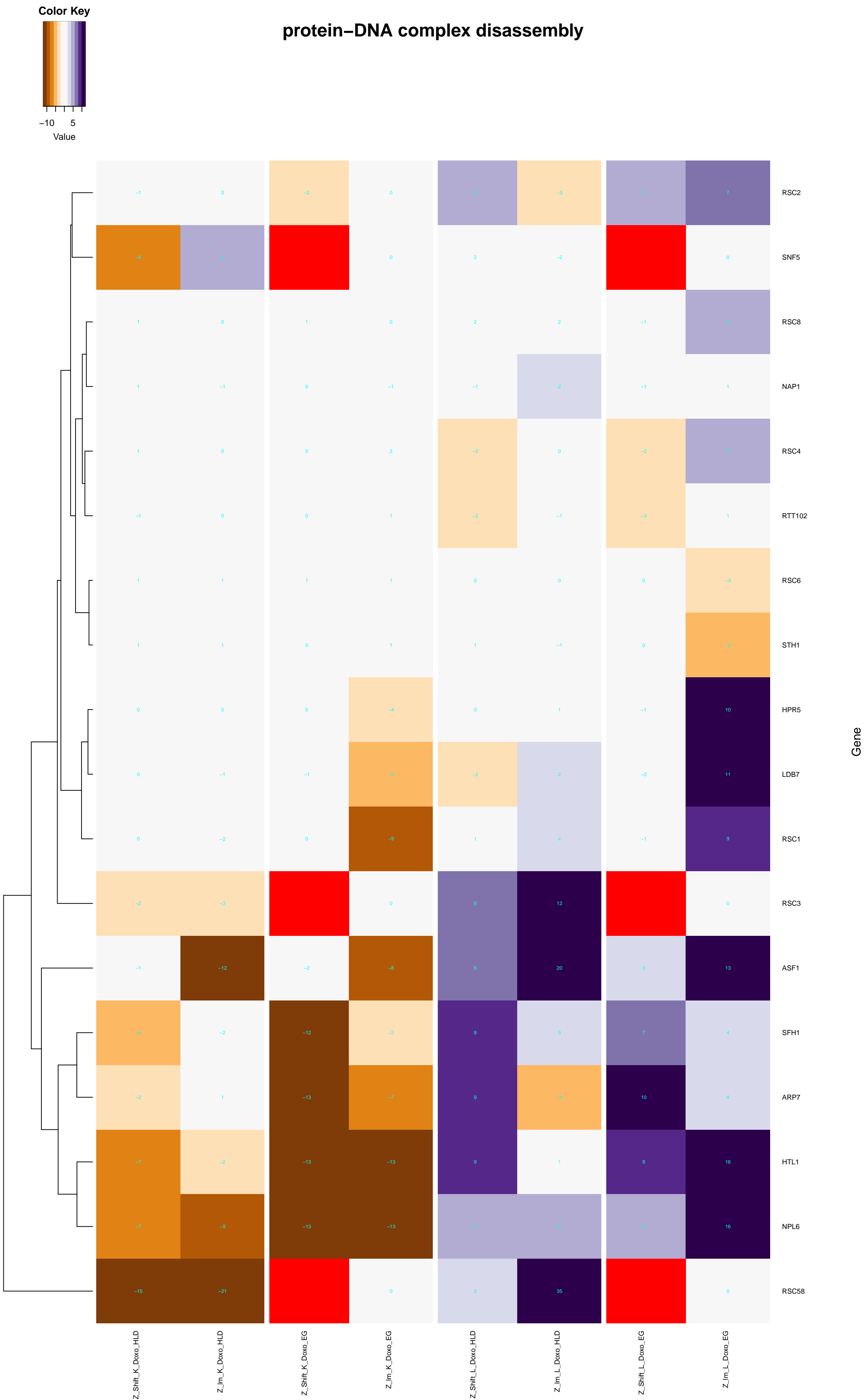

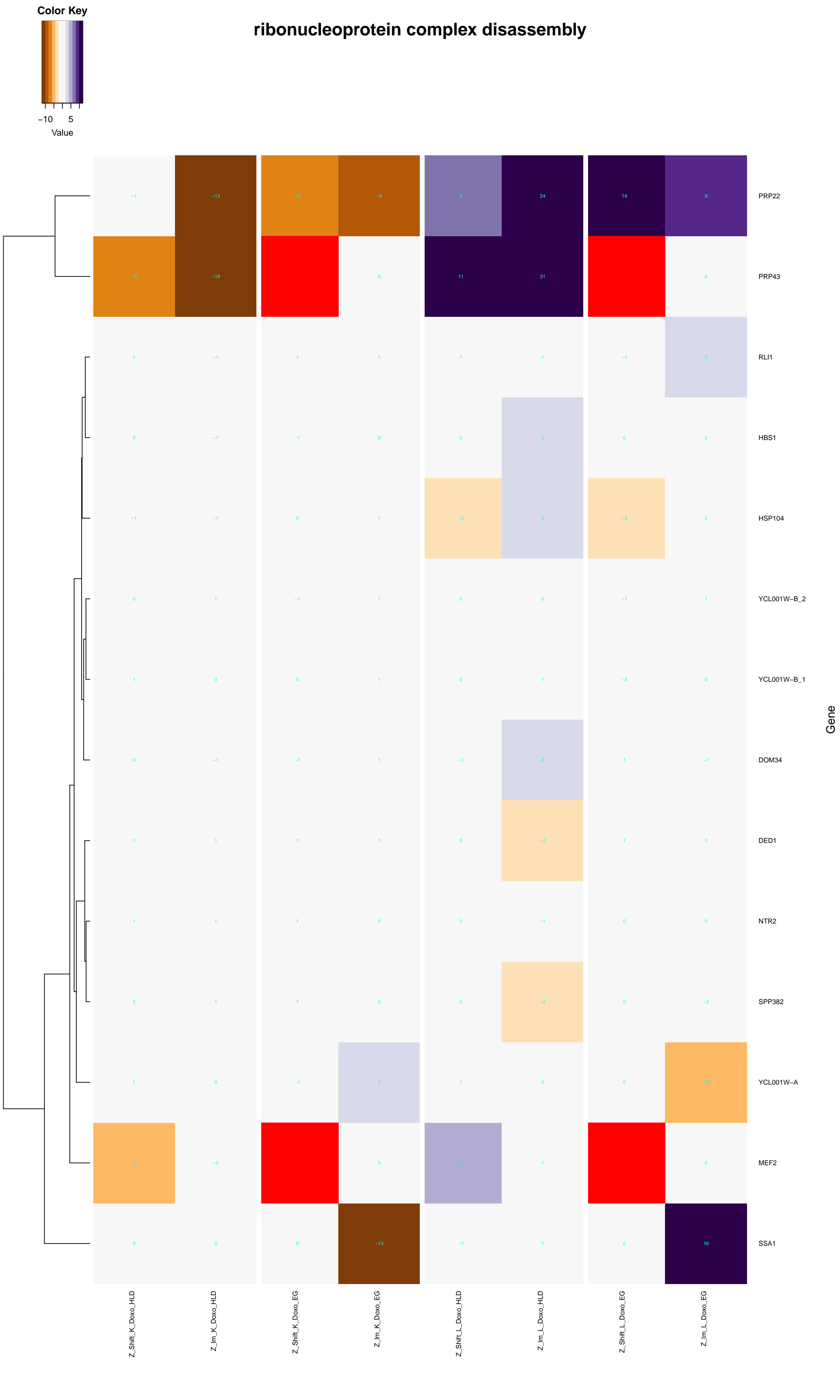

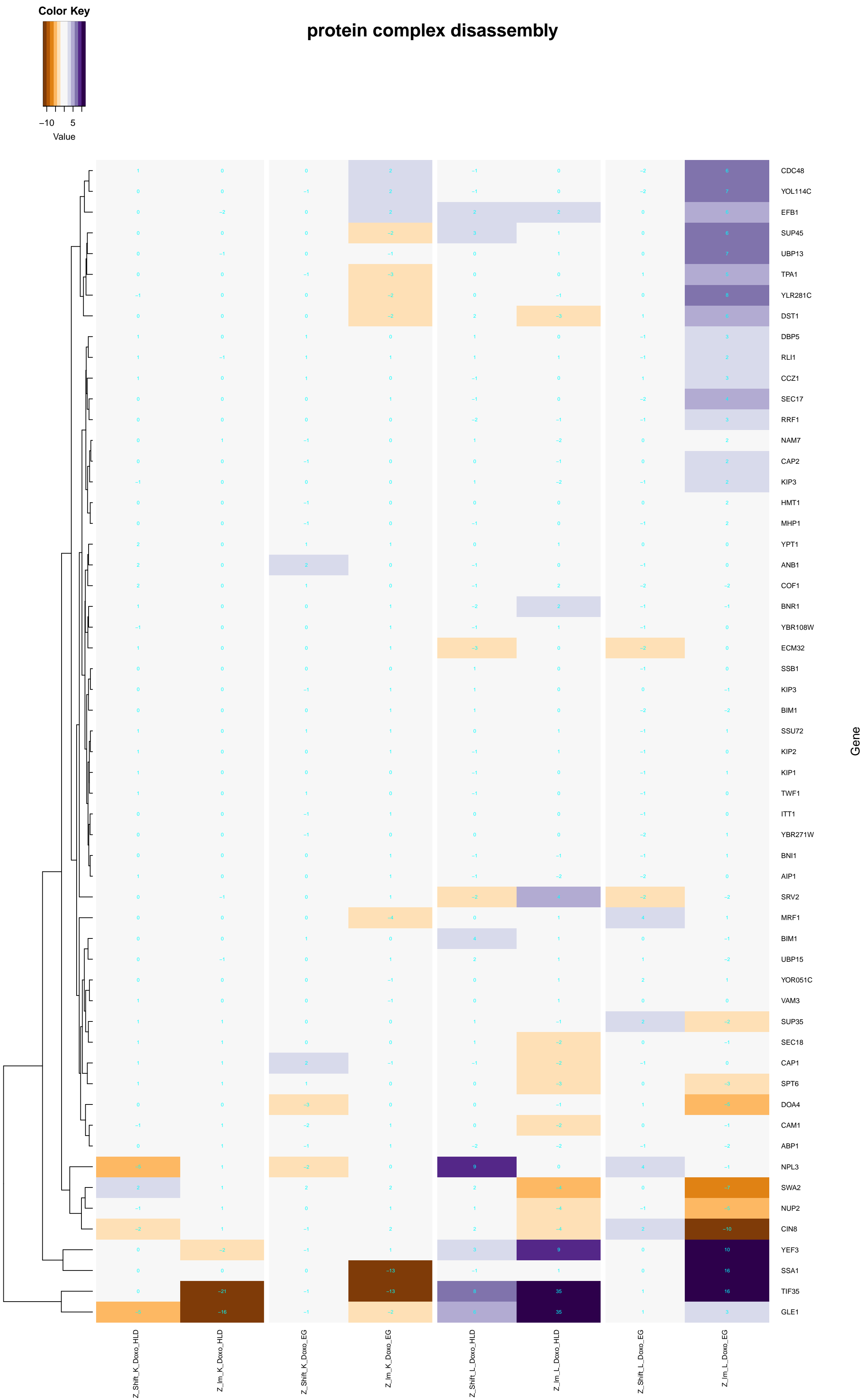

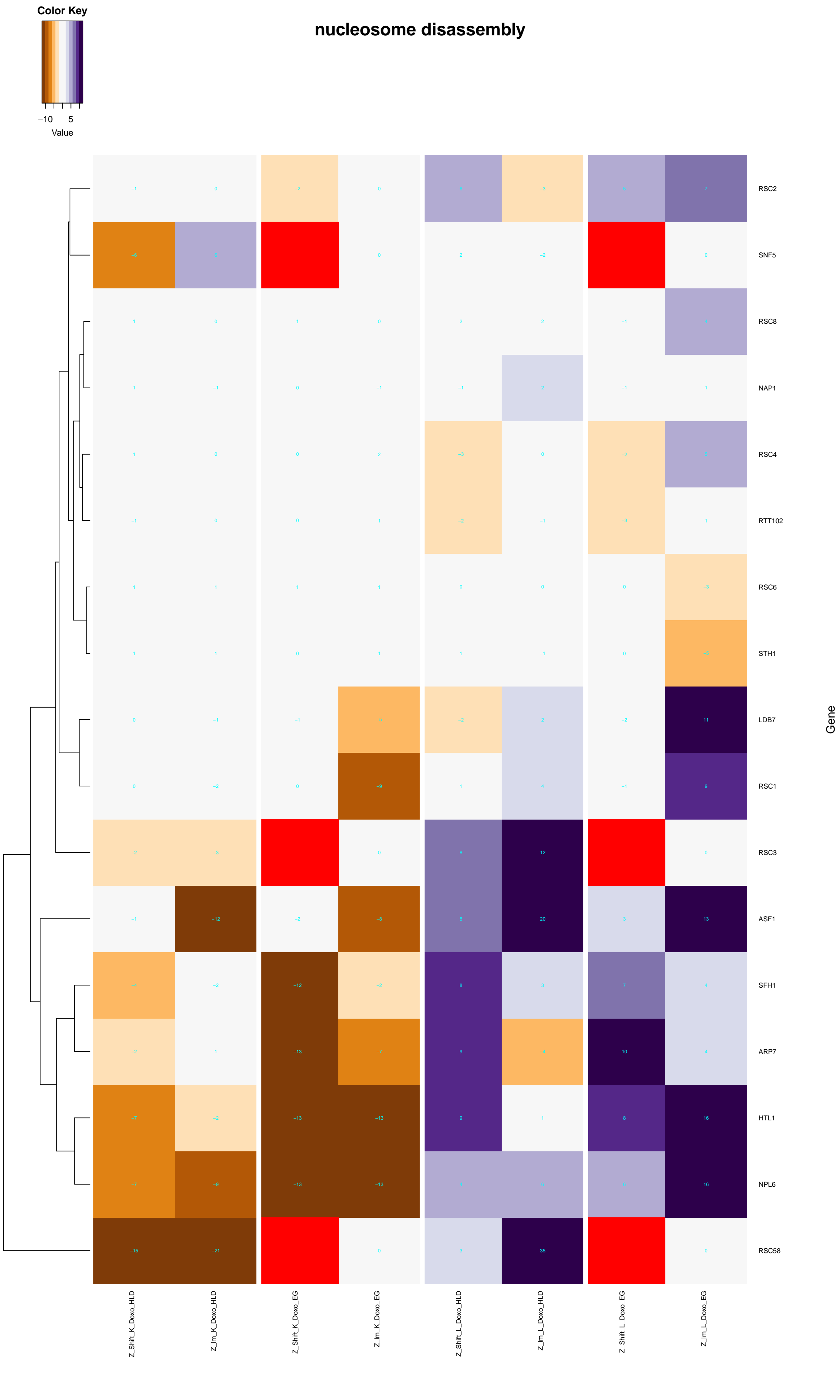

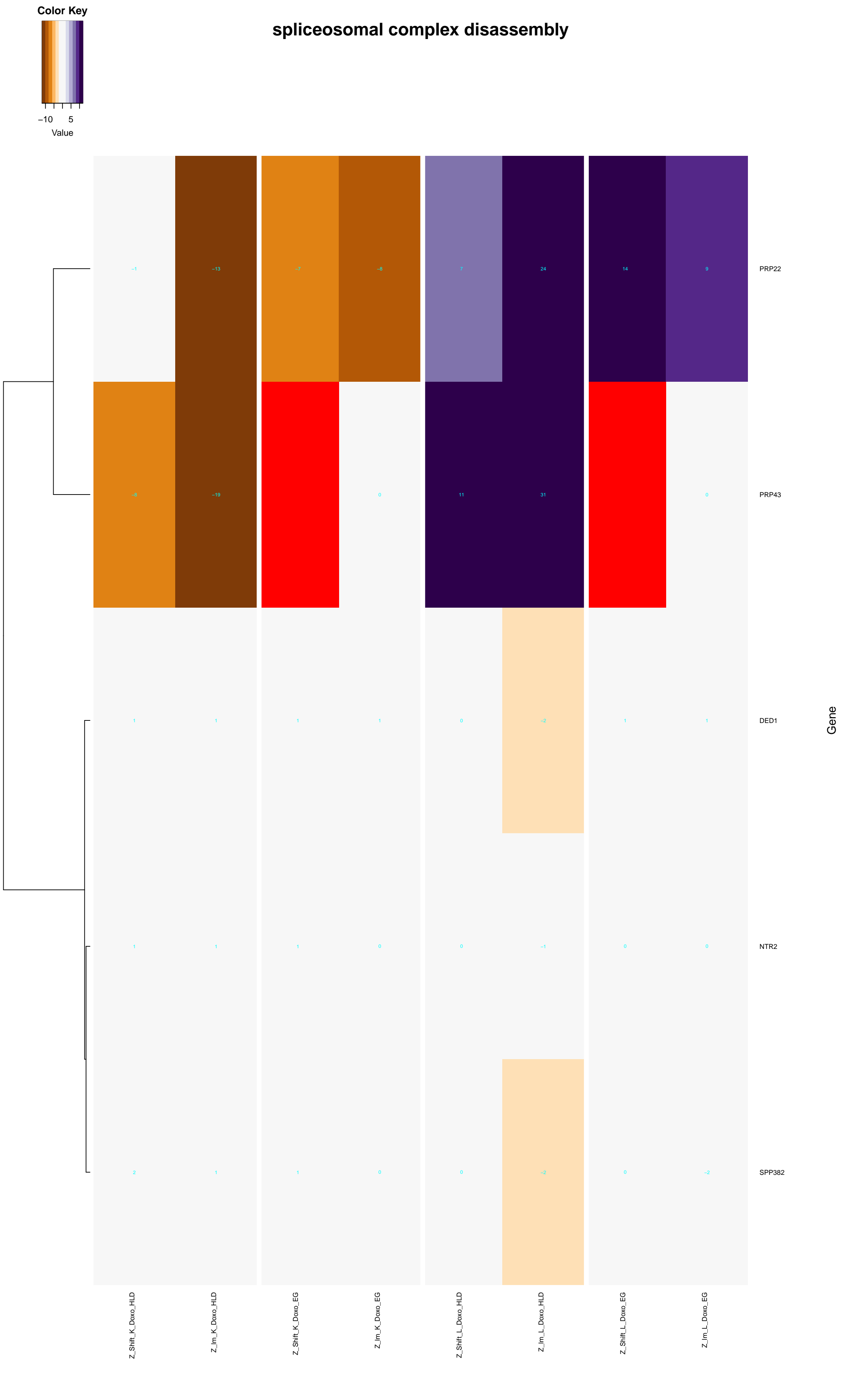

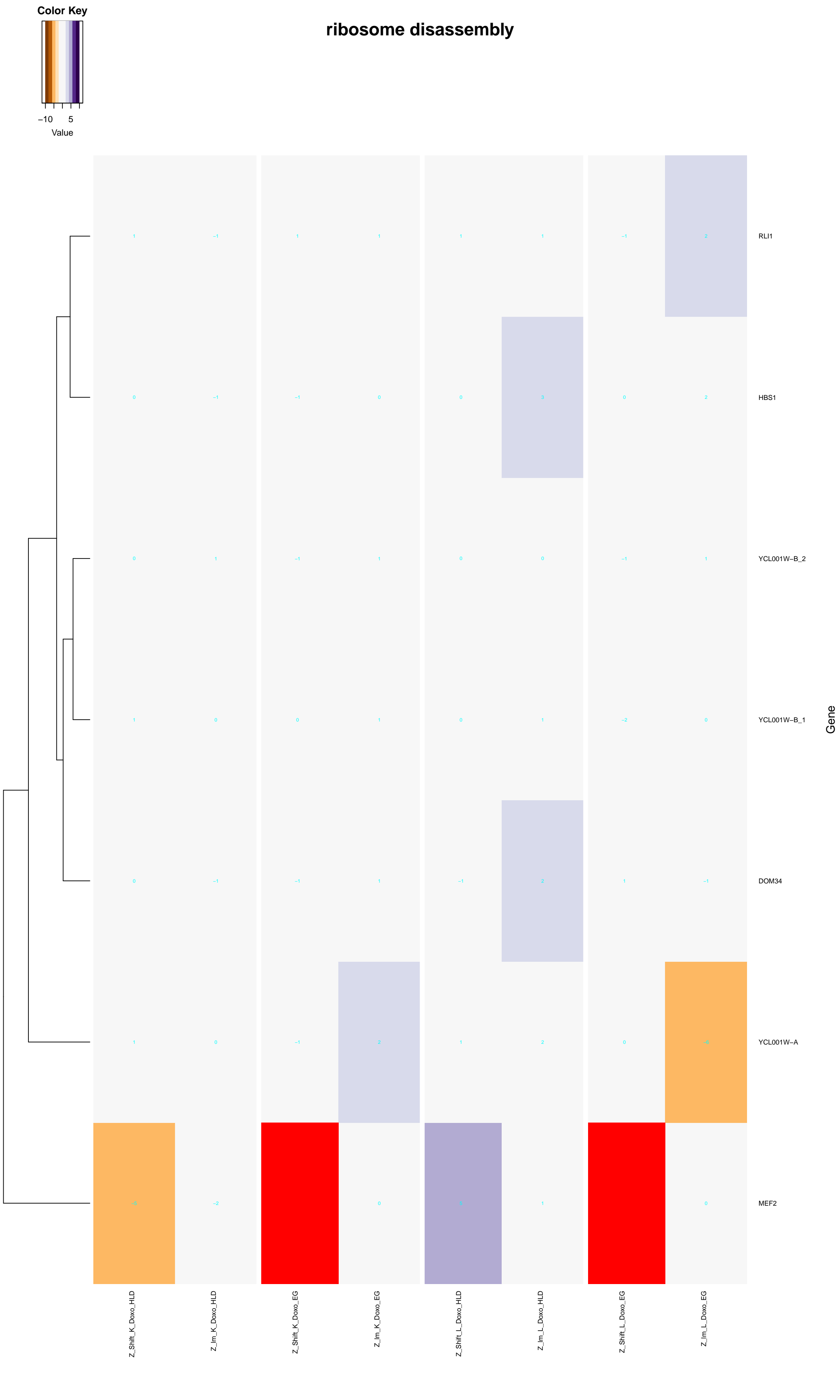

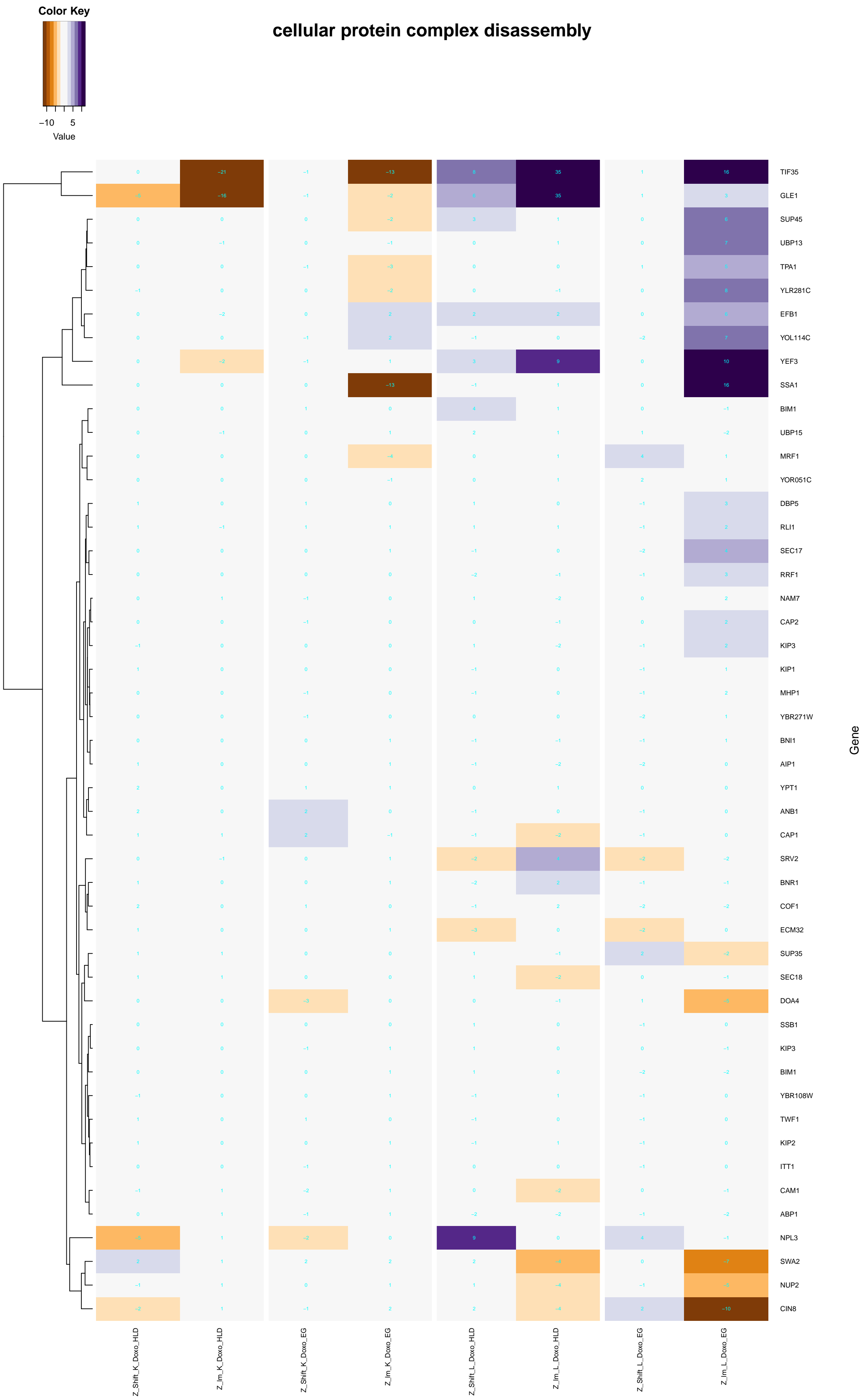

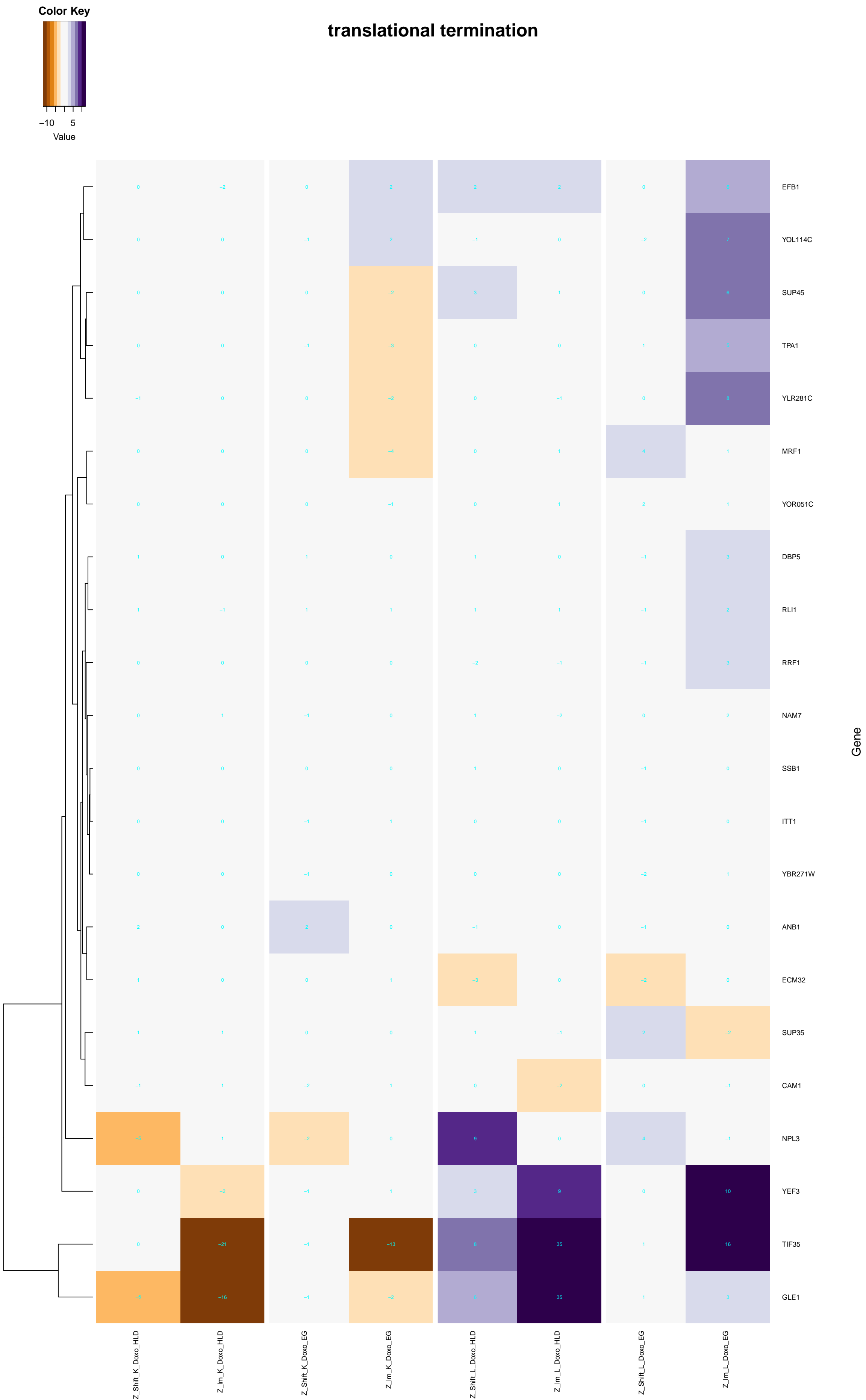

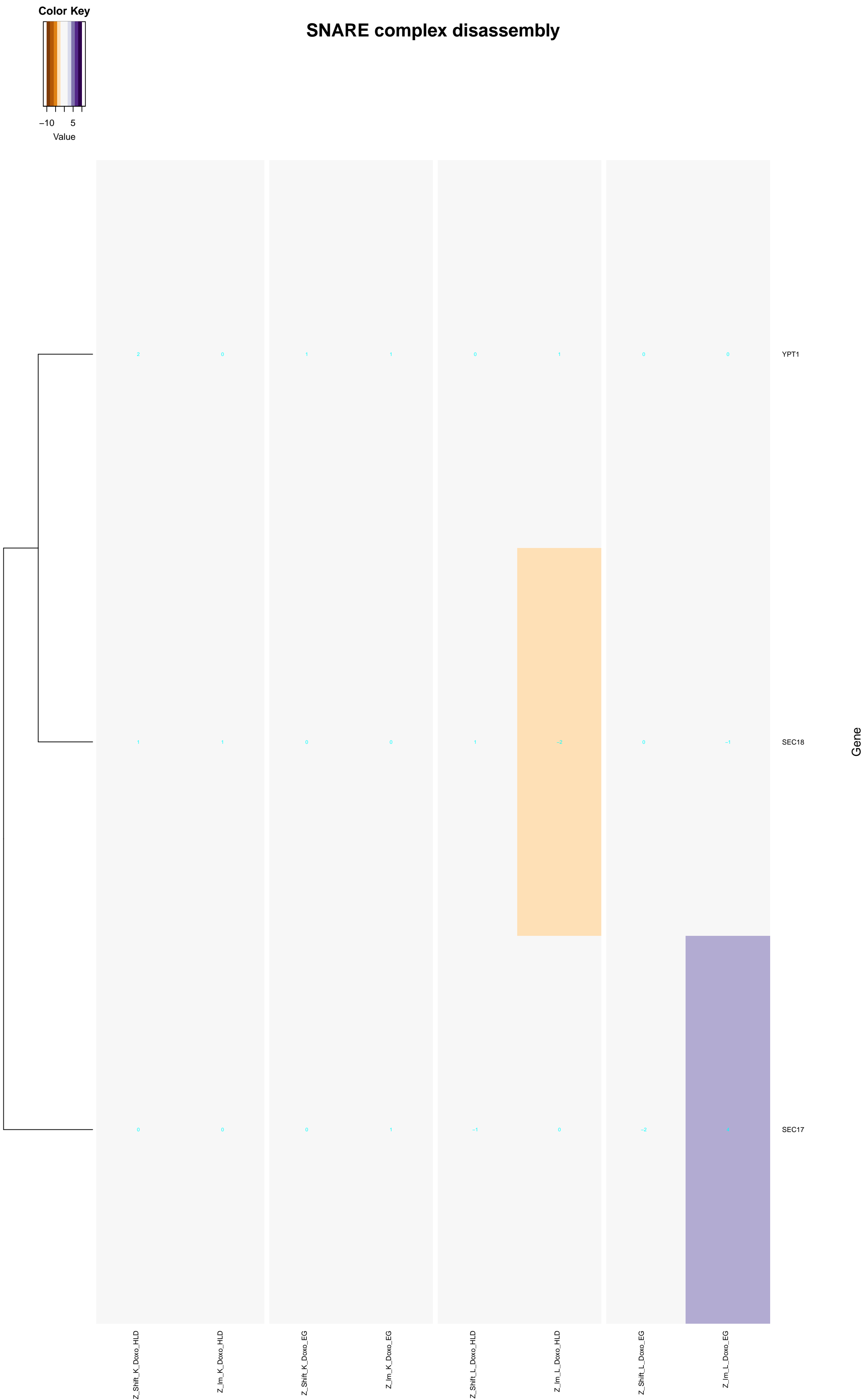

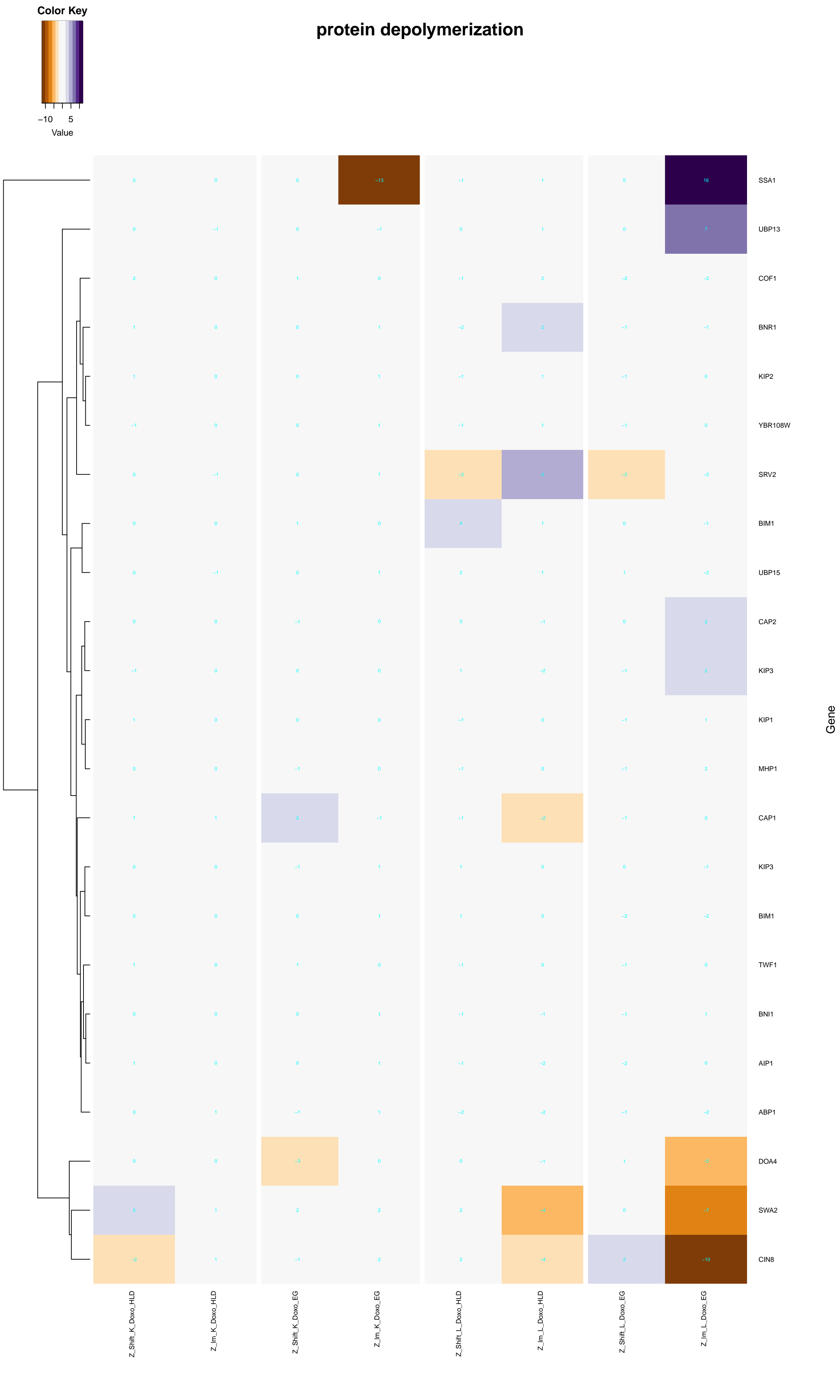

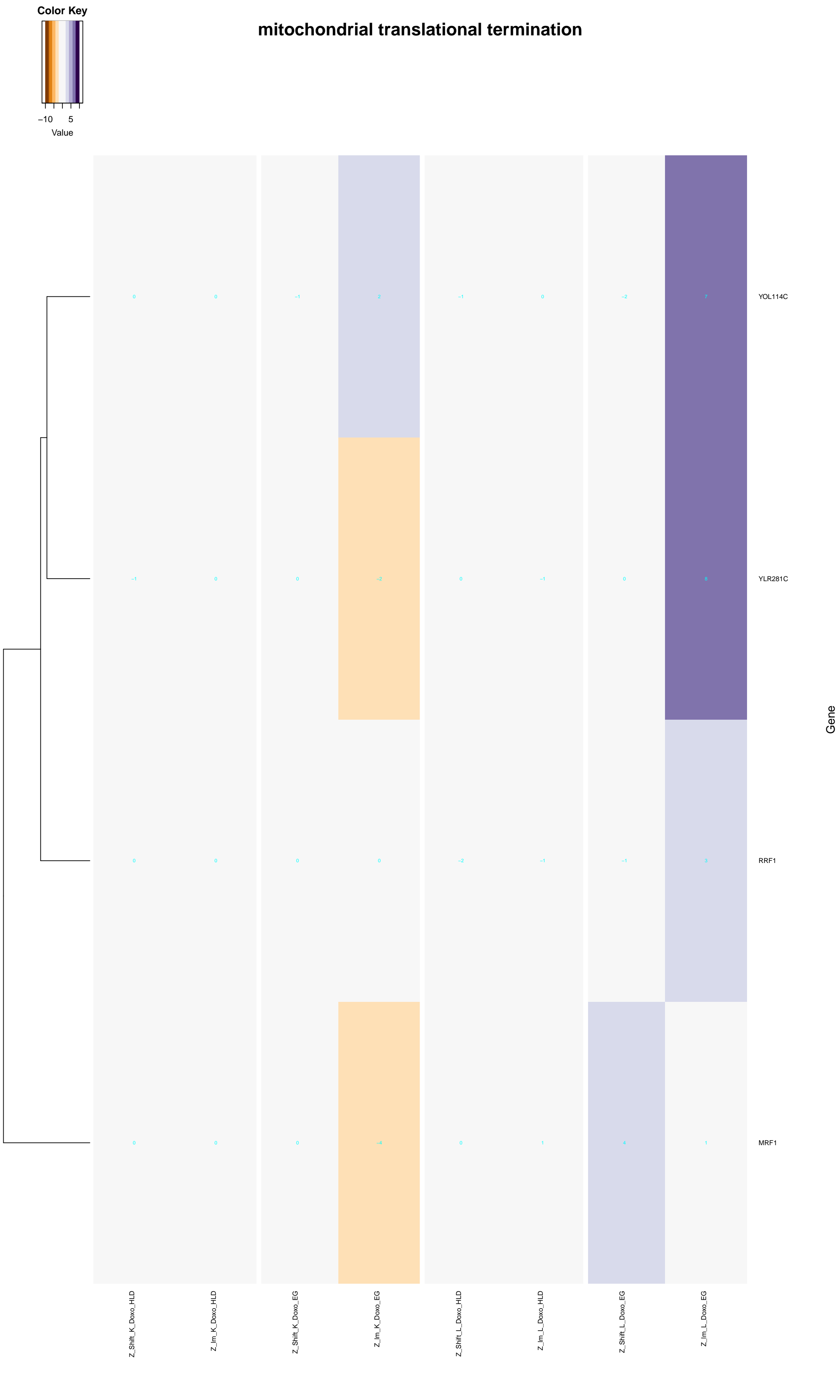

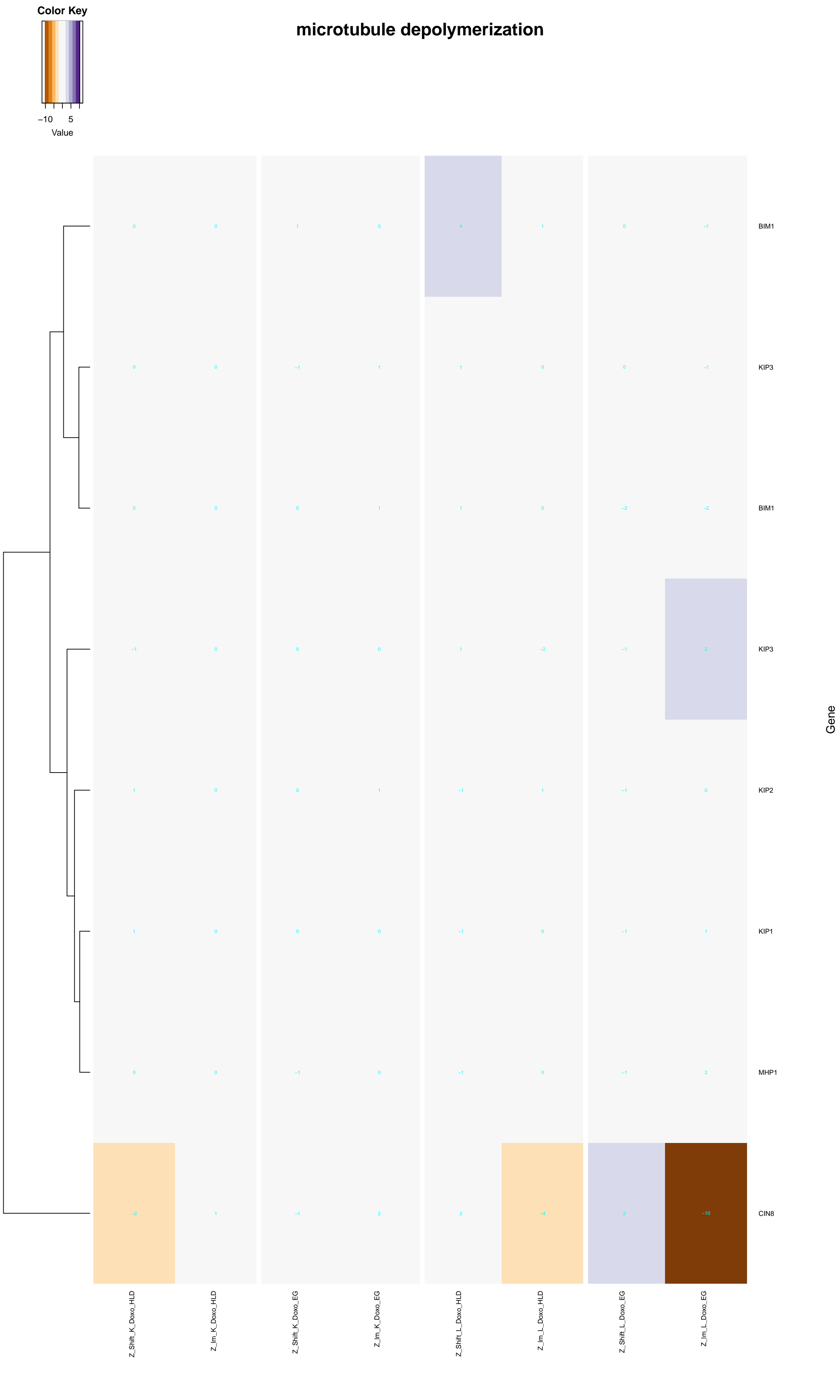

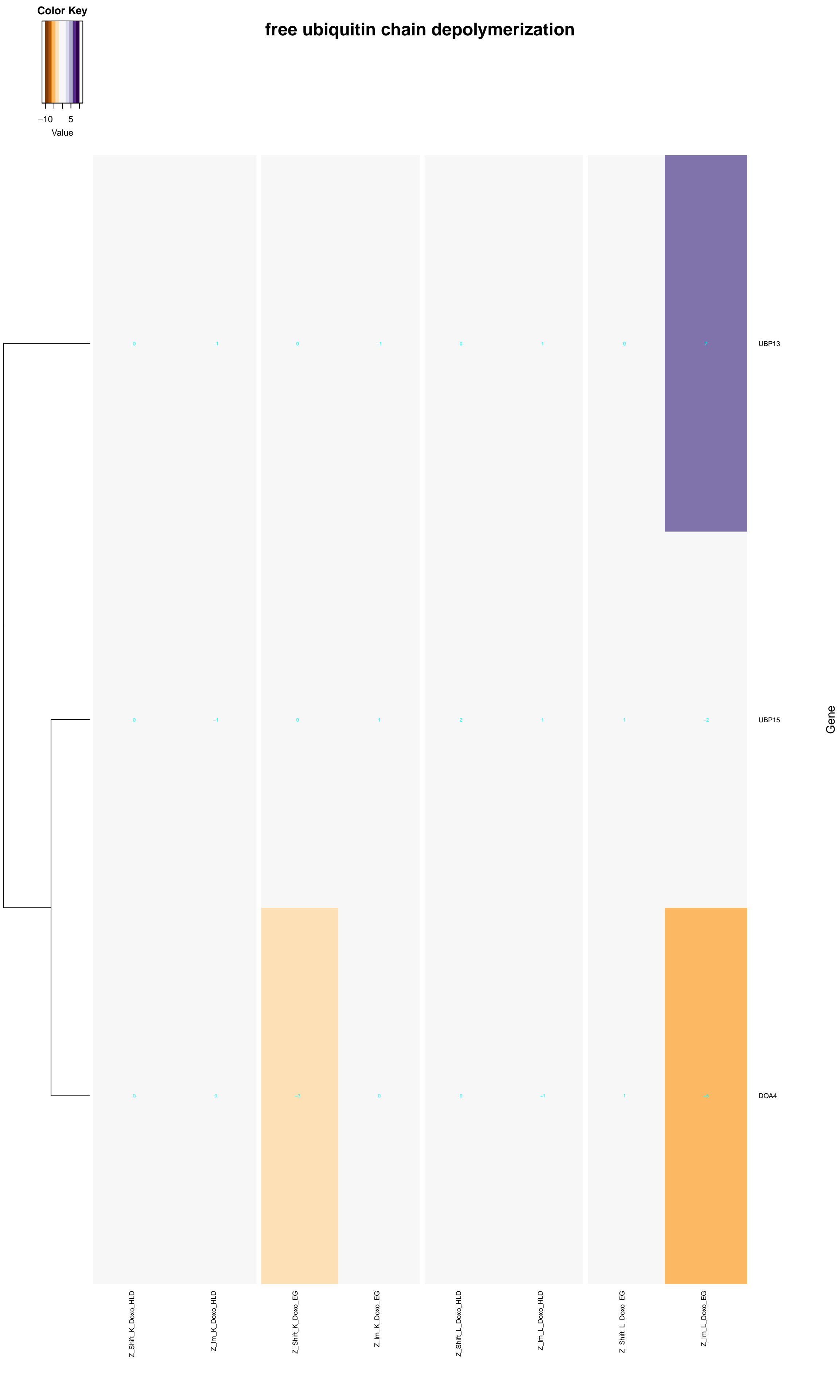

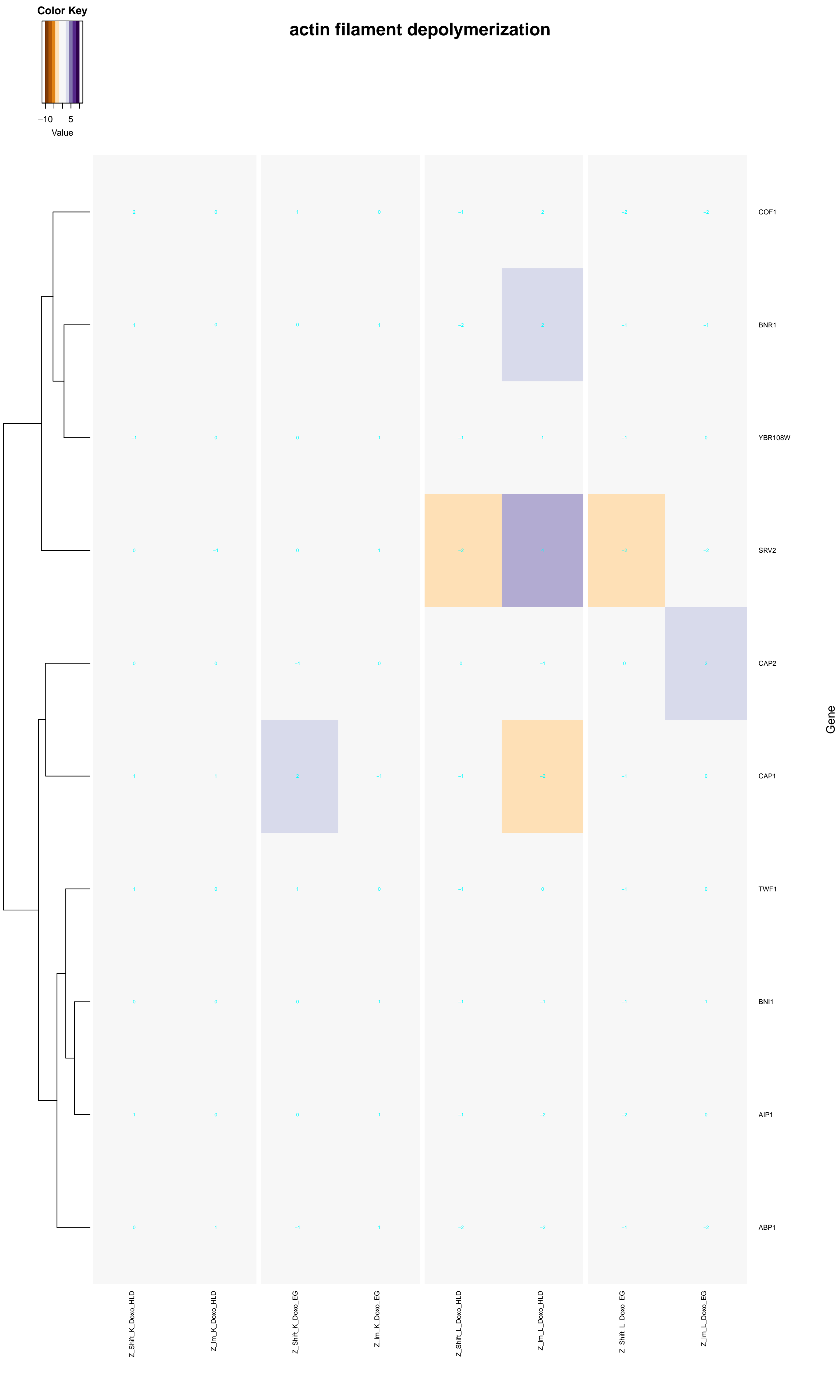

Supplement: Supplementary file 9 — Additional file 9. GO term-specific heatmaps for REMc/GTF-enriched clusters. GO term-specific heatmaps for significant GO process terms were generated as described in methods and Figs. 3 and 4. Any related child terms are presented in subsequent pages of the parent file name. GO terms with more than 100 children, with 2 or fewer genes annotated to the term, or a file size over 300KB are not shown. All heatmaps are generated with the same layout (see Figs. 3 and 4). [file 40170_2019_201_MOESM9_ESM.bz2 › Additional_File9_GOTermHeatmaps/Additional_File9_GOTermHeatmap/macromolecular_complex_disassembly.pdf]

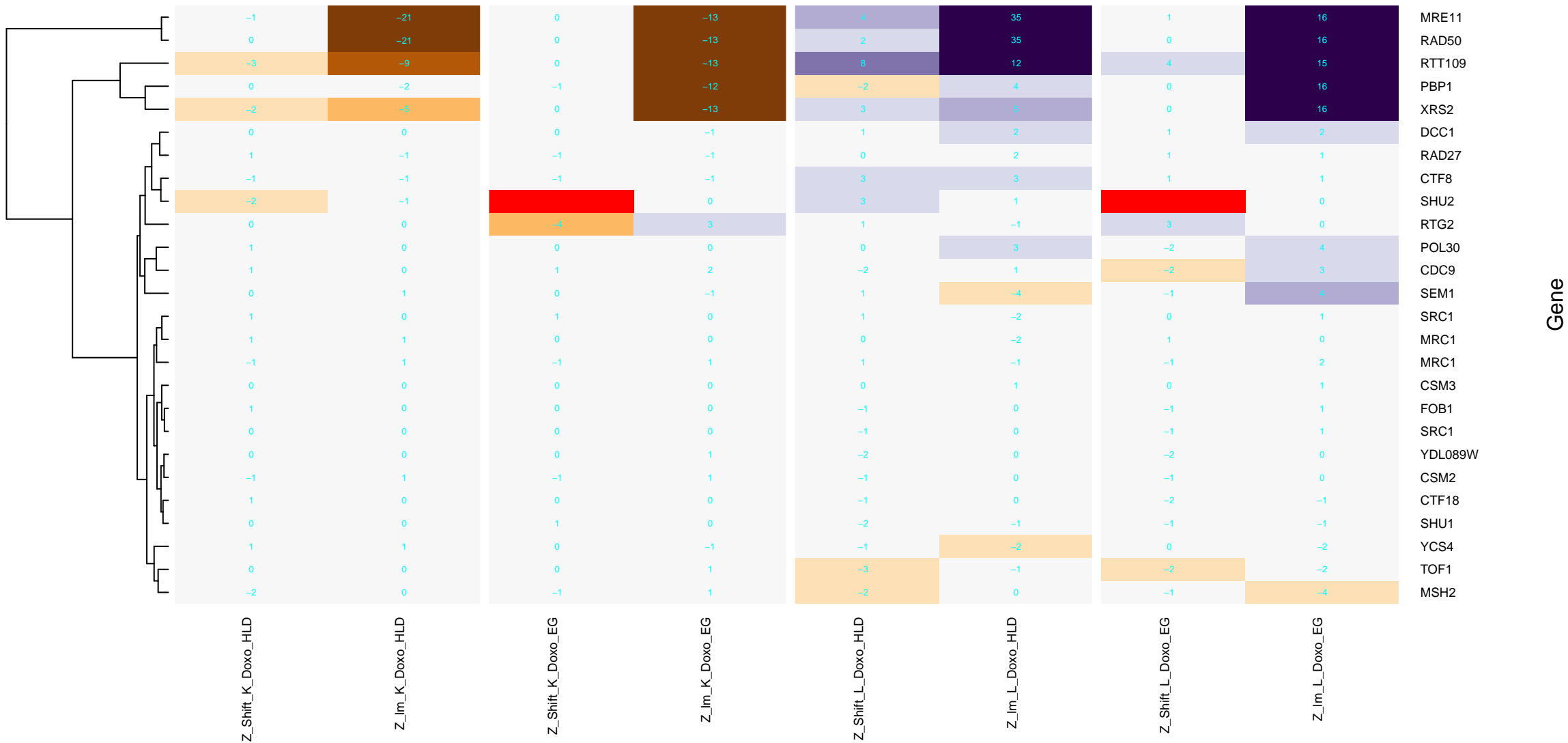

Supplement: Supplementary file 9 — Additional file 9. GO term-specific heatmaps for REMc/GTF-enriched clusters. GO term-specific heatmaps for significant GO process terms were generated as described in methods and Figs. 3 and 4. Any related child terms are presented in subsequent pages of the parent file name. GO terms with more than 100 children, with 2 or fewer genes annotated to the term, or a file size over 300KB are not shown. All heatmaps are generated with the same layout (see Figs. 3 and 4). [file 40170_2019_201_MOESM9_ESM.bz2 › Additional_File9_GOTermHeatmaps/Additional_File9_GOTermHeatmap/maintenance_of_DNA_repeat_elements.pdf]

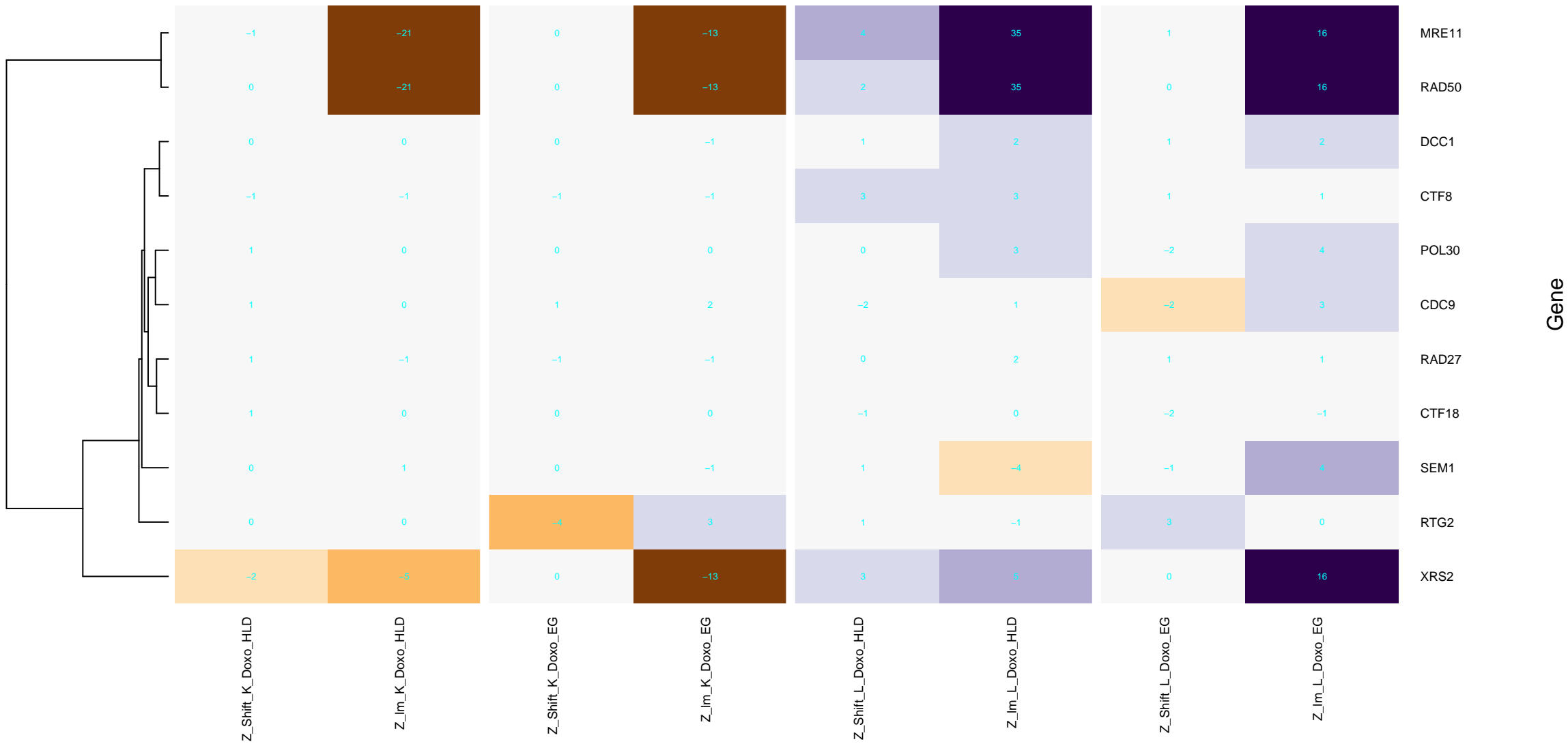

Supplement: Supplementary file 9 — Additional file 9. GO term-specific heatmaps for REMc/GTF-enriched clusters. GO term-specific heatmaps for significant GO process terms were generated as described in methods and Figs. 3 and 4. Any related child terms are presented in subsequent pages of the parent file name. GO terms with more than 100 children, with 2 or fewer genes annotated to the term, or a file size over 300KB are not shown. All heatmaps are generated with the same layout (see Figs. 3 and 4). [file 40170_2019_201_MOESM9_ESM.bz2 › Additional_File9_GOTermHeatmaps/Additional_File9_GOTermHeatmap/maintenance_of_DNA_trinucleotide_repeats.pdf]

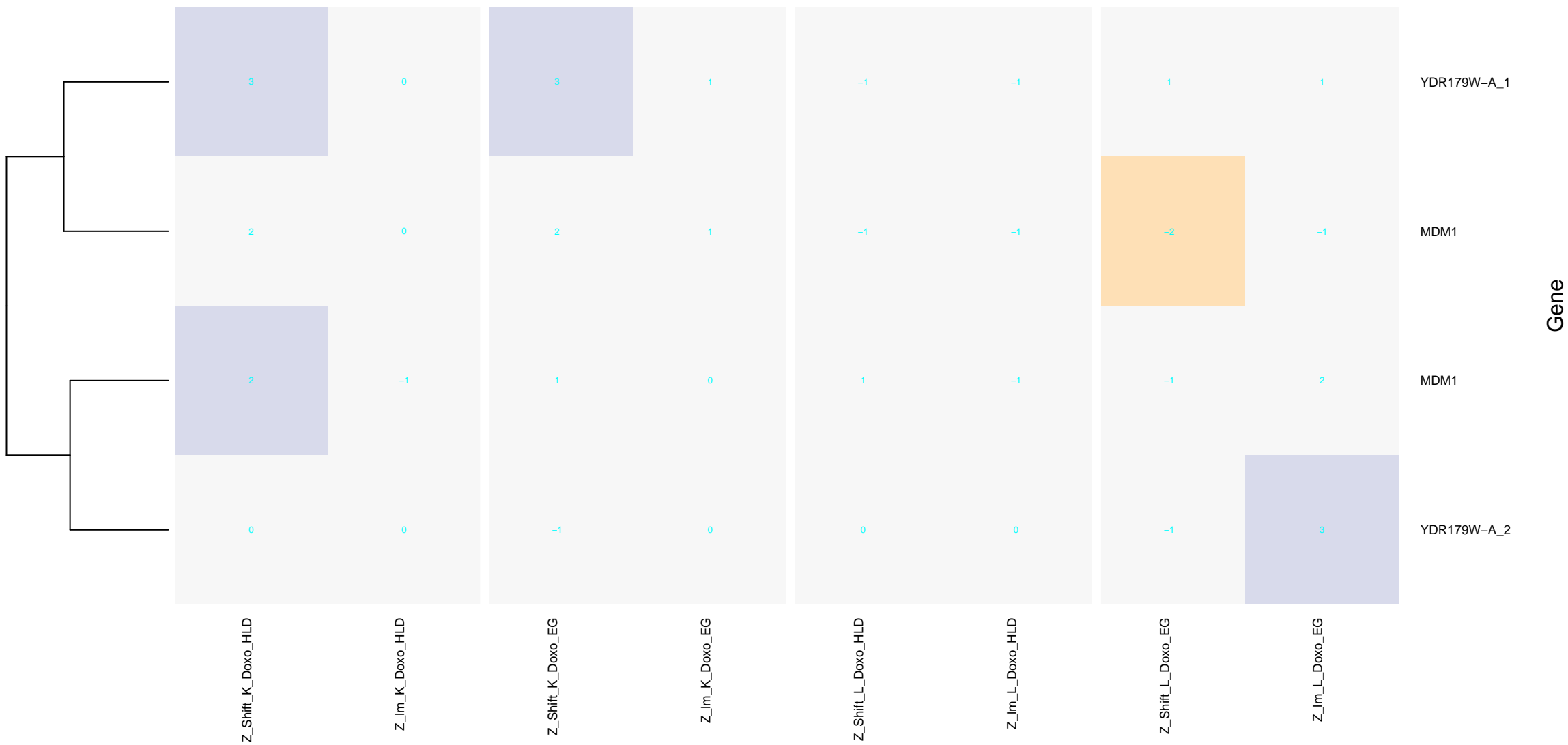

Supplement: Supplementary file 9 — Additional file 9. GO term-specific heatmaps for REMc/GTF-enriched clusters. GO term-specific heatmaps for significant GO process terms were generated as described in methods and Figs. 3 and 4. Any related child terms are presented in subsequent pages of the parent file name. GO terms with more than 100 children, with 2 or fewer genes annotated to the term, or a file size over 300KB are not shown. All heatmaps are generated with the same layout (see Figs. 3 and 4). [file 40170_2019_201_MOESM9_ESM.bz2 › Additional_File9_GOTermHeatmaps/Additional_File9_GOTermHeatmap/maintenance_of_organelle_location.pdf]

-10      5  
Value

## maturation of 5.8S rRNA

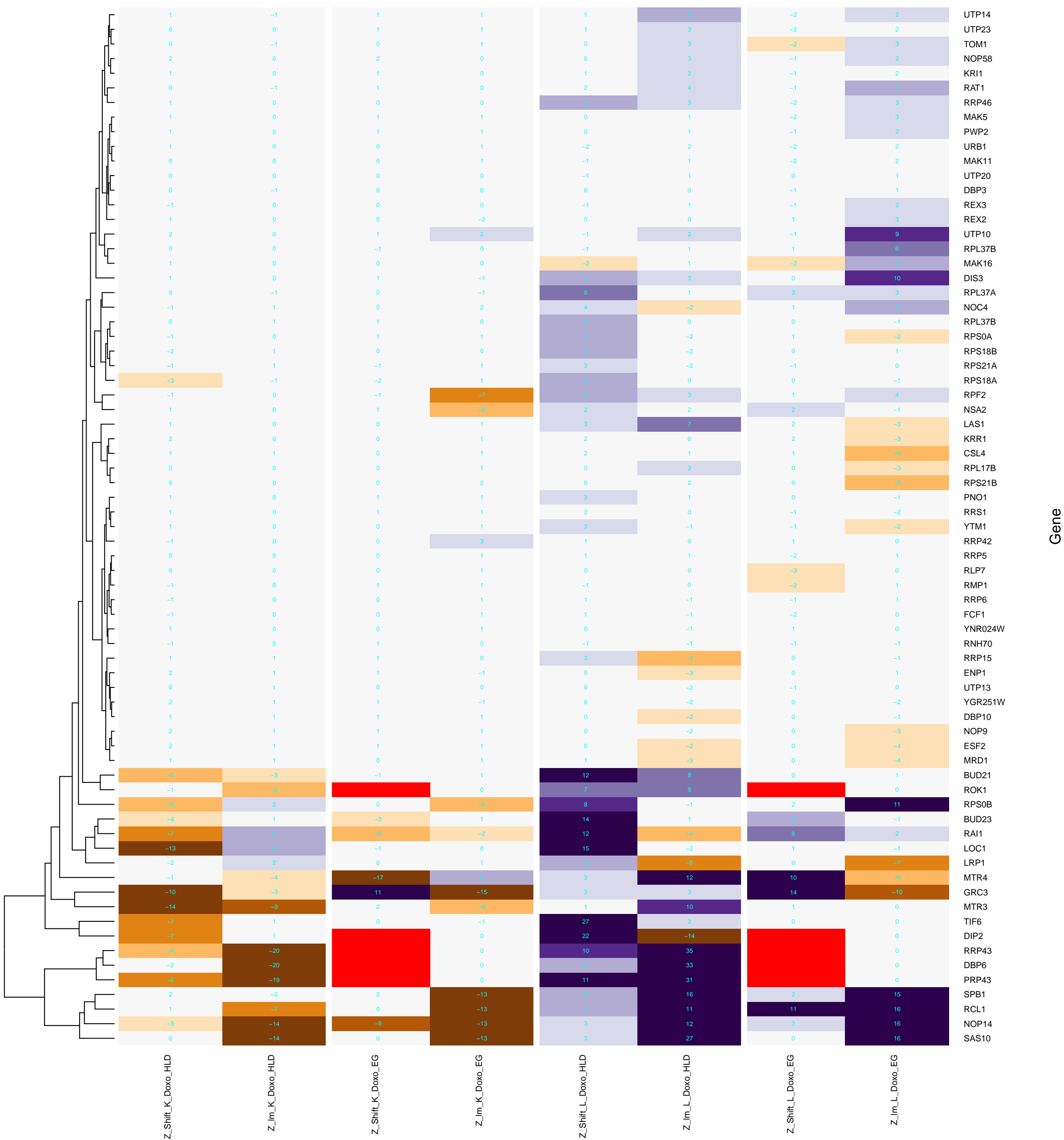

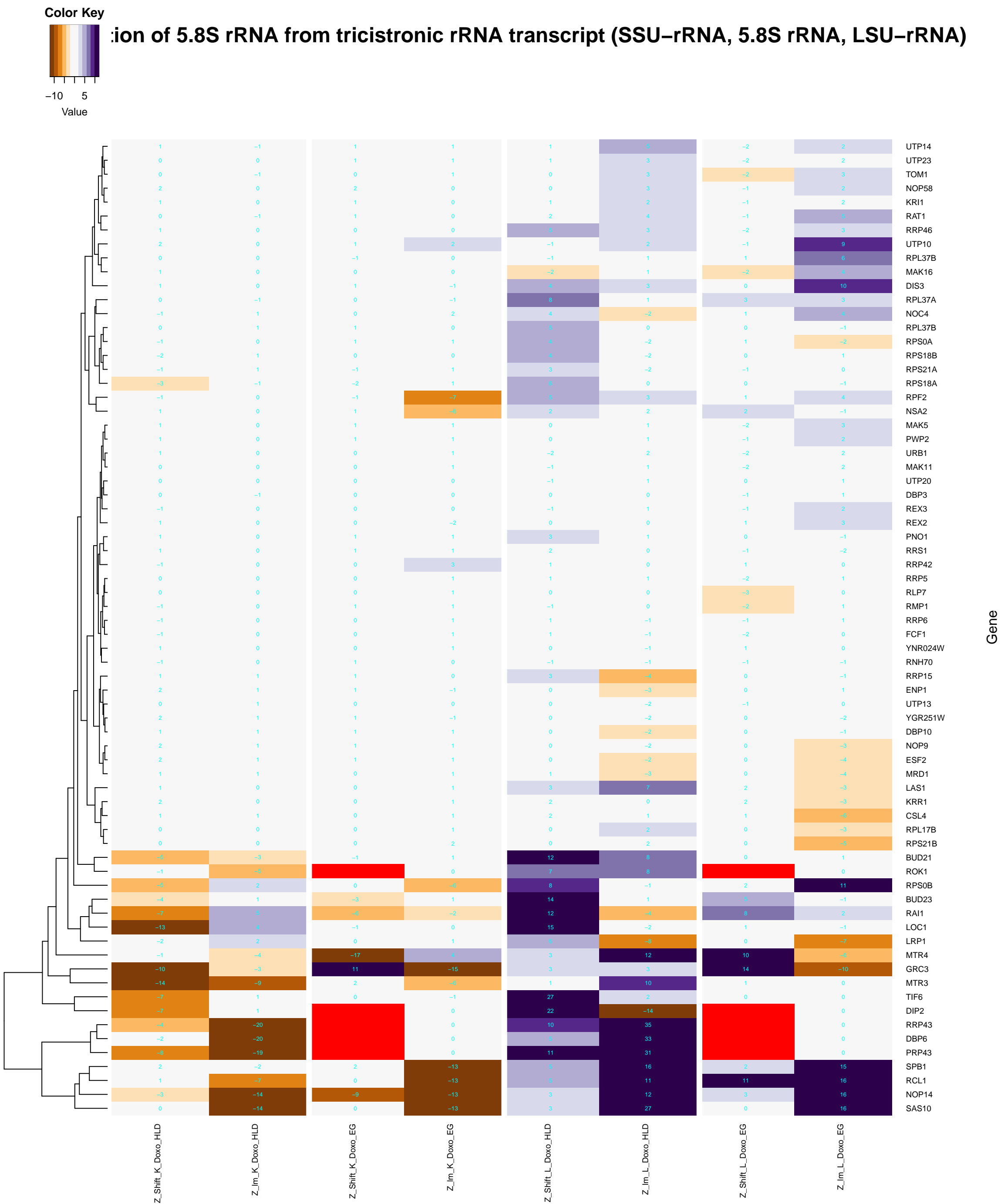

Supplement: Supplementary file 9 — Additional file 9. GO term-specific heatmaps for REMc/GTF-enriched clusters. GO term-specific heatmaps for significant GO process terms were generated as described in methods and Figs. 3 and 4. Any related child terms are presented in subsequent pages of the parent file name. GO terms with more than 100 children, with 2 or fewer genes annotated to the term, or a file size over 300KB are not shown. All heatmaps are generated with the same layout (see Figs. 3 and 4). [file 40170_2019_201_MOESM9_ESM.bz2 › Additional_File9_GOTermHeatmaps/Additional_File9_GOTermHeatmap/maturation_of_5.8S_rRNA.pdf]

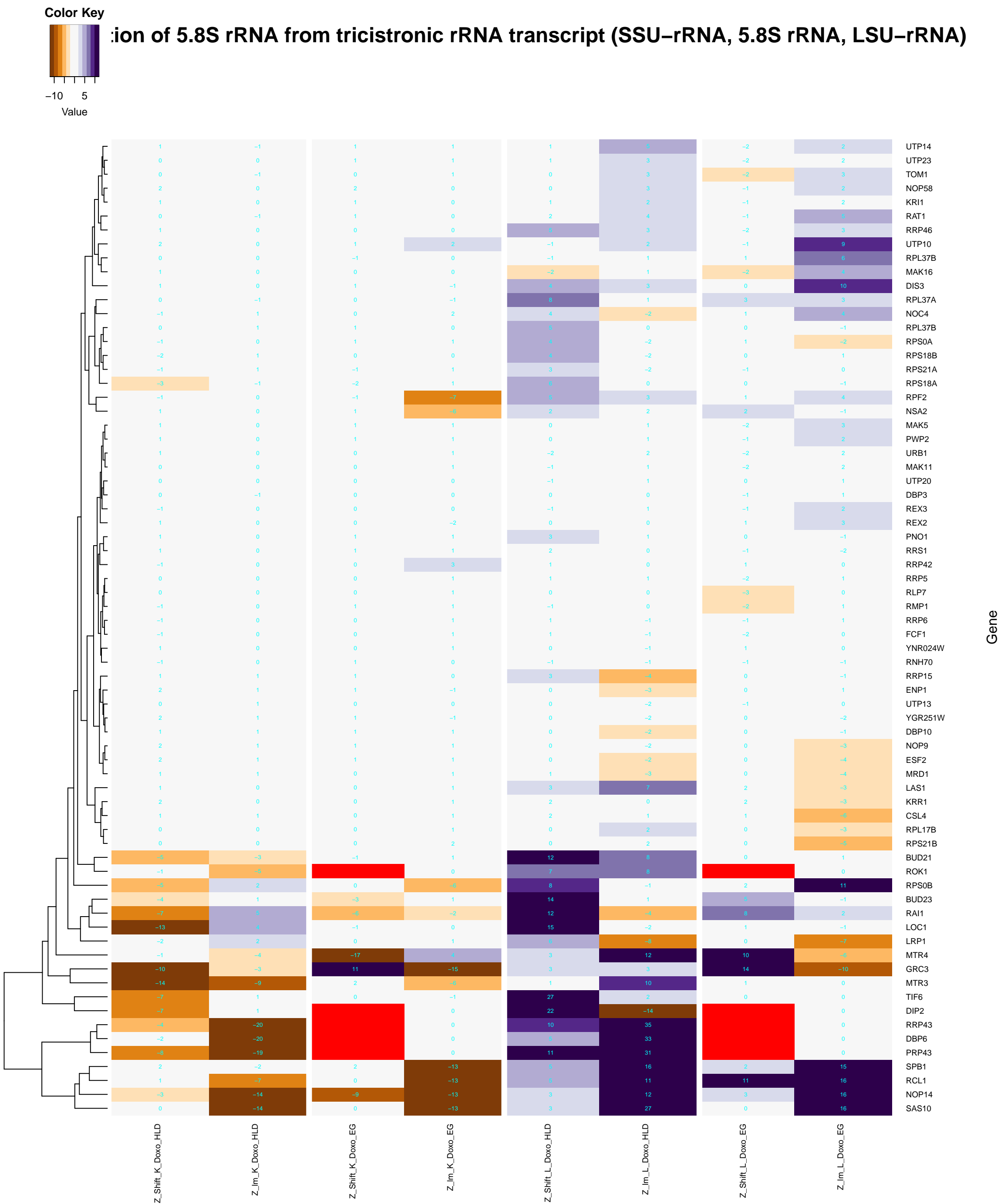

Supplement: Supplementary file 9 — Additional file 9. GO term-specific heatmaps for REMc/GTF-enriched clusters. GO term-specific heatmaps for significant GO process terms were generated as described in methods and Figs. 3 and 4. Any related child terms are presented in subsequent pages of the parent file name. GO terms with more than 100 children, with 2 or fewer genes annotated to the term, or a file size over 300KB are not shown. All heatmaps are generated with the same layout (see Figs. 3 and 4). [file 40170_2019_201_MOESM9_ESM.bz2 › Additional_File9_GOTermHeatmaps/Additional_File9_GOTermHeatmap/maturation_of_5.8S_rRNA_from_tricistronic_rRNA_transcript_(SSU-rRNA,_5.8S_rRNA,_LSU-rRNA).pdf]

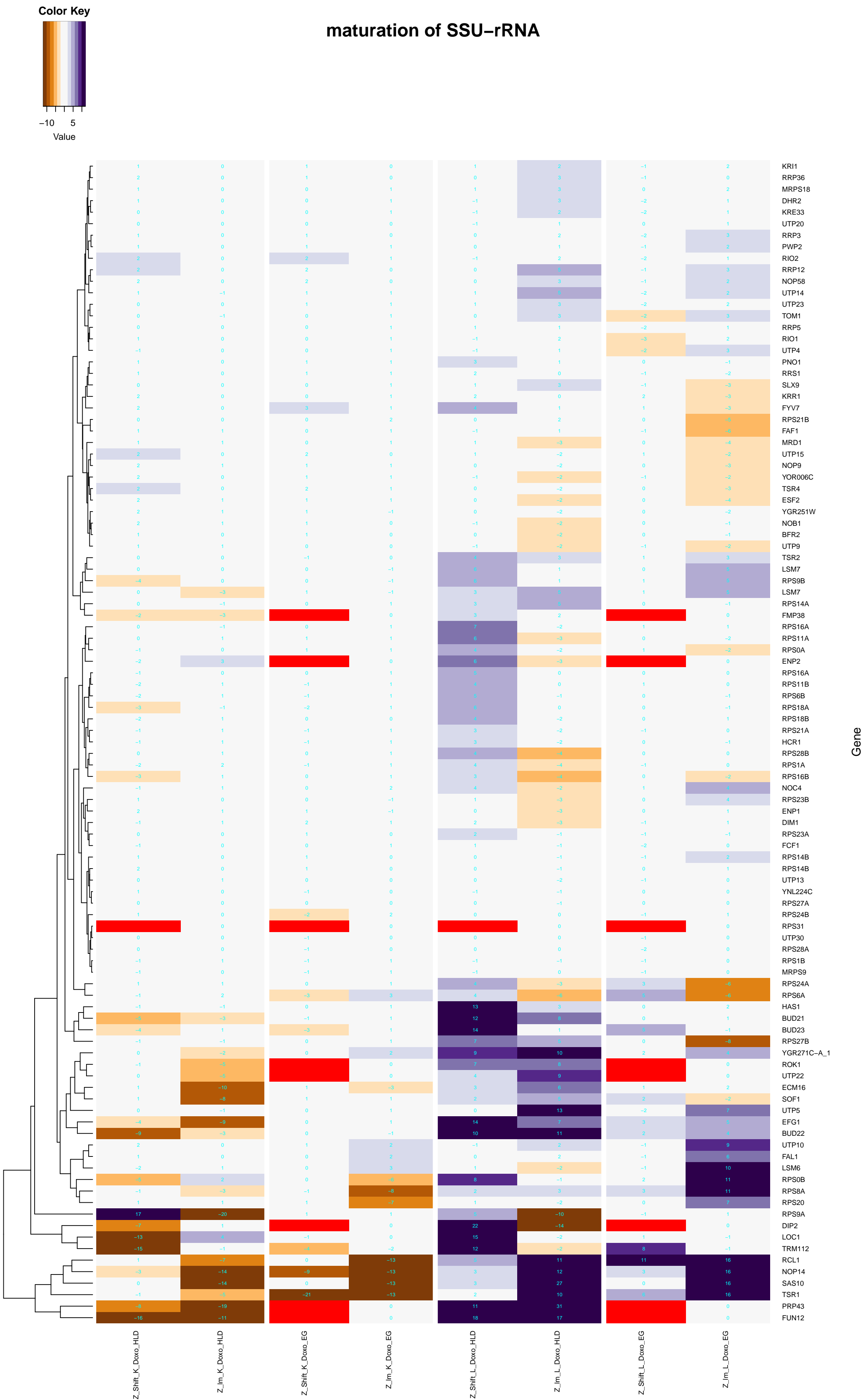

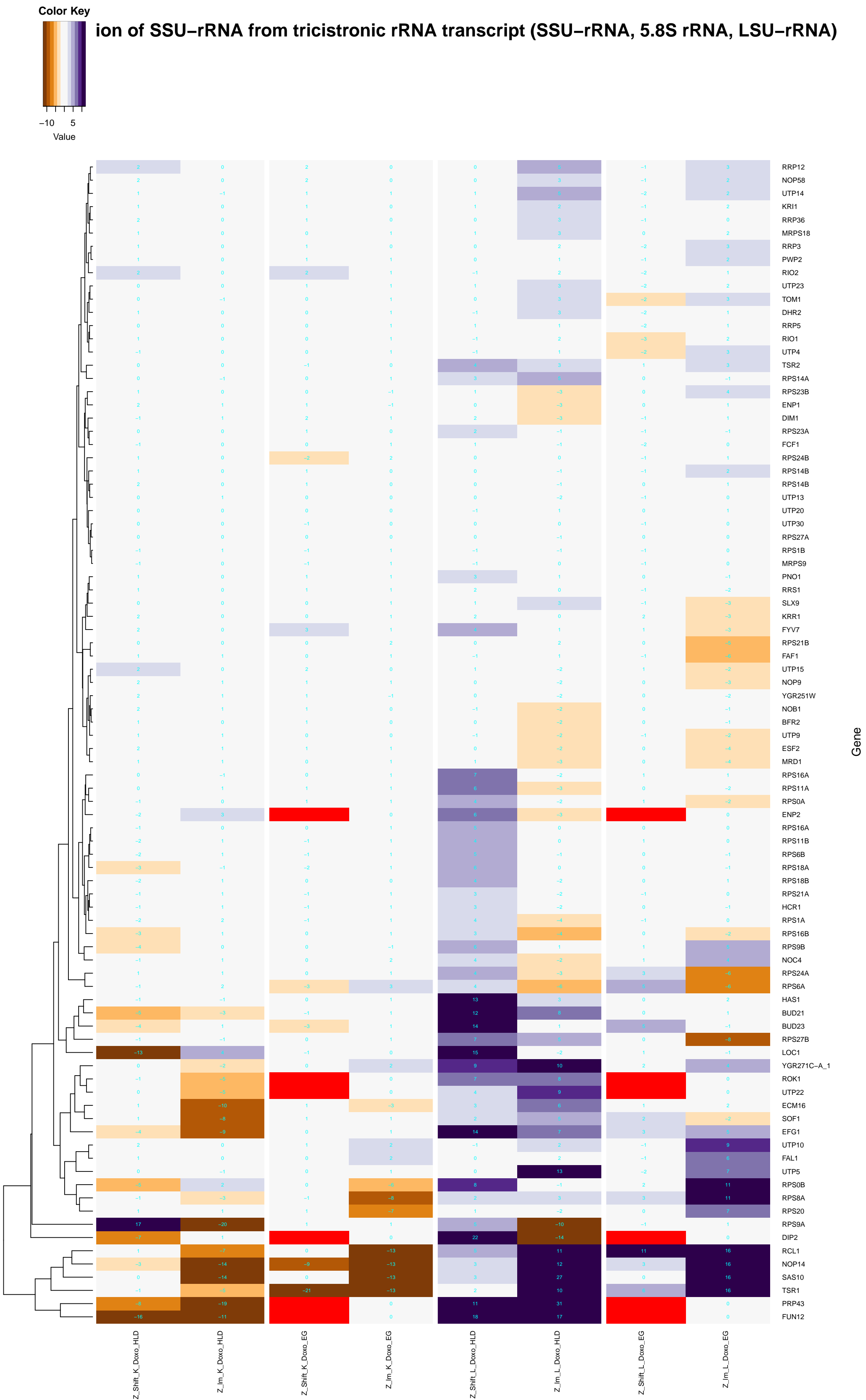

Supplement: Supplementary file 9 — Additional file 9. GO term-specific heatmaps for REMc/GTF-enriched clusters. GO term-specific heatmaps for significant GO process terms were generated as described in methods and Figs. 3 and 4. Any related child terms are presented in subsequent pages of the parent file name. GO terms with more than 100 children, with 2 or fewer genes annotated to the term, or a file size over 300KB are not shown. All heatmaps are generated with the same layout (see Figs. 3 and 4). [file 40170_2019_201_MOESM9_ESM.bz2 › Additional_File9_GOTermHeatmaps/Additional_File9_GOTermHeatmap/maturation_of_SSU-rRNA.pdf]

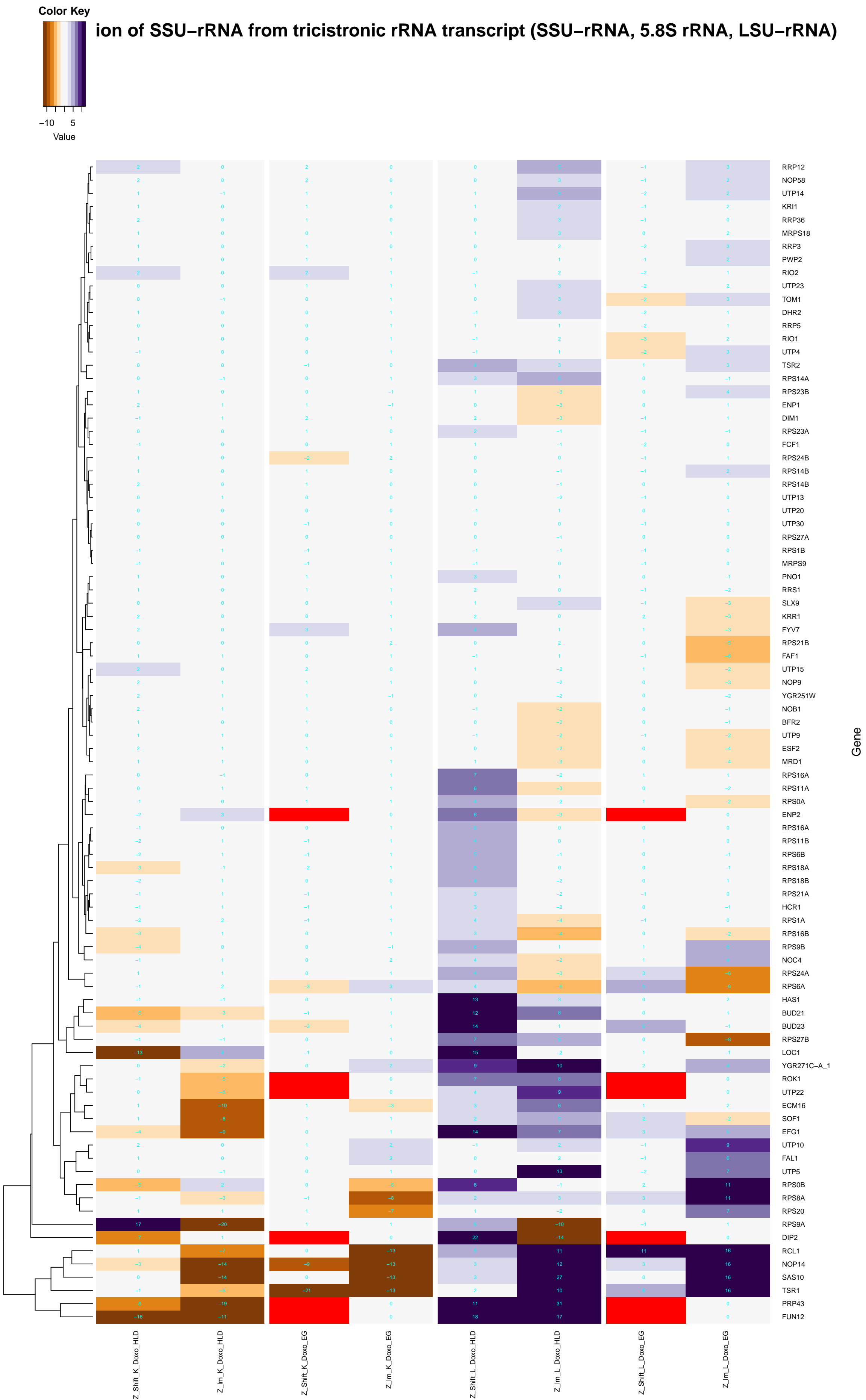

Supplement: Supplementary file 9 — Additional file 9. GO term-specific heatmaps for REMc/GTF-enriched clusters. GO term-specific heatmaps for significant GO process terms were generated as described in methods and Figs. 3 and 4. Any related child terms are presented in subsequent pages of the parent file name. GO terms with more than 100 children, with 2 or fewer genes annotated to the term, or a file size over 300KB are not shown. All heatmaps are generated with the same layout (see Figs. 3 and 4). [file 40170_2019_201_MOESM9_ESM.bz2 › Additional_File9_GOTermHeatmaps/Additional_File9_GOTermHeatmap/maturation_of_SSU-rRNA_from_tricistronic_rRNA_transcript_(SSU-rRNA,_5.8S_rRNA,_LSU-rRNA).pdf]

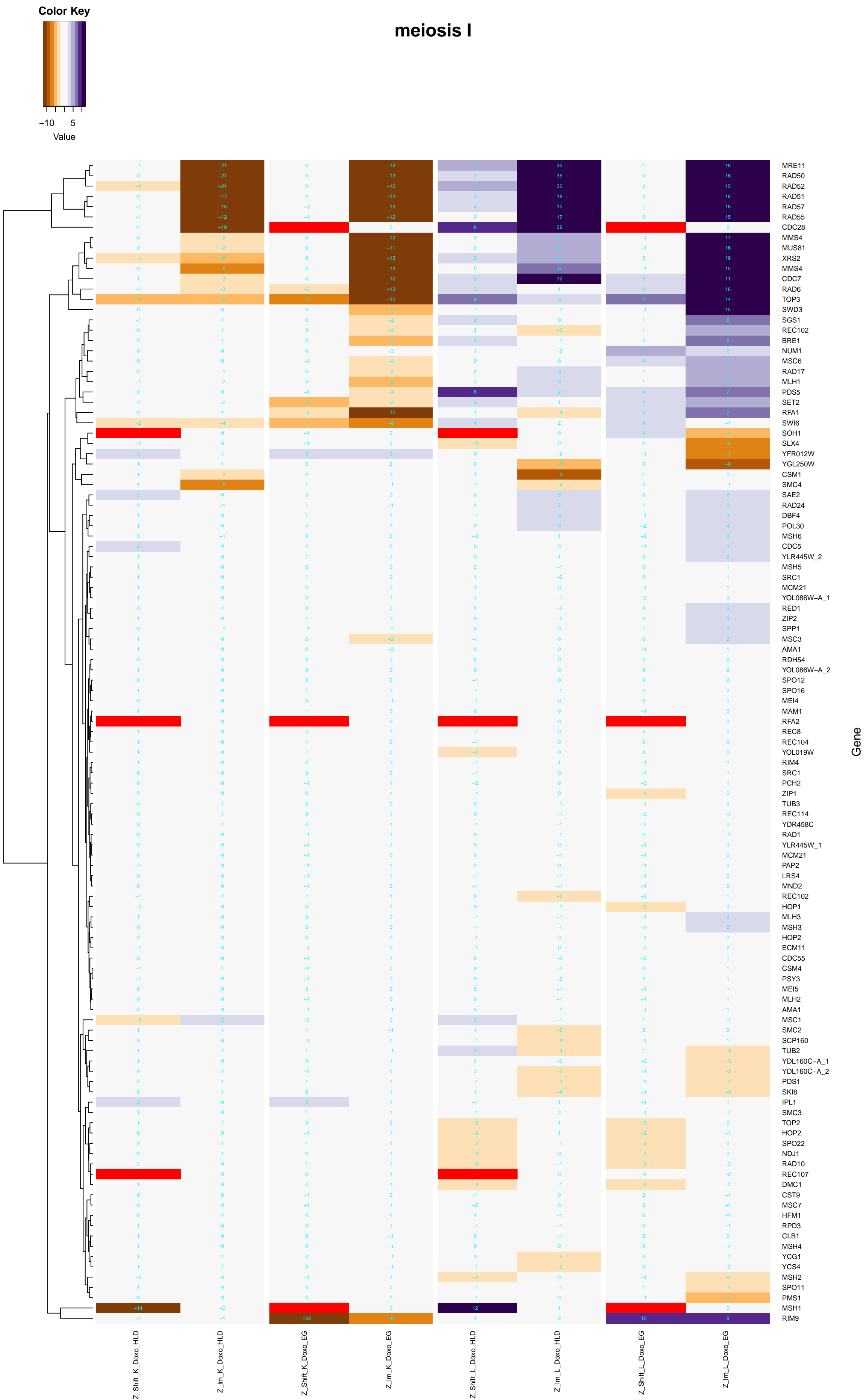

Supplement: Supplementary file 9 — Additional file 9. GO term-specific heatmaps for REMc/GTF-enriched clusters. GO term-specific heatmaps for significant GO process terms were generated as described in methods and Figs. 3 and 4. Any related child terms are presented in subsequent pages of the parent file name. GO terms with more than 100 children, with 2 or fewer genes annotated to the term, or a file size over 300KB are not shown. All heatmaps are generated with the same layout (see Figs. 3 and 4). [file 40170_2019_201_MOESM9_ESM.bz2 › Additional_File9_GOTermHeatmaps/Additional_File9_GOTermHeatmap/meiosis_I.pdf]

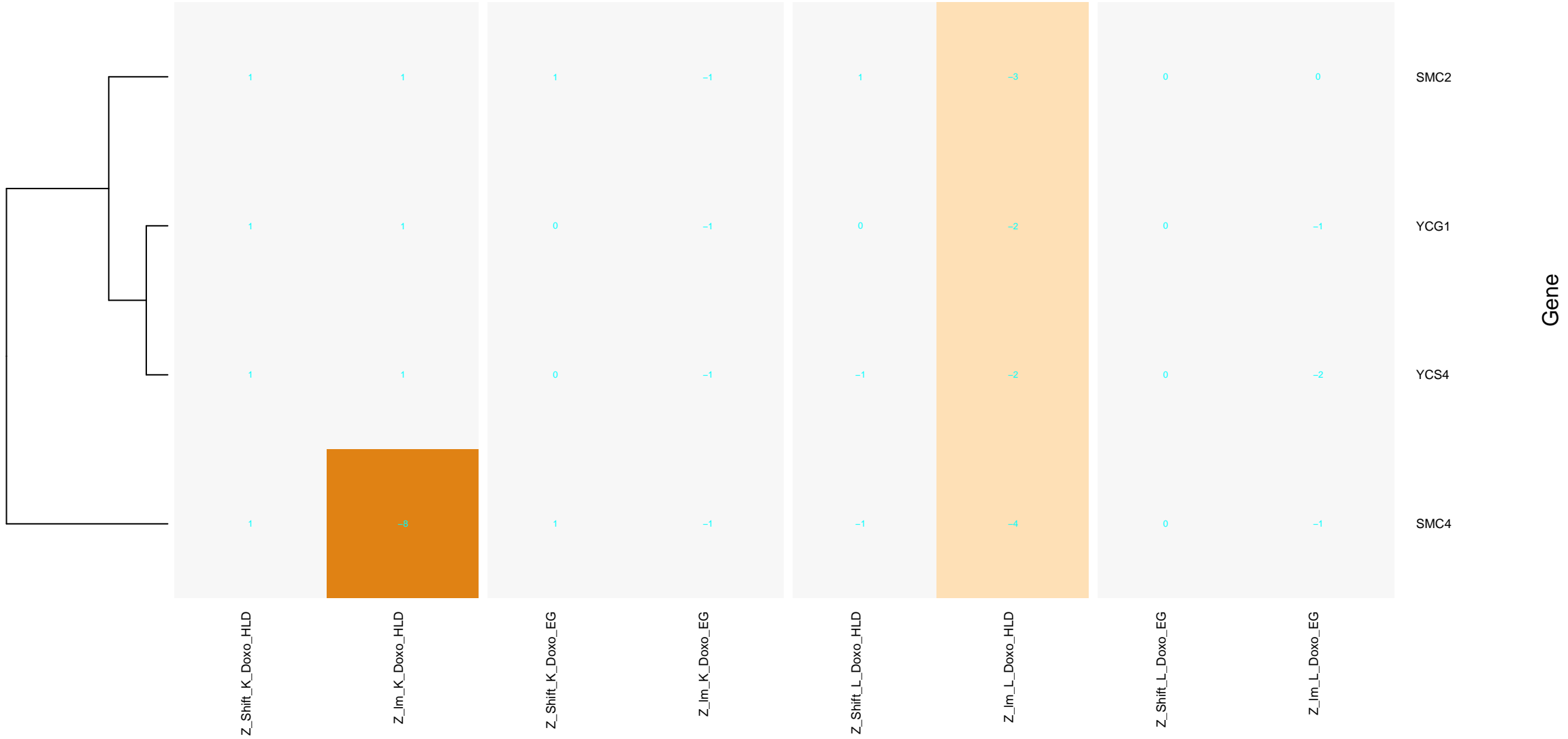

Supplement: Supplementary file 9 — Additional file 9. GO term-specific heatmaps for REMc/GTF-enriched clusters. GO term-specific heatmaps for significant GO process terms were generated as described in methods and Figs. 3 and 4. Any related child terms are presented in subsequent pages of the parent file name. GO terms with more than 100 children, with 2 or fewer genes annotated to the term, or a file size over 300KB are not shown. All heatmaps are generated with the same layout (see Figs. 3 and 4). [file 40170_2019_201_MOESM9_ESM.bz2 › Additional_File9_GOTermHeatmaps/Additional_File9_GOTermHeatmap/meiotic_chromosome_condensation.pdf]

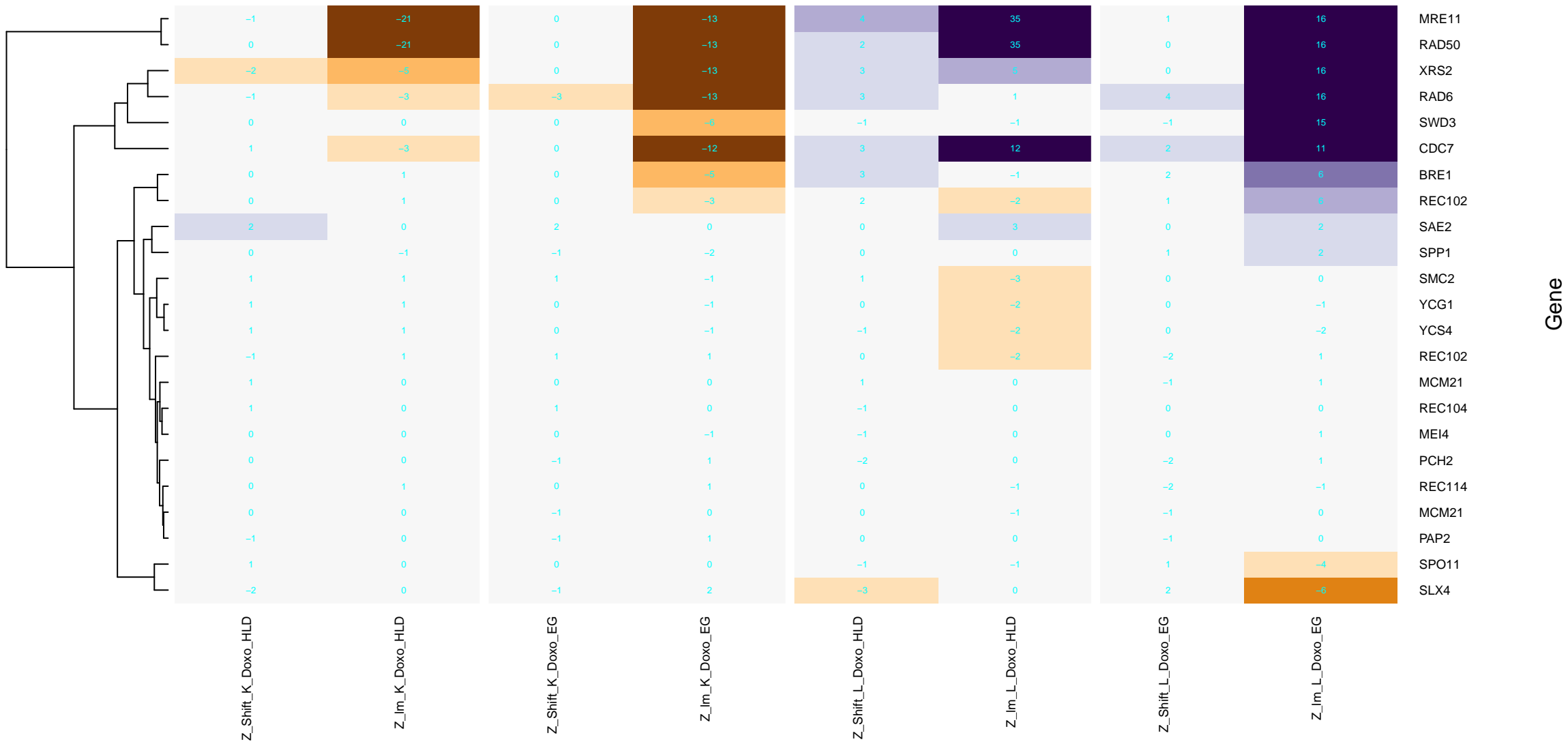

Supplement: Supplementary file 9 — Additional file 9. GO term-specific heatmaps for REMc/GTF-enriched clusters. GO term-specific heatmaps for significant GO process terms were generated as described in methods and Figs. 3 and 4. Any related child terms are presented in subsequent pages of the parent file name. GO terms with more than 100 children, with 2 or fewer genes annotated to the term, or a file size over 300KB are not shown. All heatmaps are generated with the same layout (see Figs. 3 and 4). [file 40170_2019_201_MOESM9_ESM.bz2 › Additional_File9_GOTermHeatmaps/Additional_File9_GOTermHeatmap/meiotic_DNA_double-strand_break_formation.pdf]

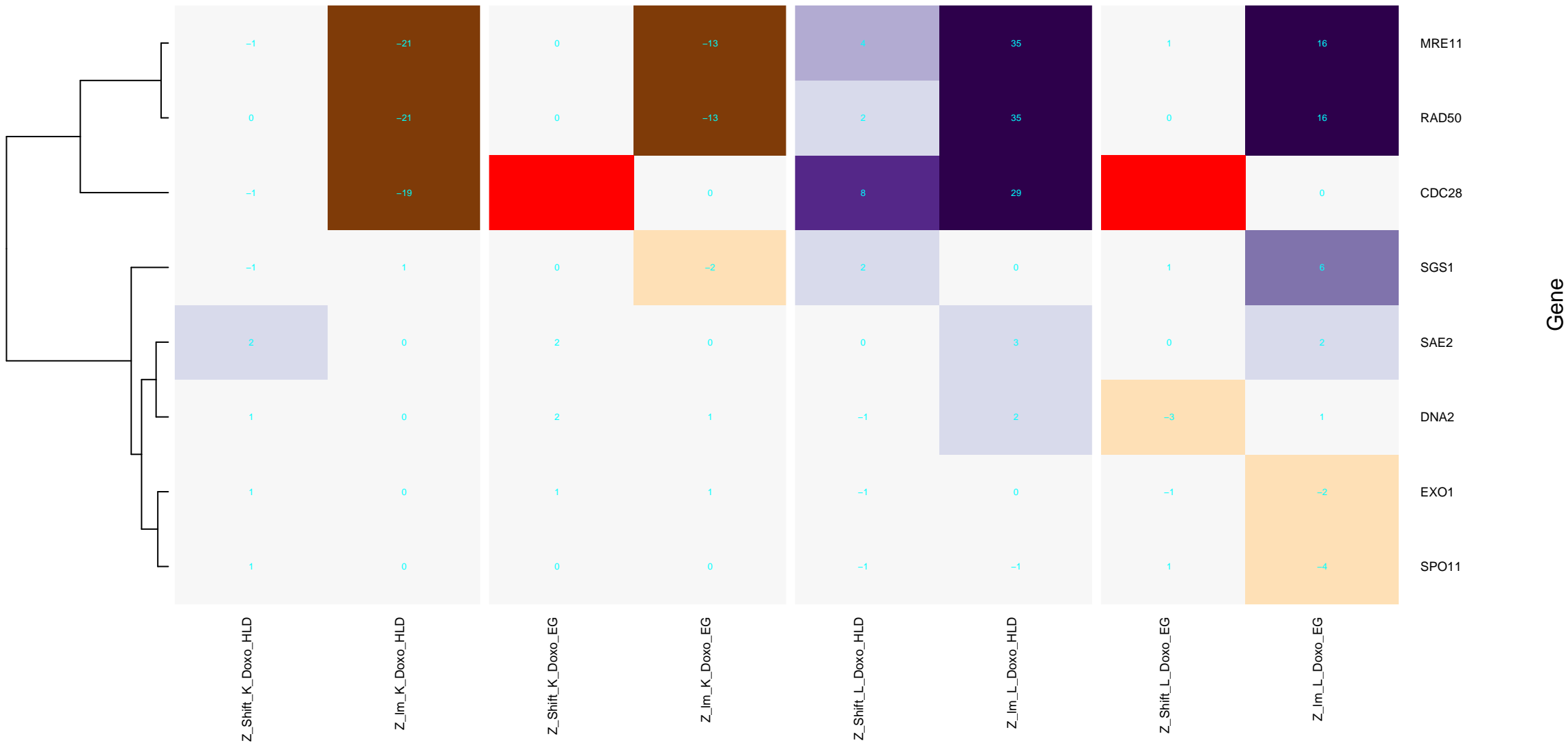

Supplement: Supplementary file 9 — Additional file 9. GO term-specific heatmaps for REMc/GTF-enriched clusters. GO term-specific heatmaps for significant GO process terms were generated as described in methods and Figs. 3 and 4. Any related child terms are presented in subsequent pages of the parent file name. GO terms with more than 100 children, with 2 or fewer genes annotated to the term, or a file size over 300KB are not shown. All heatmaps are generated with the same layout (see Figs. 3 and 4). [file 40170_2019_201_MOESM9_ESM.bz2 › Additional_File9_GOTermHeatmaps/Additional_File9_GOTermHeatmap/meiotic_DNA_double-strand_break_processing.pdf]

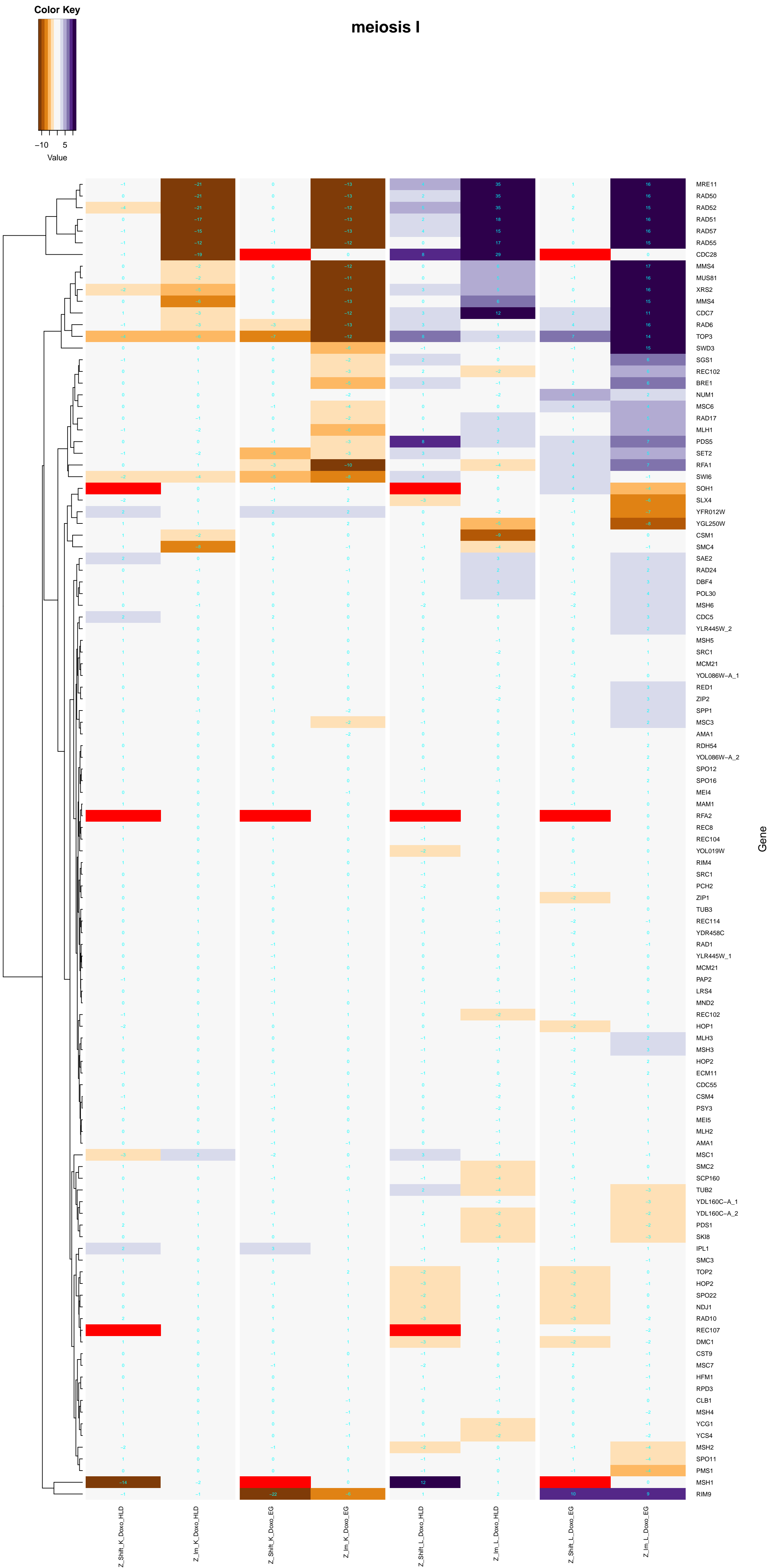

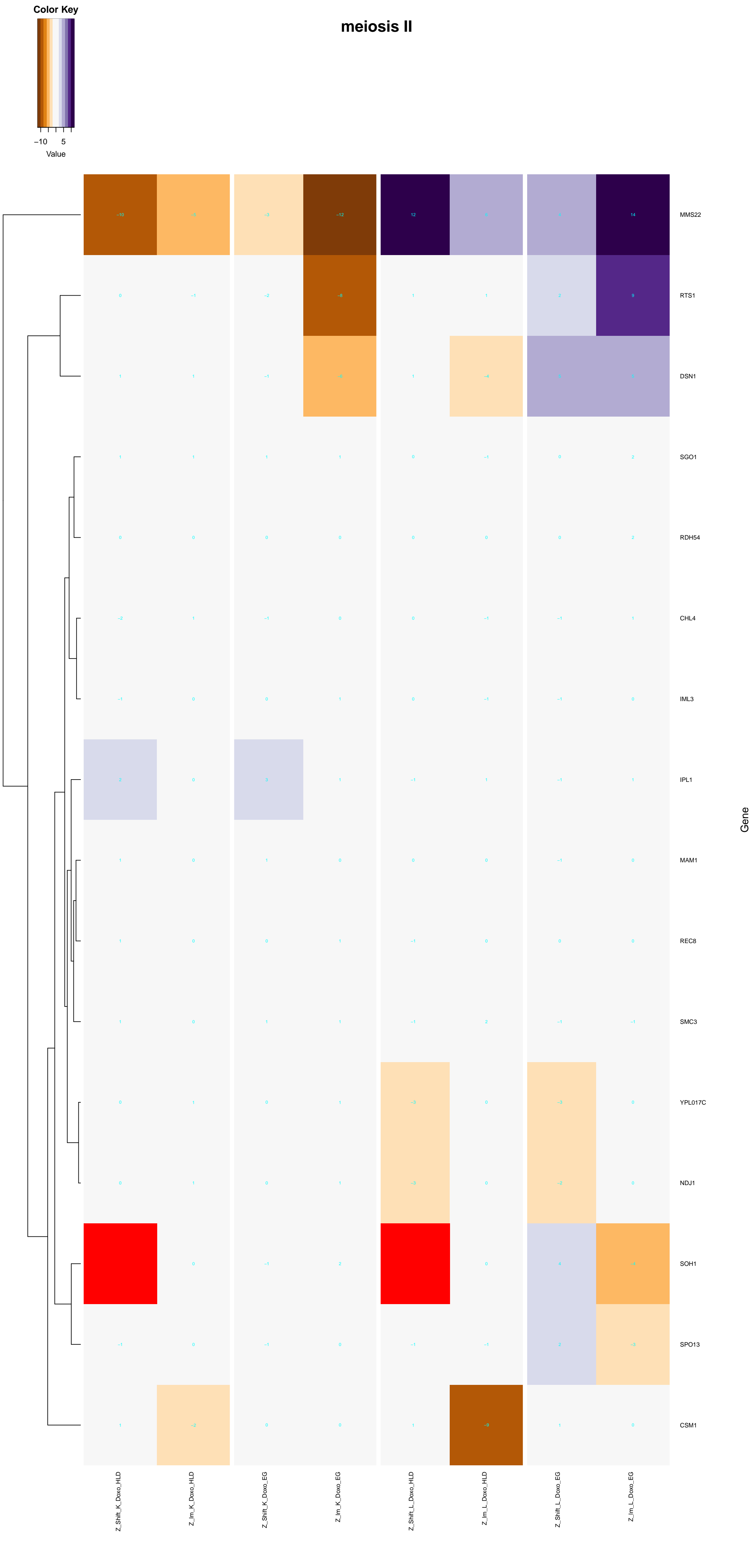

Supplement: Supplementary file 9 — Additional file 9. GO term-specific heatmaps for REMc/GTF-enriched clusters. GO term-specific heatmaps for significant GO process terms were generated as described in methods and Figs. 3 and 4. Any related child terms are presented in subsequent pages of the parent file name. GO terms with more than 100 children, with 2 or fewer genes annotated to the term, or a file size over 300KB are not shown. All heatmaps are generated with the same layout (see Figs. 3 and 4). [file 40170_2019_201_MOESM9_ESM.bz2 › Additional_File9_GOTermHeatmaps/Additional_File9_GOTermHeatmap/meiotic_nuclear_division.pdf]

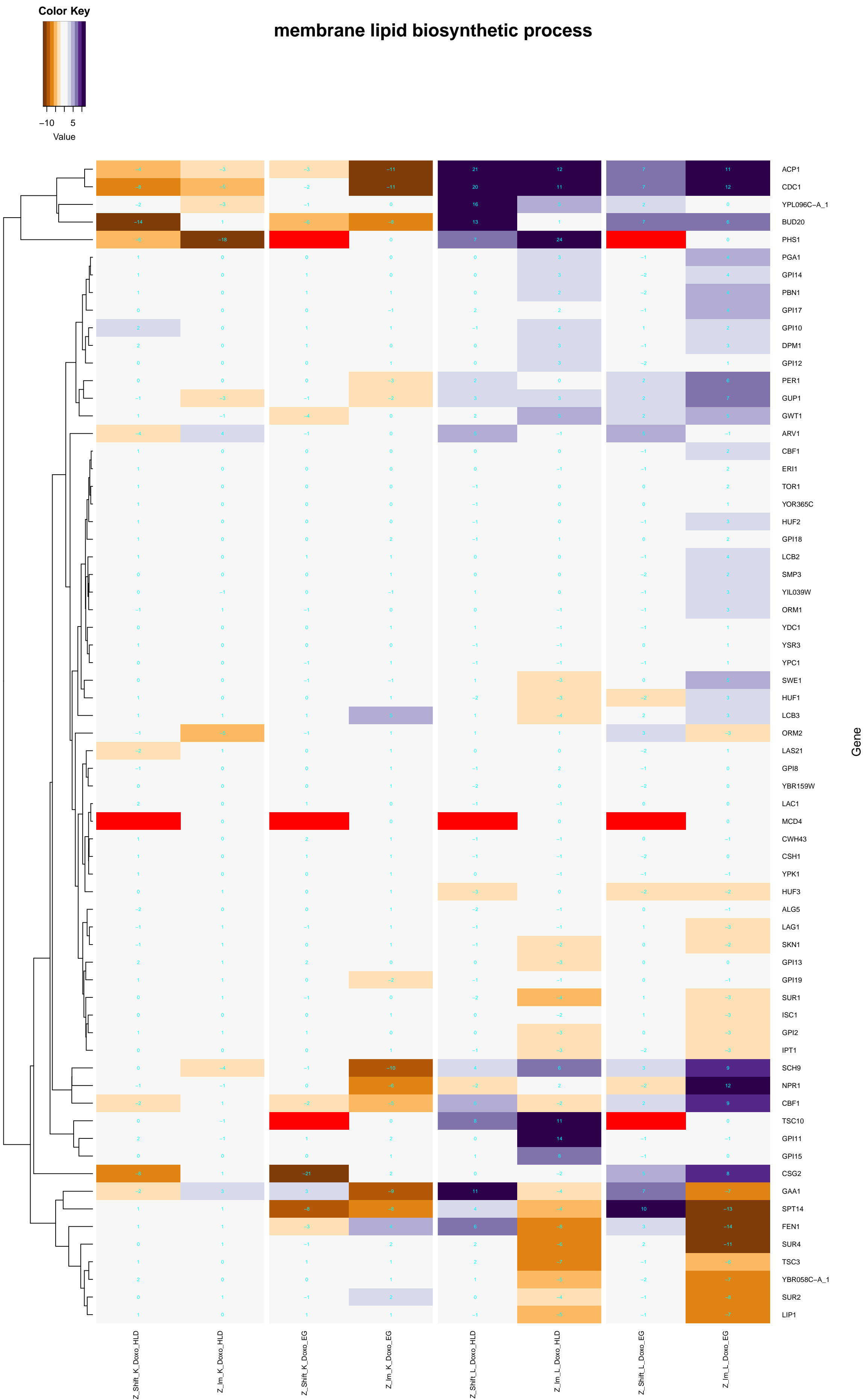

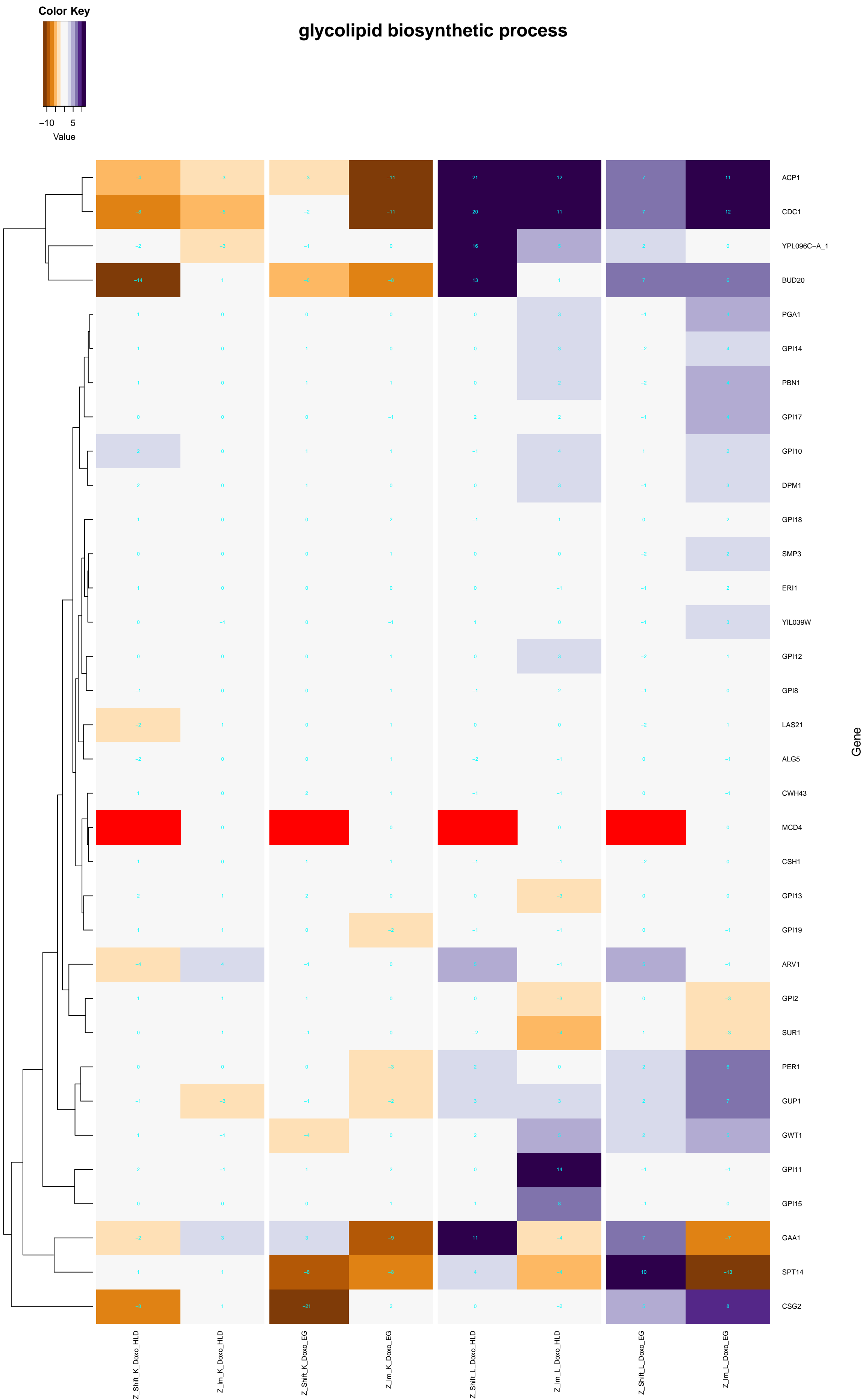

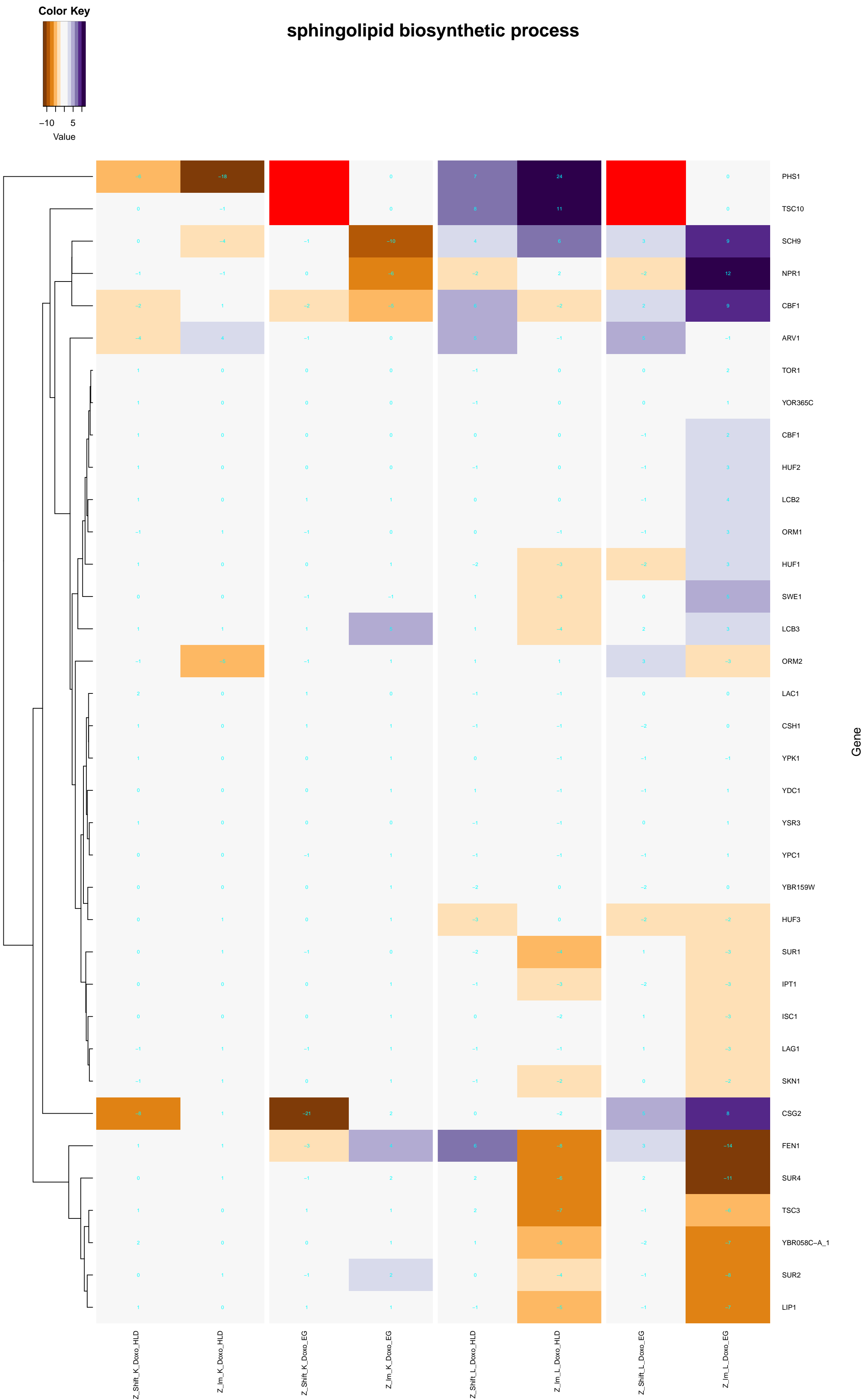

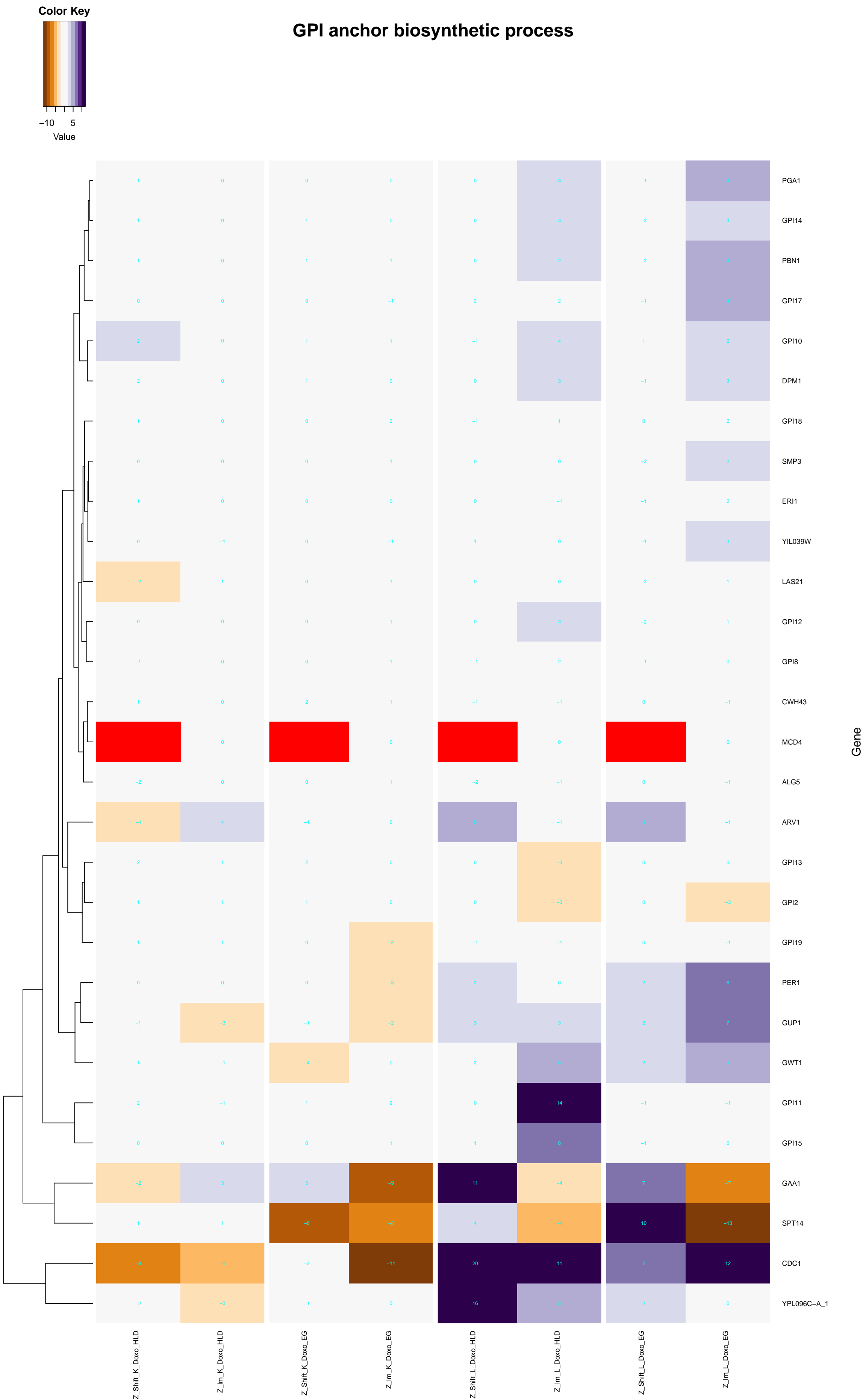

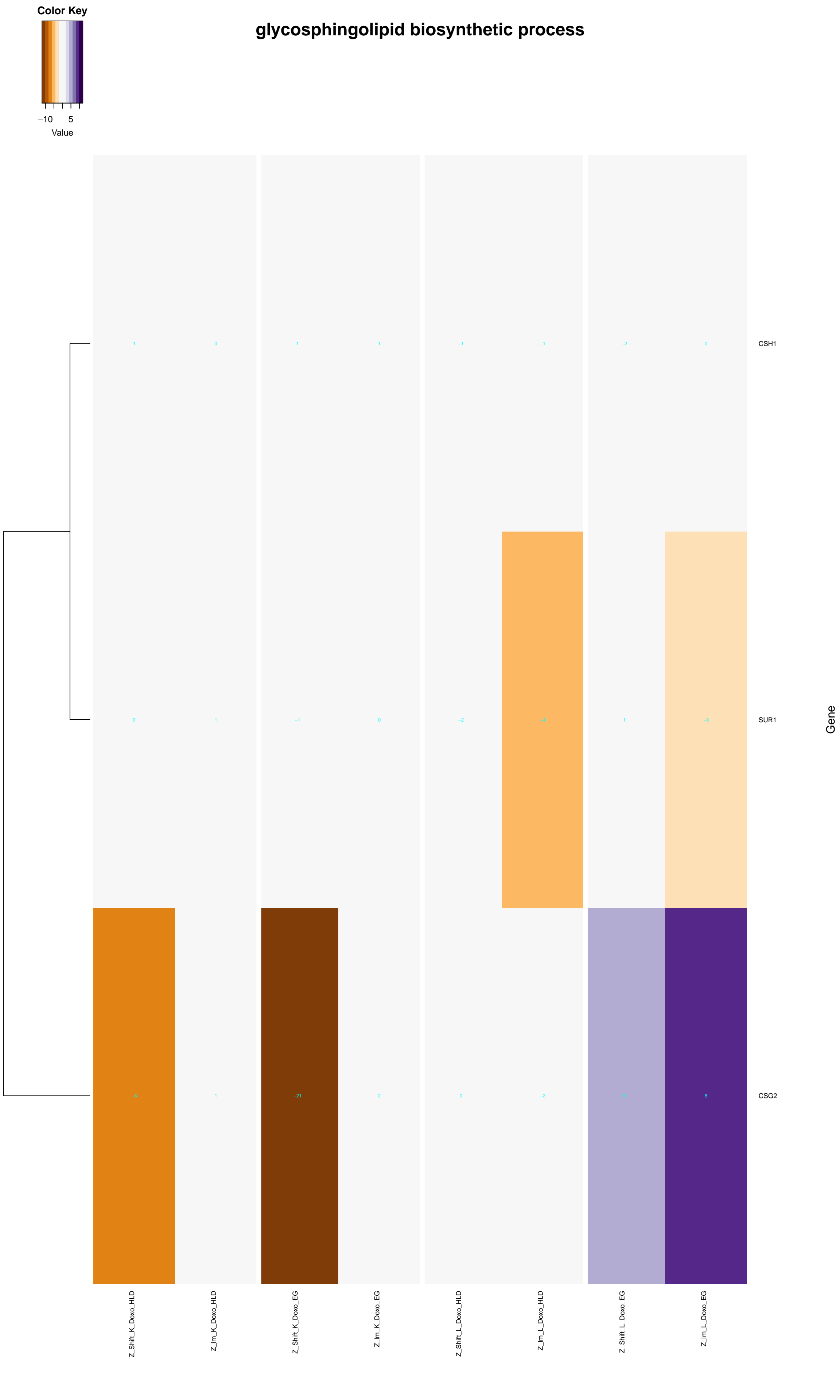

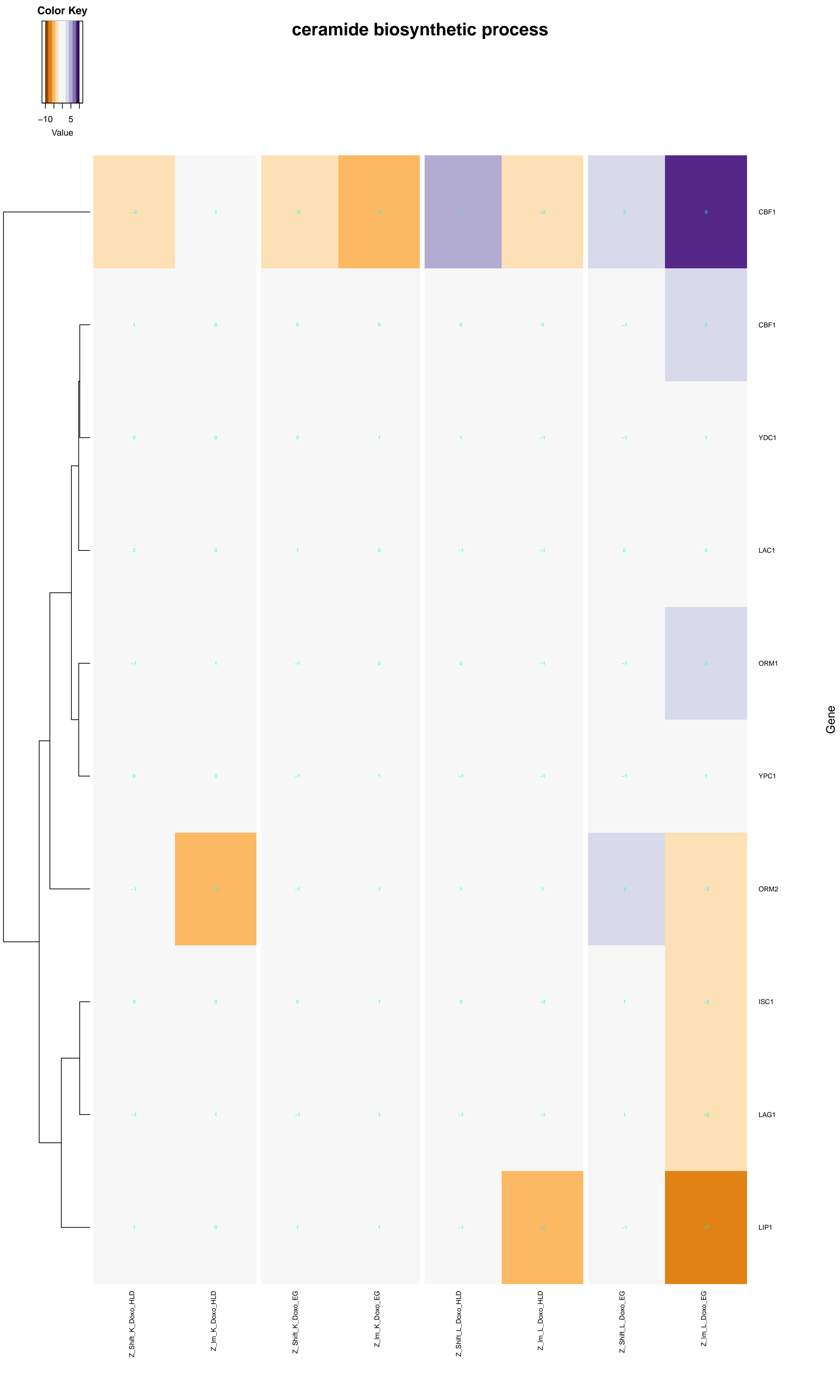

Supplement: Supplementary file 9 — Additional file 9. GO term-specific heatmaps for REMc/GTF-enriched clusters. GO term-specific heatmaps for significant GO process terms were generated as described in methods and Figs. 3 and 4. Any related child terms are presented in subsequent pages of the parent file name. GO terms with more than 100 children, with 2 or fewer genes annotated to the term, or a file size over 300KB are not shown. All heatmaps are generated with the same layout (see Figs. 3 and 4). [file 40170_2019_201_MOESM9_ESM.bz2 › Additional_File9_GOTermHeatmaps/Additional_File9_GOTermHeatmap/membrane_lipid_biosynthetic_process.pdf]

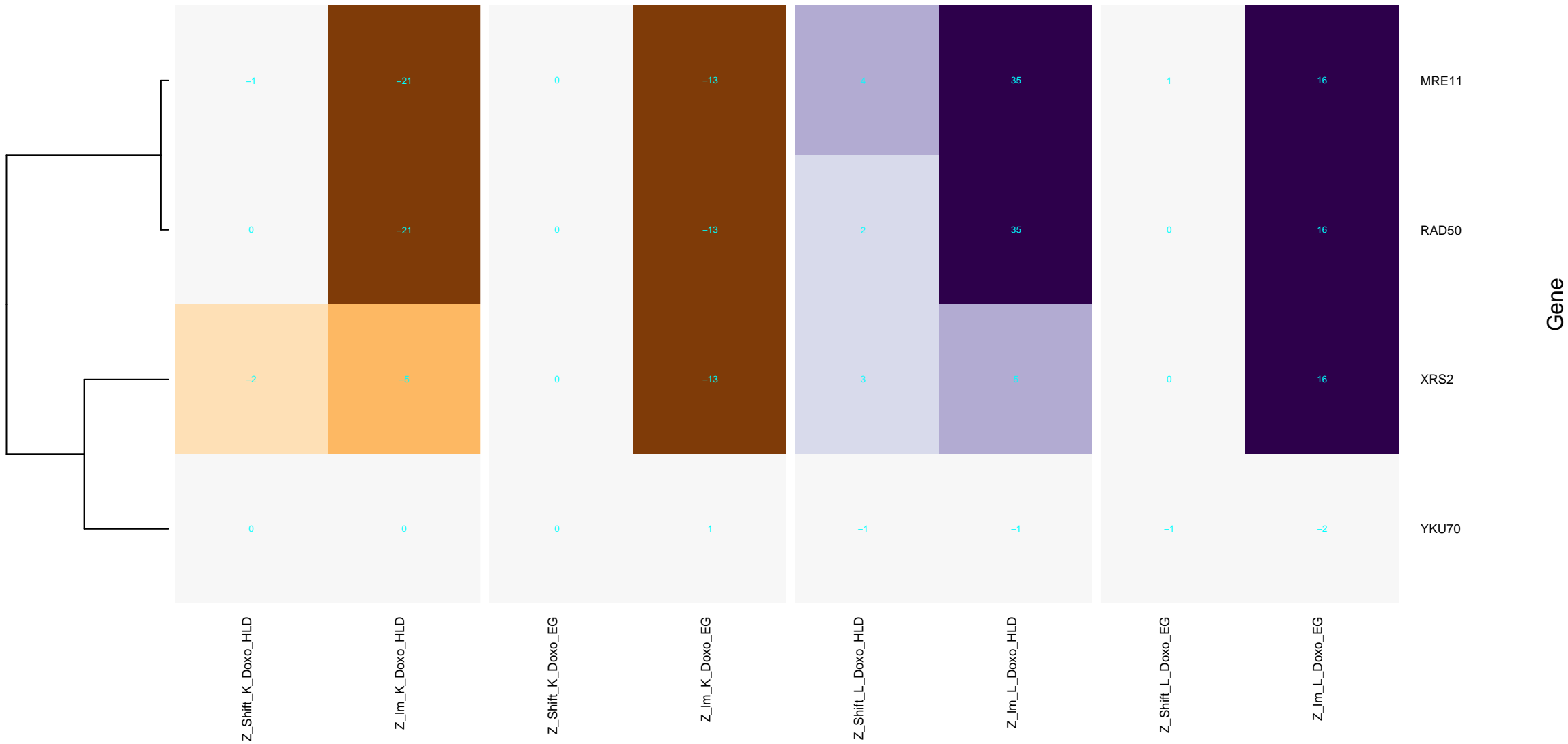

Supplement: Supplementary file 9 — Additional file 9. GO term-specific heatmaps for REMc/GTF-enriched clusters. GO term-specific heatmaps for significant GO process terms were generated as described in methods and Figs. 3 and 4. Any related child terms are presented in subsequent pages of the parent file name. GO terms with more than 100 children, with 2 or fewer genes annotated to the term, or a file size over 300KB are not shown. All heatmaps are generated with the same layout (see Figs. 3 and 4). [file 40170_2019_201_MOESM9_ESM.bz2 › Additional_File9_GOTermHeatmaps/Additional_File9_GOTermHeatmap/mitochondrial_double-strand_break_repair.pdf]

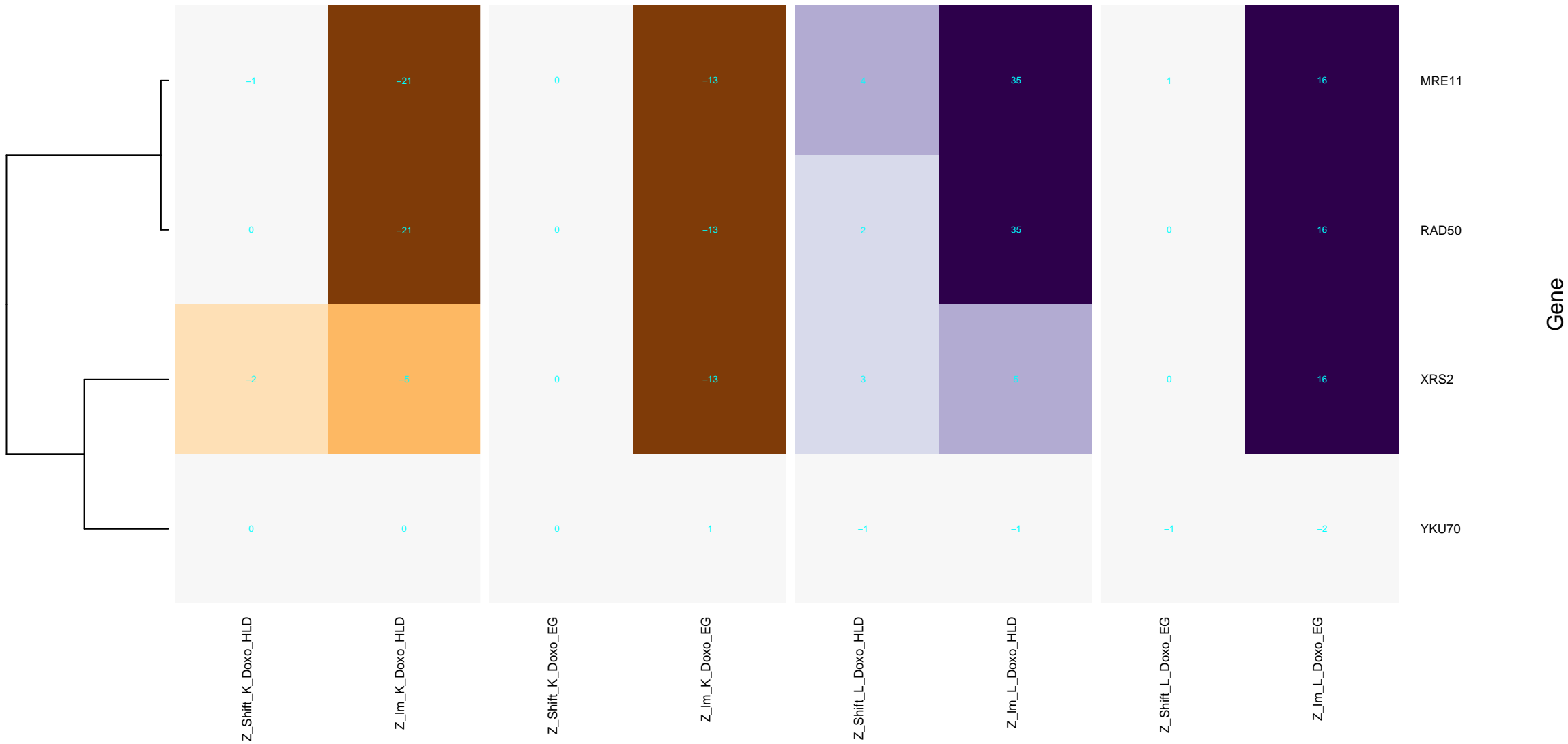

Supplement: Supplementary file 9 — Additional file 9. GO term-specific heatmaps for REMc/GTF-enriched clusters. GO term-specific heatmaps for significant GO process terms were generated as described in methods and Figs. 3 and 4. Any related child terms are presented in subsequent pages of the parent file name. GO terms with more than 100 children, with 2 or fewer genes annotated to the term, or a file size over 300KB are not shown. All heatmaps are generated with the same layout (see Figs. 3 and 4). [file 40170_2019_201_MOESM9_ESM.bz2 › Additional_File9_GOTermHeatmaps/Additional_File9_GOTermHeatmap/mitochondrial_double-strand_break_repair_via_homologous_recombination.pdf]

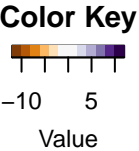

# mitochondrial genome maintenance

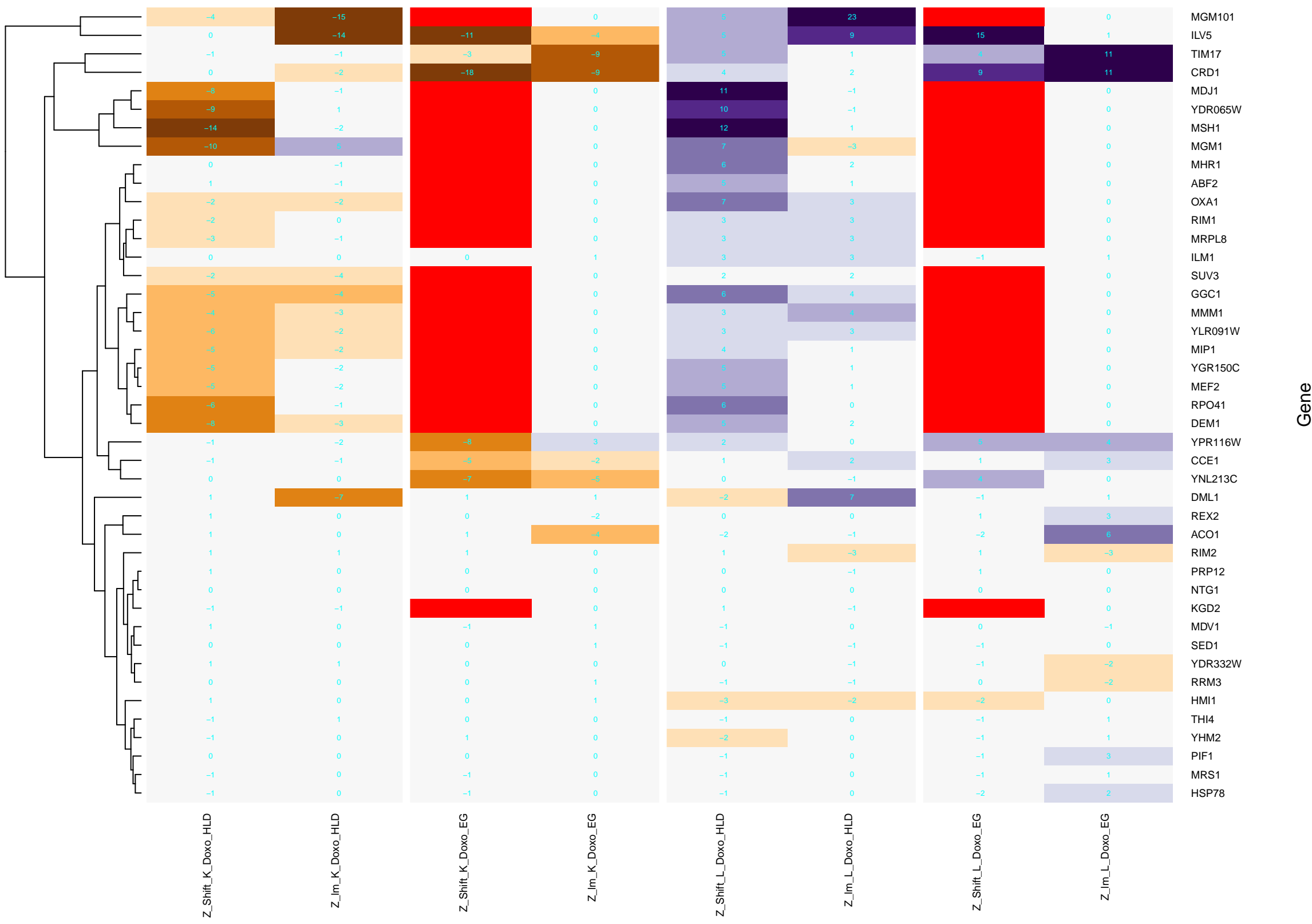

Supplement: Supplementary file 9 — Additional file 9. GO term-specific heatmaps for REMc/GTF-enriched clusters. GO term-specific heatmaps for significant GO process terms were generated as described in methods and Figs. 3 and 4. Any related child terms are presented in subsequent pages of the parent file name. GO terms with more than 100 children, with 2 or fewer genes annotated to the term, or a file size over 300KB are not shown. All heatmaps are generated with the same layout (see Figs. 3 and 4). [file 40170_2019_201_MOESM9_ESM.bz2 › Additional_File9_GOTermHeatmaps/Additional_File9_GOTermHeatmap/mitochondrial_genome_maintenance.pdf]

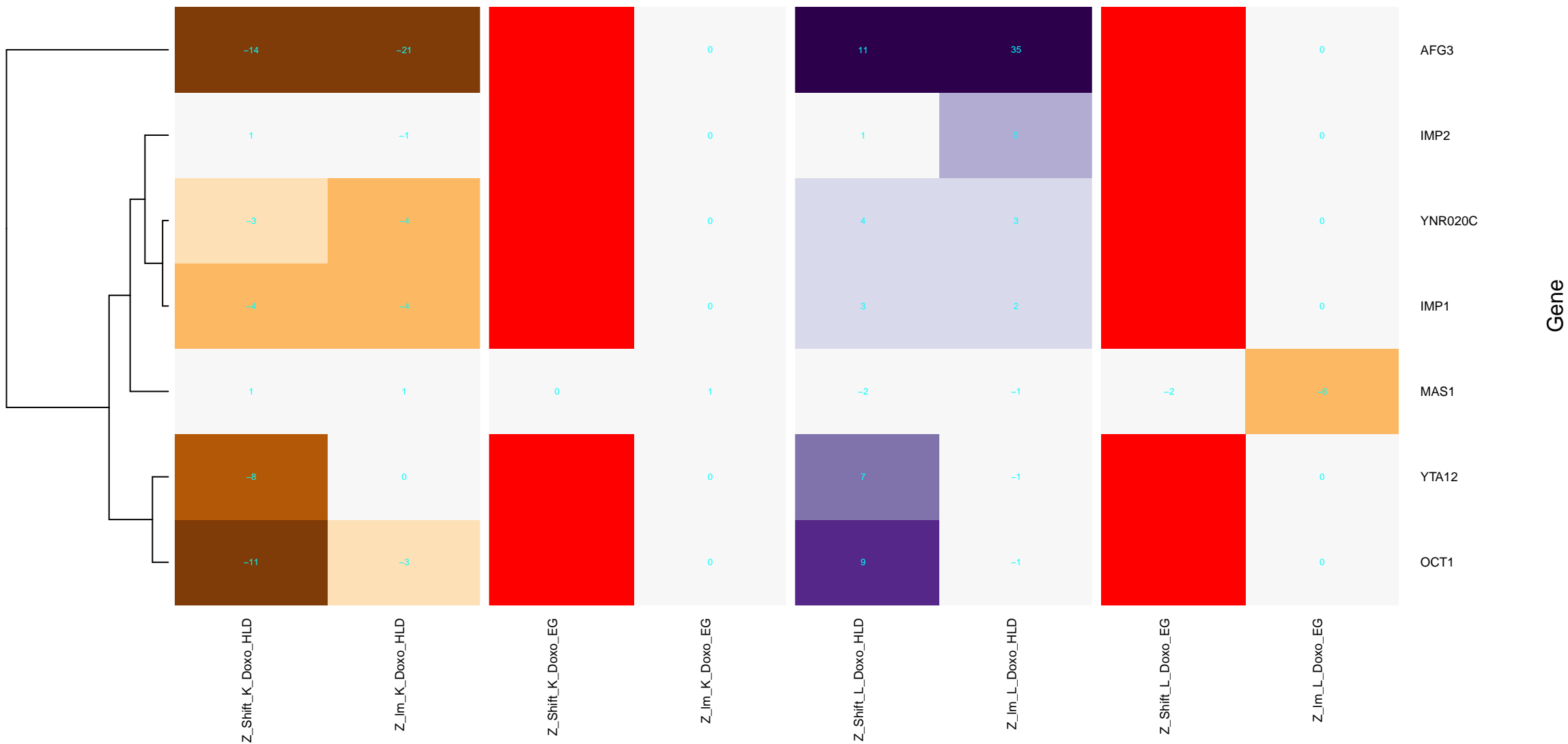

Supplement: Supplementary file 9 — Additional file 9. GO term-specific heatmaps for REMc/GTF-enriched clusters. GO term-specific heatmaps for significant GO process terms were generated as described in methods and Figs. 3 and 4. Any related child terms are presented in subsequent pages of the parent file name. GO terms with more than 100 children, with 2 or fewer genes annotated to the term, or a file size over 300KB are not shown. All heatmaps are generated with the same layout (see Figs. 3 and 4). [file 40170_2019_201_MOESM9_ESM.bz2 › Additional_File9_GOTermHeatmaps/Additional_File9_GOTermHeatmap/mitochondrial_protein_processing.pdf]

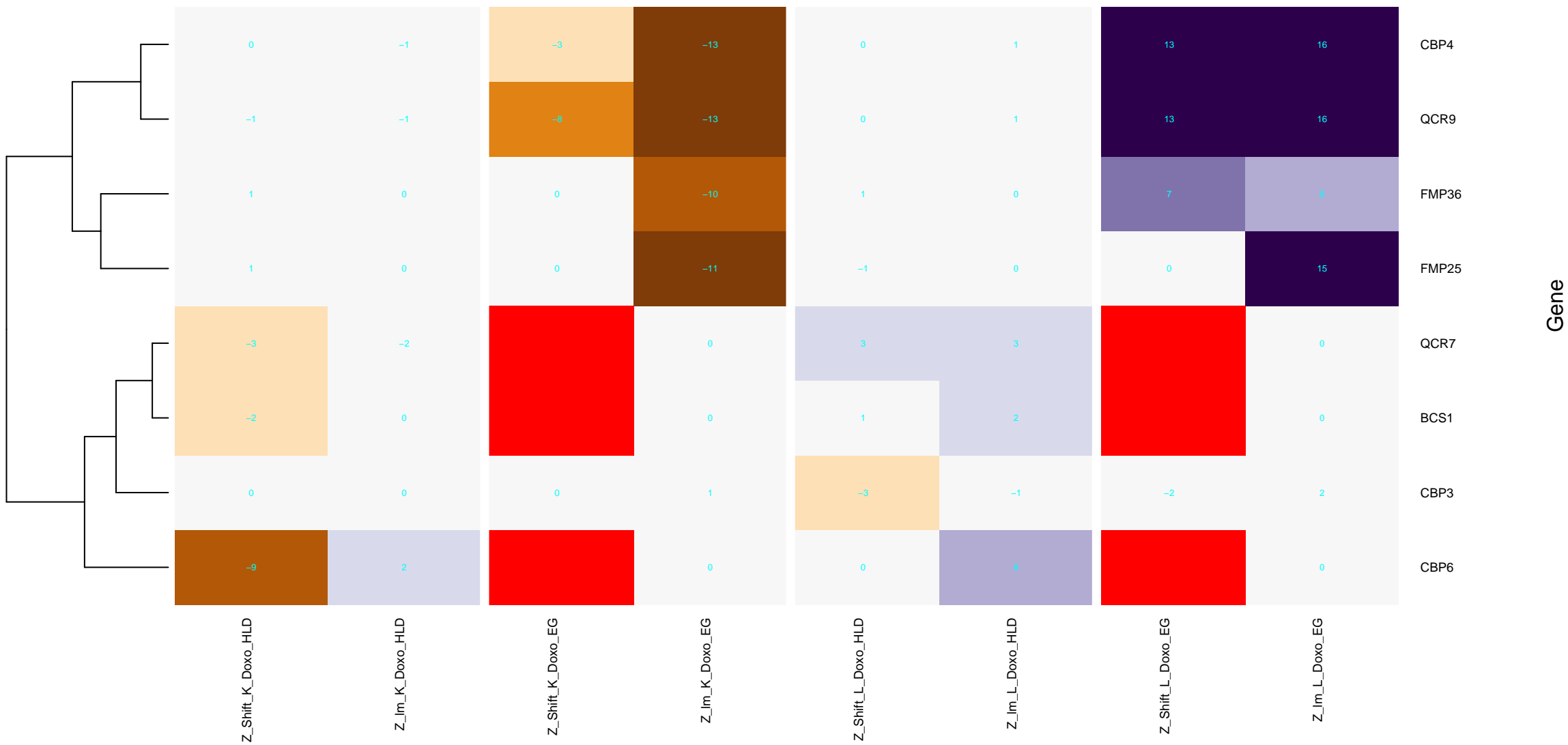

Supplement: Supplementary file 9 — Additional file 9. GO term-specific heatmaps for REMc/GTF-enriched clusters. GO term-specific heatmaps for significant GO process terms were generated as described in methods and Figs. 3 and 4. Any related child terms are presented in subsequent pages of the parent file name. GO terms with more than 100 children, with 2 or fewer genes annotated to the term, or a file size over 300KB are not shown. All heatmaps are generated with the same layout (see Figs. 3 and 4). [file 40170_2019_201_MOESM9_ESM.bz2 › Additional_File9_GOTermHeatmaps/Additional_File9_GOTermHeatmap/mitochondrial_respiratory_chain_complex_III_assembly.pdf]

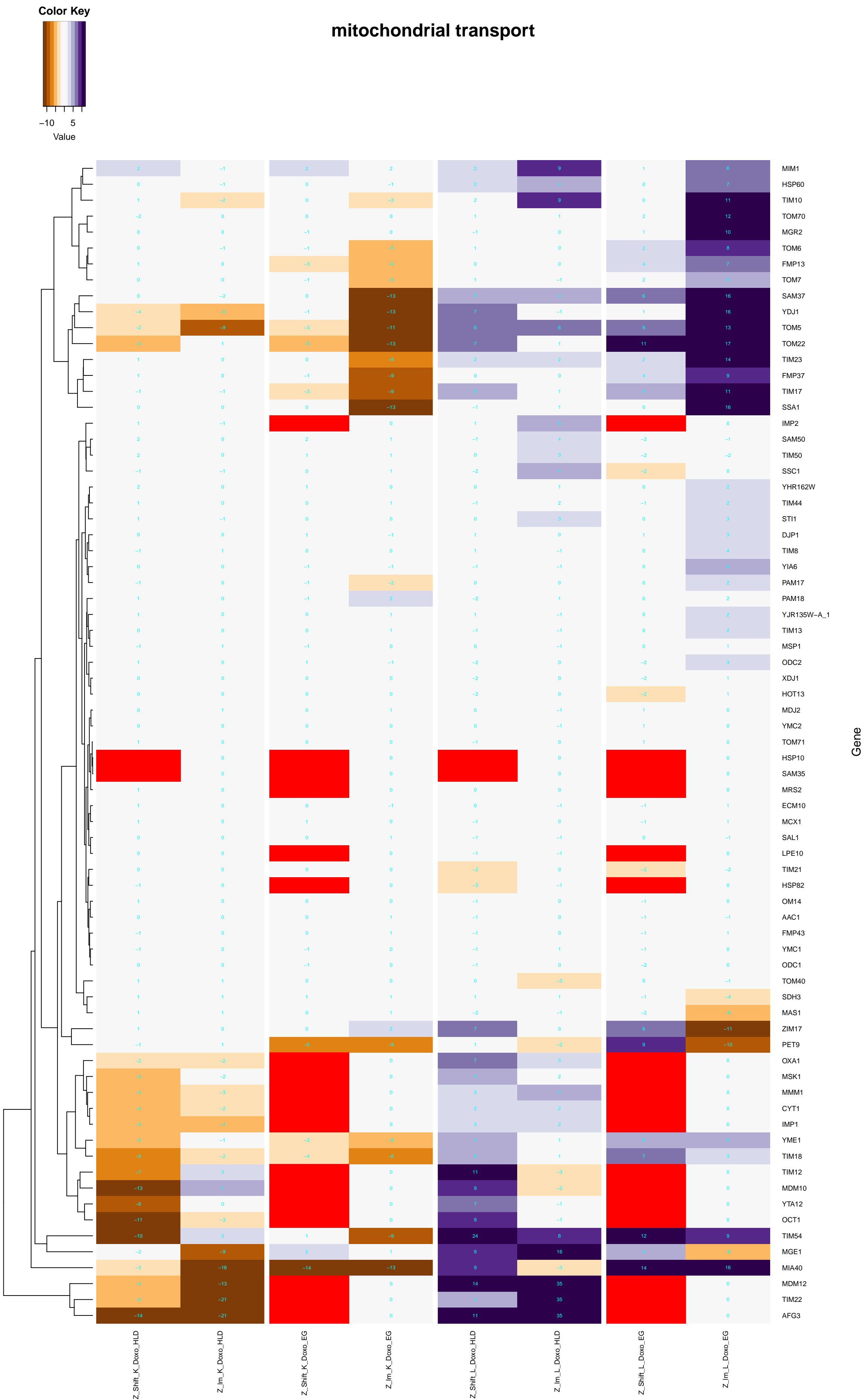

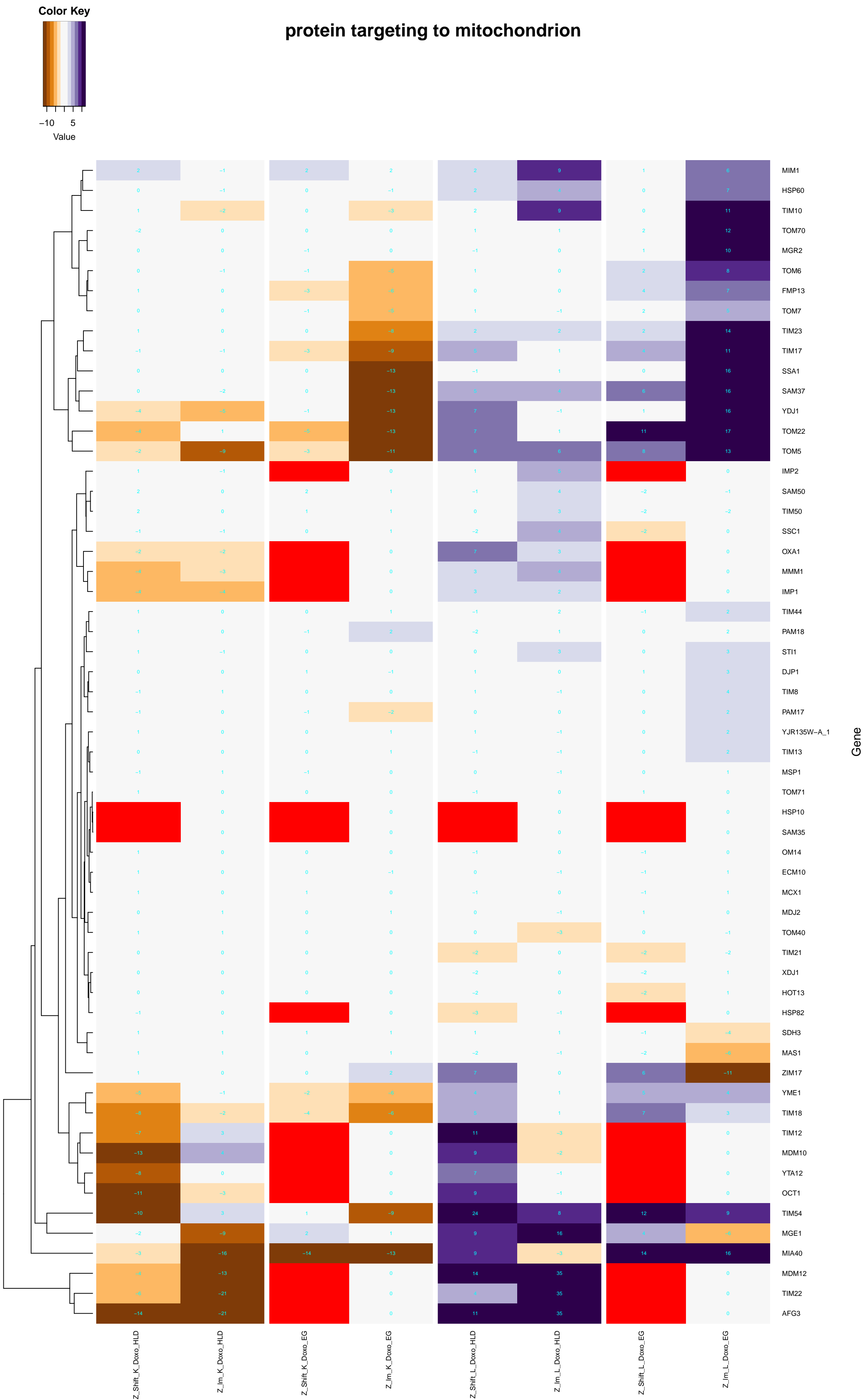

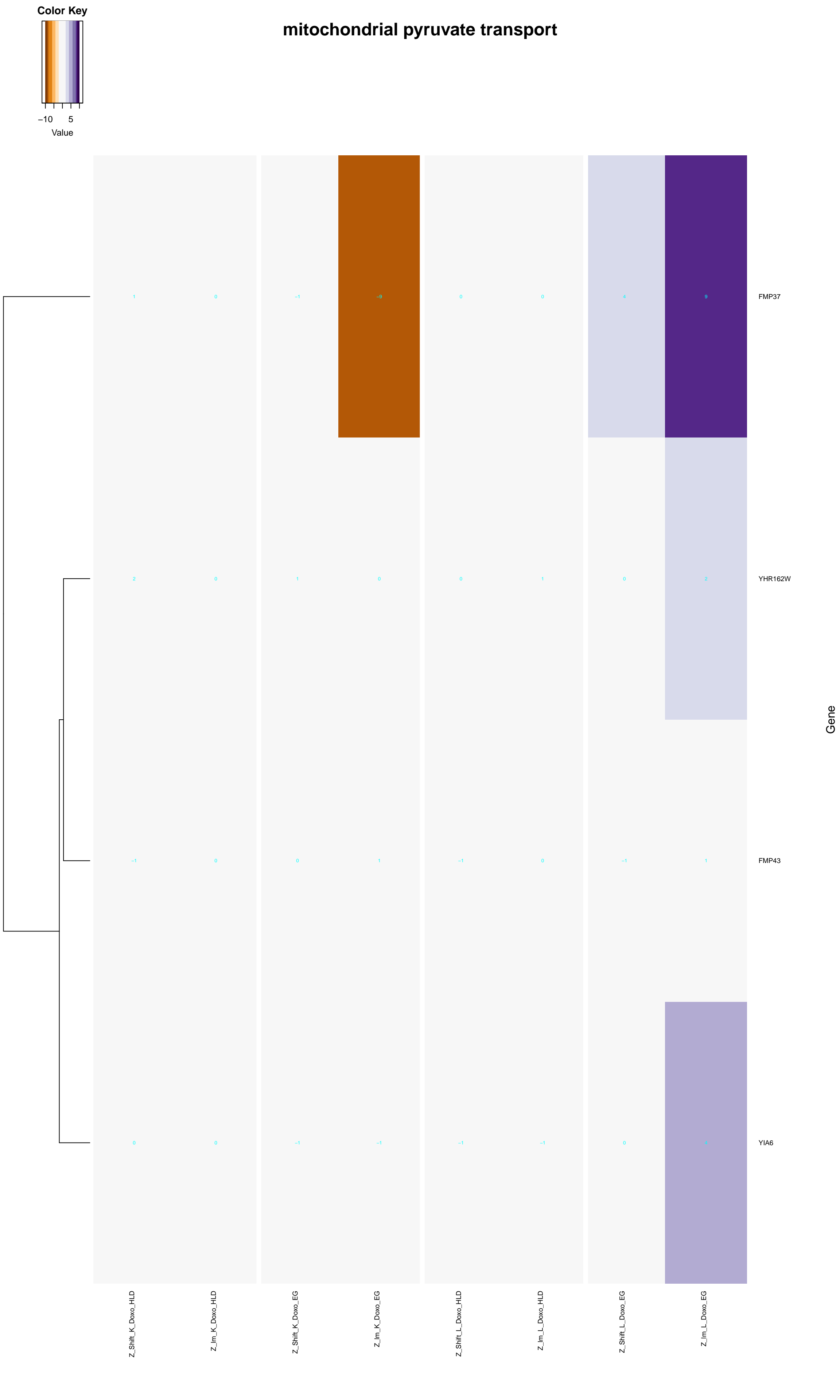

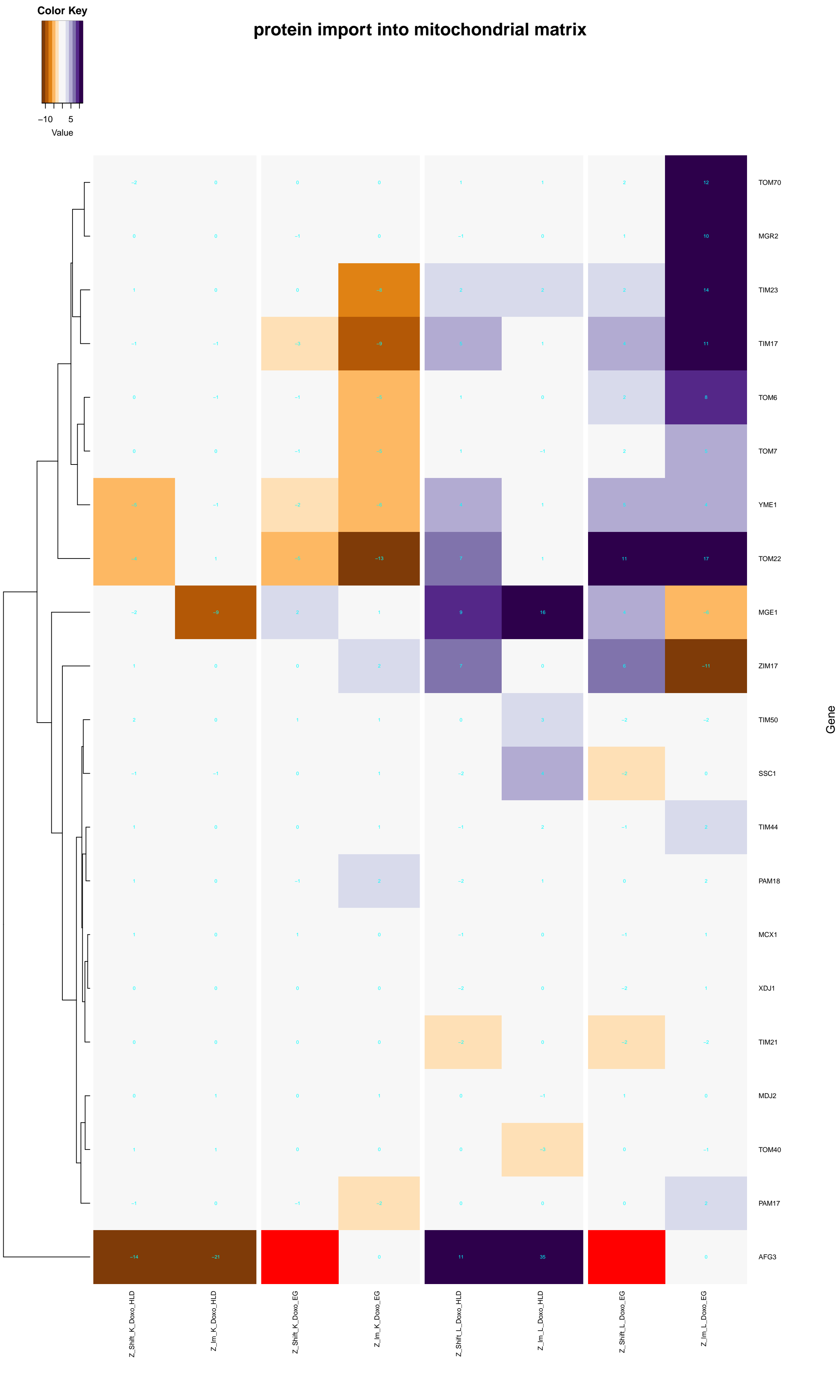

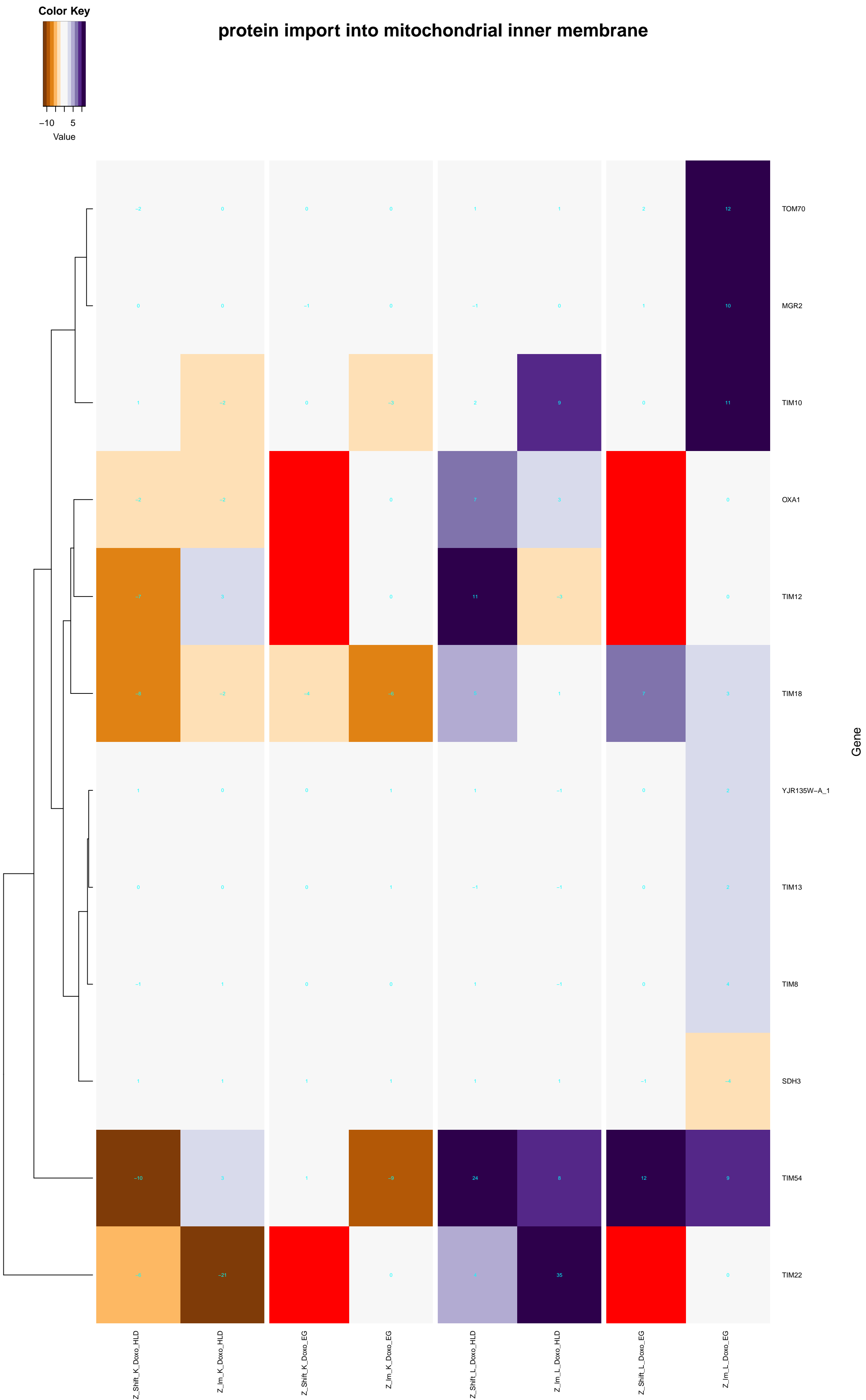

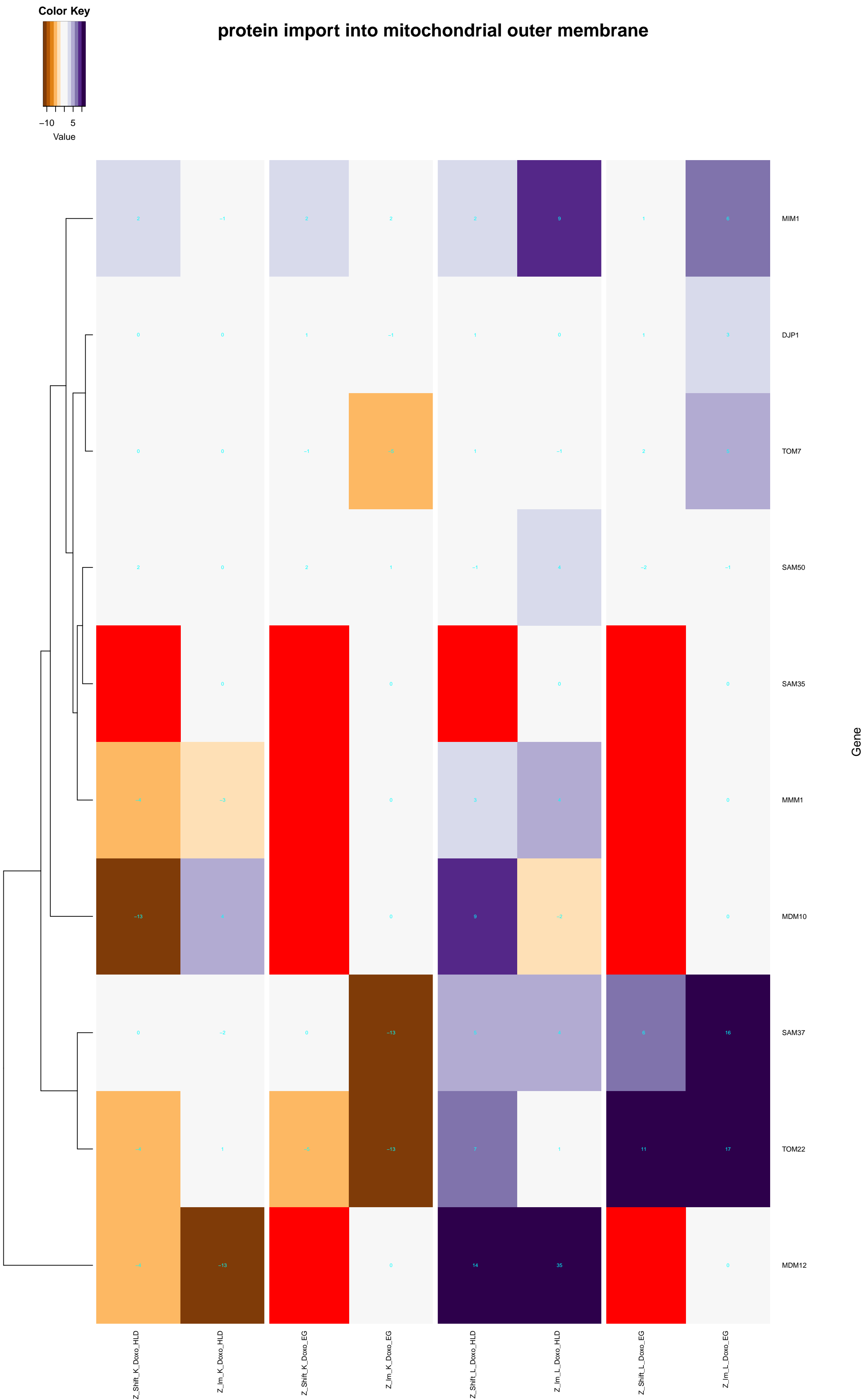

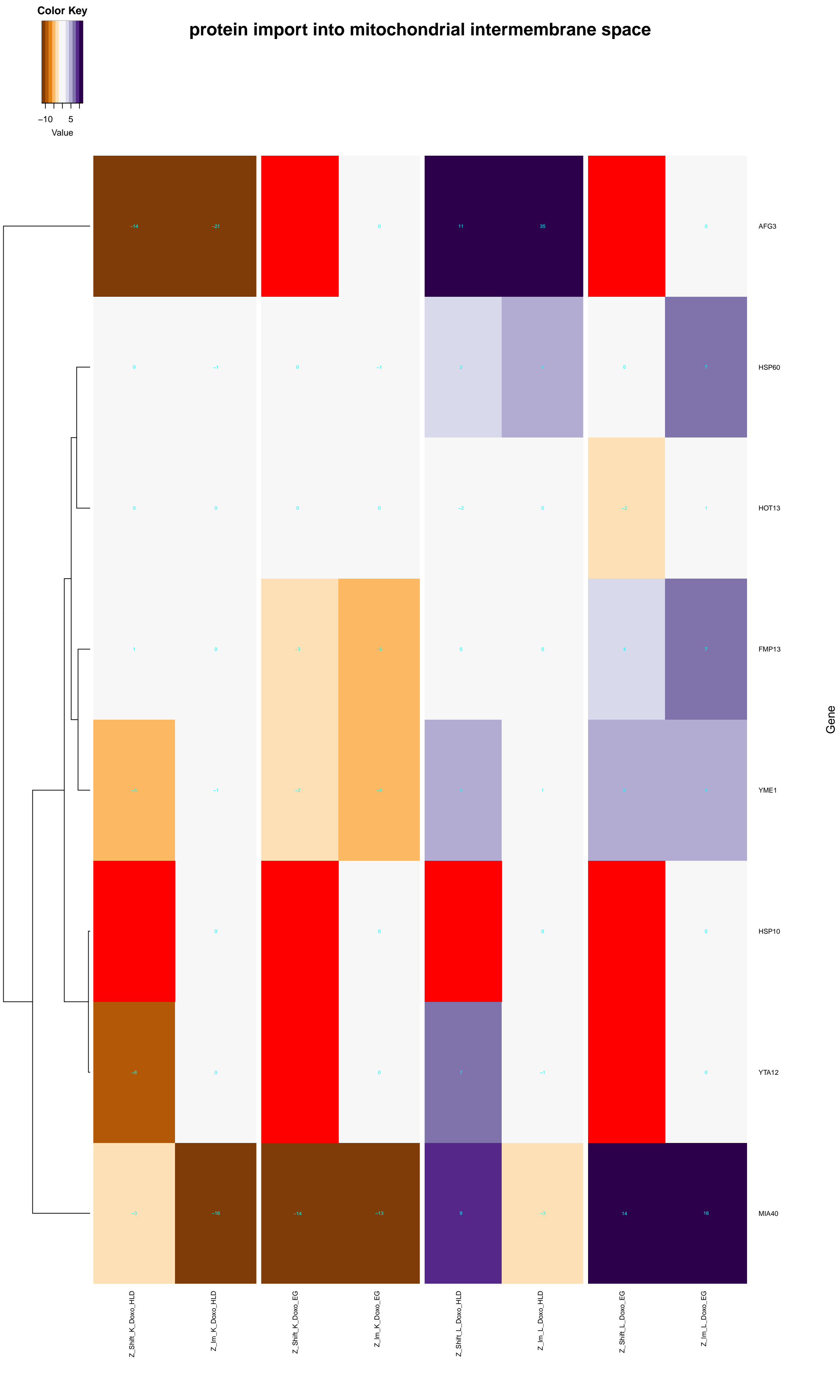

Supplement: Supplementary file 9 — Additional file 9. GO term-specific heatmaps for REMc/GTF-enriched clusters. GO term-specific heatmaps for significant GO process terms were generated as described in methods and Figs. 3 and 4. Any related child terms are presented in subsequent pages of the parent file name. GO terms with more than 100 children, with 2 or fewer genes annotated to the term, or a file size over 300KB are not shown. All heatmaps are generated with the same layout (see Figs. 3 and 4). [file 40170_2019_201_MOESM9_ESM.bz2 › Additional_File9_GOTermHeatmaps/Additional_File9_GOTermHeatmap/mitochondrial_transport.pdf]

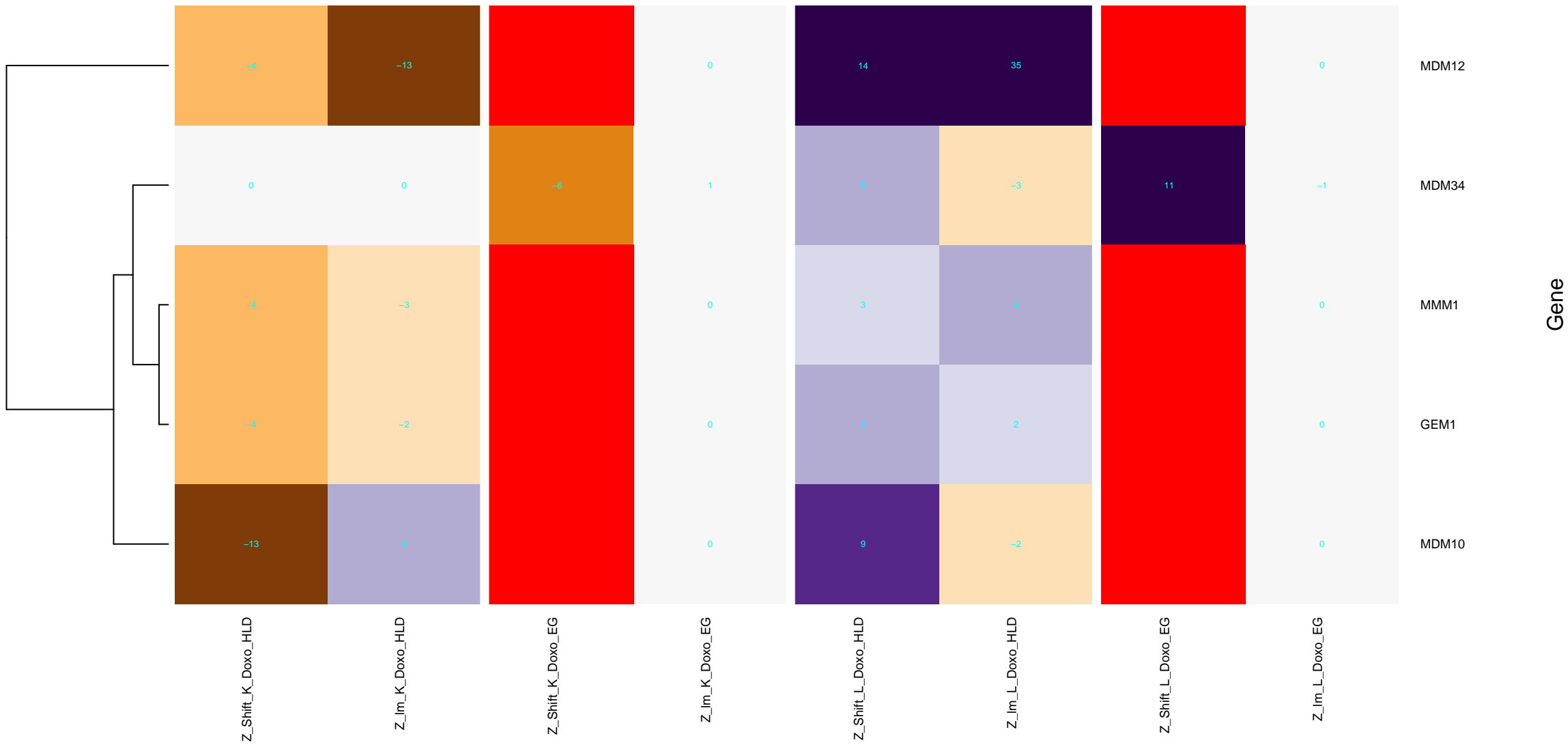

Supplement: Supplementary file 9 — Additional file 9. GO term-specific heatmaps for REMc/GTF-enriched clusters. GO term-specific heatmaps for significant GO process terms were generated as described in methods and Figs. 3 and 4. Any related child terms are presented in subsequent pages of the parent file name. GO terms with more than 100 children, with 2 or fewer genes annotated to the term, or a file size over 300KB are not shown. All heatmaps are generated with the same layout (see Figs. 3 and 4). [file 40170_2019_201_MOESM9_ESM.bz2 › Additional_File9_GOTermHeatmaps/Additional_File9_GOTermHeatmap/mitochondrion-ER_tethering.pdf]

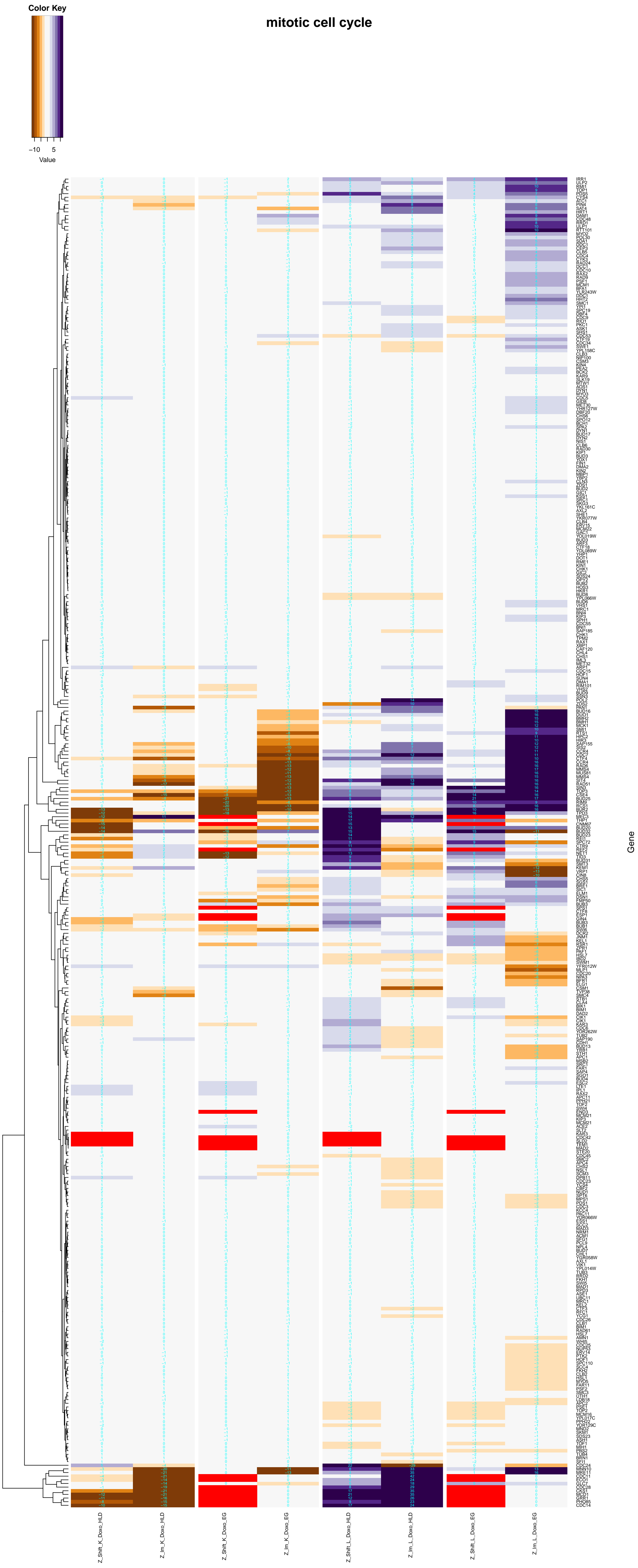

Supplement: Supplementary file 9 — Additional file 9. GO term-specific heatmaps for REMc/GTF-enriched clusters. GO term-specific heatmaps for significant GO process terms were generated as described in methods and Figs. 3 and 4. Any related child terms are presented in subsequent pages of the parent file name. GO terms with more than 100 children, with 2 or fewer genes annotated to the term, or a file size over 300KB are not shown. All heatmaps are generated with the same layout (see Figs. 3 and 4). [file 40170_2019_201_MOESM9_ESM.bz2 › Additional_File9_GOTermHeatmaps/Additional_File9_GOTermHeatmap/mitotic_cell_cycle.pdf]

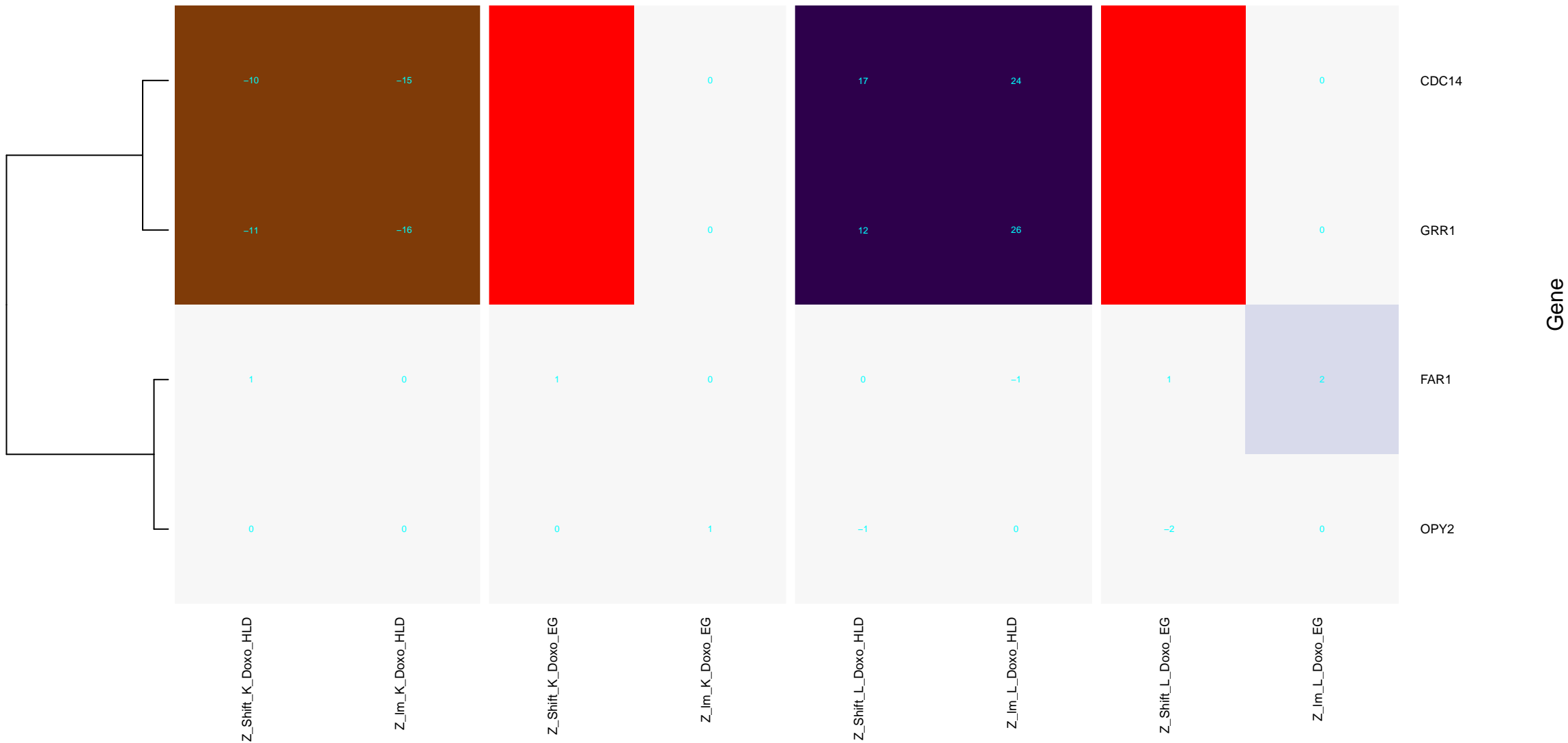

Supplement: Supplementary file 9 — Additional file 9. GO term-specific heatmaps for REMc/GTF-enriched clusters. GO term-specific heatmaps for significant GO process terms were generated as described in methods and Figs. 3 and 4. Any related child terms are presented in subsequent pages of the parent file name. GO terms with more than 100 children, with 2 or fewer genes annotated to the term, or a file size over 300KB are not shown. All heatmaps are generated with the same layout (see Figs. 3 and 4). [file 40170_2019_201_MOESM9_ESM.bz2 › Additional_File9_GOTermHeatmaps/Additional_File9_GOTermHeatmap/mitotic_cell_cycle_arrest.pdf]

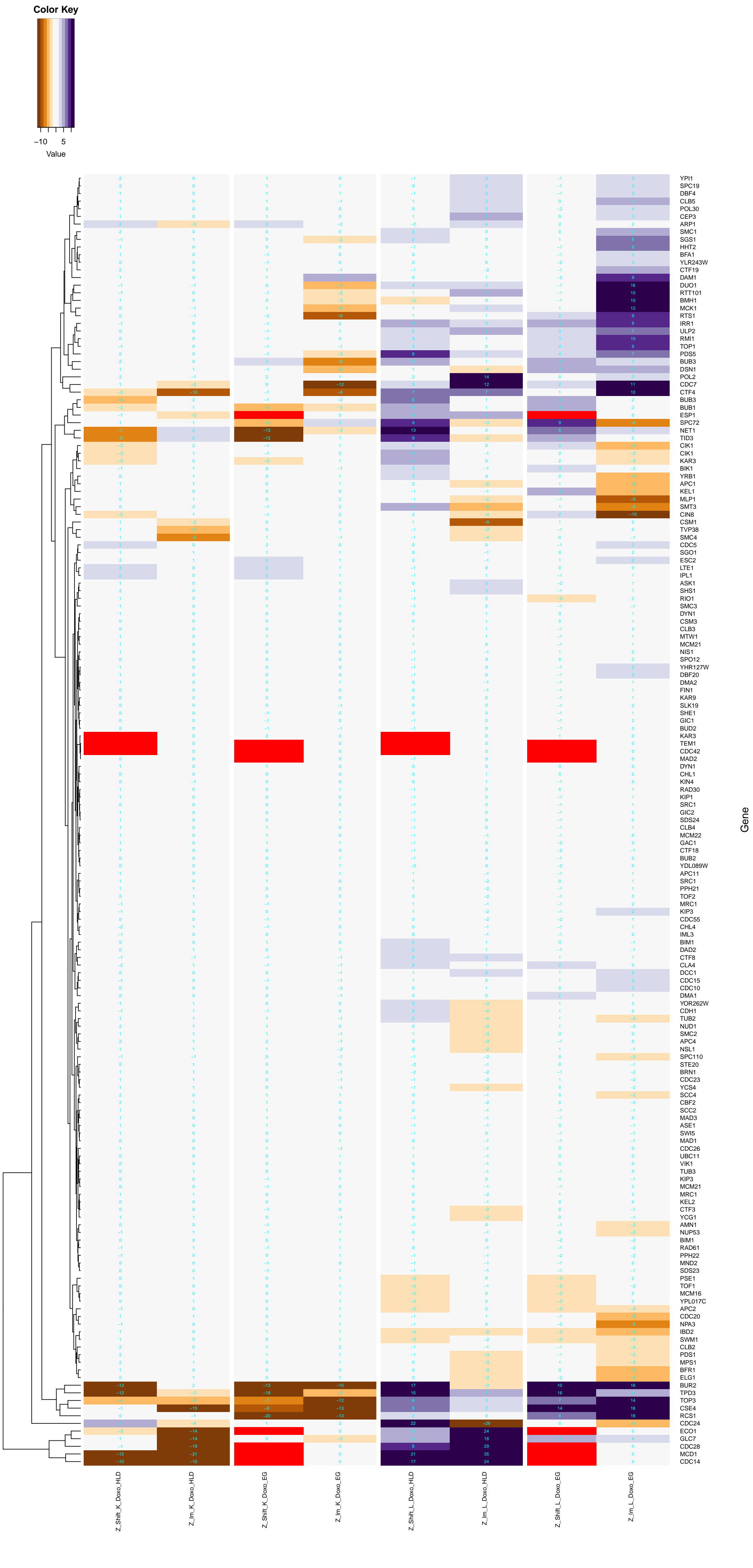

Supplement: Supplementary file 9 — Additional file 9. GO term-specific heatmaps for REMc/GTF-enriched clusters. GO term-specific heatmaps for significant GO process terms were generated as described in methods and Figs. 3 and 4. Any related child terms are presented in subsequent pages of the parent file name. GO terms with more than 100 children, with 2 or fewer genes annotated to the term, or a file size over 300KB are not shown. All heatmaps are generated with the same layout (see Figs. 3 and 4). [file 40170_2019_201_MOESM9_ESM.bz2 › Additional_File9_GOTermHeatmaps/Additional_File9_GOTermHeatmap/mitotic_nuclear_division.pdf]
