# Supplementary material for: A yeast phenomic model for the influence of Warburg metabolism on genetic buffering of doxorubicin
Source: Cancer Metab. 2019 Oct 23;7:9. doi: 10.1186/s40170-019-0201-3 (PMC6806529; doi:10.1186/s40170-019-0201-3)
Supplement: Supplementary file 11 — Additional file 11. Integration of yeast phenomic and cancer cell line pharmacogenomic data to predict human genes that modify doxorubicin toxicity in cancer cells. (A) Tables of UES and OES human genes and whether their yeast homologs were found to be deletion enhancing or deletion suppressing, respectively. (B-C) Overlap between the gCSI and GDSC1000 databases with regard to UES and OES human genes (B) across all tissues or (C) for individual tissues. Note: the intersection of UES with OES between gCSI and GDSC was used as a negative control for assessing UES and OES overlap. (D-E) Yeast phenomic doxorubicin-gene interaction profiles for homologs of human UES or OES genes, sub-classified according to interaction type (deletion enhancing or suppressing) and Warburg-dependence of the interaction, for the (D) gCSI or (E) GDSC1000 databases. Similar to Fig. 11, yeast-human homology relationships are shown to the left of heatmaps (blue - one to one; green - one to many; red - many to many). (F-I) Interactive plots for yeast-human homologs, comparing the p-value of human genes to L interaction scores for yeast counterparts in (F, G) HLD or (H, I) HLEG from (F, H) gCSI or (G, I) GDSC1000. For the standardized coefficient (‘estimate’; color gradient), a negative value (purple) indicates UES, while a positive value (orange) indicates OES. Thus, the model would predict causality for a human gene if its yeast homolog has a positive L interaction (deletion enhancing) and is colored purple (UES), or a negative L interaction (deletion suppressing) and colored orange (OES). Genes are only plotted if the human homolog was significant (p-value < 0.05). [file 40170_2019_201_MOESM11_ESM.bz2 › Additional_File11_Pharmacogenomics/C - ByTissue.pdf]

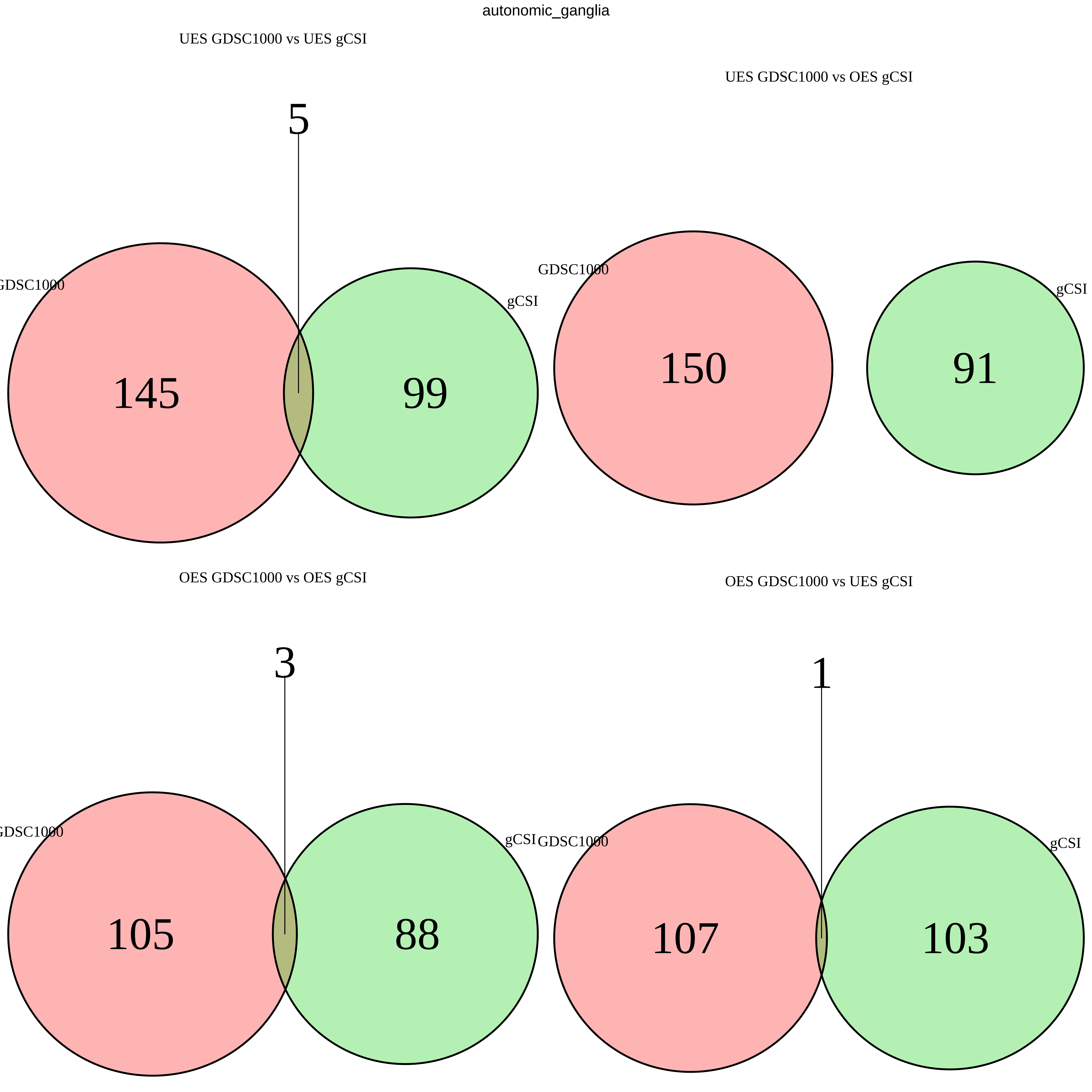
autonomic_ganglia
UES GDSC1000 vs UES gCSI
GDSC1000
gCSI
145
5
99
UES GDSC1000 vs OES gCSI
GDSC1000
gCSI
150
91
OES GDSC1000 vs OES gCSI
GDSC1000
gCSI
105
3
88
OES GDSC1000 vs UES gCSI
GDSC1000
gCSI
107
1
103

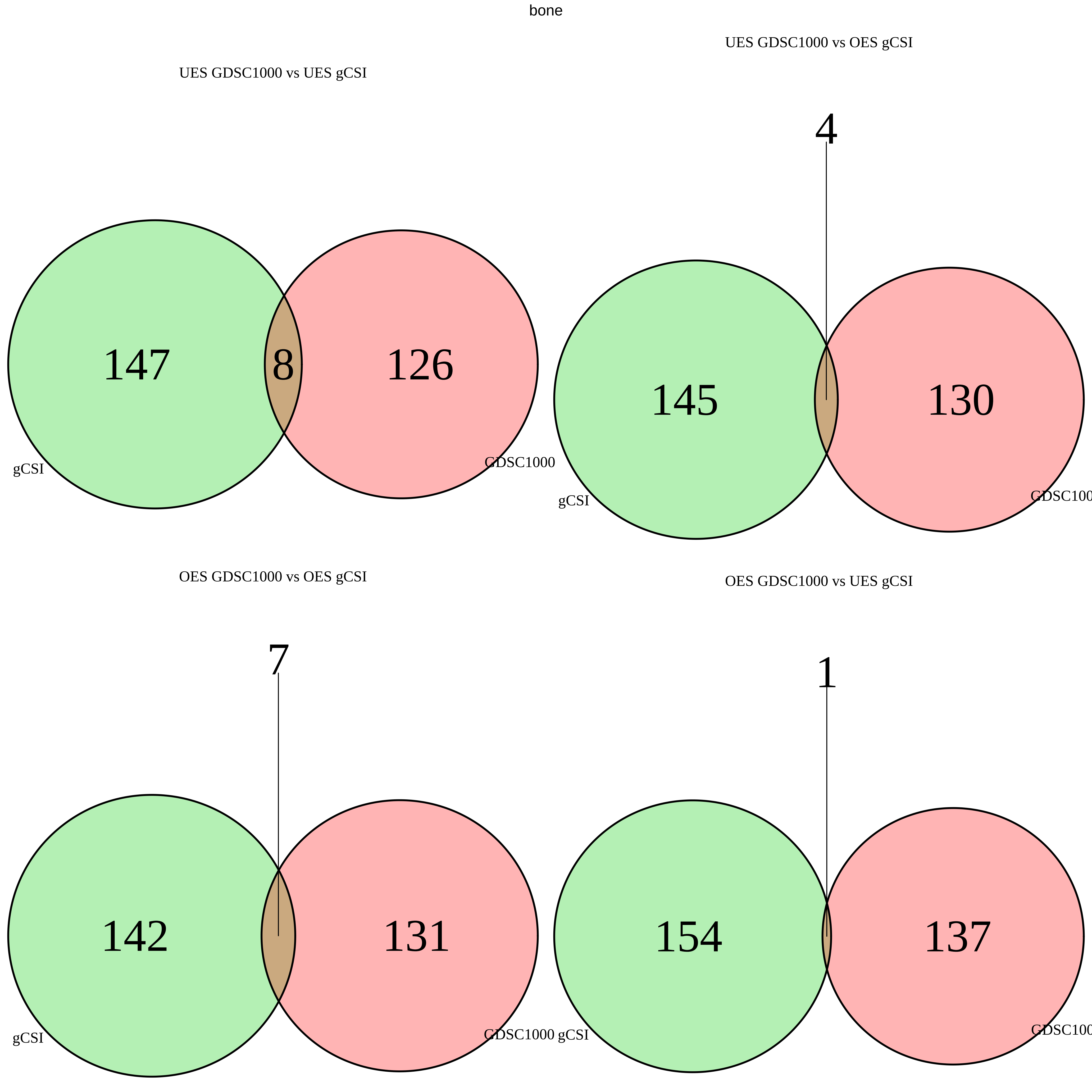
bone
UES GDSC1000 vs UES gCSI
147
8
126
gCSI
GDSC1000
UES GDSC1000 vs OES gCSI
4
145
130
gCSI
GDSC100
OES GDSC1000 vs OES gCSI
7
142
131
gCSI
GDSC1000
OES GDSC1000 vs UES gCSI
1
154
137
gCSI
GDSC100

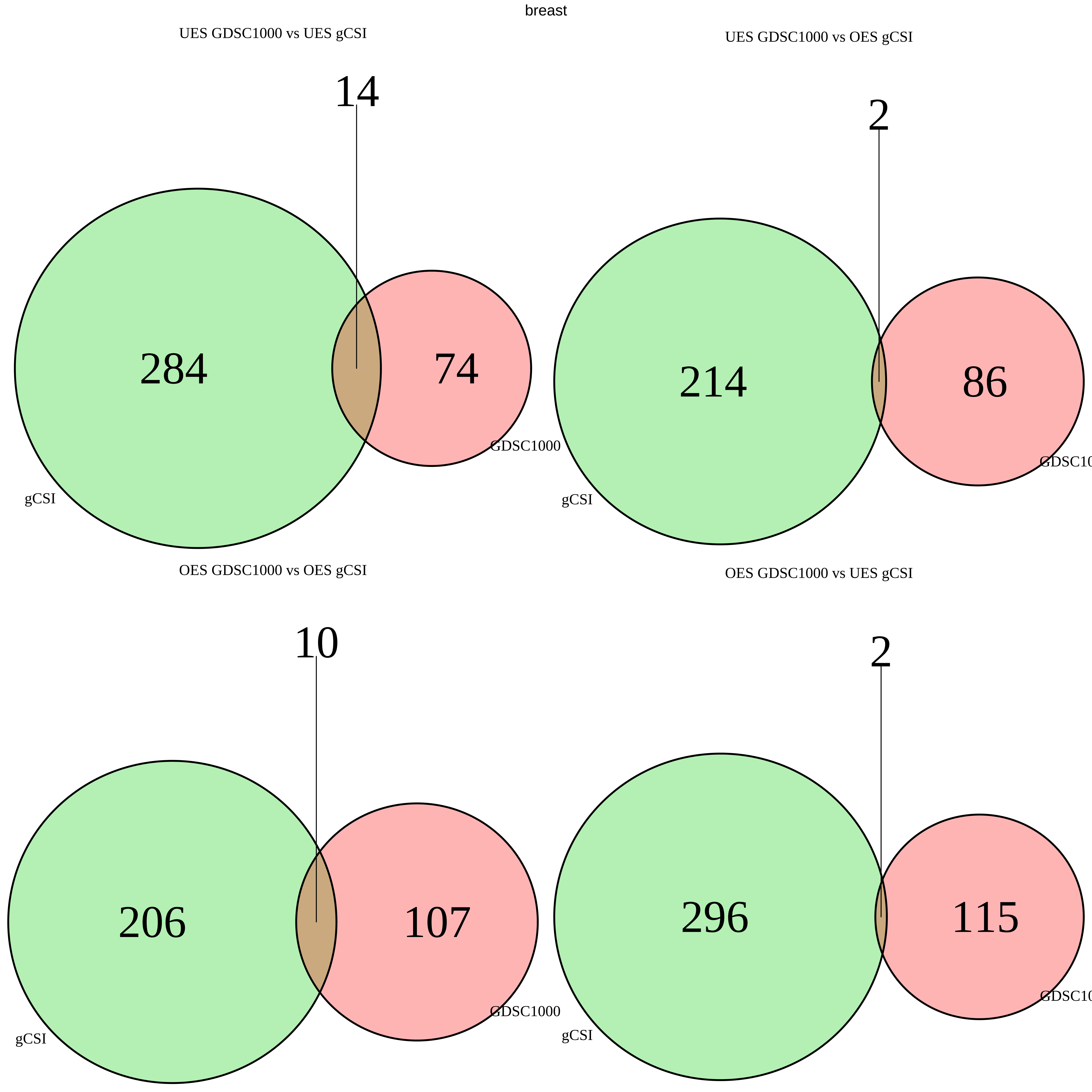
breast
UES GDSC1000 vs UES gCSI
14
284
74
GDSC1000
gCSI
UES GDSC1000 vs OES gCSI
2
214
86
GDSC10
gCSI
OES GDSC1000 vs OES gCSI
10
206
107
GDSC1000
gCSI
OES GDSC1000 vs UES gCSI
2
296
115
GDSC10
gCSI

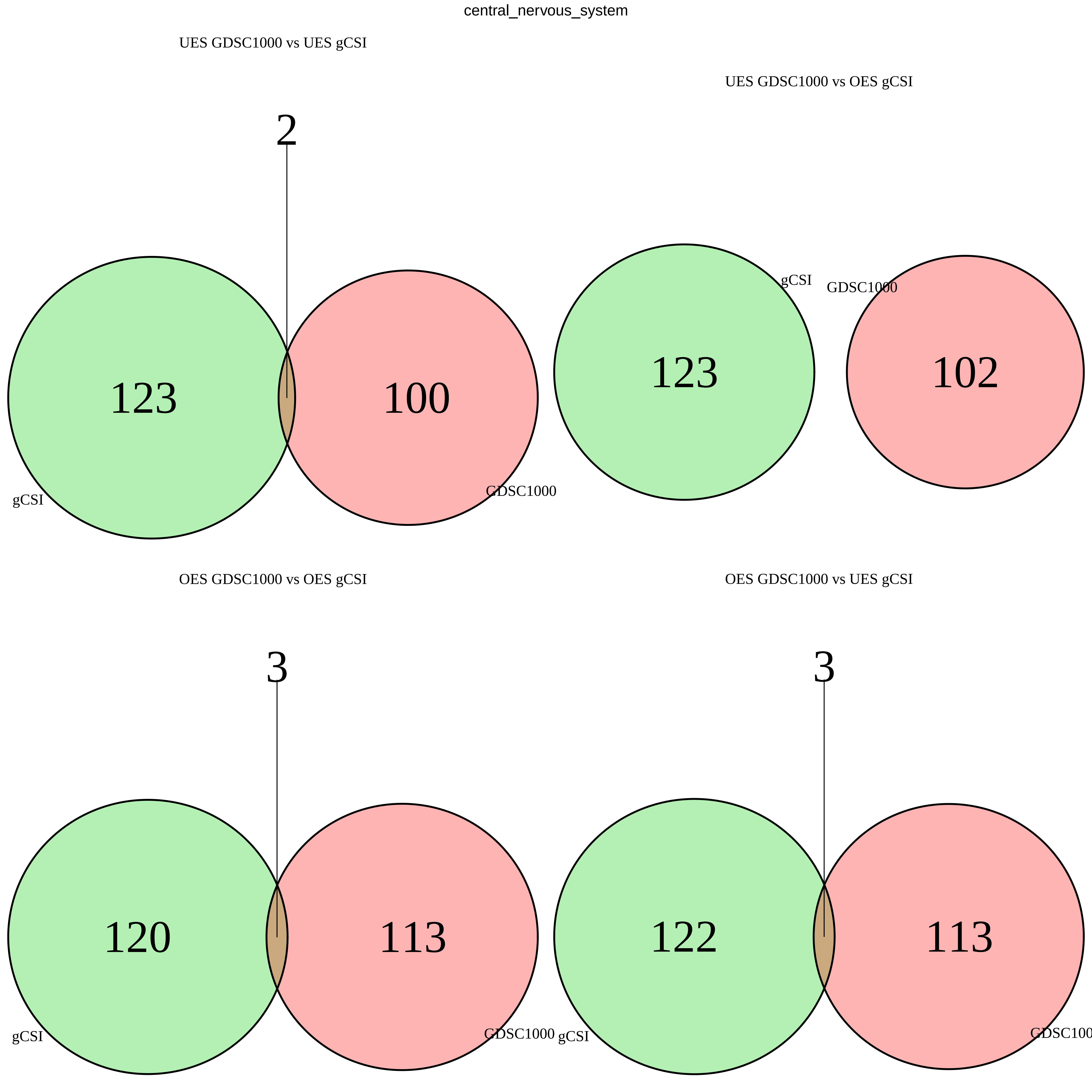
central_nervous_system
UES GDSC1000 vs UES gCSI
2
123
100
gCSI
GDSC1000
UES GDSC1000 vs OES gCSI
gCSI
GDSC1000
123
102
OES GDSC1000 vs OES gCSI
3
120
113
gCSI
GDSC1000
OES GDSC1000 vs UES gCSI
3
122
113
gCSI
GDSC100

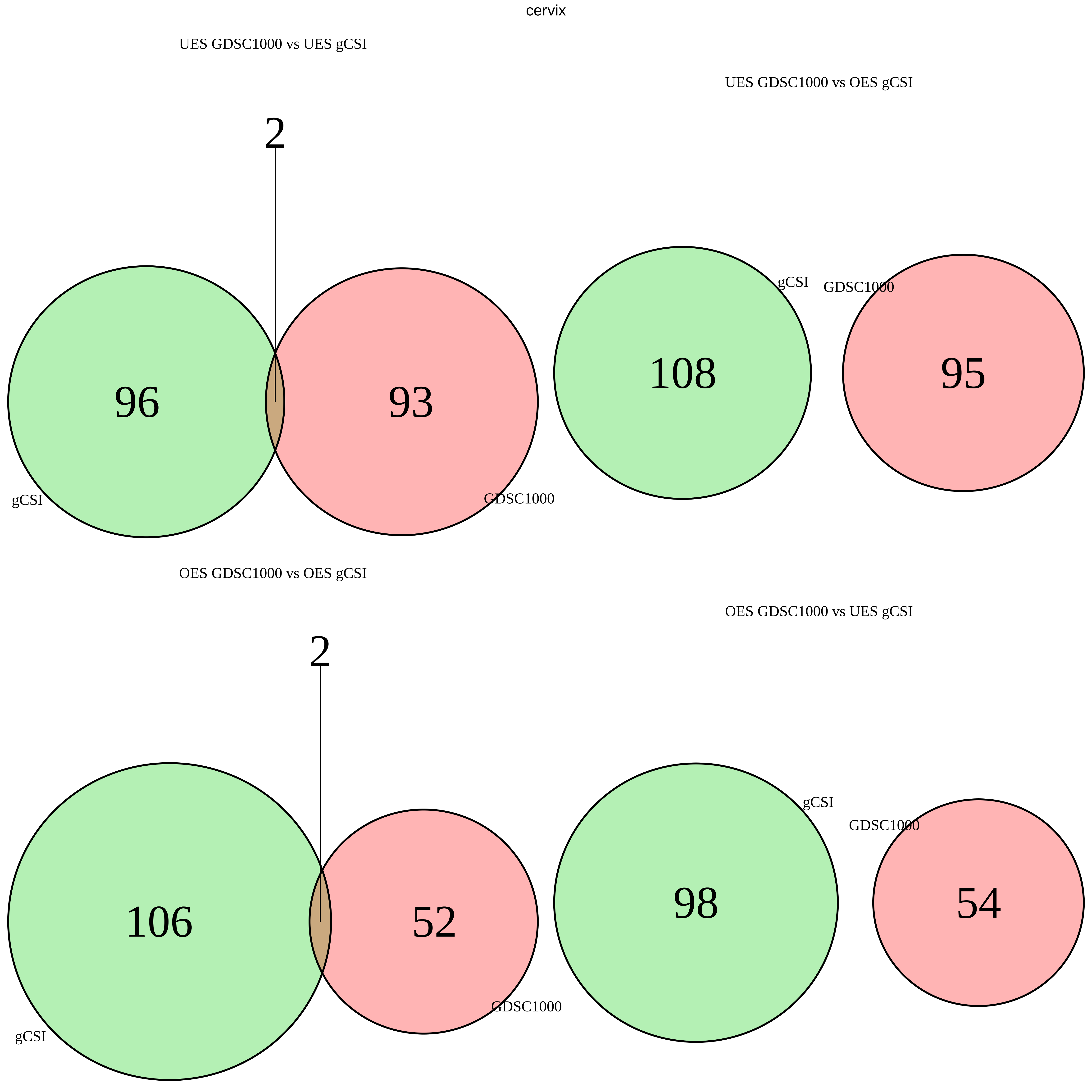
cervix
UES GDSC1000 vs UES gCSI
2
96
93
gCSI
GDSC1000
UES GDSC1000 vs OES gCSI
108
95
gCSI
GDSC1000
OES GDSC1000 vs OES gCSI
2
106
52
gCSI
GDSC1000
OES GDSC1000 vs UES gCSI
98
54
gCSI
GDSC1000

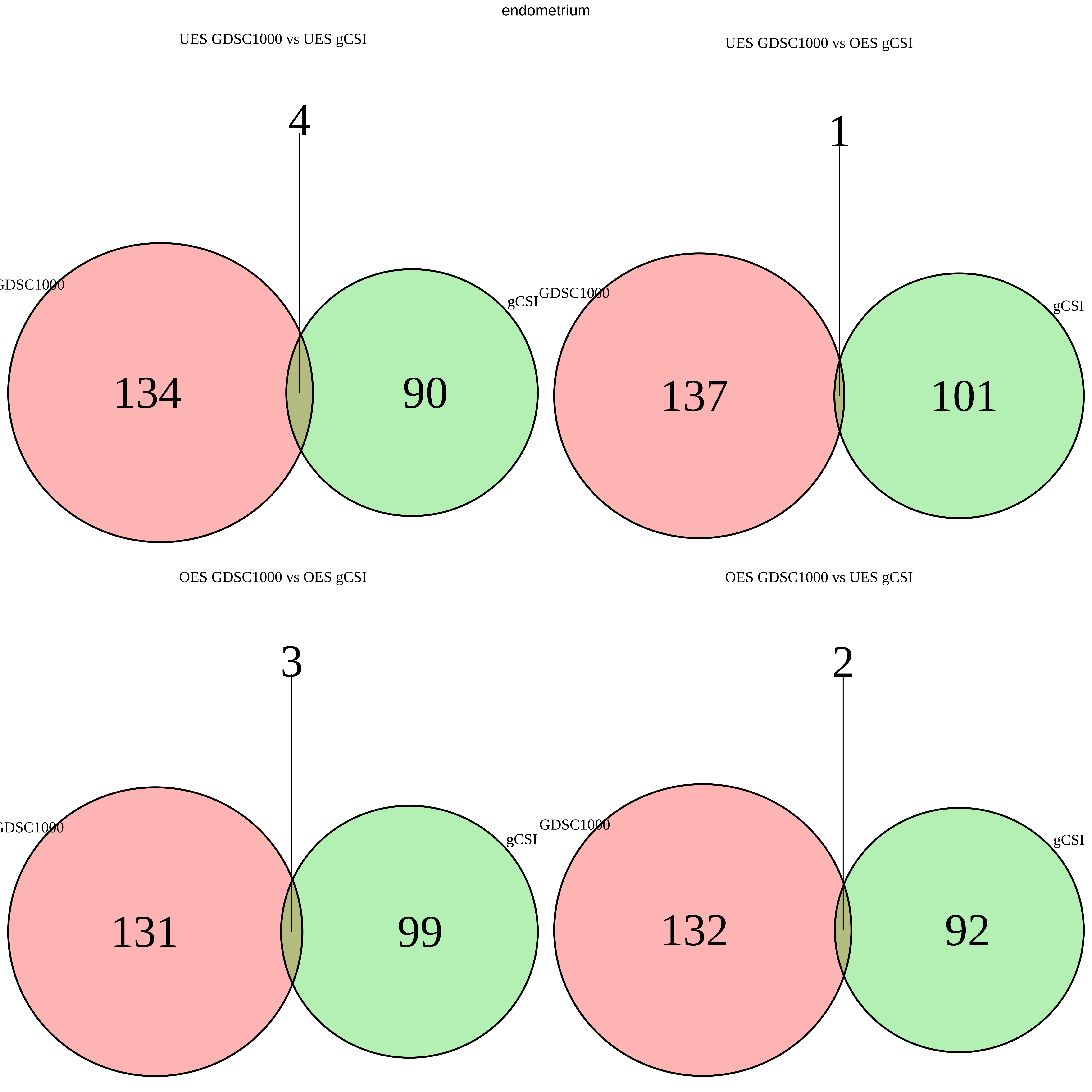
endometrium
UES GDSC1000 vs UES gCSI
4
GDSC1000
gCSI
134
90
UES GDSC1000 vs OES gCSI
1
GDSC1000
gCSI
137
101
OES GDSC1000 vs OES gCSI
3
GDSC1000
gCSI
131
99
OES GDSC1000 vs UES gCSI
2
GDSC1000
gCSI
132
92

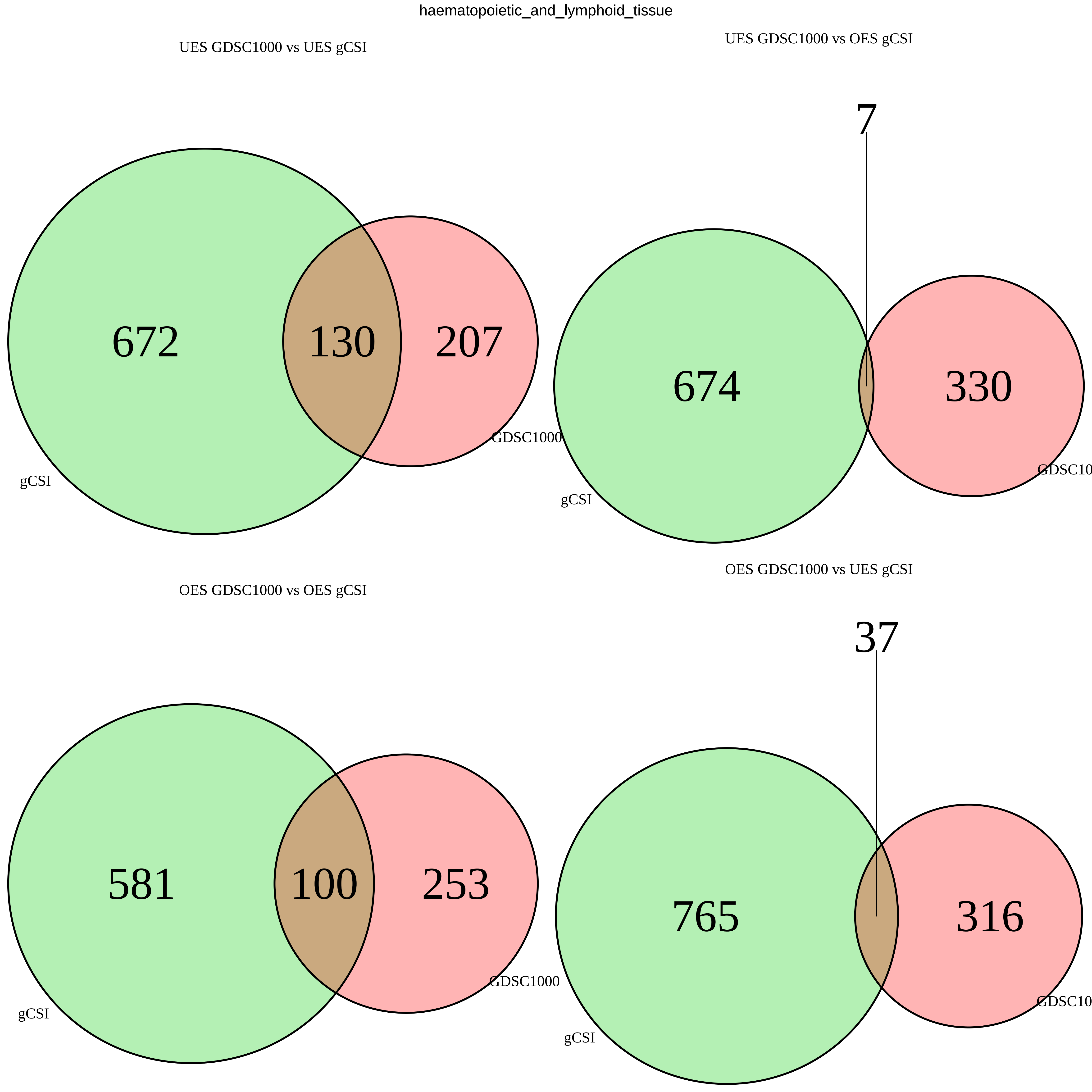
haematopoietic_and_lymphoid_tissue
UES GDSC1000 vs UES gCSI
672
130
207
GDSC1000
gCSI
UES GDSC1000 vs OES gCSI
7
674
330
GDSC10
gCSI
OES GDSC1000 vs OES gCSI
581
100
253
GDSC1000
gCSI
OES GDSC1000 vs UES gCSI
37
765
316
GDSC10
gCSI

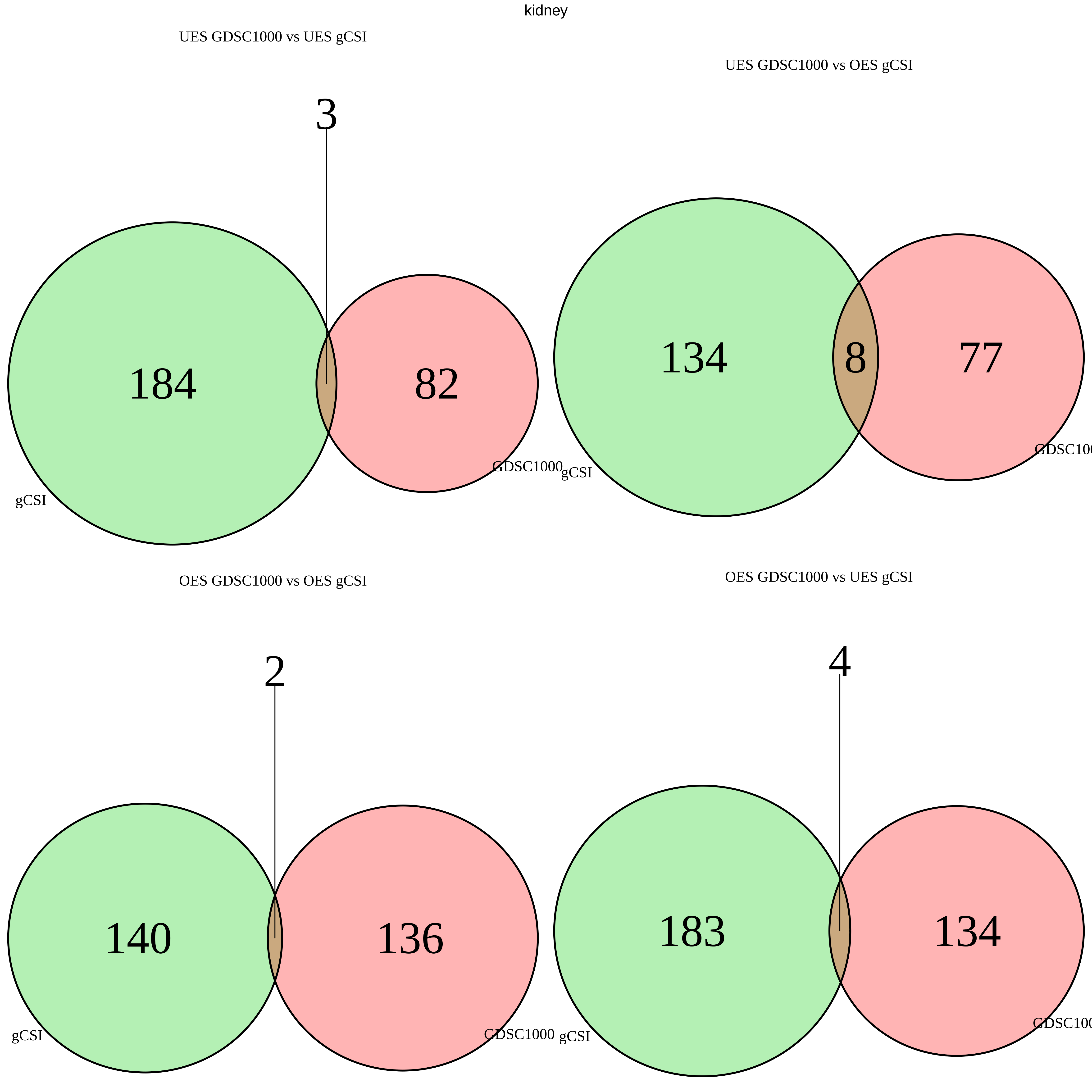
kidney
UES GDSC1000 vs UES gCSI
3
184
82
gCSI
GDSC1000
UES GDSC1000 vs OES gCSI
134
8
77
gCSI
GDSC100
OES GDSC1000 vs OES gCSI
2
140
136
gCSI
GDSC1000
OES GDSC1000 vs UES gCSI
4
183
134
gCSI
GDSC100

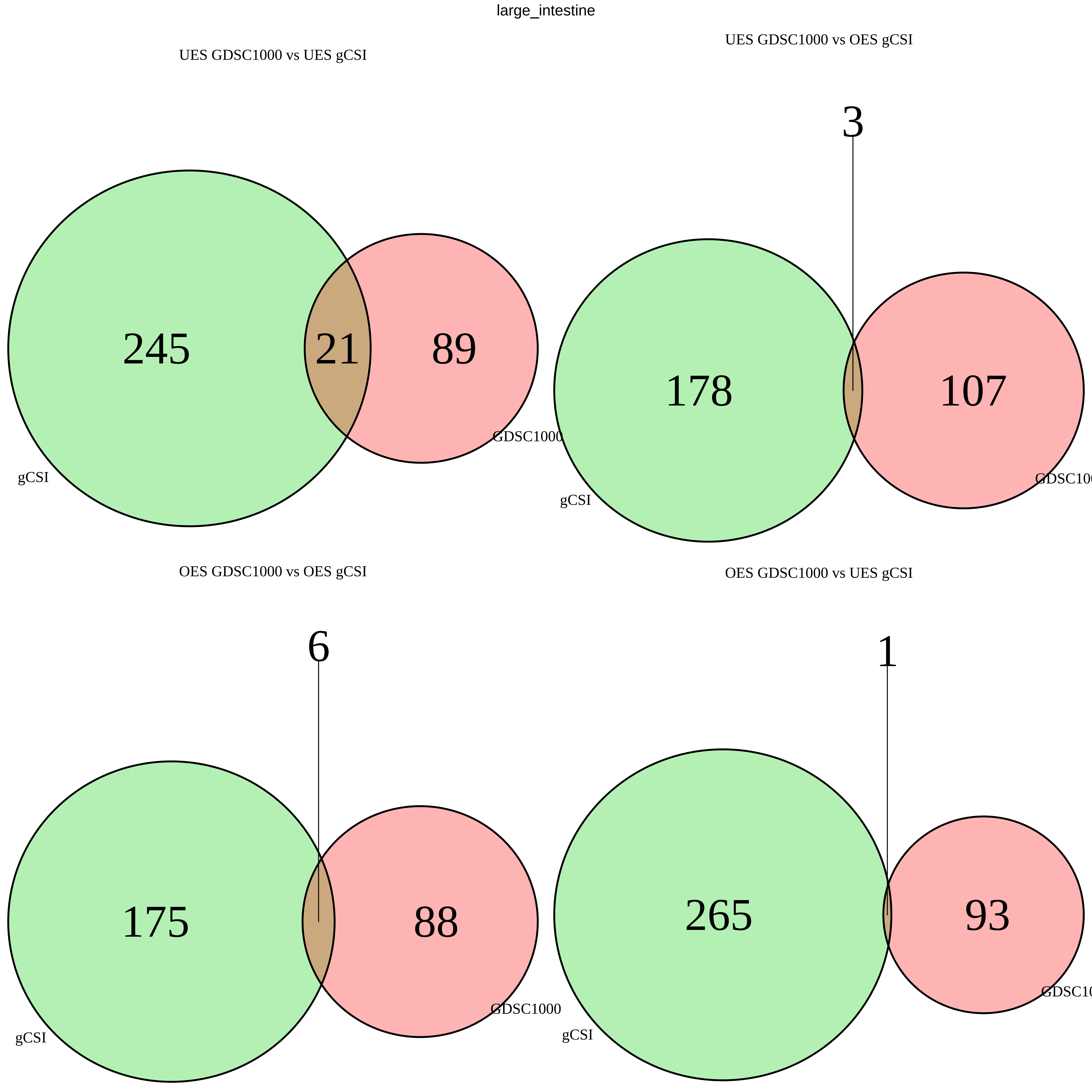

large_intestine
UES GDSC1000 vs UES gCSI
245
21
89
gCSI
GDSC1000
UES GDSC1000 vs OES gCSI
3
178
107
gCSI
OES GDSC1000 vs OES gCSI
6
175
88
gCSI
GDSC1000
OES GDSC1000 vs UES gCSI
1
265
93
gCSI

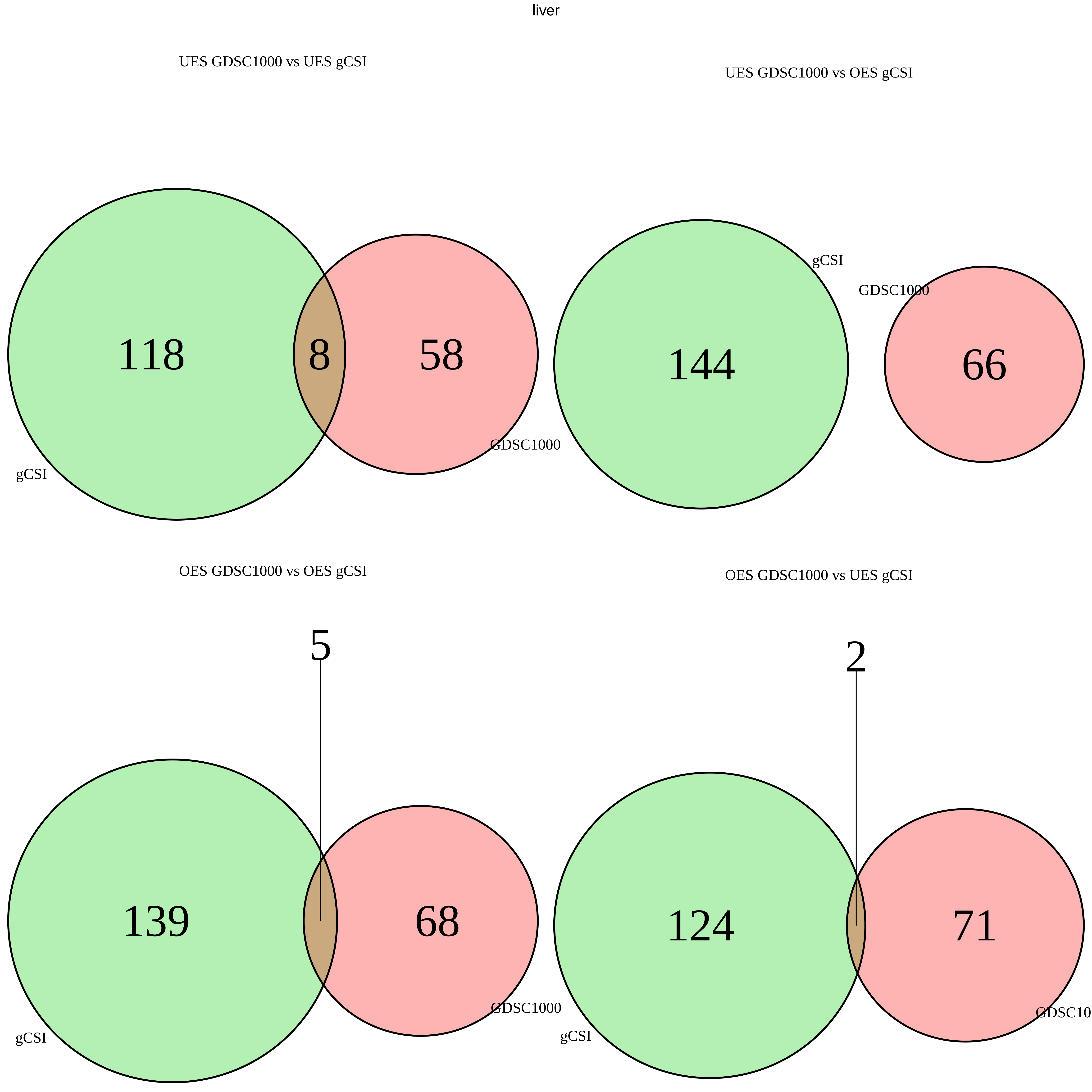
liver
UES GDSC1000 vs UES gCSI
118
8
58
gCSI
GDSC1000
UES GDSC1000 vs OES gCSI
144
66
gCSI
GDSC1000
OES GDSC1000 vs OES gCSI
5
139
68
gCSI
GDSC1000
OES GDSC1000 vs UES gCSI
2
124
71
gCSI
GDSC10

lung

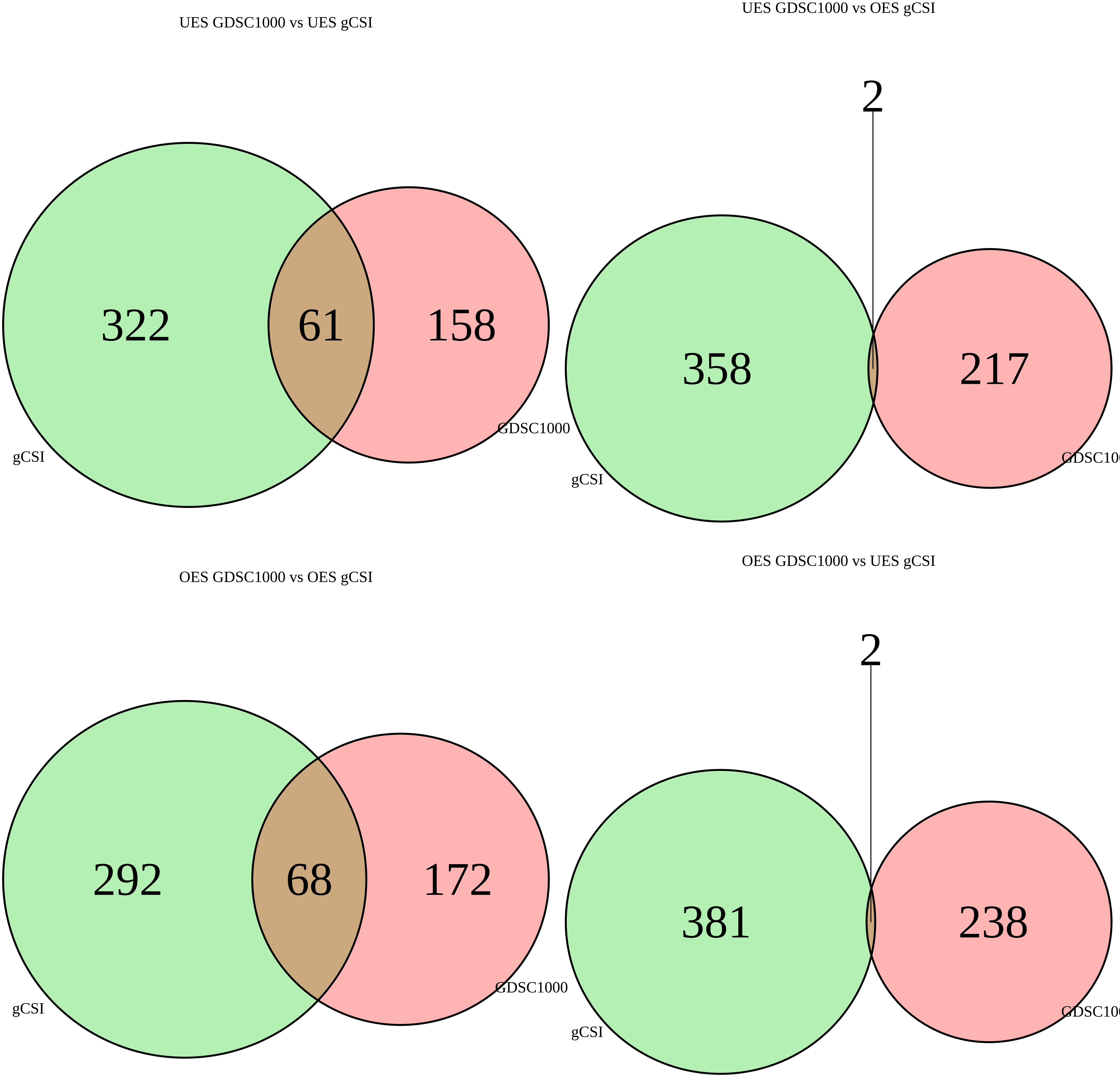
UES GDSC1000 vs UES gCSI
322
61
158
gCSI
GDSC1000
UES GDSC1000 vs OES gCSI
2
358
217
gCSI
GDSC10
OES GDSC1000 vs OES gCSI
292
68
172
gCSI
GDSC1000
OES GDSC1000 vs UES gCSI
2
381
238
gCSI
GDSC10

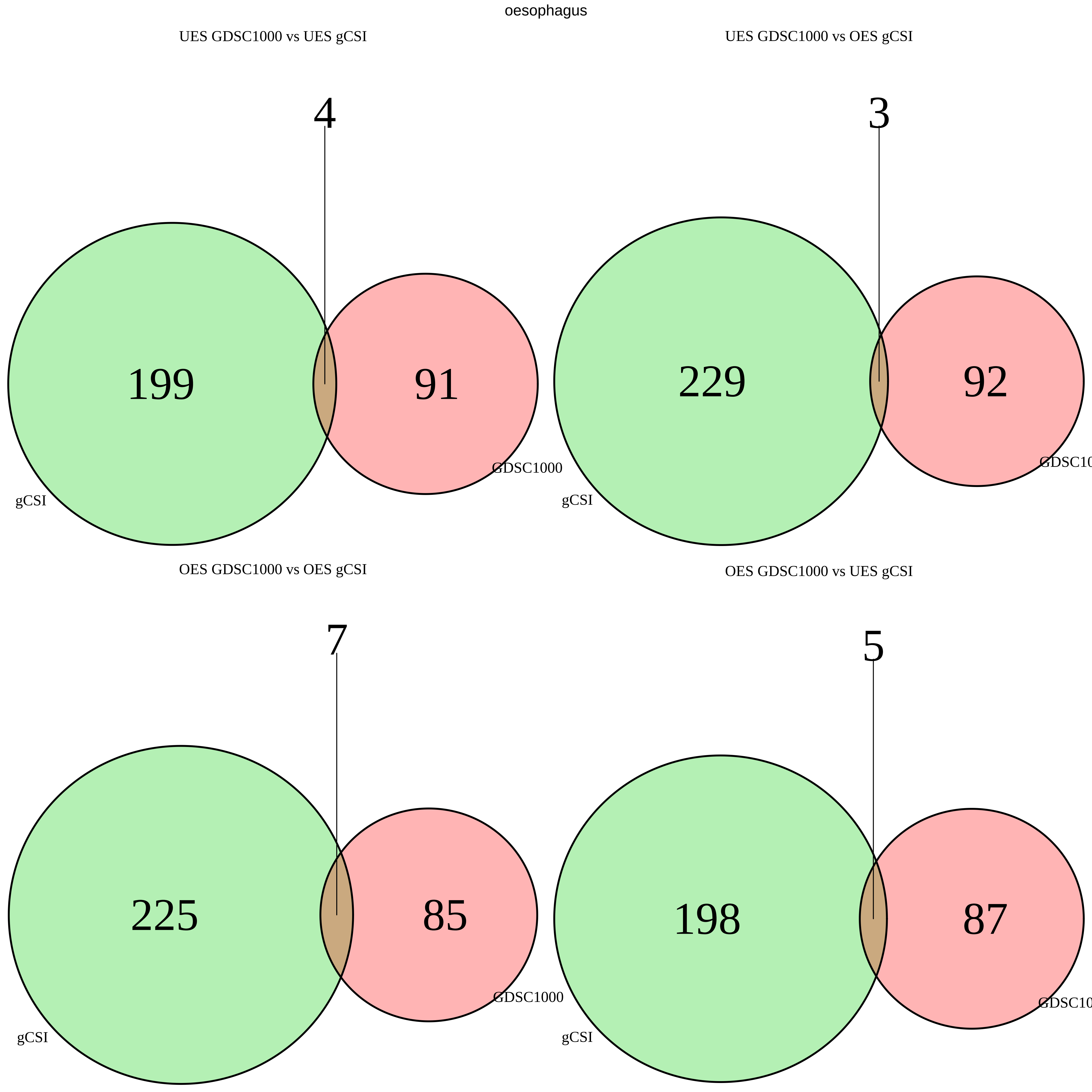
oesophagus
UES GDSC1000 vs UES gCSI
4
199
91
gCSI
GDSC1000
UES GDSC1000 vs OES gCSI
3
229
92
gCSI
GDSC10
OES GDSC1000 vs OES gCSI
7
225
85
gCSI
GDSC1000
OES GDSC1000 vs UES gCSI
5
198
87
gCSI
GDSC10

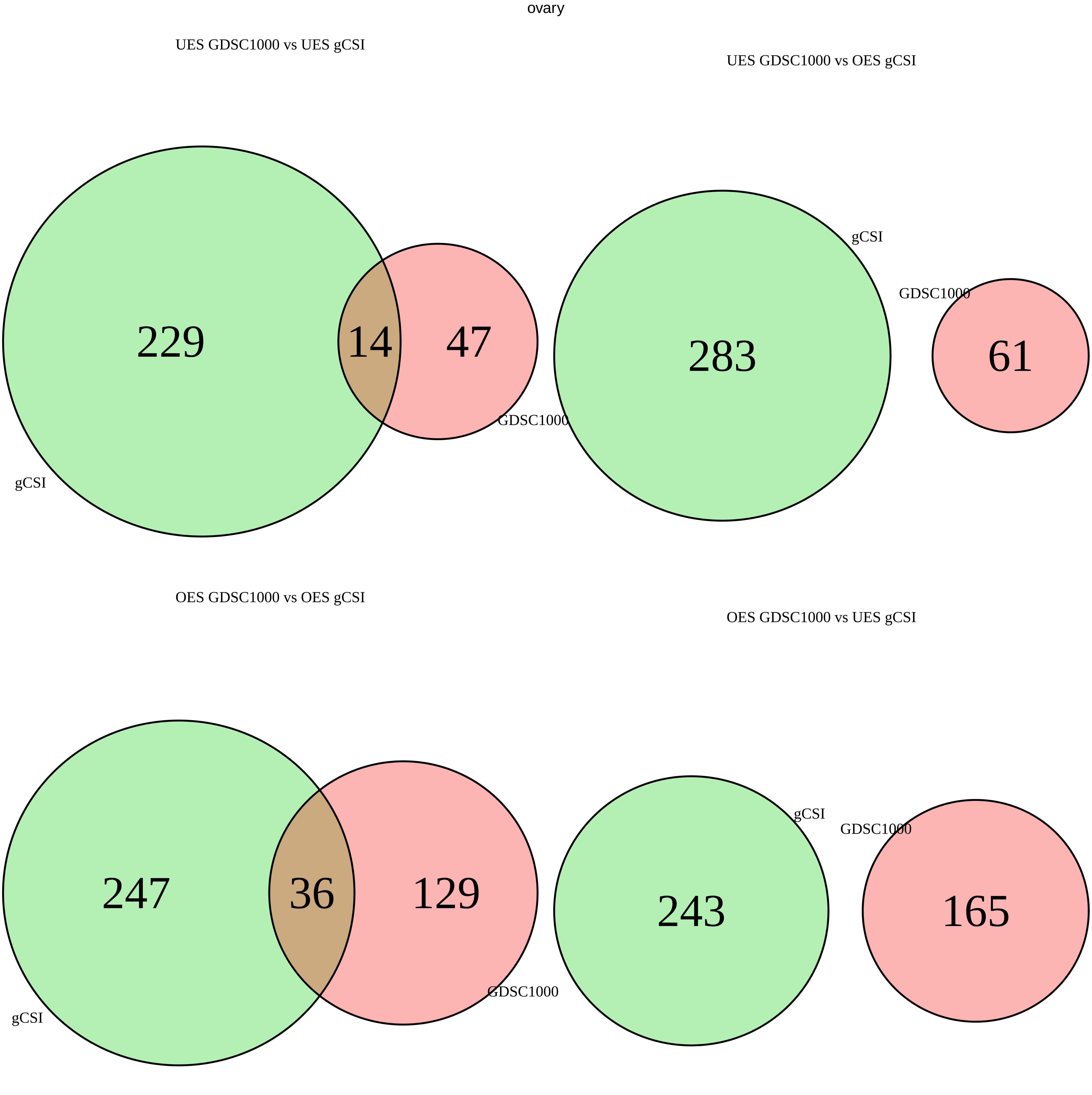
ovary
UES GDSC1000 vs UES gCSI
229
14
47
gCSI
GDSC1000
UES GDSC1000 vs OES gCSI
283
61
gCSI
GDSC1000
OES GDSC1000 vs OES gCSI
247
36
129
gCSI
GDSC1000
OES GDSC1000 vs UES gCSI
243
165
gCSI
GDSC1000

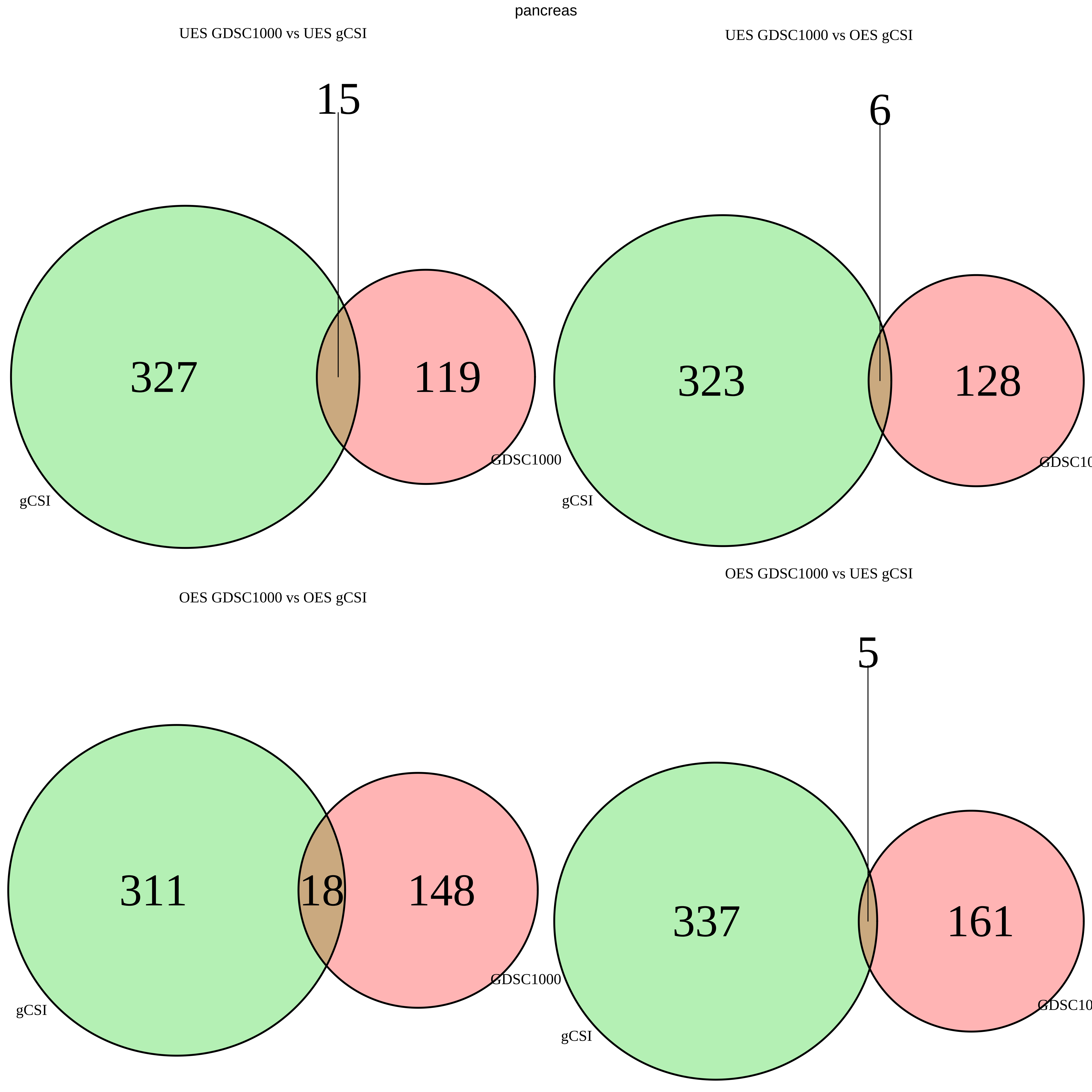
pancreas
UES GDSC1000 vs UES gCSI
15
327
119
GDSC1000
gCSI
UES GDSC1000 vs OES gCSI
6
323
128
GDSC10
gCSI
OES GDSC1000 vs OES gCSI
311
18
148
GDSC1000
gCSI
OES GDSC1000 vs UES gCSI
5
337
161
GDSC10
gCSI

skin

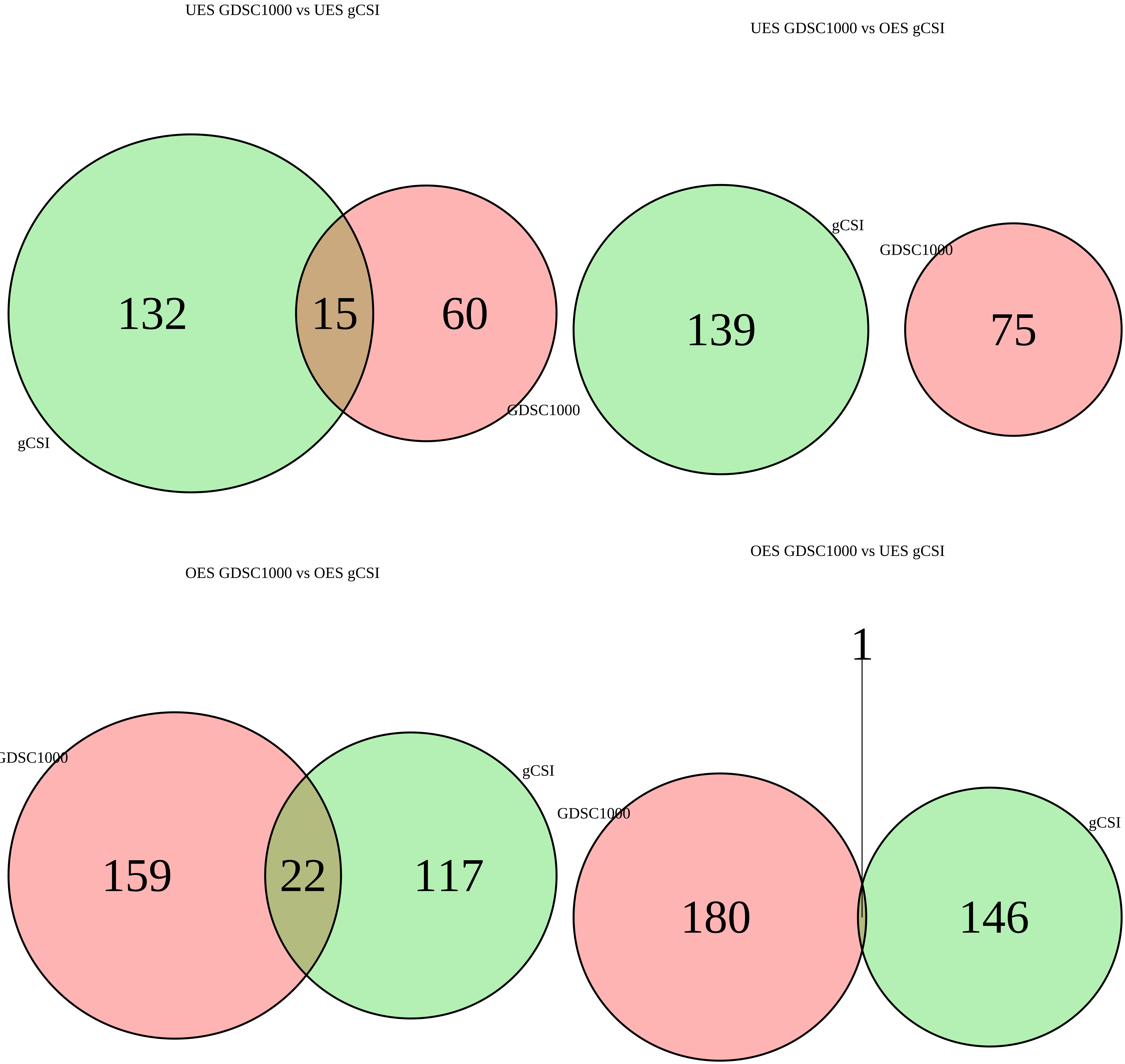

UES GDSC1000 vs UES gCSI
132
15
60
gCSI
GDSC1000
UES GDSC1000 vs OES gCSI
139
75
gCSI
GDSC1000
OES GDSC1000 vs OES gCSI
159
22
117
GDSC1000
gCSI
OES GDSC1000 vs UES gCSI
1
180
146
GDSC1000
gCSI

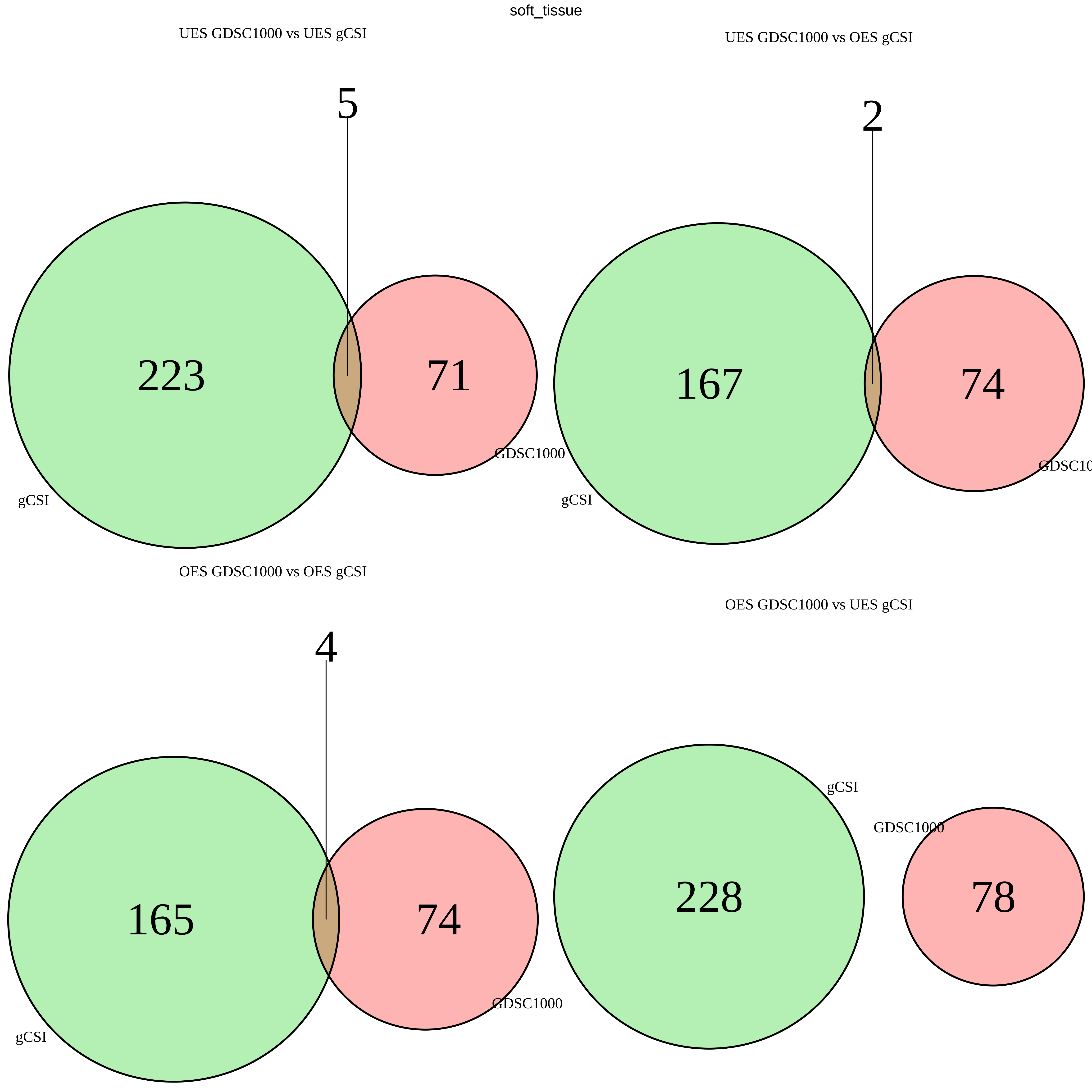
soft_tissue
UES GDSC1000 vs UES gCSI
5
223
71
GDSC1000
gCSI
UES GDSC1000 vs OES gCSI
2
167
74
GDSC10
gCSI
OES GDSC1000 vs OES gCSI
4
165
74
GDSC1000
gCSI
OES GDSC1000 vs UES gCSI
gCSI
GDSC1000
228
78

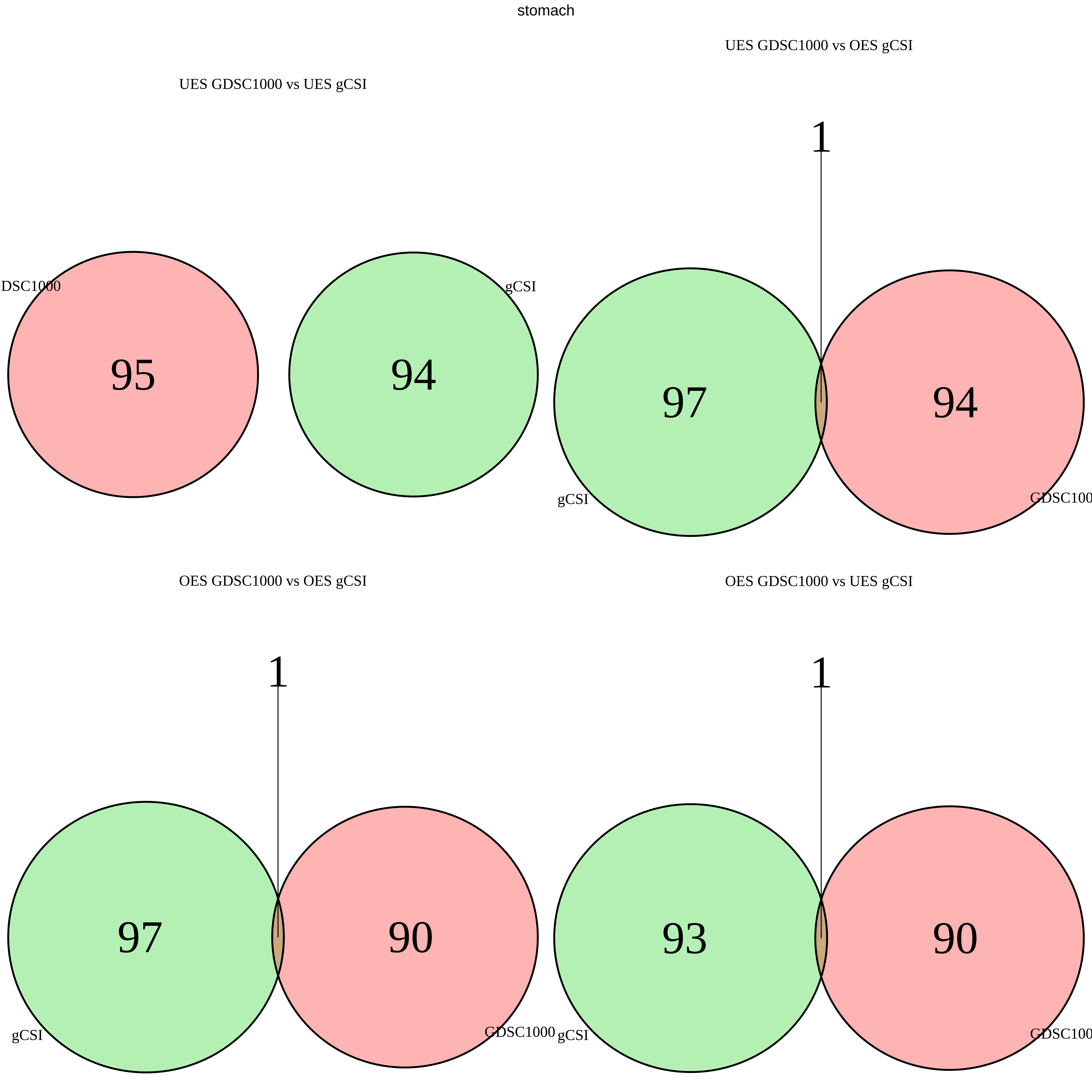
stomach
UES GDSC1000 vs UES gCSI
DSC1000
95
94
gCSI
UES GDSC1000 vs OES gCSI
1
97
94
gCSI
GDSC100
OES GDSC1000 vs OES gCSI
1
97
90
gCSI
GDSC1000
OES GDSC1000 vs UES gCSI
1
93
90
gCSI
GDSC100

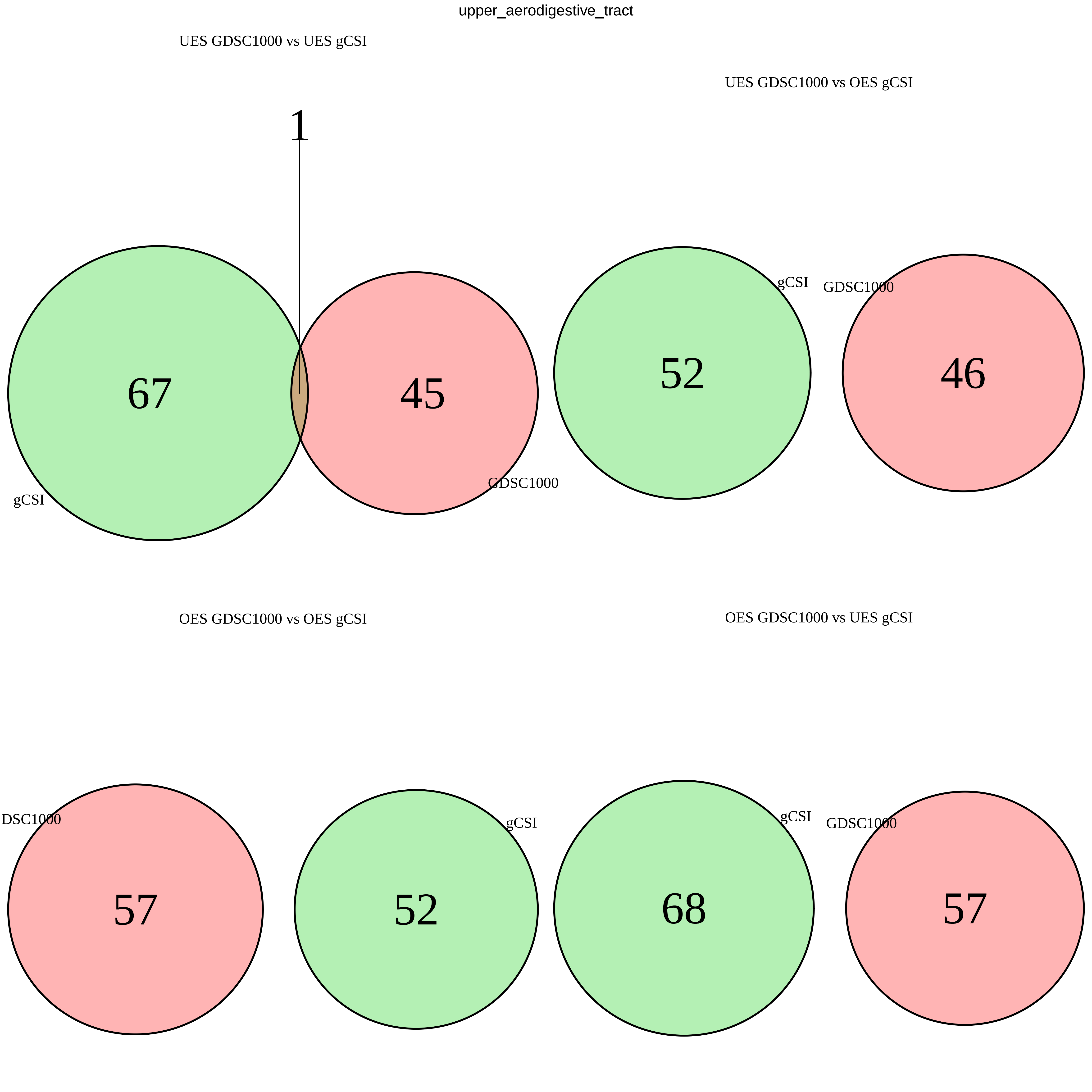
upper_aerodigestive_tract
UES GDSC1000 vs UES gCSI
1
67
45
gCSI
GDSC1000
UES GDSC1000 vs OES gCSI
52
46
gCSI
GDSC1000
OES GDSC1000 vs OES gCSI
57
52
gCSI
OES GDSC1000 vs UES gCSI
68
57
gCSI
GDSC1000

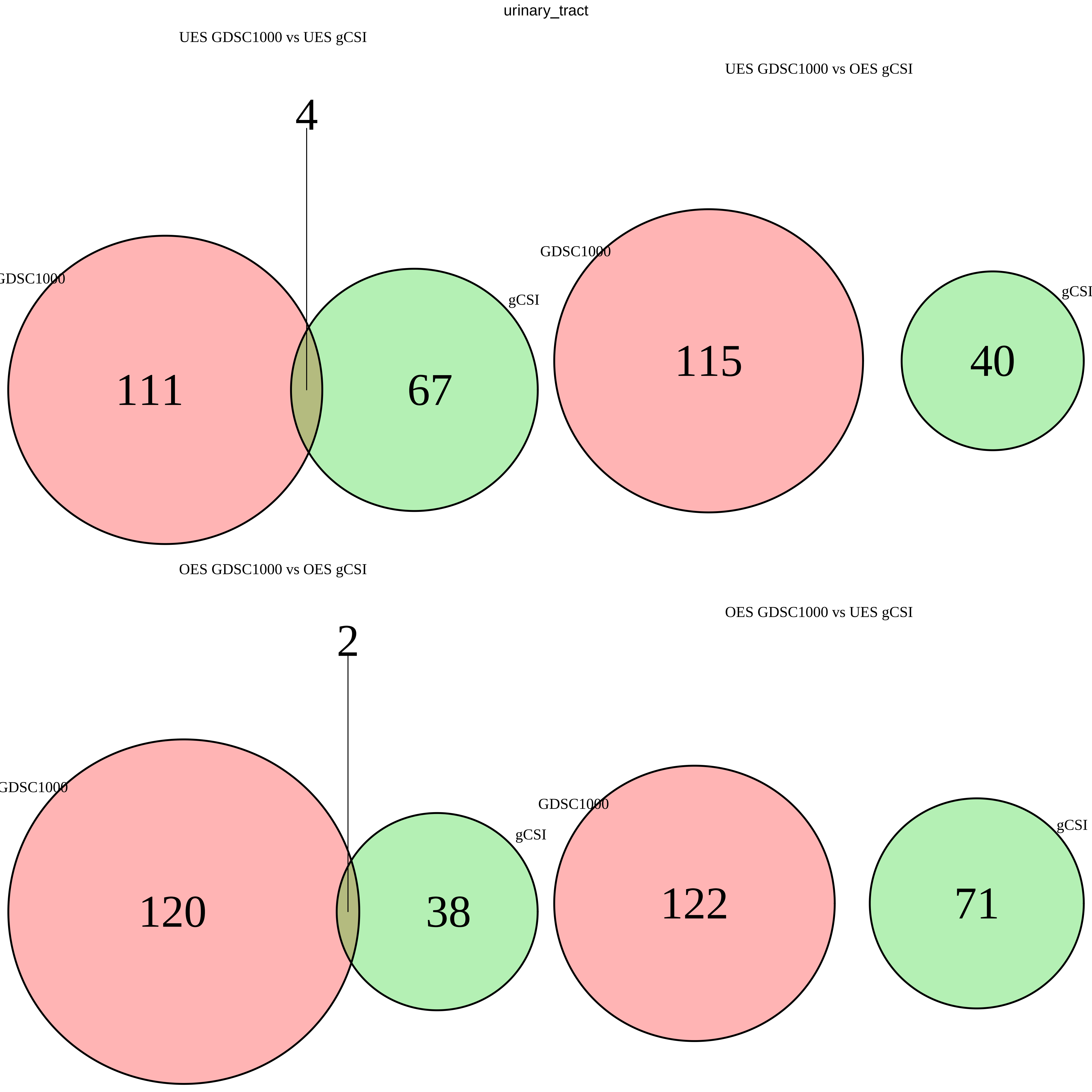
urinary_tract
UES GDSC1000 vs UES gCSI
GDSC1000
gCSI
111
4
67
UES GDSC1000 vs OES gCSI
GDSC1000
gCSI
115
40
OES GDSC1000 vs OES gCSI
GDSC1000
gCSI
120
2
38
OES GDSC1000 vs UES gCSI
GDSC1000
gCSI
122
71
