# Supplementary material for: A yeast phenomic model for the influence of Warburg metabolism on genetic buffering of doxorubicin
Source: Cancer Metab. 2019 Oct 23;7:9. doi: 10.1186/s40170-019-0201-3 (PMC6806529; doi:10.1186/s40170-019-0201-3)
Supplement: Supplementary file 11 — Additional file 11. Integration of yeast phenomic and cancer cell line pharmacogenomic data to predict human genes that modify doxorubicin toxicity in cancer cells. (A) Tables of UES and OES human genes and whether their yeast homologs were found to be deletion enhancing or deletion suppressing, respectively. (B-C) Overlap between the gCSI and GDSC1000 databases with regard to UES and OES human genes (B) across all tissues or (C) for individual tissues. Note: the intersection of UES with OES between gCSI and GDSC was used as a negative control for assessing UES and OES overlap. (D-E) Yeast phenomic doxorubicin-gene interaction profiles for homologs of human UES or OES genes, sub-classified according to interaction type (deletion enhancing or suppressing) and Warburg-dependence of the interaction, for the (D) gCSI or (E) GDSC1000 databases. Similar to Fig. 11, yeast-human homology relationships are shown to the left of heatmaps (blue - one to one; green - one to many; red - many to many). (F-I) Interactive plots for yeast-human homologs, comparing the p-value of human genes to L interaction scores for yeast counterparts in (F, G) HLD or (H, I) HLEG from (F, H) gCSI or (G, I) GDSC1000. For the standardized coefficient (‘estimate’; color gradient), a negative value (purple) indicates UES, while a positive value (orange) indicates OES. Thus, the model would predict causality for a human gene if its yeast homolog has a positive L interaction (deletion enhancing) and is colored purple (UES), or a negative L interaction (deletion suppressing) and colored orange (OES). Genes are only plotted if the human homolog was significant (p-value < 0.05). [file 40170_2019_201_MOESM11_ESM.bz2 › Additional_File11_Pharmacogenomics/D - gCSI_Heatmaps.pdf]

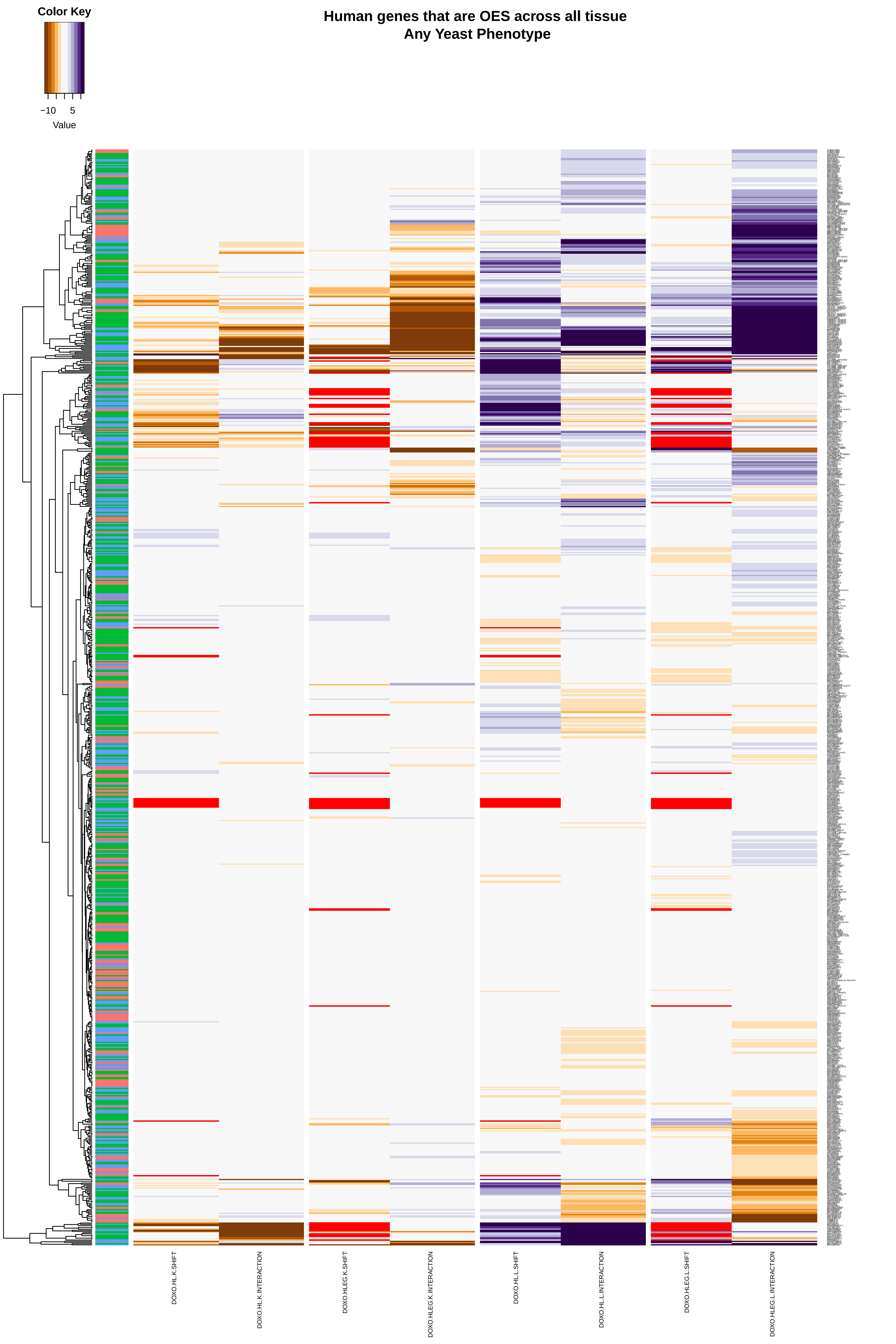

Color Key
−10
5
Value
Human genes that are OES across all tissue
Any Yeast Phenotype
DOXO.HL.K.SHIFT
DOXO.HL.K.INTERACTION
DOXO.HLEG.K.SHIFT
DOXO.HLEG.K.INTERACTION
DOXO.HL.L.SHIFT
DOXO.HL.L.INTERACTION
DOXO.HLEG.L.SHIFT
DOXO.HLEG.L.INTERACTION

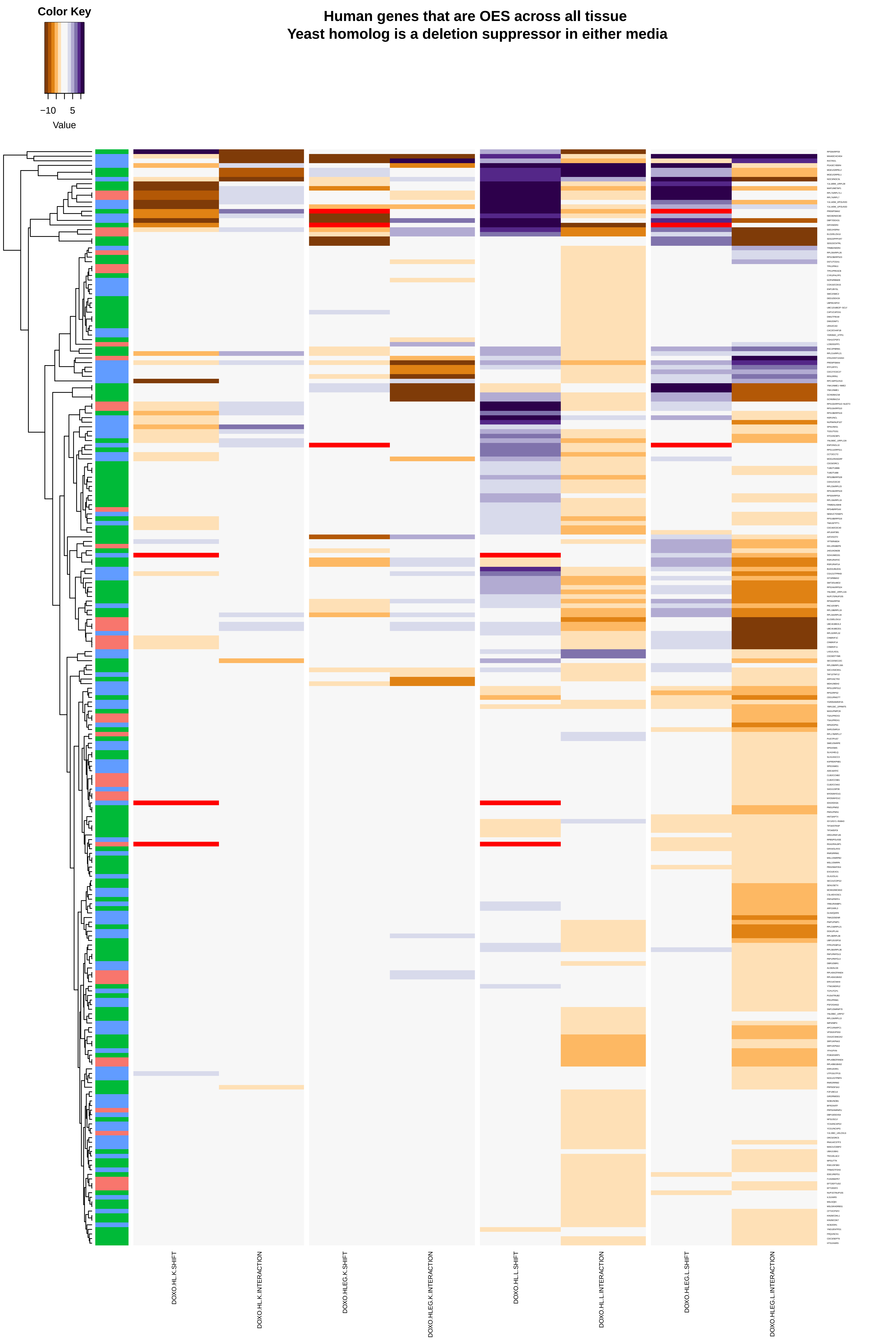
Human genes that are OES across all tissue
Yeast homolog is a deletion suppressor in either media
Color Key
−10
5
Value
DOXO.HL.K.SHIFT
DOXO.HL.K.INTERACTION
DOXO.HLEG.K.SHIFT
DOXO.HLEG.K.INTERACTION
DOXO.HL.L.SHIFT
DOXO.HL.L.INTERACTION
DOXO.HLEG.L.SHIFT
DOXO.HLEG.L.INTERACTION

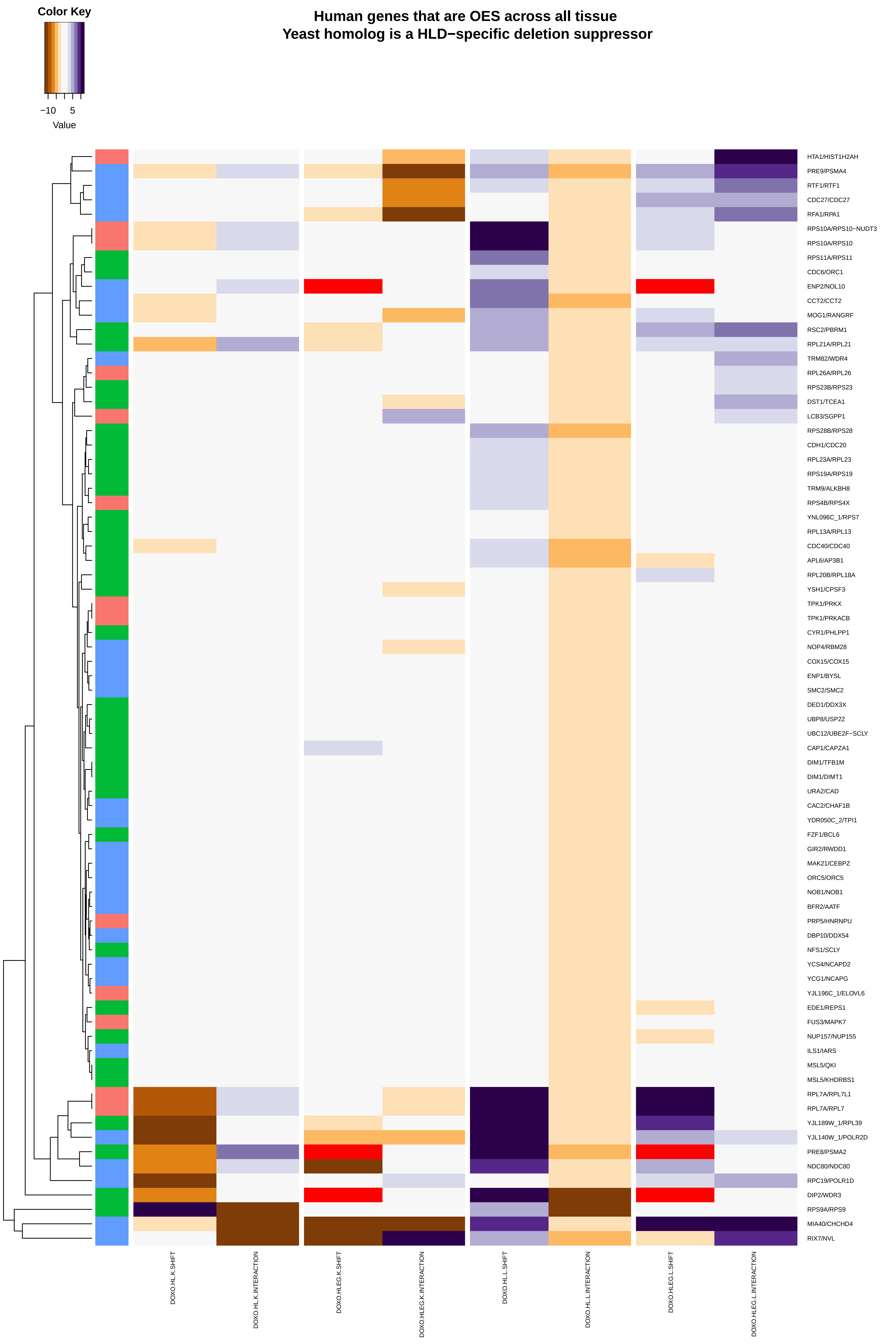
Color Key
−10
5
Value
Human genes that are OES across all tissue
Yeast homolog is a HLD−specific deletion suppressor
HTA1/HIST1H2AH
PRE9/PSMA4
RTF1/RTF1
CDC27/CDC27
RFA1/RPA1
RPS10A/RPS10−NUDT3
RPS10A/RPS10
RPS11A/RPS11
CDC6/ORC1
ENP2/NOL10
CCT2/CCT2
MOG1/RANGRF
RSC2/PBRM1
RPL21A/RPL21
TRM82/WDR4
RPL26A/RPL26
RPS23B/RPS23
DST1/TCEA1
LCB3/SGPP1
RPS28B/RPS28
CDH1/CDC20
RPL23A/RPL23
RPS19A/RPS19
TRM9/ALKBH8
RPS4B/RPS4X
YNL096C_1/RPS7
RPL13A/RPL13
CDC40/CDC40
APL6/AP3B1
RPL20B/RPL18A
YSH1/CPSF3
TPK1/PRKX
TPK1/PRKACB
CYR1/PHLPP1
NOP4/RBM28
COX15/COX15
ENP1/BYSL
SMC2/SMC2
DED1/DDX3X
UBP8/USP22
UBC12/UBE2F−SCLY
CAP1/CAPZA1
DIM1/TFB1M
DIM1/DIMT1
URA2/CAD
CAC2/CHAF1B
YDR050C_2/TPI1
FZF1/BCL6
GIR2/RWDD1
MAK21/CEBPZ
ORC5/ORC5
NOB1/NOB1
BFR2/AATF
PRP5/HNRNPU
DBP10/DDX54
NFS1/SCLY
YCS4/NCAPD2
YCG1/NCAPG
YJL196C_1/ELOVL6
EDE1/REPS1
FUS3/MAPK7
NUP157/NUP155
ILS1/IARS
MSL5/QKI
MSL5/KHDRBS1
RPL7A/RPL7L1
RPL7A/RPL7
YJL189W_1/RPL39
YJL140W_1/POLR2D
PRE8/PSMA2
NDC80/NDC80
RPC19/POLR1D
DIP2/WDR3
RPS9A/RPS9
MIA40/CHCHD4
RIX7/NVL
DOXO.HL.K.SHIFT
DOXO.HL.K.INTERACTION
DOXO.HLEG.K.SHIFT
DOXO.HLEG.K.INTERACTION
DOXO.HL.L.SHIFT
DOXO.HL.L.INTERACTION
DOXO.HLEG.L.SHIFT
DOXO.HLEG.L.INTERACTION

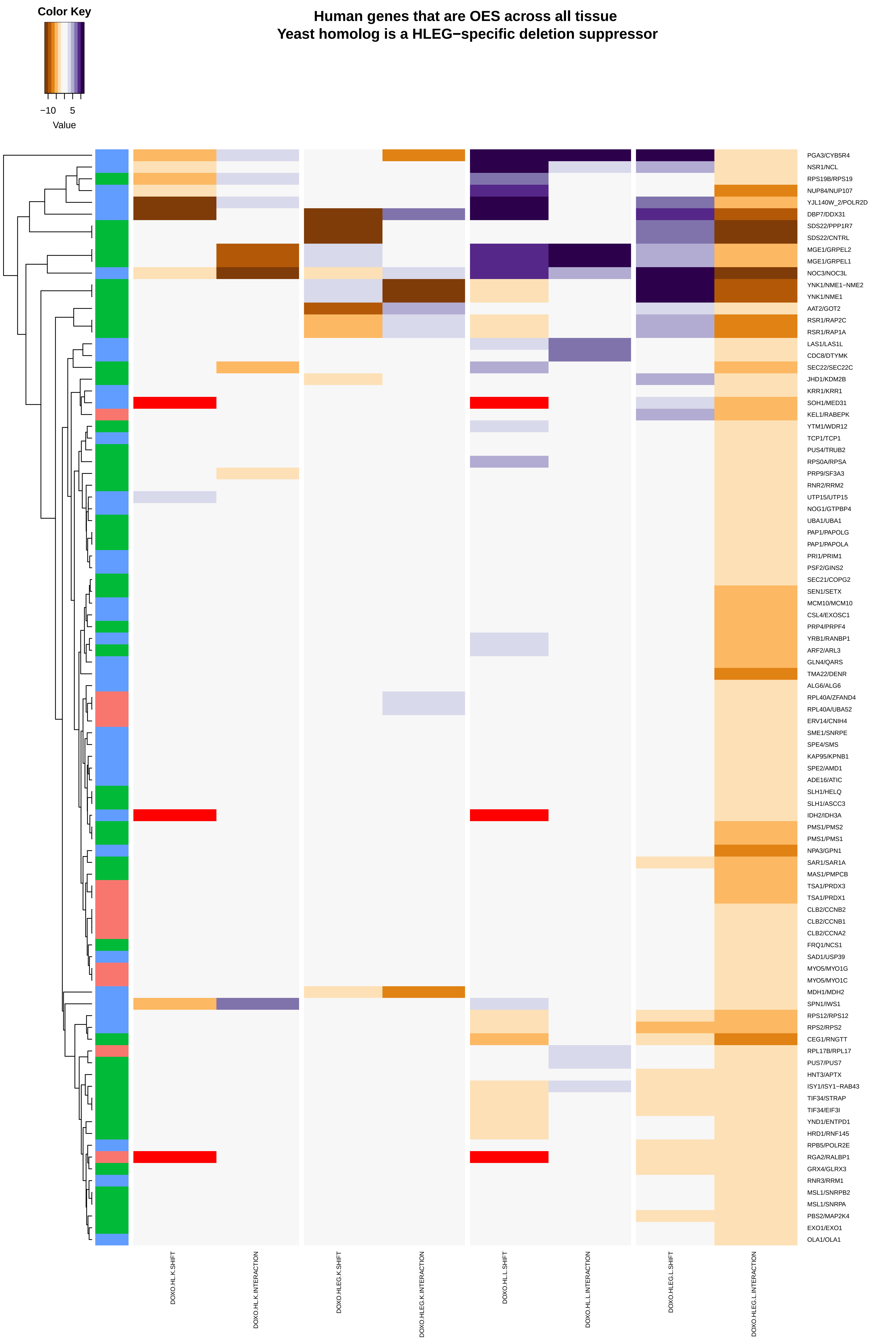

Human genes that are OES across all tissue
Yeast homolog is a HLEG−specific deletion suppressor
Color Key
−10
5
Value
PGA3/CYB5R4
NSR1/NCL
RPS19B/RPS19
NUP84/NUP107
YJL140W_2/POLR2D
DBP7/DDX31
SDS22/PPP1R7
SDS22/CNTRL
MGE1/GRPEL2
MGE1/GRPEL1
NOC3/NOC3L
YNK1/NME1−NME2
YNK1/NME1
AAT2/GOT2
RSR1/RAP2C
RSR1/RAP1A
LAS1/LAS1L
CDC8/DTYMK
SEC22/SEC22C
JHD1/KDM2B
KRR1/KRR1
SOH1/MED31
KEL1/RABEPK
YTM1/WDR12
TCP1/TCP1
PUS4/TRUB2
RPS0A/RPSA
PRP9/SF3A3
RNR2/RRM2
UTP15/UTP15
NOG1/GTPBP4
UBA1/UBA1
PAP1/PAPOLG
PAP1/PAPOLA
PRI1/PRIM1
PSF2/GINS2
SEC21/COPG2
SEN1/SETX
MCM10/MCM10
CSL4/EXOSC1
PRP4/PRPF4
YRB1/RANBP1
ARF2/ARL3
GLN4/QARS
TMA22/DENR
ALG6/ALG6
RPL40A/ZFAND4
RPL40A/UBA52
ERV14/CNIH4
SME1/SNRPE
SPE4/SMS
KAP95/KPNB1
SPE2/AMD1
ADE16/ATIC
SLH1/HELQ
SLH1/ASCC3
IDH2/IDH3A
PMS1/PMS2
PMS1/PMS1
NPA3/GPN1
SAR1/SAR1A
MAS1/PMPCB
TSA1/PRDX3
TSA1/PRDX1
CLB2/CCNB2
CLB2/CCNB1
CLB2/CCNA2
FRQ1/NCS1
SAD1/USP39
MYO5/MYO1G
MYO5/MYO1C
MDH1/MDH2
SPN1/IWS1
RPS12/RPS12
RPS2/RPS2
CEG1/RNGTT
RPL17B/RPL17
PUS7/PUS7
HNT3/APTX
ISY1/ISY1−RAB43
TIF34/STRAP
TIF34/EIF3I
YND1/ENTPD1
HRD1/RNF145
RPB5/POLR2E
RGA2/RALBP1
GRX4/GLRX3
RNR3/RRM1
MSL1/SNRPB2
MSL1/SNRPA
PBS2/MAP2K4
EXO1/EXO1
OLA1/OLA1
DOXO.HL.K.SHIFT
DOXO.HL.K.INTERACTION
DOXO.HLEG.K.SHIFT
DOXO.HLEG.K.INTERACTION
DOXO.HL.L.SHIFT
DOXO.HL.L.INTERACTION
DOXO.HLEG.L.SHIFT
DOXO.HLEG.L.INTERACTION

Human genes that are OES across all tissue
Yeast homolog is a deletion suppressor in both media

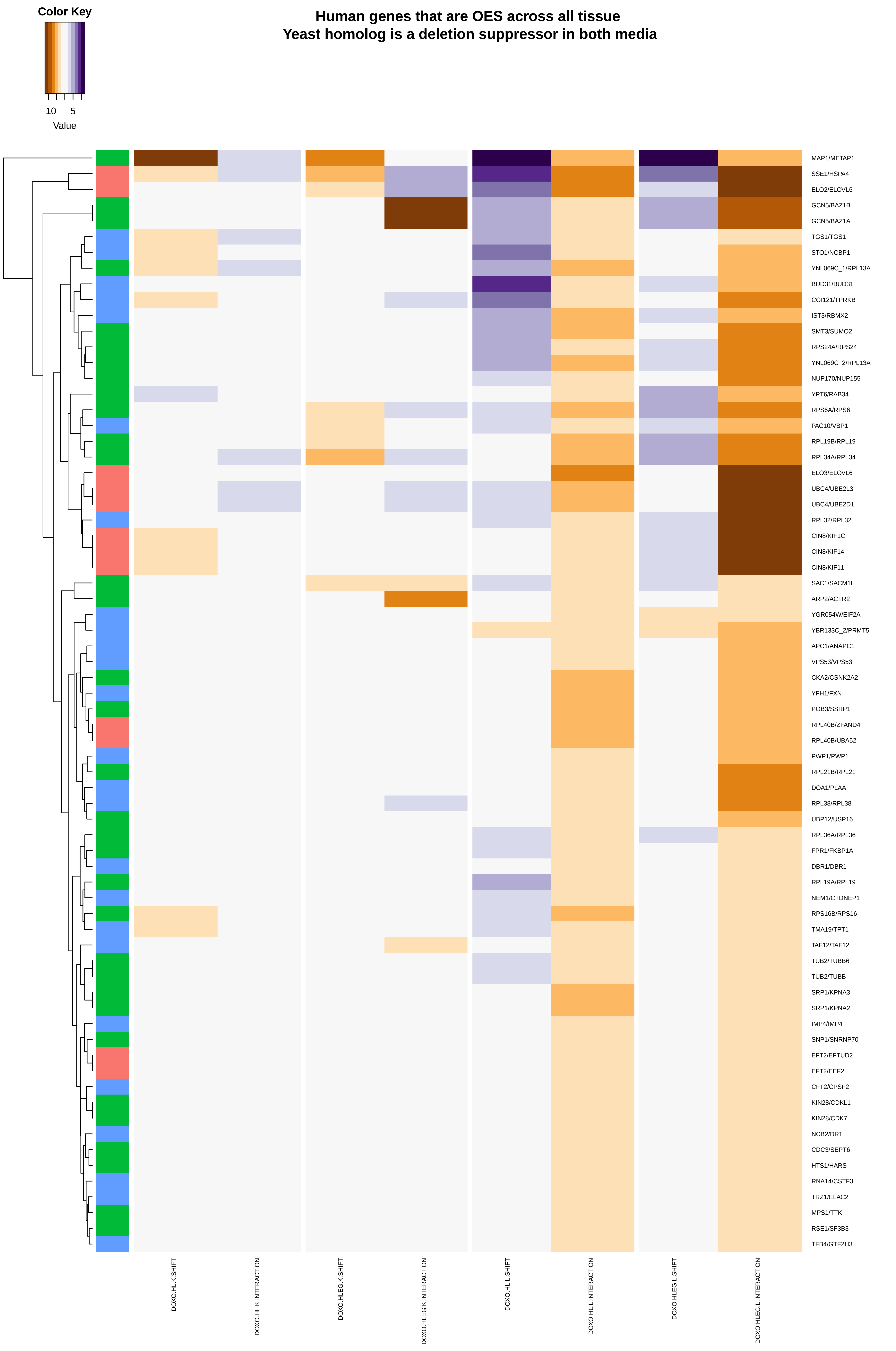

Human genes that are UES across all tissue
Any Yeast Phenotype

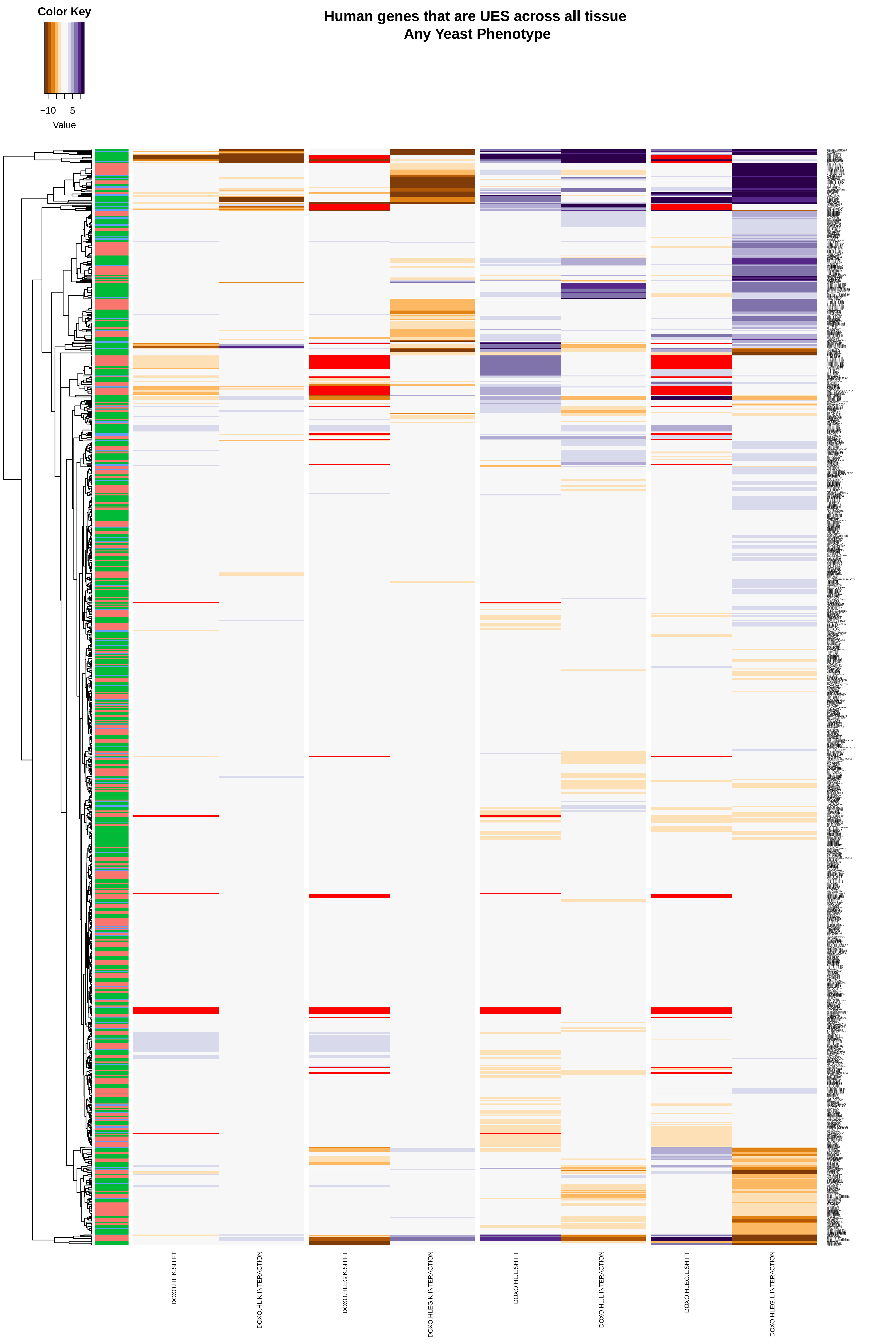

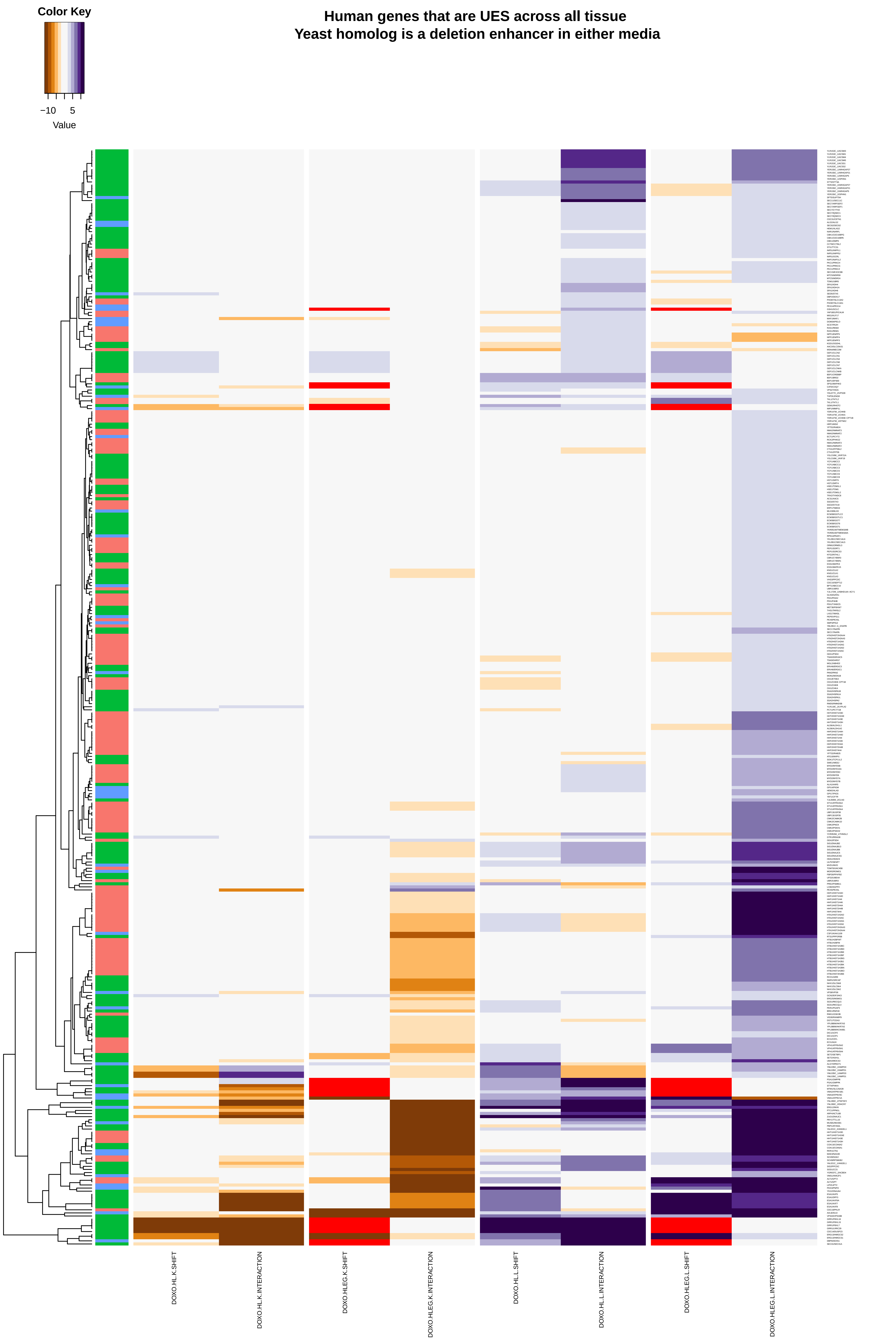
Human genes that are UES across all tissue
Yeast homolog is a deletion enhancer in either media
Color Key
−10
5
Value
DOXO.HL.K.SHIFT
DOXO.HL.K.INTERACTION
DOXO.HLEG.K.SHIFT
DOXO.HLEG.K.INTERACTION
DOXO.HL.L.SHIFT
DOXO.HL.L.INTERACTION
DOXO.HLEG.L.SHIFT
DOXO.HLEG.L.INTERACTION

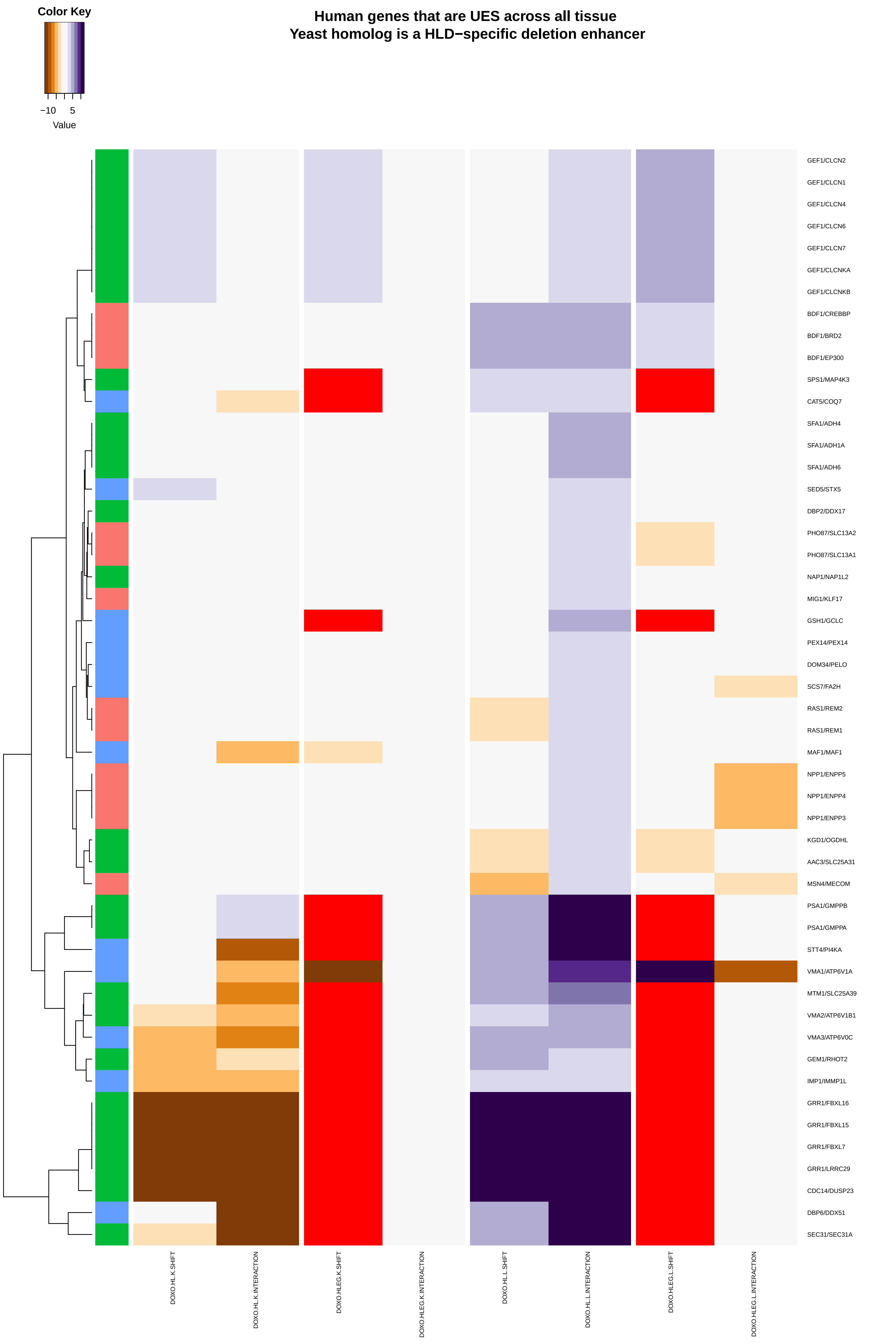
Color Key
−10
5
Value
Human genes that are UES across all tissue
Yeast homolog is a HLD−specific deletion enhancer
GEF1/CLCN2
GEF1/CLCN1
GEF1/CLCN4
GEF1/CLCN6
GEF1/CLCN7
GEF1/CLCNKA
GEF1/CLCNKB
BDF1/CREBBP
BDF1/BRD2
BDF1/EP300
SPS1/MAP4K3
CAT5/COQ7
SFA1/ADH4
SFA1/ADH1A
SFA1/ADH6
SED5/STX5
DBP2/DDX17
PHO87/SLC13A2
PHO87/SLC13A1
NAP1/NAP1L2
MIG1/KLF17
GSH1/GCLC
PEX14/PEX14
DOM34/PELO
SCS7/FA2H
RAS1/REM2
RAS1/REM1
MAF1/MAF1
NPP1/ENPP5
NPP1/ENPP4
NPP1/ENPP3
KGD1/OGDHL
AAC3/SLC25A31
MSN4/MECOM
PSA1/GMPPB
PSA1/GMPPA
STT4/PI4KA
VMA1/ATP6V1A
MTM1/SLC25A39
VMA2/ATP6V1B1
VMA3/ATP6V0C
GEM1/RHOT2
IMP1/IMMP1L
GRR1/FBXL16
GRR1/FBXL15
GRR1/FBXL7
GRR1/LRRC29
CDC14/DUSP23
DBP6/DDX51
SEC31/SEC31A
DOXO.HL.K.SHIFT
DOXO.HL.K.INTERACTION
DOXO.HLEG.K.SHIFT
DOXO.HLEG.K.INTERACTION
DOXO.HL.L.SHIFT
DOXO.HL.L.INTERACTION
DOXO.HLEG.L.SHIFT
DOXO.HLEG.L.INTERACTION

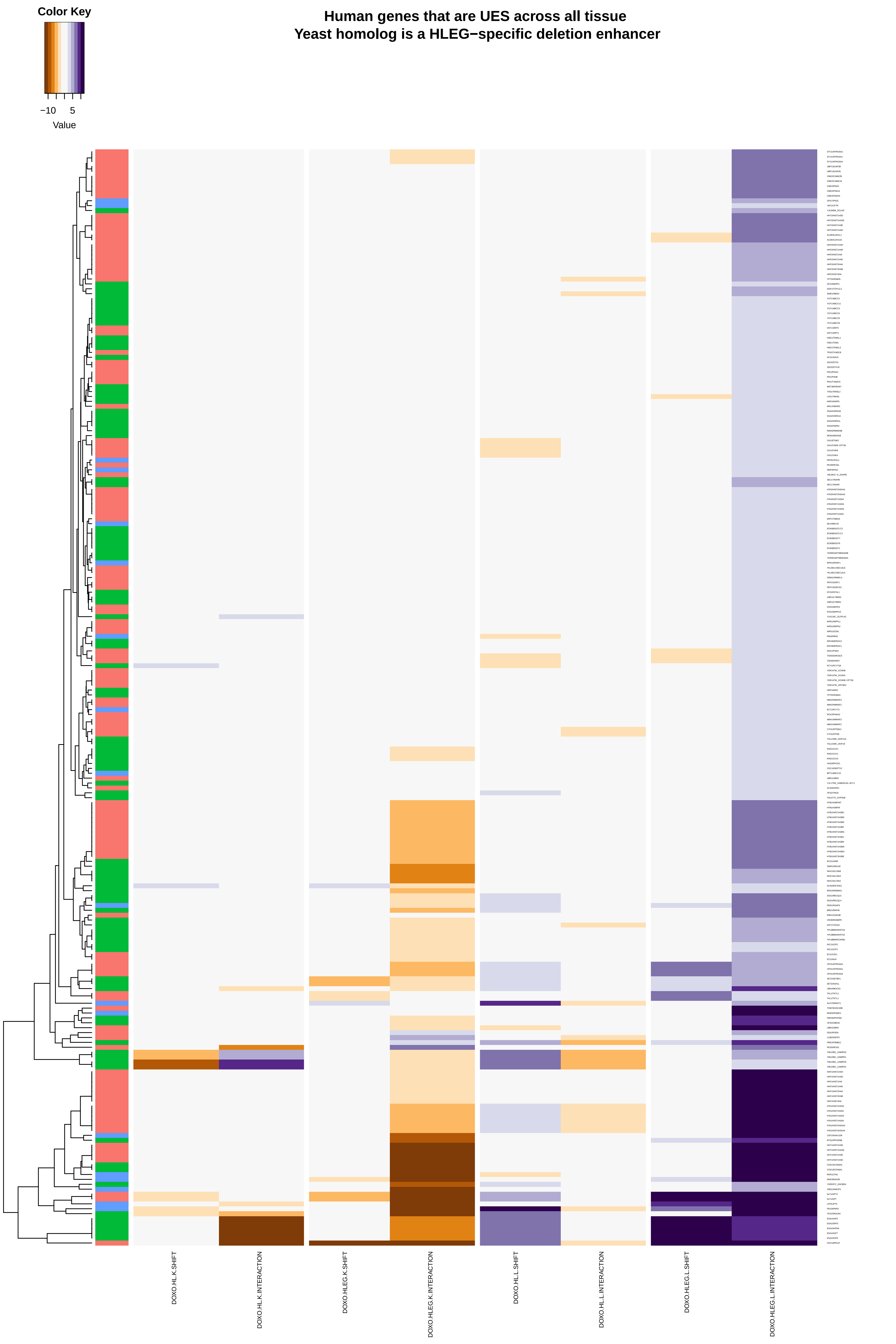
Human genes that are UES across all tissue
Yeast homolog is a HLEG−specific deletion enhancer
Color Key
−10
5
Value
DOXO.HL.K.SHIFT
DOXO.HL.K.INTERACTION
DOXO.HLEG.K.SHIFT
DOXO.HLEG.K.INTERACTION
DOXO.HL.L.SHIFT
DOXO.HL.L.INTERACTION
DOXO.HLEG.L.SHIFT
DOXO.HLEG.L.INTERACTION

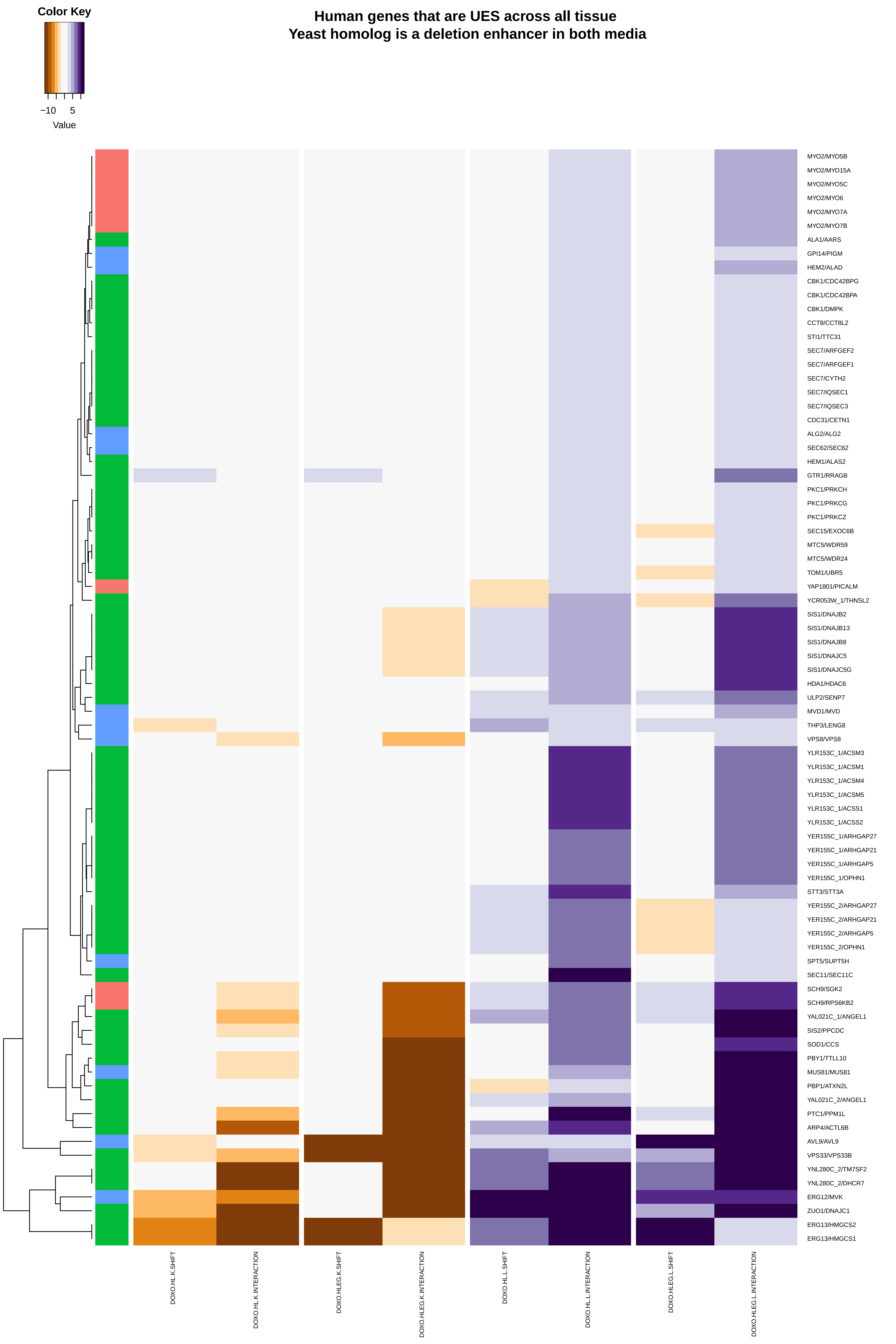

Human genes that are UES across all tissue
Yeast homolog is a deletion enhancer in both media
Color Key
−10
5
Value
MYO2/MYO5B
MYO2/MYO15A
MYO2/MYO5C
MYO2/MYO6
MYO2/MYO7A
MYO2/MYO7B
ALA1/AARS
GPI14/PIGM
HEM2/ALAD
CBK1/CDC42BPG
CBK1/CDC42BPA
CBK1/DMPK
CCT8/CCT8L2
STI1/TTC31
SEC7/ARFGEF2
SEC7/ARFGEF1
SEC7/CYTH2
SEC7/IQSEC1
SEC7/IQSEC3
CDC31/CETN1
ALG2/ALG2
SEC62/SEC62
HEM1/ALAS2
GTR1/RRAGB
PKC1/PRKCH
PKC1/PRKCG
PKC1/PRKCZ
SEC15/EXOC6B
MTC5/WDR59
MTC5/WDR24
TOM1/UBR5
YAP1801/PICALM
YCR053W_1/THNSL2
SIS1/DNAJB2
SIS1/DNAJB13
SIS1/DNAJB8
SIS1/DNAJC5
SIS1/DNAJC5G
HDA1/HDAC6
ULP2/SENP7
MVD1/MVD
THP3/LENG8
VPS8/VPS8
YLR153C_1/ACSM3
YLR153C_1/ACSM1
YLR153C_1/ACSM4
YLR153C_1/ACSM5
YLR153C_1/ACSS1
YLR153C_1/ACSS2
YER155C_1/ARHGAP27
YER155C_1/ARHGAP21
YER155C_1/ARHGAP5
YER155C_1/OPHN1
STT3/STT3A
YER155C_2/ARHGAP27
YER155C_2/ARHGAP21
YER155C_2/ARHGAP5
YER155C_2/OPHN1
SPT5/SUPT5H
SEC11/SEC11C
SCH9/SGK2
SCH9/RPS6KB2
YAL021C_1/ANGEL1
SIS2/PPCDC
SOD1/CCS
PBY1/TTLL10
MUS81/MUS81
PBP1/ATXN2L
YAL021C_2/ANGEL1
PTC1/PPM1L
ARP4/ACTL6B
AVL9/AVL9
VPS33/VPS33B
YNL280C_2/TM7SF2
YNL280C_2/DHCR7
ERG12/MVK
ZUO1/DNAJC1
ERG13/HMGCS2
ERG13/HMGCS1
DOXO.HL.K.SHIFT
DOXO.HL.K.INTERACTION
DOXO.HLEG.K.SHIFT
DOXO.HLEG.K.INTERACTION
DOXO.HL.L.SHIFT
DOXO.HL.L.INTERACTION
DOXO.HLEG.L.SHIFT
DOXO.HLEG.L.INTERACTION
