# Supplementary material for: A yeast phenomic model for the influence of Warburg metabolism on genetic buffering of doxorubicin
Source: Cancer Metab. 2019 Oct 23;7:9. doi: 10.1186/s40170-019-0201-3 (PMC6806529; doi:10.1186/s40170-019-0201-3)
Supplement: Supplementary file 9 — Additional file 9. GO term-specific heatmaps for REMc/GTF-enriched clusters. GO term-specific heatmaps for significant GO process terms were generated as described in methods and Figs. 3 and 4. Any related child terms are presented in subsequent pages of the parent file name. GO terms with more than 100 children, with 2 or fewer genes annotated to the term, or a file size over 300KB are not shown. All heatmaps are generated with the same layout (see Figs. 3 and 4). [file cytoplasmic_translation.pdf › Additional_File9_GOTermHeatmaps/Additional_File9_GOTermHeatmap/cytoplasmic_translation.pdf]

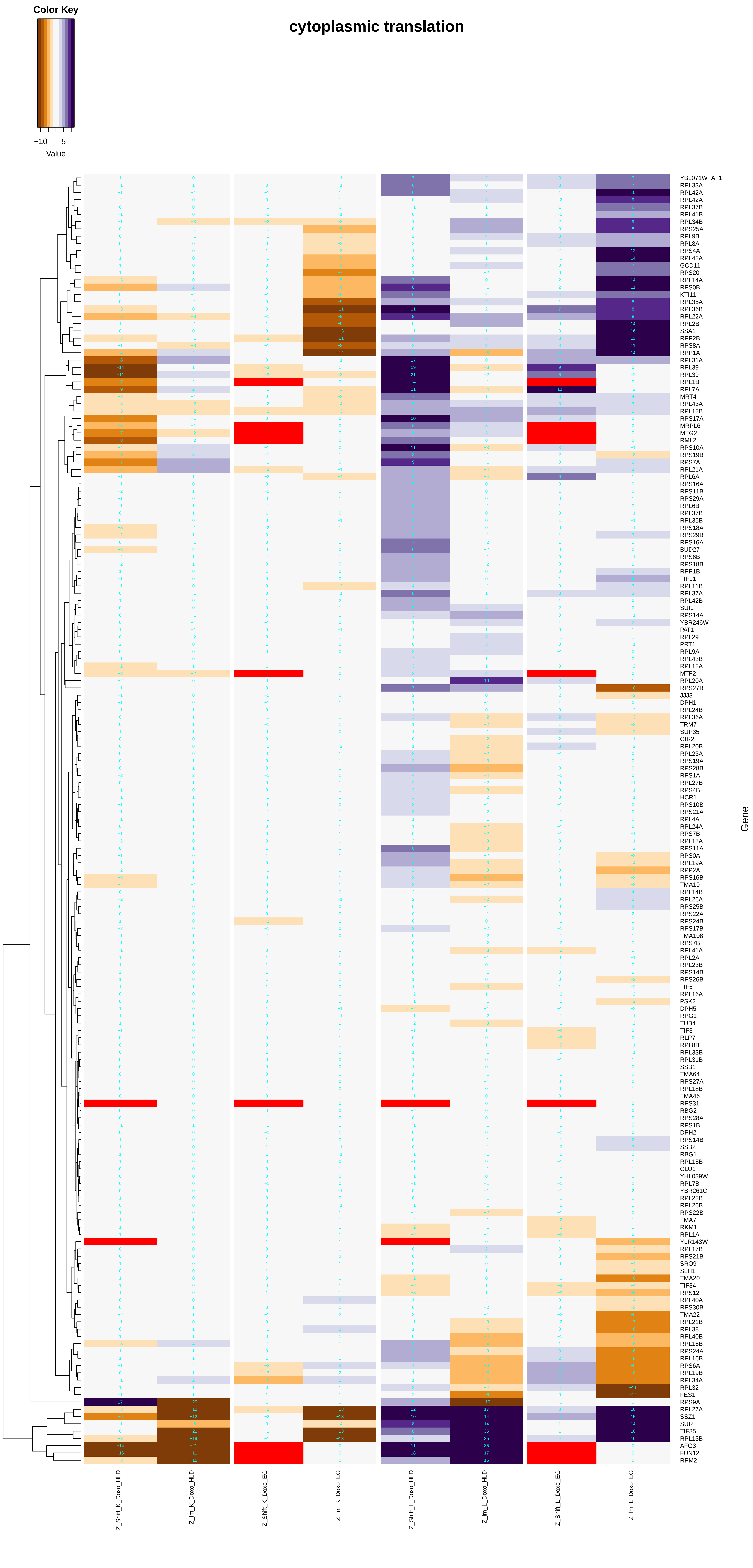

Color Key
−10 5
Value
cytoplasmic translation
Gene
YBL071W−A_1
RPL33A
RPL42A
RPL42A
RPL37B
RPL41B
RPL34B
RPS25A
RPL9B
RPL8A
RPS4A
RPL42A
GCD11
RPS20
RPL14A
RPS0B
KTI11
RPL35A
RPL36B
RPL22A
RPL2B
SSA1
RPP2B
RPS8A
RPP1A
RPL31A
RPL39
RPL39
RPL1B
RPL7A
MRT4
RPL43A
RPL12B
RPS17A
MRPL6
MTG2
RML2
RPS10A
RPS19B
RPS7A
RPL21A
RPL6A
RPS16A
RPS11B
RPS29A
RPL6B
RPL37B
RPL35B
RPS18A
RPS29B
RPS16A
BUD27
RPS6B
RPS18B
RPP1B
TIF11
RPL11B
RPL37A
RPL42B
SUI1
RPS14A
YBR246W
PAT1
RPL29
PRT1
RPL9A
RPL43B
RPL12A
MTF2
RPL20A
RPS27B
JJJ3
DPH1
RPL24B
RPL36A
TRM7
SUP35
GIR2
RPL20B
RPL23A
RPS19A
RPS28B
RPS1A
RPL27B
RPS4B
HCR1
RPS10B
RPS21A
RPL4A
RPL24A
RPS7B
RPL13A
RPS11A
RPS0A
RPL19A
RPP2A
RPS16B
TMA19
RPL14B
RPL26A
RPS25B
RPS22A
RPS24B
RPS17B
TMA10B
RPS7B
RPL41A
RPL2A
RPL23B
RPS14B
RPS26B
TIF5
RPL16A
PSK2
DPH5
RPG1
TUB4
TIF3
RLP7
RPL8B
RPL33B
RPL31B
SSB1
TMA64
RPS27A
RPL18B
TMA46
RPS31
RBG2
RPS28A
RPS1B
DPH2
RPS14B
SSB2
RBG1
RPL15B
CLU1
YHL039W
RPL7B
YBR261C
RPL22B
RPL26B
RPS22B
TMA7
RKM1
RPL1A
YLR143W
RPL17B
RPS21B
SRO9
SLH1
TMA20
TIF34
RPS12
RPL40A
RPS30B
TMA22
RPL21B
RPL38
RPL40B
RPL16B
RPS24A
RPL16B
RPS6A
RPL19B
RPL34A
RPL32
FES1
RPS9A
RPL27A
SSZ1
SUI2
TIF35
RPL13B
AFG3
FUN12
RPM2
Z_Shift_K_Doxo_HLD
Z_Im_K_Doxo_HLD
Z_Shift_K_Doxo_EG
Z_Im_K_Doxo_EG
Z_Shift_L_Doxo_HLD
Z_Im_L_Doxo_HLD
Z_Shift_L_Doxo_EG
Z_Im_L_Doxo_EG
